
JOURNAL INFORMATION
==============================
NLM Title Abbreviation: Insights Imaging
Journal ID: Insights Imaging
NLM Journal ID: 101532453

Insights into Imaging

EISSN: 1869-4101
Publisher: Springer

ARTICLE INFORMATION
==============================
PMCID: PMC8200283
PMID: 34121147
DOI: 10.1186/s13244-021-01014-5
Article ID: 1014
Article version: 2
Subjects: Meeting Abstracts

ECR 2021 Book of Abstracts

Electronic publication date: 2021 Jun 13
Publication date: 2021 Jun
Volume: 12
Issue: Suppl 2
Issue sponsor: This publication was not supported by sponsorship.
Electronic Location ID: 75
Copyright: © The Author(s) 2021
Copyright year: 2021
License: Open Access This article is licensed under a Creative Commons Attribution 4.0 International License, which permits use, sharing, adaptation, distribution and reproduction in any medium or format, as long as you give appropriate credit to the original author(s) and the source, provide a link to the Creative Commons licence, and indicate if changes were made. The images or other third party material in this article are included in the article’s Creative Commons licence, unless indicated otherwise in a credit line to the material. If material is not included in the article’s Creative Commons licence and your intended use is not permitted by statutory regulation or exceeds the permitted use, you will need to obtain permission directly from the copyright holder. To view a copy of this licence, visit http://creativecommons.org/licenses/by/4.0/.
License URL: https://creativecommons.org/licenses/by/4.0/

Conference name: European Congress of Radiology 2021
Conference location: Virtual
Conference date: 3-7 March 2021
issue-copyright-statement: © European Society of Radiology (ESR) 2021

==============================

### BOOK OF ABSTRACTS

CONTENTS

3 – 117 LIVE PROGRAMME (A)

118 – 331 ON-DEMAND PROGRAMME (B)

332 SCIENTIFIC AND EDUCATIONAL EXHIBITS (C)

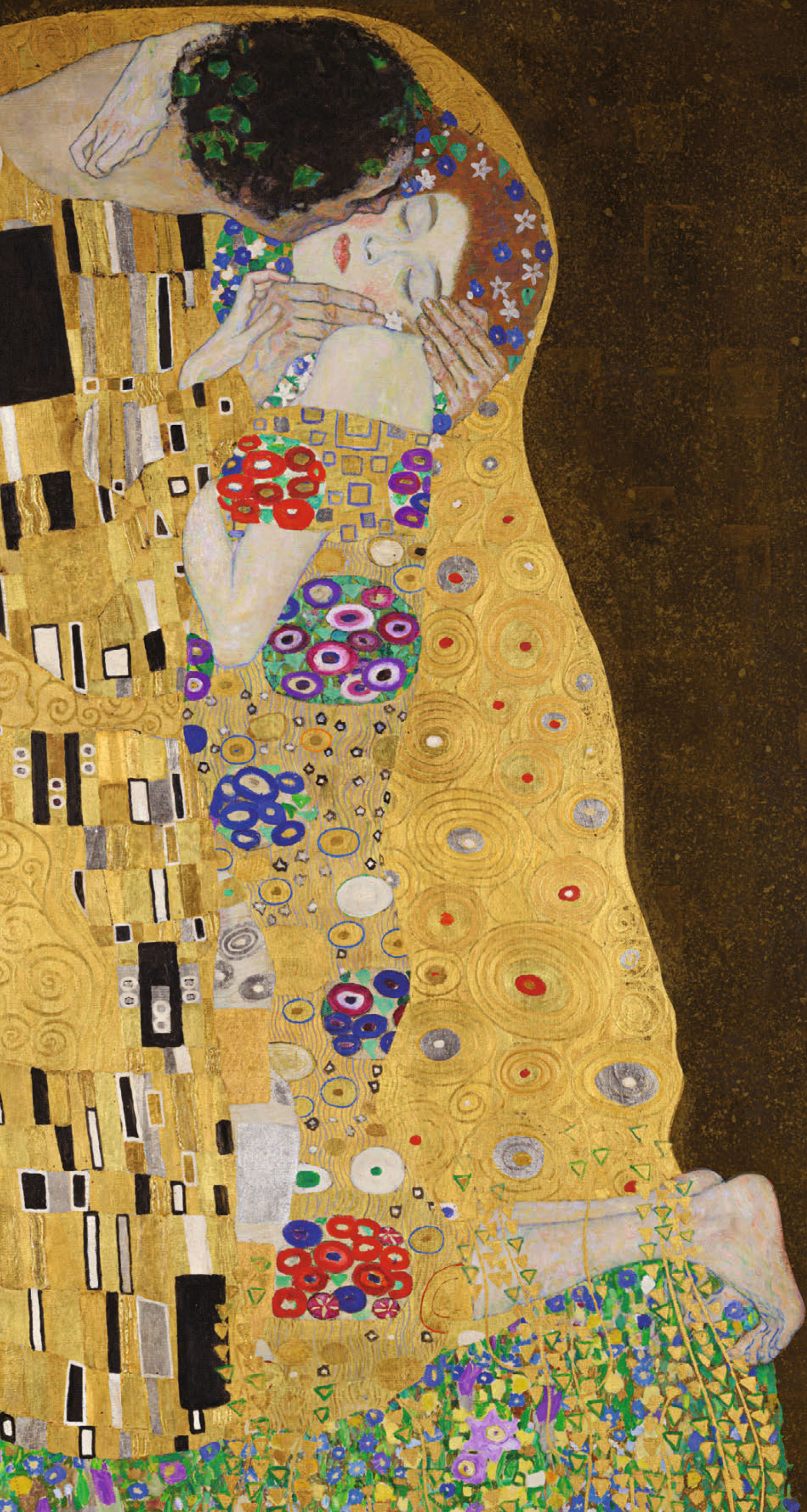

EUROPEAN CONGRESS OF RADIOLOGY

ECR 2021

myESR.org

EUROPEAN SOCIETY OF RADIOLOGY

Springer

**Disclaimer**

The ECR 2021 Book of Abstracts is published by the European Society of Radiology (ESR) and summarises the presentations accepted to be held online at the European Congress of Radiology 2021 (Vienna, Austria, March 3 - March 7, 2021). Abstracts were submitted by the authors warranting that good scientific practice, copyrights and data privacy regulations have been observed and relevant conflicts of interest declared.

Abstracts reflect the authors' opinions and knowledge. The ESR does not give any warranty about the accuracy or completeness of medical procedures, diagnostic procedures or treatments contained in the material included in this publication. The views and opinions presented in ECR abstracts and presentations, including scientific, educational and professional matters, do not necessarily reflect the views and opinions of the ESR.

In no event will the ESR be liable for any direct or indirect, special, incidental, consequential, punitive or exemplary damages arising from the use of these abstracts.

The Book of Abstracts and all of its component elements are for general educational purposes for health care professionals only and must not take the place of professional medical advice. Those seeking medical advice should always consult their physician or other medical professional.

In preparing this publication, every effort has been made to provide the most current, accurate, and clearly expressed information possible. Nevertheless, inadvertent errors in information can occur. The ESR is not responsible for typographical errors, accuracy, completeness or timeliness of the information contained in this publication.

The ECR 2021 Book of Abstracts is a supplement to Insights into Imaging (1869-4101) and published under Creative Commons Attribution 4.0 International License.

Insights Imaging (2021) 12 (Suppl 2): 75
https://doi.org/10.1186/s13244-021-01014-5
Published Online: 13 June 2021

### A

### Live Programme

Clinical Trials in Radiology (CTiR)
Coffee & Talk (open forum) Sessions (C)
ESR at Work Sessions
European Excellence in Education (E³)
EuroSafe Imaging Sessions (EU)
Hands-on Workshop (HW)
Healthcare Professionals in Focus (IF)
Joint Sessions
Meets Sessions (Meets)
Multidisciplinary Sessions (MS)
New Horizons Sessions (NH)
Plenary Lectures (PL)
Professional Challenges Sessions (PC)
Refresher Courses (RC)
Research Presentation Sessions (RPS)
Special Focus Sessions (SF)
State of the Art Symposia (SA)
Transatlantic Course of ESR and RSNA (TC)

**Wednesday, March 3 ................. 4**
**Thursday, March 4.....................25**
**Friday, March 5 .........................49**
**Saturday, March 6 .....................70**
**Sunday, March 7.......................94**

### Wednesday, March 3

08:00-09:00 CHANNEL 2

### Research Presentation Session: Artificial Intelligence & Machine Learning

### RPS 205

### Artificial intelligence (AI) in unmet clinical needs

**Moderator**
Luis Martí-Bonmatí; Valencia/ES

### RPS 205-2

**Deep learning algorithm to predict positive MRI of the sacroiliac joints according to the ASAS definition in patients with recent axial spondyloarthritis**

*C. Martin*[1], A. Bordner[1], T. Aouad[2], C. Lopez Medina[3], A. Molto[1], M. Dougados[1], H. Talbot[2], A. Feydy[1]; [1]Paris/FR, [2]Gif-Sur-Yvette/FR, [3]Cordoba/ES

**Purpose:** MRI of the sacroiliac joints (MRI-SIJ) is essential for Axial Spondyloarthritis early diagnosis. The bone marrow oedema (BME) ASAS definition of positive MRI-SIJ is used in cohorts, but the inter-reader agreement is unperfect. Our goal was to predict the MRI-SIJ status according to the ASAS definition in patients with early Axial Spondyloarthritis applying Artificial Intelligence and Machine Learning.
**Methods or Background:** Baseline patients from the DESIR cohort with full agreement for MRI-SIJ status according to the central readers were initially included. We first manually segmented the iliac and sacrum bones in 51 patients (31 for training, 6 for validation and 14 for evaluation) to learn automatic segmentation using 2 independent convolutional neuronal networks (U-Net). Then, we trained a neuronal network (ResNet-18) to detect slices with BME. The MRI-SIJ positivity in our model was defined by the presence of BME in at least 2 slices. For BME detection, we used 155 segmented MRI-SIJ (51 manually and 104 automatically). We split these patients into 105 train, 14 validation and 36 evaluation. Correlation was studied using Matthews correlation coefficient.
**Results or Findings:** Evaluation of segmentation found an Intersection Over Union of 73% for the iliac and 56% for the sacrum between manual and automatic. Correlation between central readers and our model was 0.78 for BME classification.
**Conclusion:** Our algorithm successfully segmented the iliac and sacrum bones and also achieved good results (0.78) for BME classification.
**Limitations:** These results are still not as efficient as a trained radiologist, we are optimistic that future work will be able to reach it.
**Ethics Committee Approval:** The appropriate local medical ethical committees approved this study.
**Funding for this study:** This study was conducted thanks to a grant from the Societé Française de Rhumatologie and an ASAS Research Grant.
**Author Disclosures:**
Antoine Feydy: Nothing to disclose
Clementina Lopez Medina: Nothing to disclose
Maxime Dougados: Nothing to disclose
Adrien Bordner: Nothing to disclose
Anna Molto: Nothing to disclose
Charlotte Martin: Nothing to disclose
Theodore Aouad: Nothing to disclose
M. Hugues Talbot: Nothing to disclose

### RPS 205-3

**Automatic pancreas segmentation using a novel deep learning method: evaluation on both arterial and portal phases CT**

*W. Xiheng*, H. Xue, T. Qu, X. Li, S. Cheng, J. Zhengyu; Beijing/CN
*(happyxiheng@sina.com)*

**Purpose:** To present a novel deep learning method for automatic pancreas segmentation and evaluate segmentation performance on both arterial and portal phases CT images.
**Methods or Background:** We present a deep convolutional neural network called two-stage global-local progressive fusion network for pancreas segmentation. The deep network is developed to combine global and local information of the pancreas through two modules: 1) global information guided pancreas segmentation module; 2) local information refinement pancreas segmentation module. The former guides the network to learn the position of the pancreas in the abdomen, which effectively inhibits the interference of other suspected structures. The latter further optimizes the details of the local contour of the pancreas learned by model. 179 patients with two phases preoperative CT images were retrospectively enrolled and were divided 139 for training, 20 for validation and 20 for testing. Dice similarity coefficient (DSC) was the objective evaluation index.
**Results or Findings:** The training and validation set had 95 males and 64 females, with the mean age of 53.2 years, and there were 14 males and 6 females in the testing set, with the mean age of 49.5 years. There were no significant differences either in gender or mean age of two groups (p=0.376 and 0.429, respectively). The present segmentation method was tested on the above 20 testing set. We achieved high DSC of 0.894 and 0.906 on arterial and portal phases respectively, and there was no significant difference between the two phases (p=0.068).
**Conclusion:** The proposed two-stage global-local progressive fusion network can achieve good performance of segmentation on both arterial and portal phase.
**Limitations:** Multi-center validation is still required.
**Ethics Committee Approval:** Not applicable.
**Funding for this study:** Not applicable.
**Author Disclosures:**
Jin Zhengyu: Nothing to disclose
Wang Xiheng: Nothing to disclose
Xiuli Li: Nothing to disclose
Sihang Cheng: Nothing to disclose
Taiping Qu: Nothing to disclose
Huadan Xue: Nothing to disclose

### RPS 205-4

**Multivariable diagnostic prediction model development study for gene expression profile from MRI ADC radiomics with artificial neural networks in malignant breast lesions**

B. Baysal, H. Baysal, *M. B. Eser*, M. B. Doğan, O. Alimoglu; Istanbul/TR
*(bilgineser@hotmail.com)*

**Purpose:** The purpose of this study is to develop a virtual biopsy tool by defining breast cancer with neural networks created based on MRI ADC radiomics.
**Methods or Background:** This retrospective model development study included a consecutive cohort of patients with malign breast lesions operated on from January 2015 to January 2020 in one tertiary university hospital. Of the operated 578 patients, 221 patients were included in this study. The mean patient age was 54±11 years, and 220/221 were women. Three sample size configurations were identified based on tumor size. Three observers segmented tumors on ADC maps, and an agreement was evaluated using the Dice coefficient. Predictors were determined as radiomics features (n = 851). Feature selection was based on intraclass correlation coefficient, coefficient variance, variance inflation factor, and LASSO regression analyzes. Outcomes were identified as 13 pathology results, including tumor histology, immunohistochemical markers, and gene expression profiles. A multivariable diagnostic prediction model was developed with artificial neural networks (ANN). Internal validation was performed using bootstrapping and cross-validation. ANN performance was presented as an area under the receiver operating characteristic curve (AUC) and accepted as successful if the AUC >0.85 and p-value <0.01.
**Results or Findings:** The most noticeable results of this study are - many radiomics features are not stable (93-96%), the relationship between stable features and outcomes is weak, and despite all, 16 of the 26 neural networks trained over a 1 cm3 lesion size fulfill the binary classification task.
**Conclusion:** This study successfully trained neural networks that can diagnose triple-negative and HER2-enriched breast malignant lesions over 1 cm3. This situation will be a potential development for both the patient and the clinician.
**Limitations:** Retrospective study design
**Ethics Committee Approval:** Decision number: 2020/0303
**Funding for this study:** Not applicable
**Author Disclosures:**
Begumhan Baysal: Nothing to disclose
Orhan Alimoglu: Nothing to disclose
Mehmet Bilgin Eser: Nothing to disclose
Hakan Baysal: Nothing to disclose
Mahmut Bilal Doğan: Nothing to disclose

### RPS 205-5

**Liver metastases changes analysis in CECT based on simultaneous deep learning**

A. Szeskin, A. Dodi, S. Rochman, R. Lederman, L. Joskowicz, *J. Sosna*; Jerusalem/IL
*(jacobs@hadassah.org.il)*

**Purpose:** To evaluate the performance of a novel method that simultaneously finds and quantifies the changes of liver metastasis in contrast enhanced CT (CECT) between the current and the prior scans by deep learning classification.

**Methods or Background:** We developed a novel deep learning changes analysis method inputs the current and prior CECT scans and automatically detects, segments and classifies the liver metastases. It uses a unique 3D U-Net classifier that simultaneously compares the scans and finds their differences. The network was trained on 68 pairs of clinical abdominal CECT scans from two institutions of patients with metastatic disease (colon, breast, pancreas) acquired in the venous phase with a total of 2,042 liver lesions. We quantified the performance of our method and compared it to the standard standalone approach trained on 102 scans of similar characteristics with a total of 1,739 liver metastases. We obtained manual delineations of the metastases on all scans from an expert radiologist. We report the precision and recall for the lesion detection and the Dice coefficient for the metastases segmentation.
**Results or Findings:** The simultaneous method Precision/Recall outperforms the standalone method by 5.5/4.1% and 20.5/2.2% for metastases of diameter >5mm and >10mm, respectively: simultaneous Precision/Recall of 0.76/0.75 (std=0.28/0.24) and 0.94/0.93 (std=0.13/0.16) vs. standalone Precision/Recall of 0.72/0.72 (std=0.26/0.19) and 0.78/0.91 (std=0.23/0.15) respectively. The simultaneous segmentation method Dice coefficient also outperforms the standalone method by 5.1% and 7.3% for metastases of diameter >5mm and >10mm, respectively: simultaneous 0.81 (std=0.2) and 0.88 (0.08) vs. standalone 0.78 (std=0.1) and 0.82 (0.09), respectively.
**Conclusion:** Simultaneous deep learning detection and quantitative analysis of liver metastases follow-up changes analysis in CECT outperforms standard standalone detection.
**Limitations:** Data from two institutions, test set of 40 scans
**Ethics Committee Approval:** Yes
**Funding for this study:** None
**Author Disclosures:**
Richard Lederman: Nothing to disclose
Leo Joskowicz: Nothing to disclose
Adi Szeskin: Nothing to disclose
Shalom Rochman: Nothing to disclose
Jacob Sosna: Nothing to disclose
Aviv Dodi: Nothing to disclose

### RPS 205-6

**Artificial intelligence software for flagging intracranial haemorrhage on head CT associated with reduction of mortality, readmission rate and CT turnaround time**

*D. Raskin*, C. Hoffmann, G. Twig, E. Konen, G. Yaniv; Ramat Gan/IL

**Purpose:** To assess the effect of Artificial Intelligence for flagging patients with intracranial haemorrhage in emergent care setting on patient outcomes.
**Methods or Background:** Data of all adult patients with diagnosis of ICH on head CT , admitted to the institution's emergency department (ED) was collected prior to integration of AI software and after. Study variables included demographics, clinical data, imaging data, head CT report turnaround time and inpatient length of stay. The primary variable for outcomes was 30 and 120-day mortality. Secondary variables were 120 days readmission rate, inpatient length of stay and head CT report turnaround time.
**Results or Findings:** 648 patients were eligible for the analyzed period (299 pre-AI, 349 Post-AI). Demographics, emergency severity score, type of ICH, complete blood count, comorbidities and smoking history were non-significantly different between the groups. 30 and 120 day all-cause mortality were significantly reduced post-AI (27.3% vs 16.8%, 30.8% vs 20.5%, respectively, p<0.005). Readmission rate was significantly reduced post-AI (0.37 vs 0.25, p<0.05). Head CT report turnaround time was significantly reduced post-AI (8.2 vs 7.2, p<0.007). Inpatient length of stay showed a strong trend in time reduction post-AI (7.6 vs 7.2 days, p=0.1).
**Conclusion:** Implementation of AI software for flagging patients with ICH for a year resulted in significant reduction of mortality, readmission rate and head CT report turnaround time.
**Limitations:** A retrospective study conducted in a single center.
**Ethics Committee Approval:** The study was approved by Institutional Review Board.
**Funding for this study:** No funding
**Author Disclosures:**
Eli Konen: Nothing to disclose
Chen Hoffmann: Nothing to disclose
Gilad Twig: Nothing to disclose
Daniel Raskin: Consultant at Aidoc
Gal Yaniv: Consultant at Aidoc

### RPS 205-7

**Multi-stage AI analysis system to support prostate cancer diagnostic imaging**

J. Suchánek[1], L. Rundo[1], A. Mehan[1], C. Doran[1], A. R. Padhani[2], C. Kastner[1], T. Barrett[1], E. Sala[1], *A. W. Rix*[1]; [1]Cambridge/UK, [2]Northwood/UK
*(antony.rix@lucidamedical.com)*

**Purpose:** To investigate whether Artificial Intelligence (AI) techniques could improve specificity, while maintaining sensitivity and NPV, of pre-biopsy MRI for suspected prostate cancer.
**Methods or Background:** An AI-based system was developed using a proprietary, multi-stage architecture, and trained using PROMISE12, NCI-ISBI 2013 Challenge and PROSTATEx datasets, partitioned into training, validation and held-out test sets.Accuracy was evaluated on validation and held-out test sets, and compared with literature on radiologist interpretations and similar AI/CAD approaches.
**Results or Findings:** As pre-biopsy rule-out test, the system identified patients with Gleason≥3+4 clinically significant prostate cancer (csPCa) with sensitivity 93% (95% CI 82-100%), specificity 76% (64-87%), NPV 95% (88-100%), and AUC 0.92 (0.84-0.98), using biparametric MRI (bpMRI) data from the PROSTATEx combined validation and held-out test sets (80 patients, 35% csPCa prevalence). Performance was higher on the 40-patient held-out test set. Equivalent sensitivity is reported for comparable AI/CAD systems at specificities from 6%-42%. Radiologists at Likert/PI-RADS 3 thresholds achieved per-patient sensitivity 88-93%, specificity 18-68%, and NPV 76-97%.Target identification was evaluated through submission to the PROSTATEx Grand Challenge, a blinded test with 208 radiologist-identified lesions from 140 patients. The system identified lesions containing csPCa with AUC 0.84 using bpMRI data.For prostate gland segmentation, the system achieved 92% average Dice score on held-out test cases from the PROMISE12 dataset (10 patients), in line with the state-of-the-art.
**Conclusion:** The AI system showed promising performance and specificity, suggesting it could help support exclusion of csPCa with high sensitivity and NPV, while assisting the identification of lesions to target for biopsy.
**Limitations:** Methodological and dataset differences and test set size limit comparisons. Further development, evaluation and regulatory approvals are planned.
**Ethics Committee Approval:** Exempt.
**Funding for this study:** Granta Innovation, Lucida Medical
**Author Disclosures:**
Antony William Rix: Founder at Granta Innovation Founder at Lucida Medical
Leonardo Rundo: Consultant at Lucida Medical
Christof Kastner: Nothing to disclose
Evis Sala: Founder at Lucida Medical
Jakub Suchánek: Employee at Granta Innovation
Aman Mehan: Nothing to disclose
Chris Doran: Nothing to disclose
Tristan Barrett: Nothing to disclose
Anwar R. Padhani: Nothing to disclose

**Live Q&A**

**08:00-09:00** **CHANNEL 3**

### EuroSafe Imaging Session

*Jointly organised by EuroSafe Imaging and IAEA*

### EU 6

### Building capacity and quality/safety awareness in Africa

**Moderator**
Guy Frija; Paris/FR

### EU 6-2

**Introduction (part 1)**
Shaukat Abdulrazak; Vienna/AT
*(S.Abdulrazak@iaea.org)*

### EU 6-3

**Introduction (part 2)**
May Abdel Wahab; Vienna/AT

### EU 6-4

**IAEA policy in Africa**
Peter Johnston; Vienna/AT
*(p.johnston@iaea.org)*

**Learning Objectives:**
1. To provide an overview of IAEA's efforts to promote radiation protection in patients in Africa.
2. To understand the challenges and opportunities to improve patient safety in Africa through the demonstration of successful examples.

3. To provide a roadmap for improving radiation protection of patient activities in Africa.

### EU 6-5

**Strengthening radiology in Africa: the IAEA multifactorial approach**

Diana Paez; Vienna/AT

**Learning Objectives:**
1. To provide an overview of IAEA's capacity building activities in Africa.
2. To learn how African countries can concretely benefit from IAEA support.
3. To explore synergies between IAEA, ESR, and Africa.

### EU 6-6

**African Society of Radiology (ASR) expectations**

Tarek El-Diasty; Mansoura/EG
*(teldiasty@hotmail.com)*

**Learning Objectives:**
1. To understand the role of the African Society of Radiology in promoting quality and safety in radiology practice in Africa.
2. To understand the needs of different African countries.
3. To discuss methods of promoting the exchange of knowledge between African countries via ESR and IAEA.

### EU 6-7

**EuroSafe Imaging Star concept in Africa**

Boudjema Mansouri; Algiers/DZ
*(boudjema.mansouri@gmail.com)*

**Learning Objectives:**
1. To integrate the AFROSAFE Rad Stars initiative into the AFROSAFE Rad radiation safety campaign (action plan 2019-2021).
2. To define new criteria and rates adapted for the specificities of African countries, the practice of medical imaging, and the implementation of radiation protection.
3. To learn about the impact of EuroSafe Imaging on the launch of AFROSAFE Rad Stars.

### EU 6-8

**Introducing ESR iGuide Clinical Decision Support in Africa (part 1)**

Dina Husseiny Salama; Cairo/EG
*(drdinahusseiny@yahoo.com)*

**Learning Objectives:**
1. To become familiar with the experiences and the proactive actions in the implementation of ESR iGuide in Africa. 2. To learn about the best methodologies for learning CDS in Africa. 3. To understand the future directions and suggestions towards a better clinical decision making in Africa.

### EU 6-9

**Introducing ESR iGuide Clinical Decision Support in Africa (part 2)**

Michael G. Kawooya; Kampala/UG

**Learning Objectives:**
1. To become familiar with the experiences and the proactive actions in the implementation of ESR iGuide in Africa. 2. To learn about the best methodologies for learning CDS in Africa. 3. To understand the future directions and suggestions towards a better clinical decision making in Africa.

**Live Q&A**

**08:00-09:00 CHANNEL 5**

### Research Presentation Session: Musculoskeletal

### RPS 210

### Innovative diagnostic techniques in musculoskeletal radiology

**Moderator**
Mario Maas; Amsterdam/NL

### RPS 210-2

**High-resolution 3D MRI for chondrocalcinosis detection in the knee: a prospective cohort study comparing 7 Tesla and 3 Tesla MRI with CT**

*C. Germann*, J. Galley, A. L. Falkowski, S. Fucentese, C. W. Pfirrmann, D. Nanz, R. Sutter; Zurich/CH
*(christoph.germann@googlemail.com)*

**Purpose:** To assess the detection of chondrocalcinosis in knee joints with a 3D Dual-Echo-Steady-State (DESS) sequence at 7T MRI in comparison to 3T MRI, versus CT as current cross-sectional imaging standard.
**Methods or Background:** CT and 7T MRI (DESS) of knee joints in 42 patients with radiographically known chondrocalcinosis (13 of 42 bilateral) were prospectively acquired for all included patients (N=55 knee joints). Additionally, 3T knee-MRI (DESS) was performed (N=20 knee joints). Two fellowship-trained musculoskeletal radiologists scored the images regarding presence and extent of cartilage calcification, diagnostic confidence level and sharpness of calcific deposits. As ultimate reference standard for calcifications, micro-CT of the menisci was acquired after knee arthroplasty procedure (N=1). Nonparametric tests were used to compare the different modalities. A P-value <.05 was considered to represent statistical significance.
**Results or Findings:** Compared to both 7T MRI and CT, 3T MRI detected significantly fewer cartilage calcifications (reader 1: P=.008; reader 2: P<.001). For both readers, the diagnostic confidence was significantly higher for 7T MRI than for 3T MRI (P<.001) and significantly higher for 7T MRI than for CT (P=.03). Both readers rated the delineation of calcific deposits significantly sharper in 7T MR images compared to either 3T MR) or CT (P<.001) images. Micro-CT in one patient suggested that standard-CT partly failed to detect meniscal calcifications that could be identified both in 7T MRI and micro-CT.
**Conclusion:** 3D-DESS imaging at 7T MRI is superior at detection and accurate delineation of chondral calcific deposits compared to lower field strength MRI and CT.
**Limitations:** Ultimate Reference Standard (micro-CT) only for one patient DESS sequence at 3T and 7T not complete identical (due to SAR and SNR limitations)
**Ethics Committee Approval:** approved study (cantonal ethics committee Zurich, Switzerland)
**Funding for this study:** No funding.
**Author Disclosures:**
Julien Galley: Nothing to disclose
Christian Pfirrmann: Nothing to disclose
Anna Falkowski: Nothing to disclose
Sandro Fucentese: Nothing to disclose
Daniel Nanz: Nothing to disclose
Christoph Germann: Nothing to disclose
Reto Sutter: Nothing to disclose

### RPS 210-3

**Comparison of a novel MRI technique that produces superior bone contrast with 2D CT for the assessment of glenoid bone loss**

*H. Alizai*, D. Chen, J. Chang, H. B. Ellis, P. L. Wilson; Dallas, TX/US
*(hamzaalizai@gmail.com)*

**Purpose:** FRACTURE (FFE Resembling A CT Using Restricted Echo-spacing), a 3D gradient echo pulse sequence with restricted echo-spacing combined with an automated post-processing is a technical innovation which provides superior cortical and trabecular bone contrast on MRI.
The objective of this study is to validate this technique in a cadaveric glenoid bone loss model using 2D-CT as the reference standard.
**Methods or Background:** Glenoid fractures of varying sizes were arthroscopically created in 10 cadaveric shoulders. 2D-CT and MRI FRACTURE (on 3T Philips Ingenia; Philips Healthcare, Best, Netherlands) were performed on all 10 shoulders. The CT and MRI were presented in a blinded fashion to an experienced musculoskeletal radiologist for measurement of the glenoid fracture height and width. Paired t-tests were performed to compare the measurements obtained on CT with those obtained on the MR FRACTURE images.
**Results or Findings:** There was no statistically significant difference between CT and MRI in terms of the measured glenoid fracture height (P-value=0.54). The mean height measured on CT was 23.40mm (SD=6.87) and on MRI was 22.50mm (SD=7.28). Additionally, there was no statistically significant difference between CT and MR in terms of the measured glenoid fracture width (P-value=0.86). The mean width on CT measured 7.50mm (SD=3.57) and on MRI measured 7.60mm (SD=3.50).
**Conclusion:** Measurements of glenoid fracture height and width on CT were comparable with measurements obtained using the MR FRACTURE technique, which may obviate preoperative CT in patients with anterior shoulder instability.
**Limitations:** Feasibility study with small number of subjects
**Ethics Committee Approval:** The study protocol was approved by the institutional research advisory panel. IRB approval was not required for this cadaveric model.

**Funding for this study:** The cadaveric shoulders were donated by Arthrex (Arthrex, Naples, FL, USA). Research funding provided by Scottish Rite Hospital for Children.
**Author Disclosures:**
Joseph Chang: Nothing to disclose
Hamza Alizai: Nothing to disclose
Philip L Wilson: Nothing to disclose
Henry B Ellis: Nothing to disclose
Diana Chen: Nothing to disclose

### RPS 210-4

### High-resolution US of the small but clinically relevant nerves travelling across the posterior triangle of the neck

*L. Tovt*[1], M. Pansecchi[1], F. Zaottini[1], R. Picasso[1], F. Pistoia[1], S. Sanguinetti[1], M. Miguel-Pérez[2], C. Martinoli[1]; [1]Genoa/IT, [2]Barcelona/ES

**Purpose:** The aim of this study was to describe the anatomy and the US scanning technique of the small but clinically relevant nerves travelling across the posterior triangle of the neck and to discuss the role of high frequency transducers in evaluating pathologic conditions affecting them.
**Methods or Background:** The anatomy of the suprascapular (SS), spinal accessory (SA), long thoracic (LT), phrenic (PH), supraclavicular (SC), great auricular (GA), lesser occipital (LO), and transverse cervical (TC) nerves was investigated in n=4 anatomical specimens and n=10 healthy volunteers by means of high frequency US transducers. N=7 patients with clinical symptoms suggesting neuropathy of the aforementioned nerves were successively studied. Among them, n=4 complained of pain and weakness in the shoulder girdle, n=1 felt paresthesias at the base of the neck, n=1 experienced neuropathic pain around the retromandibular region and n=1 demonstrated scapular winging.
**Results or Findings:** Cadaveric dissection successfully demonstrated the SS, SA, LT, PH, SC, GA, LO and TC. High-resolution US was able to track the anatomic course of the aforementioned nerves across the posterior triangle of the neck in all the heathy volunteers. In patients with suspected neuropathy, US demonstrated n=3 cases with enlarged SS in relation to Parsonage-Turner syndrome; n=1 case of SS impingement against a paralabral ganglion cyst; and n=3 iatrogenic neuromas affecting the SA, GA and SC respectively.
**Conclusion:** An extensive knowledge of the anatomy of the posterior triangle of the neck and high frequencies probes are required to successfully investigate patients with suspect neuropathy affecting the locoregional nerves.
**Limitations:** None
**Ethics Committee Approval:** The study was conducted in accordance with the declaration of Helsinki.
**Funding for this study:** None
**Author Disclosures:**
Federico Zaottini: Nothing to disclose
Carlo Martinoli: Nothing to disclose
Luca Tovt: Nothing to disclose
Maribel Miguel-Pérez: Nothing to disclose
Michelle Pansecchi: Nothing to disclose
Sara Sanguinetti: Nothing to disclose
Riccardo Picasso: Nothing to disclose
Federico Pistoia: Nothing to disclose

### RPS 210-5

### The role of multidimensional hip anatomy in early joint space narrowing: a multi-centric cross-sectional study

*M. Klontzas*[1], E. Volitakis[1], Ü. Aydingöz[2], K. G. Chlapoutakis[1], A. H. Karantanas[1]; [1]Heraklion/GR, [2]Ankara/TR
*(miklontzas@gmail.com)*

**Purpose:** To use multivariate modelling in order to identify anatomical factors related to hip dysplasia or femoroacetabular impingement, which can lead to early hip joint space narrowing.
**Methods or Background:** A total of 897 hip CT examinations of 457 consecutive patients <55 years old, were retrospectively examined. Age, joint space width (JSW), center-edge (CE) angle, alpha angle, anterior acetabular sector angle (AASA) and neck-shaft angle (NSA) were recorded. Dysplasia and joint space (JS) narrowing were defined with CE angle <25o and JSW<0.15 cm, respectively. Multivariate linear regression analysis and two-step supervised clustering were performed to identify factors and combinations of factors linked to early JS narrowing.
**Results or Findings:** Anatomical factors were found to differentially affect dysplastic and non-dysplastic hips. In dysplastic hips (CE<25o), AASA (P<0.005) and CE angle (P<0.032) were the only significant predictors of JS narrowing, neither of which was alone correlated to JSW, indicating that lateral and anterior dysplasia contribute together in early degeneration. In non-dysplastic hips (CE>25o), JSW was correlated to CE angle, AASA, NSA and age (P<0.001). However, in multivariate models only CE angle was independently inversely related to JSW (B=-0.002, P<0.001) indicating the importance of pincer-type impingement on early degeneration. Two-step clustering identified two relative patient clusters, one with low JSW, higher CE angle, lower NSA and higher AASA values and a second with normal JSW, low CE angle, high NSA and low AASA values (Silhouette measure 0.4).
**Conclusion:** Multidimensional anatomy differentially drives degeneration in dysplastic and non-dysplastic hips. Lateral and anterior dysplasia contribute together and pincer-type impingement contributes independently to early degeneration. Finally, combinations of CE angle, NSA and AASA can predict JSW.
**Limitations:** n/a
**Ethics Committee Approval:** Approved - University Hospital of Heraklion (Ref No 882).
**Funding for this study:** No funding received for this study
**Author Disclosures:**
Michail Klontzas: Nothing to disclose
Konstantinos G. Chlapoutakis: Nothing to disclose
Emmanouil Volitakis: Nothing to disclose
Üstün Aydingöz: Nothing to disclose
Apostolos H. Karantanas: Nothing to disclose

### RPS 210-6

### Comparison of utility for image quality improvement between compressed sensing with developed deep learning reconstruction and conventional parallel imaging in shoulder MR imaging

*Y. Obama*[1], Y. Ohno[1], K. Yamamoto[2], A. Iwase[1], T. Ueda[1], K. Murayama[1], H. Toyama[1]; [1]Toyoake/JP, [2]Otawara/JP
*(y-obama@fujit-hu.ac.jp)*

**Purpose:** To prospectively and directly determine the capability of newly developed deep learning reconstruction (DLR) for image quality improvement on MRI obtained by conventional parallel imaging (PI) and compressed sensing (CS) in patients with various shoulder diseases.
**Methods or Background:** Thirty consecutive patients with various shoulder diseases underwent MRI at a 3T MR system by conventional PI and CS, and each MR data was reconstructed with and without DLR. To determinate quantitative image quality improvement of each acquisition with and without DLR, signal-to-noise ratio (SNR) of humeral head, percentage of coefficient of variations (%CVs) and contrast-to-noise ratio (CNR) were determined by ROI measurements in all patients. For qualitative image quality assessment, two radiologists evaluated overall image quality, artifacts and diagnostic confidence level (DCL) by 5-point scoring system, and final value of each index was made by consensus of two readers. To compare the capability for reducing examination time, all MR examination times were compared by Tukey's HSD test. On qualitative image quality evaluation, inter- observer agreement of each index was firstly determined by weighted-kappa statistics. Then, all quantitative and qualitative indexes were compared among all methods by Tukey's HSD test or Wilcoxon's signed rank test.
**Results or Findings:** CS with and without DLR showed significantly shorter examination times than conventional PI (p<0.05). Overall image quality and artifact could be significantly improved with applying DLR on shoulder MRI obtained by CS and PI (p<0.05), although diagnostic confidence had no significant influence by DLR.
**Conclusion:** CS is able to significantly reduce examination time than PI. Moreover, DLR can significantly improve image quality of shoulder MRI obtained by CS at a 3T system.
**Limitations:** Sample size
**Ethics Committee Approval:** IRB comittee in Fujita Health University Hospital
**Funding for this study:** Canon Medical Systems
**Author Disclosures:**
Yuki Obama: Nothing to disclose
Kazuhiro Murayama: Grant Recipient at Canon Medical Systems
Kaori Yamamoto: Employee at Canon Medical Systems
Hiroshi Toyama: Grant Recipient at Canon Medical Systems
Takahiro Ueda: Nothing to disclose
Yoshiharu Ohno: Grant Recipient at Canon Medical Systems
Akiyoshi Iwase: Nothing to disclose

### RPS 210-7

### Evaluation of MR-derived simulated CT-like images and simulated radiographs compared to conventional radiography in patients with suspected rotator cuff injury

*G. C. Feuerriegel*, F. Kopp, D. Pfeiffer, D. C. Karampinos, B. J. Schwaiger, M. R. Makowski, K. Woertler, A. S. Gersing; Munich/DE

**Purpose:** To evaluate the diagnostic value of MR-derived CT-like images and simulated radiographs compared with conventional radiographs in patients with suspected rotator cuff injury.
**Methods or Background:** In 23 patients with suspected rotator cuff injury (mean age 52.2±18years,13 women), 3T MR-imaging of the shoulder was performed including a 3D T1-weighted gradient-echo sequence from which intensity-inverted CT-like images were derived. The inverted MRI-image volumes were processed to achieve contrast enhancement and a cone-beam

Wednesday forward projection algorithm was used to obtain simulated radiographs. Congruency of projections and image quality was graded using a 5-point Likert-scale. Two radiologists evaluated the simulated images and corresponding conventional radiographs, performing measurements of the acromiohumeral distance(AHD) and critical shoulder angle(CSA), as well as assessing semi-quantitative scores in order to grade degenerative changes using the Samilson-classification and the type of the acromion shape (Bigliani-classification).
**Results or Findings:** Measurements on simulated radiographs were comparable to those on conventional radiographs with a substantial to almost perfect inter- and intrareader agreement (κ=0.69-1.00 and κ=0.65-0.85, respectively). Between MR-derived CT-like images in combination with simulated radiographs and conventional radiographs a substantial agreement was found regarding diagnostically relevant features, assessed on Y-and AP-projections (CSA, mean+/- standard deviation 33°+/-3.3°, κ=0.84 and 0.69; AHD, mean+/-SD, 11.8 mm+/-1.9 mm, κ=0.95 and0.60; Samilson-score, κ=0.77 and 0.77; Bigliani-score, κ=0.83 and 0.67, respectively).
**Conclusion:** The visualization and assessment of diagnostically relevant features of shoulder joints was feasible using MR-derived CT-like images and simulated radiographs and findings were comparable to those of conventional radiographs. CT-like images may provide additional information on osseous abnormalities without causing any radiation exposure.
**Limitations:** Implementation of simulated radiographs into the clinical workflow may be challenging because every patient needs an MRI and post-processing using automated computerised algorithms is up to now time consuming.
**Ethics Committee Approval:** n/a
**Funding for this study:** n/a
**Author Disclosures:**
Benedikt Jakob Schwaiger: Nothing to disclose
Marcus R. Makowski: Nothing to disclose
Alexandra Sophia Gersing: Nothing to disclose
Felix Kopp: Nothing to disclose
Georg Constantin Feuerriegel: Nothing to disclose
Daniela Pfeiffer: Nothing to disclose
Dimitrios C. Karampinos: Nothing to disclose
Klaus Wörtler: Nothing to disclose

**Live Q&A**

**09:15-10:15 CHANNEL 2**

### Special Focus Session

### SF 2

### Cranial nerves: the good, the bad, and the ugly

**Moderator**
Elizabeth Loney; Halifax/UK

### SF 2-2
**The good: cranial nerve anatomy**
Jan Walther Casselman; Bruges/BE
*(jan.casselman@azsintjan.be)*

**Learning Objectives:**
1. To learn about cranial nerve anatomy.
2. To understand which imaging techniques and sequences to use to best visualise each nerve.
3. To appreciate the path each nerve takes and adjacent structures which might affect it.

**Author Disclosures:**
Jan Walther Casselman: Speaker: Philips Healhtcare

### SF 2-3
**The bad: non-neoplastic pathology**
Simonetta Gerevini; Bergamo/IT
*(sgerevini@asst-pg23.it)*

**Learning Objectives:**
1. To appreciate the wide range of non-neoplastic conditions which may affect cranial nerves.
2. To become familiar with the best techniques and sequences to use to image such conditions.
3. To see examples of such pathology in order to aid recognition if encountered during ones own practice.

### SF 2-4
**The ugly: perineural tumour spread**
Bernhard F. Schuknecht; Zurich/CH
*(bschuknecht@mri-roentgen.ch)*

**Learning Objectives:**
1. To revise those conditions more likely to present with perineural tumour spread.
2. To become familiar with the best techniques and sequences to use to image such conditions.
3. To see examples of such pathology in order to aid recognition if encountered during ones own practice.

**Live Q&A: Challenges and solutions when imaging cranial nerves**

**09:15-10:15 CHANNEL 4**

### Professional Challenges Session

### PC 6

### Radiographers meeting the challenges of the COVID-19 pandemic

**Moderators**
Carst Buissink; Groningen/NL
George Tsivtsivadze; Tbilisi/GE

### PC 6-3
**How have our clinical practices changed due to COVID-19?**
Moreno Zanardo; Milan/IT
*(moreno.zanardo@unimi.it)*

**Learning Objectives:**
1. To discuss the challenges in medical imaging brought about by the pandemic.
2. To highlight the approaches and actions being taken to return to normal clinical services.
3. To understand the potential long-term effects of the outbreak on clinical practice.

### PC 6-4
**To look back on a year of COVID-19: the impact on radiographer education**
Pete Bridge; Liverpool/UK
*(Pete.Bridge@liverpool.ac.uk)*

**Learning Objectives:**
1. To understand the ongoing educational challenges.
2. To present staff and student experiences from the past year.
3. To recognise what we have learned through a widespread move to online teaching, learning, and assessment.

**Author Disclosures:**
Pete Bridge: Board Member: Medspace.VR

### PC 6-5
**Well-being in the era of COVID-19**
Paul Bezzina; Msida/MT
*(Paul.bezzina@um.edu.mt)*

**Learning Objectives:**
1. To understand what well-being means in the COVID era.
2. To explore strategies to help radiographers reflect and renew.
3. To discuss the coping challenges of radiographers in adjusting to the crisis.

**Live Q&A: The impact of COVID on imaging practice**

09:15-10:15 CHANNEL 5

### Research Presentation Session: Cardiac

### RPS 103

### How to further refine personal cardiovascular risk stratification

**Moderator**
Jordi Broncano; Córdoba/ES

### RPS 103-2

**Exposure to environmental tobacco smoke estimated using the SHSES scale and the aortic valve calcification score in the group of patients with arterial hypertension**

*P. Gac*, M. Poreba, G. Mazur, R. Poreba; Wroclaw/PL
*(pawelgac@interia.pl)*

**Purpose:** Objective of the study was to determine the relationship between exposure to environmental tobacco smoke (ETS) estimated on the basis of the SHSES scale and the value of the aortic valve calcification score (AVCS) in people suffering from arterial hypertension (AH).
**Methods or Background:** 107 non-smokers with diagnosed and pharmacologically managed hypertension (67.16±8.48 years) were qualified for the study. The degree of exposure to ETS was assessed using the SHSES scale; cardiac computed tomography (CCT) was also performed. Using the SHSES scale, the following subgroups of patients were distinguished: A- not exposed to ETS (0 SHSES points, n=51), B- slightly exposed to ETS (1-3 SHSES points, n=14), C- moderately exposed to ETS (4-7 SHSES points, n=22) and D- significantly exposed to ETS (8-11 SHSES points, n=20). AVCS was assessed semi-automatically, based on the multiplanar reconstruction from the non-contrast phase of the CCT. The Agatston algorithm was used.
**Results or Findings:** AVCS value in the study group was 213.59±304.86. The AVCS was significantly lower in subgroup A than in subgroups C and D. In subgroup A, the lack of aortic valve calcification was observed significantly more frequently than in subgroup D. There was a positive correlation between the number of SHSES points and AVCS (r=0.37, p<0.05). Based on the ROC curve, the SHSES value was determined as the optimal cut-off point for the prediction of the absence of aortic valve calcification, amounting to 3 points. The accuracy of SHSES<3 as the predictor of AVCS=0 was set at 62.18%.
**Conclusion:** In people with AH, there is an unfavourable relationship between the amount of exposure to environmental tobacco smoke and AVCS.
**Limitations:** Small study group size. Lack of blood cotinine concentration test.
**Ethics Committee Approval:** Local Bioethic committee approval.
**Funding for this study:** Wroclaw Medical University
**Author Disclosures:**
Rafal Poreba: Nothing to disclose
Malgorzata Poreba: Nothing to disclose
Pawel Gac: Nothing to disclose
Grzegorz Mazur: Nothing to disclose

### RPS 103-3

**Measurement techniques of epicardial adipose tissue in the diagnosis of coronary heart disease**

*A. Dobrovolskij*[1], D. Austys[2], N. Valeviciene[2], R. Stukas[2]; [1]Mainz/DE, [2]Vilnius/LT
*(adobrovolskij@gmail.com)*

**Purpose:** Epicardial adipose tissue (EAT) is considered to be an important factor in the development of cardiovascular diseases. Overall, observational studies suggest a positive association between the EAT and coronary artery disease (CAD). EAT depots are estimated by measuring thickness on different sites and volume. There is a lack of research regarding the comparison between different quantification methods of EAT in diagnostic of CAD. Therefore, we aimed to compare different EAT measurement techniques in prediction of CAD.
**Methods or Background:** There were 127 CAD and 137 non-CAD adult patients investigated in this prospective study. The patients were selected according to the length of interventricular septum (interval of 100±9.5 mm). Volume and thickness of EAT depots on ventricular free walls (6 locations) and grooves (5 locations) were measured using cardiac magnetic resonance tomography images. The diagnostic ability of different EAT measurement techniques was evaluated using receiver operating characteristic (ROC) analysis.
**Results or Findings:** Overall, EAT thickness (in all locations) and volume were higher among patients with CAD (p<0.001). ROC analysis revealed that the largest area under the curve (AUC) was observed in case of the mean value of EAT thickness measurements in interventricular and atrioventricular grooves - 0.884. The AUC in case of EAT volume was 0.816, in case of EAT thickness on left and right ventricular free walls ranged between 0.524-0.656 and 0.561-0.704 respectively.
**Conclusion:** The most suitable EAT measurement in prediction of CAD was identified to be the mean value of EAT thickness in interventricular and atrioventricular grooves areas (cut-off value - 7.9 mm). The predictive power of EAT volume showed to be similar (cut-off value - 133,5 cm3), therefore, this measurement should also be considered in diagnostic of CAD.
**Limitations:** n/a
**Ethics Committee Approval:** Ethics Committee approval provided.
**Funding for this study:** n/a
**Author Disclosures:**
Nomeda Valeviciene: Nothing to disclose
Rimantas Stukas: Nothing to disclose
Andrej Dobrovolskij: Nothing to disclose
Donatas Austys: Nothing to disclose

### RPS 103-4

**Volume CT in different morphological types of Bicuspid Aortic Valve (BAV) disease as a part of personalized management**

*M. Nedevska*, N. Slavkova, V. V. Groudeva, E. Mihaylova; Sofia/BG
*(nedevska_maria@yahoo.com)*

**Purpose:** The aim of the study is to investigate how the specific morphological BAV phenotypes, aortic configuration, and hemodynamics, can be associated with different patients' outcome in BAV disease.
**Methods or Background:** For this retrospective study, we used dynamic volume CT with 320- detector- rows. Forty- five patients (20 – 72 years) with BAV disease, underwent ECG-gated cardiac CT. Multiphase reconstructions of the aorta and the aortic valve were used as a gold standard. Valve morphology was described according to the Sievers classification. Aortic dilatation pattern was described according to Cluster classification. Aortic valve orifice area, aortic regurgitation ( AR), AR fraction, left ventricular ejection fraction ( LVEF), and the extent of dilatation and hypertrophy of the left ventricle were quantified.
**Results or Findings:** The analyses of the results show that the predominant morphological phenotype of BAV is type 0- non- raphe type. 35 % of the patients had a valvular lesion with equal counts of aortic stenosis ( AS) and aortic regurgitation ( AR). Aortic dilatation was seen in 62 % of the cases, mainly affecting the ascending aorta. In 44 % there was dilatation or hypertrophy of the left ventricle with low LVEF and disturbed pump function. 10 % of the patients underwent AVR ( Aortic valve replacement) or endoprosthetic surgery. Further evaluation of the correlation between these parameters was made.
**Conclusion:** BAV disease has huge heterogeneity in radiologic and clinical appearance. The precise study and understanding of the different morphological types of BAV can give a new therapeutic and surgical approach, considering patients' specific characteristics.
**Limitations:** There are some limitations related to the relatively small number of patients and the retrospective nature of the study.
**Ethics Committee Approval:** It is approved by the local ethical committee.
**Funding for this study:** Self funding
**Author Disclosures:**
Violeta Vassileva Groudeva: Nothing to disclose
Nia Slavkova: Nothing to disclose
Evgeniya Mihaylova: Nothing to disclose
Maria Nedevska: Nothing to disclose

### RPS 103-5

**Inflammation of remote myocardium in survivors with AMI: a CMR T2-mapping study**

*K. Xu*, Y-K. Guo; Chengdu/CN
*(382781403@qq.com)*

**Purpose:** This study aimed to assess the inflammation response in remote myocardium after acute myocardial infarction (AMI) using T2-mapping and its relationship with LV remodeling.
**Methods or Background:** 54 AMI survivors were prospectively recruited and underwent cardiac magnetic resonance scans 3 days and 3 months after AMI, respectively. 25 healthy volunteers also completed the same scan. T2 mapping, late gadolinium enhancement and cine imaging were performed to measure T2 values in remote myocardium (T2remote values), enhanced size and LV functional parameters, respectively. LV remodeling was evaluated as the change in LV end-diastolic volume (LVEDV) index at follow-up scan compared with baseline.
**Results or Findings:** Compared with T2 values in normal controls (39.44 ± 2.05 ms), T2remote values of patients in baseline (40.20 ± 1.92 ms, P = 0.013) and follow-up scan (40.94 ± 2.20 ms, P < 0.01) were both higher. The T2remote values had a marginal increase in follow-up scan (0.5 ms, -0.2 ms - 1.13 ms). Between the baseline and follow-up scans, △T2remote values were correlated with △LV eject fraction (r = -0.377, P = 0.005), △LVEDV (r = 0.578, P < 0.001) and △LV end-systolic volume (r = 0.538, P < 0.001). Furthermore,

Wednesday

△T2remote values were independently associated with LV remodeling in the multivariable linear analysis (β = 0.539, P < 0.01).
**Conclusion:** T2-mapping techniques detects the inflammation response in remote myocardium after AMI, and the changes of inflammation (T2 values) are associated with LV remodeling.
**Limitations:** None
**Ethics Committee Approval:** Institutional Review Board approval was obtained
**Funding for this study:** This study was supported by the National Natural Science Foundation of China (81771887, 81771897, 81901712 and 81971586); the Program for New Century Excellent Talents in University (No. NCET-13-0386);
**Author Disclosures:**
Ke Xu: Nothing to disclose
Ying-Kun Guo: Nothing to disclose

### RPS 103-6

**Who are the patients with MAD?**
*G. Grazzini*, S. Pradella, G. Zantonelli, M. Letteriello, C. De Amicis, M. Moroni, I. Fusi, M. Acquafresca, V. Miele; Florence/IT
*(grazzini.giulia@gmail.com)*

**Purpose:** Mitral annulus disjunction (MAD) is a structural abnormality. MAD has been associated with mitral valve prolapse (MVP) and arrhythmias. The aim of this study is to evaluate the association with MVP, cardiomyopathies (CMPs), and arrhythmias in patients with MAD who underwent cardiac magnetic resonance (CMR).
**Methods or Background:** From our Picture Archiving and Communication System, we searched for all complete CMR examinations performed between January 2018 and December 2019. For each CMR, we analyzed cine steady-state free processing images acquired in the 3-chamber and 4-chamber long-axis views identifying patients with MAD. Subsequently, all the sequences of the selected CMR with MAD were analyzed evaluating any anomaly: ventricular volumes, wall thicknesses, kinetics, presence of MVP, late gadolinium enhancement (LGE), T2, T1 and extracellular volume (ECV) values, and MAD morphology. The selected patients had been tested for the following question: Did they have arrhythmias, MVP, or suspected CMP?
**Results or Findings:** We evaluated 3000 CMR. The CMR-diagnoses were: myocarditis 20%, heart attack 30%, suspected or confirmed cardiomyopathies 20%, arrhythmias 15%, valvular diseases 10%, anatomical studies before/after surgery 2%, others 3%. We selected 98/3000 CMR with MAD. All patients with MAD on CMR images had arrhythmias, 78% had a confirmed diagnosis of MVP, 45% had CMP. LGE was present in 44% of patients with MAD and native T1 and ECV values were increased in the infero-lateral basal segments in 54% of cases.
**Conclusion:** MAD was present only in patients with arrhythmias often associated with MVP or CMP and/ or LGE. In most cases, the T1 and ECV values in the maps were increased in the infero-lateral basal segments.
**Limitations:** The main limitation is that this is a retrospective study.
**Ethics Committee Approval:** The ethics committee of our institution approved this study.
**Funding for this study:** No funding
**Author Disclosures:**
Giulia Zantonelli: Nothing to disclose
Mayla Letteriello: Nothing to disclose
Manlio Acquafresca: Nothing to disclose
Giulia Grazzini: Nothing to disclose
Vittorio Miele: Nothing to disclose
Silvia Pradella: Nothing to disclose
Mario Moroni: Nothing to disclose
Isabella Fusi: Nothing to disclose
Cristian De Amicis: Nothing to disclose

### RPS 103-7

**Gender-related differences in the systemic right ventricular failure: a cross-sectional MRI study**
A. Gregov, T. Balasko Josipovic, M. Muršić, *M. Hrabak Paar*; Zagreb/HR
*(majahrabak@gmail.com)*

**Purpose:** To detect gender differences in the morphology and function of the systemic right ventricle (SRV) in patients with transposition of the great arteries (TGA).
**Methods or Background:** Cine steady-state free precession cardiac MRI sequences were acquired using a 1,5T scanner in 25 patients with SRV (age range 19-68, 13 males): 16 with atrial-switch repair for D-TGA and 9 with congenitally-corrected TGA. SRV volumetry was performed using a standard procedure. Mean longitudinal SRV strain was measured on a 4-chamber cine view, and mean circumferential SRV strain at three cine short-axis images based on feature-tracking. Late-gadolinium enhancement (LGE) scanning was performed in 22 patients and visually assessed. Numerical parameters of SRV function were compared between genders using independent samples T-test, whereas differences in LGE were assessed using Chi-Squared test.
**Results or Findings:** Male patients with SRV, compared to their female counterparts, had significantly larger indexed SRV enddiastolic volume (139±47ml/m2 vs. 104±18ml/m2, p=0.025), lower SRV ejection fraction (39±13% vs. 52±5%, p=0.003) and SRV LGE more commonly present (9/12 vs. 1/10, p=0.002). Males had lower mean longitudinal SRV strain (-11.3±2,8% vs. -16.4±2.4%, p<0.001) and mean circumferential SRV strain in the basal (-11.0±2.9% vs. -15.3±2.6%, p<0.001) and midventricular plane (-10.5±4.1% vs. -14.8±4.0%, p=0.015). There was no significant age difference between males and females.
**Conclusion:** Results of this study suggest that male patients with SRV could have worse parameters of SRV function compared to their female counterparts, but that should be confirmed in a larger group of patients.
**Limitations:** Limited number of patients, clinical differences between male and female patients have not been analyzed.
**Ethics Committee Approval:** Obtained.
**Funding for this study:** This project has received funding from the Seed Grant funding programme of the European Society of Radiology (ESR) in collaboration with the European Institute for Biomedical Imaging Research (EIBIR).
**Author Disclosures:**
Andrija Gregov: Nothing to disclose
Tihana Balasko Josipovic: Nothing to disclose
Maja Hrabak Paar: Nothing to disclose
Miroslav Muršić: Nothing to disclose

**Live Q&A**

**10:30-11:30** **CHANNEL 2**

### E³ - ECR Master Class

### E³ 1026

### CT in musculoskeletal imaging: tips and tricks

**Moderator**
Hatice Tuba Sanal; Ankara/TR

### E³ 1026-2

**A. Metal artifact reduction: does it work?**
Anagha P. Parkar; Bergen/NO

**Learning Objectives:**
1. To describe how to optimise protocols for metal artifact reduction.
2. To list the pitfalls related to metal type and to understand the strengths and weaknesses of CT vs MRI.
3. To explain how to implement metal artifact reduction effectively in clinical practice.

### E³ 1026-3

**B. Bone marrow oedema (BME): can CT get it right?**
Emma Rowbotham; Leeds/UK

**Learning Objectives:**
1. To explain the usefulness of dual-energy assessment of bone marrow.
2. To list the pitfalls in diagnosing BME on CT and to understand the strengths and weakness of CT vs MRI.
3. To explain how to implement dual-energy assessment of bone marrow effectively in clinical practice.

### E³ 1026-4

**C. 4D-CT in musculoskeletal imaging**
Pedro Augusto Gondim Teixeira; Nancy/FR
(p.teixeira@chru-nancy.fr)

**Learning Objectives:**
1. To describe how to optimise protocols for 4D-CT.
2. To list the normal and pathological findings in joints.
3. To explain the pitfalls in 4D-CT.
**Author Disclosures:**
Pedro Augusto Gondim Teixeira: Research Grant/Support: Canon Medical Systems

**E³ 1026-5**
**D. Soft tissue applications of dual-energy CT (DECT)**
Andrea S. Klauser; Innsbruck/AT
*(andrea.klauser@i-med.ac.at)*

**Learning Objectives:**
1. To describe how to optimise your protocol.
2. To list the indications of DECT for soft tissue and to understand the strengths and weakness of CT vs MRI.
3. To explain the pitfalls in DECT.

**Live Q&A**

**10:30-11:30 CHANNEL 3**

### Refresher Course: Hybrid, Molecular and Translational Imaging

*Organised by ESHIMT*

### RC 206

### Monitoring therapeutic effects: response criteria

**Moderator**
Gerald Antoch; Düsseldorf/DE

### RC 206-2
**A. Morphological response assessment: RECIST 1.1**
Melvin D'Anastasi; Msida/MT
*(melvin.danastasi@gov.mt)*

**Learning Objectives:**
1. To understand the basic concepts of morphological therapy monitoring.
2. To understand how RECIST 1.1 has simplified the previous criteria (RECIST 1.0).
3. To understand the limitations of morphological therapy monitoring.
**Author Disclosures:**
Melvin D'Anastasi: Advisory Board: Keosys Medical Imaging; Consultant: Keosys Medical Imaging

### RC 206-3
**B. Molecular response assessment: from EORTC to PERCIST**
Helmut Prosch; Vienna/AT
*(helmut.prosch@meduniwien.ac.at)*

**Learning Objectives:**
1. To understand the basic concepts of molecular therapy monitoring.
2. To understand the limitations of molecular therapy monitoring.
3. To learn of non-FDG tracer applications for therapy monitoring.

### RC 206-4
**C. Monitoring immune-therapy: iRECIST and iPERCIST**
Egesta Lopci; Rozzano/IT
*(egesta.lopci@gmail.com)*

**Learning Objectives:**
1. To understand the basic concepts of morphological therapy monitoring of immune therapies.
2. To understand the differences between various response criteria.
3. To understand the limitations of the various response criteria.
**Author Disclosures:**
Egesta Lopci: Grant Recipient: AIRC, Italian Ministry of Health; Speaker: ESMIT, MI&T congressi; Other: Royalties

**Live Q&A: Implications for clinical workflows**

**10:30-11:30 CHANNEL 4**

### ESR meets India

### Meets 2

### Radiology in India

**Moderators**
Michael H. Fuchsjäger; Graz/AT
Chellathurai Amarnath; Chennai/IN

### Meets 2-3
**Temporal lobe epilepsy imaging**
Hemant Patel; Ahmedabad/IN
*(hemantmri@gmail.com)*

**Learning Objectives:**
1. To understand the importance of imaging in partial focal seizures arising from the temporal lobe.
2. To learn the imaging pearls for establishing the diagnosis of mesial temporal sclerosis on MR imaging.
3. To discuss the updates on various advances in MR imaging such as T2 relaxometry, hippocampal volumetry, and PET MRI.

### Meets 2-4
**Interlude 1: Unity in diversity in India**

### Meets 2-5
**Imaging of CNS infection: spectrum of usual and unusual aetiologies**
Deepak P. Patkar; Mumbai/IN
*(drdppatkar@gmail.com)*

**Learning Objectives:**
1. To understand the imaging features of various CNS infections.
2. To understand the role of various imaging modalities.
3. To understand the role of advanced MRI sequences like perfusion and spectroscopy.
4. To understand the pearls and pitfalls of various imaging techniques.
5. To understand imaging findings to differentiate infections from other differentials.

### Meets 2-6
**Interlude 2: A tour of the history of radiology in India**

### Meets 2-7
**Ethics in radiology practice**
Kunnummal Mohanan; Thrissur/IN
*(mohanankdr@gmail.com)*

**Learning Objectives:**
1. To elaborate basic principles of medical ethics applied in radiology.
2. To elaborate ethical principles and diagnostic imaging: over imaging and over diagnosis.
3. To highlight about ethical principles and radiation protection.
4. To inform about ethics in interventional radiology and fetal radiology.
5. To highlight about ethics and innovative approaches in radiology.
6. To highlight ethics and radiology research.
7. To elaborate about ethics and the law.

### Meets 2-8
**Imaging in paediatric neurometabolic disease**
Chellathurai Amarnath; Chennai/IN
*(amarrd02@yahoo.co.in)*

**Learning Objectives:**
1. To review the classification of neurological metabolic diseases.
2. To highlight the important MRI findings in paediatric leucodystrophies.
3. To present some clues for differential diagnosis and an image-based approach.

**Live Q&A**

**10:30-11:30** **CHANNEL 5**

### Refresher Course: Radiographers

### RC 414

### Imaging and protecting the foetus

**Moderators**
Louise A. Rainford; Dublin/IE
Daniela Prayer; Vienna/AT

### RC 414-3

**A. Foetal ultrasound**
David Degiorgio; Msida/MT
*(degio16@gmail.com)*

**Learning Objectives:**
1. To discuss the role of ultrasound in multiple pregnancies.
2. To outline the presentation of normal anatomy and anomalies on ultrasound.
3. To explore the challenges in imaging common foetal pathologies on ultrasound.

### RC 414-4

**B. Foetal MRI**
Christina Malamateniou; London/UK
*(christina.malamateniou@city.ac.uk)*

**Learning Objectives:**
1. To discuss the current clinical applications of foetal MRI.
2. To outline the presentation of normal anatomy and anomalies on MRI.
3. To explore the challenges and optimisation considerations in foetal MRI.

### RC 414-5

**C. Protecting the foetus during emergency radiography**
Shauna Murphy; Dublin/IE
*(shauna.murphy@ucd.ie)*

**Learning Objectives:**
1. To highlight the importance of justification and record-keeping in radiographic pregnant patients in an emergency setting.
2. To discuss the value of shielding the pregnant female in an emergency setting.
3. To present advice on exposure parameter optimisation in radiography of the pregnant female in an emergency setting.

**Live Q&A**

**11:45-12:45** **CHANNEL 2**

### Refresher Course: Abdominal Viscera

### RC 501

### Colon cancer: a multidisciplinary approach

**Moderator**
Lennart K. Blomqvist; Stockholm/SE

### RC 501-2

**A. What are the expectations from the surgeon?**
Dejan Ignjatovic; Oslo/NO
*(dejan.ignjatovic@medisin.uio.no)*

**Learning Objectives:**
1. To learn about the different surgical options for colon cancer.
2. To learn the surgical nomenclature used for the different variants of surgical procedures, in particular that related to the vasculature.
3. To understand how anatomical vascular variants and lymph node involvement on CT can affect the surgical procedure.

### RC 501-3

**B. My CT protocol for staging colon cancer**
Mx J. Lahaye; Amsterdam/NL
*(MJ.Lahaye@gmail.com)*

**Learning Objectives:**
1. To demonstrate the CT acquisition protocol relevant for staging of colon cancer that also allows demonstration of bowel vasculature.
2. To learn about the vascular anatomy of the colon and nodal pathways as visualised on CT.
3. To understand how software post-processing can facilitate assessment and demonstration of vascular anatomy and lymph node involvement.

### RC 501-4

**C. Integrated staging and vascular assessment**
Anne Negaard; Lørenskog/NO
*(anne.negard@medisin.uio.no)*

**Learning Objectives:**
1. To show how a structured report can illustrate local and distant staging in colon cancer.
2. To demonstrate how the vascular assessment is performed in the same examination.
3. To present typical cases that demonstrate the importance of assessment, both in relation to the extent of disease as well as the vascular anatomy.

**Live Q&A**

**11:45-12:45** **CHANNEL 3**

### E³ - The Beauty of Basic Knowledge: Understanding MRI Technique and MRI Safety

### E³ 25A

### Basic principles and magnetic field interactions

**Moderators**
Ursula Reiter; Graz/AT
Siegfried Trattnig; Vienna/AT

### E³ 25A-2

**From the proton to the MR image: the principle of clinical MRI**
Andrew Webb; Leiden/NL
*(a.webb@lumc.nl)*

**Learning Objectives:**
1. To understand how the hydrogen nuclei in our bodies respond to the main magnet, the transmitting RF coil, and the magnetic field gradients.
2. To understand how we form an image and how image artifacts can occur.
3. To understand how to produce different contrasts in an MR image.

### E³ 25A-3

**A systematic overview on MRI interactions with magnetic and electrically conductive material**
Fritz Schick; Tübingen/DE

**Learning Objectives:**
1. To learn about the hardware components that generate different types of electromagnetic fields.
2. To learn about the interactions between the static magnetic field and magnetic material.
3. To learn about the interactions between time-varying electromagnetic fields and electrically conductive material.

**Live Q&A**

**11:45-12:45** **CHANNEL 4**

### Multidisciplinary Session

### MS 2

### Fibrotic lung disease

**Moderator**
Nicola Sverzellati; Parma/IT

### MS 2-2

**Clinical diagnosis and therapeutic options**
Nazia Chaudhuri; Manchester/UK

**Learning Objectives:**
1. To review the key clinical features of individual fibrotic lung disorders.
2. To understand the prognostic and therapeutic implications of the differential diagnosis among fibrotic lung disorders.
3. To suggest appropriate pharmacologic therapy for patients with individual fibrotic lung disorders.

**Author Disclosures:**
Nazia Chaudhuri: Advisory Board: Boehringer Ingelheim, Roche Ltd, Novartis; Research Grant/Support: BI

### MS 2-3

**Radiological and pathological diagnosis of diffuse fibrotic lung disease (part 1)**
Melanie Greaves; Manchester/UK
*(melanie.greaves@mft.nhs.uk)*

**Learning Objectives:**
1. To learn about the key CT and histologic features of fibrotic lung diseases.
2. To understand when clinic-radiological data is sufficient to make a correct diagnosis and when additional lung biopsy is required.
3. To understand the value of multidisciplinary teams in the diagnosis and management of patients with these conditions.

### MS 2-4

**Radiological and pathological diagnosis of diffuse fibrotic lung disease (part 2)**
M. Angeles Montero-Fernandez; Manchester/UK
*(amonterofer@hotmail.com)*

**Learning Objectives:**
1. To learn about the key CT and histologic features of fibrotic lung diseases.
2. To understand when clinic-radiological data is sufficient to make a correct diagnosis and when additional lung biopsy is required.
3. To understand the value of multidisciplinary teams in the diagnosis and management of patients with these conditions.

### MS 2-5

**Multidisciplinary case presentation**
Nicola Sverzellati; Parma/IT

**Live Q&A**

**11:45-12:45** **CHANNEL 5**

### Research Presentation Session: Neuro

### RPS 111

### Advances in neurovascular imaging for stroke management

**Moderator**
Aad van der Lugt; Rotterdam/NL

### RPS 111-2

**Comparison between two commercial MR perfusion software systems for mechanical thrombectomy patient selection**
*S. Pistocchi*, D. Strambo, B. Bartolini, R. A. Meuli, P. Michel, V. Dunet; Lausanne/CH
*(silvia.pistocchi@chuv.ch)*

**Purpose:** Performance of different MR perfusion software in assessing and selecting patients with acute stroke and large vessel occlusion (LVO) for mechanical thrombectomy is unclear. We compared two commercial MRI softwares, Carestream in automated (package A) and semi-automated (package B) manner and Rapid (package C), for estimating core and hypoperfusion volumes, and its influence on patients' treatment selection and outcome.
**Methods or Background:** 144 MRI from patients with anterior circulation LVO were retrospectively analyzed. All images were acquired on a Siemens VIDA 3T system and post-processed with the three packages. Standard thresholds were applied (core=ADC<620x10-6 mm2/sec; hypoperfusion=Tmax>6sec; mismatch=hypoperfusion/core). Concordance for core and hypoperfusion volumes was assessed with the Lin's test. Agreement for mismatch-based patients' classification was assessed with the Gwet's AC1 and clinical outcome was compared between groups in patients who underwent late thrombectomy.
**Results or Findings:** Mean core volume was higher in package C than in packages A and B (36.8±52.2 ml versus 22.7±38.7 ml and 19.1±35.3 ml, respectively), while mean hypoperfusion volume was lower in package C than in packages A and B (84.5±74.0 ml versus 160.3±118.9 ml and 117.9±78.4 ml, respectively). Mismatch ratio was above 1.8 in 89/142 (62.7%) using package C and in 130/142 (91.5%) patients using packages A-B, resulting in a moderate agreement (Gwet's AC1: 0.55 between C and A-B). Of 42 patients who underwent a late thrombectomy with successful recanalization (TICI 2b/3), 16 (38%) had a malignant mismatch profile using package C versus 2 (0.5%) and 3 (0.7%) using packages A and B. However, those 16 patients had a similar benefit than patients with a favorable mismatch profile.
**Conclusion:** MRI softwares for diffusion/perfusion analysis are not interchangeable and may influence patients' selection and outcome.
**Limitations:** Monocentric, retrospective.
**Ethics Committee Approval:** Approved.
**Funding for this study:** Not applicable.
**Author Disclosures:**
Bruno Bartolini: Nothing to disclose
Vincent Dunet: Nothing to disclose
Reto Antoine Meuli: Nothing to disclose
Patrik Michel: Nothing to disclose
Silvia Pistocchi: Nothing to disclose
Davide Strambo: Nothing to disclose

### RPS 111-3

**Carotid plaque characteristics predict recurrent ischemic stroke - The Plaque At RISK study: a prospective multicenter study to identify high-risk carotid plaques**
*D. H. K. Dam-Nolen*[1], M. Kassem[2], A. G. van der Kolk[3], M. I. Liem[4], D. Bos[1], P. J. Nederkoorn[4], J. Hendrikse[3], A. Van Der Lugt[1], E. Kooi[2]; [1]Rotterdam/NL, [2]Maastricht/NL, [3]Utrecht/NL, [4]Amsterdam/NL
*(h.nolen@erasmusmc.nl)*

**Purpose:** Stroke patients with symptomatic carotid artery stenosis are at high risk for recurrent stroke. Currently, treatment decisions for carotid revascularization are mainly based on degree of stenosis. However, other plaque characteristics might be better indicators of recurrent stroke, allowing for more targeted intervention. The Plaque At RISK (PARISK) study aimed to investigate which carotid plaque characteristics improve the identification of patients with increased risk of recurrent ischemic stroke.
**Methods or Background:** We included 240 patients with symptomatic 30-69% ipsilateral carotid artery stenosis in the PARISK-study, a prospective multicenter cohort study. MRI (carotid and brain) and MDCTA (carotid) were performed at baseline and after 2 years. Participants were followed for 5 years. Primary endpoint was a recurrent ipsilateral ischemic stroke or TIA. Secondary endpoint was the composite of recurrent ipsilateral ischemic stroke or TIA, and/or new ipsilateral ischemic brain lesions on follow-up MRI. Multivariable Cox-proportional hazard models were used to assess associations of intraplaque hemorrhage (IPH), ulceration, calcification percentage, and total plaque volume with the outcomes. Next, we investigated the predictive performance of these plaque characteristics by adding (combinations of) these markers to the clinically used Carotid Artery Risk (CAR) score.
**Results or Findings:** IPH presence and total plaque volume were associated with the primary (HR=3.32, 95%CI:1.11-9.91 respectively HR=1.18, 95%CI:1.03-1.35) and secondary outcome (HR=3.33, 95%CI:1.44-7.69 respectively HR=1.13, 95%CI:1.01-1.26). Ulcerations and calcification percentage were not significantly associated with the outcomes. Addition of IPH and total plaque volume to the CAR-score improved the model for the primary and secondary outcome (C-statistic increased from 0.64-0.66 to 0.70-0.78).
**Conclusion:** IPH and total plaque volume are independent risk factors for recurrent ischemic stroke or TIA in symptomatic patients with mild-to-moderate carotid artery stenosis.
**Limitations:** n/a
**Ethics Committee Approval:** Yes
**Funding for this study:** Center for Translational Molecular Medicine, Dutch Heart Foundation

**Author Disclosures:**
Aad Van Der Lugt: Nothing to disclose
Anja G. van der Kolk: Nothing to disclose
Madieke I. Liem: Nothing to disclose
Dianne H. K. van Dam-Nolen: Nothing to disclose
Paul J. Nederkoorn: Nothing to disclose
Eline Kooi: Nothing to disclose
Daniel Bos: Nothing to disclose
Jeroen Hendrikse: Nothing to disclose
Mohamed Kassem: Nothing to disclose

### RPS 111-6

**Recanalisation results of endovascular TRAP technique in acute ischaemic stroke patients**

*M. Voormolen*, M. C. Niekel, T. van der Zijden, O. d'Archambeau, T. Jardinet, T. Van Thielen, L. Yperzeele, C. Loos, A. Mondelaers; Antwerp/BE
*(maurits.voormolen@uza.be)*

**Purpose:** Improvement of endovascular recanalization procedure in patients with acute ischaemic stroke might result in better clinical outcome. Combination of stent retriever TRombectomy and catheter Aspiration during Proximal flow rest by balloonguide catheter (TRAP technique) might increase recanalisation rate with more complete recanalization, possibly resulting in better clinical outcome. Our goal was to assess safety and efficacy of endovascular recanalisation by TRAP technique in patients with acute intracranial thrombo-embolic occlusions in the anterior circulation.
**Methods or Background:** Between January 2018 and July 2020, in our hospital 185 TRAP endovascular recanalisation procedures as first pass attempt were performed in patients with acute ischaemic stroke of anterior circulation, involving 97 female (52%) and 88 male patients. Mean patient age was 70 years (range 32-92). Mean NIHSS was 17 (range 6-25). Occluded arteries were distal internal carotid (27%), M1 (54%), M2 (10%) and tandem lesions (9%).
**Results or Findings:** Recanalisation by TRAP was achieved in 170 procedures (92%). First pass recanalisation rate was 62%. Mean number of passages was 1,5 (range 1-4). Complete (TICI 3; 53%) and near complete (TICI 2B; 36%) was seen in 89%, fair recanalisation (TICI 2A) in 3% and no recanalisation (TICI 1 & 0) in 8%. Median time from groin puncture to recanalisation was 33 minutes (range 16-120). Six procedure related complications occurred (3%). Median NIHSS at 24 hours was 4 (range 1-20). Median mRS at 3 months after stroke was 2 (range 0-6).
**Conclusion:** Endovascular recanalisation with stent retriever TRombectomy and Aspiration catheter during Proximal flow rest (TRAP) in patients with acute anterior circulation ischaemic stroke is safe and shows high rates of (near-)complete recanalization.
**Limitations:** Single center not-randomised study; retrospective analysis.
**Ethics Committee Approval:** No ethics committee approval.
**Funding for this study:** No funding for this study.
**Author Disclosures:**
Maurits Voormolen: Nothing to disclose
Caroline Loos: Nothing to disclose
Annelies Mondelaers: Nothing to disclose
Maarten Christian Niekel: Nothing to disclose
Olivier d'Archambeau: Nothing to disclose
LAetitia Yperzeele: Nothing to disclose
Thomas Jardinet: Nothing to disclose
Thomas Van Thielen: Nothing to disclose
Thijs van der Zijden: Nothing to disclose

### RPS 111-7

**Semiquantitative assessment of iodine extravasation in acute ischaemic stroke after mechanical thrombectomy**

*X. Gao*, Q. Hui, B. Ming, C. C. Ma; Deyang/CN
*(724161276@qq.com)*

**Purpose:** This study aimed to use two easy and practical semiquantitative methods to assess iodine extravasation and its association with haemorrhage.
**Methods or Background:** Seventy-two patients with anterior artery obstruction were recruited. All patients underwent dual-energy CT scan immediately after mechanical thrombectomy and CT or MRI scan at follow-up. We divided these patients into two groups. Two semiquantitative methods were used to evaluate the simulated conventional CT. First, we evaluated the iodine extravasation areas by using the modified Alberta Stroke Program Early CT Score (mASPECTS). Then, we assessed the degree of iodine extravasation. Several clinical variables were collected. Student's T-test, $\chi^2$/Fisher's exact test or Kruskal-Wallis analysis was performed. A logistic regression analysis and receiver operating characteristics curve analysis were performed.
**Results or Findings:** There were 29 stroke patients having early or late haemorrhage and 43 patients without haemorrhage transformation. There were significant differences between the two groups in mRS scores (P=0.018), mASPECTS (P< 0.001) and the sign of dense iodine extravasation (P< 0.001). The multivariate logistic regression analysis showed that dense iodine extravasation (P=0.024) was significantly associated with haemorrhage transformation. The AUCs of the mASPECTS, score for dense iodine extravasation and their combination were 0.833, 0.839 and 0.850, respectively. The cut-off for mASPECTS was 6 (sensitivity of 75.86% and specificity of 81.40%). The sign of dense iodine extravasation in stroke patients for predicting haemorrhage showed a sensitivity of 72.41% and a specificity of 88.37%.
**Conclusion:** For patients after successful mechanical thrombectomy, those with a mASPECTS ≤6 and/or positive sign of dense iodine extravasation are prone to develop haemorrhage and are associated with poor outcomes.
**Limitations:** The sample is small.
**Ethics Committee Approval:** Registration number: ChiCTR1800015971
**Funding for this study:** Non.
**Author Disclosures:**
Qingtao Hui: Nothing to disclose
Bin Ming: Nothing to disclose
Chun Chun Ma: Nothing to disclose
Xin Gao: Nothing to disclose

### RPS 111-8

**The correlation between the value of ultra-high-b-value DWI and glymphatic system function after ischaemic stroke**

*L. Lin*, C. F. Sun, X. Hao, C. Li, Y. M. Yang; Shanghai/CN
*(linly_1995@163.com)*

**Purpose:** It is believed that intracisternal dynamic contrast-enhanced MRI is an efficient, in-vivo method to evaluate glymphatic system function of whole brain. In addition, ultra-high-b-value DWI (UHBV-DWI) is speculated to display aquaporins function in some neural diseases, which is an important part in glymphatic system. We aim at demonstrating whether UHBV-DWI could evaluate glymphatic system function in ischaemic stroke.
**Methods or Background:** Five Sprague-Dawley rats subjected to 90-min left middle cerebral artery occlusion were scanned by UHBV-DWI (b=2000,2500,3000,3500,4000,4500 s/mm2) and intracisternal Gd-DTPA enhanced MRI two weeks after surgery. ADC value were computed by the mono-exponential equation using GE Functool in GE Advanced Workstation. For immunohistological studies, mouse anti-AQP4 (Servicebio, GB12529), rabbit anti-GFAP (Abcam, ab33922) primary antibodies and suitable secondary antibodies were used.
**Results or Findings:** We found that T1 signal intensity of peri-lesion area (2510.00±227.26) was significantly higher than that of core-lesion area (1333.25±354.99) and that of contralateral-area (1560±137.06) at 24-hour after intracisternal Gd-DTPA injection, which meant contrast agent retention in this peri-ischaemic area. Secondly, there were obviously disordered, depolarized AQP4 and higher ADC value of UHBV-DWI in the same peri-lesion area compared with contralateral side.
**Conclusion:** It concluded that delayed clearance of waste solution by glymphatic system in peri-ischaemic area that may associated with disordered, depolarized AQP4. Moreover, UHBV-DWI as a non-invasive method could evaluate glymphatic system function after ischaemic stroke.
**Limitations:** Further experiments are needed to study mechanisms of the association between ADC value of UHBV-DWI and glymphatic system function.
**Ethics Committee Approval:** Approved by Ethics Committee of Huashan Hospital, Fudan University.
**Funding for this study:** National Natural Science Foundation (No. 81771788).
**Author Disclosures:**
Chanchan Li: Nothing to disclose
Xiaozhu Hao: Nothing to disclose
Cheng Feng Sun: Nothing to disclose
Luyi Lin: Nothing to disclose
Yan Mei Yang: Nothing to disclose

**Live Q&A**

**14:15-15:15 CHANNEL 2**

### Special Focus Session

### SF 9

### My three top tips for breast imaging

**Moderator**
Isabelle Thomassin-Naggara; Paris/FR

### SF 9-2

**Screening with tomosynthesis**

Sophia Zackrisson; Malmö/SE
*(sophia.zackrisson@med.lu.se)*

**Learning Objectives:**
1. To understand why tomosynthesis is better than mammography for breast cancer screening.
2. To appreciate the current scientific evidence of tomosynthesis in screening.
3. To acknowledge what further steps are needed before implementation in screening.

**Author Disclosures:**
Sophia Zackrisson: Patent Holder: US patent no PCT/EP2014/057372; Speaker: Siemens Healthcare AG

### SF 9-3
**Automated breast ultrasound**
Ritse M. Mann; Nijmegen/NL
*(r.mann@rad.umcn.nl)*

**Learning Objectives:**
1. To recognise artefacts specific to automated breast ultrasound (ABUS).
2. To appreciate the value of multiplanar reconstruction and the coronal spiculation pattern.
3. To gain insight into possible indications for ABUS.

**Author Disclosures:**
Ritse M. Mann: Other: please copy from other

### SF 9-4
**Complex cystic and solid lesions**
Panagiotis Kapetas; Vienna/AT
*(panagiotis.kapetas@meduniwien.ac.at)*

**Learning Objectives:**
1. To understand the difference between complex cystic and solid lesions from other cystic lesions of the breast.
2. To become familiar with complementary sonographic techniques for the accurate characterisation of cystic breast lesions.
3. To be able to properly manage complex cystic and solid lesions of the breast.

### SF 9-5
**Imaging the axilla**
Isabelle Thomassin-Naggara; Paris/FR
*(isabelle.thomassin@tnn.aphp.fr)*

**Learning Objectives:**
1. To understand the clinical role of axillary staging.
2. To learn the imaging features of abnormal lymph nodes and the criteria for biopsy.
3. To understand the importance of discriminating minimal vs advanced nodal disease.

**Author Disclosures:**
Isabelle Thomassin-Naggara: Speaker: GE, canon, guerbet, hologic

### SF 9-6
**Contrast-enhanced spectral mammography**
Eva M. Fallenberg; Munich/DE

**Learning Objectives:**
1. To understand the technical principles of contrast-enhanced spectral mammography.
2. To understand the added value of information provided by contrast-enhanced spectral mammography.
3. To become familiar with the information provided by contrast-enhanced spectral mammography and breast MRI regarding lesion morphology and enhancement.

**Author Disclosures:**
Eva M. Fallenberg: Advisory Board: GE Bayer Siemens Guerbet; Research Grant/Support: DFG Guerbet GE ; Speaker: GE Siemens Guerbet Siemens

### SF 9-7
**Stereotactic-guided biopsy**
Elisabetta Giannotti; Nottingham/UK

**Learning Objectives:**
1. To understand the indications for stereotactic-guided breast biopsy.
2. To learn about the technical aspects of stereotactic biopsy.
3. To understand the importance of marker clip placement post-biopsy.

### SF 9-8
**US-guided biopsy**
Alexandra Athanasiou; Athens/GR
*(aathanasiou@mitera.gr)*

**Learning Objectives:**
1. To understand current indications, contraindications, and possible complications of US-guided biopsy.
2. To be familiar with different available biopsy systems and to know which one to choose according to the clinical setting.
3. To learn the most important technical tips for performing a US-guided biopsy successfully.

### SF 9-9
**MRI-guided biopsy**
Gul Esen; Istanbul/TR
*(gulesenicten@gmail.com)*

**Learning Objectives:**
1. To understand the importance of pre-biopsy preparation including patient information.
2. To be familiar with how to relate lesion and needle positions.
3. To appreciate tricks for targeting lesions in complicated locations (superficial, deep, retroareolar).

### SF 9-10
**Treatment response and therapy monitoring**
Chantal Van Ongeval; Leuven/BE
*(chantal.vanongeval@uzleuven.be)*

**Learning Objectives:**
1. To learn what is important in the reporting of the treatment response.
2. To understand the accuracy of mammography, ultrasound, and magnetic resonance in the monitoring of therapy.

### SF 9-11
**Post-therapy evaluation**
Julia Camps Herrero; Valencia/ES
*(jcamps@riberasalud.es)*

**Learning Objectives:**
1. To understand and learn the different phases of fat necrosis and its imaging correlates in all modalities.
2. To know what to report in patients with breast implants and oncoplastic reconstructions.
3. To learn the different appearances of breast cancer recurrence.

**Author Disclosures:**
Julia Camps Herrero: Advisory Board: Bayer, Becton-Dickinson

**Live Q&A**

**14:15-15:15** **CHANNEL 3**

### E³ - ECR Master Class

### E³ 326
### Cardiac imaging in arrhythmia and sudden cardiac death

**Moderator**
Paulo Donato; Coimbra/PT

### E³ 326-2
**A. The role of cardiovascular magnetic resonance (CMR) in sudden cardiac death**
Hubert Cochet; Bordeaux/FR

**Learning Objectives:**
1. To become familiar with the pathophysiology of sudden cardiac death.
2. To introduce CMR biomarkers to prevent sudden cardiac death.
3. To learn how to perform and how to interpret CMR to prevent sudden cardiac death.

### E³ 326-3
**B. Preventing sudden cardiac death with CT: pure theory or new diagnostic paradigm?**
Katarzyna Gruszczynska; Katowice/PL

**Learning Objectives:**
1. To learn what to look for on CT to prevent sudden cardiac death.
2. To receive an overview of the existing evidence for performing cardiac CT in this indication.
3. To discuss the possible role of cardiac CT in preventing sudden cardiac death.

### E³ 326-4
**C. Imaging to drive electrophysiology procedures**
Antonio Esposito; Milan/IT
*(esposito.antonio@hsr.it)*

**Learning Objectives:**
1. To learn about the importance of early identification of potentially lethal arrhythmias.
2. To become familiar with the tips and tricks for successful CT/CMR in patients with arrhythmia.
3. To understand the role of imagingin the planning of electrophysiological procedures.
**Author Disclosures:**
Antonio Esposito: Speaker: Bayer

**Live Q&A: Should we screen, who should we screen, and how should we screen in order to prevent sudden cardiac death?**

**14:15-15:15 CHANNEL 5**

### Coffee & Talk (open forum) Session
*Organised by EIBIR*

### C 13
### Joint EIBIR-EURAMED session on European radiation protection research

**Moderator**
Christoph Hoeschen; Magdeburg/DE

### C 13-2
**Talk from the MEDIRAD project: understanding and evaluating the health effects of low-dose ionising radiation exposure from diagnostic and therapeutic imaging**
Guy Frija; Paris/FR

**Learning Objectives:**
1. To learn about the MEDIRAD research project.
2. To understand the risks of low-dose ionising radiation exposure.

### C 13-3
**Talk from the SINFONIA project: developing comprehensive risk appraisal for the detrimental effects of radiation exposure on patients and healthcare professionals (during the management of lymphoma or brain tumour patients)**
John Damilakis; Iraklion/GR

**Learning Objectives:**
1. To learn about the SINFONIA research project.
2. To learn about risk appraisal for radiation exposure.

### C 13-4
**Talk from the EURAMED rocc-n-roll project: developing a strategic research agenda for medical applications of ionising radiation and related radiation protection**
Christoph Hoeschen; Magdeburg/DE
*(christoph.hoeschen@ovgu.de)*

**Learning Objectives:**
1. To learn about the EURAMED rocc-and-roll project.
2. To appreciate the European radiation protection research efforts.

**Live Q&A**

**15:30-16:30 CHANNEL 2**

### E³ - ECR Master Class

### E³ 1526
### Cone-beam, 4D, and more: new diagnostic tools for vascular diseases

**Moderator**
Tobias F. Jakobs; Munich/DE

### E³ 1526-2
**A. The role of intraprocedural perfusion assessment in peripheral arterial disease**
Jim A. Reekers; Amsterdam/NL
*(jimreekers@xs4all.nl)*

**Learning Objectives:**
1. To understand the technique of intraprocedural perfusion assessment.
2. To learn how to target peripheral revascularisation therapy based on perfusion assessment.
3. To discuss the value of using intraprocedural perfusion assessment on the outcome of endovascular therapy for peripheral artery disease.
**Author Disclosures:**
Jim A. Reekers: Consultant: Philips medical

### E³ 1526-3
**B. CT 4D imaging after endovascular aortic repair (EVAR)**
Rüdiger Schernthaner; Vienna/AT
*(Ruediger.schernthaner@meduniwien.ac.at)*

**Learning Objectives:**
1. To understand the challenges of imaging follow-up after TEVAR.
2. To become familiar with the technique of 4D CT.
3. To learn how to establish treatment recommendations based on 4D CT results after TEVAR.
**Author Disclosures:**
Rüdiger Schernthaner: Research Grant/Support: Siemens Healthineers

### E³ 1526-4
**C. How cone-beam CT can change your practice in interventional radiology**
Raman Uberoi; Oxford/UK

**Learning Objectives:**
1. To understand the technique of cone-beam CT.
2. To learn about the role and applications of the cone-beam in the angio suite.
3. To discuss the influence on daily clinical practice in interventional radiology.

**Live Q&A**

**15:30-16:30 CHANNEL 4**

### Special Focus Session

### SF 5
### How to read a chest radiograph

**Moderator**
Mariaelena Occhipinti; Florence/IT

### SF 5-2
**The lungs**
Miroslav Herman; Olomouc/CZ
*(miroslav.herman@fnol.cz)*

**Learning Objectives:**
1. To review the approach to focal and diffuse lung diseases at chest radiography.
2. To appreciate the chest radiograph limitations for focal and diffuse lung diseases.
3. To demonstrate possible mistakes.

**SF 5-3**
**The hila and pulmonary vasculature**
Arjun Nair; London/UK

**Learning Objectives:**
1. To appreciate the radiographic features of the structures forming the hila.
2. To review the differences between pulmonary arteries and veins.
3. To examine patterns of distribution of pulmonary flow.

**Author Disclosures:**
Arjun Nair: Advisory Board: Aidence BV; Other: Part funded by BRC UCLH

**SF 5-4**
**The heart and mediastinum**
José Vilar; Valencia/ES

**Learning Objectives:**
1. To review the normal mediastinal lines.
2. To learn about the normal and abnormal mediastinal contour.
3. To focus on the cardio-pericardial silhouette.

**SF 5-5**
**The pleura and chest wall**
Eduardo Jose Mortani Barbosa Jr.; Philadelphia, PA/US

**Learning Objectives:**
1. To learn about the signs of air and fluid accumulation in the pleural space.
2. To appreciate how to distinguish pleural from extra-pleural lesions.
3. To review the most common chest wall lesions.

**Author Disclosures:**
Eduardo Jose Mortani Barbosa Jr.: Research Grant/Support: Siemens Healthineers

**Live Q&A: What not to miss in chest radiography?**

**15:30-16:30** **CHANNEL 5**

### Research Presentation Session: Oncologic Imaging

### RPS 116

### Novel imaging biomarkers for assessment, treatment response, and outcome prognostication of cancers

**Moderator**
Lale Umutlu; Essen/DE

**RPS 116-2**
**Gadoxetic acid MRI for the assessment of HCC response to Yttrium 90 radioembolization: correlation with histopathology**
*N. Vietti Violi*[1], J. Gnerre[2], S. Hectors[2], O. Bane[2], A. Law[2], I. Fiel[2], B. Taouli[2]; [1]Lausanne/CH, [2]New York, NY/US
*(nviettivioli@gmail.com)*

**Purpose:** To assess the diagnostic performance of gadoxetate-enhanced MRI for predicting complete pathologic necrosis (CPN) of hepatocellular carcinoma (HCC) after Yttrium 90 transarterial radioembolization (TARE), using histopathology as the reference.
**Methods or Background:** This retrospective study included 48 consecutive patients (M/F:36/12, mean age:62y) with HCC treated by TARE followed by surgery (liver resection/transplantation:14/34). All patients underwent gadoxetate MRI ≤90days of surgery. Two independent radiologists evaluated the following criteria: % of necrosis on subtraction images obtained during late arterial, portal venous and hepatobiliary phases, mRECIST, LI-RADS and apparent diffusion coefficient (ADC). Data were correlated to % necrosis on pathology. Inter-reader agreement for radiologic % necrosis was assessed using interclass correlation coefficient (ICC). ROC and DeLong analyses were used to determine and compare prediction of CPN. Correlation between radiologic and pathologic % necrosis was assessed using Pearson correlation.
**Results or Findings:** Histopathology demonstrated 71 HCCs (mean size:2.8±1.7cm) including 42 with CPN, 22 with partial necrosis and 7 with no necrosis. There was excellent inter-reader agreement for assessing radiologic degree of necrosis (ICC:0.82-0.89). Percentage of tumor necrosis, mRECIST, LI-RADS were all significant (p <0.0001) predictors of CPN (AUC:0.80-0.82 for radiologic % necrosis and AUC:0.71-0.73 for mRECIST and LI-RADS), with a significant difference observed between subtraction and LI-RADS (p=0.04) for reader 2. ADC was not a significant predictor of CPN (AUC:0.63). Radiologic % necrosis was significantly correlated to histopathologic degree of tumor necrosis (r=0.66-0.8, p<0.0001).
**Conclusion:** Image subtraction, LI-RADS and mRECIST are all significant predictors of CPN in HCC treated with TARE, with superiority of subtraction over LI-RADS for one reader. Excellent correlation was found between radiologic and pathologic percentages of necrosis.
**Limitations:** This study is limited by its relatively small sample size.
**Ethics Committee Approval:** This study was approved by local ethical committee.
**Funding for this study:** No funding
**Author Disclosures:**
Amy Law: Nothing to disclose
Mre Octavia Bane: Nothing to disclose
Isabel Fiel: Nothing to disclose
Naik Vietti Violi: Nothing to disclose
Stephanie Hectors: Nothing to disclose
Bachir Taouli: Nothing to disclose
Jeffrey Gnerre: Nothing to disclose

**RPS 116-3**
**Metabolic and transcriptomic biomarkers to predict response to neoadjuvant chemotherapy and survival in oesophageal adenocarcinoma**
*K. G. Foley*[1], A. Lavery[2], E. Napier[2], D. Campbell[2], M. Eatock[2], R. Kennedy[3], K. Bradley[4], R. Turkington[2]; [1]Llantrisant/UK, [2]Belfast/UK, [3]Craigavon/UK, [4]Cardiff/UK
*(foleykg@cardiff.ac.uk)*

**Purpose:** 18F-fluorodeoxyglucose PET-CT may guide treatment decisions in patients with oesophageal adenocarcinoma (OAC). This study evaluated the added value of maximum standardised uptake value (SUVmax) to a novel DNA-damage immune repair (DDIR) assay to improve pathological response prediction. The diagnostic accuracy of PET response and the prognostic significance of PET metrics for recurrence-free survival (RFS) and overall survival (OS) were assessed.
**Methods or Background:** Retrospective, single-centre study of OAC patients treated with neoadjuvant chemotherapy from 2003-2014. SUVmax was recorded from baseline and repeat PET-CT after completion of pre-operative chemotherapy. Logistic regression models tested the additional predictive value of PET metrics combined with the DDIR assay for pathological response. Cox regression models tested the prognostic significance of PET metrics for RFS and OS.
**Results or Findings:** 113 patients were included; 25 (22.1%) were DDIR positive and 88 (77.9%) were DDIR negative. 69 (61.1%) were PET responders (SUVmax reduction of 35%) and 44 (38.9%) were PET non-responders. After adding PET metrics to DDIR status, post-chemotherapy SUVmax (hazard ratio (HR)=0.75, p=0.02), SUVmax change (HR=1.04, p=0.003) and an optimum SUVmax reduction of 46.5% (HR=4.36, p=0.021) showed additional value for predicting pathological response. The optimised SUVmax threshold was independently significant for RFS (HR=0.47, 95%CI 0.26-0.85, p=0.012) and OS (HR=0.51, 95%CI 0.26-0.99, p=0.047).
**Conclusion:** This study demonstrated the additional value of PET metrics, when combined with a novel DDIR assay, to predict pathological response in OAC patients treated with neoadjuvant chemotherapy. Furthermore, an optimised SUVmax reduction threshold for pathological response was calculated and was independently significant for RFS and OS.
**Limitations:** Retrospective, single-centre study
**Ethics Committee Approval:** Northern Ireland Biobank (NIB12-0032) Research Ethics Committees Northern Ireland (ORECNI: 13/NI/0149).
**Funding for this study:** The Gastrointestinal Cancer Research Charitable Fund, The Cancer Research UK Experimental Cancer Medicine Centre Initiative, Invest Northern Ireland, Almac Diagnostics
**Author Disclosures:**
David Campbell: Nothing to disclose
Martin Eatock: Nothing to disclose
Eoin Napier: Nothing to disclose
Kevin Bradley: Nothing to disclose
Anita Lavery: Nothing to disclose
Kieran George Foley: Nothing to disclose
Richard Kennedy: Patent Holder at Almac Diagnostics, Employee at Almac Diagnostics
Richard Turkington: Research/Grant Support at Almac Diagnostics, Patent Holder at Queens University Belfast

### RPS 116-4

**68Ga-PSMA PET-CT for primary staging of prostate cancer and use of SUV Max as metastasis predictor**

*C. Gatica*, G. Chong, D. Barahona, D. Hasson, C. Silva; Santiago/CL
*(esperanza.troncoso@gmail.com)*

**Purpose:** The present study is based on a retrospective analysis of Gallium-68-labelled prostate-specific membrane antigen PET-CT (PET PSMA) performed in newly diagnosed, treatment-naïve prostate cancer (PCa) patients prior to definitive treatment.
To correlate the SUV Max of the primary lesion and the rest of the staging parameters.
**Methods or Background:** A retrospective study was conducted including patients with PCa staged with PSMA PET between 2014 and 2019 at our institution. The prostate specific antigen (PSA) and Gleason scores (GSC) values obtained from transrectal biopsies were collected, categorizing the patients by risk according to the D´Amico criteria.The SUV Max of the dominant prostate lesion for each patient and tumor spread were recorded.
**Results or Findings:** 231 patients were included; 19 low risk, 105 intermediate and 107 high risk. 56 patients presented regional pelvic lymphatic involvement, 23 supra-pelvic lymphatic and 23 visceral and bone metastases. A significant difference was demonstrated in the medians of SUV Max per risk group ($p < 0.001$).
The ROC curve analysis reveals an area under the curve of 0.79 for the detection of bone metastases, and using a cut-off point of SUV Max of 16, a sensitivity of 0.82 and a specificity of 0.70 are obtained.
**Conclusion:** In patients with initial diagnosis of prostate cancer, 68Ga-PSMA PET CT is a relevant staging procedure by identifying nodal and/or distant metastasis.
The SUV Max correlates with the probability of finding metastasis, which, together with PSA, could guide the biological behavior of the primary tumor and the need for more strict follow up.
**Limitations:** Retrospective study
**Ethics Committee Approval:** Not required
**Funding for this study:** Not required
**Author Disclosures:**
Daniela Barahona: Nothing to disclose
Daniel Hasson: Nothing to disclose
Guillermo Chong: Nothing to disclose
Consuelo Gatica: Nothing to disclose
Claudio Silva: Nothing to disclose

### RPS 116-5

**Gadolinium-loaded calcium phosphate nanoparticles for magnetic resonance imaging of primary hepatocellular carcinoma**

*N. Zhang*[1], C. Lu[1], Y. Du[2], J. Ji[1]; [1]Lishui/CN, [2]Hangzhou/CN
*(znn199109@126.com)*

**Purpose:** HCC is one of the most common diseases threatening human health worldwide. Timely diagnosis is extremely important. The aim of this study was to develop a novel MRI contrast agent for the targeting delivery of contrast to the tumor position.
**Methods or Background:** The MR imaging efficacy of A54-CaPNPs was conducted on the primary HCC models.
**Results or Findings:** A54-CaPNPs showed a uniform distribution with a mean diameter of about 30 nm (Fig.1). It exhibited higher longitudinal relaxivity (6.07 $mM^{-1} s^{-1}$) than Gd-DTPA (3.56 $mM^{-1} s^{-1}$) (Fig.1). The cellular uptake of the nanoparticles was in time-dependent manner. The fluorescence intensity of Bel-7402 cells incubated with A54-CaP/DOX were stronger than those of HepG2 cells incubated A54-CaP/DOX or CaP/DOX (Fig.2). The MR imaging efficiency of A54-CaPNPs was studied in primary HCC models. The mice treated with A54-CaPNPs exhibited an obviously bright contrast enhancement at the tumor position. The quantitative data suggested that the SNR of the tumor region began to increase after the injection of A54-CaPNPs, reaching more than 1.99-fold enhancement at 1 h post injection, which was higher than that of the Gd-DTPA group (Fig. 3B).
**Conclusion:** The prepared A54-CaPNPs may be a promising candidate as an MRI contrast agent for hepatic cancer diagnosis.
**Limitations:** This research is only carried out at the cellular and animal level, and the actual efficacy is difficult to confirm.
**Ethics Committee Approval:** All the animal studies were performed in accordance with the guide- lines of the Ethical Committee of Zhejiang University. The sur- gical procedures and experiment protocols were approved by the Committee for Animal Experiments of Zhejiang University.
**Funding for this study:** This study was supported by the National Natural Science Foundation of China (No. 81901852, 81901848) and Experimental Animal Science and Technology Projects of Zhejiang Province (No. 2017C37178).
**Author Disclosures:**
Yongzhong Du: Nothing to disclose
Jiansong Ji: Nothing to disclose
Nannan Zhang: Nothing to disclose
Chenying Lu: Nothing to disclose

### RPS 116-6

**Prospective study in patients with high-grade soft tissue sarcomas of extremities and trunk wall undergoing neoadjuvant treatment assessed with MRI: correlation between ADC and pathologic response**

*C. Buonomenna*, R. Vigorito, A. Casale, A. Messina, C. Morosi, A. V. Marchianò; Milan/IT
*(buonomenna.ciriaco@gmail.com)*

**Purpose:** To correlate preoperative apparent diffusion coefficient (ADC) mean value with pathologic response, particularly with residual viable tumor, in adult patients with high-risk primary, localized soft-tissue sarcomas treated with neoadjuvant chemoradiation therapy followed by surgery in a prospective randomized study
**Methods or Background:** We evaluated 60 Patients with MRI at baseline (ADC0), after one cycle of chemotherapy (ADC1) and before surgery (ADC3). Patients were studied at National Cancer Institute of Milan, with the same MR machine and same protocol. Mean ADC value was obtained by manually drawing a ROI around the margin of the whole lesion, on sections taken every 5 mm, using Philips Intellispace software. The percentage of residual viable tumor was assessed on the surgical specimen by two pathologists.
**Results or Findings:** We demonstrated a statistically significant increase of ADC values between the baseline and after therapy in all group of Patients (mean ADC0: 1.58 ± 0.37; ADC1= 1.82 ± 0.41; ADC3= 1.98 ± 0.38).
The correlation between pathological variables and ADC3 was analyzed: ADC3 was closely related to the percentage of residual viable tumor in an inversely proportional manner (Spearman's coefficient: -0.43) and to the percentage of sclerohyalinosis, which is an histological pattern of response to therapy, in a directly proportional way (Spearman's coefficient: +0.55).
The association between radiological response and ADC3 was studied: there was a significant association between ADC3 and Choi criteria (p-value: 0.036)
**Conclusion:** In our study average ADC value has proven useful for the assessment of tumor cellularity in soft-tissue sarcomas, particularly of residual viable tumor after therapy.
In clinical practice cellular changes precede morphologic changes so DWI may help to detect treatment response earlier than conventional imaging
**Limitations:** The number of patients was relatively small; heterogeneity of patient population.
**Ethics Committee Approval:** Yes.
**Funding for this study:** n/a
**Author Disclosures:**
Antonella Messina: Nothing to disclose
Carlo Morosi: Nothing to disclose
Ciriaco Buonomenna: Nothing to disclose
Raffaella Vigorito: Nothing to disclose
Alfonso Vittorio Marchianò: Nothing to disclose
Alessandra Casale: Nothing to disclose

### RPS 116-7

**Dual-layer spectral detector CT in comparison with FDG-PET/CT for the assessment of lymphoma activity**

*K. Gehling*, T. Mokry, F. L. Giesel, U. Haberkorn, H-U. Kauczor, T. F. Weber; Heidelberg/DE

**Purpose:** In patients with malignant lymphoma, disease activity is recommended to be assessed by FDG-PET/CT and the Deauville five-point scale (5-PS). With contrast-enhanced dual-layer spectral detector CT (SDCT), iodine concentrations can be quantified to investigate lesion vascularization. The purpose of this study was to explore the potential of iodine concentration as an alternative surrogate parameter for lymphoma disease activity by investigating its correlation with SUVmax and 5-PS.
**Methods or Background:** 26 patients with malignant lymphoma were retrospectively analyzed. Portal venous phase contrast-enhanced SDCT and FDG-PET/CT had been performed within a time period of at most 3 months without interim treatment. CT attenuation values (AV), absolute iodine concentrations (aIC), and iodine concentrations normalized to inferior vena cava (nIC) of lymphoma lesions were correlated with SUVmax using Spearman's rank correlation coefficient. The performance of aIC and nIC to detect lymphoma activity (defined as 5-PS > 3) was determined using receiver operating characteristics (ROC) curves.
**Results or Findings:** 63 lesions were analyzed in total, and 32 lesions were considered active according to 5-PS. AV, aIC and nIC all correlated significantly with SUVmax. The strongest correlation was observed for nIC (Spearman $\rho=0.71$; $p<0.001$). The highest area under the ROC curve (AUROC) for detecting lymphoma activity was observed for nIC (AUROC=0.814), providing sensitivity, specificity, and diagnostic accuracy of

75%, 81%, and 78%, respectively at a threshold of 0.30. ROC analysis for AV (AUROC=0.801) and aIC (AUROC=0.792) yielded similar results.
**Conclusion:** In malignant lymphomas, there is significant correlation between metabolic activity as assessed by FDG-PET/CT and iodine concentration as assessed by SDCT. Iodine concentration shows promising diagnostic performance for detecting lymphoma activity and may represent a potential imaging biomarker.
**Limitations:** Not applicable
**Ethics Committee Approval:** Not applicable
**Funding for this study:** Not applicable
**Author Disclosures:**
Theresa Mokry: Nothing to disclose
Hans-Ulrich Kauczor: Nothing to disclose
Frederik L Giesel: Nothing to disclose
Kim Gehling: Nothing to disclose
Tim Frederik Weber: Nothing to disclose
Uwe Haberkorn: Nothing to disclose

**Live Q&A**

**16:45-17:45 CHANNEL 2**

### Joint Session of the ESR and EORTC

### ESR/EORTC

### Trials and tribulations: can imaging biomarkers tell the whole bony story?

**Moderator**
Frédéric E. Lecouvet; Brussels/BE

### ESR/EORTC-2

**WB-MRI vs PET for imaging prostate cancer**
Christoph Berliner; Hamburg/DE

**Learning Objectives:**
1. To learn about the techniques of whole-body MRI and PET for assessing bone metastases from prostate cancer.
2. To appreciate the strengths and limitations of the techniques.
3. To understand when to use these techniques in the clinic.

**Author Disclosures:**
Christoph Berliner: Other: ABX-CT-301expert panel (Phase III Study of F-18-PSMA-1007 vs F-18-Fluorocholine PET)

### ESR/EORTC-3

**The impact, potentials, and possibilities of PET in breast cancer care**
Lioe Fee de Geus-Oei; Leiden/NL
*(l.f.de_geus-oei@lumc.nl)*

**Learning Objectives:**
1. To learn about the techniques of PET for assessing bone metastases from breast cancer.
2. To appreciate the strengths and limitations of the techniques.
3. To understand when to use MRI vs PET techniques in the clinic.

### ESR/EORTC-4

**WB MRI in myeloma**
Marius E. Mayerhöfer; Vienna/AT
*(marius.mayerhoefer@meduniwien.ac.at)*

**Learning Objectives:**
1. To learn about the techniques of whole-body MRI for assessing myeloma.
2. To appreciate the strengths and limitations of the techniques.
3. To understand when to use MRI for assessing myeloma in the clinic.

**Author Disclosures:**
Marius E. Mayerhöfer: Speaker: Siemens, BMS

**Live Q&A: WB-MRI vs PET for imaging bone: is there a right answer?**

**16:45-17:45 CHANNEL 3**

### E³ - ECR Master Class

### E³ 726

### Renal involvement in coronavirus infection

**Moderator**
Raymond H. Oyen; Leuven/BE

### E³ 726-2

**A. Pathophysiology of the coronavirus on the kidney**
Nicolas Grenier; Bordeaux/FR
*(nicolas.grenier@chu-bordeaux.fr)*

**Learning Objectives:**
1. To understand the impact of COVID-19 on renal function and the underlying pathophysiology.
2. To learn about the association of renal involvement and patient outcome.

### E³ 726-3

**B. Renal imaging during the COVID-19 pandemic**
Lorenzo E. Derchi; Genoa/IT
*(lorenzo.derchi1@gmail.com)*

**Learning Objectives:**
1. To become aware of ways to organise renal imaging during the COVID-19 pandemic.
2. To learn the appearance of the kidneys and how best to depict them.
3. To become familiar with what renal clinicians expect from radiologists during COVID-19 pandemic.

### E³ 726-4

**C. Renal sequelae of COVID-19 infection**
Riccardo Manfredi; Rome/IT
*(riccardo.manfredi@unicatt.it)*

**Learning Objectives:**
1. To learn the spectrum of post COVID-19 chronic kidney changes.
2. To learn how to follow up post COVID-19 chronic kidney changes.
3. To understand the impact of COVID-19 on patients with pre-existing chronic renal disease.

**Live Q&A: The impact of COVID-19 on the kidneys**

**16:45-17:45 CHANNEL 4**

### New Horizons Session

### NH 2

### Radiology sequencing: massive radiomics analysis in practice

**Moderator**
Emanuele Neri; Pisa/IT

### NH 2-2

**Radiomics and phenotyping cancer**
Nickolas Papanikolaou; Lisbon/PT
*(nickolas.papanikolaou@research.fchampalimaud.org)*

**Learning Objectives:**
1. To describe the radiomics principles and the concept of imaging phenotype.
2. To show the radiomics pipeline.
3. To explain the concept of deep learning radiomics.

**Author Disclosures:**
Nickolas Papanikolaou: Advisory Board: Advantis Medical Imaging; Owner: MRIcons LTD

### NH 2-3

**Radiomics: from research to clinics in cancer imaging**
Stefano Trebeschi; Amsterdam/NL
*(s.trebeschi@nki.nl)*

**Learning Objectives:**
1. To show how to set up a radiomics study in oncology.
2. To discuss the use of imaging data for radiomics validation in view of the GDPR.
3. To discuss the exploitation of research results for the clinical implementation of radiomics.

### NH 2-4
**Linking radiomics features to other omics data in clinical practice**
Evis Sala; Cambridge/UK

**Learning Objectives:**
1. To learn the concept of radiogenomics (how to link, heat maps, etc.).
2. To review the potential applications of radiogenomics (radiomics vs genomics, proteomics, metabolomics etc.).
3. To discuss how to analyse such big data (imaging biobanks, large repositories, etc.).

### NH 2-5
**A research spot in radiomics: the EU EucanImage project**
Karim Lekadir; Barcelona/ES
*(karim.lekadir@ub.edu)*

**Learning Objectives:**
1. To describe the EucanImage project.
**Author Disclosures:**
Karim Lekadir: Investigator: University of Barcelona; Research Grant/Support: EuCanImage H2020

**Live Q&A: How to go beyond visual perception?**

**16:45-17:45** **CHANNEL 5**

### Research Presentation Session: Physics in Medical Imaging

### RPS 113

### The ultimate goal: improvement of image quality

**Moderator**
Osvaldo Rampado; Turin/IT

### RPS 113-2
**Bin-combination-based noise reduction for metal artifact reduction in photon-counting CT**
*P. M. Trapp*, A. Byl, L. Klein, S. Heinze, H-P. Schlemmer, S. Sawall, M. Kachelrieß; Heidelberg/DE (philip.trapp@dkfz.de)

**Purpose:** To counteract the noise increase observed in photon counting CT (PCCT) artifact reduction algorithms that are based on spectral bin combinations.
**Methods or Background:** PCCT simultaneously acquires several energy bins. Combinations of these bin images can be useful for artifact reduction, e.g. to reduce metal or beam-hardening artifacts. However, the image noise increases when the artifacts are reduced. We propose a cross bilateral filter that is guided by a low noise CT image, obtained by a noise minimizing bin combination. The filter is applied to the above-mentioned artifact-reducing but noisy bin combination, which additionally constrains the filter acting as second guide image. To validate our method, PCCT acquisitions of a pig cadaver and post-mortem specimens containing hip TEPs and dental implants were performed on an experimental PCCT system (SOMATOM CounT, Siemens Healthineers) using 140 kV with two energy bins.
**Results or Findings:** The processed images simultaneously provide high contrast-to-noise-ratios (CNRs) and reduced metal artifacts. The spatial resolution is maintained since the cross bilateral filter does not filter across edges. In soft tissue of a head scan with dental implants, image noise was reduced by a factor of five by postprocessing with our cross bilateral filter compared with the original artifact-reduced image. A self-guided bilateral filter achieved a noise reduction factor of two only.
**Conclusion:** A cross bilateral filter can effectively reduce image noise in PCCT acquisitions. Spectral data showing different traits like improved CNR or reduced artifacts are always available in PCCT. However, our method is not limited to PCCT but can be applied whenever spectral data is available.
**Limitations:** A spectral CT acquisition is necessary.
**Ethics Committee Approval:** The post-mortem experiments were approved by the local ethics committee (S-021/2020).
**Funding for this study:** BMBF (grant number 13N14804, funding program: Photonics Research Germany (KMU Innovative)).
**Author Disclosures:**
Achim Byl: Nothing to disclose
Laura Klein: Nothing to disclose
Philip Maurice Trapp: Nothing to disclose
Sarah Heinze: Nothing to disclose
Heinz-Peter Schlemmer: Nothing to disclose
Marc Kachelrieß: Nothing to disclose
Stefan Sawall: Nothing to disclose

### RPS 113-3
**Model observer assessment of photon-counting-detector coronary CT angiography and comparison with energy-integrating-detector CT**
*D. C. Rotzinger*[1], D. Racine[1], F. Becce[1], E. Lahoud[2], K. Erhard[2], S. A. Si-Mohamed[3], J. Greffier[4], R. A. Meuli[1], P. C. Douek[3]; [1]Lausanne/CH, [2]Haifa/IL, [3]Lyon/FR, [4]Nimes/FR
*(david.rotzinger@chuv.ch)*

**Purpose:** To objectively evaluate photon-counting-detector (PCD) coronary CT angiography (CCTA) in vitro and investigate its image quality characteristics compared with a clinical energy-integrating-detector (EID) CT system.
**Methods or Background:** A dedicated coronary artery module inserted into an anthropomorphic thorax phantom was scanned at regular dose (10 mGy) on a prototype clinical PCD-CT and a clinically available EID-CT under various conditions of simulated patient size (small, medium, large). Images were reconstructed using a filtered back-projection algorithm with a soft-tissue kernel. We assessed noise and contrast-dependent spatial resolution with noise power spectra (NPS) and target transfer functions (TTF), respectively. We further computed detectability indexes (d’) of simulated non-calcified atherosclerotic plaques (contrast difference=300 HU) using the non-prewhitening with eye filter model observer.
**Results or Findings:** For all three patient sizes, PCD-CT provided markedly lower noise magnitude (33-37% lower NPS amplitude) and higher frequency noise (sharper noise texture). Furthermore, PCD-CT provided consistently higher spatial resolution than EID-CT (32-36% better TTF), depending on patient size. In the resultant d’ analysis, PCD-CCTA outperformed the clinical EID-CT system in all investigated experimental conditions, providing superior detectability indexes. Of note, PCD-CT reached almost perfect detectability (accuracy≈95%) for simulated plaques measuring as low as 0.5-mm-thickness (for small-sized patients), whereas EID-CT had slightly less accuracy (≈85%).
**Conclusion:** PCD coronary CT angiography is feasible not only for small or medium-sized, but even for obese subjects. Furthermore, PCD-CCTA outperformed EID-CT in quantitative analysis and might enhance the diagnostic accuracy by providing lower noise magnitude, markedly improved spatial resolution, and superior plaque detectability.
**Limitations:** Only one radiation dose level and one iodine concentration were assessed. In addition, this study encourages further development of PCD-CCTA and in vivo validation.
**Ethics Committee Approval:** Not required.
**Funding for this study:** No funding was received.
**Author Disclosures:**
Salim Aymeric Si-Mohamed: Nothing to disclose
Damien Racine: Nothing to disclose
David Christian Rotzinger: Nothing to disclose
Reto Antoine Meuli: Nothing to disclose
Philippe Douek: Nothing to disclose
Joel Greffier: Nothing to disclose
Fabio Becce: Nothing to disclose
Elias Lahoud: Employee at Philips Medical Systems
Klaus Erhard: Employee at Philips Medical Systems

### RPS 113-4
**Virtual monoenergetic images from dual-energy CT: systematic assessment of task-based image quality performance for dual-source dual-energy CT**
*A. Euler*, D. Cester, M. Eberhard, T. Frauenfelder, H. Alkadhi; Zurich/CH

**Purpose:** To systematically compare task-based image quality among virtual monoenergetic images (VMI) and linear-blended images (LBI) from dual-source dual-energy CT (DECT) as a function of contrast task, radiation dose, patient size, and lesion diameter.
**Methods or Background:** A multisize image quality phantom (MercuryPhantom4.0) was repeatedly imaged on a 192-slice dual-source DECT at 100/Sn150kV and three radiation doses (5,10,15mGy). The phantom contained a noise and a resolution module with inserts of different materials. Bone and iodine inserts emulated a high-contrast task with and without iodine. A polystyrene insert served as low-contrast task. LBI and VMI at 40-190keV were reconstructed. Noise and resolution properties were evaluated using the noise power spectrum (NPS) and task-transfer function (TTF), respectively. Task-based image quality was determined by the detectability index (d’).

**Results or Findings:** Noise magnitude increased with increasing size and decreasing radiation dose or VMI-level. It was higher for VMI at 40-60keV compared to LBI. Differences in noise texture compared to LBI were found for very low and very high VMI-levels (50 keV, 130-190keV). Resolution depended on VMI-level for the low-contrast task with a shift to higher TTF frequencies with decreasing VMI-level. No relevant difference in TTF shape was observed for the high contrast tasks. D' increased with increasing radiation dose or lesion diameter and decreasing size. For both high-contrast tasks, d' was higher for 40-80keV and lower at high keV-levels compared to LBI. For the low-contrast task, d' was higher at 70-90keV and lower at 40-60keV.
**Conclusion:** VMI at 70 to 90keV yielded comparable or improved task-based image quality compared to linear-blended images. They could offer a balanced alternative independent of the imaging task.
**Limitations:** Phantom study. Single dual-energy approach and vendor.
**Ethics Committee Approval:** Not needed because of the design as phantom study
**Funding for this study:** No funding
**Author Disclosures:**
Thomas Frauenfelder: Nothing to disclose
Matthias Eberhard: Nothing to disclose
Davide Cester: Nothing to disclose
Andre Euler: Nothing to disclose
Hatem Alkadhi: Nothing to disclose

### RPS 113-5

**Integrated investigation of radiomic features reproducibility in NSCLC patients: the impact of scanner, x-ray tube voltage and strength of reconstruction algorithms in contrast-enhanced CT images**

*L. Rinaldi*[1], S. P. De Angelis[1], S. Raimondi[1], D. A. Origgi[1], A. Lascialfari[2], M. Mariani[2], M. Cremonesi[1], M. Bellomi[1], F. Botta[1]; [1]Milan/IT, [2]Pavia/IT
*(lisa.rinaldi@ieo.it)*

**Purpose:** The influence of acquisition/reconstruction parameters on radiomic features was systematically characterised in contrast-enhanced CT images of 103 non-small-cell lung cancer (NSCLC) patients, focusing on parameter variability observed at our Institute.
**Methods or Background:** All patients were imaged at 2.5mm slice thickness, on an OptimaCT660 (25 at 120kVp, 25 at 100kVp) and on a DiscoveryCT750HD scanner (26 at 120kVp, 27 at 100kVp), from GE-Healthcare, defining 4 populations. Each acquisition was reconstructed with FBP and 5 iterative strengths. For each reconstruction, 1414 features were extracted (Pyradiomics) from the contoured lesion considering non-filtered(154), Wavelet-filtered(560) and LoG-filtered(700) images. Clinical similarity was evaluated with Chi-square and Wilcoxon rank-sum tests. Feature differences within scanners and voltages were evaluated with Wilcoxon rank-sum test. Concordance between different reconstruction settings was evaluated for each feature with Overall Concordance Correlation Coefficient (OCCC≥0.85 defining good reproducibility). Using a multivariate mixed model, the contribution of each acquisition/reconstruction parameter in feature variation was investigated and p-value assessed.Combining univariate and multivariate models, 4 behaviours were identified: OCCC≥0.85-p-value<0.05 (group1), OCCC≥0.85-p-value≥0.05 (group2, most robust features), OCCC<0.85-p-value<0.05 (group3), OCCC<0.85-p-value≥0.05 (group4, least robust).
**Results or Findings:** The 4 populations were clinically comparable. Scanner and voltage did not affect features significantly. Reconstruction algorithms affected significantly (OCCC<0.85) 16/154 (non-filtered images), 116/560 (Wavelet-filtered) and 22/700 (LoG-filtered) features. Most were in groups 1 and 3, characterised by significant trend with the algorithm strength, systematic among patients. Correction coefficients were derived from the multivariate model to reduce this dependency.
**Conclusion:** In our database, feature stability was influenced by reconstruction algorithms. Feature selection by univariate analysis may exclude potentially informative features which can be restored by integration with correction factors from multivariate analysis.
**Limitations:** Low patient number, few scanner types/vendors, manual segmentation, feature repeatability not assessed.
**Ethics Committee Approval:** Approved(UID-2412)
**Funding for this study:** Ministero della Salute-Ricerca Finalizzata(GR-2016-02362050)
**Author Disclosures:**
Francesca Botta: Nothing to disclose
Manuel Mariani: Nothing to disclose
Sara Raimondi: Nothing to disclose
Marta Cremonesi: Nothing to disclose
Simone Pietro De Angelis: Nothing to disclose
Alessandro Lascialfari: Nothing to disclose
Massimo Bellomi: Nothing to disclose
Daniela Anna Origgi: Nothing to disclose
Lisa Rinaldi: Nothing to disclose

### RPS 113-6

**Image quality characterisation of a biplane angiographic equipment with moving details and generalised spatio-temporal model observers**

*R. Villa*, N. Paruccini, V. Faccenda, P. Caricato, E. De Ponti; Monza/IT
*(raffaele.vill@gmail.com)*

**Purpose:** Characterise image quality performances of a biplane angiographic equipment, applying generalised spatio-temporal model observer with details in motion.
**Methods or Background:** Different clinical protocols, characterised by different fps, dose levels and filtration, have been considered. Contrast media flow has been simulated with the moving component of the CIRS Dynamic Thorax Phantom. Details of different diameters (diameter from 2 to 6 mm) have been used to simulate typical small vessels sizes and moved, with a speed ranging from 2.5 to 10 mm/s, in order to simulate blood flow.Images have been processed with two different Model Observers in order to simulate the perceived clinical image quality in terms of contrast detectability: a Non-Prewhitening Eye filter (NPWE) with eye Spatio-temporal contrast sensitivity function and a Channelized Hotelling Observer (CHO) with Spatio-temporal Gabor filters. Results have been compared with a series of 2AFC experiments performed by trained observers.
**Results or Findings:** Comparison with 2AFC experiments underline a good agreement (RMSE <20%) for each considered condition with both model observers.
Results suggest the validity of a dose reduction through an acquisition frame rate reduction, especially with fluoroscopy modalities that are characterised by higher frame rate.
**Conclusion:** The agreement between the results of the implemented models and the 2AFC experiments underline the potential of simulating the perceived clinical image quality with mathematical observers.
**Limitations:** Model Observer approaches required an high number of images, especially with the proposed generalised models.
**Ethics Committee Approval:** Not required
**Funding for this study:** Nothing
**Author Disclosures:**
Elena De Ponti: Nothing to disclose
Raffaele Villa: Nothing to disclose
Nicoletta Paruccini: Nothing to disclose
Paolo Caricato: Nothing to disclose
Valeria Faccenda: Nothing to disclose

### RPS 113-7

**Effect on image quality and dose reduction of a new deep learning reconstruction algorithm for CT compared with a hybrid and an advanced MBIR algorithms: a phantom study**

*J. Greffier*, D. Dabli, A. Hamard, J. Frandon, J-P. Beregi; Nimes/FR
*(joel.greffier@chu-nimes.fr)*

**Purpose:** To assess the impact on image quality and dose reduction of a new Deep Learning Image Reconstruction (DLR) algorithm compared to a hybrid (HIR) and an advanced model-based iterative reconstruction (MBIR) algorithm.
**Methods or Background:** Data acquisitions were performed at six dose levels (CTDIvol: 15/10/7.5/5/2.5/1mGy) using a standard phantom designed for image quality assessment. Raw data were reconstructed using three levels (Mild, Standard, Strong) of HIR (AIDR 3D), of MBIR (FIRST) and DLR (AiCE). Noise-power-spectrum (NPS) and task-based transfer function (TTF) were computed. Detectability index (d') was computed to model the detection of a large mass in the liver and a small calcification.
**Results or Findings:** NPS peaks were lower with DLR than with HIR or MBIR. The average NPS spatial frequency was lower with DLR than HIR but higher than MBIR for Standard and Strong levels. For acrylic insert, values of TTF50% were higher with DLR than HIR and MBIR, except for Mild level. For bone insert, values of TTF50% were higher with DLR than HIR but lower than MBIR. For both simulated lesions, d' values were higher with DLR than HIR and MBIR (except for Strong level). Using the Standard level, dose could be reduced by -78% for the small calcification and -59% for the large mass using DLR compared to HIR.
**Conclusion:** The new DLR algorithm generates an image quality with less noise and/or less smudged/smooth images and a higher detectability than the

Wednesday

HIR or MBIR algorithms, which supports its routine use in a dose optimization process.
**Limitations:** Based on the AiCE limits for abdominal images, only a soft tissue kernel and a slice thickness 0.5-mm thick were used.
**Ethics Committee Approval:** Institutional Review Board approval was not required for this study because it's a study phantom
**Funding for this study:** No
**Author Disclosures:**
Jean-Paul Beregi: Nothing to disclose
Aymeric Hamard: Nothing to disclose
Julien Frandon: Nothing to disclose
Joel Greffier: Nothing to disclose
Djamel Dabli: Nothing to disclose

**Live Q&A**

**18:00-19:00 CHANNEL 2**

### RTF - Radiology Trainees Forum

### TF

### Highlighted Lectures

**Moderators**
Martin Reim; Tartu/EE
Saif Afat; Tübingen/DE

### EU 6-2
**Introduction (part 1)**
Martin Reim; Tartu/EE
*(martin.reim@kliinikum.ee)*

**Learning Objectives:**
1. To focus on the up-to-date radiological principles in the management of acute ischaemic stroke; the diagnostic approach and interventional radiology strategies.
2. To improve the understanding of myotendinous junction anatomy and its clinical/radiological importance.
3. To encourage young radiologists in their on-call duty with tips from experienced emergency specialist.

### EU 6-3
**Introduction (part 2)**
Saif Afat; Tübingen/DE
*(saif.afat@gmx.de)*

**Learning Objectives:**
1. To focus on the up-to-date radiological principles in the management of acute ischaemic stroke; the diagnostic approach and interventional radiology strategies.
2. To improve the understanding of myotendinous junction anatomy and its clinical/radiological importance.
3. To encourage young radiologists in their on-call duty with tips from experienced emergency specialist.

### TF-5
**Stroke: from diagnosis to treatment**
Alan O'Hare; Dublin/IE
*(alanohare@beaumont.ie)*

**Learning Objectives:**
1. To learn how to approach stroke from a radiology perspective: the possibilities, indications, and limitations.
2. To appreciate and improve the collaboration to diagnose and treat the stroke patients in the most effective way.
3. To understand the importance of timely diagnostic imaging studies and access to interventional procedures.

### TF-6
**Myotendinous junction anatomy and muscle injury**
Anagha P. Parkar; Bergen/NO

**Learning Objectives:**
1. To learn the detailed anatomy of the myotendinous junction and the clinical importance of the injuries in that region.
2. To appreciate the importance of small details in the anatomy and to improve the knowledge of musculoskeletal radiology.
3. To understand the possibilities and limitation in diagnosing muscular injuries.

### TF-7
**My first night on call: tips to overcome fear**
Elizabeth Dick; London/UK

**Learning Objectives:**
1. To learn about the possible difficulties that young radiologist will face early in their career at emergency radiology departments and during the nervous first on-call shifts.
2. To appreciate the lessons learned from other professionals and to find out about the tips and tricks to facilitate their on-call work.
3. To understand how to achieve a more confident approach and avoid the possible mistakes that every radiologist might face while being on-call.
**Author Disclosures:**
Elizabeth Dick: Speaker: Guerbet

**Live Q&A: What is the most critical knowledge that young radiologists should take from your talk and this session?**

**18:00-19:00 CHANNEL 3**

### Refresher Course: Paediatric

### RC 312

### Fluoroscopy: a mainstay state of the art in paediatric radiology

**Moderator**
Johanna Aronniemi; Helsinki/FI

### RC 312-2
**A. Use of fluoroscopy in neonates with suspected gastrointestinal pathology**
Ingegerd Aagenaes; Oslo/NO
*(iaagenes@ous-hf.no)*

**Learning Objectives:**
1. To learn about the most common indications for fluoroscopy in non-urogenital queries in neonates.
2. To become familiar with the technique, findings, and pitfalls for the most common fluoroscopy non-urogenital queries in the neonate.
3. To discuss the best fluoroscopic approach in suspected bowel obstruction in a neonate.

### RC 312-3
**B. Gastrointestinal and other applications in the older child**
Stéphanie Franchi-Abella; Le Kremlin-Bicêtre/FR
*(stephanie.franchi@aphp.fr)*

**Learning Objectives:**
1. To learn about the most common indications for fluoroscopy in non-urogenital queries in infants and older children.
2. To become familiar with the most common fluoroscopy queries in this patient group.
3. To discuss the most common findings and pitfalls.

### RC 312-4
**C. Urogenital applications**
Giulia Perucca; Turin/IT
*(giuliaperucca@yahoo.it)*

**Learning Objectives:**
1. To learn about the indications for fluoroscopy in urogenital queries in children.
2. To become familiar with the technique in fluoroscopic urogenital queries.
3. To discuss the most common findings and pitfalls.

### RC 312-5
**D. When can ultrasound replace fluoroscopy?**
Simon G.F. Robben; Maastricht/NL
*(s.robben@mumc.nl)*

**Learning Objectives:**
1. To learn about the indications for dynamic ultrasound studies.
2. To become familiar with the technique of dynamic ultrasound.
3. To discuss the most common pitfalls.

**Live Q&A: When can ultrasound imaging replace fluoroscopy?**

18:00-19:00 **CHANNEL 5**

### Transatlantic Course of ESR and RSNA (Radiological Society of North America): Stroke Imaging and Endovascular Treatment: Now and the Future

### TC 128

### Current status of stroke workup and treatment

**Moderators**
Raman Uberoi; Oxford/UK
Achala Vagal; Cincinnati, OH/US

### TC 128-3

**A. Current status of endovascular management of acute ischaemic stroke: evidence and guidelines**
Raman Uberoi; Oxford/UK
*(Raman.Uberoi@ouh.nhs.uk)*

**Learning Objectives:**
1. To learn about current evidence of endovascular treatment in acute ischaemic stroke.
2. To become familiar with evidence-based guidelines (AHA/ASA and ESO/ESMINT) in stroke treatment.

### TC 128-4

**B. CT-based evaluation of acute stroke: advantages and challenges**
Achala Vagal; Cincinnati, OH/US
*(vagala@ucmail.uc.edu)*

**Learning Objectives:**
1. To learn about the advantages and challenges of CT-based stroke imaging workup.
2. To become familiar with the pitfalls of CT perfusion imaging.

**Author Disclosures:**
Achala Vagal: Research Grant/Support: NIH, Cerenovus (Research grant, ENDOLOW trial)

### TC 128-5

**C. MRI-based evaluation of acute stroke: advantages and challenges**
Max Wintermark; San Francisco, CA/US

**Learning Objectives:**
1. To learn about the advantages of MRI-based stroke imaging workup.
2. To become familiar with the challenges of MRI-based stroke imaging workup.

**Live Q&A: Strategy for acute stroke imaging and intervention**

### Thursday, March 4

08:00-09:00 CHANNEL 2

### State of the Art Symposium

### SA 1

### My future and me: personalised cardiac risk stratification

**Moderator**
Gorka Bastarrika; Pamplona/ES

### SA 1-2

**Screening asymptomatic individuals: state of the evidence and future directions**
Rozemarijn Vliegenthart; Groningen/NL
*(r.vliegenthart@umcg.nl)*

**Learning Objectives:**
1. To become familiar with the evidence for CT calcium scoring in asymptomatic individuals.
2. To learn about the current role of CT calcium scoring in primary prevention guidelines.
3. To appreciate the issues of the wide-spread use of CT calcium scoring and future directions.

**Author Disclosures:**
Rozemarijn Vliegenthart: Grant Recipient: Dutch Heart Foundation; Dutch Research Council; Research Grant/Support: Institutional Research Grant, Siemens Healthineers; Other: Associate Editor, Eur J Radiology

### SA 1-3

**Stable chest pain and current guidelines**
Giuseppe Muscogiuri; Milan/IT
*(g.muscogiuri@gmail.com)*

**Learning Objectives:**
1. To become familiar with the clinical indication of CCTA in patients with chest pain.
2. To understand the clinical management of patients following CCTA results based on the current guidelines.
3. To understand the economic impact of CCTA in clinical practice following the guidelines.
4. To appreciate the impact of CCTA in terms of prognosis.

### SA 1-4

**Risk stratification in the emergency department**
Fabian Bamberg; Freiburg/DE
*(fabian.bamberg@uniklinik-freiburg.de)*

**Learning Objectives:**
1. To learn about the role of cardiac CT in the emergency setting.
2. To become familiar with the different findings in cardiac CT in the emergency setting.
3. To understand the value of triple-rule-out CT scanning for risk stratification.
4. To learn about embedding in a non-imaging cardiac risk stratification in the emergency setting.

**Author Disclosures:**
Fabian Bamberg: Research Grant/Support: Siemens Healthineers, Bayer Healthcare; Speaker: Siemens Healthineers, Bayer Healthcare, Bracco Diagnostics

### SA 1-5

**Emerging artificial intelligence biomarkers: above the current state of the art?**
Carlo N. De Cecco; Atlanta, GA/US
*(carlodececco@gmail.com)*

**Learning Objectives:**
1. To learn about current and future applications of machine learning/artificial intelligence in cardiac imaging.
2. To understand what is meant by the terms machine learning, deep learning, and radiomics in cardiac imaging.
3. To appreciate some of the limitations of artificial intelligence biomarkers in cardiac imaging.

**Author Disclosures:**
Carlo N. De Cecco: Research Grant/Support: Siemens

**Live Q&A: How to match the best patient for the best test?**

08:00-09:00 CHANNEL 3

### Coffee & Talk (open forum) Session

### C 4

### Radiology in the cloud: advantages and threats

**Moderator**
Luis Martí-Bonmatí; Valencia/ES

### C 4-2

**Trustworthy radiology in the cloud**
Ignacio Blanquer; Valencia/ES
*(iblanque@dsic.upv.es)*

**Learning Objectives:**
1. To learn about the concept of trustworthiness in cloud services.
2. To understand the different dimensions of trustworthiness.
3. To understand the complexity of cloud services when dealing with the processing of sensitive data.

### C 4-3

**Data protection, cybersecurity, and GDPR in cloud radiology practice**
Arnon Makori; Tel Aviv/IL

**Learning Objectives:**
1. To learn about the key requirements for data protection, cybersecurity, and GDPR relating to cloud-based radiology services.
2. To understand the challenges of cybersecurity in cloud-based radiology services.
3. To understand how to evaluate data protection and GDPR compliance of cloud-based radiology services.

**Author Disclosures:**
Arnon Makori: Advisory Board: Carestream

### C 4-4

**Pseudonymisation and full anonymisation**
Karine Seymour; Labège/FR
*(kseymour@medexprim.com)*

**Learning Objectives:**
1. To understand the differences between full anonymisation and pseudonymisation and the implications with respect to GDPR.
2. To identify the roles and obligations of data controllers and data processors in the deidentification process.
3. To become familiar with the confidentiality profiles defined by the DICOM standard and existing solutions.

**Author Disclosures:**
Karine Seymour: Founder: Medexprim

**Live Q&A**

08:00-09:00 CHANNEL 5

### Professional Challenges Session

### PC 4

### Safety challenges in everyday clinical practice

**Moderators**
Andrew England; Salford/UK
Berit Verbist; Leiden/NL

### PC 4-3

**Benefit/risk communication to adult and paediatric patients: top tips**
Jonathan Portelli; Msida/MT
*(jonathan.portelli@um.edu.mt)*

**Learning Objectives:**
1. To become familiar with benefit and risk communication for adult and paediatric patients.
2. To appreciate opportunities to promote the effective communication of benefit/risk.

**PC 4-4**
**MRI safety: recent incidents and continued opportunities to improve**
Anne Dorte Blankholm; Aarhus/DK
*(anneblan@rm.dk)*

**Learning Objectives:**
1. To learn about recent incidents regarding MRI safety.
2. To become familiar with opportunities for improving MRI safety within clinical practice.
**Author Disclosures:**
Anne Dorte Blankholm: Board Member: SMRT President Elect

**PC 4-5**
**Embedding quality improvement in your everyday clinical practice**
Marie-Louise Ryan; Dublin/IE (marielouise.butler@gmail.com)

**Learning Objectives:**
1. To learn about quality improvement tools and strategies.
2. To understand how to embed quality improvements within everyday clinical practice.

**PC 4-6**
**GDPR: confidentiality and patient safety considerations for radiographers**
*Andreas Strassl*, Christian Schneckenleitner; Vienna/AT
*(andreas.strassl@meduniwien.ac.at)*

**Learning Objectives:**
1. To appreciate the legislative issues surrounding confidentiality and patient safety.
2. To understand patient safety issues pertinent to radiographers.

**Live Q&A: How to build a better safety culture among clinical radiographers?**

**09:15-10:15 CHANNEL 2**

### E³ - Rising Stars Programme: Basic Sessions
*Organised by the ESOR*

### BS 13
### Radiologic anatomy: chest

**Moderator**
Fabian Rengier; Heidelberg/DE

**BS 13-2**
**Mediastinal**
Mariaelena Occhipinti; Florence/IT

**Learning Objectives:**
1. To review the mediastinal anatomy according to old and new classifications.
2. To define "normal" and "abnormal" mediastinal anatomy.
3. To use radiologic anatomy to characterise mediastinal lesions and to stage tumours.

**BS 13-3**
**Lungs**
Cornelia M. Schaefer-Prokop; Amersfoort/NL
(cornelia.schaeferprokop@gmail.com)

**Learning Objectives:**
1. To learn how to interpret mediastinal lines and contours in chest radiography.
2. To become familiar with the "signs" in chest radiography helpful for diagnosis.
3. To learn about the normal appearance of lung parenchyma on CT and to use the anatomy of the secondary lobule for analysis of chest diseases.

**BS 13-4**
**Vasculature**
Iva Zuza; Rijeka/HR

**Learning Objectives:**
1. To identify thoracic vascular structures on chest x-rays.
2. To interpret chest vasculature, including aorta and coronary arteries, major aortic branches, and pulmonary arteries and veins, by using multidetector CT (MDCT).
3. To review and illustrate the most common variants and anomalies.
4. To discuss the challenges and limitations in interpretation based on particular CT scanning protocols.

**Live Q&A**

**09:15-10:15 CHANNEL 4**

### Refresher Course: Musculoskeletal

### RC 110
### Ultrasound in musculoskeletal radiology

**Moderator**
Andrew J. Grainger; Cambridge/UK

**RC 110-2**
**A. Musculoskeletal ultrasound in the emergency department**
Luca M. Sconfienza; Milan/IT
*(io@lucasconfienza.it)*

**Learning Objectives:**
1. To explain the indications of musculoskeletal ultrasound in the emergency department.
2. To describe differential diagnostic considerations of musculoskeletal ultrasound in the emergency department.
**Author Disclosures:**
Luca M. Sconfienza: Advisory Board: Pfizer; Speaker: Samsung Medison, Esaote SPA, Novartis, Abiogen, Janssen-Cilag

**RC 110-3**
**B. The role of ultrasound in the diagnosis of soft tissue tumours**
Maria Pilar Aparisi Gomez; Valencia/ES
*(pilucaparisi@yahoo.es)*

**Learning Objectives:**
1. To explain the ultrasound findings of soft tissue tumours.
2. To describe differentils in the diagnosis of soft tissue tumours by ultrasound.

**RC 110-4**
**C. Ultrasound elastography in musculoskeletal radiology**
Ali Balci; İzmir/TR
*(alibalciradyolog@yahoo.com)*

**Learning Objectives:**
1. To explain the techniques used in ultrasound elastography of the musculoskeletal system.
2. To describe ultrasound elastography findings in musculoskeletal radiology.

**Live Q&A: New clinical roles for musculoskeletal ultrasound**

**09:15-10:15 CHANNEL 5**

### Research Presentation Session: Abdominal Viscera

### RPS 101a
### Advances in liver imaging

**Moderator**
Giuseppe Brancatelli; Palermo/IT

**RPS 101a-2**
**LI-RADS ancillary features favouring benignity: is there a role in LR-5 observations?**

D. S. Gagliano, *R. Cannella*, F. Vernuccio, M. Antonucci, G. Brancatelli; Palermo/IT

**Purpose:** The LI-RADS algorithm allows category downgrade in presence of ancillary features (AFs) favoring benignity, even in observations that are definitively HCC (LR-5) according to major features. This study aims to assess the role of AFs favoring benignity in LR-5 observations and their impact for possible category downgrade.
**Methods or Background:** This retrospective study included 119 consecutive cirrhotic patients with at least one LR-5 observations imaged with gadoxetate disodium-enhanced MRI between 2016 and 2020. Two blinded radiologists independently evaluated the presence of AFs favoring malignancy, not HCC in particular, favoring HCC in particular, and AFs favoring benignity. Category downgrade was considered possible in presence of ≥ 1 AF favoring benignity and no AFs favoring malignancy. Correlation between lesions size and number of AFs was assessed using the Spearman's rank correlation coefficient. The Cohen's kappa (k) test was used to assess inter-reader agreement.
**Results or Findings:** Final cohort included 162 LR-5 with a mean size of 22.9±15.8 mm. Hepatobiliary phase hypointensity was the most common AF among LR-5 observations (R1: 87.7%; R2: 91.4%). AFs favoring benignity were reported in 9 (5.5%) and 15 (9.2%) observations by R1 and R2, respectively. Category downgrade was considered possible in only one (0.6%) observation by each reader. There was a statistically significant correlation between observations size and number of AFs favoring malignancy ($p<0.001$), not HCC in particular ($p\leq0.001$), and favoring HCC in particular ($p<0.001$). The inter-reader agreement ranged from poor to substantial ($k$=-0.03-0.74).
**Conclusion:** AFs favoring benignity can be encountered in LR-5 observations but they have a marginal impact for possible category downgrade.
**Limitations:** Retrospective study design, lack histopathological proof for all lesions.
**Ethics Committee Approval:** IRB-approved, informed consent was waived.
**Funding for this study:** No funding was received for this study.
**Author Disclosures:**
Federica Vernuccio: Nothing to disclose
Michela Antonucci: Nothing to disclose
Roberto Cannella: Nothing to disclose
Domenico Salvatore Gagliano: Nothing to disclose
Giuseppe Brancatelli: Nothing to disclose

### RPS 101a-3

**Non-contrast MRI for hepatocellular carcinoma detection: a systematic review and meta-analysis: a potential surveillance tool?**
M. V. Chan, *R. Huo*, N. Trieu, A. Mitchelle, J. George, E. He, A. Lee, J. Chang, J. Yang; Sydney/AU

**Purpose:** This meta-analysis investigates the diagnostic performance of non-contrast magnetic resonance imaging (MRI) for the detection of hepatocellular carcinoma (HCC).
**Methods or Background:** A systematic review was performed to May 2020 for studies which examined the diagnostic performance of non-contrast MRI (multi-sequence or diffusion-weighted imaging (DWI)- alone) for HCC detection in high risk patients. The primary outcome was accuracy for the detection of HCC. Random effects models were used to pool outcomes for sensitivity, specificity, positive likelihood ratio (LR) and negative LR. Subgroup analyses for cirrhosis and size of the lesion were performed.
**Results or Findings:** Twenty-two studies were included involving 1685 patients for per-patient analysis and 2128 lesions for per-lesion analysis. Multi-sequence non-contrast MRI (NC-MRI) using T2+DWI□T1 sequences had a pooled per-patient sensitivity of 86.8% (95%CI:83.9-89.4%), specificity of 90.3% (95%CI:87.3-92.7%), and negative LR of 0.17 (95%CI:0.14-0.20). DWI-only MRI (DW-MRI) had a pooled sensitivity of 79.2% (95%CI:71.8-85.4%), specificity of 96.5% (95%CI:94.3-98.1%) and negative LR of 0.24 (95%CI:1.62-0.34). In patients with cirrhosis, NC-MRI had a pooled per-patient sensitivity of 87.3% (95%CI:82.7-91.0%) and specificity of 81.6% (95%CI:75.3-86.8%), whilst DWI-MRI had a pooled sensitivity of 71.4% (95%CI:60.5-80.8%) and specificity of 97.1% (95%CI:91.9-99.4%). For lesions <2cm, the pooled per-lesion sensitivity was 77.1% (95%CI:73.8-80.2%). For lesions >2cm, pooled per-lesion sensitivity was 88.5% (95%CI:85.0-91.5%).
**Conclusion:** Non-contrast MRI has a moderate negative LR and high specificity with acceptable sensitivity for the detection of HCC, even in patients with cirrhosis and with lesions <2cm. Prospective trials to validate if non-contrast MRI can be used for HCC surveillance is warranted.
**Limitations:** There is a lack of prospective studies. Futhermore there is a lack of studies that subanalyse cirrhosis and steatosis which impact ultrasound but nor MRI.
**Ethics Committee Approval:** None required
**Funding for this study:** None
**Author Disclosures:**
Ruth Huo: Nothing to disclose
Michael Vinchill Chan: Nothing to disclose
Emily He: Nothing to disclose
Jessica Yang: Nothing to disclose
Alice Lee: Nothing to disclose
Jeff Chang: Nothing to disclose
Amer Mitchelle: Nothing to disclose
Nelson Trieu: Nothing to disclose
Jacob George: Nothing to disclose

### RPS 101a-4

**Multivariate analysis of radiological predictors of Beta-catenin mutation status in hepatocellular carcinoma (HCC) according to the Liver Imaging Reporting and Data System (LI-RADS)**
*J. Yousef*[1], R. Catania[2], B. Sholosh[1], S. Thangasamy[1], S. S. Monga[1], S. Wu[1], K. P. Chupetlovska[1], A. Furlan[1], G. Nebbia[1]; [1]Pittsburgh, PA/US, [2]Chicago, IL/US
*(yousefjm@upmc.edu)*

**Purpose:** In HCC, beta-catenin mutation is associated with a relatively favorable prognosis and may be a promising target for future therapies. We aimed to investigate the association between CT and MR imaging features and the beta-catenin mutation status of HCC.
**Methods or Background:** This retrospective HIPAA-compliant study included 157 subjects with pathologically proven HCC and a contrast-enhanced CT (n=82) or MRI (n=75) performed within 1-year of pathology. The beta-catenin mutation status of each HCC was: "mutated", n=22 (14%), "possibly mutated", n=25 (16%); "wild", n=110 (70%). Twenty-two imaging features were recorded by 2 experienced readers according to LI-RADS v2018; consensus was reached with a third experienced reader. Logistic regression analysis was used to compute the AUC for task 1 ("mutated" vs. "wild") and task 2 ("mutated" or "possibly mutated" vs. "wild"). The AUC represents the percentage of case-control pairs where the case has a higher logistic regression score than the control (here case refers to "mutated"). The Odds Ratios (ORs) associated with each imaging feature were then calculated.
**Results or Findings:** For task 1, the AUC value was 79.81% and the predictive features were tumor size (OR = 2.04, $p<0.05$) and corona enhancement (OR= 6.27, $p < 0.1$). For task 2, the AUC value was 71.69% and the predictive features were tumor size (OR= 1.71, $p<0.05$), corona enhancement (OR= 4.11, $p < 0.1$), and presence of nodule-in-nodule (OR= 9.16, $p<0.1$). Notably, the LI-RADS category was not predictive of the beta-catenin mutation status.
**Conclusion:** Tumor size, corona enhancement, and nodule-in-nodule show significant association with beta-catenin mutation status of HCC.
**Limitations:** Single-institutional data; relatively small cohort; qualitative imaging assessment
**Ethics Committee Approval:** IRB approved
**Funding for this study:** None
**Author Disclosures:**
Giacomo Nebbia: Nothing to disclose
Roberta Catania: Nothing to disclose
Senthur Thangasamy: Nothing to disclose
Alessandro Furlan: Nothing to disclose
Biatta Sholosh: Nothing to disclose
Satdarshan Singh Monga: Nothing to disclose
Jacob Yousef: Nothing to disclose
Shandong Wu: Nothing to disclose
Kalina Plamenova Chupetlovska: Nothing to disclose

### RPS 101a-5

**Outcome of LR-3 and LR-4 observations without arterial phase hyperenhancement at Gd-EOB-DTPA-enhanced MRI follow-up**
*I. Viola*, F. Agnello, L. Rabiolo, F. Midiri, G. Micci, M. Galia; Palermo/IT
*(ilaria.viola4@gmail.com)*

**Purpose:** The aim is to retrospectively evaluate the outcome of LR-3 and LR-4 observations without arterial phase hyperenhancement (APHE) and identify which features could predict LR-5 progression at Gd-EOB-DTPA enhanced MRI follow-up.
**Methods or Background:** 49 cirrhotic patients with 55 LR-3 and 19 LR-4 without APHE were enrolled. LR-3 and LR-4 were classified as decreased, stable or increased in category at follow-up. Images were independently analyzed by two abdominal radiologists. Presence or absence of major and ancillary LI-RADS features, LIRADS category, and observation size were evaluated. Chi-square and Fischer's exact test were used to assess if baseline LIRADS features and baseline diameter (< 10; ≥ 10 mm) were associated with LR-5 progression of LR-3 and LR-4. $P < .05$ was considered statistically significant
**Results or Findings:** Of 55 LR-3, 17 (31%) progressed to LR-5 at follow-up, 3 (6%) progressed to LR-4, 6 (11%) remained stable in category, and 29 (52%) decreased in category. Of 19 LR-4, 8 (42%) progressed to LR-5 at follow-up, 4 (21%) remained stable in category and 7 (37%) decreased to LR-3. Major and ancillary features were not significantly different among LR-3 and LR-4 that progressed to LR-5 and those that remained stable or decreased in category at follow-up. A diameter ≥ 10 mm significantly increased LR-5 progression risk of

Thursday

LR-3 (OR = 6.07; 95% CI: 0.12; 60.28]; P < .001), while LR-4 with a diameter ≥ 10 mm more likely become LR-5 at follow-up (OR = 8.95; 95% CI: 0.73; 111.8; P = .083]).
**Conclusion:** LR-3 and LR-4 observations without APHE were often downgraded or remained stable in category on Gd-EOB-DTPA-enhanced MRI follow-up. LR-3 and LR-4 observations with diameter ≥ 10 mm had an increased risk of LR-5 progression.
**Limitations:** No
**Ethics Committee Approval:** No
**Funding for this study:** Not applicable
**Author Disclosures:**
Ilaria Viola: Nothing to disclose
Giuseppe Micci: Nothing to disclose
Massimo Galia: Nothing to disclose
Federico Midiri: Nothing to disclose
Francesco Agnello: Nothing to disclose
Lidia Rabiolo: Nothing to disclose

### RPS 101a-6

**T1 reduction rate with Gd-EOB-DTPA for liver function mapping in MRI: comparison between 1.5T and 3T in a cross-sectional comparative study**

*V. C. Obmann*, D. Catucci, A. Berzigotti, C. Gräni, L. Ebner, J. T. Heverhagen, A. Christe, A. T. Huber; Bern/CH
*(verena.obmann@insel.ch)*

**Purpose:** To compare MRI T1 reduction rates between 1.5T and 3T for liver function mapping in a large cross-sectional comparative study.
**Methods or Background:** 287 consecutive patients with liver MRI and T1 mapping before and 20 minutes after intravenous (i.v.) GD-EOB-DTPA administration were included (132 on 1.5T, 132 on 3T) between 09/2018–07/2019. Patients were grouped into no chronic liver disease, chronic liver disease (CLD) and cirrhosis Child-Pugh A-C. T1 reduction rate was calculated as (native T1 – post contrast T1) / native T1 and compared between 1.5T and 3T in every patient group using a Mann-Whitney U test with Bonferroni correction. The predictive value of T1 reduction rate and cutoff values to predict CLD and cirrhosis Child A-C were determined with a receiver operating characteristic (ROC) analysis. A p-value <0.05 was defined as statistically significant.
**Results or Findings:** T1 reduction rates showed no significant difference between 1.5T and 3T in all patient groups: for no CLD the reduction rate was 0.75 vs. 0.77 (p=0.623), for CLD 0.69 vs. 0.70 (p>0.999), for Child A 0.65 vs. 0.65 (p>0.999), for Child B 0.61 vs. 0.56 (p=0.311) and for Child C 0.47 vs. 0.49 (p>0.999). T1 reduction rate showed a good predictive value for CLD (AUC=0.83), cirrhosis Child A (AUC=0.81), Child B (AUC=0.84) and Child C (AUC=0.91), all with a p-value <0.05.
**Conclusion:** MRI T1 reduction rate allows an assessment of the liver function with comparable values between 1.5T and 3T.
**Limitations:** We acknowledge limitations to our study, mainly the retrospective cross-sectional design with a relatively small sample size of Child C patients presented, while all other groups were much bigger.
**Ethics Committee Approval:** Yes.
**Funding for this study:** This project was funded by the Swiss National Science Foundation (SNF) grant # 320030_188591.
**Author Disclosures:**
Johannes T. Heverhagen: Nothing to disclose
Damiano Catucci: Nothing to disclose
Verena Obmann: Nothing to disclose
Christoph Gräni: Nothing to disclose
Lukas Ebner: Nothing to disclose
Andreas Christe: Nothing to disclose
Adrian Thomas Huber: Grant Recipient at Swiss National Science Foundation
Annalisa Berzigotti: Nothing to disclose

### RPS 101a-7

**Deep learning analysis of liver metastases changes in CECT**

A. Szeskin, A. Dodi, S. Rochman, R. Lederman, H. Fruchtman-Brot, Y. Azraq, L. Joskowicz, *J. Sosna*; Jerusalem/IL
*(jacobs@hadassah.org.il)*

**Purpose:** To compare reading of contrast enhanced CT (CECT) by radiologists to computer-aided reading using a novel method that simultaneously analyzes the changes of liver metastasis between the current and the prior scans by deep learning classification.
**Methods or Background:** We developed a novel deep learning changes analysis method inputs the current and prior CECT scans and automatically detects, segments and classifies the liver metastases. It uses a unique 3D U-Net classifier that simultaneously compares the scans and finds their differences. The network was trained on 68 pairs of scans with a total of 2,042 liver metastases. We assessed 20 pairs (current and prior) of CECT scans of patients with metastatic liver disease (colon, breast, pancreas) acquired in the venous phase. The total number of lesions was 461 (mean=11.5, std=7.8). Two independent expert radiologists performed 40 readings (20 each) to assess disease status using RECIST 1.1 with two weeks between the conventional and the computer aided evaluations. Our reference standard was defined by a third expert observer augmented with the computer-aided technique and blinded to the results of the other readers.
**Results or Findings:** Conventional reading and computer aided reading by radiologists were in consensus regarding disease status in 32 readings (14 progression, 8 stable, 10 partial response). In the other 8 readings, the computer aided reading by the radiologists made them change the disease status as defined in the reference standard to progression (6 readings) and partial response (2 readings). This was in agreement with the third reader evaluation. Automatic lesion detection and segmentations were rated as good or acceptable in 80% of the studies.
**Conclusion:** Automatic liver metastases change analysis by simultaneous comparison deep learning can improve disease classification.
**Limitations:** Studies from two institutions only
**Ethics Committee Approval:** Yes
**Funding for this study:** None
**Author Disclosures:**
Richard Lederman: Nothing to disclose
Leo Joskowicz: Nothing to disclose
Hila Fruchtman-Brot: Nothing to disclose
Adi Szeskin: Nothing to disclose
Shalom Rochman: Nothing to disclose
Jacob Sosna: Nothing to disclose
Aviv Dodi: Nothing to disclose
Yusef Azraq: Nothing to disclose

**Live Q&A**

**10:30-11:30** **CHANNEL 2**

### Refresher Course: Genitourinary

### RC 307

### The role of MRI for each therapeutic option in prostate cancer follow-up

**Moderator**
Jonathan Richenberg; Brighton/UK

### RC 307-2

**A. After radiation therapy and surgery**

Valeria Panebianco; Rome/IT
*(valeria.panebianco@uniroma1.it)*

**Learning Objectives:**
1. To understand the capabilities of MRI to identify prostate cancer recurrence after radiotherapy and radical prostatectomy.
2. To became familiar with the potential reporting/standardisation of prostate cancer recurrence after radiotherapy and radical prostatectomy.
3. To learn the clinical impact of MRI and the optimal integration with the different modalities.

### RC 307-3

**B. After focal therapy**

Jurgen J. Fütterer; Nijmegen/NL
*(jurgen.futterer@radboudumc.nl)*

**Learning Objectives:**
1. To understand the capabilities of MRI to identify prostate cancer recurrence after focal therapies.
2. To became familiar with the potential reporting/standardisation of prostate cancer recurrence.
3. To learn the clinical impact of MRI and the optimal integration with the different modalities.

**RC 307-4**
**C. During active surveillance**
Francesco Giganti; London/UK

**Learning Objectives:**
1. To understand the role of mpMRI for low and intermediate risk prostate cancer during active surveillance (AS).
2. To become familiar with the dedicated scoring (PRECISE) used to assess radiological change on serial mpMRI during AS.
3. To be aware of the current limitations of PRECISE and how to tackle the challenges ahead.

**Live Q&A: How to manage and standardise the prostate cancer follow-up with MRI**

**10:30-11:30 CHANNEL 3**

### Special Focus Session

### SF 20

### Clinical impact of artificial intelligence (AI): status report

**Moderator**
Elmar Kotter; Freiburg/DE

### SF 20-2
**AI in chest x-ray prioritisation: has it been useful?**
Carlos F. Muñoz-Nuñez; Valencia/ES
*(carlos.munoznunez@gmail.com)*

**Learning Objectives:**
1. To understand why prioritisation is useful in clinical practice.
2. To learn how AI can support prioritisation of chest x-rays.
3. To understand the limitations of AI-based prioritisation.

### SF 20-3
**Benefits of AI challenges to clinical practice: algorithms that won and made it to the clinic**
Bram Van Ginneken; Nijmegen/NL
*(bram.vanginneken@radboudumc.nl)*

**Learning Objectives:**
1. To learn about AI challenges and their organisation.
2. To understand what happens with the data collected and algorithms created during AI challenges.
3. To learn about examples of AI algorithms that made it from challenges to the clinic.

**Author Disclosures:**
Bram Van Ginneken: Research Grant/Support: Siemens Healthineers, Elekta, Sectra, Novartis, Thirona, MeVis Medical Solutions, Delft Imaging; Share Holder: Thirona

### SF 20-4
**Research action in AI: a national pilot study in France, DRIM AI**
Marc Zins; Paris/FR
*(mzins@hpsj.fr)*

**Learning Objectives:**
1. To understand the organisation of DRIM.
2. To learn about the use cases addressed by DRIM.
3. To understand what lessons can be derived from DRIM.

**Live Q&A: AI in practice and research: what are the next steps?**

**10:30-11:30 CHANNEL 4**

### ESR meets the Arabian Peninsula

### Meets 3

### Arabian Peninsula radiology: challenges and advances in regulations, education, and practice

**Moderators**
Michael H. Fuchsjäger; Graz/AT
Lamia Jamjoom; Jeddah/SA
Saqer AlTai; Muscat/OM
Usama M.H. AlBastaki; Dubai/AE

### Meets 3-5
**Saudi healthcare: new frontiers**
Sattam Saud Lingawi; Jeddah/SA
*(dr_lingawi@yahoo.ca)*

**Learning Objectives:**
1. To learn about the overall health affairs' changes planned in Saudi Arabia for vision 2030 and their impact on healthcare policymakers, providers, investors, and the general population.
2. To understand the health policies and practices' reforms in Saudi Arabia in 2020 and Saudi Arabia's new era of international openness in collaboration with international counterparts.
3. To appreciate the corrective measures in medicine in Saudi Arabia and the empowerment of youth, women, and qualified personnel in leadership, emphasising health awareness and education, active lifestyle, and preventive measures.
4. To become familiar with Saudi Arabia's private sector enablement, international investors facilitation, and public-private-partnership (PPP) programmes, transferring medical operations from government-based to private-based, starting with radiological services, teleradiology, and artificial intelligence.

**Author Disclosures:**
Sattam Saud Lingawi: Author: Health Strategic Transformation Center, Ministry of Health

### Meets 3-6
**Interlude 1: Saudi Arabia to infinity and beyond**

### Meets 3-7
**Radiology education in Oman**
Jokha Al Kalbani; Muscat/OM
*(jalkalbani@yahoo.com)*

**Learning Objectives:**
1. To provide an introduction about the evolution of the radiology education in Oman, lead both by the society and the members who are also trainers in the Omani post-graduate education board.
2. To discuss the role of the Omani Radiology society in setting regulations and imaging guidelines as well as spreading the awareness on radiation protection and safe practice in collaborations with international bodies.
3. To describe the international collaborations and emphasis on the role of the society in education and up-to-date conferences.
4. To touch on the challenges and opportunities that the society is facing both locally and internationally.
5. To provide a short description of the society's plans and the timeline for achieving them.

### Meets 3-8
**Interlude 2: Oman the past the current and the vision**

### Meets 3-9
**Challenges facing radiology in the United Arab Emirates and how to tackle them**
Taleb Almansoori; Al Ain/AE
*(taleb.almansoor@uaeu.ac.ae)*

**Learning Objectives:**
1. To introduce the evolution of the radiology sector in the United Arab Emirates.
2. To discuss the implementation of radiology science in undergraduate and post-graduate medical education.

3. To review the current residency programmes and the challenges being faced to attract medical students into the field.
4. To become familiar with the educational activities being organised for radiologists in the United Arab Emirates.

**Meets 3-10**
**Interlude 3: Cultural immersions and adventures in UAE**

**Live Q&A**

**10:30-11:30** **CHANNEL 5**

### Research Presentation Session: Hybrid, Molecular and Translational Imaging

### RPS 106

### Advances in hybrid and dual-modality imaging

**Moderator**
Sergios Gatidis; Tübingen/DE

### RPS 106-2

**Prospective comparison between 18F-FDG PET/MRI, MRI, CT and bone scintigraphy for the detection of bone metastases in the initial staging of primary breast cancer patients**
*N-M. Bruckmann*; Düsseldorf/DE

**Purpose:** To compare the diagnostic performance of 18F-FDG PET/MRI, MRI, CT and bone scintigraphy for the detection of bone metastases in the initial staging of primary breast cancer patients.
**Methods or Background:** A cohort of 154 therapy-naive patients with newly diagnosed, histopathologically proven, breast cancer was enrolled in this study prospectively. All patients underwent a whole-body 18F-FDG PET/MRI, computed tomography (CT) scan, and a bone scintigraphy prior to therapy. All datasets were evaluated regarding the presence of bone metastases. McNemar $chi^2$ test was performed to compare sensitivity and specificity between the modalities.
**Results or Findings:** Forty-one bone metastases were present in 7/154 patients (4.5%). Both 18F-FDG PET/MRI and MRI alone were able to detect all of the patients with histopathologically proven bone metastases (sensitivity 100%; specificity 100%) and did not miss any of the 41 malignant lesions (sensitivity 100%). CT detected 5/7 patients (sensitivity 71.4%; specificity 98.6%) and 23/41 lesions (sensitivity 56.1%). Bone scintigraphy detected only 2/7 patients (sensitivity 28.6%) and 15/41 lesions (sensitivity 36.6%). Furthermore, CT and scintigraphy led to false-positive findings of bone metastases in 2 patients and in 1 patient, respectively. The sensitivity of PET/MRI and MRI alone was significantly better compared with CT ($p < 0.00001$, difference 43.9%) and bone scintigraphy ($p < 0.00001$, difference 63.4%).
**Conclusion:** 18F-FDG PET/MRI and MRI are significantly better than CT or bone scintigraphy for the detection of bone metastases in patients with newly diagnosed breast cancer. Both CT and bone scintigraphy show a substantially limited sensitivity in detection of bone metastases.
**Limitations:** The number of patients with bone metastases was small.The Reference standard was also based on follow-up examinations using CT and MRI.
**Ethics Committee Approval:** This study was approved by the local ethics committee.
**Funding for this study:** Funded by Deutsche Forschungsgemeinschaft (DFG).
**Author Disclosures:**
Nils-Martin Bruckmann: Nothing to disclose

### RPS 106-3

**Ultrafast, low dose FDG oncologic whole-body PET/CT: phase 2 study findings**
*M. I. Knopp*[1], C. L. Wright[2], Y-l. Hsieh[2], E. Ives[2], K. Binzel[2], E. Folefac[2], E. D. Miller[2], P. Kneuertz[2], M. V. Knopp[2]; [1]Cincinnati, OH/US, [2]Columbus, OH/US
*(knopp.29@osu.edu)*

**Purpose:** To assess the clinical feasibility of ultrafast (2 min), low dose (185 MBq) FDG oncologic whole body imaging using digital PET/CT and adaptive regularized reconstruction in a Phase II prospective study. To perform a 2nd Phase II study at low FDG dose.
**Methods or Background:** 105 patients with an oncologic disease were prospectively enrolled prior to therapeutic interventions and FDG digital PET imaging performed 60 - 75 min p.i. using both a conventional 90s per bed position and investigational 9s per be position acquisition. The BMI was 29.5 +/- 6.8, FDG dose 5.0 +/- 0.5 and glucose level 100.4 +/- 23.9. An organ, BMI adaptive regularized reconstruction was used for the ultrafast acquisition while the 90s was performed using current system default settings. A blinded two reader, adjudicator visual and a quantitative assessment was performed.
**Results or Findings:** Due to count sparsity, regularized adaptive reconstruction was essential and enabled all cases with a BMI <35 (82/82) to be assessed as diagnostic. In the 23 cases with BMI >=35, 6 were classified as non-diagnostic , 11 as limited and 8 as diagnostic.
**Conclusion:** Ultrafast wholebody PET/CT was shown to be feasible even at low FDG dose with consistent diagnostic quality for patients with BMI <35. Further optimization of the organ adaptive regularized reconstruction may resolve the challenges at very high BMI or a compensatory increase in acquisition time should enable a consistent diagnostic quality which then needs to be validated in a Phase III trial.
**Limitations:** The selection of the adaptive PET reconstruction parameter were manually set and not yet automatically assigned based on an algorithm or DLM which will be then used in the Phase III
**Ethics Committee Approval:** Approved IRB 2013H0393, NCT02283125
**Funding for this study:** ODSA TECH 13-060, WCIBMI Development Fund, NCI R01CA195513
**Author Disclosures:**
Chadwick Lewis Wright: Nothing to disclose
Eric D Miller: Nothing to disclose
Katherine Binzel: Nothing to disclose
Michelle I Knopp: Nothing to disclose
Edmund Folefac: Nothing to disclose
Michael Vinzenz Knopp: Nothing to disclose
Yu-lung Hsieh: Nothing to disclose
Emma Ives: Nothing to disclose
Peter Kneuertz: Nothing to disclose

### RPS 106-4

**Imaging locoregional lymph nodes in newly diagnosed breast cancer patients: comparison of PET/MRI, MRI and sonography**
*J. Morawitz*[1], N-M. Bruckmann[1], F. Dietzel[1], A-K. Bittner[2], S. Mohrmann[1], L. M. Sawicki[1], K. Herrmann[2], G. Antoch[1], J. Kirchner[1]; [1]Düsseldorf/DE, [2]Essen/DE

**Purpose:** To compare breast-MRI, whole-body MRI, whole-body 18F-FDG PET/MRI and axillary sonography for the detection of axillary lymph node metastases in women with newly diagnosed breast cancer.
**Methods or Background:** This prospective double-center study included 112 patients (mean age 53.04±12.6 years) with newly diagnosed breast cancer. Patients underwent dedicated breast-MRI in prone body position, whole-body (18F-FDG PET/)MRI in supine body position and axillary sonography. Datasets were evaluated separately regarding nodal status (nodal+ vs. nodal-). Histopathology served as reference standard in all patients. The diagnostic performance of breast-MRI, whole-body MRI, whole-body PET/MRI and axillary sonography in detecting nodal positive patients was tested by creating receiver-operating-characteristic curves with a calculated area under the curve. Sensitivity, specificity, positive predictive value, negative predictive value and accuracy were calculated for all four modalities. A McNemar test was used to assess differences.
**Results or Findings:** Of all tested imaging modalities, whole-body PET/MRI showed the highest ROC-AUC with a value of 0.892. The AUC for breast-MRI, whole-body MRI and sonography were 0.782, 0.814 and 0.834, respectively. Differences between the modalities were statistically significant, e.g. whole-body PET/MRI vs. axillary sonography (p=0.013). PET/MRI showed highest sensitivity (81.8%, 95%-CI: 67.29-91.81%) while axillary sonography showed highest specificity (96.7%, 95%-CI: 80.39-99.51%). Nevertheless, all modalities missed some histopathologically proven axillary lymph node metastases.
**Conclusion:** 18F-FDG PET/MRI outperforms axillary sonography, breast-MRI and whole-body MRI in detecting axillary lymph node metastases. However, diagnostic competence does not suffice to replace histopathological sampling of axillary lymph node in primary breast cancer staging.
**Limitations:** Regarding reference standard, some samples were taken after neoadjuvant systemic therapy and therefore had to be evaluated retrospectively.
**Ethics Committee Approval:** All procedures performed were in accordance with the ethical standards of the institutional research committee.
**Funding for this study:** The study is funded by the Deutsche Forschungsgemeinschaft (DFG).
**Author Disclosures:**
Ken Herrmann: Nothing to disclose
Frederic Dietzel: Nothing to disclose
Lino M Sawicki: Nothing to disclose
Julian Kirchner: Nothing to disclose
Svjetlana Mohrmann: Nothing to disclose
Gerald Antoch: Nothing to disclose
Ann-Kathrin Bittner: Nothing to disclose

Janna Morawitz: Nothing to disclose
Nils-Martin Bruckmann: Nothing to disclose

### RPS 106-5

**Impact of PET/CT on the management of patients with oesophagal cancer: results from a PET/CT registry study**

*C. P. Reinert*, J. Sekler, C. Gani, K. Nikolaou, C. la Fougère, C. Pfannenberg, S. Gatidis; Tübingen/DE
*(christian.reinert@med.uni-tuebingen.de)*

**Purpose:** To investigate the impact of PET/CT on clinical management in patients with oesophageal cancer and its link to overall survival (OS) in a real-world setting.
**Methods or Background:** A patient cohort with advanced oesophageal cancer undergoing PET/CT was prospectively enrolled in a registry study between 04/2013 and 06/2019. Intended patient management prior and after PET/CT was documented based on standardized questionnaire data. Management changes after PET/CT were recorded including major changes concerning the treatment goal (curative vs. palliative) and minor changes (therapy adjustments). OS was analyzed for subgroups with squamous cell carcinomas (SCC) or adenocarcinomas (AC) and stratified for extent of metastatic disease and treatment goals.
**Results or Findings:** 257 patients (53 female;65.5±10.0yr.) were included. After PET/CT, major changes of intended therapy were observed in 34/257 patients (13.2%), from curative to palliative (8.2%), palliative to curative (1.9%) and from “not finally determined” to a curative (1.9%) or palliative (1.2%) concept. Minor changes were found in 62/257 patients (24.1%). Invasive procedures and additional imaging were intended in 70/257 (27.2%) and 94/257 (36.6%) patients before PET/CT and 20/257 (7.8%) and 8/257 (3.1%) patients after PET/CT. Curative therapy concepts based on PET/CT were associated with a longer OS (3.5yr.[95%CI 3.1-3.8yr.]) as compared to palliative concepts (0.9yr.[95%CI 0.6-1.2yr.];p<0.0001). Patients with SCC had a worse prognosis (2.4yr.[95%CI 2.0-2.9yr.]) as compared to patients with AC (3.2yr.[95%CI 2.7-3.7yr.];p=0.01).
**Conclusion:** In patients with advanced oesophageal cancer, PET/CT has a significant impact on clinical management by improving the selection of individualized treatment strategies and avoiding additional diagnostic procedures.
**Limitations:** Outcome data were not available for all patients.
**Ethics Committee Approval:** Reviewed and approved (Project number: 064/2013B01)
**Funding for this study:** Deutsche Forschungsgemeinschaft (DFG, German Research Foundation) under Germany's Excellence Strategy - EXC 2180-390900677
Junior Clinician Scientist Program of the Medical Faculty of Tübingen
**Author Disclosures:**
Konstantin Nikolaou: Nothing to disclose
Cihan Gani: Nothing to disclose
Sergios Gatidis: Nothing to disclose
Julia Sekler: Nothing to disclose
Christian la Fougère: Nothing to disclose
Christian Philipp Reinert: Nothing to disclose
Christina Pfannenberg: Nothing to disclose

### RPS 106-6

**Bimodal instrument markers for magnetic particle and magnetic resonance imaging**

*F. Wegner*[1], S. Cremers[2], K. Lüdtke-Buzug[1], M. A. Koch[1], T. Friedrich[1], P. Borm[2], T. M. Buzug[1], J. Barkhausen[1], M. Ahlborg[1]; [1]Lübeck/DE, [2]Düsseldorf/DE
*(franz.wegner@uksh.de)*

**Purpose:** To develop instrument markers that are visible in both, Magnetic Particle Imaging (MPI) and Magnetic Resonance Imaging (MRI).
**Methods or Background:** The instrument markers are based on two different iron oxide particle types: Resovist and Bayoxide E 8706. Varnishes containing only Resovist or both Resovist and Bayoxide E 8706 were made and applied to segments of standard nonmagnetic guide wires and catheters. Imaging experiments were performed using a preclinical, commercial MPI scanner as well as a 1 Tesla MRI system. In MPI, the guide wires were placed on a home-built nonmagnetic phantom holder. For the MRI measurements, the guide wires were placed in a silicone vessel phantom which was filled with diluted gadolinium-based contrast agent (Gadovist, 1:200).
**Results or Findings:** The bimodal markers were clearly visible in MPI and MRI. They caused circular signal voids in MRI and areas of high signal intensity in MPI. Both the signal voids as well as the areas of high signal intensity overrate the size of the markers. Markers containing Resovist alone caused only small signal voids in MRI, not sufficient for a reliable visualization.
**Conclusion:** The developed instrument markers based on two different particle types were reliably visible in MPI and MRI. This is a prerequisite for monitoring vascular interventions with MPI/MRI hybrid systems.
**Limitations:** We only investigated the markers under static conditions and did not prove the biocompatibility.
**Ethics Committee Approval:** Not applicable.
**Funding for this study:** This study was funded by the German Federal Ministry of Education and Research (BMBF) grant number 01DL17010A.
**Author Disclosures:**
Franz Wegner: Nothing to disclose
Sjef Cremers: Employee at Nano4Imaging
Kerstin Lüdtke-Buzug: Nothing to disclose
Thorsten M Buzug: Nothing to disclose
Paul Borm: Founder at Nano4Imaging
Martin A Koch: Nothing to disclose
Jörg Barkhausen: Nothing to disclose
Mandy Ahlborg: Nothing to disclose
Thomas Friedrich: Nothing to disclose

### RPS 106-7

**Feasibility and performance of free-hand single-photon computed tomography/ultrasonography for preoperative parathyroid adenoma localization: a pilot study**

*M. Champendal*, M. Jreige, M. Nicod-Lalonde, J. A. Jorge, M. Matter, G. Sykiotis, J. O. Prior; Lausanne/CH
*(Melanie.Champendal@hesav.ch)*

**Purpose:** To evaluate the feasibility of a new hybrid imaging modality, the free-hand single-photon computed tomography/ultrasonography (fhSPECT/US) for preoperative localization of parathyroid adenomas and to compare its performance with standard imaging modalities: ultrasonography and SPECT/CT.
**Methods or Background:** In this prospective pilot study, patients underwent sequentially ultrasound, parathyroid scintigraphy including SPECT/CT, and lastly fhSPECT/US allowing real-time fusion between US and gamma-camera images manipulated free-handedly by the operator. Detection rates from each imaging modality were calculated and compared using McNemar’s test; the gold standard was the histopathology result, when available, otherwise it was the imaging modality showing the highest detection rate. Challenges related to image acquisition and technique were also analyzed using descriptive methods.
**Results or Findings:** Twelve patients were included. Sensitivity of fhSPECT/US was 58%(95%CI: 32–81%) and 44%(23–67%) on a per-patient and per-lesion basis, respectively. Sensitivity results for fhSPECT/US were lower compared to US 75%(47–91%) and 63%(39–82%) and SPECT/CT 100%(75–100%) and 100%(81–100%), on per-patient and per-lesion basis, respectively. fhSPECT/US was unable to detect 9 lesions in 5 patients due to deep localization of lesions (>4.5 cm), lack of adequate image fusion due to tissue compression during US, limited spatial manipulation of the probe, and spread of detected activity.
**Conclusion:** The use of fhSPECT/US for the localization of parathyroid adenomas is clinically feasible, but requires technical improvements to increase sensitivity, which is otherwise lower than US and SPECT/CT. Technical limitations were identified and mainly related to the depth of adenoma localization and spatial manipulation of the SPECT mobile camera.
**Limitations:** The sample size, the patient population (only sick patients) and the absence of histology confirmation for all patients. Moreover, the fhSPECT/US imaging was not blinded to previous modalities results.
**Ethics Committee Approval:** This study has been approved by the local ethics committee.
**Funding for this study:** None
**Author Disclosures:**
Jose A. Jorge: Nothing to disclose
Maurice Matter: Nothing to disclose
Mario Jreige: Nothing to disclose
John O Prior: Nothing to disclose
Gerasimos Sykiotis: Nothing to disclose
Mme Marie Nicod-Lalonde: Nothing to disclose
Mélanie Champendal: Nothing to disclose

**Live Q&A**

**11:45-12:45** **CHANNEL 2**

### Lancet Oncology Commission Session

### Lancet Oncology

### Ensuring sustainable healthcare systems: The Lancet Oncology Commission on Medical Imaging and Nuclear Medicine

**Moderator**
David Collingridge; London/UK

### Lancet Oncology-3
**Goals and scope of The Lancet Oncology Commission on Medical Imaging and Nuclear Medicine**
May Abdel Wahab; Vienna/AT

**Learning Objectives:**
1. To provide information on the scope and aims of the project.
2. To provide information on the data collection methodology.
3. To provide an overview of the related efforts of the IAEA.

### Lancet Oncology-4
**Global utilisation of imaging and nuclear medicine in oncology**
Andrew Scott; Melbourne, VIC/AU

**Learning Objectives:**
1. To provide information on the results of the equipment and workforce global survey.
2. To provide information on gaps identified in low- and middle-income countries.
3. To outline technology advances that can improve the delivery of imaging to cancer patients.

### Lancet Oncology-5
**Health economic analysis of cancer imaging**
Rifat Atun; Boston, MA/US

**Learning Objectives:**
1. To outline the methodology and results of health economics analysis for imaging in cancer at a global and country level.
2. To outline the investment in imaging required for improved health outcomes.
3. To present an investment case for cancer imaging.

### Lancet Oncology-6
**Overview and recommendations**
Hedvig Hricak; New York, NY/US

**Learning Objectives:**
1. To provide a summary of results.
2. To provide recommendations for policy and investment with respect to the imaging of cancer patients at country and global levels.
3. To highlight how the commission's findings may aid in the implementation of the 2017 Cancer Resolution "Cancer prevention and control in the context of an integrated approach".

**Author Disclosures:**
Hedvig Hricak: Advisory Board: Johns Hopkins, University of Vienna, DKFZ; Board Member: IBA

**Live Q&A**

**11:45-12:45** **CHANNEL 3**

### EuroSafe Imaging Session
*Jointly organised by EuroSafe Imaging and the ECR Paediatric Subcommittee*

### EU 2

### Avoiding or reducing ionising radiation in children: the ALARA principle

**Moderator**
Erich Sorantin; Graz/AT

### EU 2-2
**An update on dose reduction techniques in paediatric CT and radiography**
Erich Sorantin; Graz/AT
*(erich.sorantin@medunigraz.at)*

**Learning Objectives:**
1. To learn about the different technical possibilities.
2. To understand how to improve the benefit/risk-ratio of children in radiography and CT.
3. To become familiar with the relevant resources for radiation protection.

### EU 2-3
**Orthopaedic imaging in children**
Jeannette Kraft; Leeds/UK

**Learning Objectives:**
1. To learn how to limit the indications for CT in skeletal imaging.
2. To learn about the possibilities of low-dose applications in skeletal imaging.
3. To appreciate the important role of ultrasound or MRI for musculoskeletal imaging in children.

### EU 2-5
**Imaging lung diseases in children and adolescents: can we avoid CT?**
Diane M. Renz; Hannover/DE
*(Renz.Diane@mh-hannover.de)*

**Learning Objectives:**
1. To appreciate the role of radiography.
2. To become familiar with the role of ultrasound in lung imaging.
3. To learn about the advantages of lung MRI.

### EU 2-6
**Avoiding radiation in our smallest patients**
Willemijn M. Klein; Nijmegen/NL
*(willemijn.klein@radboudumc.nl)*

**Learning Objectives:**
1. To learn about alternatives to radiography.
2. To learn tips an tricks to minimise ionising radiation when needed.
3. To learn the ALARA and image gently principles.

**Live Q&A**

**11:45-12:45** **CHANNEL 4**

### Special Focus Session

### SF 25

### Interventional radiology (IR) treatment for arterial occlusive disease and thrombo-embolic disease: lessons learned from head to toe

**Moderator**
Elias N. Brountzos; Athens/GR

### SF 25-2
**Revascularisation in acute ischaemic stroke**
Gregoire Boulouis; Paris/FR

**Learning Objectives:**
1. To learn how to select suitable candidates for mechanical stroke thrombectomy (MST) on the basis of the clinical and imaging evaluation.
2. To learn about basic techniques of MST and the most used tools.
3. To learn about the results and complications.

**Author Disclosures:**
Gregoire Boulouis: Advisory Board: Cerenovus 2020

### SF 25-3
**Aorta and aortic branches interventions: current evidence**
Viktor Bérczi; Budapest/HU
*(berczi@hotmail.com)*

**Learning Objectives:**
1. To learn about the indications, techniques, results, and complications of interventions for isolated aortic stenoses.
2. To learn about the indications for IR treatment of athero-occlusive lesions of the innominate and subclavian arteries (aortic arch arteries).
3. To understand the results of IR treatment of the athero-occlusive lesions of the renal arteries.

### SF 25-4
**How to treat acute and chronic mesenteric ischaemia**
Lorenzo Garzelli; Paris/FR

**Learning Objectives:**
1. To learn how to select patients with acute arterial mesenteric ischaemia for IR treatment on the basis of clinical and imaging evaluations.
2. To understand the techniques used in IR treatment of acute arterial mesenteric ischaemia and to learn about the results and complications.
3. To understand the indications for IR treatment of patients with chronic mesenteric ischaemia.
4. To learn about the techniques, results, and complications of IR treatment for chronic mesenteric ischaemia.

### SF 25-5
**Endovascular treatment of the diabetic foot: new techniques**
Marco Manzi; Padua/IT
*(marcodocmanzi@gmail.com)*

**Learning Objectives:**
1. To learn about the indications of innovative arterial access for diabetic foot IR treatment, i.e. pedal access.
2. To learn about the new tools for treating long total occlusions.
3. To learn about the indications and techniques of lithotripsy.
4. To understand the role of drug-eluting technology.
**Author Disclosures:**
Marco Manzi: Consultant: Abbott vascular, Angiodroid, BdBard,Boston Sc.,CID Alvimedica,Cook, Terumo

**Live Q&A: Are IR techniques evolving as the first-line treatment modality for atheromatous disease? Are there any exceptions to the rule?**

**11:45-12:45 CHANNEL 5**

### E³ - Rising Stars Programme: Basic Sessions
*Organised by the ESR*

### BS 11
### Musculoskeletal: lower extremities

**Moderator**
Mario Maas; Amsterdam/NL

### BS 11-2
**The degenerative hip**
Üstün Aydingöz; Ankara/TR
*(uaydingo@hacettepe.edu.tr)*

**Learning Objectives:**
1. To learn about imaging methods in degenerative hip evaluation.
2. To learn about imaging features in the degenerative hip.

### BS 11-3
**The degenerative knee**
Jan L.M.A. Gielen; Antwerp/BE
*(jan.gielen@uza.be)*

**Learning Objectives:**
1. To learn about imaging methods in degenerative knee evaluation.
2. To learn about imaging features in the degenerative knee.

### BS 11-4
**The degenerative ankle**
Apostolos H. Karantanas; Iraklion/GR
*(akarantanas@gmail.com)*

**Learning Objectives:**
1. To learn about imaging methods in degenerative ankle evaluation.
2. To learn about imaging features in the degenerative ankle.

**Live Q&A**

**14:15-15:15 CHANNEL 2**

### Joint Session of the ESR and EFOMP

### ESR/EFOMP
### High-field vs low-field MRI: time for a re-think?

**Moderators**
David J. Lurie; Aberdeen/UK
Marion Smits; Rotterdam/NL

### ESR/EFOMP-3
**Advantages of 0.5 Tesla and below: physics and technology**
Andrew Webb; Leiden/NL
*(a.webb@lumc.nl)*

**Learning Objectives:**
1. To become familiar with the low-field MRI technology.
2. To explore the advantages of low-field MRI.
3. To understand the drawbacks of low-field MRI.

### ESR/EFOMP-4
**Clinical benefits of low-field MRI**
Mathieu Sarracanie; Allschwil/CH
*(mathieu.sarracanie@unibas.ch)*

**Learning Objectives:**
1. To become familiar with the clinical applications of low-field MRI.
2. To understand the benefits of low-field MRI of the heart.
3. To understand the disadvantages of low-field MRI for clinical application.
**Author Disclosures:**
Mathieu Sarracanie: Founder: Hyperfine; Patent Holder: Hyperfine, Siemens

### ESR/EFOMP-5
**Advantages of 7 Tesla and above: physics and technology**
David Brunner; Zurich/CH
*(david.brunner@skope.ch)*

**Learning Objectives:**
1. To become familiar with the high-field MRI technology.
2. To explore the advantages of high-field MRI.
3. To understand the drawbacks of high-field MRI.
**Author Disclosures:**
David Brunner: Board Member: ISMRM Engineering Study Group; Employee: Skope MRT

### ESR/EFOMP-6
**Clinical benefits of high-field MRI**
Anja van der Kolk; Utrecht/NL
*(A.G.vanderKolk@umcutrecht.nl)*

**Learning Objectives:**
1. To become familiar with the clinical applications of high-field MRI.
2. To understand the benefits of high-field MRI of the brain.
3. To understand the disadvantages of high-field MRI for clinical application.

**Live Q&A: Does clinical value scale with magnetic field strength?**

14:15-16:30 CHANNEL 3

### E³ - Rising Stars Programme: Case-Based Diagnosis Training

### CB

### Case-Based Diagnosis Training

**Moderators**
Soraya Robinson; Vienna/AT
Klaus M. Friedrich; Vienna/AT

### CB-3
**Liver**
Asunción Torregrosa Andrés; Valencia/ES
*(asunsuso@gmail.com)*

### CB-4
**Neuro**
Daniela Prayer; Vienna/AT

### CB-5
**Musculoskeletal**
Franz Kainberger; Vienna/AT

### CB-6
**Maxillofacial**
Soraya Robinson; Vienna/AT
*(s.robinson@dzu.at)*

### CB-7
**Genitourinary**
Michael Toepker; Vienna/AT
*(mt@dz10.at)*

### CB-8
**Interlude: Air in the wrong place**
Burce Ozgen Mocan; Chicago, IL/US

### CB-10
**Head and neck**
Christian Czerny; Vienna/AT
*(christian.czerny@meduniwien.ac.at)*

### CB-11
**Chest**
Helmut Prosch; Vienna/AT
*(helmut.prosch@meduniwien.ac.at)*

### CB-12
**Spine**
Klaus M. Friedrich; Vienna/AT

### CB-13
**Gastrointestinal**
Wolfgang Schima; Vienna/AT
(wolfgang.schima@khgh.at)

### CB-14
**Breast**
Fleur Kilburn-Toppin; Cambridge/UK
*(fleur.kilburn-toppin@addenbrookes.nhs.uk)*

14:15-15:15 CHANNEL 5

### Research Presentation Session: Breast

### RPS 102

### Advances in breast cancer screening with digital breast tomosynthesis (DBT), artificial intelligence (AI), and MRI

**Moderator**
Fredrik Strand; Stockholm/SE

### RPS 102-2
**Using an AI system and breast density to quantify the short-term risk of interval cancer in a large screening cohort**
A. J. Wanders[1], W. Mees[1], N. Janssen[2], M. U. Dalmis[2], A. Rodriguez Ruiz[2], I. Sechopoulos[2], C. van Gils[3], R. Mann[2], *J-K. Van Rooden*[1]; [1]The Hague/NL, [2]Nijmegen/NL, [3]Utrecht/NL
*(j.vanrooden@hagaziekenhuis.nl)*

**Purpose:** To create a short-term breast cancer risk model combining the assessments of an artificial intelligence (AI) detection system and breast density (BD) to assess the risk of interval cancers (IC) in screening mammograms.
**Methods or Background:** This nested case-control study included DM exams of 2332 IC cases of women that participated in the Dutch breast cancer screening program, and a randomly selected group of 584 normal screening controls verified by at least 2 years of follow-up. For each mammogram, a score 1-10 indicating the risk of presence of visible abnormalities was computed by an AI system (Transpara, Screenpoint Medical), and BD was computed using automated software. A neural network-based model was trained combining the AI score and BD to predict the short-term risk of IC in screening mammograms using 10-fold cross-validation. The area under the receiver operating characteristics curve (AUC) of the AI system, breast density, and the risk model for selecting women at risk for developing IC at immediately preceding screening mammograms was estimated with 95% confidence intervals and compared using T-test for paired samples.
**Results or Findings:** The AUC of the neural-network risk model to detect IC in screening was 0.801 (0.774-0.827), higher (P<0.01) than the AI system or BD alone (0.741 (0.713-0.769) and 0.695 (0.660-0.730), respectively). At 90% specificity, the risk model achieved a sensitivity of 49.7% (43.2%-56.2%), higher (P<0.01) than the AI system or BD alone, (42.2% (37.1%-47.4%) and 23.2% (16.1-30.3%), respectively).
**Conclusion:** Combining breast density and AI assessments improves the selection of women at risk for developing IC over either method alone, allowing for the correct prediction of about half of the IC at 90% specificity.
**Limitations:** The risk model needs validation on external cohorts.
**Ethics Committee Approval:** Approved by institutional review board
**Funding for this study:** Not applicable
**Author Disclosures:**
Willem Mees: Nothing to disclose
Mehmet Ufuk Dalmis: Employee at ScreenPoint Medical
Carla van Gils: Nothing to disclose
Alejandro Rodriguez Ruiz: Employee at ScreenPoint Medical
Natasja Janssen: Employee at ScreenPoint Medical
Ritse Maarten Mann: Consultant at ScreenPoint Medical
Alexander J.T. Wanders: Nothing to disclose
Ioannis Sechopoulos: Nothing to disclose
Jan-Kees Van Rooden: Nothing to disclose

### RPS 102-3
**Using AI with single reading in screening: a simulation of the impact on tumour characteristics of detected cancers**
N. Janssen, S. van Winkel, A. Rodriguez Ruiz, N. Karssemeijer, I. Sechopoulos, *R. Mann*; Nijmegen/NL
*(r.mann@rad.umcn.nl)*

**Purpose:** To compare the tumour characteristics of screen-detected cancers when one human reader is replaced by an artificial intelligence (AI) system in a double reading breast cancer screening program.
**Methods or Background:** A consecutive cohort of 23,035 digital mammography exams with 159 screen-detected (SD), 48 interval (IC) and 62 next-round-SD cancers (NRSD) was collected from the Dutch screening program. An AI system (Transpara, ScreenPoint Medical) processed all exams and assigned a recall/no recall decision at the same recall rate as a radiologist (3.0%). Histopathological types (invasive ductal/lobular carcinoma (IDC/ILC), ductal carcinoma in situ (DCIS)), grade, and mean radiological size (95% CI) of all cancers were reported for double reading and single reading + AI.

**Results or Findings:** Double reading detected 159 SD, with mean size of 13.6mm (95% CI:12.4-14.9mm). These were 61.0% IDC, 10.1% ILC, 19.5% DCIS, 7.5% other invasive and 1.9% unknown. Single reading + AI recalled 179 cancers (154 SD, 15 IC, 10 NRSD), with mean size 14.2mm (95% CI:13.1-15.4mm) and 62% IDC, 12.8% ILC, 16.2% DCIS, 7.3% other invasive and 1.7% unknown. Double reading and single reading + AI detected similar proportions of grade-3 cancers: 25% vs. 21.8% (p=0.48). The five SD that were missed by single reading + AI were all grade-3, (3 DCIS, 1 ILC, 1 IDC). The 15 recalled IC by single reader + AI were all invasive, and 20% were grade-3, 33% grade-2, 40% grade-1 and 7% unknown. Of the 10 NRSD, 10% were grade-3, 20% grade-2, 60% grade-1 and 10% unknown, with 1 DCIS.
**Conclusion:** Introducing AI in screening could lead to higher cancer detection rates with similar tumour characteristics as with double reading of mammograms.
**Limitations:** No visual examination of identified AI findings was performed.
**Ethics Committee Approval:** Institutional review board.
**Funding for this study:** Not applicable.
**Author Disclosures:**
Suzanne van Winkel: Nothing to disclose
Alejandro Rodriguez Ruiz: Employee at ScreenPoint Medical
Natasja Janssen: Employee at ScreenPoint Medical
Ritse Maarten Mann: Consultant at ScreenPoint Medical
Ioannis Sechopoulos: Nothing to disclose
Nico Karssemeijer: CEO at ScreenPoint Medical

### RPS 102-4

**The potential of AI to replace a first reader in a double reading breast cancer screening programme: a feasibility study**

N. Janssen[1], A. Rodriguez Ruiz[1], C. Mieskes[2], N. Karssemeijer[1], *S. H. Heywang-Köbrunner*[2]; [1]Nijmegen/NL, [2]Munich/DE
*(sylvia.heywang@referenzzentrum-muenchen.de)*

**Purpose:** To evaluate the impact of replacing the double human reading of digital mammograms (DM) with a single human reading and an artificial intelligence (AI) system in a breast cancer screening programme.
**Methods or Background:** A randomly selected screening cohort of 2314 DM exams with 114 biopsy-proven screen-detected cancers was retrospectively collected from a single center, which involved independent double human reading and consensus in case of disagreement. Normal mammograms were defined as free of screen-detected cancer by double reading. No follow-up information about interval cancers was available. All exams were processed by an AI system (Transpara, ScreenPoint Medical), assigning an Exam Score 1-10 which represents an increasing risk of containing visible abnormalities. An AI-based screening scenario was retrospectively investigated in which the second reader was replaced with AI (operating at the same sensitivity). Specificity and workload were compared between original double reading and the AI-based scenario using a McNemar test.
**Results or Findings:** Double human reading recalled 505 women to consensus, with 112 detected cancers and 393 false positives (specificity 82.1%, CI: 80.5-83.7). AI-based screening resulted in 550 recalled women to consensus, with 112 detected cancers and 438 false positives (specificity 80.1%, CI: 78.4-81.7, relative reduction compared to double reading of 2.0%, CI: -0.3-4.3%, P=0.08).
**Conclusion:** Replacing a human reader with AI in a double reading breast cancer screening programme could be an effective strategy to halve radiologists workload while maintaining the same sensitivity, particularly in case of a shortage of readers. This strategy potentially comes at the cost of more false positive cases sent to consensus.
**Limitations:** Due to the retrospective character of this study, it was not possible to assess the benefit of AI in an actual screening setting.
**Ethics Committee Approval:** IRB approval was obtained
**Funding for this study:** Not applicable
**Author Disclosures:**
Sylvia H. Heywang-Köbrunner: Nothing to disclose
Alejandro Rodriguez Ruiz: Employee at ScreenPoint Medical
Natasja Janssen: Employee at ScreenPoint Medical
Christoph Mieskes: Nothing to disclose
Nico Karssemeijer: CEO at ScreenPoint Medical

### RPS 102-5

**The potential for AI to replace a reader in a double reading breast cancer screening programme**

S. van Winkel, N. Janssen, A. Rodriguez Ruiz, N. Karssemeijer, I. Sechopoulos, *R. Mann*; Nijmegen/NL
*(r.mann@rad.umcn.nl)*

**Purpose:** To evaluate the impact of replacing the double human reading with a single human reading and an artificial intelligence (AI) system in a breast cancer screening programme.
**Methods or Background:** A consecutive cohort of 23,035 digital mammography (DM) exams with 159 screen-detected (SD), 48 interval (IC) and 62 next-round SD cancers (NRSD) was collected from the Dutch screening programme, which involved double human reading. An AI system (Transpara, ScreenPoint Medical) processed all cases, assigning a level of suspicion to each exam on a continuous scale. AI-based screening was retrospectively investigated in which the second reader was replaced with AI, using the human recall rate as operating point. Sensitivity and recall rate were computed for the first reader, AI alone, AI-based screening and double reading.
**Results or Findings:** The first reader recalled 694 cases (3.0%, CI = 2.8-3.2%), including 138 SD, 2 IC and 1 NRSD (sensitivity 52.4%, CI =46.3-58.5%). AI alone detected 127 SD, 14 IC and 10 NRSD (sensitivity 56.1%, CI = 50.0-62.2%). There were 1012/23035 (4.4%) discrepant assessments between AI and the first reader. AI-based screening recalled 1200 cases (5.2%, 95% CI = 4.9-5.5%), detecting 154 SD, 15 IC and 10 NRSD (sensitivity 66.5%, CI = 60.6-72.2%). Double reading (including consensus) recalled 724 cases (3.1%, CI = 2.9-3.4%), with 159 SD (sensitivity 59.1%, CI = 53.0-65.0%).
**Conclusion:** The use of AI as independent reader could halve the radiologists workload and potentially increase sensitivity and lower recall rate, if an effective arbitration process can be implemented to assess discrepant exams.
**Limitations:** Due to incomplete data we were unable to include all NRSD in this series, hence the sensitivity of the AI system may be underestimated.
**Ethics Committee Approval:** Approved by the institutional review board
**Funding for this study:** Not applicable
**Author Disclosures:**
Suzanne van Winkel: Nothing to disclose
Alejandro Rodriguez Ruiz: Employee at ScreenPoint Medical
Natasja Janssen: Employee at ScreenPoint Medical
Ritse Maarten Mann: Consultant at ScreenPoint Medical
Ioannis Sechopoulos: Nothing to disclose
Nico Karssemeijer: CEO at ScreenPoint Medical

### RPS 102-6

**Interval breast cancer in a screening programme: comparison between women screened with two-dimensional digital mammography or tomosynthesis**

*C. Pulido Carmona*, S. Romero Martin, J. L. L. Raya Povedano, E. Elías Cabot, M. Álvarez Benito; Cordoba/ES

**Purpose:** Several studies have demonstrated that digital breast tomosynthesis (DBT) in the screening setting is associated with an increase of cancer detection rates with fewer false-positive findings compared to two-dimensional digital mammography (DM). However, the few studies published to date do not show significant differences in the rate of interval cancers. The aim of this study is to determine if the rate of interval breast cancers differ for DM versus DBT.
**Methods or Background:** Screening studies carried out in a screening unit from January 2015 to December 2016 were reviewed retrospectively. Different sources were examined in order to find the screening-detected cancers (if diagnosed within 2 years of a positive screening examination) and interval cancers (if diagnosed withing 2 years of a negative screening examination). Screening outcomes such as interval cancer rate (ICR/1000 negative screens) and screening sensitivity were calculated. Moreover, cancers characteristics were compared (DM inverval cancers vs. DBT interval cancers and, on the other hand, interval cancers vs. screening-detected cancers).
**Results or Findings:** 43256 women were screened, 24863 with DM and 18393 with DBT. 309 breast cancers were diagnosed: 247 were screening-detected cancers and 62 interval cancers. The rate of interval cancers was higher for DM than for DBT (1.94 vs. 0.76 per 1000 negative examinations, p=.001). Screening sensitivity of DBT was higher than DM (87% vs. 75%, p=.008). There were no significant differences in tumor charateristics between interval cancers on DM versus DBT (p=.051-1). Nevertheless, there were significant differences (p<.05) in tumor characteristics between interval cancers and screening-detected cancers showing worse prognosis characteristics the first ones.
**Conclusion:** The rate of interval cancers is lower for DBT than for DM. Interval cancers show worse prognosis characteristics in comparison with screening-detected cancers.
**Limitations:** Explained later
**Ethics Committee Approval:** No
**Funding for this study:** None
**Author Disclosures:**
Marina Álvarez Benito: Nothing to disclose
Cristina Pulido Carmona: Nothing to disclose
Sara Romero Martin: Nothing to disclose
Esperanza Elías Cabot: Nothing to disclose
Jose Luis Raya Povedano: Nothing to disclose

### RPS 102-7

**Re-attendance in the second breast MRI screening round of the DENSE trial and reasons for discontinuation**

*S. G. A. Veenhuizen*, I. Laseur, M. F. Bakker, S. V. de Lange, R. M. Pijnappel, W. B. Veldhuis, C. van Gils; Utrecht/NL
*(s.g.a.veenhuizen@umcutrecht.nl)*

**Purpose:** To describe determinants of re-attendance and reasons for discontinuation in the second screening MRI round of the DENSE trial among women with extremely dense breast tissue.
**Methods or Background:** Of the women participating in the first screening round, we calculated the re-attendance rate in the second round according to subgroups of patient characteristics. Women who discontinued MRI screening, were asked to provide one or more reasons for this.
**Results or Findings:** Women who had a negative mammography two years after the first MRI screening round (4310/4783= 90.1%), were re-invited for MRI. The re-attendance rate was 71.8% among all women, and 79.7% among those re-invited. The re-attendance rate was higher among women with higher education, a healthier lifestyle, and women who experienced little or no pain, discomfort or anxiety at the first MRI. Re-attendance was lower in women with a false-positive MRI in the first screening round (65.8%) than in women with a negative MRI (74.5%, p-value=0.01). Women mostly mentioned MRI-related inconveniences as a reason to discontinue screening (43.7%).
**Conclusion:** Women of lower educational status, or who had an unpleasant MRI experience in the previous screening round were less likely to re-attend in another screening round. More emphasis should be put on improving MRI experience to increase the re-attendance rate in supplemental MRI screening.
**Limitations:** 5.5% of the women did not re-attend mammography for unknown reasons and thus were not re-invited for MRI.
**Ethics Committee Approval:** Approved by Dutch Minister of Health, Welfare and Sport (2011/19); participants gave written Informed Consent.
**Funding for this study:** UMC Utrecht, ZonMw, Dutch Cancer Society, Dutch Pink Ribbon/A Sister's Hope, Stichting Kankerpreventie Midden-West, Bayer AG Pharmaceuticals, and in kind by Volpara Health Technologies.
**Author Disclosures:**
Stéphanie Vera de Lange: Author at UMC Utrecht
Carla van Gils: Consultant at Bayer, Grant Recipient at All are mentioned in the abstract
Stefanie Gerlinda Anita Veenhuizen: Author at UMC Utrecht
Isabelle Laseur: Author at UMC Utrecht
Wouter B. Veldhuis: Other at Imaging department UMC Utrecht
Ruud M Pijnappel: Other at Director at the LRCB
Marije F. Bakker: Author at UMC Utrecht

**Live Q&A**

**15:30-16:30 CHANNEL 2**

### ESR Audit and Standards Session

### ESR Audit

### The role of clinical audit in radiology service improvement

**Moderator**
David C. Howlett; Eastbourne/UK

### ESR Audit-2
**Radiology service improvement: what can be learned from industry?**
Steven Lee; Guildford/UK (steven.lee_1@philips.com)

**Learning Objectives:**
1. To understand the concepts of quality/service improvement.
2. To appreciate the experience of industry in service improvement.
3. To highlight the benefits for radiology departments of effective processes of quality improvement.

### ESR Audit-3
**The EU Basic Safety Standards Directive and related clinical audit requirements**
Alexandra Karoussou-Schreiner; Luxembourg/LU (alexandra.schreiner@ms.etat.lu)

**Learning Objectives:**
1. To appreciate the roles of HERCA and inspection.
2. To highlight the importance of clinical audit as defined within the EU-BSS.
3. To consider the findings and implications of ESR EU-BSS uptake surveys.

### ESR Audit-4
**Development of European radiological clinical audit: the role of the national societies**
Andrea G. Rockall; London/UK (a.rockall@imperial.ac.uk)

**Learning Objectives:**
1. To review the structure and function of the ESR national societies network.
2. To appreciate the concepts of internal vs external audit and audit with external direction.
3. To consider future development of pan-European radiology clinical audit networks.

**Live Q&A: Quality improvement and radiology: how to enhance clinical audit practice?**

**15:30-16:30 CHANNEL 4**

### E³ - ECR Master Class

### E³ 226

### Radiomics and radiogenomics in breast cancer

**Moderator**
Thomas H. Helbich; Vienna/AT

### E³ 226-2
**A. What is it and how does it work?**
Katja Pinker-Domenig; New York, NY/US
*(pinkerdk@mskcc.org)*

**Learning Objectives:**
1. To understand the definitions of radiomics and radiogenomics.
2. To understand how the information is generated from images and genomics.
3. To learn about the newest literature on the topic.
**Author Disclosures:**
Katja Pinker-Domenig: Board Member: EUSOBI; Research Grant/Support: Research Grants: * Digital Hybrid Breast PET/MRI for Enhanced Diagnosis of Breast Cancer (HYPMED). H2020 - Research and Innovation Framework Programme PHC-11-2015 # 667211-2. Role: Co-PI * A Body Scan for Cancer Detection using Quantum Technology (CANCERSCAN) H2020-FETOPEN-2018-2019-2020-01 # 828978. Role: Co-PI * Multiparametric 18F-Fluoroestradiol PET/MRI coupled with Radiomics Analysis and Machine Learning for Prediction and Assessment of Response to Neoadjuvant Endocrine Therapy in Patients with Hormone Receptor+/HER2+ Invasive Breast Cancer. Jubilee Funds of the Austrian National Bank # Nr: 18207. Role: PI * Deciphering breast cancer heterogeneity and tackling the hypoxic tumor microenvironment challenge with PET/MRI, MSI and radiomics. The Vienna Science and Technology Fund # LS19-046. Role: PI Katja Pinker is supported in part through the NIH/NCI Cancer Center Support Grant P30 CA008748 and a grant from the Breast Cancer Research Foundation.; Speaker: - European Society of Breast Imaging - Siemens Healthineers - IDKD 2019

### E³ 226-3
**B. Can it be clinically useful?**
Matthias Dietzel; Erlangen/DE

**Learning Objectives:**
1. To understand the current challenges in estimating prognosis in breast cancer.
2. To appreciate when radiomics and radiogenomics can add to the clinical picture.
3. To learn about their potential role for personalised medicine.

### E³ 226-4
**C. AI-improved breast imaging "omics"**
Ritse M. Mann; Nijmegen/NL
*(r.mann@rad.umcn.nl)*

**Learning Objectives:**
1. To learn about the basic definitions of AI.
2. To discuss the possible applications of AI beyond detection.
3. To appreciate when the addition of AI may improve "omics".
**Author Disclosures:**
Ritse M. Mann: Advisory Board: Bayer, BD, Screenpoint, Transonic imaging, Siemens; Grant Recipient: Bayer, BD, Siemens, Screenpoint, Koning, Medtronic, Volpara, Seno medical

**Live Q&A: Are we ready to integrate prognostic imaging into the tumour board?**

**15:30-16:30 CHANNEL 5**

### CTiR 1
### Clinical Trials in Radiology 1

**Moderators**
Marc Dewey; Berlin/DE
Regina G.H. Beets-Tan; Amsterdam/NL

### CTiR 1-3
**Utilization of coronary CT angiography across Europe in the last decade: insights from the European ESCR MR/CT registry**

*J. Uhlig*[1], B. Foldyna[2], L. Lehmkuhl[3], L. Natale[4], R. Vliegenthart[5], J. Lotz[1], M. Gutberlet[6]; [1]Goettingen/DE, [2]Boston, MA/US, [3]Bad Neustadt/DE, [4]Roma/IT, [5]Groningen/NL, [6]Leipzig/DE
*(johannes.uhlig@med.uni-goettingen.de)*

**Purpose:** To analyze the utilization of coronary computed tomography angiography (CTA) across Europe in the last decade.
**Methods or Background:** The multinational, multicenter MR/CT Registry of the European Society of Cardiovascular Radiology (ESCR) was queried for CTA studies performed for suspected coronary artery disease (CAD) in adults between 1/2010 and 12/2019. CTAs, proportions between academic vs. non-academic centers, patient and scan characteristics between 2010–2014 vs. 2015–2019 were compared.
**Results or Findings:** Overall, 215 sites submitted 64,387 coronary CTAs (mean 300 CTAs/site). The number of sites and cases/site increased by 76% and 46% from 2010-2014 to 2015-2019. While academic centers submitted most records (53%) in 2010–2014, non-academic centers dominated the second study half (71% in 2015–2019). Initially, most CTAs were performed in elderly men (men: 60.3%; mean age: 60.4 years) with high (31%) and intermediate (63%) pretest probability, resulting in a relatively high prevalence of any CAD (42%) and multivessel disease (62%).From 2015-2019, 95% of patients had low or intermediate PTP with a slightly lower prevalence of any CAD (41%, p=0.03) but substantially less advanced disease (56% multivessel disease, p<0.001). Overall image quality was excellent or good in 90.1% of cases at relatively low radiation exposure (median 5.7 mSv) and a low number (0.4%) of acute adverse scan-related events.
**Conclusion:** Coronary CTA is increasingly utilized across Europe, gradually driven by non-academic centers. CTA-performing sites increasingly adhere to guidelines, reflected by the increasing use of coronary CTA for CAD exclusion in those with low and intermediate PTP. Despite widespread utilization, image quality and favorable acute safety profile are maintained.
**Limitations:** Limitations include missing details on CAD extent, clinical patient presentation and long-term follow-up.
**Ethics Committee Approval:** Leipzig University IRB.
**Funding for this study:** The ESCR MRCT Registry received unrestricted educational funding from Siemens, Bayer, Philips, and Bracco.
**Author Disclosures:**
Lukas Lehmkuhl: Nothing to disclose
Matthias Gutberlet: Speaker at Bayer HealthCare, Bracco Imaging, Siemens HealtCare, Philips Healthcare, GE Healthcare, Circle Health
Rozemarijn Vliegenthart: Nothing to disclose
Luigi Natale: Nothing to disclose
Johannes Uhlig: Speaker at Bayer Health
Borek Foldyna: Nothing to disclose
Joachim Lotz: Nothing to disclose

### CTiR 1-4
**Discussant**
Mecit Kantarci; Erzurum/TR
*(akkanrad@hotmail.com)*
**Author Disclosures:**
Mecit Kantarci: Nothing to disclose

### CTiR 1-5
**Coronary computed tomography versus invasive coronary angiography in patients with stable chest pain referred for invasive coronary angiography with intermediate pretest probability of coronary artery disease (DISCHARGE): 1-year follow-up of an open-label, parallel-group, multicentre trial**

*P. Maurovich-Horvat*[1], M. Bosserdt[2], K. F. Kofoed[3], M. Dewey[2], on behalf of The DISCHARGE Trial Investigators[2]; [1]Budapest/HU, [2]Berlin/DE, [3]Copenhagen/DK
*(maurovich-horvat.pal@med.semmelweis-univ.hu)*
***No abstract available***
**Author Disclosures:**
Klaus F. Kofoed: Grant Recipient at Rigshospitalet, University Copenhagen, AP Møller og hustru Chastine McKinney Møllers Fond, Danish Heart Foundation, Danish Agency for Science, Technology and Innovation and Canon Medical Corporation Research/Grant Support at EU grant (EC-GA 603266).
Pál Maurovich-Horvat: Research/Grant Support at EU grant (EC-GA 603266). Other at Neumann Medical Ltd.
Marc Dewey: Patent Holder at Patent on fractal analysis of perfusion imaging (jointly with Florian Michallek, PCT/EP2016/071551, USA Notice of Allowance 15/759,586) licensed to Florian Michallek and Marc Dewey. Research/Grant Support at EU grant (EC-GA 603266). Grant Recipient at DFG (Heisenberg Programme, BIOQIC, Fractal analysis myocardial perfusion, SPP-Radiomics), Berlin University Alliance, Berlin Institute of Health. Equipment Support Recipient at Institutional master research agreements with Siemens, General Electric, Philips and Canon.
on behalf of The DISCHARGE Trial Investigators: Research/Grant Support at EU grant (EC-GA 603266).
Maria Bosserdt: Research/Grant Support at EU grant (EC-GA 603266).

### CTiR 1-6
**Discussant**
Michelle Williams; Edinburgh/UK
**Author Disclosures:**
Michelle Williams: Nothing to disclose

### CTiR 1-7
**Incidental findings on routine preoperative unenhanced chest computed tomography prior to cardiac surgery in the multicenter randomized controlled CRICKET trial**

*W. G. Knol*[1], A. den Harder[2], L. de Heer[2], M. Polos[3], P. Maurovich-Horvat[3], T. Leiner[2], G. P. Krestin[1], A. Bogers[1], R. P. J. Budde[1]; [1]Rotterdam/NL, [2]Utrecht/NL, [3]Budapest/HU
*(w.knol@erasmusmc.nl)*

**Purpose:** To describe the prevalence of incidental findings when implementing routine screening for aortic calcification using unenhanced computed tomography prior to cardiac surgery.
**Methods or Background:** In the multicenter randomized controlled CRICKET study 862 adult patients scheduled for cardiac surgery were randomized 1:1 to undergo a preoperative unenhanced chest CT-scan in addition to standard of care (SoC+CT) or standard of care (SoC) alone, which included a chest-radiograph. In this sub-analysis, all incidental findings on the chest radiograph and CT-scan were included if preoperative work-up or follow-up was required.
**Results or Findings:** Incidental findings were observed in 11.0% (n=47, 95% CI [8.2% - 14.3%]) of patients in the SoC+CT group and in 3.7% (n=16, 95% CI [2.1% - 6.0%]) of patients in the SoC-group (p<0.001). In the SoC+CT group further preoperative work-up was required in 3.3% (n=14), vs 1.4% (n=6) in the SoC-group (p=0.07). In the SoC+CT-group, clinically relevant findings were observed in 1.2% (n=5): a lung cancer urging change from surgery to a percutaneous approach (n=2); sarcoidosis (n=1); an aortic aneurysm requiring concomitant surgery (n=1); and a patient with splenomegaly and liver cirrhosis, ultimately leading to cancellation of surgery (n=1). In the remaining nine patients no consequent treatment was required. Follow-up was required in 7.7% (n=33) of SoC+CT patients, because of pulmonary nodules (n=17), pulmonary ground glass opacities (n=5), other pulmonary abnormalities (n=5), mediastinal lymphadenopathy (n=1) or non-pulmonary findings (n=5).
**Conclusion:** In this population of patients scheduled for cardiac surgery the addition of routine CT-screening will lead to clinically relevant incidental findings in 1.2% of patients. This comes at a cost of additional diagnostic imaging and the need to follow-up on incidental findings of unclear significance in about a tenth of patients.
**Limitations:** n/a
**Ethics Committee Approval:** MEC-UMC Utrecht: 13-692/M
**Funding for this study:** ZonMW-Grantno. [837001403]
**Author Disclosures:**
Ricardo P. J. Budde: Nothing to disclose
Annemarie den Harder: Nothing to disclose
Wiebe G Knol: Nothing to disclose
Ad Bogers: Nothing to disclose
Tim Leiner: Nothing to disclose
Gabriel P. Krestin: Nothing to disclose
Linda de Heer: Nothing to disclose
Pál Maurovich-Horvat: Nothing to disclose
Miklos Polos: Nothing to disclose

### CTiR 1-8
**Discussant**
Monika Arzanauskaite; Liverpool/UK
**Author Disclosures:**
Monika Arzanauskaite: Nothing to disclose

### CTiR 1-9
**Combination of coronary computed tomography angiography and clinical probability for the prediction of obstructive coronary artery disease: international, multicenter meta-analysis**
*V. Wieske*, O. N. behalf of Collaborative Meta-Analysis of Cardiac CT (COME-CCT) Consortium; Berlin/DE
*(viktoria.wieske@charite.de)*

**Purpose:** To investigate if the combination of coronary computed tomography angiography (CTA) and pragmatic clinical probability models improves the prediction of the presence of obstructive coronary artery disease (CAD).
**Methods or Background:** Overall, 5332 patients who were clinically referred to invasive coronary angiography (ICA) with suspected CAD based on stable chest pain in 65 prospective diagnostic accuracy studies were included in this prospective multicenter analysis. Pragmatic estimation of clinical probability of obstructive CAD was based on age, sex, and chest pain type. The prediction models were compared using the area under the receiver-operating-characteristic curve (AUC). Multivariable logistic regression with a dataset-specific random intercept to account for clustering was applied to the clinical prediction models.
**Results or Findings:** Discriminative ability of the clinical prediction model decreased with less typical chest pain (typical or atypical angina (AUC 0.70, 0.68 to 0.71) vs. nonanginal or other chest discomfort (0.63, 0.60 to 0.65); $p < 0.001$). CTA alone had a discriminative ability for CAD of 0.81 (0.80 to 0.82, $p < 0.001$). Adding CTA to the clinical COME-CCT prediction model resulted in a significantly improved diagnostic discrimination with an AUC of 0.86 (0.85 to 0.87, $p < 0.001$). This improvement was equally seen in patients with typical or atypical angina (0.85, 0.84 to 0.86, $p < 0.001$) and nonanginal or other chest discomfort (0.88, 0.86 to 0.89, $p < 0.001$).
**Conclusion:** Incorporating CTA into the clinical prediction results in a good discriminative ability for all types of chest pain.
**Limitations:** This analysis did not include a physiologic assessment of the individual relevance of obstructive CAD and the cohort represents those clinically referred to ICA.
**Ethics Committee Approval:** Individual studies included in the collaboration were approved by the local ethics committees.
**Funding for this study:** German Federal Ministry of Education and Research (01KG1110).
**Author Disclosures:**
Viktoria Wieske: Nothing to disclose
O N behalf of Collaborative Meta-Analysis of Cardiac CT (COME-CCT) Consortium: Research/Grant Support at Grant support from the FP7 Program of the European Commission for the randomized multicenter DISCHARGE trial (603266-2, HEALTH-2012.2.4.-2).

### CTiR 1-10
**Discussant**
Marton Kolossvary; Budapest/HU
*(martonandko@gmail.com)*
**Author Disclosures:**
Marton Kolossvary: Nothing to disclose

**16:45-17:45** **CHANNEL 2**

### Special Focus Session

### SF 7

### My three top tips: pearls and pitfalls in abdominal imaging

**Moderator**
Nikolaos Kartalis; Stockholm/SE

### SF 7-2
**Liver**
Yves Menu; Paris/FR

**Learning Objectives:**
1. To identify key imaging findings in the evaluation of the liver.
2. To understand the importance of appropriately performed examination protocols.
3. To become familiar with the most common pitfalls and how to avoid them.

### SF 7-3
**Bile ducts**
Miriam Klauß; Heidelberg/DE

**Learning Objectives:**
1. To identify key imaging findings in the evaluation of the bile ducts.
2. To understand the importance of appropriately performed examination protocols.
3. To become familiar with the most common pitfalls and how to avoid them.

### SF 7-4
**Pancreas**
Celso Matos; Lisbon/PT (celso.matos@fundacaochampalimaud.pt)

**Learning Objectives:**
1. To identify key imaging findings in the evaluation of the pancreas.
2. To understand the importance of appropriately performed examination protocols.
3.To become familiar with the most common pitfalls and how to avoid them.

### SF 7-5
**Colon/rectum**
Andrea Laghi; Rome/IT (andrea.laghi@uniroma1.it)

**Learning Objectives:**
1. To identify key imaging findings in the evaluation of the colon and rectum.
2. To understand the importance of appropriately performed examination protocols.
3. To become familiar with the most common pitfalls and how to avoid them.
**Author Disclosures:**
Andrea Laghi: Speaker: Bracco, Bayer, GE Healthcare, Merck

### SF 7-6
**Spleen**
Ali Devrim Karaosmanoglu; Ankara/TR
*(alidevrim76@yahoo.com)*

**Learning Objectives:**
1. To identify key imaging findings in the evaluation of the spleen.
2. To understand the importance of appropriately performed examination protocols.
3. To become familiar with the most common pitfalls and how to avoid them.

### SF 7-7
**Kidney**
Kevin Murphy; Cork/IE

**Learning Objectives:**
1. To identify key imaging findings in the evaluation of the kidney.
2. To understand the importance of appropriately performed examination protocols.
3. To become familiar with the most common pitfalls and how to avoid them.

### SF 7-8
**Adrenal**
Giles Boland; Boston, MA/US
*(gboland@partners.org)*

**Learning Objectives:**
1. To identify key imaging findings in the evaluation of the adrenal.
2. To understand the importance of appropriately performed examination protocols.
3. To become familiar with the most common pitfalls and how to avoid them.

### SF 7-9
**Prostate**
Harriet C. Thoeny; Fribourg/CH
*(Harriet.thoeny@h-fr.ch)*

**Learning Objectives:**
1. To identify key imaging findings in the evaluation of the prostate.
2. To understand the importance of appropriately performed examination protocols.
3. To become familiar with the most common pitfalls and how to avoid them.
**Author Disclosures:**
Harriet C. Thoeny: Advisory Board: Bayer

**Live Q&A: How can we really avoid mistakes from imaging pitfalls?**

**16:45-17:45** **CHANNEL 3**

### Refresher Course: Radiographers

### RC 114

### Communicating imaging justification

**Moderator**
Paul Bezzina; Msida/MT

### RC 114-3

**A. The role of radiographers as practitioners**

Shane J. Foley; Dublin/IE
*(shane.foley@ucd.ie)*

**Learning Objectives:**
1. To become familiar with the EU Basic Safety Standards (BSS) directive on the role of the practitioner.
2. To understand the role of radiographers as practitioners.
3. To explore the future role of radiographers as practitioners.

### RC 114-4

**B. Teamwork in the justification process**

Kristin Bakke Lysdahl; Kongsberg/NO
*(Kristin.Bakke.Lysdahl@usn.no)*

**Learning Objectives:**
1. To highlight the importance of radiographers working within a multidisciplinary team.
2. To provide practical advice for radiographers in performing justification.
3. To present the different roles of radiographers as practitioner in different countries.

### RC 114-5

**C. The challenges of referral guideline implementation**

Amanda Martin; Manchester/UK

**Learning Objectives:**
1. To introduce the iRefer and ESR iGuide referral guidelines.
2. To present the challenges in implementing referral guidelines for radiography.
3. To discuss the advantages and limitations of referral guideline implementation.

**Live Q&A: How best can we progress the issue of justification in the current clinical environment?**

**16:45-17:45** **CHANNEL 5**

### Research Presentation Session: Cardiac

### RPS 203

### Advanced new techniques in cardiovascular imaging

**Moderator**
Thomas Weikert; Basle/CH

### RPS 203-2

**Semi-supervised learning for vessel segmentation in coronary angiography**

*J-W. Hong*[1], C-K. Lee[2], Z-G. Qui[1], Z-Z. Li[1], P-H. Tseng[1]; [1]Taipei/TW, [2]Hsinchu/TW
*(page19920610@gmail.com)*

**Purpose:** Invasive coronary angiography (CAG) remains the gold standard for diagnosing coronary artery disease (CAD). To determine treatment plans automatically within the foreseeable future, the first step is vessel segmentation. All current studies focus on supervised learning, where labeling takes professionals lots of time. Semi-supervised learning (SSL) takes advantage of adopting unsupervised learning from a huge amount of unlabeled data. In this study, we are the first to design SSL for vessel segmentation to surpass supervised learning performance.
**Methods or Background:** Our SSL method is composed of the student and teacher models. Random data argumentation from contrast, brightness, sharpness, or additive Gaussian noise is utilized. In the same iteration, two different parameters of the same argumentation input to the student and teacher models. We take the teacher prediction as a fake label to guide the student prediction and compute their consistency loss. If this image is labeled, the segmentation loss between the label and the student prediction is calculated. After updating the student model's weights, we update the teacher model's weights by the exponential moving average method. Our data is collected and labeled at National Taiwan University Hospital.
**Results or Findings:** We utilize the DenseNet121 U-Net for our baseline using supervised learning, including applying additional four times of data augmentation. On 50, 100, and 200 training labeled images for training, the F1-score of supervised learning is 83.19%± 0.43%, 85.04% ± 0.25%, and 86.49% ±0.20% under three-fold validation, respectively, while our proposed method improves to 84.63%±0.84%, 86.97% ±0.35%, and 88.06%±0.14%.
**Conlusion:** With accurate vessel segmentation from SSL, our method with 100 labels outperforms the supervised learning with 200 labels, saving half of required labels.
**Limitations:** Validation using a higher number of labels, requiring further annotation.
**Ethics Committee Approval:** By NCTU-REC-109-014F
**Funding for this study:** By MOST 109-2314-B-002-257-MY3
**Author Disclosures:**
Zhi-Gao Qui: Nothing to disclose
Chih-Kuo Lee: Nothing to disclose
Po-Hsuan Tseng: Nothing to disclose
Zheng-Zhang Li: Nothing to disclose
Jhen-Wei Hong: Nothing to disclose

### RPS 203-4

**Calcium scoring using virtual non-contrast images from a dual-layer spectral CT: comparison to real non-contrast data and evaluation of proportionality factor in a large patient collective**

*F. G. Gassert*, F. T. Gassert, C. E. von Schacky, M. R. Makowski, J. Nadjiri; Munich/DE

**Purpose:** Determination of coronary artery calcium scoring (CACS) in non-contrast computed tomography (CT) images has been shown to be an important prognostic factor in Coronary Artery Disease (CAD). The objective of this study was to evaluate the accuracy of CACS from virtual non-contrast (VNC) imaging generated from spectral data in comparison to standard (true) non-contrast (TNC) imaging in a representative patient cohort with clinically approved software.
**Methods or Background:** 103 patients referred to coronary CTA with suspicion of CAD were investigated on a dual-layer spectral detector CT (SDCT) scanner. CACS was calculated from both TNC and VNC images by software certified for medical use. Patients with a CACS of 0 were excluded from analysis.
**Results or Findings:** Mean age of the study population was 61±11 years with 48 male patients (67%). Inter-quartile-range of clinical CACS was 22-282. Correlation of measured CACS from true- and VNC images was high (0.95); p < 0.001. The slope was 3.83, indicating an underestimation of VNC CACS compared to TNC CACS by that factor. Visual analysis of Bland-Altman plot of CACS showed good accordance of both methods after correction of VNC CACS by the above mentioned factor.
**Conclusion:** In clinical diagnostics of CAD, determination of CACS is feasible using VNC images generated from spectral data obtained on a dual layer spectral detector CT. When multiplied by a correction factor results were in good agreement with the standard technique. This could enable radiation dose reductions by obviating the need for native scans typically used for CACS.
**Limitations:** CT based true non-contrast imaging was used as reference standard. Artifacts caused by beam hardening and blooming could also occur in native scans.
**Ethics Committee Approval:** Approval of the Institutional Review Board had been obtained prior to this study
**Funding for this study:** n/a
**Author Disclosures:**
Florian Tilman Gassert: Nothing to disclose
Claudio E. von Schacky: Nothing to disclose
Marcus R. Makowski: Nothing to disclose
Felix Gerhard Gassert: Nothing to disclose
Jonathan Nadjiri: Nothing to disclose

### RPS 203-5

**A new software for automatic cardiac lesions identification and classification based on CMR-LGE imaging analysis**

*G. Grazzini*, S. Pradella, M. Letteriello, C. De Amicis, P. Tortoli, S. Bettarini, S. Busoni, L. N. N. Mazzoni, V. Miele; Florence/IT
*(grazzini.giulia@gmail.com)*

**Purpose:** The aim of this study is to test a new semiautomated software FLORA (For Late gadOlinium enhanced aReas clAssification) developed to identify and distinguish ischemic from non-ischemic myocardial alterations on cardiac magnetic resonance late gadolinium enhancement (CMR-LGE) images and evaluate its accuracy in assessing the transmural extent of the ischemic lesions.
**Methods or Background:** 120 patients undergone complete CMR were retrospectively selected (40 myocardial ischemic, 40 non-ischemic cardiomyopathies, and 40 negatives). For each patient, a radiologist selected a single slice of the short-axis-LGE sequence and segmented the epicardial and endocardial border. Subsequently, FLORA identified as pathological the pixels with intensity higher than the automatic estimated threshold value (calculated using two different methods: double-gaussian fit method and "fixed-shift" method). The pixel clusters overlapping the endocardial border with an extension>5 mm are classified as ischemic; the pixels without overlap as non-ischemic; while the clusters with overlap<5mm as a mixed pattern. Finally, a quantitative estimate of the transmural extent is provided.
**Results or Findings:** 120 patients, mean age52±34, male91. Considering the software performance in lesions identification, only one false negative has emerged. Ischemic myocardial lesions have been correctly classified in 85/95% (double-gaussian fit method/fixed-shift method). Non-ischemic changes are correctly recognized in 70/82.7%. FLORA showed a very good agreement in the determination of the degree of transmurality with the radiologist (Cohen's Ks 0.855/0.931).
**Conclusion:** The new semiautomated software FLORA showed very high sensitivity in the identification of myocardial LGE. It showed good agreement with the radiologist in the classification of myocardial LGE correctly identifying most ischemic lesions and a very good agreement in the determination of the degree of transmurality.
**Limitations:** FLORA can confuse some motion artifact patterns as LGE
**Ethics Committee Approval:** The ethics committee of our institution approved this study
**Funding for this study:** No funding
**Author Disclosures:**
Mayla Letteriello: Nothing to disclose
Giulia Grazzini: Nothing to disclose
Silvia Bettarini: Nothing to disclose
Vittorio Miele: Nothing to disclose
Silvia Pradella: Nothing to disclose
Lorenzo Nicola N Nicola Mazzoni: Nothing to disclose
Simone Busoni: Nothing to disclose
Paolo Tortoli: Nothing to disclose
Cristian De Amicis: Nothing to disclose

### RPS 203-6

**Precision of left ventricular cardiac output quantification among cine, 2D flow and 4D flow techniques**

*T. Emrich*[1], A. Kilburg[2], A. Ese[2], M. C. Halfmann[2], U. J. Schoepf[1], K-F. Kreitner[2], A. Varga-Szemes[1]; [1]Charleston, SC/US, [2]Mainz/DE
*(tilman.emrich@gmail.com)*

**Purpose:** Cardiac output (CO) plays an important role in assessing health and disease. CMR cine imaging is considered as reference standard for the determination of CO. 2D flow has been shown to have a good correlation with cine-based CO in a swine model, however, both methods tend to overestimate invasive CO-measurement. The purpose of this study was to compare these traditional CO-measurement methods to 4D flow-based assessment in healthy volunteers.
**Methods or Background:** In this prospective study, 49 healthy volunteers (28.0±7.1years,24 men) underwent CMR at 3T. Cine imaging was performed by a conventional GRAPPA accelerated bSSFP sequence, while 2D flow was assed using a conventional phase-contrast sequence with a VENC of 150 cm/s. A GRAPPA accelerated 4D flow-prototype was used to acquire 4D flow datasets covering the aorta. Cine-based CO was calculated by multiplying left ventricular stroke volume by the heart rate. Flow-based CO was expressed by multiplying aortic forward flow by heart rate.
**Results or Findings:** Mean cine-based CO (5.8±1.1l/min) was comparable to CO measured by 2D flow (5.7±1.0l/min, p=0.201). CO-measurements by 4D flow (5.4±0.9l/min) significantly underestimated CO in comparison to cine-based CO (p=0.003; mean of differences -0.38l/min), and 2d flow-based CO (p<0.001, mean of differences -0.25 /min). However, 4D flow-based CO showed good correlation and good to excellent agreement to cine (r=0.781,ICC=0.828) and 2D-flow based CO measurements (r=0.880,ICC=0.914).
**Conclusion:** 4D flow-based evaluation demonstrated systematic underestimation of CO in comparison to standard clinical techniques, but showed strong correlation and agreement. Recently published animal data suggest that standard clinical techniques overestimate CO when compared to invasive measurements, therefore further validation of 4D flow-based estimation of CO against invasive measurements are needed.
**Limitations:** Application in healthy volunteers, no invasive correlation in this healthy volunteers study.
**Ethics Committee Approval:** Approval number: 837.477.14
**Funding for this study:** Not applicable
**Author Disclosures:**
Uwe Joseph Schoepf: Consultant at Bayer, Consultant at HeartFlow, Consultant at Guerbet, Consultant at Bracco, Consultant at Siemens Healthineers, Consultant at Elucid Bioimaging
Tilman Stephan Emrich: Speaker at Siemens Healthineers
Moritz Christian Halfmann: Nothing to disclose
Anton Kilburg: Nothing to disclose
Amir Ese: Nothing to disclose
Karl-Friedrich Kreitner: Nothing to disclose
Akos Varga-Szemes: Research/Grant Support at Siemens Healthineers, Consultant at Elucid Bioimaging, Consultant at Bayer

### RPS 203-8

**Automated assessment of cardiac pathologies on cardiac MRI using T1-mapping and late gadolinium enhancement sequences with deep learning**

*C. E. von Schacky*, F. G. Gassert, S. Foreman, F. T. Gassert, J. S. Kirschke, K-L. Laugwitz, M. R. Makowski, J. Nadjiri; Munich/DE

**Purpose:** A deep learning (DL) model that automatically detects cardiac pathologies on cardiac MRI may help streamline diagnostic workflow. Therefore, the aim of this study was to develop a DL model to detect cardiac pathologies on cardiac MRI using T1-mapping and late gadolinium phase sensitive inversion recovery (LGE) sequences.
**Methods or Background:** Subjects in this retrospective study (n=200, age 53.6 ± 19.9 years, 68 women) were either diagnosed with a cardiac pathology (n=137) including acute and chronic myocardial infarction, myocarditis, dilated cardiomyopathy, and hypertrophic cardiomyopathy, or classified as normal (n=63). Cardiac MR imaging included T1-mapping and LGE sequences. Subjects were split 65/15/20% for training, validation, and testing. The DL models were based on a pretrained DenseNet-161 and implemented in PyTorch. Performance was evaluated on the test set with sensitivity, specificity, and accuracy. Gradient-weighted class activation maps (Grad-CAMs) visualized the decision-making process of the model.
**Results or Findings:** On LGE images, the DL model achieved a sensitivity, specificity, and accuracy of 100%, 38%, and 88%, respectively. On T1-mapping images, the DL model achieved a sensitivity, specificity, and accuracy of 78%, 54%, and 70%, respectively. Grad-CAMs demonstrated that the DL model focused on the myocardium and the cardiac pathology when evaluating an MR image.
**Conclusion:** The developed DL models were able to reliably detect cardiac pathologies on cardiac MR images. The model that used T1-mapping images achieved an accuracy of 70%, while the model that used LGE images achieved an accuracy of 88%. The diagnostic performance of T1 mapping alone is particularly of note since it does not require a contrast agent and can be acquired quickly.
**Limitations:** No further characterization of the cardiac pathologies.
**Ethics Committee Approval:** The local ethics committee approved this retrospective study.
**Funding for this study:** Supported by the Clinician Scientist Program at TUM.
**Author Disclosures:**
Florian Tilman Gassert: Nothing to disclose
Claudio E. von Schacky: Nothing to disclose
Jan S Kirschke: Nothing to disclose
Karl-Ludwig Laugwitz: Nothing to disclose
Marcus R. Makowski: Nothing to disclose
Felix Gerhard Gassert: Nothing to disclose
Jonathan Nadjiri: Nothing to disclose
Sarah Foreman: Nothing to disclose

**Live Q&A**

**18:00-19:00** **CHANNEL 2**

### E³ - Advanced Courses: Neuro

### E³ 618

### Demyelination potpourri or "antibody-mediated autoimmunity"?

**Moderator**
Tarek A. Yousry; London/UK

### E³ 618-2

**A. Circumventricular organs and neuromyelitis optica spectrum disorders (NMOSD)**

Majda M. Thurnher; Vienna/AT
*(majda.thurnher@meduniwien.ac.at)*

**Learning Objectives:**
1. To learn about circumventricular organs.
2. To understand the imaging findings in NMOSD.
3. To learn how to differentiate between demyelinating diseases.

**E³ 618-3**
**B. Multiple sclerosis (MS): views from the rooftop**
Frederik Barkhof; Amsterdam/NL

**Learning Objectives:**
1. To learn about the currently valid MS diagnostic criteria and how to use them in clinical routine.
2. To discuss the "perfect" MS protocol, including contrast use.
3. To learn how and when to use artificial intelligence programs for MS.

**E³ 618-4**
**C. Acute disseminated encephalomyelitis (ADEM) and similar parainfectious syndromes**
Andrea Rossi; Genoa/IT
*(andrearossi@gaslini.org)*

**Learning Objectives:**
1. To discuss the clinical background in ADEM and ADEM-related diseases.
2. To learn the different imaging patterns in ADEM.
3. To adopt the checklist for reporting in suspected ADEM.
**Author Disclosures:**
Andrea Rossi: Speaker: Telemedicine Clinic Academy

**Live Q&A**

**18:00-19:00 CHANNEL 3**

### Refresher Course: Emergency Imaging

### RC 417

### Penetrating injuries: understanding mechanisms and effects

**Moderator**
Mari T. Nummela; Helsinki/FI

**RC 417-2**
**A. Penetrating injury: blades, bullets, and beyond**
Nupur Verma; Gainesville, FL/US

**Learning Objectives:**
1. To become familiar with knife/sharp object wounds and how to detect their effect on tissues.
2. To understand the mechanism of injury at different velocities.
3. To recognise the predictable and unpredictable trajectories of bullets and flying fragments within the body.

**RC 417-3**
**B. Penetrating injuries of the upper body: head, neck, chest, and upper limbs**
Ken Linnau; Seattle, WA/US
*(klinnau@u.washington.edu)*

**Learning Objectives:**
1. To develop a systematic approach for reviewing the relevant structures in a patient with a surface wound.
2. To recognise occult signs of penetrating injury.
3. To learn how to help clinicians evaluate and re-evaluate a patient with a penetrating injury.

**RC 417-4**
**C. Penetrating injuries of the lower body: diaphragm, abdomen, pelvis, and lower limbs**
Elizabeth Dick; London/UK

**Learning Objectives:**
1. To seek out constellations of obvious and occult injuries in pelvic penetrating trauma, including neurovascular and perineal injuries.
2. To become familiar with the spectrum of vascular injuries including active bleeding, pseudoaneurysms, dissection and thrombus formation, and arteriovenous fistulas.
3. To recognise where radiologists can add value and guide the surgical approach.
**Author Disclosures:**
Elizabeth Dick: Speaker: Guerbet

**Live Q&A**

**18:00-19:00 CHANNEL 4**

### Research Presentation Session: Interventional Radiology

### RPS 109

### Improving interventional radiology (IR) procedure results through advanced technology

**Moderator**
Frederic Deschamps; Villejuif/FR

**RPS 109-2**
**MRI-guided percutaneous cryoablation of small renal masses: automated 3D margin assessment using intraoperative MR-MR image fusion and correlation with local outcome**
N. S. de Jager[1], T. J. van Oostenbrugge[1], T. Paetz[2], S. F. M. Jenniskens[1], J. J. Futterer[1], H. F. Langenhuijsen[1], *C. G. Overduin*[1]; [1]Nijmegen/NL, [2]Bremen/DE
*(kristian.overduin@radboudumc.nl)*

**Purpose:** To evaluate three-dimensional treatment margins of magnetic resonance imaging (MRI)-guided percutaneous cryoablation of small renal masses (SRMs) and determine correlation with local outcome.
**Methods or Background:** Retrospective analysis was performed on 31 patients who underwent percutaneous MRI-guided cryoablation for 33 SRMs (size: 0.9-4.5 cm). Tumors and corresponding ice-ball volumes were segmented on intraprocedural pre- and post-ablation MR images using Software Assistant for Interventional Radiology (SAFIR) software. After MRI-MRI co-registration, 3D ablation margins were automatically quantified. Minimal ablation margin was defined as the smallest three-dimensional distance between the tumor and ice-ball surface, where negative values indicate incomplete coverage. Local tumor progression (LTP) after cryoablation was assessed on follow-up imaging.
**Results or Findings:** Median follow-up was 16 months (range: 1-58). Local control after cryoablation was achieved in 27 tumors (82%) and LTP occurred in 6 (18%) respectively. Minimal ablation margin was significantly smaller for cases with vs. without LTP (-6.9±3.5 vs. 2.6±1.7 mm, P<.001). No LTP was observed in patients with a minimal margin >0 mm. Cases with LTP had significantly larger tumor diameters vs. those without LTP (4.1±0.5 vs. 2.9±0.9 cm, P=.003). All negative treatment margins occurred in tumors >3cm. No significant differences were found for other baseline parameters.
**Conclusion:** Minimal treatment margin appears a strong predictor of outcome after MRI-guided cryoablation of SRMs. Radiologically complete coverage (smallest margin >0 mm) was associated with absence of local recurrence. Intraoperative use of automated 3D margin analysis can be a valuable tool in predicting therapy success during percutaneous renal cryoablation procedures.
**Limitations:** Single-center retrospective study with limited sample size.
**Ethics Committee Approval:** This retrospective study was approved by the local IRB. Informed consent was waived.
**Funding for this study:** None
**Author Disclosures:**
Tim J. van Oostenbrugge: Nothing to disclose
Torben Paetz: Nothing to disclose
Hans F. Langenhuijsen: Nothing to disclose
Christiaan G Overduin: Nothing to disclose
Sjoerd Franciscus Maria Jenniskens: Nothing to disclose
Jurgen J. Futterer: Nothing to disclose
Drs Nienke S. de Jager: Nothing to disclose

**RPS 109-3**
**MR-HIFU of uterine fibroids: intraprocedural visualization of the non-perfused volume with deep learning-based synthetic contrast-enhanced T1-weighted scans**
*J. D. J. Slotman*[1], L. W. Bartels[2], A. Zijlstra[1], J. van Osch[1], I. M. Nijholt[1], M. Van 'T Veer-Ten Kate[1], E. De Boer[1], R. d. van den Hoed[1], M. F. Boomsma[1]; [1]Zwolle/NL, [2]Utrecht/NL
*(d.j.slotman@isala.nl)*

**Purpose:** Gadolinium-free visualization of the non-perfused volume (NPV) after MR-guided high intensity focused ultrasound (MR-HIFU) therapy of uterine fibroids, by synthesizing contrast-enhanced T1-weighted (CE-T1w) scans from diffusion weighted imaging (DWI) using deep learning.
**Methods or Background:** BackgroundHitherto, there is still no method available to intraprocedurally assess the NPV during MR-HIFU therapy for uterine fibroids, leading occasionally to unnecessarily long ablation procedures or sub-optimal treatment outcomes. Lack of the possibility to use a gadolinium-based contrast agents between sonications inhibits intraprocedural evaluation of the NPV. Deep learning strategies could possibly allow gadolinium-free DWI-based imaging of the NPV. MethodsA deep learning image-to-image translator was trained on paired DWI/CE-T1w scans to generate synthetic CE-T1w scans that were validated on retrospective data. Four MR-HIFU radiologists independently and randomly assessed the technical success of the MR-HIFU procedure, based on the postprocedural synthetic and reference CE-T1w scan. Additionally, the radiologists were asked to estimate the NPV ratio on both scans.
**Results or Findings:** A total of 56 women were included with a paired DWI and CE-T1w scan acquired during screening, directly postprocedurally and after 6 months follow-up. Absolute agreement between the synthetic and reference CE-T1w scans in assessment of technical success by radiologists was 83%. No statistical differences were found between NPV ratio estimations based on the synthetic and reference CE-T1w scans, and the true 3D measurements ($p = 0.39$).
**Conclusion:** Synthetic CE-T1w scans allow gadolinium-free visualization of the NPV during or after MR-HIFU therapy. Ultimately, the use of these synthetic CE-T1w scans may result in more efficient MR-HIFU treatments by shortening of procedure times and reducing the number of procedures with unsuccessful outcomes.
**Limitations:** Retrospective analysis on prospectively acquired data.
**Ethics Committee Approval:** Local ethics committee approved this study.
**Funding for this study:** None.
**Author Disclosures:**
Martijn F. Boomsma: Nothing to disclose
Erwin De Boer: Nothing to disclose
Jorik Derk Jan Slotman: Nothing to disclose
Rolf d. van den Hoed: Nothing to disclose
Aylene Zijlstra: Nothing to disclose
Lambertus W. Bartels: Nothing to disclose
Miranda Van 'T Veer-Ten Kate: Nothing to disclose
Ingrid M. Nijholt: Nothing to disclose
Jochen van Osch: Nothing to disclose

### RPS 109-4

**Predictive machine learning modelling of complication risk in CT-guided biopsies: a prescription for improving procedure safety**

*E. J. M. Barbosa Jr.*, C. Murphy, H. Lee; Philadelphia, PA/US
*(eduardo.mortani@gmail.com)*

**Purpose:** CT guided transthoracic biopsy (CTTB) is a minimally invasive procedure with demonstrated excellent diagnostic yield and low complication rate. We assessed a large CTTB cohort utilizing machine learning (ML) for prediction of procedural and patient factors associated with risk of complications.
**Methods or Background:** We retrospectively identified 1487 CTTB patients in a tertiary hospital (7-years interval), in this IRB approved investigation. We gathered and coded patient demographics, lesion location (lung, pleura, mediastinum, chest wall), type of biopsy (FNA vs core biopsy, with or without co-axial technique), number of passes, presence and severity of emphysema, distance of aerated lung crossed, patient position on the table, whether the biopsy side was dependent or not, and whether the needle approach was closer to vertical or horizontal. Our outcomes were presence and severity (major, minor, none) of complications. Statistical analyses included univariate analyses with chi-square, multivariate logistic regression (LR) and ML methods such as gradient boosted trees (GBT) and bootstrap random forest (BRF) with cross-validation.
**Results or Findings:** Lung biopsies comprised 1249/1487 CTTBs. Complications occurred in 355/1487 (328/1487 minor, 26/1487 major); and included pneumothorax (261/1487), bleeding (92/1487) and air embolism (2/1487). Statistically significant predictors of complications were greater distance of aerated lung crossed, needle approach vertical, number of passes and biopsy side non-dependent (FDR logworth 2.3 - 5.2), in this order. LR, GBT and RF accuracy ranged from 76% for risk of complications to 93% for prediction of major complications.
**Conclusion:** ML methods can not only accurately predict the likelihood of CTTB complications and their severity, but more importantly can identify that technical factors matter the most, while delineating specific factors, thereby offering a prescription to optimize procedural technique and patient safety.
**Limitations:** Retrospective
**Ethics Committee Approval:** IRB approved
**Funding for this study:** None
**Author Disclosures:**
Eduardo Jose Mortani Barbosa Jr.: Research/Grant Support at Siemens, Research Support, unrelated to this study
Hwan Lee: Nothing to disclose
BA Christina Murphy: Nothing to disclose

### RPS 109-5

**Biopsy and ablation percutaneous procedures on bone lesions: efficacy of an augmented reality navigation system compared to standard CT-guidance**

*G. Castiello*, E. Faiella, G. Pacella, C. Altomare, B. B. Beomonte Zobel, R. F. Grasso; Rome/IT
*(ge.castiello@gmail.com)*

**Purpose:** To assess the efficacy of an augmented reality navigation system (SIRIO) for percutaneous biopsies and ablative treatments of bone lesions.
**Methods or Background:** Biopsy and ablation procedures of bone lesions were retrospectively analyzed, comparing those performed using SIRIO with those performed under common CT-guidance. Overall duration, number of CT scans and patient's radiation dose were analyzed. In biopsy procedures, sensitivity and specificity were also analyzed. For all procedures the size of the lesions was evaluated, considering two classes according to a dimensional cut-off of 2 cm. Mann-Whitney U test was used for statistical analysis.
**Results or Findings:** A total of 188 procedures on bone lesions were evaluated: 142 biopsies and 46 ablations. Of the biopsy procedures, 74 were performed using SIRIO and 68 under standard CT-guidance; of the ablative procedures 22 were performed using SIRIO and 24 under standard CT guidance. For biopsy procedures performed using SIRIO, we observed a significant reduction ($p<0.05$) in number of CT scans and radiation dose for both size classes and a significant reduction in procedural duration for biopsies of lesions <2 cm. Biopsies under SIRIO guidance also showed a better diagnostic efficacy. For ablations performed using SIRIO, we observed a significant reduction ($p<0.05$) in number of CT scans and in radiation dose for procedures on lesions <2 cm. No complications were observed for biopsy procedures; 3 cases of minor complications were observed for ablative procedures under standard CT-guidance (12.5%).
**Conclusion:** SIRIO augmented reality navigation system is an effective tool to guide percutaneous biopsies and ablative treatments of bone lesions compared to traditional CT-guidance methods.
**Limitations:** n/a
**Ethics Committee Approval:** All methods and procedures were carried out following the ethical standards of the institutional research committee and complied with the 1964 Helsinki declaration.
**Funding for this study:** None
**Author Disclosures:**
Gennaro Castiello: Nothing to disclose
Bruno Beomonte Beomonte Zobel: Nothing to disclose
Rosario Francesco Grasso: Nothing to disclose
Eliodoro Faiella: Nothing to disclose
Giuseppina Pacella: Nothing to disclose
Carlo Altomare: Nothing to disclose

### RPS 109-6

**Non-invasive assessment of foot perfusion in a rabbit model of atherosclerosis using dynamic volume perfusion CT with an upslope method**

S. Hur[1], *H. D. Min*[2], J. H. Lee[2], C. J. Yoon[2], W. S. Choi[2]; [1]Seoul/KR, [2]Seongnam-si/KR
*(hooney.min@gmail.com)*

**Purpose:** To evaluate the feasibility of non-invasively measuring foot perfusion using dynamic volume perfusion CT with the upslope method in rabbit models of atherosclerosis.
**Methods or Background:** New Zealand male rabbits were divided into two groups: Dietary hypercholesterolemia induced atherosclerosis (n=10) and normal diet control (n=20). A foot perfusion CT of the rabbit's foot was performed at baseline. After 5 minutes of rest, pressure cuff was applied to the left thigh and inflated to 200 mmHg for 3 minutes. After cuff deflation, a second perfusion CT was scanned to assess perfusion response to hyperemia. A color-coded perfusion map was generated for each perfusion CT scan by using an in-house dedicated analysis software. The perfusion parameters of the left posterior tibial artery, plantar dermis and the ratio of dermal perfusion value before and after cuff-induced reactive hyperemia were analyzed.
**Results or Findings:** There was no significant difference in the blood flow of plantar dermis between the atherosclerosis group and the control group at baseline. Cuff-induced reactive hyperemia in healthy rabbits significantly increased blood flow while atherosclerosis rabbits did not (ratio 0.97 vs. 1.69, $p<.001$).
**Conclusion:** Foot perfusion CT not only provides a perfusion map of the foot but also reveals impairment of reactive hyperemia in atherosclerosis rabbit model. It may be a useful tool for quantitative and functional evaluation of atherosclerotic peripheral arterial disease.
**Limitations:** The study was limited in only measuring one areas of foot perfusion due to the small size of the rabbit feet and a single dominant artery in the rabbit.
**Ethics Committee Approval:** The study was approved by the Institutional Animal Care and Use Committee of Seoul National University Hospital.
**Funding for this study:** The work was supported by Korea Health Technology R&D Project [HI18C0493] funded by the Ministry of Health & Welfare.
**Author Disclosures:**
Jae Hwan Lee: Nothing to disclose
Won Seok Choi: Nothing to disclose
Hooney D Min: Nothing to disclose
Chang Jin Yoon: Nothing to disclose
Saebeom Hur: Nothing to disclose

### RPS 109-7
**Targeting the Achilles' heel of C-arm computed tomography in transarterial chemoembolization: evaluation of a novel motion correction algorithm**
*L. S. Becker*, M. Gutberlet, S. K. Maschke, T. Werncke, C. Dewald, C. von Falck, B. C. Meyer, F. Wacker, J. B. Hinrichs; Hannover/DE

**Purpose:** Evaluation of feasibility and impact on image quality of a motion correction 3D-reconstruction prototype technique for C-arm CT(CACT) during transarterial chemoembolization(TACE).
**Methods or Background:** We included 65 consecutive CACTs acquired during TACE in 54 patients(47m,7 f; 67±11.3 years) from 01/2019-12/2019. All original CACT raw datasets (CACT_Org) underwent reconstruction with and without a prototype 3D-image reconstruction software to compensate for motion (CACT_MC_bone), additional volume punching of high-contrast objects like bones was performed(CACT_MC_no bone). Objective(image sharpness metric) and subjective criteria were used to evaluate image quality. Subjective criteria (3-point scale i and ii; 2-point scale iii-v) were assessed by two independent readers: i)vessel geometry, ii)overall image quality(IQ), iii)delineation of tumor feeders, iv)presence of foreign material induced artifacts, v)need for additional imaging. Friedman rank sum test and post-hoc analysis using pairwise Wilcoxon signed-rank test were computed. Inter-observer agreement was analysed using Cohen´s kappa.
**Results or Findings:** Objective IQ as defined by an objective image sharpness metric, increased from 273.5±28 (CACTOrg) to 328.5±55.1 (CACTMC_bone) and 331±57.8 (CACTMC_no bone; all p<0.0001). These results could largely be confirmed by the subjective analysis, which demonstrated predominantly good and moderate inter-observer agreement, with best agreement for CACTMC_no bone in all categories (e.g. vessel geometry: CACT_Org=0.51, CACT_MC_bone=0.42, CACT_MC_no bone=0.69).
**Conclusion:** Application of a motion correction algorithm was feasible for all data sets and led to a significant increase of both objective and subjective image quality parameters, offering the opportunity to improve diagnostic work-up during TACE procedures.
**Limitations:** Retrospective study at single center, manual bone segmentation
**Ethics Committee Approval:** Waived (retrospective)
**Funding for this study:** None
**Author Disclosures:**
Marcel Gutberlet: Nothing to disclose
Bernhard C Meyer: Nothing to disclose
Christian von Falck: Nothing to disclose
Cornelia Dewald: Nothing to disclose
Sabine Katharina Maschke: Nothing to disclose
Lena Sophie Becker: Nothing to disclose
Thomas Werncke: Nothing to disclose
Frank Wacker: Nothing to disclose
Jan B Hinrichs: Nothing to disclose

**Live Q&A**

**18:00-19:30 CHANNEL 5**

### Healthcare Professionals in Focus

### IF 1

### Resilient individuals: from chronic stress and burnout to happy, healthy, and resilient

**Moderator**
M.G. Myriam Hunink; Rotterdam/NL

### IF 1-2
**From Big Bang to burnout**
Witte Hoogendijk; Rotterdam/NL
*(w.hoogendijk@erasmusmc.nl)*

**Learning Objectives:**
1. To understand how we are not adapted to the stressors of modern life.
2. To appreciate the increase in chronic stress among healthcare providers due to the pandemic.

### IF 1-3
**Staying healthy, happy, and resilient during the pandemic**
Colin P. West; Rochester, MN/US
*(West.Colin@mayo.edu)*

**Learning Objectives:**
1. To appreciate the prevalence of chronic stress and burnout among healthcare professionals.
2. To understand what causes chronic stress and burnout.
3. To appreciate the effect of the pandemic on levels of stress, anxiety, depression, and burnout.
4. To understand what you can do as an individual to become and stay healthy, happy, and resilient.

**Live Q&A: Interventions at the individual level that cultivate resilience and well-being**
with the participation of: Markus F. Berger; Nottwil/CH

**19:45-20:45 CHANNEL 2**

### Special Focus Session

### SF 1

### My three top tips for CT evaluation of lung parenchyma

**Moderator**
Anagha P. Parkar; Bergen/NO

### SF 1-2
**How to differentiate cystic diseases from emphysema**
Peter Beddy; Dublin/IE

**Learning Objectives:**
1. To know what is normal aging.
2. To understand the CT imaging criteria of emphysema.
3. To know what defines a lung cyst.

### SF 1-3
**How to differentiate honeycombing from paraseptal emphysema**
Johny A. Verschakelen; Leuven/BE
*(johny.verschakelen@uz.kuleuven.ac.be)*

**Learning Objectives:**
1. To understand the CT definition of paraseptal emphysema.
2. To learn the definition of honey combing.
3. To know the significance recognising honey combing.

### SF 1-4
**How to differentiate the main cystic diseases from one another**
Anastasia Oikonomou; Toronto, ON/CA
*(anastasia.oikonomou@sunnybrook.ca)*

**Learning Objectives:**
1. To recognise the patterns of the commonest cystic lung diseases.
2. To recognise the rarer diseases.
3. To learn about the prevalence of the various diseases.

### SF 1-5
**How to differentiate bronchial dilatation from bronchiectasies**
Helmut Prosch; Vienna/AT

**Learning Objectives:**
1. To learn the definition of bronchial dilatation.
2. To learn the definition of bronchiectasies
3. To learn what to report or recommend when there is uncertainty.

### SF 1-6
**How to differentiate solid from subsolid nodules**
Felix Doellinger; Berlin/DE
*(felix.doellinger@charite.de)*

**Learning Objectives:**
1. To learn the definition and CT imaging criteria of solid nodules.
2. To learn the definition and CT imaging criteria of subsolid nodules.
3. To learn the difference in follow up of the nodules.

### SF 1-7
**How to differentiate intralobular and interlobular lines**
Thomas Frauenfelder; Zurich/CH

**Learning Objectives:**
1. To learn the definition and CT imaging criteria of intralobular lines.
2. To learn the definition and CT imaging criteria of interlobular lines.
3. To understand why it is important to recognise these patterns.

### SF 1-8
**How to differentiate centrilobular and septal nodules**
Carole Ridge; Dublin/IE
*(caroleridge@hotmail.com)*

**Learning Objectives:**
1. To learn the definition and CT imaging criteria of centrilobular nodules.
2. To learn the definition and CT imaging criteria of septal nodules.
3. To understand why it is important to recognise these patterns.

### SF 1-9
**How to differentiate ground glass from mosaic attenuation**
Marie-Pierre Revel; Paris/FR

**Learning Objectives:**
1. To learn the definition and CT imaging criteria of ground glass opacities.
2. To learn the definition and CT imaging criteria of mosaic attenuation.
3. To understand why it is important to recognise these patterns.

**Live Q&A**

**19:45-20:45 CHANNEL 3**

### Research Presentation Session: Head and Neck

### RPS 108
### Head and neck emergencies and oncology

**Moderator**
Elizabeth Loney; Halifax/UK

### RPS 108-2
**Clinical and prognostic significance of emergency MRI findings in neck infections**
*J. Heikkinen*, J. Nurminen, J. Velhonoja, H. Irjala, T. Happonen, T. Soukka, K. Mattila, J. Hirvonen; Turku/FI
*(jaheik4@gmail.com)*

**Purpose:** Due to superior soft-tissue contrast and ability to delineate abscesses, MRI has superior diagnostic accuracy in neck infections. We sought to determine the clinical and
prognostic significance of various MRI findings, focusing on specific edema patterns.
**Methods or Background:** We retrospectively reviewed imaging of 371 patients with clinically confirmed neck infection usingMRI scans performed with a 3 Tesla MRI device during five years in a tertiary emergency radiologydepartment. We correlated various MRI findings, including retropharyngeal (RPE) and mediastinal edema (ME) and abscess diameter, to clinical findings and outcomes, such as the need for intensive care unit (ICU) treatment and length of hospital stay (LOS).
**Results or Findings:** 202 out of 371 patients (54%) with neck infection showed evidence of RPE, and 81 out of 314 patients (26%) had ME. Both RPE (OR=7.9, $p<0.001$) and ME (OR=5.0, $p<0.001$) were more prevalent in patients who required ICU treatment than in those who did not. In a multivariate analysis, both RPE ($p=0.004$) and ME ($p=0.014$) predicted the need for ICU treatment, as well as maximal abscess diameter ($p<0.001$) in patients with abscess. Presence of ME ($p=0.001$) and maximal abscess diameter ($p<0.001$) were significant predictors of LOS. Even in patients with low CRP (<100 mg/L), RPE and ME predicted ICU irrespective of CRP.
**Conclusion:** In patients with neck infection, RPE, ME, and abscess diameter, as shown by emergency MRI
are significant predictors of more severe disease. These specific edema patterns represent reactive non-suppurative edema. This prognostic value supports the use of emergency MRI in patients with suspected neck infection.
**Limitations:** One limitation is the retrospective nature of the study despite the large sample size.
**Ethics Committee Approval:** Permission from the hospital district board
**Funding for this study:** Sigrid Juselius Foundation
**Author Disclosures:**
Tero Soukka: Nothing to disclose
Tatu Happonen: Nothing to disclose
Jaakko Heikkinen: Nothing to disclose
Jarno Velhonoja: Nothing to disclose
Jussi Hirvonen: Nothing to disclose
Kimmo Mattila: Nothing to disclose
Heikki Irjala: Nothing to disclose
Janne Nurminen: Nothing to disclose

### RPS 108-3
**Pitfalls and normal PET-CT variants in head and neck oncology**
*L. d. A. Defendi*, R. Marques, L. Bezerra, M. M. Ouchar Sabino, L. Bisolo, H. Tames, R. M. Loureiro, R. L. E. Gomes; São Paulo/BR
*(lariad@gmail.com)*

**Purpose:** Elucidate the current indications of Positron emission tomography (PET) with 2-[fluorine-18] fluoro-2-deoxy-D-glucose (FDG) in head and neck cancer; Review the technical aspects and recommendation of FDG-PET CT evaluation; Demonstrate false positive and false negative pitfalls of FDG-PET CT in oncology, as well as the most common artifacts that may be a confusing factor.
**Methods or Background:** Positron emission tomography (PET) with FDG is an effective tool in the diagnosis, staging, and follow-up of head and neck cancer. Nonetheless, the variable physiologic FDG uptake and distribution in the neck, as well as inflammatory conditions and surgical changes can lead to misinterpretation, especially in pos-treatment scenario.
**Results or Findings:** Teaching cases will be used to illustrate the following topics:
1. PET-FDG Protocols and Recommendations
2. False-positive Pitfalls:
- Physiologic FDG uptake (Waldeyer's ring and salivary glands; sternocleidomastoid, extra-ocular and mastication muscles; vocal cords; brown adipose tissue)
- Benign conditions (thyroid nodules; Warthin tumor)
- Inflammatory or infectious FDG uptake (odontogenic sinusitis; sternoclavicular joint arthritis; calcified atherosclerotic plaques; rhinophyma; injected facial fillers)
- Postradiation changes
- Postsurgical changes (unilateral cranial nerve palsy)
3. False-negative Pitfalls:
- Lesion proximity to high metabolic areas;
- Usual tumor presentation with low FDG uptake;
- Necrotic lymph nodes.
4. Artifacts
- Dental hardware
**Conclusion:** Radiologists must be aware of the physiologic variations and pitfalls in PET imaging analysis as well as pathophysiological mechanisms leading to potentially false-positive and false-negative assessments in head and neck cancer.
**Limitations:** Education Exhibit
**Ethics Committee Approval:** Education Exhibit
**Funding for this study:** No funding available
**Author Disclosures:**
Lorena Bezerra: Nothing to disclose
Rafael Maffei Loureiro: Nothing to disclose
Larissa de Andrade Defendi: Nothing to disclose
Hugo Tames: Nothing to disclose
Louise Bisolo: Nothing to disclose
Milena Maria Ouchar Sabino: Nothing to disclose
Rodrigo Marques: Nothing to disclose
Regina Lúcia Elia Gomes: Nothing to disclose

### RPS 108-4

**Quantitative assessment of grade of sinonasal masses using apparent diffusion coefficient values**

*V. S. Arunachalam*, S. Sharma, P. Sharma, P. Sherwani, U. Chauhan, M. Tayal; Rishikesh/IN
*(drvenkat07@gmail.com)*

**Purpose:** The advent of widespread availability of advanced cross-sectional imaging has reignited the quest for objective non-invasive characterization of Sinonasal malignancies. Aim: To compare the exponential(eADC) and true apparent diffusion coefficient(ADC) values of high and low grade sinonasal neoplasms and identify imaging markers for quantitative assessment.
**Methods or Background:** A retrospective observational study was conducted at our institute. A systematic search of case records was done in hospital information system using the keyword “sinonasal neoplasm” which yielded 54 patients over the period March, 2019 till June, 2020. Based on morphological imaging features, they were categorized as high and low grade sinonasal neoplasms. eADC and true ADC values were recorded from solid portions of tumour at three representative locations and average values calculated.
**Results or Findings:** 66%(36) of the patients were males. Tumour heterogeneity on conventional images, susceptibility artifacts as well as heterogeneous contrast enhancement favoured categorization as high grade tumour. Average exponential ADC values of high and low grade sinonasal neoplasms were 0.471 and 0.287 respectively. Average True ADC values of high and low grade gliomas were 0.891 and 1.363*10(-3) respectively. Increased cellularity of high grade lesions have contributed to lower ADC values. Heterogeneity due to necrosis, haemorrhage and calcification may be confounding factors that have contributed to relatively higher eADC values. Histological correlation was done.
**Conclusion:** Comprehensive objective expression of tumour characteristics using perfusion parameters, diffusion variables and quantitative susceptibility mapping values will provide an insight into understanding the molecular basis of carcinogenesis and predict biological behaviour.
**Limitations:** 1) Single centre cross-sectional study.
2) Changes in ADC values after treatment were not recorded.
**Ethics Committee Approval:** Yes
**Funding for this study:** None
**Author Disclosures:**
Mohit Tayal: Nothing to disclose
Udit Chauhan: Nothing to disclose
Venkata Subbaih Arunachalam: Nothing to disclose
Poonam Sherwani: Nothing to disclose
Smily Sharma: Nothing to disclose
Pankaj Sharma: Nothing to disclose

### RPS 108-5

**Frailty is related to radiological sarcopenia and the occurrence of post-operative complications in cutaneous head and neck cancer patients**

*A. T. Zwart*, L. Kok, M. van Kester, R. Dierckx, G. de Bock, A. Van Der Hoorn, G. Halmos; Groningen/NL
*(a.t.zwart@umcg.nl)*

**Purpose:** Radiological sarcopenia, or low quantity of skeletal muscle mass, is an important predictor for adverse clinical outcomes in mucosal head and neck cancer (HNC) patients, and so seems frailty. The aim was to analyse whether frailty and skeletal muscle mass are related, and to analyse their impact on the occurrence of post-operative complications in a cutaneous HNC (cHNC) population.
**Methods or Background:** cHNC patients with primary surgical treatment and CT or MRI neck imaging were included. The association between frailty, as measured with frailty indicators and a geriatric assessment, and skeletal muscle mass was analysed with a logistic and linear regression analyses with respectively radiological sarcopenia and skeletal muscle index (SMI) as dependent variables. To evaluate the impact of skeletal muscle mass on the occurrence of any-postoperative complications, also logistic regression analysis was performed. Analyses were adjusted for relevant baseline assessments.
**Results or Findings:** Fifty-seven patients were included with a mean age of 77.1 (±9.0) years. Prevalence of radiological sarcopenia was 29.8% (n=17). The G8 frailty indicator (OR 7.68, 95% CI 1.19–49.66, p=0.032), and medium to high malnutrition risk (OR 9.55, 95%CI 1.19–76.94), p=0.034) were independently related to radiological sarcopenia. None of the frailty indicators or geriatric domains were related to SMI. The G8 frailty indicator (OR 6.56, 95%CI 1.37–31.46, p=0.019) was an independent predictor for any-postoperative complications.
**Conclusion:** The G8 frailty indicator is related to radiological sarcopenia and the occurrence of post-operative complications in cHNC patients.
**Limitations:** Relatively small sample size.
**Ethics Committee Approval:** Patients were included after written informed consent in the IRB approved OncoLifeS databiobank. Anonymized data were provided in a protected environment.
**Funding for this study:** The first author was awarded with a 3-years-PhD scholarship for excellent master students from the Graduate School of Medical Sciences of the University of Groningen.
**Author Disclosures:**
Geertruida de Bock: Nothing to disclose
Laurence Kok: Nothing to disclose
Aniek Thea Zwart: Nothing to disclose
Gyorgy Halmos: Nothing to disclose
Rudi Dierckx: Nothing to disclose
Anouk Van Der Hoorn: Nothing to disclose
Marloes van Kester: Nothing to disclose

### RPS 108-7

**Laryngeal fractures patterns and associated soft tissue abnormality**

*K. Buch*, K. Takumi, H. Curtin, O. Sakai; Boston, MA/US
*(kbuch@partners.org)*

**Purpose:** Acute traumatic injuries to the larynx including fractures of the hyoid bone, cricoid and thyroid cartilage are uncommon injuries. The purpose of this study was to assess fracture and soft tissue patterns associated with laryngeal trauma.
**Methods or Background:** This was a retrospective review of patients with laryngeal fractures who presented to two level I trauma centers and underwent CT imaging. Imaging findings including fractures of the cartilaginous structures of the larynx and hyoid bone, and soft tissue abnormalities including focal hematoma, edema with non-focal hemorrhage, and additional penetrating injuries were recorded. Frequencies of fracture patterns were recorded.
**Results or Findings:** Thyroid cartilage fractures were most frequently observed occurring in 45/55 patients, cricoid fractures in 13/55 patients and hyoid fractures in 8/55 patients. Multi-site fractures were observed in 12/55 patients with thyroid-cricoid fractures occurring in 8/12 patients followed by thyroid-hyoid fractures in 2/12 patients. Most multi-site fractures occurred with focal supraglottic hematomas (10/12), supraglottic edema and non-focal hemorrhage (11/12), and focal subglottic hematoma (5/12). All 13 cricoid fractures occurred with either focal supraglottic hematoma (7), focal subglottic hematoma (4), or edema with non-focal hemorrhage (13).
**Conclusion:** Thyroid cartilage fractures were the most frequently encountered fracture followed by cricoid cartilage fractures. Cricoid fractures always occurred with soft tissue abnormalities. Recognition of fracture patterns in the setting of laryngeal trauma and associated patterns of soft tissue injury is important for practicing radiologists for early diagnosis of these conditions and reduction of associated morbidity.
**Limitations:** This study has several limitations including relatively small sample size, retrospective study design, and lack of clinical followup for several patients.
**Ethics Committee Approval:** This study was approved by our institution's review board
**Funding for this study:** None
**Author Disclosures:**
Koji Takumi: Nothing to disclose
Karen Buch: Nothing to disclose
Hugh Curtin: Nothing to disclose
Osamu Sakai: Nothing to disclose

### RPS 108-9

**Comprehensive CT data analysis in midface trauma**

*O. Pavlova*, N. S. Serova, D. Davydov, S. K. Ternovoy; Moscow/RU

**Purpose:** To assess the main criteria for comprehensive CT data analysis in midface trauma.
**Methods or Background:** A total of 115 patients with midface trauma (100%) were admitted to the hospital in 24-48 hours after the injury. CT was performed using Canon Aquilion One 640, CT data processing was performed using workstation Vitrea Core. The examined criteria of CT data analysis included orbital and sinuses volume measurements, evaluation of inferior orbital wall defects, analysis of globe position and assessment of intraorbital and face soft tissue density.
**Results or Findings:** Developed criteria for CT data analysis allowed to reveal additional traumatic increased orbital volume in 22 patients (19%); additional enophthalmos in 9 patients (8%); determination of 4 types of inferior orbital wall defects: small inferior orbital defects (n=19; 17%), moderate defects (n=33; 29%), severe defects (n=40; 35%) and total defects (n=22; 19%); assessment of intraorbital and face soft tissue density: oedema (n=64; 56%), haematoma (n=10; 9%), atrophy (n=31; 27%), no changes (n=9; 8%), p<.001.
**Conclusion:** The developed criteria for CT data analysis such as orbital and sinus volume measurements, inferior orbital wall defects evaluation, analysis of globe position and assessment of intraorbital and face soft tissue density statistically significantly increased the efficiency of diagnostics in midface trauma in order to define the correct treatment tactics.
**Limitations:** No
**Ethics Committee Approval:** Yes

**Funding for this study:** No
**Author Disclosures:**
Sergey K Ternovoy: Nothing to disclose
Dmitry Davydov: Nothing to disclose
Natalya S Serova: Nothing to disclose
Olga Pavlova: Nothing to disclose

**Live Q&A**

**19:45-20:45** **CHANNEL 4**

### Transatlantic Course of ESR and RSNA (Radiological Society of North America): Stroke Imaging and Endovascular Treatment: Now and the Future

### TC 228

### Practical stroke imaging and mimics

**Moderators**
Jean-Pierre Pruvo; Lille/FR
Achala Vagal; Cincinnati, OH/US

### TC 228-3

**A. Stroke mimics and "chameleons": how to recognise them**
Didier Leys; Lille/FR

**Learning Objectives:**
1. To learn about the definitions of stroke mimics and chameleons.
2. To understand the clinical challenges of stroke mimics.
3. To become familiar with the imaging signs and differential diagnosis of stroke mimics

### TC 228-4

**B. Practical review of stroke imaging and triage: within six hours and beyond including wake-up strokes**
Lotfi Hacein-Bey; Sacramento, CA/US
*(lhaceinbey@yahoo.com)*

**Learning Objectives:**
1. To learn about optimal patient triage in acute ischaemic stroke in early and delayed time windows.
2. To understand stroke imaging in wake-up strokes and unknown onset time.
3. To appreciate the importance of efficient workup and time metrics.

### TC 228-5

**C. Interactive case presentation**
*Lotfi Hacein-Bey*; Sacramento, CA/US, Didier Leys; Lille/FR
*(lhaceinbey@yahoo.com)*

**Learning Objectives:**
1. To learn how imaging can help in decision making in acute stroke.
2. To consolidate the knowledge gained from the session with interactive cases.

**Live Q&A**

**19:45-20:45** **CHANNEL 5**

### Research Presentation Session: Paediatric

### RPS 112

### Evaluating new imaging techniques for children

**Moderator**
Ola Kvist; Stockholm/SE

### RPS 112-2

**Fully-automated segmentation of neuroblastic tumours on multi-sequence MRI using convolutional neural networks**
*L. Cerda Alberich*, L. Marti-Bonmati; Valencia/ES
*(leogibi230@gmail.com)*

**Purpose:** In this study, we proposed an automated method based on convolutional neural networks (CNNs) for segmentation of neuroblastic tumours on multi-sequence magnetic resonance imaging (MRI).
**Methods or Background:** T1-weighted (T1W), T2-weighted (T2W) and Diffusion-weighted (DW) MRI images were collected from 127 neuroblastoma patients from three different European hospitals. We developed a multi-parametric and multi-planar U-net and trained the network based on the two-dimensional multi-sequence MRI images in the training dataset. In order to justify the effectiveness of multi-sequence MRI images, we performed an experiment with different inputs in a subset of randomly selected patients. We evaluated the model's performance by using a 5-fold cross-validation strategy and compared the results with other state-of-the-art solutions.
**Results or Findings:** The Dice similarity coefficient (DSC) values of the method using only T1W, only T2W, only DW and a multi-sequence approach of 5-fold cross-validation as different inputs were 0.732±0.064, 0.745±0.118, 0.786±0.077 and 0.841±0.038, respectively. The average DSC of the internal validation using the multi-sequence strategy was 0.830.
**Conclusion:** To summarize, we successfully proposed a fully automatic segmentation method of neuroblastic tumours based on CNNs and multi-sequence MRI images with accurate and stable performance. If further validated, our proposed method would be of use in clinical practice for management of neuroblastoma cancer.
**Limitations:** Future work with a lager sample would be necessary to further validate our model. In addition, the co-registration of T1W, T2W and DW images is still challenging. A method to overcome this limitation may be proposed in future work.
**Ethics Committee Approval:** The study was approved by all the corresponding Hospital's Ethics Committees.
**Funding for this study:** PRIMAGE (PRedictive In-silico Multiscale Analytics to support cancer personalized diaGnosis and prognosis, empowered by imaging biomarkers). Horizon 2020 | RIA (Topic SC1-DTH-07-2018) project with grant agreement no: 826494.
**Author Disclosures:**
Luis Marti-Bonmati: Nothing to disclose
Leonor Cerda Alberich: Nothing to disclose

### RPS 112-6

**CBF in term and preterm neonates: a pCASL study**
*E. Piccirilli*, V. Panara, M. Treddenti, V. Maruotti, M. Colasurdo, M. Caulo; Chieti/IT
*(ele.piccirilli@gmail.com)*

**Purpose:** The aims of our study were to evaluate CBF using pCASL in different brain regions in newborn babies of different gestational ages (GA) and how GA affects CBF.
**Methods or Background:** Brain MRI of 106 consecutive preterm and term neonates were included. The neonates were divided into 4 categories according to their GA. A standardized elliptic ROI was manually placed on the cerebral perfusion maps in the cortex of the frontal (F), parietal (P) and occipital (O) lobe, in the thalamus (T) and in the cerebellum (C) of each hemisphere, using the T2 sequence as anatomical reference. A two-sample t-test was applied to evaluate differences in CBF of each homologous ROI between hemispheres. Differences in CBF among ROIs (F, P, O, T and C) were assessed with One-way ANOVA. Pearson's correlation coefficient was calculated to evaluate the relationship between GA and mean CBF. Statistical analysis was performed with IBM SPSS Statistics ($p<0.05$).
**Results or Findings:** No significant hemispheric differences in CBF were observed ($p=0.3$). There were significant differences in CBF between F and P ($p=.009$), F and T ($p<0.05$), P and T ($p<0.05$), P and C ($p=0.046$) and O and T ($p<0.05$). A significant correlation between GA and CBF was observed, with overall CBF increasing with GA ($r=0.2$, $p=0.01$).
**Conclusion:** We demonstrated differences in CBF values among different cerebral grey matter regions in newborns, independently of their GA. The T was the region with the highest CBF, both in term and preterm neonates. Moreover, we demonstrated in vivo and non-invasively a significant correlation between CBF and GA.
**Limitations:** Manual ROIs, which potentially included white matter as well. Retrospective study.
**Ethics Committee Approval:** Study approved by the Ethics Committee of our University
**Funding for this study:** No funding was received for this work

**Author Disclosures:**
Valerio Maruotti: Nothing to disclose
Mauro Treddenti: Nothing to disclose
Eleonora Piccirilli: Nothing to disclose
Marco Colasurdo: Nothing to disclose
Valentina Panara: Nothing to disclose
Massimo Caulo: Nothing to disclose

### RPS 112-7

**RAVE-T2/T1: feasibility of a new hybrid MR-sequence for free-breathing abdominal MRI in children and adolescents**

*K. Glutig*[1], F. H. Prüfer[2], K. T. Block[3], H-J. Mentzel[1], M. M. Obmann[2]; [1]Jena/DE, [2]Basel/CH, [3]New York, NY/US

**Purpose:** To evaluate the feasibility of a RAVE-T2/T1 hybrid sequence for free-breathing MRI of the abdomen in pediatric patients.
This is a modern three-dimensional radial sequence with fat saturation and blood flow suppression. During one measurement T2- and T1-weighted contrasts are obtained in identical slice position.
**Methods or Background:** 15 MRIs of children (age 4 weeks-18years) on a 3-T System (Skyra, Siemens) were retrospectivly reviewed. All patients underwent standard axial T1 DIXON (4,06/1,24 TR/TE, 9° flip angle, TA 11 sec), T2 HASTE FS (1000/96 TR/TE, 130° flip angle, TA 85 sec) and RAVE-T2/T1 (1200 TR, 133 and 1,44 TE, 90/120° and 12° flip angle, TA 483 sec) of the upper abdomen. MRI studies were analyzed by two pediatric radiologists using a five-point Likert scale in five different categories: overall image quality, respiratory motion artefacts, clarity of portal vein wall delineation, sharpness of hepatic margin and quality of fat suppression. Wilcoxon signed-rank test and sum scores of T1 and T2 sequences were used for evaluation.
**Results or Findings:** The T2 weighted part of the RAVE-T2/T1 sequence was significantly better than the standard T2 HASTE sequence in all image quality categories: overall image quality (2.17±0.7 vs 1.77±0,7), respiratory motion artefacts (3.83±0.4 vs 2.03±0.7), portal vein clarity (3.25±0.8 vs 2.23±0.7), hepatic margin sharpness (2.43±1,0 vs 1.8±0.7). There was no significant difference in image quality for the T1 weighted sequences (for all p>0.05).
**Conclusion:** Abdominal MRI with new radial RAVE-T2/T1 hybrid sequence showed higher image quality compared to a standard T2 HASTE sequence and equivalent image quality compared to a T1 DIXON sequence. The RAVE-T2/T1 hybrid sequence may be a good tool for pediatric abdominal imaging, particularly for children with lung diseases due to the free breathing acquisition.
**Limitations:** Not required.
**Ethics Committee Approval:** None
**Funding for this study:** None
**Author Disclosures:**
Kai Tobias Block: Nothing to disclose
Markus Obmann: Nothing to disclose
Hans-Joachim Mentzel: Nothing to disclose
Katja Glutig: Nothing to disclose
Friederike Helene Prüfer: Nothing to disclose

### RPS 112-8

**Quantitative analysis of the effect of dose reduction on image metrics in 18F-FDG PET/MRI in paediatric oncology**

U. Aydos, E. Balcı, S. Gülbahar Ateş, U. O. Akdemir, *L. O. Atay*; Ankara/TR
*(l.ozlem.atay@gmail.com)*

**Purpose:** To evaluate the effect of reduced injected tracer activities on the quantitative image parameters of whole body 18F-FDG PET/MRI imaging in paediatric patients.
**Methods or Background:** 77 oncological 18F-FDG PET/MRI examinations were performed on 54 paediatric patients (standart injected activity: 1/2-dose [1.9 MBq/kg] and standart PET scan duration: 5 min per bed position). Lower activity PET images (1/3-dose [1.2 MBq/kg] and 1/4-dose [0.9 MBq/kg]) were retrospectively simulated from the originally acquired list-mode data sets by decreasing the count statistics. Objective quantitative parameters were assessed by measuring the SUVmax, SUVmean, SUVvar, SUVpeak, signal-to-noise ratio (SNR) and contrast-to-noise ratios (CNR) in each PET data set. Differences in quantitative parameters of simulated data sets were recorded as relative percentage changes compared to the original data.
**Results or Findings:** SNRs were found as significantly different among PET data sets ($p < 0.001$) and showed gradually increasing image noise with decreasing doses. CNRmax and CNRmin values did not show any significant differences among PET data sets ($p = 0.152$ and $p = 0.259$, respectively). In physiologic organs, compared to original data set, mean relative deviations of SUVmax was below 10% and of SUVmean was below 1% at 1/3-dose data set. In FDG-avid lesions, compared to original data, the mean relative deviations of SUVmean, SUVvar and SUVpeak were below 2% and of SUVmax was below 3% at 1/3-dose images. The relative deviations of SUVmax and SUVmean reached levels above 4% and 3% respectively and of SUVpeak reached levels up to above 9% at 1/4-dose images.
**Conclusion:** Our analysis showed that the reduction of injected activity to 1.2 MBq/kg can be feasible in paediatric oncologic PET/MRI, with a small relative percentage change in quantitative parameters.
**Limitations:** Low activity images were simulated retrospectively.
**Ethics Committee Approval:** Yes
**Funding for this study:** No
**Author Disclosures:**
Umit Ozgur Akdemir: Nothing to disclose
Erdem Balcı: Nothing to disclose
Lutfiye Ozlem Atay: Nothing to disclose
Uguray Aydos: Nothing to disclose
Seda Gülbahar Ateş: Nothing to disclose

### RPS 112-9

**Comparative evaluation of MRI vs CT lung in paediatric patients with chronic lung disease**

*M. A. Fouad*, R. Ibrahim; Cairo/EG
*(mona.fouad@kasralainy.edu.eg)*

**Purpose:** to study the efficacy and role of MRI lungs in diagnosis/surveillance of paediatric patients with chronic lung disease in comparison to HRCT lung.
**Methods or Background:** This cross-section prospective study included 35 pediatric cases, 29 males (83%), and 6 females (17%), referred with a chronic chest complaint, for diagnosis or follow-up. Cases with HRCT evidence of chronic lung disease were included in the study. HRCT and MRI lungs were done to all patients within a time interval of 3 days. CT and MRI images analysis were evaluated separately and blindly by two pediatric radiologists with 15 years' experience. A comparative evaluation of the two studies was done using the modified Bhalla scoring system. Then statistical analysis was done.
**Results or Findings:** All parameters of the modified Bhalla scoring system evaluated and all cases had bronchiectasis and bronchial wall thickening, 88.6% had ground-glass densities. 57% had mucus plugs and mosaic attenuation, 51.4% had centrilobular nodules. The mean scores were 11.2 (range 3-21, SD 5.3) for HRCT and 10.46 (range 4-20, SD 4.4) for MRI, with the mean difference of 0.74 points. There was a strong positive correlation between both methods, with $R(33)= 0.8366$, and a significant P-value < 0.00001. Findings of HRCT and MRI showed 78% complete agreement, A difference of 1 point appeared in 18.3%. Thus, the overall agreement between both modalities was excellent. MRI helped in primary diagnosis for 54% of cases compared to CT in 91% of cases.
**Conclusion:** MRI lungs is a comparable technique to high-resolution CT in pediatric cases, with a high agreement in bronchial disease evaluation, detection of abscesses, and honeycombing, and beneficial for case surveillance rather than primary diagnosis.
**Limitations:** Limited number of cases.
**Ethics Committee Approval:** KasrAlainy ethical committee.
**Funding for this study:** No fund received.
**Author Disclosures:**
Rania Ibrahim: Nothing to disclose
Mona Ahmed Fouad: Nothing to disclose

**Live Q&A**

**21:00-21:30** **CHANNEL 2**

### PL 1
### Plenary Lecture

**Moderator**
Michael H. Fuchsjäger; Graz/AT

### PL 1-2

**ACE2 - a rational front line therapy for COVID-19**

Josef Penninger; Vancouver/CA
*(josef.penninger@ubc.ca)*

**Live Q&A**

### Friday, March 5

08:00-09:00 CHANNEL 2

### E³ - ECR Master Class

### E³ 626

### Clinical impact of image quantification and artificial intelligence (AI)

**Moderator**
Thomas Beyer; Vienna/AT

### E³ 626-2

**A. The role of quantification in brain imaging**
Ralf Buchert; Hamburg/DE

**Learning Objectives:**
1. To learn quantification methods for brain metabolism and dementia.
2. To outline reliable quantification methods for neuroinflammation.
3. To discuss the added value of quantification.

### E³ 626-3

**B. Quantitative imaging and AI in cardiovascular diseases**
Niek Prakken; Groningen/NL

**Learning Objectives:**
1. To outline machine learning techniques and metrics for cardiac imaging.
2. To learn about quantification of cardiac and intravascular flow.
3. To understand the value of quantitative cardiac imaging for myocardial ischaemia.

### E³ 626-4

**C. Quantitative imaging and radiomics in oncology**
Gary Cook; London/UK

**Learning Objectives:**
1. To outline quantitative methods, radiomics, and machine learning in oncological molecular imaging.
2. To learn about potential targets and metrics as promising imaging biomarkers.
3. To discuss the challenges of quantitative imaging and radiomics in oncology.

**Author Disclosures:**
Gary Cook: Consultant: NanoMab, Serac Healthcare, Theragnostics Ltd

**Live Q&A: What can we quantify and what is clinically essential?**

08:00-09:00 CHANNEL 3

### Research Presentation Session: Chest

### RPS 104

### Artificial intelligence (AI) and technological improvements in chest imaging

**Moderator**
Fergus Gleeson; Oxford/UK

### RPS 104-2

**Assessment of pneumothorax detection performance with and without the aid of a deep-learning-based AI algorithm**
J. M. Sabol[1], T. Zhang[2], *A. Baenen*[2], G. Rao[2], Z. Herczeg[2], B. Heckel[2], P. Tegzes[2]; [1]Sussex, WI/US, [2]Waukesha, WI/US
*(alec.baenen@ge.com)*

**Purpose:** To evaluate clinician performance in detection of pneumothorax (PTX) on frontal chest radiographs with and without the aid of a deep-learning-based AI system relative to standalone performance of the algorithm.
**Methods or Background:** Presence of PTX in a 174-case dataset from 3 international sites was determined by two board certified radiologists with discrepancies arbitrated by a third. This challenging, enriched (48% positive) dataset included a range of PTX sizes (64% small/36% large) and co-morbidities. Inferencing for PTX was performed using a commercially available AI system that uses frontal chest x-ray and PTX classification algorithms developed from independent datasets of over 32k and 12.5k images respectively. Seven readers read each case twice: with and without the aid of AI in a Latin square crossover design with a 4-week washout period. Readers consisted of thoracic Radiologist and resident, ER physician and resident, Internal Medicine resident, and two Nurse Practitioners. Multiple-reader multiple-case ROC analysis was used to determine diagnostic accuracy of PTX detection with and without the AI system. A sensitivity/specificity analysis was conducted from separate responses on presence/absence of PTX.
**Results or Findings:** On this dataset, the AI system had standalone AUC, sensitivity, specificity performance of 0.941, 81%, and 96% respectively. Without AI, the readers had equal sensitivity and specificity of 78.6%. With AI these increased to 83.8% and 87.6% and AUC rose from 0.825 to 0.901 (p<0.036). For small PTX, improvement was markedly superior, AUC rising 0.098 (p<0.018).
**Conclusion:** Analysis shows that an AI system significantly improves performance of radiologists and bedside clinicians to detect PTX with most significant improvement for small PTX, enabling rapid intervention and improved outcomes.
**Limitations:** Outcome assessment of performance in clinical settings is required to validate these results.
**Ethics Committee Approval:** IRB oversight waived.
**Funding for this study:** Not applicable.
**Author Disclosures:**
Beth Heckel: Employee at GE Healthcare
Pál Tegzes: Employee at GE Healthcare
Gireesha Rao: Employee at GE Healthcare
Alec Baenen: Employee at GE Healthcare
John M Sabol: Nothing to disclose
Tianhao Zhang: Employee at GE Healthcare
Zita Herczeg: Employee at GE Healthcare

### RPS 104-3

**Clinical evaluation of a deep learning-based, pneumothorax (PTX) classification AI model on frontal chest x-ray images**
*R. Kurokawa*[1], K. Suzuki[1], A. Hagiwara[1], A. Baenen[2], Z. Herczeg[3], P. Tegzes[4], L. Török[4], N. Uetake[1], S. Aoki[1]; [1]Tokyo/JP, [2]Waukesha, WI/US, [3]Szeged/HU, [4]Budapest/HU

**Purpose:** To evaluate the performance of a PTX classification AI model to detect pneumothorax(es) of various sizes and locations.
**Methods or Background:** A retrospective 400 images were formed from patients(>= 18 y/o). The dataset was derived from the clinical population within an international institution not represented in the training data used during model development. Ground truth for the presence, laterality, and size of a PTX was independently assessed by two radiologists. PTX size measurement follows both the British Thoracic Society and American College of Chest Physicians standards. Inferencing was then performed by a deep learning-based algorithm developed from independent datasets of over 12,000 images.
**Results or Findings:** Radiologists deemed PTX present in 42% of the dataset, with laterality of right (48.5%), left (43.8%), and bilateral(7.7%). PTX size was determined to be small(37.3%), large (60.3%), and small + large(2.3%) in bilateral cases. The PTX classifier had an AUC of .978 (overall), .952 (small PTX),.994 (large PTX),.975 (Left PTX),.980 (right PTX) and .991 (bilateral PTX). In addition, the PTX classifier had sensitivity of 94% (overall), 86% (small PTX), 99% (large PTX), and specificity of 91% (all). The AI model accurately predicted the radiologist label using either the BTS and ACCP standards, achieving an AUC of .796 and .797 respectively. A AUC of .950 was achieved when comparing the radiologist binary size label to model mask area output.
**Conclusion:** The investigated AI model accurately localizes and quantifies PTX on frontal chest x-ray images originating from geographical regions not represented in training/testing data used during model development.
**Limitations:** The model only recognizes PTX.
**Ethics Committee Approval:** This is an institutional review board approved study.
**Funding for this study:** This is a collaborative study with GE healthcare
**Author Disclosures:**
Ryo Kurokawa: Nothing to disclose
Shigeki Aoki: Grant Recipient at JSPS Kakenhi
Pál Tegzes: Employee at GE Healthcare
Alec Baenen: Employee at GE Healthcare
Akifumi Hagiwara: Grant Recipient at JSPS Kakenhi
Nozomu Uetake: Employee at GE Healthcare
Zita Herczeg: Employee at GE Healthcare
Kazuhiro Suzuki: Investigator at GE Healthcare
Levente Török: Employee at GE Healthcare

### RPS 104-4

**Diagnostic performance of a new reconstruction technique for dual-energy CT (DECT) lung perfusion: preliminary experience in 58 patients**

J. Pinilo[1], A. Hutt[1], J-B. Faivre[1], J. Labreuche[1], T. Flohr[2], B. Schmidt[2], A. Duhamel[1], J. Remy[1], *M. Remy-Jardin*[1]; [1]Lille/FR, [2]Forchheim/DE
*(martine.remy@chru-lille.fr)*

**Purpose:** To compare DECT lung perfused blood volume images generated with monoenergetic subtraction (MonoE) with images obtained by three-compartment decomposition (Lung PBV).
**Methods or Background:** The inclusion criteria comprised: (a) a positive or negative CT angiogram for acute PE; (b) obtained on the same CT unit (Force, Siemens Healthineers); (c) in the absence of lung infiltration or destruction. Over a two-year period, 28 consecutive patients with acute PE were eligible (Group 1); the first 30 consecutive patients with a negative angiogram for acute PE were selected (Group 2). From each data set, lung PBV images (i.e., the reference standard) and MonoE images, generated by subtraction of 40 keV and 190 keV monoenergetic images using a prototype software, were reconstructed. The inter-technique comparison was undertaken at a patient (n=58) and segment (n=1106; exclusion of 54 non-analyzable segments) level.
**Results or Findings:** The distribution of scores of subjective image noise significantly differed between the two reconstructions ($p<0.0001$), with mild noise in 58.6% (34/58) of MonoE vs 25.9% (15/58) of PBV images. Detection of perfusion defects was concordant in 1104 segments (no defect: n=968; defects present: n=138) and discordant in 2 segments with a defect only depicted on MonoE images. The gradient of attenuation between perfused areas and defects was significantly higher on MonoE images compared to Lung PBV (77.7 ±20.0 HU vs 26.9 ±8.4 HU) ($p<0.0001$) with sharper edges in 8.7% of defects. Fissures were precisely identified in 77.6% of patients (45/58) on MonoE images while blurred (30/58; 51.7%) or not detectable (28/58; 48.3%) on Lung PBV images.
**Conclusion:** MonoE perfusion imaging allows significant improvement in the overall image quality and detectability of PE-type perfusion defects.
**Limitations:** Comparison was obtained by consensus between two readers.
**Ethics Committee Approval:** Waiver of patient informed consent
**Funding for this study:** None
**Author Disclosures:**
Thomas Flohr: Employee at Siemens Healthineers
Jean-Baptiste Faivre: Nothing to disclose
Julien Labreuche: Nothing to disclose
Martine Rémy-Jardin: Research/Grant Support at Siemens Healthineers
Bernhard Schmidt: Employee at Siemens Healthineers
Jacques Remy: Research/Grant Support at Siemens Healthineers
M. Alain Duhamel: Nothing to disclose
Juliette Pinilo: Nothing to disclose
Antoine Hutt: Nothing to disclose

### RPS 104-5

**Chest CT at x-ray dose using a novel noise-mitigating projection technique: diagnostic value for detecting pneumonia in immunocompromised patients**

*P. Rogalla*, S. Carey, M. May, S. Ronghe, S. Kandel, F. Sanchez Tijmes; Toronto, ON/CA
*(Patrik.Rogalla@uhn.ca)*

**Purpose:** To assess the diagnostic performance of ultra-low-dose CT using a noise-mitigating projection technique (Thoracic Tomogram, TT) to detect pneumonia in immunocompromised patients.
**Methods or Background:** With ethics board approval, 35 immunocompromised patients underwent a standard-of-care, low-dose CT (SOC) of the chest (120 kV, dose modulation) immediately followed by an ultra-low-dose CT (135 kV, target dose 0.18 mSv/75 kg), processed using a novel projection function in three planes with 2 cm thick slabs (TT) to suppress noise while enhancing inherent image contrast. Two chest radiologists evaluated the TTs alone, and then the SOC-CT in the categories: (I) opacities <1cm, (II) opacities >1cm, (III) atelectasis, (IV) pleural effusion, (V) interstitial pattern, on a scale of -4=confidently absent to +4=confidently present. Readers provided a diagnosis (fungal, atypical, bacterial, non-specific) and ranked the subjective image quality (1=non-diagnostic, 10=best). The interpretation time for TTs and the DLPs were recorded.
**Results or Findings:** Sensitivity, specificity, accuracy and AUC for reader 1/2 was 72.2%/72.2%, 88.2%/88.2%, 80.0%/80.0% and 0.88/0.85 in category (I). Reader 2 over-interpreted only 1 case in category (II) and (IV), and did not report 4 minimal atelectases (III) and 6 cases of initial interstitial pattern (accuracy, 82.9%). There was no difference in diagnosis between TT and SOC or between readers. The mean image quality and DLP (mGy*cm) was 8.42 and 19.1 for TT, and 9.42 and 103.0 for SOC-CT ($p<0.05$). The interpretation time for reader 1 and 2 was 15.2s and 14.4s, respectively.
**Conclusion:** Ultra-low-dose chest CT at X-ray dose (2 views) provides nearly identical diagnostic information to SOC-CT in immunocompromised patients in whom detection of pneumonia is paramount, at one-fifth of the radiation dose. The interpretation time appears similar to reading chest X-rays.
**Limitations:** Applicable to lung only; sample size
**Ethics Committee Approval:** Approved.
**Funding for this study:** Internal.
**Author Disclosures:**
Felipe Sanchez Tijmes: Nothing to disclose
Sean Carey: Nothing to disclose
Mary May: Nothing to disclose
Sonja Kandel: Nothing to disclose
Patrik Rogalla: Nothing to disclose
Sandeep Ronghe: Nothing to disclose

### RPS 104-6

**Comparison of capability for distinguishing metastatic from non-metastatic lymph nodes among computed DWI (cDWI), actual DWI (aDWI) and PET/CT in non-small cell lung cancer**

*Y. Ohno*[1], M. Yui[2], T. Yoshikawa[3], D. Takenaka[3], Y. Kassai[2], K. Murayama[1], H. Toyama[1]; [1]Toyoake/JP, [2]Ohtawara/JP, [3]Akashi/JP
*(yohno@fujita-hu.ac.jp)*

**Purpose:** To directly compare the capability for distinguishing metastatic from non-metastatic lymph nodes among computed DWI (cDWI) generated at different b values, actual DWI (aDWI) and FDG-PET/CT in non-small cell lung cancer (NSCLC) patients.
**Methods or Background:** 245 consecutive operable NSCLC patients (127 men, 118 women; mean age 75 years) prospectively underwent aDWI with b value at 0 and 1000 s/mm2, FDG-PET/CT, surgical treatment and pathological and follow-up examinations. In each subject, computed DWIs were generated at 400 (cDWI400), 600 (cDWI600) and 800 (cDWI800) s/mm2. According to pathological examination results, 114 metastatic nodes and 114 out of 2581 non-metastatic nodes were measured contrast ratio (CR) on each computed DWI between each lymph node and chest wall muscle, ADC on actual DWI and SUVmax by ROI measurements. To determine the feasible threshold value for each index on a per node basis, ROC analysis was performed. Then, diagnostic performance were compared among all methods by McNemar's test.
**Results or Findings:** Area under the curve (AUC) of CR at cDWI600 (AUC=0.87, $p<0.05$) was significantly larger than that of CRs at cDWI400 (AUC=0.79), cDWI800 (AUC=0.83) and SUVmax (AUC=0.81). In addition, AUC of cDWI800 was significantly larger than that of cDWI400 ($p<0.05$). On a per node basis, accuracy of CR at cDWI600 (79.4%) were significantly higher than that of others ($p<0.05$).
**Conclusion:** Computed DWI has a better potential for distinguishing metastatic from non-metastatic lymph nodes than actual DWI or PET/CT in NSCLC patients. Computed DWI would be better to be generated at 600s/mm2 in this setting.
**Limitations:** Not applicable
**Ethics Committee Approval:** Not applicable
**Funding for this study:** Research grant from Canon Medical Systems Corporation.
**Author Disclosures:**
Kazuhiro Murayama: Grant Recipient at Canon Medical SysteCorporation
Hiroshi Toyama: Grant Recipient at Canon Medical SysteCorporation
Daisuke Takenaka: Nothing to disclose
Masao Yui: Employee at Canon Medical SysteCorporation
Yoshimori Kassai: Employee at Canon Medical SysteCorporation
Takeshi Yoshikawa: Nothing to disclose
Yoshiharu Ohno: Grant Recipient at Canon Medical SysteCorporation

### RPS 104-7

**Mismatch of predicted lung volume and CT-derived lung volume in a general population cohort**

*H. J. Wisselink*, D. J. Steerenberg, G. J. Pelgrim, M. Rook, G. de Bock, R. Vliegenthart; Groningen/NL
*(h.j.wisselink@alumnus.utwente.nl)*

**Purpose:** For lung transplantation, size of donor lungs should match the intended recipient. A size mismatch may increase morbidity or mortality. Currently, size is predicted based on donor's gender and height, but this model may not adequately account for natural variation. We compared the predicted total lung capacity (TLC) with the total lung volume measured on inspiratory CT (TLV) in a general population study cohort.
**Methods or Background:** For the current analysis, 400 participants were retrospectively selected from the ongoing ImaLife study. All underwent inspiratory CT scan (third generation dual-source CT [SOMATOM Force, Siemens Healthineers], 120 kVp, 20 ref mAs, slice thickness/increment 1.0/0.7). TLV was measured with Pulmo3D (Siemens Healthineers). The predicted lung volume was calculated as $TLC\_male=(7.99\cdot height-7.08)\cdot 1000$ and $TLC\_female=(6.60\cdot height-5.79)\cdot 1000$. TLC values were compared to a linear multivariate model, using height, weight, gender, and age to predict TLV.

Friday

**Results or Findings:** Mean±SD TLC was 6.5±1.2L, while TLV was 5.5±1.3L. The difference between predicted and measured volumes was 1.2±1.2L for men and 0.6±0.8L for women.
$R^2$ for the (height-only) TLC models were -0.80 (male) and -0.21 (female), and 0.46 for the multivariate model.
**Conclusion:** There is a substantial mismatch between predicted and measured lung volumes, predicted TLC being larger. A model that includes age and weight may better predict CT-derived lung volume.
**Limitations:** No gold standard TLC was available; CT is performed in supine position which may limit maximum inspiration.
**Ethics Committee Approval:** The institutional review board approved this study, and all participants signed informed consent.
**Funding for this study:** ImaLife is supported by an institutional research grant, Siemens Healthineers. The first author is supported by KNAW grant PSA-SA-BD-01.
**Author Disclosures:**
Mieneke Rook: Nothing to disclose
Geertruida de Bock: Nothing to disclose
Rozemarijn Vliegenthart: Research/Grant Support at Siemens Healthineers
Gert Jan Pelgrim: Nothing to disclose
Hendrik Joost Wisselink: Grant Recipient at KNAW
Danielle J.D. Steerenberg: Nothing to disclose

**Live Q&A**

**08:00-09:00 CHANNEL 5**

### Refresher Course: Head and Neck

### RC 208

### Dental imaging for general radiologists

**Moderator**
Edith Vassallo; Msida/MT

### RC 208-2
**A. Dental variants: what the general radiologist needs to know**
Andy Whyte; Perth/AU
*(andywhyte7@bigpond.com)*

**Learning Objectives:**
1. To revise the anatomy of teeth and surrounding structures.
2. To learn about commonly encountered dental variants.
3. To become acquainted with how to image and report such findings.

### RC 208-3
**B. Odontogenic cysts: sorting the wood from the trees**
Nicholas Drage; Cardiff/UK

**Learning Objectives:**
1. To become familiar with different types of odontogenic cysts.
2. To learn how best to image these lesions.
3. To review those imaging features that help differentiate lesions from one another.

### RC 208-4
**C. Reporting orthopantomogram (OPT): what and what not to report**
Kaan Orhan; Ankara/TR
*(call53@yahoo.com)*

**Learning Objectives:**
1. To review indications for OPT and when to perform other imaging.
2. To learn about artefacts on OPT and how to obtain better images.
3. To understand which pathology to comment upon and why.

**Live Q&A: Personal pearls and pitfalls in reporting**

**09:15-10:15 CHANNEL 2**

### Joint Session of the ESR and EFLM

### ESR/EFLM

### Clinical use cases for integrated diagnostics for laboratory medicine and radiology

**Moderators**
Michael H. Fuchsjäger; Graz/AT
Michael Neumaier; Mannheim/DE

### ESR/EFLM-3
**Cancer: personalised diagnostics in the detection of recurrence of metastatic colorectal cancer (part 1)**
Stefan O. Schönberg; Mannheim/DE

**Learning Objectives:**
1. To learn about novel approaches towards personalised cancer follow-up.
2. To understand the importance of individualised diagnostic pathways adapted to the biological aggressiveness of cancer.
3. To appreciate the advantages of integrated diagnostics in the detection of recurrence of metastatic colorectal cancer.

### ESR/EFLM-4
**Cancer: personalised diagnostics in the detection of recurrence of metastatic colorectal cancer (part 2)**
Michael Neumaier; Mannheim/DE
*(michael.neumaier@medma.uni-heidelberg.de)*

**Learning Objectives:**
1. To understand the clinical utility and merits of different methods for the characterisation of circulating nucleic acids in the blood.
2. To learn, how synergistic workflow between radiology and laboratory medicine will advance early detection of tumour recurrence.
3. To understand, how epigenetic profiling in blood samples can assist in identification of CUP syndromes and progeny of tumours.

### ESR/EFLM-5
**Cardiovascular: personalised management of patients with acute chest pain in the emergency department based on imaging and laboratory biomarkers**
Fabian Bamberg; Freiburg/DE
*(fabian.bamberg@uniklinik-freiburg.de)*

**Learning Objectives:**
1. To understand the clinical challenge of acute chest pain management in the emergency department.
2. To learn about the evidence of cardiac CT in this setting and appreciate its limitations in the era of high sensitivity troponin assays.
3. To define workflows that use laboratory and imaging parameter to manage patients with acute chest pain
**Author Disclosures:**
Fabian Bamberg: Research Grant/Support: Bayer Healthcare, Siemens Healthineers; Speaker: Bayer Healthcare, Siemens Healthineers

### ESR/EFLM-6
**COVID-19: prediction of prognosis based on laboratory data and chest CT**
Salvatore Cappabianca; Naples/IT
*(Salvatore.CAPPABIANCA@unicampania.it)*

**Learning Objectives:**
1. To clarify the place of CT scanner in the management of COVID-19 patients.
2. To define the possibilities of CT alone in the prediction of patients' outcome.
3. To compare lung CT impairment with clinical data to increase performance in outcome prediction.

### ESR/EFLM-7
**Trauma: value of biomarkers of brain injury in the diagnosis of head trauma**
Aad van der Lugt; Rotterdam/NL
*(a.vanderlugt@erasmusmc.nl)*

**Learning Objectives:**
1. To understand the differences between blood and imaging biomarkers in brain injury.
2. To learn about the role of biomarkers in diagnosis and prognosis of brain injury.
3. To understand the advantages of integrated diagnostics in head trauma.

**Live Q&A**

**09:15-10:15** **CHANNEL 4**

### EFRS meets Austria

### Meets 5

### Embracing our profession in Austria

**Moderators**
Charlotte Beardmore; London/UK
Sabine Weissensteiner; Vienna/AT

### Meets 5-3
**Introduction: radiographers in Austria**
Sabine Weissensteiner; Vienna/AT

**Learning Objectives:**
1. To introduce the radiography profession in Austria and the priorities of the national society.
2. To introduce Austria as a country, the Austrian lifestyle, and the Austrian culture.

### Meets 5-4
**Continuing professional development for radiographers: criteria in training and education**
Fritz Friedl; Vienna/AT

**Learning Objectives:**
1. To learn about the Austrian guidelines, the need for further education, and continuous professional development (CPD).
2. To explore the use of dedicated software to support the CPD programme and the individual health professional/radiographer.
3. To understand the challenges in implementing CPD guidelines and standards.

### Meets 5-5
**Masters programmes for Austrian radiographers**
Sabine Leitner; Wiener Neustadt/AT

**Learning Objectives:**
1. To become familiar with the different degree programmes for radiographers in Austria.
2. To consider the potential scope of practice for, and impact of, master's degree graduates.
3. To discuss the career challenges for master's degree graduates.

### Meets 5-6
**Interlude: Austria: beyond ECR**

### Meets 5-7
**Radiographer research in Austria**
Gerold Unterhumer; Vienna/AT

**Learning Objectives:**
1. To learn about radiographer research activity in Austria.
2. To discuss research opportunities available to Austrian clinical and academic radiographers.
3. To explore future plans for growing radiographer research involvement in Austria.

### Meets 5-8
**Evaluation of the eye lens dose in angiography**
Anna Suppnig; Klagenfurt/AT
*(anna.suppnig@gmx.at)*

**Learning Objectives:**
1. To consider EU Council Directive 2013/59/Euratom in terms of eye lens doses and dose measurement.
2. To learn about the Austrian guidelines for eye lens dose.
3. To explore the impact of eye lens dose measurement and methods for dose evaluation.

### Meets 5-9
**MedAustron: cancer treatment with particle therapy and the radiographers' role**
Harald Hentschel; Wiener Neustadt/AT

**Learning Objectives:**
1. To introduce the use of particle therapy for cancer treatment.
2. To identify the radiographers' role within particle therapy and the wider multidisciplinary team.
3. To consider future opportunities for radiographers involved in a particle therapy service.

**Live Q&A**

**09:15-10:15** **CHANNEL 5**

### Research Presentation Session: Genitourinary

### RPS 107

### The next generation of prostate interventions using state-of-the-art imaging techniques

**Moderator**
Bernd Hamm; Berlin/DE

### RPS 107-2
**Remote-controlled manipulated transrectal biopsy of the prostate: a promising technique**
H. Raat[1], *R. Van Stiphout*[1], A. van Gorp[1], J. O. Barentsz[2]; [1]Roermond/NL, [2]Nijmegen/NL
*(R.vanstiphout@lzr.nl)*

**Purpose:** MRI targeted prostate biopsy refers to an imaging targeted "in-bore "technique rather than the traditional systematic approach of a prostate biopsy with TRUS (Trans Rectal UltraSound) . Different techniques have been established and are in use: cognitive fusion biopsy, ultrasound-MRI fusion biopsy and MRI-guided-in-bore biopsy. In the Netherlands the MRI fusion technique is the most popular although the MRI in-bore technique is the most accurate technique.
**Methods or Background:** In-bore robot controlled prostate biopsy with de remote controlled manipulator in patients with PIRADS 4 or 5 lesions on detection MRI.TRUS or MRI fusion technique have limitations in reaching all suspect prostate lesions due to little size or not reachable locations such as very lateral or anterieur location. Or lesions in very big prostates.
**Results or Findings:** The manual in-bore technique is time-consuming and therefore costly because the patient has to be moved in and out of the MRI several times. There is no risk of fusion or cognitive inaccuracy. With this device the radiologist is able to position the transrectal needleguide in the control-room without moving the patient. We report our experience with a Remote Controlled Manipulator robot in the first 300+ patients.
**Conclusion:** The technique is feasible and less time-consuming than the manual technique. It's cost-effective and the results are similar to manual MRI-guided-in-bore biopsy.
**Limitations:** Patients must be MRI compatible and not claustrofobic. Patients endure head-first prone position for approximately 20 minutes.
**Ethics Committee Approval:** Approval is granted
**Funding for this study:** There is no funding for this study
**Author Disclosures:**
Rogier Van Stiphout: Nothing to disclose
Alexander van Gorp: Nothing to disclose
Jelle O. Barentsz: Nothing to disclose
Henricus Raat: Nothing to disclose

### RPS 107-3

**MR directed biopsy (MRDB) for prostate cancer detection: in-bore vs MR-transrectal ultrasound fusion technique, performed by the same operators**

*E. Messina*, R. Campa, S. Cipollari, M. Del Monte, C. Catalano, V. Panebianco; Rome/IT
*(emanuele.messina@uniroma1.it)*

**Purpose:** To compare the difference in the detection of overall PCa and csPCa of the in-bore (MRI-TB) and TRUS/MRI fusion prostate biopsy (FUS-TB), performed by the same operator. To compare MRI-TB and FUS-TB biopsy in terms of percentage of cancer per biopsy core and of number of cores per lesion.
**Methods or Background:** 263 patients was retrospectively enrolled. Inclusion criteria: age > 18 years old, high clinical suspicion for PCa (total PSA >4 ng/mL, or >2,5 in patients with family history, and/or a positive DRE). All patients underwent multiparamentric MRI and patients were directed to MR directed biopsy (MRDB) when PI-RADS score > 3.
**Results or Findings:** Total number of biopsy targets was 393: 197/393 were biopsied with the fusion technique and 196/393 with the in-bore technique.
A total of 988 cores where taken: 542 with the fusion technique and 446 with the in-bore biopsy.
No statistical difference was found in terms of detection rate of patients biopsied: CDR with the fusion technique was 62.4% (95% CI, 53.6 – 71.2) vs. 63.2% (95% CI, 54.1 – 72.4) for patients who underwent in- bore biopsy. Mean overall cores-per-target number was 2.5, with no statistical difference between techinques (2.3 and 2.8, respectively, p > 0.05). In-bore biopsy was more accurate yielding a mean percentage of 59.4% (IQR 40.0% - 65.0%) vs. 29.0% (IQR 16.6% - 60.0%) obtained by fusion technique (wilcoxon test p-value < 0.001).
**Conclusion:** No statistically significant difference was detected in terms of cancer detection rate comparing in-bore MRI and MRI-TRUS fusion targeted prostate biopsy. MRI-TB showed highest per-core malignant cells percentage and lower mean volume of the targets compared to FU-TB.
**Limitations:** Lack of pathology based on radical prostatectomy specimens. Single-institution retrospective study.
**Ethics Committee Approval:** Approved
**Funding for this study:** Not applicable
**Author Disclosures:**
Valeria Panebianco: Nothing to disclose
Stefano Cipollari: Nothing to disclose
Emanuele Messina: Nothing to disclose
Riccardo Campa: Nothing to disclose
Maurizio Del Monte: Nothing to disclose
Carlo Catalano: Nothing to disclose

### RPS 107-4

**In-bore MRI-guided prostate biopsy in a patient group with a high likelihood of mpMRI findings: a single centre experience**

*S. Durmaz*[1], B. Coskun[1], M. Kılıc[1], E. Altınmakas[1], A. Onay[2], B. Çolakoğlu[1], D. Cengiz[1], S. Akpek[1], M. Vural[1]; [1]Istanbul/TR, [2]Ankara/TR
*(drselahattindurmaz@gmail.com)*

**Purpose:** To determine the diagnostic yield of magnetic resonance imaging (MRI)-guided in-bore biopsy in patients with high likelihood multiparametric MRI (mpMRI) findings.
**Methods or Background:** This retrospective study consisted of 277 Prostate Imaging Reporting and Data System (PI-RADS) assessment category 4 and 5 targets in 246 consecutive patients (mean, 65.7 years; median prostate-specific antigen value, 7.75 ng/mL) who had undergone MRI-guided in-bore biopsy at our institution between December 2012 and March 2020. Cohort was consisted of primary (biopsy naive) (n=200) and secondary (had at least one negative biopsy) (n=46) patients subset. Eighty-one patients who were underwent RP as a definitive treatment, needle biopsy and RP specimen Gleason scores were evaluated in terms of concordance.
**Results or Findings:** In total, 277 targets biopsied from 246 patients and overall 216 cores were positive for prostate cancer. Overall PCa detection rates were 80.5% per patient (198/246) and 78% per target (216/277). Clinically significant prostate cancer (ISUP Gleason grade ≥ 2) was found 63% of patients. Diagnosis of prostate cancer rate was 68% and 92% in cases classified as PI-RADS assessment category 4 and 5, respectively.
A total of 81 patients (%32.9) underwent radical prostatectomy as a definitive treatment in our cohort. Among these, there were upgrade in the ISUP grade in 22 patients (27.2%), there were downgrade in 7 patients (8.6)% and concordance in 52 patients (64.2%).
A median of 3.2 cores was obtained per target and percentage of cancer involvement in positive biopsy cores was 53.2%.
**Conclusion:** MRI-guided in-bore prostate biopsy has a high detection rate of csPCa in PI-RADS assessment category 4 and 5 targets. Biopsy results were satisfactory in terms of number of positive cores, cancer volume in positive cores and concordance of GS in needle biopsy and RP specimen.
**Limitations:** Not applicable
**Ethics Committee Approval:** Not applicable
**Funding for this study:** Not applicable
**Author Disclosures:**
Emre Altınmakas: Nothing to disclose
Bülent Çolakoğlu: Nothing to disclose
Metin Vural: Nothing to disclose
Sergin Akpek: Nothing to disclose
Bilgen Coşkun: Nothing to disclose
Selahattin Durmaz: Nothing to disclose
Mert Kılıc: Nothing to disclose
Duygu Cengiz: Nothing to disclose
Aslihan Onay: Nothing to disclose

### RPS 107-5

**Reasons for missing clinically significant prostate cancer by targeted magnetic resonance imaging/ultrasound fusion-guided biopsy**

*M. Klingebiel*, L. Schimmöller, T. Ullrich, C. Arsov, P. Albers, G. Antoch; Düsseldorf/DE
*(Maximilian.Klingebiel@med.uni-duesseldorf.de)*

**Purpose:** This study evaluates cases of csPCa missed by targeted biopsy (TB) and analyzes the diagnostic impact of additional systematic biopsies (SB) in a large patient collective.
**Methods or Background:** Consecutive patients with 3T multiparametric MRI of the prostate and subsequent MRI/US fusion-guided TB plus 12-core US-guided SB from 01/2014 to 04/2019 were included in this study. Primary study endpoint was the analysis of cases with csPCa missed by TB. Secondary study endpoints were PCa detection and the correlation with clinical and MRI parameters.
**Results or Findings:** In total 785 patients met the inclusion criteria. 342 patients had csPCa (median PSAD 0.29 ng/ml/cm3). In 42 patients (13%), csPCa was detected only by SB. In 36 of these cases, the localization of the positive SB cores matched cancer suspicious regions (CRS) that were previously described on mpMRI. Cases with csPCA missed by TB showed insufficient MRI segmentation in 48%, insufficient lesion registration in 67% including 21% where the center of a larger or diffuse lesion was missed, and a generally small lesion size in 14%. Median PSAD of patients with nsPCa detected by SB was 0.15 ng/ml/cm³.
**Conclusion:** Main reasons for missing csPCa by TB were insufficient prostate segmentation (prostate boundary correlation) or imprecise lesion registration (lesion transfer, tracking or matching) during MRI/US fusion biopsy. Consequently, verification of MR image quality, exact MRI lesion assessment, and advanced biopsy experience may improve accuracy. Altogether, additional SB adds limited clinical benefit, especially in men with PSAD ≤0.15 ng/ml/cm³.
**Limitations:** The histopathologically non-blinded correlation of missed PCa for retrospective image review might be a source of bias. Moreover, no postoperative results after radical prostatectomy or follow-up of included patients evaluated in this study.
**Ethics Committee Approval:** Approved by local ethic committee.
**Funding for this study:** No funding.
**Author Disclosures:**
Maximilian Klingebiel: Nothing to disclose
Tim Ullrich: Nothing to disclose
Christian Arsov: Nothing to disclose
Peter Albers: Nothing to disclose
Lars Schimmöller: Nothing to disclose
Gerald Antoch: Nothing to disclose

### RPS 107-6

**MRI and clinical validation of transperineal laser ablation of benign prostatic hyperplasia in outpatient settings: an alternative effective treatment suitable for the COVID-19 outbreak**

G. Manenti, T. Perretta, A. Calcagni, D. Ferrari, S. Vidali, *C. Ryan*, F. R. Fraioli, D. D'Amato, R. Floris; Rome/IT

**Purpose:** Transperineal laser ablation (TPLA) of the prostate is a novel, mini-invasive option for men with lower urinary tract symptoms (LUTS) due to benign prostate hyperplasia (BPH). The aim is assessing the impact of US-guided TPLA regarding urodynamic improvement and sexual function, monitoring clinical data, post-procedural complications and define imaging findings using 3T multi-parametric MRI.
**Methods or Background:** Prospective interventional pilot study in patients affected by LUTS due to benign prostatic obstruction (BPO) treated by TPLA. 44 men aged 50 or older with moderate to severe LUTS and history of refractory, intolerance or poor compliance to medical therapies. Clinical measurements included PSA, Flow rate estimation, US post-voiding volume, sexual function, SHIM Questionnaire, Quality of Life questionnaire. Adverse events were evaluated using Clavien-Dindo scale. Volume changes were measured by 3T MRI and automatic segmentation software at 1 year follow-up
**Results or Findings:** Erectile and ejaculatory functions were maintained in all patients. MRI assessed the changes over time with a 53% reduction of adenoma volume and 71% of ablated area with clinical and functional improvement in all cases. The overall adverse event rate was 7%.
**Conclusion:** Clinical monitoring and 3T mpMR imaging at 1-year follow-up confirms US-guided TPLA as a safe, manageable and effective treatment for LUTS. Due to its mini-invasive technique, it should be considered a problem-solving treatment for BPH patients during a COVID 19 outbreak
**Limitations:** A further investigation with a long-term prospective multi-centric study is necessary. Another limitation is the lack of comparison studies with gold-standard therapies.
**Ethics Committee Approval:** Procedures were conducted accordingly with the ethical standards of the institutional research committee, in accordance with the guidelines of the Declaration of Helsinki.
**Funding for this study:** This study has not received any funding.
**Author Disclosures:**
Tommaso Perretta: Nothing to disclose
Donatella Ferrari: Nothing to disclose
Antonello Calcagni: Nothing to disclose
Guglielmo Manenti: Nothing to disclose
Sofia Vidali: Nothing to disclose
Dejanira D'Amato: Nothing to disclose
Colleen Ryan: Nothing to disclose
Roberto Floris: Nothing to disclose
Federico Romeo Fraioli: Nothing to disclose

### RPS 107-7

**Rates of infection post-MRI guided target trans-rectal prostate biopsy (TRUS) during the COVID-19 pandemic**
*P. Tansey*, P. Beddy, N. Sheehy, J. F. Meaney; Dublin/IE
*(paultansey1990@gmail.com)*

**Purpose:** Rates of infection post MRI guided target Trans-rectal prostate biopsy (TRUS) are increasing globally. During the COVID-19 pandemic from March to September 2020 as per national guidelines to help reduce hospital admissions, we changed our practice to targeted biopsies only rather than the traditional target and 12 core method at our institution. Given the reduction in core biopsies it is presumed that this may lead to a reduction in the overall rates of infections. The purpose of this study is to evaluate the rate of infections between the target only biopsies from March to September 2020, to the target/systematic biopsies from the same period in 2019.
**Methods or Background:** This study was carried out at St James's Hospital Radiology department. A search was performed using NIMIS for patients who underwent MRI guided targeted TRUS biopsies. Once identified, Electronic Patient Records (EPR) was used to follow-up complications post procedure. This was identified by emergency department admissions; outpatient letters or notes from the Urology clinical nurse specialist who acted as the point of contact for patients post procedure.
**Results or Findings:** A total of 120 target biopsies were performed in the 2020 period, 4 of which developed infections. In comparison, 97 target/systematic biopsies were performed in the 2019 period, 3 of which were associated with infections. There was no significant reduction in the rates of infection between the target and target/systematic groups with a non-significant p value of 0.9206.
**Conclusion:** We found that the post TRUS biopsy infection rates are in keeping with those found internationally. There was no significant reduction in the rates of infection between the target and the target/systematic biopsy group.
**Limitations:** Size of patient cohort.
**Ethics Committee Approval:** n/a
**Funding for this study:** n/a
**Author Disclosures:**
Peter Beddy: Nothing to disclose
Niall Sheehy: Nothing to disclose
Paul Tansey: Nothing to disclose
James F Meaney: Nothing to disclose

**Live Q&A**

**10:45-11:15 CHANNEL 2**

### PL 2
### Plenary Lecture

**Moderator**
Michael H. Fuchsjäger; Graz/AT

### PL 2-2

**How to live well and thrive on performance**
Thomas Bubendorfer; Monaco/MC
*(thomas@bubendorfer.com)*

**Live Q&A**

**11:45-12:45 CHANNEL 2**

### Joint Session of the ESR and ESTRO

### ESR/ESTRO
### MR-guided radiation therapy of gynaecological cancer

**Moderators**
Regina G.H. Beets-Tan; Amsterdam/NL
Henrike G. Westerveld; Amsterdam/NL

### ESR/ESTRO-3

**Functional MRI for cervical cancer**
Evis Sala; Cambridge/UK

**Learning Objectives:**
1. To review the role of functional MRI in the treatment selection of patients with cervical cancer.
2. To understand the role of functional MRI in radiotherapy treatment planning.
3. To learn about the potential of functional MRI in assessing treatment response and predicting outcome.

### ESR/ESTRO-4

**A first experience with the MR-linac in gynaecological tumours**
Luca Boldrini; Rome/IT
*(lucaboldrini@hotmail.it)*

**Learning Objectives:**
1. To become familiar with MR-linac gynaecological cancer treatment.
2. To learn about hybrid MR-guided adaptive treatment approaches.
3. To discuss possible future developments and opportunities.
**Author Disclosures:**
Luca Boldrini: Advisory Board: Victoria IBA; Consultant: Varian Medical Systems, ViewRay Inc; Patent Holder: 202020000005950; Speaker: ViewRay Inc.

### ESR/ESTRO-5

**MR anatomy of the uterus, cervix, and pelvic floor: all you need to know for accurate contouring**
Stephanie Nougaret; Montpellier/FR

**Learning Objectives:**
1. To review the basic anatomy of the uterus, cervix, and pelvic floor.
2. To understand the relevant anatomy prior to radiotherapy for the treatment of uterine cancers.
3. To understand the relevant anatomy prior to radiotherapy in the setting of pelvic relapse.

### ESR/ESTRO-6

**Image-guided adaptive brachytherapy in gynaecological tumours**
Remi Nout; Rotterdam/NL

**Learning Objectives:**
1. To become familiar with the concepts and methodology of MRI-based image-guided adaptive brachytherapy.
2. To review the key clinical outcomes when using this technique.
3. To discuss the possible future developments and opportunities.
**Author Disclosures:**
Remi Nout: Grant Recipient: Dutch Cancer Society, Dutch Research Council, Elekta, Varian, Accuray

**Live Q&A: MR-guided radiation therapy of gynaecological cancer: how should radiologists and radiotherapists work in synergy?**

**11:45-12:45** **CHANNEL 3**

### Refresher Course: Neuro

### RC 511

### Epilepsy imaging

**Moderator**
Horst Urbach; Freiburg/DE

### RC 511-2

**A. How to investigate epilepsy**
Horst Urbach; Freiburg/DE
*(horst.urbach@uniklinik-freiburg.de)*

**Learning Objectives:**
1. To understand the pathophysiology of epilepsy.
2. To learn how to image epilepsy in clinical practice.
3. To become familiar with advanced MRI in epilepsy management.

### RC 511-3

**B. How to report malformations of the cortical development**
Pia C. Maly Sundgren; Lund/SE

**Learning Objectives:**
1. To understand the pathophysiology of cortical development.
2. To recognise the malformations of cortical development.
3. To learn how to report the malformations of cortical development.

### RC 511-4

**C. How to image first seizure in adults?**
Jérome Hodel; Paris/FR

**Learning Objectives:**
1. To learn how to optimise imaging protocol in patients with first seizure.
2. To understand the specific value of arterial spin labelling (ASL) in such patients.
3. To learn how to distinguish between acute stroke, stroke mimics and seizure using MRI.

**Live Q&A: Would artificial intelligence bridge the gap in intractable epilepsy?**

**11:45-12:45** **CHANNEL 4**

### ESR meets Austria

### Meets 1

### Beats in the mountains

**Moderators**
Michael H. Fuchsjäger; Graz/AT
Rosemarie Forstner; Salzburg/AT

### Meets 1-3

**Introduction**
Wolfgang Schima; Vienna/AT
*(wolfgang.schima@khgh.at)*

**Learning Objectives:**
1. To learn about what makes hearts beat faster in Austria.
2. To understand major risks in the mountains.
3. To appreciate hearing the land of music.

### Meets 1-4

**Cardiac risk profiling in sports**
Christian Loewe; Vienna/AT
*(christian.loewe@meduniwien.ac.at)*

**Learning Objectives:**
1. To learn about sudden cardiac death in mountain hiking.
2. To understand cardiac risk factors in sports activities.
3. To appreciate coronary CTA in cardiac risk profiling.

**Author Disclosures:**
Christian Loewe: Author: BRACCO Springer; Speaker: Siemens Healthineers

### Meets 1-5

**Alpine emergency room management**
Helmut Schöllnast; Graz/AT

**Learning Objectives:**
1. To learn about the most frequent trauma cases in alpine regions.
2. To understand imaging algorithms in shock room management.
3. To appreciate multidisciplinary case reviews.

### Meets 1-6

**Hearing loss and modern implant rehabilitation**
Gerlig Widmann; Innsbruck/AT
*(gerlig.widmann@i-med.ac.at)*

**Learning Objectives:**
1. To learn about various hearing implants.
2. To understand relevant preoperative conditions.
3. To appreciate imaging in the postoperative evaluation of implants.

**Live Q&A: Open your heart, don't break, listen**

**11:45-12:45** **CHANNEL 5**

### Research Presentation Session: Radiographers

### RPS 114

### Quality improvement in radiography

**Moderators**
Mark F. McEntee; Cork/IE
Monique Brink; Nijmegen/NL

### RPS 114-2

**A retrospective clinical audit on the quality of positioning standards in plain film radiography**
*C. Nepomuceno*, M-L. Ryan, T. Neville, J. Potočnik, L. Walsh, A. Toohey, V. Douglas, L. A. Rainford; Dublin/IE
*(chelsea.nepomuceno@ucdconnect.ie)*

**Purpose:** Clinical audit is designed to compare current practices to a proven standard, enabling healthcare providers (HCPs) to improve the quality of patient care. While there are established methods of quality assurance in radiography e.g. reject analysis, there is an absence of literature pertaining to the quality of reported images and radiography/radiology consensus. This study aimed to address the paucity of research on the standards of reported radiographs.
**Methods or Background:** Radiology reported images were collated from 4 clinical sites governed by 2 HCPs, with 51 radiography staff, 10+ radiologists. Each HCP provided access to 200 images for 5 different radiographic projections: Antero-posterior (AP) ankle, lateral knee, AP shoulder, lateral elbow and wrist. Images were prepared for review (n=2000) and 5 radiography observers with one common to both HCPs scored each image for radiographic positioning using a four-point scale: 0) Unacceptable, 1) Borderline Acceptable, 2) Definitely Acceptable, and 3) Very Good. Images indicating optimal positioning were provided to the observers.Observer findings were analysed accumulatively for each radiographic projection and each HCP. Inter-observer and intra-observer differences were calculated.
**Results or Findings:** The results identified a substantial proportion of X-rays had suboptimal positioning, 36.3% recorded scores of 1 or below. Additionally, there were significant differences related to the perception of quality between the observers, demonstrating the subjective nature of image quality review.
**Conclusion:** Preliminary data has evidenced over one-third of reported images are borderline acceptable at best. Further research is warranted into radiography practice and how radiographers and radiologists judge diagnostic acceptability to inform future reject analysis developments and protocols.
**Limitations:** A larger study is recommended to include a greater number of clinical sites and radiographic examination types.
**Ethics Committee Approval:** An institutional waiver from full ethics was confirmed.
**Funding for this study:** Global Diagnostics Ireland
**Author Disclosures:**
Laura Walsh: Nothing to disclose
Tom Neville: Nothing to disclose
Andrea Toohey: Nothing to disclose
Chelsea Nepomuceno: Nothing to disclose

Louise A. Rainford: Nothing to disclose
Vicki Douglas: Nothing to disclose
Jaka Potočnik: Nothing to disclose
Marie-Louise Ryan: Nothing to disclose

### RPS 114-3

**Radiographers' view on image quality: does years of experience or country of practice influence image quality assessment**

*E. Kjelle*[1], A. K. Schanche[2], L. Hafskjold[1]; [1]Kongsberg/NO, [2]Drammen/NO
*(elin.kjelle@usn.no)*

**Purpose:** The aim of this study was to explore differences in radiographer's assessment of image quality based on years, or country of clinical practice.
**Methods or Background:** A questionnaire with 20 cases (plain radiography image and referral text) was made available to radiographers at the EFRS research HUB during ECR 2020. The cases were divided into three categories, based on the European guidelines of image quality, "Acceptable"(8), "Could be acceptable"(11), "Should be rejected"(11). Respondents assessed the image and text and decided whether to keep or reject the image. If an image was rejected the respondent gave a reason for rejection from a list of alternatives. In addition, respondents could elaborate on their choice (both keep and reject) in free text. R was used to perform 2-tailed chi-squared tests to explore difference between groups.
**Results or Findings:** Of the 546 radiographers visiting the online research HUB, 81 radiographers participated in this survey, a response rate of 15%. 62 of these were practicing in Europe, and 46 had practiced as a radiographers for >10 years. Radiographers with <10 years clinical practice kept 65% of images compared to 62% among radiographers with >10 years clinical practice. Comparing country of practice, radiographers in European countries kept 64% of the images compared to 61% among radiographers from the rest of the world. Comparing the groups based on image quality category gave no significant difference in reject rate.
**Conclusion:** This study showed that there was no significant difference in the number of rejected images comparing years and country of practice among radiographers.
**Limitations:** The number of respondents from countries outside Europe was small weakening the analyses for place of clinical practice.
**Ethics Committee Approval:** The Norwegian Center for Research Data (Ref:987929) approved this study.
**Funding for this study:** No funding given
**Author Disclosures:**
Linda Hafskjold: Nothing to disclose
Ann Kristin Schanche: Nothing to disclose
Elin Kjelle: Nothing to disclose

### RPS 114-4

**Optimisation of the AP abdomen projection in large patients**

*S. Gatt*, J. L. Portelli, F. Zarb; Msida/MT
*(sharon.gatt.16@um.edu.mt)*

**Purpose:** To identify optimal exposure parameters, delivering the lowest radiation dose while maintaining images of diagnostic quality for the AP abdomen projection in large patients.
**Methods or Background:** The study was composed of two phases. Phase 1: an anthropomorphic phantom (20cm AP abdominal diameter) was repetitively radiographed adding 15 layers (1cm thick) of fat reaching a maximum AP diameter of 35cm. For every 5cm thickness, images were taken at 10kVp intervals (80kVp-120kVp). DAP, mAs and measurements of SNR and CNR were recorded. Phase 2: image quality evaluation of resultant images by radiographers and radiologiststhrough Absolute VGA. VGA scores were analysed using VGC curves.
**Results or Findings:** Increasing kVp from 80kVp to 110kVp was found to be the optimal kVp setting for AP abdominal diameters: < 20cm (with a 56.5% decrease in DAP and 76.2% in mAs); >20 <25cm (with a 54.2% decrease in DAP and 76.2% decrease in mAs) and >25 < 30cm (with a 29.2% decrease in DAP and 59.7% decrease in mAs). Increasing the kVp from 80kVp to 120kVp was found to be the optimal kVp setting for AP abdominal diameter < 30 > 35 cm (with a 50.7% decrease in DAP and 73.4% decrease in mAs). All this while maintaining images of diagnostic quality.
**Conclusion:** Tailoring the exposure parameters for large patients in radiography of the abdomen results in a significant dose reduction while still maintaining diagnostic image quality.
**Limitations:** Using an anthropomorphic phantom.
kVp setting was the only parameter investigated.
The simulation of fat in large patients is still not 100% equivalent to its distribution.
**Ethics Committee Approval:** University of Malta Research and Ethics Committee (UREC), reference number: 2623_27082019_Sharon Gatt.
**Funding for this study:** No specific grant from any funding agency in the public, commercial or non-profit sectors was received.
**Author Disclosures:**
Sharon Gatt: Nothing to disclose
Jonathan Loui Portelli: Nothing to disclose
Francis Zarb: Nothing to disclose

### RPS 114-5

**Weight vs age comparison of diagnostic reference levels for paediatric body computed tomography examinations**

*A. Cestnik*[1], N. Mekis[2], I. Delakis[1]; [1]Doha/QA, [2]Ljubljana/SI
*(acestnik@sidra.org)*

**Purpose:** European guidelines recommend the use of weight groups for the development of paediatric diagnostic reference levels (DRLs) for body Computed Tomography (CT) examinations. However, patient weight is not always as readily available as age. The purpose of our study was to investigate differences in using age vs. weight groupings when developing paediatric DRLs for CT body examinations at our hospital.
**Methods or Background:** Our hospital is the only tertiary paediatric hospital in the country. The CT scanners are Definition Flash (Siemens Healthcare) and dose information is automatically collected with a dose management system (Radimetrics, Bayer). Patient age was extracted from PACS and patient weight from our radiology information system. Data collected was from 120 abdomen and 320 chest CT exams of children at different ages/weights. Data was filtered carefully to ensure exam consistency. Median CTDIvol and DLP values were calculated, and weight distributions were produced for different age groups.
**Results or Findings:** DRLs developed with either weight or age groupings were comparable at younger patient ages (<4y) for chest and abdomen CT exams. At older ages, the distribution of weights was skewed towards higher values for abdominal CT exams and lower values for chest CT exams. As a result, median CTDIvol and DLP values for older age groups were slightly higher for abdomen CT exams and lower for chest CT exams, than for their respective weight groups.
**Conclusion:** Calculating DRLs based on age groups for body CT exams was a reasonable alternative to using weight groups at our site only for younger ages (<4y). At older ages, weight groupings must be used because the distribution of patient weight is skewed, likely due to demographics or referral pathology.
**Limitations:** Hospital has one type/vendor of CT scanners
**Ethics Committee Approval:** n/a
**Funding for this study:** n/a
**Author Disclosures:**
Nejc Mekis: Nothing to disclose
Ioannis Delakis: Nothing to disclose
Anja Cestnik: Nothing to disclose

### RPS 114-6

**Effect of body weight on iodination for human foetal post-mortem micro-CT imaging**

*I. C. Simcock*, S. C. Shelmerdine, N. Sebire, O. J. Arthurs; London/UK
*(ian.simcock@gosh.nhs.uk)*

**Purpose:** Micro-CT is a novel imaging technique allowing high resolution diagnostic imaging in early gestational fetuses. Internal soft tissue contrast is imparted through submersion of the fetus in an exogenous contrast agent (i.e. potassium tri-iodide (I2KI)) which passively diffuses through the foetal skin. Optimal timing of foetal submersion is vital for adequate organ visualisation but can be variable. The aim of this study was to explore the relationship between foetal size and iodination timing.
**Methods or Background:** In this 1year, single centre, prospective study, all fetuses referred for micro-CT imaging were placed in 2.5% Iodine solution (100g KI and 50g I2 in 1000ml water, diluted 1:1 with 10% formalin) and scanned on a NIKON (Tring, UK) micro-CT scanner at regular intervals until fully iodinated. Fetuses were excluded if a delay in the examination time, insufficient I2KI or weakened solution was used. Foetal body weight and gestational age were evaluated against contrast immersion time.
**Results or Findings:** In total, 157 fetuses (gestational weights 5 – 298g) were examined. There was a linear relationship between body weight and immersion time with correlation coefficient R2 0.64. Total iodination time could be predicted using the formula time(days) = 0.03 x body weight(grams) + 2.2 (foetal weight 50g equates to adequate contrast immersion time of~3.7 days).
**Conclusion:** Post-mortem foetal body weight can be used to estimate the required contrast immersion time in preparation for micro-CT using a simple formula. This can help plan suitable time for scanning in a busy imaging service.
**Limitations:** Alternative concentrations of I2KI solution and higher body weights could be investigated.
**Ethics Committee Approval:** Ethical approval gained (13/LO/1994 +17/WS/0089).
**Funding for this study:** Supported by National Institute for Health Research, Medical Research Council, Royal College of Radiologists, Great Ormond Street Hospital Children’s Charity and NIHR GOSH BRC.

**Author Disclosures:**
Ian C. Simcock: Nothing to disclose
Neil Sebire: Nothing to disclose
Susan Cheng Shelmerdine: Nothing to disclose
Owen Arthurs: Nothing to disclose

### RPS 114-7

**Plaque inflammation-related metabolism and plaque features of instability: an initial exploration using corresponding PET/CT and MRI slices**

*N. Giannotti*[1], J. McNulty[2], S. J. Foley[2], S. Murphy[2], M. Marnane[2], G. Horgan[2], E. Kavanagh[2], M. J. O'Connell[2], P. Kelly[2]; [1]Dublin/IE, Sydney/AU, [2]Dublin/IE *(nicola.giannotti@sydney.edu.au)*

**Purpose:** The current assessment of carotid atherosclerotic lesions is based on luminal stenosis measurements; however, histopathologic studies demonstrate that morphological plaque characteristics of instability and inflammation may be associated with an increased risk for cerebrovascular events. Magnetic Resonance Imaging (MRI) and Positron Emission Tomography (PET) may identify patients at a higher risk to develop ischaemic cerebrovascular events. This study investigated whether plaque 18Fluoro-deoxy-glucose/PET (18F-FDG/PET) uptake is associated with plaque MRI features such as volume, intra-plaque haemorrhage, lipid accumulation, calcification, fibrotic cap (FC) thinning at selected corresponding locations.
**Methods or Background:** Inclusion criteria were: speech/motor TIA or non-severe stroke (Rankin ≤3); ipsilateral carotid stenosis ≥50%; age ≥50 years; carotid plaque MRI and 18FDG-PET/CTA performed. The MRI slice showing the largest accumulation of lipid was selected for the analyses; consequently, selected MRI carotid plaque morphological features were semi-automatically quantified. The maximum standardised uptake value (SUVmax) was measured on co-registered PET/CT images at the corresponding slice using the carotid bifurcation as a reference.
**Results or Findings:** Twenty-five symptomatic patients (72% men, mean age 65 years) were included in the analysis. Age showed a correlation with plaque calcium volume (p=0.001). In males, plaques were thicker (p=0.04) and presented greater lipid accumulation (p=0.013). FC volume was larger in males (p=0.018), smokers (p=0.026), and in patients with hypertension (p=0.089). SUVmax was correlated with increased plaque lipid accumulation at corresponding slices (p=0.001).
**Conclusion:** This study demonstrated novel correlations of non-invasive imaging biomarkers of inflammation-related plaque metabolism with morphological MRI markers of plaque instability. If replicated, our findings may support the application of combined MRI and PET to detect vulnerable plaque in future clinical practice and randomised trials.
**Limitations:** None
**Ethics Committee Approval:** Full institutional review board approval
**Funding for this study:** Irish Health Research Board;
Irish Institute of Radiography and Radiation Therapy Board
**Author Disclosures:**
Nicola Giannotti: Nothing to disclose
Gillian Horgan: Nothing to disclose
Sean Murphy: Nothing to disclose
Michael Marnane: Nothing to disclose
Jonathan McNulty: Nothing to disclose
Martin Joseph O'Connell: Nothing to disclose
Eoin Kavanagh: Nothing to disclose
Shane J Foley: Nothing to disclose
Peter Kelly: Nothing to disclose

**Live Q&A**

**14:15-15:15 CHANNEL 2**

### ESR QuADRANT Session

*Jointly organised by the ESR Audit and Standards Subcommittee and EuroSafe Imaging*

### ESR QuADRANT

### Quality improvement through audit in diagnostic radiology, radiotherapy, and nuclear medicine: a European project on behalf of the European Commission

**Moderator**
David C. Howlett; Eastbourne/UK

### ESR QuADRANT-2

**Introduction**
David C. Howlett; Eastbourne/UK *(david.howlett@nhs.net)*

**Learning Objectives:**
1. To appreciate the importance of clinical audit.
2. To review the role of audit within the EU-BSS.
3. To examine audit-related initiatives within the ESR.

### ESR QuADRANT-3

**The European Commission perspective**
Georgi Simeonov; Luxembourg/LU

**Learning Objectives:**
1. To understand the background of the European Commission.
2. To understand the background of the tender.
3. To consider the desired outcomes of QuADRANT.
4. To appreciate the role of clinical audit in enhancing patient care.

### ESR QuADRANT-4

**Aims, scope, and overview of the project**
David C. Howlett; Eastbourne/UK *(david.howlett@nhs.net)*

**Learning Objectives:**
1. To review the structure of the project.
2. To examine the timelines and outputs of the work packages.
3. To consider the challenges and potential benefits for QuADRANT.

### ESR QuADRANT-5

**Findings and output from the initial workshop**
Adrian Brady; Cork/IE *(adrianbrady@me.com)*

**Learning Objectives:**
1. To discuss the proceedings of the initial QuADRANT workshop.
2. To highlight key outcomes and recommendations.
3. To consider how this workshop will drive remaining work packages.

**Live Q&A: How to further integrate clinical audit into European radiology practice?**

**14:15-15:15 CHANNEL 3**

### E³ - Advanced Courses: How to Improve Your Expertise in Cardiothoracic Imaging

### E³ 1319

### Cardiovascular imaging in pregnancy

**Moderator**
Jean-Nicolas Dacher; Rouen/FR

### E³ 1319-2

**A. Pulmonary embolism: optimising patient's selection and radiation protection**
Christian Loewe; Vienna/AT
*(christian.loewe@meduniwien.ac.at)*

**Learning Objectives:**
1. To learn how to adapt CT acquisition protocol during pregnancy.
2. To become familiar with alternative imaging techniques.
3. To become familiar with alternative diagnoses.
**Author Disclosures:**
Christian Loewe: Author: BRACCO Springer; Speaker: Siemens Healthineers

### E³ 1319-3

**B. Acute aortic disease in pregnancy**
Ricardo P.J. Budde; Rotterdam/NL
*(r.budde@erasmusmc.nl)*

**Learning Objectives:**
1. To learn about risk factors and prevalence of acute aortic syndromes during pregnancy.
2. To learn how to adapt CT acquisition protocol during pregnancy.
3. To become familiar with alternative imaging techniques.

**E³ 1319-4**
**C. Imaging peripartum cardiomyopathy and other cardiac complications**
Alexis Jacquier; Marseille/FR

**Learning Objectives:**
1. To learn about prevalence and clinical presentation of cardiomyopathy during pregnancy.
2. To learn an appropriate diagnostic algorithm.
3. To become familiar with prognosis and outcome.

**Live Q&A**

**14:15-15:15 CHANNEL 4**

### Coffee & Talk (open forum) Session

*Organised by the EORTC*

### C 10

### Setting up and managing imaging trials

**Moderator**
Nandita M. deSouza; Sutton/UK

### C 10-2
**Challenges of introducing imaging biomarkers as end-points into clinical trials**
Anouk Neven; Brussels/BE
*(anouk.neven@eortc.org)*

**Learning Objectives:**
1. To learn what requirements are necessary for clinical trial end-points.
2. To appreciate the difficulties in introducing imaging biomarkers as end-points.
3. To understand what needs to be addressed to overcome the challenges.

### C 10-3
**How do I share my trial data?**
Marion Smits; Rotterdam/NL
*(marion.smits@erasmusmc.nl)*

**Learning Objectives:**
1. To learn what is needed when archiving data for external use.
2. To appreciate the challenges of setting up an accessible image archive.
3. To understand the limitations.

**Author Disclosures:**
Marion Smits: Consultant: Parexel Ltd (fees paid to institution); Speaker: GE Healthcare (honorarium paid to institution)

### C 10-4
**What is the shelf-life of imaging data?**
Luc Bidaut; Lincoln/UK
*(luc.bidaut@ieee.org)*

**Learning Objectives:**
1. To learn about the use of quantitative imaging data for comparative studies over time.
2. To appreciate the speed of the evolution of imaging technology.
3. To understand the pitfalls of quantitative analysis from banked data.

**Live Q&A**

**15:30-16:30 CHANNEL 2**

### Refresher Course: Physics in Medical Imaging

### RC 113

### Blue skies and current trends in digital radiography (DR), computed tomography (CT), and interventional radiology (IR)

**Moderator**
Jonas Andersson; Umeå/SE

### RC 113-2
**A. Updates and future perspectives of DR technology**
Juha Peltonen; Helsinki/FI
*(Juha.Peltonen@hus.fi)*

**Learning Objectives:**
1. To learn about existing DR technologies.
2. To learn about the state of the art in DR imaging.
3. To predict future developments in DR imaging.

**Author Disclosures:**
Juha Peltonen: Owner: Fieldie Oy

### RC 113-3
**B. Updates and future perspectives of MG technology**
Ioannis Sechopoulos; Nijmegen/NL
*(ioannis.sechopoulos@radboudumc.nl)*

**Learning Objectives:**
1. To learn about existing MG technologies.
2. To learn about the state of the art in MG imaging.
3. To predict future developments in MG imaging.

**Author Disclosures:**
Ioannis Sechopoulos: Research Grant/Support: Siemens Healthcare, ScreenPoint, Sectra Benelux, Volpara Solutions, Canon Medical; Speaker: Siemens Healthcare

### RC 113-4
**C. Updates and future perspectives of CT technology**
Marc Kachelrieß; Heidelberg/DE
*(marc.kachelriess@dkfz.de)*

**Learning Objectives:**
1. To learn about existing CT technologies.
2. To learn about the state of the art in CT imaging.
3. To predict future developments in CT imaging.

### RC 113-5
**D. Updates and future perspectives of IR and angiography technology**
Nicholas Marshall; Leuven/BE
*(nicholas.marshall@kuleuven.be)*

**Learning Objectives:**
1. To learn about existing IR technologies.
2. To learn about the state of the art in IR.
3. To predict future developments in IR.

**Author Disclosures:**
Nicholas Marshall: Research Grant/Support: Siemens Healthineers

**Live Q&A: What can we expect from new detectors, equipment designs, and post-processing/reconstruction?**

**15:30-16:30** **CHANNEL 4**

### Special Focus Session

### SF 8

### The most challenging bowel requests and how to deal with them

**Moderator**
Marc Zins; Paris/FR

### SF 8-2

**Intestinal anastomosis: a leak or not a leak? That is the question**
Damian J.M. Tolan; Leeds/UK
*(djmtolan@doctors.org.uk)*

**Learning Objectives:**
1. To learn about the diagnostic performance of imaging methods for detecting anastomotic leaks after GI surgery.
2. To learn about the optimal CT imaging protocol.
3. To understand the treatment of anastomotic leakages and indications for abscess drainage.

**Author Disclosures:**
Damian J.M. Tolan: Grant Recipient: UK NIHR Intact Trial

### SF 8-3

**Obstructed defecation syndrome? Just do MRI-defecography**
Luis Curvo-Semedo; Coimbra/PT
*(curvosemedo@gmail.com)*

**Learning Objectives:**
1. To learn about the main functional anatomic landmarks in the radiological evaluation of obstructed defecation syndrome.
2. To address the diagnosis of anorectal dyssynergia with an emphasis on how imaging techniques such as dynamic pelvic floor MRI and evacuation proctography complement and enhance the role of anorectal physiology.
3. To understand how to use structured reporting methods for MRI of the pelvic floor in clinical practice.

### SF 8-4

**Internal hernia? Not always going along with bowel obstruction**
Sabine Schmidt Kobbe; Lausanne/CH
*(sabine.schmidt@chuv.ch)*

**Learning Objectives:**
1. To learn about the clinical findings and the radiological evaluation of the most common internal hernias.
2. To learn about the optimal CT imaging protocol.
3. To specifically discuss the challenging diagnosis of collapsed closed-loop form of small bowel obstruction from internal hernia.

**Live Q&A: How to improve diagnostic performance in challenging bowel requests?**

**15:30-16:30** **CHANNEL 5**

### CTiR 2

### Clinical Trials in Radiology 2

**Moderators**
Marc Dewey; Berlin/DE
Regina G.H. Beets-Tan; Amsterdam/NL

### CTiR 2-3

**Diagnostic accuracy of a convolutional neural network assessment of solitary pulmonary nodules compared with PET/CT and DCE-CT**
*J. R. Weir-Mccall*[1], E. Debruyn[2], S. Harris[3], N. Qureshi[1], R. Rintoul[1], F. Gleeson[4], F. J. Gilbert[1]; [1]Cambridge/UK, [2]Chicago, IL/US, [3]Southampton/UK, [4]Oxford/UK
*(jw2079@cam.ac.uk)*

**Purpose:** Solitary Pulmonary Nodules (SPNs) measuring 8-30 mm in diameter require further work-up to determine likelihood of malignancy. The purpose of the current study was to examine the diagnostic performance of a Lung Cancer Prediction Convolutional Neural Network (LCP-CNN) in nodules found on CT compared to current clinical work-up.
**Methods or Background:** This study was a post-hoc analysis of the SPUTNIK trial. This prospective multi-centre study compared the diagnostic accuracy of dynamic contrast-enhanced computed tomography (DCE-CT) with positron emission tomography (PET/CT) in SPNs measuring 8–30 mm in diameter. The LCP-CNN was designed and validated in an external cohort of non-contrast CTs. LCP-CNN risk scores were generated from the non-contrast CT images from the DCE-CT. The gold standard was histology or 2-years of follow-up. The area under the ROC curves (AUROC) when predicting cancer diagnosis were calculated using LCP-CNN score, SUVmax and DCE-CT maximum enhancement as predictive variables and compared using DeLongs test.
**Results or Findings:** 270 (68.3±8.8 years old, 49% female) participants underwent PET/CT and DCE-CT with CT data available centrally for AI-analysis. The nodule size was 15.8±5.5mm, with 61% malignant. Malignant nodules had a higher SUVmax (6.38±5.35 vs 1.95±1.83, p<0.001), a greater degree of enhancement (61.4±58.3 vs 34.7±25.4HU) and a higher LCP-CNN score (78.0±22.6 vs 41.4±29.2, p<0.001). The accuracy of the LCP-CNN on the non-contrast CT (AUROC= 0.83 (95%CI 0.79;0.88)) was superior to DCE-CT (AUROC= 0.76 (95% CI 0.69;0.82), p=0.03), and equivalent to PET/CT (AUROC= 0.86 (95%CI 0.81;0.90, p=0.35)).
**Conclusion:** A LCP-CNN algorithm provides an AUROC equivalent to PET/CT in the diagnosis of solitary pulmonary nodules. Routine incorporation of this into clinical practice may speed time to diagnosis with less resource utilization.
**Limitations:** Higher rate of malignancy than in screening trials
**Ethics Committee Approval:** Approved
**Funding for this study:** NIHR HTA Programme (grant no: 09/22/117)
**Author Disclosures:**
Elise Debruyn: Nothing to disclose
Fiona J. Gilbert: Nothing to disclose
Fergus Gleeson: Shareholder at Shareholder in Optellum
Jonathan R. Weir-Mccall: Nothing to disclose
Robert Rintoul: Nothing to disclose
Scott Harris: Nothing to disclose
Nagmi Qureshi: Nothing to disclose

### CTiR 2-4

**Discussant**
Mark O. Wielpütz; Heidelberg/DE
*(wielpuetz@uni-heidelberg.de)*
**Author Disclosures:**
Mark O. Wielpütz: Grant Recipient at Vertex, Boehringer Ingelheim

### CTiR 2-5

**Cost-effectiveness of MRI screening for women with extremely dense breast tissue**
*A. Geuzinge*[1], M. F. Bakker[2], E. Heijnsdijk[1], N. van Ravesteyn[1], W. B. Veldhuis[2], R. M. Pijnappel[2], R. Mann[3], C. van Gils[2], H. J. de Koning[1]; [1]Rotterdam/NL, [2]Utrecht/NL, [3]Nijmegen/NL
***No abstract available***
**Author Disclosures:**
Carla van Gils: Grant Recipient at Bayer AG Pharmaceuticals
Nicolien van Ravesteyn: Nothing to disclose
Eveline Heijnsdijk: Nothing to disclose
Amarens Geuzinge: Nothing to disclose
Harry J. de Koning: Nothing to disclose
Wouter B. Veldhuis: Nothing to disclose
Ritse Maarten Mann: Nothing to disclose
Ruud M Pijnappel: Nothing to disclose
Marije F. Bakker: Nothing to disclose

### CTiR 2-6

**Discussant**
Fiona J. Gilbert; Cambridge/UK
*(fjg28@cam.ac.uk)*
**Author Disclosures:**
Fiona J. Gilbert: Speaker at GE Healthcare

### CTiR 2-7

**Quality of life outcomes from a randomized controlled trial comparing drug-coated balloon to conventional balloon angioplasty for below-the-knee arteries in patients with critical limb ischaemia**
*B. S. Tan*, Z. Kun Da, A. Patel, F. Irani, D. Matchar, N. Sivapragasam, U. Pua, L. Sum, K. H. Tay; Singapore/SG

**Purpose:** To report the quality of life (QOL) results of a randomized controlled trial comparing drug-coated balloon (DCB) to conventional angioplasty (PTA) for below-the-knee (BTK) arteries in patients with critical limb ischemia (CLI).
**Methods or Background:** In this investigator-initiated, dual centre, blinded, randomized controlled trial (NCT02129634), 138 patients (93 males) with mean age of 62.6 years (std=9.9), were randomized to either DCB (n=70) or PTA

(n=68). 94.2% of the patients had diabetes and 52.9% had end stage renal failure (ESRF). QOL measured with the EQ-5D over the follow up period of 12 months were compared between the 2 groups after adjustment for baseline utility scores.
**Results or Findings:** Baseline QOL were similar in both groups. There was no significant difference in adjusted QALY gained at 12 months in both groups. The 6-month target lesion angiographic primary patency (primary endpoint) (DCB 42.9% vs PTA 38.2%, p=0.475) and 12-month limb salvage rate (DCB 75.0% vs PTA 84.6%, p=0.163.) were not statistically significant. The 12-month amputation-free survival (AFS) was however better in the PTA group (DCB 58.6% vs CB 77.9%, log rank test p = 0.012).
**Conclusion:** The use of DCB angioplasty for BTK interventions for CLI did not show superiority in the QALY gained at 12 months compared to PTA angioplasty. The 6-month angiographic patency of target lesion and 12-month limb salvage rate were also similar. AFS was however significantly different at 12 months, in favor of the PTA group.
**Limitations:** The operators were not blinded and ascertainment bias cannot be excluded.
**Ethics Committee Approval:** Yes.
**Funding for this study:** National Medical Research Council, Singapore
**Author Disclosures:**
David Matchar: Nothing to disclose
Nirmali Sivapragasam: Nothing to disclose
Leong Sum: Nothing to disclose
Kiang Hiong Tay: Nothing to disclose
Farah Irani: Nothing to disclose
Zhuang Kun Da: Nothing to disclose
Bien Soo Tan: Nothing to disclose
Ankur Patel: Nothing to disclose
Uei Pua: Nothing to disclose

### CTiR 2-8
**Discussant**
Stevo Duvnjak; Odense/DK
*(duvnjak.stevo@gmail.com)*
**Author Disclosures:**
Stevo Duvnjak: Nothing to disclose

### CTiR 2-9
**Brain atrophy and endovascular treatment effect in acute ischaemic stroke**
*S. Luijten*[1], A. van Es[2], Y. Roos[3], C. B. L. M. Majoie[3], R. van Oostenbrugge[4], W. Van Zwam[4], D. Dippel[1], A. Van Der Lugt[1], D. Bos[1]; [1]Rotterdam/NL, [2]Leiden/NL, [3]Amsterdam/NL, [4]Maastricht/NL (s.luijten@erasmusmc.nl)
***No abstract available***
**Author Disclosures:**
Aad Van Der Lugt: Grant Recipient at Dutch Heart Foundation, Dutch Brain Foundation, The Netherlands Organisation for Health Research and Development, Health Holland Top Sector Life Sciences & Health, Stryker European Operations BV, Thrombolytic Science, LLC, Penumbra, Medtronic and Cerenovus for new trials in acute stroke treatment
Sven Luijten: Nothing to disclose
Charles Bernardus Lucia Maria Majoie: Shareholder at Nico.lab Grant Recipient at CVON/Dutch Heart Foundation, European Commission, TWIN Foundation and Stryker
Diederik Dippel: Grant Recipient at Dutch Heart Foundation, Dutch Brain Foundation, The Netherlands Organisation for Health Research and Development, Health Holland Top Sector Life Sciences & Health, Stryker European Operations BV, Thrombolytic Science, LLC, Penumbra, Medtronic and Cerenovus for new trials in acute stroke treatment
Adriaan van Es: Nothing to disclose
Willem Van Zwam: Grant Recipient at Cerenovus® and Stryker®
Yvo Roos: Grant Recipient at CVON/Dutch Heart Foundation Shareholder at Nico.lab
Robbert van Oostenbrugge: Nothing to disclose
Daniel Bos: Nothing to disclose

### CTiR 2-10
**Discussant**
Johan Van Goethem; Antwerp/BE
*(johan.vangoethem@uantwerpen.be)*
**Author Disclosures:**
Johan Van Goethem: Nothing to disclose

**16:45-17:45 CHANNEL 2**

### Multidisciplinary Session

### MS 1

### Venous thromboembolic disease

**Moderator**
Gerard O'Sullivan; Galway/IE

### MS 1-2
**Haematology management in VTE: which patients for intervention, which patients for conservative? My clinical perspective**
Ruth Gilmore; Galway/IE

**Learning Objectives:**
1. To describe why some patients are more suitable for intervention and others less so.
2. To discuss the anticoagulation management around catheter directed thrombolysis and why fibrinogen even matters.
3. To discuss anticoagulation regimes post op and when to choose low molecular weight heparin vs warfarain vs NOAC.

### MS 1-3
**Patient selection and interventional options in deep vein thrombosis**
Douglas Mulholland; London/UK
*(doug2057@gmail.com)*

**Learning Objectives:**
1. To understand which patients I am keen to treat and why.
2. To show which treatment options I favour.
3. To share my worst disaster in DVT intervention and how we managed it.

### MS 1-4
**Patient selection and interventional options in pulmonary embolus?**
Stephen T. Kee; Dublin/IE
*(stephentkee@gmail.com)*

**Learning Objectives:**
1. To understand which patients I am keen to treat and why.
2. To show which treatment options I favour.
3. To share my worst disaster in PE intervention and how we managed it.

### MS 1-5
**Precise, patient-directed imaging in DVT and PE**
Carsten Arnoldussen; Maastricht/NL

**Learning Objectives:**
1. To describe the various imaging modalities for DVT and PE.
2. To describe my preferred modalities for DVT and PE.
3. To discuss future directions, particularly with respect to MRV and thrombus aging.

### MS 1-6
**Multidisciplinary case presentation**
Gerard O'Sullivan; Galway/IE
*(gerard.osullivan2@hse.ie)*
**Author Disclosures:**
Gerard O'Sullivan: Advisory Board: Medtronic Philips; Consultant: BSCI COOK MEDTRONIC BD WHITESWELL CREGANNA VETEX MARVAO; Patent Holder: VETEX ; Share Holder: VETEX Orthosensor

**Live Q&A**

16:45-17:45 CHANNEL 5

### Research Presentation Session: Oncologic Imaging

### RPS 216

### Protocol optimisation and quality control for advanced oncologic imaging techniques

**Moderator**
Helmut Ringl; Vienna/AT

### RPS 216-2

**Dose to oncologic patients in multi-phase CT examination: a single-centre survey**

*P. De Marco*, D. A. Origgi; Milan/IT
*(paolo.demarco@ieo.it)*

**Purpose:** Aim of this work is to evaluate effective dose to oncologic patients in multi-phase CT examinations.
**Methods or Background:** 18987 staging and follow-up examinations performed at our institution from January 2018 to September 2020 on 2 different scanners were collected with a dose-tracking software (Radimetrics, Bayer HealthCare) and were filtered and aggregated according to body part examined and number of acquisitions for each examinations.Numbers of examinations and relative frequency (RF) of the number of phases are reported.Effective doses (E) obtained from organ doses were calculated by the software, results are presented as median and interquartile ranges (IQR).
**Results or Findings:** 8275 Chest-Abdomen-Pelvis (CAP) examinations presented E of 13.6, 21.0, 26.9 and 33.5 mSv, for 1 to 4 phases respectively and a RF of 27%,22%,26% and 25% each (IQR 11.2-16.8, 16.5-26.2, 21.9-35.2, 26.2-43.9 mSv). 901 Abdomen exams performed with 4 (62%) or 5 (38%) phases, had E of 28.6 and 36.1 mSv, respectively (IQR 20.8-37.5 and 28.0-49.0 mSv), while 1972 Abdomen-Pelvis showed E of 10.4, 18.7, 26.9 and 33.0 mSv for 1(22%), 2(14%), 3 (26%) and 4 (38%) phases (IQR 8.1-13.9, 14.2-23.7, 21.2-35.4, 26.0-42.4 mSv). 3495 single-phase Chest exams had an effective dose of 7.6 mSv (IQR 6.0-9.7 mSv). 4344 Head-CAP examinations with 2 phases on the head and 1-to-4 phases on the body (RF 17%,24%,34% and 25%) exposed patients to median E of 17.5, 24.4, 30.5 and 36.8 mSv as number of phases increase (IQR 14.7-21.4, 19.9-30.2, 25.1-37.7, 29.6-46.1 mSv).
**Conclusion:** Oncologic patients undergoing multi-phase CTs are exposed to significant doses: considering the high numbers of examinations performed during the diagnostic and therapeutic path, the need of optimization is highly relevant and poses challenges to medical physicists, radiologists and radiographers involved.
**Limitations:** None.
**Ethics Committee Approval:** Not required.
**Funding for this study:** None.
**Author Disclosures:**
Daniela Anna Origgi: Nothing to disclose
Dipl.-Phys. Paolo De Marco: Nothing to disclose

### RPS 216-3

**Assessment of repeatability, robustness and performances of T2-mapping based radiomics features: a comparative study**

*A. Crombe*[1], X. Buy[1], F. Han[2], S. Toupin[3], M. Kind[1]; [1]Bordeaux/FR, [2]Los Angeles, CA/US, [3]Saint-Denis/FR

**Purpose:** MRI-based radiomics features are sensitive to several acquisition and post-processing parameters, including the signal intensity harmonization technique (IHT). Our aim was to investigate if a quantitative T2-mapping could help overcoming this issue.
**Methods or Background:** Twenty-six healthy adult volunteers prospectively underwent two abdominal MRI-scans at 1.5-T (MAGNETOM Aera) including radial TSE multi-echo T2-mapping (prototype) and classical TSE T2-WI with prospective motion correction. Six volumes-of-interest (VOIs) were manually drawn (liver, kidney, pancreas, muscle, bone, spleen). After sequences co-registration, initial T2-WIs were further post-processed with two IHTs: standardization and histogram-matching (HM) providing four paired datasets (initial T2-WI, standardized T2-WI, HM T2-WI and T2-mapping). The VOIs were propagated on all sequences to extract 45 RFs from MRI-1 and MRI-2 of each dataset. The influence of the datasets on RF values, test-retest reproducibility of RFs (through intra-class correlation coefficient [ICC]), robustness of RFs over multiple segmentations and correlations between k-means clustering labels and the six segmented tissues depending on the RFs dataset (through adjusted-Rand-index [ARI]) were investigated.
**Results or Findings:** 41/45 (91.1%) RFs were significantly influenced by the datasets (p-values range:<0.0001-0.192). The T2-sequence and/or its post-processing was the most influent factor on the repeatability of RFs (F-value=174.9, p-value<0.0001). In the six tissues, the test-retest ICCs from T2-mapping displayed intermediate values between initial T2-WI (lowest reproducibilities) and HM T2-WI (highest reproducibilities). The number of robust RFs was: 38/45 (84.4%) with HM T2-WI, 35/45 (77.8%) with standardized T2-WI and T2-mapping and 28/45 (62.2%) with initial T2-WI. The best discrimination of tissues was reached with the T2-mapping and the worse with HM T2-WI (ARI=0.624 and 0.152, respectively).
**Conclusion:** The best compromise between repeatability, robustness and no loss of information was obtained with the T2-mapping.
**Limitations:** No phantom; single-center; number of volunteers
**Ethics Committee Approval:** IRB-approved
**Funding for this study:** None
**Author Disclosures:**
Xavier Buy: Nothing to disclose
Amandine Crombe: Nothing to disclose
Solenn Toupin: Employee at Siemens healthineers
Michèle Kind: Nothing to disclose
Fei Han: Employee at Siemens Healthineers

### RPS 216-4

**Subsolid nodule detection in low-dose photon-counting CT of the chest**

*E. Wehrse*, L. T. Rotkopf, P. Mayer, C. P. Heussel, C. H. Ziener, S. Delorme, M. Kachelrieß, H-P. Schlemmer, S. Sawall; Heidelberg/DE
*(e.wehrse@dkfz-heidelberg.de)*

**Purpose:** To evaluate the potential of photon-counting (PC) CT for the detection of subsolid nodules (SN) in low-dose chest acquisitions compared to conventional energy-integrating (EI) CT.
**Methods or Background:** A model of lung parenchyma with SN was measured using an experimental CT equipped with an EI and a PC detector (SOMATOM CounT, Siemens Healthineers). The model consisted of 20 slices of polyurethane foam (-770 HU at 120 kV), placed in a semi-anthropomorphic thorax phantom (QRM Möhrendorf). In computationally generated randomly distributed positions, 0.7 mm holes were inserted and filled with a mixture of a water-based gel and palmitic-acid-based foam. Dose-matched acquisitions (CTDIvol,32cm  2.7 mGy) at 120 kV were performed using both detectors. Reconstruction was performed on a 1024×1024 matrix using a slice thickness of 1 mm and a B70f kernel. Two board-certified radiologists and three residents assessed the image stack with the task of identifying all SN. A nodule was considered correctly identified if the user clicked within 2.5 mm of the correct nodule position.
**Results or Findings:** Image noise was 85 HU in PC and 120 HU in EI images at the same dose and resolution. In total, 103 nodules were placed in the phantom (-340 ±120 HU). Lesion-based sensitivity is considerably higher in PC (median 51%, range 26-69%), compared to EI CT (median 40%, range 10-56%), leading to a relative increase of sensitivity of 25%. Specificity was >99.0% for both detectors.
**Conclusion:** Our results demonstrate the advantages of PC CT compared to EI CT, i.e. lower noise-levels and higher contrast-to-noise ratios at constant dose. This translates into an increase of accuracy in the detection of SN in low-dose chest CT.
**Limitations:** The influence of lung parenchyma texture, nodule shapes and sizes remains to be investigated.
**Ethics Committee Approval:** n/a
**Funding for this study:** n/a
**Author Disclosures:**
Claus Peter Heussel: Nothing to disclose
Christian Herbert Ziener: Nothing to disclose
Stefan Delorme: Nothing to disclose
Lukas Thomas Rotkopf: Nothing to disclose
Philipp Mayer: Nothing to disclose
Heinz-Peter Schlemmer: Nothing to disclose
Marc Kachelrieß: Nothing to disclose
Stefan Sawall: Nothing to disclose
Eckhard Wehrse: Nothing to disclose

### RPS 216-5

**Optimisation of contrast medium volume for abdominal CT in oncologic patients: a prospective comparison between fixed and lean body weight-adapted dosing protocols**

*B. Bracci*, M. Zerunian, G. Guido, F. Pucciarelli, C. Rucci, M. Polici, E. Lucertini, A. Laghi; Rome/IT

**Purpose:** Patient body size represents the main determinant of parenchymal enhancement and by adjusting the contrast medium (CM) dose to patient weight may be a more appropriate approach to avoid a patient over dosage of

Friday

CM. Thus, we aims to compare the performance of fixed-dose and lean body weight (LBW)-adapted contrast medium dosing protocols, in terms of image quality and parenchymal enhancement.
**Methods or Background:** One-hundred cancer patients undergoing multiphasic abdominal CT were prospectively enrolled in this multicentric study and randomly divided in two groups: patients in fixed-dose group (n=50) received 120 mL of CM (Iomeprol 350 mg/mL) while in LBW group (n=50) the amount of CM was computed according to the patient's LBW. Signal-to-noise ratio (SNR), contrast-to-noise-ratio(CNR) and contrast enhancement index (CEI) were compared in the two groups on arterial and portal-venous phase in splanchnic organs.
**Results or Findings:** LBW protocol group received a significantly lower amount of CM (103.47 ± 17.65 mL vs 120.00 ± 0.00 mL, P<0.001). Arterial kidney SNR and CNR and pancreatic CNR were significantly higher in LBW group (all P≤0.004). LBW group provided significantly higher arterial liver, kidney, and pancreatic CEI and portal-venous phase kidney CEI (all P≤0.002). Significantly lower portal-venous SNR and CNR were observed in LBW-Group (all P≤0.020).
**Conclusion:** LBW-adapted CM administration for abdominal CT reduces the volume of injected CM and improves both image quality and parenchymal enhancement. In patients affected by chronic diseases should be evaluated the option to standardize an LBW-adapted protocol in order to reduce the risks of acute kidney failure caused by contrast medium.
**Limitations:** Small population sample.
**Ethics Committee Approval:** Study approved by local institutional review board; informed consent received from all patients.
**Funding for this study:** No.
**Author Disclosures:**
Francesco Pucciarelli: Nothing to disclose
Carlotta Rucci: Nothing to disclose
Marta Zerunian: Nothing to disclose
Benedetta Bracci: Nothing to disclose
Andrea Laghi: Nothing to disclose
Michela Polici: Nothing to disclose
Gisella Guido: Nothing to disclose
Elena Lucertini: Nothing to disclose

### RPS 216-6

**Proposal for an improved HCC-diagnostic protocol for perfusion-CT quantification**
*K. Ekert*[1], M. Horger[1], W. Thaiss[2]; [1]Tübingen/DE, [2]Ulm/DE

**Purpose:** To refine the perfusion-CT (PCT) examination-protocol in patients with liver cirrhosis and suspected hepatocellular carcinoma (HCC) capable to replace 3-4-phase contrast-enhanced-CT while keeping diagnostic accuracy and qualitative assessment of tumor enhancement patterns (wash-in/wash-out) at a comparable radiation dose.
**Methods or Background:** 123 liver PCT examinations were evaluated retrospectively in 76 patients (mean age 64.8 ± 10.0 years, 6 female) between October 2015 and December 2016 for suspected HCC. PCT (temporal-resolution 1.5sec/80kVp/100mAs for 40sec) was used and arterial liver perfusion (ALP), portal-venous perfusion (PVP) and hepatic perfusion index (HPI) were quantified. Two additional spiral data sets after 80 sec were included to generate subtraction maps for arterial phase (AP, 18.5sec post injection), early (EPVP, 40sec) and late portal-venous phase (LPVP, 86sec), as well as LPVP minus EPVP to quantify wash-out.
**Results or Findings:** 141 lesions were analyzed. In 10 examinations no HCC was found at PCT. HCCs could be identified and characterized by wash-in and wash-out enhancement patterns as well as in terms of ALP (mean 45.6□22.9 mL/100mL/min), PVP (7.8□14 mL/100mL/min) and HPI (88.1□18.9%). Dose length product for perfusion-CT was 1003±89 mGy x cm, resulting in a mean effective dose of 15.05 mSv and standard abdominal four phase CT examination of 16.75 mSv.
**Conclusion:** Perfusion-CT with added late phase imaging can provide the information necessary to characterize and diagnose HCC without the need for additional equilibrium phase acquisitions with comparable radiation dose to standard abdominal three or four phase CT and additional perfusion-based tumor characterization.
**Limitations:** There was no histologic proof of HCC, but indirect confirmation by non-invasive imaging and follow-up.
**Ethics Committee Approval:** This retrospective study was approved by the ethics committee.
**Funding for this study:** No funding received.
**Author Disclosures:**
Marius Horger: Author at senior author
Kaspar Ekert: Nothing to disclose
Wolfgang Thaiss: Nothing to disclose

### RPS 216-7

**Impact of attenuation correction CT parameters on PET reconstruction**
*K. Binzel*, C. L. Wright, M. V. Knopp; Columbus, OH/US

**Purpose:** In vivo validation of equivalence of ultra-low dose attenuation CT for clinical PET reconstruction.
**Methods or Background:** Five canines underwent 18F-FDG PET imaging on a next-generation digital photon counting system (Philips Vereos). 75 minutes post-injection a 90 second/bed PET acquisition was performed over the whole body. Multiple CT series were acquired for validation. First, the standard protocol utilizing 120kVp, 150mAs, 4 and 2 mm slice thickness followed by ultra-low dose (uLD) acquisition with 80kVp, 20 (n=3) or 25 (n=2) mAs, at 4mm. The same PET acquisition was reconstructed using each different CT series.
**Results or Findings:** Blinded review found no significant differences in image quality or hot spot conspicuity among the differently reconstructed PETs. Using the full dose 4mm CT corrected PET images as reference, there was an average of 0.6% and 0.2% difference in SUVmean for background regions measured on the uLD 4mm and full dose 2mm corrected PET images, respectively. One subject had two hot lymph nodes with SUVmax measurements of 4.56, 4.49, and 4.56 for the first and 4.35, 4.31, and 4.35 for the second, for the full dose 4mm, uLD 4mm, and full dose 2mm corrected PET images, respectively.
**Conclusion:** To enable CT radiation dose reduction in PET, the use of ultra-low dose CT in this in vivo intra-individual analysis revealed a negligible impact on PET visualization and quantification.
**Limitations:** As repeat CTs in patients would not be ethical, this assessment was limited to a small number of canine subjects. Prospective trials will help validate the results.
**Ethics Committee Approval:** All imaging was performed with approval of the University Institutional Animal Care and Use Committee
**Funding for this study:** 5R01EB022134-04 and Ohio Third Frontier OSDA TECH 09-028 and 13-060 grants
**Author Disclosures:**
Chadwick Lewis Wright: Nothing to disclose
Katherine Binzel: Nothing to disclose
Michael Vinzenz Knopp: Nothing to disclose

### RPS 216-8

**Influence of the low-dose CE-MDCT scanning protocol parameters on the results of pancreatic ductal adenocarcinoma radiomic analysis**
*V. Tikhonova*, I. Gruzdev, E. V. Kondratyev, G. Karmazanovsky; Moscow/RU
*(vdovenkobc28@mail.ru)*

**Purpose:** The key to the CT-diagnosis of pancreatic ductal adenocarcinoma (PDAC) is to achieve the greatest possible contrast difference between intact pancreatic parenchyma and tumor. According to world literature low-dose CT protocol improves the visibility of pancreatic cancer compared to the standard protocol. The purpose of this study was to investigate the dependence of textural features of pancreatic ductal adenocarcinoma on using standard and low-dose CT protocol.
**Methods or Background:** The study included 52 consecutive patients with histologically confirmed PDAC who underwent CE-MDCT using standard (120 kV) or low-dose (100 kV) scanning protocols. We compared radiomics features of the same histological grade PDAC between standard and low-dose protocols using unenhanced, arterial, venous, and delayed-phase of the CT-scan.
**Results or Findings:** We calculated 52 radiomics features in all types of PDAC grade differentiation for unenhanced, arterial, venous, and delayed-phase MDCT scans. We found that less than half of the radiomic features differ statistically significantly when using different protocols in all types of tumor differentiation. Kurtosis was the most sensitive and specific parameter of radiomics for preoperative PDAC grade prediction. The diagnostic accuracy of this feature did not depend on the kV change in the patient's CT protocol.
**Conclusion:** The use of a low-dose CT protocol doesn't affect for PDAC grade prediction.
**Limitations:** The study was single-centred with standardised CE-CT protocols.
**Ethics Committee Approval:** Informed consent was obtained from all patients for using data of their studies in the clinical trial.
The institutional review board approved this prospective study.
**Funding for this study:** The reported study was funded by Russian Foundation for Basic Research according to the research project № 20-315-90092.
**Author Disclosures:**
Evgeny V Kondratyev: Nothing to disclose
Valeriya Tikhonova: Grant Recipient at The reported study was funded by Russian Foundation for Basic Research according to the research project № 20-315-90092.
Grigory G. Karmazanovsky: Nothing to disclose
Ivan Gruzdev: Nothing to disclose

**Live Q&A**

**18:00-19:00** **CHANNEL 2**

### E³ - ECR Master Class

### E³ 1726

### Post-treatment emergencies in oncologic patients

**Moderator**
Digna R. Kool; Amsterdam/NL

### E³ 1726-2
**A. Chest**
Helmut Prosch; Vienna/AT

**Learning Objectives:**
1. To become familiar with the modern approach to malignant chest tumour therapy.
2. To learn how to differentiate clinically important complications.
3. To understand how to look for the early signs of severe and urgent conditions.

**Author Disclosures:**
Helmut Prosch: Advisory Board: Boehringer Ingelheim, MSD, Roche; Speaker: Boehringer Ingelheim, Roche, BMS, MSD, AstraZeneca, Novartis

### E³ 1726-3
**B. Abdomen**
Raffaella Basilico; Chieti/IT
*(rbasilic@unich.it)*

**Learning Objectives:**
1. To learn about different therapies used in abdominal tumours.
2. To become familiar with possible complications.
3. To understand the effectiveness of imaging modalities in the evaluation of emergent complications.

### E³ 1726-4
**C. How can interventional radiologists help in the management of oncological treatment complications?**
Krzysztof K. Pyra; Lublin/PL
*(k.pyra@poczta.fm)*

**Learning Objectives:**
1. To understand the role of interventional radiology in modern tumour therapy and the complications.
2. To become familiar with interventional techniques used in the treatment of complications of different tumour therapies.
3. To learn which tumour therapy complications can be treated with interventional radiology.

**Live Q&A: What is the impact of complication findings on the continued management of oncologic patients?**

**18:00-19:00** **CHANNEL 3**

### Joint Session of the ESR and EIBIR

### ESR/EIBIR

### Funding the future of research in artificial intelligence (AI) and cardiac imaging: reports from the ESR Research Seed Grant projects

**Moderators**
Marc Dewey; Berlin/DE
Gabriel P. Krestin; Rotterdam/NL

### ESR/EIBIR-3
**Reliability of cardiothoracic ratio as an estimate of cardiac enlargement compared to gold standard MRI**
Monika Arzanauskaite; Liverpool/UK

**Learning Objectives:**
1. To learn about the reliability of cardiothoracic ratio as an estimate of cardiac enlargement.
2. To learn about the project progress.

### ESR/EIBIR-5
**MRI-assessed myocardial deformation of the systemic right ventricle in patients with transposition of the great arteries: a cross-sectional study**
Maja Hrabak Paar; Zagreb/HR
*(maja.hrabak.paar@mef.hr)*

**Learning Objectives:**
1. To learn about MRI-assessed myocardial deformation.
2. To learn about the project progress.

**Author Disclosures:**
Maja Hrabak Paar: Grant Recipient: This project has received funding from the Seed Grant funding programme of the European Society of Radiology (ESR) in collaboration with the European Institute for Biomedical Imaging Research (EIBIR)

### ESR/EIBIR-4
**RISK: risk maps using T2 mapping and diffusion MR sequences of the prostate**
Mirjam Lenz; Cologne/DE

**Learning Objectives:**
1. To learn about risk maps using T2 mapping and diffusion MR sequences.
2. To learn about the project progress.

**Author Disclosures:**
Mirjam Lenz: Employee: Philips GmbH Innovative Technologies (K. Sonnabend, R. Shahzad); Research Grant/Support: ESR Seed Grant

### ESR/EIBIR-6
**MR-based biomarkers of tumour hypoxia**
Ieva Kurilova; Amsterdam/NL
*(i.kurilova@nki.nl)*

**Learning Objectives:**
1. To learn about AI-driven MR-based imaging of tumour hypoxia.
2. To learn about the project progress.

**Author Disclosures:**
Ieva Kurilova: Research Grant/Support: ESR/EIBIR Research Seed Grant

### ESR/EIBIR-7
**Value of cardiac magnetic resonance imaging derived temporal myocardial strain pattern for non-invasive diagnosis of myocarditis**
Fabian C. Laqua; Greifswald/DE

**Learning Objectives:**
1. To learn about spatio-temporal cardiac motion patterns.
2. To learn about the project progress.

**Author Disclosures:**
Fabian C. Laqua: Research Grant/Support: European Society of Radiology and European Institute for Biomedical Imaging Research; Share Holder: FCL holds shares of companies developing and/or selling soft- and hardware in the field of artificial intelligence and machine learning

### ESR/EIBIR-8
**Impact of myocardial motion on the relationship between transmitral flow and left ventricular filling rates**
Clemens Reiter; Graz/AT
*(Clemens.Reiter@medunigraz.at)*

**Learning Objectives:**
1. To learn about compressed sensing whole-heart 4D-Flow imaging.
2. To learn about the project progress.

### ESR/EIBIR-9
**CT-based radiomics analysis to predict malignancy in patients with intraductal papillary mucinous neoplasm (IPMN) of the pancreas**
David Tobaly; Clichy/FR

**Learning Objectives:**
1. To learn about T-based radiomics analysis in cancer.
2. To learn about the project progress.

**Author Disclosures:**
David Tobaly: Research Grant/Support: Eibir

### ESR/EIBIR-10

**Towards automated TNM-staging of NSCLC in FDG-PET/CT: evaluation of combined deep learning strategies**

Thomas Weikert; Basle/CH
*(thomasjohannes.weikert@usb.ch)*

**Learning Objectives:**
1. To learn about the integration of an algorithm for automated detection of cancer.
2. To learn about the project progress.

**Live Q&A**

**18:00-19:00** **CHANNEL 4**

### Research Presentation Session: Head and Neck

### RPS 208

### Thyroid imaging and otology/audiology

**Moderator**
Berit Verbist; Leiden/NL

### RPS 208-2

**Application of medical 3D printing in cochlear implantation for precise electrode array placement: a prospective study**

*S. Agarwal*, M. Grover, M. Bagarhatta, A. Prakash; Jaipur/IN
*(srishtiagarwal243@gmail.com)*

**Purpose:** 3D printing consists of a software technology that can generate computational model from a patient's data. We have used this technology for the estimation of an individual's cochlear duct length as a basis for electrode selection.
**Methods or Background:** 3D models of cochlea are created by entering an individual's pre-operative manually segmented cochlear DICOM images from HRCT temporal bone and converting it into STL format. A 3D printer then fabricates the STL files and fuses successive 2D layers of material. The outcome data represents the exact anatomy and a proportionately increased size of the cochlea as that present in the particular individual. All relevant data protection regulations are complied with.
**Results or Findings:** Our study included 42 patients from age 0 to 3 years referred during the year 2016 to 2018, with bilateral profound to severe sensorineural hearing loss with normal cochlear anatomy on HRCT temporal bone and normal MRI brain. 3D cochlea were printed for each patient and personalized length of electrode array were evaluated. The cochlear duct length were compared to that based upon a two-dimensional CT image. The length predicted by 3D prints were significantly better ($p < 0.01$) than that calculated by two dimensional CT images, taking postoperative insertion depth as gold standard.
The patients were followed up for at least 2 years and pre and post operative Meaningful Auditory Integration Scale Score were assessed.
**Conclusion:** 3D model of cochlea helps: Open new dimensions in education in perceiving human cochlear anatomy. Precise intracochlear electrode array placement. Provides a person centric treatment and thus a better prognosis on an individual level.
**Limitations:** The small sample size is a limitation.
**Ethics Committee Approval:** Approved by ethical committee.
**Funding for this study:** Not applicable
**Author Disclosures:**
Aparna Prakash: Nothing to disclose
Meenu Bagarhatta: Nothing to disclose
Mohnish Grover: Nothing to disclose
Srishti Agarwal: Nothing to disclose

### RPS 208-4

**Does shear wave elastography definitively characterize TIRADS 3 and 4 nodules? An efficient resolution to Pandora's box of numbers**

*S. B. Grover*[1], L. Garg[1], S. Patra[1], H. Grover[2], C. Chintamani[1], G. Khanna[1]; [1]New Delhi/IN, [2]New York, NY/US
*(shabnamgrover@yahoo.com)*

**Purpose:** The present study aimed to assess diagnostic performance of previously published shear wave elastography (SWE) values for predicting malignancy in ACR-TIRADS 3 & 4 suspicious thyroid nodules using cytology/histopathology as gold standard. Secondary purpose was to determine our own SWE value cut-offs for characterizing thyroid nodules as benign or malignant.
**Methods or Background:** In this IRB approved study, 60 patients with TIRADS 3 or 4 nodules diagnosed on conventional ultrasound, were further evaluated by Shear Wave Elastography (SWE), using a Siemens S-3000 scanner. In patients with multiple nodules, single most suspicious nodule was interrogated. Lesions were classified into benign or malignant, using previously published SWE cut-off value of 85.2 kPa with higher values indicating malignancy. FNAC/histopathology was considered as gold standard. Statistical analysis was performed for sensitivity, specificity, NPV, PPV and diagnostic accuracy (DA) of the latter SWE values. ROC curve was retrospectively constructed from the SWE values as correlated with histopathology in our cohort and our own cut-off value for SWE was computed.
**Results or Findings:** By ACR criteria 29 nodules were TIRADS 3 and 31 were TIRADS 4. Using established SWE cut-offs, 27 were classified as benign and 33 as malignant. FNAC/histopathology revealed 28 as benign and 32 as malignant. Using published SWE cut-offs, sensitivity, specificity, PPV, NPV and DA was 81.3%, 96.4%, 96.3%, 81.8% and 88.3% respectively. The SWE value determined from our cohort was 74 kPa, with sensitivity, specificity, PPV, NPV and DA of 96.9%, 85.7%, 88.6%, 96.0% and 91.7% respectively.
**Conclusion:** For TIRADS 3 and 4 nodules, SWE has a good diagnostic accuracy and the lower SWE cut-off value obtained in our study differs significantly from previous studies ($p<0.05$), with superior sensitivity, NPV and diagnostic accuracy.
**Limitations:** Small sample size
**Ethics Committee Approval:** Yes
**Funding for this study:** None
**Author Disclosures:**
Chintamani Chintamani: Nothing to disclose
Sayantan Patra: Nothing to disclose
Shanam Bhandari Grover: Nothing to disclose
Geeika Khanna: Nothing to disclose
Hemal Grover: Nothing to disclose
Lovish Garg: Nothing to disclose

### RPS 208-5

**TIRADS, SRE and SWE in Indeterminate thyroid nodule characterization: what is more effective?**

*D. Fresilli*, I. Celletti, G. Polti, E. Polito, G. T. Lucarelli, P. Pacini, O. Guiban, V. Cantisani, C. Catalano; Rome/IT
*(daniele.fresilli@hotmail.it)*

**Purpose:** To assess Strain Ratio (SRE) and Shear Wave Elastography (SWE) accuracy alone and with TIRADS classification, for the risk stratification of indeterminate thyroid nodules.
**Methods or Background:** 128 Patients with 128 indeterminate nodules candidates for thyroidectomy underwent pre-operative staging neck ultrasound and were classified according to K-TIRADS score. Subsequently, semi-quantitative (SRE) and quantitative (SWE expressed in kPa) elastosonographic evaluation was performed and compared with post-thyroidectomy histology. Diagnostic performance of each method, alone and in combination, was assessed with 2x2 contingency tables and ROC curve analysis. In order to maximize the SRE and SWE sensitivity and specificity, their cut-off values were calculated using the Liu test. Bonferroni test was used to evaluate statistically significant differences with a p value <0.05.
**Results or Findings:** Sensitivity, specificity, PPV and NPV were respectively 71.4%, 82.4%, 62.5%, 87.5% for K-TIRADS baseline US, 85.7%, 94.1%, 85.7%, 94.1% for SRE and 57.1%, 79.4%, 53.3%, 81.8% for SWE (kPa expressed). SRE evaluation showed the best diagnostic accuracy compared to the SWE (kPa expressed) ($p <0.05$) and to the K-TIRADS ($p> 0.05$). The association of SRE with conventional ultrasound with K-TIRADS score increased sensitivity (92.9% vs 71.4%) but decreased the specificity than conventional US alone (76.5% vs 82.4%).
**Conclusion:** Strain US-Elastography can be associated with K-TIRADS US examination in the thyroid nodule characterization with indeterminate cytology; in fact, adding the SRE to K-TIRADS assessment increases its sensitivity and negative predictive value. However, further multicenter studies on larger population are warranted.
**Limitations:** Inter-operator variability, small study population and cases when it wasn't possibile the elastosonographic evaluation (big thyroid nodules, presence of large calcifications or large fluid areas).
**Ethics Committee Approval:** All procedures performed were in accordance with the ethical standards of the institutional and/or national research committee.
**Funding for this study:** n/a
**Author Disclosures:**
Daniele Fresilli: Nothing to disclose
Patrizia Pacini: Nothing to disclose
Ilaria Celletti: Nothing to disclose
Olga Guiban: Nothing to disclose
Giorgia Polti: Nothing to disclose
Eleonora Polito: Nothing to disclose

Giuseppe Tiziano Lucarelli: Nothing to disclose
Vito Cantisani: Nothing to disclose
Carlo Catalano: Nothing to disclose

### RPS 208-6

**Non-EPI PROPELLER DWI sequence on MRI to depict cholesteatoma in children with non-operated chronic ear disease**

*K. Lebel*[1], S. Daniel[2], M. Duval[2], C. Saint-Martin[2]; [1]Sherbrooke, QC/CA, [2]Montreal, QC/CA
*(kianalebel@gmail.com)*

**Purpose:** To show usefulness of non-EPI diffusion sequence to depict cholesteatoma in children with otoscopic evaluated high risk of cholesteatoma.
**Methods or Background:** A prospective study evaluated the accuracy of non-EPI diffusion sequences to depict cholesteatoma in pediatric chronic ear disease, in the absence of prior ear surgery. 1.5 Tesla MRIs of consecutive patients were compared to pediatric ENT follow-up exams if MRI was negative for cholesteatoma, and to pathology post-surgery if MRI was positive for cholesteatoma.
**Results or Findings:** 32 pediatric patients (15 males, 17 females), mean age 11.4 years (16 months-17 years) with chronic ear disease underwent non-EPI diffusion sequence (n=40). 2 patients (n=5) were excluded due to susceptibility artifacts from unremovable devices. Unilateral positive restricted diffusion was found on 7/35 examinations and confirmed on pathology to be cholesteatoma in 6. False positive diffusion restriction was due to superimposed acute infection and resolved on repeat MRI after antibiotics. One false negative diffusion sequence was due to the small size and the tegmen location of the cholesteatoma. The 22 patients with negative diffusion sequence (25 MRIs), were followed clinically for a mean of 39 months (3-60 months), with stable audiology tests, further healing and spontaneous closure of the tympanic membrane perforation in 14 patients or further tympanoplasty in six. Two patients were lost during follow-up.
**Conclusion:** In children with high otoscope suspicion of cholesteatoma, non-EPI Diffusion sequence has high sensitivity (85.7%) and very high specificity (96.3 %) to identify cholesteatoma. Hence, we suggest CT indication could be narrowed to patients with positive MRI, prior to cholesteatoma surgery, in order to decrease overall unnecessary radiation.
**Limitations:** This study included only 32 patients.
**Ethics Committee Approval:** Yes
**Funding for this study:** None
**Author Disclosures:**
Kiana Lebel: Nothing to disclose
Sam Daniel: Nothing to disclose
Melanie Duval: Nothing to disclose
Christine Saint-Martin: Nothing to disclose

### RPS 208-7

**DIXON-T2WI magnetic resonance imaging at 3 Tesla outperforms conventional imaging for thyroid eye disease**

*A. Ollitrault*, F. Charbonneau, M-L. Herdan, O. Berges, K. Zuber, L. Giovansili, P. Launay, J. Savatovsky, A. Lecler; Paris/FR
*(alexis.ollitrault@gmail.com)*

**Purpose:** To determine the diagnostic performances of a single DIXON-T2WI sequence compared to a conventional protocol including T1-, T2- and fat-suppressed T2-weighted MRI at 3T when assessing thyroid eye disease (TED).
**Methods or Background:** This prospective single-center study enrolled participants presenting with confirmed TED from April 2015 to October 2019. They underwent an MRI, including a conventional protocol and a DIXON-T2WI sequence. Two neuro-radiologists, blinded to all data, read both datasets independently and randomly. They assessed the presence of extraocular muscle inflammation, enlargement, fatty degeneration or fibrosis as well as the presence of artifacts. A Wilcoxon signed-rank test was used.
**Results or Findings:** 206 participants were enrolled (135/206 [66 %] women, 71/206 [34 %] men, age 52,3 +/- 13,2 years). DIXON-T2WI was significantly more likely to detect at least one inflamed EOM as compared to the conventional set: 248/412 (60 %) vs 228/412 (55 %) eyes, p=0.02. DIXON-T2WI was more sensitive and specific than the conventional set for assessing muscular inflammation: 100% versus 94.7 % and 71.2 % versus 68.5 %, respectively. DIXON-T2WI was significantly less likely to show major or minor artifacts as compared to FS T2WI: 20/412 (5 %) versus 109/412 (27 %) eyes, p<0.001 and 175/412 (42 %) versus 257/412 (62 %) eyes, p<0.001. Confidence was significantly higher with DIXON-T2WI than with the conventional set: 2.35 versus 2.24, p=0.003.
**Conclusion:** DIXON-T2WI showed higher sensitivity and specificity and showed fewer artifacts than a conventional protocol when assessing TED, in addition to higher self-reported confidence.
**Limitations:** The study was achieved in a single tertiary center specialized in ophthalmological imaging, using an optimized 3T MRI device, which might prevent the generalizability of our results. Moreover, no “true” reference standard exists for assessing orbital inflammation in TED.
**Ethics Committee Approval:** Approved
**Funding for this study:** None
**Author Disclosures:**
Frédérique Charbonneau: Nothing to disclose
Alexis Ollitrault: Nothing to disclose
Kévin Zuber: Nothing to disclose
Herdan Marie-Laure Herdan: Nothing to disclose
Pauline Launay: Nothing to disclose
Lama Giovansili: Nothing to disclose
Olivier Berges: Nothing to disclose
Julien Savatovsky: Nothing to disclose
Augustin Lecler: Nothing to disclose

### RPS 208-8

**Percutaneous laser ablation of benign thyroid nodules: a five year follow-up study**

*M. Squarcia*, M. Mora, G. Aranda, I. Halperin, F. Hanzu; Barcelona/ES
*(squarcia@clinic.cat)*

**Purpose:** The aim of the present study was to evaluate the efficacy of the laser ablation (LA) -moving shot technique( LA-MLIEI) during a 5-years follow-up and to identify possible predictors of the final outcome.
**Methods or Background:** Prospective study. Thirty outpatients (23 women/7 men) with benign symptomatic thyroid nodules were assigned to LA-MLIEI, between 2012- 2015. A single LA session was performed under real-time ultrasound (US) guidance using a 1064nm continuous wave laser at 3W.US evaluation was performed after LA-MLIEI at 1 week, 1-, 3-, 6-, and 12 months and thereafter annually till 5 years.
**Results or Findings:** Two distinct groups of patients were observed during follow-up: responders 67% (n: 20) and non-responders 33% (n: 10) ones to LA-MLIEI. The responders group initiated reduction at 1 month, with remission of symptoms and presented a 50% V reduction (□V) at 3 months of treatment, with a maximum response at 24 months, showing stability until the end of the study follow-up. The non-responder group presented a □V of less than 50% at 12 months, showing a tendency to >50% □V at 24 - 36 months, and subsequent regrowth and need for surgical treatment in 30% of this group. Higher total administered energy/V (J/V) and nodule V ≤ 20 ml were correlated with a greater □V. No severe adverse effects were observed.
**Conclusion:** LA with multiple fractional discharges and single fiber is a safe and inexpensive technique with minimal variability that allows rapid reduction after treatment and a stable response at 5 years.
**Limitations:** Relatively small numer of patients.
**Ethics Committee Approval:** The study was approved by the I ethic commitiee of Hospital Clinic Barcelona.
**Funding for this study:** No fund recived.
**Author Disclosures:**
Felicia Hanzu: Nothing to disclose
Gloria Aranda: Nothing to disclose
Irene Halperin: Nothing to disclose
Mireia Mora: Nothing to disclose
Mattia Squarcia: Nothing to disclose

**Live Q&A**

**18:00-19:30** **CHANNEL 5**

### Healthcare Professionals in Focus

### IF 2

### Resilient organisations: the role of the organisation in ensuring employee vitality, engagement, and productivity

**Moderator**
M.G. Myriam Hunink; Rotterdam/NL

### IF 2-2

**Employee silence and employee voice as well-being factors in building resilient organisations**

Anthony Montgomery; Thessaloniki/GR
*(monty5429@hotmail.com)*

**Learning Objectives:**
1. To learn about how employee silence/voice contributes to burnout among healthcare professionals.

2. To better connect clinical practice and healthcare professional well-being in developing preventive interventions.
3. To become familiar with possible interventions at the organisational level that reduces employee silence and enables sharing about work practices.

### IF 2-3
**From chronic stress to vitality and engagement**
Arnold Bakker; Rotterdam/NL
*(bakker@essb.eur.nl)*

**Learning Objectives:**
1. To understand the role of job demands and resources on employee wellbeing.
2. To understand the role of psychological safety and meaning to employee engagement.
3. To shift the focus from the prevention of chronic stress towards positive work experience.
4. To appreciate the need of a systems approach in healthcare organisations.

### IF 2-4
**How to create a resilient organisation**
Diane Sliwka; San Francisco, CA/US
*(diane.sliwka@ucsf.edu)*

**Learning Objectives:**
1. To learn about interventions at the organisational level that promote well-being.
2. To understand what effects can be expected from organisational interventions.
3. To appreciate the complexity of developing a program to foster organisational resilience.
4. To appreciate the increase in complexity due to the pandemic.

### IF 2-5
**Economic considerations**
Stijntje Dijk; Rotterdam/NL
*(s.dijk@erasmusmc.nl)*

**Learning Objectives:**
1. To understand the economic consequences of chronic stress and burnout among healthcare providers.
2. To appreciate the widespread economic effects of burnout.
3. To recognise that prevention can lead to enormous cost-savings.

**Live Q&A: Interventions at the organisational level**

**19:45-20:45 CHANNEL 2**

### E³ - Rising Stars Programme: Basic Sessions
*Organised by the ESR*

### BS 8
### Breast imaging

**Moderator**
Francesco Sardanelli; San Donato Milanese/IT

### BS 8-2
**Inflammation and benign lesions**
Tamar Sella; Jerusalem/IL
*(tamarse@hadassah.org.il)*

**Learning Objectives:**
1. To present current imaging techniques to evaluate breast inflammation and benign lesions.
2. To demonstrate the most important imaging findings.

### BS 8-3
**Lesions of uncertain malignant potential (B3)**
Mihai Lesaru; Bucharest/RO

**Learning Objectives:**
1. To become familiar with the most common imaging findings in B3 lesions.
2. To identify the most appropriate imaging modality.

### BS 8-4
**Malignant lesions**
Christiane K. Kuhl; Aachen/DE (ckuhl@ukaachen.de)

**Learning Objectives:**
1. To present current imaging techniques to evaluate malignant breast lesions.
2. To demonstrate the most important imaging findings.
**Author Disclosures:**
Christiane K. Kuhl: Speaker: Guerbet, Bayer, Bracco

**Live Q&A**

**19:45-20:45 CHANNEL 3**

### Special Focus Session

### SF 19
### Big data use and governance: top tips for radiographers

**Moderators**
Hans-Ulrich Kauczor; Heidelberg/DE
Maryann Hardy; Bradford/UK

### SF 19-3
**What can big data tell us about our clinical services? Leveraging data from the Scottish National PACS**
Jonathan McConnell; Glasgow/UK

**Learning Objectives:**
1. To understand the types of data and data assimilations undertaken.
2. To become familiar with the analysis processes.
3. To consolidate knowledge about the outcomes and impact of big data analysis.
4. To learn how radiographers can get involved with similar initiatives.

### SF 19-4
**How do you manage and protect all that data? Learning from multicentre studies**
Mark F. McEntee; Cork/IE
*(mark.mcentee@ucc.ie)*

**Learning Objectives:**
1. To understand data protection principles, data security, and encryption.
2. To appreciate the processes for data sharing
3. To learn about the communication between systems when a patient opts-out of data sharing.

### SF 19-5
**Artificial intelligence (AI): data-driven decision making in imaging**
Robin Decoster; Brussels/BE

**Learning Objectives:**
1. To learn about the applications of AI for radiography.
2. To become familiar with the data-driven decisions.
3. To consolidate knowledge about ethics ad responsibilities for AI decision-making.
4. To understand the potential augmentation of the radiographer’s role.

**Live Q&A: Should radiographers evolve to be clinical imaging computer/data scientists?**

19:45-20:45 CHANNEL 4

### Transatlantic Course of ESR and RSNA (Radiological Society of North America): Stroke Imaging and Endovascular Treatment: Now and the Future

### TC 328

### Endovascular treatment

**Moderators**
Jean-Pierre Pruvo; Lille/FR
Raman Uberoi; Oxford/UK

### TC 328-3

**A. Endovascular treatment of acute ischaemic stroke: practical pearls**
Ansgar Berlis; Augsburg/DE
*(ansgar.berlis@klinikum-augsburg.de)*

**Learning Objectives:**
1. To learn about endovascular treatment in ischaemic stroke.
2. To understand the different approaches of endovascular treatment.
3. To appreciate the importance of efficient workup and time metrics in angiosuite.

**Author Disclosures:**
Ansgar Berlis: Consultant: Microvention, Stryker, Medtronic; Speaker: Penumbra, Phenox, Medtronic

### TC 328-4

**B. Where to perform and how to organise a thrombectomy**
Jeremy Heit; Stanford, CA/US

**Learning Objectives:**
1. To learn about the number of persons and regulatory recommendations.
2. To understand the optimal organisation in angiosuite and workflow.
3. To appreciate the implications and management for continuity of care.

**Author Disclosures:**
Jeremy Heit: Advisory Board: iSchemaView; Consultant: Medtronic and MicroVention

### TC 328-5

**C. Interactive discussion with illustrative cases of endovascular thrombectomy (Part 1)**
Ansgar Berlis; Augsburg/DE
*(ansgar.berlis@klinikum-augsburg.de)*

**Learning Objectives:**
1. To learn about the tricks and tips of endovascular treatment using illustrative cases.
2. To appreciate the optimal time metrics in angiosuite.
3. To become familiar with the different approaches (stent retriever, ADAPT) and challenging cases (tandem occlusions, distal occlusions).

**Author Disclosures:**
Ansgar Berlis: Consultant: Microvention, Stryker, Medtronic; Speaker: Penumbra, Phenox, Medtronic

### TC 328-6

**C. Interactive discussion with illustrative cases of endovascular thrombectomy (Part 2)**
Gregoire Boulouis; Paris/FR

**Learning Objectives:**
1. To learn about the tricks and tips of endovascular treatment using illustrative cases.
2. To appreciate the optimal time metrics in angiosuite.
3. To become familiar with the different approaches (stent retriever, ADAPT) and challenging cases (tandem occlusions, distal occlusions).

**Author Disclosures:**
Gregoire Boulouis: Advisory Board: Cerenovus 2020

### TC 328-7

**C. Interactive discussion with illustrative cases of endovascular thrombectomy (Part 3)**
Jeremy Heit; Stanford, CA/US

**Learning Objectives:**
1. To learn about the tricks and tips of endovascular treatment using illustrative cases.
2. To appreciate the optimal time metrics in angiosuite.
3. To become familiar with the different approaches (stent retriever, ADAPT) and challenging cases (tandem occlusions, distal occlusions).

**Author Disclosures:**
Jeremy Heit: Advisory Board: iSchemaView; Consultant: Medtronic and MicroVention

**Live Q&A**

19:45-20:45 CHANNEL 5

### New Horizons Session

### NH 1

### Visualising obesity: the new epidemic

**Moderator**
Antonia Arjonilla; Madrid/ES

### NH 1-3

**Brain alterations**
Anna Maria van Opstal; Leiden/NL

**Learning Objectives:**
1. To review the state-of-the-art neuroimaging research for alterations of eating behaviours in obesity and relation to emotions.
2. To describe grey and white matter alterations in obesity.
3. To assess neuroinflammation in obesity by DTI.
4. To try to define how bariatric surgery can reverse obese related effects in the brain.

### NH 1-4

**Thoracic CT in obesity**
Susan J. Copley; London/UK

**Learning Objectives:**
1. To establish a strategy for improving quality imaging of chest plain films and chest CT.
2. To identify chest CT imaging biomarkers with prognostic implications.
3. To show the correlation between obesity and mosaic attenuation, end respiratory air trapping and tracheal shape.
4. To determine reversibility of signs of airway inflammation after bariatric surgery. Lung CT as a marker of clinical improvement.

### NH 1-5

**Cardiovascular imaging**
Rozemarijn Vliegenthart; Groningen/NL

**Learning Objectives:**
1. To achieve technical optimisation of CT cardiovascular studies in obese population.
2. To provide a strategy for optimising contrast injection (total amount and rate) and improve vessel conspicuity.
3. To review the association of obesity with coronary disease, hypertension, atrial fibrilation and heart failure.
4. To be aware of the obesity paradox.

### NH 1-6

**Fatty liver and beyond**
Federica Vernuccio; Palermo/IT
*(federicavernuccio@gmail.com)*

**Learning Objectives:**
1. To learn about liver function capacity in obesity.
2. To learn what NAFLD is and why it is clinically important.
3. To assess the feasibility of non-invasive monitorisation of fat after bariatric surgery.
4. To become familiar with the quantification of liver fat with MRI and proton density fat fraction (PDFF).

**Live Q&A: What do you think should be the role of radiology in the obesity epidemic? Should we lead the pursuit to universal access to adequate diagnostic imaging techniques for the obese population?**

**21:00-21:30** **CHANNEL 2**

### PL 3
### Plenary Lecture

**Moderator**
Michael H. Fuchsjäger; Graz/AT

**PL 3-2**
**Shaping the future of healthcare in the COVID-19 era: partnering with you for intelligent efficiency**
Kieran Murphy; Chalfont St Giles/UK

### Saturday, March 6

08:00-09:00 CHANNEL 2

### EFOMP Workshop: New European Quality Controls for State-of-the-Art Innovations in Radiology and Artificial intelligence: The Role of the Medical Physicist

### EF 1

### European quality controls (QC) in diagnostic and interventional radiology

**Moderator**
Lucie Sukupova; Prague/CZ

### EF 1-2
**Introduction**
Paddy Gilligan; Dublin/IE
*(PGilligan@materprivate.ie)*

**Learning Objectives:**
1. To learn the concept of new QC protocols.
2. To understand the opportunities provided by the standardisation of QC protocols in the optimisation process.
3. To become familiar with how the new protocols can be implemented in a practical manner.

### EF 1-3
**Developing a QC protocol in digital breast tomosynthesis**
Ruben van Engen; Nijmegen/NL
*(r.vanengen@lrcb.nl)*

**Learning Objectives:**
1. To learn why a QC protocol is necessary in breast tomosynthesis.
2. To understand the concepts of the new QC protocol, its challenges and limitations, and its relevance to diagnostic accuracy and safety.
3. To become familiar with how the new protocol can be implemented in a practical manner.

### EF 1-4
**Developing a QC protocol in interventional systems**
Annalisa Trianni; Trento/IT
*(annalisa.trianni@apss.tn.it)*

**Learning Objectives:**
1. To learn why a QC protocol is necessary in modern interventional systems.
2. To understand the concepts of the new QC protocol, its challenges and limitations, and its relevance to diagnostic accuracy and safety.
3. To become familiar with how the new protocols can be implemented in a practical manner.

### EF 1-5
**Developing a QC protocol in PET/CT and PET/MRI**
Roberta Matheoud; Novara/IT

**Learning Objectives:**
1. To learn why a QC protocol is necessary in modern hybrid systems.
2. To understand the concepts of the new QC protocol, its challenges and limitations, and its relevance to diagnostic accuracy and safety.
3. To become familiar with how the new protocols can be implemented in a practical manner.

**Live Q&A**

08:00-09:00 CHANNEL 3

### Professional Challenges Session

### PC 1

### Subspecialisation vs despecialisation

**Moderator**
Adrian Brady; Cork/IE

### PC 1-2
**Current clashes in interventional radiology**
Laura Crocetti; Pisa/IT
*(laura.crocetti@med.unipi.it)*

**Learning Objectives:**
1. To learn about the current landscape of the organisation of interventional radiology services in different European countries.
2. To discuss the need of further subspecialisation within interventional radiology.
3. To understand how education is organised and could be improved to train interventional radiologists.

### PC 1-3
**Role of new professional profiles: the role of a consultant radiographer reporting plain-film radiographs within a clinical imaging team**
Robert Milner; Rotherham/UK
*(Robert.Milner1@nhs.net)*

**Learning Objectives:**
1. To learn about how a plain-film radiographer reporting service can be safely developed.
2. To understand how a hospital can think outside the box and embrace new ways of working with no additional risk to patients or staff.
3. To appreciate how radiographers and radiologists can work together to free up the radiologist’s time for other activities and to provide a plain-film radiographer-led service that is amongst the best and most responsive in the UK.

### PC 1-4
**What do the "customers" referring physicians or patients want and how to provide it?**
Caroline Justich; Vienna/AT
*(cjustich@me.com)*

**Learning Objectives:**
1. To learn about the patients’ questions, behaviour, and needs when they come into a radiology department.
2. To understand what is necessary to support patients and doctors, what information is expected, that patients are no longer silent consumers and are now active decision makers, and to understand the needs of the referring doctors.
3. To appreciate that patients want to be supported but also want to cooperate with their doctors to identify symptoms and needs as fast as possible, and to receive the right diagnose and medication as soon as possible.
4. To become familiar with digital solutions and tools to help patients and colleagues with the right information and data.

### PC 1-5
**Are "imaging specialists" in other disciplines a threat to radiology?**
Hans-Ulrich Kauczor; Heidelberg/DE

**Learning Objectives:**
1. To consolidate knowledge about the value and uniqueness of general and subspecialised radiology.
2. To appreciate the added value of multidisciplinary teams with shared expertise.
3. To learn about the opportunities of radiologists in future workflow scenarios.
**Author Disclosures:**
Hans-Ulrich Kauczor: Research Grant/Support: Siemens, Philips, Bayer; Speaker: Philips, Astra Zeneca, MSD; Boehringer Ingelheim

**Live Q&A: How to implement subspecialisation into a department: perspectives for large and small departments**

08:00-09:00 CHANNEL 5

### Research Presentation Session: GI Tract

### RPS 101b

### The cutting edge of GI imaging

**Moderator**
Monique Maas; Amsterdam/NL

### RPS 101b-2

**Prognostic significance of MRI-detected mesorectal fat thickness in rectal cancer: a risk factor for distance metastasis**

*P. Tripathi*, Z. Li, Y. Shen, X. Hu, D. Hu; Wuhan/CN

**Purpose:** We primarily aimed to investigate relationship between MRI-detected mesorectal fat and BMI. Furthermore, investigate the impact of MRI-detected mesorectal fat distant metastasis in patients with rectal cancer.
**Methods or Background:** Post-operative T3 rectal cancer patients without previous treatment from July 2016 to December 2019 were enrolled for this study. Two blinded radiologists evaluated MRI-detected extramural vascular invasion, MRI-detected depth of tumor extension, MRI-detected mesorectal fat thickness, area within mesorectum, mesorectum thickness and fat buffer thickness. The results along with other clinical characteristics were then correlated with distant metastases to determine the risk factors. Cox regression analysis was used to estimate independent risk factors for disease-free survival (DFS).
**Results or Findings:** In 77 patients (58.9±11.69 years), 33 patients were confirmed to be mrEMVI-positive. The mesorectal thickness, area within mesorectum, area inside rectum, fat buffer area and BMI were 4.79 ± 2.86mm, 2708 ± 513.29mm2, 951.88 ± 288.42mm2, 1756.42 ± 448.56mm2 and 22.43 ± 3.70kg/m2 respectively. Area within mesorectum (p=0.02), area inside rectum (p=0.05) and fat buffer area (p=0.02) showed significant correlation with BMI. Multivariate analysis revealed only MRI-detected mesorectal fat thickness to be significantly associated with distant metastasis. A 3-year disease-free survival rates were 97.2% and 9.5% (P<0.001) in patients with mesorectal fat thickness >3.32mm and <3.32mm groups.
**Conclusion:** Area within mesorectum, area inside rectum and fat buffer area has significant correlation with BMI. MRI-detected mesorectal fat thickness is an independent risk factor for distant metastasis in rectal cancer. 3-year disease-free survival rates were lower in the patients with thin mesorectal fat.
**Limitations:** 1. Small sample size.
2. Single centre sample.
**Ethics Committee Approval:** This study was approved by the appropriate institutional review board.
**Funding for this study:** National Natural Science Foundation of China under Grant number 81571642, 81701657, 81771801 and 81801695.
**Author Disclosures:**
Xuemei Hu: Nothing to disclose
Pratik Tripathi: Nothing to disclose
Zhen Li: Nothing to disclose
Yaqi Shen: Nothing to disclose
Daoyu Hu: Nothing to disclose

### RPS 101b-3

**Crohn's disease: the correlation between US and MR-enterography - our experience**

*J. Miranda Bautista*[1], J. Llorente Peris[1], R. E. Buongermini[1], S. Lanzarote Vargas[1], P. Otermin Barrera[1], E. de Miguel Campos[1], P. Menéndez Fernández-Miranda[2]; [1]Madrid/ES, [2]Santander/ES
*(mirandabautistajuan@gmail.com)*

**Purpose:** The objectives of this study were to outline the influence of intestinal ultrasonography in the initial assessment and follow-up of patients with Crohn´s disease and to compare the findings statistically with MR enterography features (gold standard), in view of a potential subsequent implementation in the algorithms of these patients in our hospital.
**Methods or Background:** Between 2015 and 2019, 100 cases with suspected or confirmed diagnosis of Crohn's disease were treated in our Institute. Most of them underwent an MR enterography and posterior ultrasonography over a period of 15 days. In this prospective study, morphological and doppler findings, together with semiquantitative parameters in contrast-enhanced ultrasonography were assessed to quantify the extent of the affected segments, the degree of inflammatory activity and diagnosis of extraluminal complications.
**Results or Findings:** The concordance in disease activity and extension had Kappa values of 0.711 and 0.854, respectively. The mean mural thickness with MRI was 5.8, and with echo 5.2 mm. To diagnose mucosal ulcers, fistulae, abscess, and stenosis it showed a Kappa value of 0.66, 0.807, 1 and 0.647, respectively. Grade II/III color Doppler signal showed a sensitivity of 67% and a specificity of 98% in the diagnosis of disease activity (MaRIA Index >7). In contrast-enhanced US, patients with a MaRIA index <7 showed a mean AUC of 116.17 compared to 503.79 if MaRIA index > 7.
**Conclusion:** In patients with Crohn's disease, bowel US is an imaging technique whose diagnostic possibilities are in some ways comparable to those of MR. Its excellent accessibility, lower price, and better tolerance forces us to consider it as a profitable alternative in the assessment of inflammatory activity in this long-term disease.
**Limitations:** No limitations
**Ethics Committee Approval:** Scientific study approved
**Funding for this study:** Not required
**Author Disclosures:**
Rafael Emilio Buongermini: Nothing to disclose
Pablo Menéndez Fernández-Miranda: Nothing to disclose
Juan Miranda Bautista: Nothing to disclose
Silvia Lanzarote Vargas: Nothing to disclose
Paula Otermin Barrera: Nothing to disclose
Javier Llorente Peris: Nothing to disclose
Enrique de Miguel Campos: Nothing to disclose

### RPS 101b-4

**A proposal for a new prognostic grading system in achalasia using dynamic barium swallow: the FBF score**

*G. Fontanella*[1], S. Borrelli[2], A. Festa[1], S. De Lucia[1], C. A. T. Manganiello[1]; [1]Benevento/IT, [2]Atripalda/IT
*(giovanni.fontanella@hotmail.com)*

**Purpose:** To develop and evaluate a new radiographic scoring system, the FBF score, allowing for more accurate, clinically oriented prognostic grading in achalasia.
**Methods or Background:** We developed our FBF Scoring System by profiling the three achalasia subtypes described in the clinical/manometric Chicago Classification (I, Hypotonic; II, Panpressurizing; III, Spastic) into three equivalent radiographic grades, using five morphodynamical findings (bird-beak sign, dilatation, hypotonia, stasis, spasm) as scoring criteria.Between June 2017 and June 2020, 141 patients (mean age 59.2, 60.2% male) already diagnosed with achalasia at manometry, were evaluated with Dynamic Barium Swallow, reviewed in blind by two radiologists with GI experience, then graded using our FBF Score. Radiographic grading accuracy and sensitivity were evaluated using manometry data as gold standard. The five scoring criteria were each evaluated for sensitivity and specificity with regard to each radiological grade.
**Results or Findings:** Our FBF score allowed us to predict the clinical/manometric subtype with high accuracy, with sensitivity values reaching 91.3% for Subtype III, 95.4% for Subtype II and 98.03% for Subtype I, the latter being correctly diagnosed in 50/51 patients with FBFs ≥8 (presence of at least 4 combined radiographic findings). Spasm showed high specificity in Subtype III (95.65%), while stasis resulted in high sensitivity for Subtype II (93.18%).
**Conclusion:** Radiographic grading of achalasia using FBF score has shown to be highly sensible and accurate when compared to the manometry results, especially in Subtypes II and III. FBF score implementation might allow more precise morphodynamical analysis, structured reporting and better overall diagnostic support to manometry, patient stratification and therapeutic choices.
**Limitations:** This study is monocentric, patient number is limited (141).
**Ethics Committee Approval:** Our study received ethical committee approval, each patient signed informed consent before examinations
**Funding for this study:** No funding was received for this work.
**Author Disclosures:**
Carmine Augusto Tommaso Manganiello: Nothing to disclose
Simona Borrelli: Nothing to disclose
Giovanni Fontanella: Nothing to disclose
Andrea Festa: Nothing to disclose
Silvio De Lucia: Nothing to disclose

### RPS 101b-5

**The heterogeneity of skewness in T2w-MR images of locally advanced rectal cancer relates with a response to neo-adjuvant chemoradiotherapy**

A. Bevilacqua, F. Coppola, *M. Mottola*, S. Lo Monaco, A. Cattabriga, A. Guido, F. Di Fabio, L. Ricciardiello, R. Golfieri; Bologna/IT
*(margherita.mottola@unibo.it)*

**Purpose:** To investigate how the heterogeneity of skewness (HoS) in locally advanced rectal cancer (LARC) imaged with T2 weighted (T2w) MRI before neo-adjuvant chemoradiotherapy (nCRT) can allow predicting responder patients based on the Tumour Regression Grade (TRG).
**Methods or Background:** Forty patients with LARC diagnosis undergo 1.5T-MRI before nCRT. Regions of Interest (ROIs) are manually segmented in all slices of the tumour's site on the oblique axial T2w sequences. Based on the TRG assigned at the histopathological inspection of the surgical resection samples, the dataset is split into 15 responders (Rs) and 25 non-responders

Saturday

(NRs), having TRG=[0,1] and TRG=[2,3], respectively. HoS in LARC is computed on T2w values stemming from the measures of local asymmetry of each voxel. One-tail Wilcoxon rank-sum test ($p<0.05$) is considered to assess R and NR separability. Median values and interquartile ranges (IQRs) are considered to evaluate how the pre-therapy heterogeneity varies between Rs and NRs.
**Results or Findings:** HoS of Rs patients has a lower variance (IQR=0.15) than HoS of NRs ones (IQR=0.46), although median HoS has an opposite behaviour, being higher in Rs groups (4.08) than in NRs one (3.68). This yields Rs and NRs groups to be clearly separated (p~10-5), with non-overlapping IQRs.
**Conclusion:** In pre-therapy LARC imaged with T2w MRI, the higher the HoS, the better the response to nCRT. In addition, while the Rs group shows a homogeneous behaviour, the group of NRs is spread over a wider heterogeneity range, probably due to a higher variability of tumour characteristics.
**Limitations:** This study includes a limited number of patients, but the clear separation between R and NR highlights the promising role of heterogeneity, to be confirmed increasing the number of patients considered.
**Ethics Committee Approval:** n° 842/2020/Oss/AOUBo
**Funding for this study:** not Applicable
**Author Disclosures:**
Alessandra Guido: Nothing to disclose
Silvia Lo Monaco: Nothing to disclose
Rita Golfieri: Nothing to disclose
Arrigo Cattabriga: Nothing to disclose
Francesca Di Fabio: Nothing to disclose
Margherita Mottola: Nothing to disclose
Luigi Ricciardiello: Nothing to disclose
Francesca Coppola: Nothing to disclose
Alessandro Bevilacqua: Nothing to disclose

### RPS 101b-6

**Single versus split dose of Iohexol for fecal tagging in CT colonography**
*A. Meshcheryakov*, O. Pugacheva, N. Gurova, S. Morozov; Moscow/RU
*(aim.radiologist@gmail.com)*

**Purpose:** The purpose of this study is to compare the quality of fecal tagging and patients' tolerance for CT colonography (CTC) with nonionic iohexol iodinated oral contrast agents in single versus split dose.
**Methods or Background:** 60 adults with no symptoms were enrolled in this study (mean age, 72,3 years; 23 men). All patients were randomly assigned (1:1 ratio) to receive a single dose of 50 mL of iohexol 350 for fecal tagging or a split dose (25 mL + 25 mL). All other preparation components remained constant. Endpoints were the quality of fecal tagging and tolerance to the assigned bowel preparation regimen. Fluid attenuation (HU values) and homogeneity (SD values) analysis of residual colonic fluid was performed. Additionally was scored a volume of residual colonic fluid and solid stool using a previously validated 4-point scale. A Wilcoxon's matched pairs test, and the Mann-Whitney test was used for comparison of the cohorts.
**Results or Findings:** The mean fluid attenuation was higher with single-dose iohexol (849 ± 298 HU) compared with split-dose (714 ± 335 HU) ($p = 0.02$). The homogeneity of fecal tagging was 92 and 85 HU for groups 1 and 2, respectively ($p = 0.12$). The mean total colonic fluid score was 2.3, with a single dose and 2.5 with a split dose ($p = 0.53$), and the mean total colonic stool score was 1.5 and 1.7 respectively ($p = 0.72$). Tolerance was significantly better in the single-dose group (p=0,03).
**Conclusion:** Single dose iohexol 350 improves fecal tagging quality compared with spit dose with higher residual fluid attenuation and elevates patient acceptability.
**Limitations:** Our study's main limitation is the relatively small patient cohort.
**Ethics Committee Approval:** The study was approved by the local ethics committee.
**Funding for this study:** No funding for this study.
**Author Disclosures:**
Sergey Morozov: Nothing to disclose
Andrey Meshcheryakov: Nothing to disclose
Olga Pugacheva: Nothing to disclose
Nadezda Gurova: Nothing to disclose

### RPS 101b-7

**Rectal cancer with complete endoscopic response after neoadjuvant therapy: what is the meaning of a positive MRI?**
*M. J. Gollub*, J. Das, D. D. B. Bates, J. L. Fuqua III, J. S. Golia, S. Javed-Tayyab, V. Paroder, I. Petkovska, J. Garcia-Aguilar; New York, NY/US
*(gollubm@mskcc.org)*

**Purpose:** To determine the short-term outcomes of discordant tumor assessments between DWI-MRI and endoscopy in patients with treated rectal cancer when tumor-bed diffusion restriction is present ("+DWI").
**Methods or Background:** In this HIPPA compliant, IRB-approved retrospective study, rectal MRI and endoscopic reports were reviewed for patients with locally advanced primary rectal adenocarcinoma (LARC) treated with chemoradiotherapy or total neoadjuvant therapy and imaged between January 2016 and December 2019. Eligible patients had a +DWI and endoscopy within 2 weeks of each other. True positive MRI were those with tumor on endoscopy and/or biopsy (TPa) or in whom endoscopy was negative for tumor, but subsequent 3-month follow up endoscopy and DWI were both positive (TPb). The positive predictive value of DWI-MRI was calculated on a per-scan and per patient basis. DWI negative MRI exams were not explored in this study.
**Results or Findings:** 397 patients with nonmetastatic primary LARC were analyzed. After exclusions, 90 patients had 98 follow-up rectal MRI studies with +DWI. 76 patients underwent 80 MRI scans and had concordant findings at endoscopy (TPa). 17 patients underwent 18 MRI scans and had discordant findings at endoscopy (FP); among these, 4 scans in 4 patients were initially false positive (FP) but follow up MRI remained +DWI and the endoscopy turned concordantly positive (TPb). PPV was 0.86 per scan and per patient. In 4/18 (22%) scans and 4/17 (23%) patients with discordances, MRI detected tumor regrowth before endoscopy.
**Conclusion:** Although most +DWI exams discordant with endoscopy are false positive, 22% will reveal that DWI-MRI detects tumor recurrence before endoscopy.
**Limitations:** Retrospective
**Ethics Committee Approval:** Memorial Sloan Kettering Cancer Center IRB
**Funding for this study:** None
**Author Disclosures:**
Vicky Paroder: Nothing to disclose
Sidra Javed-Tayyab: Nothing to disclose
Julio Garcia-Aguilar: Nothing to disclose
Marc Jeffrey Gollub: Nothing to disclose
David Dawson Bartlett Bates: Nothing to disclose
J. Louis Fuqua III: Nothing to disclose
Iva Petkovska: Nothing to disclose
Jennifer S. Golia: Nothing to disclose
Jeeban Das: Nothing to disclose

**Live Q&A**

**09:15-10:15 CHANNEL 2**

### Refresher Course: Vascular

### RC 415

### Vasculitis and inflammation in arteries

**Moderator**
Elias N. Brountzos; Athens/GR

### RC 415-2

**A. Vasculitis and inflammation in peripheral arteries**
Thorsten Bley; Würzburg/DE
*(bley_t@ukw.de)*

**Learning Objectives:**
1. To learn about classification and aetiology.
2. To understand radiological imaging techniques and differential diagnosis.
3. To review the treatment options.

**Author Disclosures:**
Thorsten Bley: Advisory Board: Roche / Chugai / Novartis; Grant Recipient: Deutsche Forschungsgemeinschaft, DFG; Research Grant/Support: Siemens; Speaker: Roche / Chugai / Novartis

### RC 415-3

**B. Vasculitis and inflammation in neurocranial arteries**
Hans Kortman; Tilburg/NL

**Learning Objectives:**
1. To learn about radiological imaging techniques.
2. To understand aetiology and differential diagnosis.
3. To become familiar with the treatment options.

### RC 415-4

**C. Nuclear imaging of vasculitis and inflammation in arteries**
James Howard; Manchester/UK
*(james.howard@mft.nhs.uk)*

**Learning Objectives:**
1. To discuss the nuclear medicine options of inflammatory arterial disease.
2. To provide optimal nuclear medicine strategies for subacute and chronic inflammatory disease of the arteries.
3. To become familiar with the signs of inflammatory vs non-inflammatory disease of the arteries.

**Live Q&A**

**09:15-10:15 CHANNEL 4**

### Special Focus Session

### SF 17

### Male erectile dysfunction (ED)

**Moderator**
Boris Brkljačić; Zagreb/HR

### SF 17-2

**What the urologists expect from the radiologist?**
Nicola Pavan; Trieste/IT
*(nicpavan@gmail.com)*

**Learning Objectives:**
1. To understand the clinical correlations between imaging and erectile dysfunction.
2. To understand the clinical indication for imaging studies.
3. To analyse how imaging is influencing the clinical management of a patient with erectile dysfunction.

### SF 17-3

**Penile Doppler**
Paul S. Sidhu; London/UK
*(paulsidhu@btinternet.com)*

**Learning Objectives:**
1. To understand and learn to perform a dynamic penile Doppler US.
2. To analyse the spectral Doppler waveforms in erectile dysfunction.
3. To learn about the usefulness of a Doppler US in priapism.
4. To obtain an overview of other abnormalities of the penis seen on US.

**Author Disclosures:**
Paul S. Sidhu: Advisory Board: Samsung Inc; Consultant: ITREAS ; Speaker: Bracco, Siemens

### SF 17-4

**Role of MRI**
Laurence Rocher; Le Kremlin-Bicêtre/FR

**Learning Objectives:**
1. To assess penile MRI adequate protocols regarding the suspected pathology.
2. To become familiar with normal MRI findings in flaccidity and erectile status.
3. To identify pathological findings such as Peyronie's disease, fibrosis, or rare tumours involved in erectile dysfunction.

### SF 17-5

**Interventional radiology in diagnosis and management of ED**
Charles Tapping; Oxford/UK
**Author Disclosures:**
Charles Tapping: Investigator: Merit Medical; Speaker: Boston Scientific

**Live Q&A: What is the role of imaging and image-guided interventions in erectile dysfunction?**

**09:15-10:15 CHANNEL 5**

### Research Presentation Session: Artificial Intelligence & Machine Learning

### RPS 105

### Artificial intelligence (AI) and COVID-19

**Moderator**
Erik R. Ranschaert; Tilburg/NL

### RPS 105-2

**Fully automatic COVID-19 lung and pneumonia lesion segmentation from CT images**
*I. Shiri*[1], H. Arabi[1], Y. Salimi[1], A. Sanaat[1], A. Akhavanalaf[1], G. Hajianfar[2], D. Askari[2], K. R. Kalantari[2], H. Zaidi[1]; [1]Geneva/CH, [2]Tehran/IR
*(isaac.sh92@gmail.com)*

**Purpose:** We present a deep learning (DL)-based automated whole lung and COVID-19 pneumonia infectious lesions (COLI-Net) detection and segmentation from chest CT images
**Methods or Background:** We prepared 2178 ( 347’259, 2D slices) and 170 (16’890, 2D slices) volumetric CT images along with their corresponding manual segmentation of lungs and lesions, respectively, in the framework of a multi-center/multi-scanner study. All images were cropped, resized and the intensity values clipped and normalized. A residual network (ResNet) with non-square Dice loss function built upon TensorFlow was employed. The accuracy of lung and COVID-19 lesions segmentation was evaluated on an external RT-PCR positive COVID-19 dataset (7’333, 2D slices) collected at five different centers. To evaluate the segmentation performance, we calculated different quantitative metrics, including radiomic features.
**Results or Findings:** The mean Dice coefficients were 0.98±0.011 (95% CI, 0.98-0.99) and 0.91±0.038 (95% CI, 0.90-0.91) for lung and lesions segmentation, respectively. The mean relative Hounsfield unit differences were 0.03±0.84% (95% CI, -0.12 – 0.18) and -0.18±3.4% (95% CI, -0.8 - 0.44) for the lung and lesions, respectively. The relative volume difference for lung and lesions were 0.38±1.2% (95% CI, 0.16-0.59) and 0.81±6.6% (95% CI, -0.39-2), respectively. Most radiomic features had a mean relative error less than 5% with the highest mean relative error achieved for the lung for the Range first-order feature (-6.95%) and least axis length shape feature (8.68%) for lesions.
**Conclusion:** We developed an automated deep learning-guided three-dimensional whole lung and infected regions segmentation in COVID-19 patients in order to propose a fast, consistent, robust and human error immune framework for lung and pneumonia lesion detection and quantification.
**Limitations:** Not applicable
**Ethics Committee Approval:** This retrospective study was conducted with institutional review board approval.
**Funding for this study:** Swiss National Science Foundation under grant SNRF 320030_176052.
**Author Disclosures:**
Dariush Askari: Nothing to disclose
Amirhossein Sanaat: Nothing to disclose
Hossein Arabi: Nothing to disclose
Ghasem Hajianfar: Nothing to disclose
Habib Zaidi: Nothing to disclose
Azadeh Akhavanalaf: Nothing to disclose
Yazdan Salimi: Nothing to disclose
Isaac Shiri: Nothing to disclose Nothing to disclose
Kiara Rezaei Kalantari: Nothing to disclose

### RPS 105-3

**Diagnostic performance of a deep learning model ensemble for the detection of COVID-19 pneumonia on chest radiographs**
*Z. T. J. Sim*, C. H. Tan, Y. Ting, W. Huang, X. Xu, Y. Feng, X. Lei, Y. Cui, S. T. Wong; Singapore/SG
*(jordansim92@gmail.com)*

**Purpose:** During the Coronavirus disease-19 (COVID-19) pandemic, chest radiographs (CXRs) are the mainstay of initial radiological investigation in management. Artificial Intelligence (AI) algorithms for COVID-19 pneumonia detection may perform well but can degrade significantly when deployed in the real-world setting. This study is the first to test a trained COVID-19 pneumonia CXR detection model in real world setting as a proof-of-value.
**Methods or Background:** An ensemble of seven models (RadiLogic) was developed from 112,120 CXRs from the NIH Chest-14 open-source dataset, and fine-tuned using CXRs from patients suspected of COVID-19 pneumonia. Two senior radiologists with more than 10 years of clinical experience provided the reference standard. RadiLogic was thereafter integrated into the daily workflow, prioritising CXRs for expedited reporting. Area under the receiver operating characteristic curve (AUC), F1 score, sensitivity and specificity were calculated to characterise diagnostic performance.
**Results or Findings:** A total of 9,381 unique CXRs were included in the datasets. For the deployment dataset, RadiLogic achieved an AUC of 0.95 (95% Confidence Interval [CI]: 0.92, 0.96) corresponding to a specificity of 97% (95% CI: 0.97, 0.98) and sensitivity of 79% (95% CI: 0.72, 0.84). No significant degradation of diagnostic performance was encountered as a result of RadiLogic deployment, while turn-around-time for abnormal CXR reports were reduced by 20%.
**Conclusion:** In real-world clinical deployment, our ensemble of seven deep learning models expedites reporting of COVID-19 pneumonia while preserving diagnostic performance.
**Limitations:** n/a
**Ethics Committee Approval:** n/a
**Funding for this study:** n/a
**Author Disclosures:**
Yingnan Cui: Nothing to disclose
Xiaofeng Lei: Nothing to disclose
Yonghan Ting: Nothing to disclose
Cher Heng Tan: Nothing to disclose
Yangqin Feng: Nothing to disclose
Weimin Huang: Nothing to disclose
Sum Thai Wong: Nothing to disclose
Xinxing Xu: Nothing to disclose
Zheng Ting Jordan Sim: Nothing to disclose

### RPS 105-4

**Development of an artificial intelligence method to detect COVID-19 pneumonia in computed tomography images based on multi-planar reconstruction and convolutional neural networks**

H. M. Karakas[1], *G. Yildirim*[1], Y. A. Ozkaya[2], E. Sener[2]; [1]Istanbul/TR, [2]Ankara/TR
*(dr.gulsah.yildirim@gmail.com)*

**Purpose:** This study was aimed to construct an artificial intelligence system based on convolutional neural network algorithms to detect COVID-19 pneumonia on high resolution CT images. This system was built to assist radiologists in rapid and confident diagnosis of infected cases during high volume conditions of pandemic, and also to quantify disease-related lesion burden in multifactorial prognostic analyses.
**Methods or Background:** In this retrospective and single-center study, a CNN model was developed to extract visual features from volumetric chest CT exams for the detection of COVID-19. Dataset was acquired between March 2020 and July 2020. CT images of 573 RT-PCR proven COVID-19 patients were extracted from local PACS system. Images were randomly annotated by three experienced radiologists for known lesions of COVID-19 pneumonia (i.e. ground glass opacity and consolidation). Consequently, lesion contours in 407 studies were manually selected. Anisotropic Image volumes were first converted to isotropic volumes and axial, sagittal, and coronal images and labels were generated using Multi Planar Reconstruction techniques. These perpendicular data sets were trained individually using U-Net architecture. Obtained models are applied consecutively to a volumetric CT scan and suspicious lesions are extracted by intersecting the findings from three different planes.
**Results or Findings:** The multiplanar models achieved an accuracy of 92.42% on average. The specificity is found to increase from 78.68% to 88.24% when multiplanar findings were merged compared to using outputs from axial plane only.
**Conclusion:** The artificial intelligence model can accurately detect typical COVID-19 pneumonia lesions. Analyzing CT datasets from all three isoviews is found to increase specificity compared to using axial view alone.
**Limitations:** The method is validated for typical findings of COVID-19; indeterminate atypical findings are beyond the scope of this study.
**Ethics Committee Approval:** Aproval no: 2020-11-10T15_07_21.
**Funding for this study:** None
**Author Disclosures:**
Emre Sener: Nothing to disclose
Gulsah Yildirim: Nothing to disclose
Hakki Muammer Karakaş: Nothing to disclose
Yasar Alper Ozkaya: CEO at Simplex Information Technologies Co.

### RPS 105-5

**Artificial intelligence services' impact on radiologist's performance in the context of the COVID-19 pandemic**

*T. Logunova*, A. E. Andreychenko, V. Klyashtorny, K. M. Arzamasov, A. Vladzymyrskyy, S. Morozov; Moscow/RU
*(logunova.tatiana@gmail.com)*

**Purpose:** COVID-19 pandemic has led to an essential increase of chest CT examinations that necessitates to urgently report a large amount of studies in limited time with limited human resources. The purpose of the study is to determine impact of the introduction of AI-services analyzing chest CTs for COVID-19 related findings on the radiologists workflow and performance.
**Methods or Background:** Comparative analysis of the time intervals between loading a CT study into RIS and validation of the report by a radiologist in RIS was performed; the assessment of the accuracy was estimated by comparing the number of clinically significant discrepancies between radiologist and expert radiologist during clinical audit, expressed as a percentage of the total number of audited studies. In total, we analyzed the relevant parameters in 128,350 chest CT studies (36,358 studies were timely analyzed by AI) acquired and reported in the period from April 30 to August 31, 2020 in Moscow in total by 578 radiologists.
**Results or Findings:** The results of multivariate regression analysis revealed that group of radiologists periodically receiving timely results of an AI-service processed study on 7 minutes faster than group without AI ($p<0.001$). The number of discrepancies between radiologist and expert radiologist before and after the introduction of the AI-service did not reveal statistically significant differences ($p> 0.05$).
**Conclusion:** Introduction of the AI-services into the radiologists workflow to analyze chest CT during the COVID-19 pandemic increases the productivity of the radiologist, reducing the report turn-around-time, without significantly affecting the accuracy of interpretation.
**Limitations:** The reported results and conclusions are preliminary as the study data collection is still on-going.
**Ethics Committee Approval:** This study is based upon the results of the registered study (https://clinicaltrials.gov/ct2/show/NCT04489992).
**Funding for this study:** Internal.
**Author Disclosures:**
Sergey Morozov: Nothing to disclose
Anna Evgenevna Andreychenko: Nothing to disclose
Tatiana Logunova: Nothing to disclose
Anton Vladzymyrskyy: Nothing to disclose
Vladislav Klyashtorny: Nothing to disclose
Kirill Mihajlovich Arzamasov: Nothing to disclose

### RPS 105-6

**A deep learning approach to automatically segment CT manifestations of COVID-19 and non-COVID-19 pneumonia**

F. Meng[1], *R. Shahzad*[2], H. Liu[3], F. Thiele[2], H. Zhang[1], M. Perkuhn[2]; [1]Changchun/CN, [2]Cologne/DE, [3]Wuhan/CN
*(rahil.shahzad@uk-koeln.de)*

**Purpose:** To develop a deep learning based automatic framework to segment COVID-19 and non-COVID-19 manifestation in non-contrast computer tomography (NCCT) of the chest, and compare the performance with reference annotations from radiologists.
**Methods or Background:** A total of 493 CT scans of 477 patients were retrospectively included. 240 consecutive patients with positive RT-PCR for COVID-19 were collected from Wuhan No 1 Hospital. 237 community acquired pneumonia (CAP) patients were selected from the First Hospital of Jilin University between January 2019 and April 2020. As reference standard, CT manifestations of pneumonia were manually annotated by a radiologist. A 3D U-net deep learning model was trained and evaluated on 394 NCCT of 380 patients using 5-fold-cross-validation. Model performance was tested on an independent set of 99 NCCT of 97 patients. The segmentation accuracy of the model against the reference standard was evaluated using dice similarity coefficient (DSC) and volume correlation (r).
**Results or Findings:** The training set consisted of 198 COVID-19 and 196 CAP scans. Average model performance over the 5-folds was DSC=0.75 on training and DSC=0.63 on validation data. On the test set with 50 COVID-19 and 49 CAP, overall DSC was 0.60 (range 0-0.91) and volume correlation r=0.96. For the COVID-19 subset, DSC was 0.70 (range 0.03-0.91) and r=0.95. For CAP cases, DSC was 0.49 (range 0.06-0.86) and r=0.97.
**Conclusion:** The deep learning model can accurately detect and segment the CT manifestations of COVID-19 and CAP patients as compared to manual reference standard. Such a volumetric quantification is helpful for monitoring infection status.
**Limitations:** n/a
**Ethics Committee Approval:** The local ethics committee approved this retrospective study
**Funding for this study:** n/a
**Author Disclosures:**
Rahil Shahzad: Employee at Philips Healthcare
Haifeng Liu: Nothing to disclose
Huimao Zhang: Nothing to disclose
Fanyang Meng: Nothing to disclose
Frank Thiele: Employee at Philips Healthcare
Michael Perkuhn: Employee at Philips Healthcare

**Live Q&A**

10:30-11:30 CHANNEL 2

### E³ - ECR Master Class

### E³ 126

### Update of diffusion-weighted MRI

**Moderator**
Sören Rafaelsen; Vejle/DK

### E³ 126-2

**A. Technical aspects of diffusion-weighted imaging (DWI)**
Hero K. Hussain; Beirut/LB
*(hh141@aub.edu.lb)*

**Learning Objectives:**
1. To learn about the strengths and shortcomings of different DWI techniques.
2. To understand how to integrate and optimise DWI in MRI protocols.
3. To be able to avoid pitfalls in DWI sequencies.

### E³ 126-3

**B. DWI of abdominal organs**
Damiano Caruso; Rome/IT
*(dcaruso85@gmail.com)*

**Learning Objectives:**
1. To learn how to integrate DWI in your abdominal MRI protocol.
2. To understand the diagnostic value of DWI for abdominal pathologies.
3. To be able to deal with technical difficulties of DWI in the upper abdomen.

### E³ 126-4

**C. DWI of pelvic organs**
Evis Sala; Cambridge/UK

**Learning Objectives:**
1. To understand the correct implementation of DWI for pelvic imaging.
2. To learn how to avoid imaging artefacts in pelvic DWI.
3. To become familiar with typical findings and pitfalls of pelvic DWI.

**Live Q&A**

10:30-12:45 CHANNEL 3

### Hands-on Workshop: Cardiac CT

### HW

### Ready? Set! Cardiac CT!

### HW-1

**Cardiac CT: indications and how to select your patients**
Marc Dewey; Berlin/DE
*(dewey@charite.de)*

**Learning Objectives:**
1. To understand proper indications for the performance of cardiac CT.
2. To develop a systematic approach to select patients for cardiac CT.
3. To learn about use of pretest probability.

**Author Disclosures:**
Marc Dewey: Prof. Dewey has received grant support from the FP7 Program of the European Commission for the randomized multicenter DISCHARGE trial (603266-2, HEALTH-2012.2.4.-2). He also received grant support from German Research Foundation (DFG) in the Heisenberg Program (DE 1361/14-1), graduate program on quantitative biomedical imaging (BIOQIC, GRK 2260/1), for fractal analysis of myocardial perfusion (DE 1361/18-1), the Priority Programme Radiomics for the investigation of coronary plaque and coronary flow (DE 1361/19-1 [428222922] and 20-1 [428223139] in SPP 2177/1). He also received funding from the Berlin University Alliance (GC_SC_PC 27) and from the Digital Health Accelerator of the Berlin Institute of Health. Prof. Dewey is European Society of Radiology (ESR) Research Chair (2019–2022) and the opinions expressed in this article are the author's own and do not represent the view of ESR. Per the guiding principles of ESR, the work as Research Chair is on a voluntary basis and only remuneration of travel expenses occurs. Prof. Dewey is also the editor of Cardiac CT, published by Springer Nature, and offers hands-on courses on CT imaging (www.ct-kurs.de). Institutional master research agreements exist with Siemens, General Electric, Philips, and Canon. The terms of these arrangements are managed by the legal department of Charité – Universitätsmedizin Berlin. Professor Dewey holds a joint patent with Florian Michallek on dynamic perfusion analysis using fractal analysis (PCT/EP2016/071551).

### HW-2

**Cases**
Viktoria Wieske; Berlin/DE
A case with normal coronary arteries (no plaques, no stenosis).
A case with plaque(s) but without significant stenosis.
A case with one-vessel disease.

### HW-3

**Coronary arteries: anatomy and calcium scoring**
Rozemarijn Vliegenthart; Groningen/NL
*(r.vliegenthart@umcg.nl)*

**Learning Objectives:**
1. To understand normal coronary anatomy and coronary anomalies.
2. To develop a systematic approach for the evaluation of calcium score and coronary arteries.
3. To learn about plaque and stenosis evaluation.

**Author Disclosures:**
Rozemarijn Vliegenthart: Grant Recipient: Institutional Research Grant, Siemens Healthineers; Netherlands Organisation for Scientific Research; Dutch Heart Foundation

### HW-4

**Cases**
Giuseppe Muscogiuri; Milan/IT
*(g.muscogiuri@gmail.com)*
A case with benign anomalous origin of coronary artery.
A case with malign anomalous origin of coronary artery.
A case of a patient with high values of calcium score and multivessel CAD.

### HW-6

**CTA: patient preparation and how to perform cardiac CT**
Viktoria Wieske; Berlin/DE
*(viktoria.wieske@charite.de)*

**Learning Objectives:**
1. To understand why it is important to achieve a low and steady heart rate for cardiac CT.
2. To develop a systematic approach for preparing your patient for cardiac CT.
3. To learn about the use and importance of beta-blockers and nitroglycerin in the context of cardiac CT.

**Funding for this study:**
Grant support from the FP7 Program of the European Commission for the randomized multicenter DISCHARGE trial (603266-2, HEALTH-2012.2.4.-2).

### HW-7

**Cases**
Matthias Rief; Berlin/DE
*(matthias.rief@charite.de)*
A case with motion artifacts/non-diagnostic segment or vessel.
A case with high heart rate but good image quality.
A case with two-vessel disease.

**Funding for this study:**
Dr. Rief received grant support from German Research Foundation (DFG) in the Priority Programme Radiomics for the investigation of coronary plaque (Deutsche Forschungsgemeinschaft (DFG) - Projektnummer 428222922 in SPP 2177/1). Institutional master research agreements exist with Siemens, General Electric, Philips, and Canon. The terms of these arrangements are managed by the legal department of Charité – Universitätsmedizin Berlin.

### HW-8

**Analysis and CT report: how to systematically report and manage cardiac and non-cardiac findings**
Rozemarijn Vliegenthart; Groningen/NL
*(r.vliegenthart@umcg.nl)*

**Learning Objectives:**
1. To understand why it is important to systematically look for cardiac and non-cardiac findings.
2. To develop a systematic approach to analyze cardiac and non-cardiac findings.
3. To learn how not to miss important cardiac and non-cardiac findings.

**Author Disclosures:**
Rozemarijn Vliegenthart: Grant Recipient: Institutional research grant, Siemens Healthineers; Netherlands Organisation for Scientific Research; Dutch Heart Foundation

### HW-9
**Cases**
Firdaus A.A. Mohamed Hoesein; Utrecht/NL
A case with relevant extra-cardiac finding in abdomen.
A case with relevant extra-cardiac finding in the thorax.
A case with relevant cardiac finding.

**10:30-11:30 CHANNEL 4**

### Research Presentation Session: Musculoskeletal

### RPS 110

### Deep learning and novel imaging applications in musculoskeletal radiology

**Moderator**
Marc-André Weber; Rostock/DE

### RPS 110-2
**Multitask deep learning for simultaneous detection, segmentation, and classification of bone tumours on x-ray**
*C. E. von Schacky*, N. Wilhelm, V. Schäfer, Y. N. Leonhardt, C. Knebel, M. R. Makowski, K. Wörtler, R. Burgkart, A. S. Gersing; Munich/DE

**Purpose:** To use multitask deep learning for simultaneous detection, segmentation, and classification of bone tumors on x-ray.
**Methods or Background:** In this retrospective study bone tumors on x-rays prior to treatment were analyzed in patients (n=934, age 33.2±19.3y, 419 women) diagnosed with benign (n=673) and malignant (n=261) bone lesions using histopathology as standard of reference. Overall, 16 tumor entities were included: osteochondromas, enchondromas, chondroblastomas, osteoid osteomas, NOFs, giant cell tumors, chordomas, haemangiomas, simple and aneurysmatic bone cysts, fibrous dysplasia, chondrosarcomas, osteosarcomas, Ewing's sarcomas, plasma cell myelomas, and b cell NHLs. Bounding box placements and segmentations were performed by a radiology resident and reviewed by a fellowship-trained musculoskeletal radiologist. Patients were split 70%(n=654)/15%(n=140)/15%(n=140) for training, validation, testing. The multitask DL model was based on ResNeXt-101 implemented in PyTorch. Classification predictions are improved by the information of detection and segmentation, and vice versa thus capitalizing on knowledge gained by conducting each of the three tasks.
**Results or Findings:** The multitask model achieved 83% accuracy for the classification of bone lesions as malignant or benign on the test set. The model achieved an accuracy of 51% for correct subclassification into one in 16 tumor entities, placed 82% of the bounding boxes correctly with an intersection over union of 0.52±34, and had an average dice score of 0.60±37 for segmentations.
**Conclusion:** Multitask deep learning allowed for accurate detection, segmentation, and classification of bone tumors on x-ray. Multitask deep learning may improve accuracy by capitalizing on knowledge gained by conducting different tasks simultaneous.
**Limitations:** No external validation set was used.
**Ethics Committee Approval:** This study was approved by the local institutional review and informed consent was waived.
**Funding for this study:** This work was supported by the Clinician Scientist Program at Technische Universität München (Kommission für Klinische Forschung, KKF).
**Author Disclosures:**
Nikolas Wilhelm: Nothing to disclose
Claudio E. von Schacky: Nothing to disclose
Rainer Burgkart: Nothing to disclose
Marcus R. Makowski: Nothing to disclose
Valerie Schäfer: Nothing to disclose
Alexandra Sophia Gersing: Nothing to disclose
Carolin Knebel: Nothing to disclose
Yannik Niklas Leonhardt: Nothing to disclose
Klaus Wörtler: Nothing to disclose

### RPS 110-3
**Ultrahigh-resolution ultrasound in systemic sclerosis: an evaluation of digital and nailfold perfusion with a 33-9MHz probe**
*M. Pansecchi*[1], L. Tovt[1], F. Zaottini[1], R. Picasso[1], F. Pistoia[1], S. Sanguinetti[1], M. Miguel-Pérez[2], C. Martinoli[1]; [1]Genoa/IT, [2]Barcelona/ES

**Purpose:** The aim of this study was to investigate the role of spectral analysis performed by mean of ultra-high frequency US in the evaluation of the peripheral vascular bed of Systemic sclerosis (SSc) patients.
**Methods or Background:** An US machine equipped with 33-9 MHz and 18-5 MHz transducers was employed to study both of n=33 patients with SSc and n=34 healthy controls. On both hands we calculated the proximal resistive index (pRI) and the peak systolic velocity (PV) of the second interdigital artery orienting the 18-5 MHz transducer along the long axis of the vessel, on the palmar aspect of the third interdigital space. Distal resistive index (dRI) was calculated performing Doppler spectral analysis at the level of a nailfold arteriole of the third finger employing a proprietary technology based on Doppler technology and integrated within the ultrasound system [Superb Microvascular Imaging®(SMI)] which allows visualization and sampling of microvascular flows.
**Results or Findings:** Detection and sampling of nailfold vessels flow resulted generally easier in volunteers than in patients. Amongst patients, it was possible to collect dRI in n=26 cases whereas PV and pRI were obtained in n=32 of them. Compared to controls, patients demonstrated significant lower PV and higher dRI, whereas no differences were shown in pRI.
**Conclusion:** US spectral analysis is able to detect the disease-related variation in distal flow parameter in SSc patients. Further studies with larger series are needed to validate the use of high-resolution US in the investigation of the SSc peripheral vasculopathy.
**Limitations:** Further studies with larger series are needed to validate the use of high-resolution US in the investigation of the SSc peripheral vasculopathy
**Ethics Committee Approval:** The study was conducted in accordance with the declaration of Helsinki.
**Funding for this study:** None
**Author Disclosures:**
Carlo Martinoli: Nothing to disclose
Luca Tovt: Nothing to disclose
Maribel Miguel-Pérez: Nothing to disclose
Michelle Pansecchi: Nothing to disclose
Sara Sanguinetti: Nothing to disclose
Riccardo Picasso: Nothing to disclose
Federico Pistoia: Nothing to disclose
Federico Zaottini: Nothing to disclose

### RPS 110-4
**Development of deep learning models to differentiate atypical lipomatous tumours and lipomas on MR images**
*D. W. Kramp*, C. E. von Schacky, T. Tomov, F. Navarro, C. Knebel, M. R. Makowski, K. Wörtler, S. Foreman, A. Gersing; Munich/DE

**Purpose:** To develop deep learning (DL) models to differentiate atypical lipomatous tumors (ALTs) from lipomas on MRI.
**Methods or Background:** Patients included in this study (N=109, age 54.8±14.5 years, 58 women) were diagnosed with lipomas (N=64) or ALTs (N=45), using histology and analysis of the murine double minutes (MDM2) gene as standard of reference. Preoperative MR imaging included T2w and T1w sequences, and a fat-suppressed T1w sequence with contrast enhancement (T1fsgd). For final and independent testing, 20% of the patients were randomly selected and removed from the dataset. Training and validation was performed on the remaining 80% using 3-fold cross validation. Segmentations were performed by a radiology resident to be used as input to the models. In addition, preoperative MR images were assessed by one board-certified radiologist and specialist for musculoskeletal imaging with over 25 years of experience and two radiology residents, blinded to histological and clinical data. A final diagnosis ALT/Lipoma was determined using standard MR imaging characteristics (septation, tumor diameter, presence of nodular components or contrast enhancement).
**Results or Findings:** The DL model achieved a sensitivity/specificity/accuracy of 89%/85%/86% on T1w images; 67%/67%/67% on T2w images; and 80%/56%/64% on T1fsgd images. The overall sensitivity/specificity/accuracy of the resident readings was 78%/77%/77% and 89%/100%/95% of the musculoskeletal specialist readings.
**Conclusion:** A DL model developed to differentiate ALTs from lipomas on preoperative MR images achieved 89% sensitivity at 85% specificity. Compared to a musculoskeletal specialist, the DL model had the same sensitivity at a lower specificity. Compared to resident readings, the DL model had a higher sensitivity and higher specificity. This model may aid radiologists in diagnostic workflow to differentiate ALTs from lipomas.
**Limitations:** Limited sample size.
**Ethics Committee Approval:** Approval of the institutional review board.
**Funding for this study:** Clinician Scientist Program (KKF) at TUM.

**Author Disclosures:**
Claudio E. von Schacky: Nothing to disclose
Alexandra Gersing: Nothing to disclose
Marcus R. Makowski: Nothing to disclose
Tim Tomov: Nothing to disclose
Carolin Knebel: Nothing to disclose
Daniel Wolfgang Kramp: Nothing to disclose
Fernando Navarro: Nothing to disclose
Klaus Wörtler: Nothing to disclose
Sarah Foreman: Nothing to disclose

### RPS 110-5

**Diagnosis of joint invasion in patients with malignant bone tumours: value and reproducibility of direct and indirect signs on MR imaging**

*J. H. W. Bodden*, J. Neumann , A. Fingerle, M. Rasper, C. Knebel, K. Specht, C. Bollwein, A. S. Gersing, K. Wörtler; Munich/DE
*(jannis.bodden@tum.de)*

**Purpose:** To evaluate the performance and reproducibility of new and previously described MR imaging features in the diagnosis of joint invasion (JI) by malignant bone tumors.
**Methods or Background:** Pretherapeutic MR examinations of patients with(n=24) and without(n=24) pathologically proven JI by bone tumors (n=46 sarcomas,n=2 metastases) were independently assessed by three readers, blinded for pathologic outcome. Tumors were located at the shoulder(n=7), hip(n=14) or knee(n=27). On MR images, direct (intrasynovial tumor tissue(ITT), intraarticular destruction of cartilage/bone, invasion of capsular/ligamentous insertions) and indirect signs (tumor size, signal alterations of epiphyseal/transarticular bone(bone marrow replacement/edema-like), synovial contrast enhancement, joint effusion) of JI were assessed. Associations between imaging features and presence of JI were investigated using exact logistic regression models. Sensitivities, specificities, positive(PPV) and negative predictive values(NPV) were calculated, and inter- and intra-reader reproducibility was determined using Fleiss' and Cohen's κ.
**Results or Findings:** JI could be diagnosed with a sensitivity of 92%-100% and a specificity of 88%-100%, with direct visualization of ITT and destruction of intraarticular bone as the best predicting signs($OR \geq 21.25$,$P<0.01$, for each reader). Direct visualization of ITT was also highly reliable(sensitivity,specificity,PPV,NPV:92%-100%), with excellent inter- and intrareader reproducibility (κ=0.95). Epiphyseal bone marrow replacement and synovial contrast enhancement were the most sensitive indirect signs (87%-96%), but lacked specificity(29%-54%). By combining direct signs with high specificity, sensitivity was increased(96%) and specificity(100%) was maintained.
**Conclusion:** Joint invasion by malignant bone tumors can reliably be assessed on preoperative MR images with excellent sensitivity, specificity and reproducibility. Direct visualization of ITT and destruction of intraarticular bone were most valuable, while indirect signs were less reliable. Combining multiple specific signs may further increase the sensitivity while maintaining high specificity.
**Limitations:** Limitations include possible reader bias and cohort size.
**Ethics Committee Approval:** Ethics committee approved this study.
**Funding for this study:** No external funding sources.
**Author Disclosures:**
Katja Specht: Nothing to disclose
Michael Rasper: Nothing to disclose
Alexandra Sophia Gersing: Nothing to disclose
Alexander Fingerle: Nothing to disclose
Carolin Knebel: Nothing to disclose
Jannis Hubert Wilhelm Bodden: Nothing to disclose
Christine Bollwein: Nothing to disclose
Klaus Wörtler: Nothing to disclose
Jan Neumann : Nothing to disclose

### RPS 110-7

**Suprapatellar fat pad MRI abnormalities in asymptomatic subjects: what is normal?**

*A. Cosentino*[1], R. Richard[1], M. Baron[2], H. Babel[2], J. Favre[2], P. Omoumi[2]; [1]Rennaz/CH, [2]Lausanne/CH

**Purpose:** To study the prevalence of supra-patellar fat pad (SPFP) MRI alterations in a cohort of asymptomatic subjects, in correlation with a wide range of clinical/radiological evaluations, including muscle performance tests and physical activity data.
**Methods or Background:** We prospectively included 110 asymptomatic subjects from 07.2019 to 09.2020 as part of a cohort study. Inclusion criteria were no knee pain in the last year. Exclusion criteria were any previous knee disorder/procedure, including osteoarthritis. Subjects underwent knee radiographs, low-dose posture radiographs [EOS®], 3T MRI, clinical examination including quadriceps and hamstrings performance tests and physical activity quantification, monitored digitally for 1 week. Study groups were based on presence/absence of SPFP alterations: hyperintensity and mass effect (qualitative evaluation, 3 radiologists in consensus) on fluid-sensitive sequences. Correlations were tested with a total of 55 categorical/continuous clinical/radiological variables, including SPFP relative T2-signal, trochlear/patellar and lower-limb morphologic measurements. Mann-Whitney and Chi-squared test were used to compare sub-groups.
**Results or Findings:** SPFP alterations were common in asymptomatic subjects: 57% (63/110) hyperintensity and 37%(41/110) mass effect, with 27% (30/110) of subjects showing both. Among the 55 variables tested, a correlation was found only between SPFP mass-effect and increasing patellar tilt angle ($p=0.02$); SPFP hyperintensity and younger age ($p=0.04$). No other radiological, clinical or physical activity variable showed correlation with the SPFP alterations.
**Conclusion:** SPFP high signal abnormality and mass effect are common findings at knee MRI of asymptomatic subjects (57% and 37%, respectively). Their presence was correlated with only one parameter related to patello-femoral morphology, and age (for isolated SPFP hyperintensity). No other correlations were found, including with muscle power or physical activity. Therefore, they most likely represent normal variants and care should be taken not to overcall them as pathological findings.
**Limitations:** No histopathology.
**Ethics Committee Approval:** Yes
**Funding for this study:** Yes
**Author Disclosures:**
Margaux Baron: Nothing to disclose
Julien Favre: Nothing to disclose
Hugo Babel: Nothing to disclose
Raphaël Richard: Nothing to disclose
Aurelio Cosentino: Nothing to disclose
Patrick Omoumi: Nothing to disclose

**Live Q&A**

**10:30-11:30 CHANNEL 5**

### Special Focus Session

### SF 26

### Recent developments in diagnosing congenital and acquired vascular and lymphatic disorders in children: when, how, and why?

**Moderator**
Willemijn M. Klein; Nijmegen/NL

### SF 26-2

**Treatment of vascular disorders in children**

Anne Marie Cahill; Philadelphia, PA/US
*(CAHILL@email.chop.edu)*

**Learning Objectives:**
1. To learn about the indications for conservative and interventional treatment.
2. To understand the potential applications and techniques of interventions.
3. To discuss the risks and limitations.

### SF 26-3

**Diagnosing lymphatic disorders in children**

Pär Gerwins; Uppsala/SE
*(par.gerwins@akademiska.se)*

**Learning Objectives:**
1. To learn about typical lymphatic pathologies in children.
2. To learn about the techniques of MR lymphography.
3. To discuss the appropriate work-up of lymphatic disorders.

### SF 26-4

**Interventional treatment of lymphatic disorders**

Ralph Gnannt; Zurich/CH
*(ralph.gnannt@usz.ch)*

**Learning Objectives:**
1. To learn about the indications for conservative and interventional treatment.
2. To understand the potential applications and techniques of interventions.
3. To discuss the risks and limitations.

**Live Q&A: How to optimise diagnosis and treatment of lymphatic and vascular disorders in children?**

**11:45-12:45** **CHANNEL 2**

### Special Focus Session

### SF 22

### My three top tips in post-mortem imaging (PMI)

**Moderator**
Rick R. van Rijn; Amsterdam/NL

### SF 22-2
**Post-mortem imaging: how to do it**
Jeroen Kroll; Maastricht/NL
*(j.kroll@mumc.nl)*

**Learning Objectives:**
1. To discuss the role of post-mortem imaging.
2. To discuss the organisational and logistic issues you will experience.
3. To discuss the added value of trained post-mortem radiographers.

### SF 22-3
**Foetal and perinatal PMI**
Owen J. Arthurs; London/UK

**Learning Objectives:**
1. To understand how to choose the best imaging modality.
2. To learn about normal post-mortem changes.
3. To practice "safe" image interpretation.

### SF 22-4
**Paediatric PMI**
Guillaume Gorincour; Marseille/FR
*(guillaume.gorincour@gmail.com)*

**Learning Objectives:**
1. To review and explain the scope of post-mortem foetal ultrasound.
2. To describe a simple technique of perinatal post-mortem angio-CT.
3. To show the relevance of PMCT in suspected neonaticide.

**Author Disclosures:**
Guillaume Gorincour: Advisory Board: AGFA Healthcare; Consultant: Philips Ultrasound; Employee: Imadis Teleradiologie; Founder: IMAGE 2; Share Holder: DeepLink Medical; Other: Scientific Director / Imadis Teleradiologie

### SF 22-5
**Adult neuro PMI**
Thomas Ruder; Berne/CH

**Learning Objectives:**
1. To understand how post-mortem changes mimic or modify the appearance of pathology on PMI.
2. To learn how reporting PMI can improve your clinical radiology reports.
3. To become familiar with the potential for knowledge transfer between PMI and clinical radiology.

### SF 22-6
**Adult trauma and musculoskeletal PMI**
Krzysztof Wozniak; Krakow/PL
*(mpwoznia@cyf-kr.edu.pl)*

**Learning Objectives:**
1. To present issues referring to PMCT evaluation of victims of transportation accidents leading to forensic medical reconstruction of the critical event.
2. To deliver examples of PMCT documentation of firearm injuries giving the opportunity for reconstruction of the track(s) of the projectile.
3. To show results of PMCT-Angiography evaluation of stab wounds as the background of medico-legal reconstruction of injury tracks and the course of the critical event.

### SF 22-7
**Adult chest PMI**
Sue Kearney; Preston/UK
*(Susan.kearney@lthtr.nhs.uk)*

**Learning Objectives:**
1. To recognise CTPM appearances of various cardiothoracic diseases and how to use this information to offer a cause of death for the coroner.
2. To learn about the use of lung ventilation and coronary angiography.
3. To understand the limitations of CTPM and when to consider invasive autopsy.

### SF 22-8
**Adult abdomen PMI**
Sarah Heinze; Heidelberg/DE
*(Sarah.Heinze@med.uni-heidelberg.de)*

**Learning Objectives:**
1. To learn about typical post-mortem abdominal changes.
2. To recognise the common pitfalls of abdominal post-mortem imaging.
3. To understand that special knowledge for post-mortem imaging is necessary.

**Live Q&A: PMI: complement or replacement of autopsy?**

**11:45-12:45** **CHANNEL 4**

### Coffee & Talk (open forum) Session
*Organised by the ESR Value-Based Radiology Subcommittee*

### C 5

### Communicating with patients: adding value to the patient’s first encounter with radiology

**Moderator**
Adrian Brady; Cork/IE

### C 5-2
**I'm the patient, not an inanimate object: talk to me!**
Judy Birch; Poole/UK

**Learning Objectives:**
1. To learn about the importance of radiologists listening to their patients.
2. To appreciate the need to inform patients about their care at the appropriate level, taking into account their history and what they say.
3. To understand the need for radiologists to prepare background information for patients.

### C 5-3
**Ultrasound: we're touching the patients, why not talk to them too?**
Elena E. Drakonaki; Iraklion/GR *(drakonaki@yahoo.gr)*

**Learning Objectives:**
1. To appreciate the importance of a good level of communication between the radiologist and the patient during US examinations.
2. To discuss the pros and cons of communicating the results of the US scan to the patient.
3. To understand the challenges associated with verbal and non-verbal communication during US scanning.

### C 5-4
**Interventional radiology: if we don't explain what we're doing, who else should?**
Adrian Brady; Cork/IE *(adrianbrady@me.com)*

**Learning Objectives:**
1. To learn the responsibility of the interventional radiologist to explain procedures to patients.
2. To appreciate how direct explanations can alleviate patient anxiety.
3. To understand that interventional radiology represents a valuable opportunity to engage directly with patients.

**Live Q&A**

**11:45-12:45** **CHANNEL 5**

### Research Presentation Session: Emergency Imaging

### RPS 217

### Triaging and investigating patients in a pandemic: added value from ultrasound?

**Moderator**
Mariano Scaglione; Castel Volturno/IT

### RPS 217-2

**Outpatient CT-centre for emergency triage of COVID-19 patients: local experience from Saint Petersburg**

*M. Cherkashin*, N. Berezina, A. Nikolaev, D. I. Kuplevatskaya, V. Kuplevatsky, D. Lisovets, A. Boikov; Saint Petersburg/RU
*(mikhail.a.cherkashin@gmail.com)*

**Purpose:** To discuss organizational principles of outpatient CT-based triage emergency center
**Methods or Background:** Novel coronavirus infection characterized by great influence on healthcare systems all over the world. During first peak, number of patients was devastating and clinical decisions were restricted by system capacity. Hospital's emergency departments were full with COVID-patients, ambulances spent a lot of hours in lane for patient's hospitalization. In April 2020 on City level we've started with outpatient triage. Centers were equipped by CT, crash-cart, vital monitor and defibrillator, oxygen. There were consultant physician, radiographer and medical receptionist inside. Radiologists assess images remotely. Patients were admitted by ambulance in CT-center, assessed by consultant physician and based on chest CT results (severity of disease) and clinical status, decisions for hospitalization were conducted. Due to hazardous environment all staff were equipped by Level 2 PPE and strong infection control procedures were implemented.
**Results or Findings:** Since April until November 2020, 37537 suspected and laboratory tested COVID patients were triaged. We've used local radiology pneumonia severity classification (CT 1-4 points). Patients with CT 1-2 points and good clinical performance were way back to home for observation by ambulatory physician, patients with CT 3-4 points and dyspnea, SpO2 less than 94%, comorbidity etc were admitted at the hospital. During 7 months 21986 patients with COVID pneumonia were diagnosed. We've hospitalized 5532 moderate-to-severe and critical patients, 32005 were referred for home treatment.
**Conclusion:** Based on our study results we propose some sentences: outpatient COVID-19 patients triage with computed tomography is the acceptable strategy in respiratory pandemic environment; CT-triage is the fast tool for clinical decision; infection control is the key point for medical staff safety.
**Limitations:** It's ongoing study with interim results
**Ethics Committee Approval:** Study was approved by LEC
**Funding for this study:** Hospital based funding
**Author Disclosures:**
Alexey Boikov: Nothing to disclose
Mikhail Cherkashin: Nothing to disclose
Natalia Berezina: Nothing to disclose
Aleksey Nikolaev: Nothing to disclose
Vladimyr Kuplevatsky: Nothing to disclose
Dmitry Lisovets: Nothing to disclose
Daria Igorevna Kuplevatskaya: Nothing to disclose

### RPS 217-3

**Improving efficacy and efficiency in an emergency unit by using the radiology department as the entry door of COVID-19 patients from the primary care network**

J. M. Plasencia Martinez, *M. Lozano Ros*, P. Fabuel Hernández, M. d. C. Sánchez Ayala, G. Pérez Hernández, J. M. M. Garcia Santos; Murica/ES
*(marina.lozano93@gmail.com)*

**Purpose:** To reduce COVID-19 risk of contagion in non-respiratory patients in our ED, we implemented a circuit to perform immediate chest X-rays (CXR) on possible/confirmed COVID-19 patients in home confinement and telephone follow-up by GPs with an unfavorable evolution, without sending the patient to the ED.
**Methods or Background:** We classified patients into:- G1: entrance through High Resolution Radiology Supply (HRRS). Normal CXR - back home.- G2: entrance through HRRS. Pneumonic findings on CXR - ED. - G3: repiratory patients entrying directly through ED.We consider as duration of the process in G1, the period between the CXR appointment time and report validation by radiologists; and in G2 and G3, the period between the arrival of the patient to ED admission and the report signing by the emergency doctor. IBM Statistics SPSS 22 version program was used. Anova test with Bonferroni correction, Kruskal-Wallis test and Chi2 test were applied. Statistically significant differences were considered when $P<0.05$.
**Results or Findings:** From March 26th to April 17th, the number of respiratory patients attended by HRRS (419) and directly by ED (439) were similar. 65% ([325 G1+1 G2+228 G3]/849) of patients returned back home.G1 patients (0:41 ± 1:05h) stayed in hospital significantly less time than G2 and G3 subjects (5:25 ± 3:08h and 5:36 ± 4:36h; $P<0.001$), even when G2 and G3 patients were forwarded home (3:36 ± 2:58 h and 3:50 ± 3:16 h; $P<0.001$).G2 patients were hospitalized (84/93; 90.3%) more frequently than G3 (203/439; 46.2%, $P<0.001$).
**Conclusion:** A straightforward HRRS could triage and decrease respiratory patients at ED during COVID-19 pandemic, reduce waiting times and yield fast admission decisions.
**Limitations:** Not having all clinical and radiological follow-up data of patients.
**Ethics Committee Approval:** Yes
**Funding for this study:** No
**Author Disclosures:**
María del Carmen Sánchez Ayala: Nothing to disclose
Marina Lozano Ros: Nothing to disclose
Juana María Plasencia Martinez: Nothing to disclose
Jose Maria Garcia Santos: Nothing to disclose
Pablo Fabuel Hernández: Nothing to disclose
Gloria Pérez Hernández: Nothing to disclose

### RPS 217-5

**Point-of-care lung ultrasound and echocardiography simulation module for training frontline physicians to triage and monitor coronavirus patients**

*G. Bartal*, T. Avziz, I. Harel, A. Shouval, V. Levin, R. Weiss; Tel Aviv-Yafo/IL
*(gbartal@gmail.com)*

**Purpose:** POCUS (point-of-care ultrasound) is considered an alternative to chest radiography and echocardiography in COVID-19 patients. POCUS is bedside, quick for use, repeatable, low cost, and radiation-free. Training frontline and intensive care physicians to rapidly acquire POCUS skills can be critical for the success of COVID-19 patient management. Our aim was to develop and implement POCUS virtual reality simulator for hands on training of non-radiologists.
**Methods or Background:** Based on published COVID-19 lung ultrasound imaging findings, the simulated ultrasound image have been designed to visualize the typical COVID-19 findings, with a variety of patient lung pathologies, from mild to severe. Soon after the lung ultrasound module was adapted the existing echocardiography simulation module was modified and embedded into the new comprehensive simulation system. The current module includes unique lung ultrasound and Echocardiography clinical cases of patients suspected or diagnosed with COVID-19. In addition, the module is hands on and comprises COVID-19 rich ultrasound educational library material that includes videos and protocols from key opinion leaders.
**Results or Findings:** The COVID-19 POCUS module was released in only two weeks of fast-tracked development and installed on dozens of Ultrasound Mentor simulators (Simbionix, 3D Systems Healthcare) worldwide. In certain countries, mobile training units were designated to train physicians to triage patients using POCUS. Eventually, the rapid development of the simulated module helped to improve and to expand patient care during Pandemic.
**Conclusion:** Lung Ultrasound and echocardiography simulation and training solution allowed efficient, swift, and validated recruitment of primary and intensive care physicians to triage and to follow-up COVID-19 patients using POCUS techniques in the course of the Pandemic. Evaluation of clinical results and physicians' satisfaction using validated questionnaires is underway.
**Limitations:** Retrospective design.
**Ethics Committee Approval:** Not required
**Funding for this study:** No funding.
**Author Disclosures:**
Iris Harel: Employee at Simbionix 3DSysteHealthcare
Victor Levin: Employee at Simbionix 3DSysteHealthcare
Assaf Shouval: Employee at Simbionix 3DSysteHealthcare
Ran Weiss: Employee at Simbionix 3DSysteHealthcare
Gabriel Bartal: Consultant at Simbionix 3DSysteHealthcare
Tal Avziz: Employee at Simbionix 3DSysteHealthcare

### RPS 217-7

**Can ultrasound alone suffice in the evaluation of acute abdomen in adults: a comparative ultrasound versus computed tomography (hospital-based) study**

*S. Awasthi*, V. Gupta, S. B. Grover; Greater Noida/IN
*(swatiawasthi.cool08@gmail.com)*

**Purpose:** The objective of this study was to compare the sensitivity of Ultrasound (US) and Computed tomography (CT) in evaluation of acute abdomen and to assess whether Ultrasound alone will suffice in majority situations, so that radiation exposure may be minimized.
**Methods or Background:** This institutional review board approved prospective observational study comprised of 50 adult patients with acute abdomen. The exclusion criteria were trauma and pregnancy. US and CT (non-contrast and contrast enhanced) were performed by standard protocol in all patients.
**Results or Findings:** The average exposure from the PACS database for acute abdomen CT protocol, was found to be 10mSv. Although the overall sensitivity of US was 78% and that of CT was 96% with significant difference (p value – 0.01), the sensitivity of US compared to CT in diagnosing common etiologies of acute abdomen namely acute cholecystitis, acute appendicitis, intestinal obstruction, acute pancreatitis and ureteric calculi sensitivity did not differ significantly (p value of 1.00, 0.47, 0.50, 0.06, 1.00 respectively). CT was necessary only in patients with retro-caecal appendicitis, bowel obstruction cases for defining the transition point and in patients with acute pancreatitis to obtain the CT Severity index.
**Conclusion:** We propose that US should remain the primary imaging modality in all patients of acute abdomen in order to prevent radiation exposure, especially as it was found to have a sensitivity comparable to CT, in majority of the clinical situations. Moreover, it is a more cost-effective investigation. Therefore, CT should be reserved only for the minority of clinical situations where US is significantly inconclusive. We propose to extend the study for a larger cohort of patients.
**Limitations:** Small sample size
**Ethics Committee Approval:** Yes
**Funding for this study:** Not Applicable
**Author Disclosures:**
Shabnam Bhandari Grover: Nothing to disclose
Swati Awasthi: Nothing to disclose
Vishal Gupta: Nothing to disclose

### RPS 217-8

**High-resolution ultrasonographic (HRUS) evaluation of subglottic diameter for preanesthetic estimation of endotracheal tube diameter**
*N. Neha*, R. Rastogi, V. Pratap; Moradabad/IN
*(neha11aug@gmail.com)*

**Purpose:** Endotracheal intubation which is a prerequisite for variety of surgical procedures is mainly governed by the narrowest diameter of the upper airway. Size of the endotracheal tube can be calculated by physical indices or invasively by laryngoscopy. Over-size tubes lead to airway injuries while under-size tubes leads to inadequate ventilation. High-resolution ultrasonography (HRUS) not only allows objective measurement of airway dimensions but is also noninvasive & cheap technique. This study aimed to:
•Determine the accuracy of HRUS-determined diameter compared with endotracheal tube diameter used clinically during the procedure.
**Methods or Background:** The study was carried on 25 patients appearing for preanesthetic check-up for elective surgery. HRUS was performed on these patients using a high-frequency, linear-array probe. The subglottic, transverse, tracheal diameter was measured just inferior to true vocal cords at the level of cricoid arch. The diameter of endotracheal tube used clinically during the anaesthesia was be noted along with the endotracheal tube diameter determined by physical indices.The diameters thus obtained by three methods were then matched with each other to determine the accuracy of the HRUS determined diameter.
**Results or Findings:** The age of the patients ranged from 3rd to 6th decade. The accuracy of HRUS determined subglottic diameter is approximately 75% in patients below the age of 50years.
**Conclusion:** HRUS is a safe and reliable imaging tool for preanesthetic determination of subglottic diameter for ET insertion.
Clinical Relevance
Though in experienced hands, HRUS-determined subglottic diameter may not be significantly useful in decreasing the number of attempts required for determination of clinically-useful ET diameter yet it may be definitely useful for trainees / residents / inexperienced hands and in times of emergency, thus reducing the morbidity related with intubation
**Limitations:** None
**Ethics Committee Approval:** None
**Funding for this study:** None
**Author Disclosures:**
Rajul Rastogi: Nothing to disclose
Neha Neha: Nothing to disclose
Vijai Pratap: Nothing to disclose

### RPS 217-9

**Chest x-ray in the emergency department during COVID-19 pandemic descending phase in Italy: correlation with patients' outcomes**
*D. Cozzi*, C. Moroni, E. Cavigli, A. Bindi, M. Albanesi, S. Luvarà, P. Nazerian, V. Miele; Florence/IT
*(dilettacozzi@gmail.com)*

**Purpose:** The aims of the study are: to estimate admission chest X ray (CXR) accuracy during the pandemic descending phase; to identify specific CXR findings strictly associated with COVID-19 infection and to correlate lung involvement of admission CXR with patients' outcome.
**Methods or Background:** We prospectively evaluated the admission CXR of 327 patients accessed to our institute during the Italian pandemic descending phase (April-July 2020). For each CXR were searched: ground glass opacification (GGO), consolidation (CO), reticular-nodular opacities (RNO), nodules, excavations, pneumothorax, pleural effusion, vascular congestion and cardiac enlargement. For lung alterations was defined the predominance (upper/basal, focal/diffuse, central7peripheral, etc.). Then radiologists assessed if CXRs were suggestive or not for COVID-19 infection. For COVID-19 patients a prognostic score was applied and correlated with the patients' outcome.
**Results or Findings:** CXR showed 83% of specificity and 60% of sensitivity. GGO, CO, RNO and a peripheral, diffuse and basal prevalence showed good correlation with COVID-19 diagnosis. A logistic regression analysis pointed out GGO and a basal or diffuse distribution as independent predictors of COVID-19 diagnosis. The prognostic score showed good correlation with the patients' outcome.
**Conclusion:** In our study, admission CXR showed a fair specificity and a good correlation with patients' outcome. GGO and others CXR findings showed a good correlation with COVID-19 diagnosis. Besides GGO and a diffuse or bibasal distribution resulted independent variables highly suggestive for COVID-19 infection.
**Limitations:** Our study has several limitations. First, authors lack interobserver agreement in the diagnostic evaluation and in the prognostic scoring of the CXR (all decisions were reached collegially). Also, we evaluated only admission CXR and we lack follow up imaging.
**Ethics Committee Approval:** Yes (rif. CEAVC 17104) from the Ethics Committee of our Hospital in Florence.
**Funding for this study:** None
**Author Disclosures:**
Alessandra Bindi: Nothing to disclose
Vittorio Miele: Nothing to disclose
Silvia Luvarà: Nothing to disclose
Edoardo Cavigli: Nothing to disclose
Peiman Nazerian: Nothing to disclose
Chiara Moroni: Nothing to disclose
Diletta Cozzi: Nothing to disclose
Marco Albanesi: Nothing to disclose

**Live Q&A**

**14:15-15:15** **CHANNEL 2**

### ISRRT meets Singapore

### Meets 6

### Radiography (imaging and therapy) services in Singapore

**Moderators**
Donna Newman; Fargo, ND/US
Napapong Pongnapang; Bangkok/TH

### Meets 6-3

**Introduction to Singapore's healthcare system and the radiography profession**
Denise Choong Ai Wen; Singapore/SG
*(president@ssr.org.sg)*

**Learning Objectives:**
1. To learn about Singapore's population and healthcare concerns.
2. To learn about the structure and financing of Singapore's health system.
3. To understand the radiography profession as part of Singapore's healthcare system.

### Meets 6-4
**Radiology services in Singapore**
Michael Ong Kah Leong; Singapore/SG
*(michael_ong@nuhs.edu.sg)*

**Learning Objectives:**
1. To become familiar with the types of radiography and radiation therapy services provided in Singapore.
2. To understand how Singapore's population accesses radiology services

### Meets 6-5
**The Allied Health Professions Council (AHPC) and regulation of professionals**
Gabriel Leong Kok Wah; Singapore/SG

**Learning Objectives:**
1. To understand the rationale for Singapore's AHPC and how radiographers and radiation therapists are regulated.
2. To obtain an overview of the radiography career opportunities in Singapore and hiring difficulties.
**Author Disclosures:**
Gabriel Leong Kok Wah: Employee: Changi General Hospital

### Meets 6-6
**Singapore's radiography education system**
Tan Chek Wee; Singapore/SG
*(chekweetan@nuhs.edu.sg)*

**Learning Objectives:**
1. To learn about the history and current status of Singapore's radiography education system.
2. To appreciate how we are preparing for professional challenges in the future.

**Live Q&A**

**14:15-15:15 CHANNEL 3**

### E³ - The Beauty of Basic Knowledge: Breast

### E³ 24B

### Basis of breast ultrasound and multimodality readings

**Moderators**
Panagiotis Kapetas; Vienna/AT
Karen Kinkel; Chêne-Bougeries/CH

### E³ 24B-3
**Practical ultrasound of the breast: how I do it**
Alexandra Athanasiou; Athens/GR
*(aathanasiou@mitera.gr)*

**Learning Objectives:**
1. To learn about patient positioning and image quality control.
2. To understand the value of colour Doppler ultrasound and elastography.
3. To learn about applying ultrasound breast imaging reporting and data systems (BIRADS).

### E³ 24B-4
**Integrating ultrasound findings into the final mammography report**
Eva M. Fallenberg; Munich/DE

**Learning Objectives:**
1. To understand how to assess concordance between mammography and ultrasound results.
2. To learn to give a final recommendation (no further action required, short-term follow-up, biopsy, other imaging modality, etc.).
**Author Disclosures:**
Eva M. Fallenberg: Advisory Board: Siemens Bayer Guerbet; Board Member: EUSOBI DRG; Research Grant/Support: GE Guerbet Siemens; Speaker: GE Siemens Bayer Guerbet

**Live Q&A**

**14:15-15:15 CHANNEL 5**

### Research Presentation Session: Vascular

### RPS 115

### Modern techniques in vascular diagnostics

**Moderator**
Krzysztof K. Pyra; Lublin/PL

### RPS 115-4
**Contrast-enhanced ultrasound perfusion patterns, risk factors, and serum lipid signatures of vulnerable carotid plaque in predicting stroke: a cohort study of carotid stenosis in Chinese patients**
*H. Yunqian*, M. Chen; Shanghai/CN

**Purpose:** To investigate the correlation between contrast-enhanced ultrasound (CEUS) perfusion patterns, Risk factors and serum lipid signatures of carotid artery plaques with the degree of carotid stenosis.
**Methods or Background:** We screened 2039 patients with plaque of the carotid ultrasound From March to June in 2018 years. A total of 80 patients were enrolled with who underwent CEUS, CTA or DSA, complete questionnaires(including demographics and Risk factors), and serum lipid testing. Exclusion criteria were age<18 or>85 years, plaque thickness less than 1.5mm, have a history of myocardial infarction in two weeks. The CEUS perfusion pattern were divided into plaque base entering and surface entering mode.
**Results or Findings:** The 50 participants with hypertension consisted of 32 (59.3%) males and 18 (69.2%) females. The 28 participants with plaque surface entering mode by the CEUS perfusion pattern. Based on our previous study that the contrast agent enhancement levels and the CEUS perfusion patterns in plaque were significantly associated with the degree of carotid stenosis (P<0.05), we found hypertension and increasing apolipoprotein E concentration were significantly associated with plaque surface entering mode by the CEUS perfusion pattern (P<0.05).
**Conclusion:** The contrast agent mainly infuses from the surface to the interior of the plaque on CEUS are indicative of vulnerable carotid plaques, prior to that, hypertension and increasing apolipoprotein E concentration may cause the variation of the Contrast-enhanced perfusion patterns. Together these factors may provide evidence of vulnerable plaques in those with carotid artery stenosis.
**Limitations:** This study was a retrospective, single-center study. We only assessed the neovascularization of plaques qualitatively, via direct observation.
**Ethics Committee Approval:** The study protocol was approved by the Institutional Ethics Review Committee and written informed consent was obtained from all participants.
**Funding for this study:** No external funding was provided for this study.
**Author Disclosures:**
Huang Yunqian: Nothing to disclose
Man Chen: Nothing to disclose

### RPS 115-5
**Are the morphological indices of the vertebrobasilar system heritable? A twin study**
*L. Szalontai*[1], Z. Jokkel[1], T. Horvath[1], M. Piroska[1], B. Forgo[2], D. L. Tarnoki[1], A. Tarnoki[1]; [1]Budapest/HU, [2]Örebro/SE

**Purpose:** Asymmetrical vertebral artery (VA) flow and diameter are common findings and are often left dominant. This can result in asymmetrical blood flow in the basilar artery (BA), which may cause bending of the artery over time. This study investigated whether the variation of the different vertebrobasilar morphological indices that influence flow characteristics might be inherited.
**Methods or Background:** We analyzed 200 cerebral MRI scans of healthy Caucasian twins (100 pairs). Self-reported questionnaires were used for zygosity classification and to collect detailed risk factors. All twins underwent time-of-flight MRI. We reconstructed a 3D mesh of the posterior circulation from the start of the V4 segment to the basilar top (ITK snap) and subsequently analyzed the morphology of the vertebrobasilar system with an in-house software written in Python. The phenotypic variance of the different morphological parameters was decomposed into heritability (A), shared (C), and unshared (E) environmental effects (ACE analysis).
**Results or Findings:** VA V4 segment diameter, curvature, tortuosity were mainly influenced by shared (C) and unshared (E) environmental factors. Moderate BA heritability was found for length (A: 63% ; 95%CI: 45,7-75,2%) (E: 37% ; 95%CI: 24,8-54,3%) and volume (A: 60,1%; 95%CI:42,4-73,2%) (E: 39,9%; 95%CI: 26,8-57,6%), while the torsion of both arteries showed no heritability and were only influenced by unshared environment.

**Conclusion:** The length and volume of the BA showed moderate genetical influence. However, most of the measured morphological indices were influenced by shared and unshared factors, which may highlight the complex hemodynamic background of the vertebrobasilar system.
**Limitations:** ToF-MRI may produce artifacts that can influence measurements.
**Ethics Committee Approval:** The study protocol conforms to the ethical guidelines of the 1975 Declaration of Helsinki and the study protocol has been priorly approved by the Semmelweis University Ethical Committee (TUKEB 264/2019).
**Funding for this study:** Not applicable.
**Author Disclosures:**
David Laszlo Tarnoki: Nothing to disclose
Bianka Forgo: Nothing to disclose
Marton Piroska: Nothing to disclose
Tamas Horvath: Nothing to disclose
Zsofia Jokkel: Nothing to disclose
Adam Tarnoki: Nothing to disclose
László Szalontai: Nothing to disclose

### RPS 115-6

**Estimated false lumen arc length is associated with late adverse events in uncomplicated type-B aortic dissection**

M. J. Willemink[1], M. Codari[1], G. Mistelbauer[1], *J. Hop*[2], A. M. H. Sailer[1], V. Turner[1], V. Hinostroza[1], D. Mastrodicasa[1], D. Fleischmann[1]; [1]Stanford, CA/US, [2]Groningen/NL
*(j.f.hop@umcg.nl)*

**Purpose:** A recent study showed that the circumferential angle of the false lumen (FL) is independently associated with late adverse events (LAEs) in patients with uncomplicated type-B aortic dissection (uTBAD), even when added to a multivariable model that includes maximum aortic diameter. Although these results are promising, the circumferential-angle of the FL is difficult to measure. We aimed to assess whether the easy-to-measure FL-arc-length has the potential to replace circumferential angle and maximum aortic diameter for LAEs prediction in uTBAD-patients.
**Methods or Background:** uTBAD-patients who underwent a CTA≤2 weeks after dissection were retrospectively included. The conventional Cox regression model included 1 clinical feature (presence of connective tissue-disease (CTD)) and 4 imaging features: FL-circumferential-angle, maximum aortic diameter, total FL-outflow volume, and number of identifiable intercostals. Estimated FL-arc-length was calculated as(Max_Aortic_diameter*pi*False_lumen_circumferential_angle)/360. LAE was defined as aortic aneurysm formation, rapid aortic growth, aortic rupture, branch vessel ischemia, or death. Multivariable Cox-regression models were created with estimated FL-arc-length.
**Results or Findings:** 83 patients (mean age 54±16 years) were included and 14 (15%) patients had CTD. Median follow-up was 850 days (interquartiles 247-1824) and 33 (40%) patients developed LAEs. The conventional multivariable Cox-model showed hazard ratios (HRs) of 1.10(1.02-1.18), p=0.015 for maximum aortic diameter and 1.03(1.01-1.05), p=0.003 for FL-circumferential-angle. The multivariable model was updated by replacing maximum aortic diameter and FL-circumferential-angle with estimated FL-arc-length, resulting in a HR of 1.05(1.03-1.08)/mm, p<0.001. Model performance was similar with area under the curves of 0.738(0.632-0.844) for the original model and 0.733(0.627-0.839) for the updated model.
**Conclusion:** Our multicenter study showed that estimated FL-arc-length is significantly associated with LAEs and has the potential to replace FL-circumferential-angle and maximum aortic diameter in risk prediction of uTBAD-patients.
**Limitations:** Retrospective study; FL-arc-length was estimated;
**Ethics Committee Approval:** IRB-approved
**Funding for this study:** American Heart Association
**Author Disclosures:**
Dominik Fleischmann: Research/Grant Support at Siemens Healthineers
Virginia Hinostroza: Nothing to disclose
Marina Codari: Nothing to disclose
Gabriel Mistelbauer: Grant Recipient at cool IT GmbH
Valery Turner: Shareholder at Segmed, Inc
Anna M. H. Sailer: Nothing to disclose
Martin J. Willemink: Grant Recipient at Philips Healthcare Consultant at Arterys, Inc, Shareholder at Segmed, Inc
Domenico Mastrodicasa: Nothing to disclose
Joost Frederik Hop: Nothing to disclose

### RPS 115-7

**CT-derived pulmonary vascular metrics predict COVID-19 patients' prognosis**

S. Schiaffino[1], A. Cozzi[1], *M. Codari*[2], C. Bnà[3], M. Cariati[1], A. Carriero[4], A. Vanzulli[1], L. M. Sconfienza[1], F. Sardanelli[1]; [1]Milan/IT, [2]Stanford, CA/US, [3]Brescia/IT, [4]Novara/IT
*(mcodari@stanford.edu)*

**Purpose:** To evaluate the prognostic significance of CT-derived vascular and lung parenchymal features combined with clinical data acquired at emergency department (ED) admission of COVID-19 patients.
**Methods or Background:** Patients with RT-PCR-confirmed SARS-CoV-2 infection and unenhanced chest CT performed on ED admission from six hospitals were included, their outcome (discharge or death) being retrieved alongside clinical data (demographics, comorbidities, laboratory examinations), chest CT features (parenchymal involvement extent/progression, crazy paving, pleural effusion, lymphadenopathies), and CT-derived vascular metrics (pulmonary artery and ascending aorta diameters). Univariate analysis (Mann-Whitney U test), support vectors machines (SVM) and neural networks (NN) were used to build a mortality-predicting model, with a 85%:15% training-validation/test dataset split.
**Results or Findings:** Out of 897 patients (68% males, median age 66 years, interquartile range [IQR] 55–77 years), 229 (26%) patients died after a median 7 days hospitalization (IQR 4–11), dead patients showed higher pulmonary artery diameter than discharged patients (29.0 versus 27.0 mm, p<0.001), and higher median aortic diameter (36.6 mm versus 34.0 mm, p < 0.001). We used 10 input features selected after least absolute shrinkage and selection operator regression (sex, age, pulmonary consolidations, crazy paving pattern, parenchymal involvement extent, pulmonary artery and aortic diameters, pre-existent cardiovascular disease, oncological history, chronic kidney insufficiency) to build our mortality predictive model. Best predictive model was obtained using NN and yielded an area under the curve of and 0.844 (precision 0.680, recall 0.567) in the testing set. Pulmonary artery diameter was the third most important predictor after age and parenchymal involvement extent.
**Conclusion:** Pulmonary artery maximum diameter showed significant difference between discharged and dead patients, sizably contributing to prognosis-predictive model
**Limitations:** Retrospective study performed during a pandemic peak.
**Ethics Committee Approval:** Obtained for each center.
**Funding for this study:** Partial support by the Italian Ministry of Health.
**Author Disclosures:**
Andrea Cozzi: Nothing to disclose
Claudio Bnà: Nothing to disclose
Francesco Sardanelli: Advisory Board at Bracco Group, Advisory Board at Bayer Healthcare, Speaker at General Electric Healthcare
Simone Schiaffino: Other at Bracco Imaging, Speaker at General Electric Healthcare
Marina Codari: Nothing to disclose
Angelo Vanzulli: Nothing to disclose
Luca Maria Sconfienza: Nothing to disclose
Maurizio Cariati: Nothing to disclose
Alessandro Carriero: Nothing to disclose

### RPS 115-9

**Preoperative cerebral small vessel disease and carotid near-occlusion are independently associated with cerebral hyperperfusion after carotid endarterectomy**

*X. Fan*[1], Z. Lai[1], T. Lin[1], H. You[1], J. Wei[1], Z. Li[2], F. Feng[1]; [1]Beijing/CN, [2]Shanghai/CN
*(18801122923@163.com)*

**Purpose:** To determine whether preoperative cerebral small vessel disease (SVD) and carotid near-occlusion are associated with cerebral hyperperfusion (CH) after carotid endarterectomy (CEA). Another purpose was to explore the risk factors for the further development of cerebral hyperperfusion syndrome (CHS) in patients diagnosed with CH.
**Methods or Background:** Consecutive patients with carotid stenosis who underwent CEA between May 2015 and September 2020 were included. CH was defined as a cerebral blood flow increase of more than 100% compared with preoperative values on arterial spin labelling images. The grade or the number of four cerebral SVD markers (white matter hyperintensities(WMHs), lacunes, perivascular spaces and cerebral microbleeds) was evaluated based on preoperative MRI. The presence of carotid near-occlusion was defined by computed tomography angiography.
**Results or Findings:** A total of 50 patients were included and CH was observed in 12 patients(24%). Logistic regression analysis revealed that WMHs (OR=2.46, 95% CI [1.36-4.45], p=0.003), lacunes (OR=1.76, 95% CI [1.17, 2.64], p=0.007) and carotid near-occlusion (p<0.01) were independently associated with postoperative CH. CHS was occurred in 5(41.67%) of the 12 patients diagnosed with CH. The maximum systolic blood pressure within 24h after surgery of 5 CHS patients was significantly higher than that of the other 7 patients (p=0.045).
**Conclusion:** In patients with carotid stenosis, preoperative WMHs, lacunes and carotid near-occlusion can identify patients at risk of CH after CEA. Strict control of blood pressure might be the key point to prevent the progression of CHS.
**Limitations:** First, the sample size was relatively small. Secondly, our conclusions were specifically appliable to patients undergoing CEA under general anesthesia.

**Ethics Committee Approval:** This study was approved by the Medical Ethics Committee of the Peking Union Medical College Hospital.
**Funding for this study:** This study was funded by the Beijing Natural Science Foundation grant (L182067).
**Author Disclosures:**
Feng Feng: Nothing to disclose
Zhichao Lai: Nothing to disclose
Juan Wei: Nothing to disclose
Xiaoyuan Fan: Nothing to disclose
Zheng Li: Nothing to disclose
Hui You: Nothing to disclose
Tianye Lin: Nothing to disclose

**Live Q&A**

**15:30-16:30 CHANNEL 2**

### Refresher Course: Chest

### RC 404

### Thoracic manifestations of COVID-19

**Moderator**
Annemiek Snoeckx; Antwerp/BE

### RC 404-2
**A. Acute findings and late sequelae**
Nicola Sverzellati; Parma/IT

**Learning Objectives:**
1. To recognise acute findings on radiographs and CT.
2. To evaluate late findings on CT.
3. To gain confidence in identifying imaging patterns, even when PCR is negative.

### RC 404-3
**B. COVID-19: a vascular pulmonary disease**
Marie-Pierre Revel; Paris/FR
*(marie-pierre.revel@aphp.fr)*

**Learning Objectives:**
1. To learn about pulmonary vascular injury and thrombosis in COVID-19.
2. To understand the evolution of vascular pathology in the lungs.
3. To learn about optimal imaging protocols for patients in intensive care.
**Author Disclosures:**
Marie-Pierre Revel: Other: travel support from guerbet, lecture fee from MSD

### RC 404-4
**C. COVID-19 in patients with pre-existing lung diseases**
Thomas Frauenfelder; Zurich/CH

**Learning Objectives:**
1. To distinguish current pathology from pre-existing lung anomalies.
2. To understand how underlying lung disease affects prognosis in COVID-19.
3. To know when to plan follow-up after COVID-19.

**Live Q&A**

**15:30-16:30 CHANNEL 3**

### EFRS Workshop

### EFRS WS

### Radiographers and patient-centred care

**Moderators**
Charlotte Graungaard Falkvard; Frederiksberg/DK
Erik Briers; Hasselt/BE

### EFRS WS-3
**What constitutes patient-centred care in medical imaging?**
Rodrigo García Gorga; Sabadell/ES

**Learning Objectives:**
1. To define the keys concepts of patient-centred care.
2. To outline the potential benefits of patient-centred care for patients.
3. To identify the challenges and opportunities for successfully implementing patient-centred care.

### EFRS WS-4
**Successfully achieving optimal patient experience within medical imaging**
Louise Harding, Paula Park; Warrington/UK
*(louise.harding1@nhs.net), (paula.park@nhs.net)*

**Learning Objectives:**
1. To understand how implementing a quality improvement methodology can assist in improving radiographic practice and team work.
2. To appreciate how patient centred care can impact on radiographic practice and patient experience.
3. To understand how radiography research can translate into implementing changes in clinical practice.

### EFRS WS-6
**What patients require from their experience within a radiology department**
Cheryl Cruwys; Glanges/FR

**Learning Objectives:**
1. To appreciate the patient's requirements when attending imaging examinations.
2. To consider how patients can contribute to implementing more patient-centred approaches.
3. To identify the future challenges and opportunities for delivering patient-centred care within medical imaging.
**Author Disclosures:**
Cheryl Cruwys: Founder: Breast Density Matters UK

### EFRS WS-7
**What can the EFRS do at a European level to promote patient-centred care?**
Andrew England; Salford/UK
*(a.england@keele.ac.uk)*

**Learning Objectives:**
1. To outline the role of the EFRS in promoting patient-centred care.
2. To explain the EFRS initiatives for promoting patient-centred care across Europe.
3. To appreciate other opportunities for developing and promoting patient-centred care within individual organisations.

**Live Q&A: Patient-centred care is not a barrier to improving departmental efficiency**

**15:30-16:30 CHANNEL 4**

### Coffee & Talk (open forum) Session
*Organised by EIBIR*

### C 12

### Innovations in breast imaging: keys to funding success

**Moderator**
Gabriel P. Krestin; Rotterdam/NL

### C 12-2
**Innovation in the diagnosis of breast cancer using ultrasound and diffuse optics**
Pietro Panizza; Milan/IT

**Learning Objectives:**
1. To learn about the developments of the SOLUS research project.
2. To learn about and understand the benefits of combined use of ultrasound and optical tomography.

### C 12-3
**Digital hybrid breast PET/MRI for enhanced diagnosis of breast cancer**
Thomas H. Helbich; Vienna/AT

**Learning Objectives:**
1. To learn about the development of the HYPMED research project.
2. To learn about and understand the benefits of digital hybrid PET/MR in breast cancer imaging.

**Author Disclosures:**
Thomas H. Helbich: Grant Recipient: bracco, guerbet, siemens, hologic, bard, novomed

### C 12-4
**The impact of breast MRI on surgical planning and reoperation rate**
Francesco Sardanelli; San Donato Milanese/IT
*(francesco.sardanelli@unimi.it)*

**Learning Objectives:**
1. To learn about the MIPA clinical study.
2. To understand the impact of breast MRI on surgical planning and reoperation rates.

**Author Disclosures:**
Francesco Sardanelli: Advisory Board: Bayer, Bracco, General Electric; Equipment Support Recepient: General Electric

### C 12-5
**Funding opportunities under Horizon Europe**
Peter Gordebeke; Vienna/AT
*(pgordebeke@eibir.org)*

**Learning Objectives:**
1. To learn about Horizon Europe.
2. To learn about European research funding opportunities.

### C 12-6
**EIBIR's support services: key for funding success**
Pamela Zolda; Vienna/AT
*(pamela.zolda@myesr.org)*

**Learning Objectives:**
1. To learn about EIBIR's support services.
2. To understand how EIBIR can increase funding success.

**Live Q&A**

**15:30-16:30** **CHANNEL 5**

### Student Session

### S 1

### Best of ECR Student Abstract Submission

**Moderator**
Dragos Negru; Iasi/RO

### S 1-2
**Dynamic changes of the functional connectome during foetal brain development observed in utero via MRI**
*A. Taymourtash*, E. Schwartz, K-H. Nenning, D. Prayer, G. Kasprian, G. Langs; Vienna/AT
*(athena.taymourtash@meduniwien.ac.at)*

**Purpose:** A comprehensive quantitative analysis of functional foetal brain development in-utero, can advance the study of pathologies without the confounding effects of postnatal experiences, allowing for early prevention, detection, and intervention starting even before birth. In this work, we model foetal functional connectome development with a focus on thalamo-cortical connectivity derived from in-utero MRI.
**Methods or Background:** Resting-state fMRI were acquired from 72 singleton fetuses between 19 and 39 weeks of gestation. We addressed the technical challenges inherent to in-utero imaging such as motion contamination, signal non-uniformity, and low SNR with established image processing methods. Subject-specific functional connectivity maps were obtained by correlating the average time-course of the BOLD signal within cortical ROIs. After computational quality control of the resulting matrices, we excluded 24 subjects and modelled the longitudinal development of functional connectivity with a linear and a sigmoid model. The accuracy of the models was evaluated via bootstrapping over 1000 iterations.
**Results or Findings:** We found a significant increase of functional thalamo-cortical connectivity after 28th GW compared to before 26th GW, consistent with prior work regarding the invasion and penetration of the thalamocortical afferents into the cortical plate (mean Adjusted-R2: linear=0.175; sigmoid=0.148). The development of cortico-cortical connectivity was symmetric and the functional symmetry of the brain was decreased with increasing gestational age ($P<0.01$)
**Conclusion:** Recent advancements in functional MRI enabled the examination of functional connectivity development of a human fetus, in-utero. However, optimization of the imaging sequences and meticulous post-processing including mitigation of motion-related artifacts and age-specific segmentation were necessary to link FC measures to the developmental changes of the foetal brain.
**Limitations:** We compared two predefined models for the functional connectome development, and only healthy subjects were included.
**Ethics Committee Approval:** Yes.
**Funding for this study:** European Union's Horizon 2020 grant agreement No.765148.
**Author Disclosures:**
Mister Karl-Heinz Nenning: Nothing to disclose
Georg Langs: Nothing to disclose
Athena Taymourtash: Nothing to disclose
Ernst Schwartz: Nothing to disclose
Daniela Prayer: Nothing to disclose
Gregor Kasprian: Nothing to disclose

### S 1-4
**Quantification of peak blood flow velocity by four-dimensional flow and two-dimensional phase-contrast MRI compared with echocardiography: a meta-analysis**
*K. Xu*, Y-K. Guo; Chengdu/CN
*(382781403@qq.com)*

**Purpose:** Accurate measurement of peak blood velocity is required for the clinical evaluation, diagnosis, and therapeutic management of cardiovascular diseases. This meta-analysis aimed to objectively examine the agreement and correlation between four-dimensional (4D) flow magnetic resonance imaging (MRI) and traditional two-dimensional (2D) phase contrast (PC) MRI with the reference standard of echocardiography (echo) for measuring peak blood velocity in the cardiac valve and great arteries and assess if this technology offers an advantage over the traditional 2D method.
**Methods or Background:** We systematically searched for studies that evaluate the degree of correlation and agreement between 4D flow MRI or 2D PC MRI and echo retrieved from PubMed, Embase and Cochrane Library. We conducted a meta-analysis to determine the peak velocity pooled bias with 95% limits of agreement (LoA) and correlation coefficient (r) for 4D flow MRI and 2D PC MRI compared with echo.
**Results or Findings:** Ten studies that compared 4D flow MRI with echo and twelve studies that compared 2D PC MRI with echo were included. 4D flow MRI showed an underestimation with bias and 95% LoA of –0.09 (–0.41, 0.23) m/s while 2D PC MRI showed a poorer agreement with an underestimated bias and 95% LoA of -0.25 (-0.53, 0.03). 4D flow MRI and 2D PC MRI showed a strong correlation with $R = 0.80$ (95% CI 0.75, 0.84 ) $p < 0.001$ and $R = 0.83$ (95% CI 0.79, 0.87) $p < 0.001$, respectively
**Conclusion:** 4D flow MRI potentially provides improved assessment for peak velocity compared with traditional 2D methods and can therefore be considered an important complement to echo in the clinic with high agreement, particularly in those patients with complex blood flow patterns.
**Limitations:** None
**Ethics Committee Approval:** Institutional Review Board approval was not required
**Funding for this study:** None
**Author Disclosures:**
Ke Xu: Nothing to disclose
Ying-Kun Guo: Nothing to disclose

### S 1-5
**Perioperative deep learning assessment of kidneys and renal tumours**
J. Glavis-Bloom, T-L. Bui, *E. Lebby*, A. Sasani, M. bardis, C. Chahine, S. Cramer, P. Chang, R. Houshyar; Orange/US
*(elebby@hs.uci.edu)*

**Purpose:** Partial nephrectomy and percutaneous ablation, increasingly utilized to treat small renal masses, require precise preoperative imaging interpretation. We sought to develop and evaluate a convolutional neural network (CNN), a type of deep learning artificial intelligence, that can accurately determine renal tumour and kidney volumes via segmentation on single-phase computed tomography (CT).

**Methods or Background:** CT images of 319 patients were retrospectively analyzed. Two distinct CNNs were developed for (1) bounding cube localization of the right and left hemi-abdomen and (2) segmentation of renal parenchyma and tumour within each bounding cube. Training was performed on a randomly selected cohort of 269 patients. CNN performance was evaluated on the remaining 50 patients using Sorensen–Dice coefficients and Pearson correlation coefficients. Experiments were performed on a GPU-optimized workstation with a single NVIDIA GeForce GTX Titan X (12GB, Maxwell architecture).
**Results or Findings:** Median Dice coefficients for kidney and tumour segmentation were 0.970 and 0.816, respectively; Pearson correlation coefficients between CNN-generated and human-annotated estimates for kidney and tumour volume were 0.998 and 0.993 ($p < 0.001$), respectively. End-to-end trained CNNs were able to perform renal parenchyma and tumour segmentation on a new test case in an average of 5.6 seconds.
**Conclusion:** Deep learning is capable of rapidly and accurately detecting and segmenting kidneys and renal tumours on single-phase contrast-enhanced CT scans and calculating tumour and renal volumes.
**Limitations:** Our CNN's performance was affected by inclusion of many kidneys with complex cysts, some of which may have been misinterpreted as solid renal lesions.
**Ethics Committee Approval:** The University of California, Irvine Institutional Review Board approved this study.
**Funding for this study:** Cancer Research Trainee Grant, Department of Radiology, University of California Irvine School of Medicine
**Author Disclosures:**
Michelle bardis: Nothing to disclose
Ali Sasani: Nothing to disclose
Elliott Lebby: Nothing to disclose
Scott Cramer: Nothing to disclose
Chantal Chahine: Nothing to disclose
Roozbeh Houshyar: Nothing to disclose
Justin Glavis-Bloom: Nothing to disclose
Peter Chang: Shareholder at Avicenna.ai, a medical imaging startup, Founder at Avicenna.ai, a medical imaging startup
Thanh-Lan Bui: Nothing to disclose

### S 1-7

### Opinion of medical students regarding the current medical teaching methodology and trends in medical research practice in Romania: results and implications for the field of radiology

*R. C. Anicai*[1], M-A. Gaman[2]; [1]Iasi/RO, [2]Bucharest/RO
*(anicairobert98@gmail.com)*

**Purpose:** To our knowledge, med-students' opinions regarding the current medical teaching methodology/ research trends in Romania have not been assessed so far.
**Methods or Background:** Cross-sectional, multicentric study. Using a social-media platform, we distributed a survey during 01/09/2020-31/10/2020 to med-students from 11 Romanian universities. Calculated sample size: 376 respondents (population=15000; 95% CI; 5% margin of error).
**Results or Findings:** 400 med-students responded: 301(75.25%) females, 99(24.75%) males (P-value<0.001), 233(58.25%) enrolled in preclinical (2nd/3rd) and 167(41.75%) in clinical years (4th/5th/6th)[P-value<0.001]. 55.0%(N=220) evaluated the teaching system at 3/5. 40.75%(N=163) believed the biggest drawback of med-school is the exaggerated focus on theoretical knowledge versus practical aspects. 79.17%(N=316) viewed attending lectures as useless if they only listened to presentation slides being read without any interaction with the lecturers. If faced with the decision to reapply to med-school, 43.75%(N=175) would choose another field. 62.25%(N=250) said optimal practical/laboratory classes should last 50-100 minutes. 55.25%(N=221) reported that <10 students should partake in a lab simultaneously. 88.00%(N=352) considered practical examples/training the best way of learning. 49.25%(N=197) believed med-school focuses too much on memory. 38.50%(N=154) thought med-schools could have done more to adapt the teaching system to the students' needs and 49.75%(N=199) considered their training was deficient during the COVID-19 pandemic. 50.50%(N=202) thought research is important, 42.75%(N=171) believed there is a medium level of student engagement in research, 52.75%(N=211) answered that physicians should engage in research in addition to clinical duties only if wishing to pursue academic careers. 34.25%(N=137) of the respondents want to pursue a career in Romania. Implications/responses regarding the teaching of Radiology will be presented at ECR-2021.
**Conclusion:** Universities should aim to improve the current teaching methodology and involve med-students in research, including in Radiology.
**Limitations:** Not applicable.
**Ethics Committee Approval:** Not applicable.
**Funding for this study:** Not applicable.
**Author Disclosures:**
Robert Constantin Anicai: Nothing to disclose
Mihnea-Alexandru Gaman: Nothing to disclose

### S 1-8

### Can we stimulate medical students' evidence-based and cost-effective diagnostic reasoning in radiology? Impact of a Choosing Wisely approach

*C. V. Souza Freire*, G. F. Saffe, M. E. R. d. A. D. d. Pinho, M. E. B. De Siervi, C. Freitas Lins; Salvador/BR
*(camilafreire17.2@bahiana.edu.br)*

**Purpose:** To assess a Choosing Wisely (CW) approach complementary to the teaching of Radiology as a strategy of early incentive to evidence-based and cost-effective reasoning in medical curriculum.
**Methods or Background:** CW approach was developed in parallel with Radiology classes for second-year medical students by student mentors of radiology, who are more advanced students previously trained by a Radiology professor. The chosen recommendations were inserted through clinical problems in theoretical classes by the professor and in reviews by mentors. After case discussion and following steps to diagnosis, feedback guided by best evidence and cost-effective strategies was provided. One semester later, students were invited to answer an evaluation questionnaire. Written informed consent was obtained from all subjects.
**Results or Findings:** 148 students (21±3 years, 59% female) participated in the study. 94% considered the approach "above average" or "excellent" to its purpose. 94% considered their interest "above average" or "excellent" in the proposed activities. 84% rated its efficiency as "above average" or "excellent". 96% defined it as "above average" or "excellent" as a didactic tool. 98% considered the approach "above average" or "excellent" to assist them in reflecting on cost-effective diagnostic reasoning. Additionally, 77% rated the CW approach "above average" or "excellent" on contributing to their performance on radiology exams.
**Conclusion:** CW approach was highly well-rated by students since it encourages the development of early critical and conscious diagnostic reasoning. This approach has the potential to generate future physicians who make wiser evidence-based and cost-effective decisions regarding imaging diagnosis.
**Limitations:** This is a cross-sectional study; further investigation is required to measure the longitudinal impact of this strategy.
**Ethics Committee Approval:** Ethical approval was obtained from the Ethics Committee of the University where this study was developed (CAAE: 28196820.6.0000.5544).
**Funding for this study:** This research received no external funding.
**Author Disclosures:**
Maria Eduarda Rodrigues de Araújo Dantas de Pinho: Nothing to disclose
Gabriela Fagundes Saffe: Nothing to disclose
Maria Eduarda Barreto De Siervi: Nothing to disclose
Carolina Freitas Lins: Nothing to disclose
Camila Verônica Souza Freire: Nothing to disclose

### S 1-9

### Brain effects of transcutaneous vagal nerve stimulation: a meta-analysis of neuroimaging evidence

*R. Rajiah*, K. Takahashi, Q. Aziz, J. Ruffle; London/UK
*(r.rajiah1@uni.bsms.ac.uk)*

**Purpose:** Dysfunction of the autonomic nervous system is placed at the centre of disease pathophysiology for many conditions spanning numerous body systems. Considering the pivotal role of the vagus nerve to the brain's central autonomic network, transcutaneous vagal nerve stimulation (tVNS) has been proposed as a novel therapeutic intervention to restore disease-driven autonomic disruption. However, the effect of tVNS on the brain remains unclear - a significant rate limiting step in its prospective use. We aimed to identify the reproducible neural correlates of tVNS by meta-analysis.
**Methods or Background:** A total of 157 studies were identified from the Web of Science and PubMed databases, 4 of which were appropriate for neuroimaging-based statistical analysis, comprising 60 subjects (17 male, 27 female, gender in 16 subjects not provided) aged 18-65. Using activated likelihood analysis estimation, we established brain activity changes from tVNS statistically contrasted to both null and sham stimulation.
**Results or Findings:** tVNS consistently increased activity in the bilateral insula, left thalamus, bilateral frontal pole, bilateral anterior cingulate cortex, right putamen, bilateral caudate and bilateral central opercular cortex and reduced activity in the bilateral parahippocampal gyri, bilateral hippocampi, bilateral temporal occipital fusiform gyrus and right posterior cingulate cortex (all $p<0.005$ or lower).
**Conclusion:** We provide the first meta-analytic map of tVNS brain effects by coalescence of all relevant prior brain imaging in healthy subjects and illustrate that numerous brain regions demonstrate a change in their activity sequential to tVNS. Considering the functional importance of these brain structures in regulating emotion, pain processing and autonomic activity, these findings could provide a physical correlate for the therapeutic effects observed in prior research. Further exploration to better elucidate these mechanisms is warranted.

**Limitations:** Limited number of studies were used.
**Ethics Committee Approval:** Not applicable.
**Funding for this study:** Not applicable.
**Author Disclosures:**
Kazuya Takahashi: Nothing to disclose Nothing to disclose
essor Qasim Aziz: Nothing to disclose Nothing to disclose
Rebekah Rajiah: Nothing to disclose Nothing to disclose
James Ruffle: Nothing to disclose Nothing to disclose

**Live Q&A**

**16:45-17:45** **CHANNEL 2**

### New Horizons Session

### NH 3

### Thera(g)nostics

**Moderator**
Clemens C. Cyran; Munich/DE

### NH 3-2

**Thera(g)nostics: what is it all about?**
Frederik L. Giesel; Heidelberg/DE

**Learning Objectives:**
1. To become familiar with basic hypotheses of thera(g)nostics.
2. To understand the underlying mechanisms of target selection for imaging and therapy.
3. To know about the most relevant clinical fields for theragnostic concepts.
4. To learn about the key translational challenges of thera(g)nostics towards clinical care.

### NH 3-4

**Molecular biomarkers for immuno-imaging**
Elisabeth de Vries; Groningen/NL
*(e.g.e.de.vries@umcg.nl)*

**Learning Objectives:**
1. To learn about the concept of imaging biomarkers.
2. To become familiar with the basics of immuno-imaging.
3. To know about the most relevant clinical applications of immuno-imaging.
4. To understand the main challenges and limitations of immuno-imaging for clinical translation.

**Author Disclosures:**
Elisabeth de Vries: Advisory Board: Sanofi, Daiichi Sankyo, NSABP, Pfizer, Merck (Institutional financial support for advisory boards); Research Grant/Support: Amgen, Genentech, Roche, AstraZeneca, Synthon, Servier, Nordic Nanovector, G1 Therapeutics, Bayer, Chugai Pharma, CytomX Therapeutics, Radius Health, Regeneron, Servier (Institutional Financial support for clinical trials or contracted research); Other: (Co)-chair RECIST committee, Member ESMO-MCBS Working Group, Chair ESMO Cancer Medicines Committee, Member Royal Netherlands Academy of Arts & Sciences, Member supervisory board Netherlands Comprehensive Cancer Organisation (Non-financial interests, including non remunerated activities)

**Live Q&A: Does hybrid imaging improve patient outcome?**

**16:45-17:45** **CHANNEL 4**

### E³ - ECR Master Class

### E³ 426

### Autoimmune thoracic diseases

**Moderator**
Sujal R. Desai; London/UK

### E³ 426-2

**A. Relapsing polychondritis**
Walter de Weever; Leuven/BE
*(walter.dewever@uzleuven.be)*

**Learning Objectives:**
1. To learn about the pathophysiology and clinical manifestations of this disease.
2. To learn when to suggest this diagnosis on CT.
3. To review the other causes of tracheobronchial wall thickening.

### E³ 426-3

**B. Alveolar proteinosis**
Anastasia Oikonomou; Toronto, ON/CA
*(anastasia.oikonomou@sunnybrook.ca)*

**Learning Objectives:**
1. To learn about the CT manifestations of alveolar proteinosis.
2. To review the other causes of crazy paving on CT.
3. To be aware of infectious complications, especially superinfection by Nocardia.

### E³ 426-4

**C. Goodpasture syndrome and differentials**
Anand Devaraj; London/UK

**Learning Objectives:**
1. To learn about the pathophysiology and the clinical manifestations of this disease.
2. To learn about CT signs of intra-alveolar haemorrhage.
3. To review the other causes of intra-alveolar haemorrhage.

**Author Disclosures:**
Anand Devaraj: Advisory Board: Boehringer Ingelheim; Employee: Brainomix

**Live Q&A**

**16:45-17:45** **CHANNEL 5**

### Research Presentation Session: Neuro

### RPS 211

### Infections in neuroradiology

**Moderator**
Majda M. Thurnher; Vienna/AT

### RPS 211-2

**Neurological imaging manifestations in COVID-19 patients**
*M. M. A. Rezk*[1], H. E. Elsikhry[2], K. M. Zaki[2], I. AboShady[2], B. Alkandari[2]; [1]Cairo/EG, [2]Kuwait/KW
*(medical1430@yahoo.com)*

**Purpose:** To figure out the neuroradiological findings in COVID-19
**Methods or Background:** A retrospective study include adult hospitalized patients with PCR-positivity for SARS-CoV-2, undergoing neuroimaging between March and November, 2020, were included. All examinations were systematically re-evaluated by 3 readers then descriptive statistics were calculated.
**Results or Findings:** 300 patients with COVID-19 underwent neuroimaging. Frequent findings include intra-axial susceptibility abnormalities, ischemic and macrohemorrhagic manifestations, olfactory bulb signal abnormalities, prominent perioptic nerve spaces, and enhancement of the parenchyma, leptomeninges, cranial nerves and spinal nerves .
**Conclusion:** Patients with COVID-19 had a wide spectrum of vascular and inflammatory involvement of both the central and peripheral nervous system.
**Limitations:** Subject to systematic confounders.
The lack of a control group.
**Ethics Committee Approval:** Yes
**Funding for this study:** No
**Author Disclosures:**
Hisham Elsayed Elsikhry: Nothing to disclose
Islam AboShady: Nothing to disclose
Kamal Mahmoud Zaki: Nothing to disclose
Mahmoud M. A. Rezk: Nothing to disclose
Buthaina Alkandari: Nothing to disclose

### RPS 211-3

**Adapting the UK biobank brain MRI protocol and analysis pipeline to multi-organ imaging of COVID-19 survivors**
*L. Griffanti*, M. Cassar, B. Raman, N. Filippini, F. Alfaro-Almagro, F. Sheerin, S. Neubauer, K. L. Miller, S. M. Smith; Oxford/UK

**Purpose:** To adapt the UK Biobank brain MRI acquisition protocol and analysis pipeline in order to evaluate the effects of COVID-19 as part of a multiorgan assessment.
**Methods or Background:** SARS-CoV-2 infection has been shown to damage multiple organs, including the brain. Multiorgan MRI can provide further insight on the repercussions of COVID-19 on organ health, but requires balance between data acquisition and scan duration. We adapted the UK Biobank brain MRI protocol to produce high quality images while being suitable as part of a multiorgan MRI exam. The analysis pipeline includes new imaging-derived phenotypes (IDPs) designed to assess the effects of COVID-19. This was performed in 58 COVID-19 patients post-hospital discharge and 25 controls in the C-MORE study.
**Results or Findings:** The protocol acquires high resolution T1, T2-FLAIR, diffusion weighted images, susceptibility weighted images and arterial spin labelling in 17 minutes. The automated imaging pipeline derives 263 IDPs, assessing brain anatomy (including olfactory bulb volume and intensity), perfusion and microstructural integrity. In the C-MORE study, these quantitative measures were consistent with clinical radiology reports. Our exploratory analysis revealed an increased burden of white matter hyperintensities and reduced mean diffusivity in the posterior thalamic radiations and sagittal stratum of COVID-19 survivors. These differences were more prominent in patients who received organ support.
**Conclusion:** The protocol is now used in the ‘PHOSP-COVID Post-hospitalisation COVID-19’ study: a UK national consortium to understand and improve long-term health outcomes. The pipeline is widely applicable and will help us understand the short and long-term effects of COVID-19.
**Limitations:** Small sample size, exploratory analysis.
**Ethics Committee Approval:** Ethics approval and consent from all participants were obtained.
**Funding for this study:** NIHR Oxford and Oxford Health Biomedical Research Centre, Oxford British Heart Foundation Centre of Research Excellence, UK Research Innovation and Wellcome Trust.
**Author Disclosures:**
Karla L. Miller: Nothing to disclose
Stefan Neubauer: Patent Holder at Co-author of the patents for calculating T1 (ShMOLLI) in the heart, liver and abdomen: US patent 61/630,508 licensed to Perspectum and US patent 61-630,510 licensed to Perspectum. Shareholder in Perspectum. SN was a board member and consultant to Perspectum until 2019.
Fidel Alfaro-Almagro: Nothing to disclose
Betty Raman: Nothing to disclose
Stephen M. Smith: Nothing to disclose
Mark Cassar: Nothing to disclose
Fintan Sheerin: Nothing to disclose
Nicola Filippini: Nothing to disclose
Ludovica Griffanti: Nothing to disclose

### RPS 211-4

### In vivo imaging of microglia activation using proton magnetic resonance spectroscopy in immunocompetent patients with acute toxoplasmosis: preliminary results

*A. Garkowski*[1], B. Kubas[1], M. Hładuński[1], J. Zajkowska[1], A. Moniuszko-Malinowska[1], O. Zajkowska[2], M. Kondrusik[1], U. Lebkowska[1]; [1]Białystok/PL, [2]Warsaw/PL
*(adam.garkowski@gmail.com)*

**Purpose:** It is alleged that the healthy person's immune system keeps the parasite Toxoplasma gondii (T. gondii) latent from causing illness, and it is commonly said that T. gondii infection is either asymptomatic or cause mild, self-limited illness in the immunocompetent people. However, many recent epidemiological studies suggest that in immunocompetent persons T. gondii infection is associated with a range incidence of neuropsychiatric disorders. However, there have not been any studies assessing the impact of T. gondii infection in immunocompetent patients on the central nervous system (CNS) using imaging techniques. We aimed to assess the impact of acute T. gondii infection on the CNS in immunocompetent persons using proton magnetic resonance spectroscopy (1H-MRS).
**Methods or Background:** Thirteen patients with an acute toxoplasmosis and eight healthy volunteers (control group, with excluded T. gondii infection) have been involved in the study. 1H-MRS examinations were performed with repetition time (TR) = 2000 ms, and echo time (TE) = 30 ms (3.0-T MRI scanner). Single voxels were positioned bilaterally in the basal ganglia, frontal, temporal and occipital lobes.
**Results or Findings:** We found a statistically significant increase of the mI/Cr ratio within the right frontal lobe (p=0.024), right basal ganglia (p=0.019) and in the left temporal lobe (p=0.043) in the patients with acute toxoplasmosis. We also observed significantly reduced Glx/Cr ratio in the left occipital lobe (p=0.033) and reduced NAA/Cr ratio within right (p=0.032), and left basal ganglia (p=0.001).
**Conclusion:** These metabolie alterations in patients with acute toxoplasmosis suggest the presence of microglia activation with neuronal dysfunction, and additionally glutamine-glutamate dysregulation.
**Limitations:** The sample size of patients in our study was small, which may affects the statistical results.
**Ethics Committee Approval:** Ethics committee approval obtained
**Funding for this study:** Medical University of Białystok
**Author Disclosures:**
Anna Moniuszko-Malinowska: Nothing to disclose
Bożena Kubas: Nothing to disclose
Joanna Zajkowska: Nothing to disclose
Olga Zajkowska: Nothing to disclose
Marcin Hładuński: Nothing to disclose
Maciej Kondrusik: Nothing to disclose
Urszula Lebkowska: Nothing to disclose
Adam Garkowski: Nothing to disclose

### RPS 211-6

### The impact of the COVID-19 pandemic on adult diagnostic neuroradiology in Europe

*M. Smits*[1], M. W. Vernooij[1], N. Bargallo I Alabart[2], A. Ramos Gonzalez[3], T. A. Yousry[4]; [1]Rotterdam/NL, [2]Barcelona/ES, [3]Madrid/ES, [4]London/UK
*(marion.smits@erasmusmc.nl)*

**Purpose:** The purpose of this study was to understand the impact the Covid-19 pandemic has or has had on the work, training, and wellbeing of professionals in the field of diagnostic Neuroradiology. These insights may be used to prepare or improve strategies for similar situations and address potential needs and worries that arose from the crisis.
**Methods or Background:** A survey (SurveyMonkey) was emailed to all ESNR members and associates as well as distributed via professional social media channels. The survey was held in the Summer of 2020 when the first wave had subsided in most of Europe while the second wave was not yet widespread. The questionnaire featured a total of 46 questions on general demographics, the crisis phase, the exit phase, the current situation, and the numbers of Covid-19 patients.
**Results or Findings:** 167 responses were received from 48 countries (Italy 12%, Spain 10%, Netherlands 9%) mostly from Neuroradiologists (72%), followed by General Radiologists (16%) and Residents (9%). Most commonly applied safety measures (>80%) were regular cleaning, distancing measures, and screening patients for infection. While 81% felt safe at work, fewer than 50% had sufficient personal protection equipment during the entire crisis. Only 22% had access to a fully equipped work station at home. 61% felt (much) worse during the crisis. Main worries concerned personal health and safety (72%), adapting to Covid-19 operational changes, non-work obligations (35%), personal finances (27%), job security (20%), and impact on research obligations (15%) and academic career advancement (11%). 78% followed online courses/congresses and 73% considered these a viable alternative for the future.
**Conclusion:** The Covid-19 pandemic substantially affected professional life as well as personal wellbeing of Neuroradiologists throughout Europe.
**Limitations:** 45 (27%) responses were from 23 countries outside Europe.
**Ethics Committee Approval:** n/a
**Funding for this study:** None.
**Author Disclosures:**
Tarek A. Yousry: Nothing to disclose
Meike W. Vernooij: Nothing to disclose
Ana Ramos Gonzalez: Nothing to disclose
Nuria Bargallo I Alabart: Nothing to disclose
Marion Smits: Consultant at Parexel Ltd. (reviewer fees paid to institution) Speaker at GE Healthcare (fees paid to institution)

### RPS 211-7

### A destructive duo-spinal tuberculosis in human immunodeficiency virus (HIV) positive patients

*B. Rathi*, A. Bellamkondi, M. Bagarhatta, K. Mehndiratta; Jaipur/IN
*(bhaveshrathi9@gmail.com)*

**Purpose:** Extrapulmonary (EP) TB have been largely associated with the HIV epidemic in which musculoskeletal system is the most common site with spinal involvement seen in 50% of cases. The classic destruction of disc space in spinal tuberculosis, leading to kyphosis and neurological impairment, become apparent months after initial spinal involvement.Therefore radiological examinations remains the primary tool for early diagnosis and guiding surgical treatment to avoid the siginificant morbidity associated with it.
**Methods or Background:** A Prospective comparative study of 50 spinal tuberculosis patients (confirmed by response to anti-tubercular treatment,ATT) in a tertiary care hospital were included after obtaining written informed consent.Among which 20 were HIV positive and 30 were HIV negative.The patients underwent a neurological examination,CD4 count, plain radiograph and MRI spine (standard protocol).
**Results or Findings:** There was no significant difference between HIV-positive and HIV-negative spinal TB patients with regard to demographic data (age and sex),the number of vertebrae involved(p=0.4) and site of involvement(paradiscal being most common).In terms of neurological outcome, hiv positive [16 (80%) had stage 1 and 4(20%) had stage 2 neurology] while hiv negative [12(40%) had stage 1 and 8(60%) had stage 2 neurology](p=0.005) which were statistically significant.Gibbus was seen in 8 HIV-positive and 21 HIV-negative patients (p=0.03). The median Cobb angle was 10 degree for hiv positive and 20 degree hiv negative patients (p<0.05). The amount of epidural abscess formation was greater in hiv positive compared to hiv negative where the mean abscess volume was 8.2±2 cm3 in HIV-positive and 2.8±1.2cm3 in HIV negative patients (p=0.03).
**Conclusion:** Hence larger epidural abscess formation with less vertebral body destruction was seen in hiv positive while the degree of vertebral body destruction and kyphosis was more in hiv negative patients.
**Limitations:** Small sample size
**Ethics Committee Approval:** Taken
**Funding for this study:** Nil
**Author Disclosures:**
Meenu Bagarhatta: Nothing to disclose
Kuldeep Mehndiratta: Nothing to disclose
Bhavyansh Rathi: Speaker at NIL
Ashwini Bellamkondi: Nothing to disclose

### RPS 211-8
**COVID-19 targeting the CNS in the Egyptian population: how MRI can be useful**
*A. M. R. Khalil*, T. M. Dawoud; Tanta/EG

**Purpose:** Thorough neurological assessment of positively proven COVID-19 patients who had neurological deficits by 1.5 Tesla MRI in Egyptian hospitals & quarantine centres to exclude CNS complications.
**Methods or Background:** The recent pandemic of COVID-19 caused by SARS-CoV-2 has turned the world into chaos owing to its high rate of transmissions among population. Recently viewed neurological manifestations among Egyptian patients with COVID-19 who were hospitalized in quarantine centres. Ataxia, disturbed consciousness and convulsions should be further evaluated by MRI for CNS involvement by SARS-CoV-2. As we are in a learning phase of how COVID-19 can target the CNS so, it is quite difficult to predict any particular diagnostic neurological test to ascertain the high-risk COVID-19 patients.This prospective study was conducted on 127 positive RT-PCR of SARS-COV-2 who were hospitalized in quarantine centres. MRI was performed by 1.5 Tesla MRI unit and images were reviewed on PACS by two independent trainee neuroradiologists with (10 years' experience for each).
**Results or Findings:** Ninety-one men (71.6%) and thirty-six women (28.3%) met the inclusion criteria, with a mean age of 52 years ± 10 (standard deviation) (age range, 12–78 years). The commonest neurological manifestations were disturbed conscious level (75.5%), agitation (9%) and coma (15.5%). The most frequent MRI findings were signal abnormalities located in the medial temporal lobe (43%), multifocal white matter hyperintense areas seen in FLAIR sequence with associated haemorrhagic areas (30%), ischemic lesions (22%) and microhaemorrhages were also noted.
**Conclusion:** During the current pandemic of COVID-19, radiologists should be aware of wide spectrum of MRI signs of CNS involvements with COVID-19.
**Limitations:** patients on mchanical ventilation
patients had metallic prothesis (incompatable for MRI)
**Ethics Committee Approval:** The research is approved by ethical commitee of Tanta University (33481/9/12)
**Funding for this study:** No funding for this study
**Author Disclosures:**
Alaa Mohammed Reda Khalil: Nothing to disclose
Tamer Mahmoud Dawoud: Nothing to disclose

**Live Q&A**

**18:00-19:00 CHANNEL 2**

### Multidisciplinary Session

### MS 3
### Sarcoma

**Moderator**
Jasminka Igrec; Graz/AT

### MS 3-2
**The role of pathology to diagnose soft tissue tumours**
Bernadette Liegl-Atzwanger; Graz/AT

**Learning Objectives:**
1. To describe the role of the pathologist in the multidisciplinary approach to soft-tissue tumours.
2. To learn about the changes in WHO 2020.
3. To understand the role of molecular pathology in soft tissue sarcomas.

### MS 3-3
**Added value of MRI in soft tissue sarcoma**
Jasminka Igrec; Graz/AT
*(Jasminka.Igrec@klinikum-graz.at)*

**Learning Objectives:**
1. To describe the role of radiology in the multidisciplinary approach to soft-tissue tumours.
2. To demonstrate the imaging possibilities of advanced MRI imaging techniques in sarcoma imaging.
3. To discuss the current use and future role of advanced imaging MRI techniques in sarcoma imaging.

### MS 3-4
**The dilemma of surgical margins: what do we need to solve it?**
Andreas Leithner; Graz/AT
*(andreas.leithner@medunigraz.at)*

**Learning Objectives:**
1. To describe the role of the orthopaedic surgeon in the multidisciplinary approach to soft-tissue tumours.
2. To discuss the new challenges in operative treatment of patients with sarcoma.
**Author Disclosures:**
Andreas Leithner: Research Grant/Support: Johnson&Johnson; Alphamed; Implantec

### MS 3-5
**Challenges in immunotherapy in sarcoma**
Joanna Szkandera; Graz/AT

**Learning Objectives:**
1. To describe the role of the oncologist in the multidisciplinary approach to soft-tissue tumours.
2. To explain how immunotherapy works.
3. To verify in which sarcoma subtypes immunotherapy should be used.

### MS 3-6 to MS 3-8
**Multidisciplinary case presentation (part 1), (part 2) and (part 3)**
Iva Brcic; Graz/AT
*(iva.brcic@medunigraz.at)*
Marko Bergovec; Graz/AT
*(marko.bergovec@medunigraz.at)*
Jasminka Igrec; Graz/AT
*(Jasminka.Igrec@klinikum-graz.at)*

**Live Q&A**

**18:00-19:00 CHANNEL 3**

### E³ - ECR Master Class

### E³ 1126
### Acute ischaemic stroke in the era of mechanical thrombectomy

**Moderator**
Horst Urbach; Freiburg/DE

### E³ 1126-2
**A. Mechanical thrombectomy in 2021**
Ansgar Berlis; Augsburg/DE
*(ansgar.berlis@klinikum-augsburg.de)*

**Learning Objectives:**
1. To learn about the clinical evidence of mechanical thrombectomy.
2. To become familiar with the technical aspects of mechanical thrombectomy.
3. To understand the importance of appropriate training.
**Author Disclosures:**
Ansgar Berlis: Consultant: Microvention, Stryker, Medtronic; Speaker: Penumbra, Phenox, Medtronic

**E³ 1126-3**
**B. The role of vascular imaging**
Frédéric Clarençon; Paris/FR
*(frederic.clarencon@psl.aphp.fr)*

**Learning Objectives:**
1. To know how to perform CT and MRI angiography.
2. To report vascular occlusion and collaterality.
3. To become familiar with recanalization prognosis.
**Author Disclosures:**
Frédéric Clarençon: Board Member: Artedrone; Consultant: Medtronic; Speaker: Balt, Penumbra, Stryker

**E³ 1126-4**
**C. The role of brain imaging**
Omer Eker; Lyon/FR

**Learning Objectives:**
1. To learn how to report ischaemia (necrosis and penumbra) using CT and MRI.
2. To learn how to identify patients at risk of haemorrhage.
3. To become familiar with post-thrombectomy follow-up.
**Author Disclosures:**
Omer Eker: Advisory Board: Johnson & Johnson; Consultant: BALT, STRYKER; Speaker: BALT

**Live Q&A**

**18:00-19:00** **CHANNEL 4**

### EuroSafe Imaging Session

### EU 5

### "Yield the shield": new approaches and challenges in the use of gonadal shielding during radiography in children

**Moderator**
Guy Frija; Paris/FR

**EU 5-2**
**Introduction**
Donald P. Frush; Durham, NC/US
*(donald.frush@duke.edu)*

**Learning Objectives:**
1. To provide summary justification for revisiting routine gonadal shielding during abdomiopelvic radiography in children: domain covered and why are we doing this.
2. To present the outline of topics to be covered: roles, challenges, and models for change.
3. To introduce the speakers and their qualifications.

**EU 5-3**
**The past journey of gonadal shielding and the role of regulation and the U.S. radiologist**
Donald P. Frush; Durham, NC/US
*(donald.frush@duke.edu)*

**Learning Objectives:**
1. To learn the background of gonadal shielding during radiography and the efforts and organisations currently involved in the United States.
2. To become familiar with the role of the US radiologist in changes in practice of routine gonadal shielding.
3. To understand the current regulations and guidance for the use of gonadal shielding in the U.S.

**EU 5-4**
**The current reasons for change and the role of regulation and the European radiologist**
Joanna Kasznia-Brown; Taunton/UK

**Learning Objectives:**
1. To learn about the scientific evidence supporting a change in practice for routine gonadal shielding.
2. To become familiar with the role of the European radiologist to changes in practice of routine gonadal shielding.
3. To understand the current regulations and guidance for the use of gonadal shielding in Europe.

**EU 5-5**
**Front-line culture, challenges, and strategies for change: European perspective**
Joana Santos; Coimbra/PT
*(joanasantos@estescoimbra.pt)*

**Learning Objectives:**
1. To learn about the role of the radiographer/technologist community in Europe for routine gonadal shielding (certification, training, licensing etc.).
2. To appreciate the complexities in changes to practice, including patient/caregiver engagement.
3. To identify successful models and ongoing efforts for change in practice.

**EU 5-6**
**Front-line culture, challenges, and strategies for change in shielding practice: U.S. perspective**
Rebecca Marsh; Denver, CO/US
*(REBECCA.MARSH@CUANSCHUTZ.EDU)*

**Learning Objectives:**
1. To learn about the role of the radiographer/technologist community in Europe for routine gonadal shielding (certification, training, licensing etc.).
2. To appreciate the complexities in changes in practice, including patient/caregiver engagement.
3. To identify the salient ongoing efforts and suggest models for change in shielding practice.

**Live Q&A: "Easier said than done?"**

**18:00-19:30** **CHANNEL 5**

### Healthcare Professionals in Focus

### IF 3

### The science of meditation and mindfulness

**Moderator**
M.G. Myriam Hunink; Rotterdam/NL

**IF 3-2**
**The effectiveness of mindfulness meditation**
M.G. Myriam Hunink; Rotterdam/NL
*(m.hunink@erasmusmc.nl)*

**Learning Objectives:**
1. To learn about the origins of mindfulness and how it relates to meditation, yoga, and the martial arts.
2. To appreciate the effects on the brain and body of practicing mindfulness or similar practices.
3. To learn about the results of randomized controlled trials studying mindfulness and related interventions.
**Author Disclosures:**
M.G. Myriam Hunink: Investigator: PI DESTRESS study ; Research Grant/Support: American Diabetes Association Netherlands Organization for Health Research and Development German Innovation Fund Netherlands Educational Grant (''Studie Voorschot Middelen'') Gordon and Betty Moore Foundation; Other: Royalties for textbook from Cambridge University Press

**IF 3-3**
**The effect of the pandemic and what can help**
Leonieke Kranenburg; Rotterdam/NL
*(l.kranenburg@erasmusmc.nl)*

**Learning Objectives:**
1. To understand the effect of the pandemic on chronic stress and burnout among healthcare professionals.
2. To learn about the results of mindfulness and similar interventions in dealing with stress, anxiety depression, and burnout.

**Live Q&A: Mindfulness: Hope or hype?**

19:45-20:45 CHANNEL 2

### Transatlantic Course of ESR and RSNA (Radiological Society of North America): Stroke Imaging and Endovascular Treatment: Now and the Future

### TC 428

### The future strategy for stroke thrombectomy

**Moderators**
Jean-Pierre Pruvo; Lille/FR
Raman Uberoi; Oxford/UK
Achala Vagal; Cincinnati, OH/US

### TC 428-4

**A. Addressing workforce needs: who and how to train specialists**
Hans van Overhagen; The Hague/NL
*(h.voverhagen@hagaziekenhuis.nl)*

**Learning Objectives:**
1. To learn about the number and type of specialists trained.
2. To understand the global organisation of stroke interventionists and neurologists.
3. To appreciate the future potential number of cases.

### TC 428-5

**B. The future for stroke thrombectomy: what is next?**
Mahesh V. Jayaraman; Providence, RI/US
*(MJayaraman@Lifespan.org)*

**Learning Objectives:**
1. To learn about the subgroups that were not studied in the recent stroke trials.
2. To become familiar with use of artificial intelligence in stroke.

### TC 428-6

**C. New innovations in stroke thrombectomy techniques and technology**
Stephan Meckel; Linz/AT

**Learning Objectives:**
1. To understand the limitations of current devices and techniques.
2. To appreciate the evolution in stroke thrombectomy technology.
3. To understand how the different devices and techniques may improve outcomes.

**Live Q&A**

19:45-20:45 CHANNEL 3

### Refresher Course: Imaging Informatics

### RC 205

### How artificial intelligence (AI) has changed radiology

**Moderator**
Elmar Kotter; Freiburg/DE

### RC 205-2

**A. How to integrate AI into the clinical workflow**
Tim Leiner; Utrecht/NL
*(t.leiner@umcutrecht.nl)*

**Learning Objectives:**
1. To learn how different AI systems can be integrated to support the radiological workflow.
2. To understand the benefits of integrating AI systems into the clinical routine.
3. To learn the challenges related to the AI workflow.

**Author Disclosures:**
Tim Leiner: Founder: Quantib-U; Research Grant/Support: Pie Medical, Medis, Philips Healthcare, Bayer AG; Other: Department of Radiology of Utrecht University Medical Center receives royalties from Sectra AB

### RC 205-3

**B. AI in automated organs segmentation**
Ana Jiménez-Pastor; Valencia/ES
*(anjipas@gmail.com)*

**Learning Objectives:**
1. To learn how AI has enabled automated image analysis through human-like performance in segmentation.
2. To appreciate the main AI network architectures allowing for multiple-organs segmentation.
3. To understand novel techniques for automated lesions segmentation.
**Author Disclosures:**
Ana Jiménez-Pastor: Employee: QUIBIM SL

### RC 205-4

**C. AI in detection and classification applied to workflow prioritisation**
Ramin Khorasani; Boston, MA/US

**Learning Objectives:**
1. To learn how we can make use of AI to optimise radiology workflow.
2. To appreciate the impact of AI in real productivity metrics of radiologists.
3. To understand the trustworthiness of AI for first-reads and worklists prioritisation.

**Live Q&A**

19:45-20:45 CHANNEL 4

### E³ - Rising Stars Programme: Basic Sessions

*Organised by the ESOR*

### BS 14

### Radiologic anatomy: head and neck

**Moderator**
Minerva Becker; Geneva/CH

### BS 14-2

**Neck spaces**
Nikoleta I. Traykova; Plovdiv/BG

**Learning Objectives:**
1. To learn the anatomy of the neck spaces.
2. To discuss the current imaging techniques for their evaluation.
3. To describe the imaging appearance of the most common pathological findings.

### BS 14-3

**Temporal bone**
Jan Walther Casselman; Bruges/BE
*(jan.casselman@azsintjan.be)*

**Learning Objectives:**
1. To learn which technique is best suited to visualise specific anatomical structures of the temporal bone.
2. To recognise the clinically most important anatomical structures of the middle and inner ear.
3. To learn how the anatomical structures of the temporal bone appear in the transverse, coronal, and double oblique plane.
**Author Disclosures:**
Jan Walther Casselman: Speaker: Philips Healthcare / Cefla s.a.

### BS 14-4

**Larynx**
Roberto Maroldi; Brescia/IT
*(roberto.maroldi@unibs.it)*

**Learning Objectives:**
1. To understand the anatomy and signals of the cartilage framework of the larynx.
2. To learn the surgical subdivision of the paraglottic space of the larynx into its key compartments.
3. To learn the anatomy and signals of the muscles within the larynx.

**Live Q&A**

**19:45-20:45 CHANNEL 5**

### Research Presentation Session: Radiographers

### RPS 214

### The radiography profession: a life-long learning experience

**Moderators**
Charlotte Beardmore; London/UK
Lorenzo Preda; Milan/IT

### RPS 214-3

**The occupational and psychological impact of COVID-19 on radiographers in Ireland in 2020**

*J. Creedon*[1], A. O'Loughlin[1], S. J. Foley[2]; [1]Cork/IE, [2]Dublin/IE
*(creedonjill@gmail.com)*

**Purpose:** This research aims to evaluate the impact of covid-19 on radiographers
and radiology services in Ireland over time. This includes but is not exclusive to personal protective
equipment (PPE) availabilities, workload changes, inadvertent exposures to covid-19 and
radiographers' mental health concerns.
**Methods or Background:** Imaging is required for assessing the severity and disease progression of covid-19 both in the Emergency Department (ED) and in an inpatient capacity. Radiographers are in frequent direct contact with both confirmed positive and suspected infected persons, placing them at an increased risk of exposure to the virus. The impact of covid-19 on radiographers in Ireland was evaluated through means of a two-stage electronic questionnaire distributed six weeks apart. This method focussed on collating radiographers' perspectives on working conditions during the initial covid-19 response (March, 2020) with those during the ending of initial emergency measures in Ireland (May, 2020).
**Results or Findings:** 646 survey responses were received and all six Irish health regions represented. Most radiographers (77%) reported having adequate PPE available, however almost 50% were inadvertently exposed to covid-19 positive patients without appropriate PPE. This was attributed to poor communication and testing. Anxiety levels reduced substantially six weeks into the crisis period although obvious distress was noted among some respondents. Burnout symptoms were reported by 40% of radiographers and 30% reported considering changing jobs or retiring, both credited to the covid-19 outbreak.
**Conclusion:** Clear communication regarding changing protocols and patients' infectious status are essential to safeguard radiographers and to minimise the induction of anxiety and distress. Staff mental health concerns must be addressed and burnout symptoms identified to prevent long term negative consequences of the covid-19 pandemic on radiographers and radiology services.
**Limitations:** n/a
**Ethics Committee Approval:** n/a
**Funding for this study:** n/a
**Author Disclosures:**
Jill Creedon: Nothing to disclose
Anne O'Loughlin: Nothing to disclose
Shane J Foley: Nothing to disclose

### RPS 214-4

**Radiotherapy education in the EU: the impact on practitioners' mobility and patient safety**

*J. G. Couto*[1], S. L. McFadden[2], P. McClure[2], P. Bezzina[1], C. Hughes[2]; [1]Msida/MT, [2]Jordanstown/UK
*(jose.g.couto@um.edu.mt)*

**Purpose:** Previous research showed that the lack of regulation of therapeutic radiography education across the EU leads to differences in education programmes and competence level between member states. The current study aimed to understand the impact of these differences on professional mobility and patient safety.
**Methods or Background:** Interviews with stakeholders (local and migrant professionals, clinical managers, educators, students and professional association representatives) from Finland, Poland, Portugal and United Kingdom (due to disparate education characteristics) were used in this qualitative cross-sectional study. Thematic analysis was performed using NVivo (v.12).
**Results or Findings:** Stakeholders (n=30) suggested that a combination of several characteristics have an impact on the quality of education and competency level i.e. programme duration, academic level, specialisms covered, RT-specific training, staffing and teaching methods. Differences in education hinder professional mobility from some countries. Other factors impacting mobility included: workforce scarcity or excess, registration process, language, personal and family reasons, professional growth and salary. The standardisation of education could improve mobility across countries, however, diversity in education enables an overall growth of the profession. Participants believe that professional mobility does not compromise patient safety, but regulatory bodies and employers are essential in ensuring staff competence. Some programmes lack RT-specific training, compromising safety even within the same country of graduation. Diversity in the workforce, evidence-based practice, risk assessment, and multidisciplinarity improve care, while language issues can compromise safety.
**Conclusion:** Stakeholders should consider the programme characteristics mentioned above when designing educational programmes to improve patient care and safety and facilitate professionals' mobility.
**Limitations:** A larger sample of countries would improve the picture of education across Europe.
**Ethics Committee Approval:** This study was approved by the Research Ethics Committee at Ulster University.
**Funding for this study:** Part of the SAFE EUROPE project, funded by the ERASMUS+ Programme.
**Author Disclosures:**
Ciara Hughes: Nothing to disclose
Patricia McClure: Nothing to disclose
Sonyia Lorraine McFadden: Nothing to disclose
Paul Bezzina: Nothing to disclose
Jose Guilherme Couto: Nothing to disclose

### RPS 214-5

**Anticipating the role of touchless technologies within diagnostic radiography: results of an international questionnaire**

*A. England*[1], J. D. Thompson[2], E. Littler[3], J. Tugwell-Allsup[4], E. Edwards[1]; [1]Keele/UK, [2]Manchester/UK, [3]Warrington/UK, [4]Bangor/UK
*(a.england@keele.ac.uk)*

**Purpose:** Digital healthcare technology is evolving at a rapid rate. Touchless technologies (TT), involving gestures and voice commands, are being incorporated into daily life. The aim of this study was to survey the anticipated role of TTs within diagnostic radiography.
**Methods or Background:** An online questionnaire was developed, piloted and deployed to radiographers as part of an international radiology congress. Eligible respondents were radiographers, either qualified or in-training. The survey covered ten themes relating to the role of TTs, including perceptions of examination scenarios where TTs might be useful. Results were analysed using descriptive and inferential statistics, together with thematic analysis.
**Results or Findings:** 155 radiographers completed the survey. 100 (64.9%) were female and clinical experience ranged from 0 to 40 (mean 13.5) years. 54 (35.1%), had a Bachelor's degree, with respondents being from 23 different countries (five continents). 121 (79.1%) respondents personally owned or intended to purchase TTs. 89 (84.8%) of respondents indicated that they would use TTs, if available on current imaging equipment. 25 (16%) respondents reported that they have access to TTs within their departments. 88 (81.5%) and 67 (65%) respondents reported that they saw voice and gesture controls as being key in improving exam efficiency.
**Conclusion:** Participants anticipate a positive role for touchless technologies within diagnostic radiography. Access to such technology is not yet widely available. Voice activated technologies were favoured over gesture-based aids for improvements in examination efficiency.
**Limitations:** Questionnaires were delivered only in English and as such would have limited responses from non-native English speaking countries. Responses were not received from all countries, particularly those in South America and as such results cannot be considered as being fully global.
**Ethics Committee Approval:** This project was approved by the University of Salford.
**Funding for this study:** This project received no funding.

**Author Disclosures:**
Emma Edwards: Nothing to disclose
Jenna Tugwell-Allsup: Nothing to disclose
Andrew England: Nothing to disclose
Emily Littler: Nothing to disclose
John David Thompson: Nothing to disclose

### RPS 214-6

**An understanding of Irish radiographers' opinions on the implementation of artificial intelligence into radiography practice**

*M-L. Ryan*, J. McNulty; Dublin/IE (marielouise.ryan@ucd.ie)

**Purpose:** Artificial Intelligence (AI) is on the cusp of revolutionizing radiography internationally. Literature has raised positive aspects, challenges and concerns from different medical imaging professionals. The purpose of this work was to understand Irish radiographers understanding of AI, their opinions on implementation, education required and effects on the profession.
**Methods or Background:** A web-based questionnaire was designed, and all Irish public and private hospitals were contacted to participate, together with social media promotion. Participation was anonymous and voluntary.
**Results or Findings:** 318 Irish radiographers participated. 95% of respondents were comfortable working with technology. 93.2% were familiar with the concept of AI and 76.6% with the concept of machine learning. 65% of respondents agreed that implementing AI would be an opportunity to redefine the radiographers role. 70.2% of respondents agreed that AI will enhance the quality of service provided by radiographers. 47.6% of respondents had some ethical concerns about AI, with a large majority (80.6%) saying AI should be regulated by national legislation. 94.71% of respondents noted it was important for radiographers to be involved IN AI tool development, with 68.3% having moderate trust in AI tools. Regarding education, 19.6% had some AI training. 83% felt that training should occur at undergraduate level, with 66.5% suggesting CPD.36.1% felt that AI will have a significant impact on the profession within the next 5 years. A large majority (86.16%) were favorable to AI implementation in radiography practice in Ireland.
**Conclusion:** Whilst support for AI integration is largely positive, discussion need to happen around key areas such as trust, ethical concerns and regulation.
**Limitations:** Data is self-stated opinion only, which is a common bias in survey research.
**Ethics Committee Approval:** This project was reviewed in line with the institutional Ethics Review process.
**Funding for this study:** No specific funding.
**Author Disclosures:**
Marie-Louise Ryan: Nothing to disclose
Jonathan McNulty: Nothing to disclose

### RPS 214-7

**An investigation of radiographers' perceptions of the scope of advanced practice roles in radiography**

*D. Catania*[1], N. Giannotti[2], A. Roletto[1], M. D. Davis[3]; [1]Milan/IT, [2]Sydney/AU, [3]Dublin/IE (cataniadiego@hotmail.com)

**Purpose:** This study investigated how advancements in radiography practice are perceived by European radiographers and how advanced roles are currently practiced internationally. The information provided baseline data to assist in determining how barriers to professional role advancements may be reduced. This insight is to be used to progress role development opportunities within Radiography, with the aim of optimising professional work practice post COVID-19.
**Methods or Background:** An online survey containing fifteen questions was developed and administered via the EFRS Research Hub2020. The survey included questions on participants' demographics, education, active memberships, current radiography skills, advanced radiography practice roles available and how these roles could be progressed in their country of work and the main positives for advancing roles.
**Results or Findings:** Eighty-three participants completed and submitted the survey. The majority of participants were members of the European Federation of the Radiography Societies or European Society of Radiology. More than half of the participants considered reporting X-rays as one of the main advancements for the radiography profession, this was followed by being promoted as a clinical specialist (22%) and delivery of intravenous administration (22%). The majority of the participants stated that advanced practice roles should be supported by postgraduate studies (22%) and collaboration with radiologists/in-house training (17% respectively).
**Conclusion:** An insight into radiographers' perceptions of advanced practice was achieved. The advancements in radiography practice identified have the potential to assist radiology departments to streamline processes and optimise the necessary resources to provide a comprehensive response to the continual increase in use of medical imaging globally and to respond efficiently to healthcare demands, as the COVID-19 pandemic continues, in its' aftermath and as Radiology services recover overtime.
**Limitations:** None
**Ethics Committee Approval:** Obtained in the form of an exemption from full ethical review
**Funding for this study:** None
**Author Disclosures:**
Diego Catania: Nothing to disclose
Nicola Giannotti: Nothing to disclose
Andrea Roletto: Nothing to disclose
Michaela Dawn Davis: Nothing to disclose

**Live Q&A**

**21:00-21:30** **CHANNEL 2**

### PL 4

### Plenary Lecture

**Moderator**
Michael H. Fuchsjäger; Graz/AT

### PL 4-2

**A global perspective on oncologic imaging: why should we care?**

Hedvig Hricak; New York, NY/US
**Author Disclosures:**
Hedvig Hricak: Advisory Board: University of Vienna, Johns Hopkins, DKFZ; Board Member: IBA

**Live Q&A**

### Sunday, March 7

**08:00-09:00** **CHANNEL 2**

### Research Presentation Session: Genitourinary

### RPS 202

### Beyond conventional breast imaging

**Moderator**
Alexandra Athanasiou; Athens/GR

### RPS 202-3

**DCE-MRI radiomics analysis in differentiating luminal A and luminal B breast cancer molecular subtypes**

*O. Lafcı*, P. S. Öztekin, P. Celepli, P. N. N. Koşar; Ankara/TR
*(oguz-oguzz@hotmail.com)*

**Purpose:** The aim of the present study was to investigate the association between Luminal A and Luminal B molecular subtypes and radiomics features of dynamic contrast-enhanced magnetic resonance imaging (DCE-MRI) in patients with invasive ductal cancer.
**Methods or Background:** 73 patients with histopathologically proven invasive ductal cancer (IDC) were selected. Tumors were classified into molecular subtypes: Luminal A (ER-positive and/or progesterone receptor (PR)-positive, human epidermal growth factor receptor type 2 (HER2) -negative, proliferation marker Ki-67< 20) and Luminal B (ER-positive and/or PR-positive, HER2-positive or HER2-negative with high Ki-67 ≥20). A total of 81 tumoral lesions were evaluated on T1-weighted fat-suppressed sagittal post-contrast late-phase MRI images after the required "pre-process" steps and 3D segmentation were made. 43 radiomics features including: 1 conventional, 4 shape, 6 histogram, 7 Gray Level Co-occcurance Matrix (GLCM), 11 Gray Level Run Length Matrix (GLRLM), 3 Neighborhood Gray Level Difference Matrix (NGLDM), 11 Gray Level Zone Length Matrix (GLZLM) were extracted by using the software LIFEX.
**Results or Findings:** A statistically significant difference was found in radiomics properties including Histogram-skewness; Shape volume, voxel; GLCM-energy, entropy.log2, entropy.log 10; GLRLM-GLNU, RLNU, HGRE; NGLDM-Busyness; GLZLM-SZE, GLNU, HGZE, ZLNU between two different molecular subtypes.
**Conclusion:** Radiomics properties that may distinguish Luminal A and Luminal B molecular subtypes of IDC were determined. The radiomics features were thought to reflect the intratumoral heterogeneity in molecular subtypes. It has been predicted that the characterization of Luminal A and Luminal B tumors may be made non-invasively by radiomics analysis.
**Limitations:** Retrospective study design, the limited number of patients, one center study
**Ethics Committee Approval:** Approved by the local ethics committee.
**Funding for this study:** None
**Author Disclosures:**
Pelin Seher Öztekin: Nothing to disclose
Pinar Celepli: Nothing to disclose
Oğuz Lafcı: Nothing to disclose
Pinar Nercis Nercis Koşar: Nothing to disclose

### RPS 202-4

**Shear wave and strain elastography to reduce unnecessary biopsies in breast cancer diagnostics**

*A. Pfob*[1], R. G. Barr[2], T. Bruckner[1], C. Büsch[1], Z. Alwafai[3], C. S. Balleyguier[4], D-A. Clevert[5], V. F. Duda[6], M. Gonçalo[7], I. Gruber[8], M. Hahn[8], P. Kapetas[9], R. Ohlinger[3], M. J. Rutten[1]0, M. Tozaki[11], S. Wojcinski[12], G. Rauch[13], J. Heil[1], M. Golatta[1]; [1]Heidelberg/DE, [2]Ravenna, OH/US, [3]Greifswald/DE, [4]Villejuif/FR, [5]Munich/DE, [6]Marburg/DE, [7]Coimbra/PT, [8]Tuebingen/DE, [9]Vienna/AT, [10]'S-Hertogenbosch/NL, [11]Kagoshima/JP, [12]Bielefeld/DE, [13]Berlin/DE
*(andre.pfob@med.uni-heidelberg.de)*

**Purpose:** Minimally invasive biopsies are indicated to rule out malignancy for all unclear breast lesions with >2% likelihood of malignancy (BI-RADS© 4 or higher). Biopsies find up to 90% of lesions to be benign, which causes unnecessary physical and psychological distress for patients and costs for health care systems. We evaluated the feasibility of combining shear wave and strain elastography (2D-SWE + SE), a new ultrasound technique, to recategorize BI-RADS© 3 or 4 lesions to reduce the number of unnecessary biopsies without impairing the breast cancer detection rate.
**Methods or Background:** We analyzed prospective, multicenter, international data from 1288 women with breast lesions rated by conventional 2D B-mode US as BI-RADS© 3 to 4c undergoing 2D-SWE and SE. All patients underwent histopathologic evaluation which was the reference standard. Reduction of unnecessary (benign) biopsies after recategorizing with 2D-SWE + SE and missed malignancies were the outcome measures. A maximum of 2% missed tumors after recategorizing was the safety endpoint analogous to BI-RADS© 3 definition by ACR.
**Results or Findings:** Histopathologic evaluation showed malignancy in 368 of 1288 lesions.
Following the traditional BI-RADS© assessment, 53.80% (495 of 920 patients) underwent an unnecessary biopsy (biopsy in benign lesion).
After recategorizing BI-RADS© 4a lesions with 2D-SWE + SE (2D-SWE cut-off 3.70 m/s), 34.78% (320 of 920 patients) underwent an unnecessary biopsy - unnecessary biopsies were reduced by 35.35% (320 vs. 495). Malignancies in the new BI-RADS© 3 cohort were missed in 1.96% (12 of 612 patients).
**Conclusion:** In breast cancer diagnostics, adding 2D-SWE + SE with a cutoff of 3.70 m/s could help reduce the number of unnecessary minimally invasive biopsies by approximately 35%, avoiding treatment burden for patients, providers and health care systems.
**Limitations:** Not applicable
**Ethics Committee Approval:** Yes:S-429/2015
**Funding for this study:** Siemens Medical Solutions USA, Inc
**Author Disclosures:**
Markus Hahn: Nothing to disclose
Mitsuhiro Tozaki: Nothing to disclose
Christopher Büsch: Nothing to disclose
Dirk-André Clevert: Nothing to disclose
Michael Golatta: Nothing to disclose
Thomas Bruckner: Nothing to disclose
Sebastian Wojcinski: Nothing to disclose
Ralf Ohlinger: Nothing to disclose
Panagiotis Kapetas: Nothing to disclose
Zaher Alwafai: Nothing to disclose
Matthieu J.C.M. Rutten: Nothing to disclose
André Pfob: Nothing to disclose
Ines Gruber: Nothing to disclose
Geraldine Rauch: Nothing to disclose
Corinne S. Balleyguier: Nothing to disclose
Manuela Gonçalo: Nothing to disclose
Volker F. Duda: Nothing to disclose
Joerg Heil: Nothing to disclose
Richard G. Barr: Nothing to disclose

### RPS 202-5

**Accuracy and reproducibility of contrast-enhanced mammography (CEM) in the assessment of response to neoadjuvant chemotherapy (NAC) in breast cancer patients with calcifications in the tumour bed**

*V. Iotti*, S. Ravaioli, V. Marchesi, G. Besutti, M. Ragazzi, M. Revelli, R. Vacondio, P. Giorgi Rossi, P. Pattacini; Reggio Emilia/IT
*(valentina.iotti@ausl.re.it)*

**Purpose:** To evaluate CEM accuracy and reproducibility in the detection and measurement of residual tumor after NAC in BC patients with calcifications in the tumor bed, using pathological examination of the surgical specimen as the reference standard.
**Methods or Background:** All consecutive BC patients receiving NAC from 2012 to 2020 who presented pathological calcifications in the tumor bed at diagnosis and underwent CEM before and after NAC, were included. The pathological reference standard included presence/absence, size, and type (invasive and/or in-situ) of residual tumor. CEM were retrospectively reviewed by two independent radiologists, describing absence/presence and size of residual disease according to contrast enhancement (CE) only and to CE plus calcification. Discordant cases were discussed with a third radiologist.
**Results or Findings:** Of the 36 included patients (median age 52 years), 28 (77.8%) had invasive and 5 (13.9%) in-situ only residual carcinoma at pathology of surgical specimen.
CEM sensitivity for invasive residual tumor increased (from 85.7%,95%CI=67.3-96%, to 96.4%,95%CI=81.7-99.9) and specificity decreased (from 62.5%,95%CI=24.5-91.5% to 14.3%,95%CI=0.4-57.9%) when considering CE+calcifications instead of CE-only; in the detection of in-situ only residual tumor, false negatives decreased (from 3 to 0) and false positives increased (from 1 to 2).
For residual tumor detection the inter-reader reproducibility was 100%.
Considering CE+calcifications versus CE-only, the concordance in residual tumor diameter estimation between CEM and pathology increased ($R^2$ from 0.38 to 0.45), reflecting a decrease in underestimations, while inter-reader concordance decreased ($R^2$ from 0.79 to 0.66).
**Conclusion:** CEM allows a direct combined evaluation of CE and calcification in the tumor bed after NAC.
The evaluation of CE+calcification showed higher sensitivity and decreased underestimation of residual tumor size than considering CE-only, but worsened specificity.
**Limitations:** Retrospective imaging classification; small sample.
**Ethics Committee Approval:** AVEN Ethic Committee (n.2020/0119203).
**Funding for this study:** None.
**Author Disclosures:**

Matteo Revelli: Nothing to disclose
Valentina Iotti: Speaker at GE Healthcare, Consultant at Bayer
Pierpaolo Pattacini: Nothing to disclose
Moira Ragazzi: Nothing to disclose
Vanessa Marchesi: Nothing to disclose
Rita Vacondio: Nothing to disclose
Paolo Giorgi Rossi: Nothing to disclose
Giulia Besutti: Nothing to disclose
Sara Ravaioli: Nothing to disclose

### RPS 202-6

**Applicability of the Kaiser score clinical decision rule to contrast-enhanced mammography (CEM) interpretation of breast lesions: a comparison with interpretation according to the BI-RADS**

*A. A. Marzogi*[1], P. Clauser[2], I. Biondić Špoljar[3], A. Korajac[2], P. Kapetas[2], T. H. Helbich[2], P. A. Baltzer[2]; [1]Makkah/SA, [2]Vienna/AT, [3]Zagreb/HR
*(alaamarzogi@live.com)*

**Purpose:** The objective of this study was to test the applicability of the Kaiser Score (KS) in the characterization of breast lesions detected at contrast enhanced mammography (CEM) and compare its results to ínterpretation according to BI-RADS.
**Methods or Background:** IRB-approved retrospective, cross-sectional, single-center study. Patients who underwent CEM (1 ml/kg of iodine contrast medium (400 mgI/ml) between July-2018 and October 2020 were included. Exclusion criteria were absence of histology (reference standard), no delayed-phase image (cranio-caudal view of the side of pathology acquired after the four standard projections). Two radiologists (8 and 5 years of breast imaging experience), blinded to patient data and histopathology results, evaluated the breast lesions and separately assigned KS and BI-RADS. A ROC-curve analysis was performed.
**Results or Findings:** Among 200 patients who underwent CEM, 116 lesions were included (70 malignant (60%), 46 benign (40%)). The areas under the curve (AUC) using the KS and BI-RADS for Reader 1 were 82% (95% confidence interval (CI): 73.8% to 88.5%) and 72.0% (95% CI: 63.6% to 80.5%) ($p < 0.05$), respectively. For Reader 2 the AUC using the KS and BI-RADS were 83.7% (95% CI: 75.7 % to 89.9%), and 71.7% (95% CI: 62.6% to 79.7%) ($p < 0.05$), respectively. The KS´s AUC was superior to BI-RADS for both readers ($p < 0.05$).
**Conclusion:** The KS is applicable to lesions detected on CEM and showed a superior accuracy compared to interpretation according to BI-RADS.
**Limitations:** No.
**Ethics Committee Approval:** IRB-approved
**Funding for this study:** No
**Author Disclosures:**
Pascal A.T. Baltzer: Nothing to disclose
Iva Biondić Špoljar: Nothing to disclose
Alaa Abdulrahman Marzogi: Nothing to disclose
Aida Korajac: Nothing to disclose
Thomas H. Helbich: Nothing to disclose
Panagiotis Kapetas: Nothing to disclose
Paola Clauser: Speaker at for siemens healthcare gmbh

### RPS 202-7

**A multimodal hybrid 18F-FDG PET/MRI radiomics model discriminating biological properties of malignant and benign breast lesions**

*V. Romeo*[1], P. Clauser[2], P. Kapetas[2], P. A. Baltzer[2], S. Rasul[2], P. Gibbs[3], K. Pinker-Domenig[3], R. A. Woitek[4], T. H. Helbich[2]; [1]Naples/IT, [2]Vienna/AT, [3]New York, NY/US, [4]Cambridge/UK
*(valeria.romeo@unina.it)*

**Purpose:** To develop an artificial intelligence (AI)-based radiomics model applied to simultaneous 18F-FDG PET/MRI for differentiation of malignant and benign breast lesions.
**Methods or Background:** In this IRB-approved, prospective, single institution study, 102 patients with 120 breast lesions (BI-RADS 0, 4/5: 101 malignant, 19 benign) detected by ultrasound and/or mammography were included and underwent hybrid 18F-FDG PET/MRI of the breast. Quantitative parameters and radiomics features (first, second, higher order) were extracted from dynamic contrast-enhanced (tumor Mean Transit Time (MTT), Volume Distribution, Plasma Flow), diffusion-weighted (DWI) (tumor ADCmean and contralateral breast parenchyma), PET (tumor SUVmax, mean and minimum, SUVmean of ipsi- and contralateral breast parenchyma) and T2-weighted imaging. Different AI models were developed using a fine gaussian support vector machine (SVM) classifier with a 5-fold cross validation, using different combinations of quantitative parameters and radiomics features to obtain the highest accuracy for breast cancer diagnosis. The performance of the best radiomics model was compared with that of expert readings by a radiologist and a nuclear medicine physician using the McNemar test.
**Results or Findings:** Eight radiomics models were developed. The integrated model combining MTT and ADCmean of breast lesion with radiomics features extracted from PET and ADC images obtained the highest accuracy for breast cancer diagnosis (AUC 0.983, accuracy: 94.8%, sensitivity:95.3%, specificity: 94.3%, PPV:94.3%, NPV:95.3%). The performance of the SVM classifier was better (AUC 0.983) but not significantly different from the performance of the expert radiologist and nuclear medicine physician (AUC 0.868, p=0.508).
**Conclusion:** An AI-based radiomics model combining quantitative perfusion and DWI parameters with radiomics features extracted from simultaneous 18F-FDG PET/MRI images characterizes malignant and benign breast lesions with high accuracy.
**Limitations:** Small sample size
Lack of external validation
Unbalanced distribution of benign and malignant breast lesions
**Ethics Committee Approval:** IRB-approved
**Funding for this study:** EU grant
**Author Disclosures:**
Pascal A.T. Baltzer: Nothing to disclose
Katja Pinker-Domenig: Nothing to disclose
Valeria Romeo: Research/Grant Support by the ESOR "Bracco Fellowship"
Thomas H. Helbich: Grant Recipient at EU Grant
Ramona Anna Woitek: Nothing to disclose
Sazan Rasul: Nothing to disclose
Panagiotis Kapetas: Nothing to disclose
Peter Gibbs: Nothing to disclose
Paola Clauser: Speaker at Siemens

### RPS 202-8

**MRI radiomics signatures for the prediction of risk of breast cancer recurrence: correlation with genomic recurrence scores**

C. Rossi[1], A. Bitencourt[1], P. Gibbs[2], R. Lo Gullo[2], *I. Daimiel Naranjo*[2], J. V. M. Horvat[2], S. Thakur[2], E. A. Morris[2], K. Pinker-Domenig[2]; [1]Sao Paulo/BR, [2]New York, NY/US

**Purpose:** To correlate radiomic features from breast MRI and Oncotype DX recurrence scores (RS) in patients with early-stage estrogen-recpetor (ER)-positive/human epidermal growth factor receptor 2 (HER2)-negative invasive breast cancer.
**Methods or Background:** This was a retrospective study between November 2016-September 2018. Inclusion criteria were: early-stage breast ER-positive/HER2-negative breast cancer, axillary node-negative, pre-treatment breast MRI, and available Oncotype DX RS. Exclusion criterion was insufficient image quality preventing radiomics analysis. Dynamic contrast-enhanced (DCE) images were reviewed and three-dimensional tumor segmentation for radiomics analysis (first post-contrast T1-weighted sequence) was performed. Radiomics parameters were calculated using publicly available CERR software, and measures of accuracy, including sensitivity, specificity, negative predictive value (NPV) and positive predictive value (PPV), were estimated. Oncotype DX recommendations for stratification were used: Low-risk (little-no benefit from chemotherapy): patients>50 years; RS≤25. Patients≤50 years; RS≤15; Intermediate-risk (chemotherapy may be offered): patients≤50 years; RS=16-25; high-risk (candidates for chemotherapy) RS≥26.
**Results or Findings:** 175 tumors were analyzed. Oncotype DX RS comprised 87 low-risk cases, 67 intermediate-risk cases and 21 high-risk cases. Significant differences were found between low/intermediate-risk (154) and high-risk (21) cases for 12 radiomics features. After minority class size boosting via adaptive synthetic sampling, LASSO regression was used to determine the top five parameters for model development: robust median absolute deviation(first order), hgce (NGLDM), joint max(GLCM), zp(SZM) and lzlgle (SZM). A support vector-machine model with 5-fold cross-validation yielded 86.5% accuracy, 98.6% sensitivity, 76.0% specificity, 80.5% PPV and 95.9% NPV.
**Conclusion:** Radiomics signatures from DCE-MRI showed high accuracy identifying patients at high-risk of recurrence and could be used as non-invasive alternative to genomic RS to select patients who will benefit from chemotherapy.
**Limitations:** Small sample size. Single center data.
**Ethics Committee Approval:** IRB-approved. Informed consent was waived.
**Funding for this study:** NIH/NCI Cancer Center Support Grant(P30 CA008748). Breast Cancer Research Foundation.
**Author Disclosures:**
Joao Vicente Machado Horvat: Nothing to disclose
Carolina Rossi: Nothing to disclose
Almir Bitencourt: Nothing to disclose
Katja Pinker-Domenig: Nothing to disclose
Elizabeth A. Morris: Nothing to disclose
Roberto Lo Gullo: Nothing to disclose
Sunitha Thakur: Nothing to disclose
Peter Gibbs: Nothing to disclose
Isaac Daimiel Naranjo: Nothing to disclose

**Live Q&A**

08:00-09:00 CHANNEL 3

### Joint Session of the ESR and EFSUMB

### ESR/EFSUMB

### New options of diagnosis and treatment in MSK ultrasound

**Moderators**
Dirk André Clevert; Munich/DE
Vito Cantisani; Rome/IT

### ESR/EFSUMB-3
**Ultrasound-guided MSK interventional procedures: what is the evidence?**
Luca M. Sconfienza; Milan/IT
*(io@lucasconfienza.it)*

**Learning Objectives:**
1. To understand the basic methods for ultrasound-guided MSK interventional procedures.
2. To show the interventional techniques in the major joints.
3. To present the current evidence on the main ultrasound-guided MSK interventional procedures.

**Author Disclosures:**
Luca M. Sconfienza: Advisory Board: Pfizer; Speaker: Samsung Medison, Esaote SPA, Abiogen, Novartis, Janssen-Cilag

### ESR/EFSUMB-4
**Application of ultrasound in intra-articular pathology**
Esperanza Naredo; Madrid/ES
*(enaredo@ser.es)*

**Learning Objectives:**
1. To review the main intra-articular indications for musculoskeletal ultrasound (including injections).
2. To exemplify the main ultrasound-evaluable intra-articular pathologies
3. To present the standardised ultrasound assessment of these pathologies.

### ESR/EFSUMB-5
**Application of ultrasound in extra-articular pathology**
Daniela Fodor; Cluj-Napoca/RO
*(dfodor@umfcluj.ro)*

**Learning Objectives:**
1. To review the main extra-articular indications for musculoskeletal ultrasound.
2. To exemplify the tendon, enthesis, and muscle pathology.
3. To present the current knowledge in these pathologies.

### ESR/EFSUMB-6
**US elastography in MSK: is there a role?**
Elena E. Drakonaki; Iraklion/GR
*(drakonaki@yahoo.gr)*

**Learning Objectives:**
1. To present the basic technique of EUS in MSK.
2. To present the current indications in MSK based on the recent EFSUMB guidelines.
3. To discuss future directions and potential applications in MSK.

**Live Q&A**

08:00-09:00 CHANNEL 5

### Multidisciplinary Session

### MS 4

### Rectal cancer treatment: which are the clinically relevant questions?

**Moderator**
Regina G.H. Beets-Tan; Amsterdam/NL

### MS 4-2
**From the surgeons**
Geerard L. Beets; Amsterdam/NL

**Learning Objectives:**
1. To understand the relevant clinical questions.
2. To learn about the current status of rectal cancer surgery and organ preservation.
3. To learn what we can expect in the years to come.

### MS 4-3
**From the radiation oncologists**
Corrie Marijnen; Amsterdam/NL

**Learning Objectives:**
1. To become familiar with the various neoadjuvant treatment strategies (long or short course chemoradiotherapy, induction, or consolidation chemo).
2. To learn about the value of these strategies in the era of organ preservation.

### MS 4-4
**Multidisciplinary case presentation**
Regina G.H. Beets-Tan; Amsterdam/NL
*(r.beetstan@nki.nl)*

**Live Q&A**

09:15-10:15 CHANNEL 2

### E³ - ECR Master Class

### E³ 1226

### Artificial intelligence (AI) applications in the growing body

**Moderator**
Tomas Sakinis; Oslo/NO

### E³ 1226-2
**A. In times of limited data**
Tomas Sakinis; Oslo/NO
*(sakinis.tomas@gmail.com)*

**Learning Objectives:**
1. To understand the basic principles of big data collection.
2. To learn about the organisation of big data.
3. To discuss the challenges in obtaining and managing big data in the paediatric population.

**Author Disclosures:**
Tomas Sakinis: CEO: Artificial Intelligence AS; Owner: Artificial Intelligence AS

### E³ 1226-3
**B. Neuroimaging**
Stavros Stivaros; Manchester/UK
*(Stavros.Stivaros@manchester.ac.uk)*

**Learning Objectives:**
1. To understand the principles of AI applications in paediatric neuroimaging.
2. To learn about how deep learning networks can be used for segmentation, detection, and characterisation in paediatric neuroimaging.
3. To discuss the challenges of AI in paediatric neuroimaging.

### E³ 1226-4
**C. Current and emerging chest applications**
Steven Schalekamp; Nijmegen/NL

**Learning Objectives:**
1. To understand the current spectrum of available AI applications for chest radiology.
2. To learn about the emerging AI applications within pediatric chest radiology.
3. To discuss the future directions of AI in pediatric chest radiology.

**Live Q&A: How can paediatric radiologists keep up with adult radiologists in applying AI to clinical practice?**

09:15-10:15 CHANNEL 5

### Special Focus Session

### SF 21

### Lung cancer screening implementation in Europe: where are we now?

**Moderator**
Annemiek Snoeckx; Antwerp/BE

### SF 21-2

**Pivotal aspects for a successful lung cancer screening programme**
Annemiek Snoeckx; Antwerp/BE
*(Annemiek.Snoeckx@uza.be)*

**Learning Objectives:**
1. To describe those key elements that are essential for lung cancer screening programmes to be successful and impact on the care of those who are potentially screened.
2. To highlight the multifaceted role of radiologists in a lung cancer screening programme.

### SF 21-3

**Croatia**
Ante Marusic; Zagreb/HR
*(ante.marusic@kbc-zagreb.hr)*

**Learning Objectives:**
1. To demonstrate the complexity of preparation, implementation, and management of a national lung cancer screening program (LCS).
2. To demonstrate the structure of the national LCS in Croatia through integrated care.
3. To demonstrate the applied quality control in the ongoing LCS in Croatia.

### SF 21-4

**Hungary**
Anna Kerpel-Fronius; Budapest/HU
*(kerpel.anna@gmail.com)*

**Learning Objectives:**
1. To learn about implementing screening from a one-centre study to several institutions.
2. To understand Hungary-specific aspects of screening.
3. To become familiar with the Hungarian LDCT LCS implementation programme.

### SF 21-5

**Poland**
Mariusz Adamek; Gdansk/PL
*(mariusz.adamek@gmail.com)*

**Learning Objectives:**
1. To demonstrate how a nationwide programme may originate from a couple of local screening programmes.
2. To indicate the importance of proper structuring of a programme.
3. To become familiar with the cloud-based AI-powered platform created for the Polish programme.
**Author Disclosures:**
Mariusz Adamek: Advisory Board: Medtronic; Share Holder: NovellusDx

### SF 21-6

**Russia**
Sergey Morozov; Moscow/RU

**Learning Objectives:**
1. To learn about the different stages of implementing a lung cancer screening programme in Russia.
2. To show the role of artificial intelligence in lung cancer screening.
3. To identify the transition problems from a pilot project to a large-scale programme and possible solutions.
**Author Disclosures:**
Sergey Morozov: Advisory Board: Philips, Agfa; CEO: CDT Moscow; Speaker: Siemens

### SF 21-7

**France**
Marie-Pierre Revel; Paris/FR
*(marie-pierre.revel@aphp.fr)*

**Learning Objectives:**
1. To provide an overview of the ongoing lung cancer screening studies in France.
2. To learn about the pilot programme conducted in the Somme district.
3. To explain how French radiologists are getting prepared for large scale screening.
**Author Disclosures:**
Marie-Pierre Revel: Other: lecture fees from MSD Francen Travel support from Guerbet

### SF 21-8

**Germany**
Claus Peter Heussel; Heidelberg/DE

**Learning Objectives:**
1. To provide an update on the lessons learned from LC-screening in Germany so far.
2. To understand the German-specific aspects of screening organisation and radiation protection and funding.
3. To become familiar with the German LC-screening future.

### SF 21-9

**United Kingdom**
Anand Devaraj; London/UK

**Learning Objectives:**
1. To understand the current progress in lung cancer screening implementation in the UK.
2. To understand training and radiology requirements for reading in the UK.
3. To review the challenges and successes of implementation to date.
**Author Disclosures:**
Anand Devaraj: Advisory Board: Boehringer Ingelheim; Employee: Brainomix

**Live Q&A: What are the remaining obstacles for LCS implementation?**

10:30-11:30 CHANNEL 2

### Refresher Course: Breast

### RC 102

### Breast tomosynthesis in screening: where do we stand?

**Moderator**
Sophia Zackrisson; Malmö/SE

### RC 102-2

**A. Implementation of DBT in screening in the US**
Emily Conant; Philadelphia, PA/US
*(Emily.Conant@pennmedicine.upenn.edu)*

**Learning Objectives:**
1. To review the US retrospective studies on DBT in screening.
2. To understand how DBT works in different screening settings.
3. To be oriented about the current use of DBT in screening in the US.
**Author Disclosures:**
Emily Conant: Advisory Board: Hologic, Inc.; Research Grant/Support: Hologic, Inc.

### RC 102-3

**B. Evidence from the prospective trials of DBT**
Nehmat Houssami; Sydney, NSW/AU

**Learning Objectives:**
1. To review the prospective trials of DBT.
2. To discuss the strengths and limitations of DBT in screening.
3. To understand what evidence is needed to implement DBT in a population screening setting.

**RC 102-4**
**C. Optimisation of reading DBT: an overview**
Ioannis Sechopoulos; Nijmegen/NL
*(ioannis.sechopoulos@radboudumc.nl)*

**Learning Objectives:**
1. To learn about the different reading protocols that can be used in DBT screening.
2. To learn how different reading strategies may impact detection, recalls, and reading time.
3. To learn about possible approaches to optimise DBT reading.

**Author Disclosures:**
Ioannis Sechopoulos: Research Grant/Support: Siemens Healthcare, ScreenPoint, Sectra Benelux, Volpara Solutions, Canon Medical; Speaker: Siemens Healthcare

**Live Q&A: Has the time come to introduce screening with DBT in Europe?**

**10:30-11:30 CHANNEL 3**

### State of the Art Symposium

### SA 3

### Assessment of incidental and screen-detected pulmonary nodules

**Moderators**
Mathias Prokop; Nijmegen/NL
Ieneke Hartmann; Rotterdam/NL

### SA 3-3
**Diagnosis and overdiagnosis of subsolid nodules**
Arjun Nair; London/UK

**Learning Objectives:**
1. To become familiar with the characteristics of subsolid nodules and their risk of cancer.
2. To learn about the differential diagnosis of subsolid nodules.
3. To understand current management strategies for the diagnosis, classification, and follow-up of subsolid nodules.

**Author Disclosures:**
Arjun Nair: Advisory Board: Aidence BV; Other: Part funded by BRC UCLH

### SA 3-4
**Diagnosis and follow-up of solid nodules**
Anna Rita Larici; Rome/IT
*(annarita.larici@unicatt.it)*

**Learning Objectives:**
1. To become familiar with the characteristics of solid nodules and their risk of cancer.
2. To learn about the differential diagnosis of solid nodules.
3. To understand current management strategies for the diagnosis, classification, and follow-up of solid nodules.

### SA 3-5
**Radiomics and AI performance in lung nodule assessment**
Guillaume Chassagnon; Paris/FR

**Learning Objectives:**
1. To become familiar with the deep learning methods used and their possibilities.
2. To learn what the current state-of-the-art applications of AI are in detection, classification, and follow-up of pulmonary nodules.
3. To appreciate what the impact of these advancements may be on the role of radiologists.

**Live Q&A: The requirements of a nodule MDT board**

**10:30-11:30 CHANNEL 4**

### ESR meets Armenia

### Meets 4

### Blooming radiology in an ancient nation

**Moderators**
Michael H. Fuchsjäger; Graz/AT
Kostan Galumyan; Yerevan/AM

### Meets 4-3
**Introduction: History of Armenian radiology**
Kostan Galumyan; Yerevan/AM
*(kostmed@rambler.ru)*

**Learning Objectives:**
1. To learn about the history and current state of radiology in Armenia.
2. To learn about the educational system and opportunities for residents in Armenia.
3. To get acquainted with typical clinical cases in Armenian radiology practice.
4. To become familiar with the achievements of radiology and its perspectives in Armenia.

### Meets 4-4
**Interlude 1: Armenian folk music**

### Meets 4-5
**Practicing modern radiology in Armenia**
Hovhannes Vardevanyan; Yerevan/AM
*(hovovar@gmail.com)*

**Learning Objectives:**
1. To learn about specific cases predominantly observed in Armenia.
2. To become familiar with everyday workflow of Armenian radiological clinics.
3. To learn about the difficulties faced by radiologists in Armenia.

### Meets 4-6
**Interlude 2: Armenian folk dances**

### Meets 4-7
**New opportunities for radiology residents**
Meri Talanyan; Yerevan/AM
*(marytalanyan@gmail.com)*

**Learning Objectives:**
1. To learn about challenges of radiology residents in Armenia.
2. To become familiar with residency programmes and opportunities.

### Meets 4-8
**Interlude 3: Armenian culinary treats**

### Meets 4-9
**Future vision of Armenian radiology**
Narek Matinyan; Yerevan/AM
*(matinyan@doctor.com)*

**Learning Objectives:**
1. To learn about perspectives for improving radiological practice in different regions of Armenia.
2. To learn about the potential of artificial intelligence in Armenian radiology.
3. To become familiar with new ideas for radiology education.

**Live Q&A: New paths for growth of the Armenian radiology**

**10:30-11:30** **CHANNEL 5**

### Research Presentation Session: Genitourinary

### RPS 207

### Up-to-date reporting and data systems (RADS) in the pelvis

**Moderator**
Subramaniyan Ramanathan; Doha/QA

### RPS 207-2

**Diagnostic performance of ultrasound based ACR O-RADS versus IOTA simple rules risk (SRrisk) calculation tool for malignancy risk stratification of adnexal pathology**
*S. B. Grover*, S. Patra, H. Grover, P. Mittal, G. Khanna; New Delhi/IN
*(shabnamgrover@yahoo.com)*

**Purpose:** The aim of this study was to compare the diagnostic performance between ultrasound (US) based ACR O-RADS (ovarian-adnexal reporting and data system) & IOTA simple rules risk (SRrisk) calculation tool score for assigning malignancy risk in patients with adnexal pathology.
**Methods or Background:** In this Institutional Review Board approved prospective study, 102 consenting patients with adnexal tumors were recruited. All patients underwent US examination on the same equipment, by two different evaluators: first & second author. O-RADS risk category & IOTA SRrisk calculation tool score was assigned to each tumor by consensus. Reference standard was histopathology obtained from biopsied/operated tumors; for the rest, it was a biannual survey for 12 months. Sensitivity, specificity, positive & negative predictive values, & diagnostic accuracy was compared between both risk stratification systems.
**Results or Findings:** Of the evaluated tumors, 40 were benign & 62 were malignant. O-RADS risk classification could be applied to all evaluated tumors, IOTA SRrisk was not applicable in two of the evaluated tumors. Overall sensitivity, specificity, PPV & NPV for ACR O-RADS was found to be 100%, 55%, 77.5% & 100%, while for IOTA SRrisk calculation tool it was 90%, 80%, 87.1% & 84.2% respectively. Diagnostic accuracy for IOTA SRrisk calculation tool was 86%, for ACR O-RADS, 82.6%.
**Conclusion:** Both ACR O-RADS & IOTA SRrisk calculation tool classification show good diagnostic performance for malignancy risk stratification of adnexal tumors. Although, diagnostic accuracy of IOTA SRrisk calculation tool is better than ACR O-RADS, the latter is superior for prediction of benignity. Definitive classification of benignity is critical, since these tumors need only conservative management.
**Limitations:** The limitation in our study was a relatively small sample size and patients being evaluated from a single Institution.
**Ethics Committee Approval:** Yes.
**Funding for this study:** Not Applicable
**Author Disclosures:**
Sayantan Patra: Nothing to disclose
Shabnam Bhandari Grover: Nothing to disclose
Geetika Khanna: Nothing to disclose
Hemal Grover: Nothing to disclose
Pratima Mittal: Nothing to disclose

### RPS 207-5

**Preoperative detection of VI-RADS score 5 reliably identifies extravesical extension of urothelial carcinoma of the bladder and predicts significant delayed time to cystectomy**
M. Bicchetti, *M. Pecoraro*, F. Del Giudice, E. Messina, C. Catalano, V. Panebianco; Rome/IT
*(pecoraro.martina1@gmail.com)*

**Purpose:** (I)To determine VI-RADS score 5 accuracy in predicting locally advanced bladder cancer (BCa), to identify those patients who could avoid the morbidity of deep TURBT in favour of histological sampling-TUR prior to radical cystectomy (RC). (II)To explore the predictive value of VI-RADS score 5 on time-to-cystectomy outcomes.
**Methods or Background:** We retrospectively reviewed patients' ineligible or refusing cisplatin-based combination neoadjuvant chemotherapy who underwent mpMRI of the bladder prior to staging TURBT followed by RCfor muscle-invasive BCa. Sensitivity, specificity, PPV and NPV were calculated for VI-RADS score 5 vs. score 2–4 cases to assess the accuracy of mpMRI for extravesical BCa detection (≥pT3). VI-RADS scoreperformance was assessed by ROC curve analysis. K statistic was calculated for mpMRI and pathological diagnostic agreement.The risk of delayed TTC was assessed using multivariable logistic regression model.
**Results or Findings:** 149 T2–T4a, cN0–M0 patients included. VI-RADS 5 demonstrated sensitivity, specificity, PPV and NPV, in detecting extravesical disease of 90.2% (95% CI 84–94.3), 98.1% (95% CI 94–99.6), 94.9% (95% CI 89.6–97.6) and 96.4% (95% CI 91.6–98.6). The AUC was 94.2% (95% CI 88.7–99.7) and inter-reader agreement was Kinter 0.89. Mean (SD) TTC was 4.2 (2.3) and 2.8 (1.1) months for score 5 vs 2–4, ($P<0.001$). VI-RADS score 5 independently increased risk of delayed TTC (odds ratio 2.81, 95% CI 1.20–6.62).
**Conclusion:** VI-RADS is valid and reliable in differentiating patients with extravesical disease from those with muscle-confined BCa before TURBT. Detection of VI-RADS 5 was found to predict significant delay in TTC independently from other clinicopathological features.
**Limitations:** (I)Retrospective single centre study with a limited number of observed locally advanced cases. (II)diagnostic performance and the effect of predicting factors (ORs) could be influenced by the sample size.
**Ethics Committee Approval:** EC approved
**Funding for this study:** Not applicable
**Author Disclosures:**
Emanuele Messina: Nothing to disclose
Marco Bicchetti: Nothing to disclose
Valeria Panebianco: Nothing to disclose
Martina Pecoraro: Nothing to disclose
Francesco Del Giudice: Nothing to disclose
Carlo Catalano: Nothing to disclose

### RPS 207-6

**ORADS: a winding road of standardisation and an aid in differentiating benign ovarian masses from malignant ones - how and why?**
*S. K. Kale*, S. Sharma, S. Rahman, A. M. Nizar, S. Khan; Bengaluru/IN
*(drsudhir.kale@asterhospital.com)*

**Purpose:** To familiarize radiologist with Ovarian-Adnexal Reporting and Data System (O-RADS).
ORADS will lead to standardization in reporting, higher accuracy in classification of lesion as malignant or benign, and to provide a basic management recommendation.
**Methods or Background:** Most of the incidentally detected ovarian lesions are cystic, benign and asymptomatic and often resolve spontaneously. Only few ovarian lesions are suspicious and rarely malignant. USG is a preliminary imaging tool but may have different interpretations among radiologists regarding complexity. Classifying them according to risk of malignancy can decrease the number of further unnecessary imaging.
**Results or Findings:** Ovarian-Adnexal Reporting is a new and effective lexicon which uses a non-invasive, readily available, cheap imagining modality USG to suggest likelihood of malignancy and providing guidance for management.
**Conclusion:** Unlike other lexicon O-RADS is developed for the patient at average risk of malignancy which increases sensitivity of detection of ovarian carcinoma.
**Limitations:** Not applicable
**Ethics Committee Approval:** Not applicable
**Funding for this study:** Not applicable
**Author Disclosures:**
Sayana Rahman: Nothing to disclose
Sukrity Sharma: Nothing to disclose
Saarah Khan: Nothing to disclose
Sudhir Kumar Kale: Nothing to disclose
Ajmal Mohamed Nizar: Nothing to disclose

### RPS 207-7

**Revisiting O-RADS: an illustrated guide with magnetic resonance correlation**
*L. d. A. Defendi*, V. M. Brito, F. M. A. Coelho, E. E. Dutenhefner, A. Talans, R. Baroni; São Paulo/BR
*(lariad@gmail.com)*

**Purpose:** 1. Conduct a brief review of American and European guidelines on diagnosis and management of adnexal lesions;
2. Describe the imaging features of main benign and malignant adnexal lesions on ultrasound (US) and magnetic resonance (MRI);
3. Review the O-RADS lexicon
4. Illustrate the risk classification system with teaching cases
**Methods or Background:** A reliable imaging method to approach adnexal lesions would reduce the number of unnecessary laparotomies in benign cases. O-RADS is a recently published classification of malignancy risk that presents a standardized lexicon to describe the sonographic aspects of lesions involving the ovaries or tubes. Magnetic resonance imaging is widely used in the follow-up of adnexal lesions and in cases of diagnostic doubts since it is highly sensitive in assessing their features and thus determining their malignancy.

**Results or Findings:** The following topics will be discussed:1. Review of American and European guidelines: GI-RADS Risk Classification,O-RADS Risk Classification, ADNEx MR Scoring System, ACOG Practice Bulletin on Evaluation and Management of Adnexal Masses2. MRI protocols - evaluation of the female pelvis3. O-RADS risk classification system: Understanding the lexicon, Illustrated O-RADS categories4. Sonographic findings with MRI correlations: Endometrioma, Hydrosalpinx, Ovarian torsion mimicking adnexal tumor, Fibroma, Teratoma, Cystadenofibroma, Serous Cystadenoma, Serous Cystadenocarcinoma, Mucinous cystadenoma, Endometrioid carcinoma, Serous Mucinous Borderline tumor, Imature teratoma, Leydig cell tumor5. Current state of the literature regarding O-RADS use
**Conclusion:** Radiologists must be familiar with the imaging aspects of various adnexal lesions and the proposed risk classification systems to ensure an accurate diagnosis and avoid unnecessary surgical procedures.
**Limitations:** Educational Exhibit
**Ethics Committee Approval:** Educational Exhibit
**Funding for this study:** This Educational Exhibit did not receive funding.
**Author Disclosures:**
Fernando Morbeck Almeida Coelho: Nothing to disclose
Vanessa Mizubuti Brito: Nothing to disclose
Eliane Eliza Dutenhefner: Nothing to disclose
Aley Talans: Nothing to disclose
Ronaldo Baroni: Nothing to disclose
Larissa de Andrade Defendi: Nothing to disclose

**Live Q&A**

**11:45-12:45 — CHANNEL 2**

### RTF Quiz

### Beating heart of radiology

**Moderators**
Marco Francone; Rome/IT
*(marco.francone@uniroma1.it)*
Christian Loewe; Vienna/AT
*(christian.loewe@meduniwien.ac.at)*
**Author Disclosures:**
Christian Loewe: Author: BRACCO Springer; Speaker: Siemens Healthineers

**11:45-12:45 — CHANNEL 3**

### Refresher Course: Oncologic Imaging

### RC 416

### Cancer becoming a chronic disease: role of imaging and radiomics

**Moderator**
Melvin D'Anastasi; Msida/MT

### RC 416-2
**A. Imaging surveillance: the pros and the cons**
Christiane K. Kuhl; Aachen/DE
*(ckuhl@ukaachen.de)*

**Learning Objectives:**
1. To understand which imaging techniques are suitable for cancer surveillance.
2. To learn when to perform surveillance imaging in different cancers.
3. To become familiar with the limitations of surveillance imaging.

**Author Disclosures:**
Christiane K. Kuhl: Speaker: Guerbet, Bracco, Bayer

### RC 416-3
**B. Early detection of prostate cancer recurrence**
Hebert Alberto Vargas; New York, NY/US

**Learning Objectives:**
1. To understand the clinical impact of early recurrence detection.
2. To learn the role of MRI for early lesion detection.
3. To learn the complementary value of PET.

### RC 416-4
**C. Radiomics for outcome prediction and prognostication**
Georg Langs; Vienna/AT

**Learning Objectives:**
1. To become familiar with radiomics analysis techniques in oncologic imaging.
2. To understand the concept and methodologies for image-based outcome prediction.
3. To learn how to integrate radiomics results and clinical-risk models for prognostication.

**Author Disclosures:**
Georg Langs: Employee: contextflow GmbH; Founder: contextflow GmbH; Research Grant/Support: Novartis, Siemens Healthineers, NVIDIA; Share Holder: contextflow GmbH

**Live Q&A: How to generate evidence for imaging in cancer surveillance?**

**11:45-12:45 — CHANNEL 4**

### ESR eHealth and Informatics Subcommittee Session

*Jointly organised with the ESR Patient Advisory Group (ESR-PAG)*

### ESR eHealth

### How to enhance radiologist-patient communication through IT

**Moderators**
Birgit Bauer; Abensberg/DE
Peter Mildenberger; Mainz/DE

### ESR eHealth-3
**Using patient portals for communicating with patients**
Christoph D. Becker; Geneva/CH

**Learning Objectives:**
1. To understand the communication needs of patients and radiologists.
2. To learn how patient portals are used to collect patient data and consent.
3. To know how patient portals can help to communicate results.

### ESR eHealth-4
**How to enhance patients' perception of radiology**
Birgit Bauer; Abensberg/DE

**Learning Objectives:**
1. To understand how to use patient portals for the communication with patients.
2. To understand how patients perceive radiology and how to enhance patient's perception of radiology.
3. To learn how technology can help radiologists to team up with patients.

### ESR eHealth-5
**Teaming up with our patients**
Elmar Kotter; Freiburg/DE
*(elmar.kotter@uniklinik-freiburg.de)*

**Learning Objectives:**
1. To learn how IT can be used to reduce information collection from the patient.
2. To understand the principle of chatbots and to analyse their potential value for radiology.
3. To explain how reports adapted to patients needs can be derived from standard reports.

**Live Q&A: Communication between patients and radiologists in five years**

11:45-12:45 CHANNEL 5

### Research Presentation Session: Paediatric

### RPS 212

### New insight into classical problems in paediatric radiology

**Moderator**
Thekla von Kalle; Stuttgart/DE

### RPS 212-2

**Bone marrow quantitative MRI in children with leukaemia**

*N. Kriventsova*, D. Kupriyanov, P. Menshchikov, D. Litvinov, G. Novichkova, G. Tereshchenko; Moscow/RU
*(nataliastrumila@gmail.com)*

**Purpose:** The aim of this study was to establish a normal bone marrow FF values in healthy children and changes in this parameter in patients with acute leukemia (AL).
**Methods or Background:** Our research contained 16 healthy volunteers from 7 to 16 years (median of age 13.1±2.9 years) and 17 patients with acute leukemia from 4 to 17 years (median of age 11.5±2.7 years). The study was performed using Philips Achieva 3T MRI scanner. FF values in both patient and control groups were evaluated using 2 methods: mDixon-Quant (TR = 6.5 ms, 6 echo times starting from TE = 1.2 ms with 0.8 ms increment, coronal slices which include Ilium) and proton single-voxel MR spectroscopy (PRESS, TE/TR = 39/2000 ms ). Spectroscopic voxels in size of 10×10×10 mm were located in the right Ilium bone. FF values were quantified using built-in Philips console programs for both methods.
**Results or Findings:** The FF in the bone marrow of healthy volunteers by the method of mDixon-Quant was 45.66% ± 10.27%, and by spectroscopy – 36.07% ± 12.22%. In children with the AL, the content of fat decreases sharply to 3.08% ± 2.6% in mDixon-Quant and to 3.14% ± 3.0% in spectroscopy. Difference between the parameters of healthy volunteers and patients with leukemia according to each method was statistical significance by T-test ($p \leq 0.01$). Two methods had strong correlation (Pearson's r = 0,82, p<0,05).
**Conclusion:** Bone marrow fat fraction values in patients with acute leukemia (AL) is significantly lower than in healthy children. mDixon-Quant and spectroscopy are effective for estimating the fat fraction in the bone marrow.
**Limitations:** No limitations
**Ethics Committee Approval:** The study was approved by the Ethics Committee.
**Funding for this study:** The study was not funded
**Author Disclosures:**
Dmitry Litvinov: Nothing to disclose
Petr Menshchikov: Nothing to disclose
Galina Tereshchenko: Nothing to disclose
Galina Novichkova: Nothing to disclose
Dmitry Kupriyanov: Nothing to disclose
Nataliia Kriventsova: Nothing to disclose

### RPS 212-3

**The bright bronchus sign in in-vivo foetal MRI of congenital lung lesions: a multi-center study**

*F. Prayer*[1], A. Mehollin-Ray[2], C. Cassady[2], G. Gruber[3], A. Scharrer[1], A. Hojreh[1], H. Prosch[1], D. Prayer[1], G. Kasprian[1]; [1]Vienna/AT, [2]Houston, TX/US, [3]Krems an der Donau/AT

**Purpose:** To characterize the 'bright bronchus sign' (BBS) in in-vivo fetal MRI of a multi-center cohort of fetuses with congenital lung lesions.
**Methods or Background:** For this retrospective multi-center study, 292 fetuses and 381 fetal MRI scans were allocated to two groups: group A with, and group B without postnatal CT and/or pathologic lesion workup. Fetal MRI scans were reviewed for the BBS, i.e. a tubular or branching T2-hyperintense structure close to the pulmonary vessels within a lung lesion. Postnatal CTs were reviewed for bronchial obstruction. BBS frequency in different lung lesions, gestational ages, and sensitivity, specificity, PPV, NPV and acccuracy of the BBS with regard to bronchial obstruction on CT were analyzed.
**Results or Findings:** In group A (64 fetuses, 96 MRI scans), BBS was found at 23+6 (20+1 - 38+3) GW in one of 25 congenital pulmonary airway malformation, 14 of 15 bronchial atresia, 9 of 17 bronchopulmonary sequestration, two of six hybrid lesion, and none of one bronchogenic cyst cases. Sensitivity, specificity, PPV, NPV, and accuracy of the BBS for isolated or BPS-associated bronchial obstruction were 64.9%, 95.7%, 96.0%, 62.9%, and 76.7%, respectively. In group B (228 fetuses, 285 fetal MRI scans), BBS was found at 26+6 (19+2 to 36+3) GW in 57 of 75 BA, 55 of 74 BPS, seven of 55 CPAM, six of eleven HL, two of nine BC, and four of four CHAOS cases.
**Conclusion:** The BBS is a frequent and highly specific fetal MR image marker of isolated or bronchopulmonary sequestration-associated bronchial obstruction, and may improve prenatal differential diagnosis of congenital lung lesions.
**Limitations:** Fetal MRI scans from different centers were retrospectively reviewed by different radiologists.
**Ethics Committee Approval:** This study was approved by the respective institutional review boards.
**Funding for this study:** This study received no funding.
**Author Disclosures:**
Helmut Prosch: Nothing to disclose
Florian Prayer: Nothing to disclose
Anke Scharrer: Nothing to disclose
Christopher Cassady: Nothing to disclose
Amy Mehollin-Ray: Nothing to disclose
Azadeh Hojreh: Nothing to disclose
Gerlinde Gruber: Nothing to disclose
Daniela Prayer: Nothing to disclose
Gregor Kasprian: Nothing to disclose

### RPS 212-4

**Contrast-free MRI protocol for pituitary assessment in children with growth or puberty disorders**

*M. Michali Stolarska*[1], A. Tukiendorf[2], A. Zacharzewska-Gondek[1], J. Chrzanowska[2], J. Jaców[1], J. Bladowska[1]; [1]Wrocław/PL, [2]Wroclaw/PL
*(marta.michali@gmail.com)*

**Purpose:** Most of the pituitary MRI examinations in children with growth or puberty disorders (GPD) might not require gadolinium-based contrast agent (GBCA) administration.
**Methods or Background:** Retrospective single-center study that includes re-analysis of contrast-enhanced pituitary MRI examinations of a group of children treated in a tertiary reference center.The group of 567 patients met the following inclusion criteria: age below 18 years; growth or puberty disorders. MRI examinations were divided into: Part1 - before contrast administration (reference group) and Part2 - after contrast administration. The differences in the visibility of focal lesions between groups were statistically analyzed.
**Results or Findings:** The overall frequency of Rathke's cleft cysts was 11.6%, ectopic posterior pituitary 3.5% and microadenomas 0.9%. Lesions visible before contrast administration accounted for 85% of cases. Lesions, which were not visible before and diagnosed only after contrast injection accounted for only 0.18% of all patients. Statistical analysis showed the advantage of antero-posterior (AP) pituitary dimension over other criteria in determining the appropriateness of using contrast in pituitary MRIs. The AP dimension was the most significant factor in logistic regression analysis: OR=2.23, 95%CI, 1.35 to 3.71, p-value=0.002 and in ROC analysis: AUC:72.9% with cut-off value 7.5 mm, with sensitivity/specificity rates: 69.2%/73.5%.
**Conclusion:** In most cases, the use of GBCA in pituitary MRI in children with GPD is unnecessary.
The advantages of GBCA omission include: shortening the time of MRI examination and of general anesthesia; saving time for other examinations, thus increasing the availability of MRI for waiting children and acceleration in their further clinical management.
**Limitations:** First, it has a retrospective design; second, none of our patients underwent surgery, therefore histopathological confirmation of the diagnoses made by MRI was not possible.
**Ethics Committee Approval:** Local ethics committee approval - opinion No. 117/2019
**Funding for this study:** None
**Author Disclosures:**
Marta Michali Stolarska: Nothing to disclose
Andrzej Tukiendorf: Nothing to disclose
Jagoda Jaców: Nothing to disclose
Joanna Chrzanowska: Nothing to disclose
Anna Zacharzewska-Gondek: Nothing to disclose
Joanna Bladowska: Nothing to disclose

### RPS 212-6

**Does sevoflurane sedation or intravenous propofol administration in children lead to "pseudo" leptomeningeal enhancement in the brain on 3-Tesla MRI?**

*K. Khandwala*, K. Hilal, S. S. M. Anwar, F. Khan, S. Rashid; Karachi/PK

**Purpose:** The rationale of our study is prominent leptomeningeal contrast enhancement (LCME) is noted on MRI in some sedated children, but based on clinical notes and CSF analysis, they neither were acutely ill nor exhibited meningeal signs. Our study aims to find out whether sevoflurane sedation or propofol anesthesia in children leads to 'pseudo' LCME on 3 Tesla MRI.
**Methods or Background:** A retrospective cross sectional study was conducted in which MRI examinations performed for pediatric patients (aged 0-8 years) on a 3 Tesla system were reviewed. The degree of leptomeningeal contrast enhancement (LCME) was graded and evaluated by two radiologists. Interobserver scores were calculated by using Cohen's Kappa. Correlations were assessed using Pearson's correlation test.
**Results or Findings:** A total of 63 pediatric patients were ultimately included. 42 patients were sedated with sevoflurane, 15 were anesthetized with propofol and 6 with a combination of both. There were statistically significant and inverse moderate correlations between patient age and LCME grade, and between weight and LMCE grade (r 0.35 each, P<0.05). Neither the type of anesthesia administered (sevoflurane vs propofol) nor the sedation dosage or duration significantly correlated with the LMCE grade. Almost perfect interobserver agreement (Kappa: 0.83) was found between the two radiologists for degree of LCME.
**Conclusion:** Pseudo LMCE is relatively common on post contrast MR imaging of children sedated by intravenous propofol or inhalational sevoflurane, and therefore should not be mistaken for meningeal pathology. We found that this effect inversely correlates with age and weight. This phenomenon may be attributable to the immaturity of younger children's vasculature or increased permeability of the blood brain barrier, but needs to be studied further with larger prospective cohorts.
**Limitations:** Retrospective single center study, with selection biases.
**Ethics Committee Approval:** Taken.
**Funding for this study:** None.
**Author Disclosures:**
Kiran Hilal: Nothing to disclose
Kumail Khandwala: Nothing to disclose
Faheemullah Khan: Nothing to disclose
Shayan Sirat Maheen Anwar: Nothing to disclose
Saima Rashid: Nothing to disclose

### RPS 212-7

**Audit of yield of magnetic resonance imaging in idiopathic scoliosis**
*A. T. Foran*, M. Hanley, D. Mulholland, S. Ryan; Dublin/IE

**Purpose:** Adolescent Idiopathic Scoliosis(IS) relates to a curvature of the spine with an unknown aetiology that occurs in 1-5.4% of children. Established guidelines for the use of MRI in AIS do not exist. Some literature proposes that all children with idiopathic scoliosis should have MRI with a yield of significant findings up to 15%. Others recommend MRI for specific indications only such as an atypical curve, curves of a significant magnitude or those with clinical neurological findings on examination
**Methods or Background:** We performed a retrospective review of patients undergoing an initial MRI for AIS over a 4 year period to assess the yield of performing MRI in these patients. We included patients aged 12 -16, referred through the National Scoliosis referral pathway to our service. We also identified clinical findings or risk factors that may be associated with a positive finding. We categorised patients by the King-Moe Classification and the Lenke Classification.
**Results or Findings:** 159 MRIs performed for presumed adolescent idiopathic scoliosis were identified during the 4 year period November 2015-November 2019. 18 of these had positive findings including Chiari I malformation, syrinx, cerebellar tonsillar ectopia and spondylolisthesis.
**Conclusion:** Indications for MRI scanning in presumed adolescent idiopathic scoliosis remains unclear. 9% of patients in our cohort had clinical findings which could potentially change management. Based on these findings it seems reasonable to perform an MRI in this patient population to assess for underlying abnormalities to help guide management decisions.
**Limitations:** Our study is limited as it is a retrospective study.
**Ethics Committee Approval:** Local approval from the ethics committee was obtained for this audit.
**Funding for this study:** None
**Author Disclosures:**
Ann Therese Foran: Nothing to disclose
Stephanie Ryan: Nothing to disclose
Douglas Mulholland: Nothing to disclose
Marion Hanley: Nothing to disclose

### RPS 212-8

**Ultrasound (US) and magnetic resonance imaging (MRI) predictive role of long-term medical outcome in native liver survivor patients with biliary atresia (BA) after kasai portoenterostomy (KP)**
*M. Caruso*, F. Di Dato, R. Liuzzi, C. Mollica, V. Romeo, G. Vallone, P. P. Mainenti, R. Iorio, S. Maurea; Naples/IT (caruso.martina90@gmail.com)

**Purpose:** To assess the role of US and MRI in predicting long-term medical outcome in native liver survivor patients with BA after KP.
**Methods or Background:** BA, a progressive fibro-obliterative bile duct disease, leads to cirrhosis in absence of KP and/or liver transplantation. Patient follow-up includes clinical and laboratory parameters and abdominal US. MRI has also been proposed. Twenty-four retrospectively enrolled patients were divided in two groups according to clinical and laboratory data at initial evaluation after KP (median follow-up =9.7 years) as with ideal (Group 1; n=15) or non-ideal (Group 2; n=9) medical outcome. All patients were re-evaluated after 4 years using clinical and laboratory indices. US and MRI studies were qualitatively analyzed assessing imaging signs suggestive of chronic liver disease.
**Results or Findings:** At re-evaluation, 6 patients (40%) of Group 1 changed their medical outcome in non-ideal (Group 1A); the other 9 patients (60%) remained ideal (Group 1B). The AUC was 0.84 and 0.87 for US and MRI scores to predict long-term medical outcome with the best cut-off value score >4 for both modalities (p=0.89). In patients of Group 1, the mean time to change the medical outcome in non-ideal at re-evaluation was 43.5+/-2.3 months. In Group 2, 6 (67%) patients showed a clinical disease progression, while the other 3 (33%) patients were unchanged as non-ideal status.
**Conclusion:** In BA patients with ideal medical outcome after KP, US and MRI studies may both predict long-term medical outcome change.
**Limitations:** The study population is small, but BA is a relatively rare disease and the correlative availability of the imaging studies is limited. A larger, randomized prospective trial should be addressed.
**Ethics Committee Approval:** The local Institutional Review Board approved the study, the need for informed consent was waived.
**Funding for this study:** None.
**Author Disclosures:**
Fabiola Di Dato: Nothing to disclose
Valeria Romeo: Nothing to disclose
Raffaele Liuzzi: Nothing to disclose
Pier Paolo Mainenti: Nothing to disclose
Gianfranco Vallone: Nothing to disclose
Carmine Mollica: Nothing to disclose
Raffaele Iorio: Nothing to disclose
Martina Caruso: Nothing to disclose
Simone Maurea: Nothing to disclose

**Live Q&A**

**14:15-15:15 CHANNEL 2**

### Special Focus Session

### SF 12

### Liver transplantation: imaging and intervention

**Moderator**
Andrzej Cieszanowski; Warsaw/PL

### SF 12-2

**Preoperative: what to know**
Valérie Vilgrain; Clichy/FR

**Learning Objectives:**
1. To learn how imaging impacts on clinical decision making for liver transplantation.
2. To understand the selection criteria for transplantation in HCC patients.
3. To appreciate the role of biopsy and interventional radiology in pre-transplant patient management.

### SF 12-3

**Postoperative and follow-up: what to expect and what goes wrong**
Rossano Girometti; Udine/IT
*(rgirometti@sirm.org)*

**Learning Objectives:**
1. To appreciate the early and late complications of liver transplantation.
2. To understand the role of multimodality imaging for the diagnosis.
3. To understand the prognostic factors that may change patient management.

### SF 12-4

**The role of interventional radiology in treating complications**
Allan Bloom; Jerusalem/IL
*(allan@hadassah.org.il)*

**Learning Objectives:**
1. To learn about the complications of liver transplantation that are treatable through interventional radiology.
2. To understand the clinical results emphasising the failure rates of IR techniques.
3. To appreciate the future direction of IR in the liver.

**Live Q&A: Can radiology expand its role for patient management?**

**14:15-15:15** **CHANNEL 3**

### Refresher Course: Cardiac

### RC 203

### The radiologist at the centre of the heart team

**Moderator**
Tim Leiner; Utrecht/NL

### RC 203-2

**A. The value of CT/MRI in non-surgical cardiac valve replacement**
Rodrigo Salgado; Antwerp/BE
*(rodrigo.salgado@uza.be)*

**Learning Objectives:**
1. To understand the clinical indications for transcatheter interventions in different cardiac valves.
2. To understand the difficulties in correctly assessing cardiac valvular anatomy and its therapeutic implications.
3. To correctly identify and report key issues which may influence procedural success and patient prognosis.

### RC 203-3

**B. Diagnosing native and prosthetic heart valve endocarditis: how can the radiologist help?**
Gudrun Feuchtner; Innsbruck/AT
*(Gudrun.Feuchtner@i-med.ac.at)*

**Learning Objectives:**
1. To understand the aetiology and clinical presentation of endocarditis.
2. To understand the current guidelines for diagnosis and treatment and the role of CT/MRI imaging.
3. To become familiar with common and uncommon imaging presentations of endocarditis and its pitfalls.

**Author Disclosures:**
Gudrun Feuchtner: Advisory Board: Sanofi Aventis, Amgen

### RC 203-4

**C. The radiologist as a member of the heart team: what the guidelines say and future perspectives**
Jean-Nicolas Dacher; Rouen/FR
*(jndacher@gmail.com)*

**Learning Objectives:**
1. To understand the role of multidisciplinary heart teams.
2. To learn how heart teams are implemented in the current guidelines.
3. To evaluate the current position of the radiologist, and the challenges ahead.

**Author Disclosures:**
Jean-Nicolas Dacher: Consultant: Takeda, Bayer, Microport; Investigator: Takeda Shire, Microport; Speaker: Siemens, GE HealthCare, Bayer

**Live Q&A: How to empower the radiologist's clinical role in the setting of a heart team?**

**14:15-15:15** **CHANNEL 5**

### Research Presentation Session: Physics in Medical Imaging

### RPS 213

### Patient dose: the neverending story

**Moderator**
Ioannis Sechopoulos; Nijmegen/NL

### RPS 213-2

**Dose reduction in photon-counting CT by ultra-high resolution acquisitions compared to today's iterative reconstructions: combined benefits of the "iodine effect" and the "small pixel effect"**
*S. Sawall*, L. Klein, E. Wehrse, C. Amato, J. Maier, C. H. Ziener, H-P. Schlemmer, S. Heinze, M. Kachelrieß; Heidelberg/DE
*(stefan.sawall@dkfz.de)*

**Purpose:** To evaluate the potential noise/dose-reduction of an ultra-high resolution (UHR) photon-counting (PC) detector compared to today's iterative reconstructions of energy-integrating (EI) data.
**Methods or Background:** Images of an abdomen phantom with different sizes equipped with iodine vials were acquired at the SOMATOM CounT (Siemens Healthineers, Germany) CT system, an experimental CT housing an EI and a PC detector. 120 kV acquisitions were performed at matched CTDI with tube currents between 35 to 300 mAs using the EI detector and the UHR-mode of the PC detector. Resolution-matched reconstructions of EI data were performed using an iterative reconstruction (SAFIRE strength 1-5) while the PC data were reconstructed using filtered backprojection (FBP). The "small pixel effect" predicts a noise-reduction in the PC images and the "iodine effect" an increased iodine contrast in PC data. Noise and contrast-to-noise ratio (CNR) were compared to conventional FBP reconstructions of EI data at the same dose and spatial resolution. All results are verified in a post-mortem angiography study.
**Results or Findings:** UHR acquisitions allow for a dose reduction of 58% compared to EI-FBP at the same dose and resolution. EI-SAFIRE reconstructions allow for a dose reduction of 25%-87%. The noise level found in UHR reconstructions is similar to EI-SAFIRE(2) while the CNR is similar to EI-SAFIRE(3) due to the "iodine effect". These results hold for all phantom sizes and were confirmed in a post-mortem angiography study.
**Conclusion:** Thanks to the iodine effect and the small pixel effect, photon-counting UHR allow for a dose-reduction similar to iterative reconstructions while using conventional FBP thereby providing classical, well-defined image quality metrics.
**Limitations:** No patient data are available since the described experiments would require multiple measurements.
**Ethics Committee Approval:** The post-mortem experiments were approved by the local ethics committee (S-021/2020).
**Funding for this study:** Not applicable.
**Author Disclosures:**
Laura Klein: Nothing to disclose
Christian Herbert Ziener: Nothing to disclose
Sarah Heinze: Nothing to disclose
Carlo Amato: Nothing to disclose
Heinz-Peter Schlemmer: Nothing to disclose
Marc Kachelrieß: Nothing to disclose
Stefan Sawall: Nothing to disclose
Eckhard Wehrse: Nothing to disclose
Joscha Maier: Nothing to disclose

### RPS 213-3

**Radiation risk minimising tube current modulation (rmTCM) for x-ray computed tomography**
*J. Maier*[1], L. Klein[1], S. Sawall[1], C. Liu[2], A. Maier[2], M. Lell[3], M. Kachelrieß[1];
[1]Heidelberg/DE, [2]Erlangen/DE, [3]Nuremberg/DE

**Purpose:** Tube current modulation (TCM) aims at minimizing radiation dose while maintaining image quality. In contrast to clinical implementations that are based on the minimization of the mAs product, this study investigates the potential of modulating the tube current to minimize the radiation risk instead.
**Methods or Background:** TCM adjusts the tube current during gantry rotation as well as along z-direction. In today's clinical CT systems, the modulation curve is determined according to the patient attenuation that is estimated either from the topogram or by an online feedback loop. To extend the concept of TCM to also take into account the radiation risk, additional prior knowledge of the dose distribution is necessary. Here, we use the deep dose estimation (DDE) algorithm that estimates patient-specific dose distributions, and thus the effective dose, in real-time using a neural network. Including DDE in the conventional optimization routine allows to determine a TCM curve that minimizes the radiation risk. The performance of the proposed approach was evaluated for clinical CT simulations using different tube voltages, different anatomical regions, and different scan trajectories.
**Results or Findings:** Compared to a conventional TCM that minimized the mAs product, rmTCM is able to reduce the effective dose by about 12% on average for 70 kV scans, 8% for 120 kV scans, and 7% for 150 kV scans. In any case, the highest effective dose reductions of up to 20% were achieved in the thorax region.
**Conclusion:** Recent developments in the field of deep learning, such as the deep dose estimation, open new options for automatic TCM. Using this additional prior knowledge allows to modulate the tube current to minimize the effective dose, and thus, to further reduce the radiation risk of CT examinations.
**Limitations:** Na
**Ethics Committee Approval:** Na
**Funding for this study:** Na

**Author Disclosures:**
Chang Liu: Nothing to disclose
Laura Klein: Nothing to disclose
Andreas Maier: Nothing to disclose

Michael Lell: Nothing to disclose
Marc Kachelrieß: Nothing to disclose
Stefan Sawall: Nothing to disclose
Joscha Maier: Nothing to disclose

### RPS 213-4

**The VCT_BREAST virtual clinical trial for 2D and 3D x-ray breast imaging and dosimetry**

*G. Mettivier*[1], A. Sarno[1], K. Bliznakova[2], A. Hernandez[3], X. Jia[4], Y. Lai[4], B. Golosio[5], M. C. Staffa[1], J. M. Boone[3]; [1]Naples/IT, [2]Varna/BG, [3]Sacramento, CA/US, [4]Dallas, TX/US, [5]Cagliari/IT
*(mettivier@na.infn.it)*

**Purpose:** We present the VCT_BREAST project, for the realization of virtual clinical trials (VCTs) for imaging and dosimetry research in breast computed tomography (BCT), digital breast tomosynthesis (DBT) and digital mammography (DM).
**Methods or Background:** VCT_BREAST is an ongoing Monte Carlo (MC) based VCT project for performing in-silico x-ray examinations of the breast. Virtual imaging of the compressed or uncompressed breast is simulated using imaging techniques that closely replicate clinical exams in terms of breast anatomy, system geometry, x-ray spectrum, imaging detection process and absorbed dose. The MC simulations replicate the 2D or 3D exam and run on both a CPU platform and a GPU OpenCL based cross-platform adopting GPU accelerating engines for simulation of the imaging datasets and 3D maps of the glandular dose. Patient-derived, anatomically realistic, computational phantoms of the uncompressed breast are generated using a tissue classification algorithm on BCT images. Correspondingly, we derived compressed breast models, via a computational tissue compression process. The dataset comprised 150 voxelized 3D breast phantoms and 60 compressed phantoms. Additional patient-like phantoms will be generated following a data augmentation scheme, by means of a machine learning computational network.
**Results or Findings:** Glandular dose distributions and virtual exams for BCT, DBT and DM were simulated using exemplary patient-derived anatomically realistic breast phantoms with different characteristics (shape, size and glandularity) derived from clinical BCT scans.
**Conclusion:** VCT_BREAST aims at VCTs for technology assessment and innovation in particular in DBT and BCT. This work presents the first VCT project for combined 2D and 3D x-ray breast imaging on a cohort of virtual patients, showing the present status of the project focused on the development of the software and hardware platform as well as assessing its overall performance.
**Limitations:** No
**Ethics Committee Approval:** Not applicable
**Funding for this study:** Not applicable
**Author Disclosures:**
Xu Jia: Nothing to disclose
Maria Carla Staffa: Nothing to disclose
John M. Boone: Nothing to disclose
Bruno Golosio: Nothing to disclose
Youfang Lai: Nothing to disclose
Kristina Bliznakova: Nothing to disclose
Giovanni Mettivier: Nothing to disclose
Antonio Sarno: Nothing to disclose
Andrew Hernandez: Nothing to disclose

### RPS 213-5

**Primary results of large-scale size-specific dosimetry in chest radiography**

*A. S. L. Dedulle*, N. Fitousi, H. Bosmans; Leuven/BE
*(an.dedulle@qaelum.com)*

**Purpose:** Although the need for personalized dosimetry in Computed Tomography has been highlighted in literature, it has not been extensively investigated in projection radiography. In earlier work we have shown how to estimate a water equivalent diameter (WED) from DICOM-tags in projection images. This study presents the first results using this WED to select size-specific dose conversion factors.
**Methods or Background:** Chest posterior-anterior radiographies performed between 2015 and 2019 (2671 examinations) were extracted with a dose management software (DOSE, Qaelum) and the WED was estimated. Patient-specific effective dose and organ doses (lungs, breasts, thyroid) were calculated by applying size-specific conversion factors. The conversion factors were generated from 44 voxel models of various sizes using a dedicated Monte Carlo framework.
**Results or Findings:** The estimated WED ranged from 19cm to 38cm (median:26cm). Mean patient-specific effective dose was 12±3µSv (range:6µSv-36µSv), with increasing dose for increasing WED ($p<0.05$). If, as routinely done, only 1 standard-size conversion factor would be used, the increase would be steeper and only attributed to the increase of dose area product (DAP) for larger patients. Lung dose was the highest among the organs investigated (range:13µSv-81µSv), followed by the thyroid dose (range:3µSv-31µSv) and both correlated with WED (R2=0.59-0.80). The breast dose (range:3µSv-12µSv) had weak correlation with WED: R2 of 0.01 (males) and 0.23 (females), which can be linked to the position of breast closer to the detector and the corresponding shielding effect.
**Conclusion:** A novel methodology made large-scale size-adjusted dosimetry for chest radiography feasible, by using only information available through dose management systems. Evaluating the patient-specific effective dose for different sizes shows the limitation of older methods that were only based on the change in exposure parameters.
**Limitations:** Not applicable.
**Ethics Committee Approval:** Not applicable.
**Funding for this study:** PhD grant VLAIO [HBC.2016.0233].
**Author Disclosures:**
An Saskia Luc Dedulle: Employee at Qaelum NV, Research/Grant Support at VLAIO grant (HBC.2016.0233)
Hilde Bosmans: Founder at Qaelum NV, Board Member at Qaelum NV
Niki Fitousi: Employee at Qaelum NV

### RPS 213-6

**Impact of dose reduction and iterative reconstruction on spectral performance of a dual-source CT system: task-based image quality and iodine quantification assessment**

*D. Dabli*, J. Frandon, P. Akessoul, A. Belaouni, A. Hamard, J-P. Beregi, J. Greffier; Nîmes/FR
*(Djamel.Dabli@chu-nimes.fr)*

**Purpose:** To assess the impact of dose reduction and the use iterative reconstruction (ADMIRE) on image quality in low-energy virtual monochromatic images (VMIs) and on iodine quantification from a Dual-source Dual Energy CT (DSCT).
**Methods or Background:** Two CT phantoms were scanned with a third generation DSCT using classical parameters of abdomen-pelvic examination for three dose levels (CTDIvol: 11/8/5 mGy). Raw-data were reconstructed using Filtered Back Projection (FBP) and two levels of ADMIRE (A3/A5). The noise-magnitude and noise-texture was assessed using the Noise power spectrum (NPS) and the spatial resolution using the task-based transfer function (TTF), for six low energy levels on VMIs. The iodine concentration accuracy was assessed with four different concentrations (2, 5, 10, 15 mg/ml) using root-mean-square deviation (RMSDiodine).
**Results or Findings:** Noise-magnitude was reduced between 40 to 70 keV by -80%±1%, -83%±1% and -86%±1% with FBP; A3 and A5, respectively. The mean spatial frequency of the NPS peaked at 70 keV and decreased as ADMIRE level increased. TTF values at 50% were highest at 40 keV and shifted towards lower frequencies as the keV increased. The difference between the measured and theoretical concentrations increased for 5 mGy level at 2 and 5 mg/ml. The RMSDiodine were similar for 11 and 8 mGy (0.35 mg/ml) and increased at 5 mGy (0.42 mg/ml).
**Conclusion:** At low keV on VMIs, the study confirmed that iterative reconstruction reduced the noise magnitude, improved the spatial resolution. No effect of ADMIRE on iodine quantification accuracy but this accuracy decreased at low dose for low iodine concentration.
**Limitations:** One of the five kVp pairs available on this DSCT for a single standard reconstruction kernel was used and no assessment of the phantom sizes impact.
**Ethics Committee Approval:** Not concerned.
**Funding for this study:** No funding.
**Author Disclosures:**
Jean-Paul Beregi: Nothing to disclose
Aymeric Hamard: Nothing to disclose
Julien Frandon: Nothing to disclose
Joel Greffier: Nothing to disclose
Philippe Akessoul: Nothing to disclose
Asmaa Belaouni: Nothing to disclose
Djamel Dabli: Nothing to disclose

### RPS 213-7

**Lens dose measurement during CT exams with new plastic scintillation dosimetry system**

*C. Popotte*[1], M. Munier[2], D. Paul[3]; [1]Strasbourg/FR, [2]Entzheim/FR, [3]Lyon/FR

**Purpose:** Assess the dose delivered to the lens during CT exams in real time.
**Methods or Background:** Plastic scintillation dosimeters (PSD) from Fibermetrix company where used on a Somatom Edge (Siemens) CT scan in order to monitor the following dose indexes CTDIvol and DLP, and absorbed dose. As the lens diameter vary between 6,5mm and 9mm, especially according to the patient’s age, the influence of acquisition parameters on the dose received in the lens area was evaluated with plastic scintillating point dosimeters ranging from 5mm to 15mm of length. IVIscan® PSD and CT

displayed dose indexes were compared for routine skull CT exams and for about 40 anthropomorphic phantom acquisitions.
**Results or Findings:** Discrepancies up to +50% between the measured dose by the IVIscan® PSD and the CTDIvol calculated by the CT scan were observed for patients receiving CT scans in the lens area. An investigation using IVIscan® measurements tools allowed to identify the origin of these discrepancies as mA modulation with patient positioning issues. When changing the pitch for the anthropomorphic phantoms from 0,6 to 1,5 the dose relative standard variation was, for a 15mm long point PSD, 1,9% and 6,9% respectively. Same measurements realized with a 5mm long point PSD showed a relative standard variation of 2,7% and 8,9% respectively. Using a 5mm long point PSD, variations up to 30% were observed in the lens region with a pitch of 1,5.
**Conclusion:** The study showed that sometimes dose variations in the lens area cannot be assessed by the usual dose indexes.
**Limitations:** Based on these results, further investigations has to be carried using point PSD to get a closer understanding of dose variations in the lens area depending on both anatomical and physical parameters.
**Ethics Committee Approval:** Approved.
**Funding for this study:** None.
**Author Disclosures:**
Christian Popotte: Nothing to disclose
Mme. Mélodie Munier: Founder at Fibermetrix
Didier Paul: Nothing to disclose

**Live Q&A**

**15:30-16:30 CHANNEL 2**

### ESR EIBALL Session

### ESR EIBALL

### Navigating an imaging biomarker from concept into the clinic

**Moderator**
Nandita M. deSouza; Sutton/UK

### ESR EIBALL-2

**Launching a biomarker (Biomarker x) successfully into clinical practice**
Marion Smits; Rotterdam/NL
*(marion.smits@erasmusmc.nl)*

**Learning Objectives:**
1. To learn what makes a successful biomarker.
2. To appreciate the necessary steps and processes required.
3. To understand the pathway used in the case of a successfully launched biomarker.

**Author Disclosures:**
Marion Smits: Consultant: Parexel Ltd, honorarium paid to institution; Speaker: GE Healthcare, honorarium paid to institution

### ESR EIBALL-3

**Global networking to promote biomarker development and use**
Xavier Golay; London/UK

**Learning Objectives:**
1. To learn about the relevant groups involved in biomarker development and validation.
2. To appreciate the on-going networking activities.
3. To understand the gaps in knowledge and how networking could address them.

**Author Disclosures:**
Xavier Golay: CEO: Gold Standard Phantoms

### ESR EIBALL-4

**Exploiting artificial intelligence (AI) for rapid biomarker development, validation, and interpretation**
Angel Alberich-Bayarri; Valencia/ES

**Learning Objectives:**
1. To learn how AI has the potential for identifying and validating biomarkers.
2. To appreciate the role of AI in data driven interpretation.
3. To understand the limitations in AI-driven biomarker interpretation.

**Author Disclosures:**
Angel Alberich-Bayarri: CEO: QUIBIM S.L.

**Live Q&A: When should imaging biomarkers be used as decision-support tools?**

**15:30-16:30 CHANNEL 3**

### E³ - ECR Master Class

### E³ 826

### Implants in the head and neck

**Moderator**
Alexandra Borges; Lisbon/PT

### E³ 826-2

**A. Otological implants**
Berit Verbist; Leiden/NL
*(b.m.verbist@lumc.nl)*

**Learning Objectives:**
1. To become familiar with the types and locations of otological implants.
2. To learn about optimal imaging strategies for such implants.
3. To become acquainted with the key imaging features to report.

**Author Disclosures:**
Berit Verbist: Research Grant/Support: insitutional: Cochlear, Advanced Bionics

### E³ 826-3

**B. Cosmetic facial implants**
Polly Richards; London/UK

**Learning Objectives:**
1. To become familiar with the types of compounds used in cosmetic facial implants.
2. To learn about the types of procedures patients may have undergone.
3. To recognise the relevant imaging features and post-procedural complications.

### E³ 826-4

**C. 3D printed implants in the skull: what's new and what's required?**
Maurice Mommaerts; Brussels/BE

**Learning Objectives:**
1. To learn about differing types of dental and maxillofacial implants.
2. To be familiar with the imaging appearance of such implants.
3. To understand what the surgeon wants to know in your report.

### E³ 826-5

**Case presentation**
Alexandra Borges; Lisbon/PT
*(borgalexandra@gmail.com)*

**Live Q&A**

**15:30-16:30 CHANNEL 4**

### Professional Challenges Session

### PC 5

### The state-of-the-art of everyday radiation: benefit-risk communication

**Moderators**
Paddy Gilligan; Dublin/IE
Catherine Owens; London/UK

### PC 5-3

**The patient has a right to know: what the EU Directive 2013/59 tells us**
Carmel J. Caruana; Msida/MT
*(carmel.j.caruana@um.edu.mt)*

**Learning Objectives:**
1. To learn about the ethical and legal rights and obligations for patients and staff regarding risk.
2. To understand who has to communicate the benefit-risk information.
3. To become familiar with how the new standards can be implemented in a practical manner.

### PC 5-4
**The patient's perspective is all that matters: what do they need to hear?**
Erik Briers; Hasselt/BE
*(erikbriers@telenet.be)*

**Learning Objectives:**
1. To learn why a patient-centred approach is important.
2. To understand the guidelines and the recommendations of the ESR patient advisory group.
3. To become familiar with how the new standards can be implemented in a practical manner for different patient groups.

### PC 5-5
**Communicating risks with parents and caretakers of paediatric patients**
Elly Castellano; London/UK
*(elly.castellano@rmh.nhs.uk)*

**Learning Objectives:**
1. To learn the importance of the communication of benefit-risk information to this sensitive group.
2. To understand the challenges and how they might be overcome.
3. To become familiar with how the above can be achieved in a practical manner.

### PC 5-6
**Radiation risk in pregnancy: what should the woman be informed of?**
Franz Kainberger; Vienna/AT

**Learning Objectives:**
1. To learn the importance of the communication of benefit-risk information to this radiosensitive cohort.
2. To understand the challenges and how they might be overcome.
3. To become familiar with how the above can be achieved in a practical manner.

**Live Q&A: Do we need to improve our risk communication skills? How?**

**15:30-16:30 CHANNEL 5**

### Research Presentation Session: Chest

### RPS 204

### Body composition, COVID-19, and interstitial diseases in chest imaging

**Moderator**
Jürgen Biederer; Heidelberg/DE

### RPS 204-2
**Can sarcopenia on preoperative chest computed tomography predict cancer-specific and all-cause mortality following pneumonectomy for lung cancer? A bi-institutional study**
F. Troschel[1], *F. J. Fintelmann*[2], T. Muley[3], F. Eichhorn[3], T. D. Best[2], A. Troschel[2], H. Winter[3], H. A. Gaissert[2], C. P. Heussel[3]; [1]Münster/DE, [2]Boston, MA/US, [3]Heidelberg/DE

**Purpose:** Sarcopenia on preoperative computed tomography (CT) has been associated with overall survival (OS) following pneumonectomy for non-small cell lung cancer (NSCLC). We hypothesized that thoracic sarcopenia independently predicts worse cancer-specific survival (CSS) and OS.
**Methods or Background:** This retrospective cohort study included consecutive pneumonectomies for primary NSCLC performed at Massachusetts General Hospital and Heidelberg University Hospital from 2010 to 2018. Muscle cross-sectional area was quantified on standard preoperative chest CT at three thoracic vertebral levels (T8, T10 and T12). Sarcopenia was defined as two standard deviations below the muscle cross-sectional area at T10 of healthy controls. Time-to-event analyses adjusted for age, body mass index, Charlson Comorbidity Index, forced expiratory volume in 1 second in % predicted, induction therapy, sex, smoking status, tumor stage, and laterality were performed. The likelihood-ratio test was used to assess whether the addition of sarcopenia improved model fit.
**Results or Findings:** 367 included patients were predominantly male with a median age of 62 years. Most patients underwent standard pneumonectomy for stage IIIA (46%) and squamous cell histology (58%). Sarcopenia was present in 104 patients (28%). After a median follow-up of 20.5 months (interquartile range, 9.2-46.9), 183 of 367 patients (50%) had died and 133 deaths (72.7%) were due to cancer. Sarcopenia was associated with shorter CSS (hazard ratio [HR] 1.7, p=0.008) and OS (HR 1.7, p=0.003). Sarcopenia improved model fit (p=0.009).
**Conclusion:** This bi-institutional study demonstrates that sarcopenia on preoperative chest computed tomography is an independent risk factor for CSS and OS following pneumonectomy for NSCLC.
**Limitations:** First, retrospective design. Second, imbalance between the two centers with one contributing significantly more patients. Third, inability to include race/ethnicity because this variable is not commonly collected in Europe.
**Ethics Committee Approval:** Yes
**Funding for this study:** None
**Author Disclosures:**
Thomas Muley: Nothing to disclose
Florian Eichhorn: Nothing to disclose
Claus Peter Heussel: Nothing to disclose
Fabian Troschel: Nothing to disclose
Till D. Best: Nothing to disclose
Florian J. Fintelmann: Grant Recipient at American Roentgen Ray Society
Hauke Winter: Nothing to disclose
Henning A. Gaissert: Nothing to disclose
Amelie Troschel: Nothing to disclose

### RPS 204-3
**The impact of chest CT body composition parameters on clinical outcomes in COVID-19 patients**
*G. Besutti*[1], M. Pellegrini[1], M. Ottone[1], E. Bonelli[1], G. Ligabue[2], M. Cantini[2], G. Guaraldi[2], P. Giorgi Rossi[1], P. Pattacini[1]; [1]Reggio Emilia/IT, [2]Modena/IT

**Purpose:** To evaluate the impact of chest CT body composition parameters at emergency room presentation on COVID-19 outcomes and disease severity.
**Methods or Background:** All consecutive COVID-19 patients who underwent chest CT at the emergency room from 27/02/2020 to 13/03/2020 were included. Pectoralis muscle density (MD), and total, visceral, and intermuscular adipose tissue areas (TAT, VAT, and IMAT) at T7-T8 vertebrae were retrospectively measured on chest CT and included in models adjusted for sex, age, and calendar period. Outcomes were: C-reactive protein (CRP), oxygen saturation (SO2), and CT disease extension at presentation, hospitalization, mechanical ventilation (MV) and/or death, death within 45 days.
**Results or Findings:** We included 318 patients (median age 65.7 years, females 37.7 %); 205 (64.47%) hospitalizations and 58 (18.24%) deaths occurred, and 97 (30.5%) patients died or needed MV. Decreased MD was a risk factor for hospitalization and MV/death, with a borderline effect on death (OR for the first vs last quartile=1.409;95%CI=0.399-4.973). Increased TAT, VAT, and IMAT were risk factors for hospitalization and MV/death, with a borderline effect of VAT and IMAT on death (OR for the last vs first quartile=3.096;95%CI=0.809-11.852, OR for the last vs first quartile=1.615;95%CI=0.431-6.053, respectively). All these parameters were similarly associated with worst SO2 and CT disease extension, while the association with CRP was stronger for VAT. Mediation analysis showed that approximately 3% of the effect of age on death was mediated by decreased MD.
**Conclusion:** Sarcopenia and ectopic fat accumulation are associated with COVID-19 severity and outcomes. VAT is associated with inflammatory reaction in COVID-19. Sarcopenia appears to be one of the mechanisms for the extremely strong effect of age on COVID-19 mortality.
**Limitations:** Observational single-center study; height was not available hence skeletal muscle index was not calculated.
**Ethics Committee Approval:** AVEN (number:2020/0045199).
**Funding for this study:** None.
**Author Disclosures:**
Pierpaolo Pattacini: Nothing to disclose
Massimo Pellegrini: Nothing to disclose
Guido Ligabue: Nothing to disclose
Efrem Bonelli: Nothing to disclose
Michele Cantini: Nothing to disclose
Paolo Giorgi Rossi: Nothing to disclose
Giulia Besutti: Nothing to disclose
Marta Ottone: Nothing to disclose
Giovanni Guaraldi: Nothing to disclose

### RPS 204-4

**Post-COVID-19 interstitial lung syndrome (P-COILS): HRCT findings after six months**

*D. Cozzi*, E. Cavigli, C. Moroni, A. Bindi, S. Luvarà, V. Luzzi, S. Tomassetti, V. Miele; Florence/IT
*(dilettacozzi@gmail.com)*

**Purpose:** Chronic-pulmonary HRCT changes after COVID-19 remains unexplored.The aim of the study is to describe HRTC interstitial changes occurring 6-months after the admission for COVID-19.
**Methods or Background:** This is a single-center interim-analysis of a larger prospective-multicenter-national trial involving 12 Italian referral centers for ILDs. We describe the first cases enrolled in September-October 2020 at Careggi University Hospital, Florence(IT).A team of dedicated-thoracic radiologists evaluated at 6-months all patients that recovered from COVID-19.The presence of significant lung involvement was defined accordingly to current “interstitial-lung-abnormalities” definition as non-dependent abnormalities affecting more than 5%of any lung zone.
**Results or Findings:** Finally 89 HRCT were evaluated: 49/89(55%) had a complete remission from lung involvement. Of the 40 subjects(45%) with persistent changes at HRCT, we found 22(24.7%)with subpleural-lines and 21(23.6%)with interstitial thickening with perilobular pattern.Ground-glass-opacities were still appreciable in 14 (15.7%)and traction bronchiectasis in 13(14.6%).Only 3(3.4%)had a OP-like pattern and 1.1% a UIP-like fibrosis.One patient(1.1%) had cystic changes in lower lobes.
**Conclusion:** These preliminary results show that almost a half(45%) of patients that recovered from COVID-19 have persistent HRCT changes 6-months after the acute infection.A small minority(1%) present with the typical UIP pattern observed in IPF.The vast majority presents with subpleural/perilobular lines, mild ground-glass and signs of fibrotic changes (traction bronchiectasis), possible expression of the slowly-healing OP/DAD pattern observed in the acute phase.
**Limitations:** These are preliminary-descriptive results of a limited-number of cases from a national trial still ongoing.The trial was designed to define the risk factors for development of long-term-pulmonary sequelae of COVID-19, detect possible novel biomarkers for disease progression(including BAL and histopathology assessment).The clinical meaning of the observed HRCT changes remains unclear: we can’t exclude that the minority of UIP pattern detected might be expression of a pre-existing non-recognized UIP/IPF.
**Ethics Committee Approval:** Yes(Ref.PCOILS)
**Funding for this study:** None
**Author Disclosures:**
Alessandra Bindi: Nothing to disclose
Vittorio Miele: Nothing to disclose
Silvia Luvarà: Nothing to disclose
Sara Tomassetti: Nothing to disclose
Edoardo Cavigli: Nothing to disclose
Valentina Luzzi: Nothing to disclose
Chiara Moroni: Nothing to disclose
Diletta Cozzi: Nothing to disclose

### RPS 204-5

**Pulmonary cysts as a diagnostic indicator of Birt-Hogg-Dubé syndrome in patients with renal cell carcinoma**

*A. H. Ashok*, T. J. Sadler, S. Scullion, S. Karia, J. Babar, S. J. Marciniak, G. Stewart, E. Maher, M. T. A. Wetscherek; Cambridge/UK

**Purpose:** Birt–Hogg–Dubé syndrome (BHD) is an autosomal dominant disorder, characterised by skin fibrofolliculomas, lung cysts, spontaneous pneumothorax and increased risk of renal cell carcinoma(RCC). The incidence of BHD in RCC has not been systematically evaluated in previous studies. The purpose of our study was to assess the presence of pulmonary cysts in patients who underwent nephrectomy for RCC as a potential indicator of BHD syndrome.
**Methods or Background:** We retrospectively assessed the presence of lung cysts on chest CT of all patients with records of nephrectomy for RCC in the past 6 years. We investigated if the patients fulfilled the diagnostic criteria in the European BHD Consortium statement (1 major criteria: genetically confirmed Folliculin germline mutation, or two minor criteria: multiple/lower zone predominant lung cysts, and either age <50 years-old at diagnosis of RCC, multiple/bilateral RCC, or particular RCC histology).
**Results or Findings:** Among the 1288 patients with RCC, 120 (9.3%, age at time of diagnosis 61±11 years, M:F 88:32) had at least one pulmonary cyst. Twenty-seven patients (2.1%) fulfilled the BHD consortium criteria: three cases had genetically confirmed BHD, while 24 patients (1.9%) met at least two minor criteria: <50 years old at the time of diagnosis (n=20), multifocal or bilateral RCC (n=4), mixed type tumour at histology (n=2). Further genetic evaluation in patients with suspicious imaging appearance of BHD is in progress.
**Conclusion:** This is the first study to investigate the incidence of BHD in a large cohort of RCC. Although BHD remains a rare condition, patients with a combination of RCC and pulmonary cysts may need further clinical and genetic workup and radiologists should be aware of this diagnosis.
**Limitations:** Genetic data not available in all patients.
**Ethics Committee Approval:** This work was approved as service improvement project.
**Funding for this study:** None
**Author Disclosures:**
Timothy Sadler: Nothing to disclose
Eamonn Maher: Nothing to disclose
Stephen Scullion: Nothing to disclose
Maria Teodora Antuaneta Wetscherek: Nothing to disclose
Sumit Karia: Nothing to disclose
Judith Babar: Nothing to disclose
Abhishekh Hulegar Ashok: Nothing to disclose
Stefan J. Marciniak: Nothing to disclose
Grant Stewart: Nothing to disclose

### RPS 204-6

**The effects of anticoagulant treatment on thromboembolic lesions at CTPA in patients with acute pulmonary embolism with an ultimate diagnosis of CTEPH**

*N. J. Braams*[1], G. Boon[2], F. de Man[1], P. den Exter[2], L. Kroft[2], L. F. Beenen[1], F. Klok[2], H. J. Bogaard[1], L. J. Meijboom[1]; [1]Amsterdam/NL, [2]Leiden/NL
*(n.braams@amsterdamumc.nl)*

**Purpose:** Chronic thromboembolic pulmonary hypertension (CTEPH) is described as the most severe consequence of acute pulmonary embolism (APE). However, signs of chronic PE may already be present at the APE CT pulmonary angiogram (CTPA), which may predispose for CTEPH. The effect of anticoagulant treatment on various thromboembolic lesions is yet unknown. We aimed to investigate the effect of anticoagulant treatment on various thromboembolic lesions at CTPA in patients with APE with an ultimate diagnosis of CTEPH.
**Methods or Background:** We analyzed baseline CTPA (i.e. at APE diagnosis) and follow-up CTPA in 41 patients with CTEPH (cases) and 124 patients without CTEPH at follow-up (controls). All patients received anticoagulant treatment. Segmental pulmonary arteries were scored as normal or affected (as defined by a total occlusion by thrombus, central thrombus, mural thrombus, web or tapered pulmonary artery) by experienced chest radiologists.
**Results or Findings:** Central thrombi resolved after anticoagulant treatment for APE, while mural thrombi or total occlusions by thrombi either resolved or transformed to a web or tapered pulmonary vessel at follow-up. Webs and tapered pulmonary arteries did not resolve at follow-up and were only present in the CTEPH group at baseline. In addition, the number of webs and tapered pulmonary arteries increased at follow-up in CTEPH patients (resp. from 18 to 33% and 9 to 19%; both p<0.001), probably as remnants of central thrombi, mural thrombi or total occlusion by thrombi at baseline.
**Conclusion:** None of the webs and tapered segmental pulmonary arteries resolved after anticoagulant treatment for APE. Therefore, webs and tapered segmental pulmonary arteries indicate chronic morphology, and should raise awareness for CTEPH in patients with persistent dyspnea after anticoagulant treatment for acute PE.
**Limitations:** Not applicable
**Ethics Committee Approval:** Not applicable
**Funding for this study:** Research grant Actelion Pharmaceuticals
**Author Disclosures:**
Natalia Joanna Braams: Nothing to disclose
Paul den Exter: Nothing to disclose
Erik Klok: Nothing to disclose
L.J.M. Kroft: Nothing to disclose
Harm Jan Bogaard: Nothing to disclose
Frances de Man: Nothing to disclose
L. J. Meijboom: Nothing to disclose
L.F.M. F.M. Beenen: Nothing to disclose
Gudula Boon: Nothing to disclose

### RPS 204-7

**Systematic review and meta-analysis of CT-derived bronchial parameters**

*I. Dudurych*[1], S. Muiser[1], N. McVeigh[2], H. Kerstjens[1], M. van den Berge[1], M. de Bruijne[3], R. Vliegenthart[1]; [1]Groningen/NL, [2]Dublin/IE, [3]Rotterdam/NL

**Purpose:** CT-derived bronchial parameters are obtained via diverse methodologies with conflicting results among various populations. To determine the current methods for bronchial parameters we conducted a systematic review, and pooled bronchial parameters for never-smoking, smoking, asthma and COPD populations to determine reference values.
**Methods or Background:** MEDLINE and Embase databases were systematically searched (last date 13th October 2020). Methodologic data from studies were summarised. Bronchial parameters of Percentage Wall Area (WA%), Wall Thickness (WT), square root of the wall area at hypothetical airway at internal perimeter of 10mm (Pi10) and Luminal Area (Ai) were pooled according to population and compared.

**Results or Findings:** The methodologic review included 162 articles, of which 61 were included for meta-analysis. Most studies (85/162 articles) used multiplanar reconstruction for bronchial parameter measurement. Full-Width Half-Max algorithm was used the most (46/162 articles). Upper and lower lobe 3rd generation airways were most studied. The largest populations were current-smokers (14,926) and patients with COPD (12,618), followed by asthma (2,542) and never-smoking (1,045) populations. Mean pooled parameters were as follows: Pi10 (mean SD 3.97±0.39mm), WT (2.39±0.83mm) and Ai normalised to Body Surface Area (12.46±4mm²) and were generally larger in never-smokers, while COPD had the largest WA% (63.34±7.42%, n=5,563). WA% discriminated between all populations (all comparisons p <0.001) with the exception for comparison between current-smoking and asthma populations(p=0.11).
**Conclusion:** Bronchial parameters are derived with diverse methodology, with a focus on larger airways in the COPD and smoker populations. The most discriminative bronchial parameter was percentage wall area for all but smoking vs asthma populations.
**Limitations:** Pooled analysis assumes a normal distribution. There was insufficient information for subgroup (e.g. sex, age) analysis.
**Ethics Committee Approval:** Not applicable.
**Funding for this study:** Project funded by by IMDI (Innovative Medical Devices Initiative)/ZonMW (Netherlands Organisation for Health Research and Development).
**Author Disclosures:**
Ivan Dudurych: Nothing to disclose
Marleen de Bruijne: Nothing to disclose
Susan Muiser: Nothing to disclose
Maarten van den Berge: Nothing to disclose
Rozemarijn Vliegenthart: Nothing to disclose
Niall McVeigh: Nothing to disclose
Huib Kerstjens: Nothing to disclose

**Live Q&A**

**16:45-17:45 CHANNEL 2**

### Special Focus Session

### SF 13

### Can we limit gadolinium use in neuroimaging?

**Moderator**
Alejandro Rovira-Cañellas; Barcelona/ES

### SF 13-2
**Gadolinium: an environmental issue**
Gerard Stroomberg; Nieuwegein/NL
*(stroomberg@riwa.org)*

**Learning Objectives:**
1. To learn about the potential toxic effect of environmental free gadolinium.
2. To appreciate that gadolinium is an emerging contaminant in water resources.
3. To understand the need of monitoring gadolinium distribution in tap waters.

### SF 13-3
**Gadolinium deposition: an update**
Dragan A. Stojanov; Nis/RS
*(drstojanov@gmail.com)*

**Learning Objectives:**
1. To learn about the mechanisms involved in gadolinium deposition in the brain.
2. To understand stability differences of the types of gadolinium chelates and their relationship to gadolinium deposition in the brain.
3. To learn about the potential long-term adverse health effects of gadolinum deposition in the brain.

### SF 13-4
**I don't need gadolinium**
Tarek A. Yousry; London/UK

**Learning Objectives:**
1. To understand the rationale for reducing repetitive injections of gadolinium-based contrast agents.
2. To identify clinical situations in which gadolinium-based contrast agents have no added value for diagnostic and monitoring purposes.
3. To learn about the potential of unenhanced MRI techniques with quantitative image analysis to obtain gadolinium-analog information.

### SF 13-5
**I always need gadolinium**
Nicoletta Anzalone; Milan/IT
*(anzalone.nicoletta@hsr.it)*

**Learning Objectives:**
1. To appreciate the excellent safety profile and tolerance of gadolinium-based contrast agents.
2. To learn that there is no documented clinical risk related with gadolinium deposition in the brain.
3. To learn about the added clinical value of gadolinium MRI for diagnosis and monitoring purposes in different neurologic disorders.
**Author Disclosures:**
Nicoletta Anzalone: Consultant: bracco, Bayer; Speaker: Bracco, Bayer

**Live Q&A: Towards practice harmonisation**

**16:45-17:45 CHANNEL 3**

### Coffee & Talk (open forum) Session
*Organised by EuroSafe Imaging*

### C 15

### Image quality and dose: a practical guide for radiologists

**Moderators**
Werner R. Jaschke; Innsbruck/AT
Annalisa Trianni; Trento/IT

### C 15-3
**Image quality in CT: the medical physicist's perspective**
Annalisa Trianni; Trento/IT
*(annalisa.trianni@apss.tn.it)*

**Learning Objectives:**
1. To learn how to quantify image quality.
2. To appreciate the relation between dose and image quality.
3. To understand how image reconstruction influences image quality.

### C 15-4
**Image quality in CT: the radiologist's perspective**
Mathias Prokop; Nijmegen/NL
*(mathias.prokop@radboudumc.nl)*

**Learning Objectives:**
1. To learn how to assess image quality and how to avoid artefacts.
2. To appreciate the important role of image quality and diagnostic accuracy.
3. To understand that an appropriate image quality can be achieved at rather low dose levels for many clinical tasks.
**Author Disclosures:**
Mathias Prokop: Patent Holder: 4D noise suppression, licensed to Canon Medical Systems; Research Grant/Support: Canon Medical Systems, Siemens Healthineers; Speaker: Canon Medical Systems, Siemens Healthineers

### C 15-5
**Lung cancer screening using CT**
Matthys Oudkerk; Groningen/NL

**Learning Objectives:**
1. To learn how to optimise CT protocols for lung cancer screening.
2. To appreciate the important role of dose reduction.
3. To understand the risks associated with screening by CT.
**Author Disclosures:**
Matthys Oudkerk: Author: NEJM , Lancet etc Nelson study; Board Member: Institute of Diagnostic Accuracy; Employee: University of Groningen; Grant Recipient: KNAW, ERC,Horizon 2020, etc

**Live Q&A**

16:45-17:45 CHANNEL 5

### Research Presentation Session: Interventional Radiology

### RPS 209

### Liver oncology

**Moderator**
Dimitrios Filippiadis; Athens/GR

### RPS 209-2

**Balloon occluded TACE (B-TACE) vs DEM-TACE for HCC: a single center retrospective case control study**

P. Lucatelli, *B. Rocco*, G. De Rubeis, M. Corona, L. Teodoli, A. Cannavale, P. G. Nardis, M. Bezzi, C. Catalano; Rome/IT
*(biancarocco.br@gmail.com)*

**Purpose:** Compare oncological results and safety profile of balloon micro-catheter transarterial chemoembolization (b-TACE) and drug-eluting-microsphere (DEM-TACE) in patients with hepatocellular-carcinoma (HCC)
**Methods or Background:** This is a case-control, retrospective, single-center study. Between January-2015/March-2019, 149 patients with 226 HCC were treated, 22 patients (35 HCC) with b-TACE and 127 with DEM-TACE (191 HCC). Embolization protocol was standardized (sequential 100±25 and 200±25 µm microspheres). Results were evaluated by modified-responseevaluation-criteria-in-solid-tumor [mRECIST] at 1, 3-6 and 9-12 months and time to recurrence after complete response [TTR] at 1 years. Cox's regression weighted with tumor dimensions was performed. Adverse events (AEs) were recorded
**Results or Findings:** mRECIST oncological response at all time points (1, 3-6 and 9-12 months) for both treatments were similar, with the exception of Objective response rate at 9-12months. Objective response at 1 and 3-6 months between b-TACE vs DEM-TACE [23/35(65.7%) vs 119/191(62.3%), 21/29(72.4%) vs 78/136(57.4%) (p>0.05), respectively].
On the contrary, at 9-12 months, it was significantly higher in b-TACE subgroup than
DEM-TACE (15/19[78.9%] vs 48/89[53.9%], p=0.05). TTR for complete response at
1 year had a better trend for b-TACE vs DEM-TACE (278.0 days [196.0-342.0] vs 219.0 days [161.0-238.0], OR 0.68 [0.4-1.0],p=0.10). The use of balloon microcatheter reduced the relative risk of the event of recurrence by 0.63 [CI95% 0.38-1.04]; p=0.07). No significant differences were found in AEs rate
**Conclusion:** b-TACE showed a trend of better oncological response over DEM-TACE with and longer TTR with a similar adverse events rate, in patients presenting with larger tumors
**Limitations:** The nature of the study is retrospective and observational without randomization; groups were not homogenous, though this limitation was overcome by weighting differences as co-variate in statistical analysis.
**Ethics Committee Approval:** This study was approved by ethical review board of our institution
**Funding for this study:** No fundings were received
**Author Disclosures:**
Bianca Rocco: Nothing to disclose
Mario Bezzi: Nothing to disclose
Mario Corona: Nothing to disclose
Gianluca De Rubeis: Nothing to disclose
Pier Giorgio Nardis: Nothing to disclose
Alessandro Cannavale: Nothing to disclose
Leonardo Teodoli: Nothing to disclose
Pierleone Lucatelli: Nothing to disclose
Carlo Catalano: Nothing to disclose

### RPS 209-4

**Feasibility of intra-arterial Lu-177 DOTA TATE peptide receptor radionucleotide therapy in metastatic neuroendocrine tumours with liver dominant disease**

*M. Palaniswamy*, M. Uthappa; Chennai/IN
*(drmohnishp@gmail.com)*

**Purpose:** Purpose was to assess the safety, tolerability and efficacy of intra arterial Lu-177 DOTA TATE therapy in patients with liver dominant metastasis in well differentiated NETS.
**Methods or Background:** Four patients with well differentiated NET liver dominant metastasis4 cycles of 200mCi of Lu-177 DOTATATE therapy intra arterially at interval of 8 weeks Through selective hepatic arterial catheterization. All patients received bilobar hepatic arterial infusions in the same sitting. All patients were administered aminoacid infusions for 4-6 hours starting pretherapy and continued post therapy infusions for nephroprotection. Plan to cover with long acting octeotide therapy was instituted in case of endocrine crisis.Their complete blood counts, liver function, renal function and coagulation profiles were assessed within 2 weeks prior to each treatment cycle, in the immediate post therapy period and within 6 weeks post treatment. Radiological assessment and tumor markers were also assessed pre and post treatment.
**Results or Findings:** Significant reduction in serum chromogranin A levels Partial reduction in size and tracer uptake in all patients were noted.
None of the patients experienced any acute side effects or unexpected complication during therapy or in the immediate post therapy period .
No radiation induced lung injuryor renal toxicity seen during the course of therapy and even after the last cycle.
Liver functions remained stable with improved quality of life.
**Conclusion:** Three patients showed partial response. One patient showed stable disease (PERCIST)
Survival benefits will be evaluated after 1 year of follow up.
**Limitations:** Patients with Renal disease could not be included in the study
**Ethics Committee Approval:** Approved
**Funding for this study:** It was funded by the hospital.
**Author Disclosures:**
Mohnish Palaniswamy: Consultant at Gleneagles Global Health City
M.C. Uthappa: Consultant at Gleneagles Global Health City

### RPS 209-5

**Transarterial chemoembolisation in patients with hepatocellular carcinoma: impact of low-dose doxorubicin on post-embolisation syndrome and overall survival**

*A. A. A. Bessar*; Zagazig/EG
*(ahmedawadbessar@gmail.com)*

**Purpose:** Introduction: No chemotherapeutic agents have been standardized for transarterial chemoembolization (TACE). Moreover, doxorubicin, the most commonly chemotherapeutic agent has no standard optimal dosage in TACE procedures.
Objectives: To compare effects of low versus currently used doses of doxorubicin in TACE on post-embolization syndrome (PES) and overall survival (OAS) in advanced hepatocellular carcinoma (HCC) patients.
**Methods or Background:** Patients and methods: Prospective interventional study between 10/2014 and 3/2018 on patients with primary HCC, on top of cirrhosis due to hepatitis C infection, who underwent palliative TACE. Patients were divided into 2 groups: group A received 50 mg and group B received 100 mg of doxorubicin TACE. The outcomes were PES, individual manifestations of PES, time-to-be free from PES, and tumor response to TACE at 1-, 3-, and 6-month post-procedure, and OAS.
**Results or Findings:** Results: Twenty-eight patients received 68 TACE procedures with 15 (Group A) and 13 (Group B) patients palliated with 50mg and 100mg of doxorubicin, respectively. Mean age at first procedure was 58.9 (±6.8) years. Males constitute 85.7% of patients and 27 (96.4%) patients had repeated TACE. visual analgesia score and pain duration were significantly different between both groups (P<0.0001). Median duration of fever was shorter in Group A than Group B (p=0.003). No statistically differences between the 2 groups in tumor response to TACE and OAS. Doxorubicin dose was statistically correlated with duration of pain, fever, VAS score and vomiting.
**Conclusion:** Conclusion: Lower dose of doxorubicin was associated with fewer PES symptoms and duration compared with 100 mg without effects on tumor response nor OAS.
**Limitations:** Not specified
**Ethics Committee Approval:** Not specified
**Funding for this study:** Not specified
**Author Disclosures:**
Ahmed Awad Abdelaziz Bessar: Nothing to disclose

### RPS 209-6

**Circulating tumour cells in hepatocellular carcinoma under interventional radiological treatment: cTACE vs MWA**

*T. J. Vogl*, L. Riegelbauer, E. Oppermann, H. Ackermann, S. Stein, W. Bechstein, M. Juratli; Frankfurt/DE
*(t.vogl@em.uni-frankfurt.de)*

**Purpose:** To investigate the short-term dynamics of circulating tumor cells (CTCs) in patients with hepatocellular carcinoma (HCC) under conventional transarterial chemoembolization (cTACE) or microwave ablation (MWA). The short-term dynamics were compared with the clinical course of the patients using a novel method for the isolation and detection of CTCs.
**Methods or Background:** Blood samples from 18 patients with HCC who underwent cTACE (n=8) or MWA (n=10) were analyzed. Intravenous blood was taken before and immediately after the radiological interventions to isolate CTCs and quantify them by flow cytometry. CTCs were considered to be those cells that were negative for the marker CD45 and positive for the markers

ASGPR, CD146, and CD274 (PDL1). Follow-up studies of up to 2 years after the radiological intervention were evaluated.
**Results or Findings:** The number of CTCs after radiological intervention (post=0.9 CTCs/ml of blood) was on average lower than that before therapy (pre=1.2 CTCs/ml of blood). The number of CTCs decreased more in patients treated with MWA (0.4 CTCs/ml of blood) than in those who received cTACE (0.2 CTCs/ml of blood). The 2-year survival rate in the group receiving cTACE (37.5% died) was lower than that in MWA patients (0% died).
**Conclusion:** The therapeutic benefit of cTACE and MWA in patients with HCC is emphasized by decreasing CTCs and the clinical course of the patients.
**Limitations:** Small sample size
**Ethics Committee Approval:** The study was approved by the ethical committee
**Funding for this study:** No funding
**Author Disclosures:**
LInda Riegelbauer: Nothing to disclose
Mazen Juratli: Nothing to disclose
Thomas J. Vogl: Nothing to disclose
Wolf Bechstein: Nothing to disclose
Stefan Stein: Nothing to disclose
Elsie Oppermann: Nothing to disclose
Hanns Ackermann: Nothing to disclose

### RPS 209-8

**Efficacy and safety of TACE combined with sorafenib plus immune checkpoint inhibitors for the treatment of intermediate and advanced TACE-refractory hepatocellular carcinoma: a retrospective study**
*L. Zheng*, S. Fang, F. Wu, Z. Zhao, J. Ji; Lishui/CN
*(zhengliyun@zju.edu.cn)*

**Purpose:** The study aims to retrospectively investigate the efficacy and safety of sorafenib combined with TACE (TACE+Sor) vs. TACE+Sor plus immune checkpoint inhibitors (TACE+Sor+ICIs) in treating intermediate and advanced TACE-refractory hepatocellular carcinoma.
**Methods or Background:** From January 2016 to June 2020, 51 eligible patients with intermediate or advanced TACE-refractory HCC received TACE+Sor (n=29) or TACE+Sor+ICIs (n=22). The differences in tumor response, adverse events, progression-free survival, and overall survival were compared between the two groups. Factors affecting PFS and OS were determined by Cox regression.
**Results or Findings:** The DCR was higher in the TACE+Sor+ICIs group than in the TACE+Sor group (81.82% vs 55.17%, P=0.046). Compared with the TACE+Sor group, PFS and OS were prolonged in the TACE+Sor+ICIs group (mPFS: 16.26 vs 7.30 months, P<0.001; mOS: 23.3 vs 13.8 months, P=0.012). Multivariate analysis showed that BCLC stage, alpha-fetoprotein and treatment were independent factors of PFS; BCLC, Child-Pugh class, ablation after disease progression and treatment were independent predictive factors of OS. Four patients in the TACE+Sor+ICIs group and three patients in the TACE+Sor group suffered from dose reduction or interruption (18.18% vs 10.34%, P=0.421).
**Conclusion:** The therapeutic schedule of TACE+Sor+ICIs demonstrated efficacy and safety in advanced TACE-refractory HCC.
**Limitations:** As a retrospective study, it has all of the defects inherent to this type of study design. For example, the background of patients, including financial capability, education, and cognition of liver cancer, have an impact on the choice of therapy by patients and physicians. In addition, the limitation of the sample size of patients and the length of follow-up also had a significant impact on the outcome.
**Ethics Committee Approval:** approved by the ethics committee of Lisui Hospital, Zhejiang University, China.
**Funding for this study:** National Natural Science Foundation of China (81803778); Zhejiang Natural Science Foundation/exploration project Q (LQ20H160055)
**Author Disclosures:**
Fazong Wu: Author at conducted all experiments, analyzed the data and wrote the manuscript
Shiji Fang: Author at conducted all experiments, analyzed the data and wrote the manuscript
Jiansong Ji: Author at conducted all experiments, analyzed the data and wrote the manuscript
Liyun Zheng: Speaker at designed the study and revise this manuscrip
Zhongwei  Zhao: Author at conducted all experiments, analyzed the data and wrote the manuscript

**Live Q&A**

**18:00-19:00 CHANNEL 2**

### Coffee & Talk (open forum) Session

### C 6

### Professional regulation and opportunities across Europe

**Moderator**
Charlotte Beardmore; London/UK

### C 6-2

**Professional regulation: a tool or a barrier for harmonisation and free movement**
Louise A. Rainford; Dublin/IE
*(louise.rainford@ucd.ie)*

**Learning Objectives:**
1. To gain an overview of the role of professional regulators.
2. To appreciate how professional regulators function and the importance of their work.
3. To understand professional regulatory implications regarding free movement and qualification recognition.

### C 6-3

**Harmonisation of professional knowledge, skills, and competencies across Europe**
Andrew England; Salford/UK
*(a.england@keele.ac.uk)*

**Learning Objectives:**
1. To learn about the differences in professional knowledge, skills, and competencies for radiographers across Europe.
2. To appreciate the need for harmonising knowledge, skills, and competencies across Europe.
3. To understand the methods for harmonising knowledge, skills, and competencies.

### C 6-4

**The EU Basic Safety Standards (BSS) directive: progressing or restricting the harmonisation of the radiographer profession across Europe**
Joana Santos; Coimbra/PT
*(joanasantos@estescoimbra.pt)*

**Learning Objectives:**
1. To understand radiographers' responsibilities and duties according to the EU Basic Safety Standards (BSS) directive.
2. To gain an overview of the BSS radiographer's radiation protection role across countries.
3. To become familiar with different countries strategies in order to promote the harmonisation of the radiographer profession.

### C 6-5

**The Nordic continuing professional development (CPD) system: a tool to facilitate harmonisation and free movement**
Claus Brix; Frederiksberg/DK
*(Claus@radiograf.dk)*

**Learning Objectives:**
1. To learn about how CPD can be used to support ongoing and post qualification development of the profession.
2. To understand the necessary importance of CPD in the development of clinical skills.
3. To become familiar with the work and implementation of CPD in Nordic countries.

**Live Q&A**

18:00-19:00 CHANNEL 3

### Special Focus Session

### SF 15

### How to succeed in brachial plexus imaging

**Moderator**
Felix P. Kuhn; Zurich/CH

### SF 15-2

**High-resolution ultrasound: techniques and pitfalls**
Carlo Martinoli; Genoa/IT
*(55904@unige.it)*

**Learning Objectives:**
1. To learn about the technical possibilities in high-resolution ultrasound.
2. To understand the potential pitfalls.
3. To appreciate the possibilities to extend and improve your ultrasound skills.
4. To become familiar with different clinical cases.

### SF 15-3

**MRI protocol optimisation: different information from different MR sequences (incl. DTI)**
Carlos Torres; Ottawa, ON/CA
*(catorres@toh.on.ca)*

**Learning Objectives:**
1. To learn about the different types of MR pulse sequences to assess the brachial plexus.
2. To understand how to improve your MR protocols.
3. To appreciate what information can be extracted from different MR sequences (incl. DTI).
4. To become familiar with 3D high-resolution imaging.

### SF 15-4

**MRI in clinical routine: radiological/clinical correlation**
Simonetta Gerevini; Milan/IT
*(gerevini.simonetta@hsr.it)*

**Learning Objectives:**
1. To learn about the correlation between image findings and the clinical presentation.
2. To understand the importance of pertinent clinical information.
3. To appreciate the additional information which you may be able to provide to your clinical colleagues.
4. To become familiar with different clinical cases.

### SF 15-5

**Case review: a particularly interesting case (part 1)**
Felix P. Kuhn; Zurich/CH

### SF 15-6

**Case review: a particularly interesting case (part 2)**
Carlo Martinoli; Genoa/IT
*(55904@unige.it)*

### SF 15-7

**Case review: a particularly interesting case (part 3)**
Carlos Torres; Ottawa, ON/CA
*(catorres@toh.on.ca)*

**Live Q&A: How to find your way in this complex anatomy**

18:00-19:00 CHANNEL 4

### Research Presentation Session: Vascular

### RPS 215

### Vascular and interventional radiology

**Moderator**
Viktor Bérczi; Budapest/HU

### RPS 215-2

**The majority of major amputations after resuscitative endovascular balloon occlusion of the aorta are associated with pre-admission trauma**
*S. R. Levin*, A. Farber, P. Burke, T. Brahmbhatt, A. Richman, K. Twomey, J. Siracuse; Boston, MA/US
*(scott.levin@bmc.org)*

**Purpose:** Resuscitative endovascular balloon occlusion of the aorta (REBOA) is potentially life-saving. However, reports of associations with limb loss and mortality have called its safety into question. We aimed to evaluate patient and hospital characteristics associated with major amputation and mortality among patients undergoing REBOA.
**Methods or Background:** The U.S. National Trauma Data Bank (2015-2017) was queried for patients who presented to trauma centers and underwent REBOA. We determined associations with major amputation and in-hospital mortality.
**Results or Findings:** A total of 316 patients underwent REBOA. Mean age was 45±20 years. Most patients presented to Level I trauma centers (72%) after blunt injuries (79%) with an average injury severity score (ISS) of 31±15, indicating major trauma. In 15 patients (5%), there were 18 major amputations – 7 above-knee and 11 below-knee. Eleven amputations were either traumatic amputations (73%) or mangled limbs requiring amputation within 24 hours (27%). Among remaining amputations, 71% were associated with ipsilateral vascular/orthopedic extremity injuries. Comparing patients with and without amputations, demographics/comorbidities and hospital characteristics were similar.There were 110 deaths (35%). Amputation prevalence was similar between patients who died vs. survived (3.6% vs. 5.3%, P=.5). Prehospital cardiac arrest (OR 8.47,95% CI 1.47-48.66), penetrating vs. blunt trauma (OR 5.5,95% CI 1.05-28.82), decreased Glasgow Coma Scale score (OR 1.18,95% CI 1.05-1.32), increased age (OR 1.06,95% CI 1.03-1.1), and increased ISS (OR 1.05,95% CI 1-1.1) were associated with higher mortality.
**Conclusion:** Despite concerns about prohibitive limb complications of REBOA, baseline injuries appear to be the primary cause of limb loss. Injury type and severity, as well as initial hemodynamic derangements were associated with mortality after REBOA.
**Limitations:** Retrospective study.
**Ethics Committee Approval:** The Boston University Institutional Review Board and American College of Surgeons Committee on Trauma approved this study.
**Funding for this study:** Not applicable.
**Author Disclosures:**
Peter Burke: Nothing to disclose
Kathryn Twomey: Nothing to disclose
Jeffrey Siracuse: Nothing to disclose
Tejal Brahmbhatt: Nothing to disclose
Scott Ross Levin: Nothing to disclose
Aaron Richman: Nothing to disclose
Alik Farber: Nothing to disclose

### RPS 215-3

**Computed tomography in extracorporeal membrane oxygenation (ECMO): scan techniques, normal findings, and complications**
*H. Çelik*[1], J. Weir-McCall[2], A. Barker[2], L. L. D'Errico[2], T. H. Yu[2], S. Sultana[2], I. R. Okam[2], S. K. B. Agrawal[2]; [1]Izmir/TR, [2]Cambridge/UK
*(hakkicelikmd@gmail.com)*

**Purpose:** To review the principles of performing computed tomography (CT) in extracorporeal membrane oxygenation (ECMO), and review the normal findings of patients on ECMO, and complications related to this.
**Methods or Background:** ECMO is a modified cardiopulmonary bypass to support life, permitting further treatment and recovery during severe cardiac or pulmonary failure. Due to the artificial circulatory environment of patients on ECMO, this can provide unique challenges in obtaining diagnostic quality CT images. Further complications are not uncommon. For this review, CT scans from over 200 patients acquired over three years were reviewed to curate the best exemplars of cases with complications related to ECMO.
**Results or Findings:** This review will cover the basics of ECMO, including the differences between venovenous (VV) and venoarterial (VA) ECMO and it will equip the reader with scanning techniques. We will then examine the expected normal range of findings post-initiation of ECMO therapy and a range of complications arising from this process.
The most frequent complication during ECMO is haemorrhage.
A variety of complications can occur during cannulation, including vessel perforation with haemorrhage, arterial dissection, pseudoaneurysm formation, cardiac injury, and incorrect cannula location. Pleural effusion, pneumothorax, pneumomediastinum, hemomediastinum, hemopericardium, and subcutaneous emphysema can be seen due to traumatic cannula insertion or malpositioned cannula.
Clot formation in the circuit is a common complication. Systemic thromboembolism due to thrombus formation within the extracorporeal circuit is a complication that can be devastating.
CT images exemplifying these findings will be shared in the presentation.

**Conclusion:** Complications related to ECMO are not uncommon, some of which can be life-threatening. Awareness of normal CT findings, and these complications is essential for the accurate and early detection of these.
**Limitations:** Not applicable
**Ethics Committee Approval:** Not applicable
**Funding for this study:** No funding received for this study.
**Author Disclosures:**
Hakki Çelik: Nothing to disclose
Jonathan Weir-McCall: Nothing to disclose
Luigia Luigia D'Errico: Nothing to disclose
Sadia Sultana: Nothing to disclose
Sanjay Kumar Bobby Agrawal: Nothing to disclose
Ikenna Raymond Okam: Nothing to disclose
Tak Houng Yu: Nothing to disclose
Allanah Barker: Nothing to disclose

### RPS 215-4

**Retrograde peroneal artery approach to infra-inguinal arterial chronic total occlusion**

*L. Patrone*[1], G. Falcone[2], T. Bisdas[3], D. Akhtar[1], F. Fanelli[2], E. Blessing[4]; [1]London/UK, [2]Florence/IT, [3]Athens/GR, [4]Karlsbad/DE
*(Lorenzo.patrone@nhs.net)*

**Purpose:** The aim of this study is to assess the feasibility, safety and effectiveness of retrograde PA access and to provide some technical details on the effective use and management of this retrograde access.
**Methods or Background:** We retrospectively analyzed 137 consecutive patients (137 limbs) (84 males [61,3%]; median age, 76,8± 10,9 years old; range 44-94 years) who underwent endovascular recanalization of infra-inguinal arteries for symptomatic PAD-CLTI using a retrograde PA access in case of a failed antegrade attempt. The target of the recanalization via the PA access was the superficial femoral artery (SFA) and/or popliteal artery in 58 pts (42,3%), the peroneal artery and/or the tibio-peroneal trunk in 25 patients (18,2%), and both segments in 54 pts (39,4%).
**Results or Findings:** The retrograde puncture of the PA was successful in 98,5% cases. In 8,8% cases backflow was obtained but the intraluminal delivery of the guidewire failed. The supplemental use of the retrograde approach allowed a successful retrograde lesion crossing in 90,5 % of the patients. In all these cases to deliver adjunctive therapy (including plain balloon angioplasty, drug eluting balloons, bare metal stents, drug eluting stents, atherectomy) was possible, as from operators' choice.
In the majority of the cases (92,7 %) a sheat-less approach was pursued while in 7,3% of cases a 4Fr sheath was inserted. At the final angiogram haemostasis at puncture site was successfully achieved in 137 cases (100%). The total rate of retrograde puncture-related complication rate was 2,2%.
**Conclusion:** The retrograde recanalization of infra-inguinal arterial CTO via the peroneal access is a safe and effective bailout technique, useful in the most challenging cases of recanalization of the infra-inguinal vessels.
**Limitations:** Retrospective study.
**Ethics Committee Approval:** Not needed
**Funding for this study:** No
**Author Disclosures:**
Erwin Blessing: Consultant at Abbott, Shockwave, Cardinal Health
Fabrizio Fanelli: Consultant at Philips, Cook
Lorenzo Patrone: Consultant at Abbott, Terumo, Pathfinder Medical, Shockwave
Daoud Akhtar: Nothing to disclose
Giammarco Falcone: Nothing to disclose
Theodosios Bisdas: Consultant at Shockwave, Medtronic

### RPS 215-5

**3D-arterial analysis software and CEUS in the assessment of severity and vulnerability of carotid atherosclerotic plaques in comparison with CTA and histology**

*D. Fresilli*, G. Polti, E. Polito, G. T. Lucarelli, O. Guiban, S. Cardaccio, P. Pacini, V. Cantisani, C. Catalano; Rome/IT
*(daniele.fresilli@hotmail.it)*

**Purpose:** To assess multiparametric Ultrasound (US) efficacy in the evaluation of carotid plaque vulnerability and stenosis measurement.
**Methods or Background:** 134 patients with carotid plaques to undergo endarterectomy were enrolled. All carotid plaques were evaluated with Color-Doppler-US, CEUS and 3D ultrasound using 3D Arterial analysis software and were compared with CTA for estimating carotid stenosis and with histology for the plaque vulnerability assessment. 3D-Arterial Analysis provided a colour map based on plaque vulnerability and a volumetric stenosis evaluation.
**Results or Findings:** 94 vulnerable plaques at histological examination were identified. CDUS, 3D-Arterial Analysis software, CEUS and CTA were able to detect 77, 84, 82 and 82 vulnerable plaques respectively, with a sensitivity of 82%, 89%, 87% and 87% and a specificity of 90% for CDUS and 100% for CTA, CEUS and 3D Arteriale Analysis. CTA has identified 84 severe stenosis of which 83 were correctly evaluated by 3D-Arterial Analysis software and CEUS, with a sensitivity of 99% and specificity of 100% and 71 by CDUS with a sensitivity of 85% and specificity of 80%.
**Conclusion:** The preliminary results of our study confirm that the integration of the new ultrasound softwares, CEUS and 3D Arterial Analysis, to the eco-color-Doppler examination can improve the management of the carotid plaque. However, further studies with larger populations are needed in order to establish whether they can be useful to direct the patient towards surgical procedures and / or can be a screening tool to stratify early those patients who could develop vulnerable plaques.
**Limitations:** Large 3D probe size, sulfur allergy and sampling bias.
**Ethics Committee Approval:** All procedures performed were in accordance with the ethical standards of the institutional and/or national research committee.
**Funding for this study:** n/a
**Author Disclosures:**
Daniele Fresilli: Nothing to disclose
Patrizia Pacini: Nothing to disclose
Sara Cardaccio: Nothing to disclose
Olga Guiban: Nothing to disclose
Giorgia Polti: Nothing to disclose
Eleonora Polito: Nothing to disclose
Giuseppe Tiziano Lucarelli: Nothing to disclose
Vito Cantisani: Nothing to disclose
Carlo Catalano: Nothing to disclose

### RPS 215-6

**Carotid plaque score associated with the presence and severity of coronary atherosclerosis**

*Y. Tang*, M. Chen, H. Yunqian; Shanghai/CN
*(yhtangsz@163.com)*

**Purpose:** To determine whether carotid plaque score(CPS) counted by the number of ultrasound-detected plaques and carotid intima-media thichness(IMT) are associated with the presence and severity of coronary atherosclerosis.
**Methods or Background:** 153 patients were enrolled in this study with confirmed or suspected coronary heart disease who were scheduled to undergo coronary angiography and carotid ultrasound. CPS was calculated by summation of the number of plaques in the common, bifurcation, and internal carotid artery segments. Measurements of the right and left commom carotid far wall approximately 1cm proximal to the carotid bifurcation were taken to derive carotid IMT and the thicker one was recorded. Coronary severity was evaluated by Gensini score and the number of diseased coronary arteries. The correlation of CPS and carotid IMT with the presence and severity of coronary atherosclerosis was assessed.
**Results or Findings:** Patients with diseased coronary arteries had higher CPS than those with no diseased coronary artery, $P<0.01$. According to Gensini score tertiles CPS in low Gensini score group was significantly lower than that in middle-high Gensini score group (0.94±1.10 vs 1.74±1.59, $p=0.002$). However, although the carotid IMT of middle-high Gensini score group was increased , there was no statistically significant difference in the carotid IMT between different Gensini score groups. Logistic regression analysis showed that CPS was independently associated with the severity of coronary atherosclerosis (OR=1.637, 95%CI: 1.149-2.333, $p=0.006$).
**Conclusion:** CPS calculated by summation of the number of plaques was related to the presence and severity of Coronary Atherosclerosis. CPS detection has potential value for noninvasively identifying the severity of coronary atherosclerosis.
**Limitations:** This was a cross-sectional study with a small sample size, which might partially account for some statistically non-significant correlations.
**Ethics Committee Approval:** This study protocol was approved by the clinical ethics committee.
**Funding for this study:** n/a
**Author Disclosures:**
Yuhua Tang: Nothing to disclose
Huang Yunqian: Nothing to disclose
Man Chen: Nothing to disclose

### RPS 215-7

**3D-printing for preoperative planning and modeling of physician-modified branched stent-grafts for the complex treatment of the aortic arch thoracic aneurysms: one centre's experience**

*M. Wojtuń*, M. P. Kawa, P. Rynio, P. Gutowski, A. Falkowski, A. Kazimierczak, T. Jędrzejczak, Ł. Wójcik, S. Waligórski; Szczecin/PL
*(wojtunmaciek@gmail.com)*

**Purpose:** Study was to sum up own experience, outcomes of thoracic endovascular aortic repair(TEVAR) for anatomically-complicated aneurysms in aortic arch using three-dimensional(3D)printing to guide application of physician-modified branched stent-grafts.This innovative procedure was

Sunday developed in our Cardio-Vascular DiseasesCenter located in University Hospital of PomeranianMedicalUniversity in Szczecin,Poland. Our institution is tertiary referral clinical centre for patients with complex aortic pathology.Our group previously performed 3Dprinting application for modeling physician-modified branched stent-grafts for treatment of AAA in 35patients, permitting our surgeons and radiologists were familiarized to this technology that aids planning,reduces time of surgery,decreasing intra-and post-operative complications,augmenting recovery. However,there was lack of knowledge whether 3Dprinting can be used to help guide treatment of aortic arch aneurysms,that are highly complicated pathology.
**Methods or Background:** Patients with aortic arch aneurysms were treated in our center.Preoperative angio-CT from all 10patients were prospectively collected and digitally reconstructed using dedicated software.Next, 3D-model of aortic arch aneurysm was printed,according to corresponding angio-CT.Furthermore, physician-modified branched stent-grafts were prepared as indicated by personalized 3D-printedAorta model.
**Results or Findings:** All angio-CTimages matched with their 3D-printed models were discussed among vascular-and cardio-surgeons, vascular-trained radiologist before and after TEVAR,whether anatomical reliability of 3Dmodels was sufficient.Due to preoperative existence of 3D-printed models the personally-tailored stent-grafts modified by physicians in the operating room could be made without significant errors.
**Conclusion:** We demonstrated utility and feasibility of a 3D-printed template of aortic arch in construction of fenestrated modified stent-grafts.Complex procedure was technically successful. 3D
models would make a great contribution on surgical planning and intraoperative modification of branched stent-grafts in patients with anatomically-complicated aneurysms. However,further radiological research and reconstruction imaging techniques development are needed.Especially, dedicated study is required for advanced assessment and accuracy of 3D-printed models.
**Limitations:** Without limitations.
**Ethics Committee Approval:** Retrospective study, approved.
**Funding for this study:** There was no special funding.
**Author Disclosures:**
Maciej Wojtuń: Nothing to disclose
Arkadiusz Kazimierczak: Nothing to disclose
Milosz P. Kawa: Nothing to disclose
Łukasz Wójcik: Nothing to disclose
Szymon Waligórski: Nothing to disclose
Piotr Gutowski: Nothing to disclose
Aleksander Falkowski: Nothing to disclose
Paweł Rynio: Nothing to disclose
Tomasz Jędrzejczak: Nothing to disclose

**Live Q&A**

**18:00-19:30 CHANNEL 5**

### Healthcare Professionals in Focus

### IF 4

### Make your own resilience and well-being plan

**Moderators**
Michael H. Fuchsjäger; Graz/AT
M.G. Myriam Hunink; Rotterdam/NL

### IF 4-2
**Keynote speaker: Surviving in times of chaos: creating your personal resilience/well-being plan**
Peter Moskowitz; Palo Alto, CA/US

**19:45-20:45 CHANNEL 2**

### E³ - Rising Stars Programme: Basic Sessions

*Organised by the EFRS*

### BS 1

### Challenges in cross-sectional imaging

**Moderator**
Carst Buissink; Groningen/NL

### BS 1-2
**Technical challenges in MRI: overcoming metal artefacts**
Jean-Philippe Dillenseger; Strasbourg/FR

**Learning Objectives:**
1. To provide an overview of the most common metal artefacts in MRI.
2. To highlight the importance of limiting the impact of metal artefacts on safety and diagnosis.
3. To provide practical suggestions on how to limit artefacts in MRI.

### BS 1-3
**Challenges in contrast administration to vulnerable patients in CT**
Cathrine Helgestad Kristiansen; Oslo/NO
*(Cathrine.Helgestad.Kristiansen@ahus.no)*

**Learning Objectives:**
1. To learn how to identify vulnerable patients.
2. To identify the challenges in contrast administration to paediatric and elderly patients.
3. To provide strategies for reducing the risks in the administration of contrast to vulnerable patients.

### BS 1-4
**An overview of technical and physical artefacts in ultrasound**
Barbara Kraus; Vienna/AT
*(barbara.kraus@fh-campuswien.ac.at)*

**Learning Objectives:**
1. To provide an overview of the most common artefacts in US.
2. To highlight the importance of limiting the impact of artefacts on diagnosis.
3. To provide practical suggestions on how to limit artefacts in US.

**Live Q&A: How to ensure quality control in cross-sectional imaging in daily practice?**

**19:45-20:45 CHANNEL 3**

### E³ - ECR Master Class

### E³ 1626

### Whole-body MRI (and PET/MRI)

**Moderator**
Davide Prezzi; London/UK

### E³ 1626-2
**A. Metastatic bone prostate cancer**
Frédéric E. Lecouvet; Brussels/BE

**Learning Objectives:**
1. To define imaging strategies in newly diagnosed, recurrent, and advanced disease.
2. To learn to use MET-RADS-P guidelines for whole-body MRI acquisition, reading, and response assessment.
3. To illustrate recent therapeutic advances: i.e. oligometastatic disease and precision medicine approaches.

### E³ 1626-3
**B. Multiple myeloma**
Ioana G. Lupescu; Bucharest/RO
*(ilupescu@gmail.com)*

**Learning Objectives:**
1. To become aware of the value of whole-body MRI in myeloma.
2. To understand current imaging protocols.
3. To learn about its roles: detect, monitor, and provide prognostic information.

### E³ 1626-4
**C. Lymphoma**
Marius E. Mayerhöfer; Vienna/AT
*(marius.mayerhoefer@meduniwien.ac.at)*

**Learning Objectives:**
1. To introduce the role of whole-body MRI and PET/MRI for imaging lymphoma subtypes.
2. To know its value for detection, assessment of response, and prognosis.
3. To understand the current advantages and limitations of whole-body MRI and PET/MRI.

**Author Disclosures:**
Marius E. Mayerhöfer: Speaker: Siemens, BMS

**Live Q&A**

**19:45-20:45 CHANNEL 4**

### Research Presentation Session: Emergency Imaging

### RPS 117

### The emergency department sees some of the most complex patients: how radiology can help

**Moderator**
Elika Kashef; London/UK

### RPS 117-3
**Accuracy of MRI imaging for acute abdominal pain in pregnancy**
*B. Buckley*, M. C. Murphy, F. Delaney, T. Geoghegan, C. G. Cronin; Dublin/IE

**Purpose:** The aim of this study is to quantify the accuracy of MRI in detecting acute appendicitis in a pregnant population in a single tertiary referral centre.
**Methods or Background:** A retrospective review was carried out of all MRI abdomen studies performed on pregnant patients for assessment of appendicitis between 2017 and 2020. All MRI reports were reviewed for the presence of appendicitis or identification of a normal appendix, its size and the presence of secondary signs of inflammation. These were compared with intra-operative reports and histology where available, follow up outpatient records and clinical notes.
**Results or Findings:** Between 2017 and 2020, 22 MRI abdomens we performed on pregnant patients for the assessment of acute appendicitis. Of these, 7 patients were identified at time of MRI to have acute appendicitis with 6 of those undergoing appendectomy with confirmed acute appendicitis on histology reports. 1 patient was reported to have a normal visualised appendix which was subsequently demonstrated to be acute appendicitis on histology. This yielded an accuracy in diagnosis of 93% with a sensitivity of 85% and specificity of 95%. The appendix was not visualised in 10 patients.
**Conclusion:** MRI is a safe and highly accurate tool for the assessment of acute appendicitis in pregnancy. Our institution demonstrated an accuracy of 93% in diagnosing acute appendicitis at time of MRI with a high negative predictive value of 95%.
**Limitations:** None.
**Ethics Committee Approval:** Not required. Completely anonymised data.
**Funding for this study:** None.
**Author Disclosures:**
Carmel Geraldine Cronin: Nothing to disclose
Tony Geoghegan: Nothing to disclose
Mark Conal Murphy: Nothing to disclose
Bryan Buckley: Nothing to disclose
Francis Delaney: Nothing to disclose

### RPS 117-4
**Multidetector-row CT (MDCT) findings in patients with non-occlusive mesenteric ischaemia (NOMI): influence of vasoconstrictive agents**
*A. B. Topolsky*, O. Pantet, L. Liaudet, J-F. Knebel, A. Denys, S. Schmidt; Lausanne/CH

**Purpose:** To find out if there is an influence of vasoconstrictive agents (VCA) on MDCT signs of vasoconstriction and bowel ischemia detected in patients with non-occlusive mesenteric ischemia (NOMI).
**Methods or Background:** This 8-years single-center retrospective study included all patients with histopathologically proven NOMI and MDCT examination performed ≤48h prior to surgical management. Two blinded radiologists jointly reviewed the MDCT examinations for presence and distribution of bowel ischemia signs, abdominal organ infarct, mesenteric vessels size and regularity, and ancillary vascular findings.VCA administration, clinical and biochemical data, risk factors, and outcome were subsequently retrieved.Subgroups comparisons were performed.
**Results or Findings:** Of the 90 patients (59 males, mean age 65), 44.4% (n=40) had received VCA before MDCT.
Overall mortality was 32% (n=29), with no significant difference between groups (p>0.05).
In the VCA group, superior mesenteric artery (SMA) caliber was significantly smaller (p=0.014) and vasoconstriction of its branches was more commonplace (p=0.048). Although neither the presence nor the extent of bowel ischemia significantly correlated with VCA administration, abdominal organ infarcts were more frequent (p=0.005) and involved more organs (p=0.016).
The VCA group had lower mean arterial pressure (p=0.006) and lower hemoglobin levels (p<0.001). Inflammatory biomarkers, namely leukocytosis (p=0.005) and CRP (p<0.001), were significantly higher. Of the tested organ-failure biomarkers, only serum creatinine (p<0.012) and urea (p<0.036) differed significantly, while there was no difference for serum lactate and other arterial-blood gas test results.
**Conclusion:** VCA administration in NOMI patients is associated with more severe SMA vasoconstriction and abdominal organ infarct, without decreased survival.
**Limitations:** This is a single-center retrospective study.
**Ethics Committee Approval:** This single-centre retrospective study was approved by our institutional ethics committee.
**Funding for this study:** None.
**Author Disclosures:**
Alban Denys: Nothing to disclose
Jean-François Knebel: Nothing to disclose
Olivier Pantet: Nothing to disclose
Antoine Blagoev Topolsky: Nothing to disclose
Lucas Liaudet: Nothing to disclose
Sabine Schmidt: Nothing to disclose

### RPS 117-5
**Thoraco-abdominopelvic CT in the evaluation and follow-up of trauma patients admitted to the emergency department: a single-institute experience**
*D. D. Ekizalioğlu*, E. Güler, A. Z. Öcek, M. Songür Kodik, M. Harman, N. Elmas; İzmir/TR
*(duygudogaeao@gmail.com)*

**Purpose:** To investigate the frequency, imaging findings, and follow-up of adult patients who were admitted to the emergency department(ED) due to trauma and had CT of the thorax, abdomen, and pelvis(CT-TAP).
**Methods or Background:** CT-TAP scans of patients who were admitted to the ED due to trauma between January 2019 and January 2020 were retrospectively evaluated. Trauma-related imaging findings were investigated. The hospitalization and interventions that the patients underwent were searched from the electronic medical records. The patients who had follow-up CT-TAP were identified and the examinations were reviewed.
**Results or Findings:** A total of 985 trauma patients who had CT-TAP in the ED were identified. In 436 patients (44.3%)(150 women, 286 men; mean age 47±19 years) trauma-related imaging findings were detected. 549(55.7%) patients (161 women, 388 men; mean age 41±17 years) had normal CT-TAP. Bone injury was the most commonly identified imaging finding as it was positive in 334 out of 436 patients (76.6%). Lung injury was detected in 85 (19.5%) and pneumothorax was found in 77 (17.7%) patients. 68 (15.6%) patients had retroperitoneal hemorrhage, 31 (7.1%) had splenic injury and 29 (6.7%) had liver injury. Presence of bone injury was associated with both thoracic and abdominopelvic injuries; however these associations were moderate according to effect size (ES=0.402, ES=0.306, p<0.001, respectively). 201 (46.1%) patients with trauma-related imaging findings were hospitalized. There was progression in the follow-up CTs of 40 (9.2%) patients. 74 (17%) cases underwent radiological or surgical intervention.
**Conclusion:** Trauma-related imaging findings were detected in 44.3% of the patients who had CT-TAP in the ED. Bone injury was the most commonly identified imaging finding.
**Limitations:** The retrospective design and the lack of comparison with clinical findings of trauma.
**Ethics Committee Approval:** It was approved by institutional review board.
**Funding for this study:** None.

**Author Disclosures:**
Meltem Songür Kodik: Nothing to disclose
Duygu Doğa Ekizalioğlu: Nothing to disclose
Nevra Elmas: Nothing to disclose
Aslı Zeliha Öcek: Nothing to disclose
Ezgi Güler: Nothing to disclose
Mustafa Harman: Nothing to disclose

### RPS 117-6

**An audit on the use of CT angiography for cases of suspected acute aortic syndrome referred from the emergency department in an Irish teaching hospital**

*D. T. Ryan*, C. Doherty, B. Snow, J. Morrow, R. Macdermott, D. Murphy; Dublin/IE
*(davidtryan@hotmail.com)*

**Purpose:** Acute aortic syndrome (AAS) is a common presentation to ED with CT angiography (CTA) being the investigation of choice. Despite having a defined protocol in our institution, deviation from this is common. We audited the use of CTA for cases of suspected AAS, looking at the protocols used, radiation dose and positivity rate.
**Methods or Background:** Retrospective study of 106 consecutive CTA requests referred from our Emergency Department (ED) for possible AAS from March 2019 – January 2020. We excluded patients with multiple scans (e.g. CT pulmonary angiograms or CT brains), those who underwent dedicated cardiac CTs and non-ED requests. We audited protocols used, radiation dose and positivity rate.
**Results or Findings:** 106 cases of suspected AAS were included. The average age was 62 years old with a male predominance (64%). 69% of studies included a shortened scan range, such as the thorax only. 29% omitted a delayed phase, incorporating just a non-contrast (NC) and arterial phase. 4.7% were positive for AAS. 26% had another cause for symptoms such as pneumonia or pulmonary emboli. 24% had clinically significant incidental findings such as lung cancers or adrenal masses. Average effective dose for all scans was 15.6mSv and for our original triphasic CT thorax, abdomen and pelvis (TAP) protocol was 23.5mSv. The delayed phase was non-contributory in all positive AAS cases. Changing to just a NC thorax (for intramural haematoma assessment) and arterial phase TAP has achieved a new average affective dose of 11.7mSv or dose reduction of 50%.
**Conclusion:** We propose standardising CTA protocols for suspected cases of acute aortic syndrome to a non-contrast thorax and arterial phase TAP to optimise dose, without decreasing diagnostic yield.
**Limitations:** Retrospective study, excluded those with multiple scans, small sample size.
**Ethics Committee Approval:** Approved
**Funding for this study:** None
**Author Disclosures:**
Barbara Snow: Nothing to disclose
Cathal Doherty: Nothing to disclose
David Thomas Ryan: Nothing to disclose
Joseph Morrow: Nothing to disclose
Roisin Macdermott: Nothing to disclose
David Murphy: Nothing to disclose

### RPS 117-7

**Retroperitoneal haematoma: from the CT room to the interventional radiology suite**

*D. Herrán De La Gala*, T. Cobo Ruiz, D. Castanedo Vázquez, J. Azcona Sáenz, P. Menéndez Fernández-Miranda, A. Pérez del Barrio, E. Julián Gómez, J. Jordá Lope, B. García Martínez; Santander/ES
*(herrandario@gmail.com)*

**Purpose:** To review the retroperitoneal anatomy and its appearance on CT.
To describe the appropriate CT protocol for evaluating retroperitoneal hematomas.
To review the different etiologies of retroperitoneal hematoma.
To describe imaging pitfalls that can lead to false positives and false negatives in evaluating active bleeding.
**Methods or Background:** The retroperitoneum it's a space delimited anteriorly by the posterior parietal peritoneum and posteriorly by the posterior abdominal wall. It can be divided in three spaces: anterior pararenal space, perirenal space and posterior pararenal space. Retroperitoneal hemorrhage can be the source of significant but clinically occult blood loss. There are different conditions that can lead to retroperitoneal hematoma (RH) and we must address the presence or absence of active bleeding on CT imaging.
**Results or Findings:** Trauma is the leading cause of RH. In the absence of trauma history, underlying conditions must be scrutinized in order to justify the hematoma: iatrogenic, abdominal aorta aneurysms, inferior vena cava rupture, renal or suprarenal lesions, hemorrhagic pancreatitis or, lastly, spontaneous retroperitoneal bleeding. It is recommended to evaluate RH in multi-phase CT scan, where non-enhanced acquisitions are key. Evaluating the presence of active bleeding is imperative to discuss whether the patient might benefit from endovascular treatment or not.
**Conclusion:** -Non-enhanced CT scan is imperative while evaluating RH.
-Describing and assessing the RH distribution maybe useful in finding its origin.
-In the absence of trauma history, we must search for underlying conditions that can justify the RH.
-Describing the presence of active bleeding and its possible origin helps in evaluating the need for endovascular treatment.
**Limitations:** Not applicable
**Ethics Committee Approval:** Not applicable
**Funding for this study:** Not applicable
**Author Disclosures:**
Amaia Pérez del Barrio: Nothing to disclose
Pablo Menéndez Fernández-Miranda: Nothing to disclose
Juan Jordá Lope: Nothing to disclose
Javier Azcona Sáenz: Nothing to disclose
Teresa Cobo Ruiz: Nothing to disclose
Beatriz García Martínez: Nothing to disclose
David Castanedo Vázquez: Nothing to disclose
Elena Julián Gómez: Nothing to disclose
Darío Herrán De La Gala: Nothing to disclose

### RPS 117-8

**Perceive the "invisible": enhancing the visibility of foreign body in soft tissue injuries in x-ray**

*W. Tam*; Leeds/UK
*(tws_winnie19972004@hotmail.com)*

**Purpose:** Foreign bodies (FB) injuries can cause serious complications if unremoved (1,2,3). The visibility of low-density FBs, in which soft-tissue has a similar density to, making FB detections difficult in x-ray. The aim is to investigate how to enhance the contrast between the two, and in turn, increase the chance of FBs recognition.
**Methods or Background:** A literature review, evaluating if there is an up-to-date advice on this matter. Literature on non-medical x-ray usage was also consulted to learn if medical imaging can utilise some of these techniques to aid detection.
**Results or Findings:** The availability of up-to-date literature on imaging technique for FB injuries is limited. The contrast between the FB and its surrounding soft-tissue must be higher than the just-noticeable-difference-threshold of human eyes for distingushment. The just-noticeable-difference-thresholds for monochrome is 0.08 (4,5), compares to 0.025, 0.026 and 0.023 for red, blue and green respectively (6). By colourising monochromic images, a smaller difference between shades can be perceived and potentially aids the differentiation of FBs to their surrounding soft-tissue. Fovea, the area that provides the sharpest vision, only contains colour-detecting photoreceptors (7). Thus, pseudocolour enhances the subtle differences (8,9), and increases the likelihood for FBs identifications. Pseudocolour is widely used in non-medical x-ray images to assist detection (10,11,12).
**Conclusion:** There are no evidence-based suggestions on imaging technique for low-density FB injuries. Pseudocolour is commonly used in other industries for detection. Shockingly, an extremely limited amount of literature is currently available on the potential of pseudocolour in medical images, further research is urgently needed.
**Limitations:** The amount of literature reviewed was not optimal due to the insufficient literature availability. But this signified the need of further investigation, for the sake of best patient care and profession advancement.
**Ethics Committee Approval:** Na
**Funding for this study:** Na
**Author Disclosures:**
Winnie Tam: Nothing to disclose

**Live Q&A**

**19:45-20:45** **CHANNEL 5**

### Coffee & Talk (open forum) Session

*Organised by the ESR Subcommittee on Undergraduate Education*

### C 1

### Attracting the next generation of radiologists: exploring ways forward

**Moderator**
Laura Oleaga Zufiría; Barcelona/ES

### C 1-2
**Future radiologists: the importance of early engagement**
Sofia Gourtsoyianni; Athens/GR
*(sgty76@gmail.com)*

**Learning Objectives:**
1. To be aware of the different strategies of early engagement across Europe.
2. To appreciate the benefits and challenges to effective early engagement.

### C 1-3
**Undergraduate radiology societies: the UK experience in bringing together future radiologists**
Jim Zhong, Rosemary Ho; Leeds/UK

**Learning Objectives:**
1. To learn the role and process of setting up undergraduate radiology societies.
2. To appreciate the benefits of early engagement through undergraduate societies.

### C 1-4
**Undergraduate and postgraduate degrees: do they bring undergraduates into radiology?**
Vicky J. Goh; London/UK
*(vicky.goh@kcl.ac.uk)*

**Learning Objectives:**
1. To be aware of undergraduate and postgraduate degrees involving radiology.
2. To understand if these are effective in bringing doctors into radiology.
**Author Disclosures:**
Vicky J. Goh: Research Grant/Support: Siemens Healthcare

### C 1-5
**Current ESR education initiatives: an update**
Carlo Catalano; Rome/IT
*(Carlo.Catalano@uniroma1.it)*

**Learning Objectives:**
1. To be aware of ESR's activities in attracting new doctors into radiology.
2. To be aware of ESR's activities for radiologists in training.

**Live Q&A: Attracting the next generation: how to achieve more in Europe?**

### B

### On-Demand Programme

$E^3$ - Rising Stars Programme: Basic Sessions (BS)
$E^3$ - Rising Stars Programme: Student Sessions (S)
$E^3$ - The Beauty of Basic Knowledge ($E^3$)
$E^3$ - European Diploma Prep Sessions ($E^3$)
Special Focus Sessions (SF)
State of the Art Symposia (SA)
Professional Challenges Sessions (PC)
Coffee & Talk (open forum) Session (C)
Refresher Courses (RC)
Research Presentation Sessions (RPS)
$E^3$ - Advanced Courses ($E^3$)
EFOMP Workshop (EF)
EuroSafe Imaging Sessions (EU)

### E³ - Rising Stars Programme: Basic Sessions

*Organised by the EFRS*

### BS 2

### Introducing elastography

**BS 2-1**
**Ultrasound elastography techniques**
Malene Roland Vils Pedersen; Vejle/DK
*(malene.roland.vils.pedersen@rsyd.dk)*

**Learning Objectives:**
1. To explain the principles of elastography technique in US.
2. To discuss the advantages and disadvantages of the different types of elastography techniques in US.
3. To provide practical tips and tricks in the application of the technique

**BS 2-2**
**MRI elastography techniques**
Claude Portanier Mifsud; Msida/MT

**Learning Objectives:**
1. To explain the principles of elastography technique in MRI.
2. To discuss advances in MR elastography.
3. To provide practical tips and tricks in the application of the technique.

**BS 2-3**
**Clinical applications of elastography**
Allison McGee; Dublin/IE

**Learning Objectives:**
1. To indicate clinical areas for elastography examinations.
2. To indicate clinical indications of elastography examinations.
3. To consider the associated risks of elastography techniques.
**Author Disclosures:**
Allison McGee: Employee: Employee of an Educational Institution; Speaker: Invited to contribute to ECR 2021

### E³ - Rising Stars Programme: Basic Sessions

*Organised by the EFRS*

### BS 3

### Radiation dose and image quality optimisation

**BS 3-1**
**Patient shielding in diagnostic radiography: is it effective?**
Nejc Mekis; Ljubljana/SI

**Learning Objectives:**
1. To explain the difference between in-plane and out-of-plane shielding.
2. To explain the pros and cons of shielding.
3. To provide an updated overview of position statements on shielding.

**BS 3-2**
**Principles of radiation dose optimisation**
Joana Santos; Coimbra/PT
*(joanasantos@estescoimbra.pt)*

**Learning Objectives:**
1. To emphasise the need for radiation dose optimisation.
2. To discuss the role of diagnostic reference levels (DRLs) in the dose optimisation strategy.
3. To understand the importance of radiation dose monitoring.

**BS 3-3**
**Image quality evaluation methods**
Mark F. McEntee; Cork/IE
*(mark.mcentee@ucc.ie)*

**Learning Objectives:**
1. To provide an overview of image quality requirements in diagnostic imaging.
2. To become familiar with the available image quality assessment methods.
3. To become aware of appropriate image quality analysis.

### E³ - Rising Stars Programme: Basic Sessions

*Organised by the EFRS*

### BS 4

### Equality, diversity, and inclusion (EDI) issues for radiography

**BS 4-1**
**An introduction to the concept**
Louise A. Rainford; Dublin/IE
*(louise.rainford@ucd.ie)*

**Learning Objectives:**
1. To introduce the Athena Swan.
2. To understand the importance of the Athena Swan for radiography.
3. To emphasise how the Athena Swan can impact positively on radiography training centres.

**BS 4-2**
**Application within radiography curricula**
Ruth Strudwick; Ipswich/UK
*(r.strudwick@uos.ac.uk)*

**Learning Objectives:**
1. To incorporate EDI in radiography curricula.
2. To discuss EDI considerations, including clinical skills labs and clinical.
3. To demonstrate the impact on the student code of conduct.

**BS 4-3**
**Ensuring appropriate patient care in the clinical environment**
Karen Knapp; Exeter/UK
*(K.M.Knapp@exeter.ac.uk)*

**Learning Objectives:**
1. To discuss EDI policies in the clinical environment.
2. To understand the requirements of CPD to support clinical radiographers and appreciate the topic.
3. To safeguard patient rights.

### E³ - Rising Stars Programme: Basic Sessions

*Organised by the EFRS*

### BS 5

### Radiography: raising the profile of our profession

**BS 5-1**
**Being a professional: the EFRS perspective and why it matters**
Jonathan McNulty; Dublin/IE
*(jonathan.mcnulty@ucd.ie)*

**Learning Objectives:**
1. To discuss radiography as a profession.
2. To understand the importance of being a professional.
3. To be aware of the EFRS perspective of radiographers as professionals.

**BS 5-2**
**Using social media to inform others about our profession**
Graciano Paulo; Coimbra/PT
*(graciano@estescoimbra.pt)*

**Learning Objectives:**
1. To provide an overview of what social media is, what is available, and how to identify which is the best for promoting the profession.
2. To learn how to maximise social media use in promoting radiography amongst health professionals.
3. To learn how to maximise social media use in promoting radiography amongst the general public.

**BS 5-3**
**A radiographers' perspective: the importance of highlighting the role of radiographers to patients**
Charlotte Beardmore; London/UK
*(CharlotteB@sor.org)*

**Learning Objectives:**
1. To discuss the relevance of the radiographer's role within the health care system.
2. To highlight issues patients may have in distinguishing between radiology and radiography.
3. To discuss whether the radiographer's responsibility is in satisfying the patient's expectations.

### E³ - Rising Stars Programme: Basic Sessions
*Organised by the ESR*

### BS 6
### Abdominal viscera: liver imaging

**BS 6-1**
**Benign liver lesions**
Andrea Laghi; Rome/IT
*(andrea.laghi@uniroma1.it)*

**Learning Objectives:**
1. To present current imaging techniques for evaluation of benign liver lesions.
2. To describe the typical features of benign liver lesions.
**Author Disclosures:**
Andrea Laghi: Speaker: Bracco, GE Healthcare, MSD, Bayer, Guerbet

**BS 6-2**
**Malignant liver lesions**
Ioana G. Lupescu; Bucharest/RO
*(ilupescu@gmail.com)*

**Learning Objectives:**
1. To present the current imaging techniques for evaluation of malignant liver lesions.
2. To become familiar with the typical findings of malignant liver lesions.

**BS 6-3**
**MRCP: state-of-the-art diagnosis of the biliary system**
Ahmed Ba-Ssalamah; Vienna/AT
*(ahmed.ba-ssalamah@meduniwien.ac.at)*

**Learning Objectives:**
1. To present technical aspects and indications of MRCP.
2. To become familiar with the typical MRCP findings in benign and malignant biliary diseases.

### E³ - Rising Stars Programme: Basic Sessions
*Organised by the ESR*

### BS 7
### Genitourinary

**BS 7-1**
**Imaging of the uterus**
Riccardo Manfredi; Rome/IT
*(riccardo.manfredi@unicatt.it)*

**Learning Objectives:**
1. To learn about the uterine anatomy.
2. To present the current imaging techniques for the evaluation of the uterus.
3. To demonstrate the most important findings of common uterine pathologies.

**BS 7-2**
**Imaging of the adnexa**
Sanja Stojanovic; Novi Sad/RS

**Learning Objectives:**
1. To learn about the adnexa anatomy.
2. To present the current imaging techniques for the evaluation of the adnexa.
3. To demonstrate the most important findings of adnexal pathologies.

**BS 7-3**
**Imaging gynaecological emergencies**
Rosemarie Forstner; Salzburg/AT
*(r.forstner@salk.at)*

**Learning Objectives:**
1. To present current imaging techniques for the evaluation of gynaecological emergencies.
2. To illustrate the spectrum of imaging features in gynaecological emergencies in non- pregnant and pregnant women.

### E³ - Rising Stars Programme: Basic Sessions
*Organised by the ESR*

### BS 9
### Cardiovascular imaging

**BS 9-1**
**Acute coronary artery syndrome**
Maja Pirnat; Maribor/SI

**Learning Objectives:**
1. To present current imaging techniques to evaluate the coronary arteries.
2. To demonstrate the most important imaging findings.

**BS 9-2**
**Imaging in non-ischaemic cardiomyopathy**
Jens Bremerich; Basle/CH
*(jens.bremerich@usb.ch)*

**Learning Objectives:**
1. To present the imaging techniques to evaluate the myocardium.
2. To demonstrate the most important findings.

**BS 9-3**
**Aortic dissection**
Karl-Friedrich Kreitner; Mainz/DE
*(Karl-Friedrich.Kreitner@unimedizin-mainz.de)*

**Learning Objectives:**
1. To present the imaging techniques to evaluate the aorta.
2. To demonstrate the most important findings.

### E³ - Rising Stars Programme: Basic Sessions
*Organised by the ESR*

### BS 10
### Neuroradiology

### BS 10-1
**Stroke**
E. Turgut Tali; Ankara/TR
*(turgut.tali@gmail.com)*

**Learning Objectives:**
1. To learn about imaging features in haemorrhagic stroke.
2. To learn about imaging features in ischaemic stroke

### BS 10-2
**Infection and demyelinating diseases**
Laura Oleaga Zufiría; Barcelona/ES
*(lauraoleaga@gmail.com)*

**Learning Objectives:**
1. To learn about imaging features in brain infections.
2. To learn about imaging features in demyelinating diseases.

### BS 10-3
**Tumours**
Marion Smits; Rotterdam/NL
*(marion.smits@erasmusmc.nl)*

**Learning Objectives:**
1. To learn about imaging features in benign tumours.
2. To learn about imaging features in neoplastic disorders.

**Author Disclosures:**
Marion Smits: Consultant: Parexel Ltd, honorarium paid to institution; Speaker: GE Healthcare, honorarium paid to institution

### E³ - Rising Stars Programme: Basic Sessions
*Organised by the ESOR*

### BS 12
### Radiologic anatomy: abdomen

### BS 12-1
**Small bowel**
Stuart A. Taylor; London/UK

**Learning Objectives:**
1. To appreciate the embryological development of the small bowel.
2. To learn about the anatomy of the small bowel relevant to radiological imaging.
3. To understand the pitfalls of the normal small bowel appearance during imaging interpretation.

**Author Disclosures:**
Stuart A. Taylor: Consultant: alimentiv

### BS 12-2
**Anorectal**
András Palkó; Szeged/HU
*(palkoand@gmail.com)*

**Learning Objectives:**
1. To review the normal anatomy of the anorectum.
2. To learn how well depicted the different anatomical landmarks of the anorectum are with different available imaging methods.
3. To discuss the clinical scenarios for which it is necessary to be aware of the anatomy of the anorectum.

### BS 12-3
**Peritoneum and mesentery**
Panos K. Prassopoulos; Thessaloniki/GR
*(pprasopo@auth.gr)*

**Learning Objectives:**
1. To review the radiologic anatomy of peritoneal ligaments, mesenteries, and omenta on cross-sectional imaging.
2. To describe the most clinically important peritoneal compartments and fluid collections.
3. To discuss the key role of anatomy in the dissemination of malignancies or the restriction of inflammatory processes in the abdomen.

### E³ - Rising Stars Programme: Student Session

### S 2
### Student Session 2

### S 2-1
**Machine learning can predict left ventricular dilatation in non-gated contrast enhanced CT thorax**
*A. Asif*, P. F. P. Charters, B. J. Hudson, J. C. L. Rodrigues; Bath/UK

**Purpose:** The study purpose was to determine the diagnostic accuracy of a fully automated machine-learning (ML) derived tool at detecting left ventricular (LV) dilatation on non-ECG gated contrast-enhanced CT of the thorax relative to contemporaneous cardiac magnetic resonance (CMR) gold-standard.
**Methods or Background:** Consecutive patients (n=77) with contrast-enhanced CT thorax and CMR within a 30-day interval were included. LV dilatation was defined against age, sex and body surface area (BSA) indexed normal values with analysis by a Cardiac Radiologist using cmr42 (Circle Cardiovascular Imaging Inc., Calgary, Canada). All CTs were sent for automated ML-derived analysis that segments the ventricular chambers, presenting maximal LV diameter from trans-axial images (Imbio, Minneapolis, USA). Area under the receiver operator curve (AUC-ROC) analysis identified the automated LV diameter thresholds with ≥90% sensitivity and highest specificity and ≥90% specificity with highest sensitivity.
**Results or Findings:** Automated CT analysis was feasible in all patients (45 men, 32 women, age 61±14 years, mean CT-CMR interval 10±8 days). Relative to CMR gold standard, 45% (35/77) had LV dilatation and 23% (18/77) had RV dilatation. AUC-ROC for automated absolute LV diameter was 0.712 (95% CI 0.59-0.83), p=0.001: absolute LV diameter >41.9mm 91% sensitive and 28% specific for LV dilatation and absolute LV diameter >52.4mm 91% specific and 51% sensitive. Automated LV diameter indexed to BSA improved performance (AUC-ROC 0.738 (95% CI 0.62-0.85), p<0.0001: indexed LV diameter >20.8mm 91% sensitive and 21% specific for LV dilatation and indexed LV diameter >28.0mm 91% specific and 49% sensitive.
**Conclusion:** Automated ML-derived analysis of non-ECG gated contrast enhanced CT thorax compares favourably with detecting LV dilatation relative to CMR gold standard and could be applied routinely to screen for dilated cardiomyopathy.
**Limitations:** Retrospective, single centre.
**Ethics Committee Approval:** Approved by trust audit committee.
**Funding for this study:** Nil
**Author Disclosures:**
Pia Charters: nothing to disclose
Ashar Asif: Nothing to disclose
Benjamin James Hudson: Nothing to disclose
Jonathan Rodrigues: Other: RUH is a Centre of Excellence for Imbio with use of Imbio without charge

### S 2-2

**Dual-layer spectral detector CT to study the correlation between pericoronary adipose tissue and coronary artery stenosis**

*L. X. Zhu*, Y. Dang, X. Lu, Y. Hou; Shenyang/CN

**Purpose:** To investigate the relationship of pericoronary adipose tissue with coronary artery stenosis in patients with coronary heart disease on dual-layer spectral detector CT（SDCT）
**Methods or Background:** Retrospectively collected 99 patients who used SDCT to perform coronary CTA on suspected coronary heart disease. According to random block design，all patients were divided into three groups according to the degree of coronary artery stenosis: normal (no stenosis), no significant stenosis (the degree of stenosis was < 50%) and significant stenosis (the degree of stenosis was ≥ 50%) with 33 persons in each group. Compare the effect of lesion stenosis on pericoronary adipose tissue（PCAT），quantitatively evaluate the narrowest part of the lesion pericoronary adipose tissue: fat attenuation index (FAI40kev), attenuation index (PCATi40keV = (CTFAI40keV - CTSAT40keV) / CTSAT40keV, SAT: subcutaneous adipose tissue)，spectral curve slope (λHU= (CTFAI40keV - CTFAT70keV) /30), effective atomic number (Eff-Z) and epicardial fat volume (EFV).
**Results or Findings:** Each index of PCAT was related to the degree of coronary artery stenosis. The difference in PCAT index between the normal group and the significant group, the non-significant group and the significant group were statistically significant, while the EFV difference between the normal group and the non-significant group, the normal group and the significant group were statistically significant. Both FAI40kev and λHU were statistically significant in the significant stenosis group (P <0.05).
**Conclusion:** This study showed that the PCAT and EFV indicators based on SDCT were significantly different between the normal and atherosclerotic groups and between the groups with different atherosclerotic degrees. Based on FAI40kev and λHU was a risk factor for significant coronary stenosis. Its association with significant coronary stenosis was better than EFV.
**Limitations:** Single-center small sample research, small sample size
**Ethics Committee Approval:** Not applicable
**Funding for this study:** Not applicable
**Author Disclosures:**
Xiaomei Lu: Nothing to disclose
Yuxue Dang: nothing to disclose
Yang Hou: Nothing to disclose
MA Long Xiao Zhu: Nothing to disclose

### S 2-3

**Diagnostic models for detection of intrauterine growth restriction and placental insufficiency severity, based on magnetic resonance imaging of placenta**

B. Moradi, E. Tabibian, M. A. Kazemi, M. Shirazi, M. Chavoshi, *S. Rashedi*; Tehran/IR

**Purpose:** Placental insufficiency is regarded as the primary cause of intrauterine growth restriction (IUGR). Here we aimed to provide a diagnostic model based on different parameters of placental MRI to detect IUGR, as well as the severity of placental insufficiency.
**Methods or Background:** We included 44 fetuses with appropriate weight for gestational age (AGA) and 46 singleton pregnancies with documented IUGR defined as the estimated fetal weight (EFW) below the 10th percentile. By using Doppler ultrasonography, IUGR cases were divided into two groups: 1. IUGR with severity signs: birth weight<3rd centile, or cerebroplacental centile<5th, or abnormal umbilical/uterine artery pulsatile index; and 2. Non-severe IUGR without any of this criterion. For all of the three groups, MRI was performed (T1, T2, and diffusion-weighted sequences), and its parameters were compared between AGA and IUGR and two IUGR groups. Two diagnostic models consisting of significant predictors were developed, and their performance was investigated with accuracy metrics.
**Results or Findings:** The severity signs were detected in 25 (54.3%) IUGR cases. All demographic characteristics were almost equal and comparable among the groups. The diagnostic model for the differentiation of IUGR from AGA revealed an acceptable performance (area under the receiver operating characteristic curve(AUC)=0.794). Moreover, the logistic regression model for detection of the severity signs was even better with AUC=0.842 and consisted of three main predictors: I. Placental Infarct Percent> 10% (OR=8.26[95% CI: 1.54 - 44.23], p=0.014); II. Placental Shape (plate-like vs. globular) (OR= 5.80[95% CI: 1.35 - 24.75], p=0.018); and III. Placental Area>5400mm2 (OR=3.21 [95% CI: 0.63 - 16.37], p=0.016).
**Conclusion:** MRI parameters can differentiate IUGR from AGA and, more precisely, assess the placental insufficiency severity in IUGR fetuses.
**Limitations:** The major limitation of this study was the limited sample size.
**Ethics Committee Approval:** n/a
**Funding for this study:** None.
**Author Disclosures:**
Behnaz Moradi: Nothing to disclose
Sina Rashedi: Nothing to disclose
Elnaz Tabibian: Nothing to disclose
Mahboobeh Shirazi: Nothing to disclose
Mohammadreza Chavoshi: Nothing to disclose
Mohammad Ali Kazemi: Nothing to disclose

### S 2-4

**Association between tears of the posterior root of the medial meniscus and far posterior femoral condyle osteoarthritis**

*S. S. Ashok*, R. Sebro, M. Uriell; Philadelphia, PA/US

**Purpose:** Evaluate whether tears of the posterior horn of the medial meniscus root ligament (PHMM RL) are associated with osteoarthritis of the far posterior femoral condyles (FPFC).
**Methods or Background:** Retrospective review of 1158 patients who underwent arthroscopy identified 49 patients with PHMM RL attachment tears. Review of preoperative MRI studies identified advanced osteoarthritis involving the medial and lateral FPFC. Controls (n=48) had no meniscal tears confirmed by arthroscopy. Cases and controls were age- and sex-matched 1:1. One case was excluded due to absence of control. The International Cartilage Research Society (ICRS) MRI cartilage grade was recorded for medial and lateral FPFC. Associations were evaluated using univariate and multivariable conditional logistic regression analyses.
**Results or Findings:** There were 10 men (20.8%) in each case and control group with median age 53 years (range, 21–67). Medial FPFC ICRS Grade 2 or higher lesions were present in 34 (70.8%) cases and 16 (33.3%) controls. Lateral FPFC ICRS Grade 2 or higher lesions were present in 24 (50%) cases and 14 (28.2%) controls. Increased BMI was associated with PHMM RL tears (OR=1.11, 95% CI [1.01, 1.22], P = 0.020). MRI was 81.2% (39/48) sensitive and 91.2% (44/48) specific for detection of PHMM RL tears. PHMM RL tears were associated with Grade 2 or higher medial FPFC osteoarthritis (OR=10.00, 95% CI (2.34, 42.78), P < 0.001). This association remained after adjusting for BMI (OR=11.79, 95% CI [2.46, 56.53], P = 0.002). An association between PHMM RL tears and lateral FPFC osteoarthritis persisted after adjusting for BMI (OR =3.00, 95% CI [1.07, 8.37], P = 0.036).
**Conclusion:** PHMM RL tears are associated with advanced osteoarthritis of the FPFC. Radiologists identifying FPFC osteoarthritis should look carefully for PHMM RL tears.
**Limitations:** None.
**Ethics Committee Approval:** IRB approval.
**Funding for this study:** None.
**Author Disclosures:**
BA Samantha Sharon Ashok: Nothing to disclose
Matthew Uriell: Nothing to disclose
Ronnie Sebro: Nothing to disclose

### S 2-5

**Study of knee anatomical variables as risk factors predisposing to anterior cruciate ligament injury using MRI morphometry**

*S. G. Maheshwari*, R. S. Kuber, A. Kharat, R. Arkar; Pune/IN

**Purpose:** The anterior cruciate ligament (ACL) is the most frequently injured ligament of the knee and some people are at greater risk of tearing their ACL than others.
Objectives:
To assess anatomic variables of distal femur and proximal tibia morphology such as medial and lateral condyle width, intercondylar notch width, notch index,tibial platue slopes and depth respectively between the anterior cruciate ligament(ACL)injured and non- injured patient on conventional MRI. Are these variables risk factors for ACL injury?
**Methods or Background:** Case-control study of ACL injured and non-injured patients presented to Department of Radiology at D Y Patil Medical college, Pune. Knee MRIs of 450 patients were studied (225 cases of ACL injured and 225 controls with normal ACL) after matching for age, sex and predisposing factor. Distal femoral and proximal tibia morphology was studied with respect to different parameters on multiplanar MRI. Statistical analysis was done and Odds ratio calculated.
**Results or Findings:** In this group,there was significant bony morphology difference between ACL injured and non-injured patients. Significant difference of bicondylar width (P=0.004) and intercondylar notch width (P=0.001) between male and female patients were noted. Among the male group between injured and non-injured ACL patients there was significant difference notch width index (P=0.001), medial posterior tibial slope (P=0.007) and medial plateau depth (P=0.003) are noted. There is narrower notch width in ACL injured men respect to non-injured. (Odd Ratio=3.02-cases compared OR=5.2-control). There were no significant differences lateral posterior tibial slopes.

**Conclusion:** From this study we concluded that ACL injured men have narrower notch width index and shallow medial tibia depth and steeper MPTS which appear important risk factors. Identifying these risk factors helps in stratification non-intervention and intervention strategies of ACL injuries.
**Limitations:** Previous Surgery, Septic-Hemophilic and Degenerative arthritis altering knee anatomy.
**Ethics Committee Approval:** Yes
**Funding for this study:** None
**Author Disclosures:**
Rajesh S Kuber: Nothing to disclose
Amit Kharat: Nothing to disclose
Sagar Ganeshprasad Maheshwari: Nothing to disclose
Rahul Arkar: Nothing to disclose

### S 2-6

**Association between enlarged perivascular spaces and cerebral blood flow, brain volume, and cardiovascular disease risk in elderly**
*S. Liu*, B. Hou, H. You, Y. Zhu, C. Ma, Z. Zuo, F. Feng; Beijing/CN

**Purpose:** To evaluate the association between enlarged perivascular spaces in basal ganglia (BG-EPVS) and gray matter volume (GMV), GM cerebral blood flow (GM-CBF), and cardiovascular disease (CVD) risk in cognitively intact elderly individuals.
**Methods or Background:** 285 participants were recruited with the criteria of age≥55-year-old, MMSE score≥27, right handedness, and free of brain disorders. MRI was performed at 3T using pseudo-continuous arterial spin labeling and sagittal T1-weighted sequence for GM-CBF and GMV analysis. Images from a subset of the participants (set A; n=254) were used to investigate the relationship between the severity of BG-EPVS and GMV/GM-CBF, while images from another subset (set B; n=254) were used to reveal the relationship between the severity of BG-EPVS and the CVD risk. The BG-EPVS was identified according to standards for Reporting Vascular changes on neuroimaging. Ten-year CVD risk for individuals was determined from Framingham risk score. Based on CVD, participants were divided into a high-risk group (>20% risk) and a low-risk group (≤20% risk) for statistical analysis.
**Results or Findings:** In subset A, severe BG-EPVS was associated with significantly decreased GMV and GM-CBF in multiple cortical regions, such as the frontal and temporal gyrus (P<0.05), but increased CBF in the bilateral caudate nucleus and putamen (P<0.05). In subset B, participants with severe BG-EPVS had higher CVD risk than participants with mild BG-EPVS (66.1% vs. 46.0%; P=0.009).
**Conclusion:** In cognitively intact elderly individuals, severe BG-EPVS was associated with increased CVD risk and widespread changes in GMV and GM-CBF. Early identification of BG-EPVS could be useful for better understanding the pathophysiology of EPVS-related diseases.
**Limitations:** This cross-sectional analysis did not make allowances for causal inferences.
**Ethics Committee Approval:** Peking Union Medical College Hospital
**Funding for this study:** Chinese Academy of Medical Sciences Innovation Fund for Medical Sciences
**Author Disclosures:**
Feng Feng: Nothing to disclose
Sirui Liu: Nothing to disclose
Hui You: Nothing to disclose
Chao Ma: Nothing to disclose
Yicheng Zhu: nothing to disclose
Zhentao Zuo: Nothing to disclose
Bo Hou: Nothing to disclose

### S 2-7

**Alterations in selected regions of brain white matter with the biomarker anisotropy fraction derived from the diffusion tensor in patiens with clinical diagnosis of post-traumatic stress disorder**
*K. E. Abundiz Bibiano*, C. V. Baena; Mexico City/MX

**Purpose:** To identify alterations in selected brain white matter regions with the biomarker anisotropy fraction derived from the diffusion tensor in military personnel with a clinical diagnosis of post-traumatic stress disorder (PTSD).
**Methods or Background:** Cross-sectional, as well as cases and controls. Military personnel who were exposed to high impact events with clinical diagnostic criteria for PTSD and healthy controls not belonging to the armed forces. Total sample of 35 patients with a clinical diagnosis of PTSD and 20 healthy patients, non-probabilistic sample for convenience from January to December 2018 with simple magnetic resonance imaging of the skull with acquisition of conventional sequences and the advanced diffusion tensor sequence and quantify the fraction of anisotropy.
**Results or Findings:** In a group of 20 healthy controls, the measurements of the anisotropic fraction biomarker derived from the diffusion tensor had a normal distribution according to the Kolmogorov-Smirnov statistic, the controls with positive alcoholism differed from those with negative alcoholism at the T. Long level. Right upper [0.54 +/- 0.01 versus 0.53 +/- 0.009, p = 0.03]. In the group of cases, there was no difference in the means of the biomarker in any region according to the severity of stress as chronic or acute. With the exception of the left prefrontal cortex, in all other regions the means of the biomarker differ significantly between cases and controls, with greater accentuation at the level of the hippocampus and amygdala.
**Conclusion:** We can conclude that there is a decrease in the anisotropy factor in patients with post-traumatic stress disorder compared to healthy patients, markedly at the level of the amygdala and hippocampus.
**Limitations:** No ONE
**Ethics Committee Approval:** Approved.
**Funding for this study:** NO oNE
**Author Disclosures:**
Karla Estela Abundiz Bibiano: Nothing to disclose
Claudia Viviana Baena: Nothing to disclose

### S 2-8

**Analysis of compression real-time strain elastography efficacy in differentiation of functional and inflammatory colonic diseases in children**
*E. A. Paleonaya*[1], N. J. Nelassov[2], M. Morgunov[2], T. Korkoshka[2], A. Palenui[2], A. Zubov[1], O. Eroshenko[2], A. Nechaeva[2]; [1]Donetsk/UA, [2]Rostov/RU

**Purpose:** To assess if transabdominal ultrasound-based compression real-time strain elastography in children help to differentiate functional/inflammatory colonic pathology in acute stage.
**Methods or Background:** 79 children (mean age 9.8+1.4 years) were examined. There were 20 healthy children (1-st subgroup) without any signs of intestinal disorders and values of fecal calprotectin (FC) <50 µg/g and 59 children with clinical presentation of colonic pathology (25 with normal values of FC (<50 µg/g; 2-d subgroup), 24 with borderline levels of FC (50–120 µg/g; 3-d subgroup), 9 with elevated values of FC (>120 µg/g; 4-th subgroup) and with verified by colonoscopy diagnosis of IBD). RTE measurements were performed during exam of ascending, transverse and descending colon at the sites of detection of mostly affected colonic segments. The outcome of RTE was classified according to a colorimetric categorical classification method proposed by Giannetti A. et al (2013).
**Results or Findings:** In all healthy children RTE revealed category 1B of wall stiffness (normal colonic wall stiffness). 84.0% of children in subgroup 2 had normal intestinal wall stiffness (1B), indicating the presence of functional pathology. 83.3% of children in 3-d subgroup had 2BA category of wall stiffness, which is characteristic for either functional pathology or remission stage of IBD. In patients of 4-th subgroup in 88.9% of cases was found 1A category of wall stiffness (stiff colonic wall). While using FC as comparator sensitivity and specificity of RTE in separation of children with IBD from others in acute stage of disease appeared to be 88.9% and 98.0% (p=.0001).
**Conclusion:** RTE can effectively differentiate functional and inflammatory colonic pathology in children in acute stage of disease.
**Limitations:** During the study, the limitations were not violated.
**Ethics Committee Approval:** The ethics Committee approved the study.
**Funding for this study:** The research was not funded.
**Author Disclosures:**
A. Zubov: Investigator: the research process
Palenui A. Andrew Palenui: Investigator: the research process
Nikolai J Nelassov: Investigator: the research process
Maxim Morgunov: Investigator: the research process
O. Eroshenko: Investigator: the research process
Tatiana Korkoshka: Investigator: the research process
Elisaveta Andrevna Paleonaya: Author: The Research
A. Nechaeva: Investigator: the research process

### E³ - Rising Stars Programme: Student Session

### S 3

### Student Session 3

### S 3-1

**The breast is yet to come: review of symptomatic recall to screening. Our experience at the South East Scotland Breast Screening Centre**
*N. Tai*, G. P. Babu, E. G. Breaden, B. Coiley, H. Dissanayake, B. Ingabire, Y. Papadopoulos, L. Street; Edinburgh/UK

**Purpose:** In Scotland, breast screening is offered to women (50-70 year-old) triennially. Women are recalled for mammographic abnormalities and symptoms. This audit project aimed to review the effectiveness of the current approach to symptomatic recall. A secondary objective was to establish an effective GP referral pathway for women who report symptoms at screening.
**Methods or Background:** Review of a database of 47,340 screenings between November 2017 and October 2018 identified 2,132 recall cases; 166 were symptomatic. Data analysis was performed to categorise symptoms into lumps, discharge, dimpling and non-specific causes. Excel was used to statistically analyse percentage of cases in each symptom category. Information on GP referrals was obtained from patient records.
**Results or Findings:** Symptomatic recall rate was 7.8% (166 symptomatic recalls out of 2132 recall cases). 51% of symptomatic women reported lumps, 6% discharge, 25% dimpling and 18% non-specific. Positive predictive value for cancers was low at 2.4% (only 4 out of 166 symptomatic women) with all lesions visible mammographically. Only 49% of symptomatic patients were assigned to the appropriate patient pathway ie. referral to GP.
**Conclusion:** Outcome of study showed that no cancers detected from symptomatic recalls were occult mammographically. We established that the current approach to symptomatic recall is not cost-effective. Additionally, women face emotional stress when recalled for screening assessments. A standardised approach to symptomatic recall was lacking across various screening centres and this evidence has helped us develop one. This also enabled education and training of mammographers to assign women to appropriate GP referral pathways. The project outcomes have been accepted nationally and is being incorporated into practice.
**Limitations:** There was an exclusion of a small number of cases due to technical reasons but this was not statistically significant.
**Ethics Committee Approval:** Not applicable
**Funding for this study:** Not applicable
**Author Disclosures:**
Beth Coiley: Nothing to disclose
Lara Street: Nothing to disclose
Ellen Grace Breaden: Nothing to disclose
Yasmina Papadopoulos: Nothing to disclose
Hathsu Dissanayake: Nothing to disclose
Benissa Ingabire: Nothing to disclose
Gauri Priya Babu: Nothing to disclose
Natalie Tai: Nothing to disclose

### S 3-2

**How branching scenario tool can transform and improve radiology education? A cross-sectional study**
*M. E. B. De Siervi*, M. E. R. d. A. D. d. Pinho, G. F. Saffe, C. V. Souza Freire, C. Freitas Lins; Salvador/BR

**Purpose:** To evaluate the application of "Branching Scenario" as an interactive tool for teaching radiology for medical students.
**Methods or Background:** "Branching scenario" was developed by student mentors of radiology guided by a radiologist through H5P plug-in and it was made available weekly by Moodle platform to medical students. This tool is based on a clinical case followed by multiple-choice questions about the probable diagnosis and the imaging tests to be requested. The chosen answer determines the following question that will be seen in order to dialogue with the clinical-radiological reasoning developed by the student. After that, feedback is received about the adequacy of answers and cost-conscious handling for the case. After one semester, students were invited to answer an evaluation questionnaire. Written informed consent was obtained from all subjects.
**Results or Findings:** 147 students (21±3 years, 59% female) participated in the study. From that sample, 89% considered the digital platform's clarity to approach radiology above the average or excellent. 87% expressed to have facility to use the platform. 80% considered Branching Scenario as a helpful or very helpful tool in order to take correct conduct with their patients in their future medical practice. 89% considered that the digital interactive platform contributed to the realization of academic tests. 91% considered that association between clinical cases and radiology enabled greater learning.
**Conclusion:** "Branching Scenario" tool allows the creation of clear and interactive radiological contents that can increase the learning potential of students.
**Limitations:** This is a descriptive study, further investigation would be required to measure the impact of the tool.
**Ethics Committee Approval:** Ethical approval was obtained from the Ethics Committee of the University where this study was developed (CAAE: 28196820.6.0000.5544).
**Funding for this study:** Not applicable.
**Author Disclosures:**
Maria Eduarda Rodrigues de Araújo Dantas de Pinho: Nothing to disclose
Gabriela Fagundes Saffe: Nothing to disclose
Maria Eduarda Barreto De Siervi: Nothing to disclose
Carolina Freitas Lins: Nothing to disclose
Camila Verônica Souza Freire: Nothing to disclose

### S 3-3

**Photoacoustic imaging in evaluating early intestinal ischemia and reperfusion injury with rat models**
*R. Wang*; Yunnan/CN

**Purpose:** It is still a challenge to distinguish whether the damaged intestine is viable or not in the treatment of acute mesenteric ischemia. In this study, photoacoustic imaging (PAI) was used to observe the activity of intestinal tissue after ischemia and reperfusion injury in rats.
**Methods or Background:** Forty male SD rats were randomly divided into four groups: sham operated (SO) group, 1 h ischemia group, 2 h ischemia group and ischemia-reperfusion (I/R) group. In the ischemia group, superior mesenteric artery was isolated and clamped for 1 h and 2 h, respectively. In I/R group, after ischemia for 1 hour, the clamp was removed and reperfused for 1 hour. Immediately after the establishment of the animal model, PAI examination was performed. After PAI examination, the small intestine was collected for histopathology.
**Results or Findings:** The PAI derived parameters of 2 h ischemia group were significantly different from those of SO group and I/R group ($P<0.05$). The levels of Hb, HbR, map760 and map 840 were increased in different degrees in ischemia group, especially in 2 h ischemia group (compared with SO group, $P<0.01$). In immunohistochemistry, compared with SO group, BAX increased significantly in 2 h ischemia group ($P<0.01$). TUNEL staining showed that the number of apoptotic positive nuclei in 2 h ischemia group was significantly higher than that in SO group ($P<0.05$).
**Conclusion:** PAI can be used as an effective tool to detect acute intestinal ischemia injury and quantitatively evaluate tissue viability.
**Limitations:** The main limitation of our study is that the intestinal I/R model with mesenteric artery ligation can not fully simulate simulate clinical intestinal ischemia.
**Ethics Committee Approval:** Approved by Animal Ethics Committee of Kunming Medical University.
**Funding for this study:** Supported by National Natural Science Foundation of China (NSFC Grants 82060313 and 81760316).
**Author Disclosures:**
Rui Wang: Nothing to disclose

### S 3-4

**Percutaneous microwave ablation (MWA) of small-sized hepatocellular carcinoma (HCC) under CT-guidance**
*H. Adwan*, T. J. Vogl; Frankfurt am Main/DE

**Purpose:** To evaluate the safety and efficacy of CT-guided percutaneous microwave ablation (MWA) as a potentially curative treatment for small-sized (≤2 cm) Hepatocellular carcinoma (HCC) according to therapy response, survival and complication rates.
**Methods or Background:** In our study, we retrospectively included 87 patients (16W and 71M, mean age: 63.8±10.8yrs) with 130 small HCC lesions, which were treated by CT-guided MWA. All cases were evaluated according to diameter, post-ablation volume, technical success, complete ablation rate, therapy response, overall survival (OS) rates, progression-free survival (PFS) rates, complication rate. For evaluation of response to MWA treatment MRI was performed post-ablation.
**Results or Findings:** The mean axial diameter of the tumor and post- ablation volume were 1.4 cm and 32.6 mL, respectively. We have achieved 100% technical success in all abations. Complete ablation was achieved in 97.7% (127/130) of the tumors. The median follow-up time was 2.75 years. In this period, 4.6% (4/87) of the patients developed local tumor progression (LTP) and 36.8% (32/87) of the patients developed intrahepatic distant recurrence (IDR). The 1-, 3-, and 5-year OS rates were 94.3%, 66.4% and 53.8%, respectively. The 1-, 3-, and 5-year PFS rates were 70.5%, 46.4% and 33%, respectively. The rate of complications was 4.6% (4/87). There were no peri-procedural deaths reported.
**Conclusion:** CT-guided MWA seems to be an effective and safe treatment for small-sized HCC. Patients with small-sized HCC, that underwent percutaneous MWA had a long OS time as well as low rates of LTP and complications.
**Limitations:** Retrospective study.
**Ethics Committee Approval:** Our study was approved.
**Funding for this study:** No funding.
**Author Disclosures:**
Thomas J. Vogl: Nothing to disclose
Hamzah Adwan: Nothing to disclose

### S 3-5

**Common and distinct grey matter alterations in adolescent major depressive and bipolar disorders**
*X. Long*; Chengdu/CN

**Purpose:** Adolescence (10–19 years) is a crucial period for developing affective disorders such as major depressive disorder (MDD) and bipolar disorder (BD). Although dysfunction of some emotion-related brain areas is implicated in these two diseases, the structural alterations in adolescent patients are inconsistent, and the neural correlate similarities and differences between adolescent MDD and adolescent BD patients remain unclear.
**Methods or Background:** Meta-analyses of whole-brain voxel-based morphometry findings were conducted in adolescent MDD and adolescent BD patients separately, followed by a quantitative meta-analytic comparison of the two groups.
**Results or Findings:** Sixteen studies of a total of 263 adolescent MDD and 263 adolescent BD patients were included in the current meta-analysis. Conjunction analysis showed greater volumes in the right caudate nucleus in both groups of adolescent patients. On the other hand, adolescents with MDD had greater middle frontal gyrus, orbital frontal gyrus, amygdala, and striatum volumes. Smaller volumes of the insula and superior frontal gyrus were only observed in adolescents with BD compared to healthy adolescent controls.
**Conclusion:** Our findings confirmed that an increase of the grey matter volume in the right caudate nucleus may be a common pattern underlying adolescent MDD and BD, while disease-specific grey matter alterations in the fronto-limbic circuitry may reflect their different neural bases. This study unveiled important differences and conjunctions between adolescent MDD and BD and specific biomarkers of the two groups.
**Limitations:** We did not perform meta-regression analyses of treatment status, mood states, and illness duration.
**Ethics Committee Approval:** NA, not applicable
**Funding for this study:** This study was supported by the National Natural Science Foundation of China (Grant Nos. 81971595 and 81771812), the Innovation Spark Project of Sichuan University (No. 2019SCUH0003) and 1·3·5 project for disciplines of excellence–Clinical Research Incubation Project, West China Hospital, Sichuan University (No. 2020HXFH005).
**Author Disclosures:**
Xipeng Long: Nothing to disclose

### S 3-6

### Clinical reasoning and radiology: untapped potential for medical students

*J. C. Lui*, C. Chew; Glasgow/UK

**Purpose:** Neurological cases used to create an interactive online resource with Storyline© to enhance the Neurology teaching. Students choose required information within history, examination, management and investigation. Each decision in the diagnostic process is evaluated, illustrating how differential diagnosis changes. Immediate feedback is provided with each choice. The key investigation, CTBrain, can be reviewed repeatedly. Each case concludes with management, a summary slide and 3 reflective questions.
**Methods or Background:** Modern clinical medicine is increasingly reliant on Radiology. It is important we teach our students the role it plays in the diagnostic process. The combination of Clinical Stories with Radiology is a potent way to teach Clinical Reasoning. We present our initial experience using Storyline© to interactively guide the student to work through a patient's presentation, clinical findings, investigations, imaging (where appropriate!) and results to arrive at a diagnosis.
**Results or Findings:** This learning resource has been extremely well received by students. Feedback comments include: "Good thinking about how the history/presentation can change when different or new factors were added into consideration"; "Good systematic approach to learning"; "Relevant to real cases "; "Easy to know where to improve"
**Conclusion:** Clinical Reasoning has been linked with Patient Safety and is now mandated by the GMC to be included explicitly in the medical school curriculum. While it is ubiquitous to how doctors perform their job and arrive at the diagnosis, there is as yet no consensus on exactly how best to teach this important subject. Radiology, together with computing software produced an online, interactive learning resource to teach Clinical Reasoning has proved extremely popular with the students. The student co- author found the process very fulfilling. The student input ensured the material is relevant and student-centric.
**Limitations:** None
**Ethics Committee Approval:** Not required
**Funding for this study:** None
**Author Disclosures:**
Jessica Claresta Lui: Nothing to disclose
Cindy Chew: Nothing to disclose

### S 3-7

### Selecting optimal kernels for QCT using phantom simulation results

*Z. Artyukova*, A. Petraikin, K. Akhmad, D. Semenov, A. Smorchkova, N. Kudryavtsev, F. A. Petriaikin, S. Morozov; Moscow/RU

**Purpose:** Compare kernels (CT convolution algorithms) with varying degrees of beam hardening correction for QCT study in phantom modeling and in patients.
**Methods or Background:** The studies were performed on a 64-row scanner. We use kernels for a body with (FC08) and without (FC17) beam hardening correction. The semi-anthropomorphic phantom with a wide range of $K_2HPO_4$ (50-550 mg/ml) was used. Asynchronous QCT was used to determine the bone mineral density (BMD). Additionally, we analyzed QCT data of five patients who had normal BMD of «femoral neck» and «total hip» regions.
**Results or Findings:** When we compared a BMD dependency measured by QCT vs preset value for the kernels FC08 and FC17 in phantom study, we did not discover any statistically significant difference in slope angle of inclination and intercept of the regression lines ($p>0.05$) for the range of 50-200 mg/ml. When scanning was performed with the FC17 kernel for the range of 250-550 mg/ml, the difference in slope of the regression lines was significant ($p <0.05$) and there was no difference when comparing the intercept ($p = 0.10$). Based on the data from the phantom study, the results of scanning five patients (right and left hip, n=10) were simulated and compared with the initial data. The use of the FC08 kernel led to the insignificant mean underestimation of BMD, assessed by the T-score (-0.24±0.02 SD), while the use of the FC17 kernel led to the significantly more pronounced ($p <0.05$) decrease in BMD (-0.94±0.07 SD).
**Conclusion:** For asynchronous QCT body filters with beam hardening correction kernels should be chosen (FC08). Kernels without beam hardening correction (FC17) can lead to the significant underestimation of BMD results.
**Limitations:** No limit
**Ethics Committee Approval:** Not subject
**Funding for this study:** The reported study was funded by RFBR №20-015-00260.
**Author Disclosures:**
Sergey Morozov: Nothing to disclose
Kate Akhmad: Nothing to disclose
Zlata Artyukova: Nothing to disclose
Nikita Kudryavtsev: Nothing to disclose
Alexey Petraikin: Nothing to disclose
Dmitry Semenov: Nothing to disclose
Anastasiia Smorchkova: Nothing to disclose
Fedor Alekseevich Petriaikin: Nothing to disclose

### S 3-8

### A retrospective observational study on spectrum of inferior vena cava and renal vein anomalies: embryologic and surgical considerations

*V. Varadharaj*, R. Ramachandran, K. Athiyappan, V. Sai; Chennai/IN

**Purpose:** To demonstrate various congenital anomalies of inferior vena cava and its tributaries with embryological correlation and surgical consideration, which aided in further course of management.
**Methods or Background:** CT-Philips brilliance 16 and GE revolution EVO-128. Single Centre based Retrospective imaging analysis of congenital anomalies of IVC and its tributaries in all age groups for a period of five years from 2016-2020. Congenital anomalies of the inferior vena cava (IVC) and its tributaries have become more commonly recognized in asymptomatic patients. The embryogenesis of IVC is a complex process involving the formation of several anastomosis between three paired embryonic veins. The result is the numerous variations in the basic venous network of the abdomen and pelvis. Although most of the IVC anomalies are clinically silent and are usually discovered incidentally on abdominal imaging, aberrations may be responsible for significant hemorrhage or damage to vascular structures during surgeries. Aberrant vessels may be mistaken for lymphadenopathy and may be biopsied. Hence, accurate characterization of systemic venous anomalies plays a vital role in the appropriate selection of surgical approach or interventional procedure.
**Results or Findings:** The overall incidence of congenital anomalies of IVC and its tributaries were found to be 0.4% with incidence of each subtype as follows: 1. Interruption of IVC - 5.2% , 2. Retro aortic left renal vein-33.3% 3.Duplication of IVC- 1.3% , 4. Circumaortic left renal vein- 1.7% , 5.Left IVC-5.2% , 6.Retrocaval ureter- 33%, 7.Absent infra renal segment of the IVC-0.0%
**Conclusion:** IVC anomalies are commonly encountered due to increased use of cross sectional imaging modalities. A working knowledge of IVC and renal vein anomalies is essential in designing an effective treatment plan from interventional standpoint and to avoid diagnostic pitfalls.
**Limitations:** n/a
**Ethics Committee Approval:** n/a
**Funding for this study:** n/a
**Author Disclosures:**
Kumaresh Athiyappan: Author: Guidance
Venkata Sai: Author: Guidance
Rajoo Ramachandran: Author: Guidance
Vinu Varadharaj: Author: Principal investigator

### E³ - Rising Stars Programme: Student Session

### S 4

### Student Session 4

### S 4-1

**A nomogram strategy for identifying the subclassification of IDH1 mutation and ATRX expression loss in in low-grade gliomas**

*S. Wu*[1], X. Zhang[2], Z. Yao[1], Y. Zhang[1], T. Qiu[1], Y. Ren[1]; [1]Shanghai/CN, [2]Xi'an/CN

**Purpose:** To construct a radiomics nomogram based on multiparametric MR for noninvasively determining molecular status of isocitrate dehydrogenase 1 mutation (IDH1(+)) and loss of nuclear alpha thalassemia/mental retardation syndrome X-linked expression (ATRX(–)) in patients with low-grade gliomas (LGGs).
**Methods or Background:** A total of 111 eligible LGG patients LGG patients with IDH1(+) (n = 76) and IDH1(–) (n = 35), divided into a training set (n = 78) and a validation set (n = 35) for IDH (+) prediction task. LGG patients with IDH1(+) were further subclassified into two subtypes of ATRX(–) (n = 38) and ATRX(+) (n = 38) for the ATRX (-) prediction task. 250 radiomic features were extracted on each tumor volume of interest from T2 fluid-attenuated inversion recovery (T2 FLAIR), contrast-enhanced T1 weighted imaging, arterial spin labeling (3D-ASL)-derived cerebral blood flow (CBF), DWI-derived apparent diffusion coefficient (ADC), and exponential ADC (eADC). Then, a radiomics signature was selected using Elastic Net regression model. Combined with clinical risk factors, a radiomics nomogram was constructed based on a multivariate logistic regression model.
**Results or Findings:** The presented radiomics nomogram exhibited promising discrimination for IDH mutation (C-index: training sets= 0.881, validation sets=0.9) and ATRX expression loss (C-index: training sets= 0.863, validation sets=0.840). The favorable calibration and decision curve analysis indicated its clinical usefulness.
**Conclusion:** The presented nomogram combining multiparametric radiomics features and with clinical risk factor are favorable in identifying molecular subtypes of IDH1 and ATRX in LGGs.
**Limitations:** Relatively small population.
**Ethics Committee Approval:** Approved by the Ethics Review Board of our hospital.
**Funding for this study:** Science and Technology Support Project for Medicine sponsored by Science and Technology Commission of Shanghai Municipality; Contract grant number: 18411967300.
**Author Disclosures:**
Zhenwei Yao: Nothing to disclose
Yan Ren: Nothing to disclose
Xi Zhang: Nothing to disclose
Yong Zhang: Nothing to disclose
Shiman Wu: Nothing to disclose
Tianming Qiu: Nothing to disclose

### S 4-2

**Advantage of 3D over 2D dense neural network architecture for the MRI-based classification of focal liver lesions**

*R. Stollmayer*, B. K. Budai, M. Boostani, Z. Tánczik, B. Körmendy, E. Hartmann, I. Kalina, V. Bérczi, P. N. Kaposi-Novák; Budapest/HU

**Purpose:** To compare the accuracy of 2D dense convolutional networks (2D-DenseNets) vs. 3D-DenseNets trained on multi-sequence MRI-scans for the classification of focal liver lesions.
**Methods or Background:** In this study, we retrospectively collected T2W, gadoxetate disodium-enhanced portal venous phase and hepatobiliary phase MRI scans from patients with focal nodular hyperplasias (FNH), hepatocellular carcinomas (HCC) or metastases (MET). The final dataset consisted of n=33 FNHs, n=42 HCCs and n=35 METs. Three axial slices of each tumor were selected and the corresponding sequences of each slice were combined into three-channel JPG files and used as input for a 2D-DenseNet264 network. To train a 3D-DenseNet264 model, the scans of each tumor were stacked 4D Nifti file inputs. The trained models were tested on 10 tumors from each class. Prediction accuracies of the models were compared using receiver operating characteristic curve (AUROC), specificity, sensitivity, positive predictive values (PPV), negative predictive values (NPV), and f1 scores for each class.
**Results or Findings:** Average AUROC values of 0.79 and 0.84 were achieved using the 2D vs. the 3D model. Mean PPV, sensitivity, NPV, specificity and f1 scores were 0.73, 0.73, 0.87, 0.87, 0.73 for the 2D model and 0.88, 0.87, 0.94, 0.93, 0.86 for the 3D model.
**Conclusion:** Our results indicate that 3D-DenseNets are superior to 2D-DenseNets for the multi-sequence MRI-based classification of focal liver lesions.
**Limitations:** The current study is limited by the number of cases used for model training and validation.
**Ethics Committee Approval:** The study has been approved by the institutional ethics committee of our university (SE-RKEB 136/2019).
**Funding for this study:** The authors received no funding for this study.
**Author Disclosures:**
Pál N. Kaposi-Novák: Nothing to disclose
Erika Hartmann: Nothing to disclose
Ildikó Kalina: Nothing to disclose
Róbert Stollmayer: Nothing to disclose
Viktor Bérczi: Nothing to disclose
Zsófia Tánczik: Nothing to disclose
Mehdi Boostani: Nothing to disclose
Bettina Katalin Budai: Nothing to disclose
Borbála Körmendy: Nothing to disclose

### S 4-3

**Deep convolutional neural network for predicting kidney tumour malignancy**

*A. Obuchowski*, B. O. Klaudel, R. Karski, B. Rydziński, M. Glembin, P. Jasik, P. Syty; Gdańsk/PL

**Purpose:** According to the statistics, up to 15-20% of removed solid kidney tumors turn out to be benign in postoperative histopathological examination, despite having been identified as malignant by a radiologist. The aim of the research was to limit the number of unnecessary nephrectomies of benign tumors.
**Methods or Background:** We propose a machine-aided diagnostic system for kidney tumor malignancy identification that can serve as a second opinion source to prevent potentially unnecessary surgical procedures. CT images of a cross-section of a tumor were used to identify tumor malignancy. The system utilizes a deep convolutional neural network as a feature extractor. The network was trained on approximately 15,000 CT images of arterial phase coming from 349 solid kidney tumors. We introduced medical image colorization to improve the knowledge transfer, as the network was pretrained on natural RGB images. We used subtype max-pooling to map predicted histopathological subtype to malignant/benign classification, to improve the network's accuracy over binary classification.
**Results or Findings:** The algorithm achieved F1-score of 90% and accuracy of 87% for the identification of benign and malignant tumors. Achieved specificity of 100% was essential to the study, as the goal of the research was to help reduce the number of tumors misdiagnosed as malignant. We discovered that image colorization can improve accuracy up to 10 pp.
**Conclusion:** We present a machine-aided diagnostic system to classify malignancy of renal tumors. Our method has proved to be a valuable source of second opinion assisting in the decision whether or not nephrectomy should be performed.
**Limitations:** We are planning to explore how the colorization affects images coming from other domains.
**Ethics Committee Approval:** Ethics committee approval was not required.
**Funding for this study:** "e-Pionier - wykorzystanie potencjału uczelni wyższych na rzecz podniesienia innowacyjności rozwiązań ICT w sektorze publicznym"
**Author Disclosures:**
Bartosz Rydziński: Nothing to disclose
Paweł Syty: Nothing to disclose
Barbara Olga Klaudel: Nothing to disclose
Patryk Jasik: Nothing to disclose
Mateusz Glembin: Nothing to disclose
Aleksander Obuchowski: Nothing to disclose
Roman Karski: Nothing to disclose

### S 4-4

**Teaching ultrasound-guided peripheral venous catheter placement through immersive virtual reality: an explorative pilot study**

*N. L. Andersen*, R. Overgaard Jensen, S. Posth, C. Laursen, R. Jørgensen, O. Graumann; Odense/DK

**Purpose:** Immersive virtual reality (IVR)-based training is gaining ground as an educational tool in healthcare. Combining IVR with well-established educational methods could potentially increase competency and autonomy in ultrasound (US)-guided peripheral venous cannulation. The aim of this study was to examine the impact of adding IVR training to a course in US-guided peripheral venous cannulation.
**Methods or Background:** Medical students from University of Southern Denmark without former standardized US education were recruited voluntarily to participate in a pilot study, designed as a randomized controlled trial.

Primary outcome was the proportion of successful peripheral venous cannulations in a phantom. Secondary outcomes included the proportion of surface punctures on the phantom and procedure time. Participants (n=19) received e-learning on basic US before randomization to either IVR (n=10) or no further training (n=9). The additional IVR training consisted of 10 virtual scenarios on US-guided peripheral venous catheter (PVC) placement. Participants were subsequently assessed in peripheral venous cannulation by a blinded supervisor.
**Results or Findings:** The proportion of successful peripheral venous cannulations in the IVR group was 100%, 80%, and 40% for the 1st, 2nd, and 3rd PVC, respectively. In total, 73% of their cannulations were successful. In the control group, the proportion of successful cannulations was 44%, 22%, and 0% for the 1st, 2nd, and 3rd PVC, respectively. In total, 22% of their cannulations were successful. The proportion of successful cannulations was significantly higher in the IVR group compared to the control group for the 1st and 2nd PVC and in total.
**Conclusion:** This pilot study showed that the proportion of successful peripheral venous cannulations significantly increased after e-learning and IVR training on US-guided PVC placement on a phantom compared to e-learning on its own.
**Limitations:** Not applicable
**Ethics Committee Approval:** Not applicable
**Funding for this study:** Not applicable
**Author Disclosures:**
Ole Graumann: Nothing to disclose
Rasmus Jørgensen: Nothing to disclose
Stefan Posth: Nothing to disclose
Nanna Lind Andersen: Nothing to disclose
Rune Overgaard Jensen: Other: CEO and co-founder of VitaSim, the company providing materials and software for the study.
Christian Laursen: Nothing to disclose

### S 4-5

**The status of diversity in canadian radiology: where we stand and what can we do about it**

*K. Lebel*[1], E. Hillier[2], L. B. Spalluto[3], W. W. Yap[4], K. Keglowitsch[2], K. Darras[5], C. J. Yong-Hing[5]; [1]Sherbrooke, QC/CA, [2]Edmonton, AB/CA, [3]Nashville, TX/US, [4]Abbotsford, BC/CA, [5]Vancouver, BC/CA

**Purpose:** 1) Review the current data on gender, race and indigenous representation in the Canadian field of radiology 2) Review proposed solutions and strategies in the current litterature to increase diversity in the field of radiology
**Methods or Background:** Radiology has been identified as one of the medical fields with the least gender, racial, and ethnic diversity. Despite the demonstrated benefits of gender and race diversity in medicine, including innovation, empathy and improved patient outcomes, diversity in radiology in Canada is still lacking. Many solutions for national associations and radiology departments to improve diversity (gender, racial and indigenous) have been proposed. We review the current literature and available data on diversity within radiology in Canada, including the status of gender, race/ethnicity, and Indigenous people. We also present potential solutions to increase diversity.
**Results or Findings:** In 2019, women represented around 63% of current medical canadian graduates. However, within Canadian radiology practices, only 31.6% of radiologists are women. Women are also underrepresented in academic positions and the widening gender gap is present at higher academic ranks, indicating that women may not advance through academic hierarchies at the same pace as men. Although data on racial diversity in Canadian radiology practices is currently lacking, the representation of visible minorities in the general Canadian population is not reflected across Canadian radiology practices. Similarly, despite the Canadian Truth and Reconciliation Commission calling for action to increase the number of Indigenous healthcare workers, Indigenous people remain underrepresented in medicine and radiology.
**Conclusion:** The importance of increasing diversity in radiology has gained recognition in recent years. Leadership commitment is required to implement these programs to increase diversity in radiology in Canada with the ultimate goal of improving patient care.
**Limitations:** None
**Ethics Committee Approval:** Not necessary
**Funding for this study:** None
**Author Disclosures:**
Kiera Keglowitsch: Nothing to disclose
Lucy B Spalluto: Nothing to disclose
Medical Elizabeth Hillier: Nothing to disclose
Kiana Lebel: Nothing to disclose
Charlotte J. Yong-Hing: Nothing to disclose
Wan Wan Yap: Nothing to disclose
Kathryn Darras: Nothing to disclose

### S 4-6

**Evaluation of dysphagia in patients with head and neck cancer after treatment by real-time magnetic resonance imaging based on compressed sensing**

*G. Xiaobin*; Kunming/CN

**Purpose:** To investigate the use of real-time magnetic resonance imaging (RT-MRI) based on compressed sensing(CS) in the evaluation of swallowing function in patients with head and neck cancer after treatment.
**Methods or Background:** Fifty patients with dysphagia after the treatment of head and neck cancer were examined by RT-MRI and video fluoroscopic swallowing studies (VFSS) combined with Swallowing-Related Quality of Life Questionnaire (SWAL-QoL) and flexible endoscopic evaluation of swallowing (FEES), the location and changes of dysphagia were quantitatively analyzed.
**Results or Findings:** RT-MRI equally revealed dysphagia compared to VF and FEES and correlated well with the SWAL-QoL. Only RT-MRI allowed precise time measurements and identification of the respective tissue morphology. The time of swallowing, oral delivery time and hyoid bone forward displacement measured by RT-MRI were different from the reference values.
**Conclusion:** RT-MRI is safe and equally capable as VFSS to identify the cause of dysphagia in patients with head and neck cancer after treatment.Advantages of RT-MRI include visualization of soft tissue, more reliable timing analysis, and lack of X-ray exposure. To provide a new evaluation index and theoretical basis for human swallowing function, and contribute to improve dysphagia caused by head and neck tumors and treatment.
**Limitations:** There is no more in-depth analysis of the causes of dysphagia combined with pathology.
**Ethics Committee Approval:** The ethical committee of Yunnan Cancer Hospital reviewed and approved the research plan.
**Funding for this study:** Supported by The National Natural Science Foundation of China（NSFC） (Project No.: 81760316)
**Author Disclosures:**
Guo Xiaobin: Nothing to disclose

### S 4-7

**Infrapopliteal endovascular intervention and the angiosome concept: intra-operative real-time assessment of foot regions blood volume guides and improves direct revascularisation**

*J. Ma*; Beijing/CN

**Purpose:** There is no consensus for determining which vessel should be revascularized in patients with multiple diseased infrapopliteal arteries. We conducted a study to assess whether direct revascularization (DR) will lead to better perfusion improvement than indirect revascularization (IR).
**Methods or Background:** We performed a prospective single-center observational cohort study in patients treated in the Department of Vascular Surgery of Peking Union Medical College Hospital from November 2016 to April 2019. Twenty-seven patients treated with endovascular intervention were included. The intraoperative parenchymal blood volume of different foot regions was obtained for each patient using C-arm CT before and after intervention.
**Results or Findings:** The intervention procedure significantly increased the overall blood volume (48.95 versus 81.97 ml/1000 ml, p=0.002). Patients with direct revascularization had a 197% blood volume increase while patients with indirect revascularization had a 39% increase (p=0.028). The preoperative blood volume was higher in patients with mild symptoms than in patients with severe symptoms (58.20 versus 30.45 ml/1000 ml, p=0.039).
**Conclusion:** Overall blood volume can rise significantly after the intervention. Revascularizing the supplying vessel of the ischemic area directly will result in better perfusion improvement than restoring blood supply through the collateral circulation. Preoperative blood volume is associated with preoperative symptoms.
**Limitations:** First, this was a prospective observational cohort study with a limited sample size. In addition, deviations existed because of foot deformation during the endovascular procedure and movement during image acquisition.
**Ethics Committee Approval:** The study was approved by the ethics committee of Peking Union Medical College Hospital.
**Funding for this study:** This study has received funding by Non-profit Central Research Institute Fund of Chinese Academy Of Medical Science, Beijing Municipal Science and Technology Commission "clinical application of capital characteristics" and National College Students' Innovation and Entrepreneurship Training Program.
**Author Disclosures:**
Jiangyu Ma: Nothing to disclose

**S 4-8**
**Linear mixed-effects models for estimation of pulmonary metastasis growth rate: implications for CT surveillance in patients with sarcoma**
*U. Isidro*[1], L. O'Brien[2], R. Sebro[1]; [1]Philadelphia, PA/US, [2]Waterville, ME/US

**Purpose:** No data exist on optimal surveillance interval for chest CT for detecting metastases in sarcoma patients. This study aimed to estimate pulmonary metastasis growth rate.
**Methods or Background:** Retrospective review of 95 sarcoma patients with pulmonary metastases treated at an academic tertiary-care center from 2000-2019. Age, sex, primary tumor size, grade, subtype, size and volume of the pulmonary metastasis over successive chest CT scans were recorded. Multivariate linear mixed-effects models with random effects for each pulmonary metastasis and patient were used to estimate pulmonary metastasis growth rate and volume doubling time.
**Results or Findings:** Maximal primary tumor size and patient age at diagnosis, tumor grade, and tumor type were not statistically significant predictors of pulmonary nodule growth. Chemotherapy decreased rate of pulmonary nodule growth. 95% of untreated pulmonary metastases are expected to grow less than 6 mm in 6.4 months. There was significant intrapatient and interpatient variation in pulmonary metastasis growth rate. Volume doubling time for pulmonary metastases assuming an exponential model was 143 days.
**Conclusion:** Assuming a 2 mm nodule is the smallest reliably detectable nodule by CT, the data suggest an untreated pulmonary metastasis is expected to grow to 8 mm in 8.4 months. Tumor size, grade and sarcoma subtype did not significantly alter pulmonary metastasis growth rate; chemotherapy slowed the growth rate.
**Limitations:** Retrospective study of patients treated at a single tertiary care academic institution and subject to ascertainment bias. All sarcoma subtypes were not represented. All metastases were not histologically diagnosed via biopsy. There was heterogeneity in chemotherapy regimens administered. The small sample size limits ability to detect small differences between pulmonary metastasis growth rate for sarcoma subtypes.
**Ethics Committee Approval:** Reviewed and approved by the local IRB. Signed informed consent from each patient was waived.
**Funding for this study:** None
**Author Disclosures:**
Ulysses Isidro: Nothing to disclose
Ronnie Sebro: Nothing to disclose
Liam O'Brien: Nothing to disclose

### E³ - The Beauty of Basic Knowledge: Breast

### E³ 24A
### Basics of mammography

### E³ 24A-1
**Cookbook for image analysis and interpretation**
Anne Tardivon; Paris/FR
*(anne.tardivon@curie.net)*

**Learning Objectives:**
1. To learn to assess mammographic image quality and mammographic breast density.
2. To recognise and name the main imaging findings using the breast imaging reporting and data system (BIRADS) lexicon.

### E³ 24A-2
**Differences in mammography techniques and image analysis**
Sophia Zackrisson; Malmö/SE
*(sophia.zackrisson@med.lu.se)*

**Learning Objectives:**
1. To learn about the technique and purpose of tomosynthesis compared to digital mammography.
2. To understand the differences in reading a screening and diagnostic mammography.
3. To become familiar with work-up possibilities after a positive screening mammography, taking into account psychological consideration.
**Author Disclosures:**
Sophia Zackrisson: Patent Holder: US patent no PCT/EP2014/057372; Speaker: Siemens Healthcare AG

### E³ - The Beauty of Basic Knowledge: Breast

### E³ 24C
### Basics of breast MRI

### E³ 24C-1
**When is breast MRI indicated and what protocol to use?**
Ritse M. Mann; Nijmegen/NL
*(r.mann@rad.umcn.nl)*

**Learning Objectives:**
1. To become familiar with the guidelines for accepted indications of breast MRI.
2. To learn the basic protocol for screening and diagnostic breast MRI.
**Author Disclosures:**
Ritse M. Mann: Other: copy

### E³ 24C-2
**How to read breast MRIs**
Federica Pediconi; Rome/IT
*(federica.pediconi@uniroma1.it)*

**Learning Objectives:**
1. To understand morphological and kinetic findings.
2. To learn the value of diffusion-weighted imaging.
3. To learn about reporting by using the breast imaging reporting and data system (BIRADS).

### E³ - The Beauty of Basic Knowledge: Breast

### E³ 24D
### Basics of interventional breast imaging

### E³ 24D-1
**Ultrasound-guided interventional imaging: when and how?**
Mary C. Mahoney; Cincinnati, OH/US
*(mahonemc@ucmail.uc.edu)*

**Learning Objectives:**
1. To learn about cyst aspiration and biopsy techniques.
2. To understand the contraindication and complications.
3. To understand the correlation between imaging and pathology.
**Author Disclosures:**
Mary C. Mahoney: Board Member: RSNA, ACR; Other: Editorial Board CDR

### E³ 24D-2
**When and how to biopsy under mammographic guidance?**
Dragana Djilas-Ivanovic; Sremska Kamenica/RS
*(djilas.dr@gmail.com)*

**Learning Objectives:**
1. To learn about the techniques and limitations.
2. To become familiar with the quality control of stereotactic- or tomosynthesis-guided breast biopsy.

### E³ 24D-3
**When and how to biopsy under MRI guidance?**
Pascal A.T. Baltzer; Vienna/AT

**Learning Objectives:**
1. To learn about the techniques and limitations.
2. To become familiar with the quality control of MRI-guided breast biopsy.

### E³ - The Beauty of Basic Knowledge: Breast

### E³ 24E

### How to deal with common clinical breast symptoms

**E³ 24E-1**
**The acute painful breast**
Mihai Lesaru; Bucharest/RO

**Learning Objectives:**
1. To learn about the choice of imaging modality.
2. To understand the management options and treatment of common breast lesions causing pain, including mastitis.

**E³ 24E-2**
**How to manage nipple discharge**
Isabelle Thomassin-Naggara; Paris/FR
*(isabelle.thomassin@tnn.aphp.fr)*

**Learning Objectives:**
1. To identify causes of nipple discharge unrelated to the breast or physiology.
2. To learn about the choice and sequence of imaging modalities to identify breast lesions causing nipple discharge.
3. To understand the spectrum of common benign and malignant breast lesions in relation to nipple discharge.

**Author Disclosures:**
Isabelle Thomassin-Naggara: Speaker: GE, Hologic, Guerbet, canon; Other: Siemens

### E³ - The Beauty of Basic Knowledge: Understanding MRI Technique and MRI Safety

### E³ 25B

### Static magnetic fields

**E³ 25B-1**
**MRI at high-field strengths: advantages and clinical challenges**
Anja van der Kolk; Utrecht/NL
*(A.G.vanderKolk@umcutrecht.nl)*

**Learning Objectives:**
1. To get familiar with the advantages when moving to higher filed strength.
2. To know the clinical benefits of higher field strengths.
3. To understand the challenges of higher field strengths.

**E³ 25B-2**
**MRI safety: risks with static magnetic fields**
Jacques Felblinger; Lyon/FR
*(j.felblinger@chu-nancy.fr)*

**Learning Objectives:**
1. To understand the risks of attraction for ferromagnetic material.
2. To understand the risks of torque for ferromagnetic material.
3. To understand how the manufacturer evaluates medical devices in MRI conditions.

**Author Disclosures:**
Jacques Felblinger: Founder: Healtis

### E³ - The Beauty of Basic Knowledge: Understanding MRI Technique and MRI Safety

### E³ 25C

### Time varying electromagnetic fields

**E³ 25C-1**
**Coils: important players in MRI**
Franz Schmitt; Erlangen/DE
*(schmittfranz53@gmail.com)*

**Learning Objectives:**
1. To understand the difference between transmit and receive coils.
2. To understand why local receive coils provide a better image quality, i.e. signal-to-noise ratio.
3. To understand the importance of local multi-receive coils for accelerated MRI.

**Author Disclosures:**
Franz Schmitt: Consultant: Flywheel.io

**E³ 25C-2**
**MRI safety: risks with high frequency and gradient fields**
Siegfried Trattnig; Vienna/AT
*(siegfried.trattnig@meduniwien.ac.at)*

**Learning Objectives:**
1. To learn about the risks associated with high frequency field, in particular the different types of burns.
2. To learn how to avoid high frequency burns in clinical MRI examinations.
3. To understand the risks of peripheral nerve stimulation and how to avoid them.

### E³ - The Beauty of Basic Knowledge: Understanding MRI Technique and MRI Safety

### E³ 25D

### MRI in the presence of implants

**E³ 25D-1**
**Sequence techniques and protocols**
Reto Sutter; Zurich/CH

**Learning Objectives:**
1. To recognise the common types of metal-induced artifacts in MRI and to identify the diagnostic pitfalls.
2. To apply effective basic techniques for reducing MRI metal artifacts in turbo spin echo sequences and fat saturated sequences.
3. To learn about the advanced techniques reducing through-plane distortions in MRI and their clinical impact.

**Author Disclosures:**
Reto Sutter: Author: Breitenseher Publisher; Other: Balgrist University Hospital has an academic research collaboration with Siemens Healthineers and Balzano Informatik.

**E³ 25D-2**
**MRI implant safety assessments**
Nadia Oberhofer; Bozen/IT
*(NADIA.OBERHOFER@sabes.it)*

**Learning Objectives:**
1. To understand and learn how to minimise the risk related to active electronic implants in an MRI environment.
2. To understand and learn how to minimise the risk related to passive implants in patients and staff in an MRI environment.
3. To understand the role of equipment choices in reducing the risk related to implants in an MRI environment.
**Author Disclosures:**
Nadia Oberhofer: Equipment Support Recipients: Metrasens, Kopp Development

### E³ - The Beauty of Basic Knowledge: Understanding MRI Technique and MRI Safety

### E³ 25E

### Working in the MRI environment

**E³ 25E-1**
**Intraoperative/interventional MRI: methods and applications**
Andreas Melzer; Leipzig/DE
*(andreas.melzer@uni-leipzig.de)*

**Learning Objectives:**
1. To learn about the technological prerequisites for interventional and intraoperative MRI, including MRI systems overviews, requirements, and recommendations for safe use.
2. To become familiar with the safety of interventional and surgical devices in the MRI environment.
3. To learn about appropriate workflow and enabling equipment handling for effective and safe MRI-guided procedures.
**Author Disclosures:**
Andreas Melzer: Grant Recipient: EU FP7, H2020 and BMBF; Investigator: EU FP7, H2020 and BMBF; Research Grant/Support: EU FP7, H2020 and BMBF

**E³ 25E-2**
**MRI safety education and awareness**
Titti Owman; Lund/SE
*(Titti.Owman@gmail.com)*

**Learning Objectives:**
1. To understand the importance of MRI safety education and why it must be repeated and updated regularly.
2. To clarify who needs MRI safety education, the different levels of education, and how to create an efficient and sufficient educational organisation.
3. To discuss how to improve awareness of the MRI safety risks and the necessity of reporting incidents.

### E³ - European Diploma Prep Sessions

### E³ 123

### Gastrointestinal and abdominal

**E³ 123-1**
**A. Hepatobiliary system**
Giuseppe Brancatelli; Palermo/IT
*(gbranca@yahoo.com)*

**Learning Objectives:**
1. To become familiar with the imaging features of diffuse liver diseases.
2. To understand the imaging features of focal liver lesions.
**Author Disclosures:**
Giuseppe Brancatelli: Consultant: Bayer; Speaker: Bayer, GE

**E³ 123-2**
**B. Pancreas and spleen**
Andreas Schreyer; Brandenburg/DE
*(andreas.schreyer@mac.com)*

**Learning Objectives:**
1. To understand the anatomy, normal variants and congenital disorders of the pancreas.
2. To become familiar with the causes and imaging features of benign and malignant pancreatic tumours.
3. To understand the imaging features of acute and chronic pancreatitis and its potential complications.
4. To learn the causes and imaging features of focal and diffuse splenic abnormalities.
**Author Disclosures:**
Andreas Schreyer: Advisory Board: Takeda; Consultant: Bayer Vital GmbH; Share holder: Siemens Healthineers; Speaker: Bayer Vital GmbH, Siemens Healthineers

**E³ 123-3**
**C. Imaging of the gastrointestinal tract**
Siobhan A. Whitley; Bury St Edmunds/UK

**Learning Objectives:**
1. To become familiar with the anatomy, normal variants, and congenital disorders of the gastrointestinal tract.
2. To understand the imaging features of the most commonly encountered causes of an acute abdomen.
3. To learn typical radiological manifestations of inflammatory, infective, and ischaemic bowel diseases.
4. To become familiar with the staging of tumours of the gastrointestinal tract and to understand the role of different imaging modalities in diagnosing and staging.

### E³ - European Diploma Prep Sessions

### E³ 223

### Neuro

**E³ 223-1**
**A. Congenital and white matter disorders of the brain**
Andrea Rossi; Genoa/IT
*(andrearossi@gaslini.org)*

**Learning Objectives:**
1. To understand the development, normal anatomy, and normal variants of the brain.
2. To become familiar with common congenital disorders of the brain and neurocutaneous syndromes.
3. To learn the imaging features and differential diagnoses of white matter disease, inflammation, and neurodegeneration.

**E³ 223-2**
**B. Neurovascular disorders and trauma of the brain**
Meike Vernooij; Rotterdam/NL
*(m.vernooij@erasmusmc.nl)*

**Learning Objectives:**
1. To become familiar with the normal anatomy and normal variants of the craniocervical arterial and venous system.
2. To learn the causes and imaging features of stroke, haemorrhage, and other common vascular lesions of the brain, and their relevance to interventional neuroradiology.
3. To understand the imaging features of traumatic injury to the brain.
**Author Disclosures:**
Meike Vernooij: Board Member: ESNR

**E³ 223-3**
**C. Tumours of the brain and spine**
Majda M. Thurnher; Vienna/AT
*(majda.thurnher@meduniwien.ac.at)*

**Learning Objectives:**
1. To understand the normal anatomy and normal variants of the spine, spinal cord, and nerve roots.
2. To learn about the imaging features of benign and malignant tumours of the neurocranium.
3. To become familiar with the imaging features of benign and malignant tumours of the spine.

### E³ - European Diploma Prep Sessions

### E³ 323

### Principles of imaging and radiation protection

**E³ 323-1**
**A. Principles of computed tomography**
Elly Castellano; London/UK
*(elly.castellano@rmh.nhs.uk)*

**Learning Objectives:**
1. To understand the physical basis of image formation of computed tomography and of the physics of helical, multidetector, and dual-source CT.
2. To learn the scale of Hounsfield units and the principle of window centre and width.
3. To become familiar with modern CT technology.
4. To understand the principles of optimising CT protocols with a focus on patient dose reduction.

**E³ 323-2**
**B. Principles of magnetic resonance imaging**
Dominique Sappey-Marinier; Lyon/FR
*(Dominique.Sappey-Marinier@univ-lyon1.fr)*

**Learning Objectives:**
1. To become familiar with the physical basis of image formation in MRI including the principles of pulse sequences and relaxation times.
2. To become familiar with the typical appearance of tissues, organs, and main pathological processes of the most commonly used sequences in MRI.
3. To learn typical artefacts on MRI and to discuss their respective causes.
4. To understand absolute or relative contraindications against MRI and safety issues in the MR environment with regard to patients and staff.

**E³ 323-3**
**C. Radiation protection**
John Damilakis; Iraklion/GR

**Learning Objectives:**
1. To understand the phenomena of x-ray interaction with matter and their effect on image quality and dose.
2. To provide an overview of patient and staff dosimetry in diagnostic and interventional radiology.
3. To understand the basic principles of radiation protection as outlined by the ICRP (International Commission on Radiological Protection).
4. To become familiar with the concepts and tools for dose management in radiology with regard to adult and paediatric patients.

### E³ - European Diploma Prep Sessions

### E³ 423

### Chest

**E³ 423-1**
**A. Fundamentals of chest imaging**
Okka Hamer; Regensburg/DE
*(okka.hamer@klinik.uni-regensburg.de)*

**Learning Objectives:**
1. To learn the anatomy, including normal "lines and stripes", of the lung parenchyma, mediastinum, and pleura, and to confidently identify these on radiographs.
2. To gain an in-depth understanding of the signs of chest radiography pathology.
3. To learn about differential diagnoses and pitfalls in chest radiography.
**Author Disclosures:**
Okka Hamer: Speaker: Boehringer-Ingelheim, Roche

**E³ 423-2**
**B. Inflammation of the lung**
Daria Manos; Halifax, NS/CA

**Learning Objectives:**
1. To understand the imaging features and differential diagnoses of diffuse fibrotic lung disease.
2 To learn the common causes of acute and chronic diffuse airspace disease.
3. To become familiar with thoracic diseases in immunocompetent, and immunocompromised patients.

**E³ 423-3**
**C. Lung and pleural malignancy**
Peter Beddy; Dublin/IE

**Learning Objectives:**
1. To become familiar with the differentiation of solitary and multiple pulmonary nodules and benign and malignant neoplasms.
2. To become familiar with the imaging features of pleural malignancy.
3. To understand the role of different imaging modalities, including hybrid imaging, in diagnosing and staging neoplasms of the chest.

### E³ - European Diploma Prep Sessions

### E³ 523

### Musculoskeletal

**E³ 523-1**
**A. Traumatic disorders of the musculoskeletal system**
Andrew J. Grainger; Cambridge/UK

**Learning Objectives:**
1. To develop an understanding of the underlying mechanisms of injury in appendicular skeletal trauma and to correlate it with imaging findings.
2. To gain an appreciation of typical fracture patterns and their accompanying soft tissue injuries.
3. To understand the benefits and disadvantages of different imaging modalities in assessing trauma involving the skeleton and soft tissue in differing age groups.

**E³ 523-2**
**B. Bone tumours**
Marc-André Weber; Rostock/DE
*(marc-andre.weber@med.uni-rostock.de)*

**Learning Objectives:**
1. To learn about the typical imaging features of common bone tumours.
2. To understand the typical imaging features of "don't touch" lesions.
3. To become familiar with the imaging manifestations of haematological disorders.

**E³ 523-3**
**C. Degenerative and inflammatory disorders of the spine**
Monique Reijnierse; Leiden/NL
*(m.reijnierse@lumc.nl)*

**Learning Objectives:**
1. To learn about the imaging features and clinical features of degenerative diseases of the spine.
2. To become familiar with the typical imaging manifestations of infection, inflammation, and metabolic diseases of the spine.
3. To understand the application of different imaging modalities.

### E³ - European Diploma Prep Sessions

### E³ 623
### Breast

### E³ 623-1
**A. Fundamentals of mammography**
James Tanner; Cambridge/UK

**Learning Objectives:**
1. To understand the anatomy, normal appearances, variants, and abnormalities of the female breast.
2. To become familiar with the technical aspects of diagnostic mammography, especially in regard to dose and image quality.
3. To become familiar with the principles of current practice and risk/benefit analysis in breast cancer screening.

### E³ 623-2
**B. Breast cancer diagnosis and interventions**
Tamar Sella; Jerusalem/IL
*(tamarse@hadassah.org.il)*

**Learning Objectives:**
1. To learn about the differentiation of benign and malignant breast disease.
2. To understand the principles and basic application of standardised diagnostic categorisation systems such as BI-RADS® (the ACR breast imaging reporting and data system).
3. To become familiar with indications, contraindications, and technical aspects of image-guided interventional breast procedures.

### E³ 623-3
**C. Advanced imaging of the female breast**
Eva M. Fallenberg; Munich/DE

**Learning Objectives:**
1. To understand the role of advanced imaging techniques in the evaluation of the breast.
2. To learn to use the added value of new techniques for lesion classification in mammography, ultrasound, and MRI.
3. To recognise the major imaging challenges for common indications of breast imaging.
**Author Disclosures:**
Eva M. Fallenberg: Advisory Board: Bayer Siemens Guerbet; Board Member: EUSOBI DRG; Grant Recipient: DFG; Research Grant/Support: GE Guerbet; Speaker: GE Siemens Bayer Guerbet

### Special Focus Session

### SF 3
### The prince that was promised: status of PET/MRI after the first 10 years

### SF 3-1
**Complementary information: the key to success?**
Lale Umutlu; Essen/DE

**Learning Objectives:**
1. To appreciate the protocols that can be used for PET/MRI imaging to maximise information gained in oncology imaging.
2. To understand how the morphological and functional aspects of MRI and molecular information from PET can be synergistic to give a better outcome for oncology imaging.

### SF 3-2
**Impact of PET/MRI on cancer patient management: the evidence**
Onofrio A. Catalano; Boston, MA/US
*(onofriocatalano@yahoo.it)*

**Learning Objectives:**
1. To understand the evidence that exists for the use of PET/MRI in oncologic imaging practice.
2. To become familiar with some of the clinical indications of PET/MRI in oncological practice.
3. To appreciate potential advantages of combined PET/MRI in oncologic imaging.

### SF 3-3
**Prostate cancer imaging with PSMA-PET/MRI: the killer application?**
Marcus R. Makowski; Berlin/DE

**Learning Objectives:**
1. To become familiar with the protocols that can be used with PET/MRI in prostate cancer imaging.
2. To learn about the current literature supporting the use of PET/MRI in prostate cancer.
3. To appreciate the strengths and weaknesses of PET/MRI in prostate cancer imaging.

### Special Focus Session

### SF 4
### CT-derived fractional flow reserve: hope or hype in cardiac imaging?

### SF 4-1
**Fractional flow reserve: an introduction**
Ricardo P.J. Budde; Rotterdam/NL
*(r.budde@erasmusmc.nl)*

**Learning Objectives:**
1. To explain the basic concept of fractional flow reserve in coronary arteries.
2. To review the available techniques for CT-derived fractional flow reserve.
3. To discuss how CT-derived fractional flow reserve is done in clinical practice.
**Author Disclosures:**
Ricardo P.J. Budde: Research Grant/Support: Heartflow. Siemens

### SF 4-2
**CT-derived fractional flow reserve: available techniques and current clinical evidence**
Gianluca Pontone; Milan/IT

**Learning Objectives:**
1. To learn about different solutions of FFRct.
2. To learn how FFRct can improve diagnostic accuracy.
3. To learn how the integration of FFRct in the diagnostic pathway is cost effective.

### SF 4-3
**CT and MRI perfusion: how do they compare?**
Fabian Bamberg; Freiburg/DE
*(fabian.bamberg@uniklinik-freiburg.de)*

**Learning Objectives:**
1. To learn about the technical background of CT and MRI-based perfusion imaging.
2. To understand the differences in imaging protocols between the two approaches.
3. To become familiar with the current level of scientific evidence on the value of CT and MRI-based perfusion imaging.
**Author Disclosures:**
Fabian Bamberg: Grant Recipient: Bayer Healthcare, Siemens Healthineers; Speaker: Bayer Healthcare, Siemens Healthineers

### Special Focus Session

### SF 6

### Tumour profiling: established and novel MRI techniques

### SF 6-1

**Perfusion: neovascularisation and aggressiveness**
Vicky J. Goh; London/UK
*(vicky.goh@kcl.ac.uk)*

**Learning Objectives:**
1. To understand how DCE enables improved cancer detection by revealing microvasculature and perfusion.
2. To become familiar with quantitative analysis of signal intensity-time curves after intravenous contrast agent administration in order to reveal cancer specific perfusion-related parameters.
3. To appreciate the diagnostic potential of DCE to detect significant prostate cancer.

**Author Disclosures:**
Vicky J. Goh: Research Grant/Support: Siemens Healthcare

### SF 6-2

**DWI: tissue architecture and cell viability**
Ravikanth Balaji; Chennai/IN
*(ravikanthbalaji@gmail.com)*

**Learning Objectives:**
1. To understand how DWI enables improved cancer detection by revealing microstructural tissue features.
2. To become familiar with quantitative analysis of signal intensity-curves dependent on b-values to reveal quantitative tissue parameters (i.e. IVIM, ADC, and kurtosis).
3. To appreciate the diagnostic potential of DWI to detect and characterise cancer.

### SF 6-3

**CEST: protein content and cellular metabolism**
Linda Knutsson; Lund/SE
*(linda.knutsson@med.lu.se)*

**Learning Objectives:**
1. To understand the basic physical principles of CEST MRI.
2. To become familiar with the (patho-)biologic correlates of CEST contrast.
3. To learn about clinical applications in oncology.

### SF 6-4

**Fingerprinting: the future of functional MR imaging**
Siegfried Trattnig; Vienna/AT
*(siegfried.trattnig@meduniwien.ac.at)*

**Learning Objectives:**
1. To understand the rf pulse design of MR fingerprinting sequences (MRF).
2. To appreciate how MRF enables the quantifying of tissue properties.
3. To appreciate clinical applications in oncology.

### Special Focus Session

### SF 14

### Chest emergencies

### SF 14-1

**Dyspnoea: how to approach the diagnosis of diffuse lung abnormalities on CT**
Juan José Arenas-Jiménez; Alicante/ES
*(j.arenasjimenez@gmail.com)*

**Learning Objectives:**
1. To become familiar with the characteristic radiologic patterns on CT of diffuse lung abnormalities that can be diagnostic for a specific disease.
2. To understand the importance of the clinical and laboratory background when interpreting CT with diffuse changes in patients with acute dyspnoea.
3. To learn about how to use a proposed checklist combining clinical and radiological information to narrow the differential diagnosis in patients with acute dyspnoea and diffuse lung abnormalities on CT.

### SF 14-2

**Non-cardiac chest pain: how to proceed?**
Johann Baptist Dormagen; Oslo/NO
*(uxjorm@ous-hf.no)*

**Learning Objectives:**
1. To become familiar with the clinical features and patient characteristics in acute and chronic non-cardiac chest pain (NCCP) and with common and less common causes of NCCP.
2. To understand the clues and pitfalls when imaging the chest for vascular emergencies, including dissection, pulmonary embolism, and less common non-vascular conditions in patients presenting with NCCP.
3. To appreciate the importance of different imaging modalities for differential diagnoses of NCCP.

### SF 14-3

**Acute haemoptysis: causes and radiological treatment**
Mark O. Wielpütz; Heidelberg/DE
*(wielpuetz@uni-heidelberg.de)*

**Learning Objectives:**
1. To appreciate that haemoptysis can be a life-threatening pulmonary emergency with high mortality, is symptomatic of an underlying severe pulmonary disease, and requires immediate diagnosis and treatment.
2. To learn about contrast-enhanced multi-detector computed tomography with CT angiography as a diagnostic modality in cases of acute haemoptysis to gain information on the underlying pulmonary disease, bleeding site, and vascular anatomy of the bronchial arteries.
3. To understand that bronchial artery embolisation is a safe and potentially lifesaving therapy in severe haemoptysis.
4. To become familiar with the fact that false embolisation in spinal branches of bronchial arteries is the most serious complication of bronchial artery embolisation.

**Author Disclosures:**
Mark O. Wielpütz: Research Grant/Support: Vertex, Boehringer Ingelheim

### Special Focus Session

### SF 16

### Risk-adjusted screening for breast cancer

### SF 16-1

**Methods to assess breast cancer risk**
Mireille Broeders; Nijmegen/NL
*(m.broeders@lrcb.nl)*

**Learning Objectives:**
1. To become familiar with the current risk prediction models for breast cancer and their potential use in screening.
2. To understand the most important measures used to determine how well a risk model performs.
3. To learn about the ongoing research to further improve models to assess breast cancer risk.

**Author Disclosures:**
Mireille Broeders: Employee: Professor of Personalized Cancer Screening, Radboudumc, Nijmegen, the Netherlands; Scientific Supervisor, Dutch Expert Centre for Screening, Nijmegen, the Netherlands; Speaker: Agreements: Hologic and Siemens

### SF 16-2

**Short-term, image-based breast cancer risk prediction model**
Per Hall; Stockholm/SE
*(per.hall@ki.se)*

**Learning Objectives:**
1. To argue why the same risk models should not be used for individualised prevention and early detection of breast cancer.
2. To understand why early detection risk models must include mammographic features and should not have a longer time span than the screening interval.
3. To show why risk assessment should be repeated and performed each time a woman performs screening mammography.
**Author Disclosures:**
Per Hall: Advisory Board: Atossa Therapeutics; Patent Holder: Patent pending

### SF 16-3
**Risk-based breast cancer screening programme: an example for the USA**
Karla Kerlikowske; San Francisco, CA/US
*(Karla.Kerlikowske@ucsf.edu)*

**Learning Objectives:**
1. To appreciate the differences in risk prediction for overall breast cancer risk vs intermediary breast cancer outcomes.
2. To understand how risk stratification impacts the balance of benefits and harms of screening.
3. To become familiar with risk-based screening approaches in the US.
**Author Disclosures:**
Karla Kerlikowske: Grant Recipient: NCI and PCORI

### SF 16-4
**Screening with MRI in women with dense breasts**
Carla van Gils; Utrecht/NL

**Learning Objectives:**
1. To appreciate the importance of breast cancer masking in addition to breast cancer risk.
2. To become familiar with the approaches of using breast density to personalise breast cancer screening with MRI.
3. To learn about the performance of MRI in women with dense breasts and increased breast cancer risk.
**Author Disclosures:**
Carla van Gils: Consultant: Bayer Pharma AG; Research Grant/Support: Bayer Pharma AG, Volpara Solutions (in kind); Speaker: Bayer Pharma AG

---

### Special Focus Session
*Organised by ESR eHealth and Informatics Subcommittee*

### SF 18
### Deep learning and image quality

### SF 18-1
**AI-enhanced image processing: noise reduction**
Joel Greffier; Nimes/FR
*(joel.greffier@chu-nimes.fr)*

**Learning Objectives:**
1. To gain basic understanding of noise and how to measure it in radiologic imaging.
2. To learn the principles of AI-enhanced noise reduction in medical imaging.
3. To know the advantages and inconveniences of AI-enhanced noise reduction.

### SF 18-2
**How to use deep learning to improve image quality**
Yuko Nakamura; Hiroshima/JP

**Learning Objectives:**
1. To understand how image quality in radiology is traditionally improved.
2. To learn how deep learning can be used to improve image quality.
3. To explain the advantage of using deep learning to improve image quality.

### SF 18-3
**AI helping to optimise dose and contrast agent application**
Christoph Hoeschen; Magdeburg/DE
*(christoph.hoeschen@ovgu.de)*

**Learning Objectives:**
1. To learn about the relationship between radiation dose, contrast medium application and image quality.
2. To understand how AI can be used to optimise dose and contrast medium application.

---

### Special Focus Session

### SF 23
### Organ transplantation

### SF 23-1
**Brain death and donor evaluation**
Ferdia Bolster; Dublin/IE

**Learning Objectives:**
1. To obtain an overview of brain death and the clinical determination of brain death.
2. To become aware of ancillary testing in brain death with a particular focus on multimodality radiology imaging in the determination of brain death, including digital subtraction angiography, transcranial Doppler, nuclear medicine scintigraphy, computed tomography (CT) and magnetic resonance imaging (MRI), CT and MR angiography, and CT and MR perfusion.
3. To understand the role of the radiologist in the early recognition of brain death, to review some conditions that can mimic brain death/pitfalls, and to review donor evaluation.

### SF 23-2
**Living donor evaluation: liver and kidney**
Federica Vernuccio; Palermo/IT
*(federicavernuccio@gmail.com)*

**Learning Objectives:**
1. To obtain an overview of the key surgical considerations and the role of imaging for the evaluation of living liver and kidney donors.
2. To provide an outline of the optimal CT imaging protocol used in the preoperative evaluation of living liver and kidney donors.
3. To discuss the main reportable findings for the diagnostic imaging work-up of living liver and kidney donors.

### SF 23-3
**Recipient evaluation pre- and post-Tx: liver**
Rossano Girometti; Udine/IT
*(rgirometti@sirm.org)*

**Learning Objectives:**
1. To understand the role of preoperative imaging in selecting candidates for liver transplant.
2. To understand the role of postoperative imaging in assessing transplant-related complications.
3. To summarise the main imaging algorithms and findings in pre- and post-operative scenarios.

### SF 23-4
**Recipient evaluation pre- and post-Tx: lung**
Marie-Pierre Debray; Paris/FR
*(marie-pierre.debray@aphp.fr)*

**Learning Objectives:**
1. To learn about the imaging features evaluated in recipients before lung transplantation and to understand those that may favour mono- or bi-pulmonary transplantation and those that may lead to exclusion from the waitlist.
2. To learn to recognise the normal imaging appearance of the lung allograft in the early phase after transplantation.
3. To obtain an overview of the imaging features of the most common complications after lung transplantation.
**Author Disclosures:**
Marie-Pierre Debray: Speaker: Boehringer Ingelheim

### SF 23-5
**Contrast-enhanced ultrasound in the follow-up evaluation of kidney-Tx**
Dirk André Clevert; Munich/DE
*(Dirk.Clevert@med.uni-muenchen.de)*

**Learning Objectives:**
1. To learn about actual indications and applications of CEUS in the follow-up after transplantation of the kidney.
2. To demonstrate better parameter settings for optimal technical results.
3. To illustrate the tips and tricks for technical and clinical successful examinations.
4. To become familiar with common transplantation pathologies of the kidney.
**Author Disclosures:**
Dirk André Clevert: Speaker: Bracco, Siemens, Philips, Esaote, Samsung

### Special Focus Session

### SF 24

### Oral and maxillofacial radiology

### SF 24-1
**Overview of salivary gland imaging with an emphasis on sialo-CBCT**
Chen Nadler; Jerusalem/IL
*(nadler@hadassah.org.il)*

**Learning Objectives:**
1. To learn about the sialography indications of salivary gland pathosis.
2. To become familiar with sialography's role in demonstrating the radiographic features of salivary gland pathosis, computer-aided diagnosis, and creating a unique aid for patient assessment and treatment.
3. To obtain an overview of ultrasound and sialaendoscopy for salivary gland diagnosis and interventional treatment.

### SF 24-2
**Role of imaging in the diagnosis of temporomandibular joint pathology**
Tore A. Larheim; Oslo/NO
*(t.a.larheim@odont.uio.no)*

**Learning Objectives:**
1. To appreciate normal MRI variations.
2. To become familiar with the most frequent MRI pathology (internal derangement, osteoarthritis, and inflammatory joint diseases).

### SF 24-3
**Oral and pharyngeal swallowing dysfunction among snorers, the elderly, and stroke and head and neck cancer patients**
Eva K. Levring Jäghagen; Umea/SE
*(eva.levring.jaghagen@umu.se)*

**Learning Objectives:**
1. To learn about the results from studies of oral and pharyngeal swallowing dysfunction, assessed with videofluoroscopy among snorers and patients after stroke and head and neck cancer treatment.
2. To learn about neuromuscular injuries in the oropharyngeal area among snorers.
3. To learn about the results of neuromuscular training for the improvement of swallowing function in the elderly and patients after stroke.

### State of the Art Symposium

### SA 2

### Radiological management of polytrauma

### SA 2-1
**Whole-body CT in polytrauma: are we imaging properly?**
Monique Brink; Nijmegen/NL
*(monique.brink@radboudumc.nl)*

**Learning Objectives:**
1. To learn the importance of choosing the optimal CT imaging protocol for trauma patients.
2. To understand the differences in single and dual phase imaging.
3. To appreciate the need to detect subtle but potentially fatal injuries in polytrauma patients.

**Author Disclosures:**
Monique Brink: Grant Recipient: Canon Medical Systems Europe; Speaker: Canon Medical Systems Europe

### SA 2-2
**Contrast-enhanced ultrasound (CEUS) in the follow-up of trauma patients: does it have a role?**
Annamaria Deganello; London/UK
*(adeganello@nhs.net)*

**Learning Objectives:**
1. To learn how to classify and grade solid organ injuries.
2. To become familiar with incorporating CEUS in trauma patient follow ups as an alternative to repeated CT-studies.
3. To appreciate patient selection and appropriate timing of follow up CEUSs.
4. To understand the advantages and limitations of CEUS in solid organ trauma.
**Author Disclosures:**
Annamaria Deganello: Speaker: Lecture fees Bracco

### SA 2-3
**MRI in the follow-up of solid organ injury in trauma patients**
Nupur Verma; Gainesville, FL/US

**Learning Objectives:**
1. To understand the feasibility and usefulness of MRI in solid organ injury follow up imaging.
2. To become familiar with the suitable candidates and timeline for MRI follow up with regard to severity of organ injury.
3. To understand the limitations and challenges of interpreting traumatic lesions and assessing blood products in MRI.

### SA 2-4
**Interventional management of abdominal vascular injuries**
Leena Lehti; Malmö/SE
*(leena.lehti.ll@gmail.com)*

**Learning Objectives:**
1. To learn about the patient selection and interventional treatment options for splenic, hepatic and renal injuries.
2. To become familiar with the recommended treatment options for solid organ and abdominal vascular injury.
3. To understand findings that indicate change of management from conservative to IR, from IR to surgical.
4. To understand what the interventional radiologist/surgeon needs to know.

### Professional Challenges Session

### PC 2

### Radiology reporting in the age of computer-based interpretation

### PC 2-1
**From automated measurements into meaningful reports**
Angel Alberich-Bayarri; Valencia/ES
*(angel@quibim.com)*

**Learning Objectives:**
1. To learn about the integration of reporting automation.
2. To become familiar with novel reporting methodologies.
3. To understand the role of man-machine interaction in reporting.
**Author Disclosures:**
Angel Alberich-Bayarri: CEO: QUIBIM S.L.

### PC 2-2
**Common data elements in use: a critical component for improved reporting quality**
Daniel Pinto dos Santos; Cologne/DE
*(daniel.pinto-dos-santos@uk-koeln.de)*

**Learning Objectives:**
1. To learn about the RSNA's and ACR's common data elements initiative.
2. To understand the differences and similarities between template-based structured reporting and the use of common data elements.
3. To appreciate the potential benefits of common data elements for clinical routine and scientific research.

### PC 2-3
**How to provide multi-media reports**
Florian Jungmann; Mainz/DE

**Learning Objectives:**
1. To understand how radiological reports can be enriched with information that help the clinical referrers to better capture the key messages.
2. To become familiar with the integration of schematic drawings, key images, and graphs and tables to illustrate responses in oncologic patients.
3. To learn about the challenges of implementing multi-media reports and how they can be solved.

### PC 2-4
**The value of structured information for research and quality management**
Elmar Kotter; Freiburg/DE
*(elmar.kotter@uniklinik-freiburg.de)*

**Learning Objectives:**
1. To learn about the use of structured information in research data repositories.
2. To understand how structured information can be used for the follow-up of findings.
3. To appreciate the potential of structured reporting for peer review and double-reading.

### Professional Challenges Session
*Organised by the ESR Ultrasound Subcommittee*

### PC 3
### The future of ultrasound in radiology

### PC 3-1
**Assessing point of care in ultrasound**
Maija Radzina; Riga/LV
*(mradzina@gmail.com)*

**Learning Objectives:**
1. To describe the current point of care concepts, guidelines, and technical aspects.
2. To provide an overview of pathologies and indications that can be covered by point of care ultrasound.
3. To outline the difference of diagnostic and point of care ultrasound applications and training requirements.
**Author Disclosures:**
Maija Radzina: Speaker: Canon, Bracco, Bayer

### PC 3-2
**Who should be performing ultrasound?**
Dirk André Clevert; Munich/DE
*(Dirk.Clevert@med.uni-muenchen.de)*

**Learning Objectives:**
1. To learn about who is performing ultrasound currently.
2. To understand the new technical developments in ultrasound.
3. To appreciate the advantages of ultrasound.
4. To become familiar with the future developments.
**Author Disclosures:**
Dirk André Clevert: Speaker: Bracco, Siemens, Samsung, Esaote

### PC 3-3
**What will the radiologists of the future be doing in their department?**
Paolo Ricci; Rome/IT
*(paolo.ricci@uniroma1.it)*

**Learning Objectives:**
1. To describe the organisation of an ultrasound unit in the radiology department.
2. To define how to challenge with the new ultrasound tools and facilities.
3. To identify a good method of cooperating with non-radiologists performing ultrasound.

### PC 3-4
**How can radiology adapt to the change to handheld devices?**
Vito Cantisani; Rome/IT
*(vito.cantisani@uniroma1.it)*

**Learning Objectives:**
1. To update on technical developments.
2. To define the role, limitations, and possibilities.
3. To show the actual European and non-European status.
**Author Disclosures:**
Vito Cantisani: Speaker: Bracco, Samsung, Canon

### Coffee & Talk (open forum) Session

### C 9
### Tracheobronchomalacia (TBM): optimising the diagnostic approach to this increasingly important childhood entity

### C 9-1
**The role of CT in tracheobronchomalacia**
Efthymia Alexopoulou; Athens/GR
*(ealex64@hotmail.com)*

**Learning Objectives:**
1. To learn about the indications for CT in TBM.
2. To understand optimised CT protocols.
3. To discuss the CT findings in TBM.

### C 9-2
**The role of MRI in tracheobronchomalacia**
Pierluigi Ciet; Rotterdam/NL
*(p.ciet@erasmusmc.nl)*

**Learning Objectives:**
1. To learn about the indications for MRI in TBM.
2. To understand the MRI imaging protocols.
3. To discuss the MRI findings in TBM.
**Author Disclosures:**
Pierluigi Ciet: Consultant: Vertex Pharmaceutical; Grant Recipient: ZonMW

### C 9-3
**Bronchoscopy: is it the gold standard?**
Konstantinos Priftis; Athens/GR
*(kpriftis@otenet.gr)*

**Learning Objectives:**
1. To acquire basic knowledge of bronchoscopic techniques.
2. To learn about the indications and limitations for bronchoscopy of the large airways in children.
3. To learn about typical bronchoscopic findings and therapeutic interventions in children.

### Refresher Course: Abdominal Viscera

### RC 101

### Benign and malignant lesions in "forgotten organs"

**RC 101-1**
**A. Imaging of the spleen**
Cäcilia S. Reiner; Zurich/CH

**Learning Objectives:**
1. To be aware of the most common benign and malignant lesions of the spleen.
2. To know about imaging strategies and protocols involving ultrasound, CT, and MRI.
3. To know about the management of splenic lesions.

**RC 101-2**
**B. Imaging of mesentery and omentum**
Antonella Filippone; Chieti/IT

**Learning Objectives:**
1. To understand the anatomical landmarks of the mesentery and omentum.
2. To be aware of the most common pathological findings.
3. To learn how to avoid diagnostic misinterpretation of mesentery and omentum anomalies.
**Author Disclosures:**
Antonella Filippone: Employee: Bracco Imaging

**RC 101-3**
**C. Imaging of the gallbladder**
Bart J. Op de Beeck; Antwerp/BE
*(bart.op.de.beeck@uza.be)*

**Learning Objectives:**
1. To become familiar with most common benign and malignant changes of the gallbladder.
2. To be aware of the strengths and shortcomings of different imaging techniques including ultrasound, CT, and MRI.
3. To understand diagnostic pitfalls of gallbladder imaging.

### Refresher Course: Abdominal Viscera

### RC 201

### Common benign and malignant liver lesions: unusual radiological appearance

**RC 201-1**
**A. Features of benign liver lesions**
Maria Manuela França; Porto/PT

**Learning Objectives:**
1. To be able to identify the most common benign liver lesions.
2. To learn about unusual appearances of benign liver tumours.
3. To develop an algorithm for correct identification of differentials.

**RC 201-2**
**B. Features of primary malignant liver lesions**
Giuseppe Brancatelli; Palermo/IT
*(gbranca@yahoo.com)*

**Learning Objectives:**
1. To learn about the typical features and prevalence of hepatocellular carcinomas (HCCs) and cholangiocellular carcinomas (CCCs).
2. To understand why HCCs and CCCs do not always show typical imaging features.
3. To establish a strategy for differentiating atypical HCCs and CCCs from other liver tumours.
**Author Disclosures:**
Giuseppe Brancatelli: Consultant: Bayer; Speaker: Bayer, GE healthcare

**RC 201-3**
**C. Features of liver metastases**
Asunción Torregrosa Andrés; Valencia/ES
*(asunsuso@gmail.com)*

**Learning Objectives:**
1. To review the most frequent tumours causing.
2. To understand why liver metastases can have different imaging characteristics.
3. To become familiar with post-therapeutic changes.

### Refresher Course: Abdominal Viscera

### RC 301

### Imaging of pancreatitis

**RC 301-1**
**A. Focal pancreatitis vs adenocarcinoma**
Thomas Bollen; Nieuwegein/NL
*(tlbollen@hotmail.com)*

**Learning Objectives:**
1. To learn about the clinical markers of focal pancreatitis and adenocarcinoma.
2. To understand the difficulties in differentiating these entities.
3. To learn about follow-up strategies in non-conclusive cases.

**RC 301-2**
**B. IgG4 autoimmune pancreatitis**
Ankur Arora; Worthing/UK

**Learning Objectives:**
1. To learn about the specific imaging characteristics of autoimmune pancreatitis.
2. To learn about the value of different imaging tool including US, CT, and MRI.
3. To be aware of imaging markers indicating therapeutic success in patients with autoimmune pancreatitis.

**RC 301-3**
**C. Imaging of chronic pancreatitis**
Giulia Zamboni; Verona/IT
*(gzamboni@hotmail.com)*

**Learning Objectives:**
1. To understand the strengths and weaknesses of different imaging tools.
2. To be aware of typical and atypical imaging findings in patients with chronic pancreatitis.
3. To learn about the pitfalls when evaluating patients with chronic pancreatitis.

**RC 301-4**
**D. Interventions in acute pancreatitis**
Michael M. Maher; Cork/IE
*(m.maher@ucc.ie)*

**Learning Objectives:**
1. To recognise the techniques of interventions in acute pancreatitis.
2. To understand the indications of transgastral vs percutaneous drainage.
3. To learn how to avoid complications following pancreatic interventions.

### Refresher Course: Abdominal Viscera

### RC 401

### Imaging of the biliary system

**RC 401-1**
**A. Magnetic resonance cholangiopancreatography (MRCP): state of the art**
Carlos Valls; Stockholm/SE
*(carlos.valls-duran@karolinska.se)*

**Learning Objectives:**
1. To learn about the different techniques of MRCP including their advantages and disadvantages.
2. To discuss differences of MRCP at 1.5T and 3.0T.
3. To understand how to deal with pitfalls in MRCP.

**RC 401-2**
**B. Primary sclerosing cholangitis (PSC)**
Giovanni Morana; Treviso/IT
*(gmorana61@gmail.com)*

**Learning Objectives:**
1. To be aware of the pathophysiological background of PSC.
2. To learn about typical and atypical imaging features of PSC.
3. To understand follow-up imaging strategies in patients with PSC.
**Author Disclosures:**
Giovanni Morana: Speaker: BRACCO

**RC 401-3**
**C. Biliary drainage and stenting**
Thomas K. Helmberger; Munich/DE
*(Thomas.Helmberger@klinikum-muenchen.de)*

**Learning Objectives:**
1. To understand the access routes for biliary drainage and stenting.
2. To be aware of pitfalls and complications during and after biliary interventions.
3. To learn how to evaluate therapeutic control on imaging.

### Refresher Course: Breast

### RC 202

### The radiologist and the oncoplastic surgeon: the importance of interaction for successful imaging evaluation

**RC 202-1**
**A. Oncoplastic surgical techniques: what the radiologist needs to know**
Isabel Rubio; Madrid/ES
*(irubior@unav.es)*

**Learning Objectives:**
1. To learn about the indications for oncoplastic surgery.
2. To describe the most common oncoplastic techniques, their strengths, and limitations.
3. To learn about the expected outcome and common complications after surgery.
**Author Disclosures:**
Isabel Rubio: Advisory Board: Sirius Medical

**RC 202-2**
**B. The importance of imaging when planning oncoplastic surgery**
Laura Martincich; Candiolo/IT

**Learning Objectives:**
1. To understand which imaging methods are most appropriate to use and when.
2. To learn which anatomical landmarks are more relevant in preoperative imaging.
3. To become updated on the latest imaging protocols.

**RC 202-3**
**C. Follow-up imaging of the oncoplastic breast: pearls and pitfalls**
Silvia Perez Rodrigo; Madrid/ES

**Learning Objectives:**
1. To understand which imaging methods and which intervals should be used for follow-up.
2. To learn about the most common false-positive and false-negative imaging findings.
3. To understand the role of imaging in follow-up and prognosis.

### Refresher Course: Breast

### RC 302

### Interventional breast imaging: the increasing role of the radiologist

**RC 302-1**
**A. Fine-needle aspiration cytology (FNAC), core-needle, or vacuum-assisted biopsy (VAB): what, when, and how?**
Andrew Evans; Dundee/UK
*(a.z.evans@dundee.ac.uk)*

**Learning Objectives:**
1. To get to know the different needle systems.
2. To become familiar with the potential pitfalls.
3. To be able to choose the right needle for the right indication.

**RC 302-2**
**B. Breast lesion localisation: going beyond wires**
Maja Marolt Music; Ljubljana/SI
*(mmusic@onko-i.si)*

**Learning Objectives:**
1. To appreciate the importance of image-guided preoperative wire localisation for non-palpable lesions.
2. To get to know alternative localisation techniques.
3. To become familiar with the limitations of different techniques.

**RC 302-3**
**C. Percutaneous ablation of breast cancer: a step forward**
Giovanni Mauri; Milan/IT
*(vanni.mauri@gmail.com)*

**Learning Objectives:**
1. To become familiar with the different techniques for tumour ablation.
2. To acknowledge the value of each technique for the treatment of breast lesions.
3. To identify the factors that affect success.
**Author Disclosures:**
Giovanni Mauri: Advisory Board: Boston Scientific; Speaker: GE healtcare

### Refresher Course: Breast

### RC 402

### Update on lesions with uncertain malignant potential (B3)

**RC 402-1**
**A. A wrap-up of the newest literature on the most important B3 lesions**
Zsuzsanna Varga; Zurich/CH

**Learning Objectives:**
1. To learn about the biological behaviour of the most important B3 lesions.
2. To understand the difference between B3a and B3b lesions.
3. To recognise the most worrisome entities.

### RC 402-2
**B. Imaging lesions of uncertain potential**
Magda Marcon; Zurich/CH
*(magda.marcon@usz.ch)*

**Learning Objectives:**
1. To become familiar with the most common imaging findings in B3 lesions.
2. To identify the most appropriate imaging modality.
3. To identify the factors that limit patient compliance to the management recommendations of high-risk breast lesions.

### RC 402-3
**C. How to handle them: update on B3 guidelines**
Nisha Sharma; Leeds/UK
*(nisha.sharma2@nhs.net)*

**Learning Objectives:**
1. To learn about the most recent international recommendations on the management of B3 lesions.
2. To differentiate between the results of a core-needle and a vacuum-assisted biopsy (VAB).
3. To be able to identify cases when VAB excision may pose a valid alternative to surgery.

### Refresher Course: Breast

### RC 502
### State of the art and recent developments in breast ultrasound

### RC 502-1
**A. Breast ultrasound: tell me the value of coloured images**
Corinne S. Balleyguier; Villejuif/FR
*(Corinne.BALLEYGUIER@gustaveroussy.fr)*

**Learning Objectives:**
1. To learn about the correct settings and interpretation of Doppler and elastography.
2. To understand their respective added value in lesion assessment.
3. To learn about their roll in follow-up and therapy monitoring.

**Author Disclosures:**
Corinne S. Balleyguier: Speaker: Samsung, Siemens

### RC 502-2
**B. Automated breast ultrasound (ABUS): the right add-on in screening dense breasts**
Athina Vourtsis; Athens/GR
*(athinavourtsi@yahoo.gr)*

**Learning Objectives:**
1. To learn how to perform, assess image quality, interpret, and recognise artefacts.
2. To review the current literature of ABUS in women with dense breasts.
3. To understand the value of 3D multiplanar images and ABUS workflow implementation compared to handheld US (HHUS).

**Author Disclosures:**
Athina Vourtsis: Advisory Board: Medical Advisory Board of Volpara Solutions; Grant Recipient: X; Investigator: GE Precision Healthcare LLC; Other: European Liaison for DenseBreast-Info Europe

### RC 502-3
**C. Can artificial intelligence (AI) be helpful in the US-screening setting?**
Panagiotis Kapetas; Vienna/AT
*(panagiotis.kapetas@meduniwien.ac.at)*

**Learning Objectives:**
1. To understand the current status of the use of AI in US breasts screening.
2. To realise the limitations in the implementation of AI in US examinations.
3. To explore potential future applications.

### Refresher Course: Cardiac

### RC 103
### Imaging the complexity of pulmonary hypertension (PH) "syndrome"

### RC 103-1
**A. Assessment of PH by CT**
Elena Mershina; Moscow/RU
*(elena_mershina@mail.ru)*

**Learning Objectives:**
1. To review the spectrum of currently available techniques, from pulmonary angiography to CT perfusion, with spectral imaging.
2. To list typical and subtle CT imaging parenchymal and vascular features in primary and secondary diseases.
3. To review the current role of CT in the clinical guidelines for the diagnosis and treatment of pulmonary hypertension.

### RC 103-2
**B. Uncoupling right ventricular physiology with cardiovascular magnetic resonance (CMR): from early adaptation to heart failure**
Karl-Friedrich Kreitner; Mainz/DE
*(Karl-Friedrich.Kreitner@unimedizin-mainz.de)*

**Learning Objectives:**
1. To review peculiarities of right heart physiology and ventricular interdependence in pulmonary hypertension.
2. To outline early and late morpho-functional changes associated with right ventricular pressure overload.
3. To understand the primary role of CMR in the evaluation of different forms of pulmonary hypertension.

### RC 103-3
**C. Cardiac imaging to monitor therapeutic response and predict outcome**
Maria Nedevska; Sofia/BG
*(nedevska_maria@yahoo.com)*

**Learning Objectives:**
1. To discuss the advances in treatment and importance of imaging to guide therapy.
2. To understand the predictive role of CT and MRI.
3. To analyse the importance and respective roles of CT and MRI to monitor response to therapy.

### Refresher Course: Cardiac

### RC 403
### Cardiac imaging in special patient populations

### RC 403-1
**A. Cardiovascular evaluation of the pregnant patient**
Birgitta K. Velthuis; Utrecht/NL
*(b.k.velthuis@umcutrecht.nl)*

**Learning Objectives:**
1. To learn about the normal physiological changes of the cardiovascular system during pregnancy.
2. To understand the impact of pregnancy on existing and newly developed cardiomyopathies.
3. To learn how to use CT/MRI for a pregnant patient with an acute cardiovascular problem.

**Author Disclosures:**
Birgitta K. Velthuis: Board Member: ESCR

**RC 403-2**
**B. Cardiotoxicity of recreational and therapeutic drugs**
Elie Mousseaux; Paris/FR
*(elie.mousseaux@egp.ap-hop-paris.fr)*

**Learning Objectives:**
1. To understand the effects of recreational drugs on the cardiovascular system.
2. To understand the cardiac consequences of different therapeutic agents used in oncology.
3. To establish a comprehensive CT/MRI imaging protocol for the evaluation of acute and chronically ill patients.

**RC 403-3**
**C. Systemic infection/inflammation and the heart with special considerations for imaging during a pandemic**
Marco Francone; Rome/IT
*(marco.francone@uniroma1.it)*

**Learning Objectives:**
1. To understand the aetiology and pathophysiology of different forms of myocarditis.
2. To learn about the cardiovascular implication for patients during the COVID-19 pandemic.
3. To become familiar with the different CT/MRI imaging characteristics of cardiac inflammation and its prognostic implications.

### Refresher Course: Chest

### RC 204

### Thoracic emergencies: part 2

**RC 204-1**
**A. Emergencies in thoracic oncology**
Cornelia M. Schaefer-Prokop; Amersfoort/NL

**Learning Objectives:**
1. To learn about various causes of dyspnoea in thoracic oncology.
2. To learn about cardiac complications of thoracic malignancies.
3. To learn how to suggest an appropriate differential diagnosis.

**RC 204-2**
**B. Haemoptysis**
Antoine Khalil; Paris/FR
*(antoine_khalil@yahoo.fr)*

**Learning Objectives:**
1. To understand the role of multidetector CT angiography.
2. To become familiar with the anatomy of bronchial arteries.
3. To learn how to optimise the planning of endovascular interventions.
**Author Disclosures:**
Antoine Khalil: Speaker: Guerbet Canon

**RC 204-3**
**C. Non-ischaemic cardiac emergencies**
Christian Loewe; Vienna/AT
*(christian.loewe@meduniwien.ac.at)*

**Learning Objectives:**
1. To learn how to evaluate acute pericarditis.
2. To learn how to explore patients with suspected myocarditis.
3. To learn about takotsubo features on MRIs.
**Author Disclosures:**
Christian Loewe: Author: BRACCO Springer; Speaker: Siemens Healthineers

**RC 204-4**
**D. Postoperative and iatrogenic complications**
Jonathan D. Dodd; Dublin/IE

**Learning Objectives:**
1. To learn about the complications after thoracic surgery.
2. To review the complications of percutaneous interventional radiology.
3. To review illustrative cases.

### Refresher Course: Chest

### RC 504

### Pleural disease revisited

**RC 504-1**
**A. Pleural plaques and mimickers**
Catherine Beigelman; Lausanne/CH

**Learning Objectives:**
1. To learn about the pathophysiology of pleural plaques.
2. To recognise typical findings and differentials on radiographs.
3. To recognise typical findings and differentials on CT.

**RC 504-2**
**B. Malignant mesothelioma**
Ieneke Hartmann; Rotterdam/NL
*(i.j.c.hartmann@gmail.com)*

**Learning Objectives:**
1. To learn when to suspect mesothelioma on radiographs.
2. To recognise imaging features highly suggestive of mesothelioma on CT.
3. To learn the role of PET-CT and MRI in diagnosing mesothelioma.

**RC 504-3**
**C. Other pleural neoplasms**
Ting Ting Zhang; Brighton/UK

**Learning Objectives:**
1. To learn how to differentiate pleural metastases from mesothelioma.
2. To learn how to recognise benign pleural tumours.
3. To learn about other rarer pleural malignancies.

### Refresher Course: Imaging Informatics

### RC 105

### How to manage data: moving patients' data across hospitals, regions, and countries

**RC 105-1**
**A. Transferring patients' data across hospitals, regions, and countries: pros and cons**
Elmar Kotter; Freiburg/DE
*(elmar.kotter@uniklinik-freiburg.de)*

**Learning Objectives:**
1. To learn the benefits and risks of patients' data sharing between centres.
2. To discuss the use of novel technologies allowing data sharing.
3. To understand the differences in data policy across hospitals and European countries.

**RC 105-2**
**B. Anonymised vs pseudo-anonymised data**
Timo De Bondt; Antwerp/BE
*(timo.debondt@gmail.com)*

**Learning Objectives:**
1. To learn basic general data protection regulation (GDPR) law concepts.
2. To appreciate the difference between anonymised and pseud-anonymised data.
3. To understand the relevance of anonymisation and pseudo-anonymisation for clinical studies.

**Author Disclosures:**
Timo De Bondt: Employee: Vinçotte - Controlatom

### RC 105-3
**C. Management of data for research projects and clinical trials**
Daniele Regge; Turin/IT
*(daniele.regge@ircc.it)*

**Learning Objectives:**
1. To learn how to design infrastructures for big projects and multi-centre trials.
2. To appreciate the different researcher roles linked to data in clinical studies.
3. To see practical examples of success stories in research projects.

**Author Disclosures:**
Daniele Regge: Consultant: Kardo, Radmetrix

### RC 105-4
**D. Federated learning: will artificial intelligence models travel to the images?**
Daniel Rubin; Stanford, CA/US

**Learning Objectives:**
1. To learn about the federated learning concept.
2. To discuss the benefits and risks of federated learning vs centralised repositories.
3. To observe the current technical implementations allowing for practical use of federated learning.

### Refresher Course: Hybrid, Molecular and Translational Imaging
*Organised by ESHIMT*

### RC 106
### Advancing clinical hybrid imaging

### RC 106-1
**A. Cost-effectiveness of hybrid imaging**
Barbara M. Fischer; London/UK
*(Malene.fischer@kcl.ac.uk)*

**Learning Objectives:**
1. To discuss why clinicians often consider hybrid imaging as the expensive choice.
2. To obtain knowledge of the basic concepts of health economy.
3. To present and discuss examples of cost-effectiveness studies in the use of PET/CT in oncology.
4. To evaluate how hybrid imaging may be incorporated as a cost-effective tool into routine clinical practice.

### RC 106-2
**B. PET/MRI in clinical routine**
Vicky J. Goh; London/UK
*(vicky.goh@kcl.ac.uk)*

**Learning Objectives:**
1. To learn about the basic concepts underlying PET/MRI.
2. To understand the potential clinical advantages of combining PET and MRI.
3. To discuss how PET/MRI may find a role as a routine clinical test in the future.

**Author Disclosures:**
Vicky J. Goh: Research Grant/Support: Siemens Healthcare; Speaker: ESOR

### RC 106-3
**C. PET/MRI in the context of radiation treatment**
Jiri Ferda; Plzen/CZ
*(ferda@fnplzen.cz)*

**Learning Objectives:**
1. To learn about the role of MRI and PET/MRI in radiation oncology.
2. To understand how MRI and/or PET/MRI can be used to monitor radiotherapy response.
3. To appreciate the role of hybrid imaging in targeting and modulating radiation therapy.

### Refresher Course: Genitourinary

### RC 107
### Imaging of benign female pelvis

### RC 107-1
**A. Benign ovarian lesion characterisation**
Rosemarie Forstner; Salzburg/AT
*(r.forstner@salk.at)*

**Learning Objectives:**
1. To understand the role of ultrasound and MRI in the characterisation of benign ovarian lesions.
2. To learn the imaging findings that allow the radiologist to recognise a mass of ovarian origin.
3. To become familiar with the different benign ovarian entities and different histological types of benign tumours.

### RC 107-2
**B. Unusual appearances of uterine leiomyomas**
Deniz Akata; Ankara/TR
*(dakata@hacettepe.edu.tr)*

**Learning Objectives:**
1. To understand the role of ultrasound and MRI in the characterisation of leiomyomas, as distinct from adenomyosis.
2. To become familiar with the different histological subtypes of leiomyomas and forms of degeneration.
3. To understand the role of MRI in distinguishing unusual leiomyomas from malignant entities.

### RC 107-3
**C. Congenital malformations of the uterus**
Cristina Maciel; Porto/PT
*(tina_maciel@yahoo.com)*

**Learning Objectives:**
1. To understand how to optimise MRI protocols for the work-up of female genital tract congenital anomalies.
2. To become familiar with the different classification of female genital tract congenital anomalies.
3. To learn how to present a reporting MRI checklist according to the specific malformation, complications and associated anomalies.

### Refresher Course: Genitourinary

### RC 207
### Imaging of recurrent gynaecological cancer

### RC 207-1
**A. Cervical and vulvar carcinoma post-therapy**
Lucia Manganaro; Rome/IT

**Learning Objectives:**
1. To become familiar with MRI expected pelvic changes after cervical and vulvar carcinoma treatment.
2. To be aware of the possible complications associated with treatment.
3. To learn the MRI features of recurrent cervical and vulvar carcinoma in the pelvis, in lymph nodes and of distant metastatic disease.

### RC 207-2
**B. Accessing recurrent endometrial cancer**
Milagros Otero García; Vigo/ES
*(milagros.otero.garcia@sergas.es)*

**Learning Objectives:**
1. To become familiar with MRI expected pelvic changes after endometrial carcinoma treatment.
2. To be aware of the possible complications associated with endometrial carcinoma treatment.
3. To learn the MRI features of recurrent endometrial carcinoma in the pelvis, in lymph nodes, and of distant metastatic disease.

### RC 207-3
**C. Recurrent ovarian cancer**
Andrea G. Rockall; London/UK

**Learning Objectives:**
1. To learn the indications and techniques for follow-up imaging in ovarian cancer.
2. To recognise the appearances and pitfalls of recurrent ovarian cancer on CT.
3. To become familiar with the appearances of recurrent ovarian cancer on MRI.

### Refresher Course: Head and Neck

### RC 108
### Imaging of 'glands' in the head and neck

### RC 108-1
**A. Lacrimal gland**
Ata Siddiqui; London/UK
*(ata.siddiqui@nhs.net)*

**Learning Objectives:**
1. To review the anatomy of the lacrimal glands and their structure.
2. To consider how best to image the lacrimal glands.
3. To become acquainted with common lacrimal gland pathology and imaging features.

### RC 108-2
**B. Salivary gland**
Roberto Maroldi; Brescia/IT
*(roberto.maroldi@unibs.it)*

**Learning Objectives:**
1. To become familiar with salivary gland anatomy and structure.
2. To learn how best to image these glands and indications for different modalities.
3. To review common salivary gland pathologies and their imaging features

### RC 108-3
**C. Thyroid ultrasound: solving the pitfalls**
Steve Colley; Birmingham/UK
*(Steve.Colley@uhb.nhs.uk)*

**Learning Objectives:**
1. To review the TI-RADS classification system.
2. To learn about pitfalls and issues associated with it.
3. To understand how to overcome these and develop logical imaging strategies.

### RC 108-4
**D. Parathyroid**
Jenny Hoang; Baltimore, MD/US
*(jennykh@gmail.com)*

**Learning Objectives:**
1. To review the anatomy and location of parathyroid glands.
2. To become familiar with available imaging modalities and understand which to use and when.
3. To learn about common pathologies and imaging features.

### Refresher Course: Head and Neck

### RC 308
### Imaging of head and neck trauma

### RC 308-1
**A. Facial trauma**
Elizabeth Loney; Halifax/UK

**Learning Objectives:**
1. To consider a simplified approach to reporting facial trauma scans.
2. To understand differing types of Le Fort fractures and key imaging review areas.
3. To become acquainted with mandibular fractures, types, and complications.

### RC 308-2
**B. Laryngeal trauma**
Minerva Becker; Geneva/CH

**Learning Objectives:**
1. To become familiar with the causes of laryngeal trauma and classification systems.
2. To learn how best to image the larynx in such situations and the challenges that can occur.
3. To review the imaging features of laryngeal trauma and consider its complications.

### RC 308-3
**C. Orbital trauma**
Pim De Graaf; Amsterdam/NL

**Learning Objectives:**
1. To review the relevant orbital anatomy.
2. To understand how best to image orbital trauma.
3. To consider the relevant imaging findings in the acute and chronic setting.

### RC 308-4
**D. Skull base trauma**
Stephen Connor; London/UK

**Learning Objectives:**
1. To briefly review the relevant skull base anatomy.
2. To understand the types and locations of traumatic injuries to the skull base.
3. To consider how to image such patients and how to review the imaging findings in acute trauma and of associated sequelae.

### Refresher Course: Head and Neck

### RC 408
### Granulomatous disease and its mimics in the head and neck

### RC 408-1
**A. Infectious granulomatous disease**
Salman Qureshi; Doha/QA

**Learning Objectives:**
1. To review the causes of infectious granulomatous disease.
2. To reflect on the available imaging modalities and to describe the pros and cons of each.
3. To become familiar with the imaging features of such conditions in the head and neck.

### RC 408-2
**B. Non-infectious granulomatous disease**
Soraya Robinson; Vienna/AT
*(s.robinson@dzu.at)*

**Learning Objectives:**
1. To become familiar with non-infectious granulomatous conditions in the head and neck.
2. To learn how best to image these conditions.
3. To review the imaging features of such pathologies.

**RC 408-3**
**C. Granulomatous disease mimics**
Burce Ozgen Mocan; Chicago, IL/US

**Learning Objectives:**
1. To consider the spectrum of diseases which may mimic granulomatous conditions.
2. To discuss the optimal imaging pathways.
3. To review the imaging appearances of such pathology and how reflect on how they might be differentiated from other conditions.

### Refresher Course: Musculoskeletal

### RC 210

### Musculoskeletal imaging in rheumatology

**RC 210-1**
**A. Crystal arthropathies**
Monique Reijnierse; Leiden/NL
*(m.reijnierse@lumc.nl)*

**Learning Objectives:**
1. To describe the imaging findings of crystal arthropathies.
2. To learn about differentials, choice of modalities, and possible clinical consequences of crystal arthropathies.

**RC 210-2**
**B. MRI of inflammatory diseases of the spine and sacroiliac joints**
Anne Cotten; Lille/FR
*(anne.cotten@chru-lille.fr)*

**Learning Objectives:**
1. To describe the MRI findings of inflammatory diseases in the axial skeleton.
2. To learn about differentials of inflammatory diseases of the axial skeleton.

**RC 210-3**
**C. Joint inflammation in children and adolescents**
Nele Herregods; Ghent/BE

**Learning Objectives:**
1. To explain the imaging and diagnostic considerations in inflammatory arthropathies in children and adolescents, including the choice of modalities.
2. To list the imaging findings and differentials in inflammatory arthropathies in children and adolescents.

### Refresher Course: Musculoskeletal

### RC 310

### The unstable joint

**RC 310-1**
**A. Shoulder instability**
Klaus Wörtler; Munich/DE
*(klaus.woertler@tum.de)*

**Learning Objectives:**
1. To describe the morphological and epidemiological aspects of shoulder instability.
2. To explain new trends in the imaging of shoulder instability.

**RC 310-2**
**B. Elbow instability**
Reto Sutter; Zurich/CH

**Learning Objectives:**
1. To describe the imaging features of acute elbow dislocations.
2. To explain the imaging hallmarks of chronic elbow instability.

**Author Disclosures:**
Reto Sutter: Author: Breitenseher Publisher; Other: Balgrist University Hospital has an academic research collaboration with Siemens Healthineers and Balzano Informatik.

**RC 310-3**
**C. Joint instability of the lower extremities**
Maria Tzalonikou; Athens/GR
*(tzalonikou3@yahoo.gr)*

**Learning Objectives:**
1. To explain the imaging findings of hip and knee dislocations.
2. To describe the imaging findings of ankle instability.

### Refresher Course: Musculoskeletal

### RC 410

### My top three tips for musculoskeletal ultrasound imaging

**RC 410-1**
**A. My top three tips for ultrasound of the shoulder**
Magdalena S. Posadzy; Poznan/PL
*(magdalenaposadzy@gmail.com)*

**Learning Objectives:**
1. To explain the utility of standardised views.
2. To describe how to recognise common pathologies.
3. To list pitfalls and explain how to avoid them.

**RC 410-2**
**B. My top three tips for ultrasound of the elbow**
Hussain Al-Bulushi; Muscat/OM
*(e_240@hotmail.com)*

**Learning Objectives:**
1. To explain the utility of standardised views.
2. To describe how to recognise common pathologies.
3. To list pitfalls and explain how to avoid them.

**RC 410-3**
**C. My top three tips for ultrasound of the wrist and hand**
Elena E. Drakonaki; Iraklion/GR
*(drakonaki@yahoo.gr)*

**Learning Objectives:**
1. To explain the utility of standardised views.
2. To describe how to recognise common pathologies.
3. To list pitfalls and explain how to avoid them.

**RC 410-4**
**D. My top three tips for ultrasound of the hip**
Emma Rowbotham; Leeds/UK
*(emmarowbotham@doctors.org.uk)*

**Learning Objectives:**
1. To explain the utility of standardised views.
2. To describe how to recognise common pathologies.
3. To list pitfalls and explain how to avoid them.

**RC 410-5**
**E. My top three tips for ultrasound of the knee**
Amanda Isaac; London/UK

**Learning Objectives:**
1. To explain the utility of standardised views.
2. To describe how to recognise common pathologies.
3. To list pitfalls and explain how to avoid them.

**RC 410-6**
**F. My top three tips for ultrasound of the ankle and foot**
Carlo Martinoli; Genoa/IT
*(55904@unige.it)*

**Learning Objectives:**
1. To explain the utility of standardised views.
2. To describe how to recognise common pathologies.
3. To list pitfalls and explain how to avoid them.

### Refresher Course: Musculoskeletal

### RC 510
### MRI of the knee

**RC 510-1**
**A. ACL injury: associated soft tissue injuries**
Patrick Omoumi; Lausanne/CH
*(patrick.omoumi@chuv.ch)*

**Learning Objectives:**
1. To explain the mechanism of an ACL tear, focusing on the associated soft tissue injuries to the menisci, posteromedial, and posterolateral corners.
2. To describe the MRI appearances of associated soft tissue injuries, their clinical importance, and potential pitfalls.

**RC 510-2**
**B. ACL repair: MRI of a normal graft and the complications**
Christian W.A. Pfirrmann; Zurich/CH

**Learning Objectives:**
1. To describe the normal anatomy and MRI appearances of an ACL repair by graft surgery.
2. To explain the imaging appearances of normal maturation and pathological conditions involving ligament repair.
3. To list the most valuable imaging findings that correlate with clinical complications.

**RC 510-3**
**C. Anterior knee pain**
P. Diana Afonso; Lisbon/PT
*(p.diana.a@gmail.com)*

**Learning Objectives:**
1. To describe the normal anatomy and MRI appearances of the anterior knee structures, including the extensor mechanism, fat pads, and synovial plicae.
2. To explain the imaging appearances of pathological conditions that present with anterior knee pain.

**RC 510-4**
**D. Image-guided interventions around the knee**
Silvia Mariani; L'Aquila/IT
*(mari.silvia@hotmail.it)*

**Learning Objectives:**
1. To describe the different imaging modalities that can be used and their advantages.
2. To explain different conditions that benefit from image-guided interventions around and within the knee.
3. To list which structures and mistakes to avoid.

### Refresher Course: Musculoskeletal

### RC 610
### Musculoskeletal tumours

**RC 610-1**
**A. Bone tumours and tumour-like conditions**
Paul O'Donnell; Stanmore/UK
*(paulodonnell@nhs.net)*

**Learning Objectives:**
1. To learn about the imaging findings of bone tumours.
2. To describe how to differentiate benign from malignant bone lesions.

**RC 610-2**
**B. Soft tissue tumours**
Alberto Bazzocchi; Bologna/IT
*(abazzo@inwind.it)*

**Learning Objectives:**
1. To explain the imaging findings of soft tissue tumours.
2. To describe differential diagnostic considerations in imaging soft tissue tumours.

**RC 610-3**
**C. Tumours of the spinal column**
Filip M.H.M. Vanhoenacker; Antwerp/BE
*(filip.vanhoenacker@telenet.be)*

**Learning Objectives:**
1. To explain the imaging findings of spinal tumours.
2. To describe differential diagnostic considerations in imaging spinal tumours.

### Refresher Course: Musculoskeletal

### RC 710
### Musculoskeletal infection

**RC 710-1**
**A. Imaging osteomyelitis: an update**
Jan Fritz; Baltimore, MD/US

**Learning Objectives:**
1. To describe the role of imaging in diagnosing osteomyelitis.
2. To explain the role of intravenous contrast medium material in diagnosing osteomyelitis.
3. To list the differential diagnostic considerations in imaging osteomyelitis.
**Author Disclosures:**
Jan Fritz: Advisory Board: Siemens, GE, Synthetic MR, ImageBiopsy Labs, Mirata Pharma; Grant Recipient: Siemens AG, BTG International, Microsoft Corporation, Zimmer Biomed, DePuy Synthes, Synthetic MRI, QED; Patent Holder: Siemens, Johns Hopkins

**RC 710-2**
**B. Soft tissue infections**
Silvia Martin; Palma de Mallorca/ES
*(silvia.m.martin@gmail.com)*

**Learning Objectives:**
1. To describe the role of imaging in soft tissue infections.
2. To explain the role of intravenous contrast medium material in diagnosing soft tissue infections.
3. To list the differential diagnostic considerations in imaging soft tissue infections.

**RC 710-3**
**C. Septic arthritis**
Radhesh Lalam; Oswestry/UK
*(radhesh.lalam@rjah.nhs.uk)*

**Learning Objectives:**
1. To describe the role of imaging in septic arthritis.
2. To explain the role of intravenous contrast medium material in diagnosing septic arthritis.
3. To list the differential diagnostic considerations in imaging septic arthritis.

### Refresher Course: Musculoskeletal

### RC 810

### Essential measurements and classifications in musculoskeletal joint imaging

**RC 810-1**
**A. Shoulder**
Üstün Aydingöz; Ankara/TR
*(uaydingo@hacettepe.edu.tr)*

**Learning Objectives:**
1. To describe essential measurements and classifications that need to be made in shoulder imaging.
2. To explain how these measurements/classifications relate to pathophysiology and management-related decision making.

**RC 810-2**
**B. Elbow-wrist-hand**
Milko C. De Jonge; Utrecht/NL
*(milkodejonge@gmail.com)*

**Learning Objectives:**
1. To describe the essential measurements and classifications that need to be made in elbow, wrist, and hand imaging.
2. To explain how these measurements/classifications relate to pathophysiology and management-related decision making.

**RC 810-3**
**C. Hip**
Florian Schmaranzer; Berne/CH
*(florian.schmaranzer@insel.ch)*

**Learning Objectives:**
1. To describe the essential measurements and classifications that need to be made in hip imaging.
2. To explain how these measurements/classifications relate to pathophysiology and management-related decision making.

**RC 810-4**
**D. Knee**
Christoph Rehnitz; Heidelberg/DE
*(Christoph.Rehnitz@med.uni-heidelberg.de)*

**Learning Objectives:**
1. To describe the essential measurements and classifications that need to be made in knee imaging.
2. To explain how these measurements/classifications relate to pathophysiology and management-related decision making.

**RC 810-5**
**E. Ankle-foot**
Richard Fawcett; Leeds/UK

**Learning Objectives:**
1. To describe essential measurements and classifications that need to be made in ankle and foot imaging.
2. To explain how these measurements/classifications relate to pathophysiology and management-related decision making.

### Refresher Course: Neuro

### RC 111

### Craniocervical junction

**RC 111-1**
**A. Congenital abnormalities**
Andrea Rossi; Genoa/IT
*(andrearossi@gaslini.org)*

**Learning Objectives:**
1. To become familiar with the congenital bone anomalies and variants of the craniocervical junction.
2. To learn about associated cerebellar and spinal cord abnormalities.
3. To illustrate craniocervical anomalies in syndromes.

**RC 111-2**
**B. Trauma**
Guillaume Bierry; Strasbourg/FR

**Learning Objectives:**
1. To be able to categorise the spectrum of fractures.
2. To develop an understanding of the biomechanics and management options.
3. To appreciate the imaging findings in ligamentous craniocervical injuries.

**RC 111-3**
**C. Systemic diseases and tumours**
Karl-Olof Løvblad; Geneva/CH
*(Karl-Olof.Lovblad@hcuge.ch)*

**Learning Objectives:**
1. To recognise craniocervical injuries in rheumatoid arthritis.
2. To differentiate systemic diseases from other pathology involving the craniocervical junction.
3. To become familiar with retro-odontoid pseudotumours without rheumatoid arthritis.

### Refresher Course: Neuro

### RC 211

### Sellar and parasellar lesions

**RC 211-1**
**A. Anatomy, variants, and function of the pituitary gland**
Seamus Looby; Dublin/IE

**Learning Objectives:**
1. To review the anatomy of the sellar and parasellar region.
2. To recognise sellar variants.
3. To be able to appropriately image the patient with diabetes insipidus.

**RC 211-2**
**B. Imaging of sellar and suprasellar lesions**
Zoran Rumboldt; Rovinj-Rovigno/HR
*(puz3@yahoo.com)*

**Learning Objectives:**
1. To recognise pituitary and sellar neoplasms other than adenoma.
2. To describe perisellar and suprasellar pathology.
3. To provide an overview of sellar and suprasellar childhood lesions.

**RC 211-3**
**C. Post-treatment findings and follow-up**
Fabrice Bonneville; Toulouse/FR
*(bonneville.f@chu-toulouse.fr)*

**Learning Objectives:**
1. To become familiar with the different types of pituitary adenoma.
2. To define the most effective imaging strategy for pituitary adenoma.
3. To learn about the role of MRI after surgical and/or medical treatment.

### Refresher Course: Neuro

### RC 311

### Neuromuscular imaging

**RC 311-1**
**A. How to image patients with neuromuscular disease**
Marc-André Weber; Rostock/DE
*(marc-andre.weber@med.uni-rostock.de)*

**Learning Objectives:**
1. To become aware of the recent developments in muscle imaging.
2. To understand the role of imaging in muscle atrophy.
3. To become familiar with the expectations of the neurologist.

**RC 311-2**
**B. How to report congenital myopathies**
Anna Pichiecchio; Pavia/IT
*(anna.pichiecchio@mondino.it)*

**Learning Objectives:**
1. To appreciate muscle MRI findings in neuromuscular disorders.
2. To learn about structured reporting in neuromuscular diseases.
3. To understand the role of various imaging modalities in the workup of congenital myopathies.

**Author Disclosures:**
Anna Pichiecchio: Advisory Board: Sanofi G-enzyme; Consultant: Sanofi G-enzyme (Prex)

**RC 311-3**
**C. How to recognise adult neuromuscular disease**
Sachit Shah; London/UK

**Learning Objectives:**
1. To learn how to apply the optimal imaging protocol.
2. To appreciate the role of quantitative imaging in genetic and acquired myopathies.
3. To illustrate the role of brain imaging in neuromuscular disorders.

### Refresher Course: Neuro

### RC 411

### Aging brain imaging: essential clues for diagnosis

**RC 411-1**
**A. Brain aging: what is normal?**
Meike Vernooij; Rotterdam/NL
*(m.vernooij@erasmusmc.nl)*

**Learning Objectives:**
1. To understand the importance for radiologists to have knowledge on the process of brain aging.
2. To learn what is 'normal' and what is considered 'abnormal' aging in brain imaging.
3. To illustrate how this knowledge can be incorporated into reading and reporting brain scans in clinical practice.

**RC 411-2**
**B. Imaging of abnormal cognitive deterioration: Alzheimer's and beyond**
Alexandre Krainik; Grenoble/FR

**Learning Objectives:**
1. To understand the relationships between imaging features and memory, executive, and speech impairments.
2. To learn how to recognise abnormal brain imaging features and patterns useful for clinical practice.
3. To provide essential clues for reporting atrophy, grey, and white matter abnormalities.

**RC 411-3**
**C. Imaging of abnormal motor deterioration: Parkinson's and beyond**
Sven Haller; Geneva/CH

**Learning Objectives:**
1. To understand the pathophysiology of the main extrapyramidal syndromes and neurodegenerative diseases.
2. To learn how to recognise related brain imaging abnormalities.
3. To provide essential clues for reporting and discussing differential diagnoses.

**Author Disclosures:**
Sven Haller: Advisory Board: EPAD; Consultant: WYSS, SPINEART; Speaker: GE

### Refresher Course: Neuro

### RC 611

### Cerebrospinal fluid (CSF) and glymphatic system imaging

**RC 611-1**
**A. How to identify Virchow-Robin spaces and glymphatic system disorders**
Nivedita Agarwal; Rovereto/IT
*(niveditaaga@gmail.com)*

**Learning Objectives:**
1. To understand the pathophysiological disorders of Virchow-Robin spaces.
2. To learn how to optimise glymphatic system imaging.
3. To be able to report enlarged perivascular spaces.

**RC 611-2**
**B. How to diagnose cerebrospinal fluid deficit**
Horst Urbach; Freiburg/DE
*(horst.urbach@uniklinik-freiburg.de)*

**Learning Objectives:**
1. To understand the pathophysiology of CSF production.
2. To recognise malformations of cortical development.
3. To learn how to report malformations of cortical development.

**RC 611-3**
**C. How to report cerebrospinal fluid excess**
Joanna Bladowska; Wroclaw/PL
*(joanna.bladowska@umed.wroc.pl)*

**Learning Objectives:**
1. To understand the pathophysiology of CSF circulation.
2. To be able to diagnose the different types of CSF excess.
3. To understand the relationships between CSF excess and intracranial hypertension.

### Refresher Course: Paediatric

### RC 112

### Learning from common errors and discrepancies in paediatric radiology

**RC 112-1**
**A. Pitfalls to avoid in musculoskeletal imaging**
Susan Cheng Shelmerdine; London/UK

**Learning Objectives:**
1. To understand how learning from mistakes improves clinical care.
2. To review common pitfalls in musculoskeletal imaging.
3. To learn how to avoid mistakes concerning bone marrow signal in children.

**RC 112-2**
**B. Pitfalls to avoid in chest imaging**
Thomas Robert Semple; London/UK
*(tsemple@doctors.org.uk)*

**Learning Objectives:**
1. To understand how learning from mistakes improves clinical care.
2. To review common pitfalls in chest imaging.
3. To learn how to avoid mistakes concerning lines and tubes.
**Author Disclosures:**
Thomas Robert Semple: Consultant: Boehringer-Ingelheim and Calyx; Research Grant/Support: Vertex and Chiesi pharmaceuticals; Speaker: Vertex pharmaceuticals

**RC 112-3**
**C. Pitfalls to avoid in paediatric abdominal imaging**
Magdalena Wozniak; Lublin/PL
*(mwozniak@hoga.pl)*

**Learning Objectives:**
1. To understand how learning from mistakes improves clinical care.
2. To review common pitfalls in abdominal imaging.
3. To learn how to avoid mistakes in patients with acute abdominal pain.

**RC 112-4**
**D. Pitfalls to avoid in neuroimaging**
Andrea Rossi; Genoa/IT
*(andrearossi@gaslini.org)*

**Learning Objectives:**
1. To understand how learning from mistakes improves clinical care.
2. To review common pitfalls in neuroimaging.
3. To learn how to avoid mistakes concerning ventricles and subarachnoid spaces.

### Refresher Course: Paediatric

### RC 212

### Imaging the small ones: tips and tricks in neonatal imaging

**RC 212-1**
**A. Neuroimaging in the neonate**
Elida Vázquez; Barcelona/ES
*(evazquez@vhebron.net)*

**Learning Objectives:**
1. To learn the most common neurological queries in neonatology.
2. To understand the technical challenges of US and MRI in neonatal imaging.
3. To discuss the key findings that may be useful for differential diagnosis.

**RC 212-2**
**B. Neonatal renal dilatation**
Jim Carmichael; London/UK

**Learning Objectives:**
1. To learn how to investigate antenatal renal dilatation.
2. To understand congenital abnormalities leading to renal dilatation and how to investigate them.
3. To present a protocol for investigation and follow-up of renal dilatation in the neonate.

**RC 212-3**
**C. Neonatal jaundice**
Helen Woodley; Leeds/UK
*(h.woodley@nhs.net)*

**Learning Objectives:**
1. To understand the aetiologies of neonatal jaundice such as biliary atresia.
2. To appreciate the key features for diagnosis.
3. To discuss appropriate management.

**RC 212-4**
**D. Imaging the neonatal bowel**
Anne M.J.B. Smets; Amsterdam/NL
*(a.m.smets@amsterdamumc.nl)*

**Learning Objectives:**
1. To recognise common bowel abnormalities in neonates on radiographs.
2. To learn how to use ultrasound to investigate necrotising enterocolitis and bowel perforation.
3. To discuss developmental bowel abnormalities.

**RC 212-5**
**E. Imaging lines and tubes in neonates**
Ola Kvist; Stockholm/SE
*(ola.kvist@sll.se)*

**Learning Objectives:**
1. To appreciate the normal course of lines and tubes in neonates on radiographs.
2. To learn how to use ultrasound to assess line position.
3. To discuss the complications of lines and tubes in neonates using radiographs and ultrasound.

### Refresher Course: Paediatric

### RC 412

### Imaging of key queries in children: an evidence-based approach

**RC 412-1**
**A. When a child is unusually short: work-up and the role of the paediatric radiologist**
Alistair D. Calder; London/UK
*(Alistair.Calder@gosh.nhs.uk)*

**Learning Objectives:**
1. To learn about possible causes of short stature.
2. To understand the indications for imaging and the typical radiological investigations.
3. To discuss a structured approach to diagnosis and how to seek help.

**RC 412-2**
**B. Respiratory tract infections**
Pierluigi Ciet; Rotterdam/NL
*(p.ciet@erasmusmc.nl)*

**Learning Objectives:**
1. To learn about the role of imaging in childhood respiratory tract infections.
2. To understand the indications and limitations of radiography, CT, and ultrasound.
3. To discuss the typical radiological findings and their clinical implications in a child with a respiratory tract infection.
**Author Disclosures:**
Pierluigi Ciet: Consultant: Vertex Pharmaceutical; Grant Recipient: ZonMW

**RC 412-3**
**C. Large and small heads: when and how to image**
Ümit Yaşar Ayaz; Mersin/TR
*(umityasarayaz@yahoo.com)*

**Learning Objectives:**
1. To learn the indications for imaging in deviating head circumference.
2. To understand examination techniques and imaging algorithms.
3. To appreciate the most common pathologies and their clinical implications.

**RC 412-4**
**D. The limping child**
Ignasi Barber; Esplugues de Llobregat/ES
*(ibarber@hsjdbcn.org)*

**Learning Objectives:**
1. To learn about the most important differential diagnosis in a limping child.
2. To understand the indications for imaging and choice of imaging modalities in a child with a limp.
3. To appreciate the most important findings and 'red flag' features.

### Refresher Course: Paediatric

### RC 512

### Polytrauma management

**RC 512-1**
**A. US, CT, and MRI in paediatric polytrauma: when and how to use?**
Maria Raissaki; Iraklion/GR
*(mraissaki@yahoo.gr)*

**Learning Objectives:**
1. To discuss the justification for imaging in paediatric polytrauma patients.
2. To learn about the imaging protocols and current guidelines in paediatric polytrauma management.
3. To discuss the potential applications and limitations of US, CT, and MRI in paediatric polytrauma patients.

**RC 512-2**
**B. Cerebral and spinal trauma in children: what the surgeon needs to know**
Stavros Stivaros; Manchester/UK

**Learning Objectives:**
1. To learn about the imaging patterns of cerebral and spinal trauma in children.
2. To discuss the indications for imaging in paediatric cerebral and spinal trauma.
3. To become familiar with basic and advanced imaging protocols.

**RC 512-3**
**C. Thoracic and abdominal trauma in children: what the surgeon needs to know**
Ilias Tsiflikas; Tübingen/DE
*(ilias.tsiflikas@med.uni-tuebingen.de)*

**Learning Objectives:**
1. To learn about the imaging patterns of thoracic and abdominal trauma in children.
2. To discuss the indications for imaging in paediatric thoracic and abdominal trauma.
3. To become familiar with basic and advanced imaging protocols.

### Refresher Course: Physics in Medical Imaging

### RC 413

### Radiation dose monitoring systems (RDMS): from commissioning to effective use

**RC 413-1**
**A. Supply and commissioning of an RDMS to meet all your needs**
Niki Fitousi; Leuven/BE
*(niki.fitousi@qaelum.com)*

**Learning Objectives:**
1. To understand how to select the right system for each hospital.
2. To learn how data should be validated after being collected.
3. To understand how derived quantities are calculated.
**Author Disclosures:**
Niki Fitousi: Employee: Qaelum NV

**RC 413-2**
**B. How to manage the data and extract the relevant information**
Osvaldo Rampado; Turin/IT
*(orampado@cittadellasalute.to.it)*

**Learning Objectives:**
1. To learn about the methods of data extraction.
2. To be able to query the database with different filters.
3. To understand how to use this data to guide optimisation actions.
**Author Disclosures:**
Osvaldo Rampado: Other: I'm a user of Physico (emme esse) radiation dose monitoring system

**RC 413-3**
**C. Monitoring and analysis of patients with high cumulative risks**
Jenia N. Vassileva; Vienna/AT
*(J.N.Vassileva@gmail.com)*

**Learning Objectives:**
1. To learn the strategies of defining attention levels and possible intervention thresholds.
2. To understand how to correlate cumulative dose indices with cumulative risks.
3. To evaluate which procedures and clinical pathways involve high cumulative risks.

### Refresher Course: Physics in Medical Imaging

### RC 513

### Quantification of magnetic resonance imaging parameters in clinical practice

**RC 513-1**
**A. Parameter mapping in MRI: acquisition methodologies**
Lars G. Hanson; Copenhagen/DK

**Learning Objectives:**
1. To learn about available acquisition methodologies for the mapping of MRI parameters: T1, T2, T2*, and magnetic susceptibility.
2. To understand the current limitations of different approaches in a clinical setting.
3. To be able to select suitable phantoms that assure reproducibility and accuracy when performing quantitative measures.

**RC 513-2**
**B. Towards optimisation and standardisation of MRI techniques for diffusion MRI of the body**
Alberto Torresin; Milan/IT
*(alberto.torresin@unimi.it)*

**Learning Objectives:**
1. To learn about diffusion MRI in body applications.
2. To understand how to perform quantitative diffusion MRI in body applications.
3. To evaluate the performance of different MRI techniques.

**RC 513-3**
**C. Quantitative, multi-parametric MRI as a useful biomarker in the clinic**
Ferdia A. Gallagher; Cambridge/UK
*(fag1000@cam.ac.uk)*

**Learning Objectives:**
1. To learn about the use of quantitative biomarkers in medical images.
2. To understand how quantification improves decision making in a clinical context.
3. To evaluate the open issues that need to be tackled to exploit such techniques fully.

**Author Disclosures:**
Ferdia A. Gallagher: Consultant: AstraZeneca; Grant Recipient: GSK; Research Grant/Support: GE Healthcare

### Refresher Course: Radiographers

### RC 214

### Imaging and advanced practice in radiotherapy

**RC 214-1**
**A. CT in radiotherapy**
Rafaela Guisantes; Coimbra/PT
*(rafaela_guis@hotmail.com)*

**Learning Objectives:**
1. To explain the specific radiotherapy requirements of CT simulation.
2. To understand the key principles of optimisation when performing CT simulation.
3. To be aware of the impact of patient preparation and positioning during CT simulation in the treatment planning process.

**RC 214-2**
**B. MRI in radiotherapy**
Vesna Mekis; Ljubljana/SI

**Learning Objectives:**
1. To understand the importance of MRI simulation in the treatment planning process.
2. To explain specific radiotherapy requirements of MRI simulation and to consider the role of the MRI linear accelerator.
3. To discuss future perspectives of MRI in radiotherapy.

**RC 214-3**
**C. Advanced practice in radiotherapy**
Sue Mercieca; Msida/MT
*(susan.mercieca@um.edu.mt)*

**Learning Objectives:**
1. To review the current advanced practice opportunities in radiotherapy.
2. To explore the potential opportunities for radiographers in radiotherapy practice.
3. To discuss the importance of education, training, and continuous professional development for radiographers specialising in radiotherapy.

### Refresher Course: Radiographers

### RC 314

### Developments and practice in ultrasound

**RC 314-1**
**A. Advances in ultrasound transducer technology**
Damir Suhonjic; Vienna/AT

**Learning Objectives:**
1. To become familiar with the latest technologies in ultrasound transducer technology.
2. To explore the consequences of different transducer technologies in examination protocols.
3. To explore the use of the latest technologies in ultrasound in daily routine practice.

**RC 314-2**
**B. Technical quality management in ultrasound**
Saso Arnuga; Ljubljana/SI
*(saso.arnuga@zf.uni-lj.si)*

**Learning Objectives:**
1. To become familiar with technical quality management in ultrasound.
2. To outline the benefits of technical quality management in ultrasound.
3. To identify challenges and opportunities for radiographers/sonographers in implementing technical quality management.

**RC 314-3**
**C. Perspectives of radiographers performing ultrasound**
Gill Harrison; London/UK

**Learning Objectives:**
1. To provide an overview of the radiographer/sonographer role in ultrasound.
2. To recognise the barriers to the successful implementation of radiographers performing ultrasound.
3. To explain the specific requirements of radiographers performing ultrasound.

**Author Disclosures:**
Gill Harrison: Investigator: Chair of an ultrasound survey working group for the European Federation of Radiographer Societies

### Refresher Course: Radiographers

### RC 514

### Updates in forensic imaging

**RC 514-1**
**A. An overview of best practice for radiographers in forensic imaging**
Jonathan McNulty; Dublin/IE
*(jonathan.mcnulty@ucd.ie)*

**Learning Objectives:**
1. To understand the key aspects of the radiographers' role in forensic imaging.
2. To discuss the importance of the continuity of evidence and record keeping during forensic examinations.
3. To highlight other elements of current best-practice guidelines.

**RC 514-2**
**B. Paediatric forensic imaging**
Niamh Kirk; Belfast/UK
*(niamh.kirk@belfasttrust.hscni.net)*

**Learning Objectives:**
1. To appreciate the role of multi-modality imaging in paediatric forensic cases.
2. To become familiar with the current guidelines on imaging in cases of suspected physical abuse/non-accidental injuries.
3. To recognise the potential challenges in obtaining cooperation from relatives.

**RC 514-3**
**C. Optimising post-mortem CT examinations and protocols**
Jeroen Kroll; Maastricht/NL
*(j.kroll@mumc.nl)*

**Learning Objectives:**
1. To explore the range of applications for CT in forensic imaging.
2. To evaluate the approaches to the optimisation of workflow in post-mortem CT.
3. To discuss tips and tricks to optimise your protocols for a range of forensic applications.

### Refresher Course: Vascular

### RC 115

### Venous thrombotic disease

**RC 115-1**
**A. Acute deep vein thrombosis: diagnosis, interventional radiology (IR) treatment, and outcomes**
Geert Maleux; Leuven/BE
*(Geert.Maleux@uzleuven.be)*

**Learning Objectives:**
1. To learn about the clinical symptoms and diagnosis of deep vein thrombosis (DVT).
2. To become familiar with the different conservative and IR treatment options.
3. To discuss the short-, mid-, and long-term outcomes.
**Author Disclosures:**
Geert Maleux: Speaker: Terumo, Sirtex, Boston Scientific; Other: proctor Terumo & Sirtex

**RC 115-2**
**B. Diagnosis and treatment of central venous occlusions**
Salah D. Qanadli; Lausanne/CH

**Learning Objectives:**
1. To learn about clinical symptoms and the appropriate diagnostic tools.
2. To become familiar with which patients IR treatment is indicated.
3. To understand how to treat a central venous occlusion using IR methods.

**RC 115-3**
**C. Chronic deep vein thrombosis: diagnosis, IR treatment, and outcomes**
Romaric Loffroy; Dijon/FR
*(romaric.loffroy@chu-dijon.fr)*

**Learning Objectives:**
1. To learn about the clinical symptoms and diagnosis of chronic DVT.
2. To become familiar with preinterventional imaging and appropriate treatment planning.
3. To discuss different IR techniques and outcomes.

### Refresher Course: Oncologic Imaging

### RC 116

### Peritoneal carcinomatosis: the role of imaging in detection and treatment planning?

**RC 116-1**
**A. Detection and characterisation: tips and tricks**
Laure S. Fournier; Paris/FR

**Learning Objectives:**
1. To become familiar with imaging strategies for the assessment of peritoneal carcinomatosis.
2. To discuss multimodal concepts for detection and characterisation.
3. To learn about the limitations of non-invasive imaging approaches.

**Author Disclosures:**
Laure S. Fournier: Research Grant/Support: Invectys, Novartis, Philips; Speaker: Sanofi, Novartis, Jannssen, General Electric; Other: congress sponsorship Guerbet

**RC 116-2**
**B. Surgical view: cytoreductive surgery**
Anne-Sophie Bats; Paris/FR
*(anne-sophie.bats@aphp.fr)*

**Learning Objectives:**
1. To understand the surgeon's view on imaging strategies.
2. To learn about surgical strategies for the treatment of peritoneal carcinomatosis.
3. To highlight the relevant information from imaging for decision making.

**RC 116-3**
**C. Imaging in treatment planning and follow-up**
Giulia Zamboni; Verona/IT
*(gzamboni@hotmail.com)*

**Learning Objectives:**
1. To become familiar with the role of imaging for systemic and local treatment planning.
2. To discuss the value of imaging modalities.
3. To understand the limitations and pitfalls of imaging.

### Refresher Course: Oncologic Imaging

### RC 316

### Role of imaging in immunotherapy: tumour response assessment and complications

**RC 316-1**
**A. What is immunotherapy and (how) does it work?**
Clarisse Dromain; Lausanne/CH
*(Clarisse.Dromain@chuv.ch)*

**Learning Objectives:**
1. To discuss the concept of immunotherapy treatment in cancer.
2. To learn the different types of immunotherapies.
3. To understand the challenges of assessing immunotherapy response.
**Author Disclosures:**
Clarisse Dromain: Advisory Board: IPSEN

**RC 316-2**
**B. Challenges in measuring immunotherapy response**
Thorsten Persigehl; Cologne/DE
*(thorsten.persigehl@uk-koeln.de)*

**Learning Objectives:**
1. To understand the limitations of RECIST and to become aware of the immune response criteria.
2. To learn about the specifics of immune-related RECIST.
3. To become familiar with pseudoprogression and hyperprogression.
**Author Disclosures:**
Thorsten Persigehl: Advisory Board: BMS; Author: Review; Speaker: MINT, Philips, Siemens, BMS, BioNtech, Roche, Bayer

**RC 316-3**
**C. Functional and molecular imaging: FDG and beyond**
Vicky J. Goh; London/UK
*(vicky.goh@kcl.ac.uk)*

**Learning Objectives:**
1. To understand the current role of [18F]FDG-PET in immunotherapy response assessment.
2. To become familiar with the concepts of Immuno-PET.
3. To learn the applications of novel Immuno-PET tracers.
**Author Disclosures:**
Vicky J. Goh: Research Grant/Support: Siemens Healthcare

**RC 316-4**
**D. Complications of immunotherapy**
Oliver Lukas Sedlaczek; Heidelberg/DE

**Learning Objectives:**
1. To learn which immunotherapies frequently lead to complications.
2. To become familiar with which life-threatening complicationscan be caused by immunotherapies.
3. To learn when immunotherapy-related complications commonly occur.

### Refresher Course: Emergency Imaging

### RC 117

### Acute neurological presentations

**RC 117-1**
**A. Acute central nervous system (CNS) infections**
Maureen Dumba; London/UK

**Learning Objectives:**
1. To learn about imaging preferences in the setting of acute CNS infections.
2. To become familiar with the imaging features of the various CNS infections.
3. To understand the role of emergency radiology in the diagnosis and follow-up of patients with acute CNS infections.
**Author Disclosures:**
Maureen Dumba: Board Member: ESER and BSER; Employee: UCL, London

**RC 117-2**
**B. Usual and unusual imaging findings of stroke patient**
Cem Calli; Izmir/TR
*(cem.calli@gmail.com)*

**Learning Objectives:**
1. To learn about the common imaging findings of ischaemic and haemorrhagic stroke.
2. To understand the arterial and venous causative mechanisms and become familiar with unusual imaging findings to prevent missed stroke cases.
3. To understand the 'time is brain approach' in the imaging evaluation of patients with stroke.

**RC 117-3**
**C. Neurological emergency imaging: which sequence and when?**
Danielle Byrne; Dublin/IE
*(dbyrne.radiology@gmail.com)*

**Learning Objectives:**
1. To understand the terminology.
2. To appreciate the rationale and value of the different imaging modalities and sequences available for acute neurology.
3. To understand which imaging modality and sequence is most useful in each scenario.

### Refresher Course: Emergency Imaging

### RC 217

### Chest emergencies

**RC 217-1**
**A. Imaging of acute chest pain**
Paola Franchi; Teramo/IT
*(paegiodi@hotmail.com)*

**Learning Objectives:**
1. To learn how acute chest pain should be assessed in emergency imaging.
2. To learn how to optimise the CT protocols.
3. To understand the main pitfalls in the imaging assessment of acute chest pain.

**RC 217-2**
**B. Infection in the lung: the role of the emergency radiologist in the management of the patient**
Mariano Scaglione; Castel Volturno/IT

**Learning Objectives:**
1. To differentiate lung infection from other lung pathologies.
2. To learn about specific imaging findings of different infectious processes in the lung.
3. To learn how to differentiate bacterial, viral, and fungal infections.

**RC 217-3**
**C. Acute aortic syndrome: from imaging to management**
Ferco Henricus Berger; Toronto, ON/CA
*(fhberger@gmail.com)*

**Learning Objectives:**
1. To understand the different forms of acute aortic syndrome, their prognosis, and potential treatments.
2. To distinguish the high-risk features of acute aortic syndromes.
3. To learn about proper imaging protocol selection.

### Refresher Course: Emergency Imaging

### RC 317

### New technologies in emergency imaging

**RC 317-1**
**A. New frontiers in emergency radiology: better imaging, less radiation - how to optimise dose reduction**
Mehmet Ruhi Onur; Ankara/TR
*(ruhionur@yahoo.com)*

**Learning Objectives:**
1. To describe radiation limiting strategies (including noise reducing image reconstruction algorithms, automatic tube modulation, optimum z-axis coverage) and their impact on image quality in emergency CT.
2. To explain the effect of CT scanning parameters on radiation dose and image quality and to illustrate specific radiation dose output decreasing strategies.
3. To assess the importance of CT dose monitoring and workflow by dose management systems and to be familiar with diagnostic reference levels (DRL) of CT examinations.
4. To suggest consistent solutions to common problems in emergency CT that increase radiation dose and decrease image quality.

**RC 317-2**
**B. Postprocessing CT programmes (ASPECT, cerebral blood flow imaging, cardiac function assessment programmes, lung analysis, and cinematic rendering)**
Steven P. Rowe; Baltimore, MD/US
*(srowe8@jhmi.edu)*

**Learning Objectives:**
1. To become familiar with the novel postprocessing CT imaging techniques and their contributions in the diagnosis of emergency settings, and their effect on patient management.
2. To understand the trade off between time taken for a post processing technique vs the clinical benefit in predicting the prognosis.
3. To be able to explain how techniques such as cinematic rendering can help patients and clinical staff to understand radiological findings more clearly.
**Author Disclosures:**
Steven P. Rowe: Consultant: Progenics Pharmaceuticals; Founder: Precision Molecular, Inc., PlenaryAI, Inc.; Grant Recipient: Progenics Pharmaceuticals; Patent Holder: Precision Molecular, Inc., PlenaryAI, Inc.; Share holder: Precision Molecular, Inc., PlenaryAI, Inc.

**RC 317-3**
**C. Role of dual-energy CT in bone injuries**
Hatem Alkadhi; Zurich/CH

**Learning Objectives:**
1. To review the basic technical principles of dual-energy CT for bone imaging.
2. To understand the utility of dual-energy CT in bone injury evaluation in terms of management of the patients.
3. To learn the advantages and shortcomings of dual-energy CT in patients with skeletal disease.

### RC 317-4

**D. Utility of the clinical decision support system (CDS) in emergency radiology**

Alexander Goehler; Essen/DE
*(agoehler@post.harvard.edu)*

**Learning Objectives:**
1. To learn what a CDS is and how it works.
2. To understand how a CDS can help clinicians to eliminate inappropriate investigations or procedures and adhere to the latest practice guidelines.
3. To recognise the concerns that a CDS may constrain clinical management and understand the benefits a good CDS can bring to the individual patient pathway.

### Research Presentation Session: Abdominal Viscera

### RPS 201a

### Infection and inflammation in the abdomen

### RPS 201a-1

**Relationship between obesity and Covid-19: could an abdominal CT scan predict disease severity?**

*V. Rizzo*[1], F. Pediconi[1], S. Schiaffino[2], A. Cozzi[2], F. Galati[1], C. Catalano[1], F. Sardanelli[2]; [1]Roma/IT, [2]Milan/IT
*(nican66@gmail.com)*

**Purpose:** The aim of our study is to investigate the relationship between Covid-19 and obesity, to document the role of different fat distribution in the severity of lung disease and in the admission in intensive care units.
**Methods or Background:** In a retrospective multicenter study, we examined clinical features, the need of mechanical ventilation and admission in ICU for SARS-CoV-2, the severity of lung disease analyzed by a chest CT and the evaluation of obesity estimating visceral and subcutaneous adipose tissue by an abdominal CT scan. Lung involvement was evaluated calculating the percentage of affected parenchyma. Obesity degree was classified by evaluation of visceral adipose tissue area (VAT area) and subcutaneous adipose tissue area (SAT area) at third lumbar vertebra (L3) lower margin. Lung score with VAT and SAT were compared between patients with and without ICU admission using the Mann–Whitney U test, while multivariate binary logistic regression was used to evaluate the association between VAT area, VAT score, and SAT areas, in predicting ICU admission.
**Results or Findings:** Multivariate binary logistic regression identified VAT score as best predictor of ICU admission (odds ratio 4.307, 95%CI 0.709–26.153) followed by sex (OR 2.783, 95%CI 0.378–20.517) and pulmonary score (OR 1.253, 95%CI 0.982–1.598), with a resulting AUC of 0.821 ($p<0.001$).
**Conclusion:** Our work suggests that a high VAT increase the risk of ICU admission for COVID-19, due to its action of promoting chronic inflammation and probably because the adipose tissue can be a viral reservoir. Obese patients infected by SARS-CoV-2 should be considered a high-risk class and could be considered to extend chest CT scan to the L3 level, to perform a quantification of visceral adipose tissue to predict the risk of dangerous complications.
**Limitations:** Not applicable
**Ethics Committee Approval:** Not applicable
**Funding for this study:** Not applicable
**Author Disclosures:**
Andrea Cozzi: Nothing to disclose
Veronica Rizzo: Nothing to disclose
Francesco Sardanelli: Nothing to disclose
Simone Schiaffino: Nothing to disclose
Francesca Galati: Nothing to disclose
Federica Pediconi: Nothing to disclose
Carlo Catalano: Nothing to disclose

### RPS 201a-2

**2D shear wave elastography (SWE) performance versus vibration-controlled transient elastography (VCTE/fibroscan) in the assessment of liver stiffness in chronic hepatitis**

*A. M. Osman*, A. El Shimy, M. M. Abd El Aziz; Cairo/EG
*(Dr_osman80@yahoo.com)*

**Purpose:** Assessment of liver stiffness and fibrosis affects the management strategy. Multiple non-invasive tools are now available to offer an adequate alternative to biopsy.
**Methods or Background:** We compared the performance of 2D shear wave elastography (SWE) to the transient elastography/fibroscan as a non-invasive tool in the prediction of liver stiffness. This is a prospective study of 215 patients confirmed by serology to have positive virus C or B infection. 2D SWE was done followed by vibration controlled transient elastography (VCTE) known as fibroscan at the same session. Biopsy results were collected.
**Results or Findings:** The mean age was 51.07 years ± 6.07 SD. 5 cases were excluded due to insufficient data. Fibroscan failed in 30 cases out of 210 cases (failure rate of 14.3%) compared to only 12 patients (6.7% failure rate) while using SWE. Only 180 patients completed the study to the result analysis. SWE results showed significant agreement to the fibroscan results with 86.7% agreement with a tendency for overestimation of the degree of fibrosis (11.7%). The efficacy of SWE was highest during the assessment of patients with F0 (98.9%), F1 (97.8%) and F4 (93.3%) respectively and relatively low in F2 (92.8%) and F3 (90.6%).
**Conclusion:** 2D SWE is a relatively recent non-invasive tool in the assessment of liver fibrosis which can be used as an alternative to the fibroscan with almost similar diagnostic performance when fibroscan is not capable to obtain adequate results such as in obesity and ascites.
**Limitations:** Absent control group due to the difficulty to get a biopsy from a healthy person. US is operator dependent and we didn't assess the inter-operator variability.
**Ethics Committee Approval:** Faculty of Medicine- Ain Shams University Ethical Committee with informed written consents were taken from all patients.
**Funding for this study:** Not available
**Author Disclosures:**
Ahmed El Shimy: nothing to disclose
Mohamed M. Abd El Aziz: Nothing to disclose
Ahmed M. Osman: Nothing to disclose

### RPS 201a-3

**Quantitative edge analysis of pancreatic margins in patients with chronic pancreatitis: a correlation with exocrine function**

*A. Grecchi*, M. C. Ambrosetti, A. Ambrosetti, A. Amodio, G. Zamboni, G. Mansueto; Verona/IT
*(annamaria.grecchi88@gmail.com)*

**Purpose:** To correlate the computer-aided analysis of pancreatic margins with exocrine function measured with fecal elastase values in patients with chronic pancreatitis (CP).
**Methods or Background:** The hospital registries were searched for patients with confirmed diagnoses of CP with fecal elastase values and abdomen MRI performed in our Institute in less than 1 year. We identified 123 Patients divided into 3 groups of 41 patients each based on the fecal elastase value: group A with fecal elastase <100 µg/g; group B with fecal elastase between 100 and 200 µg/g; group C with fecal elastase > 200 µg/g. An in-house software was developed and computer-assisted quantitative edge-analysis was performed on pancreatic margins in Fat Suppressed T1-Weighted images of the pancreas with chronic inflammation changes, obtaining the root mean square deviation SD of the actual border from the average boundary line. Border SD values were compared across groups using the Kruskal-Wallis test and correlation between border SD values and fecal elastase values were tested with the Spearman test.
**Results or Findings:** A significant difference in SD values was observed between the three groups ($p<0.0001$). A significant correlation was observed between SD values and elastase values with $\rho$ =0.5945.
**Conclusion:** Quantitative edge-analysis with dedicated software may stratify Patients with chronic pancreatitis according to the degree of exocrine insufficiency, potentially contributing to the morphological and functional staging of this pathology.
**Limitations:** Small number of cases
**Ethics Committee Approval:** Formal IRB approval not required for retrospective studies
**Funding for this study:** None
**Author Disclosures:**
Giulia Zamboni: Nothing to disclose
Giancarlo Mansueto: Nothing to disclose
Antonio Amodio: Nothing to disclose
Annamaria Grecchi: Nothing to disclose
Maria Chiara Ambrosetti: Nothing to disclose
Ambrosetti Alberto Ambrosetti: Nothing to disclose

### RPS 201a-4

**Timing of CT scanning in acute pancreatitis: failure to follow guidelines supported by evidence**

*C. Banziger*[1], M. Sharifpour[2], A. Ali[1], I. Zealley[1]; [1]Dundee/UK, [2]Leicester/UK
*(carina.banziger@nhs.scot)*

**Purpose:** Initial management of acute pancreatitis is supportive. Current guidelines recommend a delay of up to 6 days (and at least 3 days) before performing CT in patients with a clinical/biochemical diagnosis of acute pancreatitis (AP). In order to see if evidence leads to a change in practice we have repeated a previous study performed at our institution (Dobbs, 2016) which provided evidence supporting these guidelines: CT scans performed at <6 days did not lead to any surgical or radiological interventions.
**Methods or Background:** Single centre retrospective cohort study comparing intervention rates precipitated by early CT (<6 day of admission) and CT timing per current guidelines (≥6 days of admission) in patients with a clinical/biochemical diagnosis of AP from October 2016 to April 2019.
**Results or Findings:** 229 patients fulfilled the inclusion criteria, 179 patients (78%) were scanned at <6 days and 50 patients (22%) at ≥6 days. One intervention was performed on the basis of an ear-ly CT scan (<6 days). The annualised rate of 'first' CT scan has increased from 61 per year to 87 per year. The proportion of 'early' CT scan has increased from 35% to 51%. The frequency of intervention has reduced from 9% to 2%.
**Conclusion:** Only one intervention precipitated an early CT scan. Despite evidence from our previous study, our rate of early CT scans has increased from 35% to 51%. These changes suggest a reduced threshold for CT scanning with no corresponding increase in rate of therapeutic interventions. The change in practice has not resulted in an increase in the rate of targeted therapeutic interventions which improve outcomes in AP.
**Limitations:** The timescale recommendations for initial CT in acute pancreatitis vary (between guide-lines from 3-6 days). However this study has utilised the longest recommended delay and still found that patient management was not altered by early CT scans.
**Ethics Committee Approval:** Approved by Caldicott Guardian
**Funding for this study:** None
**Author Disclosures:**
Adnan Ali: Nothing to disclose
Meedya Sharifpour: Nothing to disclose
Carina Banziger: Nothing to disclose
Ian Zealley: Nothing to disclose

### RPS 201a-5

**The diagnostic value of dual-layer spectral detector CT in negative gallbladder stones**

*J. Bai*, Y. Qiao; Taiyuan/CN
*(bjq19834516901@163.com)*

**Purpose:** To investigate the diagnostic value of dual-layer spectral detector CT in negative gallbladder stones.
**Methods or Background:** From June 2019 to July 2020, 63 patients with negative gallbladder stones were collected from the First Hospital of Shanxi Medical University and were underwent enhanced upper abdominal CT scanning with dual-layer spectral detector CT. The TNC images, the effective atomic number images, monochromatic energy images (40keV—200keV) and the VNC images were gained in the post-processing workstation. The CT attenuation of negative stones and the adjacent bile in the monochromatic energy images as well as the effective atomic number of images were measured, and the independent sample t-test was used for statistical analysis. The contrast noise ratio (CNR) of bile and negative stones in TNC and VNC were calculated. The paired sample t-test was used for statistical analysis. Receiver Operating Curve (ROC) was used to assess the accuracy of the effective atomic number in differentiating stones from bile.
**Results or Findings:** The CT values of gallbladder negative stones are different from that of bile in the monochromatic energy images (40keV and 200keV). The effective atomic number of negative stones (6.74±0.41) was significantly lower than bile (7.22±0.18). The CNR of gallbladder negative stones and bile in the VNC and the TNC were 1.10±0.43 and 0.44±0.34 respectively ($P < 0.01$). Taking 7.11 as the cut-off point of the effective atomic number, its specificity and sensitivity were 83.92% and 82.32% respectively, and the area-under-curve AUC was 0.90.
**Conclusion:** The monochromatic energy images (40keV, 200keV), effective atomic number images and VNC obtained by dual-layer spectral detector CT have high value in the diagnosis of negative stones, and improve the detection rate of negative gallbladder stones.
**Limitations:** Not applicable
**Ethics Committee Approval:** Not applicable
**Funding for this study:** Not applicable
**Author Disclosures:**
Jingqi Bai: Nothing to disclose
Ying Qiao: Nothing to disclose

### RPS 201a-6

**Shear wave elastography of the liver in correlation with sustained virological response in patients with chronic hepatitis C: a prospective follow-up study**

*A. D. Rónaszéki*, B. K. Budai, B. Csongrády, V. Orban, A. Folhoffer, F. Szalay, G. Györi, P. N. Kaposi-Novák; Budapest/HU
*(ronaszeki.aladar@gmail.com)*

**Purpose:** The purpose of our study was to observe that direct-acting antiviral (DAA) therapy has significant effect on serum biomarkers and liver stiffness values (LS).
**Methods or Background:** 35 individuals with chronic hepatitis-C infection and attained sustained virological response (SVR) after antiviral therapy were enrolled. 32 patients received DAAs and three patients were treated with interferon-based regimens. The LS was measured with shear-wave elastography (SWE) at the beginning of DAA treatment and at 48 weeks after the end of treatment (EOT48). The fibrosis-4 (FIB-4) scores were calculated based on laboratory tests. The METAVIR score and the varices needing treatment (VNT) were determined based on the LS values. The clinical variables between pre- and post-treatment groups were compared with Fisher's exact test and Student's t-test.
**Results or Findings:** There was a significant difference between the baseline LS (mean±standard deviation=2.59±0.89 m/s) and LS at EOT48 (1.90±0.50m/s; $P<0.001$). Compared to the baseline status, the METAVIR score also showed significant improvement after successful antiviral therapy (F0/1=2, F2=1, F3=7, F4=25; $P<0.028$ vs. F0/1=9, F2=2, F3=10, F4=14). Moreover, the FIB4 score also indicated less fibrosis after therapy (2.04±1.12) than at baseline (3.51±2.24; $P<0.018$). Meanwhile, there was a significant decrease in the number of patients with high-risk of VNT at EOT48 vs. at baseline (4 vs. 15; OR=0.17 95% confidence interval (CI)=0.05-0.59, $P<0.007$).
**Conclusion:** In conclusion, SWE indicates significant resolution of liver fibrosis when chronic hepatitis C patients are in SVR, which also coincides with lower risk of VNT.
**Limitations:** The limitation was the low members of patients. Longer post-treatment follow-up is needed to fully understand the clinical impact of the present findings.
**Ethics Committee Approval:** The study has been approved by the institutional ethics committee of our university (SE-TUKEB 163/2017).
**Funding for this study:** The authors received no funding for this study.
**Author Disclosures:**
Vince Orban: Nothing to disclose
Anikó Folhoffer: Nothing to disclose
Pál N. Kaposi-Novák: Nothing to disclose
Barbara Csongrády: Nothing to disclose
Aladár David Rónaszéki: Nothing to disclose
Gabriella Györi: Nothing to disclose
Bettina Katalin Budai: Nothing to disclose
Ferenc Szalay: Nothing to disclose

### Research Presentation Session: Abdominal Viscera

### RPS 301a

### Abdominal diagnosis and imaging

### RPS 301a-1

**Iterative denoising and image enhancement in dynamic contrast-enhanced gradient echo Magnetic Resonance Imaging (MRI) of the abdomen: improvement of image quality and diagnostic confidence**

*S. Gassenmaier*[1], J. Herrmann[1], D. Nickel[2], S. Kannengiesser[2], S. Afat[1], A. Othman[1]; [1]Tübingen/DE, [2]Erlangen/DE

**Purpose:** To investigate the impact of a novel iterative denoising and image enhancement technique in T1-weighted dynamic contrast-enhanced (DCE) gradient echo MRI of the abdomen on image quality, noise levels, diagnostic confidence, and lesion detectability.
**Methods or Background:** Fifty patients who underwent an MRI with DCE imaging of the abdomen between June and August 2020 were included. For DCE imaging a series of three volume interpolated breath-hold examinations (VIBE) was performed. The raw data of all DCE imaging studies was processed using standard reconstruction (DCES) and using an iterative denoising and image enhancement approach (DCEDE). All imaging studies were randomly reviewed by two radiologists independently regarding noise levels, arterial contrast, sharpness of vessels, overall image quality, lesion detectability, and diagnostic confidence using a Likert-scale ranging from 1 – 4 with four being the best.
**Results or Findings:** Arterial contrast and sharpness of vessels were rated superior by both readers with a median of 4 in DCEDE versus a median of 3 in DCES (p<0.001). Furthermore, noise levels as well as overall image quality were rated higher with a median of 4 in DCEDE compared to a median of 3 in DCES (p<0.001). Lesion detectability was evaluated to be superior in DCEDE with a median of 4 versus DCES with a median of 3 (p<0.001). Consequently, diagnostic confidence was also rated to be superior in DCEDE with a median of 4 versus DCES with a median of 3 (p<0.001).
**Conclusion:** Iterative denoising and image enhancement is feasible in DCE imaging of the abdomen providing superior arterial contrast, noise levels, and overall image quality. Furthermore, lesion detectability and diagnostic confidence were significantly improved using this reconstruction method.
**Limitations:** Retrospective study design. No quantitative SNR measurements were possible.
**Ethics Committee Approval:** IRB approval obtained.
**Funding for this study:** None.
**Author Disclosures:**
Judith Herrmann: Nothing to disclose
Stephan Kannengiesser: Employee: Siemens Healthcare GmbH
Saif Afat: Nothing to disclose
Dominik Nickel: Employee: Siemens Healthcare Gmbh
Ahmed Othman: Nothing to disclose
Sebastian Gassenmaier: Nothing to disclose

### RPS 301a-2

**Acoustic radiation force impulse (ARFI) elastography for the pancreatic allograft: looking for normal reference values**

*C. Bassaganyas Vancells*, A. Darnell, J. C. Soler Perromat, A. Soler, P. Ventura-Aguiar, M. Cuatrecasas, M. T. Rodrigo, J. Ferrer, A. Garcia Criado; Barcelona/ES
*(bassaganyas@clinic.cat)*

**Purpose:** Acoustic radiation force impulse (ARFI) quantification is a non-invasive ultrasonographic technique that allows quantification of the tissue elasticity. It has been studied in the native pancreas although no reports in pancreatic allograft have been published. The purpose of this study is to determine normal reference ARFI values in pancreatic grafts and its variations over time.
**Methods or Background:** Prospective study including all pancreas transplants performed in our centre between October 2016 and January 2020. All pancreatic allografts were evaluated by ARFI at 1 week, 3 weeks and 12 months after surgery. A protocol allograft biopsy was performed immediately after ARFI evaluation at 3 weeks and 12 months post-transplantation. Besides, ARFI quantification was also performed whenever a biopsy was done. Patients with postoperative complications or rejection were excluded from the analysis.
**Results or Findings:** During this period, 120 ARFI evaluations were performed in 44 patients. Three patients had postoperative complications at the time of the evaluation (5 evaluations) and 36 rejection episodes were registered. Finally, 78 ARFI evaluations were measured in healthy pancreatic grafts. ARFI values (mean±SD) were as follow: immediately postoperative time (0-11 days) 1.7±0.7 m/s, late postoperative time (12-53 days) 1.53 ±0.56 m/s and older than two months (>61 days) 1.08±0.3 m/s. Statistically significant differences were found between the immediately postoperative time and grafts older than two months (p<0.05).
**Conclusion:** Pancreatic allograft stiffness varies over the post-transplantation time, showing higher values during the immediate postoperative time, which tend to decrease over time. Two months after transplantation, ARFI values are similar to those previously reported in native pancreas.
**Limitations:** Single-center study which limited the number of patients.
**Ethics Committee Approval:** Approved by Hospital Clínic de Barcelona ethics committee.
**Funding for this study:** Supported by a grant of the Instituto de Salud Carlos III (PI 16/00167).
**Author Disclosures:**
Pedro Ventura-Aguiar: Nothing to disclose
Joana Ferrer: Nothing to disclose
Miriam Cuatrecasas: Nothing to disclose
Alexandre Soler: Nothing to disclose
Juan Carlos Soler Perromat: Nothing to disclose
Clara Bassaganyas Vancells: Nothing to disclose
Anna Darnell: Nothing to disclose
María Teresa Rodrigo: Nothing to disclose
Mª Angeles Garcia Criado: Nothing to disclose

### RPS 301a-3

**Association of MRI-based adrenal gland volume and impaired glucose metabolism in a population-based cohort study**

*E. A. Askani*; Freiburg im Breisgau/DE

**Purpose:** Type 2 diabetes (T2DM) and prediabetes exert a stressful metabolic condition, inducing chronic activation of the hypothalamic-pituitary-adrenal (HPA) axis. Chronic activation of HPA axis leads to hypercortisolism and adrenocortical growth. Thus, the aim of this study was to assess adrenal gland volume (AGV) by MRI and to study its role as an indirect marker of HPA axis activation in prediabetes and diabetes.
**Methods or Background:** Subjects enrolled in a prospective case-control study underwent a 3T MRI, including T1w-VIBE-Dixon. For assessment of AGV, boundaries of each adrenal gland were delineated manually in axial reconstructions of T1w-VIBE-Dixon sequences and corrected on coronal and sagittal reconstructions. Univariate and multivariate associations were derived.
**Results or Findings:** Among 375 subjects included in the study (58.4% male, 56.1±9.1 years), 25.3% had prediabetes, 13.6% T2DM and average AGV was 5.6 ± 2.1 ml.
Fasting plasma glucose and glucose tolerance were significantly associated with AGV (β: 0.41, 95%-CI:[0.2, 0.6] and β: 0.53, 95%-CI:[0.3, 0.7]; all p<0.001). After adjustment for age, sex and BMI, only T2DM remained associated with increased AGV compared to healthy subjects (OR:1.41, 95%-CI:[1.11, 1.78]) and showed a significantly increased AGV compared to prediabetes (OR:1.35, 95%-CI:[1.07, 1.69]). No significant difference was found between prediabetes and healthy controls after adjustment for age, sex and BMI.
**Conclusion:** T2DM is significantly associated with increased AGV, independent of age, sex and BMI, whereas the association of prediabetes and AGV is confounded by BMI. AGV may be used as a marker of impaired glucose metabolism and HPA axis dysfunction and may thus merit dedicated research.
**Limitations:** No outcome data available.
**Ethics Committee Approval:** Yes.
**Funding for this study:** German Research Foundation.
**Author Disclosures:**
Esther Adele Askani: Nothing to disclose

### RPS 301a-4

**Iodine concentration derived from dual energy CT: reference values and physiological distribution**

*D. Zopfs*[1], J. Graffe[1], R. P. Reimer[1], S. Lennartz[1], S. Schäfer[2], J. Borggrefe[3], D. Maintz[1], S. Haneder[1], N. Grosse Hokamp[1]; [1]Cologne/DE, [2]Heidelberg/DE, [3]Minden/DE

**Purpose:** Quantitative iodine concentration (IC) derived from dual-energy-CT is increasingly used for detection and characterization of lesions. Information on physiological distribution is an important prerequisite for any imaging biomarker, however, data on reference values of IC is sparse. Aim of this study was to define reference values for IC using a large cohort of healthy individuals.
**Methods or Background:** 571 spectral-detector-CT examinations of the chest and abdomen in portal-venous phase with a fixed amount of intravenous contrast agent were retrospectively included. All examinations were screened for absence of tumor burden, which was confirmed by follow-up imaging after at least three months. Absolute IC values were determined using ROI in parenchymatous organs (n=25), lymph nodes (n=6) and vessels (n=3). All absolute IC values were normalized to the abdominal aorta to retrieve iodine perfusion ratios (IPR) and thus to adjust for the volume of contrast agent to body weight.
**Results or Findings:** IC values showed a varation with age, gender and BMI in lymph nodes, parenchymatous organs and vessels (range: 0.0±0.0mg/ml – 6.6±1.3mg/ml). After normalization to the abdominal aorta, no relation between IPR and BMI persisted. Yet, significant variations with genders and age lasted, with a tendency towards a decline in IPR with increasing age (e.g. liver – 18-44yrs/≥64yrs: 0.50±0.11/0.43±0.10, p≤0.05).
**Conclusion:** This study provides reference values obtained from a large-scale cohort for IC in abdominal organs, lymph nodes and large vessels. The variation of IC with age and gender should be taken into account when applying absolute iodine thresholds.
**Limitations:** - non-visible tumor burden may be present - results only account for portal-venous phase examinations on a dual-layer spectral detector CT
**Ethics Committee Approval:** Written informed consent was waived
**Funding for this study:** This work was partly funded through the Else Kröner-Fresenius-Stiftung (2018_EKMS.34 to Nils Große Hokamp).
**Author Disclosures:**
Simon Lennartz: Research/Grant Support: Philips Healthcare
Josefine Graffe: Nothing to disclose
Stefan Haneder: Nothing to disclose
David Maintz: Speaker: Philips Healthcare
David Zopfs: Nothing to disclose
Nils Grosse Hokamp: Research/Grant Support: Philips Healthcare
Sebastian Schäfer: Employee: Mint Medical GmbH
Jan Borggrefe: Speaker: Philips Healthcare
Robert Peter Reimer: Nothing to disclose

### RPS 301a-5

**Multiparametric MR-analysis of the spleen with Gd-EOB-DTPA: can it be used for the assessment of portal hypertension in the daily clinical routine?**

*D. Catucci*, V. C. Obmann, A. Berzigotti, C. Gräni, L. Ebner, J. T. Heverhagen, A. Christe, A. T. Huber; Bern/CH

**Purpose:** To determine whether multiparametric T1 mapping of the spleen with Gd-EOB-DTPA can be used to distinguish between patients with portal hypertension and patients without portal hypertension.
**Methods or Background:** 251 consecutive abdominal MRI scans with T1 mapping of the spleen pre and post Gd-EOB-DTPA administration in equilibrium phase were analyzed between September 2018 and July 2019. The patients were then divided into groups based on the absence or presence of portosystemic collaterals. Delta T1 (dT1) of the spleen was calculated as (native T1 spleen – post contrast T1 spleen) / native T1 spleen. Groups were compared using Student’s t-test.
**Results or Findings:** At 1.5T 52 patients had portosystemic collaterals (CL), while 82 patients had no collaterals (NCL). At 3T 48 patients had collaterals (CL), while 69 patients had no collaterals (NCL). At both 3T and 1.5T delta T1 of the spleen was significantly higher in patients with collaterals than in patients without collaterals (mean ± standard deviation 0.35 ± 0.08 vs. 0.31 ± 0.08 with p= 0.0055 and mean ± standard deviation 0.35 ± 0.06 vs. 0.31 ± 0.07 with p= 0.0129).
**Conclusion:** This study shows that dT1 of the spleen may be used to discriminate patients with and without portal hypertension using T1 mapping of the spleen with Gd-EOB-DTPA.
**Limitations:** We acknowledge limitations to our study, mainly the retrospective study design and the lack of the hepatic venous pressure gradient (HPVG) as another classification criterion.
**Ethics Committee Approval:** Yes.
**Funding for this study:** This project was funded by the Swiss National Science Foundation (SNF) grant # 320030_188591.
**Author Disclosures:**
Johannes T. Heverhagen: Nothing to disclose
Damiano Catucci: Nothing to disclose
Verena Obmann: Nothing to disclose
Christoph Gräni: Nothing to disclose
Lukas Ebner: Nothing to disclose
Andreas Christe: Nothing to disclose
Adrian Thomas Huber: Nothing to disclose
Annalisa Berzigotti: Nothing to disclose

### RPS 301a-6

**Reproducibility of attenuation measurements in virtual unenhanced images derived from three different DECT scanners**

*S. Lennartz*, A. Parakh, J. Cao, A. Kambadakone; Boston, MA/US
*(simon.lennartz@uk-koeln.de)*

**Purpose:** To investigate the longitudinal reproducibility of VUE attenuation measurements on three different dual-energy CT (DECT) scanner types.
**Methods or Background:** 137 patients with repeated portal-venous, abdominal DECT either on a rapid kV switching (rsDECT; n=46), a dual-layer detector (dlDECT; n=43) and a dual-source scanner (dsDECT; n=51) were included. ROI-based measurements of VUE attenuation and corresponding contrast-enhanced attenuation in virtual monoenergetic images (VMI) were conducted in the liver, spleen, kidneys, aorta, portal vein and subcutaneous fat. Reproducibility of VUE attenuation was analyzed by calculating the mean absolute differences between the first and second scan. Measurements pairs with differences ≤ 10 HU were considered reproducible. Influence of VMI on VUE reproducibility was analyzed using linear regression.
**Results or Findings:** The scanner-specific cohorts showed similar age (p-range:0.35-0.99), sex (p-range:0.68-1), body weight (p-range:0.26-0.87), body diameter (p-range:0.34-0.76), and inter-scan time (p-range:0.52-0.83). 94.9% of VUE measurements were reproducible for rsDECT, 93.8% for dlDECT and 90.6% for dsDECT. The scanner-specific cohorts showed similar mean absolute inter-scan differences in VUE attenuation in the liver/portal vein/fat (rsDECT vs. dsDECT:p=0.27/0.16/0.81; rsDECT vs. dlDECT: p=0.74/0.17/0.82; dlDECT vs. dsDECT: p=0.82/0.95/0.99). For the spleen, kidney and aorta, the inter-scan differences were significantly lower in rsDECT (spleen: 2.8±2.0 HU, kidney: 3.4±2.0 HU, aorta: 4.4±3.3 HU) and dlDECT (spleen:2.4±1.6 HU, kidney: 2.6±2.3 HU, aorta: 4.0±3.6 HU) compared to dsDECT (spleen: 5.0±3.6 HU, kidney: 5.6±3.7 HU, aorta: 6.4±4.3 HU; p<0.05 for all). Inter-scan differences in VMI attenuation significantly influenced inter-scan differences in VUE attenuation (p<0.001, t-ratio:4.34).
**Conclusion:** There was high longitudinal reproducibility of VUE attenuation in all scanner types. The influence of contrast-enhanced attenuation on VUE reproducibility and higher inter-scan differences in dsDECT should be further investigated.
**Limitations:** Retrospective study; limited sample size.
**Ethics Committee Approval:** IRB-approved
**Funding for this study:** Deutsche Forschungsgemeinschaft (DFG, German Research Foundation; 426969820 to Simon Lennartz)
**Author Disclosures:**
Simon Lennartz: Research/Grant Support: Koninklijke Philips N.V.
Avinash Kambadakone: Research/Grant Support: GE Healthcare Research/Grant Support: Koninklijke Philips N.V.
Jinjin Cao: Nothing to disclose
Anushri Parakh: Nothing to disclose

### Research Presentation Session: Abdominal Viscera

### RPS 401a

### Abdominal cancers and metastases

### RPS 401a-1

**Hepatic steatosis has no effect in diagnosis accuracy of LI-RADS v2018 categorisation of hepatocellular carcinoma in MR imaging**

*S. Yao*, Y. Wei, Z. Ye, J. Chen, T. Duan, Z. Zhang, B. Song; Chengdu/CN
*(shandy.yao@foxmail.com)*

**Purpose:** To determine whether hepatic steatosis (HS) affects the accuracy of the diagnosis of hepatocellular carcinoma (HCC) using magnetic resonance imaging (MRI) based on the Liver Imaging Reporting and Data System (LI-RADS) v2018.
**Methods or Background:** 130 patients with 184 pathologically proven HCCs who underwent Gd-EOBDTPA-enhanced liver MRI were included and classified as HS and non-HS groups according to quantitative MRI-proton density fat-fraction (PDFF). HS was defined as MRI-PDFF>5.6%. Two radiologists accessed HCC features and assigned LI-RADS categories in MRI independently based on LI-RADS v2018. Frequencies of HCC major features and sensitivities for the LR assignment between the two groups as well as the interobserver agreement between the two radiologists were assessed.
**Results or Findings:** Major features including arterial hyperenhancement (APHE), enhancing “capsule” and nonperipheral "washout" observed between HS and non-HS groups were not significantly different (75.76% vs. 78.82%, p=0.896; 54.55% vs. 52.98%, p=0.870; and 78.79% vs. 81.46%, p=0.807, respectively), as well as the assessment of observation size (p=0.059). No significant difference in sensitivities of LR-5 assignment between the two groups (63.64% vs. 72.85% for reader 1, p=0.296; 60.6% vs. 72.19% for reader 2, p=0.21). Interobserver agreement between the two radiologists showed almost perfect in LR 5 assignment (κ=0.895, P<0.05) and size observation (ICC=0.999, P<0.05).
**Conclusion:** The diagnosis of HCC based on LI-RADS v2018 in MRI is of great comparable accuracy regardless of HS, in which there is no significant difference in either the major imaging features or LR categorizations.
**Limitations:** The comparison was between with and without HS groups, not analyzing on the degree of HS severity.
**Ethics Committee Approval:** This retrospective study was approved by the Institutional Review Board with written informed consent obtained.
**Funding for this study:** Research Grant of National Nature Science Foundation of China and Science (Grant number 81971571 and 81771797).
**Author Disclosures:**
Yi Wei: Nothing to disclose
Ting Duan: Nothing to disclose
Shan Yao: Nothing to disclose
Zhen Zhang: Nothing to disclose
Jie Chen: Nothing to disclose
Zheng Ye: Nothing to disclose
Bin Song: Nothing to disclose

### RPS 401a-2

**Quantitative iodine concentration of dual layer spectral CT on differential diagnosis of hyper-enhancing lesions in patients at high risk for hepatocellular carcinoma**

*W. Shi*[1], W. Zhang[1], W. Deng[2], W. Mao[1], J. Wang[1]; [1]Guangzhou/CN, [2]Shanghai/CN
*(wenkeyshi@163.com)*

**Purpose:** To evaluate the value of quantitative iodine concentration (IC) of Dual Layer Spectral CT (DLSCT) on differential diagnosis of hyper-enhancing lesions in patients at high risk for Hepatocellular carcinoma (HCC)
**Methods or Background:** Nineteen patients at high risk for HCC with 26 hyper-enhancing focal liver lesions including 14 HCCs and 12 non-HCC lesions with biopsy-proven and/or follow-up who underwent contrast enhancement (370 mg/ml iodine) scanning by DLSCT were involved in this study. Parameters contained IC, normalized IC (NIC) from both arterial phase (AP) and portal venous phase (PVP), and arterial enhancement fraction (AEF). The parameters were compared between HCC and non-HCC lesions using Independent-Sample T test or Mann-Whitney test. The parameters with significant difference were also evaluated via receiver operating characteristic (ROC) curve
**Results or Findings:** The values of IC and NIC of 14 HCCs in PVP were both significantly lower than those of 12 non-HCC lesions, which were 2.43±0.78 versus 3.68±1.10 (P=0.002), and 0.48±0.14 versus 0.58±0.08 (P=0.049), respectively. While the values of IC and NIC between HCC and non-HCC lesions in AP were similar, which were 2.65±1.36 versus 2.41±1.43 (P=0.663), 0.17±0.08 versus 0.18±0.09 (P=0.708), respectively. AEF value was higher in HCC lesions (1.19 ±0.68) than in non-HCC group (0.77±0.59), but with no significant difference (P=0.099). The ROC curve analysis of IC and NIC in PVP showed that the area under curve was 0.845 and 0.705, with optimal sensitivity and specificity 71.43% and 83.33%, 57.14% and 83.33% respectively
**Conclusion:** IC and NIC in PVP derived from DLSCT may have the value to differentiate HCC from non-HCC lesions at high risk for HCC
**Limitations:** This study performed at one institution with a small sample size, and did not contain some relatively rare lesions, including lymphoma, neuroendocrine tumor
**Ethics Committee Approval:** None
**Funding for this study:** None
**Author Disclosures:**
Weibin Mao: Nothing to disclose
Wenqi Shi: Nothing to disclose
Jin Wang: Nothing to disclose
Wu Zhang: Nothing to disclose
Weiwei Deng: Nothing to disclose

### RPS 401a-3

**Chemical and paradigm shift on the imaging of adrenal lesions**

*G. Freire*[1], E. Bandeira[2], V. A. G. Herédia[3], A. P. Matos[2], M. Ramalho[3]; [1]Loures/PT, [2]Lisboa/PT, [3]Almada/PT

**Purpose:** Historically several small studies have reported high sensitivity and specificity of Magnetic Resonance Chemical-shift Imaging (CSI) in diagnosing adrenal adenomas due to a superior sensitivity to detect smaller quantities of fat. Nevertheless, contrast-enhanced CT remains the method of choice for characterizing adrenal lesions. This study intended to assess the CSI's ultimate diagnostic performance using the largest-ever consecutive cohort.
**Methods or Background:** Our sample comprised 480 patients (mean age: 64,3 ± 12,0; 249 females) with 560 consecutive adrenal lesions, including 450 adenomas and 110 non-adenomas (86 metastases, 14 pheochromocytomas, 8 adrenal cortical carcinomas, 1 lipid-poor myelolipoma, and 1 oncocytoma). CSI was performed between Jan-08 and Dec-19. The adrenal signal intensity index (ASII) was calculated for all lesions. Statistical analyses were performed (Student's t-test and Receiver Operating Characteristic curve analysis).
**Results or Findings:** Mean ASII of adrenal adenomas (58.31 ± 20.74) was significantly higher (p < 0.001) than that of the non-adenomas (2.05 ± 8.41). Only 17 of the 450 adenomas (3.8%) were lipid-poor, and only 1 non-adenoma (liposarcoma metastasis) had an ASII higher than 16.5%. CSI showed a sensitivity and specificity of 95.6% and 98.2%, respectively (AUC = 0.980) for differentiating adenomas from nonadenomas (cut-off value of 13.784).
**Conclusion:** Lipid-poor adenomas represent less than 4% of all adenomas on MRI. An ASII >13.784% can be used to distinguish adenomas from non-adenomas. The high diagnostic performance of CSI should render it a different role in the adrenal incidentaloma algorithm.
**Limitations:** Most adenomas and metastases were not histologically proven and were diagnosed based on volumetric stability over 12 months.
**Ethics Committee Approval:** Institutional review board approval was obtained for this retrospective single-center study. The need for informed consent was waived.
**Funding for this study:** No funding was requested.
**Author Disclosures:**
António Pedro Matos: Nothing to disclose
Gonçalo Freire: Nothing to disclose
Miguel Ramalho: nothing to disclose
Eduardo Bandeira: Nothing to disclose
Vasco Andresen Guimarães Herédia: Nothing to disclose

### RPS 401a-4

**Differentiating between peritoneal metastases and ectopic splenic tissue with radiomics in spectral CT**

*F. C. Hasse*, E. Wehrse, L. T. Rotkopf, T. F. Weber, H-P. Schlemmer, H-U. Kauczor, T. Mokry; Heidelberg/DE

**Purpose:** Accessory spleens and splenosis can be challenging to differentiate from peritoneal metastases in initial or post-splenectomy imaging. Aim of this study was to assess quantifiable differences in spectral CT between ectopic splenic tissue and peritoneal metastases in oncologic patients applying radiomics.
**Methods or Background:** This is a retrospective study on 70 consecutive patients with histologically or clinically confirmed peritoneal metastases and/or ectopic splenic tissue like accessory spleens or splenosis, who underwent contrast-agent enhanced venous phase spectral CT of the abdomen. All ectopic spleens (Group A) and peritoneal metastases without macroscopic necrosis or calcifications (Group B) were 3D-segmented manually by two radiologists in consensus on monoenergetic 80 keV images. We performed a radiomics analysis using 107 features from the PyRadiomics library on the segmentations. Two-tailed t-test was used for statistical analysis.
**Results or Findings:** 27 men and 43 women were included (median age 54 y), among them 36 with peritoneal metastases (in total 146 segmented lesions), 25 with accessory spleens and 9 with splenosis (in total 36 segmented ectopic spleens). 48 out of 107 features showed statistically significant differences between the two groups, among them 14 out of the 18 first order features. The first order features mean, 10th and 90th percentile (p<0.01) of CT values, were significantly higher in Group A. Group A also showed higher sphericity (p<0.01) and higher Gray-level Co-occurrence Matrix (GLCM) contrast (p<0.001). Interestingly, apart from sphericity, surface-to-volume-ratio and elongation there were no significant differences among the 14 features of shape.
**Conclusion:** Differences between macroscopically homogenous peritoneal metastases and ectopic splenic tissue can be shown in contrast enhanced venous phase spectral CT using radiomics.
**Limitations:** Small sample size in splenosis group.
**Ethics Committee Approval:** Approval granted by the Ethics Committee of the Medical Faculty Heidelberg.
**Funding for this study:** No funding received.
**Author Disclosures:**
Theresa Mokry: Nothing to disclose
Hans-Ulrich Kauczor: Nothing to disclose
Lukas Thomas Rotkopf: Nothing to disclose
Tim Frederik Weber: Nothing to disclose
Felix Christian Hasse: Nothing to disclose
Heinz-Peter Schlemmer: Nothing to disclose
Eckhard Wehrse: Nothing to disclose

### RPS 401a-5

**Local image variance based on diffusion-MRI may facilitate grading and prognosis in hepatocellular carcinoma: a retrospective study**

*H. Wu*, W. Zhang; Chongqing/CN
*(cefradine1005@gmail.com)*

**Purpose:** The preoperative study aimed to evaluate the potential for local image variance (LIV) analysis based on diffusion-weighted imaging for histological grading and prognosis of the hepatocellular carcinoma (HCC).
**Methods or Background:** 66 patients with known grades were recruited. Also, a follow-up survey was performed post the hospital discharge. Apparent diffusion coefficient (ADC) parameter derived from DWI were used to manually draw regions of interest of lesions, as well as ROIs of healthy tissues used for normalization. In these ROIs, intratumoural hypointensities of ADC was quantified with LIV. The LIV analysis was conducted according to Steiner translation theorem. The discriminating potential of ADC-LIV method for HCC grading prediction was evaluated using ROC curves. Also, KM survival analysis were introduced for the prognosis predication. A log-rank test was used to determine the statistical significance of difference in observed population survival time.
**Results or Findings:** ROC analysis revealed that the ADC-LIV at tumor mass had great ability to discriminate HCC grading, with a sensitivity of 86.9% and a specificity of 93.7% (AUC=.94). In addition, KM survival analysis yielded significant difference in survival time observed from the populations dichotomized by low (<0.632 ) and high (>0.632) LIV value ( log-rank test P < 0.05)
**Conclusion:** The ADC-LIV method was proven to be capable for prediction of HCC grading and prognosis.
**Limitations:** The main limitations is the validating accuracy of the ADC-LIV by biopsy or pathologic sampling. Thus, the method should be used to analyze the patient-derived tumor xenograft model and give a better solution to demonstrate the clinical relevance.
**Ethics Committee Approval:** Our study was approved by the institutional review board of the Army Medical Center of PLA and was conducted in accordance with Health Insurance Portability and Accountability Act regulations.
**Funding for this study:** No funding.
**Author Disclosures:**
Weiguo Zhang: Nothing to disclose
Hao Wu: Nothing to disclose

### Research Presentation Session: Abdominal Viscera

### RPS 501a

### Hepatic imaging

### RPS 501a-1

**Postoperative single-Sequence (PoSSe) MRI: imaging work-up for drainage indication of collections after hepatopancreaticobiliary surgery**

*U. Fehrenbach*[1], T. A. Auer[1], W. Schöning[1], M. Schmelzle[1], C. Jürgensen[1], T. Malinka[1], M. Bahra[1], D. Geisel[1], T. Denecke[2]; [1]Berlin/DE, [2]Leipzig/DE
*(uli.fehrenbach@charite.de)*

**Purpose:** Fluid collections due to anastomotic leakage are a common complication after hepatopancreaticobiliary (HPB) surgery and are usually treated with drainage. We conducted a study to evaluate imaging work-up with a postoperative single-sequence (PoSSe) MRI for the detection of collections and indication of drainage.
**Methods or Background:** Forty-six patients who developed signs of leakage after HPB surgery were prospectively enrolled. Each patient was examined by abdominal sonography and our PoSSe MRI protocol (axial T2-weighted HASTE only). PoSSe MRI examination time (from entering to leaving the MR scanner room) was measured. Sonography and MRI were evaluated regarding the detection and localization of fluid collections. Each examination was classified for diagnostic sufficiency and an image-based recommendation if drainage is necessary or not was proposed. Imaging work-up was evaluated and compared to actual performed drainage procedures and further clinical course.
**Results or Findings:** Sonography, as first line modality, detected 21 focal fluid collections and allowed to decide about the need for drainage in 41% of patients. The average time in the scanning room for PoSSe MRI was 9:23min [7:50-13:32min]. PoSSe MRI detected 46 focal collections and allowed therapeutic decisions in all patients. Drainage was recommended in 25 patients (54%) and performed in 21 patients (100% sensitivity and 84% specificity). No patient needed further imaging to optimize the treatment.
**Conclusion:** The PoSSe MRI approach is feasible in the early postoperative setting after HPB surgery. Imaging work-up regarding drainage of collections was successful in all patients and our proposed PoSSe MRI algorithm provides an alternative to the standard work-up.
**Limitations:** Small patient cohort (explorative study)
**Ethics Committee Approval:** Approval was granted by the IRB of our institution (Internal reference: EA4/029/18).
**Funding for this study:** This research did not receive any grant from funding agencies in the public, commercial, or not-for-profit sectors.
**Author Disclosures:**
Christian Jürgensen: Nothing to disclose
Timm Denecke: Nothing to disclose
Wenzel Schöning: Nothing to disclose
Uli Fehrenbach: Nothing to disclose
Marcus Bahra: Nothing to disclose
Dominik Geisel: Nothing to disclose
Timo Alexander Auer: Nothing to disclose
Thomas Malinka: Nothing to disclose
Moritz Schmelzle: Nothing to disclose

### RPS 501a-2

**Thin-section contrast-enhanced gradient echo T1-weighted MRI of the liver using CS-VIBE: its image quality and lesion detectability in comparison with standard-section imaging using CAIPIRINHA-VIBE**

*J. Yoo*, J. M. Lee, J-H. Yoon, I. Joo, S. J. Park, H-J. Kang; Seoul/KR

**Purpose:** To investigate the practical value of thin-section volumetric interpolated breath-hold (VIBE) sequence using compressed sensing (CS) of gadoxetic acid-enhanced liver MRI in comparison with standard-section Controlled Aliasing in Parallel Imaging Results in a Higher Acceleration (CAIPIRINHA)-VIBE sequence regarding lesion conspicuity and detectability and image quality.
**Methods or Background:** This retrospective study included 143 consecutive patients who underwent gadoxetic acid-enhanced MRI for the evaluation of focal liver lesions. Portal venous phase (PVP) images were obtained twice using thin-section) CS-VIBE technique (acceleration factor [AF], 6.5; slice thickness [ST], 1 mm) followed by standard-section CAIPIRINHA-VIBE technique (AF, 4; ST, 3 mm). Two board-certified radiologists independently reviewed the images and assessed lesion conspicuity and detectability and image quality on a 5-point scale. Lesion detectability of two sequences was compared by calculating jackknife alternative free-response receiver operating characteristics (JAFROC).
**Results or Findings:** Thin-section CS-VIBE images showed superior lesion conspicuity (p<0.001 in reviewer 1; p=0.004 in reviewer 2), and better anatomic details ( p<0.001] in both reviewers) to standard-section CAIPIRINHA-VIBE images. Thin-section CS-VIBE sequences demonstrated decreased motion artifact (p<0.001 in reviewer 1; p=0.046 in reviewer 2), but showed higher image noise (p<0.001 in reviewer 1; p<0.001 in reviewer 2), and increased CS/PI-related artifacts ([p=0.012 in reviewer 1; p<0.001 in reviewer 2). Consequently, both image sets showed comparable overall image quality (4.08 vs. 3.86 [p=0.255] in reviewer 1; 4.13 vs. 4.03 [p=0.148] in reviewer 2). Lesion detectability was not significantly different between two sequences (pooled figures-of-merit estimated with JAFROC analysis; 0.715 vs. 0.716, p=0.956).
**Conclusion:** Thin-section CS-VIBE sequence in the PVP of gadoxetic acid-enhanced liver MRI provided higher lesion conspicuity and comparable lesion detectability, compared with standard-section CAIPIRINHA-VIBE sequence.
**Limitations:** Selection bias is inevitable owing to the retrospective nature
**Ethics Committee Approval:** Approved by Institutional Review Board
**Funding for this study:** None
**Author Disclosures:**
Ijin Joo: Nothing to disclose
Sae Jin Park: Nothing to disclose
Jeong Min Lee: Nothing to disclose
Hyo-Jin Kang: Nothing to disclose
Jeongin Yoo: Nothing to disclose
Jeong-Hee Yoon: Nothing to disclose

### RPS 501a-3

**Combined ultrasound elastography vs computed tomography in patients with non-alcoholic fatty liver disease**

*D. Y. Venidiktova*; Smolensk/RU
*(92darv@gmail.com)*

**Purpose:** To compare diagnostic effectiveness of combined ultrasound elastography (“Combi-elasto”) and computed tomography (MDCT) in quantitative liver evaluation in overweight patients.
**Methods or Background:** In 2018–2020 298 patients of gastroenterological department took part in prospective study at the base of Smolensk Clinical City Hospital No. 1 (Russia). All the patients went through the multiple detector computed tomography of abdominal area (according to their main disease). The inclusion criteria are the signs of liver steatosis according to MDCT (liver attenuation<40 HU), visceral obesity (cross-sectional area at L4-L5>131 cm2). 184 patients (91 women (49,45%), 93 men (50,54%) were accepted, the average age was 48,52 ± 6,45 years. All these patients went through “Combi-elasto” of liver with quantitatine evaluation of liver steatosis, inflammation, fibrosis. Liver biopsy with histological evaluation with SAF scale was used as a reference standard (n=57). Control group (n=104) - patients without obesity and liver steatosis.
**Results or Findings:** All patients according to the “Combi-elasto” had enlarged liver sizes, increased echogenicity, and decreased sound conductivity. Patients according to the ultrasound attenutation in the liver parameter had minimal steatosis S1 (n = 85, 46,2%), moderate S2 (n = 46, 25%), severe S3 (n = 53, 28,8%). Liver inflammation: minimal A1 (n = 44, 23,9%), moderate A2 (n = 29, 15,8%), severe A3 (n = 48, 26%). Liver stiffness: clinically insignificant F1-F2 (n = 12, 6,5%), clinically significant F3 (n = 28, 15,2%), cirrhosis F4 (n = 19, 10,3%). The sensitivity and specificity of “Combi-elasto” – 92,4% and 90,3%.
**Conclusion:** Combined ultrasound elastography is effective method for quantitative evaluation of liver steatosis, inflammation and fibrosis in overweight patients and can be used in detection and monitoring of non-alcoholic fatty liver disease.
**Limitations:** Radiologist's experience in "Combi-elasto" is highly important.
**Ethics Committee Approval:** Yes
**Funding for this study:** Personal
**Author Disclosures:**
Daria Yuryevna Venidiktova: Nothing to disclose

### RPS 501a-5

**Quantitative ultrasound radiofrequency data analysis for the assessment of hepatic steatosis in nonalcoholic fatty liver disease**

*S. K. K. Jeon*, J. M. Lee, I. Joo, S. J. Park; Seoul/KR

**Purpose:** To investigate diagnostic performance of quantitative ultrasound (QUS) parameters for assessment of hepatic steatosis in patients with nonalcoholic fatty liver disease (NAFLD) using magnetic resonance imaging proton density fat fraction (MRI-PDFF) as the reference standard.
**Methods or Background:** In this prospective study, 120 patients with clinically suspected NAFLD were enrolled. Participants underwent ultrasound (US) examination for radiofrequency (RF) data acquisition and liver MRI for PDFF measurement. Using the RF data analysis, attenuation coefficient (AC) at tissue attenuation imaging (TAI) and scatter-distribution coefficient (SC) at tissue scatter-distribution imaging (TSI) were measured. Correlation between QUS parameters (AC and SC) and MRI-PDFF was evaluated using Pearson correlation coefficients. Diagnostic performance of AC at TAI and SC at TSI for detecting hepatic fat content ≥5% (MRI-PDFF ≥5%) and hepatic fat content ≥10% (MRI-PDFF ≥10%) were assessed by receiver operating characteristic (ROC) analysis. Significant clinical or imaging factors associated with AC and SC were analyzed using linear regression analysis
**Results or Findings:** Participants were classified with MRI-PDFF <5% (n=38), 5-10% (n=23), and ≥10% (n=59). AC and SC were significantly correlated with MRI-PDFF (r=0.659 and 0.727, P<0.001 for both). For detecting hepatic fat content ≥5% and hepatic fat content ≥10%, the area under the ROC curves (AUCs) of ACwere 0.861 and 0.835, and of SC were 0.964 and 0.935, respectively. In multivariate linear regression analysis, MRI-PDFF was an independent determinant of AC and SC.
**Conclusion:** AC at TAI and SC at TSI derived from quantitative US RF data analysis yielded a good correlation with MRI-PDFF and provided good performance for detecting hepatic steatosis and assessing its severity in NAFLD.
**Limitations:** Validation is required
**Ethics Committee Approval:** Approved by IRB, and written informed consent was obtained from all participants
**Funding for this study:** Supported by a research grant from Samsung Medison
**Author Disclosures:**
Ijin Joo: Nothing to disclose
Sae Jin Park: Nothing to disclose
Jeong Min Lee: Nothing to disclose
Sun Kyung Kyung Jeon: Nothing to disclose

### RPS 501a-6

**Relationship between variant biliary and vascular anatomy in living liver donors**

*L. Masood*, S. Sayeed, S. Aslam, B. Y. Faiz; Islamabad/PK
*(laibamasood66@gmail.com)*

**Purpose:** Purpose of this study was to investigate correlation of hepatic vascular variants in live liver donors with established biliary variant anatomy.
**Methods or Background:** After IRB approval, two radiologists reviewed preoperative CT volumetry scans and MRCPs from 165 live liver donors having variant biliary anatomy. Classification of conventional or variant hepatic artery, hepatic veins and portal veins was done based on CT volumetry scans while biliary anatomy was characterized based on MRCP images.
**Results or Findings:** Out of 165 subjects having variant biliary anatomy, conventional anatomy involving hepatic arteries were seen in 88(53.3%) and portal veins in 125(75.8%) subjects. Right inferior hepatic vein was found in 7(4.2%) and 32(19.3%) having accessory hepatic venous drainage of segment VI or VII or both. 40(24%) liver donors had variant portal venous anatomy and 77(46%) had hepatic artery variations. Significant association was seen between variant biliary drainage and hepatic arterial supply with p value = 0.032.
**Conclusion:** Detailed preoperative evaluation of biliary and vascular anatomy of liver donors is essential with regards to surgical planning in selecting suitable candidates, need for vascular or biliary reconstruction allowing surgeon to prepare well in time and anticipate the possible complications which may result in bile leak, bile duct stricture, vascular compromise and eventual graft failure.
**Limitations:** This is a single center study with a relatively limited sample volume, multi centric collaborative studies are needed to consolidate our findings.
**Ethics Committee Approval:** Study was performed after approval from institution review board and written consent was waived.
**Funding for this study:** None
**Author Disclosures:**
Belqees Yawar Faiz: Nothing to disclose
Samreen Aslam: Nothing to disclose
Sana Sayeed: Nothing to disclose
Laiba Masood: Nothing to disclose

### RPS 501a-7

**Dynamic volumetric CT liver perfusion: a comparative analysis of liver perfusion results of donors and recipients after liver transplantation**

F. Nazyrov, A. Ikramov, N. Djuraeva, A. Devyatov, A. Amirkhamzaev, U. R. Salimov, *N. Vakhidova*, X. Shamirzaev, K. Abdukhalimova; Tashkent/UZ
*(sidikovan@yandex.ru)*

**Purpose:** To compare the perfusion MSCT parameters of the liver after orthotopic liver transplantation(LT) with a control group of donors.
**Methods or Background:** Dynamic volume perfusion MSCT of the liver was performed in 88 donors and 16 recipients. We performed a comparative analysis of perfusion MSCT of the liver(arterial and portal perfusion, hepatic perfusion index) in the recipient before and after liver transplantation on the 7th day after surgery, and also 3 months after LT.
**Results or Findings:** The volume of portal perfusion(PP) in recipients with liver cirrhosis(LC) was amounted to 150.2±4.0ml/100ml³/min versus 180.5±2.4ml/100ml³/min(t=6.58;p<0.001) from donors. After LT PP increased to 185.6±4.7ml/100ml³/min. Arterial perfusion(AP) increased compensatory and if in healthy people this indicator was 20.8±0.7ml/100ml³/min, then with LC-34.1±1.1 ml/100ml³/min(t=10,56;p<0.001). After LT the AP decreased and amounted to 21.8±0.6ml/100ml³/min. At the same time, the difference in indicators before and after LT ranged from -7.9-19.6 ml/100 ml³/ min with an average value of-12.3±0.9ml/100 ml³/min(t=13.95;p<0.001). These values, as well as in relation to portal perfusion, no longer had significant differences in comparison with the data in donors(t=1.14;p>0.05). In turn, when comparing the results after LT in recipients with the initial data in donors, a significant difference in the hepatic perfusion index(HPI) was observed 12.3%(13.8±0.3% for donors and 15.5±0.3% for recipients;t=4.10;p<0.001).
**Conclusion:** In relation to the indicators in donors in recipients before LT, the value of arterial perfusion was 64% higher, whereas after LT this indicator differed only by 4.8%, which was not significant.PP was initially 16.8% lower, and after LT increased to virtually normal values in relation to healthy people with a difference of only 2.8%. The HPI was 81.8% higher than in donors. After LT this difference was only 12.3%.
**Limitations:** within the Institution rules
**Ethics Committee Approval:** Approved by Institution
**Funding for this study:** Institution
**Author Disclosures:**
Adham Ikramov: Nothing to disclose
Umid Ravshanovich Salimov: Nothing to disclose
Xakim Shamirzaev: Nothing to disclose
Andrey Devyatov: Nothing to disclose
Khanum Abdukhalimova: Nothing to disclose
Aybek Amirkhamzaev: Nothing to disclose
Nigora Djuraeva: Nothing to disclose
Academician Feruz Nazyrov: Nothing to disclose
Nargiza Tulkinovna Vakhidova: Nothing to disclose

### Research Presentation Session: GI Tract

### RPS 201b

### Conditions in the GI tract

### RPS 201b-3

**Assessment of lymph node status and tumour response after chemoradiation therapy in locally advanced rectal cancer: comparison of region of interest methods of intravoxel incoherent motion parameters**

*Y. Yuan*, H. Pu, X-L. Chen, H. Li; Chengdu/CN

**Purpose:** To assess the diagnostic performance of region of interest (ROI) methods of intravoxel incoherent motion diffusion-weighted imaging (IVIM-DWI) for determining lymph node metastases (LNM) and tumor response after chemoradiation therapy (CRT) in locally advanced rectal cancer (LARC).
**Methods or Background:** 52 patients underwent preoperative IVIM-DWI before and after CRT. IVIM-DWI parameters apparent diffusion coefficient (ADC), slow diffusion coefficient (D), fast diffusion coefficient (D*), perfusion-related diffusion fraction (f) and their percentage changes [Δ%]) were obtained according to three ROI protocols: whole-volume, single-slice and small samples. IVIM parameters were compared between LNM negative and positive groups, between pathological complete response (pCR) and non-pCR groups, and between good and poor response groups. Areas under the receiver operating characteristic curves (AUCs) were calculated to evaluate diagnostic performance.
**Results or Findings:** Interobserver agreement were good for pre- and post-CRT whole-volume ROI and single-slice ROI (intraclass correlation coefficient [ICC], 0.775-0.953), and moderate for small samples ROI (ICC, 0.581-0.905). As for LNM, AUCs for whole-volume ROI-derived post-f, post-D, and Δ%D were higher than that of the other IVIM parameters (AUC,0.753-0.760 vs. 0.658-0.733). As for pCR, AUCs for whole-volume ROI-derived Δ%D were higher than that of the other IVIM parameters (AUC,0.878 vs. 0.727-0.759). As for good response, AUCs for whole-volume ROI derived Δ%D were higher than that of the other IVIM parameters (AUC,0.872 vs. 0. 640-0.822).
**Conclusion:** Δ%D was equally to post-D and post-f values derived from whole-volume ROI in discriminating LNM after CRT. Whole-volume ROI-derived Δ%D

values provided high diagnostic performance for evaluating pCR and good response.
**Limitations:** It was a retrospective study with a rather small sample size and lacked more lower b-values, especially b-values< 200 s/mm2.
**Ethics Committee Approval:** Approved by the institutional review board.
**Funding for this study:** Sichuan Science and Technology Program (No. 2019YFS0437) and (No.20GJHZ0105)
**Author Disclosures:**
Yi Yuan: Nothing to disclose
Hong Pu: Nothing to disclose
Hang Li: Nothing to disclose
Xiao-Li Chen: Nothing to disclose

### RPS 201b-4

**Preoperative measurement of the hiatal surface with MDCT: impact on surgical planning**

*G. Trillò*[1], M. Rengo[1], S. Badia[1], D. M. Bellini[1], I. Carbone[2], S. Picchia[1], S. Vicini[1], A. Capodanno[1], A. Laghi[2]; [1]Latina/IT, [2]Rome/IT

**Purpose:** To evaluate the accuracy and reproducibility of Hiatal Surface Area (HSA) measured on dedicated Multidetector computed tomography (MDCT) acquisition, in patients previously subjected to laparoscopic sleeve gastrectomy (LSG) with hiatal hernia (HH), compared to surgical measurement.
**Methods or Background:** Fifty-two candidates for laparoscopic antireflux surgery were prospectively included in the study. The quantification of HSA was performed on MDCT images acquired during the administration of iodinated contrast media. Measurements were performed by nine readers with different experiences. Results were compared with intraoperative measurements by means of linear regression. Reproducibility was evaluated with intra and inter reader agreement by means of Intraclass correlation coefficient (ICC) among nine readers with different experience.
**Results or Findings:** A moderate correlation between HSA measured intraoperatively and on MDCT acquired during swallowing was observed (R2= 0.59, p<0.0034). An excellent correlation between HSA measured intraoperatively and on MDCT acquired during strain was observed (R2=0.93, p<0.0001) resulting in a good agreement between two methods (κ =0.73). All patients were correctly classified by MDCT according to HSA cut off value as determined by surgery. An excellent correlation between HSA measurements obtained on MDCT, by expert and less experienced readers and intraoperatively was observed (R2= 0.94 and 0.83). Inter-reader agreement and intra-reader agreement were excellent (ICC= 0.95 and κ = 0.93).
**Conclusion:** HSA can be accurately measured on MDCT images. This method is reproducible and minimally influenced by reader experience. The preoperative measurement of HSA has potential advantages for surgeons in term of correct approach to hiatal defects in obese patient.
**Limitations:** We didn’t investigate the effect of surgery on symptoms or the relapse of HH. Another limit is single center study design.
**Ethics Committee Approval:** The study was approved by the Institutional Review Board and informed consent was obtained.
**Funding for this study:** None.
**Author Disclosures:**
Giovanni Trillò: Nothing to disclose
Iacopo Carbone: Nothing to disclose
Simone Vicini: Nothing to disclose
Marco Rengo: Nothing to disclose
Andrea Laghi: Nothing to disclose
Antonio Capodanno: Nothing to disclose
Davide Maria Bellini: Nothing to disclose
Stefano Badia: Nothing to disclose
Simona Picchia: Nothing to disclose

### RPS 201b-5

**Radiomic's contribution to MR Imaging of patients with LARC (Locally Advanced Rectal Cancer-stage IIa-IIIc AJCC) in the evaluation of long-term outcome**

M. G. Belgrano[1], *I. Fiorese*[1], M. Costa[2], I. Gandin[1], C. Marrocchio[1], M. A. A. Cova[1]; [1]Trieste/IT, [2]Udine/IT

**Purpose:** To perform a radiomic study on MR imaging to create a model able to predict the pathological response of patients with LARC on pre neoadjuvant treatment images.
**Methods or Background:** Between the 1st of January 2007 and the 31st of December 2017, pretreatment MR images of Patients with LARC who underwent neoadjuvant therapy before surgical intervention and histologic evaluation of the TRG (Tumor Regression Grade) on the surgical specimen were collected. Two radiologists identified the lesions and proceeded to their segmentation. In addition, 1cm3 sphere was delineated in the most representative area of the tumor. The radiomic features were extracted from the volumes thus obtained and the statistical analysis was separately performed for the entire lesion and for the single sphere. The clinical outcome was the TRG grade. After a univariate analysis the features with a significance level < 0,05 were selected and then reduced to a singe variable (PC1) with the Principal Component Analysis. The PC1 and the T stage of the lesion were included in the model whose performance has been evaluated with the AUC-ROC curve.
**Results or Findings:** Thirty-nine patients (M:23, F:16, median age:70) were selected. For each patient 227 features were extracted. Ten features were selected from the entire lesion and 5 from the single sphere evaluation (p<0,05) and then reduced to the variable PC1. The models obtained demonstrated a significant association between the PC1 and the clinical outcome with good performance both in the entire lesion (p-value = 0,0157, AUC-ROC = 0,731) and in the single sphere (p-value = 0,0229, AUC-ROC = 0,743).
**Conclusion:** There is a correlation between radiomics features and pathological response of patients with LARC. The sphere could significantly shorten and simplify radiomic studies'ecexution.
**Limitations:** Number of Patients
**Ethics Committee Approval:** Approved
**Funding for this study:** None
**Author Disclosures:**
Matilde Costa: Nothing to disclose
Manuel Gianvalerio Belgrano: Nothing to disclose
Cristina Marrocchio: Nothing to disclose
Ilaria Fiorese: Nothing to disclose
Maria Assunta A. Cova: Nothing to disclose
Ilaria Gandin: Nothing to disclose

### RPS 201b-6

**The significance of preoperative computed tomography (CT) features in the prediction of overall survival in gastric cancer: a retrospective analysis**

*I. Kizildag Yirgin*, S. M. Ertürk, I. Dogan, S. Vatansever; Istanbul/TR
*(incikizil@hotmail.com)*

**Purpose:** The objective of this study was to evaluate the role of preoperative computed tomography (CT) findings in predicting overall survival (OS) in patients operated for gastric cancer.
**Methods or Background:** The cases of 101 patients with gastric cancer (68 men, 33 women; age range, 29-82 years; median, 61 years) who underwent CT before surgery were retrospectively evaluated. Two radiologists reviewed multiplanar reconstruction images to measure invasion depth of the tumor (T stage), the number of pathological lymph nodes (N stage), the long diameter of the lesion, the localization of the tumor and the attenuation values of tumor in the arterial and venous phases (HUs). Post operative pathology findings, resection status (R0,R1), pathologic T stage, N stage, grade, histopathological subtype were recorded. All CT-derived parameters and clinicopathological variables associated with OS were analysed by univariate analysis, followed by multivariate and receiver operator characteristics (ROC) analysis.
**Results or Findings:** Multivariate cox regression analysis showed that non of the preoperative CT findings correlated with the OS. The survival rate after resection was worse for the R1 group and high grade group than for the R0 group and low grade group (p:0.001,p:0.005 respectively). N stage and long diameter of the lesion on CT imaging correctly predicted R1 resection ( AUC,0.697; sensitivity,63%; specificity, 88%, AUC,0.734; sensitivity,18%; specificity, 76% respectively).
**Conclusion:** R1 resection is correlated with lower OS in operated gastric cancer. CT findings including the long diameter of the tumor and the number of pathological lymph nodes, can predict R1 resection.
**Limitations:** This is a retrospective study and number of participants were small.
**Ethics Committee Approval:** The study protocol was approved by our institutional ethics committee .
**Funding for this study:** No
**Author Disclosures:**
Inci Kızıldağ Yırgın: Nothing to disclose
Izzet Dogan: Nothing to disclose
Sezai Vatansever: Nothing to disclose
Sükrü Mehmet Ertürk: Nothing to disclose

### Research Presentation Session: GI Tract

### RPS 301b

### Liver and the pancreas

### RPS 301b-1

**Quantification of liver steatosis with 3rd generation dual source dual energy CT: a comparison with pathology after liver resection**

*A. Agostini*, M. Tufillaro, A. Borgheresi, L. Ottaviani, R. Rossi, F. Puccio, A. Mandolesi, M. Vivarelli, A. Giovagnoni; Ancona/IT
*(dott.andrea.agostini@gmail.com)*

**Purpose:** To correlate the quantification of liver steatosis estimated on Dual Source Dual Energy CT (dsDECT) with pathological specimens after liver resection.
**Methods or Background:** Inclusion criteria: patients >18 years old, underwent to liver surgery between January 2018 and January 2020, with an abdominal contrast-enhanced dsDECT within 1 month before resection, were retrospectively included. Exclusion criteria: liver cirrhosis, absence of contrast-enhanced dsDECT or pathological data. dsDECT were performed with a 3rd generation Dual Source scanner (80-100/150Sn kV, modulated mA), with triphasic contrast-enhanced study (370 mg[I]/ml, 1,3 ml/kg body weight). Liver steatosis was quantified with three-material decomposition algorithm from arterial and venous dsDECT datasets; liver attenuations on virtual non-contrast (VNC) images from both datasets were recorded. The pathological specimens were reviewed by two liver pathologists in consensus. Steatosis from dsDECT and liver attenuation on VNC were compared to pathology with Receiver Characteristic Curves (ROC) Analysis (endpoint: pathological steatosis ≥30%).
**Results or Findings:** 48 patients (M/F=30/18, mean age: 65 years old) were retrospectively included; 20/48 were resected for colorectal metastases and 15/48 for cholangiocarcinoma. dsDECT datasets showed significantly higher areas under the curve (AUC 0.872-0.891) than attenuation values on VNC (AUC 0.801-0.813) at ROC analysis, with comparable sensitivity (84%) and higher specificity (86%-91% vs 65-75%).
**Conclusion:** Liver steatosis can be accurately quantified on dsDECT datasets, with better performance of quantification with three-material decomposition than VNC attenuation values.
**Limitations:** Retrospective study, single center, small sample.
**Ethics Committee Approval:** The study was approved bay the local IRB and the infromed consent wass waived.
**Funding for this study:** None.
**Author Disclosures:**
Marianna Tufillaro: Nothing to disclose
Francesca Puccio: Nothing to disclose
Andrea Agostini: Speaker: Siemens Healthineers
Alessandra Mandolesi: Nothing to disclose
Roberta Rossi: Nothing to disclose
Alessandra Borgheresi: Nothing to disclose
Letizia Ottaviani: Nothing to disclose
Andrea Giovagnoni: Nothing to disclose
Marco Vivarelli: Nothing to disclose

### RPS 301b-2

**Radiomics signature of acute pancreatitis in CT**

*S. Bette*[1], J. Decker[1], P. Woznicki[1], S. Gölder[1], B. Geißler[1], C. Scheurig-Münkler[1], T. J. Kroencke[1], B. Baessler[2], F. Schwarz[1]; [1]Augsburg/DE, [2]Zürich/CH

**Purpose:** Acute pancreatitis (AP) remains a potentially life-threatening disease. In early diagnostic workup, the role of CT is to establish the diagnosis, assess severity, and detect potential complications. The aim of our study was to develop and evaluate an automatic machine learning model for AP detection in CT images.
**Methods or Background:** This retrospective analysis included 300 patients who had undergone contrast-enhanced CT of the abdomen between 2015 and 2020 with a clinical suspicion for AP (AP confirmed: n = 169; AP excluded: n = 131). Automatic pancreas segmentation was performed using a pre-trained deep learning model, based on 3D U-Net architecture. Subsequently, radiomic features were extracted from the region of interest using pyradiomics. The 10 most determinant and least correlated features were selected with the mRMR algorithm and a Random Forest classifier was trained to perform classification using the features. The model was evaluated using 5-fold cross-validation and a temporally separated patient cohort.
**Results or Findings:** The machine learning model achieved high predictive performance with an AUC of 0.91 (95% CI: 0.85-0.94) for AP detection in the test set. Accordingly, the method achieved a sensitivity of 88% (95% CI: 76%-97%) and a specificity of 79% (95% CI: 62%-93%) for the detection of the AP.
**Conclusion:** Radiomics-based machine learning model allows for the detection of acute pancreatitis with high diagnostic accuracy and might facilitate the correct diagnosis in clinical routine. We are planning to further extend the analysis with more clinical outcomes and evaluate the model in a follow-up prospective study.
**Limitations:** This is a single-center retrospective study.
**Ethics Committee Approval:** This retrospective study was approved by the local IRB.
**Funding for this study:** PD Dr. Bette and PD Dr. Schwarz have received funding from the university of Augsburg for this study.
**Author Disclosures:**
Frau Stefanie Bette: Nothing to disclose
Herr Christian Scheurig-Münkler: Nothing to disclose
Herr Stefan Gölder: Nothing to disclose
Frau Bettina Baessler: Nothing to disclose
Piotr Woznicki: Nothing to disclose
Thomas J. Kroencke: Nothing to disclose
Florian Schwarz: Nothing to disclose
Herr Josua Decker: Nothing to disclose
Herr Bernd Geißler: Nothing to disclose

### RPS 301b-3

**Impact of meal intake on the liver stiffness assessed by MRE in healthy volunteers**

M. Obrzut[1], *V. Atamaniuk*[1], J. Chen[2], R. Ehman[2], B. Obrzut[1], M. Cholewa[1], K. Gutkowski[1]; [1]Rzeszów/PL, [2]Rochester, MN/US
*(vitaliyacera500@gmail.com)*

**Purpose:** Chronic liver diseases are a severe health problem worldwide. They lead to liver fibrosis and, if not treated, to liver cirrhosis. However, this dynamic process may be reversible when detected early. Magnetic Resonance Elastography (MRE) is a non-invasive method for evaluating liver stiffness, which allows detecting liver fibrosis on its early stages. It has almost replaced invasive liver biopsy, which has been a gold standard in the diagnosis of liver fibrosis for many years. To eliminate potential changes in the liver stiffness caused by a postprandial increase in blood flow, current clinical protocols recommend performing MRE examination at the earliest 4 hours after food intake. Nevertheless, this relationship has not been properly studied yet.
**Methods or Background:** This study aimed to determine how the food intake affects liver stiffness and if it can be the reason of incorrect clinical diagnoses. A total of 100 healthy volunteers (36 men and 64 women; age range 20-32 years, mean age 22.9 years) were enrolled in this study. A 1.5T GE Optima clinical MRI scanner was used to assess the liver stiffness.
**Results or Findings:** The highest average liver stiffness value was recorded 30 minutes after a meal. Then a gradual decrease was observed. Still, even at 2h30m after meal, the liver stiffness was higher than that measured with empty stomach: 2.41 vs. 2.25 kPa.
**Conclusion:** Therefore, to avoid false positive results and the subsequent unnecessary diagnostics and/or treatment, we recommend performing MRE exams aimed to assess liver stiffness at least 3 hours after meal.
**Limitations:** Narrow age range.
**Ethics Committee Approval:** The study was approved by the Ethics Committee at the University of Rzeszów (No. 10/01/2019) and conformed to the ethical guidelines of the 1975 Declaration of Helsinki (6th revision, 2008).
**Funding for this study:** Not applicable.
**Author Disclosures:**
Marzanna Obrzut: Nothing to disclose
Marian Cholewa: Nothing to disclose
Jun Chen: Other: Resoundant
Richard Ehman: Other: Resoundant
Vitaliy Atamaniuk: Nothing to disclose
Krzysztof Gutkowski: Nothing to disclose
Bogdan Obrzut: Nothing to disclose

### RPS 301b-5

**Multiparametric-MRI of liver disease progression in preclinical animal models**

*F. Baskaya*, T. Nolte, S. Koletnik, A. Rix, V. Schulz, W. Lederle, F. Kiessling; Aachen/DE
*(ferhanbaskaya@gmx.de)*

**Purpose:** Chronic liver diseases (CLD) are associated with inflammation and changes in cell and tissue structure including fat content and activity of hepatocytes and macrophages. Thus, our goal was to investigate whether longitudinal multiparametric MRI can sensitively assess these changes in two CLD animal models of different etiology.
**Methods or Background:** In the NASH model, 32 mice received a high-fat diet over 24 weeks. In the fibrosis model, 20 animals were injected twice weekly intraperitoneally with CCl4 for 8 weeks. 37 healthy animals served as controls.
Longitudinal multiparametric imaging was performed at 7T MRI (Bruker BioSpec 70/20) using T1/T2-relaxometry, morphological T1w/T2w-imaging and fat-selective imaging. Furthermore, T1w and T2*w DCE/DSC-MRI were applied using PrimovistR and VivoTraxTM, respectively. Imaging data were validated by serological and immunohistological analyses.
**Results or Findings:** In the NASH-model, fatty liver degeneration was semiquantitatively assessed by fat-selective MRI (wk0: 3.9±1.0a.u., wk16: 33.7±13.1a.u., p=0.0043) and reflected by decreasing native T1-relaxation times (wk0: 1570±101ms, wk16: 816±214ms, p=<0.0001). While for CCl4 these changes are absent, a prolongation of T2-relaxation times was found in both models (NASH: wk4: 21.9±2.0ms, wk16: 28.6±4.6ms, p=0.0451; CCl4: wk0: 19.1±1.3ms, wk4: 21.2±0.6ms, p=0.0340) indicating inflammation-related edema. Furthermore, only in the NASH-model the uptake of PrimovistR and VivoTraxTM decreased suggesting either reduced delivery of the contrast agents to hepatocytes or macrophages, respectively, or disturbed cellular internalization. ALT/AST levels were increased in diseased animals in both models.
**Conclusion:** Non-invasive MRI with commercially available contrast agents can sensitively assess pathophysiological changes in the liver that can help to classify CLD and to track its progression. In the future, these extended MRI examinations may also be used to monitor treatments and to reduce the numbers of liver biopsies.
**Limitations:** n/a
**Ethics Committee Approval:** n/a
**Funding for this study:** BMBF-LiSyM GrantNo.031L0041
DFG, SFB1382, project Q1, project-ID 403224013
**Author Disclosures:**
Fabian Kiessling: Nothing to disclose
Wiltrud Lederle: Nothing to disclose
Teresa Nolte: Nothing to disclose
Ferhan Baskaya: Nothing to disclose
Volkmar Schulz: Nothing to disclose
Anne Rix: Nothing to disclose
Susanne Koletnik: Nothing to disclose

### RPS 301b-6

**Influence of contrast medium on the CT textural parameters in hepatic metastases and in normal liver: a radiomics analysis**

*F. Fiz*, G. Costa, N. Gennaro, L. La Bella, A. Boichuk, G. Torzilli, L. Balzarini, A. Chiti, L. Viganò; Milan/IT
*(francesco.fiz.nm@gmail.com)*

**Purpose:** Textural features (TF) of colorectal liver metastases (CLM) are usually extracted from portal venous phase of CT (PVP), but the impact of contrast enhancement on their values is unclear. We investigated whether significant differences of CLM/liver TF values exist between PVP and basal phase (BP).
**Methods or Background:** Patients undergoing CLM resection (2017-2020) were considered. On the PVP, we drew the following VOI: CLM (up to 5 lesions, Tumor-VOI); a 5-mm rim of parenchyma around CLM (Margin-VOI); a 2-ml sample of non-tumoral liver parenchyma distant from CLM (Liver-VOI). VOI were then copied onto the BP. Forty-five TF were extracted from each VOI (LIFEx®).
**Results or Findings:** 409 CLM in 162 patients were analyzed. Most TF were significantly different between PVP and BP in Tumor-VOI (38/45, 85%) and Margin-VOI (41/45, 91%); less so in Liver-VOI (26/45, 58%, p<0.001).
In PVP, entropy increased and energy decreased in Tumor-VOI (log10-entropy 0.93±0.10 vs 0.85±0.14 in BP; energy 0.14±0.03 vs 0.18±0.04; p<0.001) and in Margin-VOI (0.89±0.11 vs 0.85±0.12; 0.16±0.04 vs 0.18±0.04, p<0.001). These parameters remained stable in Liver-VOI. Remaining first-order TF were modified by enhancement in all VOI. Modification of Tumor-VOI TF was independent of tumor size. Comparing VOIs in BP, entropy and energy of Tumor- and Margin-VOI were not significantly different, while Liver-VOI had lower entropy and higher energy (0.76/0.21, p<0.001). In PVP, an entropy and energy gradient was observed (entropy: Tumor>Margin>Liver; energy: Tumor<Margin<Liver, p<0,001).
**Conclusion:** Contrast enhancement on CT had a relevant effect on TF values of CLM, independently from size, while did not affect entropy and energy of liver parenchyma. TF of peritumoral tissue had modification similar to Tumor-VOI, despite its apparently normal radiological aspect.
**Limitations:** Retrospective, monocentric.
**Ethics Committee Approval:** Obtained.
**Funding for this study:** None.
**Author Disclosures:**
Guido Torzilli: Nothing to disclose
Alexandra Boichuk: Nothing to disclose
Ludovico La Bella: Nothing to disclose
Francesco Fiz: Nothing to disclose
Arturo Chiti: Nothing to disclose
Nicolò Gennaro: Nothing to disclose
Guido Costa: Nothing to disclose
Luca Viganò: Nothing to disclose
Luca Balzarini: Nothing to disclose

### RPS 301b-7

**Liver volume parameters measured by Computed Tomographic (CT) portography may be used to predict the severity of esophageal varices in cirrhotic patients**

*S. Wan*[1], B. Song[1], X. Zhang[2]; [1]Chengdu/CN, [2]Beijing/CN
*(604775856@qq.com)*

**Purpose:** To investigate whether liver lobe volume indices and the ratio of caudate lobe volume(CV) to total liver volume(CV/TLV) on computed tomographic portography(CTP) could noninvasively predict the severity of esophageal varices(EV) in cirrhotic patients.
**Methods or Background:** Sixty-one consecutive cirrhotic patients from January 2020 to October 2020 were enrolled in this study. All participants underwent CTP to measure liver volume indices, with endoscopy as reference standard. According to endoscopic results, patients were divided into a non-conspicuous EV group(mild-to-moderate EV,n=29) and a conspicuous EV group(severe EV,n=32). Total liver volume(TLV), right liver lobe volume(RV), left liver lobe volume(LV), caudate lobe volume(CV) were measured using Medical Image Processing software(Shanghai United Imaging Healthcare), and the ratio of CV to TLV(CV/TLV) was calculated. Statistical analyses and the receiver operating characteristic(ROC) curves were performed to determine whether and how these parameters could predict the severity of EV.
**Results or Findings:** The indices of CV and CV/TLV were statistically significant(P<0.05), CV/TLV could best distinguish mild-to-moderate EV from severe EV, with an area under ROC curve(AUC) of 0.96, a sensitivity of 92.3% and a specificity of 100%, and the cutoff value was 5.2%. CV could also distinguish mild-to-moderate EV from severe EV, with an AUC of 0.94, a sensitivity of 84.6% and a specificity of 92.0%, and the cutoff value was 51.05cm$^3$.
**Conclusion:** Liver morphological changes may correlate with the severity of EV, in which, liver volume parameters of CV and CV/TLV measured on CTP have great potential to predict EV severity and be used as an alternative to invasive endoscopy.
**Limitations:** Some selection bias may introduce disagreement between observers due to the subjective endoscopic grading system, and some interval progression of disease cannot be ruled out.
**Ethics Committee Approval:** This study had approval from West China Hospital.
**Funding for this study:** Not applicable
**Author Disclosures:**
Xin Zhang: nothing to disclose
Shang Wan: Nothing to disclose
Bin Song: Nothing to disclose

### RPS 301b-8

**Liver iron concentration in healthy volunteers evaluated by MRI**

M. Obrzut[1], *V. Atamaniuk*[1], K. Glaser[2], J. Chen[2], R. Ehman[2], B. Obrzut[1], M. Cholewa[1], K. Gutkowski[1]; [1]Rzeszów/PL, [2]Rochester, MN/US
*(vitaliyacera500@gmail.com)*

**Purpose:** Iron overload is a common clinical condition, which leads to progressive liver fibrosis or even cirrhosis. It has numerous causes, including thalassemia, hereditary hemochromatosis, and myelodysplasia. Liver biopsy is one of the reference tests used to quantify Liver Iron Concentration (LIC). Still, this procedure has significant limitations and is associated with several potential risks. Magnetic Resonance Imaging (MRI) and assessment of the R2* relaxation rate can become an alternative to liver biopsy, but it cannot be used as a diagnostic tool without accurate R2* data corresponding to standard LIC value. For this reason, this study aimed to evaluate the normative values of liver R2* in healthy volunteers.
**Methods or Background:** A total of 100 healthy volunteers (36 men and 64 women; age range 20-32 years, mean age 22.9 years) were enrolled in this study. A 1.5T GE Optima clinical magnetic resonance scanner was used to assess the R2* rate. Images for measuring the LIC and liver fat concentration were obtained using the IDEAL-IQ technique for liver imaging.
**Results or Findings:** The Mean (SD) liver R2* was 28.34 (2.25) s−1 (95% CI, 27.78– 28.90, range 23.67–33.00 s−1) in females, 29.57 (3.20) s−1 (95% CI, 28.49–30.66, range 23.93–37.77 s−1) in males, and 28.72 (2.69) s−1 (range 23.67–37.77 s−1) in the whole group.
**Conclusion:** R2* value in this particular population with a high proportion of young women did not exceed 38 s−1. In the absence of fibrosis or steatosis, liver stiffness and fat fraction did not show any relationship with R2*.
**Limitations:** Narrow age range; high proportion of women.

**Ethics Committee Approval:** The study was approved by the Ethics Committee at the University of Rzeszow (No. 10/01/2019) and conformed to the ethical guidelines of the 1975 Declaration of Helsinki (6th revision, 2008).
**Funding for this study:** Not applicable.
**Author Disclosures:**
Marzanna Obrzut: Nothing to disclose
Marian Cholewa: Nothing to disclose
Jun Chen: Other: Resoundant
Richard Ehman: Other: Resoundant
Kevin Glaser: Other: Resoundant
Vitaliy Atamaniuk: Nothing to disclose
Krzysztof Gutkowski: Nothing to disclose
Bogdan Obrzut: Nothing to disclose

### RPS 301b-9

**Free-breathing contrast-enhanced multiphase MRI of the liver in patients with a high risk of breath-holding failure**
J. S. Kim, *E. S. Choi*; Seoul/KR
*(eunnysunny11@gmail.com)*

**Purpose: Purpose:** To compare image quality of cartesian k-space sampling volumetric interpolate examination with compressed sensing(XD-VIBE) and Golden-Angle radial sampling with denoising technique(GRASP) in patients with a high risk of breath-holding failure.
**Methods or Background:** Methods: A total of 67 patients with high risk of breath-holding failure underwent XD-VIBE(n=33) or GRASP(n=34) with optimized protocol. Overall image quality, motion artifact, liver edge sharpness and on arterial phase and portal-venous phase were evaluated by two radiologists on a 4-point score. We also evaluated signal intensity ratio of liver at late arterial phase to aorta at peak enhancement and detection rate of hypervasular lesion.
**Results or Findings:** Results: Overall image quality score, artifact score and liver edge sharpness of XD-VIBE and GRASP on arterial and portal phase was not significantly different(p=0.070-0.397). Four(reviewer1, 12.1%) and seven patients (reviewer2, 21.2%) were received score 1 (nondiagnostic quality and reexamination needed) in XD-VIBE group whereas one patient (reviewer2, 2.9%) was received score 1 in GRASP group. Ratio of aorta and liver of GRASP was significantly higher than that of XD-VIBE (0.32±0.10 vs 0.47±0.13). The hypervascular lesion detection rate of XD-VIBE (86.7 %) was higher than GRASP (57.1%) on arterial phase without statistical significant difference(p=0.081).
**Conclusion:** Overall image quality, motion artifact and liver edge sharpness on arterial and portal-venous phase of XD-VIBE and GRASP was not significantly different, although XD-VIBE could be seen non-diagnostic quality image than GRASP. In the case of GRASP, the degree of enhancement of liver parenchyma on late arterial phase compared to aorta could be higher than XD-VIBE and this has the potential to reduce detection of hypervascular lesion on arterial phase.
**Limitations:** First, this was retrospective study.
**Ethics Committee Approval:** This retrospective study approved IRB.
**Funding for this study:** None
**Author Disclosures:**
Jin Sil Kim: nothing to disclose
Ewha Womans University Eun Sun Choi: nothing to disclose

### Research Presentation Session: Breast

### RPS 302

### Breast MRI

### RPS 302-1

**Intra- and inter-reader agreement of diffusion-weighted magnetic resonance imaging for breast cancer detection: a multi-reader study**
*M. Montesano*, F. Pesapane, A. Rotili, S. Penco, L. Nicosia, V. Dominelli, C. Trentin, G. M. Agazzi, E. Cassano; Milan/IT
*(marta.mon@hotmail.it)*

**Purpose:** In order to utilize DWI as a reliable biomarker for detecting breast cancer through unenhanced MRI, we evaluated the inter-reader reproducibility and the inter-reader agreement between high-experience and low-experience readers.
**Methods or Background:** Consecutive breast MRI performed, in a single centre, from February to September 2016, were retrospectively evaluated. Exclusion criteria included: bilateral breast implants, absence of DWI sequences in the acquisition protocol, breast surgery performed in other hospitals and lack of regular follow-up. Four radiologists, divided in two groups according to the different experience, independently evaluated only DWY and ADC maps. Histopathology results or >1-year imaging follow-up was used as reference standard. Agreement was assessed through the calculation of intra-reader and inter-reader reproducibility using Cohen and Fleiss K statistics.
**Results or Findings:** Out of 1,131 examinations, 378 women (49±11 years old) were included, 13% (51/1131) with unilateral mastectomy, totalling 705 breasts. Intra-reader reproducibility was moderate to high in all readers (K=0.63–0.77) reflecting the overall proportion of lesions with concordant scoring. High-experience readers (R1, R2) showed specific agreement occurring in 90.7% (656/724) while low-experience readers (R3, R4) in 50.1% (368/724) of breasts. We reported higher intra-reader agreement for high-experience readers (κ=0.72–0.78) compared to low-experience readers (κ=0.48–0.69). Inter-reader agreement for all readers was moderate (κ=0.56) while it was substantial (κ=0.74) for high-experience readers and fair (κ=0.37) for low-experience readers. Pairwise agreement between low-experience readers and high-experience readers was moderate (κ=0.60) demonstrated superior scoring agreement between high-experience readers (p=0.003).
**Conclusion:** Breast MR based on DWI has a good reproducibility but may be assessed only by dedicated radiologists in referral/specialized centers.
**Limitations:** The inherent retrospective and monocentric nature of the study and the lack of independence from individual variability of the results.
**Ethics Committee Approval:** IRB approved.
**Funding for this study:** No funds.
**Author Disclosures:**
Chiara Trentin: Nothing to disclose
Anna Rotili: Nothing to disclose
Luca Nicosia: Nothing to disclose
Silvia Penco: Nothing to disclose
Valeria Dominelli: Nothing to disclose
Filippo Pesapane: Nothing to disclose
Enrico Cassano: Nothing to disclose
Giorgio Maria Agazzi: Nothing to disclose
Marta Montesano: Nothing to disclose

### RPS 302-2

**Dedicated axillary MRI-based radiomics analysis for the prediction of axillary lymph node metastasis in breast cancer**
*R. Granzier*[1], S. Samiei[1], A. Ibrahim[1], S. Primakov[1], M. B. I. Lobbes[1], R. G. H. Beets-Tan[2], T. van Nijnatten[1], H. C. Woodruff[1], M. Smidt[1]; [1]Maastricht/NL, [2]Amsterdam/NL
*(r.granzier@maastrichtuniversity.nl)*

**Purpose:** To predict preoperative axillary lymph node metastasis in breast cancer patients using clinical models and radiomics models based on unenhanced T2-weighted (T2W) dedicated axillary MRI features with node-by-node analysis
**Methods or Background:** From August 2012 until October 2014, women who had undergone a dedicated axillary T2W MRI(3.0T), followed by axillary surgery, were retrospectively identified, and available clinical data were collected. All axillary lymph nodes were manually delineated on the T2W MR images, and quantitative radiomics features were extracted from the delineated regions. The data was partitioned patient-wise to train 100 models using different splits for training and validation cohorts to account for multiple lymph nodes per patient and class imbalance. Features were selected in training cohorts using recursive feature elimination with repeated 5-fold cross-validation, followed by the development of random forest models. The performance of the models was assessed using the area under the receiver operating characteristic curve (AUC).
**Results or Findings:** 75 women with 511 axillary lymph nodes were included. On final pathology, 36 of the lymph nodes had metastasis. A total of 105 original radiomics features were extracted from the T2W MR images. Each cohort split resulted in a different number of lymph nodes in the training cohorts and a different set of selected features. The performance of the 100 clinical and radiomics models showed a wide range of AUC values between 0.41-0.74 and 0.48-0.89 in the training cohorts, respectively, and between 0.30-0.98 and 0.37-0.99 in the validation cohorts, respectively. With these results, it was not possible to obtain a final prediction model.
**Conclusion:** Clinical characteristics and dedicated axillary MRI-based radiomics with node-by-node analysis did not contribute to the prediction of axillary lymph node metastasis in breast cancer.
**Limitations:** Skewed data, manual delineation
**Ethics Committee Approval:** Approved
**Funding for this study:** Not applicable

**Author Disclosures:**
Marjolein Smidt: Nothing to disclose
Marc B I Lobbes: Nothing to disclose
Sanaz Samiei: Nothing to disclose
Henry Christian Woodruff: Nothing to disclose
Abdalla Ibrahim: Nothing to disclose
Regina G. H. Beets-Tan: Nothing to disclose
Sergey Primakov: Nothing to disclose
Thiemo van Nijnatten: Nothing to disclose
Renée Granzier: Nothing to disclose

### RPS 302-4

**Potential of MR lymphography for lymph nodes (LN) staging in breast cancer**

A. Christenhusz, F. Simonis, N. de Vries, M. Hofman, A. Dassen, B. ten Haken, *L. Alic*; Enschede/NL
*(l.alic@utwente.nl)*

**Purpose:** Differentiation between healthy and malignant lymph nodes in breast cancer
**Methods or Background:** Magnetic nanoparticles (MNs) enhanced MR lymphography is used for a minimally invasive preoperative detection of lymph nodes (LN). However, 80% of surgically dissected LNs are negative for metastases. This study assesses the potential of MN-enhanced MR lymphography for preoperative LN staging. Sixteen invasive breast cancer patients (8 with and 8 without metastases) were included in this study. Prior to ex vivo MRI, the patients received an intra-tumoral MN-injection containing 1.1-4.4 mg iron oxide. MRI settings include: T1-weighted (T1w,TR/TE = 12.74/5.86ms) turbo field echo and T2-weighted (T2w, TR/TE = 2500/80ms) spin echo sequences. All SLNs were pathologically processed and H&E stained using a 5 level step-serial sectioning (200 μm intervals). Healthy and malignant LNs were classified using signal intensities of ex vivo T1w and T2w scans quantified by four first order statistical (FOS) features (mean, median, skewness, entropy), five morphological features (volume, eccentricity, curvature, circularity, spiculation), and four co-occurrence texture features (variance, correlation, angular moment, inverse moment). The significance was assessed using student's t-test or Mann-Whitney U test. The four most significant features were used in a multiple discriminant analysis (MDA).
**Results or Findings:** Statistical significance in classifying healthy and metastatic LN was reached by texture feature (variance) based upon solely T1w-images, and by FOS features (median) and morphological (eccentricity) features based upon solely T2w-images. MDA revealed a prediction sensitivity of 85.7% and specificity of 100%.
**Conclusion:** MN-enhanced MR lymphography provides sufficient classification accuracy to discriminate between healthy and metastatic LNs.
**Limitations:** The limitations include the relatively low number of patients included, limited spatial resolution and the non- quantitative character of MRI sequences.
**Ethics Committee Approval:** Ethical committee approval (NL49285.044.14) registered in the Dutch Trial Register (NTR4903).
**Funding for this study:** InMediValue (under award number 122207)
**Author Disclosures:**
Anke Christenhusz: Nothing to disclose
Marieke Hofman: Nothing to disclose
Lejla Alic: Nothing to disclose
Anneriet Dassen: nothing to disclose
Bennie ten Haken: Nothing to disclose
Nynke de Vries: Nothing to disclose
Frank Simonis: Nothing to disclose

### RPS 302-5

**MRI-derived tumour-to-breast volume is associated with the extent of breast surgery**

*A. Cozzi*, V. Magni, G. Della Pepa, S. Carriero, D. Spinelli, L. A. Carbonaro, S. Schiaffino, F. Sardanelli; Milan/IT
*(andrea.cozzi@gmail.com)*

**Purpose:** To assess the correlation between magnetic resonance imaging (MRI)-derived tumour-to-breast volume ratio (TBVR) and the performed surgical strategy (breast-conserving surgery versus mastectomy).
**Methods or Background:** All patients who had a biopsy-proven diagnosis of malignancy and who underwent pre-operative breast MRI demonstrating a mass-like lesion between January 1st, 2013 and May 31st, 2019, were retrieved. TBVR was obtained using a fully manual method for segmenting the tumour volume (TV) and a growing region semiautomatic method for segmenting the whole breast volume (WBV). Two specifically trained residents (R1 and R2), each of them with 2 years of breast MRI experience, independently segmented T1-weighted images in 51 cancer cases in 51 patients (median age 57 years). Segmentation times and interobserver TBVR reproducibility was calculated. Mann-Whitney U test, Spearman correlation, and Bland-Altman statistics were used.
**Results or Findings:** Breast conserving surgery was the surgical choice in 31/51 (61%) patients, while 20/51 (39%) underwent mastectomy of the affected breast. Strong correlations between TV and final pathology were found (Reader 1 $\rho=0.748$, $p<0.001$; Reader 2 $\rho=0.778$, $p<0.001$). Median TBVR was 2.08‰ (interquartile range 0.70‰–9.13‰) for Reader 1, 2.28‰ (interquartile range 0.71‰–9.61‰) for Reader 2, with an 84% inter-reader reproducibility. Median segmentation times were 54 s for WBV and 141 s for TV. Significantly lower TBVR values were observed in the breast conserving surgery group (median 1.14‰, interquartile range 0.49‰–2.55‰) than in the mastectomy group (median 10.52‰, interquartile range 2.42‰–14.73‰) for both readers ($p<0.001$).
**Conclusion:** TBVR and its related metrics can be obtained from breast MRI with good to high inter-reader reproducibility, also demonstrating a strong direct relation between TBVR and subsequent surgical planning.
**Limitations:** Single center, retrospective study, limited sample size, limited experience of the two readers.
**Ethics Committee Approval:** Obtained (July 18th, 2019).
**Funding for this study:** None.
**Author Disclosures:**
Gianmarco Della Pepa: Nothing to disclose
Andrea Cozzi: Nothing to disclose
Francesco Sardanelli: Grant Recipient: Bayer Healthcare Research/Grant Support: General Electric Healthcare Speaker: Bracco Group
Serena Carriero: Nothing to disclose
Diana Spinelli: Nothing to disclose
Simone Schiaffino: Speaker: General Electric Healthcare Other: Bracco Imaging
Luca Alessandro Alessandro Carbonaro: Nothing to disclose
Veronica Magni: Nothing to disclose

### RPS 302-6

**Towards contrast-free perfusion imaging in breast: feasibility of velocity-selective arterial spin labeling in breast cancer patients**

*S. L. Franklin*[1], H. Dankers[1], C. Bos[2], M. van Osch[1], N. Voormolen[1]; [1]Leiden/NL, [2]Utrecht/NL
*(s.l.franklin@lumc.nl)*

**Purpose:** Breast MRI is a sensitive and specific tool in detection of breast cancer. Therefore, breast MRI is extremely valuable in breast cancer screening, especially in women at increased risk. Dynamic contrast-enhanced (DCE-) MRI after administration of intravenous gadolinium-based contrast agents (GBCA) is an essential component of multi-parametric breast MR. However there are concerns related to repeated GBCA administration, e.g. retention, patient discomfort and cost. Velocity Selective Arterial Spin Labeling (VS-ASL) provides a measure of perfusion and vascularity without requiring GBCAs. VS-ASL is more suitable for the breast than traditional ASL-methods since it can deal with slow blood flow and a complicated vascular bed by labeling blood directly in the region of interest.
**Methods or Background:** In this study ten patients (3 screening and 7 staging) received a VS-ASL scan in addition to a regular MR-protocol including ultrafast DCE-MRI and DWI. Scans were read by an experienced breast radiologist, and the relative VS-ASL signal increase at the tumor location was calculated.
**Results or Findings:** All five mass lesions showed a clear perfusion increase on VS-ASL, anatomically concordant with perfusion on ultrafast DCE-MR within 10 seconds after enhancement of the mammary artery. Two out of three non-mass lesions showed a moderate perfusion increase on VS-ASL,one did not. Interestingly, some scans showed a perfusion increase in a segmental pattern leading towards the tumor location in an adjacent slice, suggesting that VS-ASL may provide additional information than is currently available.
**Conclusion:** Concluding, VS-ASL is a very promising technique for a GBCA-free multi-parametric breast MRI protocol, adding specific vascular information.
**Limitations:** Small group-size, the technique needs optimizations to deal with dense breast better.
**Ethics Committee Approval:** This study was approved by the local ethics committee and informed consent was obtained from all participants.
**Funding for this study:** NWO ttw
**Author Disclosures:**
Suzanne Lisa Franklin: Nothing to disclose
Clemens Bos: Nothing to disclose
Matthias van Osch: Nothing to disclose
Nora Voormolen: Nothing to disclose
Henrike Dankers: Nothing to disclose

### RPS 302-7

**Apparent diffusion coefficient mapping vs. the Kaiser score in the assessment of breast lesions: a multicentre comparison**

*M. Dietzel*[1], K. B. Krug[2], P. Clauser[3], M. Hellmich[2], D. Maintz[2], M. Uder[1], H. Bickel[3], T. H. Helbich[3], P. A. Baltzer[3]; [1]Erlangen/DE, [2]Cologne/DE, [3]Vienna/AT

**Purpose:** We compared the diagnostic-performance of apparent-diffusion-coefficient (ADC) mapping with the Kaiser-score (KS) .
**Methods or Background:** In this IRB approved multicentric study, consecutive patients from three different centers receiving standardized multiparametric breast-MRI for standard nonscreening-indications were included. Experienced radiologists retrospectively interpreted the examinations in consensus and applied the KS to every histologically verified lesion. Corresponding mean-ADC was measured (Wielema 4° region-of-interest). KS and ADC were combined, yielding the KS+ score as described previously. Diagnostic-accuracy was evaluated by the area-under-the-receiver-operating characteristics curve (ROC) and compared (KS vs. ADC vs. KS+; DeLong-test). Likewise the potential to avoid unnecessary biopsies was compared based on established high sensitivity thresholds (McNemar-test).
**Results or Findings:** 450 lesions in 414 patients (mean-age: 51.5 years, interquartile-range: 42–60.8 years) were included, with 219 lesions being malignant [48.7%; 95%confidence-interval (CI): 44%–53.4%]. The KS (ROC: 0.915, CI: 0.886–0.939) outperformed the ADC (ROC: 0.848; CI: 0.811–0.880; P<0.001). The use of the KS+ (ROC: 0.918; CI: 0.889–0.942) improved the performance slightly, but without significant difference to a single KS or ADC reading (P=0.64). When applying high sensitivity thresholds for avoiding unnecessary biopsies, the KS and ADC achieved equal sensitivity (97.7% for both; cutoff values: >4 for KS, ≤1.4×10–3mm2/s for ADC). However, the rate of potentially avoidable biopsies was higher when using the KS (specificity: 65.4% for KS vs. 32.9% for ADC; P < 0.0001). The KS was superior to the KS+ in avoiding unnecessary biopsies.
**Conclusion:** The KS outperformed the ADC in distinguishing benign from malignant breast lesions. The KS may avoid twice as many unnecessary biopsies. The combination of KS and ADC does not improve diagnostic-performance.
**Limitations:** Our data requires prospective validation, with special focus to clinical context (high risk? background-enhancement?) and technical developments (new pulse-sequences? artificial intelligence?).
**Ethics Committee Approval:** n/a
**Funding for this study:** n/a
**Author Disclosures:**
Pascal A.T. Baltzer: Nothing to disclose
Hubert Bickel: Nothing to disclose
David Maintz: Nothing to disclose
Thomas H. Helbich: Nothing to disclose
Matthias Dietzel: Nothing to disclose
Michael Uder: Nothing to disclose
Martin Hellmich: nothing to disclose
Kathrin Barbara Krug: Nothing to disclose
Paola Clauser: Nothing to disclose

### RPS 302-8

**The Kaiser score helps residents outperforming specialists in interpreting breast MRI**

*N. Pötsch*, A. Korajac, P. D. Stelzer, P. Kapetas, R-I. Milos, T. H. Helbich, P. Clauser, P. A. Baltzer; Vienna/AT

**Purpose:** Due to its high sensitivity, DCE MRI of the breast (bMRI) is increasingly used for both screening and assessment purposes. Differentiating benign from malignant enhancements, even in the hands of experts, remains challenging. The “Kaiser Score” (KS) was shown to facilitate characterization of enhancing breast lesion, potentially avoiding misdiagnosis and unnecessary biopsies. The aim was to evaluate the diagnostic performance of residents using the KS compared to expert radiologists in interpreting bMRI.
**Methods or Background:** Three board certified radiologists, with more than 15 years of experience, interpreted bMRI according to the five-point BI-RADS scale. The same studies were read by three radiology residents, without specific training in breast imaging, using the KS. To ensure comparability, the residents were aware of the location of the lesions rated by the experts. All readers were blinded to clinical information. Histology was used as gold standard. Ratings were dichotomized assigning (i) BI-RADS 1-3 corresponding to a KS 1-4 to benign and (ii) BI-RADS 4 and 5 related to a KS 5-12 to suspicious. Statistical analysis was done by comparing the AUC of the ROC curves.
**Results or Findings:** A total of 80 women (mean 54.3 ± 11.2 years) with 93 lesions (32 benign, 61 malignant) were included. There was no significant difference in the performance between the three expert readers (AUC 0.723-0.742, p>0.72) and a borderline significance in the performance in-between the three residents (AUC 0.842-0.928, p=0.03 resp. p>0.08). The rating of each resident using the KS significantly outperformed the experts' ratings using the BI-RADS scale (p <0.01 – 0.05).
**Conclusion:** Using the KS allowed untrained residents to achieve better results in interpreting bMRI than expert readers using BI-RADS.
**Limitations:** Retrospective analysis, cancer enriched database.
**Ethics Committee Approval:** Approved by local ethics committee.
**Funding for this study:** None.
**Author Disclosures:**
Pascal A.T. Baltzer: Nothing to disclose
Nina Pötsch: Nothing to disclose
Herr Philipp Dominik Stelzer: Nothing to disclose
Aida Korajac: Nothing to disclose
Thomas H. Helbich: Nothing to disclose
Ruxandra-Iulia Milos: Nothing to disclose
Panagiotis Kapetas: Nothing to disclose
Paola Clauser: Nothing to disclose

### RPS 302-9

**Multidimensional diffusion MRI of breast tumours: initial findings**

*I. Daimiel Naranjo*[1], A. Reymbaut[2], P. Brynolfsson[2], S. Thakur[1], R. Lo Gullo[1], D. Topgaard[2], E. A. Morris[1], K. Pinker-Domenig[1]; [1]New York, NY/US, [2]Lund/SE

**Purpose:** To apply multidimensional diffusion (MDD) MRI to breast tumors, quantifying their composition through imaging biomarkers related to cell densities, shapes, and orientations.
**Methods or Background:** Sixteen patients with suspected breast tumors consented to undergo a breast-MRI including MDD using a customized spin-echo EPI sequence performed on a GE MR750 3T scanner with 16-channel breast coils. Histopathology was used as gold standard yielding 1 invasive lobular carcinoma, 2 ductal carcinomas in situ, 8 invasive ductal carcinomas (IDC) and 5 IDCs with extensive in situ component. Per-case parameter maps were generated featuring MDD-derived metrics such as “mean size” E[Diso] (comparable to conventional ADC), “mean shape” E[D∆2] (measuring microscopic anisotropy) and signal fractions from tissue-specific bins associated with elongated cells (fbin1), isotropic cells (fbin2) and free water (fbin3). Tumor ROIs were also drawn in glandular tissue using b=700s/mm2 MDD images (Sb700).
**Results or Findings:** Cancers were characterized by low E[Diso], and high Sb700, E[D∆2], and fbin1. Tumor-ROI analysis yielded a 10th percentile of ADC=1±0.46 x10-3mm2/s, in agreement with literature. It also gave E[D∆2]=0.47±0.15 and fbin1=0.53±0.27 significantly different than values in glandular tissue, E[D∆2]=0.27±0.11 and fbin1=0.17±0.14, respectively (Mann-Whitney U-test, p<0.001). Importantly, tumors were invisible in fractional anisotropy (FA) maps, because they lack orientational coherence (quantified by orientational order parameter OP).
**Conclusion:** MDD could distinguish breast cancer from glandular tissue within feasible scan times, providing both conventional ADC consistent with literature and novel metrics reporting on specific microstructural tissue features. Further studies evaluating correlation with histopathology/immunohistochemistry are guaranteed.
**Limitations:** Small number of cases prevented detailed correlation of conventional and MDD-derived metrics with tumor grade/molecular profile.
**Ethics Committee Approval:** Memorial Sloan Kettering IRB-approved.
**Funding for this study:** NIH/NCI Cancer Center Support Grant(P30CA008748), Breast Cancer Research Foundation, Swedish Foundation for Strategic Research (ITM17-0267), Swedish Research Council (2018-03697) and Foundation Alfonso Martin Escudero.
**Author Disclosures:**
Katja Pinker-Domenig: Nothing to disclose
Elizabeth A. Morris: Nothing to disclose
Patrik Brynolfsson: Employee: RWI holds patents related to the described methods
Roberto Lo Gullo: Nothing to disclose
Sunitha Thakur: Nothing to disclose
Daniel Topgaard: Shareholder: RWI
Isaac Daimiel Naranjo: Nothing to disclose
Alexis Reymbaut: Employee: RWI holds patents related to the described methods

### RPS 302-10

**Positive MRI findings in breast cancer patients with pathologic complete response after neoadjuvant chemotherapy**

*S. Erdemli*[1], F. Taşkin[1], İ. H. Erdoğdu[2], F. Tokat[1], K. Behzatoğlu[1], N. Meydan[2], L. Özer[1], A. Arıcan[1], C. Uras[1]; [1]Istanbul/TR, [2]Aydın/TR
*(srvterdemli@gmail.com)*

**Purpose:** To evaluate the MRI findings of breast cancer patients received neoadjuvant chemotherapy(NAC) with radiologic partial response(RPR) and pathologic complete response(PCR)

**Methods or Background:** In this two-center, retrospective study, imaging and histopathology records of breast cancer patients with RPR and PCR after NAC were evaluated. MRI interpretation included lesion morphology at baseline, changes in morphology, size, and contrast uptake kinetics. PCR was defined as the absence of invasive tumor cells in the resected breast specimen.
**Results or Findings:** MRI examinations were performed in 108 women before and after NAC. RPR were found in 72 HER-2-positive (66.7%:39 hormone receptor (HR) positive, 33 HR negative), 21 (19.4%) HR-positive/HER-2-negative, and 15 (13.9%) triple-negative (TN) breast cancer patients. Findings of MRI after NAC were 53 (49%) non-mass enhancement (NME), 39 (36%) foci, and 16 (%15) masses. Ductal carcinoma in situ (DCIS), lobular carcinoma in situ, sclerosing adenosis, and fibrosis were the most common lesions found in specimens. The most common MRI findings were foci in HER-2-positive disease and NME in HR-positive and TN disease.
**Conclusion:** While interpreting MRI after NAC in breast cancer patients, the presence of contrast enhancement in the original tumor region is considered as RPR. In PCR cases, positive MRI findings can be related to the presence of DCIS or other benign proliferative conditions in the original tumor region.
**Limitations:** The retrospective nature and the limited number of patients are the major limitations of our study.
**Ethics Committee Approval:** We applied for institutional ethics committee approval.
**Funding for this study:** No funding was received for this work.
**Author Disclosures:**
İbrahim Halil Erdoğdu: Nothing to disclose
Servet Erdemli: Nothing to disclose
Cihan Uras: Nothing to disclose
Kemal Behzatoğlu: Nothing to disclose
Füsun Taşkin: Nothing to disclose
Leyla Özer: Nothing to disclose
Ali Arıcan: Nothing to disclose
Nezih Meydan: Nothing to disclose
Fatma Tokat: Nothing to disclose

### RPS 302-11

**Feasibility of computer-assisted triaging of MRI-guided biopsy in preoperative breast-cancer patients**

*H. Wang*, B. van der Velden, M. A. A. Ragusi, W. B. Veldhuis, M. Viergever, E. Verburg, K. Gilhuijs; Utrecht/NL

**Purpose:** Additional breast lesions (i.e., incidental lesions in addition to the index cancer) detected on MRI pose challenges in the preoperative workup of patients with early breast cancer. Our aim is to assess the sensitivity of computer-assisted triaging of patients with additional breast lesions on preoperative breast MRI to MRI-guided breast biopsy with high specificity.
**Methods or Background:** We investigated 316 consecutive patients with early breast cancer who received preoperative multiparametric breast MRI between 2013 and 2016, 82 patients had 101 additional lesions (51 were benign and 50 were malignant). We collected 4 clinical features, and 46 MRI radiomic features from T1-weighted dynamic contrast-enhanced (DCE) imaging, high-temporal-resolution DCE, T2-weighted, and diffusion-weighted imaging. A multiparametric computer-aided diagnosis (CAD) model was constructed from this training set using 10-fold cross validated ridge regression. The sensitivity was calculated at operating point corresponding to 98% specificity. The model calibration performance was evaluated by calibration-plot analysis and goodness-of-fit tests. The model was tested in an independent cohort of 187 consecutive patients from 2017 and 2018. In this testing cohort, 45 patients had 55 additional lesions (23 were benign and 32 were malignant).
**Results or Findings:** The multiparametric CAD identified 48% of the malignant additional lesions with specificity of 98%. Calibration analysis indicated the model was well fitted. In the testing cohort, the specificity was 96% and sensitivity 44% at the operating point of the training set.
**Conclusion:** Our multiparametric CAD demonstrated potential to triage patients to MRI-guided breast biopsy. Malignant additional lesions were identified with near-perfect specificity in approximately half the population of preoperative patients originally indicated for a breast biopsy. In the remaining half, patients would still continue to MRI-guided biopsy to confirm absence of malignancy.
**Limitations:** No external validation
**Ethics Committee Approval:** Ethical review waived
**Funding for this study:** None
**Author Disclosures:**
Hui Wang: Nothing to disclose
Kenneth Gilhuijs: Nothing to disclose
Max Viergever: Nothing to disclose
Bas van der Velden: Nothing to disclose
Wouter B. Veldhuis: Nothing to disclose
Erik Verburg: Nothing to disclose
Max A A Ragusi: Nothing to disclose

### RPS 302-12

**Breast MR biomarkers in benign and malignant tumours of the diseased and contralateral healthy breast**

R. E. Ochoa Albíztegui[1], J. V. M. Horvat[1], S. Thakur[1], I. Daimiel Naranjo[1], R. Lo Gullo[1], E. A. Morris[1], T. H. Helbich[2], *K. Pinker-Domenig*[1]; [1]New York, NY/US, [2]Vienna/AT
*(eochoa.albiztegui@gmail.com)*

**Purpose:** To assess if imaging biomarkers at dynamic contrast-enhanced magnetic resonance imaging (DCE-MRI) are different for the contralateral healthy breast than the diseased breast in patients with benign and malignant tumors.
**Methods or Background:** One hundred consecutive patients who underwent DCE-MRI of the breast for the assessment of a BI-RADS 4-5 lesion on conventional image were included in this IRB approved study. Biomarkers for healthy and diseased breast were evaluated by two independent readers and recorded as BPE (background parenchymal enhancement) and fibroglandular tissue (FGT). Histopathology was used as standard of reference. Appropriate statistics were used to assess the difference between biomarkers on patients with benign and malignant lesions.
**Results or Findings:** There were 33 benign and 67 malignant breast lesions. In patients with malignant tumors, BPE was minimal in 42/51, mild in 16/33, moderate 6/11, and marked 3/5. In patients with benign tumors, BPE was 9/51, mild in 17/33, moderate 5/11, and marked 2/5. BPE of contralateral healthy breast (p=0.01) and BPE of diseased breast (p=<0.01) was statistically significantly different between benign and malignant tumors. FGT of contralateral healthy breast (p=0.26) and diseased breast (p=0.19) showed no difference between benign and malignant tumors.
**Conclusion:** BPE is decreased in patients with malignant tumors in the contralateral healthy breast. There is no difference in FGT of contralateral healthy breast in patients with benign and malignant tumors. BPE in both breasts (disease and healthy breast) is significantly different in malignant and benign lesions.
**Limitations:** Patients were from a single center.
**Ethics Committee Approval:** Yes, Approved.
**Funding for this study:** The Breast Cancer Research Foundation, and Susan G. Komen
**Author Disclosures:**
Joao Vicente Machado Horvat: nothing to disclose
Katja Pinker-Domenig: nothing to disclose
Thomas H. Helbich: nothing to disclose
Elizabeth A. Morris: nothing to disclose
Roberto Lo Gullo: nothing to disclose
Sunitha Thakur: nothing to disclose
Isaac Daimiel Naranjo: nothing to disclose
R. Elena Ochoa Albíztegui: nothing to disclose

### RPS 302-14

**Adding b2500 diffusion-weighted imaging to BI-RADS improves specificity of breast MRI**

*L. Saccenti*, Z. Jolibois, M. Scholer, C. De Bazelaire; Paris/FR

**Purpose:** Evaluation of diagnostic performances of visual b2500 DWI assessment in addition to conventional breast MRI protocol
**Methods or Background:** This single-institution retrospective study included patients who underwent a breast MRI examination on a 1.5T system from May 2017 to February 2020. The examination was composed of a conventional protocol (T1 and T2-weighted sequences, dynamic-contrast enhanced imaging and b0-b800 DWI) and of a b2500 DWI sequence. Lesions were classified based on the conventional protocol using BI-RADS categories. 3 independent radiologists then assessed visually b2500 DW images. The diagnostic performances of b2500 DWI assessment, BI-RADS categories, ADC value and of a model combining b2500 DWI assessment and BI-RADS categories, were evaluated by using ROC analysis. Specificity and sensitivity were computed.
**Results or Findings:** 260 patients with 212 malignant and 100 benign lesions were included. Interobserver agreement for b2500 DWI assessment was substantial (Fleiss Kappa = 0.77). Area under the curve (AUC) of ROC curve of the model combining b2500 DWI and BI-RADS was 0.84 versus 0.81 for BI-RADS alone (P=0.34). Specificity of the combined model was higher than BI-RADS alone (73% versus 25%, P<0.001) but sensibility was lower (94% versus 100%, P<0.001). AUC of b2500 DWI was higher (0.81 than AUC of ADC (0.71, P=0.14). Specificity of b2500 DWI was higher than ADC (69% versus 48%, P<0.001) but sensibility was lower (93% versus 94%, P=0.04).
**Conclusion:** Visual assessment of b2500 DWI has substantial interobserver agreement. Combining b2500 DWI assessment to BI-RADS categories significantly improves specificity of breast MRI compared to BI-RADS categories alone. Visual assessment of B2500 DWI also presents higher specificity than ADC values.
**Limitations:** Only patients with histopathologic results were included
Single-institution study with over representation of high-risk patients

**Ethics Committee Approval:** French Ethics Committee for Research in Imaging (CRM-2005-090)
**Funding for this study:** None
**Author Disclosures:**
Margaux Scholer: Nothing to disclose
Zoe Jolibois: Nothing to disclose
Laetitia Saccenti: Nothing to disclose
Cedric De Bazelaire: Nothing to disclose

### RPS 302-15

**Evaluation of high-risk (B3) breast lesions on MRI: the role of conventional and texture analysis features in predicting upgrade to malignancy**

*T. Aybal*[1], O. Buğdaycı[1], M. E. Aribal[1], H. Kaya[1], M. U. Ugurlu[1], C. Ilgin[1], B. Ö. Demirci[2]; [1]Istanbul/TR, [2]Siirt/TR
*(tahsinaybal89@gmail.com)*

**Purpose:** To investigate the potential of malignancy associated with high-risk breast lesions using breast MRI characteristics, ADC measurements and texture analysis parameters.
**Methods or Background:** Our retrospective study included 40 patients with 41 lesions who were diagnosed with a high-risk lesion after needle biopsy. All patients underwent surgery. According to histopathological results of surgical excision, patients were divided into two groups as those diagnosed with malignancy and those who did not. MRI characteristics of the breast and the lesions (breast pattern, background parenchymal enhancement, lesion type and size, lesion kinetic pattern) were recorded. ADC values of the lesions were measured. Texture analysis of lesions was performed.
**Results or Findings:** Fourteen lesions (34.1%) were upgraded to malignancy. The median ADCmean values in the group with malignancy were 1.114 x 10-3 mm2/s vs 1.383 x 10-3 mm2/s in the non-malignant group which was statistically significant (p <0.001). When the cut-off value for the mean ADC was taken as 1.163 x 10-3 mm2/s, the sensitivity and specificity were % 71.4 and % 85.2, respectively. The AUC value of ADCmean was calculated as 0.844 by ROC analysis. Among the texture analysis parameters, kurtosis values obtained from images on the ADC map and first subtracted dynamic contrast-enhanced (DCE) series and contrast values obtained from images on the second subtracted DCE series were found to be statistically significant (p = 0.016 ; p = 0.019 and p= 0.045 respectively) between malignant and non-malignant groups.
**Conclusion:** ADC measurements and texture analysis parameters provide useful diagnostic information in determining which high-risk breast lesions will upgrade to malignancy.
**Limitations:** Retrospective study and low number of samples
**Ethics Committee Approval:** The local Medical Ethical Committee permission (09.2019.255) was obtained.
**Funding for this study:** No funding was received for this work.
**Author Disclosures:**
Mustafa Erkin Aribal: Nothing to disclose
Handan Kaya: Nothing to disclose
Mustafa Umit Ugurlu: Nothing to disclose
Can Ilgin: Nothing to disclose
Onur Buğdaycı: Nothing to disclose
Burcu Özdemir Demirci: Nothing to disclose
Tahsin Aybal: Nothing to disclose

### RPS 302-17

**Associations of non-contrast breast MRI biomarkers with benign and malignant breast tumours**

R. E. Ochoa Albíztegui[1], J. V. M. Horvat[1], S. Thakur[1], I. Daimiel Naranjo[1], R. Lo Gullo[1], E. A. Morris[1], T. H. Helbich[2], *K. Pinker-Domenig*[1]; [1]New York, NY/US, [2]Vienna/AT
*(eochoa.albiztegui@gmail.com)*

**Purpose:** To investigate non-contrast MRI biomarkers and their association with breast tumors.
**Methods or Background:** Two readers independently evaluated 100 consecutive patients who underwent multiparametric breast MRI at 3T with DCE and DWI for the assessment of a BI-RADS 4/5 lesion. Breast tumors were assessed for localization (in FGT, in fat or at the fat/FGT plane), T2 signal intensity, presence of tumoral edema and DWI signal intensity pattern (homogeneous, heterogeneous, rim). The amount of fibroglandular tissue (FGT) and background parenchymal enhancement (BPE) was recorded. Appropriate statistics to determine associations of un-enhanced MRI biomarkers with malignancy were performed.
**Results or Findings:** There were 33 benign and 67 malignant lesions. Malignant and benign tumors localization was significantly different p=<0.01. Malignant tumors were less likely localized as the FGT (n=2, 2.9%) and more likely localized in fat (n=57, 85%) or at the fat/FAT plane (n=8, 11.9%). Odds Ratio (OR) of malignant tumor localization in fat is 19.99 (p=<0.01) and fat/FG plane 13.57 (p=<0.01). Malignant tumors showed a rim pattern OR 14.7 (n=10, 14.9%) and an heterogeneous DWI pattern OR 8.4 (n=40, 59%) rather than an homogeneous DWI pattern (n=17, 25%). Peritumoral edema was skin in 30 (44.77%) of malignant tumors OR 12 (95%CI 2-56) p=<0.01 and in only 2 (6.25%) of benign tumors. Both benign (78%) and malignant (86%) tumors presented mostly with minimal/mild BPE (p=0.010). FGT was not different between benign and malignant tumors (p=0.186).
**Conclusion:** Malignant tumors are more likely to be located at the FGT/fat plane presenting with peritumoral edema and DWI heterogeneous or rim signal intensity pattern than benign tumors.
**Limitations:** Single center study.
**Ethics Committee Approval:** Yes.
**Funding for this study:** The Breast Cancer Research Foundation, and Susan G. Komen
**Author Disclosures:**
Joao Vicente Machado Horvat: Nothing to disclose
Katja Pinker-Domenig: Nothing to disclose
Thomas H. Helbich: nothing to disclose
Elizabeth A. Morris: Nothing to disclose
Roberto Lo Gullo: Nothing to disclose
Sunitha Thakur: Nothing to disclose
Isaac Daimiel Naranjo: Nothing to disclose
R. Elena Ochoa Albíztegui: Nothing to disclose

### RPS 302-18

**Feasibility study of 2D MR fingerprinting (MRF) of breast cancer**

*E. Zanderigo*, T. Nolte, M. Broeckmann, D. Truhn, V. Schulz, C. K. Kuhl; Aachen/DE
*(ezanderigo@ukaachen.de)*

**Purpose:** To establish and validate 2D blurring-corrected magnetic resonance fingerprinting (MRF) of breast cancer and normal fibroglandular tissue (FGT).
**Methods or Background:** On a clinical 1.5T system, 14 women with breast cancer underwent a standardized multiparametric MRI protocol including a dynamic series before and 4 times after 0.1mmol/kg-BW Gadobutrol. Before and after the dynamic series, a single-slice 2D MRF sequence was performed over the area of the breast cancer. Three MRF trains with a length of 500 TR intervals, variable flip angles, constant TR=21 ms, and different but constant TE (TE1/TE2/TE3 = 4.6/6.9/9.2 ms) were acquired using undersampled spiral readout. After 3-pt Dixon water-fat separation and spiral off-resonance blurring correction, fat signal was discarded and water T1 and T2 maps were reconstructed. For further quantitative analysis of MRF data, a ROI was drawn that included the tumor on subtraction images and copied onto the MRF voxel grid; for analysis of FGT, a ROI was placed into the contralateral, healthy breast. The differences between mean T1 and T2 values of breast cancer vs. FGT and the respective difference after contrast injection were tested for significance by two tailed student t-test, setting alpha=5%.
**Results or Findings:** In agreement with our expectation, pre-contrast T1 values of breast cancer were significantly longer than those of healthy FGT (mean, 1147±12 ms vs. 1052±95 ms) (p=0.007); the same was true for T2 values (83±14 ms vs 73±12 ms) (p=0.03). Post-contrast T1 values of healthy tissue were significantly longer than those of cancerous one (mean 690.31±200.34 ms vs. 367.32±121.49 ms) (p=0.0005). Post contrast T2-values were not significantly different.
**Conclusion:** Our quantitative T1 and T2 times of breast cancer and normal FGT before and after contrast injection are in agreement with expectations.
**Limitations:** n/a
**Ethics Committee Approval:** n/a
**Funding for this study:** n/a
**Author Disclosures:**
Eloisa Zanderigo: Nothing to disclose
Christiane K. Kuhl: Nothing to disclose
Teresa Nolte: Nothing to disclose
Volkmar Schulz: Nothing to disclose
Daniel Truhn: Nothing to disclose
Mirjam Broeckmann: Nothing to disclose

### RPS 302-19

**Background parenchymal enhancement on breast MRI in patients undergoing neoadjuvant chemotherapy: the association with tumour response and recurrence-free survival**

*C. Costanza*, M. Durando, G. Bartoli, G. Mariscotti, L. Bergamasco, P. Fonio; Turin/IT
*(dott.claudiacostanza@gmail.com)*

**Purpose:** To investigate whether background parenchymal enhancement (BPE) on dynamic contrast-enhanced magnetic resonance imaging (DCE-MRI) is associated with patients' outcomes following neoadjuvant chemotherapy (NCT) in locally advanced breast cancer

**Methods or Background:** We retrospectively included 93 consecutive women (mean age 51 years) with unilateral invasive breast cancer, who underwent NCT between March 2014 and December 2019. All patients underwent breast MRI examination before, during and post-NCT, before curative surgery. MRI features (BPE of the contralateral breast, lesions' characteristics) and clinicopathologic features (menopausal status, breast density, histological type, molecular subtype, nuclear grade) were analyzed. Patients were grouped according to BPE category (high[moderate or marked] or low[minimal or mild]). Cox regression modeling was used to determine associations between MRI features and recurrence-free survival(RFS) after controlling for clinicopathologic variables
**Results or Findings:** The pathological tumor response after NCT was complete (pCR) in 36/93(38.7%) patients and non-complete(non-pCR) including partial and no-response in 57/93(61.3%). Considering pCR, we found statistically significant association with HER2 positive cases. Other clinical-pathological characteristics had no statistically significant association with tumor response. The mean follow-up period was 27 months. Twenty recurrences occurred(11 loco-regional,9 distant). On multivariate analysis, high BPE on pre-NCT MRI was an independent variable associated with recurrence. Patients with dense breast had a poorer RFS than patients with non-dense breast, with statistically significant difference (p=0,01), as well as patients with triple negative cancer than patients with other molecular subtypes(p=0,03), patients with high pre-NCT BPE than patients with low pre-NCT BPE (p=0,01) and patients with high nuclear grade cancer(p=0,02)
**Conclusion:** Our results suggest that BPE on pre-NCT DCE-MRI may have potential as a predictor of long-term outcomes in breast cancer patients who undergo NCT, representing a risk factor significantly associated with recurrence
**Limitations:** Single-institution retrospective study
**Ethics Committee Approval:** Not applicable
**Funding for this study:** Nothing
**Author Disclosures:**
Manuela Durando: Nothing to disclose
Giovanna Mariscotti: Nothing to disclose
Paolo Fonio: Nothing to disclose
Laura Bergamasco: Nothing to disclose
Claudia Costanza: Nothing to disclose
Germana Bartoli: Nothing to disclose

### RPS 302-20

**Preoperative staging in breast cancer: contrast-enhanced MRI vs digital breast tomosynthesis (DBT) evaluated together with unenhanced MRI (UE-MRI)**

*V. Rizzo*, F. Galati, G. Moffa, E. Kripa, C. Caramanico, G. Panzironi, F. Pediconi; Rome/IT
*(nican66@gmail.com)*

**Purpose:** To evaluate the accuracy in tumor extent, size assessment and detection of additional lesions of Digital Breast Tomosynthesis (DBT)+Unenhanced Magnetic Resonance Imaging (UE-MRI) compared to Postcontrast-MRI, in women with known breast cancer. Histology is considered the gold standard.
**Methods or Background:** A retrospective analysis was performed on 79 patients with histological diagnosis of breast cancer, who had performed DBT projections and 3.0 T MRI over 2016-2017 in our Institution. Two breast radiologists, with 15 and 7 years of experience respectively, reviewed DBT and MRI (including DWI, T2-w and T1-w, before and after contrast agent administration, sequences) in separate reading sections, unaware of the final histological examination. MRI and DBT+UE-MRI sensitivity, PPV and accuracy were calculated. Inter-reader agreement was evaluated using Cohen's Kappa. Spearman correlation were used to evaluate post-contrast MRI vs Histology, DBT+UE-MRI vs Histology and post-contrast MRI vs DBT+UE-MRI lesions tumor size agreement to histological results.
**Results or Findings:** Of a total of 79 patients, 104 lesions were confirmed on histological examination. Post-contrast MRI showed 100% sensitivity, 96% PPV and 96% accuracy while DBT+UE-MRI achieved an average sensitivity of 81%, PPV of 92% and accuracy of 77%. Regarding inter-reader agreement, k was substantial for MRI (k=0,69) and DBT+UE-MRI (k=0,75) for detection rate. Lesions size Spearman correlation coefficient was 0.97 for Post-Contrast MRI vs Histology, 0.92 for DBT+UE-MRI vs Histology, (p-value<0.0001). MRI vs DBT+UE-MRI regression coefficient was 0.83.
**Conclusion:** In preoperative study of breast cancer post-contrast MRI is the most accurate imaging technique. However, DBT evaluated together with unenhanced sequences showed to be very accurate, with a good sensitivity and tumor size assessment and it could be a valid tool for preoperative staging when the use of contrast agents is contraindicated.
**Limitations:** Not applicable
**Ethics Committee Approval:** Not applicable
**Funding for this study:** Not applicable
**Author Disclosures:**
Giuliana Moffa: Nothing to disclose
Veronica Rizzo: Nothing to disclose
Claudia Caramanico: Nothing to disclose
Giovanna Panzironi: Nothing to disclose
Francesca Galati: Nothing to disclose
Endi Kripa: Nothing to disclose
Federica Pediconi: Nothing to disclose

### RPS 302-21

**Comparison of 3 abbreviated breast MRI protocols in a screening population: a reader study**

*I. Allajbeu*[1], R. Pintican[2], P. L. Moyle[1], V. Papalouka[1], G. C. Baxter[1], F. J. Gilbert[1]; [1]Cambridge/UK, [2]Cluj/RO
*(ia359@cam.ac.uk)*

**Purpose:** To evaluate the diagnostic performance of abbreviated breast (ABB) MRI with a non contrast examination and with a subtracted first post contrast MIP only.
**Methods or Background:** A retrospective reader study was conducted using breast MRI examinations from 52 patients with 26 (50%) normal examinations, biopsy proven benign pathology in 12 (23%) and breast cancer <15 mm in size in 14 (27%). The three MRI protocols (ABB-MRI - T1WI pre and post contrast, T2WI, DWI/ADC; T2WI + DWI/ADC; subtracted first post contrast MIP) were independently evaluated by 3 radiologists (R - beginner with 2 years experience and P & V - readers with > 10 years experience). BIRADS grading was used to score each protocol into suspicious (BIRADS 4,5) or non-suspicious (BIRADS 1,2,3). At least 4 week gap was left between protocols.
**Results or Findings:** Diagnostic accuracy was 0.90 (experienced) and 0.86 (beginner) for MIP only protocol, 0.94 (experienced) and 0. 88 (beginner) for ABB MRI protocol, and 0.86 for non contrast protocol, both experienced and beginner. There was a good agreement between readers with the MIP series with an intraclass correlation coefficient of 0.842 (95% CI 0.743 – 0.906), p<0.001. Good agreement between readers was found with the ABB-MRI protocol, intraclass correlation coefficient being 0.858 (95% CI 0.773 – 0.915), p<0.001.The best agreement was found with the T2/DWI protocol, the intraclass correlation coefficient being 0.900 (95% CI 0.842 – 0.939), p<0.001. The average reading time was shortest for MIP (12 sec), followed by T2+DWI (34 sec) and ABB-MRI protocol (41 sec).
**Conclusion:** Diagnostic accuracy was highest for the ABB MRI protocol. The non-contrast protocol achieved similar results for all 3 readers but was consistently poorer
**Limitations:** One center Study
Small number of patients
**Ethics Committee Approval:** Mixed case
**Funding for this study:** Na
**Author Disclosures:**
Penelope Laura Moyle: Nothing to disclose
Fiona J. Gilbert: Research/Grant Support: GE funding for research Grant Recipient: GE Funding for Research Investigator: GE Funding for research
Iris Allajbeu: Nothing to disclose
Vasiliki Papalouka: Nothing to disclose
Roxanna Pintican: Nothing to disclose
Gabrielle Carmen Baxter: Nothing to disclose

### Research Presentation Session: Breast

### RPS 402

### Diagnosing breast cancer

### RPS 402-1

**Diagnostic performance of non-invasive imaging for assessment of axillary pathologic complete response after neoadjuvant systemic therapy in clinically node-positive breast cancer: a meta-analysis**

*C. M. de Mooij*[1], S. Samiei[1], M. B. I. Lobbes[2], K. Keymeulen[1], T. van Nijnatten[1], M. Smidt[1]; [1]Maastricht/NL, [2]Sittard-Geleen/NL
*(c.demooij@maastrichtuniversity.nl)*

**Purpose:** Neoadjuvant systemic therapy (NST) can lead to downstaging of axillary lymph node disease. Imaging can potentially provide information about the axillary response to NST and, consequently, tailor the surgical management. The purpose of this study was to perform a systematic review and meta-analysis to determine the diagnostic performance of current noninvasive imaging modalities for assessment of axillary response after NST in clinically node-positive breast cancer patients.

**Methods or Background:** PubMed and Embase were searched for studies that compared noninvasive imaging after NST with axillary surgery outcome to identify axillary response in patients with initial pathologically proven axillary lymph node metastasis. Two reviewers independently screened the studies and extracted the data. A meta-analysis was performed by computing the pooled sensitivity, specificity, positive predictive value (PPV), and negative predictive value (NPV).
**Results or Findings:** Thirteen studies describing 2380 patients were included for final analysis. Of these patients, 1322 had undergone axillary ultrasound, 849 breast MRI, and 209 whole-body 18F-FDG PET-CT. The overall axillary pathologic complete response (pCR) rate was 39.5% (941/2380). For axillary ultrasound, the pooled sensitivity, specificity, PPV, and NPV were 65%, 69%, 77%, 50%, respectively. For breast MRI, the pooled sensitivity, specificity, PPV, and NPV were 60%, 76%, 78%, 58%, respectively. For whole-body 18F-FDG PET-CT, the pooled sensitivity, specificity, PPV, and NPV were 38%, 86%, 78%, 49%, respectively.
**Conclusion:** The diagnostic performance of current noninvasive imaging modalities is limited to accurately assess axillary response after NST in clinically node-positive breast cancer patients.
**Limitations:** The most important limitations of this meta-analysis are the heterogeneity between the included studies and the differences in NST regimens, type of axillary surgery, and definition of axillary pCR among the included studies.
**Ethics Committee Approval:** Not applicable.
**Funding for this study:** No funding to disclose.
**Author Disclosures:**
Marjolein Smidt: Nothing to disclose
Kristien Keymeulen: Nothing to disclose
Cornelis Maarten de Mooij: Nothing to disclose
Marc B I Lobbes: Nothing to disclose
Thiemo van Nijnatten: Nothing to disclose
Sanaz Samiei: Nothing to disclose

### RPS 402-2

**Assessment on contrast-enhanced cone beam breast CT (CE-CBBCT): characteristics of breast cancers**
*Y. Ma*, A. Liu, Z. Ye; Tianjin/CN
*(myue0205@163.com)*

**Purpose:** To assess the CE-CBBCT characteristics of breast cancers compared with breast MRI and mammograms.
**Methods or Background:** This retrospective study enrolled the patients who underwent preoperative CE-CBBCT, breast MRI and mammography, and confirmed pathologically as breast cancer between January 2017 to August 2020. A set of characteristics of breast cancers were evaluated by consensus double reading using the BI-RADS atlas as guidance, including morphology features, kinetic features and microcalcification characteristics. The CE-CBBCT images, MRI images and mammograms were assessed separately. The agreements on characteristics assessment of breast cancers between CE-CBBCT and another two modalities were calculated by ICC and Kappa coefficient, while the discrepancies were assessed by Chi-square test.
**Results or Findings:** A total of 60 malignant lesions were enrolled. On CE-CBBCT images, the assessment of breast composition category indicated almost perfect agreement degree with mammograms (Kappa=0.833), while the background parenchymal enhancement showed moderate degree with MRI (Kappa=0.563). CE-CBBCT images showed comparable multifocal lesions detection with MRI, better than mammograms (92.86% and 100% vs. 42.86%, $P<0.05$). For morphology characteristics, CE-CBBCT images showed substantial agreement with MRI on both mass (Kappa=0.687-0.747) and non-mass (Kappa=0.714) lesions. The CE-CBBCT images showed moderate to substantial degree on enhancement pattern and degree (0.600-0.714) of lesions. For microcalcification, CBBCT images presented comparable detection rate and substantial agreement on characteristics assessment (Kappa=0.621-0.631) with mammograms.
**Conclusion:** CE-CBBCT images combined the morphology and part of hemodynamic features on MRI and microcalcification characteristics on mammograms, indicating that the corresponding BI-RADS lexicons could be used to describe CE-CBBCT characteristics associated with breast cancers.
**Limitations:** This study was based on a relatively small data set.
**Ethics Committee Approval:** Institutional Review Board approval was obtained.
**Funding for this study:** The National Key R&D Program of China (grant 2017YFC0112600) and Tianjin Science and Technology Major Project (No. 19ZXDBSY00080).
**Author Disclosures:**
Yue Ma: Nothing to disclose
Zhaoxiang Ye: Nothing to disclose
Aidi Liu: Nothing to disclose

### RPS 402-3

**Diagnostic performance of 18F-FDG PET/CT and DCE-MRI of the breast in assessment of early response to neoadjuvant chemotherapy (NAC) in locally advanced braest cancer (LABC) patients**
*A. S. Abdelghani*, S. T. Hamed, E. Hussein, S. Lasheen, M. Hussein, Y. G. Ebrahim; Cairo/EG
*(alaa.radio2013@gmail.com)*

**Purpose:** Evaluation the performance of DCE-MRI of breast &18 F-FDG PETCT regarding the assessment of early response to NAC in LABC patients and pCR prediction.
**Methods or Background:** Forty patients with locally advanced breast cancer who underwent NAC were screened, however twenty-five only were included in final analysis. Both PET/CT and DCE-MRI were performed before and during NAC. Different PET-CT parameters as well as size measurements and functional parameters by DCE-MRI were used. Patients were classified into pathologic complete response (pCR) and pathologic non complete response (non-pCR). PET/CT and MRI findings were compared in correlation to post-operative pathology.
**Results or Findings:** The sensitivity and specificity of MRI in assessment LABC response to NAC in correlation with pathological data were 100% ($p<0.001$) and 12.5% ($p=0.18$) respectively. The corresponding values for PET/CT were 94.1% ($p<0.001$) and 25% ($p=0.18$), however the estimated overall accuracy for both MRI and PETCT was the same measuring 72%. The sensitivity and specificity of MRI in assessment axillary lymph nodes(ALN) response to NAC in correlation with pathological data were 83% and 50% respectively. The corresponding values for PET/CT were 47.4% and 68.4% respectively, however estimated overall accuracy of PETCT was more than of MRI 64% and 56% respectively. Significant predictors factors for pCR were longest diameter of lesion, ADC value and maximum enhancement in baseline MRI, SUVmax and SUV mean in baseline PETCT.
**Conclusion:** DCE-MRI had a higher sensitivity in predicting pCR in locally advanced breast cancer after NAC in the primary breast mass and ALN. The diagnostic accuracy of MRI and PETCT in detecting pCR of LABC patients to NAC was the same, however PETCT was more accurate in detecting pathological response of ALN to NAC.
**Limitations:** Small number
**Ethics Committee Approval:** Local scientific ethical committe approval
**Funding for this study:** No fund
**Author Disclosures:**
Soha Talaat Hamed: nothing to disclose
Mai Hussein: Nothing to disclose
Elshaymaa Hussein: Nothing to disclose
Alaa Sobhi Abdelghani: nothing to disclose
Youstina Gerges Ebrahim: nothing to disclose
Shimaa Lasheen: Nothing to disclose

### RPS 402-5

**Cone-beam breast CT features associated with intrinsic subtypes of HER2-positive breast cancer according to hormone receptor status**
*Y. Zhu*, Y. Ma, Y. Zhang, Z. Ye; Tianjin/CN
*(zhuyueqiang1985@126.com)*

**Purpose:** To investigate the relationship between cone-beam breast CT (CBBCT) features and HER2-positive (HER2+) breast cancer subtypes according to hormone receptor (HR) expression.
**Methods or Background:** Preoperative CBBCT of patients were retrospectively evaluated in 124 surgically resected HER2+ breast cancer between January 2018 and December 2019. A set of CBBCT descriptors was semiquantitatively assessed by consensus double reading. Multivariable logistic regression analysis using backward elimination (BEA) with the Wald criterion was performed to identify independent predictive factors of HR status in HER2+ breast cancer. Principle component analysis (PCA) was used to determine characteristics that might differentiate HER2+ subtypes. Receiver operating characteristic (ROC) curve analyses were conducted to determine the predictive capability.
**Results or Findings:** HR-HER2+ accounted for 46.8% (58/124) of patients. Based on BEA, pathologic grade (OR = 11.178; $P = 0.015$), Ki-67 index (OR = 4.6; $P = 0.011$), concavity (OR = 0.012; $P = 0.038$), and with non-mass enhancement (NME) (OR = 5.913; $P = 0.046$) were confirmed as independent indicators. PCA showed that with NME and concavity were the most important for differentiation. ROC curve analyses showed that CBBCT features combined with clinicopathologic characteristics (AUC = 0.855) were superior to clinicopathologic characteristics alone (AUC = 0.729, $P=0.015$).
**Conclusion:** CBBCT features in combination with clinicopathologic characteristics can be used to prognosticate HER2+ subtypes better than use of clinicopathologic characteristics alone. It has the potential to be used for the diagnosis and treatment decision in HER2+ breast cancer patients.
**Limitations:** We limited our analyses to surgically resected and non-preoperative treated specimens.

**Ethics Committee Approval:** The institutional review board approved this study, and written informed consent was obtained from all patients.
**Funding for this study:** National Key R&D Program of China (No. 2017YFC0112600 and 2017YFC0112601), National Natural Science Foundation of China (No. 81571671)
**Author Disclosures:**
Yue Ma: Nothing to disclose
Yueqiang Zhu: Nothing to disclose
Zhaoxiang Ye: Nothing to disclose
Yuwei Zhang: Nothing to disclose

### RPS 402-6

**Parameters of breast DTI (MD and FA) in high-grade and low-grade invasive ductal carcinoma**
*M. Nadrljanski*, Z. Milosevic, V. Urban, I. Krusac; Belgrade/RS
*(dr.m.nadrljanski@gmail.com)*

**Purpose:** To assess potential diagnostic use of breast DTI parameters (MD – mean diffusivity; FA – fractional anisotropy) in patients with invasive ductal carcinoma (IDC): a.) against the breast parenchyma and b.) between the subgroups of high-grade (G3) vs. lower grade (G1-G2) tumours.
**Methods or Background:** Prospective analysis of breast MRI (N=30) included patients with unilateral lesions: high-grade IDC (n1=15) and lower grade IDC (n2=15). All patients were examined with 1.5T MRI system with DTI, DWI (b50, 850) and DCE-MRI. ADC, FA and MD were computed for all lesions and FA and MD were computed for the contralateral breast parenchyma. Mann Whitney U test was performed to assess the differences.
**Results or Findings:** MD and FA were significantly different between IDC and parenchyma (1.00+/-0.05 vs. 1.62+/-0.02, p<0.00001; 0.31+/-0.02 vs. 0.32+/-0.02, p=0.001). No significant differences were obtained between n1 and n2 for MD and FA (0.99+/-0.06 vs. 1.01+/-0.05, p=0.215; 0.30+/-0.02 vs. 0.31+/-0.02, p=0.153), while the differences in ADC were significant between the two subgroups (1.00+/-0.07 vs. 1.08+/-0.06, p=0.006). Unlike FA, MD was significantly different between n1 and parenchyma and n2 and parenchyma (0.99+/-0.06 vs. 1.62+/-0.02, p<0.00001; 1.01+/-0.05 vs. 1.63+/-0.02, p<0.00001).
**Conclusion:** DTI parameters MD and FA are significantly different between IDC and breast parenchyma. No significant differences are obtained between high-grade and low-grade IDC lesions in terms of MD and FA.
**Limitations:** Retrospective, single-center study on a small number of patients. Technical limitations may include1.5T field strength in DTI.
**Ethics Committee Approval:** Referent board approval obtained for retrospective analysis.
**Funding for this study:** None.
**Author Disclosures:**
Zorica Milosevic: Nothing to disclose
Vladimir Urban: Nothing to disclose
Iva Krusac: Nothing to disclose
Mirjan Nadrljanski: Nothing to disclose

### Research Presentation Session: Breast

### RPS 502

### Breast ultrasound

### RPS 502-1

**Predicting breast cancer response to neoadjuvant chemotherapy with ultrasound strain elastography and contrast-enhanced ultrasound: a nomogram integrating Ki-67 and ultrasound features**
*Q. Liu*, L. Tang, M. Chen; Shanghai/CN
*(xiaoya1007@sjtu.edu.cn)*

**Purpose:** To explore whether conventional ultrasound, elastography and contrast-enhanced ultrasound (CEUS) combined with histopathology can monitor the efficacy of neoadjuvant chemotherapy (NAC) for breast cancer (BC), and develop a Nomogram prediction model monitoring response to NAC.
**Methods or Background:** From February 2010 to November 2015, 91 BC patients who received NAC were recruited. The maximum diameter, stiffness, CEUS features before and after NAC were assessed. Core biopsy, surgical pathology immunophenotype and Miller-Payne (MP) evaluation were documented. Univariate and multivariate analysis was performed using ROC analysis and logistic regression analysis.
**Results or Findings:** There were 37 cases showed pCR(MP=1,2,3) and 54 of non-pCR(4,5). Interobserver agreement between sonographers shows good consistency. The changes of maximal diameter were correlated with MP(P<0.05). The sensitivity (SEN), specificity (SPE), and area under the receiver operating characteristic (ROC) curve (AUC) of baseline size predicting pCR were 57.40%, 70.30%, and 0.64 (p=0.024). Baseline Ki-67 index of pCR group is significantly higher than that of non-pCR group (P=0.029), and the ROC analysis of baseline Ki-67 indicate the SEN, SPE, and AUC of 51.70%, 78.00%, and 0.638 (p=0.050). When combined with size, CEUS features, stiffness and Ki-67 of baseline, the ROC curve shows good performance with SEN, SPE, and AUC of 70.00%, 76.19%, 0.821 (p=0.004). Incorporate the change of characteristics into multivariate regression analysis, the results demonstrate excellent performance (SEN 100.00%, SPE 95.24%, AUC 0.986, P=0.000).
**Conclusion:** The change of the maximum size was correlated with MP score, which can provide reference to predict efficacy of NAC and evaluate residual lesions. When combining with elastography, CEUS and Ki-67, better performance in predicting pathological response was showed.
**Limitations:** Sample size and qualitative analysis.
**Ethics Committee Approval:** Institutional Review Board approval and written informed consent was obtained.
**Funding for this study:** Natural Science Foundation of Shanghai (No. 18ZR1434800)
**Author Disclosures:**
Lei Tang: Nothing to disclose
Qi Liu: Nothing to disclose
Man Chen: Nothing to disclose

### RPS 502-2

**Initial targeted ultrasound in women with focal breast complaints: what about tomosynthesis?**
*L. Appelman*[1], S. Siebers[1], P. Appelman[2], S. Go[3], P. Bult[1], M. Broeders[1], M. van Oirsouw[4], R. M. Mann[1]; [1]Nijmegen/NL, [2]Tienhoven/NL, [3]Alkmaar/NL, [4]Utrecht/NL

**Purpose:** To assess the potential of initial targeted ultrasound in the diagnostic work-up of women with symptomatic localized breast disease, and the supplemental value of tomosynthesis in this setting.
**Methods or Background:** In this multicenter prospective study the standard breast imaging protocol in women 30 years and older, presenting with focal breast complaints in three different hospitals in the Netherlands, was reversed. Initially, ultrasound was performed and when required lesions were biopsied. Subsequently the tomosynthesis was evaluated. We assessed cancer detection related to the focal complaint based upon initial ultrasound alone and the combination. Furthermore, we evaluated the frequency of findings elsewhere in the breast by tomosynthesis.
**Results or Findings:** Between September 2017 and June 2019 a total of 1961 women were enrolled (mean age 47.1 years). Based upon initial ultrasound alone, in 1588 women (81%) the complaints were due to normal fibroglandular tissue or benign findings (BI-RADS 1 or 2). In 2 of these women (0.1%), the tomosynthesis led to detection of a malignant lesion at the site of the complaint (sensitivity 99%). In 373 women (19%) initial ultrasound was judged as suspicious and followed by biopsy, yielding benign results in 181 women (48.5%) and malignant results in 192 women (51.5%) (specificity 90%). In women without cancer at the site of the complaint tomosynthesis yielded findings requiring additional investigations in 63 patients (3.2%), 8 of these were malignant (0.4%, 3 DCIS, 5 invasive).
**Conclusion:** Initial targeted ultrasound is accurate and specific in the assessment of focal breast complaints. Tomosynthesis in this setting must be regarded as a screening examination with a low diagnostic yield.
**Limitations:** No limitations
**Ethics Committee Approval:** Approved
**Funding for this study:** ZonMw
**Author Disclosures:**
Mireille Broeders: Nothing to disclose
M van Oirsouw: Nothing to disclose
S. Siebers: Nothing to disclose
Ritse Maarten Mann: Nothing to disclose
Peter Appelman: Nothing to disclose
Linda Appelman: Nothing to disclose
S. Go: Nothing to disclose
Sir P Bult: Nothing to disclose

### RPS 502-3

**Comparison of ultrasound second-look and digital breast tomosynthesis second-look in the detection of additional lesions at presurgical CESM**
*C. Bellini*[1], G. Bicchierai[1], F. Amato[2], E. Savi[1], D. De Benedetto[1], F. Di Naro[1], V. Miele[1], J. Nori[1]; [1]Florence/IT, [2]Palermo/IT
*(1chiarabellini@gmail.com)*

**Purpose:** To compare Ultrasounds second-look (US-SL) and Digital Breast Tomosynthesis second-look (DBT-SL) in the detection of additional lesions (ALs) at presurgical CESM.

**Methods or Background:** We retrospectively included among patients who underwent CESM for presurgical staging at our center, from September 2016 to December 2018, 121 women with 128 additional lesions (ALs). These ALs underwent US-SL and DBT-SL, to evaluate the performance of each technique and of the combination of both. ALs in CESM images were evaluated according to type of enhancement (focus, mass or non-mass), size (<10 mm or >10mm) and level of suspicion (BIRADS 3, 4 or 5). Our gold standard was post-bioptic histology and post-surgical specimen or >24-month negative follow-up. McNemar test was used for statistical analysis.
**Results or Findings:** Out of 128 ALs an imaging correlate was found for 71 (55.47 %,) at US, for 79(61.72%) at DBT, for 53 (41.41 %) at DBT and US, for 97 (75.78%) at US and/or DBT. DBT-SL proved a higher detection rate vs. US-SL in non-mass enhancement (NME) pattern on CESM (p:0.0325) and in DCIS histologic subtype (p:0.0081). Adding DBT-SL improved the performance of US-SL alone in the overall sample (p:<0,0001) and in every subcategory identified; adding US-SL to DBT-SL improved the dectectability of ALs in the overall sample and in every category except for NME (p:0.0833), foci (p:0.0833) and B3 lesions (p:0.3173).
**Conclusion:** Combined imaging second look (DBT-SL + US-SL) for CESM ALs is superior to DBT-SL only and US-SL only. In B3 lesions, NME and foci the analysis of a larger sample could determine if adding US-SL to DBT-SL is unnecessary.
**Limitations:** Retrospective study; not blinded; readers knew site and histology of index lesion; limited sample.
**Ethics Committee Approval:** Our ethics committee approved this study.
**Funding for this study:** Not needed.
**Author Disclosures:**
Elena Savi: Nothing to disclose
Giulia Bicchierai: Nothing to disclose
Federica Di Naro: Nothing to disclose
Jacopo Nori: Nothing to disclose
Vittorio Miele: Nothing to disclose
Francesco Amato: Nothing to disclose
Chiara Bellini: Nothing to disclose
Diego De Benedetto: Nothing to disclose

### RPS 502-4

**Breast ultrasound radiomic features: potential for prediction of response to neoadjuvant chemotherapy at baseline**

*P. Kapetas*, C. Sodano, P. Clauser, T. H. Helbich, M. Bernathova, R-I. Milos, P. A. Baltzer; Vienna/AT
*(panagiotis.kapetas@meduniwien.ac.at)*

**Purpose:** Exploratory study to evaluate whether radiomics features acquired from pre-therapeutic, B-mode breast ultrasound (US) can predict response to neoadjuvant chemotherapy (NAC).
**Methods or Background:** 97 patients with histologically confirmed invasive breast cancer who underwent NAC were included in this retrospective monocentric study. A representative B-mode US image of each tumor from the baseline, pre-treatment examination was selected. The baseline examinations had been performed with a variety of US devices. Tumors were segmented by a dedicated breast radiologist and overall 851 radiomic features were extracted. Radiomic features included first-order statistics, textural and wavelet features. LASSO regression was applied to reduce the number of features in the prediction model. Multivariate logistic regression was used to identify independent predictors of response to NAC. An exploratory area under the ROC curve (AUC) was used for the evaluation of the diagnostic performance. Postoperative pathology served as the standard of reference. Pathological complete response (pCR) was defined as the lack of invasive tumor in the breast or metastases in the axillary lymph-nodes. To rule-out interobserver segmentation variability, 30 cases were independently segmented by a breast radiology fellow.
**Results or Findings:** 44 patients (45.36%) achieved a pCR. After reduction of the radiomic features, 14 features remained. Among these, 3 wavelet radiomic features were shown by multivariate logistic regression to be independent predictors of response to NAC. The model demonstrated an AUC of 0.777 (95%CI: 0.681-0.855). At a predicted probability value of >18.2%, the presence of a residual tumor could be excluded with a sensitivity of 100%.
Segmentations showed substantial reproducibility with an intraclass correlation coefficient of 0.765.
**Conclusion:** Radiomic features acquired from baseline B-mode breast US examinations have the potential to predict response to NAC.
**Limitations:** Limited patient number.
**Ethics Committee Approval:** Yes.
**Funding for this study:** None.
**Author Disclosures:**
Pascal A.T. Baltzer: Nothing to disclose
Thomas H. Helbich: Nothing to disclose
Maria Bernathova: Nothing to disclose
Ruxandra-Iulia Milos: Nothing to disclose
Panagiotis Kapetas: Nothing to disclose
Claudia Sodano: Nothing to disclose
Paola Clauser: Nothing to disclose

### RPS 502-5

**Strain elastography in breast ultrasound in addition to US-BI-RADS classification**

*A. A. M. Orlando*[1], M. Di Marco[1], A. Cirino[1], R. Ienzi[1], M. Midiri[1], T. V. Bartolotta[2]; [1]Palermo/IT, [2]Cefalù/IT
*(orlandoalessiamed@hotmail.it)*

**Purpose:** To assess the role of qualitative (Tsukuba score, TS) and/or semi-quantitative (Strain Ratio, SR) Strain Elastography (SE) assessment in adjunct to US-BI-RADS classification in the characterization of Focal Breast Lesions (FBLs)
**Methods or Background:** 185 FBLs (3,5–45,2 mm) in 185 patients (mean age: 52,7 years) who underwent B-Mode breast-US, were evaluated with SE by an experienced breast radiologist. Two indipendent reviewers (both last year radiology residents) retrospectively and in consensus assessed the same 185 FBLs, assigning to each one a BI-RADS category and a Tsukuba Score. As SOR, US-guided core-biopsy and follow-up at 24 months were considered respectively for BI-RADS 4-5 and 2-3 FBLs. A cut-off for SR was calculated with the Youden method. A comparison between TSs (chi-square test) and SR (T-Student) between benign and malignant FBLs was performed. Diagnostic accuracy, Sensitivity, Specificity, Negative and Positive Predictive Values (NPV and PPV) for each BI-RADS re-classification resulting from the adjunct of TS (BI-RADS+TS), SR (BI-RADS+SR), and of concordant TS and SR (BI-RADS+TS/SR) were calculated and differences were evaluated using the chi-square test.
**Results or Findings:** 106/185 were benign and 79/185 were malignant FBLs. TS4-5 FBLs (72/185) were more frequently malignant (76.4%, p<0.0001) and TS1-2-3 (113/185) benign (78.8%, p<0.0001). Malignant FBLs showed higher SR mean value than benign ones (4.49 vs 2.73, p<0.0001). SR cut-off value was 2.51. A significative improvement in specificity was revealed with BI-RADS+TS and BI-RADS+TS/SR re-classification (71.7% and 67.9% vs 56.6%; p=0.002 and p=0.025), but with a significant decrease in sensitivity for BI-RADS+TS (83.5% vs 93.7%, p=0.0022).
**Conclusion:** BI-RADS reclassification, downgrading BI-RADS 4a FBLs to BI-RADS 3 when TS and SR values were concordant, could led to a significative improvement in specificity without a significative decrease in sensitivity.
**Limitations:** Monocentric retrospective study
**Ethics Committee Approval:** Obtained
**Funding for this study:** None
**Author Disclosures:**
Alessia Angela Maria Orlando: Nothing to disclose
Massimo Midiri: Nothing to disclose
Mariangela Di Marco: Nothing to disclose
Raffaele Ienzi: Nothing to disclose
Alessandra Cirino: Nothing to disclose
Tommaso Vincenzo Vincenzo Bartolotta: Nothing to disclose

### RPS 502-6

**Concordance between breast core needle biopsy and postoperative histopathology in B4 cases**

*V. Urban*, M. Nadrljanski, D. Pavlovic Stankovic, I. Petkovic, I. Krusac, K. Obradovic, N. Adzic, Z. C. Milosevich; Belgrade/RS

**Purpose:** To analyse concordances between B4 category after ultrasound-guided breast core needle biopsy and final histopathology.
**Methods or Background:** A retrospective analysis of all ultrasound-guided core needle biopsies at our institution between 2015. and 2020. was performed. The eligibility criteria were B4 lesions that underwent surgical treatment and received a final histopathology. T-test for independent samples and Fisher's test were used for calculating statistical significances.
**Results or Findings:** Out of 52 patients with post-biopsy B4 category, 40 (76.92%) underwent surgical excision. Their post-biopsy histopathology suggested 31 (77.5%) invasive cancers (IC), 3 (7.5%) DCIS, 4 (10%) papillary carcinomas, and 2 (5%) lobular carcinomas. On final histopathology, 34 (85%) cases were malignant, and 6 (15%) cases were benign; 30 (75%) had IC, and 4 (10%) had in situ cancer. Out of all IC, 16 (53.33%) were invasive ductal carcinomas (IDC), 4 (13.33%) were invasive lobular carcinomas (ILC), 2 (6.67%) were mixed ductal/lobular carcinomas, 3 (10%) were invasive tubular carcinomas, 1 (3.33%) was invasive tubulo-lobular carcinoma, 2 (6.67%) were invasive papillary carcinomas, 1 (3.33%) was medullary carcinoma, and 1 (3.33%) was metaplastic carcinoma. Patients with benign outcomes were significantly younger than patients with malignant outcomes (45.50±10.17 vs. 58.47±13.42 respectively, p=0.03). All patients with BI-RADS 4c and 5 had a final malignant diagnosis. Statistical significances between post-biopsy and postoperative histopathologies were found for suspicious IC and final malignancy (p=0.016), suspicious IC and final IC (p=0.002), suspicious DCIS and final DCIS (p=0.028), and for suspicious papillary carcinoma and final benign histology (p=0.008).
**Conclusion:** All BI-RADS 4c and 5 lesions classified as B4 were malignant; associations between post-biopsy suspicious IC and final malignancy/IC, and post-biopsy suspicious papillary carcinoma and final benign histology were highly statistically significant, which could impact further management.
**Limitations:** n/a
**Ethics Committee Approval:** n/a
**Funding for this study:** n/a
**Author Disclosures:**
Katarina Obradovic: Nothing to disclose
Dragana Pavlovic Stankovic: Nothing to disclose
Zorica C Milosevich: Nothing to disclose
Ivana Petkovic: Nothing to disclose
Iva Krusac: Nothing to disclose
Nina Adzic: Nothing to disclose
Mirjan Nadrljanski: Nothing to disclose
Vladimir Urban: Nothing to disclose

### RPS 502-7

**Analysis of functional imaging biomarkers in breast carcinoma**

*B. C R*, B. Raghavan, G. Sivaramalingam, S. Viswanathan; Chennai/IN
*(banupriyacr11@gmail.com)*

**Purpose:** • To find the association of SUV and ADC values with the pathological grading and molecular subtype of breast malignancy.
• To evaluate the correlation of grade of malignancy with background parenchymal uptake (BPU), background parenchymal enhancement (BPE) & fibro glandular tissue (FGT) of the contralateral normal breast.
**Methods or Background:** Patients with biopsy-proven breast carcinoma underwent whole-body PET-CT and MRI at our institute. Imaging findings were correlated with the grade of malignancy, hormone receptors and molecular subtypes.
**Results or Findings:** 53 patients were included with mean age of 49.3 years. Positive correlation was found between tumor grade and Mean SUVmax (p-value - 0.003). Statistically significant variation in mean SUVmax was seen among ER/PR receptor status with low values in ER+ tumors (7.72+5.46, p-value – 0.043) and PR+ tumors (7.15±5.11, p-value – 0.001). Higher mean SUVmax was seen in TNBC (12.27±4.22) and significant correlation was seen between each molecular subtype (p-value – 0.02). No association was made between the ADC values and tumors with different grades of malignancy. However, there was significant variation in ADC values among different molecular subtypes (p-value –0.09). Higher ADC values were seen in HER2 Enriched tumours (1.032±0.25) and low ADC values were seen in luminal A molecular subtype (0.798±0.13). There was no statistically significant correlation between background SUV of the contralateral breast with grade of malignancy, hormone receptor status or molecular subtypes.
**Conclusion:** PET CT and MRI can be used as a complementary imaging tool for non-invasive assessment of the aggressiveness and biological characteristics of tumor such as grade, Hormone receptor status and to differentiate molecular subtypes.
**Limitations:** Uneven distribution of histologic subtype and molecular subtypes among the patients.
**Ethics Committee Approval:** Acquired
**Funding for this study:** Not required
**Author Disclosures:**
Geethapriya Sivaramalingam: Nothing to disclose
Sathyasree Viswanathan: Nothing to disclose
Banupriya C R: Nothing to disclose
Bagyam Raghavan: Nothing to disclose

### RPS 502-8

**Can shear-wave elastography (SWE) be used in predicting breast histopathological gradings?**

*W. L. Ng*[1], N. N. B. Ab Mumin[2], N. B. Omar[1], K. Rahmat[1]; [1]Kuala Lumpur/MY, [2]Sungai Buloh/MY
*(wei.lin@ummc.edu.my)*

**Purpose:** To evaluate the diagnostic performance of shearwave elastography (SWE) of breast lesions in predicting histopathological gradings.
**Methods or Background:** Women with suspicious breast lesions underwent B-mode ultrasound and SWE of the breast prior to core needle biopsy and breast surgery. Quantitative SWE parameters measured included Emax, Emean, Emin and Eratio (kPa). Diagnostic performance of the parameters were assessed based on final surgical histopathology. Lesions were categorized to their pathologic types including ductal carcinoma in situ (DCIS) and grade I to III invasive ductal carcinoma (IDC). Other pathologies were excluded due to small number. Kruskal-Wallis test was used for correlation analysis. ER, PR, HER2 status and axilla lymph node (ALN) metastasis were evaluated.
**Results or Findings:** Total of 173 patients with 147 invasive carcinoma and 26 ductal carcinoma in situ (DCIS) were analysed. Tumor grades included DCIS (n = 26), IDC grade I (n =30), IDC grade II (n = 69) and IDC grade III (n =48). Larger size of breast lesions showed increase in histological grade (p<0.05). There was no statistically significant difference between the quantitative SWE parameters, ER, PR and HER2 status with the histological grade (p>0.05). However there is statistically significant association between the histopathological grades of the breast lesion with ALN metastasis with p-value of <0.05.
**Conclusion:** This study did not show statistically significant results with SWE and histopathological gradings. However there is a positive correlation between size and ALN metastasis with histopathological gradings.
**Limitations:** Small sample size.
**Ethics Committee Approval:** This study was approved by the Medical Research Ethics Committee (MRED), University Malaya Medical Centre, MREC ID NO: 201879-6469
**Funding for this study:** Research Grant-Faculty Program, Project No: GPF06C-2018
**Author Disclosures:**
Norlia Binti Omar: Nothing to disclose
Wei Lin Ng: Nothing to disclose
Kartini Rahmat: Nothing to disclose
Nazimah Ab Mumin: Nothing to disclose

### RPS 502-9

**Detection of focal breast vascularisation with microvascular flow technique: a comparison with conventional colour and power doppler**

*A. A. M. Orlando*[1], M. I. Schillaci[1], L. Spatafora[1], M. Di Marco[1], A. Cirino[1], R. Ienzi[1], M. Midiri[1], T. V. Bartolotta[2]; [1]Palermo/IT, [2]Cefalù/IT
*(orlandoalessiamed@hotmail.it)*

**Purpose:** To compare MV-Flow technique with Color-Doppler and Power-Doppler in the detection of vascularization of Focal Breast Lesions (FBLs).
**Methods or Background:** An experienced breast radiologist performed breast US in 180 patients detecting 180 solid FBLs (3.5-45.2 mm) and saved separate cine-loops for B-mode, CD, PD and MV-Flow examination, which were evaluated off-line by two independent reviewers (R1 and R2). Reviewers assessed lesion vascularity as absent or present, classifying vascularity patterns into: a) internal, b) vessels in rim c) combined pattern. Differences in vascularization patterns rates among CD, PD and MV-Flow were calculated using the chi-square test. K-scores with Fleiss method were calculated to assess intra- and inter-observer agreement.
**Results or Findings:** 103/180 FBLs were benign and 77/180 were malignant. For both readers, vascularity detection increased with MV-Flow (77.2% and 77.8%) vs CD (52.2% and 56.7%) and PD (53.9% and 56.7%) (p<0.00001) when evaluating all 180 FBLS, and when assessing either benign FBLs or malignant ones, even with statistical significance (p<0.05). With MV-Flow, for both readers, benign FBLs showed more frequently absence of vascularity (p=0.0195 and p=0.0104), rim (p=0.0032 and p=0.0052) or combined pattern (p=0.0131 and p=0.0369), whereas malignant lesions showed higher prevalence of internal pattern (p<0.0001 for both). With MV-Flow penetrating vessels detection, more frequent in malignant FBLs than in benign ones (p<0.00001), increased significantly for both readers (p<05). Inter-reader agreement ranged from good to very good, with the lowest k-score of 0.8550 for MV-Flow (vs 0.7882 and 0.7472 for CD and PD). Intra-observer agreement demonstrated k-scores ranging from good to very good.
**Conclusion:** MV-Flow significantly increased the detection of vascularization in FBLs, regardless the experience of the readers or the nature of FBLs.
**Limitations:** Only qualitative assessment No comparison with tumoral microvessel density
**Ethics Committee Approval:** Obtained
**Funding for this study:** None
**Author Disclosures:**
Alessia Angela Maria Orlando: Nothing to disclose
Luigi Spatafora: Nothing to disclose
Massimo Midiri: Nothing to disclose
Mariangela Di Marco: Nothing to disclose
Maria Ilenia Schillaci: Nothing to disclose
Raffaele Ienzi: Nothing to disclose
Alessandra Cirino: Nothing to disclose
Tommaso Vincenzo Vincenzo Bartolotta: Nothing to disclose

### Research Presentation Session: Breast

### RPS 602

### Mammography and breast cancer

### RPS 602-1

**Impact of COVID-19 pandemic on breast imaging practice: results of a survey from an institutional department in north-western Italy**

*A. Timpani*, G. Bartoli, M. Durando, A. Frigerio, A. Luparia, V. Marra, L. Milanesio, D. Tota, P. Fonio; Turin/IT
*(alessandra.timpani@gmail.com)*

**Purpose:** The study's purpose was to investigate working style changes adapting routine practice in Breast Imaging Services during the first COVID-19 pandemic.
**Methods or Background:** A web-based survey of 20 multiple-choice questions was designed and distributed on April 2020 to healthcare staff of three Breast Imaging Services(screening and diagnostic) of the same Radiology Department and returned in one week. The questionnaire collected information regarding organization adaptations, case volume changes, personal protective equipment (PPE) use, personal exposure, staff resiliency to COVID-19 and future challenges. All responses were collected and analysed anonymously.
**Results or Findings:** We recorded complete responses from 61%Radiologists (Residents and Consultants),28%Radiographers and 11%Office Employers. During the first COVID-19 two months' Italian lockdown, all participants reported a decline in mammography volume (89%), partly due to patients' withdrawal, no changes in MR examinations and 12% increase in interventional pre-operative procedures. The interaction with other specialists (Oncologists and Surgeons) remained mainly unchanged (82%). 90% of the staff advised that suspended activities had to be rebooked in the ordinary schedule or with additional sessions. More of 55% of the staff was exposed to COVID-19 positive patients and then tested, with negative results. Nearly all responders reported limited availability of PPE and that testing for COVID healthcare staff had to be warranted.
**Conclusion:** First COVID-19 pandemic has drastically impacted the practice of breast imaging,reducing mammography volumes both in screening and diagnostic setting, otherwise maintaining pre-operative oncologic work-up and symptomatic cases management. Breast Imaging Services faced significant risks mainly from virus diffusion by asymptomatic (not previously recognized) COVID positive patients and from inadequate PPE availability and use. This survey was a local snapshot that would benefit from comparison studies with other regions and countries and with the second ongoing pandemic upsurge.
**Limitations:** Small sample size
Monocentric study
**Ethics Committee Approval:** Not applicable
**Funding for this study:** No funding
**Author Disclosures:**
Manuela Durando: Nothing to disclose
Donatella Tota: Nothing to disclose
Paolo Fonio: Nothing to disclose
Luisella Milanesio: Nothing to disclose
Alfonso Frigerio: Nothing to disclose
Alessandra Timpani: Nothing to disclose
Andrea Luparia: Nothing to disclose
Vincenzo Marra: Nothing to disclose
Germana Bartoli: Nothing to disclose

### RPS 602-2

**CEM-guided biopsy: an alternative to MRI-guided breast biopsy**

*R. Alcantara Souza*, E. N. Arenas Rivera, M. Pitarch Diago, B. Ejarque, M. C. Posso Rivera; Barcelona/ES
*(rodials@hotmail.com)*

**Purpose:** To describe the technical aspects and our initial experience with the use of contrast enhanced mammography (CEM) for percutaneous breast interventional procedures in a Teaching Hospital in Spain.
**Methods or Background:** This is a retrospective evaluation of prospectively collected data. Since October 2019, we have been selecting patients with enhancing lesions for a clinical validation study of an upright stereotactic breast biopsy unit with dual-energy acquisition. Patients have been treated in our Hospital, that serves a population of approximately 350,000 inhabitants. We describe here the management of enhancing only lesions, reporting our initial experience with the CEM-guided biopsy. This study was approved by the Ethics Committee of our institution in September 2019.
**Results or Findings:** From October 2019 to October 2020, we have performed 30 interventional procedures (in 28 patients) under CEM-guidance, 27 biopsies, all of them using a 10G vacuum-assisted needle, and 3 wire localizations. We achieved 100% target visibility, although there were some challenging findings. We used a horizontal approach with a lateral arm and a sitting position in almost 80% (24) of cases, and the procedure time ranged from 12 to 25 minutes. Regarding the histopathological results of the biopsies performed (27), we found 37% (10) of neoplasms, 15% (4) atypia, and 48% (13) benign lesions. We did not observe major adverse effects.
**Conclusion:** From our experience, CEM-guided biopsy is a safe procedure, with diagnostic results similar to those described in the literature for an MRI-guided biopsy. This novel approach has the potential to be a faster and more affordable alternative to MRI-guided biopsy, however larger prospective studies are needed.
**Limitations:** The relative small number of cases.
**Ethics Committee Approval:** Ethics committee approval Nº 2019-8890.
**Funding for this study:** There was no direct funding for this clinical validation study.
**Author Disclosures:**
Rodrigo Alcantara Souza: Speaker: GE Healthcare
Erika Natalia Arenas Rivera: Nothing to disclose
Belen Ejarque Sanahuja: Nothing to disclose
Margarita Cynthia Posso Rivera: Nothing to disclose
Mireia Pitarch Diago: Nothing to disclose

### RPS 602-3

**The results in patients diagnosed with high-risk breast lesions with image-guided needle biopsy: a multicentre retrospective study**

*Ö. Aslan*[1], A. Oktay[1], F. Taşkin[2], N. Tunçbilek[3], G. Esen[2], P. Balci[1], M. E. Aribal[2], L. Çelik[2], S. Örgüç[4]; [1]Izmir/TR, [2]Istanbul/TR, [3]Edirne/TR, [4]Manisa/TR
*(dr.ozgeaslan@gmail.com)*

**Purpose:** In this multicenter study, the goal was to document the excisional biopsy or follow-up results of high risk lesions diagnosed on image guided CNB/VAB, and evaluate the clinical, imaging and histologic features for associated malignancy risk.
**Methods or Background:** This is a retrospective multicentric study that include 1240 patients from 25 centers. The radiology records were searched for all image guided biopsies and pathology reports were reviewed in a 12 year period, between 2008 and 2020. Patients who had a diagnosis of high risk breast lesion on image guided core biopsy (tru-cut/vacuum biopsy) were reviewed. The patients who managed with an excisional biopsy or having at least 1 year follow-up documentation following the diagnosis of borderline lesion were included in the study. Needle biopsy type and sampling method were documented. The data were analysed using SPSS statistical program.
**Results or Findings:** Pathological subtypes of 1240 high-risk lesions with biopsy were 28% ADH, 45% intraductal papilloma without atypia, 9.4% intraductal papilloma with atypia, 9% flat epithelial atypia, 4.5% radial scar, 4.3% Lobuler neoplasia, 0.1% pleomorphic LCIS. In all cases, the upgrade rate to malignancy was found to be 22%. According to pathological subtypes, the upgrade rates were 40.6% in ADH, 14.8% in LN, 100% in pleomorphic LCIS, 45.7% in intraductal papilloma with atypia, 10% in intraductal papilloma without atypia, 5.4% in radial scar, and 9.3% in FEA.
**Conclusion:** The appropriate management after the diagnosis of a high risk lesion on image guided biopsy is controversial and recommendations between surgical excision and follow-up vary in different practices in these group of lesions.
**Limitations:** The number of cases could not be increased more due to cases where follow-up results could not be achieved.
**Ethics Committee Approval:** The institutional review board approval was obtained.
**Funding for this study:** None
**Author Disclosures:**
Sebnem Örgüç: Nothing to disclose
Mustafa Erkin Aribal: Nothing to disclose
Aysenur Oktay: Nothing to disclose
Özge Aslan: Nothing to disclose
Nermin Tunçbilek: Nothing to disclose
Levent Çelik: Nothing to disclose
Füsun Taşkin: Nothing to disclose
Pinar Balci: Nothing to disclose
Gul Esen: Nothing to disclose

### RPS 602-4

**The prognostic potential of mammographic growth rate of invasive breast cancer in the Nijmegen breast cancer screening cohort**

*J. Peters*[1], J. van Dijck[1], S. Elias[2], J. Otten[1], M. Broeders[1], On behalf of the IMAGINE consortium[1]; [1]Nijmegen/NL, [2]Utrecht/NL
*(Jim.Peters@radboudumc.nl)*

**Purpose:** Mammographic screening has led to increased detection of small breast cancer tumours, but only those with unfavourable biological characteristics may be life-threatening. To distinguish aggressive from indolent cancers, tumour growth rate (GR) may provide valuable prognostic information. This study addresses the association of mammographic GR with established prognostic factors and overall survival in a screened population.
**Methods or Background:** In this historic cohort study, 293 women with invasive ductal carcinoma were identified from all participants in the Nijmegen screening programme between 2000-2007. Information on patient-related factors and tumour characteristics was retrieved from patient files. Follow-up on vital status was obtained through municipalities. On the diagnostic and two prior screening mammograms, tumour volumes were estimated, assuming a spheroid shape. After comparing the fit of five growth functions, GR was calculated using a power-law growth function. Regression and survival analyses, adjusted for age, volume, density and detection mode (screen-detected or interval), were used to study associations between GR and prognostic factors as well as overall survival.
**Results or Findings:** Each one-standard-deviation increase in GR was associated with an increase in the Nottingham Prognostic Index by 0.23 [95%CI 0.10-0.35] and volume at diagnosis by 2.67 cm^3 [95%CI 0.65-470]. It was further associated with increased odds to have a tumour of unfavourable subtype (based on histologic grade and hormone receptors; OR 1.68 [95%CI 1.07-2.64]). At the end of follow-up (median 12.4 years), 78 deaths occurred. GR was not associated with overall survival (HR=1.04 [95%CI 0.80-1.34]).
**Conclusion:** Mammographic GR may indicate differences in tumour aggressiveness and shows the potential to be used as a prognostic marker if, like in screening, serial mammograms are available.
**Limitations:** A potential association of GR with cause-specific survival could not be determined.
**Ethics Committee Approval:** Not applicable
**Funding for this study:** Dutch Cancer Society (KWF11835)
**Author Disclosures:**
Jim Peters: Nothing to disclose
Mireille Broeders: Nothing to disclose
Sjoerd Elias: Nothing to disclose
Johannes Otten: Nothing to disclose
On behalf of the IMAGINE consortium: Nothing to disclose
Jos van Dijck: Nothing to disclose

### RPS 602-5

**Clinical performance of contrast-enhanced spectral mammography in pre-surgical evaluation of breast malignant lesions in dense breasts: a single-centre study**

L. Nicosia, A. C. Bozzini, V. Dominelli, S. Penco, L. Meneghetti, *M. Montesano*, A. Latronico, M. Pizzamiglio, E. Cassano; Milan/IT
*(marta.mon@hotmail.it)*

**Purpose:** To compare the efficacy of contrast-enhanced spectral mammography, with ultrasound, full field digital mammography and magnetic resonance imaging in detection and size estimation of histologically proven breast tumors.
**Methods or Background:** This study, included 160 dense breast women with at least one suspicious mammary lesion evaluated by ultrasound, full field digital mammography and magnetic resonance imaging in whom a mammary tumor was histologically proven after surgery performed at the European Institute of Oncology between January 2013 and December 2015. Following the complete diagnostic procedure, the patients were investigated by contrast-enhanced spectral mammography prior to surgery.
**Results or Findings:** Overall, the detection rate of malignant breast lesions (in situ and invasive) was 93.8% (165/176) for contrast-enhanced spectral mammography, 94.4% (168/178) for ultrasound, 85.5 (147/172) for full field digital mammography and 97.7% (173/177) for magnetic resonance imaging. Radiological measurements were concordant with the post-surgical pathological measurements of the invasive tumor (i.e., within 5 mm) in: 64.6% for contrast-enhanced spectral mammography, 62.0% for ultrasound, 45.2% for full field digital mammography ($p < 0.0001$) and 69.9% for magnetic resonance imaging ($p = 0.28$); underestimated in: 17.4% for contrast-enhanced spectral mammography, 19.6% for ultrasound, 24.2% for full field digital mammography ($p = 0.03$) and 6.7% for magnetic resonance imaging ($p = 0.0005$); and overestimated in: 16.2% for contrast-enhanced spectral mammography, 16.6% for ultrasound, 16.6% for full field digital mammography and 22.7% for magnetic resonance imaging ($p = 0.02$).
**Conclusion:** Our data suggest that contrast-enhanced spectral mammography improves on full field digital mammography and is comparable to ultrasound and magnetic resonance imaging in terms of detection sensitivity and size estimation of malignant lesions in dense breasts
**Limitations:** The study is monoinstitutional.
**Ethics Committee Approval:** The ethichs commitee approval was obtained.
**Funding for this study:** The study did not receive any funds.
**Author Disclosures:**
Luca Nicosia: Nothing to disclose
Silvia Penco: Nothing to disclose
Antuono Latronico: Nothing to disclose
Valeria Dominelli: Nothing to disclose
Anna Carla Bozzini: Nothing to disclose
Maria Pizzamiglio: Nothing to disclose
Enrico Cassano: Nothing to disclose
Lorenza Meneghetti: Nothing to disclose
Marta Montesano: Nothing to disclose

### RPS 602-6

**Accuracy of volume measurements of tumours seen in mammograms: a simulation study**

*H. Tomic*[1], B. Barufaldi[2], K. Johnson[1], S. Zackrisson[1], M. Dustler[1], A. Tingberg[1], P. Bakic[2]; [1]Malmö/SE, [2]Philadelphia, PA/US
*(hanna.tomic@med.lu.se)*

**Purpose:** To analyze estimated tumour volumes in simulated mammograms.
**Methods or Background:** Breast tumour volume is estimated to determine growth rate, which correlates with tumour aggressiveness and could, e.g., reduce unnecessary interventions. Clinically, the tumour size is commonly reported via its largest diameter. The reported size can assist volume estimation assuming spherical approximation. We simulated 42 mammograms with tumours using an open-source simulation pipeline. Each tumour was simulated as three overlapping ellipsoids. Individual tumours differed in shape by offsetting centers of ellipsoids for 0-5 mm relative to each other. The simulation provided the ground true tumour volume. A radiologist analyzed simulated mammograms, and measured the largest tumour size and the maximum size orthogonal to it. We estimated tumour volumes using spherical and ellipsoidal approximations. The spherical volume estimations were calculated using the largest size (method A) and the mean of the two measured sizes (method B). The ellipsoidal approximation (method C) used as semi-axes the measured sizes, and in the z-direction their average value. The three volume estimates were compared to the ground truth. The radiologist also rated the resemblance of simulated tumours to clinical tumours.
**Results or Findings:** The mean relative overestimation of tumour volume for method A, B, and C were 0.390, 0.331 and 0.324, respectively. Fifty-eight percent of simulated tumours resembled clinical tumours. Low clinical resemblance corresponded to large offsets in simulated tumour shape.
**Conclusion:** Our study suggests that ellipsoidal approximations (method C) improve the volume estimation accuracy by 17% vs. the largest spherical approximation (method A). This observation is important when estimating tumour growth rate from clinical images.
**Limitations:** n/a
**Ethics Committee Approval:** n/a
**Funding for this study:** Supported by the Skåne University Hospital Foundation, Swedish Fund for Clinical Research (ALF), and Marie Skłodowska-Curie Fellowship #846540.
**Author Disclosures:**
Kristin Johnson: Nothing to disclose
Anders Tingberg: Research/Grant Support: Siemens
Hanna Tomic: Nothing to disclose
Bruno Barufaldi: Nothing to disclose
Predrag Bakic: Research/Grant Support: Real Time Tomography
Magnus Dustler: Nothing to disclose
Sophia Zackrisson: Research/Grant Support: Siemens Speaker: Siemens

### RPS 602-7

**Initial experience of magnetic seed localisation in an Irish breast centre: of particular importance in the COVID era**

*L. Sweeney*, R. M. Heaney, A. O'Brien, F. Flanagan; Dublin/IE
*(laura.sweeney@live.ie)*

**Purpose:** Wire-guided localisation (WGL) has long been the mainstay of non-palpable breast lesion identification, however it has not been without its drawbacks. WGL is performed the morning of surgery, which causes scheduling/co-ordination issues between admissions, radiology and theatre. Magnetic seed localisation (MSL) has become an excellent alternative which overcomes these issues. MSL can be performed any time before surgery, resulting in greater patient-flow efficiency and reduced patient visits.
**Methods or Background:** We examined our first year of MSL experience (July 2019-July 2020), reviewing all cases in our screening/symptomatic centres combined. Our primary outcome measure was accuracy of placement of the seed relative to target lesion/clip on post-seed mammogram, taking largest distance on CC/lateral as index distance from target. We defined ‘acceptable placement’ as <=10mm of target, with further breakdown of ‘at target’(0mm), ‘very close’(<2mm), ‘close’(3-5mm) and ‘within range’(6-10mm).

**Results or Findings:** A total of 297 MSLs were performed July 2019-July 2020, 243(82%) using US guidance and 54(18%) using tomographic guidance. 285(96%) of cases were placed within acceptable range: 196(66%) at target, 46(15%) within 2mm, 31(10%) within 3-5mm and 12(4%) within 6-10mm. 12(4%) were outside of acceptable range of 10mm. 3(1%) required WGL the morning of surgery for more accurate lesion identification.
**Conclusion:** MSL is an excellent alternative to WGL, with 96% of magnetic seeds placed within 10mm of target in our first year. MSL allows for more flexibility from a logistical patient-flow point of view, allowing lesion localisation in the outpatient setting any time/date prior to the surgical date. We have found this a particularly beneficial factor in the era of COVID-19, whereby we reduced the number of patient visits and contacts in the radiology department.
**Limitations:** None
**Ethics Committee Approval:** Not required
**Funding for this study:** None
**Author Disclosures:**
Angela O'Brien: Nothing to disclose
Laura Sweeney: Nothing to disclose
Fidelma Flanagan: Nothing to disclose
Roisin Mary Heaney: Nothing to disclose

### RPS 602-8

**Quantitative analysis of enhancement parameters of breast cancer on Contrast-Enhanced Spectral Mammography (CESM): correlation with DCE-MRI and prognostic significance**
*S. Akkaya*, S. Kul; Trabzon/TR
*(selcuk.akkaya85@gmail.com)*

**Purpose:** To determine the feasibility of quantitative analysis of enhancement on contrast-enhanced spectral mammography (CESM) by comparing with dynamic contrast enhanced-MRI (DCE-MRI) and to correlate enhancement parameters with prognostic tumor markers in malignant lesions.
**Methods or Background:** 99 malignant lesions imaged with CESM and DCE-MRI were retrospectively analyzed. Lesion enhancement on CESM images was measured after manual segmentation of tumor and nearby background. Contrast-to-noise ratio [CNR= (Lesion mean intensity-Background mean intensity)/Standard deviation measured in the background] derived from sequentially obtained CC and MLO views were used as a measure of early (CNR1) and delayed (CNR2) tumor enhancement, respectively. Kinetic curve pattern was decided by comparing enhancement at early and delayed phases and grouped as persistent, stable and wash-out as in the case of DCE-MRI. To calculate percent change in enhancement intensity SD=(CNR2-CNR1/CNR1) x 100 formula was used. Early phase enhancement and kinetic pattern of the same lesions were evaluated on DCE-MRI by manually segmenting the tumor area. CESM parameters were correlated with tumor size, histologic type, grade, axillary status and immunohistochemical biomarkers. Cohen's kappa, Spearman correlation, Mann Whitney U and Chi-square tests were used.
**Results or Findings:** Early phase enhancements on CESM and MRI positively correlated ($r=0.450$, $P=0.001$). There was moderate agreement between CESM and DCE-MRI kinetic curve patterns (kappa=0.510, $P<0.001$). Early phase enhancement intensity on CESM positively correlated with tumor size, grade and Ki67 index ($P<0.040$). Wash-out kinetic curve type on CESM was associated with high histologic grade ($P<0.048$).
**Conclusion:** Quantitative analysis of enhancement on CESM images is feasible and well-correlated with DCE-MRI. These enhancement parameters might be helpful for prognostic significance of malignant tumors.
**Limitations:** The study was carried out retrospectively. The sample size was relatively limited.
**Ethics Committee Approval:** This study was approved by our ethics committee.
**Funding for this study:** No funding.
**Author Disclosures:**
Selçuk Akkaya: Nothing to disclose
Sibel Kul: Nothing to disclose

### RPS 602-9

**Identifying potential improvement points in the breast cancer multidisciplinary team meeting to facilitate development of a clinical decision support system – an interview study**
*L. Koco*, R. Mann; Nijmegen/NL
*(lejla.koco@radboudumc.nl)*

**Purpose:** Determining areas in breast cancer multidisciplinary team meetings (MDTMs) where an AI based clinical decision support system (CDSS) can be of added value according to breast cancer care specialists, may lead to inclusion of their views in the development of the CDSS, and improve its usefulness in clinical practice.
**Methods or Background:** From January 2019 to November 2020 core team members of the breast cancer MDT in three hospitals were interviewed. Semi-structured interviews were performed based on an interview guide. All interviews were recorded and transcribed verbatim. Deductive coding was performed on the transcripts. The codes were organized in categories and themes.
**Results or Findings:** In total 27 clinicians; surgeons, medical oncologists, radiotherapists, pathologists, radiologists, and specialized nurses, were interviewed. Improvement points most often mentioned were related to efficiency and missing data, during preparation and during the MDTM. Many actions are needed to find relevant patient information in the electronic medical records and redundant work is performed by the notetaker during the MDTM, which may be problematic as it was widely anticipated that the number of patients will increase. Foreseen benefits of using a CDSS would be; a clearer overview of all patient data, automatic generation of MDTM reports and clinical trial suggestions. However, conditions for using the CDSS are that; it should be up to date at all times, it provides reliable and patient specific guideline suggestions and it is editable and flexible.
**Conclusion:** Clinicians expect that using a CDSS could help to improve efficiency prior to, and during the MDTM, by affecting different aspects of the process. However, the CDSS should be up to date at all times and provide reliable, patient-specific suggestions.
**Limitations:** For each hospital one clinician per discipline was interviewed.
**Ethics Committee Approval:** Not necessary
**Funding for this study:** None
**Author Disclosures:**
Ritse Maarten Mann: Nothing to disclose
Lejla Koco: Nothing to disclose

### RPS 602-10

**Contrast-Enhanced Spectral Mammography (CESM) guided biopsy - initial clinical experience**
*A. Kornecki*, I. Ben-Nachum, M. Bhaduri, N. Khan, G. Muscedere, O. Shmuilovich; London, ON/CA

**Purpose:** Contrast Enhanced Spectral Mammography (CESM) is an emerging imaging technology with a cancer detection rate approaching that of MRI. For enhancing lesions detected by CESM and not by ultrasound or mammography, biopsy is typically performed under breast MRI guidance. A new biopsy technique has been developed to allow radiologists to perform CESM guided biopsy with the standard stereotactic- guided biopsy device.
**Methods or Background:** As the first centre in North America to use CESM-guided biopsy, the purpose of this study to assess the feasibility of the use of CESM-guided biopsy in 50 patients. This abstract will describe our initial clinical experience in the patients that have been recruited so far.
**Results or Findings:** Total of 199 enhancing lesions categorised as BI-RADS 4/5 found in 283 CESM exams obtained between June 1-October 30, 2020. 172 lesions were biopsied under US (131 malignant, 22 benign, 19 high risk lesions) and 15 were biopsied under routine stereotactic guidance (9 malignant, 2 benign, 4 high risk lesions). CESM-guided biopsy was successful in 8/10 lesions (2 malignant, 5 benign, 1 high risk lesion). 3 were initially detected by MRI and then by CESM. Two lesions did not enhance at the time of the biopsy; 1 was identified in a patient with brain metastases of unknown origin and died shortly after (PET CT was negative for breast primary) and 1 was found in a patient who underwent subsequent mastectomy for 2 biopsy proven malignancies and found to have additional lesions.
**Conclusion:** Initial experience regarding the feasibility of CESM-guided biopsy was encouraging. Lesions that were not seen were likely transient.
**Limitations:** No limitations.
**Ethics Committee Approval:** The was approved by Health Canada and Western University Research Ethic Board.
**Funding for this study:** The study is funded by GE Healthcare.
**Author Disclosures:**
Nasir Khan: Investigator: Study is funded by GE
Mousumi Bhaduri: Investigator: Study is funded by GE
Anat Kornecki: Author: Study is funded by GE
Olga Shmuilovich: Investigator: Study is funded by GE
Giulio Muscedere: Investigator: Study is funded by GE
Ilanit Ben-Nachum: Investigator: Study is funded by GE

### RPS 602-11

**Comparison of background parenchymal enhancement on contrast-enhanced spectral mammography and breast MR images**
*E. D. Luczynska*[1], W. Rudnicki[2], T. Piegza[2], M. Pawlak[2], S. Heinze[2]; [1]Rzeszow/PL, [2]Krakow/PL
*(ela.luczynska@op.pl)*

**Purpose:** Contrast enhanced spectral mammography (CESM) and magnetic resonance imaging (MRI) are comparable methods regarding sensitivity. The aim of this study is to check if analysis of background parenchymal enhancement (BPE) on CESM can improve its usefulness.
**Methods or Background:** 79 patients with breast lesions found previously on ultrasound or mammography underwent MRI and CESM. On MRI the enhancement kinetics and visual BPE were evaluated. On CESM the enhancement of lesions was as well as a quantitative level of BPH were evaluated. The obtained data were analysed and compared.
**Results or Findings:** A total of 83 lesions were identified both on MRI and CESM, including 16 (20.2%) benign and 67 (79.8%) malignant lesions. Among malignant lesions 17 (21%) were assessed as intraductal and 50 (63.3%) as infiltrating carcinomas. The study showed correlation between the level of enhancement on CESM and the type of kinetic curve on MRI and lesion enhancement on CESM as well as confirmed the fact that the BPE is important factor in both methods of imaging.
**Conclusion:** Evaluation of BPE level on CESM can help reading radiologists to define a lesion as malignant with higher probability than based only on the qualitative lesion enhancement level.
**Limitations:** Lack of data on the menstruation cycle day of the patients admitted for CESM although we didn't notice if it fluctuate during the cycle. Another limitation is a small group of analysed patients.
**Ethics Committee Approval:** Ethics committee approval OIL/KBL/17/2018.
**Funding for this study:** Nothing to declare.
**Author Disclosures:**
Tomasz Piegza: Nothing to disclose
Sylwia Heinze: Nothing to disclose
Elzbieta Dorota Luczynska: Nothing to disclose
Marta Pawlak: Nothing to disclose
Wojciech Rudnicki: Nothing to disclose

### RPS 602-12

**Incidence of malignant disease associated with pregnancy and lactation: a series of cases**

*F. J. Mercado Burgos*, M. E. Droguett; Santiago/CL
*(mercado.fdo@gmail.com)*

**Purpose:** Delaying motherhood is a growing trend in our society; this has increased the incidence of malignant breast disease detected during pregnancy and lactation. Breast ultrasound is the preferred method to study pregnant patients since it does not involve ionizing radiation. Our purpose was to determine the incidence of malignant breast disease in pregnant/lactating patients studied with breast ultrasound in our institution.
**Methods or Background:** We retrospectively reviewed all mammography and breast ultrasound studies performed on this specific population for 3.5 years (January 2017 to September 2020). We collected the images and pathology reports available on our PACS, EMR, and analyzed them.
**Results or Findings:** The sample included 1069 women, with an average age of 36 (SD 4.5 years), with a range of 25 and 53 years. 663 (62%) were 35 years and older. All were studied with ultrasound, and on cases that the radiologist deemed necessary, a mammogram or other complementary projections were requested. None of these additional studies detected new suspicious lesions. In 98% of cases (1045/1069), benign or probably benign (BIRADS 2 or 3) findings were reported. In 25/1069 cases, the results were suspicious of malignancy (BIRADS 4 and 5). 21 patients were biopsied with six malignant lesions, 4/6 of them in patients older than 35 years.
**Conclusion:** Ultrasound proved to be a sensible method for detecting lesions concerning for malignancy in this scenario. Mammography did not provide additional information, and we observed an increased incidence in the group with advanced maternal age.
**Limitations:** It is not a prospective study.
Low incidence of malignant disease.
**Ethics Committee Approval:** Not required
**Funding for this study:** Not required
**Author Disclosures:**
Fernando José Mercado Burgos: Nothing to disclose
Maria Elisa Droguett: nothing to disclose

### RPS 602-13

**The diagnostic value of contrast-enhanced 2D mammography in clinical practice**

*L. Neeter*[1], H. Raat[2], S. Meens-Koreman[2], R. Van Stiphout[2], S. Timmermans[2], M. Smidt[1], J. E. Wildberger[1], P. Nelemans[1], M. B. I. Lobbes[3]; [1]Maastricht/NL, [2]Roermond/NL, [3]Sittard-Geleen/NL
*(lidewij.neeter@mumc.nl)*

**Purpose:** Contrast-enhanced mammography (CEM) has shown to be consistently superior to digital mammography. However, current results are mainly based on systems by a specific vendor (GE Healthcare). We aimed to evaluate the diagnostic performance of a CEM system by another vendor (Hologic) in clinical practice.
**Methods or Background:** All patients in clinical practice who underwent CEM for various indications in 2019 at Laurentius Hospital (Roermond, The Netherlands) were eligible for this study, except for those in whom CEM was used as response monitoring tool. CEM was performed on a dedicated system (Selenia Dimensions, HOLOGIC). Three experienced breast imaging readers first assessed and scored the low-energy images (LE) using BI-RADS classification, before similarly assessing and scoring the CEM exams. Histopathology or one-year follow-up were the reference standard to assess true disease status. Diagnostic parameters, ROC curves, and AUCs were calculated for LE and CEM and for all readers.
**Results or Findings:** Breast cancer was diagnosed in 35 of the 152 included patients (23%). For all readers combined, sensitivity increased from 74.3% for LE to 87.6% for CEM (p=<0.0001) and specificity increased from 87.8% for LE to 94.6% for CEM (p=0.0146). The increase of the AUC from 0.872 for LE to 0.957 for CEM (p=0.001) was also significant. These outcomes are comparable to those from previous studies on other systems and meta-analyses on CEM.
**Conclusion:** The tested CEM system provided superior diagnostic performance over conventional mammography, which is in line with current knowledge. Results are comparable to published results for other clinical CEM systems.
**Limitations:** The sample size appears small and breast cancer prevalence was relatively high.
**Ethics Committee Approval:** Necessity for written informed consent was waived by our local IRB.
**Funding for this study:** This research is supported by a ZonMw DoelmatigheidsOnderzoek grant. No funding by Hologic.
**Author Disclosures:**
Steffie Timmermans: Nothing to disclose
Marjolein Smidt: Nothing to disclose
Lidewij Neeter: Nothing to disclose
Patty Nelemans: Nothing to disclose
Marc B I Lobbes: Research/Grant Support: GE Healthcare Speaker: GE Healthcare, Guerbet, and Bayer
Joachim E. Wildberger: Other: Institiutional grants - AGFA, Bayer Healthcare, Bard Medical, GE Healthcare, Optimed, Philips Healthcare, and Siemens Healthineers Speaker: Siemens Healthineers, Bayer Healthcare
Rogier Van Stiphout: Nothing to disclose
Stephanie Meens-Koreman: Nothing to disclose
Henricus Raat: Nothing to disclose

### RPS 602-14

**Sensitivity for the assessment of breast lesions in contrast enhanced spectral mammography (CESM): can we exclude malignancy?**

*A. A. Marzogi*[1], P. Clauser[2], P. Kapetas[2], R-I. Milos[2], M. Bernathova[2], T. H. Helbich[2], P. A. Baltzer[2]; [1]Makkah/SA, [2]Vienna/AT
*(alaamarzogi@live.com)*

**Purpose:** To measure the sensitivity and negative predictive value of breast lesions in CESM by correlating the enhancement intensity to the histology result of breast biopsy
**Methods or Background:** This is an IRB approved cross-sectional, retrospective study. Consecutive cases were collected from July 2018 until Oct 2020. The reference standard was histology in all cases. CEM examinations were performed with a standard technique and protocol. Two breast radiologists with 8 and 10 years of experience in breast imaging, blinded to patient data and histopathology result, qualitatively evaluated the breast lesions for enhancement intensity and a grade from (0) to (3) was given for each lesion; in which, (0)=no enhancement, (1)=subtle, (2)=moderate, (3)=distinct enhancement. ROC analysis was performed, and sensitivity and specificity were calculated by dichotomizing Grade 0, 1 enhancement as negative and grade 2, 3 enhancement as positive test result.
**Results or Findings:** Among 200 patients, 139 lesions were included (84 malignant (60%), 55 benign (40%)). For reader 1 (R1) the areas under the curve (AUC) using the grade of enhancement were 0.842 (95% confidence interval (CI), 0.771 - 0.898) and for reader 2 (R2) 0.879 (95% CI: 0.813 - 0.928). Sensitivity for R1 and R2 were as follow 78.6% (95% CI: 68.3%-86.8%) and 88.1% (95% CI: 79.2%-94.1%). Negative predictive value was 71.9% (95% CI: 62.5%-86.8%), and 79.6% (95% CI: 68.03%-87.7%). Categorical assessment of CESM enhancement demonstrated moderate sensitivity and negative predictive value.
**Conclusion:** Due to the moderate sensitivity and negative predictive value, our results indicate that CESM cannot exclude malignancy. Consequently, invasive procedures may be required for further workup of breast lesions independent from CESM results.
**Limitations:** N/a
**Ethics Committee Approval:** This is an IRB approved
**Funding for this study:** N/a
**Author Disclosures:**
Pascal A.T. Baltzer: Nothing to disclose
Alaa Abdulrahman Marzogi: Nothing to disclose
Thomas H. Helbich: Nothing to disclose
Maria Bernathova: Nothing to disclose
Ruxandra-Iulia Milos: Nothing to disclose
Panagiotis Kapetas: Nothing to disclose
Paola Clauser: Other: Speaker for Siemens healthcare GmbH

### Research Presentation Session: Breast

### RPS 702

### Artificial intelligence and machine learning in breast

### RPS 702-1

**Leveraging ipsilateral dual-view information for mass detection in mammograms using deep learning**

*M. Jie*[1], X. Lin[1], R. Ouyang[1], M. Wu[1], Z. Yang[2], Z. Cao[2], Y. Zhang[2], P. Chang[2], M. Han[2]; [1]Shenzhen/CN, [2]Palo Alto, CA/US

**Purpose:** To leverage the appearance and geometry relations of mass lesions in ipsilateral craniocaudal(CC) and mediolateral-oblique(MLO) views of one breast to improve mass detection in mammograms.
**Methods or Background:** The conventional deep learning framework typically accepts a single image input. However, the ipsilateral correlated information of CC and MLO views of one breast is well leveraged by radiologists in practice, because a mass lesion found in one CC/MLO view is highly possible to appear in the corresponding MLO/CC view. Moreover, the lesion-to-nipple distances of one mass in both views are similar. We proposed a relation module integrated with a dual-input Faster-RCNN framework to emphasize the appearance and geometry similarities of a mass showing in the ipsilateral images. The appearance feature refers to an RoI's feature map after the RoI align stage and the geometry feature is calculated based on the RoI-to-nipple distance. We also upgraded the classification and regression losses with Focal loss(FL) and Distance-IoU loss(DIoU), respectively. A total of 3582 in-house cases including malignant, benign, and normal cases, each of whom has four-view mammograms, were collected for our experiments.
**Results or Findings:** We adopt the Free-Response-Operating-Characteristic(FROC) metric to evaluate recall values at different False Positive per Image(FPI) points. Our recall values at 0.5,1.0,2.0 FPI are 0.88,0.92,0.96, respectively. We also compare our proposed relation block with a direct concatenation operation after the RoI align stage. The latter method reports the respective recall values of 0.80,0.86,0.91, worse than the performance of our proposed methods.
**Conclusion:** Our proposed dual-input deep learning framework with the learning of ipsilateral appearance and geometry relations in CC and MLO views of one breast is demonstrated to improve mass lesion detection in mammograms.
**Limitations:** The dataset size is relatively small.
**Ethics Committee Approval:** The IRB number is LL-XJS-2020011.
**Funding for this study:** n/a
**Author Disclosures:**
Zhicheng Yang: Nothing to disclose
Ma Jie: Nothing to disclose
Peng Chang: Nothing to disclose
Mingxiang Wu: Nothing to disclose
Rushan Ouyang: Nothing to disclose
Mei Han: Nothing to disclose
Yanbo Zhang: Nothing to disclose
Zhenjie Cao: Nothing to disclose
Xiaohui Lin: Nothing to disclose

### RPS 702-2

**Artificial intelligence-based breast density classifier improves mammography reporting reliability**

*A. Watanabe*[1], R. Mantey[2], C. Chim[2]; [1]Manhattan beach, CA/US, [2]La Jolla, CA/US
*(alyssa90266@gmail.com)*

**Purpose:** To demonstrate superior reliability of an AI-based tissue density classifier for mammography using an innovative semi-supervised learning (SSL) method which addresses reader variability, poor consistency of quantitative classifiers, and the complex issues in machine training based on the subjective assessment goals of BIRADS 5th-edition. The use of SSL removes human bias from the training of software.
**Methods or Background:** The AI-based density classifier (cmDensity(TM), CureMetrix.) is trained using SSL without explicit labeling, eliminating human bias. The classifier was compared to 7 MQSA qualified readers in 4-class (A-D) assessments using 792 mammograms from 3 institutions, 2 continents, and 3 vendors. Borderline exams between density classes were chosen to maximally test performance. Kappa (k) statistics at 95% confidence interval (CI) including intraclass correlation coefficient (ICC) were used for measuring inter-reader agreement, intra-reader reliability, and comparison with cmDensity. cmDensity's reliability was also tested using agreement across tomosynthesis images.
**Results or Findings:** The cmDensity agreement increased with degree of consensus (4/7 to 7/7 readers) (k=0.65,0.82,0.94,0.97). In cases with 100% reader consensus, there was near perfect agreement with cmDensity. The intra-reader reliability ranged from ICC=0.70-0.82 vs near perfect cmDensity reliability (ICC=0.99). Dense cases were correctly scored by cmDensity, despite variation in fibroglandular tissue (which is the downfall of non-AI based volumetry software). The density classifier showed high agreement in the tomosynthesis evaluation.
**Conclusion:** cmDensity shows higher reliability compared to radiologists in tissue density categorization and addresses the BIRADS 5th-edition subjective goal of reporting perceived masking effect of dense tissue. Benefits including reduced reporting variability, enhanced radiologist efficiency (including population of reports), and improved accuracy and consistency in communication of tissue density to clinicians and patients.
**Limitations:** A larger sample size could be useful.
**Ethics Committee Approval:** As a retrospective study, an IRB waiver was obtained.
**Funding for this study:** None.
**Author Disclosures:**
Alyssa Watanabe: Consultant: CureMetrix, Inc Shareholder: CureMetrix, Inc
Chiyung Chim: Employee: CureMetrix, Inc
Richard Mantey: Employee: CureMetrix, Inc

### RPS 702-3

**MRI-based radiomics for prediction to neoadjuvant chemotherapy in breast cancer: a referral centre analysis**

*F. Pesapane*, A. Rotili, L. Bianchini, F. Botta, F. Ferrari, S. Raimondi, D. A. Origgi, M. Cremonesi, E. Cassano; Milan/IT
*(filippopesapane@gmail.com)*

**Purpose:** We aimed to determine whether radiomic features extracted from breast magnetic resonance imaging (MRI) could non-invasively predict the response to neoadjuvant chemotherapy (NACT) in patients with breast cancer.
**Methods or Background:** 130 patients with breast cancer receiving NACT in a single centre (01/2017-06/2019) and undergoing breast MRI, were retrospectively evaluated. For each included patient, radiomic features were extracted within the biopsy-proven tumour on T1-weighted contrast-enhanced MRI performed before NACT. The pathological complete response (pCR) of NACT was determined based on the final surgical specimen. The association of clinical/biological and radiomic features with response to NACT was evaluated by univariate and multivariable analysis by using Random Forest and logistic regression. The performance of all models was assessed using the area under the receiver operating characteristics curve (AUC) with 95% Confidence Intervals (CI).
**Results or Findings:** 83 patients (mean [SD] age, 47.26 [8.6] years) were included. Patients with HER2+, basal-like molecular subtypes and Ki67≥20% presented more frequently a pCR to NACT; clinical/biological model AUC (95%CI) was 0.81 (0.71-0.90). Using 136 representative radiomics features selected through cluster analysis from the 1037 extracted features, a radiomic score was calculated to predict the response to NACT, with AUC (95%CI): 0.64 (0.51-0.75). Combining clinical/biological- and radiomics-model, the AUC (95%CI) was 0.83 (0.73-0.92).
**Conclusion:** MRI-based radiomic features slightly improve the pre-treatment prediction of pCR to NACT in addiction to biological characteristics. If confirmed on larger cohorts, it could be helpful to identify rightful patients for avoiding unnecessary treatment.
**Limitations:** First, the retrospective nature of the study and the limited number of patients; second, the assessment of interobserver agreement to ensure the reproducibility of the segmentation was not conducted.
**Ethics Committee Approval:** This retrospective-study was approved by the institutional review board (UID1926)
**Funding for this study:** No funds.
**Author Disclosures:**
Anna Rotili: Nothing to disclose
Linda Bianchini: Nothing to disclose
Francesca Botta: Nothing to disclose
Sara Raimondi: Nothing to disclose
Filippo Pesapane: Nothing to disclose
Marta Cremonesi: Nothing to disclose
Enrico Cassano: Nothing to disclose
Federica Ferrari: Nothing to disclose
Daniela Anna Origgi: Nothing to disclose

### RPS 702-4

**Measuring short and long-term breast cancer risk by combining mammographic texture models, an AI-based CAD system, and established risk factors**

*A. D. Lauritzen*[1], A. Rodriguez-Ruiz[2], M. C. von Euler-Chelpin[1], E. Lynge[3], I. Vejborg[1], M. Nielsen[1], N. Karssemeijer[2], M. Lillholm[1]; [1]Copenhagen/DK, [2]Nijmegen/NL, [3]Nykøbing Falster/DK
*(al@di.ku.dk)*

**Purpose:** To investigate the combined effect of mammographic texture and the exam score of an AI-based CAD system in terms of quantifying short- and long-term breast cancer risk.
**Methods or Background:** This retrospective study comprised a cohort of 52637 double-read screens of four FFDMs from the Danish Capital Region breast cancer screening program, including 154 interval cancers (IC, diagnosed 6-24 months after screening) and 808 long-term cancers (LTC, diagnosed 2-5 years after screening). For each exam, three metrics were computed. Percent mammographic density (PMD) was measured using a deep-learning-based tool. Mammographic texture-based risk (TBR) was computed using the deep-learning architecture, ResNet34, trained to detect images of women with high probability of developing cancer in the future. An AI-CAD system, Transpara v1.6, analyzed all studies providing an exam score from 0 to 10, where a high score indicates a high probability of visible malignancy. Using baseline screening mammograms, we examined risk segregation performance of the models for IC (short-term) and LTC using area under the ROC curve (AUC-ROC, 95% CI). Logistic regression was used to combine co-variates and validated using 5-fold cross-validation.
**Results or Findings:** TBR yielded an AUC-ROC of 0.69 (0.64-0.73) for ICs and 0.66 (0.64-0.68) for LTCs. The AI-CAD yielded an AUC-ROCs of 0.67 (0.62-0.71) for ICs and 0.64 (0.62-0.66) for LTCs. Combining TBR, AI-CAD, PMD, and age yielded an AUC-ROCs of 0.72 (0.68-0.76) for ICs and 0.68 (0.66-0.69) for LTCs.
**Conclusion:** Results indicate that combining texture-based risk, AI-CAD exam score, PMD, and age improved risk segregation for both ICs and LTCs compared to texture and AI-CAD alone suggesting that both systemic and localised findings can contribute to risk modelling.
**Limitations:** The cross-validated performance should be validated independently in future studies.
**Ethics Committee Approval:** n/a
**Funding for this study:** Partially Eurostars project IBSCREEN ref. 9715
**Author Disclosures:**
My Catarina von Euler-Chelpin: Nothing to disclose
Andreas David Lauritzen: Nothing to disclose
Alejandro Rodriguez-Ruiz: Employee: ScreenPoint Medical
Mads Nielsen: Nothing to disclose
Martin Lillholm: Nothing to disclose
Elsebeth Lynge: Nothing to disclose
Nico Karssemeijer: CEO: ScreenPoint Medical
Ilse Vejborg: Nothing to disclose

### RPS 702-5

**Evaluation of 3T multiparametric MRI with radiomic analysis for differentiating benign and malignant breast lesions**

*A-C. Vamvakas*, D. Tsivaka, A. Logothetis, K. Vassiou, I. Tsougos; Larissa/GR
*(alek.vamv@gmail.com)*

**Purpose:** To investigate the contribution of 3Tesla multiparametric MRI in benign and malignant breast lesions differentiation with radiomic analysis.
**Methods or Background:** In this retrospective study 109 females with histologically confirmed mass-like breast lesions (54 benign and 55 malignant), who underwent a 3Tesla MRI scanning with T2-weighted, Dynamic Contrast Enhanced T1-weighted (DCE) and Diffusion Weighted Imaging (DWI), were included. 3-dimensional lesion contours were manually drawn in T2-w, 2nd post-contrast DCE frame and Apparent Diffusion Coefficient (ADC) images with LIFEx v5.1 software and 138 radiomic features (shape, histogram, texture) were extracted. The Mann-Whitney U-test was used for initial elimination of non-informative features and the remaining 100 features were imported into a binary Logistic Regression classifier with Elastic-Net regularization. The evaluation of different feature subsets classification performance was based on 10-fold cross-validation and Receiver Operating Characteristic analysis performed in MATLAB R2019a.
**Results or Findings:** The highest classification performance (AUC=0.94) was achieved by a subset combining features from the three MRI sequences. DCE had a better performance (AUC=0.91) followed by ADC (AUC=0.86) and T2-w (AUC=0.83). We observed that histogram features were present in all feature subsets, while only DCE, T2-w and mixed subsets included texture features. Also, the number of texture features needed to maintain classification accuracy was further increased when shape features were excluded from the analysis.
**Conclusion:** Multiparametric MRI radiomic analysis holds great potential for breast cancer classification.
**Limitations:** n/a
**Ethics Committee Approval:** Institutional ethics committee approval and patient informed consent were obtained.
**Funding for this study:** This research is co-financed by Greece and the European Union (European Social Fund-ESF) through the Operational Programme «Human Resources Development, Education and Lifelong Learning 2014-2020» in the context of the project "Breast cancer assessment through advanced multiparametric imaging techniques and development of differential diagnosis software using artificial intelligence systems" (MIS5048948).
**Author Disclosures:**
Alexandros-Chrysovalantis Vamvakas: Nothing to disclose
Dimitra Tsivaka: Nothing to disclose
Andreas Logothetis: Nothing to disclose
Katerina Vassiou: Nothing to disclose
Ioannis Tsougos: Nothing to disclose

### RPS 702-6

**Diagnostic performance of AI for cancers registered in a mammography screening program: a retrospective analysis**

M. E. Seker, *Y. O. Köylüoğlu*, M. E. Aribal; Istanbul/TR

**Purpose:** The aim of this study is to evaluate the impact of AI based decision support system in radiologists' detection rates in a screening setting.
**Methods or Background:** MMG images of patients who were either diagnosed with cancer or didn't have an instance of cancer for 2 years between ages 40 and 69 were acquired from a single local screening center. A total of 110 cancer cases, of which 74 were screening detected, 27 were interval/intermediate type and 9 were missed and a control group of another 101 healthy women's MMG images who were registered as free from cancer for at least 2 years were evaluated with a web based AI algorithm which estimated a risk score between 0-100 for each breast. AI findings and radiologist decisions were analyzed separately for three subgroups; screen-detected, interval/intermediate and missed. Results were analyzed with a ROC curve to find a threshold with Youden's index and patients were relabeled accordingly. Radiologists' original findings were compared to a simulated radiologist/AI team up.
**Results or Findings:** AI predicted cancers in the cancer cohort with an AUC of 0.853 (95% CI = 0.801 – 0.905) and a sensitivity and specificity of 72.8% and 88.3%, respectively, for a risk score of 34.5 as a cut off value. AI missed 7.8% of the radiologists' true positive cases. However it has detected, an additional 70.3% of interval/intermediate and 77.7 of missed cases.
**Conclusion:** AI is effective in detecting cancers and able to increase the detection rate of interval and missed cancers. It may ameliorate the workflow of radiologists as a decision support system in a screening program by detecting a high rate of interval and missed cancers.
**Limitations:** 1.Single center study
2.Absence of inter-observer reliability statistics
**Ethics Committee Approval:** Approved
**Funding for this study:** n/a
**Author Disclosures:**
Mustafa Erkin Aribal: Nothing to disclose
Mustafa Ege Seker: Nothing to disclose
Yılmaz Onat Köylüoğlu: Nothing to disclose

### RPS 702-7

**Radiomic machine learning for predicting prognostic biomarkers and molecular subtypes of breast cancer based on the integration of tumour heterogeneity and angiogenesis properties at MRI**

*H. S. Park*[1], B. K. Seo[1], J. Y. Lee[2], K-S. Lee[3], K. R. Cho[3], O. Woo[3], E. K. Park[1], S. E. Song[3]; [1]Ansan/KR, [2]Goyang/KR, [3]Seoul/KR
*(phs9416@naver.com)*

**Purpose:** To investigate machine learning for radiomics-based prediction of prognostic biomarkers and molecular subtypes of breast cancer using quantification of heterogeneity and angiogenesis at MRI.
**Methods or Background:** This prospective study examined 291 invasive breast cancers in 288 patients who underwent breast MRI before treatment. For quantification of heterogeneity and angiogenesis, texture analysis and perfusion analysis were performed using filtration-histogram technique and the Tofts model, respectively. For each cancer, 144 heterogeneity and 16 angiogenesis parameters were extracted. Relationships between parameters and prognostic biomarkers were analyzed using six machine learning algorithms. Each machine learning model was built using only texture features, only perfusion features, or integrating both features. The model performance and importance of MRI parameters were derived.
**Results or Findings:** Texture parameters were associated with status of lymph node, hormone receptors, HER2, Ki67, tumor size, grade, and molecular subtypes ($P<0.05$). Among perfusion parameters, relative extracellular extravascular space parameter (Ve) was associated with status of hormone receptors and Ki67, tumor size, grade, and molecular subtypes (P<0.05). The random forest model integrating texture and perfusion parameters was the best for prediction: 73% and 0.73 vs 70% and 0.71 (texture parameters only), and 61% and 0.57 (perfusion parameters only) for average accuracy and area under ROC curve, respectively. The most important parameters for prediction were entropy (texture parameter) and Ve (perfusion parameter).
**Conclusion:** Radiomic machine learning, which integrates tumor heterogeneity and angiogenesis properties at MRI, can noninvasively predict prognostic biomarkers and molecular subtypes of breast cancer. Entropy and Ve are the most important parameters for this prediction.
**Limitations:** External validation wasn't performed.
**Ethics Committee Approval:** IRB approved.
**Funding for this study:** Supported by National Research Foundation (NRF) of Korea grant funded by the Ministry of Science and ICT of Korea (No. NRF-2020R1F1A1073213) and the Korea University grant (No. K2008291).
**Author Disclosures:**
Okhee Woo: Nothing to disclose
Eun Kyung Park: Nothing to disclose
Bo Kyoung Seo: Nothing to disclose
Sung Eun Song: Nothing to disclose
Kwang-Sig Lee: Nothing to disclose
Ji Young Lee: Nothing to disclose
Hyun Soo Park: Nothing to disclose
Kyu Ran Cho: Nothing to disclose

### RPS 702-8

### Radiomics analysis in breast MRI for non-invasive (in situ) breast cancer characterisation: initial results

*G. Lavazza*[1], V. Giannini[1], L. Martincich[2], M. Durando[1], G. Bartoli[1], A. Defeudis[1], D. Regge[1], P. Fonio[1]; [1]Turin/IT, [2]Asti/IT

**Purpose:** To investigate the correlation between texture analysis and radiologic and histologic characteristics of breast cancer at preoperative breast MRI. To identify an imaging biomarker for therapeutic stratification.
**Methods or Background:** Retrospective multicentric analysis including 74 patients with newly diagnosed in situ breast cancers, who preoperatively underwent 1.5T breast MRI between October 2010 and April 2020. Histologic analysis was obtained in all patients and histo-pathologic (including receptors expression) characteristics were analyzed. Two dedicated breast radiologists in consensus reviewed MRI examination and breast lesions were manually segmented on DWI and DCE sequences. Texture analysis was obtained with an "in-house" C++ software. The Mann-Whitney test was used for the statistical monoparametric analysis. Moreover, stepwise logistic regression model used as significance levels: 0.20 for adding variables and 0.05 for removing variables. Statistical significance was set at p<=0.05.
**Results or Findings:** Evaluating lesion and patients' characteristics, at DCE sequences 11 features reached statistical significance for the micropapillary in situ subtype, 7 features for the high-grade nuclear lesions, 6 features for the non-mass MRI lesions, 4 features for positive progesterone receptors lesions, and 4 features for patients candidate to mastectomy. At DWI sequence, 9 features reached statistical significance for patients candidate to mastectomy. The features considered were not able to statistically discriminate other breast lesions characteristics. The stepwise logistic regression models were are able to discriminate the previously reported lesions characteristics, with performances measured by area under ROC curve ranging between 0.79 e 0.93.
**Conclusion:** In accordance with literature, our results express the possible use of texture analysis as an imaging biomarker. The extraction of features from MR images could affect the decision-making process of the most suitable therapeutic approach and bring advantages to oncologic practice.
**Limitations:** The small sample size.
**Ethics Committee Approval:** Not applicable
**Funding for this study:** None
**Author Disclosures:**
Manuela Durando: Nothing to disclose
Daniele Regge: Nothing to disclose
Paolo Fonio: Nothing to disclose
Laura Martincich: Nothing to disclose
Giulia Lavazza: Nothing to disclose
Germana Bartoli: Nothing to disclose
Arianna Defeudis: Nothing to disclose
Valentina Giannini: Nothing to disclose

### RPS 702-9

### Machine learning approaches for optimization in MRI texture analysis of breast cancer to predict prognostic biomarkers and molecular subtypes

*H. S. Park*[1], B. K. Seo[1], E. K. Park[1], K-S. Lee[2], J. Y. Lee[3], K. R. Cho[2], O. Woo[2], S. E. Song[2]; [1]Ansan/KR, [2]Seoul/KR, [3]Goyang/KR
*(phs9416@naver.com)*

**Purpose:** To investigate machine learning approaches for optimization in MRI texture analysis of breast cancer to predict prognostic biomarkers and molecular subtypes. Texture analysis is useful for non-invasive quantification of tumor heterogeneity.
**Methods or Background:** A total of 291 invasive breast cancers in 288 patients who underwent dynamic breast MRI were enrolled. Texture analysis was performed using filtration-histogram technique with spatial scale filters (SSF; 0-without filtration, 1-2 mm-fine scale, 3-4 mm-medium scale, 5 mm-coarse scale). We measured 6 texture features (mean, standard deviation, mean of positive pixels, entropy, skewness, kurtosis) at T2-weighted(T2), pre-contrast T1-weighted(PreT1), and post-contrast T1-weighted(PostT1) imaging. 144 parameters were extracted and their relationship to pathological factors was analyzed using logistic regression and 5 machine learning models. Accuracy and the AUC (area under the ROC curve) were used to evaluate model performance.
**Results or Findings:** The random forest model was the best for predicting prognostic factors. As SSF approached 0, the superior performance of the random forest model became more apparent when compared to the logistic regression: in AUC, 0.79 vs. 0.71 for estrogen receptor status, 0.73 vs. 0.65 for Ki67, 0.77 vs. 0.70 for tumor grade, and 0.83 vs. 0.74 for molecular subtype. According to variable importance from the random forest, the top 5 important parameters were PostT1 entropy, PostT1-PreT1 entropy, T2 entropy, T2 kurtosis, PreT1 standard deviation.
**Conclusion:** Based on machine learning analysis, entropy parameters and no or fine scale filtration are recommended for texture analysis at breast MRI in predicting prognostic biomarkers and molecular subtypes of breast cancer.
**Limitations:** External validation wasn't performed.
**Ethics Committee Approval:** IRB approved.
**Funding for this study:** Supported by National Research Foundation (NRF) of Korea grant funded by the Ministry of Science and ICT of Korea (No. NRF-2020R1F1A1073213) and the Korea University grant (No. K2008291).
**Author Disclosures:**
Okhee Woo: Nothing to disclose
Eun Kyung Park: Nothing to disclose
Bo Kyoung Seo: Nothing to disclose
Sung Eun Song: Nothing to disclose
Kwang-Sig Lee: Nothing to disclose
Ji Young Lee: Nothing to disclose
Hyun Soo Park: Nothing to disclose
Kyu Ran Cho: Nothing to disclose

### RPS 702-10

### DCE-MRI radiomics analysis for breast lesions characterisation

*M. Di Marco*[1], A. A. M. Orlando[1], I. D'Angelo[2], C. Militello[2], L. Rundo[3], T. V. Bartolotta[2]; [1]Palermo/IT, [2]Cefalu/IT, [3]Cambridge/UK
*(maridimarco33@gmail.com)*

**Purpose:** To propose a radiomic signature, extracted from DCE-MRI datasets, able to predict focal breast lesions (FBLs) characterization.
**Methods or Background:** 91 FBLs (mean size: 16.85 mm; size range: 5-60 mm), found with Breast DCE-MRI in 91 patients (mean age: 47.67 years; age range: 24-72 years), underwent radiomic analysis by using a manual segmentation method performed by an experienced breast radiologist. PyRadiomics software was used to extract 107 radiomics features. A feature calibration and pre-processing step was performed to select only robust non-redundant features. An in-depth analysis was performed to build a predictive model: for this purpose, a Support Vector Machine (SVM) was trained in a nested 5-fold cross-validation scheme, by exploiting 5 different feature selection methods.
**Results or Findings:** The 5 radiomic models built showed good and similar predictive performance in terms of AUC, specificity, sensitivity and accuracy, with similar 'pattern of features' selected. Best results were given, in particular, by DGUFS feature selection method, obtaining: AUC = 0.72, sensitivity = 0.68, specificity = 0.76, and accuracy = 0.72. The best predictive model obtained by means of DGUFS feature selection method, consists of a signature composed of 4 radiomic features: Flatness, Least Axis Length and Elongation (shape features), and Dependence Non-Uniformity Normalized (Gray Level Dependence Matrix feature).
**Conclusion:** We built a radiomic predictive model able to characterize breast lesions in DCE-Breast MRI, showing promising results in terms of accuracy and specificity.
**Limitations:** Our study is a single-center study without large numbers.
Only manual annotation was considered and used, only regarding the intratumoral component.

**Ethics Committee Approval:** Ethics committee approval obtained
**Funding for this study:** None
**Author Disclosures:**
Alessia Angela Maria Orlando: Nothing to disclose
Mariangela Di Marco: Nothing to disclose
Ildebrando D'Angelo: Nothing to disclose
Carmelo Militello: Nothing to disclose
Leonardo Rundo: Nothing to disclose
Tommaso Vincenzo Vincenzo Bartolotta: Nothing to disclose

### RPS 702-11

**Using AI to identify normal cases that may not need a second reader assessment in a French breast cancer screening program (BCSP): a retrospective evaluation**

*H. Jarraya*[1], Y. Damiens[1], C. Venel[1], M. Legros[1], C. Brochart[1], J. Rais[1], M. Medjahdi[1], T. Petit[2], A. Rodriguez Ruiz[3]; [1]Arras/FR, [2]Amiens/FR, [3]Nijmegen/NL
*(hajer.jerraya@gmail.com)*

**Purpose:** To retrospectively investigate an AI system as aid to first reader to identify which cases do not need double reading because very likely normal in BCSP with digital mammography (DM).
**Methods or Background:** A cohort of 998 DM was collected and enriched with additional 43 detected cancers (52 cancers in total). All DM (and their prior) were processed by an AI system (Transpara, version 1.6.0, ScreenPoint Medical), which categorizes them on a scale 1-10 representing the risk of abnormalities. Breast density was also recorded. Different cutoff points of AI were investigated to define the largest group of screening exams with the lowest likelihood of cancer. Binomial method was used to compute 95% confidence intervals (CI) of the distributions.
**Results or Findings:** Detection wise, 49/52 detected cancers had an AI risk score of 8 or higher. Using AI to identify likely normal exams, cases with risk score 4 or lower included 0% (CI: 0-7.7) of cancers and 29% (CI: 26-32) of total screening volume. When taking breast density into account, cases with risk scores 7 or lower and with low breast density (A or B) included 0% (CI: 0-7.7) of cancers and 47% (CI: 44-50) of total screening volume.
When looking at prior exams 2 years earlier, 43% (CI: 20-70) of detected cancers already had the highest AI risk score of 10.
**Conclusion:** AI could be used in screening programs as aid to the first reader to identify 29% of very likely normal mammograms where double reading may not be necessary. Combined with breast density, this volume could be increased to 43%.
**Limitations:** More mammograms and more diversity of data (different vendors) are needed to understand the generalisability of our results.
**Ethics Committee Approval:** Internal ethics commitee approval was obtained
**Funding for this study:** None
**Author Disclosures:**
Cédric Brochart: nothing to disclose
Yoann Damiens: Nothing to disclose
Alejandro Rodriguez Ruiz: Employee: Screenpoint
Hajer Jarraya: nothing to disclose
Mylène Legros: nothing to disclose
Jamal Rais: Nothing to disclose
Coline Venel: Nothing to disclose
Mustapha Medjahdi: Nothing to disclose
Thomas Petit: nothing to disclose

### RPS 702-12

**Comparison of artificial intelligence/machine learning algorithm in reporting computed radiography (CR) and digital radiography (DR) mammography studies**

*R. Ananthasivan*[1], P. G. Patil[1], K. Garg[2], S. Bevoor Chowdaiah[1], H. H. Prabhakaran[2], K. Ravindran[2], V. Raj[1]; [1]Bangalore/IN, [2]Chennai/IN
*(rupanth@yahoo.com)*

**Purpose:** Systems utilising Artificial Intelligence (AI)/Machine Learning(ML) have shown to be highly accurate in reporting mammography studies. Digital radiography(DR) has become routine practice in most of the developed world and these AI/ML platforms have largely been trained in DR datasets. In many developing nations, Computed Radiography(CR) mammography is still in practice. We compare the accuracy of an AI system in picking up abnormalities between a CR and a DR mammography study.
**Methods or Background:** Mammography images from a tertiary referral centre and a teleradiology unit, between Nov 2018 to Feb 2020 were collated. Both CR (computed radiography) and DR (digital radiography) images were available. Images were divided into training and testing groups randomly and the training dataset was annotated by a team of senior radiologists. Cases from testing sets were reported by four radiologists and compared against the performance of the AI/ML generated reports.
**Results or Findings:** A total of 18908 examinations were available, of these 7675 were CR (41%). Training set consisted of 9960 (70% of these were CR). A total of 3042 cases (from the testing set) were reported, of these 603 (20%) were CR and 2439 were DR. Two radiologists reported 100 CR cases each. The sensitivity of the AI/ML system in reporting CR and DR was 97% vs 98% respectively. The specificity and accuracy of both CR and DR were similar at 93 and 95% respectively. There was no significant difference in the performance of the system in the CR dataset.
**Conclusion:** There is no significant difference in the interpretation of CR or DR dataset by the indigenously developed AI/ML system. High levels of accuracy were seen in both types of films.
**Limitations:** Larger adoption needs to be tested.
**Ethics Committee Approval:** Appropriate permission has been taken
**Funding for this study:** None
**Author Disclosures:**
Sanjana Bevoor Chowdaiah: Nothing to disclose
Vimal Raj: Advisory Board: CHS World
Hari Haran Prabhakaran: Founder: CHS World
Krithika Ravindran: Board Member: CHS World
Kushagra Garg: Investigator: CHS World
Rupa Ananthasivan: Nothing to disclose
Pooja G Patil: Nothing to disclose

### RPS 702-13

**Mammographic breast density assessment via deeply aggregating bilateral context information**

X. Lin[1], *M. Jie*[1], Y. Ouyang[1], M. Wu[1], S. Wu[1], Q. Diao[1], L. Huang[1], J. Xiao[1], Y. Zhang[2]; [1]Shenzhen/CN, [2]Palo Alto/US

**Purpose:** To increase the performance and reproducibility of mammographic breast density measurement, we develop an assessment network utilizing deeply aggregating bilateral contextual information.
**Methods or Background:** We collected 42154 cases of screening and diagnosis mammograms of two manufacture facilities from in-house hospital. All mammographic density categories were made sure by a senior radiologist and a junior one. In related works, density classification methods were based on a single image. In practical verification, large lesions in image did disrupt the density classifier. To tackle this problem, we proposed a novel density classifier based on residual pyramid feature network, including two branches: a single inputting classification branch, and the other is a dual inputting which aggregates bilateral contextual information to improve performance and reduce perturbation of lesions. Additionally, we only used a size 800 x 448 of Cranio-Caudal (CC) image duel to Medio-Lateral Oblique (MLO) consisting of pectoral muscle and being of a phenomenon of glandular backlog.
**Results or Findings:** To evaluate the classification performance, we had tested the model on an in-house mammogram dataset involving 1686 cases that were re-annotated by a senior radiologist. Our two- and four-classification model achieved accuracies of 0.953 and 0.858, respectively. The corresponding area under the receiver operating characteristic curve AUC and mac-AUC are 0.941 and 0.915, respectively.
**Conclusion:** As one of the most important breast cancer risk factors, breast density is usually measured on radiologist's daily work. In this retrospective study, the experimental results indicated well distinguished density categories. It's able to contribute to decrease the inconsistency of mammographic density judgment between radiologists and improve density quantification, promoting analysis of lesion nature and risk assessment of breast cancer.
**Limitations:** The bias of race difference is not well considered.
**Ethics Committee Approval:** The IRB number is LL-XJS-2020011.
**Funding for this study:** n/a
**Author Disclosures:**
Ma Jie: Nothing to disclose
Yu Ouyang: Nothing to disclose
Qingchen Diao: Nothing to disclose
Shibin Wu: Nothing to disclose
Mingxiang Wu: Nothing to disclose
Jing Xiao: Nothing to disclose
Lingyun Huang: Nothing to disclose
Yanbo Zhang: nothing to disclose
Xiaohui Lin: Nothing to disclose

### Research Presentation Session: Cardiac

### RPS 303

### Ischaemia and infarction

### RPS 303-1

**Identify ischemic, infarcted, hibernant, and normal myocardium by stress and rest T1 mapping without the application of gadolinium contrast agents**

*B. Zhuang*, M. Lu; Beijing/CN
*(zbyan10@163.com)*

**Purpose:** In chronic coronary artery disease, accurate detection of ischemic and hibernant myocardium is important because targeted revascularization improves clinical outcomes.The aim of this study was to evaluate the potential of T1 mapping at rest and during adenosine triphosphate stress for the detection of ischemic, hibernant and infarct myocardium in a swine model using pathology as gold reference.
**Methods or Background:** Twelve adult male Chinese miniature swine with the induction of chronic coronary artery stenosis were enrolled in this study. The CMR imaging was performed at four time points: baseline, 1 week-, 2 weeks- and 4 weeks- after surgery. Pre- and post-contrast T1 mapping at rest and during adenosine triphosphate stress, first-pass perfusion and stress perfusion as well as late gadolinium enhancement were performed at all imaging time points. Myocardial perfusion reserve index(MPRI) were calculated. The native T1, extracelluar volume (ECV) and their percentage were also calculated.
**Results or Findings:** The rest native T1 value of infarct, hibernant, ischemia and normal myocardium were 1586.4±159.0ms, 1350.7±165.5ms, 1218.8±153.4ms, 1101.8±107.7ms, respectively. The rest ECV value of infarct, hibernant, ischemia and normal myocardium were 73.89±8.27, 40.25±7.77, 34.09±3.00, 24.48±2.71, respectively. MPRI were associated with the change of native T1 (stress native T1 minus rest native T1, △native T1) (R=-0.45, p=0.005) and native T1 percentage (△native T1/ rest native T1) (R=-0.50, p=0.034). Besides, there were correlation observed between MPRI and the change of ECV (R=-0.41, p<0.001) and ECV percentage (R=-0.41, p=0.005).
**Conclusion:** We identified the native T1 and ECV values of four myocardium types in chronic ischemic cardiomyopathy. The rest/stress T1 mapping has the potential to detect ischemia and hibernation without the need for gadolinium contrast.
**Limitations:** There are some limitations
**Ethics Committee Approval:** This study is approved by Fuwai Hospital
**Funding for this study:** There is no funding
**Author Disclosures:**
Minjie Lu: Nothing to disclose
Baiyan Zhuang: Nothing to disclose

### RPS 303-2

**Compressed SENSE for acceleration of multi-beath-hold short-axis cine in clinical routine**

*L. Pennig*[1], A-I. Iuga[1], C. Nelles[1], S. Lennartz[1], A. Bunck[1], D. Maintz[1], K. Weiss[2], C. P. Naehle[1], J. Dörner[1]; [1]Cologne/DE, [2]Hamburg/DE
*(lenhard.pennig@uk-koeln.de)*

**Purpose:** To compare image quality and diagnostic performance of Compressed SENSE (CS) accelerated multi-breath-hold short-axis (SAx) cine for assessment of LV function, volumes, and mass to standard multi-breath-hold SAx cine using SENSE in clinical routine.
**Methods or Background:** Retrospective, single-center study of 50 consecutive patients (40.4 ± 18.1 years, 22 females; August–October 2019), who received a CMR protocol in clinical routine at 1.5T including both, CS (factor 4.5) and SENSE (factor 2) accelerated multi-breath-hold SAx cine sequences with otherwise equal scanning parameters. Two radiologists, blinded to the type of sequence and clinical data, independently performed qualitative analysis of image quality (5-point scale, 1 non-diagnostic - 5 excellent), quantitative analysis of LV volumetric and functional measurements, and visual analysis of LV wall-motion disorders (WMD).
**Results or Findings:** CS allowed for a significant reduction of overall scan time (CS: 04:31 ± 00:46 min, SENSE: 08:53 ± 01:26 min; P<.0001) with decreased number (7 vs. 14) of breath-holds and increased number of acquired slices per breath-hold (2 vs. 1). Image quality was unchanged (CS: median 4 [min 2 - max 5], SENSE: 4 [1-5]; P=.337). For all quantitative LV measurements, no significant differences (P>.05) between CS and SENSE accelerated sequences were observed while interobserver agreement was excellent for both techniques with ICCs ranging between 0.972 and 0.999. Considering SENSE as reference standard, WMD were depicted on CS cine with 100% sensitivity and specificity, respectively.
**Conclusion:** Compared to acceleration using SENSE, Compressed SENSE (factor 4.5) reduces scan time of multi-breath-hold SAx cine to 50% while maintaining high image quality and unimpaired diagnostic performance.
**Limitations:** Retrospective.
**Ethics Committee Approval:** Given.
**Funding for this study:** N/a.
**Author Disclosures:**
Simon Lennartz: Research/Grant Support: Philips Healthcare
Lenhard Pennig: Research/Grant Support: Philips Healthcare
Christian Nelles: Nothing to disclose
David Maintz: Speaker: Philips Healthcare
Andra-Iza Iuga: Nothing to disclose
Claas Philip Naehle: Nothing to disclose
Kilian Weiss: Employee: Philips Healthcare
Alexander Bunck: Nothing to disclose
Jonas Dörner: Nothing to disclose

### RPS 303-3

**Prognostic value of dipyridamole stress CMR in patients with known or suspected coronary artery disease: a long term follow-up study**

A. Meloni[1], C. Nugara[2], A. De Luca[3], C. Cavallaro[4], C. Cappelletto[3], G. Novo[2], F. Grigioni[4], G. Sinagra[3], *A. Pepe*[1]; [1]Pisa/IT, [2]Palermo/IT, [3]Trieste/IT, [4]Rome/IT
*(alessia.pepe@ftgm.it)*

**Purpose:** We determined the long-term prognostic value of dipyridamole stress-CMR in patients with known or suspected coronary artery disease (CAD).
**Methods or Background:** 246 consecutive patients (61 females, 61.96±10.05 years) were considered. Abnormal wall motion at rest and after dipyridamole, perfusion at stress and at rest, and late gadolinium enhancement (LGE) were analysed.
End-points were "major non-fatal cardiac events" (ventricular arrhythmias, coronary syndromes, heart failure hospitalization ) and cardiac death.
**Results or Findings:** An abnormal stress CMR was found in 65(26.4%) patients: 39 patients had a reversible stress perfusion defect in >one myocardial segment and 26 a reversible stress perfusion defect plus worsening of stress wall motion in comparison with rest. During a median follow up of 60.59 months, 69(28.0%) patients experienced major nonfatal cardiac events. LGE, reversible perfusion deficit, age, diabetes and family history were univariate prognosticators. In the multivariate analysis the independent predictive factors were reversible perfusion deficit (hazard ratio-HR=2.21, P=0.001) and diabetes (HR=2.21, P=0.003). Ten patients died during follow-up and reversible motion abnormality and LGE were univariate prognosticators. At multivariate analysis, LGE remained a significant prognosticator (HR=10.83, P=0.026), after adjusting for age and diabetes. When the composite end-point (cardiac events+death) was considered, both myocardial fibrosis and reversible perfusion deficit resulted to be significant univariate prognosticators, in addition to age, diabetes and family history. At multivariate analysis the independent predictive factors were reversible perfusion deficit (HR=2.26, P<0.0001) and diabetes (HR=2.59, P<0.0001).
**Conclusion:** Reversible perfusion deficit and diabetes identify patients at high risk of fatal and non fatal cardiac events. LGE is a strong predictor for death in patients with known or suspected CAD.
**Limitations:** Single-center study
**Ethics Committee Approval:** Local ethics committee
**Funding for this study:** No funding received
**Author Disclosures:**
Alessia Pepe: Nothing to disclose
Gianfranco Sinagra: Nothing to disclose
Chiara Cappelletto: Nothing to disclose
Antonio De Luca: Nothing to disclose
Giuseppina Novo: Nothing to disclose
Francesco Grigioni: Nothing to disclose
Cinzia Nugara: Nothing to disclose
Antonella Meloni: Nothing to disclose
Camilla Cavallaro: Nothing to disclose

### RPS 303-5

**Detection of myocardial fibrosis in chronic coronary heart disease by cardiac MRI**

*A. S. Abramenko*, M. Vishnyakova, M. Vishnyakova, D. Shumakov, G. Shekhyan; Moscow/RU
*(a.s.abramenko@gmail.com)*

**Purpose:** Analyze magnetic resonance imaging capabilities in imaging of diffuse and focal myocardial fibrosis in chronic coronary heart disease (CHD).
**Methods or Background:** 50 patients were examined in 2019-2020 because of CHD. Cardiac MRI including delayed enhancement (MDE) was performed on GE Optima MR450w GEM 1.5T with a surface cardiac coil. For assessment of diffusion myocardial fibrosis 2D MOLLI (3-3-5) technique was used with further semi-automatic myocardial segmentation (software CVI42) and calculation of T1 native and post contrast relaxation times, and extracellular volume fraction (ECV).
**Results or Findings:** The average T1 relaxation time in the entire study group was 1127 ± 45 ms (signs of diffuse left ventricular myocardial fibrosis). All patients showed focal fibrosis: without aneurysm formation 16%, with aneurysm formation 84%, of which 26% with thrombotic masses. In the group of patients who did not have aneurysm formation, the mean value of the native T1 value was 1080 ± 45 ms, while in patients with aneurysm formation 1125 ± 47 ms (p = 0,041). After MDE, the estimated ECV in the group of patients who did not show aneurysm formation was 41 ± 3.5% and in patients with aneurysm formation was 44 ± 2.5% (p = 0,044). Male patients showed a large myocardial mass of 164 ± 43 gr versus 126 ± 23 gr in women (p = 0.014).
**Conclusion:** CMR allows to identify and give a detailed characteristic of left ventricular fibrous changes: MDE for qualitative assessment of focal fibrosis, automatic segmental mode of analysis of left ventricular contractility, T1 mapping technique for determining diffuse myocardial fibrosis. T1 mapping showed the presence of diffuse fibrosis in the entire cohort of patients.
**Limitations:** Without limitations.
**Ethics Committee Approval:** Approved.
**Funding for this study:** No funding was received for this study.
**Author Disclosures:**
Marina Vishnyakova: Nothing to disclose
Maria Vishnyakova: Nothing to disclose
Dmitry Shumakov: Nothing to disclose
Grant Shekhyan: Nothing to disclose
Alexander S. Abramenko: Nothing to disclose

### RPS 303-6

**Assessment of the accuracy of optimised IQ.SPECT and echocardiography in assessing left ventricle ejection fraction (LVEF) measurements: a comparison with cardiac MRI on a wide range of LVEF values**
*Y. Bouchareb*, H. Al Dhuhli, H. Sulaiman, N. Tag, A. Al Riyami, J. Zabah; Muscat/OM

**Purpose:** To assess the accuracy of optimised IQ.SPECT, 2D and 3D echocardiography in patients suspected with cardiac abnormalities by comparison to cardiac MR (cMR), as a reference standard, on a wide range of LVEF measurements.
**Methods or Background:** This prospective study included twenty-three patients who underwent a Tc-99m sestamibi stress and rest SPECT/CT, 2D/3D echocardiography and cMR scans. All three scans were performed within 2 weeks. The cMR and 2D/3D echocardiography scans were processed using cardiac software packages available on the Symbia.net workstation and the Echo machine, respectively. To optimise the IQ.SPECT method, perfusion and gated raw data were reconstructed using the resolution recovery-based TurboFlash method by varying the number of iterations, number of subsets and filter size. The optimised series was considered the one which provided the closest LVEF values to cMR and improved visually perfusion images (assessed by 2 NM physicians). The IQ.SEPCT reconstructed series were processed using the Cedars-Sinai software. The LVEF measurements from optimised IQ.SPECT, routine IQ.SPECT, 2D/3D echocardiography were compared to the ones obtained by cMR (LVEF range: 26 to 73) by means of the mean percentage difference (MPD).
**Results or Findings:** In terms of LVEF measurements, a MPD of 3%, 19%, 12% and 14% was obtained between optimised IQ.SPECT (using “14-iterations, 3-subsets, 8mm Gaussian-filter”), routine IQ.SPECT (using “12-iterations, 1-subset, 10mm Gaussian-filter”), 2D- and 3D-echocardiography and cMR, respectively. The visual assessment of the optimised IQ.SPECT images provided better image quality compared to routine IQ.SPECT images. A substantial inter-operator agreement (kappa=0.74) was obtained between the 2 assessors.
**Conclusion:** The optimised IQ.SPECT method improves the accuracy of LVEF measurements compared to routine IQ.SPECT and 2D/3D echocardiography as well as provides better quality of perfusion images.
**Limitations:** Not a large patient population.
**Ethics Committee Approval:** Yes.
**Funding for this study:** Internal-Grant, Sultan Qaboos University.
**Author Disclosures:**
Naima Tag: Nothing to disclose
Yassine Bouchareb: Grant Recipient: Principal Investigator
Adil Al Riyami: Nothing to disclose
Hajir Sulaiman: Investigator: Research Assistant at Sultan Qaboos University paid from the grant.
Jawa Zabah: Nothing to disclose
Humoud Al Dhuhli: Other: Co-Principal Investigator

### RPS 303-7

**Incidental cardiac uptake in bone scintigraphy: the increased importance and association with cardiac amyloidosis**
*F. Delaney*, M. J. O'Connell; Dublin/IE

**Purpose:** Extraosseous radiotracer uptake during bone scintigraphy may signify previously undiagnosed disorders. There has been renewed focus on cardiac uptake, commonly attributed to ischaemic heart diease, in recent years due to developments in the management of cardiac amyloidosis. We review the role of scintigraphy in cardiac amyloidosis and the importance of appropriate reporting of incidental cardiac uptake.
**Methods or Background:** The recent literature on cardiac amyloidosis and bone scintigraphy is described and complemented by review of bone scintigraphy studies showing incidental cardiac uptake at our institution.
**Results or Findings:** Cardiac uptake of bone avid radiotracers is highly sensitive and specific for the diagnosis of transthyretin (ATTR) amyloidosis, an under-recognised cause of diastolic heart failure. Dedicated cardiac scintigraphy now plays a key role in the non-invasive diagnostic algorithm for ATTR amyloidosis and may help in its early detection which is increasingly important as novel therapies emerge. Myocardial ischaemia/infarction can cause cardiac uptake but this is typically more focal and may not persist 7-10 days beyond the ischaemic event. Cardiac uptake must be carefully assessed in conjunction with clinical information, therefore, to ensure it is not falsely attributed to coronary artery disease. Multiple large retrospective reviews of bone scans have consistently identified incidental cardiac uptake. While further study is required to fully define the appropriate approach to investigating this, it should prompt clinical assessment and consideration of echocardiography. We illustrate this with cases of incidental cardiac uptake leading to diagnosis of ATTR amyloidosis.
**Conclusion:** Cardiac uptake during bone scintigraphy must be carefully interpreted as there is the potential to detect subclinical ATTR amyloidosis. Cardiac scintigraphy plays a crucial and evolving role in the management of cardiac amyloidosis and an up-to-date knowledge is important for radiologists.
**Limitations:** Not Applicable
**Ethics Committee Approval:** Not Applicable
**Funding for this study:** None
**Author Disclosures:**
Francis Delaney: Nothing to disclose
Martin Joseph O'Connell: Nothing to disclose

### RPS 303-8

**Myocardial mapping as a marker of subclinical myocardial injury in patients with active Coronavirus disease 19**
*L. Marchitelli*, F. Catapano, G. Cundari, G. Pambianchi, N. Galea, C. Catalano, M. Francone; Rome/IT
*(liviamarchitelli92@gmail.com)*

**Purpose:** Subclinical myocardial injury is not uncommon in COVID-19 and recognizes a complex multifactorial pathogenesis. We hypothesized that CMR with myocardial mapping techniques would allow non-invasive detection of myocardial structural damage in patients with active infection.
**Methods or Background:** We prospectively enrolled 22 consecutive patients (18 M and 4 F) with active SARS-CoV-2 infection (positive RT-PCR on nasopharyngeal swab). In all patients, routine blood tests, including and high sensitive troponin (HsTn), were available. A CMR multiparametric evaluation was carried out including mapping sequences. CMR quantitative parameters were matched with clinical and laboratory findings, including white blood cells (WBC), C-reactive Protein (PCR) and troponin levels. Both uni- and multivariate analyses (Chi-squared, t-test, Pearson correlation coefficient and regression model) were performed to evaluate most significant predictors of myocardial injury.
**Results or Findings:** CMR T1 and T2 values were increased in 8/22 and 12/22 patients, respectively, ranging from 994 to 1210 ms for T1 values and 47 and 66 ms for T2. Left ventricle ejection fraction was impaired in 10/22 patients, ranging from 41% to 60%. All patients were classified as “mild” according to Chinese CDC clinical scoring for SARS-CoV-2 infection. A significant correlation was found between T1 map, T2map and ECVmap alterations and increase HsTn ($p<0,05$). The regression model analysis also showed a significant relationship between WBC, PCR, HsTn and T2 value.
**Conclusion:** CMR mapping predicts subclinical myocardial damage in COVID-19 active disease and correlates with WBC, PCR and HsTn levels. Underlying pathological substrate likely includes a combination of tissue edema and myocarditis-like fibrosis/necrosis changes, suggesting a potential impact of CMR findings on prognosis and clinical decision-making.
**Limitations:** Not applicable
**Ethics Committee Approval:** Not applicable
**Funding for this study:** Not applicable

**Author Disclosures:**
Giacomo Pambianchi: Nothing to disclose
Giulia Cundari: Nothing to disclose
Livia Marchitelli: Nothing to disclose
Marco Francone: Nothing to disclose
Federica Catapano: Nothing to disclose
Nicola Galea: Nothing to disclose
Carlo Catalano: Nothing to disclose

### Research Presentation Session: Cardiac

### RPS 403

### Haemodynamics and blood pressure

### RPS 403-1

**Comparison of CMR-based models for prediction of the mean pulmonary arterial pressure**

*V. Nizhnikava*, U. Reiter, G. Kovacs, C. Reiter, C. Kräuter, H. Olschewski, M. Fuchsjäger, G. Reiter; Graz/AT
*(volha.nizhnikava@gmail.com)*

**Purpose:** Various published studies present promising models for non-invasive estimation of the mean pulmonary arterial pressure (mPAP) based on parameters derived from cardiac magnetic resonance (CMR). The aim of the present study was to compare proposed models for mPAP estimation using right heart catheterization (RHC) as reference.
**Methods or Background:** 51 patients (female/male, 28/23; age, 67±9years) who underwent comprehensive native 3T CMR and RHC within 2±3 days were recruited in the prospective study. CMR protocol included cine short-axis imaging, 2- (2D), and 4-dimensional (4D) flow imaging of the pulmonary artery (PA). All functional and 2D flow measurements required for MR-based estimation of mPAP (MR-mPAP) in 14 out of 15 published models were evaluated using routine software (cvi42, Circle Cardiovascular Imaging). 4D flow-derived vortex duration along the main PA (VD) for estimation of MR-mPAP according to model MR-mPAP = 16+0.63*VD was evaluated with prototype software (4DFlow, Siemens Healthcare). Relationships between mPAP and MR-mPAPs were analyzed by correlation and Bland-Altman analysis.
**Results or Findings:** Out of 15 models 5 estimated MR-mPAPs revealed strong correlations with mPAP. The strongest correlation among the models using only cine-derived function parameters showed MR-mPAP=-4,6 + 0.23*IVSA + 16.3*VMI (IVSA, intraventricular septal angle; VMI, ventricular mass index) with r=0.82; among the models involving cine function together with 2D flow parameters it was MR-mPAP=-21.806 + 0.31*IVSA + 11.5*VMI + 0.01*minPA+0.22*RAC (minPA, minimal PA cross-sectional area; RAC, PA relative area change) with r=0.83. The strongest correlation (r=0.96) among all 15 models was found for MR-mPAP evaluated from 4D-flow data. The standard deviations of differences between MR-mPAP and mPAP for these three models were 9mmHg, 9mmHg, and 4mmHg, respectively.
**Conclusion:** CMR-derived function and flow parameters allow non-invasive mPAP estimation with 4D-flow imaging providing the most accurate results.
**Limitations:** Small number of subjects
**Ethics Committee Approval:** Obtained
**Funding for this study:** OeNB-Anniversary-Fund No.17934, ESOR Fellowship 2020
**Author Disclosures:**
Horst Olschewski: Nothing to disclose
Ursula Reiter: Nothing to disclose
Gert Reiter: Employee: Siemens Healthcare Diagnostics GmbH, Graz, Austria
Michael Fuchsjäger: Nothing to disclose
Clemens Reiter: Nothing to disclose
Gabor Kovacs: Nothing to disclose
Volha Nizhnikava: Nothing to disclose
Corina Kräuter: Nothing to disclose

### RPS 403-2

**Assessment of aortic and pulmonary flow volumes: acceleration by compressed sensing**

*U. Reiter*[1], C. Reiter[1], C. Kräuter[1], V. Nizhnikava[1], D. Giese[2], M. Fuchsjäger[1], G. Reiter[1]; [1]Graz/AT, [2]Erlangen/DE
*(ursula.reiter@medunigraz.at)*

**Purpose:** Cardiac magnetic resonance two-dimensional (2D) through-plane flow imaging during breath-holding, the well-established technique for the assessment of aortic and pulmonary flow volumes and volume ratios, could benefit from scan time reduction. The purpose of the present study was to investigate if 2D flow measurements can be substantially accelerated by compressed sensing (CS).
**Methods or Background:** 20 prospectively recruited cardiac patients without known or suspected shunts and with regular heart rhythm underwent retrospectively ECG-gated, breath-hold through-plane 2D flow imaging perpendicular to the aorta and the pulmonary artery at 3T (Magnetom Skyra, Siemens Healthcare). Both measurements were performed twice, once without CS and once employing a prototype sequence with a parameter-matched CS protocol with a CS-acceleration-factor of 7.6. Baseline-corrected aortic (Qa) and pulmonary (Qp) net flow volumes were evaluated from data sets using routine software (cvi42, Circle Cardiovascular Imaging). Relationships between results derived with and without CS were analyzed by correlation analysis; means and variances were compared employing t- and Levene test, respectively.
**Results or Findings:** Employing CS, scan times were significantly reduced (4.8±1.2s vs. 9.4±2.2s, p<0.01). Flow volumes from CS flow measurements (Qa=74±26ml; Qp=75±27ml) had no significant bias compared to measurements without CS (Qa=74±27ml, p=0.62; Qp=76±30ml, p=0.55) and demonstrated strong correlations (r=0.98 in both cases). Pulmonary-to-systemic blood flow ratios (Qp/Qa=1.02±0.11 and Qp/Qa=1.02±0.08, with and without CS, respectively) did not differ significantly, either in means (p=0.96) or in variances (p=0.10).
**Conclusion:** Compressed sensing allows for substantial acceleration of 2D flow acquisition without significant impact on the precision of derived aortic and pulmonary flow volumes.
**Limitations:** Small sample size.
**Ethics Committee Approval:** Obtained.
**Funding for this study:** OeNB-Anniversary-Fund No.17934, ESR Seed Grant 2020, ESOR Fellowship 2020.
**Author Disclosures:**
Daniel Giese: Employee: Siemens Healthcare GmbH, Erlangen, Germany
Ursula Reiter: Nothing to disclose
Gert Reiter: Employee: Siemens Healthcare Diagnostics GmbH, Graz, Austria
Michael Fuchsjäger: Nothing to disclose
Clemens Reiter: Nothing to disclose
Volha Nizhnikava: Nothing to disclose
Corina Kräuter: Nothing to disclose

### RPS 403-3

**Effects of regadenoson on the left ventricular ejection fraction in stress-cardiovascular magnetic resonance**

*A. C. Igual Rouilleault*, I. Soriano Aguadero, P. Malmierca Ordoqui, A. Paternain Nuin, J. C. Pueyo Villoslada, G. Bastarrika Alemañ, A. Ezponda Casajus; Pamplona/ES
*(aigual@unav.es)*

**Purpose:** Regadenoson is recently used as a vasodilator drug to induce stress in cardiovascular magnetic resonance (CMR). The aims of our study are: - To determine the impact of regadenoson on the hemodynamic and volumetric parameters. - To compare the pre- and post- regadenoson stress values of the ejection fraction (EF).
**Methods or Background:** We studied prospectively a total of 45 patients undergoing a stress-CMR because of clinical ischemia suspicion. Exclusion criteria included prior history of ventricular dysfunction or atrial fibrillation. Short-axis steady-state free-precession (SSFP) sequences with ECG synchronization were performed before and after the intravenous administration of regadenoson. All the images were transferred to Circle Cardiovascular software (CMR42). Ventricular volumes and EF were calculated before and immediately after the administration of the stressor. Two-tailed-p values were used for all statistical assessment and a p-value<0.05 was considered statistically significant.
**Results or Findings:** We included 45 subjects (72.1 % men, mean age 63±9.5years). 39.5% of the participants had a previous history of ischemic cardiopathy. During the stress, the average increase of heart rate was 23.3 ± 12.5 bpm and the mean decrease in systolic and diastolic blood pressure (SBP and DBP) was 8.8 ± 21.3 mmHg and 4.9 ± 9.6 mmHg, respectively. Differences were also seen analyzing ventricular volumes: after regadenoson administration, end-systolic volume decreases whereas EF and stroke volume increase.
**Conclusion:** Regadenoson entails a mild overestimation of EF and stroke volume of both ventricles. In patients undergoing stress-CMR with regadenoson in whom biventricular volumes and EF are critical components for clinical decisions, short-axis SFFP sequences should be performed before the administration of regadenoson.
**Limitations:** -High number of patients with Killip class I (90%).
-An overall quite young.
**Ethics Committee Approval:** Not applicable.
**Funding for this study:** No.

**Author Disclosures:**
Ignacio Soriano Aguadero: Nothing to disclose
Patricia Malmierca Ordoqui: Nothing to disclose
Jesus Pueyo Villoslada: Nothing to disclose
Alba Cristina Igual Rouilleault: Nothing to disclose
Ana Ezponda Casajus: Nothing to disclose
Gorka Bastarrika Alemañ: Speaker: SIEMENS Speaker: BAYER
Alberto Paternain Nuin: Nothing to disclose

### RPS 403-4

**The additive prognostic value of end-systolic pressure-volume relation by stress CMR in patients with known or suspected coronary artery disease**

A. Meloni[1], A. De Luca[2], C. Nugara[3], C. Cappelletto[2], A. Barison[1], G. Todiere[1], G. Novo[3], G. Sinagra[2], *A. Pepe*[1]; [1]Pisa/IT, [2]Trieste/IT, [3]Palermo/IT
*(alessia.pepe@ftgm.it)*

**Purpose:** We assessed for the first time the prognostic value of delta rest-stress end-systolic pressure-volume relation (ΔESPVR), an index of left ventricular contractility, by dipyridamole stress-CMR in patients with known or suspected coronary artery disease (CAD).
**Methods or Background:** We considered 196 consecutive patients (49 females, 62.74±10.66 years). Abnormal wall motion and perfusion at rest and after dipyridamole were analysed. Macroscopic myocardial fibrosis was detected by the late gadolinium enhancement (LGE) technique. The ESPVR was evaluated at rest and peak stress from raw measurement of systolic arterial pressure by cuff sphygmomanometer and end-systolic volume by biplane Simpson method.
**Results or Findings:** An abnormal stress CMR was found in 52 (26.5%) patients: 36 with reversible stress perfusion defect in >one myocardial segment and 16 with reversible stress perfusion defect plus worsening of stress wall motion in comparison with rest. LGE sequences were acquired in 170 (86.7%) patients and myocardial fibrosis was detected in 90 patients (52.9%). A ΔESPVR index≤0.02 mmHg/mL/m2 was found in 88 patients (44.9%). Mean follow-up time was 53.17±28.21 months. Cardiac events were recorded in 50 (25.5%) patients: 5 cardiac deaths, 17 revascularizations for angina or myocardial infarction, one myocardial infarction, 23 hospitalisations for heart failure or unstable angina, and 4 ventricular arrhythmias. According to the Cox analysis, diabetes, family history, LVEF, abnormal stress CMR, myocardial fibrosis, and reduced ΔESPVR were significant univariate prognosticators. In the multivariate analysis the independent predictive factors were diabetes (hazard ratio-HR=2.33, 95%CI=1.21-4.51, P=0.012), a ΔESPVR index≤0.02 mmHg/mL/m2 (HR=2.58, 95%CI=1.29-5.13, P=0.007), and myocardial fibrosis (HR=2.13, 95%CI=1.05-4.31, P=0.036).
**Conclusion:** ΔESPRV assessed by CMR provides a prognostic stratification in patients with known or suspected coronary artery disease, in addition to that supplied by diabetes and myocardial fibrosis.
**Limitations:** Single-center study
**Ethics Committee Approval:** Local ethics committee
**Funding for this study:** No funding received
**Author Disclosures:**
Alessia Pepe: Nothing to disclose
Gianfranco Sinagra: Nothing to disclose
Chiara Cappelletto: Nothing to disclose
Antonio De Luca: Nothing to disclose
Giancarlo Todiere: Nothing to disclose
Giuseppina Novo: Nothing to disclose
Cinzia Nugara: Nothing to disclose
Antonella Meloni: Nothing to disclose
Andrea Barison: Nothing to disclose

### RPS 403-5

**Realtime flow imaging for assessment of aortic and pulmonary blood flow**

*G. Reiter*[1], C. Reiter[1], C. Kräuter[1], V. Nizhnikava[1], D. Giese[2], M. Fuchsjäger[1], U. Reiter[1]; [1]Graz/AT, [2]Erlangen/DE
*(gert.reiter@siemens-healthineers.com)*

**Purpose:** Combining compressed sensing (CS) and shared velocity encoding (SVE) enables magnetic resonance two-dimensional (2D) flow imaging acquisition in realtime. The purpose of the present study was to validate realtime 2D flow imaging for the assessment of aortic and pulmonary flow volumes and their ratio against the conventional segmented technique.
**Methods or Background:** 21 prospectively recruited cardiac patients without known or suspected shunts and with regular heart rhythm underwent breath-hold through-plane 2D flow imaging perpendicular to the aorta and the pulmonary artery at 3T (Magnetom Skyra, Siemens Healthcare). Both measurements were performed once with a conventional retrospectively ECG-gated, segmented 2D flow sequence and once with a prototype realtime flow sequence employing CS and SVE acquired for 5 seconds. Baseline-corrected aortic (Qa) and pulmonary (Qp) net flow volumes were evaluated using routine software (cvi42, Circle Cardiovascular Imaging); realtime flow volumes were derived as averages of 3 heart beats. Relationships between flow volumes were analyzed by correlation analysis; means and variances were compared employing t- and Levene test, respectively.
**Results or Findings:** Flow volumes from realtime measurements (Qa=71±26ml; Qp=72±26ml) showed no significant bias compared to conventional measurements (Qa=73±27ml, p=0.28; Qp=75±30ml, p=0.12) and demonstrated strong correlations (r=0.95 for Qa, r=0.97 for Qp). Pulmonary-to-systemic blood flow ratios derived from realtime measurements (Qp/Qa=1.03±0.12) and from conventional measurements (Qp/Qa=1.03±0.09) did not differ significantly, either in means (p=0.91) or in variances (p=0.44).
**Conclusion:** Realtime 2D flow imaging allows accurate assessment of aortic and pulmonary flow volumes, suggesting the technique as fast alternative for assessment of the pulmonary-to-systemic blood flow ratio in clinical routine patients.
**Limitations:** Small sample size.
**Ethics Committee Approval:** Obtained.
**Funding for this study:** OeNB-Anniversary-Fund No.17934, ESR Seed Grant 2020, ESOR Fellowship 2020.
**Author Disclosures:**
Daniel Giese: Employee: Siemens Healthcare GmbH, Erlangen, Germany
Ursula Reiter: Nothing to disclose
Gert Reiter: Employee: Siemens Healthcare Diagnostics GmbH
Michael Fuchsjäger: Nothing to disclose
Clemens Reiter: Nothing to disclose
Volha Nizhnikava: Nothing to disclose
Corina Kräuter: Nothing to disclose

### RPS 403-6

**Functional and structural abnormalities detected in patients with sickle cell disease using cardiac magnetic resonance imaging**

*L. Tantcheu*[1], V. Chevance[2], V. Tacher[3], I. Sifaoui[4], T. D'Humières[5], P. Bartolucci[5], J-F. Deux[5]; [1]Puteaux/FR, [2]Velizy/FR, [3]Paris/FR, [4]Angers/FR, [5]Créteil/FR
*(taderlan@hotmail.com)*

**Purpose:** Cardiac involvement is a factor of poor prognosis in sickle cell disease (SCD) patients. We aim to evaluate the aspect of cardiac involvement in SCD patients using cardiac magnetic resonance imaging (MRI)
**Methods or Background:** Sixty sickle cell patients and 35 control patients were included in this prospective study and explored with 1.5 T cardiac MRI. Cine, T2 and T1 parametric (MOLLI) and LGE sequences were acquired in the short axis and long axis planes. Left (L) and right (R) ventricular ejection fraction (VEF), ventricular volumes, atrial volumes, 3D components of the strain, extracellular volume (ECV) and presence of focal area of myocardial enhancement on LGE sequences were noticed. Data are given as mean +/- SD.
**Results or Findings:** SCD patients exhibited significant dilatation of both ventricles, reduction of LVEF and RVEF and significant reduction of the 3 components of the strain in comparison to control patients (data not shown). Myocardial T2 of SCD patients was significantly higher than control patients (53 +/-3 ms vs. 49+/-2 ms ; p<0.05) and significantly correlated with left (r2=0.50 ; p<0.001.) and right (r2=0.53 ; p<0.001.) diastolic ventricular volumes. ECV of SCD patients was significantly higher than control patients (38+/-12% vs. 32+/-4% ; p<0.05), and significantly correlated with LFEF (r2=-0.35 ; p=0.03), LV diastolic volume (r2=0.41 ; p=0.06) and LV mass (r2=0.41 ; p =0.005). One SCD patients exhibited area of focal enhancement on LGE sequences (none control).
**Conclusion:** SCD patients exhibited morphologic and functional cardiac abnormalities on cardiac MRI in comparison to control patients. We noticed a raise of ECV in SCD patients that suggest diffuse myocardial fibrosis, and an increase of myocardial T2 suggesting myocardial edema.
**Limitations:** n/a
**Ethics Committee Approval:** n/a
**Funding for this study:** No funding was received for this work
**Author Disclosures:**
Pablo Bartolucci: Nothing to disclose
Vania Tacher: Nothing to disclose
Thomas D'Humières: Nothing to disclose
Islem Sifaoui: Nothing to disclose
Jean-François Deux: Nothing to disclose
Virgile Chevance: Nothing to disclose
Landry Tantcheu: Nothing to disclose

### RPS 403-7

**Normal values of the mitral valve vortex ring derived from MR 4D-flow imaging**

*C. Kräuter*[1], U. Reiter[1], C. Reiter[1], V. Nizhnikava[1], M. Masana[2], A. Schmidt[1], M. Fuchsjäger[1], R. Stollberger[1], G. Reiter[1]; [1]Graz/AT, [2]Barcelona/ES
*(corina.kraeuter@medunigraz.at)*

**Purpose:** The mitral valve (MV) vortex ring is an emerging blood flow structure for studying diastolic function, however, normal values of MV vortex ring parameters are lacking. The aim of this study was to determine normal values of MV vortex ring vorticity and kinetic energy using cardiac magnetic resonance four-dimensional phase-contrast (4D-flow) imaging and to investigate dependencies of these vortex ring parameters on age and gender.
**Methods or Background:** 46 subjects without symptoms of cardiopulmonary diseases (male/female 14/32; age 61±8 years) underwent left heart 4D-flow imaging at 3T. Pre-processing of velocity data was performed by prototype software (4DFlow, Siemens Healthcare), extraction and analysis of MV vortex rings was automatically performed by in-house software. Age dependencies of early- and late-diastolic MV vortex ring peak average vorticity and peak absolute kinetic energy as well as their ratios (curl_E, curl_A, Ekin_E, Ekin_A, curl_E/curl_A, and Ekin_E/Ekin_A, respectively) were analyzed by correlation analysis, differences between genders were analyzed by unpaired t-test or Mann-Whitney test, in case of data non-normality. p<0.05 was considered statistically significant.
**Results or Findings:** Early- and late-diastolic MV vortex rings were detected in each subject. Normal values of the MV vortex ring parameters are curl_E=81/s±12/s, curl_A=77/s±12/s, curl_E/curl_A=1.1±0.2, Ekin_E=1.6±0.6mJ, Ekin_A=1.1±0.5mJ and Ekin_E/Ekin_A=1.7±0.9. While none of the MV vortex ring parameters differed significantly between male and female, all of them correlated significantly with age, with the early-to-late-diastolic parameter ratios showing the strongest correlations (r curl_E=-0.50, r curl_A=0.38, r curl_E/curl_A=-0.65, r Ekin_E=-0.54, r Ekin_A=0.38, r Ekin_E/Ekin_A=-0.62).
**Conclusion:** Whereas MV vortex ring parameters do not depend on gender, their age-dependency has to be taken into account when comparing with diseased subjects.
**Limitations:** Only middle-aged and elderly subjects were included.
**Ethics Committee Approval:** Medical University Graz (24-126ex11/12).
**Funding for this study:** OeNB Anniversary Fund No.17934, ESR Seed Grant 2020, ESOR Fellowship 2020.
**Author Disclosures:**
Ursula Reiter: Nothing to disclose
Marc Masana: Nothing to disclose
Gert Reiter: Employee: Siemens Healthcare Diagnostics GmbH
Michael Fuchsjäger: Nothing to disclose
Clemens Reiter: Nothing to disclose
Albrecht Schmidt: Nothing to disclose
Rudolf Stollberger: Nothing to disclose
Volha Nizhnikava: Nothing to disclose
Corina Kräuter: Nothing to disclose

### RPS 403-8

**Highly accelerated compressed sensing 4D flow is feasible for intra-cardiac flow assessment**

*A. Varga-Szemes*[1], M. C. Halfmann[2], U. J. Schoepf[1], N. Jin[3], D. Dargis[1], F. Xiong[3], M. Markl[3], T. Emrich[1]; [1]Charleston, SC/US, [2]Mainz/DE, [3]Chicago, IL/US
*(vargaasz@musc.edu)*

**Purpose:** Compressed sensing (CS) is a promising acceleration technique that provides substantial reduction in image acquisition time. The aim of this study was to compare intra-cardiac flow measurements between conventional and prototype CS-based highly accelerated 4D flow acquisitions.
**Methods or Background:** Healthy volunteers (n=50) prospectively underwent whole-heart 4D flow 3T MRI with conventional GRAPPA and CS accelerated techniques with acceleration factors of 3 and 7.7, respectively. 4D flow data were post-processed by applying a valve tracking algorithm that allowed for flow quantification over all four valves (aortic [AV], mitral [MV], pulmonary [PV], and tricuspid [TV]). Flow volumes and diastolic function (E/A and E/e') were quantified. The agreement between the techniques was evaluated by Bland-Altman-analysis (BA) and intraclass correlation coefficient (ICC).
**Results or Findings:** A significant improvement in acquisition time was observed using CS compared to GRAPPA accelerated acquisition (6.7±1.3 vs. 12.0±1.3 minutes; p<0.0001). Comparisons of net forward flow measurements for all valves showed good correlation (r>0.81) and agreement (ICCs>0.89 and mean differences <9ml) between conventional and CS 4D flow with 3.3-8.3% underestimation by the CS technique. Conventional vs CS net forward flow measurements were: AV 91.3±16.5ml vs 83.9±14.8ml, BA mean difference -7.3ml, ICC=0.96, r=0.92; MV 94.0±18.4ml vs 89.3±17.2ml, BA=-4.5ml, ICC=0.91, r=0.84; PV 89.0±14.8ml vs 85.7±13.8ml, BA=-3.2ml, ICC=0.95, r=0.90; and TV 100.2±17.4ml vs 91.6±17.1ml, BA=-8.7ml, ICC=0.89, r=0.81. Further evaluation of diastolic function using mitral inflow parameters showed 3.2-17.6% error: E/A 2.2 [1.9-2.4] vs 2.3 [2.0-2.6], BA=0.08, ICC=0.82; and E/e' 4.6 [3.9-5.4] vs 3.8 [3.4-4.3], BA=-0.9, ICC=0.89.
**Conclusion:** Analysis of intra-cardiac flow patterns and evaluation of diastolic function using a highly accelerated 4D flow sequence prototype is feasible and shows underestimation of flow measurements but within clinically acceptable limits of <10%.
**Limitations:** Only healthy volunteers studied
**Ethics Committee Approval:** Approved with informed consent
**Funding for this study:** None
**Author Disclosures:**
Ning Jin: Employee: Siemens
Danielle Dargis: Nothing to disclose
Fei Xiong: Employee: Siemens
Uwe Joseph Schoepf: Consultant: Bayer, Bracco, Elucid Bioimaging, HeartFlow, Guerbet, Siemens Grant Recipient: Bayer, Bracco, Elucid Bioimaging, HeartFlow, Guerbet, Siemens
Tilman Stephan Emrich: Speaker: Siemens
Moritz Christian Halfmann: Nothing to disclose
Michal Markl: Research/Grant Support: Siemens, Circle, Cryolife Consultant: Circle
Akos Varga-Szemes: Consultant: Bayer, Elucid Bioimaging Grant Recipient: Siemens

### RPS 403-9

**Multiparametric approach to detect chronic myocardial inflammation in heart failure with reduced ejection fraction**

*M. C. Halfmann*[1], G. Laux[1], S. Benz[1], S. Schwuchow[1], C. Düber[1], W. Philip[1], K-F. Kreitner[1], T. Emrich[2]; [1]Mainz/DE, [2]Charleston, SC/US

**Purpose:** In heart failure with reduced ejection fraction (HFrEF), presence of chronic inflammation leads to myocardial remodeling and ultimately fibrosis, thus affecting prognosis and outcome. As clinical detection of inflammation remains challenging, endomyocardial biopsy (EMB) with histological and immune-histochemical quantification remains the current gold standard despite its invasive nature. In this study, a multiparametric cardiac magnetic resonance (CMR) approach was evaluated for its potential to detect chronic myocardial inflammation in HFrEF patients with EMB as a ground truth.
**Methods or Background:** 35 HFrEF-patients who underwent both CMR at 3T and EMB were retrospectively identified. Volumetric measurements, feature-tracking (FT)-strains and septal T1/T2 values were acquired. Differentiation between patients with EMB-proven inflammation (I+) and those without (I-) was evaluated by means of receiver operating characteristic curves with their respective areas under the curve (AUC).
**Results or Findings:** Median time between EMB and CMR was 1±3 days. 17 (49%) of the patients were I+. Neither FT-strains nor left ventricular mass (LVM) or T1/T2-mapping alone were significantly different between I+ and I- (p>0.05). A multiparametric approach including global circumferential strain (GCS), LVM and T1/T2-mapping reached an AUC of 0.73 with a sensitivity of 69% and specificity of 91%. This approach outperformed ejection fraction (0.60), enddiastolic volume (0.58) and LVM (0.59) as well as T1 (0.65) and T2 (0.59) mapping.
**Conclusion:** The multiparametric CMR approach was able to predict inflammation with a high specificity but suboptimal sensitivity. Currently, this approach may not replace EMB in detection of chronic myocardial inflammation. Further research is necessary to investigate additional methods, such as radiomics, to improve diagnostic accuracy of CMR in detecting myocardial inflammation.
**Limitations:** Single-center, retrospective study
**Ethics Committee Approval:** This study was approved with a waiver for informed consent (837.196.13/837.477.14).
**Funding for this study:** No outside funding was received for this study.
**Author Disclosures:**
Wenzel Philip: Nothing to disclose
Gerald Laux: nothing to disclose
Sebastian Benz: Nothing to disclose
Sören Schwuchow: Nothing to disclose
Christoph Düber: Nothing to disclose
Tilman Stephan Emrich: Speaker: Siemens Healthineers Other: Travel Support Siemens Healthineers
Moritz Christian Halfmann: Nothing to disclose
Karl-Friedrich Kreitner: Nothing to disclose

### Research Presentation Session: Cardiac

### RPS 503

### Arteriosclerosis

### RPS 503-4

**The effect of smoking status on coronary artery plaques in type 2 diabetic patients: detected by CT angiography**

*Y. Jiang*, Y. ZhiGang; Chengdu/CN
*(jiangyu_nsmc@163.com)*

**Purpose:** We aimed to explore characteristics of coronary artery plaques in type 2 diabetes mellitus (DM) detected by CTA and relationship of plaques in DM between different smoking status.
**Methods or Background:** Plaque types, luminal stenosis, segment involvement score (SIS) and segment stenosis scores (SSS) were evaluated and compared between groups. Multivariable analysis was performed to assess potential effect of smoking on performance of coronary artery plaques in DM patients.
**Results or Findings:** 1063 DM patients were found to have coronary plaques on CTA. Compared to never smoke, smoke group showed hgheri odds ratio [OR] of obstructive coronary artery disease, multi-segment disease, diseased vessel≥3, SIS≥4 and SSS≥7 (OR of 1.569, 1.518, 1.427, 1.670 and 1.513; 95%CI of 1.198-2.055, 1.010-2.283, 1.082-1.883, 1.263-2.209 and 1.149-1.992; respectively), smoking duration (≥40 years) was associated with an increasing OR of obstructive disease, multi-vessel disease, multi-segment disease, diseased vessel≥3, SIS≥4 and SSS≥7 (OR of 1.811, 2.067, 2.992, 1.635, 2.180 and 2.030; 95% CI of 1.195-2.742, 1.124-3.802, 1.449-6.186, 1.070-2.499, 1.468-3.237 and 1.376-2.997; respectively), and quit smoking was associated with a lower OR of SIS≥4 (OR, 1.596; 95%CI, 1.144-2.227) and SSS≥7 (OR, 1.432; 95%CI, 1.032-1.987) than current smoking group.
**Conclusion:** DM patients with smoking history and with longer pack-years are significantly associated with obstructive and more extensive coronary artery plaques, and quit smoking may reduce the risk of the obstructive or relatively more extensive coronary artery disease.
**Limitations:** There was a potential selection bias for the study was single-centered and retrospective. Patients with coronary artery disease but without DM were not enrolled into study.
**Ethics Committee Approval:** Institutional Review Board of West China Hospital, Sichuan University approved this study. Written informed consent was waived.
**Funding for this study:** 1·3·5 project for disciplines of excellence, West China Hospital, Sichuan University (ZYGD18013)
**Author Disclosures:**
Yu Jiang: Nothing to disclose
Yang ZhiGang: Nothing to disclose

### RPS 503-5

**Myocardial native T1 mapping and correlations with clinical and CMR parameters in patients with systemic sclerosis**

A. Meloni[1], L. Gargani[1], C. Bruni[2], M. Gobbo[3], G. D'Angelo[1], N. Martini[1], G. Sinagra[3], M. Matucci-Cerinic[2], *A. Pepe*[1]; [1]Pisa/IT, [2]Florence/IT, [3]Trieste/IT
*(alessia.pepe@ftgm.it)*

**Purpose:** We evaluated the prevalence of cardiac involvement by native T1 mapping and its correlation with clinical and CMR parameters in systemic sclerosis (SSc) patients.
**Methods or Background:** Fifty-one SSc patients (51.8±13.7 years, 42 females) and 51 healthy subjects matched for age/sex were included. The CMR protocol included: cine, T1 mapping by MOLLI, T2 mapping by multi-echo fast-spin-echo sequence, LGE, and STIR T2-weighted sequences. Native T1 and T2 values were assessed in all 16 myocardial segments and the global value was the mean.
**Results or Findings:** Global native T1 values were significantly higher in SSc patients than in healthy subjects. In patients native T1 values were significantly lower in males than in females and inversely correlated with age. Twenty-three(45.1%) patients had an increased global heart T1 value (males:>1060; females:>1085 ms). Of them, 14(60.9 %) patients showed positive LGE. Frequency of cardiovascular risk factors, indices of disease activity/chronicity, biochemical parameters, and cardio-active therapy were comparable between patients with normal and elevated T1. Patients with elevated T1 had significantly higher left ventricular (LV) end-diastolic volume index, stroke volume index, cardiac output, and global heart T2 values. Replacement myocardial fibrosis was detected in 24(47.1%) patients and they showed significantly higher global heart native T1 values. Positive T2-weighted images for myocardial oedema were found in 5(9.8%) patients, all with increased global heart native T1 value. Patients with oedema had significantly higher native global heart T1 values.
**Conclusion:** Elevated native T1 values are frequent in SSc patients and are associated with inflammation, replacement fibrosis, and increased LV dimension. CMR T1 mapping seems to be a sensitive parameter to include in the routine clinical assessment of SSc patients for detecting earlier pejorative cardiac involvement.
**Limitations:** Single-center study
**Ethics Committee Approval:** Local ethics commitee
**Funding for this study:** No funding received
**Author Disclosures:**
Alessia Pepe: Nothing to disclose
Gianfranco Sinagra: Nothing to disclose
Nicola Martini: Nothing to disclose
Luna Gargani: Nothing to disclose
Antonella Meloni: Nothing to disclose
Gennaro D'Angelo: Nothing to disclose
Cosimo Bruni: Nothing to disclose
Marco Gobbo: Nothing to disclose
Marco Matucci-Cerinic: Nothing to disclose

### RPS 503-6

**Prognostic utility of coronary CT angiography-derived plaque information on long-term outcome in patients with and without diabetes mellitus**

*V. Brandt*[1], C. Tesche[2], M. Bauer[1], M. Baquet[3], F. Straube[3], B. Brück[3], U. J. Schoepf[1], E. Hoffmann[3], U. Ebersberger[3]; [1]Charleston, SC/US, [2]Dortmund/DE, [3]Munich/DE
*(verena.brandt@gmx.net)*

**Purpose:** To investigate the long-term prognostic value of coronary CT angiography (cCTA)-derived plaque information on major adverse cardiac events (MACE) in patients with and without diabetes mellitus.
**Methods or Background:** 64 patients with diabetes (63.3±10.1 years, 66% male) and suspected coronary artery disease (CAD) who underwent cCTA were matched with 297 patients without diabetes according to age, sex, cardiovascular risk factors, statin and antithrombotic therapy. Major adverse cardiac events (MACE) were recorded. cCTA-derived risk scores and plaque measures were assessed. The discriminatory power to identify MACE were evaluated using multivariable regression analysis and the area under the receiver-operating characteristics curve (AUC).
**Results or Findings:** After a median follow-up of 5.4 years, MACE occurred in 31 patients (8.6%). In patients with diabetes, cCTA risk scores and plaque measures were significantly higher compared to non-diabetic patients (all p<0.05). The following plaque measures were predictors of MACE in diabetic patients (odds ratio [OR]): segment stenosis score (OR 1.31, p=0.002), low-attenuation plaque (OR 5.89, p=0.023), and >=2 high-risk features (OR 6.32, p=0.017), and in non-diabetic patients: segment stenosis score (OR 2.08, p=0.004), and >=2 high-risk features (OR 5.14, p=0.024). Discriminatory power of CT scores and plaque measures in diabetic patients were significantly better than that of obstructive CAD and Framingham risk score (all p<0.05).
**Conclusion:** Diabetes is associated with a significantly higher degree of high-risk plaque features, which have independent predictive value for MACE. cCTA-derived plaque information portends improved risk stratification in diabetic patients beyond cCTA stenosis grading alone.
**Limitations:** Single center study.
Small number of patients were included, which may incur selection bias.
**Ethics Committee Approval:** Approved by the institutional review board and the need for written informed consent was waived due to the retrospective nature of this investigation.
**Funding for this study:** No funding.
**Author Disclosures:**
Verena Brandt: Nothing to disclose
Ellen Hoffmann: Nothing to disclose
Christian Tesche: Research/Grant Support: Tesche has received speaker's fees from Siemens Healthineers
Ullrich Ebersberger: Nothing to disclose
Benedikt Brück: Nothing to disclose
Uwe Joseph Schoepf: Research/Grant Support: Schoepf receives institutional research support from Astellas, Bayer, GE, and Siemens.
Florian Straube: Nothing to disclose
Maximilian Bauer: Nothing to disclose
Moritz Baquet: Nothing to disclose

### RPS 503-7

**320-row MDCT: correlation of abdominal and epicardial adipose tissue with CA as a predictor of acute coronary events**

A. Ikramov, *N. Djuraeva*, R. Ibadov, Z. Khaybullina, M. Maksudov, K. Abdukhalimova, A. Sultanov; Tashkent/UZ
*(xanummuzik@yandex.ru)*

**Purpose:** Determination of the correlation between the ratio of abdominal and epicardial fat to the amount of calcium in the coronary arteries (Ca-score) as a predictor of acute coronary events (ACE).
**Methods or Background:** 260 patients(90 women and 170 men) at the age of 55±12 years with a history of ACE inhibitors were examined. All patients underwent scanning of the abdominal cavity at the level of the navel and at the level of the ventricles of the heart along the short axis using 320-rowMDCT with determination of and Ca-score and the following indicators of abdominal and epicardial adipose tissue: TFA(total fat area), VFA(visceral fat area), EAT (epicardial accessory tissue), VFA/TFA, EAT/TFA.
**Results or Findings:** The studies were based on the ratio of VFA / TFA of the abdominal region and EAT / TFA of the epicardial region to the Agatson calcium index. Ca-score 0-50 was in 30% of all patients, 100-450-31%,> 450-39% (p <0.05). As the Ca-score increased, there was an increase in the VFA / TFA and EAT / TFA ratios in linear correlation (p<0.05). In the abdominal cavity, it was found that with a Ca-score of 0-50 VFA/TFA is 0.25±0.2 (p<0.05), with 100-450 - 0.48 ± 0.25 (p<0, 05), for> 450-0.53±0.3 (p<0.05). When studying the ratio of EAT/TFA of the epicardial region, it was determined that with a Ca-score of 0-50 EFA/TFA is 0.1±0.025(p<0.05), with 100-450-0.13±0.03(p<0.05), for>450-0.22±0.2(p<0.05). These indicators are typical for 80-85% of patients. In the rest of the patients, this dependence was not determined.
**Conclusion:** The ratio of VFA/TFA and EAT/TFA of the abdominal and epicardial regions respectively correlates with the degree of calcification of the coronary arteries(p<0.05) and can be a reliable marker in predicting the severity of acute coronary events.
**Limitations:** within the Institution rules
**Ethics Committee Approval:** Approved by Institution
**Funding for this study:** Institution
**Author Disclosures:**
Adham Ikramov: Nothing to disclose
Ravshan Ibadov: Nothing to disclose
Khanum Abdukhalimova: Nothing to disclose
Alisher Sultanov: Nothing to disclose
Muzaffar Maksudov: Nothing to disclose
Nigora Djuraeva: Nothing to disclose
Zarina Khaybullina: Nothing to disclose

### RPS 503-8

**MSCT structural analysis of atherosclerotic plaque and visceral fat area/total fat area in patients with functionally significant and insignificant stenosis of coronary arteries**

A. Ikramov, *N. Djuraeva*, Z. Khaybullina, M. Maksudov, K. Abdukhalimova, A. Sultanov; Tashkent/UZ
*(nika.kt@rambler.ru)*

**Purpose:** To study the differences in the composition and percentage of atherosclerotic plaques(ASP)in individuals with functionally insignificant(FIS) and functionally significant stenosis(FSS)of coronary arteries(CA)according to volume CT coronarography (CTCA)with the determination of the correlation with the VFA/TFA(Visceral fat area/total fat area).
**Methods or Background:** Volume CTCA were performed on 320-row MDCT to 144 patients. The degree of stenosis was determined using multisegment analysis using the CAD-RADs system. The amount and percentage of components of ASP: fatty, connective tissue and calcined were determined. The indicators of plaque burden(PB)and the structure of the ASB were studied: the volume(Vmm³)and the proportion(V%)of the components in the composition of the ASP, as well as the VF/TFA.
**Results or Findings:** It was revealed that PB with FIS was 53.4±8.2, and with FSS was 62.6±5.3.Volume(mm³)of the fat component: with FIS56.86±8.3, with FSS72.66±5.5(p<0.05). The volume of the connective tissue component in cases FIS is 95.6±9.5, and for FSS is 125.3±25 (p<0.05). The volume of the calcined component in patients with FIS is 51.7±1.4, and in patients with FSS 52.6±1.5 (p<0.05). The % of the fat component in case of FIS is 30.4±3.3 and for FSS is 29±5.5(p<0.05). The % of the connective tissue component in patients with FIS is 49.7±2.3, and for FSS is 50.5±2.5(p<0.05). The % of the calcined component in cases of FIS is 20.6±3.5, and in cases of FSS is 20.5±5.2. The ratio of VFA/TFA with FIS stenosis 0.3±0.025, with FSS stenosis 0.52±0.065.
**Conclusion:** The plaque burden and the volume ratio(mm³)of the adipose tissue and connective tissue of ASP are significantly higher in FSS CA(CAD-RADs<3)and by determining the correlation with the ratio of the fat depot in the abdominal region of VFA/TFA(p<0.05). Whereas the volume ratio (mm3)Ca and the percentage of ASP components are equal(p<0.05)in the different groups, which is confirmed by the data of PB,V (mm³),(%) and VF/TFA.
**Limitations:** within the Institution rules
**Ethics Committee Approval:** Approved by Institution
**Funding for this study:** Institution
**Author Disclosures:**
Adham Ikramov: Nothing to disclose
Khanum Abdukhalimova: Nothing to disclose
Alisher Sultanov: Nothing to disclose
Muzaffar Maksudov: Nothing to disclose
Nigora Djuraeva: Nothing to disclose
Zarina Khaybullina: Nothing to disclose

### Research Presentation Session: Cardiac

### RPS 603

### Artificial intelligence and machine learning in cardiac imaging

### RPS 603-1

**Development and validation of a fully convolutional neural network for automated left ventricular myocardium segmentation on cardiac ECG 4DCT enhanced images**

*G. Gong*[1], T. Song[2], H. Yin[2], Y. Guo[1]; [1]Jinan/CN, [2]Beijing/CN
*(gongguanzhong@163.com)*

**Purpose:** To develop and evaluate the performance of a convolutional neural network for automatic segmentation of the left ventricular (LV) myocardium on ECG 4DCT enhanced scan.
**Methods or Background:** A total of 236 ECG 4DCT enhanced scans from 11 patients were retrospectively collected. The LV myocardium areas were manually segmented as standard reference. The labeled images were further divided into a training dataset (25444 images from 178 CT scans) and a validation dataset (6980 images from 58 CT scans). A modified U-Net model using Xception network as backbone architecture was proposed for our purpose. The model performance was quantified by the Dice similarity coefficient (DSC). Correlation coefficient and Bland-Altman analysis were used for agreement evaluation between automated and reference values on per-slice and per-segment analyses.
**Results or Findings:** The predicted LV volume (71.3 ± 12.8 cm3) showed good consistency with manual segmentation result (69.1 ± 14.4 cm3), achieving average DSCs of 0.852 ± 0.152 and 0.869 ± 0.060 for the LV myocardium per-slice and per-segment, respectively. The automated predicted measurements strongly correlated with reference values in per-slice (R = 0.936, 95% CI: 0.933~0.939) and per-segment (R = 0.987, 95% CI: 0.978~0.992) analyses. Bland-Altman analysis showed good agreement between the automatic and reference values in per-slice (-3.2% ± 7.6) and per-segment (-1.5% ± 7.9) analyses.
**Conclusion:** We have developed a deep learning model for automated LV myocardium segmentation with good performance. This method has the potential to replace time-consuming manual segmentations in large-scale studies.
**Limitations:** This model needs to be validated in prospective studies with a larger sample size from multicenter to acquire high-level evidence for the clinical application usefulness.
**Ethics Committee Approval:** This study was approved by IRB, with a waiver of the written informed consent requirement.
**Funding for this study:** Not applicable.
**Author Disclosures:**
Yujie Guo: Nothing to disclose
Hongkun Yin: Nothing to disclose
Guanzhong Gong: Nothing to disclose
Tian Song: nothing to disclose

### RPS 603-2

**Evaluation of machine-learning algorithms to predict postoperative outcomes in asymptomatic patients with moderate-severe primary mitral regurgitation**

*E. Andrikopoulou*[1], Y-H. Fang[1], T. Denney[2], B. Pat[1], M. Ahmed[1], L. Dell'Italia[1]; [1]Birmingham, AL/US, [2]Auburn, AL/US
*(eandrikopoulou@uabmc.edu)*

**Purpose:** Despite adherence to guidelines, 20% of patients with primary mitral regurgitation (PMR) will experience postoperative left ventricular (LV) systolic dysfunction [LV ejection fraction less (LVEF) than 50%]. We hypothesize that a machine learning (ML) model will allow for its prediction.
**Methods or Background:** Clinical, demographic and imaging (cardiac magnetic resonance, CMR) data from 46 symptomatic PMR patients undergoing mitral valve surgery and 43 asymptomatic PMR patients without intervention were included. The symptomatic subgroup underwent a follow-up

CMR 6 months postoperatively. Preoperative CMR data were used as input to a two-level decision tree model. Resubstitution and leave-one-out were used to validate the model. Following an exhaustive search, the two optimal predictors for postoperative LVEF <50% were determined.
**Results or Findings:** 14.7% of men and 41.6% of women experienced a reduction in their postoperative LVEF. Given our sample size, the binary classification tree method was used as the predictive model. We found that the best decision tree used a LVEF threshold of 58% and LV sphericity index threshold of 1.25. The resubstitution evaluation showed a 72.7% sensitivity, 97% specificity and 91% accuracy; the leave-one-out validation yielded a 54.6% sensitivity, 94% specificity and 85% accuracy. When applying this tree to the asymptomatic cohort, 12 out of the 43 subjects (28%) were predicted with postoperative drop in LVEF.
**Conclusion:** The fraction (28%) of predicted poor outcome aligns with previous reports in postoperative PMR and implies that some asymptomatic patients would benefit from early intervention. A ML-based decision model could help guide management in moderate-severe asymptomatic PMR.
**Limitations:** Lack of data to validate whether the prediction in the asymptomatic cohort is accurate.
**Ethics Committee Approval:** Not required
**Funding for this study:** NIH grants R01HL60707 and R01HL54816 to LJD, and Specialized Center for Clinically Oriented Research in Cardiac Dysfunction P50HL077100.
**Author Disclosures:**
Mustafa Ahmed: Nothing to disclose
Thomas Denney: Nothing to disclose
Betty Pat: Nothing to disclose
Efstathia Andrikopoulou: Nothing to disclose
Louis Dell'Italia: Nothing to disclose
Yu-Hua Fang: Nothing to disclose

### RPS 603-3

**Evaluation of the effect of type II diabetes mellitus on left ventricular function in patients with hypertension by 3.0 T magnetic resonance imaging**

*W-F. Yan*, Z-G. Yang, Y-K. Guo, X-M. Li, J. Li, J. Wang, K. Shi, Y. Gao; Chengdu/CN
*(315847112@qq.com)*

**Purpose:** The purpose of this study was to explore the effect of type II diabetes mellitus (T2DM) on the systolic and diastolic function of left ventricular (LV) in patients with essential hypertension (HTN) by the strain and volume-time curve parameters from cardiac magnetic resonance.
**Methods or Background:** One hundred and seventeen essential hypertension patients (46 with T2DM and 71 without T2DM) and forty-five normal individuals were enrolled in this study. Volume-time curve parameters, including the peak ejection rate (PER), peak ejection time (PET), peak filling rate (PFR), peak filling time from end systolic (PFT), PER normalized to EDV (PER/EDV), PFR normalized to EDV (PFR/EDV), and left ventricular stain parameters, including left ventricular radial strain (GRPS), circumferential strain (GCPS) , longitudinal peak strain (GLPS) were measured and compared. Multivariate linear regression analyses were performed to determine the effects of T2DM on LV diastolic dysfunction in patients with hypertension.
**Results or Findings:** GLP、PFR、PFR/EDV deteriorated significantly from controls, through HTN(T2DM−), to H□N(□2DM+) □roup; G□PS i□H□N(□2DM+) group was lower than those in both HTN(T2DM–) and control groups. PFT in HTN (T2DM-)group and HTN (T2DM+) group were longer than those in control group. Multiple regression analyses considering covariates of systolic blood pressure, age, sex and BMI demonstrated that T2DM was independently associated with PFR/EDV ( $\beta = 0.177$, $p = 0.01$).
**Conclusion:** Type 2 diabetes exacerbates left ventricular systolic and diastolic dysfunction in hypertensive patients. The impairment of cardiac function is more obviously reflected in the change of left ventricular volume during diastole.
**Limitations:** The present study was a single-center, retrospective study.
**Ethics Committee Approval:** This study was approved by the Clinical Trials and Biomedical Ethics Committee of West China Hospital of Sichuan University.
**Funding for this study:** The 1·3·5 project for disciplines of excellence, West China Hospital, Sichuan University (ZYGD18013).
**Author Disclosures:**
Jin Wang: Nothing to disclose
Ying-Kun Guo: nothing to disclose
Jiang Li: Nothing to disclose
Ke Shi: Nothing to disclose
Yue Gao: Nothing to disclose
Wei-Feng Yan: Nothing to disclose
Xue-Ming Li: Nothing to disclose
Zhi-Gang Yang: Nothing to disclose

### RPS 603-4

**Machine learning can predict pulmonary hypertension in patients undergoing contrast-enhanced CT thorax**

*P. F. P. Charters*, W. Brown, J. Rossdale, O. Slegg, J. Willis, G. Robinson, R. MacKenzie Ross, J. Suntharalingam, J. C. L. Rodrigues; Bath/UK
*(pia.charters@nhs.net)*

**Purpose:** CT pulmonary angiography (CTPA) is often used in suspected pulmonary hypertension (PH). Right ventricular dilatation is a feature of PH. The study purpose was to determine the diagnostic accuracy of a fully-automated machine learning (ML) derived tool at detecting PH in patients with suspected PH referred to a specialist centre relative to invasive right heart catheterisation (RHC) as gold standard and to compare to contemporaneous trans-thoracic echo (TTE).
**Methods or Background:** Retrospective analysis of 162 consecutive patients over 25 months with suspected PH who underwent CTPA and TTE within 12 months of RHC. TTE were graded ‘low’, ‘intermediate’ or ‘high’ likelihood of PH (British Society of Echocardiography). CTPAs were uploaded to fully-automated ML-derived image analysis software for ventricular segmentation and calculation of the RV/LV diameter ratio. RV/LV measurements were compared with RHC pressure measurements. PH diagnosis was made using RHC gold standard.
**Results or Findings:** RV/LV ≥1 was 86% sensitive and 40% specific for PH when scanned within 12 months of RHC, whilst ‘high’ likelihood TTE was 61% sensitive and 86% specific. In a subgroup analysis of 34 ‘intermediate’ likelihood TTEs, 28 had PH at RHC and RV/LV >1 was present in 82% (23) of these. Area under the receiver-operating-curve for RV/LV diameter for PH diagnosis was 0.723 (95th CI 0.609-0.836), p = 0.001. RV/LV diameter >0.95 achieved 90% sensitivity and 30% specificity and RV/LV diameter >1.33 achieved 90% specificity and 39% sensitivity for diagnosis of PH.
**Conclusion:** In patients with suspected PH referred to a specialist centre, automated RV/LV thresholds may help rule in and rule out PH and may have diagnostic utility when TTE is ‘intermediate’ for PH.
**Limitations:** ‘Low’ likelihood TTEs did not all have RHC.
**Ethics Committee Approval:** Approved by trust audit committee.
**Funding for this study:** Nil
**Author Disclosures:**
James Willis: Nothing to disclose
Graham Robinson: Other: The Royal United Hospital is a Centre of Excellence for Imbio and is not remunerated for use.
Oliver Slegg: Nothing to disclose
Pia Charters: Other: The Royal United Hospital is a Centre of Excellence for Imbio and is not remunerated for use.
Jennifer Rossdale: Nothing to disclose
Rob MacKenzie Ross: Nothing to disclose
William Brown: Other: The Royal United Hospital is a Centre of Excellence for Imbio and is not remunerated for use.
Jay Suntharalingam: Nothing to disclose
Jonathan Rodrigues: Other: The Royal United Hospital is a Centre of Excellence for Imbio and is not remunerated for use.

### Research Presentation Session: Cardiac

### RPS 703

### Myocardial deformations

### RPS 703-1

**Left ventricular strain can predict outcomes of pulmonary valve replacement in patients with repaired tetralogy of Fallot**

*B. Zhuang*, M. Lu; Beijing/CN
*(zbyan10@163.com)*

**Purpose:** Pulmonary valve replacement (PVR) is effective in reducing the right ventricular volume and retaining right ventricular function in patients with repair tetralogy of Fallot (rTOF). However, prognostic outcomes after PVR surgery are still mixed. Key factors that determine outcomes after PVR in rTOF patients are largely unknown. Our study aims to analyze whether pre-PVR strain and strain rate are associated with long-term outcomes after PVR in patients with rTOF.
**Methods or Background:** This prospective study enrolled 41 asymptomatic rTOF patients who required PVR due to moderate or severe pulmonary regurgitation. The feature tracking parameters were measured pre-operatively based on cine sequences. Adverse events including death due to any cause, cardiac transplantation, syncope caused by arrhythmias, redo PVR, symptomatic heart failure and persistence ventricular tachycardia were documented during follow-up. ROC analysis was performed and Kaplan-Meier curves were drawn with log-rank statistics.
**Results or Findings:** During 3.0±1.5 years of follow-up, 10 adverse events were recorded. The 3-year event-free survival rate was 63% for patients with a pre-operative radial strain (RS)>23.87% (p<0.001), 93% for patients with a pre-operative circumferential strain (CS)<-15.55% (p<0.001), 63% for patients with a pre-operative longitudinal strain(LS)<-9.7% (p<0.001). As for strain rate, the 3-year event-free survival rate was 68% for patients with a pre-operative systolic radial strain rate (RSRs) >1.53s-1 (p=0.003), 68% for patients with a pre-operative early diastolic radial strain rate (RSRe) <-1.73 s-1 (p<0.001) and 59% for patients with a pre-operative early diastolic longitudinal strain rate (LSRe) >0.42 s-1 (p<0.001).
**Conclusion:** The RS, CS, LS, RSRs, RSRe, LSRe before PVR are important prognostic factors for adverse events. Identifying these factors can help recognize patients at increased risk of adverse events.
**Limitations:** There are some limitations
**Ethics Committee Approval:** This study is approved by Fuwai Hospital
**Funding for this study:** There is no funding
**Author Disclosures:**
Minjie Lu: Nothing to disclose
Baiyan Zhuang: Nothing to disclose

### RPS 703-2

**Right left ventricular blood pool T2 ratio as an innovative screening tool for the identification of left to right shunts in patients with dilated right ventricle: results from a bi-centre study**

*V. Brandt*[1], V. Bordonaro[2], T. Emrich[1], A. Varga-Szemes[1], M. C. Halfmann[3], T. Schoeler[3], U. J. Schoepf[1], A. Secinaro[2]; [1]Charleston, SC/US, [2]Rome/IT, [3]Mainz/DE
*(verena.brandt@gmx.net)*

**Purpose:** Left-to-right shunts (L-R-Shunt) lead to volume overload of the right ventricle(RV), resulting in adverse cardiac events. CMR is able to detect L-R-Shunts by comparison of phase-contrast measurements of pulmonary and aortic flow, which is not routinely performed in clinical examinations. Since T2 is sensitive to blood-oxygenation, we hypothesized that T2 mapping is able to detect blood-oxygenation changes between the ventricles caused by L-R-Shunts. The aim of this study was to evaluate T2 mapping for L-R-Shunt detection in patients with dilated RV compared to healthy volunteers.
**Methods or Background:** We analyzed the T2-ratio of the RV/LV blood-pool and Qp/Qs-ratio by phase-contrast-imaging from 21 patients with L-R-shunts. Healthy volunteers (HV,n=51) and patients with dilated right ventricles without L-R-Shunts (e.g.ARVC,n=21) were used as control groups. Qp/Qs-ratio was calculated by dedicated measurements of pulmonary and aortic flow. T2 ratio of the LV/RV blood-pool was calculated from mid-ventricular T2-Maps.
**Results or Findings:** The Qp/Qs-ratios in the L-R-Shunt vs the control groups were 1.9±0.9 and 1.0±0.0, respectively (p<0.001). End-diastolic RV volume was significant higher compared to HV (122.5±42.0ml and 94.4±16.2 ml,p=0.004), but not in comparison to patients with RV dilatation (122.5±42.0ml and 127.8±34.6ml,p=0.247). T2 RV/LV-Ratio was significantly increased in patients with L-R-Shunts (0.90±0.08) compared to HV(0.74±0.10,p<0.001) and patients with dilated RV (0.73±0.12,p<0.001). A ratio over 0.76 showed 100% sensitivity and 56% specificity for detection of L-R-Shunts, resulting in a negative predictive value of 100%. T2 RV/LV-ratio was strongly correlated with the shunt-severity(r=0.863,p<0.001).
**Conclusion:** T2 RV/LV ratio has the potential to become a fast and reliable parameter to detect or rule-out L-R-Shunts with strong correlation to shunt-severity, independent of RV volume. Such diagnostic tool enables to avoid unnecessary phase-contrast measurements in cases with RV dilatation of unknown etiology.
**Limitations:** Bicenter, retrospective
**Ethics Committee Approval:** Approval-number: (837.196.13/837.477.14)
**Funding for this study:** Not available
**Author Disclosures:**
Verena Brandt: Nothing to disclose
Aurelio Secinaro: Nothing to disclose
Theresia Schoeler: Nothing to disclose
Uwe Joseph Schoepf: Consultant: Bayer, Guerbet, Elucid, Siemens Healthcare, Bracco, HeartFlow
Veronica Bordonaro: Nothing to disclose
Tilman Stephan Emrich: Speaker: Siemens Healthineers
Moritz Christian Halfmann: Nothing to disclose
Akos Varga-Szemes: Consultant: Bayer Healthcare Research/Grant Support: Siemens Healthineers Consultant: Elucid Bioimaging

### RPS 703-3

**Impact of short-term mapping parameter modification on left ventricle remodelling: Miami study**

*A. Palmisano*[1], D. Vignale[1], R. Faletti[2], M. Gatti[2], G. Peretto[1], N. Galea[3], M. Francone[3], F. De Cobelli[1], A. Esposito[1]; [1]Milan/IT, [2]Turin/IT, [3]Rome/IT
*(palmisano.anna@hsr.it)*

**Purpose:** Myocarditis is a multifaceted disease with variable outcome, ranging from complete recovery to end-stage dilated cardiomyopathy. Imaging prognosticators are widely unknown. Aim was to evaluate the value of microstructural alteration using mapping parameters in the prediction of unfavorable remodeling.
**Methods or Background:** Eighty-eight patients with diagnosis of acute myocarditis at multimodality approach (lab test, ECG, echocardiography and CMR) underwent 1.5 T CMR at baseline, 2-months after symptom onset and after 12-months. CMR protocol included 2009 and 2018 Lake Louise criteria. Forty-five healthy volunteers were used as control group.
**Results or Findings:** Clinical presentation was infart-like in 69 patients, heart failure in 14 and arrhythmic in 5 patients. T2 mapping showed best diagnostic performances at baseline (0.97 vs 0.82 and 0.85 for native T1 and ECV), 2-months CMR (0.91 vs 0.66 and 0.78 for native T1 and ECV) and 12-months CMR (0.86 vs 0.48 and 0.69 for native T1 and ECV). Patients with infart-like presentation had more intense but more focal damage, with higher LGE burden (8% vs 3% and 2%, p=0.03) but lower number of myocardial segment with mapping alteration (p=0.02 for T1; p=0.03 for T2, n.s for ECV). Moreover, most of them (55/69) had a complete healing at 2-months CMR. Patients with depressed ejection fraction (EF<55%) at 12-months had more than 10 myocardial segments with altered native T1, T2 and ECV values at baseline and short-term CMR, and higher ECV at 12-months CMR (32% vs 27%).
**Conclusion:** Larger myocardial damage by mapping techniques is associated to higher risk of long-term myocardial dysfunction.
**Limitations:** Endomyocardial biopsy was not performed in all patients.
**Ethics Committee Approval:** The study is approved by the Institutional review board.
**Funding for this study:** The study was granted by Young Research Award of the Italian Ministry of Health (GR-2013).
**Author Disclosures:**
Davide Vignale: Nothing to disclose
Giovanni Peretto: Nothing to disclose
Antonio Esposito: Nothing to disclose
Francesco De Cobelli: Nothing to disclose
Riccardo Faletti: Nothing to disclose
Anna Palmisano: Nothing to disclose
Marco Gatti: Nothing to disclose
Marco Francone: Nothing to disclose
Nicola Galea: Nothing to disclose

### RPS 703-4

**Myocardial remodelling in the first year after cardiac transplant**

*C. Obst*, M-E. Stelzmüller, A. Zuckermann, C. Loewe, D. Beitzke; Vienna/AT

**Purpose:** Heart transplantation (HTX) is a lifesaving procedure for patients with end stage-heart failure. Cardiac magnetic resonance imaging (CMR) is emerging as a promising imaging method to follow up myocardial remodelling after HTX. The aim of this study is to investigate short term cardiac remodelling using multiparametric CMR in the first year after HTX.
**Methods or Background:** Thirty-one patients who underwent CMR on a 1.5 Tesla system 3 and 12 months after HTX were included. Quantitative markers and left ventricular ejection fraction (LVEF) were derived from short axis steady-state free precision (SSFP) sequences. Native T1 and T2 mapping was performed at three short axis slices.
**Results or Findings:** There was no statistically significant difference in T1 (p=0.931), T2 (p=0.386) and the left ventricular mass (p= 0.203) three and twelve months after HTX. However both the LVEF (60.1% vs. 67.2%) and RVEF (51.7% vs. 59.3%) improved significantly (p=0.007 and 0.006) twelve months after transplantation. Endsystolic volume in both the left (51.1 vs. 34.5 ml) and right (63.3 vs. 45.8 ml) ventricles decreased statistically significant (p<0.001 and p=0.048). A reduction of the enddiastolic volume of the left ventricle (126.0 vs 103.6 ml) and the right ventricle (109.9 vs. 92.1 ml), with a p-value of 0.002 vs 0.003 respectively, was observed.
**Conclusion:** In the first year after HTX significant improvements in cardiac function and a reduction in LV and RV size can be observed by CMR. However this is not accompanied by a change in myocardial water content assed by T2 mapping or a relevant change in T1 mapping values potentially representing structural remodelling.
**Limitations:** Eventually low number of cases.
**Ethics Committee Approval:** Study was approved by the local ethics committee of the Medical University of Vienna.
**Funding for this study:** There was no special funding.

**Author Disclosures:**
Christian Obst: Nothing to disclose
Marie-Elisabeth Stelzmüller: Nothing to disclose
Dietrich Beitzke: Nothing to disclose
Andreas Zuckermann: Nothing to disclose
Christian Loewe: Nothing to disclose

### RPS 703-5

**Left atrial functional analysis is superior to standard volumetric assessment in hypertensive heart disease patients with myocardial hypertrophy**

T. Emrich[1], S. Altmann[2], B. Yacoub[1], M. C. Halfmann[2], *C. Reichardt*[2], U. J. Schoepf[1], C. Düber[2], K-F. Kreitner[2], A. Varga-Szemes[1]; [1]Charleston, SC/US, [2]Mainz/DE
*(constantin.reichardt@gmx.de)*

**Purpose:** Chronic hypertension-related LV myocardial adaptation results in myocardial fibrosis, leading to diastolic/systolic dysfunction. Besides standard volumetric parameters, time-volume and strain analysis of the atria were used. The purpose of this study was to compare diagnostic performance of volumetric versus advanced functional atrial parameters to differentiate healthy volunteers (HV) from patients with hypertensive heart disease (HHD) and correlate these parameters to the severity of LV hypertrophy.
**Methods or Background:** 42 HHD patients and 50 HV who had previously undergone CMR (3T) were included in this retrospective study. Atrial time-volume analyses based on long-axis cine acquisitions and atrial strain values were acquired using a commercially available post-processing software. Reservoir (TEF and reservoir strain), conduit (PEF and conduit strain) and booster (AEF and booster strain) function of the LA were calculated.
**Results or Findings:** HHD patients had significantly higher LV myocardial mass (78.1±17.7 g/m2 vs 54.6±9.8 g/m2,p<0.001)and lower EF (53.2±12.1% vs 62.3±5.3%,p<0.001)than HV.Maximal/Minimal atrial volumes, as well as AEF showed no significant differences between HHD and HV (p>0.05). The evaluation of reservoir and conduit function demonstrated significant differences in time-volume and strain parameters between the groups (all p<0.001). Diagnostic performance to differentiate HHD from HV was comparably high for all reservoir and conduit functional parameters of the LA (AUC:0.931-0.966;DeLong p>0.05).PEF and conduit strain demonstrated a significant negative correlation with myocardial mass (r=-0.562 and r=-0.674).
**Conclusion:** Functional parameters of atrial function by time-volume analysis and atrial strain imaging are superior to standard volumetric assessment of the LA to discriminate HHD patients from HV. Parameters from time-volume and strain analysis can be used interchangeably. The correlation between LA function and LV myocardial mass can be used as a marker of diastolic dysfunction and is a potential parameter for treatment monitoring.
**Limitations:** Single-center,retrospective study,lack of invasive/histological validation
**Ethics Committee Approval:** Approval-Number: 837.196.13/837.477.14
**Funding for this study:** None
**Author Disclosures:**
Uwe Joseph Schoepf: Other: Elucid Bioimaging Other: Bayer Healthcare Other: HeartFlow Other: Guerbet Other: Siemens Healthcare (Travel support) Other: Bracco
Christoph Düber: Nothing to disclose
Constantin Reichardt: Nothing to disclose
Tilman Stephan Emrich: Speaker: Siemens Healthcare (Travel support)
Moritz Christian Halfmann: Nothing to disclose
Basel Yacoub: Nothing to disclose
Karl-Friedrich Kreitner: Nothing to disclose
Akos Varga-Szemes: Other: Siemens Healthcare (Travel support) Consultant: Elucid Bioimaging Consultant: Bayer Healthcare
Sebastian Altmann: Nothing to disclose

### RPS 703-6

**Comparison of Cardiac Magnetic Resonance Imaging Findings between Immune Checkpoint Inhibitor-Induced Myocarditis and Presumed Viral Myocarditis**

*F. Cadour*, A. Varoquaux, P. Habert, A. Jacquier, J. Cautela, F. Thuny, U. Scemama; Marseille/FR
*(farah.cadour@hotmail.fr)*

**Purpose:** Immune checkpoint inhibitor-induced myocarditis (IIM) is one of the most serious cancer therapy-related toxicities and represents a new diagnostic challenge. Imaging findings of this rare condition have never been compared to those of presumed viral myocarditis (PVM). This study sought to compare cardiac magnetic resonance imaging (CMR) findings between IIM and PVM.
**Methods or Background:** This retrospective case-control study analyzed 46 patients with acute myocarditis (28 patients with PVM and 18 patients with IIM) confirmed by CMR according to the updated Lake Louise criteria. Covariates of interest were extracted from medical records, and CMR evaluation included late gadolinium enhancement (LGE), T1 and T2 mapping, and extracellular volume (ECV) values.
**Results or Findings:** The subepicardial region was the most common site of LGE localization in both groups. However, the LGE distribution was significantly different between the two groups (p=0.005). In comparison to PVM patients, IIM patients more frequently had midwall LGE (3% vs. 39%, p=0.003) and LGE at the basal anteroseptal (3% vs. 44%, p=0.001), basal inferoseptal (14% vs. 44%, p=0.04), and midinferoseptal (14% vs. 27%, p=0.02) segments. The global, maximum, and elevation values of T2, native T1, and ECV were not significantly different between PVM and IIM patients.
**Conclusion:** PVM and IIM have different LGE patterns on CMR imaging. Septal and midwall myocardial involvement was more frequently observed in patients with IIMs.
**Limitations:** In addition to its retrospective nature, one of the main limitations of our study is the absence of systematic EMB.
**Ethics Committee Approval:** The study was approved by the local ethics committee (RGPD/APHM 2020-132), and informed consent was obtained from all patients.
**Funding for this study:** This study was supported by Assistance Publique – Hôpitaux de Marseille.
**Author Disclosures:**
Paul Habert: Nothing to disclose
Farah Cadour: Nothing to disclose
Ugo Scemama: Nothing to disclose
Alexis Jacquier: Nothing to disclose
Arthur Varoquaux: Nothing to disclose
Franck Thuny: Nothing to disclose
Jennifer Cautela: Nothing to disclose

### RPS 703-7

**Feature tracking vs speckle tracking in the assessment of myocardial deformation in highly trained endurance athletes**

*B. Domenech Ximenos*[1], M. Sanz-de la Garza[1], Á. Sepulveda[2], D. Lorenzatti[1], F. Simard[3], R. J. J. Perea Palazon[1], S. Prat-Gonzalez[1], M. Sitges[1]; [1]Barcelona/ES, [2]Santiago/CL, [3]Montreal, QC/CA
*(bl.domenech@gmail.com)*

**Purpose:** Assessment of myocardial deformation integrated with cardiac dimensions provides a comprehensive assessment of the ventricular remodeling patterns that might be induced by cumulative effects of intensive exercise. Although strain measurements using speckle tracking echocardiography (STE) have been widely reported, feature tracking (FT) methodology assessed by cardiac magnetic resonance (CMR) has not been standardized, and its accuracy is not yet fully validated. Our aim was to compare FT accuracy and reproducibility to STE in highly-trained endurance athletes.
**Methods or Background:** 93 athletes (>12hours training/week at least during the last 5years) and 72 matched controls (52%male, mean age 35±5.1years) underwent a resting CMR and an echocardiography to assess biventricular global longitudinal strain(GLS) by CMR-FT and STE.
**Results or Findings:** Strain values(both CMR-FT and STE) proportionally decreased with increasing ventricular volumes potentially depicting the increased volume and functional biventricular reserve that characterize highly-trained athletes' heart. Strain values were lower when assessed by CMR-FT as compared to STE(p<0.001), with good reproducibility for the LV (bias=3.94%,LOA=±4.27%) but wider variability for RV strains.
**Conclusion:** Both methods were comparable when measuring LV strain but not RV strain. Strain values were significantly lower when assessed by FT as compared to STE, which was expected due to the lower in-plane spatial and temporal resolution of FTvsSTE, which is particularly relevant for the complex anatomy of the RV.
**Limitations:** This study only assessed myocardial deformation analysis by FT and STE, and we did not include CMR tagging method.
**Ethics Committee Approval:** The Ethics Committee of our Institution approved the study.
**Funding for this study:** This work was partially funded by grants from AGAUR (M. Sanz-de la Garza, MD, PhD), Plan Nacional I. D, Del Programa Estatal de Fomento De La Investigación Científica y Técnica de Excelencia, Subprograma De Generación Del Conocimiento, Ministerio de Economía y Competitividad 2013 (grant number DEP2013–44923-P).
**Author Disclosures:**
Rosario Jesús Perea Palazon: Nothing to disclose
Daniel Lorenzatti: Nothing to disclose
Maria Sanz-de la Garza: Nothing to disclose
Marta Sitges: Nothing to disclose
Álvaro Sepulveda: Nothing to disclose
François Simard: Nothing to disclose
Blanca Domenech Ximenos: Nothing to disclose
Susanna Prat-Gonzalez: Nothing to disclose

### RPS 703-8

**Cardiac Erdheim-Chester disease: disease localization and impact on myocardial fibrosis**

*M. Gambardella*[1], A. Palmisano[2], D. Vignale[2], C. Campochiaro[2], A. Tomelleri[2], F. De Cobelli[2], L. Dagna[2], A. Esposito[2]; [1]Naples/IT, [2]Milan/IT
*(mic.gambardella@libero.it)*

**Purpose:** To characterize with Cardiac Magnetic Resonance (CMR) the site and amount of ECD cardiac involvement and its impact on function and fibrosis.
**Methods or Background:** Twenty-six biopsy-proven ECD patients (59 ± 13 years, 77% males) were enrolled between 2009 and 2019. All patients underwent CMR at 1.5 T using a standardized protocol to define cardiac ECD extension, Left Ventricle (LV) function and myocardial scar burden (+3SD).
**Results or Findings:** Right atrio-ventricular (AV) groove involvement was the most frequent (75% of cases; 31 ± 23 mL) followed by right atrial pseudo-mass (54%; 23.5 ± 19.1 mL), superior (38%; 16.5 ± 14.3 mL) and inferior (23%; 12.4 ± 6.7 mL) cava vein infiltration. Left AV groove and anterior interventricular groove involvement were less frequent (15%; 9.1 ± 5.8 mL and 15%; 7.8 ± 2.8 mL, respectively). Most of patients had pericardial effusion (54%; 138 ± 135.8 mL) with pericardial thickening in 25% cases. LGE was present in 19 patients (median 21% [IQR, 12-46%]) mostly involving basal and mid-ventricular infero-septal (18/19) and inferior (8/19) walls. LGE presence was associate to right AV groove involvement (p < 0.001). Indexed LV End Diastolic Volume, Mass and Ejection Fraction values were normal. Coated aorta was present in 60% cases.
**Conclusion:** ECD mostly involved the right atrio-ventricular groove which is associated to inferior LV fibrosis.
**Limitations:** Relatively small sample size.
**Ethics Committee Approval:** Approved by Institutional Review Board and waiver of consent for retrospective study.
**Funding for this study:** No funding was recived.
**Author Disclosures:**
Davide Vignale: Nothing to disclose
Lorenzo Dagna: Nothing to disclose
Antonio Esposito: Nothing to disclose
Alessandro Tomelleri: Nothing to disclose
Anna Palmisano: Nothing to disclose
Francesco De Cobelli: Nothing to disclose
Michele Gambardella: Nothing to disclose
Campochiaro Corrado Campochiaro: Nothing to disclose

### RPS 703-9

**Cardiac resynchronisation in treating the patients with left ventricular non-compaction cardiomyopathy (LVNC): the role of CMR in the prognosis of outcome**

*E. Mershina*, R. Myasnikov, O. Kulikova, S. Koretskiy, O. Larina, M. Kharlap, P. Pilius, V. Sinitsyn, O. Drapkina; Moscow/RU
*(elena_mershina@mail.ru)*

**Purpose:** LVNC is a rare disease characterized by a structural abnormality of the myocardium with uncertain etiology and pathogenesis. Patients with LVNC and heart failure with low ejection fraction (HFrEF) are treated with the same therapy as the patients with other reasons of HFrEF. However, the cardiac resynchronization therapy (CRT) in treating the patients with LVNC, left bundle branch block (LBBB) and HFrEF remains insufficiently studied. The aim of our study was to demonstrate the prognostic role of LGE CMR results before CRT-D application in patients with LVNC.
**Methods or Background:** The diagnosis of LVNC was proven by Echo- (Chin, Jenni, Stollberger) and CMR- (Petersen, Grothoff) criteria of non-compaction. LGE CMR was used to demonstrate intramyocardial fibrosis. EchoCG was used to monitor the response after CRT-D installation. The echo response (ER) was defined as a decrease in left ventricular end systolic volume (LVESV)≥15%.
**Results or Findings:** We studied the group of 19 patients (m/w–9/10, mean age 56 years[40;65]) with LVNC (6 pts with family history of LVNC), HFrEF – mean LVEF 28% [23;30]. All the patients had LBBB with median QRS 160 ms. 9 of 19 pts were ER; 8 of 19 pts (42%) were super-responders, with the decrease of LVESV ≥30%. The number of segments with intramyocardial fibrosis foci were significantly less in the ER group in comparison to non-responders, 12 vs 76 (p<0.001). Ration of NC mass to the compacted myocardium mass was higher in the group of non-responders: 26.4% vs 21.5% (p<0.1).
**Conclusion:** In the patients with LVNC the presence of intramyocardial fibrosis defined with use of LGE CMR was an independent prognostic factor for worse clinical response after CRT-D installation.
**Limitations:** Small group of patients; retrospective study
**Ethics Committee Approval:** Approved by the Moscow University Ethics Committee.
**Funding for this study:** No
**Author Disclosures:**
Maria Kharlap: Nothing to disclose
Polina Pilius: Nothing to disclose
Olga Larina: Nothing to disclose
Valentin Sinitsyn: Nothing to disclose
Olga Kulikova: Nothing to disclose
Elena Mershina: Nothing to disclose
Oksana Drapkina: Nothing to disclose
Roman Myasnikov: Nothing to disclose
Sergei Koretskiy: Nothing to disclose

### Research Presentation Session: Cardiac

### RPS 803

### Angiography

### RPS 803-1

**Pre and postcontrast pericoronary CT attenuation values with regards to coronary stenosis**

*C. B. Monti*, D. Capra, C. Parietti, G. Florini, M. Zanardo, F. Sardanelli, F. Secchi; Milan/IT
*(caterinab.monti@gmail.com)*

**Purpose:** To assess pre- and post-contrast attenuation values of pericoronary adipose tissue (pCAT) with regards to coronary artery stenosis in patients undergoing cardiac CT (CCT) for suspected coronary artery disease (CAD).
**Methods or Background:** All patients who underwent CCT between January 2016 and June 2019 were retrospectively included. Patients who did not have both pre- and post-contrast scans, or whose CCTs presented with relevant artifacts were excluded. A round region of interest with a 1 cm diameter was placed in the post-contrast scan around each of the three main coronary arteries on the slice that best depicted all. Such segmentations were then thresholded for fat (-190 to -30 Hounsfield units), and subsequently registered on the basal scan. Data are presented as median and interquartile range (IQR).
**Results or Findings:** A total of 480 patients were included. CAD patients were older than those without (70 years, IQR 63–76 versus 62 years, 54–69, p<0.001), and more often males (70% versus 56%, p=0.001). For all coronary arteries both pre- and post-contrast pCAT attenuations were higher in CAD patients than in those without (p=0.001 both pre- and post-contrast in the right coronary artery, p<0.001 both pre- and post-contrast in the interventricular descending artery and the basal circumflex artery), except for the post-contrast, pCAT around the circumflex artery where there was no significant difference (p=0.131).
**Conclusion:** pCAT attenuation is higher in CAD patients, likely due to disease-related processes such as inflammation and adipose tissue beiging or fibrosis.
**Limitations:** Retrospective, single-centre.
**Ethics Committee Approval:** The study was approved by the local Ethics Committee.
**Funding for this study:** The study was partially supported by BLINDED.
**Author Disclosures:**
Giorgia Florini: Nothing to disclose
Francesco Secchi: Nothing to disclose
Francesco Sardanelli: Speaker: Bayer, General Electric, Bracco Grant Recipient: Bayer, General Electric, Bracco Advisory Board: Bracco
Caterina Beatrice Monti: Nothing to disclose
Moreno Zanardo: Nothing to disclose
Carlo Parietti: Nothing to disclose
Davide Capra: Nothing to disclose

### RPS 803-2

**A spectrum of extracardiac vascular anomalies on CT angiography in pediatric patients of congenital heart disease and its correlation with intraoperative findings**

R. Anand, *M. Penuganchiprolu*, B. Aggarwal; Delhi/IN
*(manasasmc@gmail.com)*

**Purpose:** To evaluate the spectrum of CT angiography (CTA) in various extra cardiac vascular anomalies in pediatric patients of congenital heart disease and its correlation with operative findings.
**Methods or Background:** This is a retrospective study wherein two hundred fifty four consecutive patients with known congenital heart disease who were referred for CTA as a part of their regular clinical work up from January 2017 to December 2019 were included. Operative findings were also recorded.
**Results or Findings:** The vascular anomalies on CTA were found in 191 patients giving an incidence of 75.1% with MAPCAs showing the highest incidence (34.61%) followed by left pulmonary artery origin stenosis (11.4%) and interrupted IVC (9.4%). Out of 254 patients 220 patients underwent surgery. On operation vascular anomalies were found in 156 patients giving an incidence of 71.1%. Most common vascular anomaly detected was MAPCAs. The most common congenital heart disease detected was TOF on both CTA and operation.
**Conclusion:** As patients of congenital heart diseases also present with a wide range of associated extra cardiac vascular findings which range from non-significant to those affecting the clinical outcome of patients. Assessment of extra-cardiac vessels is mandatory at the time of performing these studies as these findings are important for giving a complete diagnosis and for treatment planning in some cases.
**Limitations:** -As pediatric patients cannot hold their breath, image quality was compromised in few cases. -Few vascular anomalies were missed due to overlapping of adjacent vascular structures.
**Ethics Committee Approval:** Appropriate consents were obtained during the primary study and surgery. It is a retrospective study only the recorded data was used for analysis.
**Funding for this study:** No funding has been provided for this study.
**Author Disclosures:**
Reena Anand: Nothing to disclose
Bharat Aggarwal: Nothing to disclose
Manasa Penuganchiprolu: Nothing to disclose

### RPS 803-3

**Cardiac event prediction by evaluating the variation of perivascular adipose tissue in serial coronary CT angiography**
*R. Gao*, Y. Gao, H. Han, K. Zhou, B. Lu; Beijing/CN
*(ruochen.gao@miracle.ict.ac.cn)*

**Purpose:** To evaluate the variation of perivascular adipose tissue (PVAT) for cardiac event prediction in patients with suspected coronary artery disease (CAD) who underwent serial coronary CT angiography (CCTA).
**Methods or Background:** One hundred and eleven patients with suspected CAD underwent serial CCTA due to new or worsening symptoms were enrolled. Patients were followed-up for the incidence of cardiac events defined as cardiac death, coronary revascularization, nonfatal myocardial infarction and hospitalization due to unstable angina. Perivascular fat attenuation mapping was automatically done around the proximal right coronary artery (RCA), the left anterior descending artery (LAD), and the left circumflex artery (LCX) using our own developed algorithm. The variation of PVAT (∆PVAT) was defined as the difference between PVAT2 and PVAT1.
**Results or Findings:** 111 patients (mean age 59.9 years ± 10.1, male 65.8%) were followed-up with 612.3 days ± 200.6, 6 patients were lost. Twenty-eight patients had cardiac events including 16 coronary revascularization, 5 myocardial infarction and 7 hospitalization. ∆PVAT of LAD, LCX and RCA in patients without events were -2.72 (-7.93, 3.27), -2.94 (-7.00, 2.16), -2.57 (-8.65, 2.00), which were all significantly lower than those with events［ 1.05, (-3.21, 4.81), 1.37 (-3.64, 5.07), 1.23 (-6.71, 4.61), respectively, all $p < 0.05$］. In patients underwent events, PVAT of the three vessels were all increased / decreased in the second CCTA in 10 (35.7%)/5 (17.9%) patients, which was significantly higher/lower than those without events (3 (3.9%), 26 (33.7%), both $p < 0.05$).
**Conclusion:** The variation of PVAT is a novel indicator to predict cardiac events. With the increase of PVAT in the major three coronary arteries, the risk of cardiac event also grows.
**Limitations:** Our results require further prospective studies in large cohorts
**Ethics Committee Approval:** This retrospective study was approved by the Ethics Committee.
**Funding for this study:** None
**Author Disclosures:**
Yang Gao: Nothing to disclose
Bin Lu: Nothing to disclose
Hu Han: Nothing to disclose
Ruochen Gao: Nothing to disclose
Kevin Zhou: Nothing to disclose

### RPS 803-4

**The orientation of the right superior pulmonary vein affects outcome after pulmonary vein isolation**
*M. Vecsey-Nagy*, N. Szegedi, J. Simon, B. Szilveszter, S. Herczeg, M. Kolossvary, P. Maurovich-Horvat, B. Merkely, L. Geller; Budapest/HU

**Purpose:** No data is available on the impact of PV orientation on the long-term success rates of point-by-point PVI. We sought to determine the impact of PV anatomy and orientation on atrial fibrillation (AF)-free survival in patients undergoing PVI using radiofrequency point-by-point technique.
**Methods or Background:** We retrospectively included 448 patients who underwent initial point-by-point radiofrequency ablation for AF at our department. Left atrial CT-angiography (CTA) was performed before each procedure. PV anatomical variations, ostial parameters (area, effective diameter and eccentricity), orientation and their associations with 24-month AF-free survival were analyzed.
**Results or Findings:** PV anatomical variations and ostial parameters were not predictive for AF-free survival (all p>0.05). Univariate analysis showed that female gender (p=0.025) was associated with higher rates of AF recurrence, ventral-caudal (p=0.002), dorsal-cranial (p=0.034) and dorsal-caudal (p=0.042) orientation of the right superior PV (RSPV), on the other hand, showed an association with lower rates of AF recurrence, as compared to the reference ventral-cranial orientation. In multivariate analysis, both female gender [odds ratio (OR) 1.83, 95% CI 1.15-2.93, p=0.011] and ventral-caudal RSPV orientation, compared with ventral-cranial orientation, proved to be independent predictors of 24-month recurrence (OR 0.37, 95% CI 0.19-0.71, p=0.003).
**Conclusion:** Female gender and ventral-caudal RSPV orientation have an impact on long-term arrhythmia-free survival. Assessment of PV orientation may be a useful tool in predicting AF-free survival and may contribute to a more personalized management of AF.
**Limitations:** This is a single center, retrospective study with a limited number of patients.
**Ethics Committee Approval:** The requirement for informed consent was waived due to the retrospective
nature of the data.
**Funding for this study:** This research did not receive any specific grant from funding agencies.
**Author Disclosures:**
Nándor Szegedi: Nothing to disclose
Milán Vecsey-Nagy: Nothing to disclose
Bela Merkely: Nothing to disclose
Szilvia Herczeg: Nothing to disclose
Marton Kolossvary: Nothing to disclose
Judit Simon: Nothing to disclose
Bálint Szilveszter: Nothing to disclose
Pál Maurovich-Horvat: Nothing to disclose
Laszlo Geller: Nothing to disclose

### RPS 803-5

**Advanced myocardial tissue characterisation with cardiac CT in patients presenting with low-risk troponin positive chest pain**
*D. Vignale*[1], A. Palmisano[1], M. Gatti[2], R. Faletti[2], M. Tadic[3], F. Moroni[1], F. De Cobelli[1], A. Esposito[1]; [1]Milan/IT, [2]Turin/IT, [3]Belgrade/RS
*(vignale.davide@hsr.it)*

**Purpose:** To evaluate the diagnostic yield of Late Iodine Enhancement (LIE) acquisition added to triple-rule-out CT (TRO-CT) performed in patients presenting with acute symptoms and troponin elevation without clinical/electrocardiographic criteria for acute myocardial infarction (AMI).
**Methods or Background:** 80 consecutive patients with: 1)acute chest pain or equivalent; 2)troponin elevation; 3)no diagnostic criteria for AMI, underwent TRO-CT to rule-out obstructive coronary artery disease (CAD-RADS≥4), pulmonary embolism (PE), or acute aortic syndromes (AAS). Patients with negative TRO-CT underwent LIE scan to: 1) evaluate presence/pattern of LIE; 2) quantify the myocardial extracellular volume fraction (ECV). Invasive coronary angiography and cardiac magnetic resonance were used as reference standard.
**Results or Findings:** Forty two out of 80 patients (42/80) were male, median age=69 years [IQR=52-78]. Peak median troponinT was 49.0 ng/L [IQR=25-151]. Reported symptoms were chest pain (n=51[64%]), dyspnoea (n=17[21%]), syncope (n=9[11%]), sudden cardiac arrest (n=3[4%]). TRO-CT identified: obstructive CAD 31(39%); AAS 1(1%); PE 6(8%). Additional non-vascular diagnosis were: cardiac metastasis 1(1%); pericarditis 2(3%). LIE acquisition was performed in TRO-negative patients (39/80). 17(21%) had non-ischemic LIE suggestive for myocarditis; 2(3%) ischemic LIE with no-reflow suggestive for MINOCA; 5(6%) dilated cardiomyopathy [1 ischemic LIE; 2 non-ischemic LIE; 2 without LIE]; 3(4%) massively increased ECV suggestive for amyloidosis, 4(5%) apical ballooning without scar suggestive of Takotsubo. 8(10%) patients had both negative TRO and LIE; troponin dispersion was attributed to atrial fibrillation (5[6%]), kidney disease (2[3%]), and not found in 1(1%).
**Conclusion:** LIE acquisition increases the diagnostic value of TRO-CT, finding a diagnosis in 79% of TRO-negative patients.
**Limitations:** Single-centre study. Relatively small sample size.
**Ethics Committee Approval:** Retrospective study approved by the institutional review board.
**Funding for this study:** No funding received.

**Author Disclosures:**
Davide Vignale: Nothing to disclose
Antonio Esposito: Nothing to disclose
Francesco Moroni: Nothing to disclose
Riccardo Faletti: Nothing to disclose
Anna Palmisano: Nothing to disclose
Francesco De Cobelli: Nothing to disclose
Marijana Tadic: Nothing to disclose
Marco Gatti: Nothing to disclose

### RPS 803-6

**CT based coronary artery access evaluation for a valve in valve TAVI patients using 3D modelling, 3D printing and mixed reality visualisation**

*A. A. Zlahoda-Huzior*[1], A. Khokhar[2], F. Ponticelli[2], K. Kotarba[1], A. Laricchia[2], F. Giannini[2], D. Dudek[1]; [1]Krakow/PL, [2]Cotignola/IT
*(adriana.zlahoda@medapp.pl)*

**Purpose:** Coronary access (CA) following valve-in-valve (ViV) transcatheter aortic valve implementation (TAVI) may be extremely difficult due to the presence of two valvular prostheses. CA evaluation is usually performed using fluoroscopic images or computed tomography (CT) scans which often do not provide a comprehensive three-dimensional (3D) estimation of the volume and geometry of the structures. Accordingly, we propose the enhanced and specific visualization of regions of interest using 3D modeling, printing and Mixed Reality (MR).
**Methods or Background:** CT scans of 20 patients after ViV-TAVI with the ACURATE neo valve (Boston Scientific) were collected using Siemens SOMATOM Definition Flash scanner (512x512 pixels, spacing < 0.4 mm, slice thickness 0.3 mm). Semi-automatic data segmentation was performed using Slicer3D. Segmentation results were transformed into 3D mesh models constituting the base of 3D graphics, printings and holograms.
**Results or Findings:** 3D graphic models were presented on the flat screen with the possibility of translation, rotation, zooming or changing the material texture settings. Patient-specific, real-size 3D printings were performed in PolyJet technology using Stratasys J720 printer (CadXpert, Poland). Sufficient radiolucency of the tissue-like materials allowed to perform CA re-engagement tests under fluoroscopy. Holographic visualizations of 3D light-printed models were presented in CarnaLife Holo system (MedApp, Poland) connected to Microsoft HoloLens headset.
**Conclusion:** Enhanced visualization of ViV-TAVI data will likely become crucial for pre-procedural planning offering faster, easier and more flexible data analysis. Additionally, bench-side testing on 3D printed models may facilitate the selection of catheter and adjunctive materials prior to the procedure.
**Limitations:** Further tests and clinical validation of proposed 3D visualization techniques should be performed to confirm the usefulness of the method.
**Ethics Committee Approval:** NA for the study.
**Funding for this study:** AGH UST Dean’s Grant, the subsidy from the Minister of Science and Higher Education,16.16.120.773, task 60
**Author Disclosures:**
Francesco Giannini: Nothing to disclose
Dariusz Dudek: Nothing to disclose
Alessandra Laricchia: Nothing to disclose
Arif Khokhar: Nothing to disclose
Francesco Ponticelli: Nothing to disclose
Adriana Anna Zlahoda-Huzior: Employee: MedApp S.A.
Katarzyna Kotarba: Nothing to disclose

### RPS 803-8

**Environmental tobacco smoke exposure estimated using the SHSES scale, and feature tracking computed tomography derived left ventricular global longitudinal strain in hypertensive patients**

*P. Gac*, M. Poreba, P. Macek, G. Mazur, R. Poreba; Wroclaw/PL
*(pawelgac@interia.pl)*

**Purpose:** Aim of the study was to assess the relationship between ETS and feature tracking computed tomography-derived left ventricular global longitudinal strain (LV GLS) in patients with hypertension.
**Methods or Background:** 103 non-smokers with arterial hypertension were included in the study. The group consisted of 49 females and 54 males with the mean age of 67.73 ±8.84 years. Environmental tobacco smoke exposure was assessed with The Secondhand Smoke Exposure Scale (SHSES). LV GLS was measured on dynamic multidetector computed tomography using feature tracking technology. In accordance to SHSES scale patients were divided into subgroups: subgroup A– no ETS exposure (SHSES= 0 points, n=48), subgroup B– low ETS exposure (SHSES= 1-3 points, n=16), subgroup C– medium ETS exposure (SHSES= 4-7 points, n=21) and subgroup D– high ETS exposure (SHSES= 8-11 points, n=18).
**Results or Findings:** Within the study group the mean peak of LV GLS was 15.84 ±3.85% and the mean SHSES score was 3.37 ±3.96. Peak of LV GLS was statistically significantly lower in subgroup D than in subgroup A (A: 17.35 ±3.31%, D: 14.21 ±3.01%, p<0.05). There was a negative linear correlation between the exposure to ETS expressed by the SHSES scale and peak of LV GLS (r = -0.35, p<0.05). Regression analysis showed that higher SHSES score, higher age, higher diastolic blood pressure and higher LDL cholesterol concentration are independent risk factors for lower peak of LV GLS values. Contrary, the use of β-blockers appeared to be independent protecting factor against lower peak of LV GLS values.
**Conclusion:** There is an unfavourable relationship between ETS exposure estimated using the SHSES scale and LV GLS in hypertensive patients.
**Limitations:** Small study group size. Lack of blood cotinine concentration test.
**Ethics Committee Approval:** Local Bioethic committee approval.
**Funding for this study:** Wroclaw Medical University
**Author Disclosures:**
Rafal Poreba: Nothing to disclose
Malgorzata Poreba: Nothing to disclose
Pawel Gac: Nothing to disclose
Grzegorz Mazur: Nothing to disclose
Piotr Macek: Nothing to disclose

### Research Presentation Session: Cardiac

### RPS 903

### Cardiac diagnosis and outcomes

### RPS 903-1

**Impact of timing of hematocrit evaluation for calculation of ECV in cardiac magnetic resonance**

*C. Reiter*, G. Reiter, C. Kräuter, V. Nizhnikava, A. Schmidt, D. Scherr, M. Fuchsjäger, U. Reiter; Graz/AT
*(Clemens.Reiter@medunigraz.at)*

**Purpose:** Cardiac magnetic resonance (CMR) imaging assessment of myocardial extracellular volume (ECV) requires the knowledge of blood hematocrit (HCT). In clinical routine, available HCT values are often non-recent. Aim of this study was to compare ECV derived from HCT assessed immediately before CMR and from routine laboratory data.
**Methods or Background:** 27 subjects undergoing routine contrast-enhanced 3T CMR (Magnetom Skyra, Siemens Healthcare) for whom blood laboratory including HCT (lab-HCT) within 90 days was available, were enrolled in this prospective study. Blood samples were drawn immediately before CMR imaging (onsite-HCT) using a point-of-care-meter system (StarTrip, Nova Biomedical). Global ECV was determined for both HCT values by segmenting three native and post-contrast Modified Look-Locker inversion recovery (MOLLI) T1-maps in short-axis orientation using routine software (cvi42, Circle Cardiovascular Imaging). Relationships between parameters based on lab-HCT and onsite-HCT were analyzed by Bland-Altman and correlation analysis. p<0.05 was considered statistically significant.
**Results or Findings:** Mean time between CMR and lab-HCT was 16±23 days. There was no significant bias between onsite-HCT (40±5%) and lab-HCT (40±7%, p=0.32), standard deviation of differences was 3%, and HCT values showed a strong correlation (r=0.89). Moreover, there was no significant correlation between the differences of HCT values and the time span between HCT assessments. ECV values showed no significant bias (onsite-HCT: ECV 27.5±4.6%, lab-HCT: ECV 27.7±4.9%; p=0.35), standard deviation of differences was 1.3%, and their correlation was strong (r=0.95).
**Conclusion:** As neither a significant difference nor a large variability in ECV was observed, the use of routine laboratory HCT values might be feasible for routine ECV assessment.
**Limitations:** Small sample size. No post-CMR HCT measurements were performed.
**Ethics Committee Approval:** Obtained.
**Funding for this study:** OeNB-Anniversary-Fund No.17934, ESOR Fellowship 2020, MedUni Graz Foerderungsstipendien 2020.
**Author Disclosures:**
Ursula Reiter: Nothing to disclose
Gert Reiter: Employee: Gert Reiter is an employee of Siemens Healthcare Diagnostics GmbH, Austria.
Michael Fuchsjäger: Nothing to disclose
Daniel Scherr: Nothing to disclose
Clemens Reiter: Nothing to disclose
Albrecht Schmidt: Nothing to disclose
Volha Nizhnikava: Nothing to disclose
Corina Kräuter: Nothing to disclose

### RPS 903-2

**Novel virtual non-contrast reconstruction for coronary calcium scoring derived from coronary CT angiography using dual-layer dual-energy CT**

*M. C. Langenbach*[1], N. Grosse Hokamp[1], A. Bunck[1], J. Holz[1], E. Langzam[2], D. Maintz[1], L. Pennig[1]; [1]Cologne/DE, [2]Haifa/IL
*(marcel.langenbach@me.com)*

**Purpose:** To investigate assessment of calcified coronary plaques by calcium scoring (CaSc) using a novel virtual non-contrast (VNC) reconstruction algorism derived from contrast-enhanced coronary CT angiography (cCTA) compared to true non-contrast (TNC) images.
**Methods or Background:** Twenty consecutive patients (median age 55 years, range 30-86, 6 female) receiving cCTA with suspicion of coronary artery disease were included in this retrospective, single-center trial. All patients were scanned with the same dual-layer dual-energy CT using a standardized acquisition protocol in clinical routine including both, TNC scan for CaSc and contrast-enhanced cCTA. In all patients, VNC-reconstructions were calculated from cCTA images using a novel post-processing algorism discriminating calcified plaques in coronary arteries from contrast media and adjacent soft tissue. VNC images were calculated with identical imaging parameters as TNC scans. . Two radiologists independently analyzed VNC and TNC images regarding CaSc (by standard postprocessing software using the proprietary image viewer) as well as image quality, plaque delineation and beam hardening artifacts using 5-point Likert scales (5=excellent).
**Results or Findings:** In all patients, reconstruction of VNC-images was performed successfully. For TNC and VNC images image quality (mean score 4.1 vs. 4.2, p=0.832) and delineation of calcified plaques (mean score 4.1 vs. 4.4, p=0.798) was excellent. Compared to TNC, beam hardening artifacts were significantly reduced in VNC-images allowing a clearer delineation of calcified plaques (mean score 3.2 vs. 4.3, p=0.02). Calculated Agatston-Score showed higher values for TNC vs. VNC (172.9±247.0 vs. 76.4±107.7, p=0.385) with strong correlation of respective values (rho=0.853, p<0.001).
**Conclusion:** VNC reconstructions of contrast-enhanced cCTA enable a reliable detection and evaluation of calcified coronary plaques without additional acquisition of a native CT-scan. Reduction of beam-hardening artifacts in VNC-images potentially allows a more exact evaluation by Agatston-Score.
**Limitations:** Retrospective
**Ethics Committee Approval:** Approved
**Funding for this study:** Software support (Philips)
**Author Disclosures:**
Jasmin Holz: Nothing to disclose
Lenhard Pennig: Nothing to disclose
David Maintz: Nothing to disclose
Nils Grosse Hokamp: Nothing to disclose
Eran Langzam: Employee: Philips
Marcel Christian Langenbach: Research/Grant Support: Guerbet GmbH
Alexander Bunck: Nothing to disclose

### RPS 903-3

**Myocardial native T1 mapping and cardiac outcomes in thalassemia major**

A. Meloni[1], N. Martini[1], A. De Luca[2], V. Positano[1], L. Pistoia[1], D. Visceglie[3], S. Gentili[4], G. Sinagra[2], *A. Pepe*[1]; [1]Pisa/IT, [2]Trieste/IT, [3]Bari/IT, [4]Arezzo/IT
*(alessia.pepe@ftgm.it)*

**Purpose:** The T2* cardiovascular magnetic resonance (CMR) is the gold standard for the non invasive detection of myocardial iron overload (MIO). The native myocardial T1 mapping has been proposed as a complementary tool, thanks to its higher sensitivity to small amounts of iron. We explored the clinical impact of T1 mapping for detecting cardiac complications in thalassemia major (TM).
**Methods or Background:** We considered 146 patients (87 females, 38.7±11.1 years) consecutively enrolled in the Extension-Myocardial Iron Overload in Thalassemia Network. Three parallel short-axis slices of the left ventricle were acquired in end-diastole by a Modified Look-Locker Inversion recovery sequence with a 3(3s)3(3s)5 scheme. The native T1 values in all 16 myocardial segments were obtained and the global value was the mean.
**Results or Findings:** Twenty-one patients had an history of cardiac complications: 11 heart failure, 8 arrhythmias (7 supraventricular, 1 ventricular), and 2 pulmonary hyperthension. Patients with cardiac complications had significantly lower global heart T1 values (879.3±121.9 ms vs 963.2±98.5 ms; P<0.0001) but comparable T2* values (33.32±11.66 ms vs 37.17±9.15 ms; P=0.116). Cardiac complications were more frequent in the group of patients with reduced global heart T1 value (males:<928 ms, females:<989 ms) compared to the group with normal global heart T1 value (71.4% vs 39.5%; P=0.009). Odds ratio for cardiac complications was 3.8 (95%CI=1.3-10.9) for patients with reduced global heart T1 value versus patients with normal global heart T1 value.
**Conclusion:** We found out a significant association between decreased native global heart T1 values and a history of cardiac complications, suggesting that an early detection of myocardial iron burden by native T1 can support the clinicians in modifing chelation therapy earlier.
**Limitations:** No prospective
**Ethics Committee Approval:** Local Ethics committee
**Funding for this study:** No-profit support from Chiesi Farmaceutici S.p.A. and ApoPharma Inc.
**Author Disclosures:**
Alessia Pepe: Research/Grant Support: Chiesi Farmaceutici S.p.A., ApoPharma Inc. Speaker: Chiesi Farmaceutici S.p.A.
Gianfranco Sinagra: Nothing to disclose
Nicola Martini: Nothing to disclose
Antonio De Luca: Nothing to disclose
Sara Gentili: Nothing to disclose
Antonella Meloni: Speaker: Chiesi Farmaceutici S.p.A.
Laura Pistoia: Speaker: Chiesi Farmaceutici S.p.A.
Domenico Visceglie: Nothing to disclose
Vincenzo Positano: Nothing to disclose

### RPS 903-4

**Functional analysis of the left atrium provides high diagnostic accuracy to detect Fabry's disease and correlates with disease severity**

*S. Altmann*[1], M. C. Halfmann[1], C. Reichardt[1], K-F. Kreitner[1], C. Düber[1], B. Yacoub[2], U. J. Schoepf[2], A. Varga-Szemes[2], T. Emrich[2]; [1]Mainz/DE, [2]Charleston, SC/US

**Purpose:** Fabry's disease (FD) is leading to increased myocardial mass and shortened myocardial T1, resulting in pathological alteration of myocardial geometry and function. Data concerning correlation and diagnostic performance comparing markers of disease severity, determined by myocardial mass, T1 and left atrial (LA) function, using cardiac MRI has not been demonstrated. The aim of this study was to evaluate changes in LA strain in correlation to FD severity and demonstrate the improvement in diagnostic performance by combining those parameters for myocardial involvement.
**Methods or Background:** 3T CMR of fifty-seven FD patients (17 pre-hypertrophic stage,40 with cardiac involvement) and 50 healthy volunteers were included in this retrospective study. Global longitudinal strain of LA was calculated from 2-,3-,and 4-chamber cine acquisitions using feature-tracking technique. Group comparisons were performed using Mann-Whitney-U test and Pearson's correlation coefficient. Receiver operating characteristic (ROC) curve analysis was used to calculate area under the curve (AUC). DeLong method was used for pairwise to determine differences between AUCs.
**Results or Findings:** There were significant differences between healthy volunteers and FD patients in T1, myocardial mass, and LA reservoir strain (all p<0.001). LA reservoir strain correlated with T1 (r=0.421,p=0.023) and inversely correlated with left ventricular myocardial mass (r=-0.489,p<0.001). Combining LA function parameters with myocardial mass and T1 improved diagnostic performance to detect FD in every disease stage, providing excellent diagnostic performance (AUC=0.93, sensitivity: 91.8%, specificity: 86.1%), outperforming conventional parameters used to assess myocardial involvement.
**Conclusion:** LA strain parameters have potential to complement current multiparametric approaches to diagnose myocardial involvement in FD, especially in the pre-hypertrophic phase. Incorporating LA strain parameters into FD diagnostic work-up would allow better evaluation of disease severity and progression, potentially improving initiation of ERT and monitoring of treatment success.
**Limitations:** Single-center, retrospective, no histological validation
**Ethics Committee Approval:** Approval-Number:837.196.13/837.477.14
**Funding for this study:** Funding not applicable
**Author Disclosures:**
Uwe Joseph Schoepf: Other: Elucid Bioimaging (Other Activities) Other: HeartFlow (Other Activities) Other: Siemens Healthcare (Other Activities) (Travel support) Other: Bayer Healthcare (Other Activities) Other: Bracco (Other Activities) Other: Guerbet (Other Activities)
Christoph Düber: Nothing to disclose
Constantin Reichardt: Nothing to disclose
Tilman Stephan Emrich: Speaker: Siemens Healthcare (Travel Support)
Moritz Christian Halfmann: Nothing to disclose
Basel Yacoub: Nothing to disclose
Karl-Friedrich Kreitner: Nothing to disclose
Akos Varga-Szemes: Consultant: Bayer Healthcare Consultant: Elucid Bioimaging Other: Siemens Healthcare (Other Activities) (Travel support)
Sebastian Altmann: Nothing to disclose

### RPS 903-5

**Myocardial tissue characterization by T2 mapping in thalassemia major**

A. Meloni[1], N. Martini[1], R. Borrello[1], V. Positano[1], L. Pistoia[1], M. Murgia[2], C. Paci[3], R. Pedrinelli[1], *A. Pepe*[1]; [1]Pisa/IT, [2]Oristano/IT, [3]Arezzo/IT

**Purpose:** We compared myocardial T2 against T2* in patients with thalassemia major (TM) for myocardial iron characterization.
**Methods or Background:** 133 TM patients (79 females, 38.4±11.3 years) enrolled in the Extension Myocardial Iron Overload in Thalassemia Network underwent MRI. T2 and T2* images were acquired, respectively, with multi-echo fast-spin-echo and gradient-echo sequences. Global heart T2 and T2* values were obtained by averaging the values in all 16 myocardial segments. Data acquired on 80 healthy volunteers were used to establish the normal T2 range (males:48-56 ms;females:50-57 ms) and the lower limit of normal for global heart T2* (32 ms).
**Results or Findings:** A significant correlation was detected between global heart T2 and T2* values (R=0.577; P<0.0001). Out of the 113(84.9%) patients with a normal global heart T2* value, none had a decreased global heart T2 value, while 58(51.3%) had an increased T2 value. Out of the 20 patents with a decreased global heart T2* value, only 10(50%) had also a reduced T2 value. Conversely, 9(45.0%) had a normal global heart T2 value and one(4.5) showed an increased T2 value. The 59 patients with increased global heart T2 value were significantly older than the remaining patients (40.8±10.5 vs 36.4±11.6 years; P=0.019)
**Conclusion:** All patients with decreased T2 value had also a decreased T2* value and in half of the patients iron load was undetected by T2, suggesting that T2 mapping does not offer any advantage in terms of sensitivity for MIO assessment. However, more than half of TM patients had an increased T2 value, thus may be caused by the presence of myocardial inflammation and/or edema. So, T2 mapping could reveal subclinical myocardial involvement in TM patients.
**Limitations:** Single-center study.
**Ethics Committee Approval:** Local Ethics Committee
**Funding for this study:** No-profit support from Chiesi Farmaceutici and Apopharma
**Author Disclosures:**
Alessia Pepe: Research/Grant Support: Chiesi Farmaceutici S.p.A., ApoPharma Inc. Speaker: Chiesi Farmaceutici S.p.A.
Nicola Martini: Nothing to disclose
Roberto Pedrinelli: Nothing to disclose
Antonella Meloni: Speaker: Chiesi Farmaceutici S.p.A.
Rita Borrello: Nothing to disclose
Mauro Murgia: Nothing to disclose
Laura Pistoia: Speaker: Chiesi Farmaceutici S.p.A.
Cristina Paci: Nothing to disclose
Vincenzo Positano: Nothing to disclose

### RPS 903-6

**Diagnostic value of cardiothoracic ratio in the assessment of the heart in patients with non-ischemic cardiomyopathy: comparison with cardiovascular magnetic resonance**

*P. Simkus*[1], A. Banišauskaitė[2], J. Noreikaite[1], M. Gutierrez[1], D. McCreavy[1], D. Penha[1], M. Arzanauskaite[1]; [1]Liverpool/UK, [2]Kaunas/LT
*(pauliusimkus@gmail.com)*

**Purpose:** To determine reliability of the cardiothoracic ratio (CTR) as a simple method to assess the cardiac size and function in patients with non-ischemic cardiomyopathy (NICM).
**Methods or Background:** Cardiovascular magnetic resonance (CMR) is the gold standard for cardiac volumetric and functional assessment. We used CMR to evaluate whether the long-standing and widely available CTR (with a cut-off value of 50% in a posteroanterior chest radiograph) can be a valuable method for predicting heart size. CTR derived from chest radiographs (CXR) was compared to CMR volumetric and functional parameters. The sample (n=91) consisted of two groups: patients with NICM (n=66) and a control group with normal CMR parameters (n=25).
**Results or Findings:** The mean CTR in the NICM group was 50.74±5.49% (CTR was increased in 35 patients, 53%) and significantly higher compared to the control group (45.28±4.74%). On CMR, LVEDVi, LV indexed mass, LA indexed volume, LA indexed area and RA indexed area were higher and LVEF and RVEF were lower in patients with non-ischemic cardiomyopathy (p<0.05). In patients with non-ischemic cardiomyopathy, the greatest CTR relationship was determined to LVEDVi, LA indexed volume and LA indexed area (ρ=0.405, ρ=0.478 and ρ=0.467, respectively), however the strength of correlation was moderate.
**Conclusion:** CTR was significantly higher in patients with NICM compared to the control group. Nevertheless, an increased CTR is not specific for NICM. The weak correlation noted between CTR and CMR parameter limits CTR reliability for reflecting the underlying cardiac volumetric and functional characteristics.
**Limitations:** Heterogeneous sample (several types of NICM were included in the study).
**Ethics Committee Approval:** IRB approval has been obtained.
**Funding for this study:** Funded by the ESR Research Seed Grant.
**Author Disclosures:**
Monika Arzanauskaite: Nothing to disclose
Jurate Noreikaite: Nothing to disclose
Diana Penha: Nothing to disclose
Audra Banišauskaitė: Nothing to disclose
Paulius Simkus: Nothing to disclose
Manuel Gutierrez: Nothing to disclose
David McCreavy: Nothing to disclose

### RPS 903-8

**Cardiothoracic ratio derived from cardiac MRI: how does it correlate with chest radiographs?**

*P. Simkus*, A. Banišauskaitė, J. Noreikaite, M. Gutierrez Gimeno, D. McCreavy, D. Penha, J. Holemans, M. Arzanauskaite; Liverpool/UK

**Purpose:** To determine the association between cardiothoracic ratio (CTR) from posteroanterior chest radiography (CXR) and cardiovascular magnetic resonance (CMR) imaging
**Methods or Background:** CTR is a well-known criterion to evaluate the heart on CXR; CTR>50% is considered abnormal. CMR can provide a thorough assessment of the heart, and a simple CTR. Studies of 305 patients who had a CXR and a CMR within 1 month were retrospectively analysed. CTR was obtained on CMR axial sequences and compared to the CTR of CXR. The calculation was the same in both techniques: the largest horizontal heart diameter was divided by the largest horizontal internal thoracic cage diameter.
**Results or Findings:** The mean CTR was lower on CXR than on CMR (49.47±5,07% and 55.36±5.47%, respectively, p<0.001). In 279 cases (91.5%) the CTR measured on CMR was larger than the one measured on CXR. CTR on CXR was 37% to 65%, vs. 41% to 72% on CMR. There was a strong positive correlation between CTR comparing the two modalities (ρ=0.665, p<0.001). Using the standard cut-off value of 50%, the CXR-CTR was normal in 191 cases (62.6%) and increased in 114 cases (37.4%). CMR-CTR was normal in 49 (16.1%) and increased in 256 cases (83.9%). Abnormal CTR>50% only weakly correlated on CXR and CTR (statistically significant), φ=0.283, p<0.05. An additional cut-off value of CTR>55% on CMR showed a better, but still weak correlation (φ=0.503, p<0.05).
**Conclusion:** Although CTR values were significantly higher on CMR compared to CXR there was a strong correlation between the techniques. However, abnormal CXR-CTR correlated weakly with abnormal CMR-CTR.
**Limitations:** A single centre study; CMR was acquired in supine position and in expiration; posteroanterior CXR in erect position and inspiration.
**Ethics Committee Approval:** IRB approval obtained
**Funding for this study:** Funded by the ESR Research Seed Grant
**Author Disclosures:**
Monika Arzanauskaite: Nothing to disclose
Jurate Noreikaite: Nothing to disclose
Diana Penha: Nothing to disclose
Manuel Gutierrez Gimeno: Nothing to disclose
John Holemans: Nothing to disclose
Audra Banišauskaitė: Nothing to disclose
Paulius Simkus: Nothing to disclose
David McCreavy: Nothing to disclose

### Research Presentation Session: Chest

### RPS 304

### Chest CT

### RPS 304-1

**Computational fluid dynamics in predicting progressive aortic dilatation following type A aortic surgical dissection**

*Y. Zhu*, X. Y. Xu, G. Asimakopoulos, A. Gambaro, R. Ulrich, J. Pepper, S. Mirsadraee; London/UK
*(yu.zhu14@ic.ac.uk)*

**Purpose:** This study aimed to examine the role of hemodynamic parameters, such as true/false lumen pressure difference and wall shear stress, in progressive aortic dilatation following type A aortic dissection (TAAD) surgical repair.
**Methods or Background:** This retrospective study is based on the validated database of patients with repaired TAAD at Royal Brompton and Harefield hospitals. All patients were initially treated by replacing the ascending aorta alone. 40 patients underwent a minimum of 2 follow-up computed tomography angiography (CTA) following surgical repair were reviewed, whereas 23 were deemed unsuitable for computational fluid dynamic (CFD) simulations due to inadequate CT image quality or coverage. A total of 17 patients were finally analysed and divided into two groups: stable (N = 9), and unstable (N = 8). Based on post-surgery CTA scans, three-dimensional patient-specific models were reconstructed for detailed CFD analysis. Geometric and hemodynamic parameters were evaluated and compared between the two groups using Mann-Whitney U test.
**Results or Findings:** Patients with progressive aortic dilatations were found to have significantly higher luminal pressure difference, as expressed by the maximum pressure differences (0.9 [0.5, 2.3] vs 6.7[4.6, 10.9] mmHg; P=0.001), as well as fewer number of re-entry tears (5[3.3, 7.5] vs 1.5[1, 2.8]; P = 0.02).
**Conclusion:** CFD is a promising technique based on standard care clinical imaging that may assist in predicting the risk of progressive aortic dilatation following TAAD repair. Further large cohort studies are warranted to validate these findings.
**Limitations:** Small sample size and rigid wall assumption of CFD simulations.
**Ethics Committee Approval:** Ethic approval was obtained on 04/05/2020 from the Institutional Committee of Health Research Authority (HRA) and Health and Care Research Wales (HCRW), with a REC reference of 20/WM/0145.
**Funding for this study:** n/a
**Author Disclosures:**
Yu Zhu: Nothing to disclose
Xiao Yun Xu: Nothing to disclose
Saeed Mirsadraee: Nothing to disclose
Alessia Gambaro: Nothing to disclose
John Pepper: Nothing to disclose
Rosendahl Ulrich: Nothing to disclose
George Asimakopoulos: Nothing to disclose

### RPS 304-2

**Dual energy computerised tomography pulmonary angiography (DECTPA) quantifies lobar iodine distribution in patients with severe emphysema**

*N. Jeyin*, S. P. Padley, S. R. Desai, K. Wechalekar, M. Polkey, N. Hopkinson, P. Shah, S. Kemp, C. A. Ridge; London/UK
*(nidhishj98@gmail.com)*

**Purpose:** To compare DECTPA lobar enhancement in patients with severe emphysema to perfusion scintigraphy (PS).
**Methods or Background:** Lung volume reduction (LVR) improves lung function in patients with severe emphysema. Patient selection is key to its success and is dependent on accurate quantification of (1) disease distribution using non contrast computed tomography (CT) and (2) lobar perfusion using the gold standard, single photon emission CT perfusion scintigraphy (SPECT-PS). Dual energy CT pulmonary angiography (DECTPA) is hypothesised to provide a surrogate marker for lobar perfusion, potentially streamlining the assessment paradigm. Between 2018-2020, 152 patients (89 male, 64.5 ± 8.6 years) with emphysema underwent DECTPA. Perfused blood volume (PBV) segmentation estimated lobar enhancement (eXamine, Siemens Healthineers). Pearson correlation and Bland-Altman analysis provided inter-modality comparison with lobar segmented SPECT-PS using Prism8, GraphPad. Effective dose, adverse reactions, and processing times were recorded.
**Results or Findings:** There is very strong agreement between lobar perfusion using DECTPA compared to SPECT-PS (r= 0.86, p<0.01). Individual lobar comparison also showed strong correlation (r= 0.81, 0.79, 0.83, 0.81, 0.81 for the right upper, middle, lower, left upper and lower lobes, respectively, p<0.01). Mean bias was 0.044; LoA = -11.667, 11.75. 123 of 152 DECTPA(81%) did not require manual correction, taking 113±3 seconds to process. DECTPA manual segmentation took 528±56 seconds in 18 patients with anatomic distortion.
**Conclusion:** Lobar segmentation of DECTPA data provides a novel "one stop" assessment of pulmonary lobar perfusion.
**Limitations:** Manual segmentation considerably increases DECTPA processing time; a potential barrier to implementation into clinical practice.
**Ethics Committee Approval:** Institutional review board approval was obtained for this retrospective study which waived the need for consent.
**Funding for this study:** The authors are thankful to the Royal Brompton and Harefield Hospitals Charity for financial support in the purchase of dual energy advanced processing software.
**Author Disclosures:**
Nidhish Jeyin: Nothing to disclose
Carole A Ridge: Nothing to disclose
Pallav Shah: Nothing to disclose
Michael Polkey: Nothing to disclose
Kshama Wechalekar: Nothing to disclose
Samuel Kemp: Nothing to disclose
Nicholas Hopkinson: Nothing to disclose
Sujal R. Desai: Nothing to disclose
Simon Peter Padley: Nothing to disclose

### RPS 304-3

**Biphasic split-bolus protocol for routine contrast-enhanced chest CT imaging: a comparison with a conventional early phase-single bolus technique**

*M. Kul*[1], D. Kuru Öz[1], A. Gürsoy Çoruh[1], F. Özalp Ateş[1], B. Gülpınar[1], Ç. Uzun[1], K. Ç. K. Atasoy[2]; [1]Ankara/TR, [2]Istanbul/TR
*(melahatkul@yahoo.com)*

**Purpose:** To present our routine contrast-enhanced chest CT protocol with a split-bolus injection technique achieving combined early-and delayed phase images with a single aquisition, and to compare this technique with a conventional early-phase single-bolus chest CT protocol we formerly used at our institution, in terms of attenuation of great thoracic vessels, pleura, included upper abdominal structures, contrast-related artifacts, and image quality.
**Methods or Background:** A total of 210 patients, who underwent routine contrast-enhanced chest CT examination aquired with either conventional early-phase single-bolus technique (group A,n=102) or biphasic split-bolus protocol (group B,n=108) were retrospectively included. Attenuation measurements were made by two radiologists independently on mediastinal window settings using a circular ROI at the following sites: main pulmonary artery(PA) at its bifurcation level, thoracal aorta(TA) at the level of MPA bifurcation, portal vein(PV) at porta hepatis, coeliac trunk, left and right hepatic lobe, and if present, thickened pleura (>2mm) at the level with the most intense enhancement. Contrast-related artifacts were graded by both reviewers independently. Background noise was measured and CNRs of the liver and TA were calculated.
**Results or Findings:** While mean enhancement of thoracic vessels and coeliac trunk did not differ significantly between both groups (p >0.05), mean enhancement of pleura, liver parenchyma and PV were significantly greater in group B for both observers (p<0.001). Perivenous artifacts limiting evaluation were less frequent in group B than in A. No significant difference was detected in CNRTA, whereas CNRliver was higher in group B (p<0.05).
**Conclusion:** Split-bolus injection protocol facilitates simultaneous attenuation of early and delayed enhancing thoracal and upper abdominal structures in a single chest CT aquisition, and thus, might raise diagnostic confidency.
**Limitations:** First, this is a retrospective study. Second, cardiac output-body weight of the patients, which can influence contrast enhancement, were not assessed.
**Ethics Committee Approval:** Approved.
**Funding for this study:** None.
**Author Disclosures:**
Melahat Kul: Nothing to disclose
Başak Gülpınar: Nothing to disclose
Diğdem Kuru Öz: Nothing to disclose
Funda Özalp Ateş: Nothing to disclose
Kayhan Çetin K. Atasoy: Nothing to disclose
Ayşegül Gürsoy Çoruh: Nothing to disclose
Çağlar Uzun: Nothing to disclose

### RPS 304-4

**Contrast-enhanced MRI for lung nodule detection and characterization in a high-risk population: a comparison with low-dose CT**

*L. Qian*, M. O. Wielpütz, B. Jobst, O. v. Stackelberg, H-U. Kauczor, J. Biederer; Heidelberg/DE
*(lipb37@163.com)*

**Purpose:** To investigate contrast enhanced MRI (CE-MRI) for the detection and characterization of lung nodules compared with low-dose CT (LDCT) in a high-risk population.
**Methods or Background:** 177 patients with baseline LDCT scan from a large multicenter COPD study (COSYCONET) who had at least 1 pulmonary nodule were included. All subjects underwent CE-MRI and LDCT on the same day. Nodule incidence, long and short axis, location and characteristics (solid, part-solid or ground glass) and Lung-RADS category were assessed on CT and MRI separately and in a blinded fashion. MRI findings were correlated with LDCT as standard of reference.

**Results or Findings:** A total of 510 nodules were detected by LDCT, of which were 479 solid, 10 part-solid and 21 purely GGO nodules. For solid nodules, MRI detected 152 of 292 nodules 3-5mm, 74 of 100 nodules 6-7mm, 52 of 60 nodules 8-14mm, and 24 of 27 solid nodules ≥15mm. For sub-solid nodules, MRI detected 5 of 10 part-solid nodules and 2 of 21 GGO nodules. Sensitivity and positive predictive value (PPV%) of MRI for solid nodules detection was 52.1/75.2% for 3-5mm, 74.0/91.3% for 6-7mm, 86.7/89.7% for 8-14mm and 88.9/100% for ≥15mm. Among sub-solid nodules, sensitivity and PPV% of MRI detection was 50.0/83.3% for part-solid nodules and 9.5/100% for GGO respectively.
**Conclusion:** CE-MRI performs moderately well for the detection of small pulmonary nodules. Detection rate was acceptable for nodules 8 mm and larger, which is the size to trigger further workup.
**Limitations:** The benign and malignant classification of pulmonary nodules has not been characterized by surgery/biopsy or follow-up.
**Ethics Committee Approval:** This study was approved by the ethics committee of the coordinating centre in Heidelberg (Institutional Review Board of the Medical Faculty of the University of Heidelberg, Germany, S-656/2012)
**Funding for this study:** COSYCONET, Clinicaltrials.gov identifier NCT01245933
**Author Disclosures:**
Mark O. Wielpütz: Nothing to disclose
Jürgen Biederer: Nothing to disclose
Hans-Ulrich Kauczor: Nothing to disclose
Oyunbileg von Stackelberg: Nothing to disclose
Li Qian: Nothing to disclose
Bertram Jobst: nothing to disclose

### RPS 304-5

**Visual and quantitative CT airway assessment in phenotyping severe asthma for precision therapy**
*E. Robinson*[1], S. M. Mak[2], B. Kent[2], D. Jackson[2], A. Nair[2]; [1]Bristol/UK, [2]London/UK

**Purpose:** To identify visual and quantitative (qCT) differences in severe asthmatics (SevA) eligible for standard optimised (ST) versus biologic (BT) therapies Assess relationships between obstructive lung function and visual/qCT airway remodelling.
**Methods or Background:** 93 SevA CTs were retrospectively visually scored for per lobe airway remodelling features, including wall thickening (vWT). qCT software was used to measure wall thickness (WT), mean diameter (MD), wall area (WA), luminal area (LA) and total area (TA). Differences between groups, and correlations between physiologic parameters and CT metrics, were analysed.
**Results or Findings:** Proximal mucus impaction was lower in patients on ST (p=0.01). vWT and low attenuation areas were not significantly different between ST and BT. Conversely, of qCT metrics, MD (p=0.03) and TA (p=0.03) were significantly greater in BT, while WT, WA and LA were not. Both vWT (r=-0.27) and LAA (r=-0.34) were inversely correlated with FEV1, while vWT (r=0.41) inversely correlated with FEV1/FVC. No quantitative measures correlated with obstructive indices.
**Conclusion:** In SevA, visual CT evaluation of airway inflammation and small airways obstruction provides a proxy for severity of obstruction. qCT total airway dimensions potentially identify those with greater airway inflammation who may require BT.
**Limitations:** Sample size- limited population as the use of biological agents are uncommon, however our sample size is in line with others in the literature. Selection bias- those with abnormalities seen on other modalities e.g. X-ray or bloods more likely to have HRCT therefore more severe disease Cross-sectional- transient changes especially consolidations not captured and can give no longitudinal data Subjective identification of abnormality on CT scan according to previous definition
**Ethics Committee Approval:** HRA review of governance and legal compliance (18/HRA/1611)
**Funding for this study:** None
**Author Disclosures:**
Arjun Nair: Nothing to disclose
Sze Mun Mak: Nothing to disclose
Elizabeth Robinson: Nothing to disclose
Brian Kent: Nothing to disclose
David Jackson: Nothing to disclose

### Research Presentation Session: Chest

### RPS 404

### Tissue characterisation and image verification

### RPS 404-1

**CT fluoroscopy for hook-wire localisation of pulmonary nodules before thoracoscopic surgery: retrospective analysis of efficacy and safety**
*C. Sicuso*, G. Vatteroni, E. Lanza, D. Poretti, F. Lutman, F. D'Antuono; Rozzano/IT
*(claudioesse@hotmail.it)*

**Purpose:** To evaluate the efficacy and safety of CT fluoroscopy-guided hook-wire localization before thoracoscopic surgery and to investigate the risk factors associated with dislocation.
**Methods or Background:** Between June 2016 and October 2019, 94 patients (56F, 38M) with small non-palpable lung nodules (mean diameter:11±5mm; range: 5-33) underwent preoperative percutaneous localization by CT fluoroscopy-guided hook-wire system (MOON, Biopsybell), followed by thoracoscopic surgery. Clinical data, radiological characteristics, procedural and dosimetric (estimated as dose length product - DLP) features and pathological findings were evaluated. Clinical and procedure-related variables were investigated to determine the risk factors for dislocation.
**Results or Findings:** Out of 94 nodules, 5 (5.3%) were pure ground-glass (GGOs), 23 (24.2%) partially solid GGOs, and 66 (69.5%) solid lesions. The mean length of the procedure was 6.5±2.5 min (range: 3-13), with a mean distance from the pleural surface of 5.7±6.2 mm (range: 0-30). Median DLP including pre and post helical CT scan to identify the nodule and to check for the hook-wire position was 565 mGy*cm (range: 212-2017) while median procedural DLP was 40 mGy*cm (range: 8-1094). At the end of the procedure 12 (12.7%) asymptomatic pneumothorax, 3 (3.2%) mild parenchymal hemorrhage and 2 (2%) early wire dislodgement were observed, while late dislodgement during surgery was found in 11 (12%) cases. Thoracoscopic surgery was performed in 91 cases (97%), while in 3 cases conversion to a thoracotomy approach was required. Pathologic evaluation revealed 37 (39%) primary lung cancers, 38 (40%) metastases, 18 (19%) benign lesions and 1 (1%) lung meningioma.
**Conclusion:** Hook-wire localization is a fast, low-dose procedure that enables lung nodule resection using thoracoscopic approach with a high percentage of success and acceptable minor complication rate.
**Limitations:** n/a
**Ethics Committee Approval:** n/a
**Funding for this study:** n/a
**Author Disclosures:**
Felice D'Antuono: Nothing to disclose
Fabio Lutman: Nothing to disclose
Ezio Lanza: Nothing to disclose
Giulia Vatteroni: Nothing to disclose
Dario Poretti: Nothing to disclose
Claudio Sicuso: Nothing to disclose

### RPS 404-3

**Heterogeneity of structural changes in bronchiectasis patients assessed using the bronchiectasis scoring technique for CT (BEST-CT)**
J. Meerburg[1], M. De Bruijne[1], A. Garcia-Uceda Juarez[1], E-R. Andrinopoulou[1], M. Kemner van de Corput[1], S. Elborn[2], M. Tunney[2], J. Chalmers[3], *H. A. W. M. Tiddens*[1]; [1]Rotterdam/NL, [2]Belfast/UK, [3]Dundee/UK
*(h.tiddens@erasmusmc.nl)*

**Purpose:** To develop a sensitive quantitative CT scoring method to phenotype and quantify structural lung changes in bronchiectasis (BE) patients
**Methods or Background:** Recent CT-scans of BE patients with P.aeruginosa enrolled in the iBEST study were collected. Inclusion criteria: inspiratory volumetric CT-scans and slice thickness ≤3mm. Grid cells were annotated on 10 axial slices per CT, scores expressed in % total lung volume. Scoring items in hierarchical order: consolidation/atelectasis (ATCON), BE with mucus plugging (BEMP), BE without MP (BE), airway wall thickening (AWT), mucus plugging (MP), ground-glass opacities (GGO), emphysema/bullae (EMBUL), healthy airways (HA), and healthy parenchyma (HP). Composite scores: %TBE (%BEMP+%BE), airway disease (%AD) (%TBE+%MP+%AWT) and %DIS (all but %HA and %HP) were calculated. ICCs were assessed. BEST-CT scores were compared with the Hartmann-CT scores, intra-branch tapering, spirometry parameters, bronchiectasis severity index (BSI), quality of life (QoL), and exacerbations during the iBEST study. Results: median (interquartile range).

**Results or Findings:** 84 CTs were included. Age 66 [58-74] years, female 64%, FEV1 59[46-70]%Pred, FVC 72[64-88]%Pred, BSI 10[7-13]. BEST-CT subscores: %TBE 3.0[1.4-5.1], %AD 6.5[3.4-11.8], and %DIS 9.4[6.0-17.7]. Intra-airway tapering was assessed 262[178-326] airways/CT. Intra- and inter-ICCs for %TBE, %AD and %DIS were excellent. Correlations between %TBE and Hartmann BE-score, (r=0.69, p<0.001) and FEV1 (r=-0.24, p=0.027). Less tapering correlated with higher %TBE score (r=-0.04, p=0.002), higher Hartmann BE-score (r=-0.29, p=0.009), and lower FEV1 (r=-0.36, p<0.001). No significant correlations between %TBE, %TMP, %AD and %DIS and BSI, QoL scores (n=55), and exacerbations.
**Conclusion:** BEST-CT is a reproducible CT scoring method to quantify and phenotype BE patients. Considerable heterogeneity of structural changes was observed.
**Limitations:** Retrospective data collection, CT acquisition not standardised
**Ethics Committee Approval:** Yes
**Funding for this study:** The Innovative Medicines Initiative (IMI) and EFPIA companies under the European Commission funded project, iABC (grant 115721)
**Author Disclosures:**
Stuart Elborn: Nothing to disclose
Jennifer Meerburg: Nothing to disclose
Antonio Garcia-Uceda Juarez: Nothing to disclose
Eleni-Rosalina Andrinopoulou: Nothing to disclose
Michael Tunney: Nothing to disclose
James Chalmers: Other: J.D. Chalmers reports research grants for CO and personal fees from GlaxoSmithKline and Boehringer Ingelheim, research grants for CO from AstraZeneca, research grants for CO and personal fees from Pfizer, research grants for bronchiectasis and personal fees from Bayer Healthcare and Grifols, consulting fees from Napp and the Aradigm Corporation, and grants and personal fees from Insmed, outside the submitted work.
Mariëtte Kemner van de Corput: Nothing to disclose
Harm A W M Tiddens: Research/Grant Support: Tiddens has received unconditional research grants from Gilead Sciences Inc, Chiesi pharmaceuticals, Novartis, Vertex, and Vectura, and honoraria and travel expenses for lectures and participation in expert panels from Novartis, Gilead, and Vertex Patent Holder: Tiddens owns a joint patent with Perth for PRAGMA-CF
Marleen De Bruijne: Other: de Bruijne reports grants from Netherlands Organisation for Scientific Research (NWO), during the conduct of the study; grants and non-financial support from Quantib, grants and non-financial support from Cosmonio, outside the submitted work

### RPS 404-4

**Hystogram based analysis in progressive phenotype pattern: is early phenotype of progression dependent by HRCT quantification?**

*F. Tiralongo*, F. Galioto, S. Toscano, A. Vancheri, G. Sambataro, L. A. Mauro, C. Vancheri, S. Palmucci, A. Basile; Catania/IT
*(tiralongofrancesco91@hotmail.it)*

**Purpose:** To investigate relationship between histogram-based HRCT indexes and Pulmonary Function Tests (PFTs) in Interstitial Lung Diseases (ILD) patients having progressive and non-progressive phenotype.
**Methods or Background:** 28 patients having two HRCT (baseline and 1 year) and two PFTs were retrospectively investigated. Quantitative histogram-based HRCT indexes were calculated; strength of association between HRCT indexes and PFTs was investigated using Pearson correlation. Patients were divided into two groups, based on the presence or absence of progressively fibrosing phenotype – which was assessed on the basis of: relative FVC decline >10%, or relative 5-10% FVC decline and increased HRCT fibrosis. HRCT indexes were compared between the two groups using the U-test; within each group, Wilcoxon analysis was performed for comparison between baseline and follow-up HRCT indexes. ROC analysis was performed for predicting the presence of the progressively fibrosing phenotype.
**Results or Findings:** At baseline, moderate correlation was found – with r=0,55 for FVC and kurtosis and r=0,45 for DLco and kurtosis. Positive moderate correlations were observed considering skewness and PFTs, whereas negative moderate correlations were reported between HAA% and PFTs. We found more heterogenous results between quantitative indexes and PFTs collected at one-year follow-up, with r values ranging from 0.64 up to 0.32; in progressive subgroup, r was 0.38 for DLCO and kurtosis, and 0.43 for FVC and kurtosis. HRCT indexes were not statistically different between the 2 groups – with a p of 0.40 (baseline) and 0.48 (follow-up) for kurtosis; within each subgroup, Wilcoxon analysis did not report significant differences for quantitative indexes. ROC analysis reported a sensitivity of 54.5% and a specificity of 75%.
**Conclusion:** Heterogeneity of correlation found at 1-years seems to suggest that progression could be independent from quantitative HRCT assessment.
**Limitations:** n/a
**Ethics Committee Approval:** n/a
**Funding for this study:** n/a
**Author Disclosures:**
Federica Galioto: Nothing to disclose
Letizia Antonella Mauro: Nothing to disclose
Antonio Basile: Nothing to disclose
Stefano Toscano: Nothing to disclose
Gianluca Sambataro: Nothing to disclose
Ada Vancheri: Nothing to disclose
Stefano Palmucci: Nothing to disclose
Francesco Tiralongo: Nothing to disclose
Carlo Vancheri: Nothing to disclose

### RPS 404-5

**Evaluation of radiological IPF distribution in males and females: a preliminary evaluation**

A. Vancheri, *F. Tiralongo*, M. Palermo, F. Galioto, G. Fazio, G. Vignigni, G. Sambataro, S. Palmucci, A. Basile; Catania/IT
*(tiralongofrancesco91@hotmail.it)*

**Purpose:** To investigate differences of radiological pattern distribution in male and female IPF patients.
**Methods or Background:** 102 IPF patients having HRCT scan were investigated: for their enrollment, Pulmonary Function Tests (PFTs) acquired nearest to the HRCT examinations, GAP and stage disease were achieved. Quantitative histogram-based analysis was performed providing HRCT indexes – which included kurtosis, skewness, High Attenuation Areas (HAA% -250, HAA% -600), Mean Lung Density (MLD) and volume. Indexes were obtained for: whole lung, right lung, left lung, and for upper, middle and basal pulmonary regions. HRCT indexes were compared between males (n=56) and females (n=46) using the Mann-Whitney test; comparisons were also performed after adjustment for stage of disease.
**Results or Findings:** In all stages – whole lung volume, left and right lung volumes, and volumes of upper, middle and lower portion of each lung – were lower in female patients (median of 2.5 in females, 3.7 in males, p<0.0001); a statistical difference was found considering MLD values of the left middle portion (p=0.05). No others differences were found. At stage I – the whole lung HAA% -250 values, the left and right lung HAA% -250 values, the left and right basal, middle and upper portion HAA% -250 values were higher in females (p ranging from 0.007 to 0.05); the left middle region MLD value in females was lower (p=0.04). In right basal region, female kurtosis was higher (1.1 vs 0.3, p=0.02); also female skewness was higher (1.2 vs 1, p=0.06). At stage II-III, HRCT indexes didn't show statistical differences.
**Conclusion:** In females, disease seems to be more homogeneously distributed; at stage I, lower kurtosis values of right basal lung were found in males, probably due to higher respiration movements.
**Limitations:** n/a
**Ethics Committee Approval:** n/a
**Funding for this study:** n/a
**Author Disclosures:**
Giovanna Vignigni: Nothing to disclose
Federica Galioto: Nothing to disclose
Monica Palermo: Nothing to disclose
Antonio Basile: Nothing to disclose
Giulia Fazio: Nothing to disclose
Gianluca Sambataro: Nothing to disclose
Ada Vancheri: Nothing to disclose
Stefano Palmucci: Nothing to disclose
Francesco Tiralongo: Nothing to disclose

### RPS 404-6

**Bayesian network approach to diagnose subtypes of interstitial lung disease**

*W. D. Lindsay*, J. Gee, W. T. Miller; Philadelphia, PA/US
*(lindsay@seas.upenn.edu)*

**Purpose:** Obtaining a specific, timely diagnosis of Interstitial Lung Disease (ILD) is a challenging clinical problem, with the majority of ILD patients being misdiagnosed at least once. The purpose of our study was to train a machine learning model to accurately diagnose subtypes of ILD using radiographic features extracted by radiologists from High Resolution Computed Tomography (HRCT) scans of the chest.
**Methods or Background:** Data from 533 patients diagnosed with ILD at our institution were used to train four types of Bayesian network diagnosis models based on 13 key imaging features extracted from HRCT scans of the chest. A training set of 70% of our total dataset was used during model development, reserving 30% of the data for testing. General and chest radiologists were asked to list their top three differential diagnoses for a subset of cases in the dataset. Machine learning models were compared against both groups of radiologists using top1 accuracy and top3 accuracy.

**Results or Findings:** The Naive Bayes diagnosis model performed best both in terms of top1 accuracy (60.2%) and top3 accuracy (90.1%), exceeding the performance of both general radiologists (top1 accuracy of 30.5%, top3 accuracy of 48.1%) and chest radiologists (top1 accuracy of 43.2% and top3 of 60.5%) when evaluated on the 30% test set.
**Conclusion:** In ILD, where specific diagnosis is challenging, our machine learning based diagnosis engine combined with radiologist extracted features outperformed radiologists alone in terms of top1 and top3 diagnosis accuracy. With further validation, we hope this model may help contribute to faster, more accurate diagnosis for patients suffering from ILD.
**Limitations:** Single institution dataset, requires manual feature extraction, limited sample size
**Ethics Committee Approval:** Approved by institutional review board, performed in compliance with patient privacy laws
**Funding for this study:** None
**Author Disclosures:**
William David Lindsay: Nothing to disclose
James Gee: Nothing to disclose
Wallace Thomas Miller: Nothing to disclose

### Research Presentation Session: Chest

### RPS 504

### Diagnosing conditions of the chest

### RPS 504-1

**Prenatal MRI evaluation of congenital diaphragmatic hernia**
*H. Morkovkina*, R. Tammo, T. Yalynska, O. Halchenko; Kiev/UA
*(morkovkina@ukr.net)*

**Purpose:** Congenital diaphragmatic hernia (CDH) is one of the most common non-cardiac fetal intrathoracic anomalies. Morbidity and mortality are high due to pulmonary hypoplasia with which it is associated.
**Methods or Background:** This study included 68 fetal MRI scans of CDH which underwent at the Ukrainian Children Cardiac Center during the period 1 January 2014 to November 2020. The fetuses were 19-38 weeks gestational age (mean 30,3 weeks). MRI was performed in a 1.5-Tesla magnet using steady-state free precession, T2- and T1-weighted sequences.
**Results or Findings:** MRI diagnosed left CDH (58), right (9), and bilateral CDH (1) and most of them were agreed with the postnatal diagnosis. Eight newborns died a few hours after birth. The following MRI parameters were measured: total chest volume, mediastinal volume, expected lung volume, predicted lung volume, percentage of liver herniated into the fetal thorax (liver up/down; percentage of herniated liver volume). Between 8% and 45 % of the predicted lung volume were identified (mean 25%). MRI measured lung ratio has a higher prognostic accuracy than with ultrasound. The fetal liver was easily demonstrated with MRI in all fetuses and was herniated into the chest in 25 of the 68.
**Conclusion:** Fetal MR imaging has proved valuable for anatomic assessment, determining the position of the liver and visualizing lung tissue in fetuses who have CDH, calculation of lung volume and liver herniation measurements. These findings predict are important for prenatal counseling, estimating prognosis and perinatal planning, including the need for ECMO.
**Limitations:** Contraindications for a pregnant woman: the presence in the body of a metal, hearing aid, pacemaker or other electronic implants, epilepsy or claustrophobia, as well as the first trimester of pregnancy.
**Ethics Committee Approval:** Done
**Funding for this study:** There was no funding from industry and/or a funding agency
**Author Disclosures:**
Tetyana Yalynska: Nothing to disclose
Hanna Morkovkina: Nothing to disclose
Raad Tammo: Nothing to disclose
OleksanHalchenko: Nothing to disclose

### RPS 504-2

**Diagnostic value of initial chest CT findings for the need of ICU treatment/intubation in patients with COVID-19**
L. Büttner, A. Aigner, F. N. Fleckenstein, C. M. Hamper, M. Jonczyk, B. Hamm, O. Scholz, *G. Böning*; Berlin/DE

**Purpose:** Computed tomography (CT) is used for diagnosing COVID-19 and there is evidence that deep learning or volumetry can help detecting patients in need of intensive care unit (ICU) or ventilation therapy. However, those tools are not available in all hospitals. The aim of this study was to investigate whether a semi-quantitative method for measuring pulmonary involvement in CT scans could contribute to allow early identification of COVID-19 patients likely to require ICU treatment and intubation.
**Methods or Background:** The initial CT scans of 28 patients tested positive for SARS-CoV-2 during the early phase of the pandemic at our level I center were retrospectively analyzed. The extent of lung involvement on CT was classified both subjectively and with a simple semi-quantitative method measuring the affected area at three standardized levels. Competing risks Cox regression was used to identify factors associated with the time to ICU admission and intubation. Their potential diagnostic ability was assessed with ROC/AUC analysis.
**Results or Findings:** A 10% increase in the affected lung parenchyma area increased the instantaneous risk of intubation (HR=2.00) and the instantaneous risk of ICU admission (HR 1.73). The semi-quantitative measurement outperformed the subjective assessment diagnostic ability (AUC=85.6% for ICU treatment, 71.9% for intubation).
**Conclusion:** A simple measurement of the affected lung area in initial CT scans of COVID-19 patients may allow the early identification of patients likely to need ICU treatment/intubation and thus help make optimal use of limited resources in hospitals.
**Limitations:** The study is limited by a small sample size, a short inclusion period and the exclusive use of initial CT scans.
**Ethics Committee Approval:** This retrospective study was approved by the Local Ethics Committee of our hospital.
**Funding for this study:** This research received no external funding.
**Author Disclosures:**
Christina Maria Hamper: Nothing to disclose
Bernd Hamm: Nothing to disclose
Annette Aigner: Nothing to disclose
Martin Jonczyk: Nothing to disclose
Oriane Scholz: Nothing to disclose
Laura Büttner: Nothing to disclose
Georg Böning: Nothing to disclose
Florian Nima Fleckenstein: Nothing to disclose

### RPS 504-3

**Diagnostic value of dual-energy chest x-ray in immunocompromised patients to rule out pneumonia: initial results**
*F. Sanchez Tijmes*, S. Kandel, M. May, S. Ronghe, P. Rogalla; Toronto, ON/CA
*(fsancheztijmes@gmail.com)*

**Purpose:** To evaluate the diagnostic value of a novel single exposure portable multilayer detector (KA-Imaging) for generating dual-energy X-rays of the chest for the detection of pneumonia in immunocompromised patients.
**Methods or Background:** With ethics board approval, immunocompromised patients (leukemia, HSCT) received both the standard-of-care (SOC) low-dose CT and a dual-energy X-ray (DEX) of the chest in two views (pa, lateral), back-to-back. The SOC served as the reference standard. Three images were reconstructed from the DEX data: conventional X-ray (CR), bone-only and soft-tissue image (DE). Two experienced chest radiologists reviewed the conventional X-rays first and were then provided with the two DE images. The readers reported opacities, the size and location, pneumonia (yes, no, unsure), and the confidence level for the correct diagnosis (1=unsure, 5=confident). After unblinding the DE images, readers indicated the change in diagnosis (if present) and their confidence level (same scale). The subjective DE image quality was rated as well (1=poor, 5=best). The radiation dose for DEX was recorded.
**Results or Findings:** The first 33 patients of an ongoing trial were included. For reader1/2 and CR versus CR+DE, the sensitivity, specificity, accuracy, and area-under-the-curve (ROC analysis) were 57.9.5%/68.4% versus 63.2%/73.7%, 78.6%/85.7% versus 85.7%/92.9%, 66.7%/75.8% versus 72.7%/81.8%, 0.61/0.77 versus 0.72/0.76, respectively. The mean image quality was 3.7 and 3.4 for readers 1 and 2, respectively. The average DAP (mGy*cm) was 296 for pa and 555 for the lateral view.
**Conclusion:** The initial evaluation suggests that dual-energy X-ray provides adequate clinical image quality on conventional reconstructions; the additional bone and soft-tissue images improve the diagnostic accuracy; however, more data are required to determine the robustness of this novel technology in clinical practice.
**Limitations:** Sample size, DEX images reconstructed off-line.
**Ethics Committee Approval:** Ethics board approval.
**Funding for this study:** Funded by Ontario Bioscience Innovation Organization.
**Author Disclosures:**
Felipe Sanchez Tijmes: Nothing to disclose
Mary May: Nothing to disclose
Sonja Kandel: Nothing to disclose
Patrik Rogalla: Nothing to disclose
Sandeep Ronghe: Nothing to disclose

### RPS 504-4

**Diagnosis and quantification of chronic thromboembolic pulmonary hypertension using gated spectral CT imaging - assessment of accuracy in 10 consecutive patients**

*P. A. Ball*; Belfast/UK
*(peterandrewball@me.com)*

**Purpose:** Assess the accuracy and reproducibility of a one stop imaging modality for Chronic Thromboembolic Pulmonary Hypertension (CTEPH) using a low dose ECG gated Spectral CT imaging
**Methods or Background:** 10 consecutive patients referred for investigation of CTEPH were scanned using a modified dose modulated retrospective gated CTPA protocol. The images were reviewed both for perfusion using Iodine mapping and RV function using thick slice reconstructions from the lose dose modulated studies. Lung findings and measurements of RVOT and PA ratios were also reviewed. Findings were compared with complementary imaging including ECHO, V/Q and MR findings
**Results or Findings:** There was a high level of agreement between the between the Iodine perfusion on CT and V/Q. The CT had the added information of vessel luminal assessment (filling defects, recanalisation etc) and vessel size quantification. There was also a high level of agreement between the ECHO measurements and the functional reconstructions on the gated CT of Ejection fraction, wall thickness and RV ejection fraction on Bland Altman scatter plot. The DLP for the gated study was higher than the normal ungated CTPA however is similar when the normal CTPA exposure and V/Q dose are taken in combination
**Conclusion:** The pilot study suggests that a Spectral gated CTPA approach to investigation in CTEPH is a robust and potentially more accurate, cost effective method of investigation
**Limitations:** Small study at present
**Ethics Committee Approval:** None
**Funding for this study:** None
**Author Disclosures:**
Peter Andrew Ball: Speaker: Phillips Healthcare

### RPS 504-5

**Computed tomography pulmonary angiography for the diagnosis of pulmonary embolism: diagnostic accuracy of a reduced scan range**

*J. Schmid*, E. Nagy, J. Mandl, M. Janisch, A-K. Kaufmann-Bühler, M. Eibisberger, E. Janek, C. Reiter, N. Softic, H. Guss, G. Adelsmayr; Graz/AT

**Purpose:** Computed tomography pulmonary angiography (CT-PA) is frequently applied in the diagnostic workup of pulmonary embolism (PE), even in highly radiosensitive patient populations, such as children or pregnant women. Based on the direct proportional relationship between radiation dose and scan length, narrowing the scan range is one well-known approach to reduce radiation dose. This study investigates the sensitivity of CT-PA with a reduced scan range for the detection of PE, compared to a standard scan range covering the entire lung.
**Methods or Background:** We retrospectively analysed 529 CT-PA scans with suspected PE. A reduced scan range was defined based on the topogram, where the cranial slice was set at the upper edge of the aortic knob and the caudal slice at the level of the lower diaphragm. Apparently reduced intravascular contrast, which was likely explained by artefacts, was not considered as PE. CT scans without definite PE or with significant artefacts affecting arterial branches proximal to a segmental level were excluded.
**Results or Findings:** We included 454 CT-PA scans of 438 patients (mean age 63.4±17.4 years, 46.5% female) in our analysis. Median dose length product was 269.5[115.3-512.1]mGy*cm. Comparing the original and reduced scan range, mean scan length was significantly reduced by 48.1% (268±30 vs. 139±26mm, p<0.001). Single emboli outside the reduced range in addition to emboli within were found in 11 scans (2.4%), while only one scan had emboli exclusively outside the reduced range. The resulting sensitivity of CT-PA with reduced scan range was 99.78% (95% confidence interval: 98.58-99.99) for detecting any PE.
**Conclusion:** Reducing CT-PA scan range, as described above, is a simple and effective means to reduce radiation dose without a relevant loss of sensitivity for detecting PE.
**Limitations:** Retrospective design.
**Ethics Committee Approval:** Obtained.
**Funding for this study:** None.
**Author Disclosures:**
Ann-Katrin Kaufmann-Bühler: Nothing to disclose
Martin Eibisberger: Nothing to disclose
Jakob Mandl: Nothing to disclose
Helmuth Guss: Nothing to disclose
Clemens Reiter: Nothing to disclose
Michael Janisch: Nothing to disclose
Elmar Janek: Nothing to disclose
Eszter Nagy: Nothing to disclose
Nina Softic: Nothing to disclose
Gabriel Adelsmayr: Nothing to disclose
Johannes Schmid: Nothing to disclose

### Research Presentation Session: Chest

### RPS 604

### Cancers in the chest

### RPS 604-1

**Combining radiomics with domain-adversarial machine learning to predict EGFR mutation in non-small cell lung cancer patients on CT images with and without contrast enhancement**

*X. Yang*, P. Yu, M. Liu, H. Dai, C. Wang; Beijing/CN
*(yxy18161659153@126.com)*

**Purpose:** To develop and validate the method that combines domain-adversarial machine learning with radiomics signature extracted from both the enhanced and non-enhanced CT images in order to identify EGFR mutations.
**Methods or Background:** This study involved 393 patients with NSCLC who underwent molecular testing. The validation set included 73 patients with both the enhanced and non-enhanced CT scans. The remaining 320 patients were assigned to the training set (167 non-enhanced CTs and 153 enhanced CTs), Radiomics features were extracted through manual segmentation of the entire tumor. We combined such features with domain-adversarial method to build a multi-task multi-layer perceptron (MT-MLP) model in order to eliminate feature differences between enhanced and non-enhanced CTs. The optimization of MT-MLP model was supervised by two tasks, namely EGFR mutation prediction and CT type prediction. To compare, we also constructed a standard MLP model that only predicted EGFR mutations. ROC curve analysis and Delong test were used to compare the predictive performances of different models.
**Results or Findings:** A total of the 1015 radiomics features were selected. The AUCs of the MLP model in the validation set based on enhanced and non-enhanced CT radiomics features were 0.702 (CI: 0.642-0.776) and 0.648 (CI: 0.570-0.717). The corresponding results of the MT-MLP model were 0.764 (CI: 0.706-0.829) and 0.756 (CI: 0.695-0.826). Comparing to the MLP model, the performance of the MT-MLP model in predicting EGFR mutations was improved in different CT types, especially on non-enhanced CTs (p = 0.042).
**Conclusion:** Domain-adversarial machine learning combining with radiomics signature extracted from enhanced and non-enhanced CT images showed excellent performance for predicting EGFR mutant, which may become a helpful non-invasive biomarker to guide therapies.
**Limitations:** We performed a single center.
**Ethics Committee Approval:** This study has been proved by ethnic community
**Funding for this study:** No funding
**Author Disclosures:**
Pengxin Yu: Nothing to disclose
Chen Wang: Nothing to disclose
Min Liu: Nothing to disclose
Huaping Dai: Nothing to disclose
Xiaoyan Yang: Nothing to disclose

### RPS 604-2

**Quantitative measurements with computed tomography and pulmonary function test as predictors of surgical resectability in patients affected by pleural mesothelioma**

*A. Modugno*, A. Bellini, C. Giraudo, A. S. Fraia, R. Stramare, A. Dell'Amore, F. Rea; Padua/IT
*(modugno.a@libero.it)*

**Purpose:** To evaluate if pulmonary volumes and tumor thickness, measured with Computed Tomography (CT) and Total Lung Capacity (TLC%) can predict surgical resectability in patients with pleural mesothelioma.

**Methods or Background:** Patients affected by pleural mesothelioma referring to our tertiary center from 1994 to 2020, who underwent a pre-surgical CT with a slice thickness <3mm were included in this retrospective study. One radiologist expert in thoracic imaging measured tumor thickness according to the mRECIST criteria (i.e., up to six measurements of tumor thickness perpendicular to the chest wall/mediastinum) and computed pulmonary volumes using a semi-automatic method (3D Slicer Open Source Software). TLC% was also recorded and patients were classified according to the residual tumor. To assess any difference, the Mann-Whitney and the exact Fisher's test were used respectively for continuous and categorical variables. Multivariate analyses with odds ratio were applied to assess the predictive value of the examined variables (p<0.05 applied level of significance for each analysis).
**Results or Findings:** Fifty-eight patients with mesothelioma met the inclusion criteria (43 R1, mean age 64 years, 25 males; 22 were R2, mean age 63 years, 14 males). TLC% and tumor thicknes demonstrated to be independent predictors of surgical resectability (p=0.024; OR=0.917 and p=0.030; OR1.018) using as cut-offs < 77.5% and > 120.5 mm, respectively. Tumor's thicknesses showed a statistically significant difference between R1 (mean 57 mm, range 0-165; IQR 36-99) and R2 patients (mean 133 mm, range 34-241; IQR 70-181) (p=0.0012). No differences occurred comparing affected and healthy lung volumes (p>0.05).
**Conclusion:** Tumor thickness, measured according to the mRECIST criteria, and TLC% can be considered as predictors of tumor resectability in patients with pleural mesothelioma.
**Limitations:** n/a
**Ethics Committee Approval:** Yes
**Funding for this study:** n/a
**Author Disclosures:**
Anna Sara Fraia: Nothing to disclose
Andrea Dell'Amore: Nothing to disclose
Alice Bellini: Nothing to disclose
Roberto Stramare: Nothing to disclose
Chiara Giraudo: Nothing to disclose
Antonella Modugno: Nothing to disclose
Federico Rea: Nothing to disclose

### RPS 604-3
**Retrospective evaluation of CT findings of pulmonary toxicities in cancer patients treated with immunotherapy**
*C. Mazza*, F. Sandomenico, S. Cappabianca, I. Simonetti, P. A. Ascierto, M. Mattace Raso, R. Galdiero, A. Petrillo; Naples/IT
*(mazza.carmine89@gmail.com)*

**Purpose:** To analyze CT patterns of interstitial pneumonitis and sarcoid-like reactions as pulmonary immune-related adverse events, in patients treated with immune checkpoint inhibitors.
**Methods or Background:** A retrospective study was performed on 156 cancer patients (87 men and 69 women), treated with immune checkpoint inhibitors (anti-PD-1, anti-PD-L1, anti-LAG-3, anti-CTLA-4). Chest CT scans were reviewed from two radiologists to evaluate patterns and extension of pneumonitis. Pattern CT were evaluated according to the classification of interstitial pneumonitis of American Thoracic Society/European Respiratory Society ATS/ERS: acute interstitial pneumonia (AIP), cryptogenic organizing pneumonia (COP), hypersensitivity pneumonia (HP), non-specific interstitial pneumonia (NSIP), acute respiratory distress syndrome (ARDS).
**Results or Findings:** Among 156 patients treated with ICI, 20 patients developed pneumonitis and 4 patients developed sarcoid-like reactions. CT patterns were AIP in 8, COP in 5, HP in 4, NSIP in 2, ARDS in 1 and sarcoid-like reactions in 4 patients. The AIP pattern was most common in all tumors and treatment. This study also assessed the severity of pneumonitis and of sarcoidosis-like reactions and, among 24 patients were grade 1° in 16 patients, grade 2° in 7 patients and grade 5° in 1 patient according to Common Terminology Criteria for Adverse Events (CTCAE v5.0). Treatment of immune-related pneumonitis includes discontinuation or not of immunotherapy and administration of oral or intravenous corticosteroids, depending on the severity of CT findings of pneumonitis.
**Conclusion:** Radiologist must be aware of these possible adverse events and their CT patterns, in order to promptly communicate them to the oncologist for appropriate therapeutic options.
**Limitations:** None
**Ethics Committee Approval:** Yes
**Funding for this study:** None
**Author Disclosures:**
Mauro Mattace Raso: Nothing to disclose
Igino Simonetti: Nothing to disclose
Antonella Petrillo: Nothing to disclose
Salvatore Cappabianca: Nothing to disclose
Fabio Sandomenico: Nothing to disclose
Roberta Galdiero: Nothing to disclose
Carmine Mazza: Nothing to disclose
Paolo Antonio Ascierto: Nothing to disclose

### Research Presentation Session: Chest

### RPS 704
### COVID-19 findings

### RPS 704-1
**A prognostic model for death in covid-19 patients presenting to the emergency room: the added value of computed tomography**
*G. Besutti*, L. Spaggiari, M. Ottone, E. Bonelli, R. Bonacini, S. Canovi, T. Fasano, P. Giorgi Rossi, P. Pattacini; Reggio Emilia/IT
*(giulia.besutti@libero.it)*

**Purpose:** To develop a prognostic model for death in COVID-19 patients using clinical and CT variables.
**Methods or Background:** Consecutive patients who presented to the emergency room between February 27 and March 23, 2020 for suspected COVID-19, underwent chest CT, and confirmed SARS-CoV-2 positive at RT-PCR were included in this retrospective study. CT disease extension was visually graded as < 20%, 20-39%, 40-59%, or ≥ 60%. The association between clinical and CT variables with death was estimated with multivariable Cox proportional hazards models; model performance was assessed using k-fold cross-validation for the area under the ROC curve (CvAUC).
**Results or Findings:** Of the 866 included patients (median age 59.8, women 39.2%), 93 (10.74%) died. Clinical variables significantly associated with death in multivariable model were age, male sex, HDL cholesterol, dementia, heart failure, vascular diseases, time from symptom onset, neutrophils, LDH, and oxygen saturation level (SO2). CT disease extension was also independently associated with death (HR=7.56, 95% CI=3.49; 16.38 for ≥ 60% extension vs. <20%). CvAUCs were 0.927 (bootstrap bias corrected-95%CI=0.899-0.947) for the clinical model and 0.936 (bootstrap bias corrected-95%CI=0.912-0.953) when adding CT extension.
**Conclusion:** A prognostic model based on clinical variables is highly accurate in predicting death in COVID-19 patients. Adding CT disease extension to the model scarcely improves its accuracy.
**Limitations:** Lack of a validation cohort to test the generalizability of the model; lack of D-dimer values
**Ethics Committee Approval:** AVEN ethical committee (number: 2020/0045199)
**Funding for this study:** None
**Author Disclosures:**
Lucia Spaggiari: Nothing to disclose
Tommaso Fasano: Nothing to disclose
Pierpaolo Pattacini: Nothing to disclose
Riccardo Bonacini: Nothing to disclose
Simone Canovi: Nothing to disclose
Efrem Bonelli: Nothing to disclose
Paolo Giorgi Rossi: Nothing to disclose
Giulia Besutti: Nothing to disclose
Marta Ottone: Nothing to disclose

### RPS 704-2
**Application of multiple radiological classifications to differentiate COVID-19 related pneumonia from the non-COVID-19 disease in a population with a low prevalence of symptomatic COVID-19 patients**
*L. A. Carbonaro*[1], A. L. Nanni[2], P. Gemma[1], A. Cozzi[1], A. Vanzulli[1]; [1]Milan/IT, [2]Pavia/IT
*(luca.carbonaro@gmail.com)*

**Purpose:** We aimed to compare five chest CT categorization systems for the differential diagnosis between COVID-19 pneumonia and non-COVID-19 disease in a population with low prevalence of COVID-19 pneumonia.
**Methods or Background:** 98 chest CT examinations (83 with a non-COVID-19 pulmonary disease diagnosis and 15 with COVID-19 diagnosis) were independently evaluated by two readers applying 5 classification systems (RSNA Statement, COVID-RADS, CO-RADS, Luo score, and Li score) then compared with Cohen's κ and ROC analysis. Sensitivity, specificity, positive predictive value (PPV), and negative predictive value (NPV) were calculated using high or low thresholds of COVID-19 suspicion and compared with the McNemar's test.
**Results or Findings:** Inter-reader agreement was good (Cohen's κ = 0.721) for RSNA Statement, and moderate to good (Cohen's κ ≥ 0.507) for CO-RADS, COVID-RADS, Luo score, and Li score. No statistical difference was found among the AUCs of the ROC analysis of the five classifications for both readers (P ≥ 0.166). Applying a high threshold for COVID-19 suspicion, sensitivity among the five categorization systems ranged 40%–83%, specificity 79%–98%, accuracy 79%–89%, PPV 42%–82%, and NPV 90%–96%.

Applying a low threshold for COVID-19 suspicion, sensitivity among the five systems ranged 80%–90%, specificity 18%–81%, accuracy 58%–81%, PPV 17%–44%, and NPV 90%–97%.
**Conclusion:** In a cohort with low prevalence of COVID-19 pneumonia, all evaluated categorization systems showed similar diagnostic accuracy, the highest specificity and PPV being achieved using the Li score categorization system with a high threshold for COVID-19 suspicion.
**Limitations:** Retrospective design; relatively small sample size.
**Ethics Committee Approval:** Ethics committee approval was obtained in the framework of a local COVID-19 research project. Informed consent was waived for all patients.
**Funding for this study:** None.
**Author Disclosures:**
Andrea Cozzi: Nothing to disclose
Pietro Gemma: Nothing to disclose
Anna Lisa Nanni: Nothing to disclose
Angelo Vanzulli: Nothing to disclose
Luca Alessandro Alessandro Carbonaro: Nothing to disclose

### RPS 704-3

**Coronavirus disease 2019 (COVID-19): the optimisation of iterative reconstructions for pulmonary evaluation in low-dose, high-pitch, dual-source acquisition with spectral shaping at 100 kV**

A. Agostini, *A. Borgheresi*, M. Carotti, L. Ottaviani, M. Badaloni, C. Floridi, A. Giovagnoni; Ancona/IT

**Purpose:** To optimize the use of iterative reconstructions (ADMIRE) in low-dose, high-pitch, dual-source chest CT acquisitions with tin filter at 100 kV in patients with COVID-19.
**Methods or Background:** Patients with confirmed COVID-19 undergoing to unenhanced chest CT on a 3rd generation dual source scanner between March 12th and March 25th, 2020, were prospectively included. The CT protocol included a Dual Energy (DECT, 90/150Sn kVp) and a dual source (LDCT) acquisition (100Sn kVp). The DECT was reconstructed with ADMIRE3; the LDCT with ADMIRE=0,3,5. Two blinded radiologists evaluated in consensus the DECT and the LDCT for image quality, motion artifacts, and pulmonary elementary lesions on 5-point scales. A third radiologist calculated the signal-to-noise ratio (SNR) of lung, trachea, muscle and fat. The effective doses were calculated. Statistical analysis was performed with non-parametric tests (Friedman, Mann-Whitney).
**Results or Findings:** 60 patients (45M/15F, median age 69 y.o.) were included. SNR was significantly higher in DECT than LDCT with any IR. The subjective quality and elementary findings had significantly higher scores in DECT (≥4/5); among LDCT, the IR=3 had the highest score (≥3/5) in all cases. In LDCT, the motion artifacts and effective doses (0.35 mSv vs 4.44 mSv) were significantly lower than DECT ($p<0.0001$).
**Conclusion:** The low dose CT protocol at medium IR strength (ADMIRE3) achieved acceptable image quality with significant reduction of radiation dose and motion artifacts in patients with COVID-19.
**Limitations:** Single center, small cohort.
**Ethics Committee Approval:** The study was approved by the local IRB, and the informed consent was acquired.
**Funding for this study:** None
**Author Disclosures:**
Chiara Floridi: Nothing to disclose
Andrea Agostini: Nothing to disclose
Myriam Badaloni: Nothing to disclose
Marina Carotti: Nothing to disclose
Alessandra Borgheresi: Nothing to disclose
Letizia Ottaviani: Nothing to disclose
Andrea Giovagnoni: Nothing to disclose

### RPS 704-4

**Pulmonary arterial thrombosis 3 months after a SARS-CoV2 infection: Frequency on dual-energy ct (dect) angiographic examinations in 55 patients**

*M. Remy-Jardin*, L. Duthoit, S. Fry, T. Perez, N. Bautin, J-B. Faivre, J. Remy, A. Scherpereel, A. Duhamel; Lille/FR
*(martine.remy@chru-lille.fr)*

**Purpose:** To evaluate pulmonary vascular abnormalities 3 months after a SARS-Cov2 infection.
**Methods or Background:** Among the 320 patients with confirmed COVID-19 hospitalized between March and April 2020 who participated in a systematic follow-up at 3 months, 67 patients with residual respiratory symptoms were referred for specialized follow-up. Fifty-five of them (30 males; 25 females; mean age: 60.4 yr) underwent a DECT angiographic examination on a 3rd-generation dual-source CT system (Force, Siemens Healthineers) with reconstruction of perfusion images (Lung PBV).
**Results or Findings:** All patients had partial (n=40; 72.7%) or complete (n=15; 27.3%) resolution of COVID-19 lung infiltration. CT angiographic images were normal in 52 patients (52/55; 94.6%) and showed non-obstructive thrombi in 3 patients (3/55; 5.4%) at the level of (a) one (n=1) and two (n=1) segmental arteries of a single lobe; and (b) central and peripheral arteries of both lungs (n=1); none showed features of lung infarction. Lung perfusion was rated as non-interpretable in 2 patients (3.6%; shoulder prosthesis/morbid obesity), normal in 17 patients (30.9%) and abnormal in 36 patients (65.5%), the latter group comprising 32 patients with residual COVID-19 opacities (32/55; 58%) and 4 patients with normal lung parenchyma (4/55; 7%). Perfusion abnormalities consisted of (a) patchy perfusion defects in both lungs (30/36; 83%), (b) PE-type perfusion defects (6/36; 16.6%) in presence (n=1) or absence of proximal thrombosis (n=5); and (c) focal areas of hypoperfusion (2/36; 5.5%).
**Conclusion:** DECT depicted non-obstructive proximal thrombosis in 5.4% of patients and perfusion abnormalities suggestive of widespread microangiopathy in 65.5% of patients. Lung microcirculation was abnormal in 7% of patients with a normal lung parenchyma.
**Limitations:** No correlation was possible between CT and PFTs.
**Ethics Committee Approval:** Waiver of patient informed consent
**Funding for this study:** None
**Author Disclosures:**
Jean-Baptiste Faivre: Nothing to disclose
Stéphanie Fry: Nothing to disclose
M. Thierry Perez: Nothing to disclose
Martine Rémy-Jardin: Research/Grant Support: Siemens
Nathalie Bautin: Nothing to disclose
Louise Duthoit: Nothing to disclose
Jacques Remy: Research/Grant Support: Siemens
M. Alain Duhamel: Nothing to disclose
M. Arnaud Scherpereel: Nothing to disclose

### RPS 704-5

**Developing a chest low-dose CT protocol for COVID-19: a phantom study**

*I. Blokhin*, V. Chernina, T. Korb, A. P. Gonchar, O. O. Aleshina, A. Nikolaev, S. Morozov, V. Gombolevskiy; Moscow/RU

**Purpose:** To determine a threshold value of automatic tube current modulation noise index below which there is a chance to miss an onset of ground-glass opacities (GGO) in COVID-19, thus reducing patient dose without affecting the diagnostic outcome.
**Methods or Background:** First, 25 CT studies of an anthropometric phantom with BMI=29 were performed using different automatic tube current modulation (SureExp.3D). Then we retrospectively evaluated chest CT (N=22) and calculated the density difference between the GGO and unaffected tissue. Finally, the clinical image evaluation results were matched to the phantom study results to determine the minimum noise index threshold value.
**Results or Findings:** The minimum difference in density between the GGO and normal lung tissue at the onset of COVID-19 was 252 HU with 95% CI (252 - 349), p=0.00001. This corresponds to the SureExp.3D noise index of 36 as measured at Th11-12 levels.
**Conclusion:** We established a SureExp.3D noise index for Canon scanner without iterative reconstructions, allowing for reduction of radiation dose by 80%. Further dose reduction is not recommended for patients with BMI=29 suspected of having COVID-19, as this might compromise image quality and COVID-19 detection.
**Limitations:** Although the LungMan Phantom N1 anthropometric phantom is similar to the human body in many features, there is no way to adjust the inspiration depth affecting healthy lung tissue density. Second, we used the adapter plates to simulate a patient with BMI=29. The proposed threshold will entail a lower radiation dose in lower BMI, but not for those with BMI>29.
**Ethics Committee Approval:** The IEC approval was not required.
**Funding for this study:** No funding was received for this work.
**Author Disclosures:**
Anna P. Gonchar: Nothing to disclose
Sergey Morozov: CEO: CEO of Research and Practical Clinical Center for Diagnostics and Telemedicine Technologies of the Moscow Health Care Department
Tatiana Korb: Nothing to disclose
Valeria Chernina: Nothing to disclose
Olga Olegovna Aleshina: Nothing to disclose
Alexander Nikolaev: Nothing to disclose
Ivan Blokhin: Nothing to disclose
Victor Gombolevskiy: Nothing to disclose

### RPS 704-6

**Incidental multi-detector computed tomography (MDCT) findings in patients with COVID-19 pneumonia**

*A. El-Badrawy*; Mansoura/EG
*(adelelbadrawy@hotmail.com)*

**Purpose:** To investigate the frequency and significance of incidental MDCT findings in patients with COVID-19 pneumonia.
**Methods or Background:** Medical records of 2,364 COVID-19 patients who underwent chest MDCT scanning were reviewed. All patients proved to be infected with COVID-19 by RT-PCR. Incidental findings were determined from imaging reports. Radiologist coded such findings as benign or clinically significant require further evaluation. These findings are either pulmonary or extra-pulmonary. We analyzed the spectrum of incidental findings and identified potentially significant.
**Results or Findings:** Incidental findings were noted in 257/2364 (10.87%) patients. Follow-up imaging was recommended in 196 patients (8.29%). The prevalence of potentially significant abnormalities was highest for hepato-biliary findings 76/2364 (3.2%), followed by cardiovascular findings 44/2364 (1.86), and breast 38/2364 (1.6%). The prevalence of cancers among screened participants was 48/2364 (2%). The most common malignancy was hepato-cellular carcinoma (20/2364), followed by breast (11/2364), bony thoracic cage (8/2364), bronchogenic carcinoma (7/2364) and lymphoma (2/2364).
**Conclusion:** Incidental findings are commonly identified on MDCT in patients with COVID-19 pneumonia. These findings have a significant impact on treatment and follow up imaging. Further evaluations are needed to avoid the risk of missing malignancy.
**Limitations:** Limitation of our study includes retrospective of our analysis
**Ethics Committee Approval:** The study was approved by the institutional research ethics review committee (Mansoura University/Faculty of Medicine/ Egypt). IRB reference number is “R.20.11.1057 ".
**Funding for this study:** This manuscript is made by the author’s own work without receiving any funding.
**Author Disclosures:**
Adel El-Badrawy: nothing to disclose

### RPS 704-7

**Evaluation of the diagnostic performance and interobserver agreement of CO-RADS classification in COVID-19 pneumonia**

*M. Koşar Tunç*, N. Kış, O. Ince, H. Kurtul Yildiz, H. Önder; Istanbul/TR
*(meliskosar@hotmail.com)*

**Purpose:** To evaluate the diagnostic accuracy of Coronavirus Disease 2019 (COVID-19) Reporting and Data System (CO-RADS) classification in patients suspected of having COVID-19 and to determine the interobserver agreement between radiologists with different years of experience.
**Methods or Background:** Between March and May 2020, 209 chest CT scans of patients with clinically suspected COVID-19 who underwent both chest CT and RT-PCR tests were included in our retrospective study. The images were separately evaluated in consensus by two groups of radiologists with blinding to RT-PCR results. Group 1 consisted of two residents; group 2 consisted of two specialists with at least 15 years of experience. Sensitivity, specificity, and area under the receiver operating characteristic curve (AUC) were calculated. Interobserver agreement was calculated using Kappa statistic by comparing two groups of radiologists.
**Results or Findings:** In 149 of the 209 patients, RT-PCR results were positive. In 302 of 418 (72.2%) observations, there was absolute agreement in the assignment CO-RADS category. A discrepancy of one CO-RADS category was seen in 38 (18.2%) of 418, of two CO-RADS categories in 8 (3.9%) of 418, of three categories in 9 (4.3%) of 418 observations. Interobserver agreement in CO-RADS grading was moderate (k = 0.55). CO-RADS was able to distinguish patients with positive PCR results from those with negative PCR results with an average AUC of 0.75 in group 1 and an average AUC of 0.81 in group 2 (Cut-off value > CO-RADS 3). There was a significant difference in both groups ($p<0.001$).
**Conclusion:** The diagnostic performance of CO-RADS classification for predicting the pulmonary involvement of COVID-19 was very good with significant interobserver agreement.
**Limitations:** Our patient population was small.
It is known that despite negative RT-PCR test results COVID-19 can be diagnosed.
**Ethics Committee Approval:** Yes
**Funding for this study:** None
**Author Disclosures:**
Melis Koşar Tunç: nothing to disclose
Naciye Kış: nothing to disclose
Hakan Önder: nothing to disclose
Hülya Kurtul Yildiz: nothing to disclose
Okan Ince: nothing to disclose

### RPS 704-8

**Pulmonary embolism and coronavirus disease 2019 pneumonia: evidence from the first Italian epicentre**

*E. Mercanzin*, P. A. Bonaffini, P. N. Franco, A. Bonanomi, C. Valle, S. Sironi; Milan/IT
*(e.mercanzin@campus.unimib.it)*

**Purpose:** Our aims were to assess pulmonary embolism (PE) features on chest computed tomography angiography (CTA) in Coronavirus disease 2019 (COVID-19) pneumonia patients and to evaluate its correlations with comorbidities, relevant laboratories tests, pulmonary stages and outcomes.
**Methods or Background:** We retrospectively evaluated 114 COVID-19 patients with pneumonia (mean age 61 years; 26.3% females) who underwent CTA examinations for suspicious PE. PE features (distribution in one or multiple pulmonary lobes; involvement of the pulmonary trunk and/or main pulmonary arteries), pulmonary stages (early; progressive; peak; absorption), comorbidities, D-dimer levels (DD), coagulation tests and outcomes (hospitalized; discharged; died) were investigated. Their corresponding correlations were tested.
**Results or Findings:** PE was diagnosed in 65/114 patients (57%); in 16.9% of cases, pulmonary trunk and/or main arteries were involved. Progressive (24.6%) and peak (67.7%) pulmonary stages were the most frequent. Over a short-term follow-up (60 days) 25 patients (21.9%) were still hospitalized, 72 (63.2%) have been discharged and 17 patients (14.9%) were dead. At least one comorbidity affected 88/114 patients (77.2%). DD showed a significant correlation with PE: in particular, if the main arteries were involved, DD increased ($p = 0.003$). A significant relationship between pulmonary stages and DD was also noted.
**Conclusion:** PE represents a major concern in COVID-19 patients. CTA accurately describes PE features and provides information about concurrent pulmonary stages in COVID-19 pneumonia, according with DD. Therefore, imaging has a crucial role in the overall assessment and management of patients.
**Limitations:** A retrospective study with selection bias as only patients with severe pneumonia were included.
**Ethics Committee Approval:** This study was approved by our institutional ethics committee.
**Funding for this study:** No funding was received for this work.
**Author Disclosures:**
Elisa Mercanzin: Nothing to disclose
Alice Bonanomi: Nothing to disclose
Clarissa Valle: Nothing to disclose
Pietro Andrea Bonaffini: Nothing to disclose
Paolo Niccolò Franco: Nothing to disclose
Sandro Sironi: Nothing to disclose

### RPS 704-9

**Incidental discovered COVID-19 pneumonia in asymptomatic cancer patients undergoing follow-up multi-detector CT**

*A. El-Badrawy*; Mansoura/EG
*(adelelbadrawy@hotmail.com)*

**Purpose:** To investigate the frequency and imaging findings of incidental discovered COVID-19 pneumonia in asymptomatic cancer patients undergoing follow up MDCT.
**Methods or Background:** This retrospective study was conducted for 1122 cancer patients who underwent follow up MDCT scanning without suspicious of COVID-19 infection. Thoracic CT examinations were reviewed. CT images were analyzed to confirm the presence of findings consistent with COVID-19 pneumonia and qualify disease extent. The severity of lung affection was evaluated according to Bernheim et al. (2020) guidelines. Patient with suspicious findings underwent RT-PCR. Clinical and laboratory data re-evaluated.
**Results or Findings:** Among 1122 cancer patients underwent follow up MDCT scanning, 46/1122 (4.1%) patients (28 women and 18 men: median age, 60 years) revealed images highly suggestive of COVID-19 pneumonia. All 46 cases were confirmed by RT-PCR. All had increase CRP and lymphopenia. Disease extent on CT was minimal in 5/46 patients, mild in 31/46 patients, moderate in 7/46 patients and severe in 3/46 patients.
**Conclusion:** Incidental diagnosis of COVID-19 pneumonia on follow up cancer patient about 4.1%. This finding has a significant impact on treatment. Proper evaluation of chest CT images in cancer patient, are needed to avoid the risk of missing incidental discovered COVID-19.
**Limitations:** Limitation of our study includes small numbers of our patients.
**Ethics Committee Approval:** The study was approved by the institutional research ethics review committee (Mansoura University/Faculty of Medicine/ Egypt). IRB reference number is “R.20.11.1056 ".

**Funding for this study:** This manuscript is made by the author's own work without receiving any funding.
**Author Disclosures:**
Adel El-Badrawy: Nothing to disclose

### RPS 704-10

**A spectrum of atypical HRCT chest imaging features in COVID 19 patients – a revelation**

*R. Arora*; Delhi/IN
*(arorarohit0810@gmail.com)*

**Purpose:** To identify and categorize the additional Atypical imaging features detected in COVID 19 patients from eastern India.
**Methods or Background:** HRCT images of 1300 COVID-19 patients without any known co-morbid conditions and showing positive HRCT findings were analyzed and evaluated for prevalence of atypical imaging features. HRCT images were categorized into typical, atypical and indeterminate. Further the additional atypical features were evaluated.
**Results or Findings:** Out of 1300 patients, 320 (24.6%) patients showed atypical imaging features, 860 patients (64.6%) were in the Typical and 140 (10.7%) were in the indeterminate category. Amongst patient with atypical imaging features we found that isolated lobar or segmental consolidation without associated GGO's prevalent in 5.6% patients, discrete pulmonary nodules which includes both centrilobular and tree-in bud nodular patterns in 42%, mediastinal / hilar lymphadenopathy in 9.3%. About 11% patients had pleural effusion and 1.2% demonstrated pneumothorax and pneumomediastinum. Linear or sub segmental atelectasis was noted in 66% patients.
**Conclusion:** Significantly higher additional atypical features like atelectatic bands & subpleural curvilinear atelectasis (66%), followed by discrete nodules (42%) were associated with COVID 19 diagnosis in absence any known co-morbid conditions. We propose that additional imaging features which have not been categorized under any of the existing four groups, be incorporated in either a mixed category or added to any of the existing groups, in the current imaging based classification for COVID 19.
**Limitations:** No long duration follow-up scans were done in our study population.
**Ethics Committee Approval:** Approved
**Funding for this study:** Not Applicable
**Author Disclosures:**
Girendra Shankar: Nothing to disclose
Kamal Sen: Nothing to disclose
Rohit Arora: Nothing to disclose
Sunny Swaraj: Nothing to disclose
Jagadeesh Kuniyil: Nothing to disclose
Dwarampudi Sindhu Reddy: Nothing to disclose
Sangram Panda: Nothing to disclose

### RPS 704-11

**A composite index considering CT disease extension and days from symptom onset at emergency room presentation predicts hospital readmission after emergency room discharge in COVID-19 patients**

*G. Besutti*, M. Ottone, F. Luppi, O. Duric, M. Galli, V. Curcio, A. Nitrosi, P. Giorgi Rossi, P. Pattacini; Reggio Emilia/IT
*(giulia.besutti@libero.it)*

**Purpose:** To find baseline predictive factors for emergency room (ER) readmission with hospitalization in COVID-19 patients who were initially discharged from ER.
**Methods or Background:** All consecutive COVID-19 patients admitted to the ER between March 2 and March 31,2020, and discharged for home treatment, were included. Age, sex, comorbidities (alone and combined in Charlson index), days from symptom onset, signs and symptoms, laboratory examinations, CT findings (including CT disease extension as visual percentage) at the moment of first ER admission were collected and tested as predictive factors for ER readmission with hospitalization within 45 days, by means of univariate and multivariate models reporting incidence rate ratio (IRR) with 95%CI. A disease progression rapidity index dividing CT extension by the days from symptom onset plus three days lag was also considered.
**Results or Findings:** We included 454 patients (median age 54 years, 47% females), 82 (18.1%) were readmitted to the ER and among them 56 (12.3%) were hospitalized. Only age (IRR=1.03,95%CI=1.01-1.05), Charlson index 3 vs 0 (IRR=4.79,95%CI=1.58-14.52) days from symptom onset (IRR for one day increase=0.86,95%CI=0.78-0.94), and CT extension (IRR for 1% increase=1.03,95%CI=1.01-1.06) were associated in multivariate model with readmission with hospitalization. When replacing the latter two with the composite index (IRR for unit increase =1.28,95%CI=1.12-1.46), the variance explained by the model slightly decreased (R2 from 0.12 to 0.10).
**Conclusion:** A disease progression rapidity index combining CT extension and days from symptom onset at first ER presentation may predict disease worsening, i.e. a patient with 20% CT parenchymal involvement 1 day after symptom onset has the same risk of a person with 50% after 7 days.
**Limitations:** Findings depend on the criteria for home discharge applied to our cohort, studies on other populations are needed.
**Ethics Committee Approval:** AVEN (number:2020/0045199)
**Funding for this study:** None
**Author Disclosures:**
Mariagiulia Galli: Nothing to disclose
Pierpaolo Pattacini: Nothing to disclose
Olivera Duric: Nothing to disclose
Andrea Nitrosi: Nothing to disclose
Vittoria Curcio: Nothing to disclose
Francesco Luppi: Nothing to disclose
Paolo Giorgi Rossi: Nothing to disclose
Giulia Besutti: Nothing to disclose
Marta Ottone: Nothing to disclose

### RPS 704-12

**COVID-19 disease beyond pulmonary manifestation: a radiological overview**

*R. I. E. Yasin*[1], W. A. Gouda[1], E. A. G. Ibrahim[2]; [1]Menofia/EG, [2]Cairo/EG
*(rabab_yasin@outlook.com)*

**Purpose:** Our study focuses on the incidence of different extrapulmonary imaging findings in patients with COVID-19 pneumonia.
**Methods or Background:** COVID-19 disease was primarily described as a pandemic of respiratory illness, however with the disease progression variable cases with extrapulmonary manifestations have been reported all over the world. SARS-CoV-2 infection can affect different body systems with the neurologic, abdominal, thromboembolic, cardiac, mediastinal and hematological manifestations had been reported in many literatures. The understanding of these multisystemic involvement are being better understood as the pandemic progresses.
**Results or Findings:** 3500 proved COVID-19 patients were enrolled in our study 2670 (76.3%) males and 830 (23.7 %) females with mean age was 48.11 ± 10.18 years old, ranging from 20-82 years. COVID-19 associated extrapulmonary imaging findings were reported in 159 patients (4.5%) with the neurological findings were the most frequent extrapulmonary manifestations and detected in 98 cases (61.6%), followed by 46 cases (29%) with abdominal findings and 15 (9.4 %) cases with limb ischemia.
**Conclusion:** COVID-19 infection showed wide spectrum of multisystemic involvement and imaging modality have a great role in the assessment of disease dissemination to avoid any untreated causes of morbidity/ mortality.
**Limitations:** Not applicable
**Ethics Committee Approval:** The study protocol was approved by the local Ethics Committee
**Funding for this study:** Not applicable
**Author Disclosures:**
Walaa Abdullah Gouda: Nothing to disclose
Rabab Ibrahim Elsayed Yasin: Nothing to disclose
Enas Abdel Ghany Ibrahim: Nothing to disclose

### RPS 704-13

**Coronary and total thoracic calcium: strong predictors of COVID-19 patients outcome**

*V. Nicoletti*[1], A. Palmisano[1], D. Vignale[1], R. Leone[1], C. Gnasso[1], M. Toselli[2], A. Cereda[2], F. Giannini[2], A. Esposito[1]; [1]Milan/IT, [2]Cotignola/IT
*(nicoletti.valeria@hsr.it)*

**Purpose:** The study aim was to evaluate the role of coronary and total thoracic calcium on in-hospital mortality in COVID-19 patients.
**Methods or Background:** Since December 2019, an acute respiratory disease, the coronavirus disease 2019 (COVID-19), has spread worldwide becoming a global public health emergency. Early identification of high-risk patients could permit a tailored approach and improve disease management. Coronary, thoracic aorta and aortic valve calcium are validated long-term predictors of cardiovascular events and all-cause mortality and can be easily measured from a non-gated chest computer tomography (CT). However, their prognostic role in the context of an acute and systemic inflammatory disease, such as COVID-19, has not been previously investigated. We retrospectively enrolled 1093 consecutive patients hospitalized for COVID-19 in 17 Italian hospitals, all with an admission chest CT performed to evaluate pneumonia severity. Coronary, thoracic aorta, aortic valve and total thoracic calcium were qualitatively and quantitatively assessed (Agatston calcium scoring and calcium volume methodology).
**Results or Findings:** Compared to survivors, non-survivors had higher coronary artery [Volume (487.79±565.34 vs 207.77±406.81, $p<0.001$); Agatston (467.76±570.92 vs 206.80±424.13 mm2, $p<0.001$)], aortic valve [Volume (322.45±390.90 vs 98.27±250.74 mm2, $p<0.001$; Agatston 337.38±414.97 vs 111.70±282.15, $p<0.001$)] and thoracic aorta [Volume

(3786.71±4225.57 vs 1487.63±2973.19 mm2, p<0.001); Agatston (4688.82±5363.72 vs 1834.90±3761.25, p<0.001)] calcium scores. Coronary artery calcium (HR 1.308; 95% CI, 1.046-1.637, p=0.019) and total thoracic calcium (HR 1.975; 95% CI, 1.200-3.251, p=0.007) resulted to be independent predictors of in-hospital mortality.
**Conclusion:** Calcium assessment of coronary, aortic valve and thoracic aorta on non-gated chest CT is an easy and useful tool to predict the in-hospital mortality risk of COVID-19 patients.
**Limitations:** This a retrospective study and includes only patients who underwent CT for lung assessment, being potentially not reflective of all COVID-19 patients.
**Ethics Committee Approval:** Approved
**Funding for this study:** None
**Author Disclosures:**
Francesco Giannini: Nothing to disclose
Valeria Nicoletti: Nothing to disclose
Davide Vignale: Nothing to disclose
Antonio Esposito: Nothing to disclose
Anna Palmisano: Nothing to disclose
Marco Toselli: Nothing to disclose
Alberto Cereda: Nothing to disclose
Riccardo Leone: Nothing to disclose
Chiara Gnasso: Nothing to disclose

### RPS 704-14

**CT chest in novel COVID-19 pandemic: how can it help diagnose and predict patient's ICU needs?**
*A. M. M. Abdrabou*, A. M. Osman, A. Yassin, R. Hashim; Cairo/EG
*(arabou2000@hotmail.com)*

**Purpose:** COVID-19 infection is a worldwide pandemic since December 2019. This study aimed to assesses the CT accuracy in the detection of COVID-19 cases, evaluate the CT manifestations, and try to find a relation between the CT severity and the management
**Methods or Background:** A retrospective study was conducted on 600 patients, presented with symptoms suspicious for COVID-19 infection from March till the end of June 2020. 466 patients were included, 340 patients were confirmed COVID-19 infection by PCR. We followed the RSNA recommendations in CT reporting to be correlated with the PCR. The different CT findings were analyzed. Finally, the CT severity score was correlated with the patient's management
**Results or Findings:** CT sensitivity was 92.6% while the PCR is the reference. We found an increase in specificity with the increase in the level of CT probability. The CT high probability findings showed 97.6% specificity. GGO was the commonest CT findings 85.9% with a high incidence of bilateral, peripheral, and multilobar involvement (88%, 92.8%, and 92.8% respectively). Consolidation was found in 81.5% between ICU patients being predominant in 66.7% of the ICU cases. CT severity score was significantly higher in ICU patients with score ≥ 14
**Conclusion:** CT can be used as a rapid and sensitive investigation to detect COVID-19 infection. COVID-19 infection is typically presented as bilateral peripheral ground-glass opacities. CT severity score and consolidation can be used as parameters for ICU need
**Limitations:** It was difficult to collect detailed clinical and laboratory data about the patients due to pandemic status. So, we used the decision taken for the patient's management as a reflection of their clinical condition
**Ethics Committee Approval:** Faculty of medicine - Ain Shams University ethical committee approval
**Funding for this study:** No fund
**Author Disclosures:**
Reham Hashim: Nothing to disclose
Ahmed Mohammed Mahmoud Abdrabou: Nothing to disclose
Aya Yassin: Nothing to disclose
Ahmed M. Osman: Nothing to disclose

### RPS 704-15

**Quantitative chest CT and radiography substantially improve prognostic prediction of severe COVID-19 in multiparametric statistical models, when compared to clinical variables**
*E. J. M. Barbosa Jr.*[1], H-M. Jung[1], R. Yang[1], W. Gefter[1], F. Ghesu[2], S. Liu[2], A. Mansoor[2], S. Grbic[2], S. Vogt[3]; [1]Philadelphia, PA/US, [2]Princeton, NJ/US, [3]Malvern, PA/US
*(eduardo.mortani@gmail.com)*

**Purpose:** To evaluate whether severe COVID-19 can be predicted by statistical models leveraging clinical, quantitative chest CT (qCT) and quantitative chest radiograph (qCXR) airspace disease (AD) variables, obtained at initial diagnosis.
**Methods or Background:** We retrospectively selected 86 COVID-19 patients (SARS-CoV2 positive, March-July 2020) at a tertiary hospital in the USA, who underwent chest CT and CXR within 48 hrs. Clinical variables (CVs) included age, BMI, PaO2, C-reactive protein; imaging variables included qCT volumetric percentage AD (POv) and qCXR area-based percentage AD (POa), assessed by 2 expert thoracic radiologists and a deep convolutional neural network (CNN). Our prognostic outcome was the need for ICU admission. We developed 3 pairs of Random Forest (RF) and Logistic Regression (LR) models: using CVs known to be associated with worse prognosis (Model I); using only age and imaging variables (Model II); and using a dimension-reduced set of best predictor variables (Model III). RF and LR results were summarized respectively as out-of-bag error rate (OOB) and ROC AUC.
**Results or Findings:** 42/86 patients did not require ICU admission, whereas 44/86 did. Model I had the worst performance (AUC=0.739 [0.634-0.844]; OOB=42%), with only age as a statistically significant predictor. Model II demonstrated substantially improved performance (AUC=0.833 [0.748-0.918]; OOB=25%). Model III was the best overall (AUC=0.858 [0.782-0.943]; OOB=25%). Model II and III results did not change significantly when POv was replaced by POa.
**Conclusion:** Severe COVID-19 can be accurately predicted with only age and quantitative AD imaging metrics at presentation. Quantitative imaging contributes to prognostication to a greater extent than the set of CVs, and qCXR metrics can replace qCT without loss of prognostic performance. Our results could assist with resource allocation to foster better outcomes for COVID-19 patients.
**Limitations:** Retrospective study
**Ethics Committee Approval:** IRB approved
**Funding for this study:** None
**Author Disclosures:**
Hae-Min Jung: Nothing to disclose
Florin Ghesu: Employee: Siemens
Sebastian Vogt: Employee: Siemens
Eduardo Jose Mortani Barbosa Jr.: Research/Grant Support: Siemens Research Grants
Awais Mansoor: Employee: Siemens
Sasa Grbic: Employee: Siemens
Rochelle Yang: Nothing to disclose
Warren Gefter: Research/Grant Support: Siemens Research Grants
Siqi Liu: Employee: Siemens

### RPS 704-16

**Impact of COVID-19 on the lung cancer diagnostic pathway in Liverpool: an audit**
*D. Duvva*, M. Khan, M. Arzanauskaite, M. Ledson; Liverpool/UK
*(dileepduvva@doctors.org.uk)*

**Purpose:** The first nationwide lockdown introduced in the UK has led to deferment of routine diagnostic work with most hospitals and primary services prioritising only urgent and symptomatic cases for diagnostic pathways resulting in concerns of diagnostic delay and subsequent higher cancer related deaths. In this study, we aimed to review the functioning of diagnostic pathways of Liverpool Lung Cancer Unit (LLCU) during the lockdown comparing to its performance in 2019.
**Methods or Background:** Liverpool Lung Cancer Unit (LLCU), one of the largest Lung cancer units in the UK provides for investigations, diagnosis and treatment and inpatient services at both Royal Liverpool University hospital and Liverpool Heart and Chest Hospital (LHCH) and importantly outpatient services through the virtually operated Rapid Access Lung Service (RALS). It has extended its diagnostic and outpatient services to Aintree hospital, another major tertiary centre in Liverpool city from 27th of March till the 9th of June. Data was collected from PACS, CRIS and the Somerset Cancer Registry to identify and cross reference all the entries in the diagnostic pathways.
**Results or Findings:** The total number of coded images for suspected lung cancer, referrals from GPs and outpatient services have increased by 85%, 19% and 78% whilst the referrals from the emergency units surged by a 141%. However, the total number of patients seen by RALS has seen a decline of 18% during the same period.
**Conclusion:** The LLCU "straight to CT model" was operating efficiently during the spring 2020 UK lockdown. Whilst it successfully managed patients from its own catchment, it also was able to extend its virtual platform based RAL services another tertiary hospital catering for the needs of a population of the whole of Liverpool City.
**Limitations:** n/a
**Ethics Committee Approval:** n/a
**Funding for this study:** None
**Author Disclosures:**
Monika Arzanauskaite: Nothing to disclose
Dileep Duvva: nothing to disclose
Muhammad Khan: Nothing to disclose
Martin Ledson: Nothing to disclose

**RPS 704-17**
**COVID-19-related acute respiratory distress syndrome: HRCT findings within two months after resolution of the infection**
*E. Baratella*, N. Starvaggi, C. Marrocchio, S. Tollot, F. Giudici, M. A. A. Cova; Trieste/IT
*(elisa.baratella@gmail.com)*

**Purpose:** To evaluate the imaging findings on high-resolution CT of patients with COVID-19-related ARDS.
**Methods or Background:** Between March and September 2020, patients with a previous diagnosis of COVID-19-related ARDS who had performed a CT scan within 8 weeks since a negative RT-PCR on nasopharyngeal swabs were retrospectively identified. The following CT findings were evaluated and described according to their segmental distribution: ground glass opacities (GGO), consolidations, reticulations, bronchiectasis/bronchiolectasis, linear bands, loss of pulmonary volume and pleural effusion. Radiological findings were correlated with age, sex, positive swab days, hospitalization days and spirometry results. The t-student or Mann-Whitney tests were used for continuous variables, while categorical variables were compared by the Chi-Square independence test or Fischer's exact test. To evaluate the linear correlation between age and number of CT findings per patient, the Spearman correlation coefficient was calculated. Statistical tests were two-tailed and the significance level was considered for p-value<0.05.
**Results or Findings:** Forty-four patients (M:32, F:12, median age:64) were included. Alterations of the pulmonary parenchyma were observed in 97,7% of patients. Linear bands were the most common findings (84%), followed by GGO (75%), reticulations (34%), bronchiolectasis (32%), consolidations (30%), bronchiectasis (30%) and volume loss (25%). Imaging findings had a symmetric distribution and both lower lobes were the most affected ones.
**Conclusion:** Our study describes the short-term HRCT findings of COVID-19-related ARDS after resolution of the infection. Reticulations can be an early sign of post-ARDS fibrosis, as described in previous coronavirus outbreaks.
**Limitations:** Retrospective nature of the study
**Ethics Committee Approval:** Ethical approval was obtained
**Funding for this study:** This study has not received funding
**Author Disclosures:**
Elisa Baratella: Nothing to disclose
Fabiola Giudici: Nothing to disclose
Cristina Marrocchio: Nothing to disclose
Saverio Tollot: Nothing to disclose
Maria Assunta A. Cova: Nothing to disclose
Natalia Starvaggi: Nothing to disclose

**RPS 704-18**
**Chest CT texture-based radiomics analysis in differentiating COVID-19 from other interstitial pneumonia**
*C. Rucci*, M. Zerunian, T. Polidori, G. Guido, E. Lucertini, B. Bracci, M. Polici, A. Laghi; Rome/IT
*(carlottarucci@gmail.com)*

**Purpose:** To evaluate the ability of Texture Analysis in differentiating COVID-19 pneumonia from pneumonia of other etiology on Chest-CT.
**Methods or Background:** One hundred and twenty patients admitted to Emergency Department, from March 8, 2020 to April 25, 2020, with suspicious of COVID-19 that underwent Chest-CT, were retrospectively analyzed. All patients presented CT findings indicative for interstitial pneumonia. Sixty patients with positive COVID-19 real-time reverse transcription polymerase chain reaction (RT-PCR) and sixty patients with negative COVID-19 RT-PCR were enrolled. CT Texture analysis (CTTA) was manually performed using dedicated software by two Radiologists in consensus and textural features on filtered and unfiltered images were extracted as follows: mean intensity, standard deviation (SD), entropy, mean of positive pixels (MPP), skewness, kurtosis. Non-parametric Mann Whitney test assessed CTTA ability to differentiate positive from negative COVID-19 patients. Diagnostic criteria was obtained from ROC curves.
**Results or Findings:** Unfiltered CTTA showed lower values of mean density, MPP and kurtosis in COVID-19 positive patients compared to negative patients (p=0,041; 0,004 and 0,002 respectively). On filtered images, fine and medium texture scales were significant differentiators; fine texture scale being most significant where COVID-19 positive patients had lower SD (p= 0.004) and MPP (p=0.004) compared to COVID-19 negative patients. A combination of the significant texture features could identify the patients with positive COVID-19 from negative COVID-19 with a sensitivity of 60% and specificity of 80% (p=0.001).
**Conclusion:** Preliminary evaluation suggests potential role of CTTA in distinguishing COVID-19 pneumonia from other interstitial pneumonia on Chest-CT.
**Limitations:** Retrospective nature, single-center design and small sample size.
**Ethics Committee Approval:** This study was approved by our local institutional review board and written informed consent was obtained from all study participants.
**Funding for this study:** No.
**Author Disclosures:**
Carlotta Rucci: Nothing to disclose
Marta Zerunian: Nothing to disclose
Benedetta Bracci: Nothing to disclose
Tiziano Polidori: Nothing to disclose
Andrea Laghi: Nothing to disclose
Michela Polici: Nothing to disclose
Gisella Guido: Nothing to disclose
Elena Lucertini: Nothing to disclose

### Research Presentation Session: Artificial Intelligence & Machine Learning

### RPS 305

### AI in the diagnosis and treatment of COVID

**RPS 305-1**
**Prognostic modelling of COVID-19 patients risk stratification using chest CT radiomic features and machine learning**
*I. Shiri*[1], M. Pakbin[2], Y. Salimi[1], A. Sanaat[1], A. Akhavanalaf[1], S. Sandoughdaran[3], K. R. Kalantari[3], H. Arabi[1], H. Zaidi[1]; [1]Geneva/CH, [2]Qom/IR, [3]Tehran/IR
*(isaac.sh92@gmil.com)*

**Purpose:** A wide range of radiological examinations, including chest CT, are currently used for clinical diagnosis and prognosis of COVID-19 patients. In current work, we developed a risk stratification model for COVID-19 patients using radiomics features extracted from chest CT images and machine learning
**Methods or Background:** We enrolled 2525 volumetric CT images of COVID-19 patients, including 1992 alive and 533 deceased patients. Eighty percent of the patients were used for training whereas 20% were kept for external validation. 3D volumes of whole lung and COVID-19 lesions were segmented using a deep learning-based algorithm. Radiomic features were extracted from the segmented lung and infectious lesions from CT images. Maximum relevance minimum redundancy (MRMR) and XGBoost were used for feature selection and risk stratification. The metrics adopted for model performance assessment include the area under the receiver operating characteristic curves (AUC), accuracy, sensitivity and specificity.
**Results or Findings:** Our results indicated that the combined model (Lung + Lesion) had the highest prognostic capability with AUC = 0.94 ± 0.021, 0.91 ± 0.03 accuracy, 0.90 ± 0.072 sensitivity and 0.90 ± 0.02 specificity.
**Conclusion:** We developed a radiomics and machine learning-based risk stratification model for COVID-19 patients. The results demonstrated high prognostic performance in terms of discrimination between alive and deceased COVID-19 patients. The proposed model has significant potential for improved management of COVID-19 patients.
**Limitations:** Not applicable
**Ethics Committee Approval:** This retrospective study was conducted with institutional review board approval. Formal written consent was waived owing to the nature of the study.
**Funding for this study:** Swiss National Science Foundation under grant SNRF 320030_176052
**Author Disclosures:**
Saleh Sandoughdaran: Nothing to disclose
Amirhossein Sanaat: Nothing to disclose
Hossein Arabi: Nothing to disclose
Habib Zaidi: Nothing to disclose
Azadeh Akhavanalaf: Nothing to disclose
Masoumeh Pakbin: Nothing to disclose
Yazdan Salimi: Nothing to disclose
Isaac Shiri: Nothing to disclose
Kiara Rezaei Kalantari: Nothing to disclose

### RPS 305-2

**Deep learning-based diagnostic model for COVID-19 pneumonia detection from chest CT images**

*I. Shiri*[1], M. Pakbin[2], Y. Salimi[1], A. Sanaat[1], A. Akhavanalaf[1], S. Sandoughdaran[3], K. R. Kalantari[3], H. Arabi[1], H. Zaidi[1]; [1]Geneva/CH, [2]Qom/IR, [3]Tehran/IR
*(isaac.sh92@gmil.com)*

**Purpose:** Chest CT imaging has emerged as a useful tool for early diagnosis and longitudinal follow up of COVID-19 patients. In this work, we developed a deep learning-based diagnostic model of COVID-19 disease.
**Methods or Background:** We used 2332 3D CT images of COVID-19 patients, including 1010 PCR-positive COVID-19 pneumonia and 1322 non-COVID-19 pneumonia (bacterial and viral) patients. Eighty percent of the subjects were used for training whereas 20% were kept for external validation. All images were cropped and resized according to outcome of lung segmentation and the intensity values clipped and normalized. A 3D deep residual convolutional neural network (RCNN) built in PyTorch was employed for classification. The metrics adopted for model performance assessment included the area under the receiver operating characteristic curves (AUC) and accuracy.
**Results or Findings:** A mean AUC and accuracy of 0.88±0.05 and 0.87±0.02 were achieved for distinguishing PCR-positive COVID-19 and non-COVID-19 pneumonia when using the 3D-RCNN.
**Conclusion:** We developed a deep learning-based classification model of COVID-19 patients using a deep learning algorithm. The results in terms of discriminating PCR-positive COVID-19 and non-COVID-19 pneumonia patients are promising.
**Limitations:** Not applicable
**Ethics Committee Approval:** This retrospective study was conducted with institutional review board approval. Formal written consent was waived owing to the nature of the study.
**Funding for this study:** Swiss National Science Foundation under grant SNRF 320030_176052
**Author Disclosures:**
Saleh Sandoughdaran: nothing to disclose
Amirhossein Sanaat: Nothing to disclose
Hossein Arabi: Nothing to disclose
Habib Zaidi: Nothing to disclose
Azadeh Akhavanalaf: Nothing to disclose
Masoumeh Pakbin: Nothing to disclose
Yazdan Salimi: Nothing to disclose
Isaac Shiri: Nothing to disclose
Kiara Rezaei Kalantari: Nothing to disclose

### RPS 305-3

**From no data to big data: development of a management system for imaging and data analysis of chest X-Rays in patients diagnosed COVID-19 in Mexico**

E. Hernandez Rangel, A. Negreros, *B. A. Gonzalez Vazquez*, M. Francisco, G. Elizondo Riojas; Monterrey/MX
*(bagzzvzz@gmail.com)*

**Purpose:** To develop an open-access chest X-Ray´s and data storage platform in patients diagnosed with COVID-19, for deep neural networks and machine learning tools creation in Mexico. To collaborate with different hospitals, medical centers, and universities in the construction a national imaging database with COVID-19 in Mexico.
**Methods or Background:** In most Latin America Countries, there is a poor representations in image datasets, because of the lack of IT resources. We developed a graphical user interface for image and data storage, patient de-identification, image curation and annotation, formatting annotation output, for chest X-rays of patients diagnosed with COVID-19. Certified radiologist from all the country were in charge to upload, curate, annotate and segment ROI with COVID-19 pneumonia most common findings.
**Results or Findings:** We received a total of 688 x-ray images from different medical centers. We developed 15 convolutional neural network (CNN) models, which were tested against expert radiologists to find the best model, and we are in the validation process of the most accurate CNN´s.
**Conclusion:** Developing countries lack the access to robust IT and state of the art technologies. Having access to user friendly platforms to create image datasets will bring more representation of these countries.
**Limitations:** Among our most important limitations is that we couldn't include some hospitals specially in rural areas because they lack EHR or PAC´s systems
**Ethics Committee Approval:** IRB approval
**Funding for this study:** None
**Author Disclosures:**
Guillermo Elizondo Riojas: Nothing to disclose
Manuel Francisco: Nothing to disclose
Eduardo Hernandez Rangel: Nothing to disclose
Barbara Aimee Gonzalez Vazquez: Nothing to disclose
Adrian Negreros: Nothing to disclose

### RPS 305-5

**Usefulness of the Botkin.AI platform for screening non-calcified pulmonary nodules in patients with chest CT scans performed during the COVID-19 pandemic**

*P. Pilius*, I. Drokin; Saint Petersburg/RU
*(polishka1903@gmail.com)*

**Purpose:** During the coronavirus disease pandemic, the number of chest CT scans performed for assessment of the lung changes increased dramatically. Our aim was a retrospective analysis of chest CT studies on the Botkin.AI platform, which is trained to search for features of malignant neoplasms in the lungs using artificial intelligence.
**Methods or Background:** Chest CT scans that we use for analysis were performed in patients undergoing hospital treatment for laboratory-confirmed coronavirus infection. Since the sensitivity of nodules detection is reduced against the background of pronounced lungs opacities, the first stage of all studies was processed on the Botkin.AI platform, which is trained to recognize and quantify such syndromes as ground glass opacities and consolidation. Studies in which the total percentage of both lung affection was more than 5% and less than 25% were used for further analysis by the platform for the presence of pulmonary nodules.
**Results or Findings:** In 170 chest CT studies in patients with coronavirus infection, the platform found non-calcified solid nodules, 19 of which were larger than 6 mm in diameter and required additional dynamic monitoring. The sensitivity of artificial intelligence in detecting nodules was higher than that of a radiologist (respectively 88.4% and 81%, $p<0.005$). The false-positive rate for the AI results was 12%.
**Conclusion:** The Botkin.AI Platform can be used for additional screening of pulmonary nodules in patients who have performed Chest CT scans in order to assessment of viral pneumonia affection during the coronavirus pandemic. The sensitivity of detecting nodules using artificial intelligence is higher than that of a radiologist.
**Limitations:** For better evaluation of benefit of clinical use of the platform it's required to follow-up patients with suspicious pulmonary nodules and check whether some of them will grow.
**Ethics Committee Approval:** Approved
**Funding for this study:** Botkin.AI
**Author Disclosures:**
Ivan Drokin: Nothing to disclose
Polina Pilius: Nothing to disclose

### RPS 305-6

**Multi-modal trained artificial intelligence solution to triage chest x-ray for COVID-19**

*T. Tan*[1], B. Das[2], R. Soni[3], M. Fejes[4], S. R. Ranjan[2], P. Tegzes[4], L. Ferenczi[4], R. Mullick[2], G. Avinash[3]; [1]Eindhoven/NL, [2]Bangalore/IN, [3]San Ramon, CA/US, [4]Budapest/HU
*(tao.tan@ge.com)*

**Purpose:** Artificial intelligence (AI) assisted X-ray (XR) based applications for triaging and monitoring COVID patients efficiently is seen as a promising solution. We demonstrate a functional AI model to triage by inferencing using a single x-ray image, while the deep-learning model is trained on labeled paired and unpaired X-ray and CT data.
**Methods or Background:** Our experiment leverages multi-center paired (48hr.) and unpaired CT and XR data. To avoid domain shift in evaluation, we only use in-house data for testing. Three-class data includes COVID-19 (974 train images/113 validation images/268 testing images), pneumonia (10175/531/119) and negative (17068/3731/34). From unpaired and paired CT data, we created synthetic X-ray (SXR) to significantly augment training image pool. With projection and registration, we transferred the CT annotations to paired XR with manual fine-tuning. The transferred annotations were used for training. Our deep-learning model takes frontal X-ray as input and outputs location of the disease regions and classification.
**Results or Findings:** Compared to X-ray only training, the multi-modal training improves area under receiver operating characteristic curve (AUC) from 0.89 to 0.93 and segmentation DICE from 0.59 to 0.62.
**Conclusion:** Multi-modal training learns from CT and benefits the model inferencing on X-rays for COVID-19 diagnosis and segmentation.
**Limitations:** Polymerase chain reaction (PCR) tests are used as ground-truth but PCR is not 100% reliable.
**Ethics Committee Approval:** IRB oversight waived.
**Funding for this study:** Not applicable.

**Author Disclosures:**
Gopal Avinash: Employee: GE healthcare
Tao Tan: Employee: GE healthcare
Mate Fejes: Employee: GE healthcare
Lehel Ferenczi: Employee: GE healthcare
Bipul Das: Employee: GE healthcare
Pál Tegzes: Employee: GE healthcare
Sohan R. Ranjan: Employee: GE healthcare
Ravi Soni: Employee: GE healthcare
Rakesh Mullick: Employee: GE healthcare

### RPS 305-7

**AI for COVID: an Italian multicentre study for the prediction of clinical outcomes in COVID-19 patients by applying artificial intelligence to chest x-ray examinations**

N. C. D'Amico[1], G. Valbusa[2], D. Sona[3], M. Cellina[4], E. Stellato[4], S. Panella[4], C. Biasibetti[5], D. Cozzi[6], *M. Alì*[4]; [1]Rome/IT, [2]Colleretto Giacosa/IT, [3]Genova/IT, [4]Milan/IT, [5]Pavia/IT, [6]Florence/IT
*(marco.ali90@gmail.com)*

**Purpose:** The second wave of SARS-CoV-2 has arrived. Although major improvements have been made in therapies, there are no ways to predict the clinical evolution of patients. We propose a machine learning (ML) and radiomics tools for predicting the clinical evolution of COVID patients from chest x-ray (CXR), integrating in the analysis blood and other clinical data including comorbidities obtained at admission. These tools aim to support: i) high risk patients management, and ii) hospital resources management (e.g. Intensive Care Unit (ICU) beds).
**Methods or Background:** CXR images and clinical data of 823 COVID19-patients were collected from a network of Italian hospitals. According to their required treament and outcome, patients were divided into two groups: mild (no ventilatory support) and severe (non-invasive ventilation support or ICU, or dead). Images and clinical data were analyzed using 3 ML methods and radiomics: support vector machine (SVM), logistic regression (LR) and decision trees (DT). Model were validated with 10-fold and centre-wise cross-validation.
**Results or Findings:** Females (32%) were significantly older than males: 70 (57–80) vs 64 (53–74) years. 87% of patients had at least one comorbidity. Significant differences between severe and mild groups were: Age (years): 70 (60–79) vs 60 (49–72), LDH (U/L): 405 (310-527) vs 271 (214-323), O2 (%): 92 (87-96) vs 96 (94-98), D-dimer: 820 (438-2056) vs 549 (262-909) and others. Distributions were reported as median and interquartile range. The 3 predictive models based on SVM, LR and DT showed 74%, 75%, 65% and 73%, 75%, 60% accuracy with 10-fold and center-wise crossvalidation respectively.
**Conclusion:** ML applied to CXR images and clinical data showed robust results in predicting COVID-19 patients outcome. Raw data will be made open-access to the scientific community.
**Limitations:** Retrospective design
**Ethics Committee Approval:** Approval was obtained on 7.APR.2020
**Funding for this study:** None
**Author Disclosures:**
Diego Sona: Nothing to disclose
Giovanni Valbusa: Nothing to disclose
Marco Alì: Nothing to disclose
Elvira Stellato: Nothing to disclose
Silvia Panella: Nothing to disclose
Michaela Cellina: Nothing to disclose
Natascha Claudia D'Amico: Nothing to disclose
Diletta Cozzi: Nothing to disclose
Carla Biasibetti: Nothing to disclose

### Research Presentation Session: Artificial Intelligence & Machine Learning

### RPS 405

### AI in neuroradiology

### RPS 405-1

**Towards a comprehensible convolutional neural network for the detection of Alzheimer's disease in magnetic resonance images**

*D. Cantré*, M. Dyrba, M. Hanzig, M-A. Weber, S. Teipel; Rostock/DE
*(daniel.cantre@med.uni-rostock.de)*

**Purpose:** Convolutional neural networks (CNN) achieve high diagnostic accuracy for detecting Alzheimer's disease (AD) dementia based on MRI scans. However, they are not yet applied in clinical routine, one reason being lack of suitable methods for model comprehensibility. Recently developed methods for deriving CNN relevance maps may facilitate model comprehensibility through visualization with high spatial resolution.
**Methods or Background:** We trained a CNN for the detection of AD in N=663 T1-weighted MPRAGE MRI scans of patients with AD or mild cognitive impairment (MCI) and evaluated the accuracy of the models via cross-validation, and in three further independent samples, including in total N=1655 cases. For intuitive model inspection, we implemented a web-interface with interactive visualization of 3D CNN relevance maps. Finally, we evaluated the association of relevance scores and hippocampus volume to validate the clinical utility of this approach.
**Results or Findings:** We reached excellent group separation for AD vs. controls with an AUC≥0.92 and lower results for MCI vs. controls with an AUC≈0.75 across three independent datasets. Relevance maps indicated that hippocampal atrophy was considered the most informative region for AD detection by the CNN, with additional contributions from atrophy in other cortical and subcortical regions. Relevance scores within the hippocampus were highly correlated with hippocampal volume, with a Pearson correlation of r≈-0.81.
**Conclusion:** The strong correlation of hippocampus relevance scores and hippocampus volume, as well as the performance achieved in independent samples, indicate a high validity of the CNN models. The relevance maps indicating discriminative brain regions, as visualized by our dedicated interface, highlighted atrophy in regions we expected a-priori. Given that the CNN models were learnt purely data-driven, based on the MRI scans and diagnosis labels, this strengthens the comprehensibility of the models.
**Limitations:** N.a.
**Ethics Committee Approval:** Positive
**Funding for this study:** N.a.
**Author Disclosures:**
Martin Dyrba: Nothing to disclose
Stefan Teipel: Nothing to disclose
Moritz Hanzig: Nothing to disclose
Marc-André Weber: Nothing to disclose
Daniel Cantré: Nothing to disclose

### RPS 405-4

**Detection of ischaemic stroke presence and classification of territorial type in diffusion-weighted MRI by deep learning**

*I. Ö. Koska*, Y. K. K. Çetinoğlu, M. E. Uluç, M. A. Selver, M. F. Gelal; Izmir/TR
*(ozgurkoska@icloud.com)*

**Purpose:** Experimenting with ischemic stroke detection and territorial classification task using our own institutional data by using deep learning
**Methods or Background:** A total of 421 diffusion magnetic resonance images of 271 patients with acute stroke and 150 normal cases obtained between January 2017 and April 2020 were included in the study. A stroke diagnosis dataset was created with 900 slices with stroke and 900 normal slices. In the classification of stroke according to vascular territories, 1717 positive slices are obtained and divided into three classes as middle cerebral artery (MCA) infarction, posterior circulation (PC) infarction, and border zone (BZ) infarction. MobileNetV2 and EfficientNet-B0 CNN architectures were selected for the training of datasets.
**Results or Findings:** In the diagnosis of ischemic stroke, an accuracy of 96% was achieved with MobileNetV2 and 93% with EfficientNet-B0 . In the classification of stroke as MCA, PC or BZi, 93% accuracy was achieved with the MobileNetV2 and 87% with the EfficientNet-B0 . Sensitivity, specificity and PPV of MobileNetV2 were 98%, 93.5%, and 88.29% for MCA infarction group, 92%, 98.5%, and 96.84% for PC infarction group, 89%, 97,5%, and 94,68% for BZ infarction group, respectively. Sensitivity, specificity and PPV of EfficientNet-B0 were 98%, 93.5%, and 88.29% for MCA infarction, 92%, 98.5%, and 96.84% for PC infarction, 89%, 97,5%, and 94,68% for BZ infarction, respectively.
**Conclusion:** This study showed that CNN models are successful methods that can be used both in the detection of acute stroke and stroke classification according to vascular territories.
**Limitations:** All the datasets were obtained from single institution, this may introduce some selection bias. Annotation of the data is a labor intensive process. In the limited time settings we could not obtain ACA infarcts segmentation maps.
**Ethics Committee Approval:** Not applicable
**Funding for this study:** Not applicable
**Author Disclosures:**
Mustafa Alper Selver: Nothing to disclose
Yusuf Kenan KENAN Çetinoğlu: Nothing to disclose
Ilker Özgür Koska: Nothing to disclose
Mustafa Fazil Gelal: Nothing to disclose
Muhsin Engin Uluç: Nothing to disclose

### RPS 405-5

**Multivariable diagnostic prediction model to detect hydrocephaly from T2-weighted MRI radiomics and conventional features with artificial neural networks in colloid cysts**

B. Hoşgören Atalay, *M. B. Doğan*, M. B. Eser; Istanbul/TR
*(mbilaldogan@hotmail.com)*

**Purpose:** We aim to train neural networks to detect hydrocephaly in patients with colloid cyst based on T2 weighted MRI radiomics.
**Methods or Background:** This retrospective model development study included a cohort of 40 patients with colloid cysts in one tertiary university hospital. The mean patient age was 54.08±16.57 years, and 25/40 (62.5%) were women. We segmented cysts on axial T2 weighted MRI, and evaluated conventional features based on the colloid cyst risk score (i.e., patient age, headache, FLAIR hyperintensity, dimensions, location of the cyst). Predictors were identified as radiomics features (n = 851) and conventional features (n = 10). Feature selection was based on coefficient variance (CoV), variance inflation factor (VIF), and LASSO regression analysis. The outcome was identified as hydrocephaly (11/40, 27.5%). Models were developed with artificial neural networks (ANN) with three diagnostic prediction models. The first model included radiomics features, the second model included conventional features, and the third model included all of the features. Internal validation was performed using bootstrapping and cross-validation.
**Results or Findings:** The 779 of 851 features were eliminated with CoV analysis (> 0.15). Another 23 features were eliminated by VIF analysis due to collinearity. Most of the radiomics features were found to be unstable (n = 802, 94%). Conventional features detected hydrocephaly with AUC = 0.87, p < 0.001, sensitivity: 82%, specificity: 93%. Radiomics predicts hydrocephaly with AUC = 0.88, p < 0.001, sensitivity: 92%, specificity: 97%. Third model's AUC was 0.99, p <0.001, sensitivity: 91%, specificity: 100%.
**Conclusion:** This study was successful in training neural networks that can detect hydrocephaly on patients with colloid cysts.
**Limitations:** The small sample size is the limitation of this study. Studies involving a larger population are required to confirm these results fully.
**Ethics Committee Approval:** n/a
**Funding for this study:** n/a
**Author Disclosures:**
Başak Hoşgören Atalay: Nothing to disclose
Mehmet Bilgin Eser: Nothing to disclose
Mahmut Bilal Doğan: Nothing to disclose

### RPS 405-6

**The predictive power of MRI VASARI features for identifying the mutation status of multiple genes in glioblastoma using machine learning algorithms**

*A. Haddadi Avval*[1], G. Hajianfar[2], S. Mostafaei[3], M. Oveisi[4], I. Shiri[5], H. Zaidi[5]; [1]Mashhad/IR, [2]Tehran/IR, [3]Kermanshah/IR, [4]Vancouver, BC/CA, [5]Geneva/CH
*(atlas.ha78@gmail.com)*

**Purpose:** Several gene mutations have been shown to be associated with the prognosis of patients with glioblastoma multiforme (GBM). We aimed to evaluate the predictive power of MRI-based features, named Visually Accessible Rembrandt Images (VASARI) in the mutational status of the tumor genes using machine learning (ML).
**Methods or Background:** MR images of 188 patients along with their demographic and clinical data (e.g. tumor biopsy results and mutation status of five genes) were obtained. The data were randomly split into 80% and 20% as training and testing datasets, respectively. We defined a set of 54 features utilizing VASARI features. the selected features with the most important values and highest rankings were depicted using principal component analysis (PCA), Random Forest, support vector machine (SVM), and the Chi-square test. Our multivariate analysis involved 16 different ML algorithms applied to the whole feature set to extract the area under the ROC curve (AUC), sensitivity, and specificity for assessing models' performance.
**Results or Findings:** The enhancement quality, proportion enhancing, and lesion size feature appeared as the most frequent among our ranking lists generated through feature selection methods in the mutation prediction of each five genes. Among ML classifiers, SVM (AUC: 0.68), QDA (AUC: 0.60), AdaBoost (AUC: 0.70), MLP (AUC: 0.68), and Decision Tree (AUC: 0.66) classifiers obtained the highest AUC for the prediction of the mutation status in ATRX, CIC, EGFR, TP53 and 1p19q deletion status, respectively.
**Conclusion:** Our study demonstrated that machine learning helps in the detection of mutant genes in GBM tumors. Understanding tumor genomics enables better management and prognostication of patients with glioblastoma.
**Limitations:** Our limitations include a small sample size and a lack of external validaiton.
**Ethics Committee Approval:** Not applicable
**Funding for this study:** Swiss National Science Foundation under grant SNRF 320030_176052
**Author Disclosures:**
Shayan Mostafaei: Nothing to disclose
Atlas Haddadi Avval: Nothing to disclose
Ghasem Hajianfar: Nothing to disclose
Habib Zaidi: Nothing to disclose
Mehrdad Oveisi: Nothing to disclose
Isaac Shiri: Nothing to disclose

### RPS 405-7

**Application of deep learning for a reliable MRI-based diagnosis for progressive supranuclear palsy (PSP)**

J-E. Meissner[1], P. Mann[1], H. J. Michaely[2], *J. R. Opalka*[1], A. Lemke[1]; [1]Berlin/DE, [2]Karlsuhe/DE
*(j.opalka@mediaire.de)*

**Purpose:** To quantify the performance of a new Deep Learning (DL)-based algorithm in differentiating Progressive Supranuclear Palsy (PSP) patients from healthy individuals and patients with Parkinson's disease (PD).
**Methods or Background:** Quantitative brain volumetry was carried out with our new DL-based algorithm. It was tested on 248 patients that consist of 221 healthy patients (age 61.3y±10.1y, source: Parkinson's Progression Markers Initiative, PPMI), 46 patients with confirmed PD (age 64.2y±8.7y, source: PPMI) and 11 patients with confirmed PSP (age 68.8y±8.4y, source: internal). Images were acquired on 1.5T/3.0T MR-scanner using 3D-T1w images. Brain volumetry and quantitative comparison against a normal model was performed for: whole brain, grey&white matter, frontal, parietal, occipital, temporal lobe, hippocampus, mesencephalon, pons and all ventricles. Furthermore, the midbrain-to-pons-ratio (MtPR) was calculated in 3D. Performance was tested with respect to the algorithm's ability to correctly identify (i) PSP against healthy patients and (ii) PSP against those with confirmed PD based on percentiles. For that, ROC was used to calculate the corresponding AUC and sensitivity/specificity on the best performing regions.
**Results or Findings:** In the case of (i), calculations yielded highest AUC for: mesencephalon (0.944+/-0.049) and 3D MtPR (0.896+/-0.129) while for (ii), calculations yielded highest AUC for: mesencephalon (0.915+/-0.084) and the occipital lobe (0.893+/-0.085). Resulting sensitivity/specificity calculations on the best performing region yielded values of 0.91/0.89 for (i) and of 0.91/0.87 for (ii).
**Conclusion:** We successfully applied a DL approach to correctly identify PSP patients against healthy and PD patients. Our algorithm is comparable to the conventional 2D segmentation where both a reduced mesencephalon and MtPR are signs for PSP (NEUROLOGY_2005;64:2050–2055). In combination with a fast evaluation (<4mins) our algorithm is a promising tool to aid in the diagnosis of PSP in clinical routine.
**Limitations:** No
**Ethics Committee Approval:** No
**Funding for this study:** No
**Author Disclosures:**
Jan-Eric Meissner: Employee: mediaire GmbH
Philipp Mann: Employee: mediaire GmbH
Jens Ruediger Opalka: Employee: mediaire GmbH
Andreas Lemke: CEO: mediaire GmbH
Henrik J Michaely: Shareholder: mediaire GmbH

### RPS 405-8

**Aneurysmal subarachnoid haemorrhage: deep learning assessment vs radiologist in aneurysm size and volume**

*L. Pennig*, U. C. I. Hoyer, C. E. S. Zaeske, R. Shahzad, M. Perkuhn, M. Schlamann, C. Kabbasch, J. Borggrefe, L. Goertz; Cologne/DE
*(lenhard.pennig@uk-koeln.de)*

**Purpose:** To investigate the potential clinical performance of deep learning enabled fully automatic segmentation of aneurysms in aneurysmal subarachnoid hemorrhage (aSAH) on computed tomography angiography compared to radiologists.
**Methods or Background:** In this retrospective single-center study, 3 deep learning models (DLMs) were trained on 68 patients with 79 aneurysms treated for aSAH (2016-2017) with their outputs being combined using ensemble learning (DLM-Ens). Further, the DLM-Ens was evaluated on an independent test set of 104 patients treated for aSAH between 2013 and 2015 on which it performed 3D voxel-wise segmentations of aneurysms. Two radiologists with four (R1) and two years (R2) of experience in diagnostic neuroradiology manually measured the largest diameter of aneurysms in the test set in three dimensions. Correlations between manual measurements and deep learning generated segmentations were calculated using Pearson's correlation coefficient (r).
**Results or Findings:** In the test set, the DLM-Ens segmented 105 of 126 aneurysms (detection sensitivity of 83%) accurately. Considering the segmented aneurysms, there was a strong correlation for maximum diameter of aneurysms between the DLM (mean 6.2 ± 5.4 mm) and manual measurements (R1: 6.4 ± 2.6 mm, r=0.739; R2: 6.6 ± 2.5 mm, r=0.751), which was slightly lower than correlation between readers (r=0.868). Aneurysm volume correlated even stronger between deep learning generated segmentations (mean 124.6 ± 182.9 mm3) and volumes calculated based on manual measurements (R1: 132.1 ± 185.9 mm3, r=0.898; R2: 134.7 mm3, r=0.911), which was comparable to interreader correlation (r=0.934).
**Conclusion:** Compared to human measurements, deep learning provides strong correlation of maximum diameter and volume of aneurysms. Hence, it may be of valuable assistance to the treating physician in patients with aSAH, for which accurate 3D geometrical characterization of aneurysms is required for treatment planning.
**Limitations:** Retrospective.
**Ethics Committee Approval:** Approved.
**Funding for this study:** N/a.
**Author Disclosures:**
Rahil Shahzad: Employee: Philips Healthcare
Christoph Kabbasch: Nothing to disclose
Lenhard Pennig: Research/Grant Support: Philips Healthcare
Charlotte Elisabeth Sigrid Zaeske: Nothing to disclose
Jan Borggrefe: Speaker: Philips Healthcare
Ulrike Cornelia Isabel Hoyer: Nothing to disclose
Lukas Goertz: Nothing to disclose
Marc Schlamann: Nothing to disclose
Michael Perkuhn: Research/Grant Support: Philips Healthcare

### RPS 405-9

**Clinical performance of a deep learning model for detection of aneurysms in subarachnoid haemorrhage compared to human readers**

*L. Pennig*[1], U. C. I. Hoyer[1], A. Krauskopf[2], R. Shahzad[1], M. Perkuhn[1], M. Schlamann[1], C. Kabbasch[1], J. Borggrefe[1], L. Goertz[1]; [1]Cologne/DE, [2]Düsseldorf/DE

**Purpose:** To investigate the potential clinical use of a deep learning model (DLM) for fully automatic detection of aneurysms in patients with aneurysmal subarachnoid hemorrhage (aSAH) on computed tomography angiography by comparing and merging its diagnostic performance with human readers.
**Methods or Background:** In this retrospective single-center study, three different DLMs based on DeepMedic were trained on 68 patients with 79 aneurysms treated for aSAH (2016-2017) with their outputs being combined applying ensemble learning (DLM-Ens). The DLM-Ens was evaluated on an independent test set consisting of consecutive patients treated for aSAH between 2013 and 2015. Ground truth (GT) of the test set without annotations was presented to three blinded radiologists (reader 1: 13, reader 2: 4, and reader 3: 3 years of experience in diagnostic neuroradiology).
**Results or Findings:** One-hundred-and-twenty-six aneurysms (mean diameter: 6.1±5.4 mm) of 104 patients (mean age: 55.4±14.3 years, 66 females) comprised the GT of the test set. With a sensitivity of 83.3%, the DLM-Ens achieved to human readers (reader 1: 91.2%, reader 2: 86.5%, and reader 3: 86.5%; P=.313) comparable sensitivity, which showed a moderate interrater agreement (Fleiss κ of 0.502). When combining the findings of human readers with deep learning generated detections, sensitivity increased to nearly 100% (reader 1: 97.6%, reader 2: 97.6%, and reader 3: 96.0%; P=.024) resulting in an almost perfect interrater agreement (Fleiss κ of 0.878).
**Conclusion:** In aSAH, DLM-Ens detects aneurysms with to human readers comparable sensitivity and significantly increases their detection rate to almost 100% while leading to an almost perfect interrater agreement. Hence, it provides a valuable adjunct to physicians' performance in emergency imaging requiring fast and accurate diagnosis to optimize patient treatment.
**Limitations:** Retrospective.
**Ethics Committee Approval:** Approved.
**Funding for this study:** N/a.
**Author Disclosures:**
Rahil Shahzad: Employee: Philips Healthcare
Christoph Kabbasch: Nothing to disclose
Lenhard Pennig: Research/Grant Support: Philips Healthcare
Jan Borggrefe: Speaker: Philips Healthcare
Alexandra Krauskopf: Nothing to disclose
Ulrike Cornelia Isabel Hoyer: Nothing to disclose
Lukas Goertz: Nothing to disclose
Marc Schlamann: Nothing to disclose
Michael Perkuhn: Employee: Philips Healthcare

### RPS 405-11

**K-space based deep learning reconstruction empowers 50% acceleration of MR spine imaging: a prospective multicentre multireader trial**

*L. N. Tanenbaum*[1], S. Bash[2], M. Thomas[3], M. Fung[3], M. Lebel[3]; [1]New York, NY/US, [2]Woodland Hills, CA/US, [3]Waukesha, WI/US
*(nuromri@gmail.com)*

**Purpose:** This prospective, multicenter, multireader study evaluates the impact on perceived image quality of 50% scan-time reduced spine MRI reconstructed with deep learning (DL).
**Methods or Background:** With IRB approval and patient consent, 50 consecutive patients underwent standard-of-care (SOC) and accelerated (FAST) spine MRI exams acquired from a GE 3T Architect scanner. DL processing of the FAST scan data set (FAST-DL) was performed using an FDA-cleared CNN based, DL image enhancement product - Air Recon DLTM. The k-space based tool offers powerful denoising, sharpness enhancement and elimination of some artifacts such as truncation ringing. Two neuroradiologists were presented with the different image series as paired side-by-side datasets. Datasets were blinded and randomized in sequence and left-right display order. Image features were preference rated on a 5-point Likert scale.
**Results or Findings:** FAST-DL was qualitatively better than SOC for perceived signal-to-noise ratio (SNR), sharpness and artifacts. FAST-DL and SOC were better than FAST for all assessed features. No anatomic aberration or data loss was observed on the DL processed images.
**Conclusion:** DL enables 50% spine MRI scan time reduction as well as what radiologists perceive as enhanced image quality with benefits in SNR, image sharpness, and artifact reduction over SOC and FAST images without DL processing, providing gains in efficiency and portending practice utility for routine use.
**Limitations:** Small sample size. Limited numbers of cervical and thoracic exams.
**Ethics Committee Approval:** IRB approved.
**Funding for this study:** None
**Author Disclosures:**
Marc Lebel: Employee: GE Heathcare
Mary Thomas: Employee: GE Heathcare
Suzie Bash: Nothing to disclose
Lawrence N Tanenbaum: Speaker: GE Heathcare
Maggie Fung: Employee: GE Heathcare

### RPS 405-12

**Deep learning enables 60% accelerated volumetric brain MRI while preserving quantitative performance: a prospective multicentre multireader trial**

*S. Bash*[1], C. Airriess[2], L. Wang[3], G. Zaharchuk[4], E. Gong[3], T. Zhang[3], S. Dupont[3], A. Shankaranarayanan[3], L. N. Tanenbaum[1]; [1]Woodland Hills, CA/US, [2]San Diego, CA/US, [3]Menlo Park, CA/US, [4]Stanford, CA/US
*(suzie.bash@radnet.com)*

**Purpose:** Evaluating the impact on both image quality and quantitative consistency (NeuroQuant™) of 60% accelerated volumetric MRI scans processed with a DICOM-based deep learning (DL) tool (SubtleMR™) compared to standard of care (SOC).
**Methods or Background:** 40 subjects underwent brain MRI on 6 scanners (5 institutions). SOC and accelerated (FAST) datasets were acquired for each subject. FAST scans were enhanced with DL processing (FAST-DL). Both SOC and FAST-DL were subjected to quantitative analysis with NeuroQuant™ and classification by a neuroradiologist into disease categories. Quantitative biomarker concordance of SOC and FAST-DL was assessed. For subjective assessment, randomized, side-by-side, multiplanar datasets (360 series) were presented blinded to 2 neuroradiologists. Images were preference rated on a 5-point Likert scale for apparent signal-to-noise ratio, image sharpness, artifacts, lesion conspicuity, image contrast, and gray-white differentiation.
**Results or Findings:** FAST-DL was superior to SOC for quality across all features despite 60% scan time reduction (p<0.05). Both FAST-DL and SOC were superior to FAST for all quality features. There was high inter-reader agreement of quality ratings. Paired T-test analysis demonstrated excellent agreement of quantitative data between the SOC and FAST-DL datasets. Linear Regression and Bland-Altman analysis demonstrated strong concordance in quantitative values across conditions (normal, MCI, Alzheimer's). There was 100% agreement in disease classification for the SOC and FAST-DL datasets (n=29 normal/MCI and n=11 dementia).
**Conclusion:** DL reconstruction allows 60% scan time reduction while maintaining high volumetric quantification accuracy, consistent clinical classification, and superior image quality compared with SOC. This trial supports the reliability, efficiency, and utility of DL based enhancement for quantitative imaging. Shorter scan times may boost utilization of volumetric quantitative MRI in routine clinical settings.
**Limitations:** Evaluation of only one quantitative imaging product. Small sample size.

**Ethics Committee Approval:** IRB approved
**Funding for this study:** Subtle Medical and Cortechs
**Author Disclosures:**
Tao Zhang: Employee: Subtle Medical
Greg Zaharchuk: Founder: Subtle Medical
Enhao Gong: Founder: Subtle Medical
Suzie Bash: Advisory Board: Subtle Medical and Coretechs
Long Wang: Employee: Subtle Medical
Chris Airriess: CEO: Coretechs
Lawrence N Tanenbaum: Advisory Board: Subtle Medical
Sara Dupont: Employee: Subtle Medical
Ajit Shankaranarayanan: Employee: Subtle Medical

### RPS 405-13

**Automated segmentation and reconstruction of the optic nerves and chiasm in MR images using deep learning**

*Y. Zhang*, C. Chen, J. Xu; Chengdu/CN

**Purpose:** To develop and evaluate a novel automatic algorithm for the segmentation and reconstruction of the optic nerves and chiasm from MR images.
**Methods or Background:** In this retrospective study, a convolutional neural network based on the 3D U-Net architecture was trained in a data cohort of 4800 three-dimensional T2-weighted images and was validated in 800 MR images. Optic nerves and chiasm were manually segmented by experienced radiologists for comparison. Performance of the automatic segmentation was evaluated with Dice coefficient. Volume rendering was employed to automatically reconstruct the optic nerves and chiasm.
**Results or Findings:** Compared with the manual segmentation, the 3D U-Net model represented feasible performances with Dice coefficients of 0.75 in the validation set. Moreover, the volume rendering model reconstructed the optic nerves and chiasm with the sophisticated surface and clearly defined structures.
**Conclusion:** A novel and accurate method based on deep learning technology has been proposed for the automatic segmentation and reconstruction of the optic nerves and chiasm in MR images.
**Limitations:** There are several limitations in the present study. First, the study cohort was relatively small. This study is currently being continued with larger MR images datasets, and it is expected to generate more accurate and robust models. Second, the MR images included were all collected from a single institution, and our results need to be validated in external datasets in the future.
**Ethics Committee Approval:** This retrospective study was approved by Ethics Committee of Sichuan University.
**Funding for this study:** This work was supported by 1.3.5 project for disciplines of excellence, West China Hospital, Sichuan University (ZYJC18007); Key research and development project of science and technology department of Sichuan Province (2019YFS0392).
**Author Disclosures:**
Yang Zhang: Nothing to disclose
Chaoyue Chen Chaoyue Chen: Nothing to disclose
Jianguo Xu: Nothing to disclose

### RPS 405-14

**Multiparametric deep learning model segmentation for glioblastoma on multi-institutional clinical trial data sets: feasibility, opportunities and challenges**

*M. V. Knopp*[1], R. Shahzad[2], C. Nealer[1], R. Jacko[1], P. Robson[1], J. Gorski[1], M. M. Knopp[3], M. Ahrens[4], M. Perkuhn[2]; [1]Columbus, OH/US, [2]Cologne/DE, [3]Munich/DE, [4]Aachen/DE
*(knopp.16@osu.edu)*

**Purpose:** To assess the feasibility of deep-learning model based automated glioblastoma tumor segmentation algorithms on large multi-institutional aggregated clinical trial data sets. To demonstrate both opportunities as well as challenges for the assessment in multi-site clinical trials.
**Methods or Background:** We serve as the imaging core service (IROC) for the NCI NCTN to collect, curate imaging studies and support utilization of analytical tools. Within this secure framework, coded brain MRI imaging studies acquired in several neuro-oncologic clinical trials of patients diagnosed with glioblastoma (GB) were aggregated and 3423 imaging timepoints included. Using a scalable environment orchestrated via a portal system several deep learning models (DLM) utilizing T1w, T2w, FLAIR, and CE T1w images with co-registration, brain mask segmentation, normalization and resampling, segmented the GB tumor compartments.
**Results or Findings:** Inconsistencies and variability in MR acquisition and labeling were extensive. All trained DLM tools were successfully applied. A multi reader review assessed the segmentation performance using a likert scale and feature descriptors. Performance of the tools were consistently lower than reported by the developers. Evaluating DLM tools on diverse multi-center based dataset appears essential to confirm technical feasibility and performance.
**Conclusion:** The use of DLM model for segmentation of glioblastoma tumor features is feasible even in highly diverse large-scale data sets of multi-institutional trials. More consistent MRI acquisitions and labeling is essential and can be ensured by appropriate QC approaches. The ability to upscale DLM tools for glioblastoma segmentation to a national clinical trial environment was demonstrated.
**Limitations:** DLM based segmentation dependent on the quality of the original annotations and data sets. This highlights that input QC as well as data diversity for development is essential and should be disclosed.
**Ethics Committee Approval:** Approved 2017H0429, 2013H0304 NCT03490656
**Funding for this study:** ODSA TECH 13-060 Award, NCI 5U24CA180803
**Author Disclosures:**
Rahil Shahzad: Employee: Philips
Conner Nealer: Nothing to disclose
Richard Jacko: Nothing to disclose
Marcel M Knopp: Nothing to disclose
Justin Gorski: Nothing to disclose
Parker Robson: Nothing to disclose
Michael Vinzenz Knopp: Nothing to disclose
Martin Ahrens: Employee: Philips
Michael Perkuhn: Employee: Philips

### RPS 405-2

**Volumetric analysis of tumour compartments on longitudinal MRI of glioblastoma patients using deep learning**

*M. Perkuhn*, R. Shahzad, F. Thiele, M. Schlamann, C. Kabbasch, J. Borggrefe; Cologne/DE
*(michael.perkuhn@uk-koeln.de)*

**Purpose:** In patients with glioblastoma (GB), MRI is essential for diagnosis, treatment planning and treatment monitoring. Volumetric assessment of tumor burden is expected to improve patient management. However, manual segmentation is time-consuming and suffers from high interrater-variability. This study aimed to evaluate deep learning models (DLM's) for fully-automatic GB segmentation over the course of treatment.
**Methods or Background:** 332 studies from 41 subjects were included, each subject had a pre-operative, a post-operative, and multiple follow-up studies. All studies comprised T1, T2, FLAIR, and contrast-enhanced (CE) T1 sequences. Edema (ED), contrast-enhancing tumor (CET), necrosis (NC) and tumor-cavity (TC) were manually delineated by experts using consensus reading. Multi-parametric, multi-label, 3D CNN DLM's were trained and evaluated according to treatment stage and CET volumes.
**Results or Findings:** On pre-operative studies the average CET volume was 27.3±18.2ml. The DLM detected CET with accuracy=100% and dice=0.78±0.15; ED with accuracy=100%, dice=0.86±0.1 and NC accuracy=85%, dice=0.56±0.31. Volume correlation (r) was 0.90(CET), 0.96(ED) and 0.84(NC). On post-operative studies 28 subjects had confirmed CE residual tumor (CE-RT) with a volume of 0.86±1.93ml. DLM detected CE-RT >0.5ml(n=16) with accuracy=82%; >1ml(n=12) accuracy=94%; >2ml(n=6) accuracy=96%, dice=0.43±0.25(CE-RT>2ml). ED with accuracy=100%, dice=0.75±0.16(ED) and TC accuracy=96%, dice=0.64±0.22(TC). Volume correlations (r) was 0.71(CE-RT), 0.97(ED) and 0.84(TC). On follow-up, 242 studies had confirmed CE recurrent tumors with a volume of 6.1±13.8ml. DLM detected CET >0.5ml(n=207) with accuracy=78%; >1ml(n=168), accuracy=76%; >2ml(n=113) accuracy=91%, dice=0.53±0.22(CET>2ml). ED with accuracy=100%, dice= 0.66±0.19 and TC/NC accuracy=91%, dice=0.48±0.27. Volume correlations (R) were 0.80(CET), 0.91(ED), 0.76(TC/NC).
**Conclusion:** Fully-automatic assessment of GB tumor compartments on routine clinical data is possible with high correlation to manually traced volumes. This approach could overcome the limitations of two-dimensional tracking and improve the radiologist's workflow in GB assessment.
**Limitations:** n/a
**Ethics Committee Approval:** The local ethics committee approved this retrospective study
**Funding for this study:** n/a
**Author Disclosures:**
Rahil Shahzad: Employee: Philips Healthcare
Christoph Kabbasch: Nothing to disclose
Jan Borggrefe: Nothing to disclose
Frank Thiele: Employee: Philips Healthcare
Marc Schlamann: Nothing to disclose
Michael Perkuhn: Employee: Philips Healthcare

### Research Presentation Session: Artificial Intelligence & Machine Learning

### RPS 505

### AI in oncology

### RPS 505-2

**Spatio temporal risk prediction of focal bone lesion evolution in multiple myeloma**

*R. Licandro*[1], J. Hofmanninger[1], M. Perkonigg[1], S. Röhrich[1], M-A. Weber[2], M. Wennmann[3], L. Kintzelé[3], B. Menze[1], G. Langs[1]; [1]Vienna/AT, [2]Rostock/DE, [3]Heidelberg/DE
*(roxane.licandro@meduniwien.ac.at)*

**Purpose:** The earliest possible detection of focal bone lesions is key to facilitate timely treatment decisions in patients with Multiple Myeloma (MM). Here we propose a machine-learning based routine to assess the risk of MM precursor bone regions to evolve towards focal lesions within a definable timeframe in the future.
**Methods or Background:** Longitudinal T1 weighted whole body MRI (wbMRI) of 63 patients were acquired with overall 170 annotated locations, which evolved to either diffuse or focal lesions. Here, we propose a methodology, which first automatically segments bones in wbMRI and subsequently predicts future focal lesion progression within the detected bone. The proposed network architecture “Asymmetric Conditioned Cascade U-Net” identifies early signatures of emerging lesions in dependence of the prediction time and visualizes risk locations as full body risk score maps.
**Results or Findings:** We evaluated the segmentation and predictive performance in the thoracic (1) and pelvic/femoral (2) skeletal system. The bone segmentation performance reported a mean Dice Score of 0.67 (1) and 0.78 (2). Baseline scans predicted new lesions in the follow up scan with a mean AUC of 0.82 (1) and 0.96 (2) and progressive lesions with a mean AUC of 0.94 (1) and 0.96 (2), respectively.
**Conclusion:** We propose an evolution risk predictor for both new and progressive lesions based on a baseline scan and visualize associated high-risk locations accordingly. Our findings indicate hidden imaging markers beyond lesion size, currently used for categorization and risk stratification in MM, and that evolution trajectories of lesion progression can be encoded using the proposed network.
**Limitations:** We observed that partial volume artifacts and anomalous bone regions (without future lesion progression) are the main triggers for false positive predictions.
**Ethics Committee Approval:** Heidelberg EK
**Funding for this study:** DFG (WE 2709/3-1 and ME 3511/3-1) and FWF (I2714-B31)
**Author Disclosures:**
Georg Langs: Research/Grant Support: Novartis Pharmaceuticals Corporation Shareholder: contextflow GmbH Research/Grant Support: Siemens Healthineers
Bjoern Menze: Nothing to disclose
Sebastian Röhrich: Consultant: contextflow GmbH
Johannes Hofmanninger: Research/Grant Support: Siemens Healthineers
Matthias Perkonigg: Research/Grant Support: Novartis Pharmaceuticals Corporation
Marc-André Weber: Nothing to disclose
Markus Wennmann: Nothing to disclose
Laurent Kintzelé: Nothing to disclose
Roxane Licandro: Nothing to disclose

### RPS 505-3

**A multi-institutional comparative effectiveness analysis of AI-assisted vs. current practice methods for advanced cancer imaging evaluation**

*A. D. Smith*[1], B. Allen[2], A. Abou Elkassem[1], R. Mresh[1], S. Lirette[3], A. Farag[1], M. Anderson[1], A. M. Khalaf[1]; [1]Birmingham/US, [2]Durham, NC/US, [3]Jackson, MS/US
*(andrewdennissmith@uabmc.edu)*

**Purpose:** To compare the effectiveness of artificial-intelligence (AI)-assisted vs. current practice methods for advanced cancer longitudinal imaging response evaluation.
**Methods or Background:** For this multi-institutional retrospective study, 24 radiologists collectively interpreted body CT images in triplicate from 120 consecutive patients with serial imaging of advanced cancer treated by systemic therapy. Current-practice included manual tumor measurements and dictated text-based reports. For the AI-assisted method, custom software utilized AI for tumor measurement, anatomic labelling, and localization at follow up and automatically categorized tumor response per RECIST 1.1 and displayed longitudinal data in a graph, table, and key images. Oncologists (N=20) collectively reviewed 120 final reports in quadruplicate and categorized response. Comparative effectiveness metrics included: accuracy (agreement between oncologist and radiologist final response category), major errors, efficiency, and inter-observer agreement. A post-study survey of opinions was conducted.
**Results or Findings:** The accuracy of the report impression was 77% for current-practice and 100% for AI-assisted (p<0.001). Average percent of major errors per time point was 27.5% (99/360) for current-practice and 0.3% (1/360) for AI-assisted (p<0.001), corresponding to a 99% reduction. Average radiologist read times were 18.7 min for current-practice and 9.8 min for AI-assisted (p<0.001), with AI-assisted nearly twice as fast. Radiologist total agreement on final report response category was 52% (62/120) for current-practice and 75% (90/120) for AI-assisted (p<0.001), corresponding to a 45% relative increase in inter-reader agreement. 96% of radiologists and 100% of oncologists preferred the AI-assisted method and reporting.
**Conclusion:** AI-assisted imaging evaluation and reporting of advanced cancer longitudinal response is more effective than current-practice methods with manual measurements and text-based reports.
**Limitations:** Source of CT images was from a single institution.
**Ethics Committee Approval:** IRB-approved study
**Funding for this study:** Funded by the University of Alabama at Birmingham
**Author Disclosures:**
Rafah Mresh: Nothing to disclose
Ahmed M Khalaf: Nothing to disclose
Asser Abou Elkassem: Nothing to disclose
Michael Anderson: Nothing to disclose
Ahmed Farag: Nothing to disclose
Andrew Dennis Smith: Owner: Radiostics LLC Speaker: Canon Medical Speaker: GE Grant Recipient: General Electric Owner: AI Metrics LLC
Seth Lirette: Nothing to disclose
Brian Allen: Shareholder: AI Metrics LLC

### RPS 505-4

**Machine learning models applied to whole-body MRI in the staging of cancer: the MALIBO study**

*A. Fagan*[1], X. Li[1], S. A. Taylor[1], G. J. Wengert[2], E. Aboagye[1], M. Investigators[1], A. Rockall[1]; [1]London/UK, [2]Vienna/AT
*(faganai@tcd.ie)*

**Purpose:** Whole-body MRI (WB-MRI) has been shown to have similar accuracy to standard staging for lung and colon cancer, but is more efficient and cost effective. It is, however, not widely performed, and can be time consuming to report. The MALIBO study concerns the development of a machine learning (ML) model in order to aid detection of primary cancer and metastases on WB-MRI.
**Methods or Background:** The model chosen was a convolutional neural network (CNN), trained on 226 cases from the NIHR Streamline study. Expert and non-expert readers were provided with all available MRI sequences, and the cases were read in a random order, both with and without ML output.
**Results or Findings:** Clinical validation used 188 evaluable WB-MRI studies (including lung and colon cancer), 50 of which had metastatic disease. For experienced readers, per-patient specificity and sensitivity for detection was 86.2% and 66.0% with ML support, and 87.7% and 70% without support. For inexperienced readers, per-patient sensitivity with ML support was 73.3% and 60% without. None of these results were statistically significant. ML assistance reduced WB-MRI read time by 1.95 minutes (95%CI:-3.05,-0.85;p=0.001).
**Conclusion:** In this first large study on the use of WB-MRI with machine learning in the diagnosis and staging of colorectal and lung cancers, we have found a reduction in reading time with ML assistance. Interestingly, there was no difference in diagnostic test accuracy. This work represents a significant first step in the development of a complex machine learning algorithm for interpretation of WB-MRI in oncological imaging.
**Limitations:** The data set was heterogeneous (from 16 different centres). There was also a relatively low number of metastatic lesions, which limited the training data.
**Ethics Committee Approval:** ICREC reference: 15IC2647.
**Funding for this study:** Funding was from the NIHR (EME stream), funder reference number: 13/122/01
**Author Disclosures:**
Stuart Andrew Taylor: Nothing to disclose
Xingfeng Li: Nothing to disclose
Aisling Fagan: Nothing to disclose
Georg J Wengert: Nothing to disclose
Malibo Investigators: Nothing to disclose
Eric Aboagye: Nothing to disclose
Andrea Grace Rockall: Nothing to disclose

### RPS 505-5

**Magnetic resonance imaging (MRI) radiomic features to predict tumour aggressiveness and outcomes in patients with endometrial cancer (EC)**

*J. Russell*, X. Li, D. Marcus, N. Soneji, S. Ghaem-Maghami, I. Lavdas, E. Aboagye, N. Bharwani, A. Rockall; London/UK

**Purpose:** The purpose of this study is to determine whether MRI radiomic features can predict tumour aggressiveness and outcomes in patients with EC, thus determining treatment selection in a clinical setting.
**Methods or Background:** In this ethically approved retrospective cohort study, a total of 445 patient scans were evaluated and divided into training and validation sets (80:20). Tumour volumes were manually segmented from T2-weighted sagittal MR images using the image segmentation tool ITK-SNAP. Radiomic features were extracted and delineated to predict (i) type, grade and stage of tumour, and (ii) overall survival and recurrence of EC. Twenty machine learning algorithms were used to classify the features. Stratified five-fold cross validation was used to calculate accuracy and determine the best model.
**Results or Findings:** In total, 969 radiomic features were extracted. Prediction of high-risk EC was 0.73 using a K(=5)nearest neighbours classifier (KNN) model. Prediction of EC type, grade (high versus low) and LVSI were 0.80, 0.81 and 0.77 respectively. Five-year survival was predicted with almost 80% accuracy. 139 features (16 following least absolute shrinkage and selection operator (LASSO) regression) were significantly associated with progression-free survival.
**Conclusion:** Results demonstrate that radiomic features derived from preoperative MRI studies are significantly associated with high-risk disease and reduced survival in EC. Prospective validation of this model will be required with larger datasets. It is hoped that prediction of high-risk histological features, overall survival and recurrence in EC patients will translate into better treatment selection.
**Limitations:** Several limitations of the study include a lack of standardisation of MR scan parameters as well as intra- and inter-observer variability associated with manual image segmentation. Five-year survival is also influenced by multiple patient and treatment factors that cannot be accounted for by radiomic feature analysis.
**Ethics Committee Approval:** Granted
**Funding for this study:** None
**Author Disclosures:**
Ionnis Lavdas: Nothing to disclose
Neil Dev Raju Soneji: Nothing to disclose
Xingfeng Li: Nothing to disclose
James Russell: Nothing to disclose
Sadaf Ghaem-Maghami: Nothing to disclose
Diana Marcus: Nothing to disclose
Eric Aboagye: Nothing to disclose
Andrea Grace Rockall: Nothing to disclose
Nishat Bharwani: Nothing to disclose

### RPS 505-6

**Differentiating between invasive and non-invasive breast carcinomas in digital breast tomosynthesis using deep convolutional neural networks**

*D. Shimokawa*[1], K. Shibuya[1], K. Takahashi[1], T. Usuzaki[1], M. Kadowaki[1], T. Fukuda[2], K. Oba[2], E. Takaya[2], T. Ueda[1]; [1]Sendai/JP, [2]Tokyo/JP
*(lucklmn7@icloud.com)*

**Purpose:** To develop a deep convolutional neural network to differentiate invasive and non-invasive breast carcinoma from digital breast tomosynthesis (DBT).
**Methods or Background:** Four hundred eighty-nine patients who were suggested breast carcinoma on screening mammography, admitted and performed DBT at St. Luke's International Hospital from 2019/03/01-2019/8/31 were enrolled. From 489 enrolled patients, 187 patients who were proved presence of breast carcinoma by needle biopsy and were performed surgery were included in this study. Pathological results revealed 25% and 75% of the patients showed non-invasive and invasive breast carcinoma, respectively. Xception (F. Chollet et al. IEEE Conference on Computer Vision and Pattern Recognition, 2017, pp. 1800-1807) was used for the AI model to differentiate non-invasive and invasive breast carcinoma from DBT images. To assess performance of the model, we used 5-fold cross-validation where 80% and 20% data was applied for training and validation, respectively. In cross-validation assessment, the dataset was randomly shuffled in each training process. Each training process of cross-validation was repeated 5 times and the average validation accuracy was calculated.
**Results or Findings:** Average of the validation accuracy showed 0.902 (max: 0.948, min: 0.850).
**Conclusion:** Our research suggested AI model can differentiate non-invasive and invasive breast carcinoma from DBT images, which is significantly associated with outcome of breast carcinoma patients.
**Limitations:** This study only enrolled limited number of patients.
**Ethics Committee Approval:** The institutional review board of Tohoku University approved this study and waived the requirement for informed consent.
**Funding for this study:** This work was supported by JST CREST Grant Number 17-171024528, Japan.
**Author Disclosures:**
Ken Oba: Nothing to disclose
Eichi Takaya: Nothing to disclose
Daiki Shimokawa: Nothing to disclose
Takuya Ueda: Nothing to disclose
Toshinori Fukuda: Nothing to disclose
Mizuki Kadowaki: Nothing to disclose
Kiichi Shibuya: Nothing to disclose
Takuma Usuzaki: Nothing to disclose
Kengo Takahashi: Nothing to disclose

### RPS 505-7

**Differentiation of solid renal masses based on radiomic features from contrast-enhanced CT scan: a retrospective study**

C. Mosquera, *M. Aineseder*, F. N. Diaz, R. Lopez Grove, A. Beresnak, S. Benitez, A. Jurado, P. Garcia Marchiñena, J. A. Ocantos; Buenos Aires/AR
*(martina.aineseder@hospitalitaliano.org.ar)*

**Purpose:** To develop and evaluate the performance of radiomic features processed with ML model for oncocytoma (ONC) and renal carcinoma (RCC) differentiation.
**Methods or Background:** We included histologically proven cases of RCC and ONC from Jan/2015 to Dec/2017, from patients who underwent surgical resection and had a preoperative contrast-enhanced computed tomography scan acquired with standard protocol in unenhanced, corticomedullary, nephrographic, and excretory phases. For every mass we obtained a segmentation mask in each contrast-phase and extracted 70 radiomic features from each mask, obtaining a total of 280 features per mass. Patients were split into two independent sets: training(85%) and test(15%). The training set was used to adjust four Random Forest classification models corresponding to the four contrast phases. We selected the best model by a hyperparameter grid search with 5-folds cross-validation on the training set. We calculated diagnostic metrics on the test set, using the histological tumor type as a reference standard to assess performance.
**Results or Findings:** Final dataset:104 RCC and 58 ONC, from 159 patients. We observed that the median and the skewness of gray-level distribution across the renal mass have a high influence on the model prediction. We evaluated the mean prediction score on 23 test samples, calculated as the average over model outputs from the available contrast-phases on each sample. It showed an area under the curve of 0.96, with a sensitivity 1.00 and specificity 0.83 for RCC diagnosis.
**Conclusion:** The differentiation between ONC/RCC is a challenging clinical task that carries the risk of delivering unnecessary surgical procedures. The technique presented in this study could become a reliable non-invasive method to characterize renal lesions that would have relevant clinical value.
**Limitations:** Retrospective study
**Ethics Committee Approval:** Ethics committee approved.
**Funding for this study:** This study had no funding from any resource.
**Author Disclosures:**
Facundo Nahuel Diaz: Nothing to disclose
Alberto Jurado: Nothing to disclose
Jorge Alberto Ocantos: Nothing to disclose
Sonia Benitez: Nothing to disclose
Roy Lopez Grove: Nothing to disclose
Martina Aineseder: Nothing to disclose
Alejandro Beresnak: Nothing to disclose
Patricio Garcia Marchiñena: Nothing to disclose
Candelaria Mosquera: Nothing to disclose

### RPS 505-8

**Advanced deep learning approach to automatically segment malignant tumours and ablation zone in liver with contrast-enhanced CT**

X. Liu[1], K. He[1], *R. Shahzad*[2], R. P. Reimer[2], F. Thiele[2], J. Niehoff[2], A. Bunck[2], H. Zhang[1], M. Perkuhn[2]; [1]Changchun/CN, [2]Cologne/DE
*(rahil.shahzad@uk-koeln.de)*

**Purpose:** To develop an automatic deep learning based method for (1)segmentation of liver tumors in arterial and venous phase for pre-treatment CT images, and (2)segmentation of liver and ablation zones in arterial and venous phase for CT images after ablation treatment. Automatic and robust segmentation of liver tumors and ablation zones would enable the evaluation of treatment success
**Methods or Background:** 252 CT images from 63 patients undergoing liver tumor ablation were included, four CT images for each patient (pre-treatment and post-treatment arterial/venous-phase). Ground truth segmentations for the liver, tumors and ablation zone were manually performed by an experienced radiologist. Data was split into a training-set (48 patients) and test-set (15 patients). A 2D residual-attention Unet (RA-Unet) was trained to segment the liver. Then, a multi-scale patch-based 3D RA-Unet was trained for segmentation of tumor and ablation zone. Image augmentations were used to increase the amount of training samples. Transfer learning approach was applied to models pre-trained on 131 CT images from the MICCAI2017(LiTS).
**Results or Findings:** On the independent test-set, the proposed method achieved an average dice of 0.96 and 0.92 for liver segmentation on arterial and venous phase, respectively . For tumor segmentation, the average dice was 0.69. For tumors >0.5cm3, sensitivity=79%, FP's/scan=0.4, dice=0.65 in arterial phase; sensitivity=86%, FP's/scan=0.6, dice=0.72 in venous phase. Average volume of FP's was 1.63cm3, average volume of FN's was 1.10 cm3. For ablation zone segmentation, sensitivity=90%, dice=0.83 in arterial phase, sensitivity=90%, dice=0.89 in venous phase.
**Conclusion:** The proposed deeplearning approach can provide automated segmentation of liver tumors and ablation zones on multi-phase (arterial and venous) and multi-time-point (before and after treatment) CT images, enabling quantitative evaluation of treatment success.
**Limitations:** n/a
**Ethics Committee Approval:** The local ethics committee approved this retrospective study
**Funding for this study:** n/a
**Author Disclosures:**
Rahil Shahzad: Employee: Philips Healthcare
Xiaoming Liu: Nothing to disclose
Frank Thiele: Employee: Philips Healthcare
Alexander Bunck: Nothing to disclose
Michael Perkuhn: Employee: Philips Healthcare
Huimao Zhang: Nothing to disclose
Julius Niehoff: Nothing to disclose
Robert Peter Reimer: Nothing to disclose
Kan He: Nothing to disclose

### RPS 505-9
**Incorporation of polymorphisms of SULF1 into a pre-treatment CT based machine-learning radiomic model to predict the risk of platinum resistance in ovarian cancer**
*X. Yi*, Y. Liu, F. Zeng; Changsha/CN
*(yixiaoping@csu.edu.cn)*

**Purpose:** To develop a machine learning model incorporating Single-Nucleotide Polymorphisms (SNPs) of Human Sulfatase 1 (SULF1) with a pre-treatment CT radiomic model, to predict platinum resistance for ovarian cancer (OC) treatment.
**Methods or Background:** A research cohort that consisted of 102 patients with clinicopathologically confirmed OC was recruited during the time period from January 2006 to February 2018. All OC patients underwent maximal cyto-reductive surgery followed by platinum based chemotherapy (treatment response: platinum-resistant or platinum-sensitive disease), were divided into two cohorts [training, n=71; validation, n=31]. We genotyped 12 SNPs of serous SULF1 for all OC patients using Mass array method before treatment. Radiomic features, SNPs data and clinicopathological data of patients were used to build differentiation models. Feature selection and predictive modeling were performed using least absolute shrinkage and selection operator (LASSO), Random Forest Classifier and Support Vector Machine methods. Model performance for predicting platinum resistance was assessed with respect to its calibration, discrimination, and clinical application.
**Results or Findings:** For prediction of platinum resistance, the approach combining the radiomics, clinicopathological data and SNP data demonstrated higher classification efficiency, with an AUC value of 0.993 (95% CI: 0.83 to 0.98) in the training cohort and 0.967 (95% CI: 0.83 to 0.98) in validation cohort, than the performance with only the SNPs of SULF1 model (AUC: training, 0.843 [95%CI: 0.738-0.948]; validation, 0.815 [0.601-1.000]), or with only the radiomic model (AUC: training, 0.874 [95%CI: 0.789-0.960]; validation, 0.832 [95%CI: 0.687-0.976]). This integrated approach also showed good calibration and favorable clinical utility.
**Conclusion:** A predictive model combining pretreatment CT radiomics with genomic data such as SNPs of SULF1 could potentially help to predict platinum resistance in ovarian cancer treatment.
**Limitations:** Retrospective nature of this study.
**Ethics Committee Approval:** ChiCTR-TNC-15007604
**Funding for this study:** Natural Science Foundation of Hunan Province (2018JJ2641)
**Author Disclosures:**
Feiyue Zeng: Nothing to disclose
Yingzi Liu: Nothing to disclose
Xiaoping Yi: Nothing to disclose

### RPS 505-10
**An MRI radiomics signature to distinguish benign from malignant orbital lesions**
*L. Duron*, A. Heraud, F. Charbonneau, M. Zmuda, J. Savatovsky, L. Fournier, A. Lecler; Paris/FR

**Purpose:** To differentiate benign from malignant orbital lesions using radiomics on 3T MRI examinations.
**Methods or Background:** This prospective single-center study enrolled consecutive patients presenting with an orbital lesion undergoing a 3T MRI prior to surgery from 2015 to 2019. Radiomics features were extracted from six MRI sequences. Features were selected based on their reproducibility, non-redundancy, and with a step forward feature selection method. Selected features were used to train and optimize a Random Forest algorithm on the training set (75%) with 5-fold cross-validation. Performance metrics were computed on the test set (25%) with 95% confidence intervals (95%CI). Five residents, four general radiologists and three expert neuroradiologists were asked to visually distinguish benign from malignant lesions on the test set. Performance comparisons between reader groups and the model were performed. The impact of clinical and categorizable imaging data on algorithm performance was assessed.
**Results or Findings:** Two hundred patients (116/200[58%] women and 84/200[42%] men, mean age 53.0+/-17.9 years) with 126/200[63%] benign and 74/200[37%] malignant orbital lesions were included in the study. A total of 606 radiomics features were extracted. The best model on the training set combined 8 features including ADC Mean Value, Maximum Diameter on T1-WI and texture features. Area Under the ROC curve, accuracy, sensitivity and specificity on the test set were respectively 0.869 (95%CI [0.834-0.898]), 0.840 (95%CI [0.806-0.874]), 0.684 (95%CI [0.615-0.751]), and 0.935 (95%CI [0.905-0.961]). The radiomics model outperformed all reader groups, including expert neuroradiologists (p<0.01). Adding clinical and categorizable imaging data didn't impact the performance (p=0.49).
**Conclusion:** An MRI radiomics signature is helpful to differentiate benign from malignant orbital lesions and may outperform expert radiologists.
**Limitations:** Single-center study, without Perfusion-WI. Retrospective readers' task with its cognitive biases compared to real-life clinical decisions.
**Ethics Committee Approval:** Yes: IRB2015-A00364-45
**Funding for this study:** None
**Author Disclosures:**
Frédérique Charbonneau: Nothing to disclose
Loïc Duron: Nothing to disclose
Laure Fournier: Nothing to disclose
Mathieu Zmuda: Nothing to disclose
Alexandre Heraud: Nothing to disclose
Julien Savatovsky: Nothing to disclose
Augustin Lecler: Nothing to disclose

### Research Presentation Session: Artificial Intelligence & Machine Learning

### RPS 605

### AI in breast imaging

### RPS 605-1
**A deep-learning classification model for mammogram screening**
*M. Jie*[1], X. Lin[1], R. Ouyang[1], M. Wu[1], Z. Cao[2], Y. Zhang[2], Z. Yang[2], Y. Tang[2], P. Chang[2]; [1]Shenzhen/CN, [2]Palo Alto, CA/US

**Purpose:** Mammogram screening is dominated by a massive amount of healthy patients, whose mammograms have no suspicious lesion findings. We aim to build a deep learning classification model to screen out some confidently healthy patients and to reduce the workload for radiologists in the scenario of physical examination.
**Methods or Background:** Indicated by radiologists, 16,556 patients who have at least one mammogram having suspicious lesions, and 22,625 patients whose both breasts are BI-RADS1, are collected. The train/val/test sets have 70%,10%,20% of the data split at the patient level. All patients' data are collected from three hospitals in Shenzhen, China. The model has a Siamese structure to take bilateral mammograms from the same patient, such as LCC and RCC, at one time. One of these bilateral mammograms is the main mammogram, on which the model will output the result. With a selected threshold, the model predicts the main mammogram to be healthy or suspicious. During testing, only if all mammograms of a patient are claimed healthy, will we claim her healthy. Otherwise, we mark her as a suspicious patient.

**Results or Findings:** We define a patient as false negative when, indicated by radiologists, at least one of her mammograms has suspicious findings, but the model predicts her to be healthy. Applying a threshold to keep a false-negative rate as 0, the model achieves a true-negative rate of 0.231 on the test set. This means that the model correctly screens out 23.1% healthy patients while remaining 100% sensitive to suspicious patients.
**Conclusion:** Applying this artificial intelligence-based screening system can potentially reduce the workload for radiologists by screening out some healthy mammograms.
**Limitations:** The bias caused by race difference is not well considered.
**Ethics Committee Approval:** The IRB number is LL-XJS-2020011.
**Funding for this study:** n/a
**Author Disclosures:**
Zhicheng Yang: Nothing to disclose
Ma Jie: Nothing to disclose
Peng Chang: Nothing to disclose
Mingxiang Wu: Nothing to disclose
Yuxing Tang: Nothing to disclose
Rushan Ouyang: Nothing to disclose
Yanbo Zhang: Nothing to disclose
Zhenjie Cao: Nothing to disclose
Xiaohui Lin: Nothing to disclose

### RPS 605-2

**Automated classification of breast calcification malignancy in mammograms with deep-learning**

R. Ouyang[1], *M. Jie*[1], X. Lin[1], M. Wu[1], Y. Zhang[2], Y. Tang[2], Z. Cao[2], Z. Yang[2], M. Han[2]; [1]Shenzhen/CN, [2]Palo Alto, CA/US

**Purpose:** To investigate the capability of breast calcification malignancy classification for each individual calcification region with a deep learning-based classification approach.
**Methods or Background:** We develop an attention-based deep learning model for benign and malignant calcification classification in mammograms. We apply a 400*400 pixel sliding window moves in mammogram images to extract patches, and patches with calcifications are preserved and its corresponding calcification binary mask is used as attention map. A widely used ResNet 18 is used as classification architecture, a patch embedded with attention map is assumed as the input, and biopsy malignancy label of calcifications in the patch is the training target. Two junior radiologists annotated calcification regions in mammograms, and then specified each calcification region a “benign/malignant” label with the guidance of pathology report. All the annotations were finally checked by an experienced radiologist. In total, 1776 mammogram images with both calcifications and pathology reports, collected between 2011 and 2018, were annotated. These images were classified with the ratio of 3:1:1, and then image patches, the corresponding attention maps and malignancy labels were generated as training, validation, test dataset.
**Results or Findings:** The calcification malignancy classification model was trained and validated over 32484 patches, and evaluated over 12730 patches. The developed method achieves an AUC of 0.956±0.02 (a sensitivity of 94.5±0.4% and a specificity of 80.1±0.5%) for malignant versus benign calcification classification.
**Conclusion:** This study showed that the designed calcification classification method was capable of discriminated about 94.5% of malignant calcifications from radiologists' annotated calcifications.
**Limitations:** In this study, all the mammograms were collected from only one hospital. More data sources are desired to enhance diversity.
**Ethics Committee Approval:** The IRB number is LL-XJS-2020011.
**Funding for this study:** n/a
**Author Disclosures:**
Zhicheng Yang: Nothing to disclose
Ma Jie: Nothing to disclose
Mingxiang Wu: Nothing to disclose
Yuxing Tang: Nothing to disclose
Rushan Ouyang: Nothing to disclose
Mei Han: Nothing to disclose
Yanbo Zhang: Nothing to disclose
Zhenjie Cao: Nothing to disclose
Xiaohui Lin: Nothing to disclose

### RPS 605-3

**Use of an artificial intelligence software to identify low-likelihood of cancer exams collected across four commercial digital tomosynthesis screening systems**

*E. F. Conant*[1], A. Y. Toledano[2], S. Periaswamy[3], J. W. Hoffmeister[3], R. M. Nishikawa[4]; [1]Philadelphia, PA/US, [2]Kensington, MD/US, [3]Nashua, NH/US, [4]Pittsburgh, PA/US
*(emily.conant@pennmedicine.upenn.edu)*

**Purpose:** To evaluate using a deep convolutional neural network artificial intelligence (AI) system to identify low-likelihood of cancer digital breast tomosynthesis (DBT) screening exams to triage workload. The impact of incorporating age and/or breast density in the algorithm is also evaluated.
**Methods or Background:** This retrospective study used consecutive cases from 18 U.S. and 3 French sites. The dataset comprised 506 cancer and 1,293 non-cancer cases with at least 320 days of negative follow-up. Analysis used 2,999 bootstrap samples randomly selected to match a screening population within subsets of cancer and non-cancer cases and a cancer detection rate of 6.0 per 1000 screened. Endpoints included assessment of an AI-derived case-score threshold such that the false negative rate (FNR) was 0%, overall and adjusting for age (30-49, 50-64, >64) and/or breast density (dense, non-dense). Reduction in workload was measured as percentage of all cases below threshold. Trend in triage rate by AI score decile also was estimated. Medians and confidence intervals (CIs) are reported.
**Results or Findings:** With an AI-derived case-score threshold for 0% FNR, 33.4% (95% CI: 30.7-36.3%) of exams could be triaged as low-likelihood of cancer. Triaging the 40% and 50% lowest AI scores, FNR was 1.3% (95% CI: 0.4-2.4%) and 2.8% (95% CI: 1.5-4.3%), respectively. Adjusting case-score threshold for age and breast density increased potential workload reduction by 26.5% (95% CI: 23.8%-29.2%) without increasing FNR. The incremental benefit of adding breast density to age adjustment is 7.1% (97.5% CI: 5.2-9.3%); adding age to breast density, incremental benefit is 15.9% (97.5% CI: 13.3-18.6%).
**Conclusion:** An AI system can identify 33.4% of screening DBT exams to triage with 0% FNR. Combining AI case-score with age and breast density, triage could potentially increase to 58.6%.
**Limitations:** n/a
**Ethics Committee Approval:** n/a
**Funding for this study:** iCAD, Inc.
**Author Disclosures:**
Senthil Periaswamy: Shareholder: iCAD, Inc. Employee: iCAD, Inc.
Jeffrey W Hoffmeister: Shareholder: iCAD, Inc. Employee: iCAD, Inc.
Robert M Nishikawa: Research/Grant Support: GE Heathcare Advisory Board: iCAD, Inc. Research/Grant Support: Koios Medical Other: Hologic, Inc. Research/Grant Support: Hologic, Inc. Advisory Board: maiData Corp.
Alicia Y Toledano: Consultant: iCAD, Inc.
Emily F. Conant: Advisory Board: iCAD, Inc. Grant Recipient: iCAD, Inc. Advisory Board: Hologic, Inc. Grant Recipient: Hologic, Inc.

### RPS 605-4

**Performance of indigenously build artificial-intelligence/machine-learning algorithm in reporting mammography: experience from a developing country**

*R. Ananthasivan*[1], K. Garg[2], P. G. Patil[1], S. Bevoor Chowdaiah[1], H. H. Prabhakaran[2], K. Ravindran[2], V. Raj[1]; [1]Bangalore/IN, [2]Chennai/IN
*(rupanth@yahoo.com)*

**Purpose:** Healthcare systems in developing countries are heterogeneous in their capabilities and delivery structure. Artificial Intelligence (AI)/Machine Learning (ML) systems from developed countries are often very expensive and not adaptable to local practices. Breast cancer screening programs are not well established in developed countries. We assessed the performance of an indigenously built AI/ML application in reporting mammography in a developing country.
**Methods or Background:** Mammography images from one hospital and a teleradiology setup were collated between Nov 2018 to Feb 2020. A team of senior radiologists annotated training datasets, on which AI/ML system was trained. Cases from the testing dataset were reported by four radiologists and assessed against the AI/ML generated report. Any discrepancy between the radiologist and AI report was reviewed by a panel of senior radiologists and consensus opinion was generated.
**Results or Findings:** A total of 18908 examinations were included, the training set consisted of 9960 studies (53%). Of the testing set, randomly selected 3042 (23% were CR) cases were reported by 4 radiologists. The accuracy of the AI/ML system was 95% in differentiating normal from the abnormal study. The sensitivity was 98% and specificity 94%. Commonest false-positive findings were axillary lymph nodes (58). The commonest false-negative findings were of microcalcification (33%) followed by focal asymmetry and masses obscured by dense tissue. AI/ML failed to detect subtle architectural distortion and diffuse changes like skin and parenchymal thickening.
**Conclusion:** The indigenously built AI/ML algorithm has promising results with high sensitivity and specificity in a large dataset.
**Limitations:** Wider use in different clinical set-ups need to be tested to understand universal adoption.
**Ethics Committee Approval:** Approved by local body.
**Funding for this study:** None

**Author Disclosures:**
Sanjana Bevoor Chowdaiah: Nothing to disclose
Vimal Raj: Advisory Board: CHS World
Hari Haran Prabhakaran: Founder: CHS World
Krithika Ravindran: Board Member: CHS World
Kushagra Garg: Investigator: CHS World
Rupa Ananthasivan: Nothing to disclose
Pooja G Patil: Nothing to disclose

### RPS 605-5

**Automated classification of benign and malignant breast mass in digital mammograms with deep multi-task learning**

X. Lin[1], *M. Jie*[1], R. Ouyang[1], M. Wu[1], Y. Tang[2], Y. Zhang[2], Z. Cao[2], Z. Yang[2], M. Han[2]; [1]Shenzhen/CN, [2]Palo Alto, CA/US

**Purpose:** To investigate whether the BI-RADS scores reported by radiologists could improve the automated classification performance of benign and malignant breast mass on mammography.
**Methods or Background:** We propose an end-to-end architecture for benign and malignant mass classification on mammograms using multi-task learning, by training with both binary biopsy labels from pathology reports and BI-RADS scores from radiologists. The proposed architecture aims to improve learning efficiency and prediction accuracy by learning two separate objectives from a shared representation. Namely, a 121-layer densely connected convolutional network is shared for feature learning, with a classification branch and a regression branch padded for benign/malignant classification and malignancy prediction, respectively. During training, the BI-RADS scores range from 1 to 6 (4A, 4B, and 4C are separate classes) are normalized into the range [0, 1] according to a pre-defined distance map. We design a novel multi-task loss combining classification and regression to simultaneously learn these two tasks in a unified architecture.
**Results or Findings:** The proposed method is trained and cross-validated on 5865 mammographic images. A hold-out set containing 390 images (no patient overlap) is used to evaluate the performance of the proposed method. Our method achieves an AUC of 0.915±0.03 (a sensitivity of 90.10±0.54% and a specificity of 70.05±0.37%) for benign versus malignant mass classification, compared to 0.886±0.03 using only binary labels in training ($p<0.05$).
**Conclusion:** In addition to the objective biopsy labels, subjective BI-RADS scores can provide auxiliary information in training deep neural networks for benign/malignant mass classification and malignancy prediction in a multi-task setting.
**Limitations:** BI-RADS scores are subjective and might vary across radiologists. A more thorough analysis of this variance is desired.
**Ethics Committee Approval:** The IRB number is LL-XJS-2020011.
**Funding for this study:** No
**Author Disclosures:**
Zhicheng Yang: Nothing to disclose
Ma Jie: Nothing to disclose
Mingxiang Wu: Nothing to disclose
Yuxing Tang: Nothing to disclose
Rushan Ouyang: Nothing to disclose
Mei Han: Nothing to disclose
Yanbo Zhang: Nothing to disclose
Zhenjie Cao: Nothing to disclose
Xiaohui Lin: Nothing to disclose

### RPS 605-6

**Breast lesions identification employing radial basis function on mammowave clinical data**

*S. P. Rana*[1], M. Dey[1], R. Loretoni[2], M. Duranti[3], L. Sani[3], A. Vispa[3], M. Ghavami[1], S. Dudley[1], G. Tiberi[3]; [1]London/UK, [2]Foligno/IT, [3]Perugia/IT
*(ranas11@lsbu.ac.uk)*

**Purpose:** Recently, a novel microwave apparatus for breast lesion detection (MammoWave), uniquely able to function in air with 2 antennas rotating in the azimuth plane and operating within the band 1-9 GHz has been developed. Machine learning (ML) can help to understand phenomena from the frequency spectrum collected through MammoWave in response to the stimulus, segregating breasts with and without lesions. The performance is quantified statistically for the ML augmented apparatus.
**Methods or Background:** The study comprises 61 breasts (from 34 patients), each one with the correspondent output of the radiologist's conclusion (i.e. gold standard) obtained from echography and/or mammography and/or MRI. The MammoWave examinations are performed, recording complex S21 in multi-bistatic fashion where the magnitudes show substantial discrepancy and reveal dissimilar behaviours when reflected from healthy tissues or tissues with lesions. Principal component analysis (PCA) is implemented to extract the unique quantitative responses from the frequency response for automated breast lesion identification, engaging support vector machine (SVM) with radial basis function (RBF) kernel.
**Results or Findings:** According to the radiologist conclusions, 25 breasts without lesions and 36 breasts with lesions underwent MammoWave examination. The proposed SVM model achieved the optimal accuracy, sensitivity, and specificity of 92.50%, 85.40%, and 97.40% respectively, outperforming comparable gold standards.
**Conclusion:** The proposed ML augmented MammoWave can identify the breast lesions early with high accuracy for woman of any ages and any breast density.
**Limitations:** We did not consider patients pre-menstrual information.
**Ethics Committee Approval:** Ethical Committee of Umbria, Italy (N. 6845/15/AV/DM of 14/10/2015, N. 10352/17/NCAV of 16/03/2017, N 13203/18/NCAV of 17/04/2018).
**Funding for this study:** This project has received funding from the European Union's Horizon 2020 research and innovation program under grants agreements No: 830265, 793449, 872752.
**Author Disclosures:**
Lorenzo Sani: Employee: UBT - Umbria Bioengineering Technologies, Perugia/IT
Sandra Dudley: Nothing to disclose
Maitreyee Dey: Nothing to disclose
Michele Duranti: Nothing to disclose
Mohammad Ghavami: Nothing to disclose
Alessandro Vispa: Employee: UBT - Umbria Bioengineering Technologies, Perugia/IT
Soumya Prakash Rana: Nothing to disclose
Gianluigi Tiberi: Shareholder: UBT - Umbria Bioengineering Technologies, Perugia/IT
Riccardo Loretoni: Nothing to disclose

### RPS 605-7

**BiDualNet: mammographic bilateral-view mass segmentation network**

X. Lin[1], *M. Jie*[1], R. Ouyang[1], M. Wu[1], Z. Cao[2], Y. Zhang[2], Z. Yang[2], P. Chang[2], M. Han[2]; [1]Shenzhen/CN, [2]Palo Alto, CA/US

**Purpose:** To incorporate this diagnostic prior information and facilitate the learning of the symmetry constraint, we develop a Bilateral Dual-view Network for mass segmentation.
**Methods or Background:** The proposed BiDualNet model is derived from a DeepLab v3+ structure, including a Siamese input module, a non-local(NL) module and the pixel-wise focal loss(PWFL) function. For a given mammogram, the other breast image of the same view is registered as an auxiliary input of the model. The Siamese input module consists of two NL atrous convolution modules, which share the same weights to extract features from the bilateral images in the same manner. The auxiliary feature map is then assumed as a reference and concatenated with the main feature map, and in turn the feature difference at the same location can highlight the abnormality. Finally, DeepLab v3+ decoder generates a segmentation result for the main image. We train and test the proposed model using the public Digital Database for Screening Mammography(DDSM) and our private mammogram dataset. DDSM has 2,620 patient cases collected from U.S. hospitals, and the private dataset contains 2,749 cases of Asian patients. Each case has four views of mammograms. Both datasets have normal, cancer, and benign cases, close to the practical distribution. These two datasets are randomly divided into the train/val/test sets by approximately 8:1:1, respectively.
**Results or Findings:** The proposed BiDualNet respectively achieves recall scores of 0.78,0.84,0.89 on DDSM when false positive per image(FPI) rates are 0.5,1.0,2.0. For the same FPI settings, the recall scores respectively reach 0.87,0.93,0.95 on the private dataset.
**Conclusion:** The BiDualNet considers the symmetry information for both breasts at the same view, which in turn enhances mass segmentation capability for mammograms.
**Limitations:** The dataset sizes are relatively small.
**Ethics Committee Approval:** The IRB number is LL-XJS-2020011.
**Funding for this study:** n/a
**Author Disclosures:**
Zhicheng Yang: Nothing to disclose
Ma Jie: Nothing to disclose
Peng Chang: Nothing to disclose
Mingxiang Wu: Nothing to disclose
Rushan Ouyang: Nothing to disclose
Mei Han: Nothing to disclose
Yanbo Zhang: Nothing to disclose
Zhenjie Cao: Nothing to disclose
Xiaohui Lin: Nothing to disclose

### RPS 605-10

**Text mining in Russian radiology reports**

D. Kokina, *Y. Meshalkin*, V. Gombolevskiy, A. Vladzymyrskyy, A. E. Andreychenko, S. Morozov; Moscow/RU

**Purpose:** The research was aimed to explore the possibilities of NLP, ML methods for classification of Russian free-text radiology reports in order to form large scale medical datasets and to perform a prospective agreement study between physicians and AI in radiology.
**Methods or Background:** Three use cases were studied that included: chest CT radiology reports with COVID-19 signs (28173 total reports, 21752 reports positive for COVID-19 signs) and lung nodules suspected of cancer (894 total reports, 411 reports positive for lung nodules suspected of cancer) and MMG radiology reports (1120 total reports, 289 BiRADS 0 or >3) created between 01|2019 and 08|2020. The following NLP and machine learning techniques were investigated: k-nearest neighbours model (selection of optimal hyperparameters using cross-validation technique, k=3) and keyword models. For every use case, best performing techniques were chosen based on their accuracy metrics values for the test datasets.
**Results or Findings:** The best results were achieved in classification for signs of breast cancer according to MMG radiology report data (accuracy 0.996) with keyword-based model and Covid-19-pneumonia according to CT reports (accuracy 0.993) with k-nearest neighbour model. These results are comparable to the accuracy of analogous studies in free text English, Chinese and Spanish radiology reports classification. The classification of CT radiology reports for lung nodules suspected of cancer (that were much less structured than MMG and COVID-CT reports) achieved 0.972 accuracy with the keyword-based model.
**Conclusion:** The results indicate that the Russian radiology reports can be successfully classified with the help of text mining methods (machine learning, NLP). Similar to other languages (semi-)structured reporting benefits the machine-based classification accuracy.
**Limitations:** Class imbalance per subset could bias the results
**Ethics Committee Approval:** Based on the results of the registered study (https://clinicaltrials.gov/ct2/show/NCT04489992).
**Funding for this study:** Internal
**Author Disclosures:**
Yuri Meshalkin: Author: algorithm realization
Sergey Morozov: Consultant: concept formation
Anna Evgenevna Andreychenko: Author: concept formation
Anton Vladzymyrskyy: Consultant: consultation
Victor Gombolevskiy: Consultant: data preparation
Daria Kokina: Author: data preparation

### RPS 605-11

**On the ability of an AI system to detect breast cancer earlier**

S. Pacilè[1], P. Fillard[2], *J. Lopez*[3]; [1]Nice/FR, [2]Paris/FR, [3]Newport Beach, CA/US
*(January.Lopez@hoag.org)*

**Purpose:** To investigate about the ability of an AI-system to detect breast cancer at earlier stages.
**Methods or Background:** A dataset of 2D Full-Field Digital Mammograms was retrospectively collected including in the study 16004 women. For each patient 3 consecutive screening exams were collected (delayed by one screening interval ΔT). The most recent exam for each patient was referred to as T0, and it was considered as malignant (n=408) if a biopsy proved the presence of cancer within ΔT+3 months or negative/benign (n=15596) if the subsequent screening exam was negative or triggered a negative follow-up. The inferred status was propagated back and attributed to the prior exams collected at T-1 and T-2. At T0, T-1 and T-2 the predictions of the AI system (MammoScreen v1.0.2, Therapixel) were computed and compared to the original decision (patient recalled or not) made by the radiologist.
**Results or Findings:** The sensitivity of the AI system and the radiologist at T0 was 58% and 65%, respectively. The AI system was able to predict 110 cancers at T-1 (18.57 months – 95% CI: 16.55 - 20.58) with a sensitivity of 27%, and 84 cancers at T-2 (36.93 months – 95% CI: 33.60 - 40.25) with a sensitivity of 21%. Inclusion of AI positive cases resulted in an incremental increase in recall rate of 12.6% at T-1 and 11% at T-2.
**Conclusion:** The addition of AI positive assessments to radiologist interpretations of screening mammograms has the potential to detect breast cancers 1 or 2 screening cycles earlier than without AI, with a modest increase in recall rates to approximately 11% overall.
**Limitations:** Retrospective character of the study.
**Ethics Committee Approval:** The investigation protocol was approved by an IRB.
**Funding for this study:** The study was sponsored by Therapixel.
**Author Disclosures:**
January Lopez: Nothing to disclose
Pierre Fillard: Employee: Therapixel Founder: Therapixel
Serena Pacilè: Employee: Therapixel

### RPS 605-12

**Value of technical stratification of medical datasets for AI services**

*N. Pavlov*, Y. S. Kirpichev, A. Revazyan, V. Klyashtorny, A. Vladzymyrskyy, A. E. Andreychenko, S. Morozov; Moscow/RU
*(nickvolvap@gmail.com)*

**Purpose:** Fast and reliable evaluation of AI service before its clinical implementation is needed to obtain threshold values that differentiate normal studies from pathologic ones (known as calibration). Here, we explore whether thresholds should be calculated independently for different vendor diagnostic devices.
**Methods or Background:** Two AI services were included in the study: one for mammographic BI-RADS evaluation (MG), and one for COVID-19 findings on chest computed tomography (CT). Initially, the thresholds for MG and CT were determined during calibration on retrospective data (balanced dataset consisted of 50 studies with findings related to target pathology and 50 without). Then, the optimal thresholds were evaluated for every subset of prospective data determined by scanner manufacturer, of which 6 vendors were for MG (MG1–MG6), and 5 for CT (CT1–CT5) based on an agreement study of AI and radiologists. The number of prospectively analysed studies (radiologists) in each subset: MG1: 33248 (115); MG2: 8478 (46); MG3: 336 (5); MG4: 354 (2); MG5: 136 (1); MG6: 45 (2); CT1: 74630 (580); CT2: 10032 (92); CT3: 2422 (67); CT4: 4326 (70); CT5: 651 (25).
**Results or Findings:** For CT, when compared in pairs between each subset by scanner manufacturer, the statistical difference in optimal thresholds has been significant for all ($p \leq 0.001$) but one pair of CT2–CT3 ($p=0.941$). For MG, the difference was significant as well for MG1–MG2 ($p<0.001$), MG1–MG4 ($p=0.025$) and MG2–MG4 ($p=0.002$). These findings could be explained by variation in technical features of image acquisition process such as matrix size which in a way impact AI performance.
**Conclusion:** Accuracy of AI service could be improved by defining individual thresholds for each vendor during the calibration process.
**Limitations:** Differences in total number of cases per subset could potentially bias the results.
**Ethics Committee Approval:** https://clinicaltrials.gov/ct2/show/NCT04489992.
**Funding for this study:** No funding.
**Author Disclosures:**
Sergey Morozov: Nothing to disclose
Anna Evgenevna Andreychenko: Nothing to disclose
Anton Vladzymyrskyy: Nothing to disclose
Nikolay Pavlov: Nothing to disclose
Vladislav Klyashtorny: Nothing to disclose
Anush Revazyan: Nothing to disclose
Yury S Kirpichev: Nothing to disclose

### RPS 605-13

**An image annotation software for mammography**

R. Ouyang[1], *M. Jie*[1], X. Lin[1], M. Wu[1], Y. Zhang[2], S. Wu[1], L. Huang[1], P. Chang[2], Y. Xin[1]; [1]Shenzhen/CN, [2]Palo Alto, CA/US

**Purpose:** To efficiently and effectively annotate mammogram images, we develop specific annotation software.
**Methods or Background:** Deep learning has achieved great success in various medical image analysis scenarios. While its performance highly depends on the quality and quantity of annotated data. However, general-purpose annotation tools are usually difficult to efficiently provide high-quality annotations for a specific medical image modality.
**Results or Findings:** This software enables clinically-oriented users to efficiently annotate mammograms for deep learning models. It consists of five major modules. (1) Each patient's breast images and corresponding diagnosis information can be loaded and displayed in the software. (2) It enables users to manually draw boundary curves for lesions including mass, calcification, structural distortion and asymmetry. Alternatively, there is an "AI Analysis" option in this software. This embedded pre-trained AI model automatically generates lesion contours, accelerating the annotation remarkably. (3) For each lesion and image, users can specify various clinical information, including lesion types, malignancy, BI-RADS classes, density classes, etc. (4) The annotated information is automatically written into corresponding ".json" format files. (5) The software works in label/review modes, which allows a senior radiologist to review and correct the annotation. This function ensures the high quality of the obtained annotations. Three junior and one senior radiologists have labeled and reviewed more than 2000 patients' dataset. This dataset allows AI scientists to conduct various research or pre-clinical work, such as lesion segmentation, image classification, cancer-risk estimation, etc. Based on this well-annotated dataset, our trained mass and calcifications detection models have achieved state-of-the-art performance.
**Conclusion:** The developed software can efficiently annotate mammography images, providing a large number of annotated data for training deep learning-based lesion detection and classification models.
**Limitations:** This tool is currently used in a single-center study.
**Ethics Committee Approval:** The IRB number is LL-XJS-2020011.

**Funding for this study:** n/a
**Author Disclosures:**
Ma Jie: Nothing to disclose
Peng Chang: Nothing to disclose
Shibin Wu: Nothing to disclose
Mingxiang Wu: Nothing to disclose
Rushan Ouyang: Nothing to disclose
Lingyun Huang: Nothing to disclose
Yi Xin: Nothing to disclose
Yanbo Zhang: Nothing to disclose
Xiaohui Lin: Nothing to disclose

### Research Presentation Session: Artificial Intelligence & Machine Learning

### RPS 705

### AI in abdominal imaging

### RPS 705-2

**PET-CT co-registration and classification model for oesophagal cancer**

W. Cao, C. S. G. Lui, J. van Lunenburg, K. O. Lam, P. L. H. Yu,
*W. H. K. Chiu*; Hong Kong/HK
*(kwhchiu@hku.hk)*

**Purpose:** Positron-Emission Tomography-Computed Tomography (PET-CT) is an essential imaging modality for managing patients with esophageal cancer. The objective of this study was to combine PET and CT to predict complete pathological response (pCR) after neoadjuvant chemoradiotherapy (nCRT) in patients with Esophageal squamous cell carcinoma (ESCC).
**Methods or Background:** 86 patients with locally advanced ESCC who had undergone staging PET-CT, received nCRT followed by surgery were split into training and testing sets (65:21) stratified by outcome. We first developed a model to co-registration PET and CT images allowing alignment of both sets of volumes that have different dimensions. We then conducted data augmentation to increase the number of cases in the training set eight times by rotation, flipping and re-scaling. Using the augmented dataset, we trained a network-based classification model, composing of 8 convolutional layers and 2 fully-connected layers.
**Results or Findings:** In total, 85% of subjects were male, median age 65 (22-82) and 15% female with median age 68 (40-79). pCR of primary tumour was achieved in 34 patients (40.0%). The model achieved an accuracy of 0.810, 0.846 sensitivity, 0.750 specificity, 0.846 positive predictive value (PPV), 0.750 negative predictive value (NPV) and an area under the receiver operator curve (AUROC) of 0.798 for predicting pCR based on the fused PET and CT. Using CT alone, the model only achieved the accuracy of 0.7619, 0.846 sensitivity, 0.625 specificity, 0.786 PPV, 0.714 NPV and a significantly lower AUROC of 0.736 (p=0.008).
**Conclusion:** These results indicate that integrating PET and CT images using deep learning allows performance improvement in predicting pCR compared with a single modality approach of using CT alone.
**Limitations:** Single centre study with no external validation.
**Ethics Committee Approval:** The study was approved by the HKU/HA HKW IRB.
**Funding for this study:** None
**Author Disclosures:**
Wenming Cao: nothing to disclose
Chiu Sing Gilbert Lui: Nothing to disclose
Jurgen van Lunenburg: Nothing to disclose
Ka On Lam: Nothing to disclose
Wan Hang Keith Chiu: Nothing to disclose
Philip Leung Ho Yu: Nothing to disclose

### RPS 705-3

**Radiomics analysis for the differentiation of autoimmune pancreatitis and pancreatic carcinoma in MRI**

*A. Mahnken*, L. Campisi, M. Diehl, D. Heider, A. König, T. Thormälen; Marburg/DE

**Purpose:** Focal autoimmune pancreatitis (AIP) and pancreatic cancer (PC) have many similar clinical symptoms and similar imaging features impeding accurate, noninvasive differentiation of these entities. The aim of this study was to explore whether the use of radiomics techniques on magnetic resonance imaging (MRI) data could help to differentiate between AIP, PC and normal pancreas.
**Methods or Background:** This retrospective study included data of 58 patients (17 with AIP, 21 with PC, 20 with normal pancreas). All patients obtained a pretreatment MRI with unenhanced fat-suppressed, axial T1, and axial T2 weighted scans. After labeling manually the pancreas in each scan, 120 radiomics features were extracted. Different radiomics models were tested to achieve the best differentiation between AIP, PC and healthy individuals. Finally, the random forest model showed the best results. The performance of radiomics model was evaluated by AUC.
**Results or Findings:** The AUCs of the optimal radiomics model were 92% for AIP, 81% for PC and 87% for the control group. All results were highly significant (p < 0,0001).
**Conclusion:** The combination of MRI findings and radiomics techniques might be helpful to differentiate between AIP, PC and healthy individuals in clinical practice.
**Limitations:** This study suffers some limitations. Firstly, it only comprises a small dataset with 17 AIP patients due to its rare entity. Secondly, the classes differ in age and gender. This is due the fact that AIP patients are on average younger than patients with PC. Finally, the model was trained with a cross-validation approach using the whole dataset without subsequent independent testing of the model.
**Ethics Committee Approval:** No research ethics approval was necessary for this research.
**Funding for this study:** This study was supported by the European Regional Development Fund, EFRE-Program, European Territorial Cooperation (ETZ) 2014-2020, Interreg V A, Project 191.
**Author Disclosures:**
Marie Diehl: Nothing to disclose
Dominik Heider: Nothing to disclose
Luana Campisi: Nothing to disclose
Alexander König: Nothing to disclose
Thorsten Thormälen: Nothing to disclose
Andreas Mahnken: Nothing to disclose

### RPS 705-4

**3d MRI-based radiomics features in the prediction of placenta adhesion spectrum disorders in patients with placenta previa: a machine learning preliminary study**

*F. Verde*, M. Di Stasi, R. Cuocolo, A. Stanzione, V. Romeo, L. Sarno, M. Guida, M. D'Armiento, S. Maurea; Naples/IT
*(francescoverde87@gmail.com)*

**Purpose:** To assess the reliability of a 3D segmentation method of placenta MR images for the prediction of Placenta Adhesion Spectrum (PAS) disorders in patients with placenta previa (PP) using a Machine Learning Radiomics analysis.
**Methods or Background:** 63 patients (n=44 without PAS and n= 19 with PAS) with PP who underwent MRI examination for suspicion of PAS were retrospectively selected. Whole placental tissue was manually segmented slice-per-slice on sagittal placental T2-weighted images, by a first radiologist, obtaining a volume of interest (VOI) from each patient. For feature stability testing, the same procedure was then repeated on 30 randomly selected placental MRI examinations by two additional radiologists, working independently and blinded to the original segmentations. Radiomic features were extracted from all available VOIs. ML analysis was subsequently run to identify the best performing method to correctly classify instances.
**Results or Findings:** A number of 1106 radiomics features were extracted from whole-placental tissue VOIs. Of these, 420 showed intraclass correlation coefficient values lower to 0.75.and were therefore discarded. After the train/test split (75/25%), the model training set was composed of 47 cases while the test one included 16 cases. In the training test, 10 features were finally selected due to high Pearson's Correlation Coefficient. Using these features with a Bayesian Network analysis, the final model obtained a precision, recall, F1 score and AUROC of respectively 0.925, 0.813, 0.841 and 0.893.
**Conclusion:** A predictive ML model, based on 3D placental MRI radiomics features, is highly accurate in identifying PAS in patients with placenta previa.
**Limitations:** A retrospective study at a single institution and the relatively small patient population.
**Ethics Committee Approval:** Approval by the local Institutional Review Board. Informed consent waived.
**Funding for this study:** No funding source
**Author Disclosures:**
Valeria Romeo: Nothing to disclose
Laura Sarno: Nothing to disclose
Martina Di Stasi: Nothing to disclose
Francesco Verde: Nothing to disclose
Arnaldo Stanzione: Nothing to disclose
Maurizio Guida: Nothing to disclose
Renato Cuocolo: Nothing to disclose
Maria D'Armiento: Nothing to disclose
Simone Maurea: Nothing to disclose

### RPS 705-5

**Liver fibrosis staging by deep learning: a visual-based explanation of diagnostic decisions of the model**

*Y. Yin*[1], D. Yakar[1], R. Dierckx[1], K. Mouridsen[2], T. Kwee[1], R. de Haas[1]; [1]Groningen/NL, [2]Aarhus/DK
*(y.yin@umcg.nl)*

**Purpose:** Deep learning has been proven to be able to stage liver fibrosis based on contrast-enhanced CT images. However, until now the algorithm is used as a black box and lacks transparency. This study aimed to provide a visual-based explanation of the diagnostic decisions made by deep learning.
**Methods or Background:** The liver fibrosis staging network (LFS network) was developed at contrast-enhanced CT images in the portal venous phase in 252 patients with histologically proven fibrosis stage. To give a visual explanation of the diagnostic decisions made by the LFS network, Gradient-weighted Class Activation Mapping (Grad-cam) was used to produce location maps indicating where the LFS network focuses on when predicting liver fibrosis stage.
**Results or Findings:** The LFS network had areas under the receiver operating characteristic curve of 0.92, 0.89, and 0.88 for staging significant fibrosis (F2-F4), advanced fibrosis (F3-F4), and cirrhosis (F4), respectively, on the test set. The location maps indicated that the LFS network had more focus on the liver surface in patients without liver fibrosis (F0), while it focused more on the parenchyma of the liver and spleen in case of cirrhosis (F4).
**Conclusion:** Deep learning methods are able to exploit CT-based information from the liver surface, liver parenchyma and extrahepatic information to predict liver fibrosis stage. Therefore, we suggest to use the entire upper abdomen on CT images when developing deep learning-based liver fibrosis staging algorithms.
**Limitations:** The training dataset in our study was relatively small. Because deep learning methods are data-driven, diagnostic accuracy and stability of location maps highly depend on the variety and volume of the dataset. Therefore, our results should be confirmed in larger (multicenter) studies.
**Ethics Committee Approval:** IRB statement: the study was approved by the institutional review board (IRB number: 2018/139)
**Funding for this study:** None
**Author Disclosures:**
Kim Mouridsen: Nothing to disclose
Robbert de Haas: Nothing to disclose
Thomas Kwee: Nothing to disclose
Yunchao Yin: Nothing to disclose
Derya Yakar: Nothing to disclose
Rudi Dierckx: Nothing to disclose

### RPS 705-6

**Texture analysis for diagnosis of steatohepatitis and chemotherapy-associated liver injuries: performances of a combined radiomic and clinical model**

*F. Fiz*, L. Cavinato, C. Masci, G. Costa, F. Leva, G. Torzilli, A. Chiti, L. Balzarini, L. Viganò; Milan/IT
*(francesco.fiz.nm@gmail.com)*

**Purpose:** Patients undergoing liver resection for colorectal metastases after oxaliplatin- and/or irinotecan-based chemotherapy-associated liver injuries (CALI) have a major clinical impact, but their non-invasive diagnosis is still an unmet need. To elucidate the contribution of radiomic analyses to diagnosis of sinusoidal dilatation, nodular regenerative hyperplasia (NRH) and non-alcoholic steatohepatitis (NASH).
**Methods or Background:** Patients undergoing liver resection for colorectal metastases after oxaliplatin- and/or irinotecan-based chemotherapy between January 2018 and February 2020 were retrospectively analyzed. Radiomic features were extracted from a standardized volume of non-tumoral liver parenchyma outlined in the portal phase of preoperative post-chemotherapy computed tomography (CT). A multivariate logistic-regression model was performed to identify predictors of CALI. Internal validation was applied.
**Results or Findings:** Overall, 78 patients were included. Of these, 25 (32%) had grade 2-3 sinusoidal dilatation, 27 (35%) NRH, and 14 (18%) NASH. Three fingerprints derived from radiomic features were independent predictors of grade 2-3 sinusoidal dilatation: GLRLM_f3 (OR=12.25), NGLDM_f1 (OR=7.77), and GLZLM_f2 (OR=0.53). The combined clinical/radiomic predictive model had 82% accuracy, 64% sensitivity, and 91% specificity (AUC=0.87 vs. AUC=0.77 of the model without radiomics). Three radiomic parameters were independent predictors of NRH: conventional_HUQ2 (OR=0.76), GLZLM_f2 (OR=0.05), and GLZLM_f3 (OR=7.97). The combined clinical/radiomic model had 85% accuracy, 81% sensitivity, and 86% specificity (AUC=0.91 vs. AUC=0.85 without radiomic features). One radiomic feature was associated with NASH: conventional_HUQ2 (OR=0.79). Steatohepatitis was predicted with 91% accuracy, 86% sensitivity, and 92% specificity (AUC=0.93 vs. AUC=0.83 without radiomic features). In the validation setting, accuracy was 72%, 71%, and 91% for sinusoidal dilatation, NRH, and NASH, respectively.
**Conclusion:** Radiomic analysis of liver parenchyma provides a signature that, in combination with clinical and laboratory data, improves diagnosis of CALI.
**Limitations:** Retrospective, monocentric.
**Ethics Committee Approval:** IRB-approved, need for specific consent was waived.
**Funding for this study:** None.
**Author Disclosures:**
Francesca Leva: Nothing to disclose
Guido Torzilli: Nothing to disclose
Francesco Fiz: Nothing to disclose
Arturo Chiti: Nothing to disclose
Chiara Masci: Nothing to disclose
Lara Cavinato: Nothing to disclose
Guido Costa: Nothing to disclose
Luca Viganò: Nothing to disclose
Luca Balzarini: Nothing to disclose

### RPS 705-8

**Using variable flip angle evolution to implement deep learning accelerated abdominal HASTE sequence in a single breath-hold at 3 T**

*J. Herrmann*; Tuebingen/DE
*(judith.herrmann@med.uni-tuebingen.de)*

**Purpose:** Due to numerous refocusing pulses, HASTE sequences have SAR-related speed limitations at 3T. Our purpose was to create a protocol with a deep learning (DL) accelerated single-breath-hold half-Fourier single-shot turbo spin echo (HASTE_DL) sequence for T2-weighted MRI of the abdomen at 3T. We first defined a suitable flip angle (FA) evolution with low cardiac-motion related signal loss and low specific absorption rate (SAR) in a volunteer study and implemented this FA evolution in clinical routine. Its clinical feasibility, image quality (IQ) and diagnostic accuracy compared to standard T2-weighted sequences were assessed.
**Methods or Background:** 11 volunteers were included to evaluate the technical feasibility and diagnostic performance of 9 different FA evolutions of a HASTE_DL to identify the most suitable FA evolution with reasonable SAR, and low cardiac-motion related signal loss. Suitable FA was determined and implemented in clinical routine. 51 patients who underwent clinically indicated liver/pancreas MRI at 3T were included. Two radiologists assessed HASTE_DL and standard sequence regarding IQ, noise, contrast, sharpness, artifacts, cardiac-motions related signal loss, diagnostic confidence, lesion detectability, and characterization using a Likert-Scale ranging from 1-4 with 4 being best.
**Results or Findings:** Suitable FA was defined as 130°-100°-120°-140° (HASTE_DL_100) and implemented with one lower FA (130°-90°-110°-130°; HASTE_DL_90) in clinical routine. All HASTE_DL were successfully acquired at 3 T and showed comparable IQ to standard sequences.
**Conclusion:** We successfully created and implemented a novel, single breath-hold HASTE_DL with variable FA evolutions at 3T in clinical routine. IQ and diagnostic confidence of HASTE_DL is comparable to standard T2w images and can highly accelerate TA, while staying within SAR limitations.
**Limitations:** Limitation is the relatively small sample size and single-center study design.
**Ethics Committee Approval:** The institutional review board approved this monocentric study with waiver of informed consent.
**Funding for this study:** None.
**Author Disclosures:**
Judith Herrmann: Author: Using variable flip angle evolution to implement deep learning accelerated abdominal HASTE sequence in a single breath-hold at 3T Speaker: Using variable flip angle evolution to implement deep learning accelerated abdominal HASTE sequence in a single breath-hold at 3T

### RPS 705-9

**Slice-propagated 3D gastric tumour segmentation from a single 2D annotation**

*Z-F. Chen*, I. Jiazheng, Y-T. Liu, J. Zhao, L. Zhang, L. Tang, B. Dong; Beijing/CN
*(czifan@pku.edu.cn)*

**Purpose:** To develop a deep learning model on 3D CT images to generate the volumetric segmentation of a gastric tumor from the annotation of its largest cross-section.
**Methods or Background:** In this work, 232 CT volumes of different phases are obtained from 128 gastric cancer patients, of which 98 (173 CT scans) are randomly chosen as the training set and the remaining 30 (59 CT scans) as the test set. A two-stage convolutional model is established to generate the complete 3D tumor segmentation from the annotation of its largest cross-section. In the first stage, our model iteratively segments the adjacent slices from the annotated 2D slice to eventually obtain a coarse-grained segmentation of the entire tumor. In the second stage, the model focuses on optimizing difficult pixels. Our method is established under a one-shot learning framework.

**Results or Findings:** The Dice (Kappa) coefficients between the segmentation results and the manual annotations of the two radiologists are 0.7805 (0.7799), p=0.3634, and 0.7772 (0.7766), p=0.3618, respectively, which is comparable to the inter-observer variability of 0.7971 (0.7966) between the radiologists. Our method takes about 1.1 seconds to process a CT volume on a standard GPU (the time of annotating the largest cross-section is about 40 seconds), while manual annotation takes about 7 minutes.
**Conclusion:** The proposed method can greatly enhance the efficiency of 3D gastric tumor segmentation without accuracy loss, facilitating the quantitative analysis of gastric cancer.
**Limitations:** We believe that a third doctor can be introduced to make decisions for the ambiguity between two doctors' annotations.
**Ethics Committee Approval:** Our method is consistent with the Declaration of Helsinki.
**Funding for this study:** Beijing Natural Science Foundation (Z180001).
**Author Disclosures:**
Lei Tang: Nothing to disclose
Zi-Fan Chen: Nothing to disclose
Jie Zhao: Nothing to disclose
Yi-Ting Liu: Nothing to disclose
Bin Dong: Nothing to disclose
li Jiazheng: Nothing to disclose
Li Zhang: Nothing to disclose

### RPS 705-11

**Quantitative edge analysis for the diagnosis of pancreatic carcinoma**
*M. Bariani*[1], A. Ambrosetti[2], M. C. Ambrosetti[1], G. Zamboni[1], G. Malleo[1], G. Mansueto[1]; [1]Verona/IT, [2]Padua/IT
*(matildebariani@gmail.com)*

**Purpose:** Provide a possible starting point for machine-learning recognition of pancreatic lesions based on lower-dimensional feature space.
**Methods or Background:** Quantitative techniques are increasingly acclaimed as an effective support for Multi-detector computed tomography (MDCT) image-analysis and computer-assisted diagnostics. From all Patients who underwent Multi-detector Computed Tomography (MDCT) for staging of pancreatic adenocarcinoma from January 2018 to January 2020 we selected 50 Patients (36 Males, 14 Females; mean age 63.7 years) with adenocarcinoma of the body-tail of the pancreas. Computer-assisted quantitative edge-analysis was performed on border fragments in MDCT images of neoplastic and healthy glandular parenchyma, obtaining the mean square deviation SD of the actual border from the average boundary line. SD values relative to healthy and neoplastic borders were compared by paired t-test.
**Results or Findings:** Tumoral border portions were systematically characterized by significantly lower SD values than healthy fragments (0.50 vs 1.27; p<0.001). In our series, using a threshold value of SD > 0,6855 pix showed 96% sensitivity and 96 % specificity in differentiating cancerous from healthy pancreatic edge segments.
**Conclusion:** We introduce a quantitative measure of boundary irregularity which correlates with the presence/absence of pancreatic adenocarcinoma. Quantitative edge-analysis can be promptly performed on select border fragments in MDCT images, thereby providing a useful supporting tool for diagnostics and a possible starting point for machine-learning recognition based on lower-dimensional feature space.
**Limitations:** We only analyzed a small number of patients with pancreatic adenocarcinoma (preliminary study). The technique could be less useful in small lesions embedded within the parenchyma. We calculated SD values related to a virtual measure (pixels) and not to a physical measure (millimeters).
**Ethics Committee Approval:** Informed consent for utilization of clinical and radiologic data was provided by all patients (PAD-R registry, n1101CESC).
**Funding for this study:** None
**Author Disclosures:**
Giulia Zamboni: Nothing to disclose
Matilde Bariani: Nothing to disclose
Giancarlo Mansueto: Nothing to disclose
Malleo Giuseppe Malleo: Nothing to disclose
Maria Chiara Ambrosetti: Nothing to disclose
Ambrosetti Alberto Ambrosetti: Nothing to disclose

### RPS 705-13

**Accurate assessment of the minimal ablative margin and unablated region volume after liver tumor ablation on pre- and post-procedural contrast-enhanced CT: Using deep learning and image registration**
K. He[1], R. P. Reimer[2], X. Liu[1], *R. Shahzad*[2], F. Thiele[2], J. Niehoff[2], H. Zhang[1], A. Bunck[2], M. Perkuhn[2]; [1]Changchun/CN, [2]Cologne/DE
*(rahil.shahzad@uk-koeln.de)*

**Purpose:** Currently, liver tumor ablation(LTA) has emerged as a first-line treatment approach for patients with small malignant liver tumors, especially in hepatocellular carcinoma(HCC) and colorectal liver metastasis. The ideal ablation zone should encompass the tumor by at least 5mm. To evaluate immediate treatment response after LTA, the minimal ablative margin(MAM) between the tumor and ablative margin is estimated using side-by-side comparison of CT images, which is time-consuming and suffers from high interrater-variability. To overcome these limitations in clinical routine, we propose an automatic method to assess MAM and the unabated region volume(URV).
**Methods or Background:** 55 patients with 64 malignant liver tumors(40 HCC, 24 metastasis) who underwent LTA were retrospectively included. Pre-trained deep learning models(DLM) that localize and segment the liver, tumors and ablation zone in 3D on multi-phase CT scans were used. A non-rigid registration method for the liver between pre- and post-procedural CT scans was developed. After successful segmentation and registration, MAM and URV were computed and compared between cases with and without local tumor progression(LTP) using Wilcoxon test.
**Results or Findings:** The mean tumor volume was 4.2cm3. The mean ablation zone volume was 66.2cm3. Median MAM was 3.9(0-10.5)mm and median URV 0.044(0-2.16)cm3. A total of 48(75%) lesions had an URV of <0.3cm3, 12(19%) between 0.3 and 1cm3 and 4(6%)>1cm3. Overall LTP rate was 7 of 64(10.9%) per ablated malignant liver tumor. URV significantly differed between ablated tumors with and without LTP (0.7 [0.4-2.2] vs. 0.0 [0-1.5]cm3; p<0.05), whereas MAM showed no significant difference (p>0.05).
**Conclusion:** The proposed 3D approach based on deeplearning and image registration allows for the assessment of MAM and URV, and can facilitate the evaluation of immediate treatment response after LTA.
**Limitations:** n/a
**Ethics Committee Approval:** The local ethics committee approved this retrospective study
**Funding for this study:** n/a
**Author Disclosures:**
Rahil Shahzad: Employee: Philips Healthcare
Xiaoming Liu: Nothing to disclose
Frank Thiele: Employee: Philips Healthcare
Alexander Bunck: Nothing to disclose
Michael Perkuhn: Employee: Philips Healthcare
Huimao Zhang: Nothing to disclose
Julius Niehoff: Nothing to disclose
Robert Peter Reimer: Nothing to disclose
Kan He: Nothing to disclose

### RPS 705-14

**Locally advanced rectal cancer: T2w-MRI based radiomics may detect responder patients undergoing neoadjuvant chemoradiotherapy**
A. Bevilacqua, F. Coppola, *M. Mottola*, S. Lo Monaco, A. Cattabriga, A. Cocozza, D. Cuicchi, L. Ricciardiello, R. Golfieri; Bologna/IT
*(margherita.mottola@unibo.it)*

**Purpose:** To investigate whether in locally advanced rectal cancer (LARC) treated with neo-adjuvant chemoradiotherapy (nCRT), radiomics on T2 weighted (T2w) MRI sequences can discriminate responder (R) and non-responder (NR) patients based on the Tumour Regression Grade (TRG) assigned after surgical resection
**Methods or Background:** This study retrospectively enrols 40 patients undergoing pre-therapy 1.5T-MRI. Regions of Interest (ROIs) are manually outlined in all slices of the tumour's site on T2w sequences in the oblique-axial plane, acquired with 3 mm slice thickness. Based on TRG, R patients have complete and partial nCRT response (TRG=[0,1], n°15) while NR patients have a minimal and poor nCRT response (TRG=[2,3], n°25). Eighty-four local first-order radiomic features (RFs) are extracted from tumour ROIs. To prevent overfitting, only single RFs are investigated to discriminate Rs and NRs. The most performing feature is selected through a univariate analysis guided by one-tail Wilcoxon rank-sum test (p=0.05 significance level). To assess the feature discrimination capability, ROC curve analysis is performed, through AUC computation, Youden Index (YI) for sensitivity and specificity.
**Results or Findings:** One RF measuring the local heterogeneity of T2w values within tumour ROIs discriminates Rs and NRs with p~10-5, AUC=0.90 (95%CI, 0.73-0.96), with YI=0.68 corresponding to sensitivity=80% and specificity=88%. The separation achieved highlights 3 false positives and 3 false negatives.
**Conclusion:** Pre-therapy baseline tumour heterogeneity measured from T2w-MR images has a very promising role in predicting the TRG histological classification. Patients with lower tumour heterogeneity at pre-therapy show a better response to nCRT.
**Limitations:** This study involves a small number of patients. However, one-only feature is considered and such a strong discrimination stresses the future role of the feature in a classification study.
**Ethics Committee Approval:** n° 842/2020/Oss/AOUBo
**Funding for this study:** Not applicable

**Author Disclosures:**
Silvia Lo Monaco: Nothing to disclose
Rita Golfieri: Nothing to disclose
Arrigo Cattabriga: Nothing to disclose
Adriana Cocozza: Nothing to disclose
Margherita Mottola: Nothing to disclose
Dajana Cuicchi: Nothing to disclose
Luigi Ricciardiello: Nothing to disclose
Francesca Coppola: Nothing to disclose
Alessandro Bevilacqua: Nothing to disclose

### RPS 705-15

**Deep learning for differentiation of liver cirrhosis aetiology from clinical MRI**

*S. Nowak*, A. Faron, N. Mesropyan, M. Reuter, W. Block, D. Kütting, U. I. Attenberger, J. A. Luetkens, A. M. Sprinkart; Bonn/DE

**Purpose:** To investigate whether a Deep Learning approach allows differentiation of alcohol-related vs. non-alcohol-related liver cirrhosis based on clinical T2-weighted MRI.
**Methods or Background:** Clinical MRI examinations of 465 patients with confirmed diagnosis of liver cirrhosis from 2017 to 2019 formed the data set for this retrospective monocentric study. Patients were classified according to the etiology of liver cirrhosis into (a) alcohol-related (n=221) and (b) non-alcohol-related (n=244) diseases. T2-weighted single slice images at the height of the caudate lobe were used for the analysis. The cohort was randomly divided into training (85%) and test data (15%) and the training data set was split for 5-fold cross-validation. For classification of alcohol-related and non-alcohol-related cirrhosis, two different ImageNet pre-trained Convolutional Neural Network (CNN) architectures (ResNet50, DenseNet121) were employed after upstream liver segmentation.
**Results or Findings:** The mean accuracy and mean area under the receiver operating curve (AUC) for 5-fold cross-validation were 0.74 and 0.83 for ResNet50 and 0.71 and 0.82 for DenseNet121. The highest classification performance on test data was observed for the ensemble of cross-validated ResNet50 with an accuracy of 0.75 (95% confidence interval: 0.64 - 0.85) and an AUC of 0.82 (0.72 - 0.92).
**Conclusion:** The application of a CNN supports the differentiation of alcohol-related vs. non-alcohol-related liver cirrhosis based on clinical T2-weighted MRI. This proof-of-principle study demonstrates that Deep Learning classifiers can aid in uncovering the underlying cause of liver cirrhosis, which may also guide the decision for an appropriate treatment.
**Limitations:** Although of clinical interest, the different types of etiologies of non-alcohol-related cirrhosis were not addressed due to limited number of cases per subgroup.
**Ethics Committee Approval:** The study was approved by the local institutional review board with waiver for written informed consent.
**Funding for this study:** Study was supported by BONFOR (2020-2A-04).
**Author Disclosures:**
Julian Alexander Luetkens: Nothing to disclose
Anton Faron: Nothing to disclose
Alois Martin Sprinkart: Nothing to disclose
Wolfgang Block: Nothing to disclose
Sebastian Nowak: Nothing to disclose
Ulrike I. Attenberger: Nothing to disclose
Daniel Kütting: Nothing to disclose
Martin Reuter: Nothing to disclose
Narine Mesropyan: Nothing to disclose

### RPS 705-16

**Radiomic features disclose the presence of microvascular invasion in hepatocellular carcinoma**

A. Bevilacqua, F. Coppola, *M. Mottola*, M. Renzulli, G. Vara, A. Rustici, L. V. Pastore, L. Strigari, R. Golfieri; Bologna/IT
*(margherita.mottola@unibo.it)*

**Purpose:** To investigate whether a radiomic analysis performed on hepatocellular carcinoma can reveal the presence of microvascular invasion (MVI).
**Methods or Background:** The dataset includes seventy-one early-stage HCC nodules (Milan criteria, nodule≤3 cm, without macrovascular invasion and extrahepatic spread), where forty-two are MVI positive (MVI+) and twenty-nine are MVI negative (MVI-), at histopathological report obtained after surgery. Radiomic analysis is performed on contrast-enhanced CT imaging during the arterial phase. HCC nodules were manually segmented and eighty-four local first-order radiomic features (RFs) are computed from HCC Region of Interest (ROIs). To avoid overfitting, only one couple of RFs is selected for discriminating MVI+ and MVI- into three steps: (i) a subset of RFs is first selected through LASSO; (ii) linearly correlated couples are discarded; (iii) after computing the ROC curve, the most discriminating couple is selected as that one yielding the highest AUC. The discrimination between MVI+ and MVI- is assessed through specificity and sensitivity computed at the Youden Index (YI).
**Results or Findings:** The selected couple combines a measure of local heterogeneity to mean CT image values in HCC ROIs and allows achieving an AUC=0.86, with specificity=81% and sensitivity=83% at the YI=0.64.
**Conclusion:** The radiomic analysis allows unravelling different inner properties of HCC MVI+ nodules, which show a more heterogeneous tumour pattern with respect to MVI-.
**Limitations:** This radiomic analysis was performed exclusively on arterial-phase contrast enhanced images, although it is reasonable arguing that the next inclusion of venous phase images must only improve the outcomes.
**Ethics Committee Approval:** No 197/2020/Oss/AOUBo
**Funding for this study:** Not applicable
**Author Disclosures:**
Rita Golfieri: Nothing to disclose
Luigi Vincenzo Pastore: Nothing to disclose
Giulio Vara: Nothing to disclose
Arianna Rustici: Nothing to disclose
Margherita Mottola: Nothing to disclose
Matteo Renzulli: Nothing to disclose
Lidia Strigari: Nothing to disclose
Francesca Coppola: Nothing to disclose
Alessandro Bevilacqua: Nothing to disclose

### RPS 705-18

**Influence of different adaptive statistical iterative reconstruction (ASIR-V) levels on computed tomography (CT) radiomic features**

*F. Pucciarelli*, M. Zerunian, T. Polidori, G. Guido, E. Lucertini, B. Bracci, C. Rucci, A. Laghi; Rome/IT
*(pucciarelli.fra@gmail.com)*

**Purpose:** To evaluate the influence of different levels of ASIR-V on CT Radiomic Features.
**Methods or Background:** One-hundred and twenty patients who underwent unenhanced abdominal CT scans with the same scanner (Revolution Evo, GE Healthcare, USA) were analyzed. Subsequently, raw data of filtered-back projection (FBP) were reconstructed with 10 levels of ASIR-V (from 10 to 100%). Two radiologists extracted texture features of liver and kidney tissues using two different regions of interest (ROIs) that were cloned for all eleven different iteration levels datasets. Data were extracted with TexRad Medical Imaging Software. Six different radiomic features (Mean, Standard Deviation (SD), Entropy, Mean of Positive Pixel (MPP), Skewness, Kurtosis) were extrapolated and compared between FBP and all ASIR-V levels.
**Results or Findings:** Texture analysis of the liver revealed significant differences between FBP and all ASIR-V reconstructions for mean (all $p<0.002$), SD (all $p<0.0001$), Entropy (all $p<0.0001$) and MPP (all $p<0.0001$), while no significant differences was observed for Skewness and Kurtosis between FBP and all ASIR-V reconstructions (all $p>0.45$ and all $p>0.58$, respectively). Similar results were obtained for kidney analysis with no differences for Skewness and Kurtosis (all $p>0.053$ and all $p>0.176$, respectively) and significant changes for Mean (all $p<0.0001$), SD (all $p<0.0001$), Entropy (all $p<0.0036$) and MPP (all $p<0.0001$).
**Conclusion:** No influence of iterative reconstructions algorithm was reported for skewness and kurtosis compared to FBP in liver and kidney analysis whereas Mean, SD, Entropy and MPP were significantly affected by ASIR-V. Skewness and Kurtosis may be reliable quantitative parameters.
**Limitations:** Analysis performed on image acquired without contras medium, only on healthy parenchyma and a single vendor.
**Ethics Committee Approval:** This study was approved by our local institutional review board and written informed consent was obtained from all study participants.
**Funding for this study:** No.
**Author Disclosures:**
Francesco Pucciarelli: Nothing to disclose
Carlotta Rucci: Nothing to disclose
Marta Zerunian: Nothing to disclose
Benedetta Bracci: Nothing to disclose
Tiziano Polidori: Nothing to disclose
Andrea Laghi: Nothing to disclose
Gisella Guido: Nothing to disclose
Elena Lucertini: Nothing to disclose

**RPS 705-19**

**Revealing the most suitable CT radiomics features for retrospective studies with heterogeneous datasets**

*O. Jimenez-del-Toro*[1], C. Aberle[2], M. Bach[2], R. Schaer[1], M. M. Obmann[2], E. Konukoglu[3], B. Stieltjes[2], H. Müller[1], A. Depeursinge[4]; [1]Sierre/CH, [2]Basel/CH, [3]Zurich/CH, [4]Lausanne/CH
*(oscar.jimenez@hevs.ch)*

**Purpose:** There is a strong variability of CT images when using regular clinical imaging protocols, as it is often the case in retrospective studies. Their influence on the usability of radiomics features is still unclear. This study aims to assess the stability and discriminative power of radiomics features in 240 CT series of different reconstruction parameter variations using an anthropomorphic abdominal phantom.
**Methods or Background:** A novel anthropomorphic 3D-printed radiopaque CT phantom was created based on a CT of a cancer patient. Regions-Of-Interests (ROIs) were manually annotated in normal liver tissue and 3 lesion types: cysts, hemangioma and metastasis. 86 radiomics features were extracted from the ROIs in 30 repeated phantom acquisitions for 8 groups of CT parameter settings with varying reconstruction algorithms, reconstruction kernels, slice thicknesses and slice spacings. Univariate pairwise Wilcoxon signed-rank tests assessed the statistical significance of the feature variations among the 8 groups and 4 tissue classes.
**Results or Findings:** The variations of CT parameter settings produced statistically significant differences in pairwise comparisons, with more than 70% of the 86 radiomics features showing significant differences in their value distributions. All radiomics features were able to differentiate between the 4 classes of liver tissue in more than 40% of the performed tests. In a principal component analysis, the inter-class variation from the 4 classes was larger than the intra-class variations caused by CT reconstruction parameter variations in the 8 groups.
**Conclusion:** While the tested radiomics features are influenced by CT reconstruction parameters, the features have sufficient stability and discriminative power to differentiate tissue types. The presented approach using a 3D-printed phantom could systematically analyse different scanner types in the future.
**Limitations:** Not applicable
**Ethics Committee Approval:** Not applicable
**Funding for this study:** Swiss Personalized Health Network (SPHN) QA4IQI project DMS2445, SNSF grant 205320_179069
**Author Disclosures:**
Bram Stieltjes: Nothing to disclose
Markus Obmann: Nothing to disclose
Ender Konukoglu: Nothing to disclose
Michael Bach: Nothing to disclose
Adrien Depeursinge: Nothing to disclose
Christoph Aberle: Nothing to disclose
Henning Müller: Nothing to disclose
Oscar Jimenez-del-Toro: Nothing to disclose
Roger Schaer: Nothing to disclose

**RPS 705-20**

**Deep learning-based algorithm yields high accuracy for the detection of small renal cell carcinoma on contrast-enhanced CT**

*N. Toda*[1], M. Hashimoto[1], H. Haque[1], Y. Arita[1], H. Akita[1], T. Akashi[1], H. Gobara[2], A. Nishie[3], M. Jinzaki[1]; [1]Tokyo/JP, [2]Okayama/JP, [3]Fukuoka/JP
*(ntohda@rad.med.keio.ac.jp)*

**Purpose:** Small (<=4 cm) renal cell carcinoma (RCC) is often found incidentally on imaging and to our knowledge, its automatic detection has not been widely investigated. We aimed to develop a deep learning-based algorithm for the detection of small RCC on CT.
**Methods or Background:** Our dataset consisted of 510 nephrogenic and delayed phase CT images with histologically confirmed solid single RCC between January 2005 and March 2020 from Japan Medical Image Database. These images were split into training and test samples (80% and 20%). We developed algorithms using a combination of RCC segmentation and binary classification. We also adopted kidney segmentation for extracting kidneys to improve RCC segmentation result. First, both kidneys were extracted from images by using 3D U-Net kidney segmentation model. Then, RCC-suspected slice was extracted from each kidney by using different 3D U-Net called RCC segmentation model. Finally, RCC-suspected lesion was classified as RCC-positive or RCC-negative with 2D ResNet-34. All models were independently trained from the scratch. We evaluated the performance of the model pipeline with test dataset and compared with the algorithm without kidney extraction step.
**Results or Findings:** Mean diameter of RCC in balanced test dataset was 2.8 cm. Our algorithm with kidney extraction step achieved sensitivity, specificity of 83.3%, 92.2% respectively. AUC of this algorithm was 0.907 and significantly higher than that of the algorithm without kidney extraction step.
**Conclusion:** The proposed deep learning-based algorithm was feasible with high performance for detection of small RCC on multicenter contrast-enhanced CT images. It can contribute to early detection and treatment of small RCC.
**Limitations:** Small sample size of RCC except clear cell subtype. Patients with solid benign tumor were not included as RCC-negative in this study.
**Ethics Committee Approval:** Institutional Review Board approval was obtained.
**Funding for this study:** AMED JP19lk1010025.
**Author Disclosures:**
Masahiro Jinzaki: Research/Grant Support: Radiologist
Toshiaki Akashi: Nothing to disclose
Masahiro Hashimoto: Research/Grant Support: Radiologist
Hasnine Haque: Employee: GE Healthcare
Akihiro Nishie: Nothing to disclose
Hideo Gobara: Nothing to disclose
Naoki Toda: Research/Grant Support: Ph.D student
Yuki Arita: Research/Grant Support: Radiologist
Hirotaka Akita: Nothing to disclose

### Research Presentation Session: Artificial Intelligence & Machine Learning

### RPS 805

### AI in thoraxial imaging

**RPS 805-1**

**Deep learning for improved lung cancer detection on chest radiographs: results of a multi-reader study in national lung cancer screening dataset**

*Y. Hyunsuk*[1], S. S. Lee[1], C. D. Arru[2], R. Singh[2], S. R. Digumarthy[2], M. K. Kalra[2]; [1]Seoul/KR, [2]Boston, MA/US
*(hyunsuky@lunit.io)*

**Purpose:** To assess whether AI improves the reader performance for lung cancer detection using CXRs selected from participants from NLST
**Methods or Background:** This reader study included 519 CXRs selected from the NLST data set. 75 CXRs from 68 patients with visible lung cancers and 444 CXRs belonging to 226 patients were selected. Six readers, including three radiology residents, and three board-certified radiologists, participated in the observer performance test. Performance of the readers without and with AI in terms of visible lung cancer detection and chest CT recommendation was analyzed.
**Results or Findings:** With AI, the average sensitivity of readers for the detection of visible lung cancer increased for residents, but was similar for radiologists compared to that without AI (0.61 [95%CI,0.55-0.67] vs. 0.72 [95%CI,0.66-0.77], p<0.001 for residents, and 0.78 [95%CI,0.73-0.84] vs. 0.78 [95%CI,0.73-0.84, p=1.00 for radiologists), while false-positive findings per image (FPPI) was similar for residents, but decreased for radiologists (0.15 [95%CI,0.11-0.18] vs. 0.14 [95%CI,0.11-0.17], p=0.60 for residents, and 0.36 [95%CI,0.31-0.41] vs. 0.25 [95%CI,0.21-0.30], p<0.001 for radiologists). With AI, the average rate of chest CT recommendation for visible cancer increased for residents, but was similar for radiologists (54.7% [95%CI,48.2%-61.2%] vs. 70.2% [95%CI,64.2%-76.2%], p<0.001 for residents and 72.0% [95%CI,66.1%-77.9%] vs. 74.7% [95%CI,69.0%-80.3%], p=0.11 for radiologists), while that for nonvisible cancer was similar for both groups. On CXRs of seven patients with missed lung cancer, average residents detected more lung cancer with AI (39% [2.7 of 7] vs. 71% [5.0 of 7], p<0.001).
**Conclusion:** An AI algorithm increased sensitivity and decreased false-positives for lung cancer detection for residents and radiologists, respectively. AI can enhance the value of CXRs for lung cancer screening.
**Limitations:** The prevalence of lung cancer was low, and only 98 lung cancer patients were included.
**Ethics Committee Approval:** Approved
**Funding for this study:** None
**Author Disclosures:**
Sanghyup Simon Lee: Employee: Lunit
Mannudeep K Kalra: Nothing to disclose
Chiara Daniela Arru: Nothing to disclose
Yoo Hyunsuk: Employee: Lunit Inc
Ramandeep Singh: Nothing to disclose
Subba Rao Digumarthy: Nothing to disclose

### RPS 805-2

**Computer-aided analysis of airway dimensions for objective diagnosis and monitoring of bronchiectasis and airway wall thickening**

*Q. Lv*[1], L. Gallardo Estrella[2], E-R. Andrinopoulou[1], J-P. Charbonnier[2], M. Kemner van de Corput[1], E. Van Rikxoort[2], M. De Bruijne[1], P. Ciet[1], H. A. W. M. Tiddens[1]; [1]Rotterdam/NL, [2]Nijmegen/NL
*(l.qianting@erasmusmc.nl)*

**Purpose:** Develop and validate an automated method for assessment of airway artery (AA) - dimensions on chest-Computer Tomography (CT) of Cystic Fibrosis (CF) - patients for detection and monitoring of airway wall thickening (AWT) and bronchiectasis.
**Methods or Background:** Lung diseases such as CF are characterized by AWT and bronchiectasis. Manual measurement of AA pairs on chest-CTs is a sensitive but time-consuming method to detect and monitor airways disease. Lung quantification software (LungQ, Thirona, Nijmegen) was used to identify AA-pairs and airway generations (G) to automatically quantify for each AA-pair: outer airway diameter(Aout), inner airway diameter(Ain), artery diameter(A), airway wall thickness (Awt), and the Aout-A, Ain-A, Awt-A ratios. Results presented starting at the first segmental bronchi(G1). 40 out of 70 CT scans from the Erasmus-MC CF-cohort were added to the internal training dataset to make the algorithm robust against CF abnormalities. For validation, LungQ AA-analysis was compared against a manual AA-method of chest-CTs of 11 CF-patients (median 11, [7-16] years) and of 12 age matched control chest-CTs. AA-dimensions, AA-ratios, and the area under the curve (AUC) of CF and controls for segmental generation (G1-G7) were compared between the two methods.
**Results or Findings:** The LungQ AA-analysis detected 4693 AA-pairs, against 4814 AA-pairs by manual method. For Aout/A ratio, and Awt/A ratio outcomes there was no significant difference between the AUC of the LungQ AA-analysis and the manual method from G4 to G7. AUC of Aout/A ratio (from G5 to G7) and Awt/A ratio(from G3 to G7) were above 0.8 for the LungQ AA-analysis, reflecting excellent discrimination.
**Conclusion:** The automatic AA-method showed a high accuracy to detect AWT and bronchiectasis comparable to the manual method.
**Limitations:** Further validation is currently ongoing.
**Ethics Committee Approval:** Mixed case
**Funding for this study:** Dutch CF foundation (PPP grant)
**Author Disclosures:**
Jean-Paul Charbonnier: Shareholder: shareholder at Thirona
Eleni-Rosalina Andrinopoulou: Nothing to disclose
Pierluigi Ciet: Nothing to disclose
Qianting Lv: Nothing to disclose
Mariëtte Kemner van de Corput: Nothing to disclose
Leticia Gallardo Estrella: Employee: scientists working at Thirona
Eva Van Rikxoort: Shareholder: Co-founder and shareholder Thirona
Harm A W M Tiddens: Consultant: Director ErasmusMC Lunganalysis, Consultant for Novartis, Insmed, Thirona, Translate Bio
Marleen De Bruijne: Nothing to disclose

### RPS 805-6

**Pushing the boundaries of deep learning-based image translation: creating PET images from CT images of the lung**

V. K. Venugopal, *V. Mahajan*, H. Mahajan , R. Takhar; Delhi/IN
*(vidur@mahajanimaging.com)*

**Purpose:** To create Positron Emission Tomography (PET) images from Computed Tomography (CT) images using Deep Learning based methods with a focus on the thorax.
**Methods or Background:** 3,878 PET-CT scans were pulled from PACS and anonymised. From these, ~670,000 paired images of contrast enhanced CT and PET of the lung were extracted. CECT images were used since contrast flow can provide a potential surrogate for perfusion, and thereby function, in the tissue. A pre-processing pipeline consisting of linear scaling, SUV normalization (for PET), HU conversion (for CT), and re-windowing to lung window was created. To train the deep learning model an incremental learning approach was utilised comprising a Cycle-GAN (Pix2Pix) to create a baseline PET image which is then passed through a conditional GAN (cascaded UNET with 2 UNETs). A held out test set of 49 PET-CT scans was used for clinical assessment of the model and Peak Signal to Noise Ratio (PSNR) and Structural Similarity Index (SSIM) were used for quantitative assessment of the algorithm.
**Results or Findings:** Training of the deep learning algorithm took 72 hours on two 1080ti GPUs (Nvidia, USA). On the held-out test set, the average PSNR was 16.8638 with standard deviation of 3.34 and median of 16.14 and average SSIM was 0.50 with standard deviation of 0.076 and median of 0.4919. Cases with the least SSIM were analyzed by a clinician and most such cases consisted of images of the abdomen or distorted anatomy. Further deep dive is required to establish correlations between such errors.
**Conclusion:** Our work provides emerging evidence of the fact that it might indeed be possible to extract functional information (PET images) from anatomic scans (CT scans) using deep neural networks.
**Limitations:** Small validation set
**Ethics Committee Approval:** IRB approval obtained
**Funding for this study:** None
**Author Disclosures:**
Rohit Takhar: Nothing to disclose
Harsh Mahajan : Other: Director, Mahajan Imaging Pvt Ltd
Vasantha Kumar Venugopal: Other: Research collaboration, Aikenist Other: Research collaboration, General Electric Company Other: Research collaboration, Lunit Inc. Other: Research collaboration, Predible Health Other: Research collaboration, Koninklijke Philips NV Other: Research collaboration, Qure.ai Other: Research collaboration, Oxipit
Vidur Mahajan: Other: Head of Research at CARING

### RPS 805-7

**Impact of a deep learning AI pneumothorax detection algorithm: comparison of radiologist and bedside clinician performances**

J. M. Sabol[1], T. Zhang[2], *A. Baenen*[2], G. Rao[2], Z. Herczeg[2], B. Heckel[2], P. Tegzes[2]; [1]Sussex, NJ/US, [2]Waukesha, WI/US
*(alec.baenen@ge.com)*

**Purpose:** On-device AI applications assist both radiologists and bedside clinicians. This study compares the impact of a deep-learning-based AI algorithm on detection of pneumothorax (PTX) by radiologists and bedside clinicians.
**Methods or Background:** The AI system evaluated uses frontal chest x-ray classifier and PTX classification algorithms developed from over 32k and 12.5k independent images, respectively. This study used a challenging, independent, dataset of 174 cases with a range of co-morbidities from 3 international clinical sites which was curated by 3 independent radiologists. 48% were positive for PTX, 64% were small. Readers represented clinicians reading chest x-rays including thoracic radiologist and resident, ER physician and resident, Internal Medicine resident, and two nurse practitioners. All readers read each case twice, with and without the aid of AI with minimum of 4-weeks between. PTX detection was assessed with a Multiple-Reader Multiple-Case ROC analysis and sensitivity/specificity analyzed from separate binary responses on the presence/absence of PTX.
**Results or Findings:** ROC AUC of radiologists increased by 0.015 ($p<0.703$) with aid of AI. However, for the other clinicians, the AUC increase of 0.100 was significant ($p<0.035$). Similarly, with the aid of AI, radiologists showed a 1.2% increase in sensitivity and 0.6% increase in specificity whereas for the other clinicians the sensitivity and specificity increased by 6.9% and 12.5% respectively. The significant improvement of non-radiologist clinicians was mostly attributed to improved performance on small PTX with AUC increase of 0.129 ($p<0.012$).
**Conclusion:** Although AI improved performance of all readers, increases were most significant for non-radiologist clinicians. This study supports deployment of AI tools to bedside practitioners who experience the greatest benefit.
**Limitations:** Research with more readers of each category is required to thoroughly define the impact of AI on bedside radiography.
**Ethics Committee Approval:** IRB oversight waived.
**Funding for this study:** Not applicable
**Author Disclosures:**
Beth Heckel: Employee: GE Healthcare
Pál Tegzes: Employee: GE Healthcare
Gireesha Rao: Employee: GE Healthcare
Alec Baenen: Employee: GE Healthcare
John M Sabol: Nothing to disclose
Tianhao Zhang: Employee: GE Healthcare
Zita Herczeg: Employee: GE Healthcare

### RPS 805-8

**Pediatric age estimation from thoracic and abdominal CT scout views using deep learning**

*A. Demircioglu*, L. Umutlu, K. Nassenstein; Essen/DE
*(aydin.demircioglu@uk-essen.de)*

**Purpose:** Age assessment is regularly used in clinical routine by pediatric endocrinologists to determine the physical development or maturity of children and adolescents. In this study, a deep neural network is developed to automatically estimate chronological age from thoracic and abdominal CT scout views in pediatric patients.
**Methods or Background:** In this retrospective study, 1,903 CT scout views from pediatric patients (acquired between January 2013 and December 2018) corresponding to thoracic and abdominal CT scans were collected to train a wide & deep neural network. The network was trained to predict chronological age from the CT scout views and was evaluated on an independent validation set of 494 CT scout views (acquired between January 2019 and July 2020). A

t-test was used to test whether the mean average error was smaller than 2.0 years.
**Results or Findings:** The trained model showed a mean absolute error of 1.30 +/- 1.25 years on the validation data set. The one-sided t-test was statistically highly significant (p < 0.001), i.e. the difference between the predicted and actual chronological age was less than 2.0 years The correlation coefficient was very high (R = 0.95).
**Conclusion:** The chronological age of pediatric patients can be assessed with high accuracy from thoracic and abdominal CT scout views using a deep neural network.
**Limitations:** This study was conducted at a single site using CT scanners from a single vendor. To ensure that the results are valid in other populations, external validation using data acquired with scanners from different vendors is required.
**Ethics Committee Approval:** Ethical approval for this retrospective study was granted by the local ethics committee. Written and informed consent was waived because of the retrospective nature.
**Funding for this study:** None
**Author Disclosures:**
Kai Nassenstein: Nothing to disclose
Lale Umutlu: Nothing to disclose
Aydin Demircioglu: Nothing to disclose

### RPS 805-9

**Determining age, body height and weight from thoracic and abdominal CT scout view**

*A. Demircioglu*, M. S. Kim, K. Nassenstein, L. Umutlu; Essen/DE
*(aydin.demircioglu@uk-essen.de)*

**Purpose:** To automatically determine the age, body height and weight of patients from thoracic and abdominal CT scout views using deep learning methods.
**Methods or Background:** For this retrospective study, 5000 thoracic and abdominal CT scout views (acquired between January 2016 and December 2018) were collected. A wide & deep network was trained to predict the age, body height and width from the CT scout views. Evaluation was performed on an independent validation set of 2000 CT scout views (acquired between January 2019 and July 2020) by computing the mean average error between the predictions and the true outcomes.
**Results or Findings:** For prediction of age, the model showed an accuracy of 6.8 ± 5.7 years, for body height of 4.5 ± 3.5 cm and for body weight of 4.7 ± 6.8 kg. A one-sided t-test indicated that the difference between the predicted and real outcomes to be statistically significantly in all cases, i.e. the difference is less than 10 years, 10 cm and 10 kg resp. A high correlation coefficient could be seen for all three outcomes (R = 0.80, 0.81 and 0.91 resp.).
**Conclusion:** Automated estimation of age, body height and weight from thoracic and abdominal CT scout views can be performed with good accuracy using a deep neural network.
**Limitations:** Some limitations apply to our study: While the CT scout views were acquired from several different scanner models, they all were from a single vendor. An external validation set is necessary to ensure that the trained model generalizes to data acquired from scanners from different vendors and hospital sites.
**Ethics Committee Approval:** Ethical approval for this retrospective study was granted by the local ethics committee. Written and informed consent was waived because of the retrospective nature.
**Funding for this study:** None
**Author Disclosures:**
Moon Sung Kim: Nothing to disclose
Kai Nassenstein: Nothing to disclose
Lale Umutlu: Nothing to disclose
Aydin Demircioglu: Nothing to disclose

### RPS 805-12

**Impact of destructive and bronchiectasis changes on the quality of automatic lung emphysema quantification**

*N. Griva*, P. Gavrilov; St. Petersburg/RU
*(grivamd@gmail.com)*

**Purpose:** The aim of the study is to assess the impact of infectious destructive changes (lung cavities) and bronchiectases on the quality of automatic lung emphysema quantification.
**Methods or Background:** Retrospective analysis of chest CT of 50 patients with pulmonary emphysema combined with lung cavities and bronchiectases was performed. All studies were performed on the same computed tomograph using a standard program. Initially, all CT scans was estimated in a standard "lung window" (-1200/-600HU) by two independent radiologists. For deeper analysis three different software programs were used.
**Results or Findings:** The minimum percentage of detected changes for programs A and C was 0.2%, for program B - 0.3%. Differences in program calculations in one patient ranged from 0 to 17.6%. Bronchiectases were determined in 49 patients and included in the final emphysema volume in 100% of cases by all three programs. Lung cavities were found in 19 out of the 50 patients. In most cases, this changes were considered by programs as emphysema, nevertheless program B gives a slightly better result (9 out of 19), compared to programs A (11 out of 19) and C (13 out 19). We found a significant overstatement of the results of the estimated emphysema percentage by program B, while the difference in the results of programs A and C was within the confidence interval.
**Conclusion:** The presence of lung cavities and bronchiectases in patients with emphysema significantly affects the final result of volumetric analysis. When comparing all three software products, there was a significant overestimation by program B and a good correlation between programs A and C.
**Limitations:** The CT scan did not include spirometric triggering, so we could not completely ensure that imaging studies were obtained during maximum inspiration.
**Ethics Committee Approval:** Approved
**Funding for this study:** None
**Author Disclosures:**
Pavel Gavrilov: Nothing to disclose
Nadezda Griva: Nothing to disclose

### RPS 805-13

**Automated calculation of the right ventricle to left ventricle ratio on CT for the risk stratification of patients with acute pulmonary embolism**

*R. W. Foley*, S. Glenn-Cox, G. Mynott, T. A. Burnett, W. Brown,
J. Suntharalingam, B. J. Hudson, G. Robinson, J. C. L. Rodrigues; Bath/UK

**Purpose:** To assess the feasibility of the use of artificial intelligence post-processing to calculate the right ventricle to left ventricle (RV:LV) ratio on computed tomography pulmonary angiography (CTPA) and to investigate its prognostic value in patients with acute pulmonary embolism (PE).
**Methods or Background:** Single centre, retrospective study of 101 consecutive patients with CTPA-proven acute PE. Ventricular volumes were segmented on 1mm contrast-enhanced axial slices and maximal ventricular diameters were derived for RV:LV ratio using automated post-processing software (IMBIO LLC, USA) and compared to manual analysis in two observers, via intraclass coefficient correlation analysis. Each CTPA report was analysed for mention of the RV:LV ratio and compared to the automated RV:LV ratio. 30-day all-cause mortality was recorded.
**Results or Findings:** Automated RV:LV analysis was feasible in 87% (n=88). RV:LV ratios ranged from 0.67-2.43, with 64% (n=65) >1.0. There was very strong agreement between manual and automated RV:LV ratios (ICC = 0.83, 0.77-0.88). The use of automated analysis led to a change in risk stratification in 45% of patients (n=40). The AUC of the automated measurement for the prediction of all-cause 30-day mortality was 0.77 (95% CI: 0.62 – 0.99).
**Conclusion:** The automated RV:LV ratio on CTPA can be reliably measured in the majority of patients, with perfect reproducibility. The routine use of this automated analysis in clinical practice would add important prognostic information in patients with acute PE.
**Limitations:** This study is limited by its retrospective design. The initial results of this automated analysis are promising. However, before its implementation in clinical practice, it is essential that this technique undergo robust external validation via a multicentre study with larger patient numbers.
**Ethics Committee Approval:** This study was approved by our institution's Trust Audit Committee.
**Funding for this study:** No specific funding was obtained for this study.
**Author Disclosures:**
Sophie Glenn-Cox: Nothing to disclose
Graham Robinson: Nothing to disclose
Tim Alexander Burnett: Nothing to disclose
Georgina Mynott: Nothing to disclose
William Brown: Nothing to disclose
Benjamin James Hudson: Nothing to disclose
Jay Suntharalingam: Nothing to disclose
Robert Foley: Nothing to disclose
Jonathan Rodrigues: Nothing to disclose

### RPS 805-15

**Radiomics based analysis of spectral detector CT derived pulmonary perfusion maps for the diagnosis and classification of pulmonary hypertension**

*R. J. Gertz*, L. Caldeira, R. Shahzad, J-R. Kroeger, F. Gerhardt,
S. Rosenkranz, M. Perkuhn, D. Maintz, A. Bunck; Cologne/DE
*(roman.gertz@uk-koeln.de)*

**Purpose:** To investigate if radiomics-based analysis of normal and malperfused lung regions as derived from spectral-detector CT (SDCT) can be used to identify patients suffering from pulmonary hypertension (PH) and to differentiate between chronic thromboembolic hypertension (CTEPH) and PH due to different etiologies.
**Methods or Background:** 66 consecutive patients with right heart catheter proven PH (62±16 years; male/female ratio, 21/45; CTEPH/noCTEPH 26/41) and 37 controls (62±17 years; m/f ratio, 26/11) who underwent CT pulmonary angiography on a SDCT were included. Lungs were automatically segmented into normal perfused and malperfused areas based on iodine density threshold. 222 Radiomics features were computed, 111 from each region. Two classification tasks were performed: 1)PH vs. controls; 2)CTEPH vs. PH due to different etiologies. The dataset was divided into training (~75%, 50 PH, 29 controls) and test-set (~25%, 16 PH, 8 controls). Mutual information was used for feature pre-selection, recursive feature elimination was performed and a random forest classifier trained using 5-fold-cross-validation(CV).
**Results or Findings:** In the first task, PH vs. controls, 5 features derived from the normal perfused region were opted for classification, resulting in a CV accuracy (ACC) of 0.82±0.05. In the testing dataset, an ACC of 0.79 was attained. Differentiation between CTEPH and PH due to different etiologies, however, appealed more challenging. Selecting 13 features, 10 from the normal perfused and 3 from the malperfused regions lead to a CV ACC of 0.60±0.12, while in the testing dataset, an ACC of 0.68 could be achieved.
**Conclusion:** Radiomics features derived from SDCT pulmonary perfusion maps can identify patients suffering from PH with high accuracy and show promising results differentiating between CTEPH and PH due to other etiologies.
**Limitations:** n/a
**Ethics Committee Approval:** The local ethics committee approved this retrospective study
**Funding for this study:** n/a
**Author Disclosures:**
Rahil Shahzad: Employee: Philips Research
Felix Gerhardt: Nothing to disclose
Stephan Rosenkranz: Nothing to disclose
Liliana Caldeira: Nothing to disclose
David Maintz: Nothing to disclose
Jan-Robert Kroeger: Nothing to disclose
Roman Johannes Gertz: Nothing to disclose
Alexander Bunck: Nothing to disclose
Michael Perkuhn: Employee: Philips Healthcare

### RPS 805-17

**Deep learning for classification and selection of cine CMR images to achieve fully automated quality controlled CMR analysis**
*V. Vergani*, A. King, R. Razavi, E. Puyol-Antòn, B. Ruijsink; London/UK
*(vergani.vittoria@gmail.com)*

**Purpose:** In cardiac magnetic resonance (CMR), image-quality and planning significantly affect cardiac function analysis. In this study, we developed a deep learning pipeline for automated identification and quality control (QC) of cine images from routine clinical cardiovascular magnetic resonance (CMR) exams.
**Methods or Background:** This is a multicenter, multivendor study of 3,448 subjects (114,145 CMR images). The first step of our pipeline identifies potential cine images with a set of heuristic rules (time and number of frames). The second step consisted of a convolutional neural network (CNN)-1 to classify images into conventional cine classes (4Ch, 3Ch, 2Ch, short-axis), or a class of ‘other’. This was followed by another CNN for each long-axis class (2ChCNN-2, 3ChCNN-2, 4ChCNN-2) to classify images according to quality. This was then used as a QC to select the best sequence of each class per patient. Accuracy was computed to determine performance of each CNN using a test-set (10% and 20% of the total images for CNN-1 and CNN-2). To validate the pipeline, we subsequently used it to process 50 full clinical CMR studies, not used for training, and computed Cohen’s coefficient to assess agreement with manual processing.
**Results or Findings:** Accuracy of the CNN-1 was 1.00. Accuracy of 2ChCNN-2, 3ChCNN-2, 4ChCNN-2 were 0.88, 0.87, 0.87 respectively. There was substantial agreement between manual and automated processing (K=0.74).
**Conclusion:** We developed a robust and generalisable algorithm to classify and QC-select CMR images prior to further automated analysis. To the best of our knowledge, this is the first work with this aim in the field of CMR.
**Limitations:** This is a proof of concept study. The pipeline will need to be validated on a larger cohort before widespread use for automated CMR analysis.
**Ethics Committee Approval:** N.a.
**Funding for this study:** N.a.
**Author Disclosures:**
Reza Razavi: Nothing to disclose
Bram Ruijsink: Nothing to disclose
Esther Puyol-Antòn: Nothing to disclose
Vittoria Vergani: Nothing to disclose
Andrew King: Nothing to disclose

### RPS 805-18

**Detecting the pulmonary trunk in CT scout views using deep learning**
A. Demircioglu, M. C. Stein, *M. S. Kim*, L. Umutlu, K. Nassenstein; Essen/DE

**Purpose:** CT pulmonary angiography is an important examination to diagnose or exclude pulmonary embolism. As an initial step towards automating pulmonary angiography in clinical routine, a deep neural network is trained to predict and mark the location of pulmonary trunk in the CT scout view, which is a necessary step for bolus triggering in CT.
**Methods or Background:** For training, 750 patients who have received a thoracic CT scan between March 2003 and February 2020 were randomly chosen and anonymized. In addition, a validation set of 239 CT scout views from patients between March 2020 and May 2020 was collected. The craniocaudal limits of the pulmonary trunk were identified in the 5 mm axial slices by a board-certified radiologist and carried over to the CT scout view. A conditional generative adversarial network was trained on the CT scout views to predict the area of the pulmonary trunk and slice position for bolus-triggering the CT-scan.
**Results or Findings:** Overall, the predictions of the neural network showed high precision (Dice score 0.82). On 6 out of 239 images the slice by the neural network was outside the region of the pulmonary trunk, yielding an accuracy of 97.5%. The mean distance to the slice where the pulmonary trunk was best visible was 4.7 +/- 4.0mm.
**Conclusion:** Deep learning can be used to train an automated detection of regions in CT scout views corresponding to the pulmonary trunk with high precision. Thus, it is a promising initial approach for automating the scan-procedure of CT pulmonary angiography.
**Limitations:** This study was conducted on a single site, an external validation cohort is needed to confirm the results.
**Ethics Committee Approval:** Ethical approval for this retrospective study was granted by the local ethics committee, informed consent was waived.
**Funding for this study:** None
**Author Disclosures:**
Kai Nassenstein: Nothing to disclose
Lale Umutlu: Nothing to disclose
Moon Sung Kim: Nothing to disclose
Aydin Demircioglu: Nothing to disclose
Magdalena Charis Stein: Nothing to disclose

### Research Presentation Session: Artificial Intelligence & Machine Learning

### RPS 905

### AI in musculoskeletal imaging

### RPS 905-1

**CT radiomics-based machine-learning classification of atypical cartilaginous tumours and appendicular chondrosarcomas**
*S. Gitto*[1], R. Cuocolo[2], M. Acquasanta [1], A. Cincotta[1], V. Chianca[1], D. Albano[1], C. Messina[1], A. Annovazzi[3], L. M. Sconfienza[1]; [1]Milan/IT, [2]Naples/IT, [3]Rome/IT
*(sal.gitto@gmail.com)*

**Purpose:** To investigate the diagnostic performance of computed tomography (CT) radiomics-based machine learning for classification of atypical cartilaginous tumors and higher-grade chondrosarcomas of long bones.
**Methods or Background:** One-hundred-twenty patients with surgically treated and histology-proven cartilaginous bone tumors were retrospectively included at two tertiary tumor centers. The training cohort consisted of 84 CT scans from center 1 (n=55 G1 or atypical cartilaginous tumors; n=29 G2-G4 chondrosarcomas). The external test cohort consisted of the CT component of 36 positron emission tomography-CT scans from center 2 (n=16 G1 or atypical cartilaginous tumors; n=20 G2-G4 chondrosarcomas). All tumors were manually segmented on preoperative CT using the axial image showing the maximum lesion extension. First-order, shape-based and matrix features were extracted. After dimensionality reduction, a machine-learning classifier (LogitBoost) was tuned on the training cohort using 10-fold cross validation and tested on the external cohort. In patients from center 2, the classifier’s performance was compared with preoperative biopsy using McNemar’s test.
**Results or Findings:** The classifier had 81% (AUC=0.89) and 75% (AUC=0.78) accuracy in identifying the cartilaginous tumors in the training and external test cohorts, respectively. Specifically, its accuracy in classifying atypical cartilaginous tumors and higher-grade chondrosarcomas was 84% and 78% in the training cohort, and 81% and 70% in the external test cohort, respectively. Preoperative biopsy had 64% (AUC=0.66) accuracy in center 2 (p=0.289).
**Conclusion:** Machine learning showed good accuracy in classifying atypical cartilaginous tumors and higher-grade chondrosarcomas of long bones based on CT radiomic features and could prove a valuable aid in preoperative assessment.
**Limitations:** Retrospective; lack of contrast-enhanced scans.
**Ethics Committee Approval:** Local Ethics Committee approved this retrospective study and waived the need for informed consent.
**Funding for this study:** ESSR Young Researchers Grant (first author)
**Author Disclosures:**
Marzia Acquasanta: Nothing to disclose
Vito Chianca: Nothing to disclose
Carmelo Messina: Nothing to disclose
Salvatore Gitto: Nothing to disclose
Luca Maria Sconfienza: Nothing to disclose
Antonino Cincotta: Nothing to disclose
Renato Cuocolo: Nothing to disclose
Domenico Albano: Nothing to disclose
Alessio Annovazzi: Nothing to disclose

### RPS 905-3

**Automated bone age assessment in a retrospective cohort of German children from the clinical routine: agreement between AI and expert readers**

D. Gräfe[1], A. B. Päts[1], R. Pfäffle[1], *M. DiFranco*[2]; [1]Leipzig/DE, [2]Vienna/AT
*(m.difranco@imagebiopsy.com)*

**Purpose:** The radiological determination of BA from a left-hand x-ray continues to be the reference standard for skeletal maturity assessment related to short or long stature, premature or delayed puberty, and underlying conditions. Artificial intelligence (AI) algorithms are becoming more prevalent due to the subjectivity and time-consuming nature of BA assessment. In this study we evaluate the agreement between AI and expert readers for bone age (BA) assessment according to the Greulich & Pyle method.
**Methods or Background:** Radiographs of 259 patients were analyzed retrospectively (133 males aged 2 to 17 years, 126 females aged 2 to 16 years). Two pediatric radiologists and one pediatric endocrinologist made blind reads of BA using the Greulich & Pyle (GP) method independently, and an AI-software was subsequently used to estimate BA from the same images. Agreement of AI with readers was assessed based on mean absolute deviation (MAD) and root mean squared deviation (RMSD), as well as via comparison of Bland-Altman limits of agreement (LOA).
**Results or Findings:** MAD and RMSD of AI vs. reader average (0.51 and 0.66 years) was less than that for mean of three reader pairs (0.66 and 0.86 years, $p<0.05$ for each reader pair). Bland-Altman LOA between AI and reader average was (-1.22, 1.35 years), well within the mean LOAs of the three reader pairings (-1.62, 1.75 years).
**Conclusion:** A fully automated AI software shows agreement with expert readers in BA assessment on a cohort of German children and adolescents, suggesting AI integration into the radiology workflow is possible and could lead to more efficient bone age reading.
**Limitations:** n/a
**Ethics Committee Approval:** This retrospective study was approved by the institutional review board and informed consent was waived.
**Funding for this study:** n/a
**Author Disclosures:**
Daniel Gräfe: Nothing to disclose
Anne Bettina Päts: Nothing to disclose
Roland Pfäffle: Nothing to disclose
Matthew DiFranco: Employee: ImageBiopsy Lab

### RPS 905-4

**Increasing the detection of Vertebral Fractures (VFs) using AI on CT performed for all clinical indications**

*R. Gordon*[1], B. Gordon[2], K. White[2], H. Lebel[1], E. Spitz[1], E. Blumenfeld[1], E. Elnekave[1]; [1]Shfayim/IL, [2]Salt Lake City, UT/US
*(Ronen@zebra-med.com)*

**Purpose:** We report on the diagnostic accuracy and potential for clinical impact of software which detects VFs.
**Methods or Background:** A neural network based VF detection tool was generated utilizing a training set of 1,832 CT scans including compression fractures indexed according to the Genant method. Ground truth was established by three expert radiologists. Validation was performed on a novel dataset of 611 CT scans of the chest and abdomen. To assess for clinical impact, the software was applied to 76,069 anonymized CTs acquired from a U.S. based integrated health delivery system comprising 24 hospitals. The threshold for a positive result was set at the presence of at least one moderate VF. Each corresponding CT report was evaluated by a combination of human and natural language processing. 290 studies were reviewed by two expert radiologists to verify consistent algorithmic accuracy.
**Results or Findings:** The software achieved an overall AUC of 0.95 with sensitivity 90.6% and specificity of 86.6% for the detection of VFs. VFs were noted in 4,996 (6.6%) of 76,069 radiology reports; of these, 3,167 were also detected algorithmically. VFs were detected by the software in a total of 11,890 (15.6%) CT examinations (Fig 1). Of the 290 CTs analyzed for consistent accuracy, results achieved a negative predictive value of 90.7% and positive predictive value of 58.2%. Of the 94 false positive cases, 72 (76.6%) comprised studies with mild compression fractures.
**Conclusion:** An automatic VF detection tool with high diagnostic accuracy demonstrates potential to substantially improve the current reporting gap for vertebral compression fractures, increasing the detected moderate-severe VFs by 138%.
**Limitations:** The study was limited by its retrospective nature.
**Ethics Committee Approval:** Approved by IRB
**Funding for this study:** This study was supported by research funds of Intermountain Healthcare and Zebra `medical Vision.
**Author Disclosures:**
Keith White: Nothing to disclose
Ronen Gordon: Employee: Zebra Medical Vision
Einav Blumenfeld: Employee: Zebra Medical Vision
Eldad Elnekave: Employee: Zebra Medical Vision
Eyal Spitz: Employee: Zebra Medical Vision
Hila Lebel: Employee: Zebra Medical Vision
Ben Gordon: Nothing to disclose

### RPS 905-5

**How much does non-blinded assessment shift the goalpost for AI algorithms? Exploring the hidden role of bias in AI evaluation using knee osteoarthritis grading as an example**

V. Desai[1], J. Belair[1], G. Nanda[2], *V. K. Venugopal*[2], R. Takhar[2], V. Mahajan[2], H. Mahajan [2]; [1]Philadelphia, PA/US, [2]New Delhi/IN
*(vasanthdrv@gmail.com)*

**Purpose:** To study the change in the performance of AI results when tested on ground truth established by blinded and non-blinded assessments.
**Methods or Background:** Anteroposterior views of 271 knee x-rays (542 joints) were randomly extracted from PACS and anonymized. These x-rays were analyzed using DeepKnee, an open-source algorithm based on the Deep Siamese CNN architecture that automatically predicts the presence of osteoarthritis on Knee X Rays on a 5 scale Kellgren and Lawrence system (KL). The algorithm also provides an attention map that explains the decision made by the network. These x-rays were independently read by three subspecialist MSK radiologists on the CARPL AI research platform (CARING Research, India). The KL grade for each x-ray was recorded by the radiologists, following which the AI algorithm grade was shown, and radiologists given the option to change their result. The pre-AI result and post-AI results were both recorded. Ground Truth (GT) KL grade was considered to be the one with a consensus between at least two readers. In cases with a disagreement between all three readers, the median was considered. The performance of AI was assessed using misclassification rate and change in misclassification rates for AI as well as the individual readers were compared for both phases.
**Results or Findings:** The misclassification rate of the AI algorithm reduced from 0.43 in the blinded phase to 0.37 during the non-blinded phase. The misclassification rates for the readers changed from 0.25, 0.18, and 0.22 to 0.2, 0.14 and 0.23 respectively. Krippendorff's alpha among the readers improved from 0.84 & 0.87 between the phases.
**Conclusion:** We demonstrate that the perceived performance of an algorithm can show spurious improvement when tested by radiologists in a non-blinded fashion.
**Limitations:** Small sample set
**Ethics Committee Approval:** Not required
**Funding for this study:** None
**Author Disclosures:**
Vishal Desai: Nothing to disclose
Jeffrey Belair: Nothing to disclose
Geetanjali Nanda: Nothing to disclose
Rohit Takhar: Nothing to disclose
Harsh Mahajan : Other: Research collaboration, Oxipit Other: Research collaboration, General Electric Company Other: Research collaboration, Lunit Inc. Other: Research collaboration, Aikenist Other: Research collaboration, Koninklijke Philips NV Other: Research collaboration, Qure.ai Other: Director, Mahajan Imaging Pvt Ltd Other: Research collaboration, Predible
Vasantha Kumar Venugopal: Other: Research collaboration, Lunit INC Other: Research collaboration, Predible Health Other: Research collaboration, Koninklijke Philips NV Other: Research collaboration, Oxipit Other: Research collaboration, General Electric Company Other: Research collaboration, General Electric Company Other: Research collaboration, Qure.ai

Vidur Mahajan: Other: Research collaboration, Qure.ai Other: Research collaboration, Predible Other: Research collaboration, General Electric Company Other: Research collaboration, Lunit Inc. Other: Research collaboration, Koninklijke Philips NV Other: Research collaboration, Aikenist Other: Head of Research at CARING Other: Research collaboration, Oxipit

### RPS 905-6

**Effects of interobserver variability on 2D and 3D CT- and MRI-based texture feature reproducibility of cartilaginous bone tumors**

*S. Gitto*[1], R. Cuocolo[2], I. Emili[1], L. Tofanelli[1], V. Chianca[1], D. Albano[1], C. Messina[1], M. Imbriaco[2], L. M. Sconfienza[1]; [1]Milan/IT, [2]Naples/IT
*(sal.gitto@gmail.com)*

**Purpose:** To investigate the influence of interobserver manual segmentation variability on the reproducibility of 2D and 3D unenhanced computed tomography (CT)- and magnetic resonance imaging (MRI)-based texture analysis.
**Methods or Background:** This retrospective study included 30 patients with cartilaginous bone tumors (10 enchondromas, 10 atypical cartilaginous tumors, 10 chondrosarcomas). Three radiologists independently performed manual contour-focused segmentation on unenhanced CT, T1-weighted and T2-weighted MRI by drawing both a 2D region of interest (ROI) on the slice showing the largest tumor area and a 3D ROI including the whole tumor volume. Additionally, a marginal erosion was applied to both 2D and 3D segmentations to evaluate the influence of segmentation margins. A total of 783 and 1132 features were extracted from original and filtered 2D and 3D images, respectively. Intraclass correlation coefficient ≥0.75 defined feature stability.
**Results or Findings:** In 2D vs. 3D contour-focused segmentation, the rates of stable features were 74.71% vs. 86.57% (p<0.001), 77.14% vs. 80.04% (p=0.142) and 95.66% vs. 94.97% (p=0.554) for CT, T1-weighted and T2-weighted images, respectively. Margin shrinkage did not improve 2D (p=0.343) and performed worse than 3D (p<0.001) contour-focused segmentation in terms of feature stability. In 2D vs. 3D contour-focused segmentation, matching stable features derived from CT and MRI were 65.8% vs. 68.7% (p=0.191), and those derived from T1-weighted and T2-weighted images were 76.0% vs. 78.2% (p=0.285).
**Conclusion:** 2D and 3D radiomic features of cartilaginous bone tumors extracted from unenhanced CT and MRI are reproducible, although some degree of interobserver segmentation variability highlights the need for reliability analysis in future studies.
**Limitations:** Single institution; retrospective; lack of contrast-enhanced scans.
**Ethics Committee Approval:** Local Ethics Committee approved this retrospective study and waived the need for informed consent.
**Funding for this study:** None
**Author Disclosures:**
Ilaria Emili: Nothing to disclose
Vito Chianca: Nothing to disclose
Laura Tofanelli: Nothing to disclose
Carmelo Messina: Nothing to disclose
Salvatore Gitto: Nothing to disclose
Luca Maria Sconfienza: Nothing to disclose
Massimo Imbriaco: Nothing to disclose
Renato Cuocolo: Nothing to disclose
Domenico Albano: Nothing to disclose

### RPS 905-7

**Whole-body Bone Age Assessment (BAA) based on deep-learning**

*R. Louiset*[1], A. Ducarouge[2]; [1]Saclay/FR, [2]Paris/FR
*(robin76lt@gmail.com)*

**Purpose:** Development of an AI algorithm to assess bone age from X-rays of any part of the body.
**Methods or Background:** Many scientific papers already address the Deep Learning bone age assessment problem and compare their results with the Greulich and Pyle atlas performance. We managed to reproduce these results with hand radiographs and extended the scope of our model to whole body radiographs with a wider age range. Our approach enabled us to compare each bodypart bone age assessment relevance. Furthermore, we also produced heat maps to enforce our algorithm explainability.
**Results or Findings:** The Mean Average Error (MAE) of the Deep Learning model on representative hand test set of 3 409 images was 11.37 months (std=0.2 months), compared with 11.1 months for a comparable litterature method having a different clean testing set (214 images) of developmentally normal pediatric hand radiographs. Our algorithm yields a MAE of 13.68 (std=0.45) months on growth retardation suspicious pediatric hand test set, compared with 13.53 (std=0.44) months for a cohort of random radiologists. We observed comparable metrics on other body part radiographs such as Pelvis (MAE=10.68+/-0.50), Foot (MAE=11.70+/-0.20), Leg (MAE=13.06+/-0.30).
**Conclusion:** We emphasized the relevance of using chronological age to update and improve old and biased skeletal maturity assessment atlas. We demonstrated bone age state-of-the-art litterature comparable performances on hand, broadening it upon whole body parts radiographs. Finally, we produced a method in order to highlight the zones our model relies on for explainability purposes.
**Limitations:** The MAE metric between predicted bone age and patient chronological age suffers from a natural bias and standard deviation, further studies should propose metrics to mitigate these limitations.
**Ethics Committee Approval:** None, already fully anonymized retrospective data.
**Funding for this study:** Research internship funded by Gleamer, a French AI-based medical imaging company.
**Author Disclosures:**
Robin Louiset: Other: Gleamer
M. Alexis Ducarouge: Founder: Gleamer

### RPS 905-8

**Visual, structured report of spinal trauma: evaluation of a diagnostic and decisional reasoning assistant**

*F. Lafourcade*[1], J-N. Ravey[2], N. Sans[1], M. Faruch-Bilfeld[1]; [1]Toulouse/FR, [2]Grenoble/FR
*(fphlaf@orange.fr)*

**Purpose:** To evaluate a visual structured report produced by a computerized reasoning assistant (Keydiag), compared to a traditional radiology report, in the context of spinal trauma.
**Methods or Background:** A model of spinal trauma was developed with Keydiag support. Its goal is to generate structured reports. Consecutive polytrauma whole-body CT were analyzed. Patients with complex traumatic spinal injuries were selected. Their traditionnal radiology reports were grouped together. Structured reports were produced for these patients. Completeness, surgeons' assessment of reports quality, and Keydiag reproducibility were evaluated.
**Results or Findings:** 1001 bodyscanners were performed over 1 year. Among them, 808 vertebral stages of 288 rachis were injured, 124 in a complex manner. 6 of them were not accessible for technical reasons. 118 structured reports were produced and compared with traditionnal reports. Completeness was significantly higher in the structured group (+20.7% information). The evaluation of their average overall quality was better in structured group (76%) than in the traditional group (67%). The reproducibility of the Keydiag model for AOSpine was very high (κ=0.80).
**Conclusion:** Spinal trauma structured report provides an innovative and useful aid to the radiologist, more complete and qualitative, for the benefit of his correspondents and the patient.
**Limitations:** Monocentric study.
Keydiag software was a beta version.
**Ethics Committee Approval:** Not concerned.
**Funding for this study:** None.
**Author Disclosures:**
Nicolas Sans: Nothing to disclose
Jean-Noël Ravey: Nothing to disclose
François Lafourcade: Nothing to disclose
Marie Faruch-Bilfeld: Nothing to disclose

### Research Presentation Session: Artificial Intelligence & Machine Learning

### RPS 1005

### Advances in AI

### RPS 1005-1

**A novel thick-to-thin-cut generation model for CT imaging**

*W. H. K. Chiu*, W. Cao, C. S. G. Lui, Y. Fang, A. Yap, W. K. W. Seto, P. L. H. Yu; Hong Kong/HK
*(wmingcao@hku.hk)*

**Purpose:** Constraints for Picture Archiving and Communication System mean that although Computed Tomography (CT) images are acquired in thin slices, they had to be stored as thick slices to reduce storage. This results in significant data loss that affects analyses.
**Methods or Background:** We developed a synthetic model based on generative adversarial networks (GAN): the generator turns thick-cut into thin-cut images to fool the discriminator while the discriminator tries to distinguish between generated and real data. New cost functions related to content and depth estimation are introduced to further improve the image quality. Mean squared error (MSE) and structural similarity (SSIM) were used to quantify the quality of synthetic thin-cut CT images. Dice coefficient (DC) was used to compare performances of liver lesion segmentation on real and synthetic thin-cut CT images.
**Results or Findings:** 1390 contrast-enhanced abdominal CT scans with thin (1 mm) and corresponding thick (5 mm) slices were retrieved from our institute. CT images were normalized to the range [0, 255] and split into training, validation and testing with a 6:1:3 ratio. Our algorithm achieved a MSE of 57.10, indicating the mean pixel-wise error is 7.56, and SSIM of 0.934 when thick-cut images are from maximizing pixel-wise intensity. Additionally, the segmentation performance of liver lesion on real and synthetic thin-cut CT images were 0.836 and 0.829 quantified by DC, respectively.
**Conclusion:** Our results highlight the feasibility of generating high-quality thin-cut CT images from thick-slice with no compensation in image quality. In the era of big-data mining in medical imaging, our technique can be used to retrieve historical data and applied to enhance imaging quantitative analyses.
**Limitations:** Feasibility study with no external validation
**Ethics Committee Approval:** Approved by the HKU/HA HKW IRB
**Funding for this study:** Innovation and Technology Fund and United Ally Research limited
**Author Disclosures:**
Wenming Cao: Nothing to disclose
Yanwen Fang: Nothing to disclose
Chiu Sing Gilbert Lui: Nothing to disclose
Alistair Yap: Nothing to disclose
Wan Hang Keith Chiu: Nothing to disclose
Wai Kay Walter Seto: Nothing to disclose
Philip Leung Ho Yu: Nothing to disclose

### RPS 1005-2

### Automated TICI scoring from 2D DSA images

*R. Su*[1], S. A.P. Cornelissen[1], M. van der Sluijs[1], A. van Es[2], W. Van Zwam[3], P. J. van Doormaal[1], W. J. Niessen[1], A. Van Der Lugt[1], T. van Walsum[1]; [1]Rotterdam/NL, [2]Leiden/NL, [3]Maastricht/NL
*(ruisheng.su@hotmail.com)*

**Purpose:** The Thrombolysis in Cerebral Infarction (TICI) score is a common technical outcome assessment for endovascular treatment (EVT) in acute ischemic stroke. Existing TICI scores are defined in coarse ordinal grades based on visual inspection, leading to inter- and intra-observer variation. In this work, we investigate whether such scores can be computed automatically.
**Methods or Background:** The automated scoring method consists of three stages. First, each digital subtraction angiography sequence is separated into four phases (non-contrast, arterial, parenchymal and venous phase) using a convolutional neural network. Next, a minimum intensity map is computed using the motion corrected arterial and parenchymal frames, on which the vessel, perfusion and background pixels are segmented. Finally, the autoTICI score is computed as the ratio of reperfused pixels after EVT.
**Results or Findings:** The algorithm pipeline was evaluated on a subset of 141 MR CLEAN registry subjects. The automated TICI scoring method showed good correlation with the extended TICI (eTICI) reference with an average area under the curve score of 0.81. The AUC score was 0.90 with respect to the dichotomized eTICI. In addition, autoTICI exhibited overall comparable capability to eTICI with respect to outcome prediction.
**Conclusion:** TICI scores can be quantified in an automated way.
**Limitations:** Not applicable
**Ethics Committee Approval:** The ethics committee of the Erasmus Medical Center Rotterdam evaluated the MR CLEAN Registry protocol and approved the study. All subjects gave written informed consent in accordance with the Declaration of Helsinki.
**Funding for this study:** The MR CLEAN Registry was partly funded by unrestricted grants from TWIN Foundation, Erasmus MC University Medical Center, Maastricht University Medical Center, and Amsterdam University Medical Centers. The current work was financially supported by Health-Holland (TKI Life Sciences and Health), Q-Maestro project (grant number: EMCLSH19006) and Philips Healthcare (Best, The Netherlands).
**Author Disclosures:**
Theo van Walsum: Grant Recipient: Erasmus MC
Ruisheng Su: Nothing to disclose
Aad Van Der Lugt: Grant Recipient: Erasmus MC
Pieter Jan van Doormaal: Nothing to disclose
Sandra A.P. Cornelissen: Nothing to disclose
Matthijs van der Sluijs: Nothing to disclose
Adriaan van Es: Nothing to disclose
Willem Van Zwam: Nothing to disclose
Wiro J. Niessen: Nothing to disclose

### RPS 1005-3

### Enabling privacy-preserving training of deep learning models for radiological applications using federated learning

*K. S. Younis*; Waukesha, WI/US
*(kh.younis@gmail.com)*

**Purpose:** Covid19 Pandemic highlighted the need to collaborate and quickly access whatever data is available worldwide in order to develop AI models to aid in early diagnosis. Mastering the art of collaboration is a necessity to succeed in our highly complex and networked world. Federated Learning (FL) is a privacy-preserving collaborative framework for model development that solves many problems related to quick sharing of data. The purpose of this proof of concept is to establish the infrastructure and environment utilizing an FL framework solving a real-world problem of chest X-Ray disease classification.
**Methods or Background:** The Stanford University CheXpert dataset (224 thousand images) was divided into five parts. Each image can have zero or more label “multi-label” out of 15 different labels. CheXpert challenge website reports the Area Under the average ROC curve (AUC) performance for five diseases (a) Atelectasis, (b) Cardiomegaly, (c) Consolidation, (d) Edema, and (e) Pleural Effusion. Nvidia Clara Train SDK that supports FL was installed on the server at Vanderbilt University and in the five client sites. The DenseNet121 model architecture along with the hyper-parameters were included. Each client site created the self-signed certificates and exchanged them with the server host. Connectivity and proper port selection were tested, and training was completed.
**Results or Findings:** The FL global model when tested from sites depicts overall high performance of AUC of 0.8031- 0.8034. Model Averaging works well for identically distributed data.
**Conclusion:** Federated learning is a viable tool for training deep learning models while preserving the data privacy. A DenseNet121 model for disease pattern detection in chest x-rays was able to train securely and efficiently on geographically distributed data with excellent performance.
**Limitations:** n/a
**Ethics Committee Approval:** n/a
**Funding for this study:** n/a
**Author Disclosures:**
Brad Genereaux: Employee: NVIDIA
Liangqiong Qu: Nothing to disclose
Bennett A. Landman: Nothing to disclose
Anas Abidin: Nothing to disclose
Amilcare Gentili: Nothing to disclose
Paras Lakhani: Nothing to disclose
Khaled Salem Younis: Employee: Philips
Nishank Singla: Employee: Sr. Software Engineer at GE Healthcare
Nathaniel Swinburne: Nothing to disclose
Daniel Rubin: Nothing to disclose
Vishwesh Nath: Employee: Nvidia (Applied Research Scientist)

### RPS 1005-4

### Deep scatter estimation (DSE) and correction for dual-source dual-energy CT

*J. F. J. Erath*[1], T. Vöth[2], J. Maier[2], E. Fournié[1], M. Petersilka[1], K. Stierstorfer[1], M. Kachelrieß[2]; [1]Forchheim/DE, [2]Heidelberg/DE
*(julien.erath@dkfz.de)*

**Purpose:** To correct for forward and cross scattered radiation in dual source dual energy CT.
**Methods or Background:** Dual source dual energy CT operates the two x-ray tubes at different tube voltages. Forward and cross-scattered radiation can lead to strong artifacts. To remove the scatter from the measured projections we developed two deep convolutional neural networks (CNN). The forward DSE network is estimating the forward scatter based on the actual projection. To estimate cross-scatter we use cross DSE, a CNN that uses as input the actual projection and a cross-scatter approximation. The latter is calculated through a raytracing operation. The scatter contributions are obtained through our in-house Monte Carlo simulation, simulating a dual source CT system. The CNNs are trained in dual energy mode (80 kV, 140 kV). As a quantitative evaluation we determine the lung density and the mean lung CT number for three patients and compared them to the values obtained without scatter correction and with a measurement-based scatter correction (based on additional detector rows).
**Results or Findings:** DSE is able to remove the scatter artifacts and improves the accuracy of the CT values. The mean error in the lungs without scatter correction is about 14 HU. With DSE this error is reduced to 2.5 HU. The measurement-based scatter correction reduces the error to 1.5 HU.
**Conclusion:** DSE improves the accuracy of CT numbers for quantitative CT imaging. The accuracy of the corrected CT values is similar to a measurement-based correction technique but does not need additional detector sensors.

**Limitations:** Prior to predicting the actual cross-scatter intensity, the second input to xDSE needs a low resolution reconstruction to perform the raytracing operation.
**Ethics Committee Approval:** Approved
**Funding for this study:** Siemens Healthcare
**Author Disclosures:**
Eric Fournié: Employee: Siemens Healthcare
Tim Vöth: Nothing to disclose
Julien Frank Josef Erath: Employee: Siemens Healthcare
Martin Petersilka: Employee: Siemens Healthcare
Karl Stierstorfer: Employee: Siemens Healthcare
Marc Kachelrieß: Nothing to disclose
Joscha Maier: Nothing to disclose

### RPS 1005-5

**Clinical use of artificial intelligence (AI) in radiology departments in the Netherlands: a survey**

*K. G. van Leeuwen*[1], M. Rutten[2], S. Schalekamp[1], M. De Rooij[1], B. Van Ginneken[1]; [1]Nijmegen/NL, [2]'s-Hertogenbosch/NL
*(kicky.vanleeuwen@radboudumc.nl)*

**Purpose:** There are over 150 artificial intelligence (AI) products for radiology offered, but little is known about their current clinical use. We investigated actual clinical use of AI software in radiology departments in the Netherlands.
**Methods or Background:** We consulted the radiology department of each hospital organization in the Netherlands (n=70) about their current AI implementations and plans from February-March 2020. A representative of the department was asked to fill in a questionnaire about their knowledge, experience, research and/or clinical use of commercially available CE-certified AI products for radiology (n=93).
**Results or Findings:** The response rate of the consulted hospitals was 43/70: 38/62 for general hospitals, 5/7 for academic medical centers, and 0/1 for children's hospitals. Of the respondents 30 (70%) were radiologists, 5 (12%) application or information managers, and 8 (19%), among others, clinical physicists and managers. A third (14) of the participating organizations had one to three AI applications in clinical use, with a total of 19 implementations. These implementations involved eight different vendors of which four were from the Netherlands. Most commonly used was software for bone age prediction and stroke detection. Respondents were most familiar with products aimed at neurology and cardiology. MR, CT and mammography were the most familiar modalities for AI. Most interest for clinical implementation was shown in software to triage exams. Eleven organizations (26%) had a dedicated budget for AI, either from the hospital or the department.
**Conclusion:** Even though the supply of AI software is extensive, clinical use remains limited showing that we are still in the initial stages of integrating AI in clinical practice in the Netherlands.
**Limitations:** As not all hospitals responded to our request, bias may be present.
**Ethics Committee Approval:** N.a.
**Funding for this study:** This work has not received any funding.
**Author Disclosures:**
Matthieu Rutten: Nothing to disclose
Bram Van Ginneken: Founder: Thirona Other: Mevis Medical Solutions Other: Delft Imaging Shareholder: Thirona
Kicky Gerhilde van Leeuwen: Nothing to disclose
Steven Schalekamp: Nothing to disclose
Maarten De Rooij: Nothing to disclose

### RPS 1005-6

**Qualitative and quantitative comparison of image quality of upresolved simulated fast MR acquisitions and standard of care images**

V. Batra, J. S. Chatha, *V. K. Venugopal*, R. Takhar, V. Mahajan, H. Mahajan ; New Delhi/IN
*(vasanthdrv@gmail.com)*

**Purpose:** To make qualitative and quantitative comparisons of the images generated by a super-resolving algorithm from simulated undersampled MR brain and standard of care images.
**Methods or Background:** The retrospective study was conducted on 50 MRI pathological scans acquired on Siemens Magnetom 1.5T. Two types of undersampling were done, undersampling by 3 and undersampling by 4 in the K space which simulated fast MR acquisition. These images were then converted to resemble undersampled images. Undersampling by 3 and 4 will be equivalent to acquiring an MRI signal by 1/3rd and 1/4th of the time respectively. The up-resolution is done on this simulated undersampled data by a customized CNN network based on encoder-decoder CNN with 14.8 million parameters by Aikenist. The three sets of images were randomized, and presented to two neuroradiologists for evaluation of their overall quality on a five-point Likert scale (1- nondiagnostic,2 -poor quality,3- diagnostic,4-good,5- excellent). The parameters for evaluation were grey-white matter differentiation (for CNR), delineation of pathologies, delineation of normal deep grey matter anatomy, and artifacts. The overall image quality score was then estimated for each set of images and compared. The Peak SNR and SSIM values were also calculated for each set of images with respect to the SOC images.
**Results or Findings:** The average SSIM for both the 1/3rd undersampled and 1/4th undersampled images was 0.95 & 0.97. The PSNR for the 1/3rd undersampled and 1/4th undersampled images were 36.03 dB and 33.90 dB. The average qualitative scores for the SOC, 1/3rd undersampled, and 1/4th undersampled images were 11.16, 11.11 & 10.98 out of 20.
**Conclusion:** We demonstrate that the super-resolved undersampled images are comparable to the routine SOC images both in quantitative and qualitative comparisons.
**Limitations:** Small study sample
**Ethics Committee Approval:** Not required
**Funding for this study:** None
**Author Disclosures:**
Jagneet Singh Chatha: Nothing to disclose
Rohit Takhar: Nothing to disclose
Harsh Mahajan: Other: Research collaboration, Koninklijke Philips NV Other: Research collaboration, Lunit INC Other: Research collaboration, General Electric Company Other: Director, Mahajan Imaging Pvt Ltd Other: Research collaboration, Predible Health Other: Research collaboration, Qure.ai
Vasantha Kumar Venugopal: Other: Research collaboration, Lunit Inc. Other: Research collaboration, Aikenist Other: Research collaboration, Koninklijke Philips NV Other: Research collaboration, Qure.ai Other: Research collaboration, General Electric Company Other: Research collaboration, Predible Health
Vikas Batra: Nothing to disclose
Vidur Mahajan: Other: Research collaboration, Qure.ai Other: Research collaboration, Koninklijke Philips NV Other: Research collaboration, General Electric Company Other: Head of Research at CARING Other: Research collaboration, Predible Health Other: Research collaboration, Lunit Inc. Other: Research collaboration, Aikenist

### RPS 1005-7

**How to select and fine-tune an artificial intelligence (AI) service for practical application in radiology**

*A. E. Andreychenko*, V. Gombolevskiy, A. Vladzymyrskyy, S. Morozov; Moscow/RU
*(a.andreychenko@npcmr.ru)*

**Purpose:** There are multiple AI solutions in radiology that are positioned as ready for practical clinical use by their creators. However, a wide application of AI in radiology departments at non-academic sites is yet to be proven feasible and justified. Here we propose a universal framework for selection and adjusting an AI service performance for practical use on a megalopolis scale.
**Methods or Background:** Methodologies of software testing by end-users; quality assurance for medical diagnostic equipment and diagnostic tools' accuracy assessment were used. Three routine use cases were chosen: chest Xray, mammography and chest CTs. The following AI functionality was tested: worklist's triage, pathological findings indications on the images as an additional series and report template. Prospective applications of the preselected and fine-tuned AI solutions were being assessed within the unified Moscow RIS that connects > thousand diagnostics devices and > 1400 radiologists.
**Results or Findings:** The framework resulted in 5 steps: (1) is a so-called "self-test" with a representative dataset of the dicom files of the RIS devices; (2) is a functional test by means of a small dataset to check whether all AI features work properly within the RIS; (3) is a calibration test to fine-tune an AI model diagnostic accuracy on the balanced dataset that represents population and mimics settings for prospective application; (4) is a QA test for each radiology device; (5) is a periodical monitoring.
**Conclusion:** The framework showed ability to predict well real-world performance of AI solutions in radiology, to maximize its' stability and minimize risks of failures during the prospective applications.
**Limitations:** The framework was tested on limited amount of use cases for AI in radiology.
**Ethics Committee Approval:** The work was approved by the local ethical committee.
**Funding for this study:** No funding was received for this work.
**Author Disclosures:**
Sergey Morozov: Nothing to disclose
Anna Evgenevna Andreychenko: Nothing to disclose
Anton Vladzymyrskyy: Nothing to disclose
Victor Gombolevskiy: Nothing to disclose

**RPS 1005-8**
**Evaluation of artificial intelligence (AI)-based intelligent reader of medical articles (IRMA) in radiology**
*G. Bartal*[1], V. Guryanova[2], O. Leshchinskaya[2], M. Ayal[1], L. Kokov[2], V. Sinitsyn[2]; [1]Tel Aviv-Yafo/IL, [2]Moscow/RU
*(gbartal@gmail.com)*

**Purpose:** Reading scientific papers for non-native English speakers' is time consuming and deterring. We evaluated AI based Intelligent Reader of Medical articles (IRMA) that reduces the word count by ~70% maintaining article contents. Our aim was evaluation of IRMA by non-native English speakers utilizing validated Linkert scale questionnaires.
**Methods or Background:** IRMA is based on advanced text processing techniques comprising neural network-based language models, extraction of topics from texts and graph-based text representation. A proprietary data set was used for configuration of the resulting solution. PDFs of 3 articles from leading medical journals and their summarization by IRMA have been firstly evaluated by 25 4-6th year medical students and by 15 radiologists, using validated questionnaires. Mean time of cloud-based article analysis by IRMA is about 2.5 minutes.
**Results or Findings:** Based on the analysis of questionnaires on 3 articles by 2 groups we observed several differences in using the tool. (1) As 96% of the students noted that IRMA meets well their needs, only 67% of the radiologists shared that view (p<0.01). (2) 72% of the students plan to read more than 11 articles/month using this tool, whereas only 46% intend to follow that number amongst the radiologists (p<0.05). (3) Although 93% of radiologists found that AI based summarization is accurate and does not contain plethoric information, 72% of the students conceived that impression (p<0.01). Of note is an agreement as to the complete representation of the article's main content (p<0.01).
**Conclusion:** Medical students, and radiologists alike, were significantly satisfied with IRMA, declaring that they will use it for their research and practice. However, radiologists were more selective and decisive in stating that the IRMA service is accurate and useful.
**Limitations:** Small groups of participants.
**Ethics Committee Approval:** Not required
**Funding for this study:** Algomost LLC
**Author Disclosures:**
Valeria Guryanova: Employee: Algomost
Valentin Sinitsyn: Nothing to disclose
Olga Valerievna Leshchinskaya: Nothing to disclose
Leonid Kokov: Nothing to disclose
Moshe Ayal: Consultant: Algomost
Gabriel Bartal: Consultant: Algomost

**RPS 1005-9**
**Effective integration of artificial intelligence (AI) services into PACS: radiologist requirements**
*K. M. Arzamasov*, N. Ledikhova, E. Turavilova, V. Gombolevskiy, A. Vladzymyrskyy, A. E. Andreychenko, S. Morozov; Moscow/RU
*(k.arzamasov@npcmr.ru)*

**Purpose:** Aim of our study was to determine key requirements for an effective practical integration into PACS of radiological services based on artificial intelligence (AI) and computer vision.
**Methods or Background:** Summarizing the experience of using AI services, we had identified the necessity to standardize the requirements for the operation of AI services.
**Results or Findings:** A radiologist, opening an examination in PACS, faces: 1) original (native) series; 2) series from AI (may include: generalizing image; tabular view of results; a series (copy of the original) with marks from the AI); 3) textual description in DICOM SR; 4) AI-feedback module. Each additional AI-formed series should contain: the name of the AI service, probability of pathology and contrast marking of pathological findings. Additional information is optional. If the AI service didn't detect pathological changes, additional series should contain an image with a corresponding message. The number of slices in the additional series isn't less than in the original series in case of a pathology according to AI. The textual description should contain a short user manual containing information about the pathology detected by the service and a conclusion. The conclusion should be presented in an understandable form, if possible, it should contain a clinical classification of the findings. Additional details on the AI findings are of interest, but in routine work it isn't essential. Any additional information is superfluous.
**Conclusion:** The introduced practice is aimed to create possibilities for a radiologist for a comfortable operation within PACS both with and without AI results, seamlessly weaving the results of AI into the workflow.
**Limitations:** No limitation.
**Ethics Committee Approval:** This study is based upon the results of the registered study (https://clinicaltrials.gov/ct2/show/NCT04489992).
**Funding for this study:** Internal.
**Author Disclosures:**
Sergey Morozov: Nothing to disclose
Anna Evgenevna Andreychenko: Nothing to disclose
Natalia Ledikhova: Nothing to disclose
Anton Vladzymyrskyy: Nothing to disclose
Elena Turavilova: Nothing to disclose
Kirill Mihajlovich Arzamasov: Nothing to disclose
Victor Gombolevskiy: Nothing to disclose

**RPS 1005-10**
**Optimal timing for collateral assessment using CTP data**
*J. Su*, L. Wolff, P. J. van Doormaal, D. Dippel, W. J. Niessen, A. Van Der Lugt, T. van Walsum; Rotterdam/NL
*(sujiahang@gmail.com)*

**Purpose:** To investigate whether there are optimal time points in CTP for collateral status assessment.
**Methods or Background:** Patients with ischemic stroke due to large vessel occlusion underwent baseline non-contrast CT, multiphase CT angiography and CT perfusion. Baseline clinical variables were National Institutes of Health Stroke Scale (NIHSS) and Alberta Stoke Program Early CT Score (ASPECTS). For each time point in a CTP image sequence, the vessel volume of the occluded side and its contralateral side and the arterial and venous Hounsfield Units (annotated in a spherical ROI of ICA top and confluence sinuses) were obtained, yielding five curves over time. The Collateral Score(CS) was computed at the peak amplitude of each of the curves and the first acquisition of the multiphase CTA(mCTA) images. Its association with baseline NIHSS and ASPECTS was assessed with regression analysis.
**Results or Findings:** In total 58 subjects (median age, 74 years, interquartile range, 61-83 years; 33 male) were included. CS at peak arterial enhancement was associated with NIHSS and had moderate correlation with ASPECTS (NIHSS: beta =-0.586, CI, [-0.946, -0.225]; ASPECT: beta =0.452, CI, [0.079, 0.825]). The other candidate collateral measurements were less associated with NIHSS and ASPECTS. The association of the CS at the peak arterial time enhancement were comparable the CS in the first acquisition of the mCTA.
**Conclusion:** CS at peak of arterial enhancement curve has a better association with NIHSS baseline and ASPECTS compared to the other candidate collateral measurements, and has a similar association as the mCTA collateral score. This suggests that the optimal time point for collateral assessment is at the peak of arterial enhancement.
**Limitations:** The single center image data and relatively small data size.
**Ethics Committee Approval:** n/a
**Funding for this study:** No funding was received for this work.
**Author Disclosures:**
Theo van Walsum: Nothing to disclose
Aad Van Der Lugt: Nothing to disclose
Pieter Jan van Doormaal: Nothing to disclose
Diederik Dippel: Nothing to disclose
Jiahang Su: Nothing to disclose
Wiro J. Niessen: Nothing to disclose
Lennard Wolff: Nothing to disclose

### Research Presentation Session: Hybrid, Molecular and Translational Imaging

### RPS 206

### Hybrid, molecular, and translational imaging developments

**RPS 206-1**
**AI-enhanced breast cancer molecular subtyping using simultaneous 18F-FDG PET/MRI**
*V. Romeo*[1], P. Kapetas[2], P. Clauser[2], P. A. Baltzer[2], S. Rasul[2], P. Gibbs[3], R. A. Woitek[4], K. Pinker-Domenig[3], T. H. Helbich[2]; [1]Naples/IT, [2]Vienna/AT, [3]New York, NY/US, [4]Cambridge/UK
*(valeria.romeo@unina.it)*

**Purpose:** To investigate whether an AI-based radiomics model applied to 18F-FDG PET/MRI is effective in molecular subtyping of breast cancer (BC) and specifically in discriminating triple negative (TN) from other molecular subtypes of BC.
**Methods or Background:** In this IRB-approved, prospective, single-institution study, 86 patients with 98 BC lesions (Luminal A=10, Luminal B=51, HER2+=12, TN=25) were included and underwent simultaneous 18F-FDG

PET/MRI of the breast. 3D segmentation of BC lesion was performed on T2w, DCE, DWI and PET images. Quantitative diffusion (tumor ADCmean and contralateral breast parenchyma), perfusion (tumor Mean Transit Time, Plasma Flow, Volume Distribution) and metabolic (tumor SUVmax, min, mean, SUVmean of ipsi and contralateral breast parenchyma) parameters were calculated and radiomics features (first, second, higher order) extracted. Quantitative parameters and radiomics features were selected using the LASSO regression and used by a fine gaussian support vector machine (SVM) classifier with a 5-fold cross validation for identification of TNBC lesions. Different radiomics models were built based on different combinations of quantitative parameters and/or radiomic features to obtain the highest accuracy.
**Results or Findings:** Eight radiomics models were built. The best performance (AUROC 0.887, accuracy 82.8%, sensitivity 79.7%, specificity 86%, PPV 85.3%, NPV 80.8%) was found for the model combining first order, neighbourhood gray level dependence matrix and size zone matrix-based radiomics features extracted from ADC and PET images.
**Conclusion:** An AI-based radiomics model applied to 18F-FDG PET/MRI is able to non-invasively discriminate TNBC lesions from other BC molecular subtypes with high accuracy. In a future perspective, a “virtual biopsy” might be performed using radiomics signatures.
**Limitations:** Small sample size
Lack of external validation
**Ethics Committee Approval:** IRB-Approved
**Funding for this study:** EU Grant
**Author Disclosures:**
Pascal A.T. Baltzer: Nothing to disclose
Katja Pinker-Domenig: Nothing to disclose
Valeria Romeo: Research/Grant Support: Supported by the ESOR "Bracco Fellowship"
Thomas H. Helbich: Grant Recipient: EU Grant
Ramona Anna Woitek: Nothing to disclose
Sazan Rasul: Nothing to disclose
Panagiotis Kapetas: Nothing to disclose
Peter Gibbs: Nothing to disclose
Paola Clauser: Speaker: Siemens

### RPS 206-2

**Potential role of deep learning for detection and localization of hyperfunctioning parathyroid glands in primary hyperparathyroidism on [18F]fluorocholine PET/CT**

*L. Jarabek*, J. Jamsek, A. Cuderman, S. Rep, M. Hočevar, T. Kocjan, K. Zaletel, M. Jensterle Sever, L. Ležaić; Ljubljana/SI
*(leon.jarabek@gmail.com)*

**Purpose:** [18F]fluorocholine PET/CT (FCH-PET) has excellent diagnostic performance in primary hyperparathyroidism, with experienced human readers achieving high sensitivity (91.7%) and specificity (99.7%). Interpretation of FCH-PET using deep learning in the setting of primary hyperparathyroidism has not yet been investigated. The study aimed to train and test a baseline and novel deep learning classification model for detection (CPr) and localization (CLoc) of hyperfunctioning parathyroid tissue from FCH-PET and to compare it to human diagnostic performance.
**Methods or Background:** Ninety-eight FCH-PET studies of seventy-nine patients with primary hyperparathyroidism and nineteen control patients were obtained from our clinical trial (NCT03203668). We evaluated two deep learning models: a baseline ResNet10 classifier and a novel mPETResnet10 architecture. mPETResnet10 used UNet to mask physiologically high PET signal in order to improve the performance of Resnet10. The performance of both models was evaluated with 95% CI and compared to human performance from NCT03203668 using McNemar’s test.
**Results or Findings:** Both models showed comparable performance for CPr ($p > 0.05$), with mPETResnet10 achieving 0.90[0.82–0.95] sensitivity and 0.62[0.38–0.82] specificity. mPETResnet10 performed statistically better ($p < 0.05$) for CLoc, with sensitivity of 0.81[0.72–0.89] and specificity of 0.80[0.75–0.85] whereas Resnet10 achieved 0.74[0.64–0.82] sensitivity and 0.76[0.70–0.80] specificity. Based on qualitative observations, mPETResnet10 masked the physiologic signal of salivary glands. Human performance for both tasks was significantly higher ($p < 0.001$).
**Conclusion:** We proved that deep learning could be used to identify hyperfunctioning parathyroid tissue on FCH-PET. Our improved mPETResnet10 classifier performed better than the standard Resnet10 classifier. However, performance of experienced human readers was still better than our deep learning models.
**Limitations:** Given our small dataset, the results are promising and diagnostic performance might be improved by including more cases.
**Ethics Committee Approval:** ClinicalTrials.org: NCT03203668
kme-nmec.si/: 77/11/12
**Funding for this study:** No funding was received for this study.
**Author Disclosures:**
Mojca Jensterle Sever: Nothing to disclose
Leon Jarabek: Nothing to disclose
Tomaž Kocjan: Nothing to disclose
Luka Ležaić: Nothing to disclose
Marko Hočevar: Nothing to disclose
Katja Zaletel: Nothing to disclose
Anka Cuderman: Nothing to disclose
Jan Jamsek: Nothing to disclose
Sebastijan Rep: Nothing to disclose

### RPS 206-3

**Comparison of three different dietary modifications on myocardial 18F-FDG suppression in PET/CT evaluation of patients for cardiac sarcoidosis**

*C. Ozutemiz*, Y. K. Köksel, N. Rubin, J. Froelich, D. Steinberger; Minneapolis, MN/US

**Purpose:** To compare three different dietary preparations in achievement of the best myocardial suppression (MS) for diagnosis of cardiac sarcoidosis (CS) with 18F-FDG-PET/CT.
**Methods or Background:** All 18F-FDG-PET/CT exams for CS were retrospectively reviewed. Three different diets were applied from 03/2014-12/2019. Diet-A)24h ketogenic diet with overnight fasting (n=94); Diet-B)18h-fasting (n=44); Diet-C)72h ketogenic diet with 3day/12h overnight fasting (n=98). The results of initial reports were recorded, and a separate radiologist retrospectively re-evaluated each case regarding CS (Negative, Positive, Indeterminant) and MS (Complete, Failed, Partial). Interobserver agreement was studied. MaxSUV was measured from blood-pool, liver, the most suppressed normal myocardium. Three dietary groups were compared.
**Results or Findings:** Significantly lower myocardial FDG uptake, higher blood-pool/myocardium and liver/myocardium ratios were found with Diet-C, indicating better MS ($P<0.001$). The observer found statistically superior MS with Diet-C; complete-MS:96.9% (Diet-A/Diet-B:68.1%/52.3%), failed-MS:0% (Diet-A/Diet-B:23.4%/25%), partial-MS: 3.1% (Diet-A/Diet-B:8.5%/22.7%) ($P<0.001$). Borderline statistical difference was found regarding MS per the radiology report ($P=0.052$). Regarding MS, mild to moderate agreement was found between the report and the observer ($\kappa=0.41$). Diet-C has significantly more “negative”, less “positive” and less “indeterminant” active CS diagnoses compared with other diets. The rates of negatives/indeterminants/positives according to the report and observer in Diet-A, Diet-B, and Diet-C are as follows respectively; Report A: 52.1%/4.3%/43.6%, B:34.1%/13.6%/52.3%, C:83.7%/1.0%/15.3%. Observer A: 52.1%/24.5%/23.4%, B: 27.3%/40.9%/31.8%, C: 82.7%/2.0%/15.3%. High agreement was present with the observer and the report ($\kappa=0.88$)
**Conclusion:** 72h ketogenic diet with overnight fasting is superior to other diets, providing excellent MS and allowing decreased number of indeterminant and potentially “false positive” results.
**Limitations:** Retrospective
no standard calculated diet was given
no confirmation by gold standard, because there is no gold standard.
**Ethics Committee Approval:** This retrospective study was approved by the institutional review board (STUDY00005814).
**Funding for this study:** Departmental funding for statistical analysis
**Author Disclosures:**
Nathan Rubin: Nothing to disclose
Can Ozutemiz: Nothing to disclose
Yasemin Kocabas Köksel: Nothing to disclose
Daniel Steinberger: Nothing to disclose
Jerry Walter Froelich: Grant Recipient: No activities related to the present article. Author receives scientific grant support from Siemens Healthineers for a project unrelated to this study.

### RPS 206-4

**Longitudinal MRI-analysis of body composition in melanoma: from mouse model to immune checkpoint inhibitor-treated metastatic patients**

*W. Thaiss*[1], S. Gatidis[2], J. Machann[2], K. Nikolaou[2], B. Pichler[2], M. Kneilling[2]; [1]Ulm/DE, [2]Tübingen/DE

**Purpose:** Cancer cachexia (CC) is associated with cancer progression, thus early identification would be clinically beneficial. The aim was to establish an automated MRI-based segmentation workflow defining visceral and subcutaneous adipose tissue (VAT, SCAT) and lean tissue water (LTW) in mice and human melanoma patients for therapy assessment.
**Methods or Background:** For in vivo monitoring of CC B16-melanoma bearing and healthy mice underwent longitudinal three-point DIXON MRI (days 3, 12, 17 after subcutaneous tumor inoculation). In a prospective clinical study, 18 metastatic melanoma patients underwent MRI before, 2 and 12 weeks after onset of checkpoint inhibitor therapy (CIT; n=16). We employed an in-house

MATLAB script for automated whole-body segmentation for detection of VAT, SCAT and LTW.
**Results or Findings:** B16 mice developed a reduced VAT volume compared to baseline (B16 -249.8 µl, -25%; controls +85.3 µl, +10%, p=0.003) and to healthy controls. LTW was increased in controls compared to melanoma mice. Five melanoma patients responded to CIT, 7 progressed, and 6 displayed a mixed response. Responding patients exhibited a very limited variability in VAT and SCAT in contrast to others. LTW was decreased in CIT responding patients (−3.02%±2.67%; p=0.0034) but i□□re□□ed i□ p□tie□t□with pro□re□□ive disease (+1.97%±2.19%) and mixed response (+4.59%±3.71%).
**Conclusion:** MRI-based segmentation of fat and water contents adds additional information for monitoring the development of cancer cachexia in mice and metastatic melanoma patients under treatment.
**Limitations:** Larger cohort studies are needed to take individual patient parameters into account.
**Ethics Committee Approval:** Animal experiments were performed according to the German Animal Protection Law with permission from the local authorities. The prospective human study was approved by the local ethics committee.
**Funding for this study:** Deutsche Forschungsgemeinschaft (DFG, German Research Foundation), Werner Siemens-Foundation (Zug, Switzerland), intramural funding (Medical Faculty of the Eberhard Karls Universität Tübingen).
**Author Disclosures:**
Konstantin Nikolaou: Nothing to disclose
Manfred Kneilling: Nothing to disclose
Jürgen Machann: Nothing to disclose
Sergios Gatidis: nothing to disclose
Bernd Pichler: Nothing to disclose
Wolfgang Thaiss: Nothing to disclose

### RPS 206-5

**Prospective comparison of CT and 18F-FDG PET/MRI in N and M staging of primary breast cancer patients: initial results**
*N-M. Bruckmann*; Düsseldorf/DE

**Purpose:** To compare the diagnostic accuracy of contrast enhanced (ce) thoraco-abdominal computed tomography (CT) and whole-body 18F-FDG PET/MRI in N and M staging in newly diagnosed, histopathological proven breast cancer.
**Methods or Background:** A total of 80 consecutive women with newly diagnosed and histopathologically confirmed breast cancer were enrolled in this prospective study. All patients underwent a thoraco-abdominal ceCT and a whole-body 18F-FDG PET/MRI. All datasets were evaluated separately by two radiologists blinded to clinical/follow-up data regarding suspect lesion count, localization, categorization and diagnostic confidence. Histopathological results as well as follow-up imaging served as reference standard in all patients. Differences in staging accuracy were assessed using Mc Nemar´s chi² test.
**Results or Findings:** CT rated the N stage correctly in 64 of 80 (80%, 95 % CI:70.0-87.3) patients with a sensitivity of 61.5%, a specificity of 97.6%, a PPV of 96%, and a NPV of 72.7%. Compared to this, 18F-FDG PET/MRI determined the N stage correctly in 71 of 80 (88.75%, CI:80.0-94.0) patients with a sensitivity of 82.1%, a specificity of 95.1%, a PPV of 94.1%, and a NPV of 84.8%. Differences in sensitivities were statistically significant (20.6%, CI:-0.02-40.9; p=0.008). Distant metastases were present in 7/80 patients (8.75%). 18F-FDG PET/MRI detected all of the histopathological proven metastases without any false-positive findings, while 3 patients with bone metastases were missed in CT (sensitivity 57.1%, specificity 95.9%). Additionally, CT presented false-positive findings in 3 patients.
**Conclusion:** 18F-FDG PET/MRI has a high diagnostic potential and outperforms CT in assessing the N and M stage in patients with primary breast cancer.
**Limitations:** Application of a modified reference standard, based on follow-up imaging for lesions without a histological sampling.
**Ethics Committee Approval:** This study is approved by the local ethics committee.
**Funding for this study:** Funded by Deutsche Forschungsgemeinschaft (DFG).
**Author Disclosures:**
Nils-Martin Bruckmann: Nothing to disclose

### RPS 206-7

**Is there a benefit of a late-phase PET as part of 68Ga-PSMA-11 PET/CT in patients with biochemical recurrence of prostate cancer?**
*C. C. Loberg*, J. Kirchner, N-M. Bruckmann, E. Mamlins, C. Arsov, J. Morawitz, L. Schimmöller, G. Antoch, L. M. Sawicki; Düsseldorf/DE
*(christina.loberg@med.uni-duesseldorf.de)*

**Purpose:** To assess the diagnostic value of late-phase PET scan as part of 68Ga-PSMA-11 PET/CT in patients with biochemical recurrence of prostate cancer.
**Methods or Background:** This was a retrospective trial including 33 patients (mean age 74 ± 8 y) with newly documented biochemical recurrence of prostate cancer following radical prostatectomy (PSA 6.9±12.5 ng/ml; 0.27–50.0 ng/ml). Patients underwent early-phase PET PET/CT and a late-phase PET scan performed 54+/- 12.7 min after injection of 68Ga-PSMA-11. Late-phase PET-scan was conducted after emptying urinary bladder. Images were independently evaluated by two radiologists and two nuclear medicine specialists, regarding lesion count, localization, and SUVmax followed by a consensus reading. Reference standard for lesion nature was based on histopathology and follow-up imaging.
**Results or Findings:** Whole-body early-phase PSMA PET/CT detected 93 cancer lesions in 33 patients. There were 57 nodal, 28 bone, and three lung metastases as well as seven local recurrences. Late phase PET/ CT scans detected two additional lesions in two patients, according to local recurrence. Between early- and late-phase PET, corresponding lymph node metastases showed a significant increase of SUVmax from 14.47±11.63 to 21.5±17.6 (p=0.00007). Benign lymph nodes measured in the respective regions showed a significantly lower increase of SUVmax of 1.43±0.5 to 1.65±0.47 (p=0.0014). On late phase PET/CT local recurrence and bone metastases had a SUVmax 3.83 and 1.1 times higher in early phase PET/CT.
**Conclusion:** Late-phase PETin addition to early-phase 68Ga-PSMA-11 PET/CT is useful to detect local recurrences of prostate cancer and to distinguish between malignant and benign nodes. Bone metastases and local recurrences also demonstrate a strong metabolic increase over time.
**Limitations:** It was a monocentric study with a limited amount of patients.
**Ethics Committee Approval:** The study was approved by local ethic comittee (2020-871)
**Funding for this study:** There was no funding recquired
**Author Disclosures:**
Christina Christina Loberg: Nothing to disclose
Christian Arsov: Nothing to disclose
Lino M Sawicki: Nothing to disclose
Eduard Mamlins: Nothing to disclose
Lars Schimmöller: Nothing to disclose
Julian Kirchner: Nothing to disclose
Gerald Antoch: Nothing to disclose
Janna Morawitz: Nothing to disclose
Nils-Martin Bruckmann: Nothing to disclose

### RPS 206-8

**Free-breathing 3D Stack of Stars GRE (StarVIBE) sequence for detecting pulmonary nodules in 18F-FDG PET/MRI**
*N-M. Bruckmann*; Düsseldorf/DE

**Purpose:** The free-breathing T1-weighted 3D Stack of Stars GRE (StarVIBE) MR sequence potentially reduces artifacts in chest MRI. The purpose of this study was to evaluate StarVIBE for the detection of pulmonary nodules in 18F-FDG PET/MRI.
**Methods or Background:** In this prospective study, 88 consecutive women with newly diagnosed breast cancer underwent both contrast-enhanced whole-body 18F-FDG PET/MRI and computed tomography. Patients' chests were examined on CT as well as on StarVIBE and conventional T1-weighted VIBE and T2-weighted HASTE MR sequences, with CT serving as the reference standard. Presence, size, and location of all detectable lung nodules were assessed. A Wilcoxon test was applied and Pearson's correlation coefficients were calculated.
**Results or Findings:** Out of 65 lung nodules detected in 36 patients with CT, the StarVIBE sequence was able to detect 31 (47.7%), the VIBE sequence 26 (40%) and the HASTE sequence 11 (16.8%), respectively. Overall, CT showed a significantly higher detectability than all MRI sequences combined (65 vs. 36, difference 44.6%, p<0.001). The VIBE sequence showed a significantly better detection rate than the HASTE (23.1%, p=0.001). Detection rates between StarVIBE and VIBE did not significantly differ (7.7%, p=0.27), but the StarVIBE showed a significant advantage detecting centrally located nodules (66.7% vs. 16.7%, p=0.031). There was a strong correlation in nodule size between CT and MRI sequences (HASTE: r=0.80; VIBE: r=0.77; StarVIBE: r=0.78, p<0.01).
**Conclusion:** Detectability of centrally located nodules is slightly better with StarVIBE than with VIBE. Nevertheless, all MRI analyses demonstrated considerably lower detection rates for small lung nodules, when compared to CT.

**Limitations:** Women with newly diagnosed breast cancer are generally younger and physically better constituted than most other people suffering from cancer.
**Ethics Committee Approval:** This study was approved by the local ethics committee.
**Funding for this study:** Funded by Deutsche Forschungsgemeinschaft (DFG).
**Author Disclosures:**
Nils-Martin Bruckmann: Nothing to disclose

### Research Presentation Session: Genitourinary

### RPS 307

### Genitourinary cancers

### RPS 307-1

**Quantitative diffusion-weighted MR imaging in preoperative evaluation of endometrial cancer: the correlation between apparent diffusion coefficient (ADC) and definitive histological grade**

*E. Scibilia*, P. V. Foti, F. Costa, R. Farina, S. Palmucci, R. Caltabiano, G. Broggi, A. Cianci, A. Basile; Catania/IT
*(edoardoscibilia@gmail.com)*

**Purpose:** According to the European Society for Medical Oncology guidelines, lymphadenectomy in endometrial cancer (EC) is performed on the basis of tumor stage and grade. Magnetic resonance imaging (MRI) can preoperatively assess the depth of myometrial invasion (tumor stage). Preoperative endometrial sampling is used to obtain the histopathological tumor grade, nevertheless a discrepancy between preoperative and definitive histopathological diagnosis occurs in up to 29% of cases. The aim of our study was to retrospectively investigate the value of quantitative diffusion weighted (DW) MRI with apparent diffusion coefficient (ADC) in predicting tumour grade of EC.
**Methods or Background:** 40 patients with EC were enrolled in the study. All patients underwent preoperative MRI with conventional and DWI sequences. All patients underwent surgery. For quantitative analysis, the ADC values of EC were measured and compared with definitive (surgical) histopathological diagnosis considered as the reference standard. The diagnostic performance of ADC for discriminating grade 3 from grade 1-2 tumours was analysed using a receiver-operating characteristic (ROC) curve.
**Results or Findings:** Mean ADC values of EC were significantly different among grade 1, grade 2 and grade 3 groups (Grade 1: 909.4±135.7 x 10-6 mm2 /s; Grade 2: 806.7±89.7 x 10-6 mm2 /s; Grade 3: 657.7±124.2 x 10-6 mm2 /s; p<0.001). When an ADC <753 x 10-6 mm2 /s was used as the cut-off value for distinguishing between the grade 3 and grade 1-2 groups [area under the ROC curve, 0.86], a sensitivity of 92.9% and a specificity of 83.3% were obtained.
**Conclusion:** DWI-ADC value was feasible for discriminating grade 3 from grade 1-2 tumours in EC and may be useful to guide lymphadenectomy, thus avoiding overtreatment in low-risk patients.
**Limitations:** n/a
**Ethics Committee Approval:** n/a
**Funding for this study:** n/a
**Author Disclosures:**
Renato Farina: Nothing to disclose
Antonio Cianci: Nothing to disclose
Pietro Valerio Foti: Nothing to disclose
Rosario Caltabiano: Nothing to disclose
Federico Costa: Nothing to disclose
Antonio Basile: Nothing to disclose
Edoardo Scibilia: Nothing to disclose
Giuseppe Broggi: Nothing to disclose
Stefano Palmucci: Nothing to disclose

### RPS 307-3

**Synthetic MRI for quantitative measurements of endometrial cancer and correlation with depth of myometrial invasion**

*Y. Wang*, P. Cao, E. Y. P. Lee; Hong Kong/CN

**Purpose:** The depth of myometrial invasion (MI) has prognostic value in endometrial cancer (EC). Synthetic MRI is an emerging technique that enables accurate quantitative measurements of T1/T2 maps with fast speed. The relationships between T1/T2 values and the depth of MI in EC have not been explored. The aim of this study was to measure the T1/T2 values of EC, and their relationships with MI.
**Methods or Background:** Synthetic MRI of 49 patients with EC were acquired on 1.5 T MRI scanner (Signa Explorer, GE Healthcare, Waukesha, USA). A region of interest (ROI) was delineated on the axial slice that EC appeared largest using the MAGiC software to derive the mean T1/T2 values of EC. Patients were dichotomized into Group 1 (no MI, 8/49), Group 2 (MI<50%, 28/49) and Group 3 (MI≥50%, 13/49). Kruskal Wallis test was used to test for differences among groups with subsequent pairwise Mann Whitney U (MWU) test to compare the differences in T1/T2 values between groups.
**Results or Findings:** The measured T1/T2 values of EC were 1347.0±275.8 msec and 108.1±14.7 msec, respectively. The T1/T2 values were different among the 3 groups (p = 0.037 for T1 and p = 0.030 for T2). Decreasing tendencies in T1/T2 values were observed from Group 1 to Group 3. However, pairwise MWU comparisons did not reveal significant differences between groups, except for T2 values between Group 2 and 3 (p = 0.008) with T2 value in Group 2 larger than Group 3.
**Conclusion:** The T2 value derived from synthetic MRI of EC with less than 50% MI was significantly larger than EC with more than 50% MI.
**Limitations:** Small sample size.
**Ethics Committee Approval:** Yes.
**Funding for this study:** No.
**Author Disclosures:**
Elaine Yuen Phin Lee: Nothing to disclose
Pemg Cao: Nothing to disclose
Yiang Wang: Nothing to disclose

### RPS 307-4

**Comparison of abdominal attenuation values on virtual unenhanced image obtained with renal mass to hematuria dual-energy computed tomography protocol**

*J. Cao*, S. Lennartz, A. Parakh, N. Pisuchpen, A. Kambadakone; Boston, MA/US
*(JCAO11@MGH.HARVARD.EDU)*

**Purpose:** To compare virtual unenhanced (VUE) attenuation between patients who underwent dual-energy CT with renal mass protocol(DECTRM) and patients with hematuria who received split-bolus nephrographic phase urography(DECTHT).
**Methods or Background:** A total of 105 consecutive patients who underwent DECT were included: 55 patients received DECTRM for suspected renal mass and 50 patients received DECTHT for assessing hematuria on a dual-layer detector DECT. Study inclusion period was July 2018 to November 2019. All scans were biphasic examinations comprising an unenhanced nephrographic phase acquisition. DECTRM was performed with single bolus and DECTHT with a split-bolus technique. VUE and iodine-overlay images were reconstructed from 120-kVp contrast-enhanced(CE) acquisitions of the nephrographic phase. A radiologist quantified attenuation of liver parenchyma, main portal vein, aorta, spleen, renal cortex, and pelvis on TUE, VUE, CE, and Iodine no water images. Agreement between TUE and VUE images was determined for both protocols and each anatomical region.
**Results or Findings:** The attenuation of renal cortex and pelvis were significantly different between the two protocols. VUE attenuation of the renal cortex was significantly higher in the renal mass protocol compared to the hematuria protocol (left renal cortex 61.05 vs 44.14 HU, right renal cortex 60.33 vs 44.94 HU; both p<0.05). Conversely, VUE attenuation of the renal pelvis was significantly higher in the hematuria protocol compared to the renal mass protocol (left renal pelvis 78.42 vs 29.89 HU, right renal pelvis 79.29 vs 29.10 HU; both p<0.001).
**Conclusion:** There was significant variability between the renal mass protocol and hematuria protocol regarding renal VUE attenuation, which impacts the application of established attenuation thresholds for lesion characterization.
**Limitations:** Retrospective study with a limited sample size.
**Ethics Committee Approval:** HIPAA-compliant, IRB approved.
**Funding for this study:** Research Grant, Philips
**Author Disclosures:**
Simon Lennartz: Nothing to disclose
Avinash Kambadakone: Nothing to disclose
Jinjin Cao: Nothing to disclose
Nisanard Pisuchpen: Nothing to disclose
Anushri Parakh: Nothing to disclose

### RPS 307-6

**Diagnostic accuracy of the ADNEX MRI scoring system based on a simplified MRI protocol: preliminary results of a single tertiary center retrospective study**

*S. Aslan*, S. Alanya Tosun; Giresun/TR
*(serdaraslan28@hotmail.com)*

**Purpose:** To evaluate the ADNEX MR scoring system efficacy based on a simplified MRI protocol in adnexal masses.
**Methods or Background:** In this retrospective study, 179 masses belonging to 116 cases who underwent Pelvic MRI with the diagnosis of adnexal mass between November 2019 and November 2020 were evaluated. An experienced radiologist with board certification calculated the ADNEX MR scores of adnexal masses using an MRI protocol with a simplified dynamic study. The masses were scored from 1 to 5. Sensitivity, specificity, PPV, NPV, accuracy and the area under the receiver operating characteristic (ROC) curve were calculated (cutoff for malignancy, score ≥ 4). The reference standard was histopathologic diagnosis or imaging findings during >12 months of follow-up.
**Results or Findings:** Of 179 lesions, 32 (17.8%) were malignant. The malignancy rates of masses with scores of 2, 3, 4 and 5 were 0% (0/81), 12.5% (5/40), 37.5% (18/48) and 90% (9/10) respectively. The ADNEX MR scoring system, using a simplified MRI protocol, showed 87,5% (95% CI, 61,6% - 98,4%) sensitivity, 96,2% (95% CI, 89,4% - 99,2%) specificity, 82,3% (95% CI, 60,2% - 93,5%) PPV, 97,4% (95% CI, 91,3% - 99,3%) NPV, and 94,8% (95% CI, 88,2% - 98,2%) accuracy in the malignancy prediction. The area under the ROC curves for the differentiation of benign and malignant masses were 0,969.
**Conclusion:** The ADNEX MR scoring system, even based on a simplified MRI protocol, performed well in the prediction of malignant adnexal masses. This scoring system may enable the standardization of MRI reporting on adnexal masses, thereby improving communication between radiologists and gynecologists.
**Limitations:** A retrospective, single-center study and low number of malignant lesions.
**Ethics Committee Approval:** This retrospective study was approved by our institutional ethics committee.
**Funding for this study:** No funding was received for this study.
**Author Disclosures:**
Serdar Aslan: nothing to disclose
Sebnem Alanya Tosun: Nothing to disclose

### RPS 307-7

**Contrast-enhanced ultrasound (CEUS) imaging for active surveillance of small renal masses**

*I. Baldi*, N. Morelli, S. Agostini, R. Campi, S. Serni, E. Bertelli, V. Miele; Florence/IT
*(irene.baldi@gmail.com)*

**Purpose:** To assess the safety and efficacy of contrast-enhanced ultrasound (CEUS) imaging for monitoring small (<4 cm) renal masses (SRMs) in patients undergoing active surveillance (AS) at a high-volume kidney cancer Centre.
**Methods or Background:** We selected all consecutive patients with SRMs who underwent AS for at least 6 months at our Institution between January 2014 and December 2018. CEUS was performed by two experienced genito-urinary radiologists at established time points. We identified four enhancement patterns (EP) of solid SRMs and defined as “growing SRMs” those whose size growth was > 5 mm over the AS period.
**Results or Findings:** Of 1049 patients with SRMs treated during the study period, 158 (15.1%) underwent AS. The median follow-up was 25 months (IQR 13-39). No patient died due to renal cell carcinoma (RCC) during the follow-up. Of patients undergoing AS, 43 (27.2%) underwent delayed intervention (DI) after a median follow-up of 10 months (IQR 8-19). Overall, there was a large variability in the pattern of growth of SRMs. The median SRMs size did not significantly change over the AS period. Yet, there was a significantly different EP between growing vs not growing SRMs in both patient cohorts. Notably, CEUS provided a reliable assessment of SRMs beyond their size: in particular, EP calculated during the real time CEUS examination was associated with the “growing potential” of SRMs. In addition, CEUS was especially appreciated by patients and reduced the inter-observer variability.
**Conclusion:** CEUS is a safe, reliable and cost-effective strategy for strict monitoring of SRMs undergoing AS. Furthermore the EP could help clinicians in the management of SRMs.
**Limitations:** Retrospective study. We could not evaluate the specific triggers for DI. Relatively small sample size and mid-term follow-up.
**Ethics Committee Approval:** Approved by Ethical Committee
**Funding for this study:** Not applicable
**Author Disclosures:**
Noemi Morelli: Nothing to disclose
Vittorio Miele: Nothing to disclose
Riccardo Campi: Nothing to disclose
Elena Bertelli: Nothing to disclose
Simone Agostini: Nothing to disclose
Irene Baldi: Nothing to disclose
Sergio Serni: Nothing to disclose

### RPS 307-8

**Ex vivo 1H high resolution magic angle spinning (HRMAS) NMR spectroscopy for early prostate cancer detection**

*A. Steiner*[1], S. A. Schmidt[1], C-S. Fellmann[1], M. J. Beer[1], L. L. Cheng[2]; [1]Ulm/DE, [2]Boston, MA/US
*(annabelsteiner@gmx.de)*

**Purpose:** to evaluate the diagnostic value of ex vivo 1H HRMAS NMR spectroscopy for early prostate cancer detection with focus on the predictive potential of metabolomic alterations in biopsy samples for a cancer diagnosis in the following years.
**Methods or Background:** 351 prostate biopsy patients were prospectively included in a primary study (04/2006-10/2018). Samples from each patient were analyzed with NMR spectroscopy (Bruker 14.1 Tesla, Billerica, MA, USA) and histopathologically evaluated. With regard to our research question we performed a subgroup analysis of all 351 patients and built three homogenous groups à 16 patients which we compared in our subproject: Group 1 and 2 represented patients whose NMR scanned biopsy sample was histobenign, but patients of group 1 were diagnosed with cancer in the following years, while patients of group 2 stayed histobenign until the end of the study period. Group 3 included cancer patients.
**Results or Findings:** Metabolite concentrations (e.g. polyamines, $p=0.0052$) and metabolomic profiles could separate histobenign and malignant prostate tissue. Metabolite signals (e.g. glycerophosphoethanolamine, $p=0.0027$) could also differentiate between histobenign samples of patients diagnosed with cancer in the following years and of those who stayed histobenign. Metabolite concentrations correlated with volume percentage of benign epithelia (e.g. citrate, $p=0.0008$, $r2=0.2181$) and PSA density (Valin in group 3, $p=0.0047$, $r2=0.4465$). Metabolite concentrations could also differentiate between Gleason Score 3+3=6 and 3+4=7 (e.g. polyunsaturated fatty acids n-6, $p=0.0206$).
**Conclusion:** Metabolomic information seems to hold a high potential in early prostate cancer detection. Moreover, the predictive power of metabolomic profiles could improve active surveillance strategies on the basis of single patients, thus paving the way to a high level of personalized medicine for prostate cancer.
**Limitations:** n/a
**Ethics Committee Approval:** An independent ethics committee reviewed and approved the study.
**Funding for this study:** n/a
**Author Disclosures:**
Cara-Sophie Fellmann: Nothing to disclose
Annabel Steiner: Nothing to disclose
Stefan Andreas Schmidt: Nothing to disclose
Leo L. Cheng: Nothing to disclose
Meinrad Johannes Beer: Nothing to disclose

### RPS 307-9

**Non-prostate cancer tumours: incidence on 18F DCFPyL PSMA PET/CT and uptake characteristics**

*E. Perry*[1], S. Sharma[2], T. Sutherland[2]; [1]Christchurch/NZ, [2]Melbourne/AU
*(elisa.perry@pacificradiology.com)*

**Purpose:** 18F DCFPyL PSMA PET/CT has been widely adopted in management algorithms for patients with prostate cancer (PCa). Although initially thought to be specific for PCa, multiple cases reports exist describing avidity in a variety of other malignancies. To detect the incidence and types of non-prostate cancer tumours detected on 18F DCFPyL PSMA PET/CT and describe the SUV and imaging uptake characteristics of these. Determine the influence of imaging indication on the incidence of these malignancies, with evaluation of three main cohorts referred for PSMA PET/CT; primary staging; biochemical progression; and primary diagnosis.
**Methods or Background:** The 18F DCFPyL databases of our institutions were accessed to identify patients referred for 18F DCFPyL PET/CT to investigate known or suspected prostate cancer. All reports were reviewed to identify patients with suspected incidental non-prostate cancer related tumours. These studies were then reviewed to describe the imaging features, SUV, and proportion/volume of avid tumour. Histology was obtained from medical records and pathologic databases.
**Results or Findings:** Over 1,300 studies were reviewed, with potential non-PCa tumours present in around 10%. Lung nodules comprised approximately half of these. Other tumours include renal cell carcinoma, primary bronchogenic carcinoma, recurrent breast cancer, recurrent laryngeal cancer, lymphoma, grade 4 glioma, transitional cell carcinoma and gastrointestinal stromal tumour. Branch duct IPMN was a common benign tumour.
**Conclusion:** Incidental non-PCa tumours are common with around 50% of potential tumours comprising non-avid small lung nodules. Visceral malignancy and recurrent tumours in a diverse range of organs are also frequently encountered.
**Limitations:** Retrospective review is dependent upon report quality for case identification.
**Ethics Committee Approval:** New Zealand national QA ethics. St Vincent's QA department.
**Funding for this study:** We do not require any funding for this study.
**Author Disclosures:**
Sanjana Sharma: Nothing to disclose
Tom Sutherland: Nothing to disclose
Elisa Perry: Nothing to disclose

### RPS 307-10

**68Ga-PSMA PET/CT in prostate cancer patients with biochemical recurrence after primary treatment: diagnostic performance and impact on management strategy**

*R. Wang*, R. Tian; Chengdu/CN
*(wangrangscu@qq.com)*

**Purpose:** We aimed to evaluate the diagnostic performance of 68Ga PSMA PET/CT in detecting relapse or metastasis in patients with biochemical recurrence (BCR), and to further assess the impact of 68Ga PSMA PET/CT on the management strategy.
**Methods or Background:** The PubMed, EMBASE, Cochrane Library and Web of Science databases were searched. Studies regarding the diagnostic performance of 68Ga-PSMA PET/CT in patients with BCR were included. The detection rate was pooled using a random-effects model. Studies reporting the impact of 68Ga PSMA PET/CT on the management of patients with BCR were also included. The proportion of management changes was calculated using a random-effects model. Subgroup and meta-regression analyses were performed to explore heterogeneity.
**Results or Findings:** Fifty-two studies were included. In patients with BCR, the pooled detection rate was 77%. For the prostate-specific antigen (PSA) categories of 0-0.19 ng/ml, 0.2-0.49 ng/ml, 0.5-2.0 ng/ml, 2.0-1.9 ng/ml and above 2 ng/ml, the pooled detection rates were 26%, 45%, 62%, 84% and 94%, respectively. The impact of 68Ga PSMA PET/CT on management was also studied, and the pooled proportion of management changes was 55%. Among these, increasing proportions were seen for treatments including radiotherapy, multimodal treatment, focal treatment and surgery, whereas the proportions of systemic treatment and no treatment decreased due to positive 68Ga PSMA PET/CT scans.
**Conclusion:** 68Ga PSMA PET/CT had high diagnostic accuracy in biochemically recurrent PCa, and the detection rate was positively correlated with PSA levels. Furthermore, 68Ga PSMA PET/CT had a significant impact on patient management, particularly patients being considered for systematic treatment.
**Limitations:** None.
**Ethics Committee Approval:** None.
**Funding for this study:** The National Natural Science Foundation of China (grant 81971653).
**Author Disclosures:**
Rong Tian: Nothing to disclose
Rang Wang: Nothing to disclose

### RPS 307-11

**Making an art into a science: a mathematical “Likert tool” can change PI-RADS (v2) scores into Likert scores when reporting mpMRI for prostate cancer**

*W. M. Stevens*[1], C. Parchment-Smith[2], P. Melling[2], J. T. Smith[2]; [1]Huddersfield/UK, [2]Leeds/UK

**Purpose:** To create a tool which can objectively convert PI-RADS (v2) to Likert scores, based on features shown to be predictors of clinically significant prostate cancer (CSPC).
**Methods or Background:** 791 patients referred with query prostate cancer between 2017 and 2019 were prospectively allocated a Likert score by an experienced reporter based on their PI-RADS scores, clinical and radiological features. Histology results were used to compare the predictive accuracy of both PI-RADS and Likert scoring systems. A logistic regression was run based on criteria found to predict CSPC in a (n=411) cohort of 2018-19 patients. This was used to create a “Likert tool” which, when applied to a PI-RADS score would produce a Likert score. The tool’s performance was tested against the same patients and against an earlier (n=380) 2017-18 cohort of patients.
**Results or Findings:** Assuming a policy whereby patients with a PI-RADS/Likert score of 3 or above are biopsied, the Likert score allocated by the reporter to the cohort of 411 patients (sensitivity 0.92, specificity 0.77) would have resulted in 107 fewer biopsies and 20.3% higher cancer yields than the PI-RADS score (sensitivity 0.99, specificity 0.43). Four criteria found to predict CSPC: PI-RADS (v2) score; Prostate Specific Antigen Density (PSAD); age and contrast enhancement pattern (CEP). These were used to create the “Likert tool”. Similar outcomes (111 fewer biopsies, 22.3% increase in cancer yield) could be seen when the “Likert tool” was applied to the same patient cohort (sensitivity 0.93, specificity 0.79) and to the earlier cohort.
**Conclusion:** A “Likert tool” could reduce inter-reporter variability and, if linked to an agreed biopsy policy, increase CSPC yield whilst decreasing the number of patients unnecessarily biopsied.
**Limitations:** Only a single reporter was used in this study
**Ethics Committee Approval:** n/a
**Funding for this study:** n/a
**Author Disclosures:**
William Mark Stevens: Nothing to disclose
Philip Melling: Nothing to disclose
Jonathan Smith: Nothing to disclose
Catherine Parchment-Smith: Nothing to disclose

### RPS 307-12

**Follow-up for women after treatment for cervical cancer: a study about 418 patients**

*R. Taoussi*, E. Assia, D. Anouar, S. Jelloul, A. Jadib, M. Sabiri, S. Lezar, F. Essodegui; Casablanca/MA
*(taoussi.md@gmail.com)*

**Purpose:** MRI now occupies a prominent place in the post-treatment evaluation of cervical cancer and in the detection of its complications. It is therefore useful to know the normal post-treatment MRI aspects of cervical cancer and to know how to differentiate a post-treatment change from a tumor residue.
**Methods or Background:** Descriptive retrospective study on MRI scans of 418 patients received at the Central Radiology Department of the Ibn Rushd University Hospital of Casablanca, Morocco, between January 2014 and January 2020 for post-therapeutic assessment of cervical cancer
**Results or Findings:** We identified 418 cases, the average patient age was 56.6 years [23 years - 87 years]. They were distributed as follows: 185 patients had an ad integrum restitution of the zonal anatomy, there were 31 patients with a tumor recurrence, 12 with a tumor residue, 36 patients with obvious parametrial involvement without involvement of the pelvic wall,, 28 a post-radiation proctitis, 30 ureterohydronephrosis.
**Conclusion:** MRI is an important step in the assessment, lesion characterization and loco-regional extension of cervical cancers, providing vital information in lesion staging and therapeutic planning.
**Limitations:** No limitations were found
**Ethics Committee Approval:** Yes
**Funding for this study:** No fundings were needed
**Author Disclosures:**
Daki Anouar: Nothing to disclose
Fatiha Essodegui: Nothing to disclose
Abdelhamid Jadib: Nothing to disclose
Reda Taoussi: Nothing to disclose
Mouna Sabiri: Nothing to disclose
Elberhoumi Assia: nothing to disclose
Samira Lezar: Nothing to disclose
Sarah Jelloul: Nothing to disclose

### RPS 307-13

**Role of radiomics in the evaluation of PI-RADS 3 lesions**

*D. Nicoletta*[1], P. N. Franco[2], P. A. Bonaffini[3], E. De Bernardi[2], A. Corsi[2], R. Simonini[2], S. Sironi[2]; [1]Merate/IT, [2]Milan/IT, [3]Bergamo/IT
*(dario.nicoletta1988@gmail.com)*

**Purpose:** To evaluate the role of radiomics, combined with routine clinical parameters, in identifying clinically significant tumor in PI-RADS 3 lesions in prostate MRI studies.
**Methods or Background:** Sixty-eight patients with at least one PI-RADS 3 lesion (according to PI-RADS v2.1), detected on prostate MRI on a 3T MRI scanner were retrospectively included. Final pathological analysis by means of MRI-targeted biopsy was used as a reference standard. Lesions were manually contoured in ITK-SNAP on T2 sequences and ADC maps after normalization and magnetic field inhomogeneity correction, radiomics analysis was performed in Pyradiomics with 64-bins quantization. A total of 586 radiomics features per patient were computed. Clinical features included: age, PSA, PSA density and mean ADC value in a circular 2D ROI. Clinical and radiomics features best correlated with histologic results and less correlated to each other were selected. Multivariate predictive models based on clinical features only and on both clinical and radiomics features were assessed with 17-fold cross-validation.
**Results or Findings:** At pathological analysis 25 lesions were identified as clinical significant tumors (Gleason 3+4). PSA density, mean ADC in 2D ROI and 4 radiomic features (1 from T2, 3 from ADC maps) were selected. The best clinical radiomics model (KNN model based on PSA density, T2 glcm-Autocorrelation, ADC wavelet-LLL-firstorder-Median; AUC: 0.86, σ: 1 e-02) turned out to be more predictive than the best clinical-only model (cubic SVM model based on PSA density, mean ADC value in 2D ROI; AUC: 0.81; σ: 0.02), with P-value of 2 e-03.
**Conclusion:** T2 and ADC radiomic features, combined with routine clinical parameters, might help improving the identification of clinically significant prostate tumor in PI-RADS 3 lesions.
**Limitations:** The retrospective nature of the study and the paucity of patients.
**Ethics Committee Approval:** Not required.
**Funding for this study:** Not required.
**Author Disclosures:**
Dario Nicoletta: Nothing to disclose
Pietro Andrea Bonaffini: Nothing to disclose
Elisabetta De Bernardi: Nothing to disclose
Roberto Simonini: Nothing to disclose
Paolo Niccolò Franco: Nothing to disclose
Andrea Corsi: Nothing to disclose
Sandro Sironi: Nothing to disclose

### RPS 307-14

**Risk stratification of PIRADS 3 nodules using clinical/radiological characteristics in comparison to histopathological gold standard**

*F. Monelli*[1], F. Fiocchi[2], L. Venerandi[3], M. C. Sighinolfi[2], B. M. C. Rocco[2], G. Ligabue[2], P. Torricelli[2]; [1]Reggio Emilia/IT, [2]Modena/IT, [3]Riccione/IT
*(mofilippo@hotmail.it)*

**Purpose:** PIRADS3 represents 19-36% of prostate lesion at MRI but only 6-31% are clinically significative lesions (CSL) defined by a Gleason Score≥7. Our aim was to evaluate clinical/radiological features which may help to identify PIRAD3 nodules at a higher risk of being CSL.
**Methods or Background:** We included every patient who received a 3T-MRI with detection of a PIRADS3 nodule, availability of PSA value within 1 month and histopathological characterization of the lesion within 6 months from MRI. Images have been evaluated prospectively by two radiologists who registered volume, ADC, ADC-ratio (fraction between lesion ADC and ADC of contralateral healthy gland) and PSA-density. The database was randomly subdivided into two equal cohorts. Presence of statistical differences in clinical/radiological variables between CSL and non CSL lesions were analysed in cohort-1 (n=40, CSL=3). Selected variables were used to build an algorithm to distinguish high risk from low risk nodules. This algorithm was tested on cohort-2.
**Results or Findings:** 81 nodules were included, 7 were CSL (8.64%). PSA-density and ADC-ratio had a statistical correlated with CSL (p<0.05). Optimal cut-offs in cohort-1 (cohort-1=40) to differentiate CSL from non CSL nodules were: PSA-density 0.13 and ADC-ratio 0.708 for PZ and 0.800 for TZ. The algorithm was designed to assign a high risk of CSL to nodules with both parameters positive and a low risk to nodules with both negative. Applying the algorithm to cohort-2 (n=41, CSL=4) every CSL was placed in high risk category and 13/37 non CSL were reclassified to low risk. Considering only reclassified lesions accuracy was high: Sensitivity=100% (IC 39.8%-100%), specificity=81.25% (IC 54.4%-96.0%).
**Conclusion:** PSA-density and ADC-ratio are useful to stratify the risk of CSL in PIRADS3 nodules at 3T-MRI.
**Limitations:** Low number of patients and CSL.
**Ethics Committee Approval:** AreaVastaEmiliaNord ethical committee.
**Funding for this study:** None.
**Author Disclosures:**
Maria Chiara Sighinolfi: Nothing to disclose
Pietro Torricelli: Nothing to disclose
Federica Fiocchi: Nothing to disclose
Laura Venerandi: Nothing to disclose
Filippo Monelli: Nothing to disclose
Guido Ligabue: Nothing to disclose
Bernardo Maria Cesare Rocco: Nothing to disclose

### RPS 307-15

**18F choline PET/MR to assess clinically significant disease in prostate cancer: correlation with maximum and total cancer core length obtained via template mapping biopsies**

*L. M. Davis*, A. Haroon, A. Freeman, A. Afaq, H. U. Ahmed, M. Emberton, J. Bomanji; London/UK
*(lauramaydavis@nhs.net)*

**Purpose:** To compare diagnostic accuracy of 18F Choline PET/MR against reference standard Template Mapping biopsies.
**Methods or Background:** 26 prostate lobes in 13 patients (age range 56-79; median 66 years) who had no evidence of metastatic disease on 18F Choline PET/MR and underwent TPM for evaluation of disease burden in the prostate gland and presence or absence of metastatic disease.
**Results or Findings:** Mean age was 66 years (median 66; range 56-79 years), mean PSA level 5.17 ng/mL, mean SUVmax was 3.18 (median 2.3; range ranges 0.6-12.3), mean MCCL was 1.5 mm (range 1-9 mm). Prostate cancer was identified in 9 lobes with a Gleason of 6 in 3 lobes, Gleason score of 7 in 6 lobes. In 17 lobes there was no evidence of cancer, clinically significant disease was seen in 4 lobes, and clinically insignificant disease in 5 lobes. 18F Choline PET/MR detected cancer in all 9 lobes containing either clinically significant or insignificant cancer. The 12 lobes, which were positive on PET, had cancer in 7 lobes (True positive) while in 5 lobes there was no evidence of malignancy on histology. Of the 14 lobes reported as normal, 2 had cancer on histology while in 12 lobes (True negative) there was concordance between histology and 18F Choline PET/MR.
**Conclusion:** 18F Choline PET/MR can detect clinically significant and clinically insignificant disease. In patients who have undergone HIFU treatment 18F Choline PET/MR has higher accuracy compared to PET/CT due to better anatomical delineation of post-HIFU changes. False negative cases of 18F Choline PET/MR had very small volume disease (=/<2mm MCCL and TCCL).
**Limitations:** Small sample size
Retrospective study design
**Ethics Committee Approval:** Retrospecitve analysis hence Ethics committee approval was not obtained.
**Funding for this study:** NIHR
Biomedical Research Centre
**Author Disclosures:**
Laura May Davis: Nothing to disclose
Asim Afaq: Nothing to disclose
Alex Freeman: Nothing to disclose
Jamshed Bomanji: Nothing to disclose
Athar Haroon: Nothing to disclose
Hashim U Ahmed: Nothing to disclose
Mark Emberton: Nothing to disclose

### Research Presentation Session: Genitourinary

### RPS 407

### The prostate and other glands

### RPS 407-1

**Incidence of thyroid dysfunction after iodine-based contrast medium administration and identification of risk factors**

*J. Carlqvist*[1], H. Filipsson Nyström[1], U. Nyman[2], M. Hellström[1]; [1]Göteborg/SE, [2]Lund/SE

**Purpose:** To prospectively study contrast medium (CM) effects on thyroid function.
**Methods or Background:** Reported frequencies of contrast-induced thyroid dysfunction vary greatly and are often observational and retrospective. Our aim was to prospectively study CM effects on thyroid function in a randomly selected cohort. Six hundred participants in SCAPIS (Swedish CArdioPulmonary bioImage Study), aged 50–65 years, randomly selected from the Swedish population registry, and scheduled for Coronary Computed Tomography Angiography (CCTA) with intravenous iohexol were included. Blood samples (s-TSH, s-fT4, s-fT3) were obtained immediately before and 6–8 weeks after the CCTA. Individuals with abnormal hormone levels were contacted for additional follow-up. Possible risk factors, such as thyroid peroxidase (TPO) antibodies were analyzed pre-CCTA, and thyroid ultrasound was performed to detect thyroid nodules.
**Results or Findings:** Follow-up blood samples were obtained in 448 of the 600 individuals, in 408 cases (184 men, 224 women) within 4–10 weeks after CCTA. There were no cases of overt hyperthyroidism/thyrotoxicosis or overt hypothyroidism before or after CM exposure. There was a small, yet statistically significant, mean increase of s-TSH (1.81 to 1.95 mIU/L, p<0.01) and s-fT4 (15.33 to 15.54 pmol/L, p<0.01) for the whole cohort. Eight individuals developed subclinical hypothyroidism post-CCTA. We found no correlation between presence of thyroid nodules and CM-induced thyroid dysfunction. Elevated TPO antibodies was associated with greater risk of thyroid dysfunction post-CCTA (p<0.001).

**Conclusion:** Preliminary results showed slight increase in s-TSH and s-T4 after CM exposure but no overt hyper- or hypothyroidism in this randomly selected cohort aged 50–65 years. In-depth analysis of data remains.
**Limitations:** Age span limited to 50–65 years.
**Ethics Committee Approval:** Approved by the Regional Research Ethics Committee, Gothenburg (ref nr 277-17, addendum T1036-17).
**Funding for this study:** Grants from the Swedish state (ALF agreement), Västra Götaland Region and Sahlgrenska University Hospital.
**Author Disclosures:**
Ulf Nyman: Nothing to disclose
Jeanette Carlqvist: Nothing to disclose
Helena Filipsson Nyström: Nothing to disclose
Mikael Hellström: Nothing to disclose

### RPS 407-2

**Interobserver reproducibility of the PI-QUAL scoring system for prostate MRI quality: a pilot study from the NeuroSAFE PROOF trial**

*F. Giganti*, E. Dinneen, V. Kasivisvanathan, A. Haider, A. Freeman, M. Emberton, G. Shaw, C. M. Moore, C. M. Allen; London/UK

**Purpose:** To evaluate the interobserver reproducibility of the PI-QUAL score for assessing the quality of multiparametric magnetic resonance imaging (mpMRI) in patients with prostate cancer enrolled in the multicentre NeuroSAFE PROOF trial, in which patients receive mpMRI before being randomised to robot-assisted laparoscopic prostatectomy using either NeuroSAFE or standard procedure.
**Methods or Background:** PI-QUAL is a 1-to-5 scale, where a score ≤ 2 means that the diagnostic quality is inadequate while a score ≥ 3 implies that the scan is of sufficient diagnostic quality. A PI-QUAL score ≥ 4 means that the mpMRI quality is high, and all significant lesions can be ruled in and out. Two radiologists independently used the published PI-QUAL scoring sheet in 20 different scans. Cohen's κ coefficients and percent agreement were computed.
**Results or Findings:** Sixteen out of 20 scans were conducted on a 1.5T and 4/20 on a 3T system. Seven patients were examined on a Philips, 4/20 on a GE and 9/20 on a Siemens scanner.
Reader 1 scored 6 scans as PI-QUAL 2, 9 as PI-QUAL 3 and 5 as PI-QUAL 4. Reader 2 scored 6 scans as PI-QUAL 2, 7 as PI-QUAL 3 and 7 as PI-QUAL 4. Agreement was substantial (weighted κ = 0.766 and percent agreement = 80%), especially for T2-WI, followed by DCE and DWI.
**Conclusion:** We observed substantial reproducibility in the assessment of PI-QUAL between two experts, particularly for the presence of artefacts and in the evaluation of the quality of the ADC map.
**Limitations:** For the time being, PI-QUAL offers clinicians the only available scoring system for evaluating and reporting the quality of their prostate mpMRI scans but the composition of the scoring system will need to undergo further refinements.
**Ethics Committee Approval:** Yes: 17/LO/1978
**Funding for this study:** Trial number: NCT03317990
**Author Disclosures:**
Clare Mary Allen: Nothing to disclose
Eoinn Dinneen: Nothing to disclose
Greg Shaw: Nothing to disclose
Caroline M Moore: Nothing to disclose
Alex Freeman: Nothing to disclose
Aiman Haider: Nothing to disclose
Mark Emberton: Nothing to disclose
Veeru Kasivisvanathan: Nothing to disclose
Francesco Giganti: Nothing to disclose

### RPS 407-3

**Evaluation of the diagnostic performance of biparametric MRI, with and without quantitative analysis of ADC values vs multiparametric MRI of the prostate**

*G. Carnicelli*, M. Pecoraro, E. Messina, M. del Monte, C. Catalano, V. Panebianco; Rome/IT

**Purpose:** To compare biparametric MRI, with and without quantitative analysis of ADC values, and multiparametric MRI of the prostate in the diagnosis of prostate cancer in naïve patients, evaluating the role of contrast sequences.
**Methods or Background:** 460 patients were retrospectively enrolled: they underwent MRI according to the acquisition protocol defined by PI-RADS v2.1 (T2-weighted morphological sequences, diffusion functional sequences-DWI ,and contrast sequences-DCE). The exams were evaluated by two radiologists, one senior (with more than ten years of experience in prostate MRI imaging) and one junior (with five years of experience). The cases were evaluated initially omitting the contrast sequences, without (method I) and with (method II) quantitative analysis of ADC values; after at least 6 weeks, the cases were re-evaluated by the same readers, with the addition of contrast sequences (method III). The results were stratified according to the pathological categories defined by the International Society of Urological Pathology (ISUP).
**Results or Findings:** For the senior radiologist, no statistically significant differences were demonstrated between the three methods, for all ISUP categories, in the detection of lesions classified as PI-RADS > 3. For the junior radiologist, statistically significant differences were found between the three methods for ISUP category 1 (non-clinically significant carcinoma); for ISUP categories > 1, statistically significant differences were identified between methods I vs II and I vs III, but not between II vs III. The highest inter-reader agreement was demonstrated for method III (k = 0.863).
**Conclusion:** The use of contrast media and the quantitative analysis of diffusion parameters should be performed in patients with PCa suspicion, unless the MRI examination is evaluated by an expert radiologist.
**Limitations:** Single center retrospective study.
**Ethics Committee Approval:** EC approved
**Funding for this study:** Not applicable
**Author Disclosures:**
Emanuele Messina: Nothing to disclose
Valeria Panebianco: Nothing to disclose
Giorgia Carnicelli: Nothing to disclose
Martina Pecoraro: Nothing to disclose
Maurizio del Monte: Nothing to disclose
Carlo Catalano: Nothing to disclose

### RPS 407-5

**Assessing the role of radiomic features from high b-value DWI sequences in the early detection of clinically significant prostate cancer (PCa)**

*M. Mottola*[1], A. Bevilacqua[2], F. Ferroni[2], G. Gavelli[2], D. Barone[2]; [1]Bologna/IT, [2]Meldola/IT
*(margherita.mottola@unibo.it)*

**Purpose:** To assess the role of radiomic features from high b-value DWI sequences in the early detection of clinically significant prostate cancer (PCa).
**Methods or Background:** 76 patients are retrospectively enrolled, who undergo multiparametric MRI (mpMRI) and biopsy examination, where they received a Gleason Score (GS)=3+3 representing non-clinically significant PCa (ncsPCa, n=26) or GS≥3+4 meaning clinically significant PCa (csPCa, n=50). PCa Regions of Interest (ROIs) are outlined on DWI at b=2000s/mm2 and eighty-four local first-order radiomic features are extracted. First, the LASSO-based method selects a subset of relevant features, discarding linearly correlated couples. Then, to prevent overfitting, only the couple with the lowest p-value at Wilcoxon rank-sum test is selected. A Support Vector Machine (SVM) is trained on 48 patients, validated through 3-fold Cross Validation and tested on 28 patients. ROC curve and AUC are used to assess the SVM performance, together with specificity, sensitivity, and Positive Predictive Value (PPV).
**Results or Findings:** The AUC of the ROC curve on the training set is 0.86, with specificity and sensitivity equal to 94% and 77%, respectively, whilst the AUC on the test set is 0.84 with specificity and of 75% and 90%, respectively, and PPV=90%. ncsPCa and csPCa are separated with p=0.007.
**Conclusion:** The classifier shows a very low probability of overtreatment of ncsPCa while a high PPV strongly improves the performances of clinical mpMRI used in triage pre-biopsy setting.
**Limitations:** This study has been carried out on a 3T machine.
**Ethics Committee Approval:** Not applicable
**Funding for this study:** Not applicable
**Author Disclosures:**
Domenico Barone: Nothing to disclose
Fabio Ferroni: Nothing to disclose
Margherita Mottola: Nothing to disclose
Giampaolo Gavelli: Nothing to disclose
Alessandro Bevilacqua: Nothing to disclose

### RPS 407-6

**Pharmacokinetic analysis of benign and malignant diseases of the prostate gland in MpMRI**

G. Yücel[1], *R. E. Büyüktoka*[2], Z. H. Adibelli[2], H. Mollamehmetoğlu[2], E. Sefik[2], E. Vardar[2], B. Günlüsoy[2], H. İ. Bozkurt[2]; [1]Manisa/TR, [2]Izmir/TR
*(rasiterenbuyuktoka@hotmail.com)*

**Purpose:** To evaluate the contribution of pharmacokinetic analysis to differentiate prostate cancer from benign lesions,to correlate ISUP scores with quantitative and semi-quantitative parameters.
**Methods or Background:** Total of 106 lesions who underwent MpMRI, then TRUS-guided cognitive fusion biopsy were included in this study. The MpMRI images were evaluated by a radiologist, based on PI-RADSv2.1 before the biopsy. Patients' age, PSA, PSA density (PSAd), prostate volume, location of lesions, PI-RADSv2.1 scores, pharmacokinetic analysis of normal PZ and lesions were recorded.
**Results or Findings:** The mean age, PSA, prostate volume and PSAd in this study was; 63.5±7.5, 11.68±17.34ng/ml, 62.4±38.08cc and 0.23±0.39 respectively.43 of 106 lesions were malignant. 65.1% of malignant lesions were located in the PZ and 20.9% had uncertain boundaries. There was positive correlation in between PI-RADSv2.1 scores and ISUP scores(p <0,001). Of the PI-RADS 3 lesions 22,2% were diagnosed as prostate cancer(ISUP1). Pharmacokinetic analysis proved that Ktrans and Kep mean values were the lowest in the normal PZ and the highest in the cancer group(p: <0,001).Chi2 value was the highest in the cancer group and was measured quite close to each other in the other groups. Ktrans, Kep and Chi 2 values were significantly higher in cancer patients when the prostate cancer group and the non-cancer group were compared. There was no significant difference between the mean of the other parameters.No statistically significant difference was found between ISUP groups and between ISUP 1 and ISUP>1 in terms of means of these parameters.
**Conclusion:** MpMRI shows the lesions to be biopsied, can predict tumor aggressiveness.PI-RADS is expected to evolve in the future versions, with setting up the international standards for pharmacokinetic analysis in DCE.
**Limitations:** Limited number of patients is observed.
**Ethics Committee Approval:** For this prospective study, the approval of the Ethics Committee was obtained from the local ethics committee.
**Funding for this study:** n/a
**Author Disclosures:**
Enver Vardar: Nothing to disclose
Gülşen Yücel Oğuzdoğan: Nothing to disclose
Halil İbrahim Bozkurt: Nothing to disclose
Hülya Mollamehmetoğlu: Nothing to disclose
Raşit Eren Büyüktoka: Nothing to disclose
Ertugrul Sefik: Nothing to disclose
Bülent Günlüsoy: Nothing to disclose
Zehra Hilal Adibelli: nothing to disclose

### RPS 407-7

**Can prostate biopsy sepsis be eliminated? A radiology departments experience with 215 outpatient transperineal prostate biopsies**
*J. W. Power*, C. G. Cronin, B. Hutchinson, K. O'Malley, D. T. Murphy, M. Hannan, J. Murray; Dublin/IE
*(jackwpower1@gmail.com)*

**Purpose:** To audit post-procedure sepsis after a COVID-mandated transition from transrectal ultrasound-guided biopsy (TRUS) to outpatient transperineal prostate (TP) biopsy.
**Methods or Background:** Outpatient prostate biopsy continues to be performed primarily using TRUS, despite rising antibiotic resistance and post procedural sepsis. The COVID-19 international guidance has been to avoid TRUS biopsy where possible given the increased risk of sepsis associated with the procedure. With some expertise in TP biopsy under GA, we took this opportunity to transition completely from TRUS to TP biopsy under local anaesthetic. We audited sepsis rates in 215 sequential TP prostate biopsies performed from March-October 2020 and compared them with sepsis rates in 590 TRUS biopsies performed in 2019.
**Results or Findings:** In 590 TRUS biopsies, there were 15 cases of clinical sepsis (2.5%; clinical symptoms of sepsis without blood culture growth) and 8 cases of blood stream infection (1.4%). There were 0 cases of post procedural sepsis in 215 patients who underwent TP biopsy (chi-squared test P = 0.007). There was 1 case of haematuria and 1 case of urinary retention requiring hospitalisation in the TRUS group, with no cases seen in the TP group (chi-squared test P = 0.7). In all, there were no adverse event requiring hospitalisation in the TP group.
**Conclusion:** The COVID pandemic recommendation to avoid TRUS prompted our transition to outpatient TP biopsy. This resulted in elimination of post-procedure sepsis. In the COVID era, it is more important than ever to reduce iatrogenic sepsis, both for patient safety and to avoid hospital admissions. Our experience shows that it is possible to move from outpatient radiology-delivered TRUS to an outpatient TP.
**Limitations:** None identified
**Ethics Committee Approval:** NA - no Identifiable patient material included.
**Funding for this study:** None
**Author Disclosures:**
Darra Thomas Murphy: Nothing to disclose
John Murray: Nothing to disclose
Carmel Geraldine Cronin: Nothing to disclose
Jack W. Power: Nothing to disclose
Kiaran O'Malley: Nothing to disclose
Margaret Hannan: Nothing to disclose
Barry Hutchinson: Nothing to disclose

### Research Presentation Session: Genitourinary

### RPS 507

### Reproduction in GI

### RPS 507-1

**Non-contrast MRI visualisation of penile vessels anatomy**
*I. Pyatnitskiy*, R. Ovchinnikov, S. Ivanov, B. Aleksandrov; Moscow/RU
*(i.a.pyatnitskiy@gmail.com)*

**Purpose:** 1) Examine the anatomy of the penile vessels using non-contrast MRI; 2) Evaluate various techniques to visualize the penile vessels.
**Methods or Background:** We examined two groups of patients: Group A: 20 healthy volunteers underwent non-contrast MRI in the flaccid state and PPDU (Alprostadil 10 mcg); Group B: 15 healthy volunteers underwent non-contrast MRI in the flaccid and erection states (Alprostadil) and PPDU, also five of them - contrast-enhanced MRA and CT angiography (CTA). MRI scanner: Siemens Aera 48, 1,5T, 32-Channel Body Coil. MRI protocol: T2_TSE, dynamic T2_TSE (during the erection process), 3D-T2 SPACE FS.
**Results or Findings:** We obtained high-quality MRI images with clear visualization of penile vessels: cavernous (CA), dorsal (DA), urethral arteries, deep and superficial dorsal veins. We have found the anastomoses between DA and CA in 32 volunteers (91%). The average diameters of CA and DA's branch before anastomosis in the inner part of corpus cavernosum (CC) were: 1,15±0,07 (SD) and 1,3±0,2 mm (flaccid state), 1,2±0,1 and 1,5±0,2 mm (blood filling), 1,15±0,1 and 1,4±0,15 mm (erection), respectively.
**Conclusion:** Our study provides a primary understanding of the DA anastomoses anatomy and role in the erection process. The DA branches were bigger than CA with a more substantial increase during CC blood filling, which indicates the equivalent or even more significant contribution to the blood filling of the outer part during the erection process. MRI T2_TSE and SPACE sequences provide a clear visualization of the most penile structures and vessels with high reproducibility of the PPDU, CTA, and contrast-enhanced MRA results and measurements. Probably MRI SPACE sequence may be useful for the study of arterial stenosis.
**Limitations:** The study did not include patients with erectile dysfunction.
**Ethics Committee Approval:** Approved by Ochapovsky Regional Clinical hospital №1
**Funding for this study:** Rostagroexport Ltd.
**Author Disclosures:**
Roman Ovchinnikov: Nothing to disclose
Sergey Ivanov: Nothing to disclose
Boris Aleksandrov: Nothing to disclose
Ilya Pyatnitskiy: Nothing to disclose

### RPS 507-2

**The role of magnetic resonance imaging in the evaluation of placenta accreta spectrum disorders in patients with placenta previa**
*E. Güler*, A. Öz, G. Serin, T. Köse, M. Harman, N. Elmas; Izmir/TR
*(gulerezgi@yahoo.com)*

**Purpose:** To evaluate the use of magnetic resonance imaging (MRI) in the diagnosis of placenta accreta spectrum (PAS) disorders in patients with placenta previa.
**Methods or Background:** MRI scans of patients with suspected placenta previa that were obtained between January 2016 and January 2020 were retrospectively reviewed to identify cases with PAS disorders. The diagnosis of PAS disorders was confirmed by histopathology. Two radiologists independently searched for MRI findings including intraplacental dark T2 bands, uterine/placental bulge, loss of low T2 retroplacental line, myometrial thinning, bladder wall interruption, focal exophytic placental mass, and abnormal vascularization of placental bed, that were recommended for diagnosing PAS disorders by the joint consensus statement of Society of Abdominal Radiology and European Society of Urogenital Radiology. The interobserver reliability was assessed using kappa statistic.
**Results or Findings:** One hundred two patients with placenta previa (median age: 32, range: 20-45 years) were identified. Thirty-one patients (30.4%) had histopathologically confirmed PAS disorders. For the diagnosis of PAS disorders, MRI had a sensitivity of 93.5% and specificity of 85.9%. The positive and negative predictive values were 74.3% and 96.8%, respectively. Substantial agreement was noted among the diagnosis of PAS disorders based on MRI and histopathology (k=0.741). Interobserver agreement was almost perfect for all MRI findings of PAS disorders except for the detection of bladder wall interruption, for which substantial agreement was found (k=0.790).

**Conclusion:** MRI can be a useful tool for the diagnosis of PAS disorders in patients with placenta previa. High reliability was observed for the identification of MRI findings of PAS disorders.
**Limitations:** The retrospective design of the study and lack of ultrasound evaluation for PAS disorders are the major limitations.
**Ethics Committee Approval:** It was approved by institutional review board.
**Funding for this study:** None.
**Author Disclosures:**
Ayşegül Öz Özcan: Nothing to disclose
Nevra Elmas: Nothing to disclose
Gürdeniz Serin: Nothing to disclose
Ezgi Güler: Nothing to disclose
Timur Köse: Nothing to disclose
Mustafa Harman: Nothing to disclose

### RPS 507-3

**Complex investigation of the penile erectile using dynamic MRI and PPDU**

*I. Pyatnitskiy*, R. Ovchinnikov, S. Ivanov, B. Aleksandrov; Moscow/RU
*(i.a.pyatnitskiy@gmail.com)*

**Purpose:** Complex investigation of the penile erectile process using dynamic MRI and Pharmaco-penile Doppler Ultrasonography(PPDU).
**Methods or Background:** Twelve healthy volunteers underwent MRI 1,5T during the erection process (Alprostadil,10 mcg) and PPDU twice (first – continuous exam of the cavernous artery(CA) blood flow during erection, second - deep dorsal vein (DDV)). MRI protocol: dynamic T2 TSE (each 2 min. during 15-20 min till full erection), or T2 Trufi cine (each 1-3 sec). Changes in all parts of the penis were analyzed and compared with PPDU data. Corpus cavernosum(CC) was divided into three parts: crura, middle and outer parts.
**Results or Findings:** During the erection process, CC's outer part is growing the most (from 9,4 to 74,6 ml, or + 690±95%). In the first 5-10 minutes, it actively expands to a plateau. The maximal CA blood flow (inflow, 6,2±1,1 ml/min) and maximal DDV blood flow (outflow, 18,4±3,3 ml/min) corresponds to the point of 50-60% of the max CC's outer part volume. At this moment, CC's crura and middle parts and corpus spongiosum gain the maximum of their volume. Further blood inflow and outflow fall sharply. But the excess of inflow over outflow persists, therefore CC continues to grow actively until the point of disappearance of diastolic blood flow. After equaling intracavernosal pressure and diastolic arterial pressure, CC reaches the plateau.
**Conclusion:** According to our data, this is the first report about MRI and PPDU investigation of the erection process. Dynamic MRI represents the full picture of changes as outer as inner penile parts with precise measurement data. Using data from different diagnostic methods allows achieving a more in-depth understanding of the complex physiological process, like a penile erection.
**Limitations:** Small sample size
**Ethics Committee Approval:** Approved by Ochapovsky Regional Clinical hospital №1
**Funding for this study:** Rostagroexport Ltd.
**Author Disclosures:**
Roman Ovchinnikov: Nothing to disclose
Sergey Ivanov: Nothing to disclose
Boris Aleksandrov: Nothing to disclose
Ilya Pyatnitskiy: Nothing to disclose

### RPS 507-5

**Role of sonosalpinogography in female subfertility – diagnostic or therapeutic tool?**

*N. Neha*, R. Rastogi, R. Najam, R. Agarwal, V. Pratap; Moradabad/IN
*(neha11aug@gmail.com)*

**Purpose:** Sonosalpingography (SSG) has long been in radiology as a less commonly used tool for assessing the patency of fallopian tubes in subfertile females. It's significance is undermined by laparoscopic evaluation (LE) of tubal patency as latter also allows simultaneous therapeutic procedures to restore its patency, if the obstruction exists. But LE is invasive and expensive. Hence, we evaluated the role of SSG not only in diagnosis of tubal obstruction but also its role in diagnosing the cause and if possible relieving the obstruction.
**Methods or Background:** Fifty subfertile females with normal appearing uterus and ovaries on transvaginal ultrasonography were included in our study. SSG was performed to evaluate tubal patency by recording free peritoneal spill. If peritoneal spill was absent bilaterally then the patient underwent laparoscopic evaluation. However, if unilateral or bilateral peritoneal spill was noted, then patient was recruited for assisted reproductive techniques (ART) and the results were correlated with pregnancy.
**Results or Findings:** Out of 50 patients, SSG was able to demonstrate free peritoneal spill at least unilaterally in 46 patients who conceived with ART. In rest of the four patients, with lack of bilateral spill on SSG, two revealed unilateral partial block while other two revealed bilateral tubal block. In all patients SSG correctly depicted the site of obstruction. In nine, patient it revealed PID (tubercular) by demonstrating flimsy peritubal adhesions and in 14 patients, higher pressure exerted during SSG restored the patency with sharp abdominal pain.
**Conclusion:** Our study reveals that SSG is not only a diagnostic Golden Old Tool but a New Therapeutic Platinum tool as well. Hence, SSG should be used more often and can be used to segregate patients who prudently need laparoscopic evaluation.
**Limitations:** None
**Ethics Committee Approval:** Approved
**Funding for this study:** None
**Author Disclosures:**
Rehana Najam: Nothing to disclose
Rajul Rastogi: Nothing to disclose
Ritika Agarwal: Nothing to disclose
Neha Neha: Nothing to disclose
Vijai Pratap: Nothing to disclose

### RPS 507-6

**Evaluating the role of MRU in anterior male urethral strictures**

*R. Rastogi*, N. Jain, P. Joon, V. Pratap; Moradabad/IN
*(rajulrst@yahoo.co.in)*

**Purpose:** Magnetic Resonance Urethrography (MRU) has recently gained acceptance over Retrograde Urethrography for evaluation of anterior urethral strictures as it has the additional advantage of evaluating periurethral region which may affect further management besides obviating radiation exposure. In fact, MRU has been described as a gold standard and one-stop shop for evaluating anterior urethral strictures due to its excellent soft tissue contrast & lack of operator dependence. This prospective study aimed to:
•Compare accuracy of MRU in evaluation of anterior male urethral strictures.
•Role of MRU in predicting management of anterior male urethral strictures.
**Methods or Background:** Twenty male patients with suspected anterior urethral strictures were referred to our department for MRU that was performed on 1.5T scanner following distension of the urethra by instillation of optimal amounts of sterile gel per urethram & application of soft-plastic, penile-tip clamp. High-resolution, T2 weighted images were obtained in all planes. Data related to site & length of stricture, presence or absence of spongiofibrosis with its extent and any other associated abnormality was recorded in MRU. The findings were compared to that of the intraoperative findings.
**Results or Findings:** Out of 20 patients, three patients were excluded from our study due to suboptimal MRI scan quality. Long-segment stricture in anterior urethra was detected in 14 out of 17 (82.4%) patients. In rest three cases, though the stricture was diagnosed as short-segment but revealed spongiofibrosis affecting the patient management. Thus, MRI had an overall accuracy of 100% when correlated with final management.
**Conclusion:** MRU can be used with high accuracy in anterior urethral strictures to predict the correct mode of management in anterior male urethral strictures thus preventing related morbidity due to recurrence.
**Limitations:** None
**Ethics Committee Approval:** Approved
**Funding for this study:** None
**Author Disclosures:**
Neha Jain: Nothing to disclose
Rajul Rastogi: Nothing to disclose
Pawan Joon: Nothing to disclose
Vijai Pratap: Nothing to disclose

### RPS 507-7

**Penile veins non-contrast MRI visualisation**

*I. Pyatnitskiy*, R. Ovchinnikov, S. Ivanov, B. Aleksandrov; Moscow/RU
*(i.a.pyatnitskiy@gmail.com)*

**Purpose:** Evaluate the potential use of non-contrast MRI as a diagnostic tool for penile veins examination.
**Methods or Background:** Fifteen healthy volunteers underwent MRI in the flaccid and erect states (Alprostadil 10 mcg) and PPDU. MRI scanner: Siemens Aera 48, 1,5T, 32-Channel Body Coil. MRI protocol: T2_TSE, dynamic T2_TSE (during penile erection process), 3D-T2 SPACE FS, T2 Trufi cine. T2_trufi_cine was performed during voluntary pelvic floor muscles (PFM) contraction to assess their potential influence on the veins.
**Results or Findings:** We obtained high-quality MRI images with clear visualization of penile vessels. Deep dorsal vein (DDV) had a single-branch type, but it was divided into two branches at the proximal part of the corpus cavernosum (CC) in three volunteers (20%). In all the volunteers, we have found a severe narrowing of DDV between CC and the pubic bone (PB). Before this narrowing, DDV showed significant dilation. We have compared the PPDU (in proximal outer part) and MRI (dilation and narrowing parts) DDV diameters: 2,1±0,2 (SD) mm, 3,2±0,4 mm, 1,1±0,1 mm (flaccid state), 2,8±0,2 mm, 4,8±0,4 mm, 0,8±0,1 mm (erection), respectively. The DDV diameters difference in dilation and narrowing parts was 2,9 and 6 times in flaccid and erect states, respectively. Besides, we have discovered the compression of DDV during PFM contraction.
**Conclusion:** Non-contrast MRI can be effectively used for penile veins anatomy study and potentially for the leakage assessment. We have found the severe narrowing of DDV between CC and PB. In this place, the DDV diameter declines during erection, and DDV is fully compressed during PFM contraction, which can be an essential factor in the veno-occlusive mechanism.
**Limitations:** The study did not include patients with erectile dysfunction.
**Ethics Committee Approval:** Approved by Ochapovsky Regional Clinical hospital №1
**Funding for this study:** Rostagroexport Ltd.
**Author Disclosures:**
Roman Ovchinnikov: Nothing to disclose
Sergey Ivanov: Nothing to disclose
Boris Aleksandrov: Nothing to disclose
Ilya Pyatnitskiy: Nothing to disclose

### RPS 507-8
**Role of uterine artery doppler in subfertile patients**
*N. Neha*, R. Rastogi, V. Pratap; Moradabad/IN
*(neha11aug@gmail.com)*

**Purpose:** Ultrasound (Mostly transvaginal) is the first-line investigation and can be coupled with color Doppler and 3D/4D scans. It is readily available, inexpensive, noninvasive, radiation-free, relatively less time consuming, and easily repeatable. Limitations include subjective errors, limited field of view, interference by obesity or by gaseous bowel loops, suboptimal visualization of fallopian tubes and broad ligament, failure to delineate small ovaries, and inability to obtain images in the surgical plane. Hence, this study aimed to determine the role of uterine artery doppler in subfertile females by determining the relevant RI, PI and SD ratio values.
**Methods or Background:** Thirty patients who are of reproductive age group and have been clinically labelled subfertile were evaluated by Doppler Imaging. Women of reproductive age group were divided in primary & secondary subfertility group. Transvaginal colour Doppler ultrasonography in the ovulatory phase was performed 48hours prior and after the expected date of ovulation in all patients. Pulsatility index (PI), resistive index (RI) and systolic/diastolic ratio (S/D) were recorded and noted in each case with subsequent calculation of their incidences.
**Results or Findings:** The majority of subfertile females (with both primary & secondary subfertility) had high RI, PI and SD ratio values with reversal diastolic component in spectral waveform.
**Conclusion:** Uterine artery doppler is an important imaging tool to evaluate uterine receptivity in subfertile patients and should be performed before resorting to assisted reproductive techniques. Uterine artery doppler indices can be used to objectively to assess the effects of different clinical methods aimed to improve the uterine perfusion. It can also help in selecting the patients for early institution of various therapies and different types of ART at an early stage.
**Limitations:** None
**Ethics Committee Approval:** None
**Funding for this study:** None
**Author Disclosures:**
Rajul Rastogi: Nothing to disclose
Neha Neha: Nothing to disclose
Vijai Pratap: Nothing to disclose

### Research Presentation Session: Genitourinary

### RPS 607
### Imaging of the kidney

### RPS 607-2
**Cost-effectiveness analysis of multimodal imaging in diagnosis and follow-up of intermediate complex cystic renal lesions**
*P. Spiesecke*, T. Reinhold, Y. Wehrenberg, S. Werner, A. Maxeiner, J. Busch, T. Fischer, B. Hamm, M. H. Lerchbaumer; Berlin/DE
*(Paul.spiesecke@charite.de)*

**Purpose:** Cystic renal lesions are frequent incidental findings. Due to a certain risk of malignancy, many such lesions require a follow-up diagnostic test. The present study evaluates costs and outcomes of patients with Bosniak IIF and III renal cysts detected and followed up by either contrast-enhanced computed tomography (ceCT), contrast-enhanced magnetic resonance imaging (ceMRI), or contrast-enhanced ultrasound (CEUS).
**Methods or Background:** A simulation using Markov models was implemented and performed with 10 cycles of one year each. Proportionate cohorts were allocated to Markov models by a decision tree processing specific incidences of malignancy and levels of diagnostic performance. Costs of imaging and surgical treatment were investigated using internal data of a European university hospital. Multivariate probabilistic sensitivity analysis was performed to confirm results considering input value uncertainties.Patient outcomes were measured in quality-adjusted life years (QALY), and costs as averages per patient including costs of imaging and surgical treatment.
**Results or Findings:** Compared to the gold standard of ceCT, ceMRI was more effective but also more expensive, with a resulting ICER being larger than 70,000€ per QALY gained. CEUS was dominant compared to ceCT in both Bosniak IIF and III renal cysts in term of QALYs and costs. Probabilisitic sensitivity analysis confirmed these results in the majority of iterations.
**Conclusion:** Both ceMRI and CEUS are more effective than ceCT in the diagnosis and follow-up of intermediately complex cystic renal lesions, while CEUS was clearly cost-effective.
**Limitations:** The economic results apply to a large university hospital and must be adapted for smaller hospitals.
**Ethics Committee Approval:** This study was approved by the local institutional ethics committee.
**Funding for this study:** n/a
**Author Disclosures:**
Bernd Hamm: Consultant: Canon Medical Imaging
Paul Spiesecke: Nothing to disclose
Thomas Reinhold: Nothing to disclose
Jonas Busch: Nothing to disclose
Markus Herbert Lerchbaumer: Consultant: Siemens Healthineers
Andreas Maxeiner: Speaker: Hitachi-Medical-Systems, Canon Medical Systems, Stryker and Janssen-Cilag
Thomas Fischer: Consultant: Bracco and Canon Medical Imaging
Yano Wehrenberg: Nothing to disclose
Sven Werner: Nothing to disclose

### RPS 607-3
**MRI texture analysis of transplanted kidney for the prediction of interstitial fibrosis and tubular atrophy at renal biopsy: an observational retrospective study**
*F. Monelli*[1], G. Besutti[1], F. Fontana[2], V. Trojani[1], M. Bertolini[1], G. Ligabue[2], P. Torricelli[2], P. Giorgi Rossi[1], P. Pattacini[1]; [1]Reggio Emilia/IT, [2]Modena/IT
*(mofilippo@hotmail.it)*

**Purpose:** To evaluate the accuracy of MRI texture features in predicting Interstitial Fibrosis and Tubular Atrophy (IFTA) at renal biopsy (RB), the reference standard.
**Methods or Background:** All consecutive patients in follow-up after kidney transplant who received MRI and RB within 6 months were included. RB was classified following Banff guidelines. Radiomic analysis was performed on axial T2 pre-contrast and T1 fat-suppressed post-contrast sequences. The whole renal parenchyma (PAR) was segmented and labelled on T2 and T1, renal cortex (COR) only on T2. After imaging preprocessing, PyRadiomics was used to extract radiomic features. After removal of shape features, 93 features were included and reduced using LASSO regression to produce radiomic signatures. These were introduced in Machine Learning (ML) models to test the association with IFTA (25% and 50% thresholds) and with specific histopathologic outcomes: Sclerosis, Inflammation, Glomerulitis and Peritubular Capillaritis (GCPT) (presence/absence). 5-fold cross-validation was used.
**Results or Findings:** Sixty patients were included; some patients performed multiple MRI and biopsy: 67 pairs of MRI/biopsy met inclusion criteria. Among ML models, three showed a good performance; results are reported as AUC and a value of sensitivity and specificity which optimizes the test accuracy. T2 COR “firstorder_minimum/firstorder_range/glrlm_run_entropy” for IFTA≥50% (AUC=0.77, sensitivity=73%, specificity=71%), T1 PAR “firstorder_energy” for IFTA≥25% (AUC=0.71, sensitivity=74%, specificity=51%), T1 PAR “firstorder_energy/gldm_small_dependence_low_gray_level_emphasis” for GCPT (AUC=0.74, sensitivity= 78%, specificity=68%).
**Conclusion:** We propose a method to extract renal MRI imaging radiomic features, which may have the potential to produce biomarkers for the prediction of graft diseases.
**Limitations:** Retrospective design and small sample. Further analyses are necessary to explore radiomic signature potential in the prediction of clinical rather than histopathological outcomes.
**Ethics Committee Approval:** AVEN, Number: AOU 0010167/20
**Funding for this study:** None.

**Author Disclosures:**
Pietro Torricelli: Nothing to disclose
Marco Bertolini: Nothing to disclose
Pierpaolo Pattacini: Nothing to disclose
Filippo Monelli: Nothing to disclose
Guido Ligabue: Nothing to disclose
Francesco Fontana: Nothing to disclose
Paolo Giorgi Rossi: Nothing to disclose
Valeria Trojani: Nothing to disclose
Giulia Besutti: Nothing to disclose

### RPS 607-4

**Oral hydration compared to intravenous hydration in the prevention of post-contrast acute kidney injury in patients with chronic kidney disease stage IIIb: a phase III non-inferiority study**

*A. Páez Carpio*, M. D. C. Sebastia Cerqueda, E. Guillen, B. Paño, E. Poch, L. Oleaga, C. Nicolau; Barcelona/ES
*(alfredopaezcarpio@gmail.com)*

**Purpose:** To evaluate the non-inferiority of oral hydration compared to intravenous (IV) hydration in the prevention of post-contrast acute kidney injury (PC-AKI) in patients with stage IIIb CKD referred for an elective CE-CT.
**Methods or Background:** This is a prospective, randomized, phase 3, parallel-group, open-label, non-inferiority trial. Patients were randomly assigned 1:1 to receive prophylaxis against PC-AKI either with oral hydration: 500 mL of water two hours before and 2000 mL during the 24 hours after performing CE-CT or IV hydration: sodium bicarbonate (166mmol/L) 3 mL/kg/h starting one hour before and sodium bicarbonate (166mmol/L) 1 mL/kg/h during the first hour after CE-CT. The primary outcome was the proportion of PC-AKI in the first 48 to 72 hours after CE-CT. Secondary outcomes were persistent PC-AKI, the need for hemodialysis, and the occurrence of adverse events.
**Results or Findings:** Of 264 patients randomized between January 2018 and January 2019, 114 received oral hydration, and 114 received IV hydration and were evaluable. No significant differences were found between arms in clinical characteristics or risk factors (p=0.13->0.95). PC-AKI rate was 4.4% (95%CI: 1.4-9.9%) in the oral hydration arm and 5.3% (95%CI: 2.0-11.1%) in the IV hydration arm. IV hydration arm's 90% upper bound was 10.2%. Persistent PC-AKI rate was 1.8% (95%CI: 0.2-6.2%) in both arms. No patient required dialysis during the first month after CE-CT or had adverse effects related to the hydration regime.
**Conclusion:** Oral hydration is non-inferior compared to IV hydration in the prevention of PC-AKI in patients with stage IIIb CKD referred for an elective CE-TC.
**Limitations:** Unbalanced groups due to non-stratified randomization. PC-AKI rates were lower than predicted when designing the trial.
**Ethics Committee Approval:** This study was approved by our institution's ethics committee.
**Funding for this study:** This study was not supported by any funding.
**Author Disclosures:**
Elena Guillen: Nothing to disclose
Carlos Nicolau: Nothing to disclose
Blanca Paño: Nothing to disclose
Maria Del Carmen Sebastia Cerqueda: Nothing to disclose
Laura Oleaga: Nothing to disclose
Alfredo Páez Carpio: Nothing to disclose
Esteban Poch: Nothing to disclose

### RPS 607-5

**Perfusion computed tomography in the assessment of functional outcomes after nephron-sparing surgery**

*E. Kryaneva*[1], N. Rubtsova[1], A. B. Golbitc[1], A. V. Leont'ev[1], A. D. Kaprin[2]; [1]Moscow/RU, [2]Obninsk/RU

**Purpose:** To investigate the relationship between changes of perfusion parameters of affected kidney and renal function in patients with renal cell carcinoma undergoing laparoscopic partial nephrectomy (PN).
**Methods or Background:** 68 patients with primary renal tumors were included in our study. In the first group (n=31) PN was performed without any vascular clamping. Patients included in the 2nd and 3rd groups had renal artery clamping: warm ischemia time (WIT) in the 2nd group (n=15) was 8-25 minutes, in the 3rd group (n=12) WIT was 26-45 minutes. Overall renal function was assessed by glomerular filtration rate (GFR). Split renal function measured using nuclear medicine renal scintigraphy (NMS) with 99mTc diethylene triamine penta-acetic acid . Effective renal plasma flow (ERPF) was evaluated. The following perfusion parameters of cortex and medulla of affected kidney, and ischemia zone were measured: blood volume (BV), blood flow (BF), mean transit time (MTT) and permeability surface area product (PS). ERPF, perfusion parameters and GFR were assessed at baseline pre-operative and at 4 days and 3 months post-operatively. The changes of the parameters were evaluated: early and late postoperative changes and recovery of renal function.
**Results or Findings:** The study obtained significant ($p<0.05$) correlation between changes of following perfusion parameters: BV, BF and MTT of affected kidney cortex with alterations of function of the affected kidney in a 3-day and 4-month period. There were no significant correlation ($p>0.05$) between perfusion parameters and overall renal function in a 3-day period, but they were identified in the 4-month interval.
**Conclusion:** Alterations of perfusion parameters were significantly correlated with NMS data, which allows using perfusion CT data as an independent objective method for evaluating function of the affected kidney after acute ischemic injury.
**Limitations:** n/a
**Ethics Committee Approval:** n/a
**Funding for this study:** None
**Author Disclosures:**
Elena Kryaneva: Nothing to disclose
Aleksandra Borisovna Golbitc: Nothing to disclose
Andrey Dmitrievich Kaprin: Nothing to disclose
Aleksey Victorovich Leont'ev: Nothing to disclose
Natalia Rubtsova: Nothing to disclose

### RPS 607-6

**Diagnostic value of combined magnetic resonance urography and apparent diffusion coefficient calculation in pediatric obstructive uropathy**

N. Adel Zeed[1], *E. H. A. Emara*[2], A. Sakr[1], S. Khalil[1], A. Abdel Azim[1], A. Amer[1], A. Al Gayar[1]; [1]Zagazig/EG, [2]Kafr El Sheikh/EG
*(emademara85@yahoo.com)*

**Purpose:** to assess combined role of magnetic resonance urography and ADC value measured by DWI in evaluation of renal morphology and function in pediatric obstructive uropathy and to find correlation between ADC value measured by DWI and split glomerular filtration rate(GFR) in children with obstructive uropathy.
**Methods or Background:** 40 pediatric patients with urinary tract obstruction were included. Diagnostic work up included ultrasound, voiding cytogram, MRI, Static MRU, DWI and Renal scintigraphy. The patients were divided according to the degree of renal insufficiency into group A of 26 renal units (mild renal impairment), group B of 24 renal units (moderate renal impairment) and group C of 13 renal units (severe renal impairment)
**Results or Findings:** Conventional MRI & MRU provided accurate information regarding the cause and level of obstruction as well as associated congenital anomalies. The most common cause of obstruction was pelvi-ureteric junction obstruction (12 patients) followed by megaureter (7 patients) then obstructed duplex (6 patients) and inadequate pyeloplasty (4patients). The mean ADC value of hydronepherotic kidney was (1.56+ 0.21 X10-3) which was lower than ADC value of contra-lateral non hydronepherotic kidney (2.22+ 0.15 X10-3) with high statistical significance difference (P 0.0001). mean ADC values of the 3 groups were 1.82+0.08 X10-3 for group with e GFR >50 ml /min, 1.52+0.09 X10-3 for group with e GFR (25-50 ml /min) and 1.32+0.07 X10-3 for group with e GFR <25 ml /min. There was positive correlation between renal ADC value of obstructed hyrdonephrotic kidney and renal split GRF (p 0.001) .
**Conclusion:** the combination of MRU and ADC calculation provided valuable morphological and functional information in obstructive uropathy and ADC can reflect the filtration function of obstructed hydronephrotic kidney
**Limitations:** Motion artifact
**Ethics Committee Approval:** Yes
**Funding for this study:** No funding
**Author Disclosures:**
Nesma Adel Zeed: Nothing to disclose
Ahmed Sakr: Nothing to disclose
Alaa Al Gayar: Nothing to disclose
Ahmed Abdel Azim: Nothing to disclose
Ayman Amer: Nothing to disclose
Emad Hassan Ali Emara: Nothing to disclose
Salem Khalil: Nothing to disclose

### RPS 607-7

**DECT parameters as predictors of laser energy used in lithotripsy**

*H. Fruchtman-Brot*[1], Z. Romman[2], I. Leichter[1], M. Duvdevani[1], J. Sosna[1]; [1]Jerusalem/IL, [2]Haifa/IL
*(hilafru@gmail.com)*

**Purpose:** Stones chemical composition may affect the level of energy required during lithotripsy. We aimed to assess the correlation between urinary stone values of electron density (ED) and effective atomic number (Zeff) measured on Dual-Energy Computed Tomography (DECT) with the level of energy required during lithotripsy.

**Methods or Background:** We performed a retrospective study of 30 patients (26 M, 4 F, average age 50.4 years) scanned on a single source DECT (IQON, Philips, Eindhoven-Netherlands), in which a stone was identified and treated by lithotripsy later. Hounsfield unit (HU), Zeff and ED values were measured and standard deviation (SD) was calculated. Data regarding the energy used during lithotripsy was obtained. Spearman's ρ, Mann Whitney test and F test were used.
**Results or Findings:** Stone volume was on average 444 mm3 (range 11-2162). Energy/volume used was on average 11.75 Joule/mm3 (range 0.4-30.5). Six stones had a Zeff value ≤10 compared to 24 stones with Zeff >10. Mean SD of Zeff among stones of Zeff>10 was higher compared to stones of Zeff≤10 (P 0.019). The variance of energy/stone volume was different between values of both groups (P 0.014), with Zeff≤10 showing small variance of energy level compared to Zeff>10. No significant correlation was found between the actual value of Zeff or ED and the level of energy during lithotripsy.
**Conclusion:** Stones of Zeff≤10 are more homogenous and require a rather uniform level of energy which is easier to predict comparing to stones of Zeff>10 which are more heterogeneous and require varying energy levels. DECT provides information regarding urinary tract stones homogeneity and can predict the level of energy in lithotripsy.
**Limitations:** Retrospective study, with 30 patients only
**Ethics Committee Approval:** Yes
**Funding for this study:** None
**Author Disclosures:**
Mordechai Duvdevani: Nothing to disclose
Isaac Leichter: Nothing to disclose
Hila Fruchtman-Brot: Nothing to disclose
Jacob Sosna: Nothing to disclose
Zimam Romman: Employee: Philips Healthcare Haifa Israel

### Research Presentation Session: Genitourinary

### RPS 707

### Genitourinary ultrasound

### RPS 707-1

**First-trimester assessment of placental quotient and vasculature on three-dimensional ultrasound for the prediction of foetal growth restriction**

*S. Jain*, V. Bhardwaj, R. C. Shukla, M. Jain, T. B. Singh; Varanasi/IN
*(shivijain1103@gmail.com)*

**Purpose:** To evaluate the role of placental quotient and vascular indices on three-dimensional ultrasound (3D-USG) for prediction of foetal growth restriction (FGR) in first trimester.
**Methods or Background:** 362 pregnant women with singleton foetus at gestation age (GA) of 6-9 weeks, confirmed through transvaginal ultrasound, were enrolled. At 11-14 weeks, transabdominal 3D-USG assessment of placental volume (PV), placental quotient (PQ), vascularization index (VI), flow index (FI), vascularization flow index (VFI) was done. In 340 women, follow-up was done till delivery. Diagnosis of FGR was confirmed when birth weight (BW) was <10th percentile for that GA with malnutrition clinically. Receiver operator characteristic (ROC) curves were plotted; correlation coefficients of PV, PQ, VI, FI, VFI with birth weight were calculated.
**Results or Findings:** 24.1% (82/340) pregnancies showed FGR. The mean values of PV, PQ, VI, FI & VFI were significantly lower (p<0.001) in FGR than normal (PV: 51.53 ± 8.70 vs 58.11 ± 9.98, PQ: 0.88 ± 0.03 vs 0.91 ± 0.02, VI: 4.00 ± 2.82 vs 6.12 ± 1.09, FI: 18.89 ± 5.46 vs 23.61 ± 2.7, VFI: 2.26 ± 1.64 vs 3.69 ± 0.63, respectively) with their area under ROC curve being 0.685, 0.757, 0.698, 0.756 & 0.769, respectively. Sensitivity and specificity of cut-off values for prediction of FGR for PV (63.5 ml), PQ (0.92), VI (6.5%), FI (26.0), VFI (3.98) were 90.2% and 31.8%, 91.5% and 33.7%, 90.2% and 24.0%, 90.2% and 20.9%, 90.2% and 23.3% respectively. The maximum correlation with BW was noted for VFI followed by FI and VI (r=0.492, 0.422, 0.395, respectively).
**Conclusion:** 3D-USG assessment of placental quotient and vascular indices can help in prediction of FGR in first trimester.
**Limitations:** It was tertiary hospital-based study; results cannot be generalized at community level.
**Ethics Committee Approval:** Taken
**Funding for this study:** None
**Author Disclosures:**
Shivi Jain: Nothing to disclose
Ram Chandra Shukla: Nothing to disclose
Madhu Jain: Nothing to disclose
Vritika Bhardwaj: Nothing to disclose
Tej Bali Singh: Nothing to disclose

### RPS 707-2

**Ultrasound evaluation of scar thickness for predicting the risk of uterine rupture in term women with previous cesarean sections**

*S. A. Saeed*, I. Masroor, A. Majeed; Karachi/PK
*(shaista.afzal@aku.edu)*

**Purpose:** The sonographic evaluation of lower uterine scar near term can help to assess the risk of uterine dehiscence and thus increase safety for those undergoing a trial of labor. The objective of the study was to determine the accuracy of lower uterine scar thickness measurement on ultrasound in predicting the intraoperative outcome of the uterine scar.
**Methods or Background:** This retrospective cross-sectional study conducted in the Radiology Department, Aga Khan University from January 2017 to December 2019 after exemption from the institutional ethics review committee. All patients referred for scar thickness measurements in the third trimester were included. Patients who did not undergo C-section were excluded. The demographic data, comorbidities, clinical presentation, number of prior C-sections, and interpregnancy interval was recorded in the proforma. Data were analyzed to evaluate the sensitivity, specificity, positive predictive, and negative predictive value of scar thickness measurements in predicting the probability of uterine dehiscence.
**Results or Findings:** A total of 126 patients were included. The number of previous C sections was 1 to 5 and the interpregnancy interval ranged from 1 to 11 years. In the present study at a cut-off value of 2 mm for scar thickness, the sensitivity, specificity, positive predictive value, and negative predictive value of ultrasound in predicting uterine dehiscence was 83%, 36%, 93%, and 85% respectively.
**Conclusion:** The ultrasound evaluation of lower uterine segment scar thickness showed a high sensitivity in predicting the intraoperative appearance of the lower uterine segment. The scar thickness measurement can be used as a screening test to predict the integrity of the scar.
**Limitations:** Non-availability of prior operative records, non-provision of intraoperative lower uterine segment scar measurement, and ultrasound is operator dependent
**Ethics Committee Approval:** approved
ETHICS REVIEW COMMITTEE
2020-3616-11439
**Funding for this study:** No funding received
No financial disclosure
**Author Disclosures:**
Aiman Majeed: Nothing to disclose
Shaista Afzal Saeed: nothing to disclose
Imrana Masroor: Nothing to disclose

### RPS 707-3

**Clinical study of transvaginal ultrasonography combined with clinical integration model in menopausal endometrial lesions**

*Y. M. Zhu*, M. Chen, J. Yang; Shanghai/CN
*(10344891@qq.com)*

**Purpose:** To analyze transvaginal ultrasound and clinical data, and to build a clinical integrated model to provide diagnostic basis for the evaluation of endometrial lesions of menopausal.
**Methods or Background:** A retrospective analysis was performed on 156 patients with menopausal endometrial lesions, who were divided into benign lesions group and malignant lesions group. Statistical analysis was performed to establish an integrated ultrasound clinical model.
**Results or Findings:** A total of 156 cases of patients were divided into malignant group (28 cases) and benign group (128 cases). According to univariate Logistic regression analysis, age, abnormal vaginal bleeding, endometrial thickness, endometrial echo uniformity, and endometrial blood flow signal in ultrasonic indicators were screened out for variables with clinical history with statistical significance. Multivariate stepwise logistic regression analysis was conducted to establish corresponding regression equation. Multivariate logistic regression analysis showed that age, endometrial thickness and abnormal vaginal bleeding had statistical significance (P<0.05). The diagnostic efficiency of the regression equation for endometrial benign and malignant lesions, the sensitivity, specificity and AUC of the regression model for the diagnosis of endometrial malignant lesions were 75.0%, 81.3% and 0.85, respectively.
**Conclusion:** The regression model suggested that ultrasound indicators and clinical history had a good diagnostic effect on endometrial lesions in menopause. Among them, endometrial thickness, age and abnormal vaginal bleeding had better diagnostic efficacy and can provide an important basis for the personalized diagnosis and treatment of patients.
**Limitations:** The endometrial malignant lesions studied in this project are few, with bias. The sample size will be increased for further study and observation in the future.
**Ethics Committee Approval:** The study protocol was approved by the clinical medical research ethics committee of our hospital.
**Funding for this study:** None
**Author Disclosures:**
Jun Yang: Nothing to disclose
Ya Mei Zhu: Nothing to disclose
Man Chen: Nothing to disclose

### RPS 707-4

**Comparative role of retrograde urethrography (RGU) and sonourethrography (SUG) in anterior urethral strictures**
*N. Neha*, R. Rastogi, V. Pratap; Moradabad/IN
*(neha11aug@gmail.com)*

**Purpose:** Retrograde urethrography (RGU) has been the mainstay of evaluating the anterior urethra in male patients for clinicians for a long time. However, its invasive nature and radiation exposure has paved a way for introduction of sonourethrography (SUG) that has recently started gaining acceptance. Urethral management primarily depends upon site & length of stricture (>1.5cm length of stricture requires urethroplasty), presence or absence of spongiofibrosis (presence requires urethroplasty), etc. Hence, this prospective pilot study aims for determining: • Comparative role of RGU & SUG in evaluation of anterior urethral strictures • Comparative role of RGU & SUG in predicting management of anterior urethral strictures
**Methods or Background:** Thirty patients with suspected anterior urethral strictures referred to our department were evaluated by RGU followed by SUG with a high-resolution, linear-array, transducer Data related to site & length of stricture; presence or absence of spongiofibrosis with its extent and any other associated abnormality was recorded in both RGU & SUG.
**Results or Findings:** Long-segment stricture in anterior urethra was detected in 20 out of 30 (66.7%) patients by RGU while SUG detected it in 24 out of 30 (80.0%) patients meaning thereby that 4 patients were falsely diagnosed as short-segment stricture by RGU which were in bulbar urethra. More significantly, SUG detected spongiofibrosis in addition to the accurate length of stricture required for management in 10 patients out of thirty affecting the mode of management.
**Conclusion:** SUG is an accurate imaging tool in anterior urethral stricture that not only complements RGU but also affects the mode of management thus affecting the prognosis of the patient, hence should be a routine procedure in all patients with positive findings on RGU
**Limitations:** None
**Ethics Committee Approval:** None
**Funding for this study:** None
**Author Disclosures:**
Rajul Rastogi: Nothing to disclose
Neha Neha: Nothing to disclose
Vijai Pratap: Nothing to disclose

### RPS 707-5

**Normal and asymptomatic pathological urachal remnants in adults**
*F. Loddo*, L. Anfigeno, G. Valenti, J. P. Zawaideh, L. E. Derchi; Genova/IT
*(federico.loddo@fastwebnet.it)*

**Purpose:** The urachus is an embryonic tubular structure between allantois and cloaca which obliterates after birth becoming a fibrous midline cord running from umbilicus to apex of the bladder (the median umbilical ligament). Anomalous remnants result from incomplete involution of this embryonic structure and from these inflammatory or neoplastic complications can follow. Visibility of this structure, how it appears and prevalence of asymptomatic anomalies is poorly understood in adults. The aim of our study is to establish how often and with which shape the median umbilical ligament is visible in adults and to evaluate the prevalence of urachal regression anomalies.
**Methods or Background:** We reviewed the images of 300 randomly selected patients (201 males, 99 females; age range: 14 – 96 years) referred to imaging examinations with unrelated indications between August 2019 and January 2020 (100 US, 100 CT, 100 MRI). Visibility of median umbilical ligament and of urachal regression anomalies was recorded.
**Results or Findings:** The median umbilical ligament was identified in 51% of patients studied with US, in 67% of patients studied by CT and in 71% of patients by MRI. It starts with a triangular shape originating from the anterior wall of the bladder and becomes a tubular structure cephalad. Median length was 62,55 mm. Asymptomatic anomalies were found in 8 cases (2.67%): 4 patients had urachal cyst and 4 vesico-urachal diverticula.
**Conclusion:** Our study demonstrates that normal urachal remnants are frequently visible during imaging studies in adults. Asymptomatic regression anomalies such as cysts or diverticula are relatively rare but probably more frequent than expected.
**Limitations:** Future studies will include a better division between males and females and into age groups.
**Ethics Committee Approval:** Images and data were acquired, anonymously, with the consent of the patients.
**Funding for this study:** No funding received.
**Author Disclosures:**
Federico Loddo: Nothing to disclose
Lorenzo E. Derchi: Nothing to disclose
Lorenzo Anfigeno: Nothing to disclose
Jeries Paolo Zawaideh: Nothing to disclose
Giacomo Valenti: Nothing to disclose

### RPS 707-6

**Role of intraplacental villous artery (IPVA) doppler in normal pregnancies and pregnancies with intrauterine growth restriction (IUGR)**
*N. S. Mehta*, C. Mehta; Vadodara/IN
*(nsjmrad@gmail.com)*

**Purpose:** To evaluate the intraplacental villous artery Doppler indices in normally grown foetuses and growth restricted foetuses. To evaluate the role of intraplacental villous artery Doppler in early detection of IUGR
**Methods or Background:** A quantitative prospective study of pregnant females coming for antenatal ultrasound was conducted using low frequency (3 to 5 MHz) curvilinear transducer of Philips Affinitti 50G at the Radiology Department. Singleton uncomplicated pregnancies were included. A total of 116 pregnant females were included and were scanned for 3 times between 16 to 20 weeks, 25 to 28 weeks and 32 to 36 weeks. The data was analysed with standard statistical tests.
**Results or Findings:** Out of 116 females included, 94 females had normal pregnancy outcome and 22 females developed intrauterine fetal growth restriction. There was a significant decrease in PI, RI and S/D values of IPVA and umbilical artery Doppler with advancing gestational age in normal uncomplicated pregnancies ($p < .05$) . Intraplacental Villous Artery PI, RI and S/D values were significantly higher in IUGR foetuses as compared to normal foetuses ($p < 0.05$) at all three consecutive scans. Umbilical artery PI, RI and S/D values were significantly higher in IUGR foetuses as compared to normal foetuses ($p < 0.05$) at last two scans. There was no statistically significant difference between umbilical artery PI, RI and S/D values between the two groups as early as 16 to 20 weeks.
**Conclusion:** IPVA resistance indices decrease as gestational age increases in normal uncomplicated pregnancies. Lack of decrease in IPVA resistance indices or increase in their values with advancing gestational age may be early predictor of IUGR.
**Limitations:** A tertiary hospital based study in a small sample size, results can not be generalized to community level.
**Ethics Committee Approval:** Ethics Committee approval obtained.
**Funding for this study:** n/a
**Author Disclosures:**
Nirali Satish Mehta: Nothing to disclose
Chetan Mehta: Nothing to disclose

### Research Presentation Session: Head and Neck

### RPS 308

### Ear, nose, and throat

### RPS 308-2

**The pneumatization of the sphenoid sinus, its variations and relations with surrounding neurovascular anatomic structures: a computerized tomography study**
E. Fatihoğlu, *S. Aydın*, E. Karavaş, A. Kantarci; Erzincan/TR
*(sonaydin89@hotmail.com)*

**Purpose:** The wide range of variability of sphenoid sinus (SS) pneumatization and relation with surrounding structures can result in serious complications; seeing that, the assessment of regional anatomy is essential for both surgeons and radiologists. We mainly aim to reveal the possible correlation between the SS pneumatization types and protrusion/dehiscence of the adjacent neurovascular structures in a larger population by using Computerized Tomography (CT) images.

**Methods or Background:** The type of SS pneumatization (I-IV), pneumatization of anterior clinoid process (ACP), greater wing of sphenoid (GWS), and pterygoid process (PP) was evaluated. Protrusion and dehiscence of internal carotid artery (ICA), optic nerve (ON), maxillary nerve (MN), and vidian nerve (VN) was noted.
**Results or Findings:** 1003 patients were included into the study. ICA, ON, and MN protrusions were not seen in patients with the type I or II SS on both sides. These protrusions were most frequently seen along with the type IV SS on both sides ($p<0.05$). ICA, ON, MN, and VN dehiscence was not found in any patients with the type I SS. The rate of ICA protrusion increased with presence of GWS and PP; ICA dehiscence was found to be positively correlated with ACP, GWS, and PP pneumatization.
**Conclusion:** Variations, either amount or the extent, of the pneumatization of the SS are related with the presence of protrusion/dehiscence of ICA, ON, MN and VN. Knowing and reporting these relations can decrease the rate of complications during skull base surgery.
**Limitations:** Larger patient numbers can still change the results. The study was performed in a single center. The data was acquired from CT studies only.
**Ethics Committee Approval:** The present retrospective study was approved by the institutional review board. Informed consent was waived because of retrospective nature.
**Funding for this study:** None
**Author Disclosures:**
Erdal Karavaş: Nothing to disclose
Sonay Aydın: Nothing to disclose
Erdem Fatihoğlu: Nothing to disclose
Abdulmecit Kantarci: Nothing to disclose

### RPS 308-3

**Sound therapy can modulate the functional connectivity of the auditory network**
*H. Lv*, Z. Wang; Beijing/CN
*(chrislvhan@126.com)*

**Purpose:** The functional connectivity of the auditory network is considered to be important in the development of tinnitus. We hypothesized that sound therapy, as a commonly used effective treatment for tinnitus, can modulate the functional connectivity of the auditory network.
**Methods or Background:** In this prospective observational study, we recruited 27 tinnitus patients who had undergone 12 weeks of sound therapy and 27 matched healthy controls. For the two groups of subjects, resting-state functional magnetic resonance imaging was acquired both at baseline and at the 12th week. We utilized independent component analysis and seed-based functional connectivity analysis to characterize the connectivity features within the auditory network and between the auditory network and other brain regions, respectively.
**Results or Findings:** Interaction effects between the two groups and the two scans within the auditory network were observed at the left primary auditory cortex (PAC) and secondary auditory cortex (SAC). The interaction effects were driven by increased functional connectivity in the PAC and decreased values in the SAC in tinnitus patients after treatment. Increased connections between the auditory network and limbic network and decreased values with the bilateral thalami were identified. The effects were mainly driven by the activity of the SAC rather than the activity of the PAC. Significant positive correlations between the percent improvement in the THI score and the percent change in functional connectivity were observed in the SAC-bilateral thalami.
**Conclusion:** Our study provided evidence to support the theory of a gain adaptation mechanism and that quantifies the recovered gating function of the thalamus in tinnitus patients.
**Limitations:** Patients with sham treatments are recommended to be enrolled.
**Ethics Committee Approval:** Beijing Friendship Hospital, Capital Medical University
**Funding for this study:** Grant No.61801311, 61931013 from the National Natural Science Foundation of China
**Author Disclosures:**
Zhenchang Wang: Nothing to disclose
Han Lv: Nothing to disclose

### RPS 308-4

**The risk of complications during endoscopic sinus surgery in cystic fibrosis patients: an anatomical and endoscopic study**
*M. Pietragalla*, L. G. Locatello, C. Nardi, S. Colagrande, L. Bonasera, A. L. Perrone, C. De Amicis, G. Taccetti, G. Maggiore; Florence/IT
*(michelepietragalla2@gmail.com)*

**Purpose:** CFTR mutations profoundly alter both sinonasal development and surgical anatomy. As an increasing proportion of adult cystic fibrosis (CF) patients are referred to endoscopic sinus surgery (ESS) to relieve the symptoms of chronic rhinosinusitis (CRS), the relationship between their peculiar anatomy and the risk of postoperative complications is unclear.
**Methods or Background:** Paranasal sinuses CT scans of 103 CF adult patients with CRS were compared to 100 non-CF adult patients to explore their anatomical differences. From the two groups, patients who received ESS were extracted to assess their preoperative CT scan in terms of surgically relevant variants, according to the CLOSE checklist. Clinical-surgical outcomes were compared between groups.
**Results or Findings:** CF group presented more frequently with smaller and less pneumatized paranasal sinuses and a higher Lund-Mckay score compared with controls. No anatomical differences emerged in terms of genotype stratification. Non-CF patients undergoing ESS showed a significantly deeper olfactory fossa and a more frequent supraorbital pneumatization compared to CF patients ($p<0.001$ and $p=0.032$). CF patients underwent more often aggressive surgical procedures ($p<0.001$), yet no difference in terms of postoperative adverse events was found ($p=0.645$).
**Conclusion:** Despite receiving more often aggressive ESS procedures, adult CF patients do not show an increased risk of postoperative complication.
**Limitations:** The definition of the complications remains subjective.
Single-center experience.
Only primary ESS in adult CF subjects was included.
**Ethics Committee Approval:** All procedures performed in studies involving human participants were in accordance with the ethical standards of the institutional and/or national research committee and with the 1964 Helsinki declaration and its later amendments or comparable ethical standards. Informed consent was obtained from all individual participants included in the study.
**Funding for this study:** No funding
**Author Disclosures:**
Giovanni Taccetti: Nothing to disclose
Michele Pietragalla: Nothing to disclose
Anna Lara Perrone: Nothing to disclose
Luca Giovanni Locatello: Nothing to disclose
Cosimo Nardi: Nothing to disclose
Luigi Bonasera: Nothing to disclose
Stefano Colagrande: Nothing to disclose
Giandomenico Maggiore: Nothing to disclose
Cristian De Amicis: Nothing to disclose

### RPS 308-5

**CT and MRI-based imaging features to predict response to olfactory training in post-traumatic olfactory dysfunction**
A. Altundag[1], O. Saatci[1], *S. G. Kandemirli*[2], D. Tekcan Sanli[1], D. Yildirim[1]; [1]Istanbul/TR, [2]Iowa City, IA/US
*(sedat-kandemirli@uiowa.edu)*

**Purpose:** Prognosis of posttraumatic olfactory dysfunction is poor, with medical treatment options showing limited success rates. Olfactory training (OT) has been introduced as a potential therapeutic option in olfactory dysfunction. We aimed to identify the imaging features that would predict a better response to olfactory training and create an imaging-based prognostic scale.
**Methods or Background:** We retrospectively reviewed 52 patients that underwent olfactory training at our center for posttraumatic olfactory dysfunction. Olfactory function at the time of initial presentation and at completion of olfactory training were evaluated using Sniffin' Sticks test and TDI scores were calculated. Patients were divided into responders (ROT group: 16 cases) and non-responders (n-ROT group: 36 cases) to OT based on TDI score change (cut-off 5.5 point). Morphological measurements of olfactory fossa, olfactory bulb volume and signal abnormalities, olfactory nerve filia integrity, siderosis, encephalomalacic changes in olfactory cortex and other cortical regions were reviewed.
**Results or Findings:** There was no significant difference between the two groups in terms of age, gender distribution, olfactory dysfunction duration, head-trauma severity and initial TDI scores. A model incorporating 5 variables: cribriform plate fracture, olfactory fossa depth (cut-off:4.9 mm), olfactory bulb encephalomalacia, olfactory bulb volume (cut-off:27.1 cm3) and siderosis was developed. This model had an AUC of 0.950, with a cut-off of 1 showing sensitivity of 76.5% and specificity of 97.1% to predict no response to olfactory training.
**Conclusion:** We developed an imaging-based scoring system with good specificity that can be used as an adjunctive tool for patient counseling, and optimal selection of management options.
**Limitations:** Limited patient number and retrospective nature of the study with its inherent limitations, selection bias with overall results of the study reflecting a worst-case scenario.
**Ethics Committee Approval:** Acibadem hospital
**Funding for this study:** Not available.

**Author Disclosures:**
Düzgün Yildirim: Nothing to disclose
Sedat Giray Kandemirli :
Aytug Altundag: Nothing to disclose
Deniz Tekcan Sanli: Nothing to disclose
Ozlem Saatci: Nothing to disclose

### RPS 308-6

**Idiopathic vocal cord palsy: consider tortuosity of the extracranial internal carotid artery**

*J. F. Maier*, L. Abdulhady, H. Glad; Koege/DK

**Purpose:** The cause of vocal cord palsy (VCP) is often unexplained. Clinical practice made us suspect that vocal cord palsy (VCP) can be associated with extracranial internal carotid artery (ICA) tortuosity. The study was conducted in order to find evidence for a causative relation between ICA tortuosity and VCP.
**Methods or Background:** This single-center, retrospective study comprised 66 patients with VCP (39 females, 27 males, mean age 63,9y). A total of 45 patients without VCP (16 females, 29 males, mean age 60,0y) served as control group. Patient charts were reviewed for etiology and side of VCP. CT scans were reviewed independently by two radiologists for imprint in the jugular vein (JV) caused by a tortuous ICA, considered an indirect sign of potential vagus nerve affection.
**Results or Findings:** A total of 33 patients had idiopathic VCP. ICA tortuosity causing a JV imprint with >10% JV lumen reduction was found more frequently on the paretic sides of patients with idiopathic VCP (15–24%) than in controls (3–9%), with $p<.05$ for observer 2 and $p=.07$ for observer 1. In patients with idiopathic VCP and JV imprint with >10% JV lumen reduction on one or both sides (n=9), both observers found JV imprint with >10% JV lumen reduction more frequently on the side of VCP ($p<.05$). The interobserver agreement for grading of JV imprints was kappa= 0.52, i.e. moderate agreement.
**Conclusion:** ICA tortuosity causing a JV imprint may be the cause of VCP in some of the cases of VCP currently regarded as idiopathic.
**Limitations:** Small single center study.
**Ethics Committee Approval:** Danish patient safety authority (Journal no. 3-3013-1713/1)
Danish data protection agency (Journal no. 2016-41-4761)
**Funding for this study:** This study did not receive funding.
**Author Disclosures:**
Luka Abdulhady: Nothing to disclose
Jens Friedrich Maier: Nothing to disclose
Henrik Glad: Nothing to disclose

### RPS 308-7

**Evaluation of temporal bone cholesteatoma with high resolution computed tomography (HRCT)**

*R. Rastogi*, N. Jain, V. Pratap; Moradabad/IN
*(rajulrst@yahoo.co.in)*

**Purpose:** Background: Cholesteatoma is a potentially dangerous condition affecting middle ear cavity. As high-resolution computed tomography (HRCT) of temporal bone clearly depicts the inner anatomy, it can serve as an important imaging tool in evaluating cholesteatoma for preoperative planning. Hence, this study evaluates the efficacy of pre-operative HRCT in the evaluation of patients with middle ear cholesteatoma.
**Methods or Background:** This was a prospective pilot study of 40 patients with chronic suppurative otitis media and unsafe type cholesteatoma. Each patient was subjected to full clinical evaluation, and HRCT examination prior to operative intervention. Preoperative radiological data were correlated with data related to surgical findings.
**Results or Findings:** The study showed that a high incidence of cholesteatoma in the 2nd to 4th decade of life. The scutum and lateral attic wall were the most common bony erosions in the middle ear bony wall in nearly two-third patients. The malleus was the most eroded ossicle in the middle ear in nearly 80% cases. Facial canal erosion was found in nearly one-fifth patients. Temporal bone complications were commoner than intracranial complications. When compared with operative features, HRCT findings had an accuracy of more than 90% in detecting, localizing and determining the extent of cholesteatoma and nearly 100% accuracy in demonstrating ossicular chain erosion, labyrinthine fistula and intracranial complications
**Conclusion:** HRCT scan is an excellent preoperative imaging modality for the otologist to predict ossicular status and determining patient prognosis.
**Limitations:** None
**Ethics Committee Approval:** None
**Funding for this study:** None
**Author Disclosures:**
Neha Jain: Nothing to disclose
Rajul Rastogi: Nothing to disclose
Vijai Pratap: Nothing to disclose

### RPS 308-8

**Olfactory bulb MRI findings in persistent COVID-19 anosmia**

*S. G. Kandemirli*[1], A. Altundag[2], D. Yildirim[2], D. E. Tekcan Sanli[2], O. Saatci[2]; [1]Iowa City, IA/US, [2]Istanbul/TR
*(sedat-kandemirli@uiowa.edu)*

**Purpose:** There is limited literature consisting of case reports or series on olfactory bulb imaging in COVID-19 olfactory dysfunction. A systematic imaging study with objective clinical correlation is needed in COVID-19 anosmia to better understand underlying pathogenesis.
**Methods or Background:** We evaluated 23 patients with persistent COVID-19 olfactory dysfunction. Patients included in this study had minimum 1-month duration between onset of olfactory dysfunction and evaluation. Olfactory functions were evaluated with Sniffin' Sticks Test. Paranasal sinus CTs and MRI dedicated to olfactory nerves were acquired. On MRI, quantitative measurements of olfactory bulb volumes and olfactory sulcus depth and qualitative assessment of olfactory bulb morphology, signal intensity and olfactory nerve filia architecture were performed.
**Results or Findings:** All patients were anosmic at time of imaging based on olfactory test results. Olfactory cleft opacification was seen in 73.9% of cases with a mid and posterior segment dominance. 43.5% of cases had below normal olfactory bulb volumes and 60.9% of cases had shallow olfactory sulci. 54.2% of cases had changes in normal inverted J shape of the bulb. 91.3% of cases had abnormality in olfactory bulb signal intensity in the forms of diffuse increased signal intensity, scattered hyperintense foci and microhemorrhages. Clumping of olfactory filia was seen in 34.8% of cases and thinning with scarcity of filia in 17.4%. Primary olfactory cortical signal abnormality was seen in 21.7% of cases.
**Conclusion:** Our findings support the observations of olfactory cleft inflammation in COVID-19 anosmia. Additionally, relatively high percentage of olfactory bulb degeneration suggests that direct/indirect injury to olfactory neuronal pathways may also take place.
**Limitations:** Relatively low number of patients and selection bias.
**Ethics Committee Approval:** Acibadem Hospital
**Funding for this study:** None
**Author Disclosures:**
Düzgün Yildirim: Nothing to disclose
Sedat Giray Kandemirli :
Aytug Altundag: Nothing to disclose
Deniz Esin Tekcan Sanli: Nothing to disclose
Ozlem Saatci: Nothing to disclose

### Research Presentation Session: Head and Neck

### RPS 408

### Disorders of the head and neck

### RPS 408-1

**The "jugular vein footprint sign": a strong CT imaging predictor of cervical lymph node metastasis in patients with head and neck squamous cell carcinoma**

*G. C. T. E. Garcia*, L. Lebouvier, S. Ammari, C. S. Balleyguier, F. Bidault; Villejuif/FR
*(gabriel.garcia@gustaveroussy.fr)*

**Purpose:** Radiological assessment of lymph node metastasis (LNM) is challenging in patients treated for head and neck squamous cell carcinomas (HNSCC). We present a new CT-scan sign: the "Jugular vein footprint sign" (JVFS), its definition, its frequency and its diagnostic performances to predict cervical LNM.
**Methods or Background:** This is a monocentric retrospective cohort study including consecutive patients newly diagnosed for HNSCC between 2005 and 2018 presenting only one histopathologically proven lymphadenopathy with no previous treatment for HNSCC who underwent surgery with lymph node dissection and histopathological analysis. Pre-treatment neck CT-scans were analyzed by two radiologists blinded to histopathological data. They determined whether or not a LNM was radiologically detectable, in contact with the internal jugular vein (IJV), and if a notch was imprinted on the wall of the IJV (defining JVFS). Same analysis was performed on non-metastatic lymph nodes. Among all lymph nodes in contact with the IJV sensitivity, specificity, positive predictive value and negative predictive value of the JVFS to predict histopathological LNM were computed. Inter-reader reliability was assessed using the Cohen's kappa technique.

**Results or Findings:** 208 patients were screened, 110 with a radiological LNM. Among them 43 LNM (40.6%) were in contact with the IJV as well as 39 non-metastatic lymph nodes. Specificity, sensitivity, positive predictive value and negative predictive value of the JVFS to predict nodal metastatic invasion were respectively 97.4%, 74%, 97.4% and 74.5% with a Cohen's Kappa value of 75% (p<0.001).
**Conclusion:** JVFS is a strong imaging predictor of lymph node metastasis in patients with HNSCC.
**Limitations:** Retrospective study
**Ethics Committee Approval:** Approved by our institutional review board at Gustave Roussy Cancer Center
**Funding for this study:** None
**Author Disclosures:**
Gabriel Cristobal Théophile Eudoxe Garcia: Nothing to disclose
François Bidault: Nothing to disclose
Samy Ammari: Nothing to disclose
Corinne S. Balleyguier: Nothing to disclose
Lea Lebouvier: Nothing to disclose

### RPS 408-2

**Differentiation of orbital malignant and benign tumours with dual-energy computed tomography**

*S. Luo*, Y. Sha; Shanghai/CN
*(724972064@qq.com)*

**Purpose:** To investigate the diagnostic accuracy of dual-energy computed tomography (DECT)-derived iodine concentration (IC), the slope (k) value of spectral attenuation curve, and effective atomic number (Zeff) in differentiating orbital malignant and benign tumors.
**Methods or Background:** Data from 29 patients with orbital malignant or benign tumors were retrospectively analyzed. Each patient underwent DECT examination, using a 128-slice dual-source CT scanner. Dual-energy information (IC, normalized iodine concentration (NIC), Zeff), and spectral information ( k values, calculated from virtual monoenergetic images (VMIs), reconstructed from 40 to 180 keV) were determined. Two experienced radiologists, blinded to clinical, independently analyzed conventional grayscale and virtual monoenergetic DECT series for evaluation. Quantitative analysis was performed by a third radiologist.
**Results or Findings:** A total of 29 patients (age, 48.1±14.4) with pathologically proven orbital malignant and benign tumors (malignant, n=7; benign, n=22) were evaluated. IC, NIC, Zeff and k values of malignant orbital tumors were significantly higher than the benign tumors($p < 0.05$). The qualitative image score was superior in 40 to 100 keV VMI images to conventional images. The CT attenuation was significantly higher in 80 keV reconstructed VMI images in malignant than benign tumors (56.23±9.28 HU vs 73.37±10.91 HU, $P < 0.05$, AUC, 0.91). Multivariate logistic regression analysis showed the combination of parameters (IC, NIC, Zeff, k value, VMI series at 80 keV) had overall high diagnostic performance for differentiation (AUC, 0.97; sensitivity, 95.5%; specificity, 85.7%).
**Conclusion:** Quantitative DECT analysis can be a useful technique which yields superior diagnostic accuracy when compared to conventional measurements for differentiation of orbital malignant and benign tumors.
**Limitations:** This study was performed in a single center with a relatively small sample size.
**Ethics Committee Approval:** This retrospective study was approved by the institutional review board of our hospital.
**Funding for this study:** None
**Author Disclosures:**
Yan Sha: Nothing to disclose
Siqi Luo: Nothing to disclose

### RPS 408-3

**Assessment of microcirculatory changes in normal-appearing white matter of the brain in patients with multiple sclerosis by perfusion MRI**

*Y. Stankevich*, L. Vasilkiv, O. Bogomyakova, A. Tulupov; Novosibirsk/RU
*(stankevich@tomo.nsc.ru)*

**Purpose:** To evaluate perfusion changes in normal-appearing white matter (NAWM) of the brain with demyelinating lesions of the central nervous system using the method of dynamic susceptibility contrast (DSC).
**Methods or Background:** The MR study was carried out on a MR-scanner "Ingenia" ("Philips") 3 Tesla using the method of DSC. The study included 30 healthy volunteers and 80 patients with demyelinating disease of the central nervous system (9 patients with CIS, 66 patients with RRMS and 5 patients with SPMS) over the age of 18 up to 48 years (average age was 34.6 ± 8.02 years).
**Results or Findings:** In all groups in NAWM, a significant decrease in CBF and CBV was observed in all lobes of the brain, and the severity of these changes increases with the progression of the disease: in patients with CIS CBF is reduced to 13.7% and CBV to 7.3%; the most pronounced decrease in perfusion was observed in patients with SPMS: CBF by 40% and CBV by 24.8% ($p < 0.001$); with a moderate increase in TTR and MTT by 15%.
**Conclusion:** Perfusion data complements routine MRI and provides a comprehensive assessment of changes in brain matter.
**Limitations:** All patients received immunomodulating therapy, but not a single patient received systemic corticosteroids during the study or 3 months before the study.
**Ethics Committee Approval:** The study was overseen by the local ethics committee ITC SB RAS, in compliance with the ethical principles of medical research with human participation in accordance with the Declaration of Helsinki of the World Medical Association.
**Funding for this study:** We thank Ministry of Science and Higher Education of the RF for access to MRI equipment (AAAA-A16-116121510090-5). The work was supported by Russian Science Foundation (the project #19-75-00052).
**Author Disclosures:**
Liubov Vasilkiv: Employee: Researcher
Olga Bogomyakova: Employee: Researcher
Yuliya Stankevich: Employee: Researcher
Andrew Tulupov: Employee: Researcher

### RPS 408-4

**Diagnostic accuracy of 4DCT for preoperative localisation of parathyroid adenoma**

*A. Gupta*, M. Garg, V. Aggarwal; New Delhi/IN
*(arushigupta1215@gmail.com)*

**Purpose:** Assess the diagnostic accuracy of 4DCT for localising pathologically proven parathyroid adenomas found at surgery.
**Methods or Background:** • It was a single institution retrospective study done from February 2019 to August 2020. • Inclusion criteria was availability of pathological specimen. No cases were excluded. • Each scan included a scout scanogram, a noncontrast acquisition followed by multiphasic imaging. • Diagnostic accuracy was measured by comparing CT findings to the gold standard of surgical specimen.
**Results or Findings:** A total of 35 pathologically proven cases were collected out of which 30 were found positive on 4DCT including 3 cases of multiglandular disease. For lateralization of single-gland disease, 4D-CT demonstrated an accuracy of 93%. 4D-CT revealed a suboptimal 44% sensitivity, but 100% specificity, for multigland disease.
**Conclusion:** 4DCT demonstrated high diagnostic accuracy for single and multiglandular disease. Potential difficulties include high radiation dose and necessity of iodinated contrast media.
**Limitations:** This was a retrospective study and was not designed to make a direct prospective comparison of the accuracy of sonography, sestamibi, and 4D-CT imaging for the detection of parathyroid pathology. Selection bias for cases of ectopic and multigland disease because these are more difficult to diagnose on traditional imaging modalities and thus more likely to progress to 4D-CT imaging. Although the technique of 4D-CT at our institution has been performed with high accuracy, variability in diagnostic accuracy may occur across institutions, secondary to interpreter experience and study technique.
**Ethics Committee Approval:** Yes
**Funding for this study:** No
**Author Disclosures:**
Monika Garg: Nothing to disclose
Arushi Gupta: Nothing to disclose
Vivek Aggarwal: Nothing to disclose

### RPS 408-6

**High frequency ultrasound in the assessment of thyroid nodules: a prospective trial**

*N. C. Mcaddy*[1], H. K. Yang[2], A. Rattansingh[3], A. Kielar[3], J. Schwock[3], L. Rotstein[3], J. Pasternak[3], K. Khalili[3]; [1]London/UK, [2]Seoul/KR, [3]Toronto/CA

**Purpose:** Compare high frequency ultrasound (HFU) to standard ultrasound (SFU) in categorizing thyroid nodules.
**Methods or Background:** Prospective SFU (14MHz) and HFU (24MHz) were performed on 44 patients (34:10 F:M, mean age 53.9 (range 30-76)) with 47 nodules. Additionally, microflow Doppler (SMI™), punctate echogenic foci (PEF) quantification (using Micropure™) and elastography were performed. One radiologist conducted blinded reviews of randomised raw cine images and classified nodules according to ACR TIRADs. Internal vascularity with SMI™ was quantified using a 6-point scale. Two readers independently quantified PEF with SFU, HFU and Micropure™. FNA was performed for 29/47 (62%) nodules and thyroidectomy for 18 (38%). FNAs were independently assessed by two dedicated cytopathologists, surgical specimens by an endocrine pathologist.
**Results or Findings:** Final diagnosis was benign/likely benign (AUS/FLUS) in 37/47 (78%) and malignant in 10 (21%, all by surgery). TIRADs category distribution from TR1 to 5 were 6,6,6,18,11 by SFU and 10,5,0,13,19 by HFU (p=0.003). Compared to SFU, HFU significantly changed echogenicity

(p=0.0006) and margins (p=0.002) but not composition, calcification or shape TIRADS assessment categories. In detection of nodules requiring biopsy at any size (TR5), the sensitivity/specificity of SFU & HFU were not significant. In detection of benign lesions not requiring biopsy (TR1-2) the SEN/SPE of SFU & HFU were 32%/100% and 38%/90% (NS). The AUC ROC for quantitative detection of malignant calcification by two independent readers for 14 Mhz, 24Mhz and MicroPure™ were 0.49-0.52, 0.46-0.49, and 0.35-0.41. Semiquantitative internal vessel count using microflow Doppler were not a predictor of malignancy.
**Conclusion:** HFU shows promise, improving categorization of nodules into highly suspicious and benign categories. Its use may negate need for biopsy in a higher proportion of benign lesions and facilitate earlier FNA of malignant lesions.
**Limitations:** Study size
**Ethics Committee Approval:** Yes
**Funding for this study:** None
**Author Disclosures:**
Anand Rattansingh: Nothing to disclose
Joerg Schwock: Nothing to disclose
Naami Charlotte Mcaddy: Nothing to disclose
Jesse Pasternak: Nothing to disclose
Hyun Kyung Yang: Nothing to disclose
Lorne Rotstein: Nothing to disclose
Ania Kielar: Nothing to disclose
Korosh Khalili: Nothing to disclose

### Research Presentation Session: Interventional Radiology

### RPS 309

### Advances in vascular interventional radiology

### RPS 309-1

**Portal vein embolization with N-Butyl cyanoacrylate is superior to other materials: a systematic review and meta-analysis**
*A. Ali*[1], M. Ahle[2], B. Björnsson[2], P. Sandström[2]; [1]Lancaster/UK, [2]Linköping/SE
*(adnan.ali.lancs@gmail.com)*

**Purpose:** It remains uncertain which embolization material is best for portal vein embolization (PVE). We investigated the various materials for effectiveness in inducing future liver remnant (FLR) hypertrophy, technical and growth success rates, complications and resection rates.
**Methods or Background:** A systematic review from 1998 to 2019 on materials for PVE was performed on Pubmed, Embase and Cochrane. FLR growth between the two most commonly used materials was compared in a random-effects meta-analysis. In a separate analysis using local data (n=52), n-butyl cyanoacrylate (NBCA) was compared with microparticles regarding costs, radiation-dose, and procedure time.
**Results or Findings:** From 51 papers, 2896 patients, 61.0 ± 4.0 years of age and 65% male were included in the analysis. In 61% of the patients either NBCA or microparticles was used for embolization. The remaining were treated with ethanol, gelfoam or sclerosing agents. The FLR growth with NBCA was 49.1% ± 29.7 compared to 42.2% ± 40 with microparticles (P = .037). The growth success rate with NBCA vs microparticles was 95.3%, vs 90.7% respectively, (P < .001). There were no differences in major complications between NBCA and microparticles. In the local analysis, NBCA (n=41) entailed shorter procedure time and reduced fluoroscopy time (P < .001), lower radiation exposure (P < .01) and lower material costs (P < .0001) than microparticles (n=11).
**Conclusion:** PVE with NBCA seems to be the best choice when combining growth of the FLR, procedure time, radiation exposure and costs.
**Limitations:** 1. No randomised controlled trials were available. 2. Minor complications were not reported explicitly, hence minor complications reported in our study is an underestimate. 3. Local data between NBCA and microparticles was small, but the differences were significantly in favour for NBCA.
**Ethics Committee Approval:** Not applicable.
**Funding for this study:** None.
**Author Disclosures:**
Adnan Ali: Nothing to disclose
Per Sandström: Nothing to disclose
Bergthor Björnsson: Nothing to disclose
Margareta Ahle: Nothing to disclose

### RPS 309-3

**Embolization procedures performed outside the neurological area using Phil® System: our experience**
P. Lucatelli, *L. Teodoli*, B. Rocco, S. Cipollari, P. G. Nardis, A. Cannavale, M. Bezzi, C. Catalano, M. Corona; Rome/IT
*(lteodoli@gmail.com)*

**Purpose:** To evaluate the efficacy of Phil® System liquid embolic agent during embolization procedures performed outside the neurological area.
**Methods or Background:** Forty patients with a mean age of 64.8 years underwent percutaneous embolization using the Phil® System for: splancnic pseudoaneurysms (n=11), abdominal aortic type II endoleaks (N=8), iliac aneurysm endoleak (n=2), AVM (n=2), gastric varices associated with a spleno-renal shunt (n=2), paraumbilical vein varices (n=1), intrapancreatic bleedings (n=2) and arterial bleedings in soft tissue (n=12). Phil® System consists of a co-polymer dissolved in dimethyl sulfoxide (DMSO), with different viscosities. The monomer hydroxyethylmethacrylate is used to make the polymer (PHEMA). Procedures were performed with injection of Phil 25% or Phil®30% at a rate of 0.16 ml/sec, under fluoroscopic guidance to avoid occlusion or embolization of non-target vessels. In case of high flow conditions, Phil® can also be injected in combination with coils avoiding misplacement of the Phil® solution during injection.
**Results or Findings:** Technical success was achieved in all patients. In all cases Phil® embolic device was correctly inserted into the selected branches and no embolization of collateral vessels was recorded. All soft-tissue and intrapancreatic bleedings have been arrested; all endoleaks disappeared during follow-up. Phil® provided durable AVM embolization with exclusion of the nidus and durable embolization of splancinc pseudoaneurysms. After a mean follow-up of 12 months, ranging from 3 to 24 months, no late revascularization occurred. No complications and no embolization of collaterals vessels were also recorded.
**Conclusion:** Phil® System is a valid alternative embolic material for the treatment of different peripheral pathologies. However, there are some disadvantages to consider, especially the high costs.
**Limitations:** Single center study and a small number of Patients enrolled.
**Ethics Committee Approval:** Study was approved by our institutional ethics committee.
**Funding for this study:** No funding has been received.
**Author Disclosures:**
Bianca Rocco: Nothing to disclose
Stefano Cipollari: Nothing to disclose
Mario Bezzi: Nothing to disclose
Mario Corona: Nothing to disclose
Pier Giorgio Nardis: Nothing to disclose
Alessandro Cannavale: Nothing to disclose
Leonardo Teodoli: Nothing to disclose
Pierleone Lucatelli: Nothing to disclose
Carlo Catalano: Nothing to disclose

### RPS 309-4

**Role of transcatheter endovascular embolization in non-operative management of high-grade blunt hepatic trauma and incidence of post-traumatic complications**
*A. Borzelli*, F. Pane, F. Amodio, F. Giurazza, F. Corvino, M. Silvestre, G. Cangiano, G. de Magistris, R. Niola; Naples/IT
*(antonio.borzelli@libero.it)*

**Purpose:** Evaluate feasibility, safety and clinical efficacy of selective hepatic artery endovascular embolization in hemodynamically stable patients with high grade liver blunt trauma(AAST>III)
**Methods or Background:** Between June 2017 and June 2020 we retrospectively considered 38 patients(24 M 14 F; medium age 38) with blunt trauma of abdomen and liver injury, all of them hemodynamically stable. All injuries have been studied by CT of the abdomen and classified considering AAST grades:(22)III,(11)IV,(5)V. We considered of each patient the percentage of hemoglobin value lowering, the presence of vascular lesions such as pseudoaneurysm and/or arterio-venous fistula, the entity of hemoperitoneum and the evidence of contrast blush on CT. All patients underwent selective endovascular embolization of hepatic artery and embolic agents used were coils, PVA and Gelfoam. We considered as success of procedures the absence of active bleeding at post-embolization angiogram and at follow-up CT of abdomen, with significant improvement of serum hemoglobin values, and as complications related to procedures the rebleeding rate, the incidence of hepatobiliary complications related to hepatic trauma during follow-up and the rate of eventual subsequent liver resections
**Results or Findings:** In 34 patients(90%) selective hepatic artery embolization was successful, with no evidence of rebleeding at follow-up and significant improvement of serum hemoglobin values, while 4 patients(10%)died during follow-up:2 of them for hemorrhagic shock after the embolization and the other 2 because of trauma complications of multiple systems.Among the 34 surviving patients,4 of them(12%)developed hepatobiliary complications:hepatic abscess in 2 patients, biloma in 1 and perihepatic fluid effusion in 1.All the hepatobiliary complications were successfully managed by image-guided percutaneous drainage; none of the patients underwent hepatic resection
**Conclusion:** Transcatheter endovascular embolization is a feasible, safe and clinical effective procedure in high grade blunt liver injuries, not only to stop bleedings, but also to reduce the rate of hepatic resections and hepatobiliary complications related to hepatic trauma
**Limitations:** Retrospective single center study
**Ethics Committee Approval:** Yes
**Funding for this study:** None
**Author Disclosures:**
Antonio Borzelli: Nothing to disclose
Francesco Amodio: Nothing to disclose
Fabio Corvino: Nothing to disclose
Francesco Pane: Nothing to disclose
Francesco Giurazza: Nothing to disclose
Mattia Silvestre: Nothing to disclose
Raffaella Niola: Nothing to disclose
Giuseppe de Magistris: Nothing to disclose
Gianluca Cangiano: Nothing to disclose

### RPS 309-6

**Transjugular intrahepatic portosystemic shunt placement with transsplenic balloon-puncture-technique in patients with portal vein thrombosis**

*T. C. Meine*, B. Maasoumy, E. Jaeckel, T. Werncke, S. K. Maschke, L. S. Becker, F. Wacker, B. C. Meyer, J. B. Hinrichs; Hannover/DE

**Purpose:** To evaluate the feasibility, safety and procedural characteristics of transjugular intrahepatic portosystemic shunt (TIPS) placement with transsplenic balloon-puncture-technique in patients with portal vein thrombosis (PVT).
**Methods or Background:** Eleven patients (7m,47yr) with PVT were referred for TIPS placement from the department of hepatology (8/17-4/20). TIPS placements were performed with an ultrasound-guided transsplenic access. Then, a balloon catheter was advanced via the splenic vein and dilated in an intrahepatic portal vein branch. The TIPS needle was inserted in the right hepatic vein via a transjugular access. When the balloon-puncture was performed, a guidewire was introduced through the TIPS needle and intercalated with the balloon cover. The balloon catheter was removed and the intercalated guide wire was captured at the transsplenic access site. After tract dilatation the TIPS stentgraft was placed. Transjugular access site was manually compressed. Transsplenic access site was closed with gelfoam. Demographics, technical success, clinical significant complications, transsplenic access time (TAT), procedure time (PT) and Dose-Area-Product (DAP) were recorded. Values were median(min-max).
**Results or Findings:** Patients with PVT had ethyltoxic cirrhosis (3), autoimmune hepatitis (2), essential thrombocythemia (1), congenital fibrosis (1), hepatitis B (1), primary (1) and secondary sclerosing cholangitis (1) and cryptogenic PVT (1). TIPS placement was impossible once, because no intrahepatic portal vein branch could be identified. Technical success was achieved in all other patients without clinically significant complications. TAT was 18min (11-90min), PT was 157min (98-268min) and DAP was 175Gy*m2 (6-436Gy*m2).
**Conclusion:** TIPS placement in patients with PVT via transsplenic access and balloon-puncture is time-intense, but feasible and safe.
**Limitations:** It is a retrospective study with a small number of patients and no control cohort (snare-puncture-technique).
**Ethics Committee Approval:** The institutional ethic's committee approved our study and a waiver for informed consent was given.
**Funding for this study:** None.
**Author Disclosures:**
Timo C. Meine: Nothing to disclose
Bernhard C Meyer: Nothing to disclose
Elmar Jaeckel: Nothing to disclose
Sabine Katharina Maschke: Nothing to disclose
Lena Sophie Becker: Nothing to disclose
Thomas Werncke: Nothing to disclose
Frank Wacker: Nothing to disclose
Benjamin Maasoumy: Nothing to disclose
Jan B Hinrichs: Nothing to disclose

### RPS 309-7

**Long-term results of intrasaccular flow-disrupting (WEB) devices in the treatment of intracranial wide-necked bifurcation aneurysms**

*T. Y. Kuzan*, F. Baltacioğlu; Istanbul/TR
*(tykuzan@gmail.com)*

**Purpose:** This unicentric study aims to evaluate the efficacy, safety, long-term results, and the effect of the aneurysm axis on the treatment response in patients treated with a Woven EndoBridge (WEB) device for intracranial wide-neck bifurcation aneurysm.
**Methods or Background:** Clinical, demographic, and digital subtraction angiography parameters of the patients treated with WEB device in our unit between January 2014 and February 2018 were evaluated retrospectively. The treatment response to the WEB device was assessed at different time points. The relationship between the aneurysm axis and parent artery was examined in predicting the WEB device treatment response.
**Results or Findings:** Twenty-five aneurysms of 22 patients were included in the study. Procedural peri-operative complications were observed in three of 25 aneurysms treated with the WEB device. No mortality was seen because of the aneurysm during the procedure and at two years of follow-up. Acute aneurysm occlusion was observed in five (20.0%) of 25 aneurysms in the peri-operative period. In the follow-up, an acceptable level of aneurysm occlusion was observed in 16 (66.7%) of 24 aneurysms at the sixth month, 17 (73.9%) of the 23 aneurysms at the 12th month, and 20 (87.0%) of the 23 aneurysms at the 24th month. No statistically significant relationship between the aneurysm axis and parent arteries in determining the treatment response was determined.
**Conclusion:** WEB devices have technical difficulties and rare complications. However, WEB devices provide effective and reliable endovascular treatment method with low complication and mortality rates for treating wide-necked bifurcation aneurysms that are complex and difficult to treat and have a high aneurysm occlusion rate.
**Limitations:** The sample size is relatively small.
**Ethics Committee Approval:** Approved by institutional IRB.
**Funding for this study:** No funding was received for this work. 7 aneurysms were treated within WEB-IT study.
**Author Disclosures:**
Taha Yusuf Yusuf Kuzan: Nothing to disclose
Feyyaz Baltacioğlu: Nothing to disclose

### RPS 309-8

**Depiction of mosaic perfusion in patients with chronic thromboembolic pulmonary hypertension (CTEPH) on C-arm computed tomography (CACT) compared to computed pulmonary angiogram (CTPA)**

*S. K. Maschke*, T. Werncke, C. Dewald, L. S. Becker, K. Olsson, M. Hoeper, F. Wacker, B. C. Meyer, J. B. Hinrichs; Hannover/DE

**Purpose:** To detect and distinguish different patterns of mosaic perfusion in patients with chronic thromboembolic pulmonary hypertension (CTEPH) on C-Arm computed tomography (CACT) compared to computed tomography pulmonary angiography (CTPA).
**Methods or Background:** We included 41 patients with confirmed CTEPH, undergoing CACT and CTPA within a maximum of 21 days. All datasets were independently evaluated by two readers (R1, R2) regarding mosaic perfusion patterns, presence of vascular lesions typical for CTEPH and the ratio of pulmonary arterial segments affected. Inter-observer agreement was calculated for both modalities using the intraclass-correlation-coefficient (ICC). A consensus reading was conducted afterwards and, again using the ICC, inter-modality agreement was calculated between both modalities (CACTcons vs. CTPAcons).
**Results or Findings:** On CACT and CTPA, inter-observer agreement was excellent for the perceptibility of mosaic perfusion (ICC=1), vascular lesions (ICC>0.8), the ratio of affected segments (ICC>0.7) and good for the attribution of the pattern of mosaic perfusion (ICC>0.5). Inter-modality agreement was calculated for CACTcons and CTPAcons. Inter-modality agreement was excellent for the perceptibility of mosaic perfusion (ICC=1), the present perfusion pattern (ICC=1) and vascular lesions (ICC=1). With a greater proportion of identified affected segments on CACTcons, inter-modality agreement for the ratio of affected segments was fair (ICC=0.50).
**Conclusion:** For detection and distinction of different mosaic perfusion patterns in patients with CTEPH, CACT and CTPA demonstrate high agreement.
**Limitations:** CACT and CTPA have not been acquired at the same day. CTPA has been acquired on different scanners and with slightly different protocols.
**Ethics Committee Approval:** Our local ethics committee approved this retrospective study.
**Funding for this study:** The study was funded in parts by personal grants from the “Junge Akademie”.
No grant or grant support.

**Author Disclosures:**
Karen Olsson: Nothing to disclose
Marius Hoeper: Nothing to disclose
Bernhard C Meyer: Nothing to disclose
Cornelia Dewald: Nothing to disclose
Sabine Katharina Maschke: Nothing to disclose
Lena Sophie Becker: Nothing to disclose
Thomas Werncke: Nothing to disclose
Frank Wacker: Nothing to disclose
Jan B Hinrichs: Nothing to disclose

### RPS 309-9

**Mechanical thrombectomy for acute ischemic stroke in the posterior circulation: assessment of efficacy and outcome and identification of prognostic factors**

*M. Szmygin*, M. Sojka, K. K. Pyra, P. Tarkowski, P. Luchowski, A. Drelich-Zbroja, T. Jargiełło; Lublin/PL
*(mszmygin@gmail.com)*

**Purpose:** Mechanical thrombectomy (MT) is well-established in the treatment of acute ischemic anterior circulation stroke. However, there is no evidence from randomized trials or meta-analyses that MT is safe and effective in the treatment of patients with acute ischemic posterior circulation stroke (PCS). The aim of this study was to evaluate the clinical and procedural factors associated with recanalization and outcome of patients with PCS treated with MT.
**Methods or Background:** 43 patients with PCS treated with MT were included. Data including demographics, baseline stroke severity, radiological imaging, procedure and post-procedure complications were documented. Clinical outcome was evaluated using the modified Rankin Scale (mRS).
**Results or Findings:** Median baseline National Institute of Health Stroke Scale (NIHSS) was 17. Successful recanalization was observed in 88.4% of patients. After 90 days, favorable outcome (defined as mRS 0–2) was achieved in 26 patients; six patients had an unfavorable outcome (mRs >2). Final mortality rate was 25.5%. Baseline NIHSS, onset to reperfusion time, procedure duration, and successful recanalization had a statistically significant association with outcome. Failed recanalization and occurrence of intracranial hemorrhage were found to be associated with a higher mortality rate.
**Conclusion:** MT is feasible and effective method in treatment of PCS. Baseline NIHSS and onset to reperfusion time were found to be independent predictive factors of clinical outcome.
**Limitations:** The present study has some limitations: small sample size; retrospective study design; and single-center study. There was also no unified scoring of brainstem infarction and penumbra area conducted from diagnostic imaging (e.g. posterior circulation Acute Stroke Prognosis Early CT Score [pcASPECTS]).
**Ethics Committee Approval:** This study was approved by Institutional Review Board (IRB)
**Funding for this study:** None
**Author Disclosures:**
Maciej Szmygin: Nothing to disclose
Tomasz Jargiełło: Nothing to disclose
Michał Sojka: Nothing to disclose
Piotr Tarkowski: Nothing to disclose
Piotr Luchowski: Nothing to disclose
Anna Drelich-Zbroja: Nothing to disclose
Krzysztof K. Pyra: Nothing to disclose

### RPS 309-10

**SAFARI technique for limb rescue in diabetic patients with CLI: a single center experience**

F. Pane[1], *A. Borzelli*[1], G. Cangiano[1], M. Silvestre[1], M. Coppola[1], A. Paladini[2], F. Corvino[1], F. Giurazza[1], R. Niola[1]; [1]Naples/IT, [2]Novara/IT
*(antonio.borzelli@libero.it)*

**Purpose:** Evaluate the clinical and technical efficacy of retrograde arterial access for endovascular revascularization of chronic occlusions not passable by antegrade access in patients with critical limb ischemia
**Methods or Background:** We retrospectively considered between January 2017 and April 2020 32 patients (medium age 70,4 years-old, 20 M 12 F)with critical limb ischemia and trophic lesions of lower limb. All patients underwent diagnostic angiography which showed, together with the localization of the trophic lesions, according to angiosomic distribution map, the target vessels. Retrograde access was performed employing micropuncture kit under combined ultrasonographic and fluoroscopic guidance and corroboration. After arterial retrograde access was realized, the target vessel was recanalized employing .014" guidewires and angioplasty performed; if necessary, stenting of target vessels was performed too. Technical success was considered as successful recanalization of target vessels at final angiographic control while clinical success as major amputation avoided at 6 months follow-up
**Results or Findings:** Target vessels for retrograde arterial access were:12 pedidia(38%),6 anterior tibial(18%),6 popliteal(18%),4 posterior tibial(12%),2 tibio-peroneal trunk(7%),2 peroneal(7%).In 8 patients stenting of superficial femoral artery/popliteal was performed.Technical success was reached in 28 patients(87%),clinical success in 26 patients(81%).At 6 months follow-up 1 major amputation and 5 minor amputations (3 trans-metatarsal and 2 toes) were performed;1 patient died for other comorbidities
**Conclusion:** Retrograde arterial endovascular access proved to be safe,feasible and clinical effective in diabetic patients with critical limb ischemia, as it allows significant lowering of major amputations rates with low major complications rates associated to the procedure
**Limitations:** Retrospective single center study
**Ethics Committee Approval:** Yes
**Funding for this study:** None
**Author Disclosures:**
Antonio Borzelli: Nothing to disclose
Fabio Corvino: Nothing to disclose
Francesco Pane: Nothing to disclose
Milena Coppola: Nothing to disclose
Andrea Paladini: Nothing to disclose
Francesco Giurazza: Nothing to disclose
Mattia Silvestre: Nothing to disclose
Raffaella Niola: Nothing to disclose
Gianluca Cangiano: Nothing to disclose

### RPS 309-11

**Maternal and neonatal outcome in patients with placenta previa undergoing prophylactic internal iliac arteries balloons placement prior to cesarean section**

K. K. Pyra, *M. Szmygin*, W. Dymara-Konopka, G. Pietras, M. Sojka, T. Jargiełło, B. Leszczyńska-Gorzelak; Lublin/PL
*(mszmygin@gmail.com)*

**Purpose:** Placenta previa (PP) describes an implantation abnormality with placenta implanted in the lower segment of the uterus rather than in the fundus. The aim of this study was to summarize our experience with patients diagnosed with PP and treated with prophylactic internal iliac balloon implantation prior to cesarean section and to assess maternal and fetal outcomes.
**Methods or Background:** In this retrospective, single-center study we investigated the medical records of patients with PP who underwent prophylactic internal iliac balloon implantation prior to cesarean section. Clinical information including age, history of deliveries/cesarean sections and risk factors was collected.
**Results or Findings:** A total of 30 patients were included in the study. Hysterectomy was performed in 10 cases. Median estimated blood loss (EBL) was 1.18 L. A total of 30 live infants were delivered. Mean birth weight was 2435 g. In 2 cases (7%) technical complications occurred. In 1 case (3%) bilateral common iliac thrombosis requiring urgent surgical intervention occurred. Control angiography disclosed minor post-cesarean contrast extravasation in 3 cases (10%). In all 3 patients successful embolization of uterine arteries was performed with gelatine sponge powder
**Conclusion:** Prophylactic placement of internal iliac balloons in patients with placenta previa prior to cesarean section is an effective and minimally invasive procedure that results in lowering the blood loss. Moreover, it is safe for both mother and the neonate.
**Limitations:** Our study has several limitations - its retrospective and single-center nature, limited number of participants and no control arm (patients with PP without prophylactic balloon placement) are the most important ones.
**Ethics Committee Approval:** This study was approved by IRB.
**Funding for this study:** None.
**Author Disclosures:**
Weronika Dymara-Konopka: Nothing to disclose
Maciej Szmygin: Nothing to disclose
Tomasz Jargiełło: Nothing to disclose
Michał Sojka: Nothing to disclose
Bożena Leszczyńska-Gorzelak: Nothing to disclose
Grzegorz Pietras: Nothing to disclose
Krzysztof K. Pyra: Nothing to disclose

### RPS 309-13

**Analysis of traumatic carotid artery injuries: clinical predictors of management and outcomes**

*R. T. Tomihama*, H. Boggs, D. Turay, K. Mukherjee, S. Kiang; Loma Linda, CA/US
*(rtomihama@llu.edu)*

**Purpose:** Optimal management of traumatic carotid artery injury (CAI) to preserve neurologic function remains undefined. We sought to evaluate the factors that influence management, stroke and brain death outcomes of traumatic CAI.
**Methods or Background:** A search of the PROOVIT Trauma Registry of patients >18 years of age with a diagnosis of CAI was performed at a Level 1 Trauma Center from 2013-2019. Patient demographics, injuries, the timing of presentation, Biffl Cerebral Injury Grade, Injury Severity Score (ISS), Abbreviated Injury Scale (AIS) were documented. Medical management, procedures, and brain-related outcomes were recorded.
**Results or Findings:** Carotid artery injuries were identified in 30 patients. There were 25 (83.3%) blunt and 5 (16.7%) penetrating carotid injuries. While a higher Biffl grade was predictive of vascular intervention over medical management (3.6 vs. 2.4; p = 0.03, respectively), the ISS and AIS scores were not. A higher ISS score was a predictor of stroke (33.33 vs. 22.86; p = 0.012) while Biffle grade and AIS scores were not. All stroke patients declared their neurological change on average within 11.7 hours (SD±12.7) of the presentation. In addition, a higher ISS score was predictive of brain death (38.6 vs 23.6; p = 0.004) but Biffl grade and AIS scores were not. There was a trend of eventual brain death seen in blunt trauma over penetrating trauma (36% vs 0%, p = 0.11).
**Conclusion:** Analysis of carotid artery injuries revealed certain predictors of management and clinical outcomes. A higher Biffl grade can help predict vascular intervention while a higher ISS score can help predict stroke and brain death.
**Limitations:** Given the rarer disease process, study numbers are small. Long term clinical follow up is limited
**Ethics Committee Approval:** IRB approval was waived due registry study data analysis
**Funding for this study:** None
**Author Disclosures:**
Hans Boggs: Nothing to disclose
Sharon Kiang: Nothing to disclose
Roger Takeshi Tomihama: Nothing to disclose
Kaushik Mukherjee: Nothing to disclose
David Turay: Nothing to disclose

### RPS 309-14

### Prostatic artery embolisation in benign prostatic hyperplasia: a single-centre retrospective study

*T. J. Vogl*, A. Zinn, E. Elhawash, L. S. Alizadeh, N-E. A. N-E. Mohammed, N. N. N. Naguib; Frankfurt/DE
*(t.vogl@em.uni-frankfurt.de)*

**Purpose:** To evaluate the advantages of MR angiography (MRA)-planned prostatic artery embolization (PAE) in benign prostatic hyperplasia (BPH).
**Methods or Background:** In this retrospective study MRAs of 56 patients (range: 47-82 years; mean: 67.23 ± 7.73 years) with a PAE were evaluated between 2017 and 2018. For inclusion, full information on procedure time and radiation values was necessary. In every patient 3-dimensional MRA reconstruction with maximum intensity projection was conducted in order to identify prostatic artery (PA) origin. 33 patients completed clinical and imaging follow up and were included in the clinical evaluation.
**Results or Findings:** In total there were 131 PAs with a second PA in 19 pelvic sides. PA origin was correctly identified in 108 of 131 PAs (82.44%) via MRA. In patients in which MRA allowed a PA analysis, a significant reduction of the fluoroscopy time (-26.96%, p=0.028) and of the dose area product (-38.04%, p=0.003) were detected versus those with no PA analysis prior to PAE. Intervention time was reduced by 13.15%, (p=0.249). Mean fluoroscopy time was 30.14 minutes, mean dose area product 27,748.76 µGym2 and mean entrance dose 1,553.48 mGy. Technical success was achieved in all 56 patients (100.0%); all patients were embolized on both pelvic sides. The evaluated data documented a significant reduction in IPSS (p<0.0001; mean 9.67 points).
**Conclusion:** MRA prior to PAE successfully identified PAs in 82.44% of the cases. MRA-planned PAE is an effective treatment for patients with BPH.
**Limitations:** - small sample size - retrospective nature of the study.
- only the origin of PA was analyzed and no precise analysis of the intraprostatic distribution pattern was documented
**Ethics Committee Approval:** This retrospective single-center study was approved by the ethical committee
**Funding for this study:** No funding
**Author Disclosures:**
Annette Zinn: Nothing to disclose
Nagy Naguib Naeem Naguib: Nothing to disclose
Thomas J. Vogl: Nothing to disclose
Nour-Eldin Abdelrehim Nour-Eldin Mohammed: Nothing to disclose
Elsayed Elhawash: Nothing to disclose
Leona Soraja Alizadeh: Nothing to disclose

### RPS 309-15

### Predictors of outcome after mechanical thrombectomy for acute ischaemic stroke in patients aged ≥90 years

M. Sojka, *M. Szmygin*, K. K. Pyra, P. Tarkowski, J. Wojczal, A. Drelich-Zbroja, T. Jargiełło; Lublin/PL
*(mszmygin@gmail.com)*

**Purpose:** Mechanical thrombectomy (MT) has established its role as a first-line treatment of acute ischemic stroke due to large vessel occlusions (LVO). However, patients older than 85 or even 80 are commonly excluded from large randomized controlled studies as this group was found to be associated with significantly poorer clinical outcome and increased mortality compared to younger patients. The aim of this study was to evaluate clinical and procedural factors associated with clinical outcome and mortality among nonagenarians with acute ischemic stroke treated with MT.
**Methods or Background:** This retrospective, single-center study was conducted on 38 patients with LVO treated with MT. Clinical features including baseline results, radiological imaging, procedural details and outcome results were documented and evaluated. Recanalization was assessed according to the TICI score. The clinical condition was evaluated on admission (NIHSS) and after 3 months (mRS).
**Results or Findings:** The rate of successful recanalization (TICI ≥2b) was 84.2% (32/38). Symptomatic intracranial hemorrhage was observed in 3 (7.9%) patients. After 90 days, the mortality rate was 47.4%. Favorable clinical outcome (mRs 0-2) was regained in 28.9% of the patients (11/38). Poor clinical outcome (mRs>2) was observed in 9 patients (23.7%).
**Conclusion:** Very elderly patients with LVO should not be excluded from MT even if prognosis for good clinical outcome in this age group remains low and the procedure is more challenging. Long-term outcome is predicted by stroke severity (baseline NIHSS and occluded vessel) and hospital arrival time.
**Limitations:** Firstly, retrospective design and relatively small number of nonagenarians that limits the validity of the data. Secondly, initial ASPECTS and final infarct volume were not calculated. Finally, lack of control arm consisting of patients treated with intravenous thrombolysis for direct comparison.
**Ethics Committee Approval:** Study was approved by institutional review board.
**Funding for this study:** None
**Author Disclosures:**
Maciej Szmygin: Nothing to disclose
Tomasz Jargiełło: Nothing to disclose
Michał Sojka: Nothing to disclose
Joanna Wojczal: Nothing to disclose
Piotr Tarkowski: Nothing to disclose
Anna Drelich-Zbroja: Nothing to disclose
Krzysztof K. Pyra: Nothing to disclose

### RPS 309-16

### Impact of the laboratory results on cerebral vasospasm and delayed cerebral ischaemia in aneurysmal subarachnoid haemorrhage

*V. Opancina*, R. Vojinovic, S. Jankovic; Kragujevac/RS
*(valentina.opancina@gmail.com)*

**Purpose:** The main objective of this study was to examine the laboratory values associated with an increased or decreased risk of cerebral vasospasm and delayed cerebral ischemia, which develop after aneurysmal subarachnoid hemorrhage.
**Methods or Background:** The study was designed as a cross-sectional study. The study included all patients, 18 or more years of age, who were diagnosed for the first time with aneurysmal subarachnoid hemorrhage detected on CT scans and who were treated with endovascular embolization of the aneurysm, at our institution, from January 2014 until January 2019. The following variables were examined: age, gender, Glasgow Coma Scale score and laboratory analysis (maximum recorded values and nadir values): blood count, coagulation tests and biochemistry analysis. The impact of these variables on the main outcomes (cerebral vasospasm, delayed cerebral ischemia) was investigated by univariate and multivariate logistic regression.
**Results or Findings:** In total, 66 patients were enrolled. The multivariate analysis showed that the maximum recorded INR values, in patients not receiving anticoagulant therapy and the maximum recorded white blood cells, were strongly associated with cerebrovascular spasm, increasing its chances by 4.4 and 8.4 times. In addition, maximum recorded values of the white blood cells were associated with delayed cerebral ischemia, increasing its chances by 1.4 times.
**Conclusion:** It was determined that aneurysmal subarachnoid hemorrhage creates an endocranial inflammatory condition, whose intensity is probably directly related to the formation of vasospasm and delayed ischemia. These results might have value in potential pharmacological interference for neuroinflammation as a useful strategy for the prevention of vasospasm and delayed cerebral ischemia.
**Limitations:** Not applicable

**Ethics Committee Approval:** The study was approved by the Ethics Committee of the Clinical Center Kragujevac before any of the study procedures was initiated.
**Funding for this study:** Not applicable
**Author Disclosures:**
Valentina Opancina: Nothing to disclose
Slobodan Jankovic: Nothing to disclose
Radisa Vojinovic: Nothing to disclose

### RPS 309-18

**Intravascular embolisation in four cases of intralobar pulmonary sequestration: a new treatment perspective?**

A. Paladini[1], *A. Borzelli*[2], S. Tricca[1], D. Zagaria[1], S. Bor[1], F. Pane[1], A. Carriero[1]; [1]Novara/IT, [2]Naples/IT
*(antonio.borzelli@libero.it)*

**Purpose:** To evaluate endovascular embolic treatment as an alternative therapeutic option to surgery in patients suffering from intralobar pulmonary sequestration.
**Methods or Background:** Four symptomatic patients (two with hemoptysis and two with recurrent inflammatory events) with intralobar pulmonary sequestration were treated and a systematic review of the literature on this congenital anomaly was performed. The embolization of the aberrant arteries occurred in two cases by means of metal spirals and in two cases by a liquid embolizing agent (Easyx).
**Results or Findings:** The procedure was shown to be effective in selectively reducing arterial inflow to pathological lung tissue, causing necrosis and progressive involutionary fibrosis. The intra and periprocedural complications were analyzed, as well as the long-term results obtained with clinical and radiological evaluation (TC at 1 and 6 months later). The only complication found was reported by patients treated with embolic fluid, such as a pleuritic-like pain that is limited over time. Embolization is therefore a less invasive and safer therapeutic alternative than surgical resection where morbidity and complications are greater.
**Conclusion:** Endovascular treatment has been shown to be a valid and effective therapy in patients suffering from intralobar pulmonary seizure, a result that is in line with the current literature
**Limitations:** Poor cohort of Patients
**Ethics Committee Approval:** Not requested
**Funding for this study:** None
**Author Disclosures:**
Antonio Borzelli: Nothing to disclose
Francesco Pane: Nothing to disclose
Simone Bor: Nothing to disclose
Andrea Paladini: Nothing to disclose
Alessandro Carriero: Nothing to disclose
Stefano Tricca: Nothing to disclose
Domenico Zagaria: Nothing to disclose

### RPS 309-19

**Successful endovascular embolisation of active lumbar arteries bleedings in patients with blunt trauma and huge retroperitoneal haematoma**

*A. Borzelli*[1], F. Pane[1], F. Amodio[1], M. Silvestre[1], M. Coppola[1], F. Giurazza[1], A. Paladini[2], F. Corvino[1], R. Niola[1]; [1]Naples/IT, [2]Novara/IT
*(antonio.borzelli@libero.it)*

**Purpose:** Evaluate efficacy and safety of transcatheter endovascular embolization in lumbar arteries active bleedings
**Methods or Background:** Between January 2015 and January 2020 we retrospectively considered 62 patients(37 M 25 F; medium age 43 anni) affected by huge retroperitoneal hematoma due to active arterial bleedings for blunt trauma and with clinical and laboratoristic evidence of serum hemoglobin value lowering. All patients undergone preliminar CT study evaluation followed by angiographic study and endovascular transcatheter embolization. In all cases we employed 5 Fr catheters for angiographic initial evaluation of gluteal arterial district and 2,7 Ft microcatheters for selective and super-selective distal branches of lumbar arteries catheterization. We employed as embolizing agents: metallic coils, PVA, cyanoacrylate and Spongostan, alone or in association
**Results or Findings:** In all cases the endovascular embolization was successful with a complete stop of bleedings; 9 cases required a second procedure due to recidivant bleedings. In none of the cases we observed major complications related to the procedures.
**Conclusion:** The elevated rate of technical and clinical success reported and the absence of major complications suggest that endovascular transcatheter embolization is a safe and effective minimally invasive treatment for lumbar arteries active bleedings as an alternative to surgery, expecially in very old patients, affected by many other comorbidities. It is fundamental, however,an adequate pre-treatment non-invasive imaging by CT-angiography, to correctly identify all the sources of bleedings, even the minimal, but potentially fatal, ones, to perform a more precise and faster embolization.
**Limitations:** Restrospective and monocentric study
**Ethics Committee Approval:** Yes
**Funding for this study:** None
**Author Disclosures:**
Antonio Borzelli: Nothing to disclose
Francesco Amodio: Nothing to disclose
Fabio Corvino: Nothing to disclose
Francesco Pane: Nothing to disclose
Milena Coppola: Nothing to disclose
Andrea Paladini: Nothing to disclose
Francesco Giurazza: Nothing to disclose
Mattia Silvestre: Nothing to disclose
Raffaella Niola: Nothing to disclose

### RPS 309-20

**The prelude-BTK study: novel serration angioplasty balloon for below-the-knee arteries**

*C. Wissgott*; Rendsburg/DE
*(cwissgott@gmx.de)*

**Purpose:** PRELUDE-BTK assessed safety and efficacy of the Serranator® PTA Serration Balloon Catheter in subjects with atherosclerotic disease of the BTK arteries.
**Methods or Background:** PRELUDE-BTK is a single arm, prospective, multi-center feasibility study to assess Serranator in subjects with lesions in BTK arteries. Acute angiographic data captured pre-Serranator® inflation vs. post inflation effects with 30 day and 6 month follow up. Subjects had CLTI or severe claudication, de novo stenoses or non-stented restenotic or CTOs with lengths <12cm and RVD of 2.5 - 3.5mm. Safety was a composite of MALE and POD, assessed at 30 days. Efficacy was rate of device success with final diameter stenosis <50% by visual assessment using only the Serranator. A secondary objective assessed OCT or IVUS pre- and post-treatment in up to 10 subjects to evaluate lesion characteristics and presence of serrations.
**Results or Findings:** Forty-nine subjects enrolled treating 55 lesions, 53 analyzable. Mean pre-treatment diameter stenosis was 81.3% +/- 17.2% versus post treatment of 21.8% +/- 12.7%, with 28.3% CTOs (n=15/53). Twenty six percent (n=14/53) had moderate/severe calcification. One flow limiting dissection resulted in a 1.9% (1/53) bailout stent rate. All OCT/IVUS imaged lesions showed serration effects (n=10). There were no CD TLRs at 30-days. Thirty day and 6-month data to be presented.
**Conclusion:** Acute study endpoints were achieved. PRELUDE-BTK showed exceptional lumen gain in challenging infrapopliteal lesions while minimizing arterial injury. Serranator's mechanism of action was demonstrated by OCT and IVUS showing serrations without significant dissection, allowing for an increase in lumen area.
**Limitations:** Only short-term results yet available.
**Ethics Committee Approval:** Yes.
**Funding for this study:** Sponsored by Cagent Vascular.
**Author Disclosures:**
Christian Wissgott: Research/Grant Support: Cagent Vascular. Investigator: na

### RPS 309-21

**Focal dissection repair after DCB angioplasty: 12- and 24-month results of Tack-Optimised Balloon Angioplasty (TOBA) III**

*C. Wissgott*; Rendsburg/DE
*(cwissgott@gmx.de)*

**Purpose:** TOBA III evaluated a novel dissection repair device for treating post-PTA dissection in the superficial femoral (SFA) and/or proximal popliteal (PPA) arteries.
**Methods or Background:** The prospective clinical study enrolled 201 patients: 169 patients with lesions ≤150mm and a subset of 32 patients with lesions >150-250mm. Eligibility included Rutherford classification 2-4 with de novo or non-stented restenotic target SFA/PPA lesion(s) undergoing drug-coated balloon (DCB) angioplasty. After DCB angioplasty, lesions with <30% residual stenosis and presence of ≥1 dissection were enrolled and treated. The system consists of six self-expanding nitinol implants on a 6F catheter. Each implant is 6mm in length, self-sizes to vessels 2.5-6.0mm in diameter. The 12-month endpoint were primary patency—freedom from binary restenosis and clinically-driven target lesion revascularization (CD-TLR). All imaging was adjudicated by core laboratories.
**Results or Findings:** In ≤150mm lesions, the mean lesion length was 68±42mm. The safety endpoint was met with zero major adverse events at 30 days (P<0.0001). The 12-month efficacy endpoint was also met (P<0.0001). 12-month K-M primary patency and freedom from CD-TLR were 95.0% and 97.5% respectively, with 97.7% of dissections resolved. Bailout stent in 1 (0.6%) case. In the longer lesion subset, mean lesion length was 154±56mm. In this group, 12-month K-M primary patency and freedom from CD-TLR were

89.3% and 96.8% respectively, with 98.8% of all dissections resolved. In both groups, K-M freedom from CD-TLR was sustained to 24 months at 92.3% and 82.6% for lesions ≤150mm and lesions >150-250mm respectively.
**Conclusion:** The TOBA III study met its primary endpoints in a 100% dissected vessel population and demonstrated primary patency of 95.0% at 12 months for lesions up to 15cm and 89.3% for lesions 15-25cm with sustained freedom from CD-TLR.
**Limitations:** Non-randomized trial.
**Ethics Committee Approval:** Yes.
**Funding for this study:** Sponsored by Intact Vascular.
**Author Disclosures:**
Christian Wissgott: Investigator: Intact Vascular

### RPS 309-22

**COVID-19 pandemic prompts a paradigm shift to remote training of residents via cloud-based e-simulation platform**

*G. Bartal*, S. Fargun, T. Avziz, A. Ben-Moshe, E. Yechezkel, A. Shacharit; Tel Aviv/IL
*(gbartal@gmail.com)*

**Purpose:** WHO declaration of COVID-19 Pandemic lead to the “social distancing” and prevents in-person residency academic activities. Currently, clinical education and hands-on training can be achieved only remotely and virtually. Residency programs seek an alternative way to carry out training, to achieve goals of curriculum and to complete their training program. Our aim was to develop and evaluate performance of an interactive cloud-based e-simulation platform for remote simulation and training of endovascular interventions.
**Methods or Background:** E-simulation, interactive cloud-based platform was developed allowing remote simulation for learning and practicing steps of various endovascular procedures. The desktop software was converted to a cloud service and enables trainee to remotely connect with the simulation session using personal computer. The platform allows step-by-step practice of complete endovascular procedure using standard keyboard and mouse. The virtual reality simulation includes the fluoroscopic screen, vital signs monitor, selection of endovascular tools (wire, catheters, balloons, stent/grafts) and contrast injection thus creating an entire procedure flow in a variety of endovascular techniques and patient anatomies.
**Results or Findings:** The cloud-based software comprises entire simulation package besides the physical tool manipulation and deployment, all replaced by PC controls. The system provides cognitive training of every procedure step and prepares to future hands-on simulator training during which the trainee can also practice manipulating real tools. Furthermore, e-simulation can be used for remote demonstration and training on new devices. The simulation can be practiced under real-time remote supervision which allows evaluation of performance.
**Conclusion:** Remote resident training via cloud-based e-simulation platform is timely and feasible. It allows the physician to practice different patient scenarios and simulate a full procedure experience based on the patient file. Evaluation by physicians using validated questionnaires is underway.
**Limitations:** Laboratory evaluation
**Ethics Committee Approval:** Not required
**Funding for this study:** Not required
**Author Disclosures:**
Elad Yechezkel: Employee: Simbionix 3DSysteHealthcare
Shani Fargun: Employee: Simbionix 3DSysteHealthcare
Ariel Ben-Moshe: Employee: Simbionix 3DSysteHealthcare
Alex Shacharit: Employee: Simbionix 3DSysteHealthcare
Gabriel Bartal: Consultant: Simbionix 3DSystems
Tal Avziz: Employee: Simbionix 3DSysteHealthcare

### RPS 309-23

**Saving the uterus: our experience in uterine artery embolisation for acquired uterine vascular abnormalities**

*L. Masood*, A. Rana, M. S. Ahmed, S. Haider, Z. A. Khan; Islamabad/PK
*(laibamasood66@gmail.com)*

**Purpose:** The aim of this study was to describe our experience with the diagnosis and management of acquired uterine vascular abnormalities with uterine preservation.
**Methods or Background:** 5 patients were enrolled from the hospital database who presented to our Interventional Radiology department for uterine artery embolization (UAE) with a history of iatrogenic/acquired uterine vascular abnormalities confirmed on imaging (doppler ultrasound, CT, or MRI). These included 1 patient with uterine artery pseudoaneurysm and arteriovenous fistula (AVF), 2 having uterine arteriovenous malformations (AVMs), and 2 patients with uterine AVFs. Embolization agents used were histoacryl glue mixed with lipodol, PVA particles, and gelfoam slurry. Medical records, imaging studies, and telephonic contact with patients were assessed for patient presentation, intraprocedural details, and follow up to record treatment success. Statistical analysis was performed using descriptive statistics.
**Results or Findings:** A total of 5 bilateral UAE were performed in 5 patients ranging between 28 to 33 years of age. Three of the patients presented with life-threatening bleed requiring 2 – 3 transfusions. Clinical as well as angiographic success was achieved in all patients with immediate control of hemorrhage. No complications were identified during follow-up. Restoration of normal menstrual cycle was observed in all patients after 2- 3 months. Two of the patients were able to conceive normally within one year.
**Conclusion:** Acquired/iatrogenic uterine vascular abnormalities are a rare but important cause of life-threatening hemorrhage which can be expertly managed and successfully treated using UAE, which is rapid, safe, and minimally invasive with the added advantage of fertility preservation.
**Limitations:** This is a single-center study having a limited number of patients and a lack of follow-up on imaging.
**Ethics Committee Approval:** Study was performed after approval from hospital institution review board.
**Funding for this study:** No funding.
**Author Disclosures:**
Muhammed Shozab Ahmed: Nothing to disclose
Syed Haider: Nothing to disclose
Laiba Masood: Nothing to disclose
Atif Rana: Nothing to disclose
Zahid A Khan: Nothing to disclose

### Research Presentation Session: Interventional Radiology

### RPS 409

### Advances in non-vascular interventional radiology

### RPS 409-1

**National dose reference levels in computed tomography–guided interventional procedures: a proposal**

*J. Greffier*, D. Dabli, J-P. Beregi, J. Frandon; Nimes/FR
*(joel.greffier@chu-nimes.fr)*

**Purpose:** To establish national reference levels (RLs) in interventional procedures under CT guidance as required by the 2013/59/Euratom European Directive.
**Methods or Background:** Seventeen categories of interventional procedures in thoracic, abdominopelvic, and osteoarticular specialties (percutaneous infiltration, vertebroplasty, biopsy, drainage, tumor destruction) were analyzed. Total dose length product (DLP), number of helical acquisitions (NH), and total DLP for helical, sequential, or fluoroscopic acquisitions were recorded for 10 to 20 patients per procedure at each center. RLs were calculated as the 3rd quartiles of the distributions and target values for optimization process (TVOs) as the median. RLs and TVOs were compared with previously published studies.
**Results or Findings:** Results on 5001 procedures from 49 centers confirmed the great variability in patient dose for the same category of procedures. RLs were proposed for the DLPs and NHs in the seventeen categories. RLs in terms of DLP and NH were 375 mGy.cm and 2 NH for spinal or peri-spinal infiltration, 1630 mGy.cm and 3 NH for vertebroplasty, 845 mGy.cm and 4 NH for biopsy, 1950 mGy.cm and 8 NH for destruction of tumors, and 1090 mGy.cm and 5 NH for drainage. DLP and NH increased with the complexity of procedures.
**Conclusion:** This study was the first nationwide multicentric survey to propose RLs for interventional procedures under CT guidance. Heterogeneity of practice in centers were found with different levels of patient doses for the same procedure. The proposed RLs will allow imaging departments to benchmark their practice with others and optimize their protocols.
**Limitations:** The main limitation of our study is the non-consideration of the body habitus.
**Ethics Committee Approval:** Institutional Review Board approval was obtained.
**Funding for this study:** No
**Author Disclosures:**
Jean-Paul Beregi: Nothing to disclose
Julien Frandon: Nothing to disclose
Joel Greffier: Nothing to disclose
Djamel Dabli: Nothing to disclose

### RPS 409-2

**Safety and efficacy of a new percutaneous interspinous spacer: a multicentre retrospective study**

C. Zini[1], M. Bellini[2], E. Piras[3], L. Manfré[4], A. E. De Vivo[4], *S. Marcia*[3]; [1]Firenze/IT, [2]Siena/IT, [3]Cagliari/IT, [4]Catania/IT
*(stemarcia@gmail.com)*

**Purpose:** To evaluate safety and efficacy of a new percutaneous interspinous process device (IPD) Lobster® (Techlamed, Firenze, Italy) in 147 patients with degenerative lumbar spinal stenosis(DLSS) from 3 different centers.
**Methods or Background:** From November 2016 and March 2020, 147 patients (67 male, age 72 ± 9.7 years old, range 45-91) with neurogenic intermittent claudication because of DLSS related to mono/bi-segmental lumbar central canal and/or foraminal stenosis were enrolled in the present study. Clinical outcomes were evaluated using Visual Analog Scale(VAS) and Zurich Claudication Questionnaire (ZCQ) before and after the procedure. Technical success was defined as correct placement of the IPD demonstrated with computer tomography (CT), performed immediately after treatment; spinoplasty was accomplished in patients with BMD T-score<- 2.5.
**Results or Findings:** In 178 levels (L2-L3=7; L3-L4=61; L4-L5=102; L5-S1=8) a total of 177 IPDs (8mm= 60;10 mm=90;12 mm=26;14 mm=1) were implanted; in 22 patients more than 1 level have been treated in the same session; spinoplasty was accomplished in 66 patients. We registered 99,4% of technical success: in 1 case, because of spinous process rupture during spinoplasty, the device was not implanted. In 2 cases we observed displacement of the IPD subsequently removed under fluoroscopy and replaced. No major complications were registered. 5 point of VAS reduction was registered after the procedure(mean VAS pre-procedure=7.74±0.69; mean VAS post-procedure=3.36±1.3)(p<0.001); mean pre-procedural ZCQ was 50.6±10.67 and post-procedural ZCQ was 30±1.3(p<0.001).
**Conclusion:** The Lobster®IPD is safe and effective totally percutaneous minimally-invasive decompression procedure in DLSS.
**Limitations:** Number of patients
3 National Centres
**Ethics Committee Approval:** The present study has been approved by ATS Sardegna Ethical Committee (231/2020/CE)
**Funding for this study:** No profit observational study
**Author Disclosures:**
Emanuele Piras: Nothing to disclose
Matteo Bellini: Nothing to disclose
Stefano Marcia: Nothing to disclose
Chiara Zini: Nothing to disclose
Aldo Eros De Vivo: Nothing to disclose
Luigi Manfré: Nothing to disclose

### RPS 409-3

**Percutaneous management of benign biliary strictures (BBS) after liver transplantation (LT) in paediatric patients: a singlecentre experience**

*L. Dulcetta*[1], P. Marra[2], F. S. Carbone[1], C. Sallemi[2], P. A. Bonaffini[2], S. Sironi[1]; [1]Monza/IT, [2]Bergamo/IT
*(l.dulcetta@campus.unimib.it)*

**Purpose:** To assess the role of percutaneous transhepatic cholangiography (PTC) for diagnosis confirmation and treatment of BBS after LT in pediatric patients. To evaluate technical success and short-term clinical outcome.
**Methods or Background:** Clinical, laboratory, imaging and procedural data of 52 pediatric patients (mean age 22.1 months; range 1-128), who underwent PTC after LT between 2009 and 2020 in a single center, were retrospectively reviewed. Indication to PTC was clinical suspicious of BBS based on laboratory and liver biopsy data, regardless bile ducts dilation at pre-procedural imaging.
**Results or Findings:** 122 PTCs were performed, with 2.3 procedures for each patient on average (range 1-8). Technical success of intrahepatic biliary catheterization was 99.2%; one failed catheterization was repeated successfully after 7 days. In 108/121 cases (89.2%) ductal or biliodigestive anastomosis stenosis was found at PTC. In 95/108 cases (87.9%) stenosis was treated with transluminal bilioplasty and insertion of an internal-external biliary drainage (IEBD). In 10/108 the stenosis was managed in a second attempt. The remaining 3 cases failed all attempts of bilioplasty and underwent surgery. In 13/121 cases without evident stenosis at PTC only an IEBD was placed. Procedure-related complications were: 35 cholangitis (within 72 hours of procedure), 4 bilomas, 3 sepsis and 1 septic shock. Liver function tests (compared before, 7 days and 1 month after PTC) were available for 58 procedures. Reduction in cholestasis was indicated by significantly improving in serum levels of γ-glutamyl transpeptidase ($p < .001$), total bilirubin ($p < .005$), direct bilirubin ($p < .05$) and alkaline phosphatase ($p < .05$).
**Conclusion:** PTC with bilioplasty and IEBD is feasible, has low morbidity and improves cholestasis in children who developed BBS after LT.
**Limitations:** More cases are needed to confirm the accuracy of results.
**Ethics Committee Approval:** n/a
**Funding for this study:** n/a
**Author Disclosures:**
Paolo Marra: Nothing to disclose
Pietro Andrea Bonaffini: Nothing to disclose
Claudio Sallemi: Nothing to disclose
Francesco Saverio Carbone: Nothing to disclose
Sandro Sironi: Nothing to disclose
Ludovico Dulcetta: Nothing to disclose

### RPS 409-4

**The role of dacryocystography in cases of epiphora**

*A. Posa*[1], V. Vingiani[2]; [1]Rome/IT, [2]Sorrento/IT
*(alessandro.posa@gmail.com)*

**Purpose:** To retrospectively evaluate imaging findings of patients with epiphora undergoing dacryocystography and to describe the most common findings.
**Methods or Background:** From October 2011 to March 2020, consecutive dacryocystographies performed at our Interventional Radiology service were reviewed. During this procedure, a 24-G plastic cannula is inserted through the lacrimal canal, and 2 mL or less of a radio-opaque iodinated contrast medium is slowly injected. Patients suffering from epiphora underwent this procedure to dynamically evaluate the patency of lacrimal canals, lacrimal sacs, and nasolacrimal ducts and to identify any pathological finding.
**Results or Findings:** 723 dacryocystographies were reviewed. Abnormal findings were observed in 468 exams. Lacrimal sac dilation was the most common finding (20.7% of cases) due to Beraud-Krause's valve occlusion. The lacrimal points were epithelized in 3.8% of cases, whereas the opacification of lacrimal canals alone, due to Rosenmuller's valve obstruction, was evident in 11.5% of cases. Beaded appearance of the nasolacrimal duct was seen in 16.3% of cases. Seven patients had previous lacrimal duct, turbinate, or nasal surgery.
**Conclusion:** Dacryocystography plays an essential role in the diagnostic assessment and treatment decision in patients with epiphora. Other than being diagnostic, it proves to be therapeutic in cases where obstruction is related to mucous clogs. Knowledge of pathological findings of dacryocystography is mandatory for the correct management of these patients.
**Limitations:** The retrospective nature of this study represents its main limitation.
**Ethics Committee Approval:** Ethics committee approved this study.
**Funding for this study:** No funding.
**Author Disclosures:**
Alessandro Posa: nothing to disclose
Vincenzo Vingiani: Nothing to disclose

### RPS 409-5

**Role of contrast-enhanced ultrasonography (CEUS) in percutaneous drainages of abdominal abscesses and inflammatory fluid collections not detectable at B-mode US**

*A. Borzelli*[1], F. Pane[1], F. Amodio[1], M. Coppola[1], F. Giurazza[1], F. Corvino[1], M. Silvestre[1], A. Paladini[2], R. Niola[1]; [1]Naples/IT, [2]Novara/IT
*(antonio.borzelli@libero.it)*

**Purpose:** Evaluate the role of contrast-enhanced ultrasound(CEUS)as a powerful tool and aid to better identify and define abdominal abscesses and inflammatory fluid collections, otherwise not detactable by Duplex B-mode US, to allow a more precise and safer percutaneous drainage
**Methods or Background:** We retrospectively considered, from January 2019 to June 2020, 14 patients (medium age 53,8M-6F), with CT diagnosis of abscesses and inflammatory fluid collections in paracolic-perisigmoid seat(6), in precoccygeal-presacral site (3) and perirenal-retroperitoneal site (5). In all cases, the identification of the target abscess/fluid collection by Duplex B-mode US was not possible due to intestinal bloating and gas bubbles into the abscesses, and CE-US (Sonovue, sulfur hexafluoride) was employed to better identify and delineate them, to finalise the placement of a percutaneous drainage. We considered a portal-venous and late-venous phase(60-180 sec) to identify them. All procedures were performed in angiographic suite, employing Convex probes, followed by fluoroscopic guide and corroboration. We employed CHIBA coaxial 18G needles, stiff .035" guidewires and 8-10Fr catheters for the drainage.
**Results or Findings:** Technical success was achieved in 93% (13/14) patients: in 1 patient with abscess in precoccygeal-presacral site, the placement of a drainage was not possible due to unfavourable anatomical conditions, with overlay of non-passable bone structures. In all cases, the target abscess/fluid collection was identified with more accuracy, and in 13/14 patients the placement of a percutaneous drainage was allowed with more precision and safety: for each of them, 2 samples of fluid material was collected for laboratory tests. In none of the cases major complications such as bleeding or haemorrhage due to injury to abdominal/pelvic parenchymas or great vessels occurred.

**Conclusion:** The elevated rate of technical success reported,the absence of major complications and faster performance,suggest the added value of CEUS as an advantageous tool to better identify and delineate abdominal abscesses and inflammatory fluid collections,not detactable by Duplex US,to allow a safer and more precise placement of a percutaneous drainage
**Limitations:** Restrospective Monocentric study
**Ethics Committee Approval:** Yes
**Funding for this study:** None
**Author Disclosures:**
Antonio Borzelli: Nothing to disclose
Francesco Amodio: Nothing to disclose
Fabio Corvino: Nothing to disclose
Francesco Pane: Nothing to disclose
Milena Coppola: Nothing to disclose
Andrea Paladini: Nothing to disclose
Francesco Giurazza: Nothing to disclose
Mattia Silvestre: Nothing to disclose
Raffaella Niola: Nothing to disclose

### RPS 409-6

**Comparison of perfused volume segmentation based on cone-beam CT vs 99mTc-MAA SPECT/CT for treatment dosimetry before selective internal radiation therapy using 90Y-glass microspheres**

*I. Hutuca*[1], M. Martin[1], A. Hocquelet[2], F. Debordeaux[1], L. Bordenave[1], P. Papadopoulos[1], B. Lapuyade[1], H. Trillaud[1], J-B. Pinaquy[1]; [1]Bordeaux/FR, [2]Lausanne/CH
*(ioana.hutuca@gmail.com)*

**Purpose:** Comparing the accuracy and reliability of the pre-treatment dosimetry predictions by cone-beam Computed Tomography (CBCT) versus 99mTc-labeled macroaggregated albumin (MAA) SPECT/CT in selective internal radiation therapy (SIRT) of hepatocellular carcinoma using 90Y-glass microspheres.
**Methods or Background:** We have made a retrospective study of 15 patients (7 women and 8 men) with non-resectable hepatocellular carcinoma who underwent a total of 17 SIRT procedures using 90Y-glass microspheres from December 2014 to June 2019. Pre-treatment dosimetry data were calculated from 99mTc-MAA SPECT/CT using either CBCT or 99mTc-MAA SPECT/CT to segment the perfused volume, while post-treatment dosimetry data were calculated using 90Y imaging (SPECT/CT or PET/CT). Intra-and interobserver reliabilities were evaluated by calculating Lin's concordant correlation coefficients (ρc values). The biases between pre- and post-treatment dosimetry data were investigated using the modified Bland–Altman method, and the systematic bias was evaluated using Passing–Bablok regression.
**Results or Findings:** Both methods showed good-to-excellent (ρc 0.80-0.99) interobserver reliabilities. By comparing with 90Y imaging, the median differences were 5.8 Gy (IQR: −12.7; 16.1) for DPL-CBCT and 5.6 Gy (IQR: −13.6; 10.2) for DPL 99m[illegible]-MAA SPECT/CT. The median differences were 1.6 Gy (IQR: −29; 7.53) for D[illegible]- CBCT and 9.8 Gy (IQR: −28.4; 19.9) for D[illegible]-99mTc-MAA SPECT/CT. Passing–Bablok regression analysis showed that both CBCT and 99mTc-MAA SPECT/CT had proportional biases and thus tendencies to overestimate DT and DPL at higher post-treatment doses.
**Conclusion:** CBCT is a reliable segmentation method, but it does not significantly increase the accuracy of dose prediction compared with that of 99mTc-MAA SPECT/CT. Both methods tend to overestimate the doses to tumors and perfused livers at higher doses.
**Limitations:** Retrospective, single-center study with a small number of patients.
**Ethics Committee Approval:** n/a
**Funding for this study:** n/a
**Author Disclosures:**
Panteleimon Papadopoulos: Nothing to disclose
Ioana Hutuca: Nothing to disclose
Manuel Martin: Nothing to disclose
Hervé Trillaud: Nothing to disclose
Bruno Lapuyade: Nothing to disclose
Laurence Bordenave: Nothing to disclose
Jean-Baptiste Pinaquy: Nothing to disclose
Arnaud Hocquelet: Nothing to disclose
Frederic Debordeaux: Nothing to disclose

### RPS 409-8

**Percutaneous renal cryoablation (PCA): association between tumour complexity scoring systems and outcomes**

*R. Ciabattoni*, S. Cernic, C. Marrocchio, G. Visalli, M. Rizzo, A. Piasentin, F. Giudici, M. Bertolotto, M. A. A. Cova; Trieste/IT
*(ciabattoni.riccardo@gmail.com)*

**Purpose:** To evaluate the association between renal tumor complexity, according to PADUA, RENAL and ABLATE score, and outcomes in a cohort of patients that underwent percutaneous cryoablation (PCA)
**Methods or Background:** All patients who underwent PCA between 2012 and 2020 for one or more clinically localized renal masses were included, regardless of the histology. PCA was performed by two radiologists with respectively 13 and 30 years of experience in interventional radiology. Pre-operative imaging was evaluated by 2 radiologists to determine PADUA, RENAL and ABLATE score for each lesion, based on which the lesions were classified as low-, moderate- or high-risk. The association between scores and technical success was evaluated for all lesions. Oncologic outcomes were evaluated assessing the rate of persistence or recurrence of malignant and uncharacterized lesions. Accuracy of scores was calculated using ROC analysis.
**Results or Findings:** The cohort included 174 treated lesions in 139 patients (M:98, F:41, mean age: 73 years) and a median follow up of 24,6 months. According to Padua score, 52 lesions were low risk (6-7), 64 moderate (8-9) and 58 high (>9); according to RENAL score, 69 were low risk (3-6), 93 moderate (7-9) and 12 high (>9). Mean Ablate score was 3,8. Technical success was achieved in 171/174 lesions, and only the RENAL score approached statistical significance (p=0,09) 23 patients had persistent or recurrent disease. No statistically significant difference was observed in the total scores of each scoring system between lesions that persisted/recurred and lesions that did not recur.
**Conclusion:** None of the validated score is accurate to predict the risk of recurrence. Therefore, it's necessary to develop a new score dedicated to ablation procedures to predict successfully patients' outcome.
**Limitations:** Retrospective study
**Ethics Committee Approval:** Ethical approval was obtained
**Funding for this study:** None
**Author Disclosures:**
Stefano Cernic: Nothing to disclose
Michele Bertolotto: Nothing to disclose
Michele Rizzo: Nothing to disclose
Gianluca Visalli: Nothing to disclose
Fabiola Giudici: Nothing to disclose
Cristina Marrocchio: Nothing to disclose
Andrea Piasentin: Nothing to disclose
Maria Assunta A. Cova: Nothing to disclose
Riccardo Ciabattoni: Nothing to disclose

### RPS 409-9

**Percutaneous cryoablation of thoraco-abdominal soft tissue lesions: efficacy, safety and technical aspects**

*G. Castiello*, E. Faiella, G. Pacella, C. Altomare, B. B. Beomonte Zobel, R. F. Grasso; Rome/IT
*(ge.castiello@gmail.com)*

**Purpose:** To evaluate efficacy, safety and local control of cryoablation procedures for the treatment of recurrent or metastatic tumors in thoraco-abdominal soft tissues, analyzing the technical aspects.
**Methods or Background:** 12 percutaneous cryoablation procedures in 10 patients, performed under ultrasound and/or CT-guidance for the treatment of thoraco-abdominal soft tissue lesions were retrospectively reviewed. Lesions included metastases from colorectal adenocarcinoma (n=3), lung squamous-cell carcinoma (n=1), lung small-cell carcinoma (n=1) and pancreatic adenocarcinoma (n=2), locally recurrent renal cell carcinoma (n=4) and seeding site of hepatocellular carcinoma (n=1). Efficacy of the treatment, complications rate and local control in terms of Disease-Free Survival (DFS) were analyzed.
**Results or Findings:** Mean age of patients was 68.7 years (range 58-81) and mean tumor size was 28.2 mm (range 9-53). Mean time of procedures was 65 minutes (range 30–110) with an average number of probes of 2.8 (range 1–5). Hydro-dissection was performed during 6 of the 12 procedures (50%) to protect surrounding anatomical structures. Mean ice-ball dimension, measured on CT images at the end of procedures, was 48.2 mm (range 30-90). No major complications were observed except for one case of persistent pain in the ablation site. Treatment was effective in 91.7% of the cases. Mean follow-up period was 7.9 months (range 1-12 months) with an average DFS of 6 months.
**Conclusion:** Although collection of further data is necessary, our experience suggests that cryoablation procedure is a safe and efficacious option in selected cases of recurrent or metastatic tumors of thoraco-abdominal soft tissues.

**Limitations:** n/a
**Ethics Committee Approval:** All methods and procedures were carried out following the ethical standards of the institutional research committee and complied with the 1964 Helsinki declaration.
**Funding for this study:** None
**Author Disclosures:**
Gennaro Castiello: Nothing to disclose
Bruno Beomonte Beomonte Zobel: Nothing to disclose
Rosario Francesco Grasso: Nothing to disclose
Eliodoro Faiella: Nothing to disclose
Giuseppina Pacella: Nothing to disclose
Carlo Altomare: Nothing to disclose

### Research Presentation Session: Interventional Radiology

### RPS 509

### Interventional radiology in cancer treatment

### RPS 509-1

**Surgical resection versus CT-guided percutaneous thermal ablation survival outcomes for stage I Non-Small Cell Lung Cancer (NSCLC): a systematic review and meta-analysis**
*R. Huo*, M. V. Chan, C. Cao, L. Ridley; Sydney/AU

**Purpose:** Multiple cohort studies have compared surgical resection with CT-guided percutaneous thermal ablation for patients with Stage 1 non-small cell lung cancer (NSCLC), however the results have been heterogeneous. This systematic review and meta-analysis aims to compare the survival and disease free survival (DFS) of surgery and percutaneous ablation for Stage 1 NSCLC.
**Methods or Background:** A search of five databases was performed from inception to July 2020. Studies were included if survival outcomes were compared between patients treated with CT-guided thermal ablation versus surgical resection for Stage 1 NSCLC.
**Results or Findings:** A total of eight studies were included, involving a total of 708 patients. There were no significant differences in 1- to 5-year survival between surgery versus ablation. There was significantly better 1- and 2-year DFS for surgery over ablation, but not 3- to 5-year DFS. Subgroup analysis in Stage 1A demonstrated no significant differences in 1- to 3-year survival or DFS between surgery versus ablation. There was no significant survival difference between lobectomy and microwave ablation (MWA), but there was significantly better 1- and 2-year survival with limited resection versus radiofrequency ablation (RFA).
**Conclusion:** Surgical resection of Stage 1 NSCLC remains the optimal choice. However, for non-surgical patients with Stage 1A, CT-guided thermal ablation may be an alternative which offers promising disease-free survival and overall survival. Future prospective randomised controlled trials are warranted.
**Limitations:** Firstly, most studies were retrospective in nature, which contain inherent biases such as differences in the baseline patient and tumour characteristics between the two treatment groups. To minimise the risk of bias, matched data was used when available. Secondly, there are only two studies that specifically assessed MWA outcomes.
**Ethics Committee Approval:** Not required as systematic review
**Funding for this study:** Not funding
**Author Disclosures:**
Ruth Huo: Nothing to disclose
Michael Vinchill Chan: Nothing to disclose
Christopher Cao: Nothing to disclose
Lloyd Ridley: Nothing to disclose

### RPS 509-2

**CT-Guided High-Dose-Rate Brachytherapy (CT-HDRBT) & combined transarterial chemoembolisation with irinotecan-loaded microspheres vs. CT-HDRBT in patients with unresectable colorectal liver metastases**
*F. Busch*, S. A. Friedrich, M. Jonczyk, G. Wieners, G. Böning, A. Meddeb, F. Collettini, B. Gebauer; Berlin/DE
*(felix.busch@charite.de)*

**Purpose:** To compare outcome and toxicity profiles of mono-CT-HDRBT with a combination therapy of irinotecan-TACE and CT-HDRBT in patients with irresectable colorectal liver metastases (CRLM).
**Methods or Background:** This retrospective matched-pair-analysis included 44 patients in 2 cohorts with irresectable CRLM, treated either with mono-CT-HDRBT or with a combination of irinotecan-TACE and CT-HDRBT in equal parts. Matching addressed clinical target volume (CTV), number of lesions, target dose, coverage and number of catheters per CT-HDRBT, age and sex. Time of local tumour control (LTC) and progression-free survival (PFS) were estimated using Kaplan-Meier-Method. Treatment toxicities were documented per National Cancer Institute Common Terminology Criteria for Adverse Events 4.0. Catheter-related adverse events were documented per Society of Interventional Radiology classification. Statistical analysis included the Shapiro-Wilk, the paired sample T- and the Wilcoxon test. p-values <0.05 were considered significant.
**Results or Findings:** Combination therapy resulted in longer PFS (5/2 months; p=0.004) and higher LTC rates after 12 months (68%/23%; p=0.002) compared to mono-CT-HDRBT. There are tendencies for longer LTC (17/9 months; p=0.052) and lower systemic progress rates after 12 months (64%/91% of patients; p=0.058) in patients undergoing combination therapy. No catheter-associated major or minor complications occurred. AST, ALT and their toxicity levels showed significant higher increases after combination therapy post-interventionally. Patients with monotherapy presented with a significantly higher increase in haemoglobin and total bilirubin toxicity levels.
**Conclusion:** The combination of irinotecan-TACE and CT-HDRBT shows longer LTC and PFS compared to mono-CT-HDRBT in patients with non-resectable CRLM. Irinotecan-TACE combined with CT-HDRBT shows a satisfying safety profile.
**Limitations:** - retrospective cohort allocation of the group with mono-CT-HDRBT; monocentric treatment at Charité - University Medicine Berlin; limited number of 22 patients per cohort
**Ethics Committee Approval:** Ethics committee of Charité – University Medicine Berlin (EA1/043/15)
**Funding for this study:** Charité – University Medicine Berlin
**Author Disclosures:**
Gero Wieners: Nothing to disclose
Martin Jonczyk: Nothing to disclose
Bernhard Gebauer: Nothing to disclose
Aymen Meddeb: Nothing to disclose
Stefanie Andrea Friedrich: Nothing to disclose
Felix Busch: Nothing to disclose
Federico Collettini: Nothing to disclose
Georg Böning: Nothing to disclose

### RPS 509-3

**Efficacy and safety of PD1 inhibitor combined with TACE in the treatment of huge primary live cancer**
*S. Fang*, L. Zheng, F. Wu, W. Chen, Z. Zhao, J. Ji; Lishui/CN
*(fsj1216@163.com)*

**Purpose:** To investigate the efficacy and safety of PD1 inhibitor combined with TACE in the treatment of huge primary live cancer
**Methods or Background:** From June 2016 to December 2019, the clinical data of 31 patients with huge primary live cancer were collected and analyzed. The patients were divided into the control group (n=18 cases) and the combined group (n=13 cases). The patients were followed up and the adverse events were observed. The DCR were compared. The median OS and PFS were calculated. The expression of GPC3 was detected.
**Results or Findings:** The DCR in combined group (53.8%) was higher than that in control group (22.2%). The difference was statistically significant (Z=—2.13, P=0.04). The median PFS in combined group was significantly longer than that in control group (5.0 months vs. 3.0 months ,X2=4.39, P=0.04). The median OS in combined group was significantly longer than that in control group (15 months vs. 9 months,,X2=5.51, P=0.02). The median PFS of GPC3 negative patients in combined group was higher than that in the control group (7 months vs. 5 months, X2=4.38, P=0.03). The incidence rate of rash in combine group was higher than that in control group (23.1% vs. 0.0%, (P=0.01).
**Conclusion:** The combine PD1 inhibitors with TACE is an efficacy and safety therapy for huge primary live cancer and the outcome of patients with GPC3 negative is better than those with positive patients.
**Limitations:** the defects inherent to type of retrospective study design. In addition, the limitation of the sample size of patients and the length of follow-up also had a significant impact on the outcome.
**Ethics Committee Approval:** approved by the ethics committee of Lisui Hospital, Zhejiang University, China
**Funding for this study:** National Natural Science Foundation of China (81803778); Zhejiang Natural Science Foundation/exploration project Q (LQ20H160055)
**Author Disclosures:**
Fazong Wu: Author: conducted all experiments, analyzed the data and wrote the manuscript.
Shiji Fang: Speaker: conducted all experiments, analyzed the data and wrote the manuscript, and designed the study and revise this manuscript.
Jiansong Ji: Author: designed the study and revise this manuscript.

Liyun Zheng: Author: conducted all experiments, analyzed the data and wrote the manuscript.
Weiqian Chen: Author: conducted all experiments, analyzed the data and wrote the manuscript.
Zhongwei Zhao: Author: conducted all experiments, analyzed the data and wrote the manuscript.

### RPS 509-4

**B-TARE in local advanced HCC patients: safety and feasibility**

P. Lucatelli, *C. Trobiani*, F. Cappelli, S. Ungania, G. E. E. Vallati; Rome/IT
*(Claudio.trobiani@gmail.com)*

**Purpose:** To evaluate safety and feasibility of balloon-occluded trans-arterial radioembolization (B-TARE) treatment in local advanced, unresectable, hepatocellular carcinoma (HCC) patients.
**Methods or Background:** We treated 10 Child A patients with unresectable HCC (BCLC B) using balloon-occluded trans-arterial radioembolization. Every patient underwent clinical and laboratory (Bilirubine, ALT, AST, GGT, INR, Platelets) monitoring to assess presence of intra- or periprocedural complications. Impact of balloon micro-catheter trans-arterial loco-regional treatment was analyzed using 2D and 3D dosimetry in post-procedural SPECT. Early local efficacy was evaluated on 1- and 3-month follow-up multiphasic computed tomography (CT) on the basis of m-RECIST criteria.
**Results or Findings:** Technical success was obtained for all procedures and no major complications were observed. In 2D evaluation, the activity intensity peak was 987.5 (±393.8), that shows that a high amount of Y90-microspheres was delivered to the lesion. Regarding 3D dose analysis (expression of Absorbed Dose in Gy), the mean dose <D> administered to the treated lesions was 151.6 (±53.2) with a low mean dose delivered to the normal liver (29.4 ± 5.7). Complete response (CR) was obtained at 3-months follow up in 7 out of 10 patients, with 2 Partial Response (PR) e 1 Stable Disease (SD).
**Conclusion:** In our preliminary experience B-TARE seems to be a safe and effective local therapeutic option for unresectable HCC lesions, showing a high rate of local response.
**Limitations:** Small number of patients.
**Ethics Committee Approval:** Study was approved by our institutional ethics committees.
**Funding for this study:** No funding was received for this work.
**Author Disclosures:**
Pierleone Lucatelli: Nothing to disclose
Giulio Eugenio Eugenio Vallati: Nothing to disclose
Claudio Trobiani: Nothing to disclose
Sara Ungania: Nothing to disclose
Federico Cappelli: Nothing to disclose

### RPS 509-8

**Hepatic hilar and celiac plexus nerve block as analgesia for doxorubicin-eluting beads embolisation procedures for hepatocellular carcinoma**

*A. A. A. Bessar*; Zagazig/EG
*(ahmedawadbessar@gmail.com)*

**Purpose:** The aim of the present study was to assess hepatic hilar and celiac plexus nerve block as analgesia for doxorubicin-eluting beads embolization procedures for hepatocellular carcinoma.
**Methods or Background:** A total of 92 patients underwent their first transarterial chemoembolization procedures with doxorubicin-eluting beads. Patients were grouped in two groups A and B. Patients in group A underwent hepatic hilar and celiac block procedures (HHCB group) and the control group B were referred as (sham group). All patients were diagnosed by CT or MRI and laboratory investigations. The study required a post-TACE follow-up for three weeks.
**Results or Findings:** Pain was significantly lower in group A than group B for first day and week. Regarding the doses for intra-arterial lidocaine injection, group B was significantly higher than group A. The oral morphine equivalent opioid dose in HHCB group was nearly the double dose in sham group. Patients in group A were satisfied more than group B patients.
**Conclusion:** Pre-procedural nerve block for hepatic hilar nerves and celiac plexus can result in lower intra-procedural and post-procedural pain in patients underwent DEB-TACE procedures also result in lower opioid consumption and opioid related complications in the next few weeks following TACE.
**Limitations:** 1- No data regarding the size of masses embolized to assess volume of necrosis and it could be impossible to compare between degree of pain and volume of necrosis in the tumor tissue. 2- We didn't measure the effect of pre-operative preventive opioid treatment for our patients in improving the severity of pain during and after TACE.
**Ethics Committee Approval:** This prospective, randomized, double-blind clinical trial was approved by the Committee of Institutional Review Board in Zagazig University (IRB#: 6524-6-1-2019)
**Funding for this study:** The study was funded by the national health insurance system in Egypt.
**Author Disclosures:**
Ahmed Awad Abdelaziz Bessar: Nothing to disclose

### RPS 509-10

**Chemosaturation with Percutaneous Hepatic Perfusion (CS-PHP) in patients with liver-dominant metastatic uveal melanoma: outcome and safety analysis**

*C. Dewald*[1], L. S. Becker[1], S. K. Maschke[1], T. C. Meine[1], J. B. Hinrichs[1], M. Kirstein[2], A. Vogel[1], B. C. Meyer[1], F. Wacker[1]; [1]Hannover/DE, [2]Lübeck/DE
*(dewald.cornelia@mh-hannover.de)*

**Purpose:** Chemosaturation percutaneous hepatic perfusion (CS-PHP) is a palliative treatment approach, which allows selective intrahepatic delivery of high dose melphalan while minimizing systemic toxicity via filtration of the hepatic venous blood. Aim of this study was to assess safety, response and survival of CS-PHP in patients with liver-dominant metastatic uveal melanoma (UM).
**Methods or Background:** Adverse events (AEs) were classified using Common Terminology Criteria for Adverse Events (CTCAE) v5. Overall response rate (ORR) and disease control rate (DCR) were evaluated according to RECIST 1.1. Median overall survival (mOS), median progression-free survival (mPFS) and hepatic mPFS (mhPFS) were assessed using Kaplan-Meier estimation.
**Results or Findings:** Overall, 70 CS-PHP were performed in 30 patients with metastasized UM in a salvage setting (10/2014-01/2019). ORR and DCR were 42.3% and 80.8%, respectively. Overall, mOS was 12 months (95% confidence interval (CI) 7-15), mPFS was 6 months (95% CI 4-10) and mhPFS was 6 months (95% CI 4-13). AEs most frequently included significant hematologic toxicities (87% of grade 3/4 thrombocytopenia), which were transient. Less frequent AEs were hepatic injury extending to liver failure (3%) and cardiovascular events including one case of ischemic stroke (3%).
**Conclusion:** CS-PHP is an effective treatment in selected patients with liver-dominant metastasized UM. The interventional procedure is safe. Severe hepatic and cardiovascular AEs, albeit rare, demand careful patient selection.
**Limitations:** Main limitation are the retrospective study design and the mono-centric nature of the study. Complications might be underestimated, if they occurred after discharge.
**Ethics Committee Approval:** This retrospective was approved by the local ethics committee.
**Funding for this study:** Our institute received a grant from the developer of the CS-PHP-specific catheter Delcath Systems Inc. Design, evaluation and results of the study were not influenced by Delcath Systems Inc.
**Author Disclosures:**
Timo C. Meine: Nothing to disclose
Bernhard C Meyer: Nothing to disclose
Martha Kirstein: Nothing to disclose
Arndt Vogel: Nothing to disclose
Cornelia Dewald: Nothing to disclose
Sabine Katharina Maschke: Nothing to disclose
Lena Sophie Becker: Nothing to disclose
Frank Wacker: Nothing to disclose
Jan B Hinrichs: Nothing to disclose

### RPS 509-11

**Percutaneous microwave ablation (MWA) of hepatocellular carcinoma (HCC): local tumour progression, survival and complication rate**

*T. J. Vogl*, H. Adwan, B. Panahi, T. Gruber-Rouh, N-E. A. N-E. Mohammed; Frankfurt/DE
*(t.vogl@em.uni-frankfurt.de)*

**Purpose:** To retrospectively evaluate the efficacy of microwave ablation (MWA) in the therapy of hepatocellular carcinoma (HCC) using local tumor progression, survival and complication rate.
**Methods or Background:** In this study 235 patients (54 females, 181 males; mean: 66 years; range: 56-76 years) with 418 HCC lesions were treated using CT-guided MWA. They were evaluated according to location, diameter and volume of HCC lesions, post ablation necrosis volume, used power, duration of ablation, survival time, local tumor progression and complications. For evaluation of response to MWA treatment MRI was performed post ablation.
**Results or Findings:** Pre ablation mean diameter of HCC lesions was 2.11 cm and mean volume was $4.75^3$. Mean post ablation necrosis volume was 31.51 $cm^3$. Rate of local tumor progession was 5.1% (12/235). The 1-, 3- and 5-year survival rates were 93.4%, 72.5%, and 46.3%. Complication rate was 4.2% (10/235). Median survival time was 4.5 years.

**Conclusion:** Percutaneous MWA is an effective and safe treatment method for patients with HCC with a long overall survival time, low local tumor progression and low complication rate.
**Limitations:** Retrospective study design
**Ethics Committee Approval:** Approval of the ethical committee obtained
**Funding for this study:** No funding
**Author Disclosures:**
Bita Panahi: Nothing to disclose
Tatjana Gruber-Rouh: Nothing to disclose
Thomas J. Vogl: Nothing to disclose
Nour-Eldin Abdelrehim Nour-Eldin Mohammed: Nothing to disclose
Hamzah Adwan: Nothing to disclose

### RPS 509-12

**Local ablation therapy of pulmonary tumours: a retrospective analysis of factors affecting the outcome**
*E. H. A. Emara*[1], E. Elhawash[2], B. Panahi[3], N. N. N. Naguib[3], N-E. A. N-E. Mohammed[3], T. J. Vogl[3]; [1]Kafr El Sheikh/EG, [2]Alexandria/EG, [3]Frankfurt a. Main/DE
*(emademara85@yahoo.com)*

**Purpose:** To evaluate retrospectively the factors affecting the treatment outcome of microwave ablation of lung tumors (MWA).
**Methods or Background:** 124 patients (average age 61.8 +/- 4.6 years, 59 man & 65 women) with 168 inoperable pulmonary lesions (41 primary, 127 metastatic); tumor board decision was to perform CT-guided MWA. Initial CT assessment of lesions size, volume, outlines, location in relation to hilum, vascular and bronchial structures followed by CT-guided MWA. Post-ablation follow-up contrast enhanced CT was performed at 24 hours 3,6,9 12 months then every 6 months to determine response to treatment. Certain parameters including the pre-ablation size and volume, lesion location in relation to lung hilum, pulmonary vessels or bronchial tree, pathological type and outlines of the lesions are precisely evaluated and correlated with the follow up images and long-term treatment response.
**Results or Findings:** 129 lesions (76.8%) showed complete response with no residual activity while the rest (23.2%) showed incomplete response on follow up. Certain factors have been found and suggested as early predictors for the response including the maximum diameter of the lesion as well as its volume and location in relation to hilum with scientifically better response seen with peripherally located lesions less than 3 cm in diameter (p=0.019,0.026,0.037 respectively). The pathological type and location of the lesion as well as its relation the significant vessel or bronchus and outlines of lesions in the current study didn't seem to affect the overall response (p=0.41,0.34,0.47,0.18 respectively).
**Conclusion:** Pulmonary microwave ablation therapy is a safe effective minimally invasive therapeutic tool with the preablation tumor size, volume and location in relation to lung hilum are the main determinant of the treatment efficacy
**Limitations:** Retrospective study- small number of patients
**Ethics Committee Approval:** Yes
**Funding for this study:** No fund
**Author Disclosures:**
Bita Panahi: Nothing to disclose
Nagy Naguib Naeem Naguib: Nothing to disclose
Thomas J. Vogl: Nothing to disclose
Nour-Eldin Abdelrehim Nour-Eldin Mohammed: Nothing to disclose
Elsayed Elhawash: Nothing to disclose
Emad Hassan Ali Emara: Nothing to disclose

### RPS 509-13

**Construction of a novel radiomics nomogram for the prediction of aggressive intra-segmental recurrence of HCC after radiofrequency ablation**
*J. Ji*, X. Lv, M. Chen, G. Shu, C. Kong, S. Cheng, L. Zheng, S. Fang, C. Chen; Lishui/CN
*(lschrjjs@163.com)*

**Purpose:** Construction of a Novel Radiomics Nomogram for the Prediction of Aggressive Intra-segmental Recurrence of HCC after Radiofrequency Ablation
**Methods or Background:** There were 22 patients with AIR and 36 patients without AIR enrolled in our study. MRI scans were performed before RFA and followed up for more than 6 months. The laboratory indicators and MRI features were compared and assessed. Preoperative CE-T1WI were used for radiomics analysis. The selected clinical indicators and texture features were screened out to generate the novel prediction nomogram.
**Results or Findings:** Tumor shape, ADC Value, SI of DWI and ΔSI were selected as the independent factors of AIR. Meanwhile, two radiomics features were selected  by LASSO (p<0.05), which were further integrated with the Rad-score to construct the predictive model, and AUCs were 0.941 (95% CI: 0.876-1.000) and 0.818 (95% CI: 0.576-1.000) in the training and validation cohorts, respectively. The AIR predictive model was further converted into a novel radiomics nomogram, and decision curve analysis showed good agreement.
**Conclusion:** The predictive nomogram integrated with clinical factors and CE-T1WI -based radiomics signature could accurately predict the occurrence of AIR after RFA.
**Limitations:** 1. The number of patients enrolled in this study was small. 2.The current study was a single-center, retrospective study.
**Ethics Committee Approval:** The study was approved by the institutional review board, human ethics committee of Lishui Hospital of Zhejiang University, and the requirement for informed consent was waived.
**Funding for this study:** The National Key Research and Development projects intergovernmental cooperation in science and technology of China (No. 2018YFE0126900) Key projects jointly built by provincial and ministry of the National Health Commission (No. WKJ-ZJ-1932) the Public Welfare Technology Research Projects of Zhejiang Province (Nos. LGD19H160002 and LGF19H180009) the Research and Development projects of Lishui City (Nos. 2019ZDYF17 and 2019ZDYF09)
**Author Disclosures:**
Minjiang Chen: Nothing to disclose
Chunli Kong: Nothing to disclose
Shiji Fang: Nothing to disclose
Jiansong Ji: Nothing to disclose
Shimiao Cheng: Nothing to disclose
Liyun Zheng: Nothing to disclose
Gaofeng Shu: Nothing to disclose
Xiuling Lv: Nothing to disclose
Chunmiao Chen: Nothing to disclose

### RPS 509-14

**Prediction of tumour response via pretreatment MRI radiomics-based nomogram in hepatocellular carcinoma treated with transcatheter arterial chemoembolisation**
*C. Kong*; Lishui/CN

**Purpose:** To develop and validate a pre-transcatheter arterial chemoembolization (TACE) MRI-based radiomics model for predicting tumor response in advanced hepatocellular carcinoma (HCC) patients.
**Methods or Background:** 99 advanced HCC patients (69 for training cohort and 30 for validation cohort) with TACE treatment were enrolled. MRI examination were performed before TACE, and the imageological examination were performed 3 months after TACE to evaluate the efficacy according to the mRECIST criterion. A total of 396 radiomics features were extracted from T2-weighted images of pre-TACE, and least absolute shrinkage and selection operator (LASSO) regression was applied to feature selection and model construction. The performance of the model was evaluated by receiver operating characteristic curve (ROC), calibration curve, and decision curve.
**Results or Findings:** The AFP value, Child-Pugh score and BCLC stage showed statistical differences between the TACE response (TR) and non-TACE response (nTR) patients. Six radiomics features were selected by LASSO, which were further applied to calculate the Rad-score. The AUC of the ROC based on Rad-score were 0.812 and 0.866 in the training and validation cohorts, respectively. To improve the diagnostic efficiency, the Rad-score was further integrated with the above clinical indicators to form a novel predictive nomogram. Results suggested that the AUC increased to 0.861 and 0.884 in the training and validation cohorts. Decision curve analysis showed that the radiomics nomogram was clinically useful.
**Conclusion:** The radiomics and clinical indicators-based predictive nomogram can well predict TR in advanced HCC, which can further be applied for auxiliary diagnosis of clinical prognosis.
**Limitations:** The selection bias was inevitable because the study was a single-center, retrospective study.
**Ethics Committee Approval:** The institutional review board and human ethics committee of Lishui Hospital of Zhejiang University approved the study, and the requirement for informed consent was waived.
**Funding for this study:** None
**Author Disclosures:**
Chunli Kong: Nothing to disclose

### RPS 509-15

**Role of microwave ablation in the treatment of lung tumours**
*E. H. A. Emara*[1], H. M. Abdel-Rahman[2], S. S. Hegab[2], M. Abo Elez[2], T. J. Vogl[3]; [1]Kafr El Sheikh/EG, [2]Zagazig/EG, [3]Frankfurt/DE
*(emademara85@yahoo.com)*

**Purpose: T**o evaluate prospectively the efficacy and safety of CT-guided microwave ablation in treatment of lung tumours with studying the effect pre-ablation lesions characteristics on the treatment outcome.
**Methods or Background:** This prospective study included 40 patients with 52 malignant lung lesions, underwent CT-guided microwave ablation. All patients were subjected to complete clinical examination, pre-procedural laboratory investigations & imaging evaluation. Post ablation follow up by chest CT was done after 24 hours, three, six, nine months, one year and every 6 months onwards to determine treatment response. Patients were either adequately ablated (no residual tumor activity) or had local progression (residual tumor activity).
**Results or Findings:** 44 lesions (84.6%) showed complete response to treatment and 8 lesions (15.4%) had local progression (residual activity). The median time to local tumor progression was 8.3 months. The median survival was 32 months for patient underwent MWA according to the Kaplan-Meier test. The overall survival rate at 1, 2, and 3 years was 97.5%, 90%, and 82.5%, respectively. Successful tumor ablation was significantly more frequent for lesions with a maximal axial diameter of 3 cm or smaller (P =.0001). There were no deaths during the procedure and the mortality rate within 6 months after ablation was 0%. Early postablation complications included pneumothorax (13.5%), pulmonary hemorrhage (9.6%) and postablation syndrome (3.85%), Pleural Effusion (3.85%), Hemoptysis (3.85%). Manual evacuation was done in 3 cases out of 7 sessions complicated by pneumothorax. No significant long-term complications were detected.
**Conclusion:** Percutaneous CT-guided microwave ablation therapy for management of pulmonary tumors is safe and effective minimally invasive option and can improve local tumor control and survival rate in patients who are not candidate for surgical resection.
**Limitations:** Small number of patients- heterogenicity of lung tumors
**Ethics Committee Approval:** Yes
**Funding for this study:** No funding
**Author Disclosures:**
Mona Abo Elez: Nothing to disclose
Hossam Mansour Abdel-Rahman: nothing to disclose
Thomas J. Vogl: Nothing to disclose
Sameh Saber Hegab: Nothing to disclose
Emad Hassan Ali Emara: Nothing to disclose

### RPS 509-16

**sABLATE: a simplified ABLATE score for the prediction of complications and outcome in percutaneous thermal ablation of renal lesions**

*P. Biondetti*, M. Papa, R. Colombo, A. M. Ierardi, G. Cardone, G. Carrafiello; Milan/IT

**Purpose:** To evaluate the performance of a simplified-ABLATE score (sABLATE) in predicting complications and outcome in percutaneous thermal ablation of renal lesions, when compared to RENAL, mRENAL and ABLATE scores.
**Methods or Background:** This retrospective study included 136 renal lesions in 113 patients (M:F ratio=2.5; mean age 70.8 years). 98 tumors underwent ablation at San Raffaele hospital between 01/2015 and 03/2020, 37 at San Paolo or Policlinico hospitals between 07/2016 and 03/2020. RENAL, mRENAL, ABLATE and sABLATE scores were calculated for each lesion using preprocedural imaging. Data regarding complications and follow-up were registered. Mann-Whitney U test, ROC analyses, and logistic regression analyses were used for complications. Cox-regression analyses were performed for outcome
**Results or Findings:** Mean diameter of renal tumors was 23.2mm. Mean and median RENAL, mRENAL, ABLATE and sABLATE scores were 6.8 and 7, 6.9 and 7, 5.3 and 5, and 3.5 and 3, respectively. During a mean follow-up of 21.9 months (range 1-73) we registered 7 complications, 3 cases of residual disease and 10 of local tumor progression. Mann-Whitney U test p-values for complications for RENAL, mRENAL, ABLATE and sABLATE were 0.51, 0.49, 0.66 and 0.05, respectively. ROC analyses for complications showed an AUC for RENAL, mRENAL, ABLATE and sABLATE of 0.57, 0.57, 0.55 and 0.71 respectively. Logistic regression failed to demonstrate significant associations between scores and complications. HR and p-values of Cox-regression analyses were 1.30 and 0.36 for RENAL, 1.33 and 0.35 for mRENAL, 2.16 and 0.01 for ABLATE, 2.29 and 0.004 for sABLATE.
**Conclusion:** sABLATE seems to perform better than the other scores in predicting complications, without reaching 5% significance. Both ABLATE and sABLATE were significantly associated with outcome, with sABLATE demonstrating the best performance.
**Limitations:** Retrospective nature
**Ethics Committee Approval:** IRB-approved with waiver of informed consent
**Funding for this study:** None
**Author Disclosures:**
Maurizio Papa: Nothing to disclose
Anna Maria Ierardi: Nothing to disclose
Pierpaolo Biondetti: Nothing to disclose
Roberta Colombo: Nothing to disclose
Gianpiero Cardone: Nothing to disclose
Gianpaolo Carrafiello: Nothing to disclose

### RPS 509-17

**Neutrophil to lymphocyte ratio predicts local tumour progression-free survival after microwave ablation of hepatocellular carcinoma**

*A. Della Corte*, F. Ratti, D. Grippaldi , S. Gusmini, F. Cipriani, L. Aldrighetti, F. De Cobelli; Milan/IT
*(dellacorte.angelo@hsr.it)*

**Purpose:** To evaluate the short- and long-term oncological outcome of Microwave Ablation (MWA) of HCC. Technique-, nodule- and disease-related features, as well as inflammatory markers were analyzed.
**Methods or Background:** From November 2014 to September 2020, 106 patients (155 tumors) underwent MWA with a 2450Hz/100W generator (Emprint, Medtronic) with percutaneous or intraoperative approaches. Data were collected regarding amount of delivered energy, technical approach, tumor size and location, Child-Pugh class, hepatitis etiology, multifocality, previous treatments for HCC. Inflammatory markers (neutrophil/lymphocyte ratio [NLR]) were collected pre-, post-operatively, upon patient discharge and on first follow-up visit. Endpoints were local tumor progression-free survival (LTPFS) and intrahepatic progression-free survival (IHPFS).
**Results or Findings:** 94 patients (121 nodules) satisfied inclusion criteria for analysis (technique efficacy, follow-up≥6 months), with median follow-up of 19 months (range 6-55 months). LTP occurred in 26 tumors (21.5%). At univariate analysis, risk factors for LTP included NLR1stFU (HR=1.24, p=0.004), ΔNLRPREOP-1stFU (HR=1.23 p=0.001), percutaneous approach (HR=3.3 ,p=0.05), previous TACE (HR=2.24, p=0.046) Child-Pugh B (HR=3.08, p=0.025). Upon multivariate analysis, independent predictive factors of LTP were ΔNLRPREOP-1stFU (HR=1.63 ,p=0.04) and Child-Pugh B (HR=7.56, p=0.002). IHP occurred in 51 patients (54.3%). At univariate analysis, risk factors for IHP were subcapsular location (HR=1.82, p=0.036) and multifocality (HR=1.96, p=0.021).
**Conclusion:** Disease characteristics and inflammatory status seem to influence local recurrence more than technique- and nodule-related features. Our data support the role of inflammation in development of LTP after MWA, as NLR change in response to ablation was an independent predictive factor. Inflammatory markers do not seem to play a role on distant hepatic recurrence.
**Limitations:** More data are required to define the exact mechanisms of action of post-ablation immune response.
**Ethics Committee Approval:** Retrospective study approved by Institutional Board.
**Funding for this study:** No funding was received for this study.
**Author Disclosures:**
Angelo Della Corte: Nothing to disclose
Luca Aldrighetti: Nothing to disclose
Simone Gusmini: Nothing to disclose
Federica Cipriani: Nothing to disclose
Francesco De Cobelli: Nothing to disclose
Daniele Grippaldi: Nothing to disclose
Francesca Ratti: Nothing to disclose

### Research Presentation Session: Musculoskeletal

### RPS 310

### Trauma in MSK

### RPS 310-1

**Primary arthrodesis versus open reduction and internal fixation based on clinical outcome and 2D and 3D geometrical analyses obtained from weight-bearing cone-beam CT images**

*R. Wellenberg*, N. Baboeram, F. Sanders, I. Dobbe, G. Streekstra, T. Schepers, M. Maas; Amsterdam/NL
*(r.h.wellenberg@amsterdamumc.nl)*

**Purpose:** The purpose of the study was to compare primary arthrodesis versus open reduction and internal fixation (ORIF) based on clinical outcome and 2D and 3D geometrical analyses obtained from weight-bearing (WB) cone-beam CT images.
**Methods or Background:** In this prospective study, 40 patients with surgically treated calcaneal fractures were included, consisting of 20 primary arthrodesis and 20 ORIF patients. WB cone-beam CT-images of the left and right hindfoot and forefoot were acquired on a Planmed Verity cone-beam CT-scanner with a minimum of one-year follow-up. Hindfoot and forefoot images were fused using stitching software. Automated 2D and 3D geometric analyses, i.e. calcaneal pitch and Meary’s angle were obtained for injured and healthy feet. Clinical outcomes were measured using the EQ5D and FFI questionnaires.

**Results or Findings:** Baseline criteria of patients undergoing arthrodesis or ORIF did not differ, apart from age ($p<0.005$). Calcaneal pitch and Meary's angle were not statistically different between healthy feet of arthrodesis and ORIF patients in both 2D and 3D. In 2D, calcaneal pitch of the injured foot was lower in arthrodesis patients (10.9° ± 4.5) compared to ORIF patients (13.8° ± 5.6) ($p<0.001$). Meary's angle in 2D was lower in the injured foot in arthrodesis patients (7.0° ± 5.8) compared to ORIF patients (15.5° ± 5.9) ($p=0.046$). In 3D, no statistical differences were found between both groups for both measurements. Clinical outcomes were not statistically different between both groups.
**Conclusion:** Three-dimensional WB CT imaging enables functional 2D and 3D analyses under natural load in patients with complex calcaneal fractures. Based on clinical outcome and geometrical analyses both primary arthrodesis and ORIF appear viable treatment options.
**Limitations:** Manual bone segmentation, limited follow-up period.
**Ethics Committee Approval:** Medical ethical approval was obtained (METC2019_038).
**Funding for this study:** Funding was received from Amsterdam Movement Sciences.
**Author Disclosures:**
Fay Sanders: Nothing to disclose
Tim Schepers: Nothing to disclose
Mario Maas: Nothing to disclose
Ruud Wellenberg: Nothing to disclose
Iwan Dobbe: Nothing to disclose
Geert Streekstra: Nothing to disclose
Nigel Baboeram: Nothing to disclose

### RPS 310-2

**Posterior extraarticular ischiofemoral impingement can be caused by the lesser and the greater trochanter in patients with increased femoral version**

*T. D. Lerch*[1], F. Schmaranzer[1], I. Todorski[1], S. Steppacher[1], K. Siebenrock[1], M. Tannast[2]; [1]Bern/CH, [2]Fribourg/CH
*(till.lerch@insel.ch)*

**Purpose:** Posterior extraarticular hip impingement has been described for hips with increased femoral version (FV). These patients can present clinically with lack of external rotation (ER) and extension and with a positive posterior impingement test. We evaluated patient-specific 3D-CT and asked whether range of motion (ROM) and location of osseous posterior intra-and extraarticular impingement differ between hips with increased FV and a control group using patient-specific 3D-CT.
**Methods or Background:** Osseous 3D-models based on 3D-CT scans were reconstructed of 52 hips(38 symptomatic patients) with positive posterior impingement test and increased FV(>35°). They were mainly female patients (96%) with a mean age ranging from 23 to 38 years. These included 26 hips with an increased McKibbin instability index>70, while 21 hips had isolated increased FV(>35°). The control group consisted of 20 hips with normal FV, normal AV and no valgus(CCD-angle<139°). Segmentation of the hip joint was performed using segmentation software AMIRA. Validated 3D-CT based collision-detection software for impingement-simulation was used to calculate impingement-free ROM and location of hip impingement.
**Results or Findings:** Hips with increased FV had significantly($p<0.001$) decreased extension and ER in 90° of flexion compared to the control group. Posterior impingement was located(92%) extraarticular in hips with increased FV. Posterior hip impingement occurred between the ischium and the lesser trochanter in 20° of extension and 20° ER. Impingement was located between the ischium and the greater trochanter or intertrochanteric area in 20° of flexion and 40° ER
**Conclusion:** 3D CT enables patient-specific diagnosis of posterior extraarticular hip impingement. Posterior hip impingement can be caused by the lesser and the greater trochanter or the intertrochanteric region. 3D-CT can help for surgical planning such as femoral derotation osteotomy and/or hip arthroscopy.
**Limitations:** Only osseous impingement
**Ethics Committee Approval:** Yes
**Funding for this study:** None
**Author Disclosures:**
Till Dominic Lerch: Nothing to disclose
Inga Todorski: Nothing to disclose
Florian Schmaranzer: Nothing to disclose
Simon Steppacher: Nothing to disclose
Moritz Tannast: Nothing to disclose
Klaus Siebenrock: Nothing to disclose

### RPS 310-3

**USG and MRI imaging of rotator cuff tears with surgical correlation**

*V. Rustagi *; Mumbai/IN
*(drvikashrustagi@gmail.com)*

**Purpose:** To compare the diagnostic efficacy of ultrasound and MRI in the detection of rotator cuff tears with surgical findings (arthroscopy or open surgery) as a gold standard. To describe the distribution of rotator cuff tears in terms of age, gender, symptomatology, and associated abnormalities among the study population.
**Methods or Background:** Prior ultrasound screening was done for every patient who came for MRI shoulder1.5T with clinical suspicion of rotator cuff tear. Of 75 patients who were diagnosed with a rotator cuff tear, only those 56 were included in the study group in whom arthroscopy/open surgery confirmation was available.
**Results or Findings:** STATISTICAL ANALYSIS: Univariate analysis processed by Windostat Version 9.2 was used to correlate ultrasound and MRI findings of rotator cuff tears with surgical (arthroscopy/open surgery) findings by using the Chi-Square and t-test. Sensitivity, specificity, positive, and negative predictive values were calculated. Sensitivity, specificity, positive and negative predictive values of USG is 0.8919, 0.8947, 0.9429, 0.8095 and of MRI is 0.9459, 1.0000, 1.0000, 0.9048 respectively
**Conclusion:** Among the cases, dominant arm, increasing age, male gender, and history of trauma were commonly associated with rotator cuff tears. Also, glenohumeral joint effusion, bursal effusion, degenerative changes were commonly seen associated with rotator cuff tears. Among young individuals, trauma was the common causative factor, and in elderly degenerative changes. From the present prospective study of 56 patients following inferences were drawn MRI (sensitivity-0.9459, specificity-1.00) is more sensitive and specific than USG (sensitivity-0.8919, specificity 0.8947) for detecting rotator cuff tears.
**Limitations:** There were a few limitations.
**Ethics Committee Approval:** Yes
**Funding for this study:** by Insititute
**Author Disclosures:**
Vikash Rustagi : Speaker: TNMC and BYL Nair Charitable Hospital, Mumbai

### RPS 310-4

**Fast field echo resembling a CT using restricted echo-spacing (FRACTURE): a novel MRI technique with superior bone contrast**

*H. Alizai*, B. Johnson, M. Dempsey; Dallas, TX/US

**Purpose:** Objective. Computerized tomography (CT) is the modality of choice for imaging bone, however, it utilizes ionizing radiation and suffers from poor soft-tissue contrast. Unlike CT, magnetic resonance imaging (MRI) provides excellent soft-tissue contrast but is limited in its ability to image bone. The objective of this study is to describe a new technical innovation that provides superior cortical and trabecular bone contrast on MRI.
**Methods or Background:** FRACTURE (FFE Resembling A CT Using Restricted Echo-spacing), a 3D gradient echo pulse sequence with restricted echo-spacing combined with an automated post-processing is described.
**Results or Findings:** Cases demonstrating the application and utility of this technique in diagnostic MRI performed for traumatic, inflammatory, neoplastic, and developmental conditions in pediatric patients are presented.
**Conclusion:** The cortical and trabecular bone contrast generated by FRACTURE yields clinically relevant information for diagnosis and management of a subset of patients in whom it may potentially obviate the need for a pre-operative CT scan.
**Limitations:** This is a technical report with a small number of cases presented.
**Ethics Committee Approval:** Local IRB approval was obtained.
**Funding for this study:** Not applicable.
**Author Disclosures:**
Hamza Alizai: Nothing to disclose
Brian Johnson: Employee: Philips Healthcare
Molly Dempsey: Nothing to disclose

### RPS 310-5

**Is MRI or CT more accurate in diagnosing Chopart joint injuries?**

*R. Janssen*, S-R. Yang, S. Manneck, S. Eiden, F. Amsler, J. E. Gehweiler, A. Hirschmann; Basel/CH
*(ru1jans2@gmail.com)*

**Purpose:** Is MRI or CT more accurate in diagnosing Chopart joint injuries?
**Methods or Background:** Twenty-two patients (mean age 43.7±14 years) were included after an acute Chopart joint injury. MRI and CT were retrospectively assessed by three radiologists. On CT avulsion fractures of the Chopart joint ligament insertions were evaluated. On MRI ligament integrity was assessed as follows: spring, plantar, bifurcate, dorsal calcaneocuboid and dorsal talonavicular ligaments . The McNemar-test was used to assess statistical differences ($p<0.05$). Kappa (κ) statistics were used to evaluate interrater agreement including 44 control patients without a history of recent Chopart-joint trauma.
**Results or Findings:** There was no difference in the detection of avulsion and full thickness tear between CT and MRI (p=0.125-1.000). On MRI full-thickness tears were seen in 45% (10/22) at the dorsal calcaneocuboid ligament and dorsal talonavicular ligament, and in 50% (11/22) at the bifurcate ligament. Although the spring ligament was affected in 45% there was only one full thickness tear of the medioplantar oblique component. No complete tear of the plantar ligaments were seen. On CT avulsions of the ligaments were detected in 45% (10/22) at the dorsal calcaneocuboid and the bifurcate, in 27% (6/22) at the dorsal talonavicular and in 9% (2/22) at the plantar ligaments. With exception of the plantar ligaments (CT κ, -0.038-0.482; MR κ, 0.313-0.579), interrater agreement was marginally higher on CT (κ, 0.616-1.00) than MRI (0.295-0.776).
**Conclusion:** No significant difference in the detection of Chopart-joint injuries on CT or MRI could be found. MRI may be useful in detecting associated injuries of the spring ligament complex.
**Limitations:** Time interval between injury, CT and MRI varied and already healed lesions may have been evident.
**Ethics Committee Approval:** Institutional review board approval was waived for this retrospective study.
**Funding for this study:** Unfunded.
**Author Disclosures:**
Schu-Ren Yang: Nothing to disclose
Julian Emanuel Gehweiler: Nothing to disclose
Sebastian Manneck: Nothing to disclose
Anna Hirschmann: Nothing to disclose
Ruben Janssen: Nothing to disclose
Felix Amsler: Nothing to disclose
Sebastian Eiden: Nothing to disclose

### RPS 310-6

**High-resolution ultrasound of the anterior talofibular ligament components: normal anatomy and pathologic findings in sprained ankles**

*S. Sanguinetti*[1], F. Zaottini[1], R. Picasso[1], F. Pistoia[1], M. Pansecchi[1], L. Tovt[1], M. Miguel-Pérez[2], C. Martinoli[1]; [1]Genova/IT, [2]Barcelona/ES

**Purpose:** The anterior talofibular ligament (ATFL) consists of two distinct components: a thicker superior band that lies underneath the capsule, a thinned inferior band, extraarticular and connected to the calcaneofibular ligament. The aim of this study was to evaluate the US appearance of the above fascicles and to describe their abnormalities in sprained ankles.
**Methods or Background:** Latex injection of the two ATFL components from n=10 fresh-frozen unembalmed cadaveric specimens was performed under US guidance to determine whether US is accurate to localize the two components. The number and thickness of the ATFL bands were then recorded by three readers in a series of n=20 healthy volunteers. Lastly, n=20 consecutive patients (age range, 25-54yrs) who underwent an ATFL inversion injury were evaluated. US was performed with matrix 24-8MHz US technology. Micro- and overt lateral ankle instability was measured blindly by an orthopedic surgeon.
**Results or Findings:** Cadaveric correlation showed the ATFL as single-banded (n=1), double-banded (n=8) or triple-banded (n=1). In double-banded ATFL, the superior band was twice wider and thicker than the inferior band. In healthy volunteers, the ATFL components were visualized with substantial agreement among readers (k=0,78). In the patients' group, the most common injury patterns affected the two components (65%) or the superior fascicle (35%). A higher degree of lateral ankle instability was observed in double banded tears.
**Conclusion:** High-resolution US is accurate to depict the ATFL components in normal and pathologic states.
**Limitations:** None
**Ethics Committee Approval:** The study was conducted in accordance with the declaration of Helsinki.
**Funding for this study:** None
**Author Disclosures:**
Federico Zaottini: Nothing to disclose
Carlo Martinoli: Nothing to disclose
Luca Tovt: Nothing to disclose
Maribel Miguel-Pérez: Nothing to disclose
Michelle Pansecchi: Nothing to disclose
Sara Sanguinetti: Nothing to disclose
Riccardo Picasso: Nothing to disclose
Federico Pistoia: Nothing to disclose

### RPS 310-7

**Role of diffusion tensor imaging in anterior cruciate ligament injury: a case control study**

*A. M. R. Khalil*, R. S. Aboukhadra; Tanta/EG
*(lolo20-12-1986@hotmail.com)*

**Purpose:** The purpose of this study was to evaluate quantitively the role of DTI to delineate ACL fibres by using a 1.5T MRI unit for grading of ACL injury.
**Methods or Background:** DTI of the knee was done (b = 0, 400, and 800 s/mm2, 10 diffusion directions, repeated 16 times for a total of 336 diffusion-weighted volumes) was performed at 1.5Tesla MRI unit for 30 patients and 20 persons (control group). Fractional anisotropy (FA), mean diffusivity (MD), axial diffusivity (AD), and radial diffusivity (RD) were calculated within the ACL fibres. After image acquisition, tractography was performed by two independent trainee radiologists (of 15 years and 10 years' experience in Musculoskeletal imaging respectively)
**Results or Findings:** Out of 30 patients in cases group; 13 patients (43.3%) had low grade tear of ACL fibres and 17 patients (65.6%) had high grade tear of ACL fibres. Another control group (n=20) was included in the study for comparison. FA ($r = -0.87$, $P < 0.05$) and apparent diffusion coefficient (ADC) ($r = 0.82$, $P < 0.05$) were significantly correlated with the severity of ACL injuries. The area under the curve values for differentiation between low and high-grade ACL injuries with FA and ADC were 0.972 and 0.961, respectively. No significant differences in FA and ADC between grades I and II ACL injuries or in ADC between grades III and IV, but there were significant differences in FA and ADC between two grades and between control group and different ACL injury grades.($P < 0.05$).
**Conclusion:** DTI had important role in quantitative assessment and grading of ACL injuries.
**Limitations:** No limitations for this study
**Ethics Committee Approval:** The study was approved by the research ethical committee of Tanta University (23451/3/10)
**Funding for this study:** No funding for the study
**Author Disclosures:**
Alaa Mohammed Reda Khalil: Nothing to disclose
Rania Sobhi Aboukhadra: Nothing to disclose

### Research Presentation Session: Musculoskeletal

### RPS 410

### Bone and muscle tumours

### RPS 410-1

**Role of dynamic contrast enhanced magnetic resonance imaging in differentiating benign bone tumours and tumour like conditions from malignant bone tumours**

*V. Jirankali*[1], P. Aggarwal[2], S. K. Garg[2], R. Kundu[2]; [1]Ballari/IN, [2]Chandigarh/IN
*(vivekjirankali@gmail.com)*

**Purpose:** To evaluate the usefulness of Dynamic Contrast Enhanced Magnetic Resonance Imaging in differentiating benign bone tumours and tumour like conditions from malignant bone tumours.
**Methods or Background:** Patients referred from Orthopaedic OPD to the department of Radiodiagnosis for MRI of the lesion in bone on x-ray, suspicious of a tumour/tumour like condition were taken into study. Sample size is 100. Dynamic Contrast Enhanced Magnetic Resonance Imaging (DCE-MRI) was done. Pattern of enhancement of curves (Time intensity curves) were assessed which were classified into five types.
**Results or Findings:** 18/42 (43%) benign bone tumours showed type 2 curve, 14/42 (33%) benign bone tumours showed type 4 curve and 10/42 (24%) benign tumours showed type 3 curve. 52/58 (90 %) malignant bone tumours showed type 3 curve, 4/58 (7 %) malignant tumours showed type 4 curve and 2/58 (3 %) malignant tumour showed type 2 curve. Association is statistically significant (p value < 0.001). Twenty-six malignant tumours revealed rapid early enhancement followed by a plateau phase (type 3 curve). A sensitivity of 89.7 % and specificity of 76.2 % was noted when this parameter was used to differentiate benign bone tumours and tumour like conditions from malignant bone tumours. Positive predictive value, Negative predictive value and total predictive values are 83.9 %, 84.2 % and 84 % respectively.
**Conclusion:** DCE-MRI is highly sensitive and specific with good overall accuracy in differentiating benign tumours and tumour like conditions from malignant bone tumours.

**Limitations:** The quantitative parameters of DCE-MRI were not evaluated in our study and only qualitative study was done - assessing the pattern of enhancement (type of curve).
**Ethics Committee Approval:** Institutional ethics committee approval was obtained.
**Funding for this study:** n/a
**Author Disclosures:**
Sudhir Kumar Garg: Nothing to disclose
Reetu Kundu: Nothing to disclose
Purnima Aggarwal: Nothing to disclose
Vivek Jirankali: nothing to disclose

### RPS 410-2

**Long-term diagnostic value of MRI in detecting recurrent aggressive fibromatosis at two multidisciplinary sarcoma centres**
*S. Sedaghat*[1], M. Both[1], O. Jansen[1], B. Reichardt[2]; [1]Kiel/DE, [2]Essen/DE
*(samsedaghat1@gmail.com)*

**Purpose:** To assess the diagnostic accuracy of MRI in detecting recurrent aggressive fibromatosis (AF) during long-term follow-up at two multidisciplinary sarcoma centers.
**Methods or Background:** Seventy-nine patients from two sarcoma centers were included in this IRB-approved study and were examined postoperatively using 1.5-T MRI. MRI follow-up scans were reviewed for true-positive/-negative and false-positive/-negative results. Available pathological reports and MRI follow-ups were set as reference.
**Results or Findings:** The median age of the patients was 38.1±15.3 years. Of the patients 26.6% showed recurrent AF lesions. The most common localizations of AF were the axilla/shoulder (n=15) and the thigh (n=11). From 498 postoperative MRI follow-ups, 24 true-positive, 16 false-positive, 6 false-negative, and 452 true-negative MRI follow-ups were identified. The overall sensitivity and specificity for detecting recurrent AF was 80% and 97%, respectively. There was no significant difference in the diagnostic accuracy at the two sarcoma centers. All false-negative results were found in small lesions. False-positive results mostly mimicked streaky (n=9) and small ovoid/nodular (n=5) lesions. The configuration of recurrent AF was significantly most often fascicular (50%; p=0.001-0.005).
**Conclusion:** MRI shows a high long-term diagnostic value in detecting AF recurrences. Nevertheless, radiologists should pay close attention when lesions are small, as they may remain undetected. Although the configuration of recurrent AF is most often fascicular, recurrences may also appear in different shapes.
**Limitations:** The main limitation of our study is the retrospective design. In many patients in whom biopsy was not performed, MRI follow-ups were set as reference.
**Ethics Committee Approval:** The study was approved by the local institutional review board (IRB).
**Funding for this study:** None.
**Author Disclosures:**
Sam Sedaghat: Nothing to disclose
Marcus Both: Nothing to disclose
Benjamin Reichardt: Nothing to disclose
Olav Jansen: Nothing to disclose

### RPS 410-4

**Accuracy of clinical diagnosis, imaging and biopsy in hand tumours and pseudotumours**
*F. Ruiz Santiago*, A. Martinez Martinez, J. Garcia Espinosa, A. J. Lainez Ramos-Bossini; Granada/ES
*(ferusan12@gmail.com)*

**Purpose:** To present the experience in our institution in the management of tumors and pseudo-tumors of the hand and to review the utility of different imaging methods in their diagnosis.
**Methods or Background:** 150 patients with pathological or microbiological diagnosis by surgery (n = 100) and/or core needle biopsy (n = 48), or puncture-drainage (25) were included. Of these 150 cases, 18 proved to be infectious, metabolic or inflammatory processes, and 132 were diagnosed as tumours or pseudo-tumours of the hand (85 from soft tissues and 47 from the bone). The accuracy of clinical and imaging diagnosis was evaluated, considering a "correct diagnosis" if the final pathological/microbiological diagnosis had been included among the differential diagnoses of the radiology or clinical report, respectively.
**Results or Findings:** The overall accuracy for each approach was: 25.3 % clinical, 71.9 % US, 81.8 % MRI, 87% CT, and 47.9% radiography. For soft tissue masses, the overall accuracy was: 35.3% clinical, 72.4 % US, 78.1 % MRI, 63.6% CT, and 25.0% radiography. For bone tumours: 4.2% clinical, 66.7% US, 89.2% MRI, 94.3 % CT, 83.0% radiography. A core needle biopsy was performed in 48 cases (39 US-guided and 9 CT-guided), with 32 true positives, 2 false negatives and 14 true negatives for tumour conditions. The overall diagnostic accuracy of biopsies was 95.8 %, 94.1% in soft tissue masses and 100% in bone tumours.
**Conclusion:** Imaging outperforms clinical diagnosis in terms of accuracy and should be mandatory before any interventional procedure. Imaging-guided biopsy is an efficient complementary technique when doubts persist about the nature or malignancy of the tumor.
**Limitations:** This is a retrospective study but intended to reflect real-world clinical practice.
**Ethics Committee Approval:** This study was approved by the local ethics committee (code TFG-EITM-2020).
**Funding for this study:** No funding
**Author Disclosures:**
Fernando Ruiz Santiago: Nothing to disclose
Jade Garcia Espinosa: Nothing to disclose
Alberto Martinez Martinez: Nothing to disclose
Antonio Jesus Lainez Ramos-Bossini: Nothing to disclose

### RPS 410-5

**Systematic analysis of post-treatment soft-tissue oedema and seroma on MRI in 177 sarcoma patients**
*S. Sedaghat*, O. Jansen, M. Both; Kiel/DE
*(samsedaghat1@gmail.com)*

**Purpose:** To assess post-treatment subcutaneous edema, muscle edema, and seroma in MRI after soft-tissue sarcoma (STS) resection with regard to muscle involvement of STS and therapy.
**Methods or Background:** 177 patients were included and received 1.5-T MRI follow-up after treatment. Posttreatment changes were classified according to type of therapy (therapy 1-surgery; therapy 2-surgery with radiation therapy) and primary tumor localization in soft tissue (localization 1, subcutaneous tissue; localization 2, muscle involvement). Subcutaneous and muscle edema were divided into three grades: grade 0, no edema; grade 1, low-to moderate edema; and grade 2, high-grade edema.
**Results or Findings:** After therapy 1 of a sarcoma in localization 1, patients significantly more often showed low-grade subcutaneous tissue edema and an absence of muscle edema ($p < 0.001$) than high-grade edema. The risk for grade 2 subcutaneous tissue and muscle edema significantly increased with a tumor in localization 2 (RR = 2.58, p = 0.016 and RR = 15, p = 0.0065/RR = 2.05 , p = 0.021, respectively) and after therapy 2 (RR = 15, p = 0.0087 and RR = 2.05, $p < 0.0001$, respectively). Of the patients with sarcoma in localization 2, 88% developed grade 2 muscle edema after therapy 2; 40% of the patients developed post-treatment seroma. The risk for seroma is significantly higher after surgery and radiation therapy than after surgery alone ($p < 0.001$).
**Conclusion:** High-grade postoperative subcutaneous and muscle edema are significantly associated with muscle involvement of primary STS both in patients with and without radiation therapy. The risk for seroma is significantly higher after surgery with additional radiation therapy than after surgery alone.
**Limitations:** The retrospective design is the main limitation of the current study.
**Ethics Committee Approval:** The study was approved by the responsible Ethics Committee.
**Funding for this study:** None.
**Author Disclosures:**
Sam Sedaghat: Nothing to disclose
Marcus Both: Nothing to disclose
Olav Jansen: Nothing to disclose

### RPS 410-6

**Magnetic resonance and ultrasound imaging findings of soft tissue lesions referred to a tertiary sarcoma centre: a pictorial review**
*A. Mermekli*, R. Chowdhury, R. Mansour; Oxford/UK

**Purpose:** The aim of this presentation is to demonstrate the variety of soft tissue lesions, which are referred to a tertiary sarcoma centre and to correlate their Magnetic Resonance Imaging (MRI) and Ultrasound (US) characteristics.
**Methods or Background:** All US guided soft tissue biopsies, which were performed during 12 months , via the dedicated sarcoma clinic pathway, were analysed. The respective MRI and US images were reviewed, as well as their histopathology reports.
**Results or Findings:** 146 US guided soft tissue biopsies were performed in one year via the dedicated sarcoma clinic service. The biopsied lesions included malignant and benign causes, such as high grade sarcoma (15.7%), metastases (7.5%), vascular malformations (6.2%), infection/inflammation (5.5%), fibrous/fibrofatty tissue (5.5.%), low grade myxoid tumours (5.5%), lipomata (3.4%), schwannomas (3.4%), endometriosis (2.7%), epidermoid cysts (2%) and several more.
**Conclusion:** We review the imaging characteristics of the biopsied lesions, with a view to increasing the diagnostic accuracy and reducing the number of potentially unnecessary biopsies for benign causes.

**Limitations:** Review of a larger sample may provide us with a greater variety of lesions and be more representative of our diagnostic yield.
**Ethics Committee Approval:** Not applicable.
**Funding for this study:** Not applicable.
**Author Disclosures:**
Ramy Mansour: Nothing to disclose
Rajat Chowdhury: Nothing to disclose
Asimenia Mermekli: Nothing to disclose

### RPS 410-7

**Diffusion-weighted MRI radiomics classification of spinal bone tumours**
*F. Serpi*, S. Gitto, M. Bologna, I. Emili, D. Albano, C. Messina, V. Corino, L. Mainardi, L. M. Sconfienza; Milan/IT
*(francesca.serpi@unimi.it)*

**Purpose:** The objective of this study was to classify spine bone tumors using diffusion
**Methods or Background:** This local ethics committee-approved retrospective study included 101 patients with histology-proven bone tumor of the spine (22 benign; 38 primary malignant; 41 metastatic). All tumor volumes were manually segmented by a radiology resident experienced in musculoskeletal and oncologic imaging on morphologic T2-weighted sequences. The same region of interest (ROI) was used to perform radiomic analysis on apparent diffusion coefficient (ADC) map. A total of 1702 radiomic features was considered. Feature stability was assessed through small geometrical transformations of the ROIs mimicking multiple manual delineations. Intraclass correlation coefficient (ICC) quantified feature stability. Feature selection consisted of stability-based (ICC>0.75) and significance-based selections (ranking features by decreasing Mann-Whitney p-value). Class balancing was performed to oversample the minority (i.e. benign) class. Selected features were finally used to train and test a support vector machine (SVM) to discriminate benign from malignant spine tumors using 10-fold cross validation.
**Results or Findings:** A total of 76.4% radiomic features were stable. The quality metrics for the SVM were evaluated as a function of the number of selected features. The radiomic model with the best performance and the lowest number of features for classifying tumor types included 8 features. The metrics were 78% sensitivity, 68% specificity, 76% accuracy, and AUC 0.78.
**Conclusion:** SVM classifiers based on radiomic features extracted from T2- and diffusion-weighted imaging with ADC map are promising for classification of spine bone tumors. Radiomic features of spine bone tumors show good reproducibility rates.
**Limitations:** Sample size.
**Ethics Committee Approval:** Due to the retrospective nature of this study, specific informed consent was waived.
**Funding for this study:** This research received no external funding.
**Author Disclosures:**
Valentina Corino: Nothing to disclose
Luca Mainardi: Nothing to disclose
Ilaria Emili: Nothing to disclose
Carmelo Messina: Nothing to disclose
Salvatore Gitto: Nothing to disclose
Luca Maria Sconfienza: Nothing to disclose
Marco Bologna: Nothing to disclose
Francesca Serpi: Nothing to disclose
Domenico Albano: Nothing to disclose

### Research Presentation Session: Musculoskeletal

### RPS 510

### Bone density and muscle mass

### RPS 510-1

**Decreased muscle mass can predict worse outcome in COVID-19 patients: a retrospective multicentre study**
*S. Schiaffino*[1], D. Albano[1], A. Cozzi[1], C. Messina[1], C. Bnà[2], A. Carriero[3], A. Vanzulli[1], F. Sardanelli[1], L. M. Sconfienza[1]; [1]Milan/IT, [2]Brescia/IT, [3]Novara/IT
*(schiaffino.simone@gmail.com)*

**Purpose:** To investigate the potential association between muscle mass status retrieved from chest CTs and clinical outcomes (i.e. intensive care unit [ICU] admission and death) in COVID-19 patients.
**Methods or Background:** Clinical data, CT findings and clinical outcomes were retrospectively retrieved for COVID-19 patients who underwent chest CT on admission in four hospitals in Northern Italy from February 21 to April 30, 2020. Pulmonary involvement, mediastinal lymphadenopathy, pleural effusion, attenuation and cross-sectional areas of paravertebral muscles on axial CT images at T5 and T12 vertebral level were assessed. Associations between variables in predicting ICU admission or death were investigated through multivariate linear regression. Binary logistic regression was used to obtain a predictive model, including odds ratio (OR) calculation. Performance of these models in predicting outcomes was assessed by applying them to both a training and test set, using receiver operating characteristic curve (ROC) analysis.
**Results or Findings:** We included 552 patients (364 men; median age 65 years, IQR 54–75). Muscle area at T5 level (OR=4.50; P<.001), pleural effusion (OR=3.00; P=.040), and lung involvement extent (OR=2.12; P<.001) were the best predictors of ICU admission (AUC 0.79 in training and 0.75 in test set). Muscle area at T5 level (OR=2.26; P=.005), male sex (OR=2.24; P=.012), and lung involvement extent (OR=1.61; P=.001) were the best predictors of death (AUC was 0.82 in training and 0.82 in test set).
**Conclusion:** Decreased muscle mass assessed through CT exams performed at emergency department admission is an independent predictor of ICU admission and death in COVID-19 patients.
**Limitations:** Retrospective study; high disease prevalence and disease severity during the study period; incomplete data for part of the patients.
**Ethics Committee Approval:** Approval from the Ethics Committee of each institution was obtained.
**Funding for this study:** Partially supported by the Italian Ministry of Health.
**Author Disclosures:**
Andrea Cozzi: Nothing to disclose
Claudio Bnà: Nothing to disclose
Francesco Sardanelli: Advisory Board: Bracco Advisory Board: Bayer Advisory Board: GE Healthcare
Simone Schiaffino: Speaker: GE Healthcare
Angelo Vanzulli: Nothing to disclose
Carmelo Messina: Nothing to disclose
Luca Maria Sconfienza: Nothing to disclose
Alessandro Carriero: Nothing to disclose
Domenico Albano: Nothing to disclose

### RPS 510-2

**Short-term precision error of bone strain index, a new DXA-based finite element analysis software for hip strength assessment**
*S. Tortora*, M. Acquasanta , E. Carlicchi, D. Albano, L. M. Sconfienza, C. Messina; Milan/IT

**Purpose:** Bone Strain Index (BSI) is a new finite element analysis (FEA) tool applied to hip dual energy X-ray absorptiometry (DXA) scans. Aim of this study is to assess the short-term precision error of BSI on the proximal femur, both on a phantom and patients.
**Methods or Background:** We followed the International Society for Clinical Densitometry guidelines followed for short-term precision error assessment. We performed DXA measurements on an anthropomorphic femur phantom that was scanned twice for 30 times, for a total of 60 scans. For patients' study, 30 subjects were scanned twice. BSI precision error was compared to that of bone mineral density (BMD).
**Results or Findings:** BSI reproducibility was lower compared to that of BMD both for the in-vivo and phantom study, as the precision error of BSI resulted three times higher compared to that of BMD. For the in-vivo study, the highest precision was found at total femur (TF) BMD (CoV = 1.36%, reproducibility = 96.22%), while the lowest value of precision was found for femoral neck (FN) BSI (CoV = 4.17%, reproducibility = 88.46%). For phantom measurements, the highest precision value was that of TF BMD (CoV = 0.63%, reproducibility = 98.24%), while the lowest precision was that of FN BSI (CoV = 3.08%, reproducibility = 91.48%). Reproducibility at TF was always better compared to that of the FN.
**Conclusion:** BSI precision error was about three times higher compared to BMD, confirming previous results of lumbar spine BSI. The main source of variability of this new software is supposed to be related to patient positioning.
**Limitations:** Heterogeneous patient group in terms of age, BMD and T-score values.
**Ethics Committee Approval:** Not needed. For patients' study all scans were performed during the periodic institutional precision error calculation.
**Funding for this study:** None
**Author Disclosures:**
Marzia Acquasanta: Nothing to disclose
Eleonora Carlicchi: Nothing to disclose
Carmelo Messina: Nothing to disclose
Luca Maria Sconfienza: Nothing to disclose
Silvia Tortora: Nothing to disclose
Domenico Albano: Nothing to disclose

### RPS 510-3

**Use of the fragility score measured at the femoral neck for the prediction of fragility incident fractures**

*F. A. Lombardi*, P. Pisani, D. Ciardo, M. Di Paola, F. Conversano, S. Casciaro; Lecce/IT
*(fiorella@ifc.cnr.it)*

**Purpose:** The Fragility Score (FS), calculated by Radiofrequency Echographic Multi Spectrometry (REMS) technology, estimates skeletal fragility regardless of bone mineral density (BMD). It is a dimensionless parameter from 0 to 100, proportional to the degree of fragility (Measurement 2017;101:243–249). This study evaluates the effectiveness of FS in identifying patients at risk for incident osteoporotic femoral fracture (OP-FF), and compares the FS performance with T-score ones.
**Methods or Background:** REMS and DXA scans of the femoral neck were performed in women aged between 30 and 90 years. The incidence of fragility femoral fractures was assessed during a 5-year follow-up. The ability of the FS to identify patients with incident OP-FF was evaluated and compared with the performance of T-scores obtained with DXA and REMS.
**Results or Findings:** 414 women completed the follow-up (median 40 months, interquartile range (IQR): 36-57 months). Of these, 22 patients had an incident OP-FF during follow-up. The median FS for patients with and without an incident OP-FF were 25.3 (IQR: 21.0-33.0) and 42.8 (IQR: 30.4-54.7), respectively, and the difference was statistically significant (p<0.001). Also REMS T-score were lower in patients with incident OP-FF (median value -2.5, IQR: -2.8 to -1.8) than in patients without incident OP-FF (median value -1.8 IQR: -2.4 to -1.2), with p-value=0.013, and similarly for DXA T-score values (-2.1 (IQR: -3.0 to -1.5) versus -1.7 (IQR: -2.4 to -1.2), respectively, p-value=0.033). At ROC curve analysis, the area under the curve (AUC) for FS was 0.80, statistically significantly higher than the REMS T-score AUC (0.65) and DXA T-score AUC (0.63).
**Conclusion:** The FS obtained from REMS femoral scans is an effective tool for identifying the risk of femoral fracture, with performance superior than the T-score' ones.
**Limitations:** None.
**Ethics Committee Approval:** Approved.
**Funding for this study:** None.
**Author Disclosures:**
Francesco Conversano: Nothing to disclose
Sergio Casciaro: Nothing to disclose
Delia Ciardo: Nothing to disclose
Fiorella Anna Lombardi: Nothing to disclose
Marco Di Paola: Nothing to disclose
Paola Pisani: Nothing to disclose

### RPS 510-4

**Muscle involvement in a familiar form of tubular aggregates myopathy: comparison between radiologic and radiomic analysis**

A. Lupi, *A. Favero*, S. M. Tripodi, C. Giraudo, E. Pegoraro, R. Stramare; Padua/IT
*(alessandrofavero6@gmail.com)*

**Purpose:** Congenital myopathies are a heterogeneous group of diseases affecting the skeletal muscles and characterized by high clinical, genetic and histological variability. In this scenario, magnetic resonance (MR) is becoming increasingly important, leading to the identification of the pathognomonic patterns and to the assessment of disease progression at follow-up. The aim of this study is to evaluate the MR pattern of a mutation responsible for tubular aggregates myopathy (TAM) and to determine if radiomics may identify progression of disease.
**Methods or Background:** A family affected by TAM with the histologically proven autosomal dominant mutation of the STIM1 gene, was examined. All patients underwent two pelvic girdle and lower limbs MR assessment (i.e. A and B, the latter after 5 years): twenty-two muscles were evaluated for muscular fatty replacement applying the modified Mercuri scale on the T1w images, and for edema with a four-points score (absent=0,low=1,moderate=2, severe=3) on the STIR images, to determine muscle involvement and disease progression. All dataset was extracted by two raters and inter-rater reliability was calculated. Moreover, 58 radiomic features were collected (i.e. using 3DSlicer) and t-test was performed to analyze differences between A and B MR (p<0.05).
**Results or Findings:** Six patients with TAM STIM1-mutation were included (three males and three females). Coehn's k showed substantial agreement between the two raters. In the T1w sequences, all patients showed widespread involvement (median value of the Mercuri score=2) and progression, while muscle oedema mainly affected the muscles of the legs and it was of low grade (median value of the score=1) and stable at follow-up. Two radiomic features showed statistically significant differences between A and B MR assessments.
**Conclusion:** Muscle MR and radiomics are valid tools to map muscle involvement and disease progression in STIM1 patients.
**Limitations:** Further analysis are needed to confirm these results.
**Ethics Committee Approval:** n/a
**Funding for this study:** n/a
**Author Disclosures:**
Amalia Lupi: Nothing to disclose
Silvia Maria Tripodi: Nothing to disclose
Roberto Stramare: Nothing to disclose
Alessandro Favero: Nothing to disclose
Chiara Giraudo: Nothing to disclose
Elena Pegoraro: Nothing to disclose

### RPS 510-5

**Myosteatosis is a potential indicator to predict the risk of transition to severe disease in COVID-19 patients**

*X. Yi*, W. Liao; Changsha/CN
*(yixiaoping@csu.edu.cn)*

**Purpose:** To evaluate whether myosteatosis can serve as an indicator for predicting the risk of transition to severe disease before admission among patients with COVID-19.
**Methods or Background:** A total of 234 laboratory-confirmed COVID-19 patients with initial common subtype disease at admission, with 31 of which developed to severe status during hospitalization, were retrospectively recruited between January 21th 2020 and February 19th 2020 from six designed hospitals (H1-6) in Hunan province, China. Myosteatosis features was evaluated based on initial routine chest CT at the level of twelfth thoracic vertebra. The myosteatosis nomogram was constructed by multivariable logistic regression. The performance of the nomogram was evaluated by discrimination, calibration curve, and decision curve.
**Results or Findings:** The myosteatosis features, including SM-RA and PSMFI_score fitted by PSMFI, age and gender, were significantly associated with transition risk (P<0.01 for both training and validation cohorts). The combination nomogram based on SM-RA, PSMFI_score and clinical model (CRP, SPO2 and CKP), showed better discrimination with an AUC of 0.85 [95% CI, 0.75~0.95] (and 0.84 [95% CI, 0.71 to 0.97]in validation cohort, when comparing to clinical model (0.75 in training vs. 0.74 in validation cohort). Favorable clinical utility was observed using decision curve analysis.
**Conclusion:** The myosteatosis nomogram developed in the present study provided an individualized prediction of transition risk to severe disease and helped decision making and guidance of intensive treatment for COVID-19 patients.
**Limitations:** Retrospective nature of the study
**Ethics Committee Approval:** Xiangya hospital, central south university. IRB#: 202002019
**Funding for this study:** None
**Author Disclosures:**
Weihua Liao: Nothing to disclose
Xiaoping Yi: Nothing to disclose

### RPS 510-6

**MR-based Proton-Density Fat Fraction (PDFF) differentiates between patients with and without osteoporotic vertebral fractures**

*F. T. Gassert*, F. G. Gassert, Y. N. Leonhardt, S. Kronthaler, B. J. Schwaiger, J. S. Kirschke, T. Baum, D. C. Karampinos, A. Gersing; Munich/DE

**Purpose:** To evaluate the diagnostic value of bone marrow Proton-Density Fat Fraction (PDFF) in patients with and without osteoporotic vertebral fractures and to assess whether PDFF values are a useful tool to differentiate between osteoporotic and traumatic vertebral fractures.
**Methods or Background:** Overall, 52 patients were included in this study (mean age 67.5±12.9 years, 34 women) of which 32 patients presented with one or multiple vertebral fractures of the lumbar spine (66.4±14.4y, 20 women). These patients were frequency-matched for age and sex to patients without vertebral fractures (n=20, 69.3±10.1 years, 14 women). Bone mineral density (BMD) values were derived from quantitative CT. Patients were categorized into a normal BMD group (BMD>120g/cm3) and an osteopenic/osteoporotic group (BMD<120g/cm3). A six-echo 3D spoiled gradient-echo sequence was acquired at 3T. Chemical shift encoding-based water-fat separation was performed considering a single T2* decay and PDFF maps were calculated. Fractured vertebrae were excluded. Associations between fracture status and PDFF values were assessed using multivariate linear regression models adjusting for age, sex and BMD.
**Results or Findings:** Over all patients, there was a significant correlation between the mean PDFF and BMD (r=-0.664, P<0.001), suggesting high PDFF in patients with low BMD. In the osteopenic/osteoporotic group, those patients with osteoporotic fractures had a significantly higher PDFF than those without osteoporotic fractures (adjusted mean difference [95% confidence interval] between groups: 0.20 [0.13, 0.27]; P<0.001). In patients with vertebral fractures, those with traumatic fractures had a significantly lower mean PDFF than those with osteoporotic vertebral fractures (P<0.001).

**Conclusion:** MR-based PDFF values may serve as a biomarker for osteoporosis. PDFF enables a differentiation between patients with and without osteoporotic vertebral fractures and may be useful for the differentiation between osteoporotic and traumatic vertebral fractures.
**Limitations:** n/a
**Ethics Committee Approval:** n/a
**Funding for this study:** n/a
**Author Disclosures:**
Benedikt Jakob Schwaiger: Nothing to disclose
Florian Tilman Gassert: Nothing to disclose
Alexandra Gersing: Nothing to disclose
Jan S Kirschke: Nothing to disclose
Felix Gerhard Gassert: Nothing to disclose
Thomas Baum: Nothing to disclose
Sophia Kronthaler: Nothing to disclose
Dimitrios C. Karampinos: Nothing to disclose
Yannik Niklas Leonhardt: Nothing to disclose

### RPS 510-7

**Sarcopenia as a predictor of semi- and intensive care unit hospitalisation in COVID-19 patients**

C. Giraudo, *G. Fichera*, G. Librizzi, E. Balestro, A. Cattelan, P. Navalesi, A. Vianello, R. Stramare; Padua/IT
*(gfichera90@gmail.com)*

**Purpose:** To evaluate if sarcopenia, assessed with Computed Tomography, is a predictor of semi- (SICU) and intensive care unit (ICU) hospitalization in COVID-19 patients.
**Methods or Background:** We retrospectively evaluated patients with COVID-19 treated in our tertiary center from March to August 2020 who underwent an unenhanced chest CT scan during the first three weeks of hospitalization. We collected the mean Hounsfield Unit (Hu) value of the right paravertebral muscle at the level of the 12th thoracic vertebra using a 2 cm region of interest. Information regarding the unit of hospitalization considering SICU/ICU and covid-19 wards were separately recorded. We used the odds/ratio (OR) to assess if sarcopenia, defined as Hu values <30, is a predictor of SICU/ICU admission. The Fisher's exact and the student's tests were respectively applied to evaluate if any difference regarding gender and age occurred between patients with and without sarcopenia (p<0.05).
**Results or Findings:** Seventy-six patients were examined (28 females; mean age±SD 61.5±14 years old); 56 treated in covid-19 wards and 17 in SICU/ICU. Eight out of twenty-four patients with sarcopenia were hospitalized in SICU/ICU. Patients with sarcopenia showed a higher risk of SICU/ICU admission (OR= 2.389, CI 0.786 – 7.263). Patients with muscle loss were significantly older (71.5±10 vs 56.9±13.5 years old, p=0.00) whereas there was no statistically significant difference for gender between patients with and without sarcopenia (p=1.00).
**Conclusion:** According to our preliminary results, sarcopenia can be considered a predictor of SICU/ICU hospitalization in patients with COVID-19. Further analyses including a larger sample are expected to fully assess this evidence and evaluate if sarcopenia may even contribute to the distinction of the level of required intensive care (i.e., SICU or ICU).
**Limitations:** n/a
**Ethics Committee Approval:** Yes
**Funding for this study:** n/a
**Author Disclosures:**
Giulia Fichera: Nothing to disclose
Andrea Vianello: Nothing to disclose
Roberto Stramare: Nothing to disclose
Elisabetta Balestro: Nothing to disclose
Chiara Giraudo: Nothing to disclose
Paolo Navalesi: Nothing to disclose
Annamaria Cattelan: Nothing to disclose
Giovanni Librizzi: Nothing to disclose

### RPS 510-8

**Precision and repeatability of lumbar spine and proximal femur bone mineral density evaluated using ultrasound REMS technology**

*C. Messina*, S. Gitto, S. Rapisarda, A. Corazza, D. Albano, L. M. Sconfienza; Milan/IT
*(carmelomessina.md@gmail.com)*

**Purpose:** Several quantitative ultrasound methods for a non-ionizing diagnosis of osteoporosis have been developed. Radiofrequency Echographic Multi Spectrometry (REMS) is a recent ultrasound technology that allows for the evaluation of bone density on both lumbar spine (LS) and proximal femur (FEM). REMS technology assesses a new parameter, the ultrasound-BMD (US-BMD), which is expressed as grams/cm2. Our aim is to determine the short-term intra-operator precision of US-BMD and its short-term inter-operator repeatability.
**Methods or Background:** US-BMD was evaluated on LS (L1-L4) and FEM by using a dedicated ultrasound device (EchoStation, Echolight Spa, Lecce, Italy), equipped with a 3.5 MHz convex transducer. Lumbar scans were performed using longitudinal scans with the probe positioned under the sternum (scanning time 80 seconds); femur scans were performed with longitudinal scans showing the head and neck of the hip (40 seconds). Short-term intra-operator precision and inter-operator repeatability were calculated root-mean-square coefficient of variation (RMS-CV) and least significant change (LSC) for a 95% confidence level, according to the International Society for Clinical Densitometry (ISCD) official positions. Inclusion criteria were: caucasian ethnicity, age 30–90 y, BMI < 35 kg/m2.
**Results or Findings:** A total of 36 postmenopausal women were included in this study. Intra-operator precision expressed as RMS-CV was 0.48% for lumbar spine and 0.31% for femoral neck (LSC values of 1.33% and 0.87%, respectively). Inter-operator repeatability (RMS-CV) was 0.55% for lumbar spine and 0.53% for femoral neck (LSC values of 1.52% and 1.47% respectively).
**Conclusion:** US-BMD assessed with REMS can be used to precisely monitor BMD. Ongoing studies will focus on the evaluation of US-BMD accuracy compared to that of dual energy x-ray absorptiometry, still the gold standard for osteoporosis assessment.
**Limitations:** Single-center-study
**Ethics Committee Approval:** Approved by local Ethics Committee; informed consent obtained by all subjects.
**Funding for this study:** Echolight-Spa
**Author Disclosures:**
Angelo Corazza: Nothing to disclose
Carmelo Messina: Nothing to disclose
Salvatore Gitto: Nothing to disclose
Luca Maria Sconfienza: Equipment Support Recipient: Echolight Spa
Santi Rapisarda: Nothing to disclose
Domenico Albano: Nothing to disclose

### RPS 510-11

**The clinical importance of bone marrow adipose tissue ratio on sacroiliac MRI: a comprehensive study with bone mineral density**

N. Hürsoy, *E. B. Çetin*; Rize/TR

**Purpose:** Osteoporosis imaging has critical importance in determining risks for fractures and monitoring response to treatment. Studies have focused on bone quality and showed that bone marrow adiposity linkage with bone health by using conventional and advanced MR imaging techniques such as MR spectroscopy and perfusion. However, for routine clinical practice, these methods are not useful. Hence, we aimed to evaluate bone mineral density's association with bone marrow adipose tissue with a qualitative technique.
**Methods or Background:** The patients who had both sacroiliac magnetic resonance imaging and the dual-energy X-ray absorptiometry (DEXA) scan from 2016 to 2020 were listed retrospectively. DEXA results were recorded as normal, osteopenia, and osteoporosis. A specialized musculoskeletal radiologist detected the T1-Weighted axial MR slices on S1 -2 level without the knowledge of the DEXA measurements. The images were evaluated to whether the fat deposits cover more than fifty percent of the bone area subjectively.
**Results or Findings:** 121 patients with six men (%5) and 115 women (%95) with a mean age of 54.4±10.7 years were included. There was a statistically significant difference in the sacrum's bone marrow adipose tissue deposition among bone marrow density measurements (p = 0.011). Patients with osteopenia and osteoporosis had higher bone marrow adipose tissue rates than those whose T-scores were normal.
**Conclusion:** Radiologists should examine sacroiliac joint MR images in terms of bone marrow changes and indicate osteoporosis risk in their reports. The method that we have proposed allows radiologists and clinicians to know about bone mineral density in routine practice and contributes to preventing and reducing osteoporosis-related complications.
**Limitations:** The number of patients and lack of clinical data
**Ethics Committee Approval:** This study was carried out in accordance with the World Medical Association Declaration of Helsinki.
**Funding for this study:** 0
**Author Disclosures:**
Eda Beykoz Beykoz Çetin: Author: Observer
Nur Hürsoy: Author: Observer

### Research Presentation Session: Musculoskeletal

### RPS 610

### Imaging of the joints

### RPS 610-1

**Pelvic venous congestion on sacroiliac MRI: its prevalence and association with sacroiliitis**

*Ü. Aydingöz*, A. E. Yıldız, G. Aghayeva, D. İmre, F. B. Ergen; Ankara/TR
*(ustunaydingoz@yahoo.com)*

**Purpose:** Pelvic venous congestion (PVC) can cause chronic pelvic pain, which might mimic inflammatory back pain from sacroiliitis. We aimed to determine the prevalence of PVC on sacroiliac MRI and its association with sacroiliitis.
**Methods or Background:** This retrospective cross-sectional observational study covered sacroiliac MRI examinations performed for chronic inflammatory type back back pain at our institution of all females ≥15 and <45 years of age during a 48-month period. Images were reviewed for active and/or chronic sacroiliitis (according to ASAS [Assessment of SpondyloArthritis international Society] criteria) and the presence of PVC. PVC criterion was ≥4 ipsilateral tortuous dilated parauterine veins, at least one of which measuring ≥6 mm in diameter. Exclusion criteria included studies suboptimal for diameter measurement and featuring pregnancy, hysterectomy and malignant pelvic mass(es).
**Results or Findings:** A total of 794 females (mean age, 32.1 years) were included. PVC was identified in 64 patients (prevalence, 8.1%; mean age, 33.3 years; age range, 20–44). Sacroiliitis was detected in 253 patients (prevalence, 31.9%; mean age, 32.2 years; age range, 15–44). Active and/or chronic sacroiliitis was present in 21 (32.8%) of patients with PVC, and 232 patients (31.8%) without PVC (P=0.889). In patients with PVC, mean parauterine vein diameters were 7.1 mm and 7.4 mm (range, 6–12 mm, and 6–11 mm) in subgroups without and with sacroiliitis, respectively (P=0.385).
**Conclusion:** Found in 8.1% of fertile-aged females, PVC on sacroiliac MRI is independent of the presence of active and/or chronic sacroiliitis. PVC needs to be mentioned in the sacroiliac MRI report as a differential or additional diagnostic possibility in female patients at fertile age.
**Limitations:** Retrospective study; therefore menopause status, parity information and exact duration of chronic back pain symptoms were not available in all patients.
**Ethics Committee Approval:** Yes
**Funding for this study:** None
**Author Disclosures:**
Fatma Bilge Ergen: Nothing to disclose
Duygu İmre: Nothing to disclose
Adalet Elçin Yıldız: Nothing to disclose
Gülnar Aghayeva: Nothing to disclose
Üstün Aydingöz: Nothing to disclose

### RPS 610-2

**Autologous Matrix-Induced Chondrogenesis (AMIC) treatment for patellar cartilage defects: MRI imaging correlation to clinical outcome**

*A. Antón Jiménez*, R. D. O. D. Dominguez Oronoz, M. De Albert De Delas-Vigo, A. Gimeno, M. Veintemillas, L. Casas, C. Torrents, I. Portas, N. Joshi Jubert; Barcelona/ES
*(alba.antonj@gmail.com)*

**Purpose:** The aim of this study is to demonstrate correlation between MRI imaging and short-term clinical benefit in patients with isolated patellar cartilage defects treated with AMIC surgical technique in our center.
**Methods or Background:** Retrospective study included 15 consecutive patellar cartilage defects (grade 3-4 based on ICRS) treated with AMIC technique (collagen matrix and fibrin glue) in our hospital by a single surgeon. Baseline and follow-up MRI after 1 and 2 years of treatment were blinded evaluated by 2 radiologists. The cartilage target lesion was scored based on a modified MODIC scale using 9 MRI features (defect filing, surface and signal intensity of the deficit cover, bone and cartilage integration, presence of chondral osteophyte, bone marrow edema, subchondral bone state and effusion). Therapeutic efficacy was evaluated by KOOS, Kujala and VAS scores for pain pre and post treatment. Analysis of Variance (ANOVA) was implemented as feature selector and a logistic regression model was generated.
**Results or Findings:** The study included 15 knees (8 right and 7 left) in 12 patients (7 female; mean age 40,2 years; mean BWI 26,6) who underwent AMIC surgery at our Institution. None of the patients had additional surgical procedures. All lesions were non-traumatic. Main location was lateral facet (66,7%) and mean surface volume was of 0,6 cm3. After follow-up (24 months) there was a significant improvement based on clinical scores in most of the patients. From 9 MRI imaging features; 2 (defect filling and signal intensity) correlated with clinical response. Patients with higher modified MODIC score showed better clinical outcome (p<0.05).
**Conclusion:** Change of MRI features from baseline to follow-up after treatment (2 years) correlate with patient's clinical outcome after AMIC surgical technique.
**Limitations:** None.
**Ethics Committee Approval:** Approved.
**Funding for this study:** None.
**Author Disclosures:**
Alba Antón Jiménez: Nothing to disclose
Lourdes Casas: Nothing to disclose
Nayana Joshi Jubert: Nothing to disclose
Matias De Albert De Delas-Vigo: Nothing to disclose
Rosa Dominguez Oronoz Dominguez Dominguez Oronoz: Nothing to disclose
Irene Portas: Nothing to disclose
Alfredo Gimeno: Nothing to disclose
Maite Veintemillas: Nothing to disclose
Carme Torrents: Nothing to disclose

### RPS 610-3

**Choosing the right imaging: differential diagnosis at the sacroiliac joint**

*T. Diekhoff*[1], F. Radny[1], K. Ziegeler[1], D. Poddubnyy[1], F. N. Proft[1], J. Greese[1], R. Biesen[1], K-G. Hermann[1], I. A. Eshed[2]; [1]Berlin/DE, [2]Ramat Gan/IL

**Purpose:** To analyse the diagnostic accuracy of radiography (XR), computed tomography (CT) and magnetic resonance imaging (MR) for the differential diagnosis of axial spondyloarthritis (axSpA) and mechanical disease at the sacroiliac joints (SIJ).
**Methods or Background:** 166 patients underwent XR, CT and MR of the SIJ and were scored by three musculoskeletal experts for their diagnosis in the categories axSpA, mechanical disease and normal findings. The images were separately anonymised: XR, CT and MR only, MR with XR and MR with CT. Thus, every patient was evaluated five times. The readers were blinded to identifying information and clinical and laboratory findings. The final diagnosis of the rheumatology expert served as standard of reference. Interrater reliability was assessed with Fleiss' kappa.
**Results or Findings:** Ninety-one patients were diagnosed by the rheumatologist with axSpA, 57 with mechanical disease such as osteitis condensans or osteoarthritis and 18 as SIJ unrelated disease. XR showed rather low diagnostic accuracy (65.6% sensitivity/67.6% specificity) and was outperformed by CT (76.7%/97.3%) and MR (81.3%/86.7%). Adding XR to MRI reduced the sensitivity (77.8%) with only a minor increase in specificity (88.6%). However, while adding CT to MRI reduced the sensitivity (75.6%) it substantially increased the specificity (97.3%). The interrater agreement was moderate for XR (0.5), almost perfect for CT (0.88), and substantial for MR (0.67), XR+MR (0.66) and CT+MR (0.74).
**Conclusion:** Radiography is neither sensitive nor specific for the differential diagnosis in patients with suspected axSpA. MR and CT performed equally well, while MR showed higher sensitivity (81% vs 77%) but CT higher specificity (97% vs 87%) and reliability. Adding XR to MR showed no substantial effect while adding CT improved specificity at the expense of sensitivity.
**Limitations:** SOR was influenced by imaging.
**Ethics Committee Approval:** IRB approved.
**Funding for this study:** Not applicable
**Author Disclosures:**
Robert Biesen: Nothing to disclose
Denis Poddubnyy: Nothing to disclose
Torsten Diekhoff: Nothing to disclose
Katharina Ziegeler: Nothing to disclose
Fabian N. Proft: Nothing to disclose
Kay-Geert G. Hermann: Nothing to disclose
Juliane Greese: Nothing to disclose
Iris Ariel Eshed: Nothing to disclose
Felix Radny: Nothing to disclose

### RPS 610-4

**Shear-wave elastography assessment of the coracohumeral ligament following rotator interval injection in patients with adhesive capsulitis - a cohort study**

*A. Mermekli*[1], R. Te Water Naudé[2], B. McElroy[3], J. Baxter[1], A. Pendse[1], J. Papanikitas[1], J. Teh[2], R. Hughes[1], D. McKean[1]; [1]Aylesbury/UK, [2]Oxford/UK, [3]Great Missenden/UK

**Purpose:** The aim of this study is to evaluate the changes in elasticity of the coracohumeral ligament (CHL) in patients with adhesive capsulitis of the shoulder (ACS) treated with ultrasound guided rotator interval injections.
**Methods or Background:** Shear-wave elastography (SWE) was used to evaluate the elasticity of the CHL in symptomatic and asymptomatic shoulders both in neutral position and 30° external rotation. 22 shoulders were assessed. Ultrasound-guided steroid injection via the rotator interval was administered in the symptomatic shoulders, with subsequent shoulder manipulation under local anaesthetic block. The elasticity of the CHL was re-assessed, in 14 shoulders, at 10 weeks post injection.
**Results or Findings:** In all patients, the CHL elastic modulus was larger at 30° external rotation than the neutral position. In patients with ACS, the CHL thickness and elastic modulus was significantly greater in the symptomatic shoulder in both positions. Treated patients had an excellent response, which was assessed with the Oxford Shoulder Score (OSS) and Visual Analogue Scale (VAS) pain scores. This was also associated with a trend to normalisation of the elastic modulus of the CHL in the treated shoulder.
**Conclusion:** In patients with ACS, elastography demonstrated that the CHL is stiffer in the symptomatic shoulder than the unaffected shoulder. Treatment with ultrasound guided injection via the rotator interval is associated with improvement in OSS, VAS pain scores and reduced stiffness in the CHL.
**Limitations:** This is a relatively small cohort study and a larger, age and sex matched, study would provide additional information. One consistent CHL area was analysed for this study, however different locations along the CHL for SWE measurement may result in higher CHL elasticity measurements.
The rotator cuff tendons' elasticity was not measured.
**Ethics Committee Approval:** Not applicable
**Funding for this study:** Not applicable
**Author Disclosures:**
Joseph Papanikitas: Nothing to disclose
Anniruddha Pendse: Nothing to disclose
Jonathan Baxter: Nothing to disclose
James Teh: Nothing to disclose
Bernard McElroy: Nothing to disclose
Asimenia Mermekli: Nothing to disclose
Rebecca Te Water Naudé: Nothing to disclose
David McKean: Nothing to disclose
Richard Hughes: Nothing to disclose

### RPS 610-5

**Diagnostic value of MRI symptoms in patients with diabetic arthropathy and osteomyelitis: a clinical follow-up analysis**

*A. Ageev*, J. Babushkina, E. Burleva; Ekaterinburg/RU
*(ageev.artem@gmail.com)*

**Purpose:** The purpose of the study was to evaluate diagnostic value of MRI findings usually used in diagnosis of osteomyelitis complicating diabetic osteoarthropathy in patients with diabetes mellitus.
**Methods or Background:** 44 adult outpatients with diabetes mellitus were included in this study: 28 men and 16 women underwent differentiated treatment including operative treatment in 14 cases. Patients with clinically proven osteomyelitis were included in the positive group while patients uncomplicated osteoarthropathy were included in the negative group. Following MRI findings were analyzed: 1. Bone marrow edema: subchondral and diffuse edema counted separately; 2. Bone marrow edema in weight bearing zones, including displaced cuboid and talus bones; 3. Diffusion restriction in bone marrow; 4. Diffusion restriction in soft tissue; 5. Visible with MRI skin ulceration and traveling to weight bearing zone fistula. Analyzed findings were compared to complex clinical data including post-operative histological data. ROC-analysis were applied to evaluate diagnostic value these findings.
**Results or Findings:** Area under curve as the criterion of diagnostic accuracy is decreasing in following sequence: Fistula (AUC=0,760), bone marrow DWI restriction (AUC=0,698), weight bearing bone marrow edema (AUC=0,682), soft tissue fluid collections DWI restriction (AUC=0,661), diffuse bone marrow edema (AUC=0,605), soft tissue edema (AUC=0,563)
**Conclusion:** The best MRI diagnostic finding was MRI presentation of fistula but AUC of 0,760 demonstrates insufficient diagnostic value, and this finding also can be easily detected without MRI. Therefore, no one MRI finding is recommended to be taken as sole criterion for deciding on the presence of osteomyelitis, complicating the diabetic osteoarthropathy.
**Limitations:** This research was limited by one radiologist, opinion, cross-checking multi-specialist opinion will be applied in further research
**Ethics Committee Approval:** The study assumes only data analysis and does not affect the diagnosis and treatment of patients
**Funding for this study:** No funding for this study was applied
**Author Disclosures:**
Elena Burleva: Nothing to disclose
Artem Nikiforovich Ageev: Nothing to disclose
Julia Babushkina: Nothing to disclose

### RPS 610-6

**Magnetic Resonance Imaging (MRI) and High-Resolution Ultrasonography (HRSG) to evaluate patients with carpal tunnel syndrome: which is better?**

*K. Singh*, R. Malik, R. Sarawagi; Bhopal/IN
*(drkjsrandhawa@gmail.com)*

**Purpose:** The study was conducted to evaluate the extent of agreement between MRI and HRSG in clinically or nerve conduction test (NCT) diagnosed cases of Carpal Tunnel Syndrome (CTS) and to evaluate the better modality between the two.
**Methods or Background:** The patients presenting with clinical signs & symptoms of CTS were included in the study. Total of 95 wrists of 51 patients with NCT positive/clinically positive cases of CTS were evaluated. 50 controls were taken for comparison. The same technical methodology & parameters were used to evaluate all the patients both sonographically and by MRI i.e cross sectional area (CSA) at various levels and few other parameters. Statistical analysis was done for sensitivity & specificity of various parameters.
**Results or Findings:** Kohen's Kappa Analysis, Bland Altmann Plot & Paired T test were used to determine degree of agreement between HRSG and MRI for common parameters. Analysis revealed that CSAp & CSAi were comparatively more in agreement when measured by either HRSG or MRI than CSAo & CSAd. By analyzing ROC, maximum area under the curve for HRSG was that of CSAi (0.9), subsequent cut-off was 0.095cm2(sensitivity & specificity of 88.3% and 79.7%). For the MRI parameters, most statistically significant was CSAo (0.923) and cut off for CSAo was 0.095cm2 (sensitivity and specificity of 84.1% and 91.3%).
**Conclusion:** High resolution sonography revealed increased CSAp as having most positive predictive value for the patients having clinical symptoms & positive NCT results while for MRI it was CSAd. However, analyzing cost-benefit ratio and easy availability of HRSG, MRI does not offer any significant advantages over HRSG for radiological diagnosis of Carpal Tunnel Syndrome.
**Limitations:** Limitations of this study include the relatively small sample size.
**Ethics Committee Approval:** Institutional Ethics Committee approved the study via letter IHEC-LOP/2018/MD006.
**Funding for this study:** Funded by the Department of Radiodiagnosis & Imaging, AIIMS, Bhopal
**Author Disclosures:**
Kamaljeet Singh: Nothing to disclose
Radha Sarawagi: Nothing to disclose
Rajesh Malik: Nothing to disclose

### RPS 610-7

**Magnetic resonance imaging evaluation of osteochondral allografts in the knee using the OCAMRISS score**

*B. Tintaya*, C. Sitges, J. Oliva, E. Ramirez, P. Gelber, J. Llauguer; Barcelona/ES

**Purpose:** To correlate Osteochondral Allograft MRI Scoring System (OCAMRISS) in patients undergoing osteochondral allograft transplantation in the knee with clinical outcomes.
**Methods or Background:** A retrospective observational study was performed in our center of a cohort of 37 patients that underwent osteochondral allograft transplantation in the knee from August 2017 to November 2019. All patients received an MR examination nine months after surgery. Clinical outcomes were evaluated using the following scores: International Knee Documentation Committee (IKDC), Kujala, Western Ontario Meniscal Evaluation (WOMET) and Tegner. Correlation between the OCAMRISS and clinical outcomes was calculated using Spearman's correlation coefficients.
**Results or Findings:** A correlation was observed between IKDC total score and OCAMRISS cartilage score ($\rho = 0.396$, P = 0,015), and OCAMRISS total score ($\rho = 0.476$, P = 0,003). A correlation was observed between Kujala score and OCAMRISS cartilage score ($\rho = 0.339$, P = 0,04), and OCAMRISS total score ($\rho = 0.467$, P = 0,004). A correlation was observed between WOMET score and OCAMRISS ancillary score ($\rho = 0.403$, P = 0,013). No correlation was observed between WOMET score and OCAMRISS total score. No correlation was observed between Tegner activity score and OCAMRISS total score.
**Conclusion:** The MRI scoring system (OCAMRISS) is a useful, objective and noninvasive monitoring tool for postoperative assessment after osteochondral allograft transplantation surgery.
**Limitations:** Not applicable
**Ethics Committee Approval:** Not applicable
**Funding for this study:** Not applicable
**Author Disclosures:**
Eduard Ramirez: Nothing to disclose
Pablo Gelber: Nothing to disclose
Benjamin Tintaya: Nothing to disclose
Javier Oliva: Nothing to disclose
Carla Sitges: Nothing to disclose
Jaume Llauguer: Nothing to disclose

### RPS 610-8

**Intermetatarsal bursitis: a novel feature of juxta-articular inflammation in early rheumatoid arthritis - results from a longitudinal MRI-study**

*B. van Dijk*, Y. J. Dakkak, X. Matthijssen, E. Niemantsverdriet, M. Reijnierse, A. van der Helm-van Mil; Leiden/NL
*(B.T.van_Dijk@lumc.nl)*

**Purpose:** Intermetatarsal bursae in the forefeet possess a synovial lining, similarly to joints and tendon-sheaths. Inflammation of these bursae (intermetatarsal bursitis; IMB) was recently identified as specific for early rheumatoid arthritis (RA). We hypothesised that if IMB is indeed an RA-feature, then 1) its presence associates with other measures of local inflammation (synovitis/tenosynovitis/osteitis) at diagnosis and 2) it responds to DMARD-therapy similarly as other local inflammatory measures.
**Methods or Background:** 157 consecutive early RA-patients underwent unilateral contrast-enhanced 1.5T forefoot-MRI at diagnosis. MRIs were evaluated for IMB-presence, and for synovitis/tenosynovitis/osteitis in line with the RA MRI scoring-system (summed as RAMRIS-inflammation). MRIs at 4/12/24 months were evaluated for IMB-presence and -size in patients who had IMB at baseline and received early DMARD-therapy. Logistic regression and generalised estimating equations were used for analyses at patient- and bursa-level. ACPA-stratification was performed.
**Results or Findings:** 69% of RA-patients had ≥1 IMB. In multivariable analysis on bursa-level, presence of IMB was independently associated with local presence of synovitis and tenosynovitis (OR 1.69(95%CI 1.12-2.57) and 2.83(1.80-4.44), respectively), but not osteitis. On patient-level, IMB-presence was most strongly associated with tenosynovitis (OR 2.92(1.62-5.24)). During treatment, IMB-size decreased. This decrease was associated with decrease in RAMRIS-inflammation scores; and most strongly with a decrease in synovitis but not in osteitis. Within ACPA-positive and ACPA-negative RA similar results were obtained.
**Conclusion:** IMB particularly accompanies inflammation of the synovial lining of joints and tendon-sheaths, both regarding simultaneous occurrence at diagnosis and simultaneous treatment-response. These findings suggest that IMB represents juxta-articular synovial inflammation and is a hallmark of early RA.
**Limitations:** Novel consensus-based scoring-method; relatively limited sample-size in the longitudinal part; unavailability of weight-bearing radiographs.
**Ethics Committee Approval:** Obtained (Leiden University Medical Centre ethics committee)
**Funding for this study:** European Research Council (H2020, starting grant, agreement 714312) Dutch Arthritis Society
**Author Disclosures:**
Monique Reijnierse: Nothing to disclose
Ellis Niemantsverdriet: Nothing to disclose
Bastiaan van Dijk: Nothing to disclose
Yousra J. Dakkak: Nothing to disclose
Annette van der Helm-van Mil: Nothing to disclose
Xanthe Matthijssen: Nothing to disclose

### RPS 610-9

**Test-retest repeatability and reproducibility of 3D joint space mapping at the ankle using weight-bearing computed tomography**

*T. Turmezei*[1], J. W. MacKay[1], K. Malhotra[2], A. Gee[3], G. Treece[3], K. Poole[3], M. Welck[2]; [1]Norwich/UK, [2]Stanmore/UK, [3]Cambridge/UK
*(tom@turmezei.com)*

**Purpose:** Joint space mapping (JSM) is a semi-automated image analysis tool for measuring joint space width in 3D that here we apply for the first time at the ankle.
**Methods or Background:** 23 individuals with repeat weight-bearing cone beam CT examinations of both ankles within 4 months between 2013 and 2017 were selected (13 left, 10 right) to provide a mix of pathology: 11 no metalwork; 6 metalwork fusions ipsilateral foot, 6 contralateral foot. Mean±SD interval between imaging visits was 74.0±29.6 days. JSM mapping was performed at medial talocrural (MTC), lateral talocrural (LTC), talonavicular (TN), and posterior subtalar (PST) surfaces at both visits by a single blinded operator. A second operator trained on 6 opposite ankles then repeated JSM in baseline cases. Bland-Altman analysis demonstrated test-retest repeatability and interobserver reproducibility, with results shown as global statistics as well as 3D maps.
**Results or Findings:** Surfacewise bias was 0.00 mm across all joints for test-retest repeatability and reproducibility. Repeatability limits of agreement at individual surfaces were: TN=±0.26 mm, MTC=±0.28 mm, ST=±0.34 mm, and LTC=±0.40 mm. Optimum test-retest sensitivity was less than ±0.2 mm at each central articular surface. Reproducibility limits of agreement at individual surfaces were: TN=±0.27 mm, MTC=±0.24 mm, ST=±0.31 mm, and LTC=±0.37 mm. 3D maps show mean thickness at each joint surface as well as the 3D surface distribution of repeatability and reproducibility metrics.
**Conclusion:** JSM at the ankle is repeatable and reproducible with weight-bearing cone beam CT and is feasible at these joints that feature metalwork and complex regional anatomy.
**Limitations:** 2 from the original selection of 25 failed JSM because of too much metal-induced artefact.
**Ethics Committee Approval:** Consent waiver for retrospective analysis of anonymised data and subsequent data sharing was approved by institution 2.
**Funding for this study:** None
**Author Disclosures:**
Karan Malhotra: Nothing to disclose
Andrew Gee: Nothing to disclose
Graham Treece: Patent Holder: U.S. patent US8938109B2, “Image data processing systefor estimating the thickness of human/animal tissue structures”
James William MacKay: Nothing to disclose
Matthew Welck: Shareholder: Standing CT Company
Kenneth Poole: Patent Holder: U.S. patent US8938109B2, “Image data processing systefor estimating the thickness of human/animal tissue structures”
Tom Turmezei: Advisory Board: International Society of Osteoarthritis Imaging

### Research Presentation Session: Musculoskeletal

### RPS 710

### Nerves and the spine

### RPS 710-1

**Visualizing patterns of intervertebral disc destruction: diagnostic accuracy of dual-energy computed tomography in an ex vivo spine (bio)phantom**

*J. Pohlan*, C. Stelbrink, M. Pumberger, A. D. Gruber, T. Diekhoff; Berlin/DE

**Purpose:** To analyse the diagnostic accuracy of dual-energy computed tomography (DECT) in visualizing differential patterns of intervertebral disc destruction.
**Methods or Background:** A lumbar spine from a great dane dog was used as an ex-vivo biophantom. DECT was performed in sequential volume acquisition with 80 and 135kVp tube voltage with ascending order of tube current. Intervertebral discs were imaged before, and after injection of sodium chloride solution and after ventral discectomy. Oblique axial reformations were used for further analysis. Standardized regions of interest (ROI) were placed in intervertebral disc (IVD) structures. Three blinded readers scored all images separately, i.e. intact disc, nucleus and annulus lesion.
**Results or Findings:** The analysis of the inter-rater reliability yielded an almost perfect agreement with Fleiss kappa at 0.833 (95% CI 0.831-0.835). The analysis of the combined detection accuracy of IVD damage using DECT yielded a sensitivity 91.0% (95% CI 83.6-95.8) and a specificity of 92.0% (95% CI 80.8-97.8). The positive Likelyhood Ratio was 11.4.
**Conclusion:** This work indicates a high accuracy in diagnosing differential patterns of IVD damage using DECT. These data stress the potential of DECT cMaps in imaging spine pathologies with involvement of the IVD.
**Limitations:** First, our exvivo biophantom is a spine from a great dane with different size of the IVD as compared with human IVD. Still, the similarities in morphology suggest that the dog spine provides an adequate model of the human spine. Second, the lesions in this experimental setup are only partially reflecting IVD damage in human pathology. The injection of sodium chloride solution into the IVD does not reflect enzymatic destruction of the connective tissue network of the IVD as in patients with spondylodiscitis.
**Ethics Committee Approval:** Waived
**Funding for this study:** Research grant by Canon medical services for the Dept. of radiology
**Author Disclosures:**
Achim D. Gruber: Nothing to disclose
Torsten Diekhoff: Nothing to disclose
Carsten Stelbrink: Nothing to disclose
Julian Pohlan: Nothing to disclose
Matthias Pumberger: Nothing to disclose

### RPS 710-2

**Combination of quantitative MRI fat fraction and texture analysis to evaluate spastic muscles of children with cerebral palsy**

*T. Akıncı D'Antonoli*[1], F. Santini[1], X. Deligianni[1], M. Garcia Alzamora[2], E. Rutz[3], O. Bieri[1], R. Brunner[1], C. Weidensteiner[1]; [1]Basel/CH, [2]Barcelona/ES, [3]Parkville/AU

**Purpose:** Cerebral palsy (CP) is the most common cause of physical disability in childhood. Muscle pathologies occur due to spasticity and contractures; therefore, imaging of muscles to detect pathologies is often required. Fat fraction(FF) measurement can quantify muscle fat and is often a part of standard imaging in neuromuscular dystrophies. To date, FF has been used to assess muscle fat and function in CP. In this study, we aimed to utilize a radiomics analysis of MR images along with FF analysis to differentiate affected muscles from healthy ones and to compare our radiomics model(RM) with FF model(FFM).
**Methods or Background:** A total of 9 CP patients (8-15y) and 12 healthy controls (9-16y) were prospectively enrolled(2018-2020). Multi-echo Dixon acquisition was used for FF calculation and the second echo (TE=2.87msec) was used for feature extraction. Least absolute shrinkage and selection operator(LASSO) regression was employed for feature selection. Synthetic Minority Over-sampling Technique(SMOTE) was used to overcome class imbalance. RM, FFM and combined model(CM) were built for each calf muscle in datasets with and without SMOTE. The receiver operating characteristic area under the curve(AUC) values were used to evaluate model performance.
**Results or Findings:** In total 42 legs (12 sick,30 healthy) were analyzed. The performance of RM for soleus, gastrocnemius medialis and gastrocnemius lateralis(AUC 0.94,0.91,0.88,respectively) was always better than the FFM(AUC 0.86, 0.69, 0.75,respectively). Combination of both models either had a better performance than RM or a comparable performance(AUC 0.95,0.91,0.88). SMOTE application substantially improved the performance of all models in all muscles(AUC>0.90).
**Conclusion:** In conclusion, the combination of MRI quantitative fat fraction analysis and texture analysis of muscles in CP patients is a promising tool to evaluate muscle involvement of CP in a noninvasive manner.
**Limitations:** Sample size.
**Ethics Committee Approval:** Approved by EC.
**Funding for this study:** Funded by SNSF.
**Author Disclosures:**
Francesco Santini: Nothing to disclose
Meritxell Garcia Alzamora: Nothing to disclose
Xeni Deligianni: Nothing to disclose
Tuğba Akıncı D'Antonoli: Nothing to disclose
Claudia Weidensteiner: Nothing to disclose
Oliver Bieri: Nothing to disclose
Erich Rutz: Nothing to disclose
Reinald Brunner: Nothing to disclose

### RPS 710-3

**Ultrasound-guided infiltration of the greater occipital nerve: a radiological approach to pain management in occipital neuralgia**

*N. A. Ochoa Sambrizzi*, A. Gimeno, M. De Albert De Delas-Vigo, M. Veintemillas, L. Casas, C. Torrents, L. Cuni, R. Dominguez; Barcelona/ES
*(n.ochoa.sambrizzi@gmail.com)*

**Purpose:** We present a controlled before and after study of patients diagnosed with Arnold's neuralgia in our center that received ultrasound (US) guided infiltration of the Greater Occipital nerve (GON) in the last year.
With the aim of showing the efficacy of US infiltration of the GON in the treatment of Occipital Neuralgia.
**Methods or Background:** 12 patients received a total of 20 infiltrations of botulinum toxin or a mixture of corticosteroid and local anesthetic. The follow-up was at least 4 months after the infiltrations. We compared visual analogue scale (VSA) and "Neck Disability Index" (NDI) before and after the procedure. Considering the main outcome a statistical significant reduction of the VSA, as well as an improvement of the NDI. Statistical analysis was through t-test for paired samples.
**Results or Findings:** All 12 patients completed at least 4 months follow up after the procedure. Statistical analysis showed a significant reduction of the VSA score after infiltration as well as a reduction of the NDI scale. There was a significant difference in the average of the VSA scores before and 7 days after the procedure (($p < 0.001$)) with an average reduction of 4.9 points (95% CI [3.7, 6.1]), the mean duration of the effect was 3.4 months. There was also an important difference between the averages of NDI before and after the infiltrations ($p < 0.001$), with an average reduction of 24.5% after the procedure (95% CI [17.4, 31.5]).
**Conclusion:** US guided infiltrations of the GON are an effective way to manage cervical pain in patients with occipital neuralgia and should be considered among the therapeutic options when treating these patients.
**Limitations:** Retrospective study with absence of control group
**Ethics Committee Approval:** Approved by ethics committee
**Funding for this study:** No funding was received
**Author Disclosures:**
Lourdes Casas: Nothing to disclose
Matias De Albert De Delas-Vigo: Nothing to disclose
Alfredo Gimeno: Nothing to disclose
Laura Cuni: Nothing to disclose
Maite Veintemillas: Nothing to disclose
Carme Torrents: Nothing to disclose
Rosa Dominguez: Nothing to disclose
Nicolás Andrés Ochoa Sambrizzi: Nothing to disclose

### RPS 710-4

**High-resolution ultrasound of the marginal mandibular branch of the facial nerve: normal appearance and pathological findings in a case series of postsurgical nerve damage**

*R. Picasso*[1], F. Zaottini[1], F. Pistoia[1], M. Miguel-Pérez[2], C. Martinoli[1]; [1]Genova/IT, [2]Barcelona/IT
*(riccardo.picasso@gmail.com)*

**Purpose:** The aim of this observational cross-sectional study was to validate high-resolution ultrasound (US) as an imaging modality able to map the normal marginal mandibular nerve (MMN) and recognize its abnormalities in the postoperative setting.
**Methods or Background:** A cadaveric study was performed on n=3 fresh frozen face specimens to verify the correspondence between the anatomical and US localization of the MMN. N=20 healthy volunteers (mean age= 29 years) were examined with a 22-8MHz hockey stick probe and the following parameters were collected: I)MMN caliber around the facial artery; II) length of the tract of the MMN amenable to US; III) distance between the gonion and the point where the MMN steps over the mandible body. Finally, n=3 patients with clinically confirmed MMN iatrogenic injury were evaluated with US.
**Results or Findings:** In the cadaveric study, US-guided latex injection provided identification of the MMN. In healthy volunteers, mean MMN caliber resulted 0.69±0.07mm. US was able to recognize the MMN for 3.4±1.2 cm. The nerve was visualized stepping over the mandible body 3.3±0.6 cm from the gonion. Pathologic abnormalities of the MMN were detected and characterized in three patients.
**Conclusion:** High-resolution US is a valuable diagnostic tool to identify the MMN and detect its pathologic changes. This is the first evidence that US is able to image the MMN around the mandible body in normal and pathologic states.
**Limitations:** Only a small series of patients was evaluated
**Ethics Committee Approval:** The study was conducted in accordance the declaration of Helsinki
**Funding for this study:** None
**Author Disclosures:**
Federico Zaottini: Nothing to disclose
Carlo Martinoli: Nothing to disclose
Maribel Miguel-Pérez: Nothing to disclose
Riccardo Picasso: Nothing to disclose
Federico Pistoia: Nothing to disclose

### RPS 710-5

**Diagnostics of infrapatellar saphenous neuralgia: a reversible cause of chronic anteromedial pain following knee surgery**

*S-R. Yang*, A. Schiffmann, B. K. Kovacs, F. Amsler, M. Hirschmann, A. Hirschmann; Basel/CH

**Purpose:** To evaluate the impact of diagnostic nerve block and ultrasound findings on therapeutic choices and predict the outcome after concomitant surgery in patients with suspected neuropathy of the infrapatellar branch of the saphenous nerve (IPBSN).
**Methods or Background:** A total of 55 patients (mean age 57 years ± 13.1 years) following knee surgery with intractable knee pain were retrospectively included. Ultrasound reports including ultrasound-guided anesthetic injection of the IPBSN were assessed for neuroma and postsurgical scarring. Pain severity was graded on visual analog scale (VAS 1-10) before and 40 min following the procedure. Clinical success was defined as a minimum of 50% decrease of the VAS score or an absolute VAS reduction to 1. Electronic medical records were assessed for the type of procedure (neurectomy; other than nerve associated therapy) and pain score at initial follow up consultation. Factors associated with a relevant VAS score were assessed by uni- and multivariate logistic regression and chi-square for quantitative and qualitative variables.
**Results or Findings:** Positive diagnostic nerve block yielded a sensitivity of 96.9% and a specificity of 53.3%. Strongest association was between pain relief by nerve block, positive Tinel sign ($p = 0.002$, odds ratio 8.75) and absence of any case of prosthetic instability ($p = 0.029$, odds ratio 0.17).
**Conclusion:** Selective denervation for neuromatous knee pain is beneficial in selected patients with significant VAS reduction after diagnostic nerve block. Patients without VAS reduction and sonography evidence of IPBSN pathologies need to be evaluated for other causes.

**Limitations:** Verification bias as patients were selected to undergo surgery based on the positive diagnostic nerve block result, more likely received surgery than those tested negative.
**Ethics Committee Approval:** Institutional review board approval was waived for this retrospective study by the local ethics committee.
**Funding for this study:** Unfunded.
**Author Disclosures:**
Schu-Ren Yang: Nothing to disclose
Balazs Krisztian Kovacs: Nothing to disclose
Michael Hirschmann: Nothing to disclose
Anna Hirschmann: Nothing to disclose
Felix Amsler: Nothing to disclose
Alain Schiffmann: Nothing to disclose

### RPS 710-6

**Plantar forefoot pain: ultrasound findings before and after treatment with custom-made foot orthoses**
*D. Albano*, C. Bonifacini, S. Zannoni, S. Bernareggi, C. Messina, L. M. Sconfienza; Milan/IT
*(albanodomenico@me.com)*

**Purpose:** Currently, ultrasound has not a role to assess the response to conservative treatment of metatarsalgia. No previous studies investigated how ultrasound features might change over time in these patients. We aimed to describe ultrasound findings of patients with plantar forefoot pain treated with custom-made foot orthoses.
**Methods or Background:** Twenty patients (15 females; mean age: 62.6±11years) affected by metatarsalgia in 27/40 feet underwent clinical evaluation before, three and six months after conservative treatment with custom-made full foot insole with a support proximal and an excavation below the painful metatarsals. Ultrasound was performed before and three months after the use of orthoses to examine the presence of intermetatarsal / submetatarsal bursitis, metatarsophalangeal joints effusion, anterior plantar fat pad edema, flexor tendinitis/tenosynovitis, and Morton's neuroma. Clinical response with Foot Function Index (FFI)/Visual Analogue Scale (VAS) and ultrasound features changes were evaluated with McNemar and paired Student t tests.
**Results or Findings:** Median VAS and FFI before treatment were 8[5-8.5] and 45.85[32.4-59.4], respectively. After 3 and 6 months of insoles use, both median VAS (2.5 [0-5] and 0 [0-2.75], respectively) and median FFI (7.9 [3.95-20] and 0 [0-3.95], respectively) showed a significant reduction in pain and disability (p<.001). Before treatment, ultrasound revealed 22 intermetatarsal bursitis, 16 submetatarsal bursitis, 10 joint effusions, 20 fat pad edema, 3flexor tendinitis/tenosynovitis and 3 Morton's neuromas. After 3 months of treatment, a significant decrease of intermetatarsal bursitis (7, p<.001) was observed. No significant changes were observed regarding the remaining ultrasound parameters.
**Conclusion:** Ultrasound might be able to detect some imaging features associated with the response of forefoot pain to custom-made foot orthoses, especially identifying sonographic changes of intermetatarsal bursitis.
**Limitations:** Small sample size, short follow-up, no healthy subjects as controls and no randomization in treatment arms.
**Ethics Committee Approval:** Obtained
**Funding for this study:** None
**Author Disclosures:**
Carlo Bonifacini: Nothing to disclose
Susan Bernareggi: Nothing to disclose
Carmelo Messina: Nothing to disclose
Luca Maria Sconfienza: Nothing to disclose
Stefania Zannoni: Nothing to disclose
Domenico Albano: Nothing to disclose

### RPS 710-7

**Patients with episodic migraine show increased T2 values of the trapezius muscles: an investigation by quantitative high-resolution magnetic resonance imaging**
*N. Sollmann*, P. Schandelmaier, D. Weidlich, C. Zimmer, D. C. Karampinos, F. Heinen, M. Landgraf, M. Bonfert; Munich/DE
*(nico.sollmann@tum.de)*

**Purpose:** Neck pain and dysfunction is frequent in patients with migraine. Likewise, evidence for inflammatory processes in the trapezius muscles is accumulating. However, non-invasive and objectively assessable correlates are missing in vivo.
**Methods or Background:** Twenty-one subjects with episodic migraine (mean age: 24.6±3.2 years, 18 females) and 22 controls (mean age: 23.0±2.2 years, 17 females) without any history of headache prospectively underwent physical examination and quantitative 3-Tesla MRI of the trapezius muscles. A T2-prepared turbo spin-echo (TSE) sequence was acquired for manual segmentation of the trapezius muscles and extraction of mean T2 values.
**Results or Findings:** There were no statistically significant differences regarding age, sex, body mass index, or number of myofascial trigger points (mTrPs) between groups. All patients with migraine presented with mTrPs in the trapezius muscles. T2 of the entire trapezius muscles was significantly higher in the migraine group when compared to controls (31.0±1.2 ms versus 30.1±1.0 ms; p=0.022).
**Conclusion:** Elevated T2 values of the trapezius muscles may indicate subtle inflammatory processes within musculature among patients with migraine because T2 increase is likely to stem from edematous changes. Future work may validate this finding in larger cohorts, but muscle T2 might have potential to develop into a viable in-vivo biomarker for muscular affection in migraine.
**Limitations:** The cohort demographics with a small age range and constitution of predominantly females represent this study's limitations.
**Ethics Committee Approval:** This prospective study was approved by the local Institutional Review Board.
**Funding for this study:** Not applicable.
**Author Disclosures:**
Michaela Bonfert: Nothing to disclose
Florian Heinen: Nothing to disclose
Mirjam Landgraf: Nothing to disclose
Claus Zimmer: Nothing to disclose
Paul Schandelmaier: nothing to disclose
Nico Sollmann: Nothing to disclose
Dominik Weidlich: Nothing to disclose
Dimitrios C. Karampinos: Nothing to disclose

### RPS 710-8

**Differentiation of benign and malignant vertebral compression fractures using qualitative and quantitative analysis of a single fast spin echo T2-weighted Dixon sequence**
*P. Omoumi*, S. Bacher, S. D. Hajdu, Y. Maeder, V. Dunet, T. Hilbert; Lausanne/CH
*(patrick.omoumi@chuv.ch)*

**Purpose:** To determine and compare the qualitative and quantitative diagnostic performance of a single sagittal fast spin echo (FSE) T2-weighted Dixon sequence in differentiating benign and malignant VCFs, using multiple readers and different quantitative methods.
**Methods or Background:** From July 2014 to October 2019, 90 consecutive patients with spine MRI performed prior to cementoplasty for acute VCFs were retrospectively included. VCFs were categorized as benign (n=61, mean age=76±13years) or malignant (n=29, mean age=63±13years) based on the reference standard, biopsy and/or ≥9months clinical and imaging follow-up. Qualitative analysis was independently performed by four radiologists by categorizing each VCF as either benign or malignant using only the image sets provided by FSE T2-weighted Dixon sequences. Quantitative analysis was performed using two different regions of interest (ROI1-2), and three methods (signal drop, fat fraction (FF) from ROIs, FF from maps). Diagnostic performance was compared using ROC curves analyses. Interobserver agreement was assessed using kappa statistics and intraclass correlation coefficients (ICC).
**Results or Findings:** The qualitative diagnostic performance ranged from area under the curve (AUC)=0.97 (95%CI:0.92-1.00) to AUC=0.99 (95%CI:0.98-1.0). The quantitative diagnostic performance ranged from AUC=0.82 (95%CI:0.72-0.92) to AUC=0.96 (95%CI:0.92-0.99). Pairwise comparisons showed no statistical difference in diagnostic performance (all p≥0.002, Bonferroni corrected p<0.0011). All five cases with discordance among the readers, were correctly diagnosed at quantitative analysis using ROI2. Interobserver agreement was excellent for both qualitative and quantitative analysis.
**Conclusion:** A single FSE T2-weighted Dixon sequence can be used to differentiate benign and malignant vertebral compression fractures with high diagnostic performance using both qualitative and quantitative analysis, which can provide complementary information.
**Limitations:** No correction for potential confounding effect of previous treatment.
Retrospective design.
**Ethics Committee Approval:** Regional Ethical committee approval (Commission d'éthique de la recherche sur l'être humain, Vaud).
**Funding for this study:** None.
**Author Disclosures:**
Vincent Dunet: Author: co-author
Sebastien Bacher: Author: First author
Yaël Maeder: Author: co-author
Tom Hilbert: Author: co-author
Patrick Omoumi: Author: Guarantor
Steven David Hajdu: Author: co-author

### Research Presentation Session: Neuro

### RPS 311

### Cancer in neuroradiology

### RPS 311-2

**Noninvasive differentiation of molecular subtypes of non-enhancing glioma using MRI perfusion and diffusion parameters**

*I. J. Pruis*[1], S. Koene[1], S. van der Voort[1], F. Incekara[1], A. Vincent[1], M. van den Bent[1], G. Lycklama à Nijeholt[2], S. Veldhuijzen van Zanten[1], M. Smits[1]; [1]Rotterdam/NL, [2]The Hague/NL
*(i.pruis@erasmusmc.nl)*

**Purpose:** Approximately 14-45% of non-enhancing glioma have a highly aggressive course due to glioblastoma genetic status requiring rapid intervention and treatment. The aim of this retrospective study is to distinguish the molecular profiles of non-enhancing glioma using physiological MRI.
**Methods or Background:** Ninety-five patients with non-enhancing glioma were included in whom pre-operative MRI was available including at least T2w/FLAIR, dynamic contrast susceptibility perfusion and diffusion imaging, and molecular status including 1p/19q co-deletion status and IDH mutation. Tumours were segmented semi-automatically using ITK-SNAP to derive whole tumour histograms of relative Cerebral Blood Volume (rCBV) and Apparent Diffusion Coefficient (ADC). Tumours were divided into three clinically relevant molecular subgroups; IDH mutation (IDHmt) with (n=39) or without 1p/19q co-deletion (n=38), and IDH-wildtype (IDHwt) (n=18). ANOVA and Kruskall-Wallis tests were performed using SPSS.
**Results or Findings:** rCBV (mean, median, 75th and 85th percentile) and ADC (mean, median, 15th and 25th percentile) showed significance across molecular profiles ($p<0.01$), post-hocs revealed 1p/19q intact IDHmt tumours have significantly lower rCBV compared to 1p/19q co-deleted IDHmt and IDHwt tumours (mean rCBV 1.06 (0.37-1.83) versus 1.36 (0.84-2.76) and 1.37 (0.55-2.31), $p<0.05$). Also, 1p/19q intact IDHmt tumours had significantly higher ADC compared to the two other profiles (mean ADC 1.46 (1.12-2.09) versus 1.27 (1.05-1.74) and 1.16 (0.56-1.56), $p<0.001$). No significant differences were found in ADC and rCBV parameters between 1p/19q co-deleted IDHmt and IDHwt tumours.
**Conclusion:** 1p/19q intact IDHmt tumours had significantly lower rCBV and higher ADC compared to 1p/19q co-deleted IDHmt and IDHwt tumours, indicating that MRI perfusion and diffusion parameters could be useful to determine the molecular profile of non-enhancing glioma.
**Limitations:** n/a
**Ethics Committee Approval:** Obtained from the Erasmus MC Medical Ethics Committee.
**Funding for this study:** n/a
**Author Disclosures:**
Stephan Koene: Nothing to disclose
Sebastian van der Voort: Nothing to disclose
Fatih Incekara: Nothing to disclose
Sophie Veldhuijzen van Zanten: Nothing to disclose
Arnaud Vincent: Nothing to disclose
Ilanah Johanna Pruis: Nothing to disclose
Martin van den Bent: Nothing to disclose
Marion Smits: Nothing to disclose
Geert Lycklama à Nijeholt: Nothing to disclose

### RPS 311-3

**Imaging-based classification of glioma, it's validation with survival analysis and integration with new WHO genetic glioma classification**

*A. N. Kamble*[1], N. K. Agrawal[2], S. Koundal[1], S. Bhargava[1], A. N. Kamble[3], D. A. Joyner[4], T. Kalelioglu[4], S. Patel[4], R. Jain[5]; [1]New Delhi/IN, [2]Mohali/IN, [3]Mumbai/IN, [4]Charlottesville, VA/US, [5]New York, NY/US
*(akshaykumar.kamble92@gmail.com)*

**Purpose:** Imaging-based classification of glioma independent of histological or genetic markers predicting survival of patients is an unmet need. We propose a classification which can predict survival and integrates with the new WHO genetic classification.
**Methods or Background:** Multicentric study with TCGA-LGG (199 patients), REMBRANDT (130 patients) and TCGA-GBM (262 patients) dataset of glioma were analyzed as training dataset. Based on MRI images, gliomas were classified into four types. Survival analysis was done using Kaplan Meier and Cox regression. UVA dataset with (202 patients) was analyzed to validate the classification as testing dataset. Evaluation the sensitivity and specificity of radiological types with existing histological and genetic markers was done.
**Results or Findings:** Imaging-based classification predicted survival independent of genetic or histological information in both testing and training databases. Type I glioma have good specificity in identifying 1p 19q co-deleted IDH mutated, oligodendrogliomas and grade II gliomas. Type II gliomas have good specificity identifying IDH mutant 1p 19q non co-deleted and Grade II gliomas. Type III and Type IV have good specificity in identifying Grade IV and IDH wild gliomas (p value $< 0.05$). There is a substantial interobserver agreement for new radiological classification in both training and testing databases ($\kappa > 0.6$).
**Conclusion:** Proposed 'Imaging-based radiological glioma classification' does predict the survival of patients independent of histological or genetic testing. This can especially useful in resource strained or remote healthcare facilities where this novel classification can provide the preliminary insight into prognosis and genotype of gliomas using non-invasive routine MRI scan.
**Limitations:** Limitations of our study includes retrospective design.
**Ethics Committee Approval:** TCIA achieve being open access has ethics committee approval stated on the website from the respective institutes. UVA team obtained the ethic committee approval for UVA the testing database.
**Funding for this study:** None
**Author Disclosures:**
David A. Joyner: Nothing to disclose
Sohil Patel: Nothing to disclose
Nidhi Kishore Agrawal: Nothing to disclose
Rajan Jain: Nothing to disclose
Abhaykumar Nana Kamble: Nothing to disclose
Surabhi Koundal: Nothing to disclose
Akshaykumar Nana Kamble: Nothing to disclose
Salil Bhargava: Nothing to disclose
Tuba Kalelioglu: Nothing to disclose

### RPS 311-4

**Diagnostic accuracy of ASL in comparison with DSC perfusion in the surveillance of different types of brain tumours**

*A. Lavrova*[1], W. Teunissen[2], E. Warnert[2], M. van den Bent[2], V. Cheremisin[1], M. Smits[2]; [1]St. Petersburg/RU, [2]Rotterdam/NL
*(lavrova.anya@gmail.com)*

**Purpose:** Dynamic susceptibility contrast-enhanced (DSC) perfusion is an established standard in the assessment of brain tumor perfusion. This study aims to assess the feasibility of using non-contrast arterial spin-labeling (ASL) instead of DSC.
**Methods or Background:** We included 122 (78 males, mean age 54±13) patients in this retrospective study, who underwent both ASL and DSC perfusion in the same MRI-scanning session at 3T (GE) during 2019. ASL-derived Cerebral Blood Flow (CBF) maps were created with Ready View (AW Server, GE); DSC-derived relative Cerebral Blood Volume (rCBV) maps, uncorrected for leakage, were created with Intellispace Portal (Philips). Lesions were identified as T2 and T2-FLAIR hyperintensity with or without contrast enhancement (CE). Measurements were done in Radiant DICOM Viewer by placing a region of interest of approximately 70 mm2 on the rCBV map and copying it to the contralateral normal-appearing white matter (NAWM) and to the CBF map. Pearson's correlation coefficient testing was done on the CBF and rCBV ratios of tumor versus NAWM.
**Results or Findings:** We identified 183 lesions, 120 with and 63 without CE. Correlation coefficients were as follows: glioma with (80 lesions, $r=0.648$, $p<0.001$), and without CE (56 lesions, $r=0.551$, $p<0.001$), lymphoma with (10 lesions, $r=-0.233$, $p=0.464$) and without CE (7 lesions, $r=0.02$, $p=0.952$), metastases with CE (30 lesions, $r=0.33$, $p=0.081$).
**Conclusion:** We found a strong correlation in glioma, while there was no significant correlation in lymphoma and metastases. Our findings suggest that ASL can be used instead of DSC to measure perfusion in glioma at 3T. For lymphoma and metastases, this should be further investigated in a larger group.
**Limitations:** Cases with motion (1), signal loss at the site of the tumor (2) were excluded.
**Ethics Committee Approval:** Approved by the Erasmus MC Ethics Committee.
**Funding for this study:** None.
**Author Disclosures:**
Vladimir Cheremisin: Nothing to disclose
Esther Warnert: Nothing to disclose
Anna Lavrova: Nothing to disclose
Wouter Teunissen: Nothing to disclose
Martin van den Bent: Nothing to disclose
Marion Smits: Nothing to disclose

### RPS 311-5

**Combination of magnetic resonance relaxometry and ASL perfusion in assessment of high-grade glioma subregions and surrounding brain tissue**

*I. Chekhonin*, A. Batalov, A. Tyurina, E. Pogosbekian, P. Nikitin, A. Bykanov, N. Zakharova, D. Pitskhelauri, I. N. Pronin; Moscow/RU
*(Ivan-Chekhonin@yandex.ru)*

**Purpose:** To assess the capability of MR relaxomery and ASL perfusion to differentiate between regions of malignant gliomas and areas of surrounding brain tissue.
**Methods or Background:** 27 patients older than 18 years with newly diagnosed contrast-enhancing supratentorial glial tumors were enrolled. The MRI protocol (1.5 T) contained conventional stuctural MRI (including contrast-enhanced images), pseudocontinuous ASL perfusion and magnetic resonance image compilation (MAGiC). Before tumor resection, the following ROIs on relaxometric and ASL maps were chosen: - area of maximal perfusion within contrast-enhancing tumor part (1); - perifocal infiltrative edema (non-enhancing T2 FLAIR hyperintensive) zone (2) - normal-appearing tumor-adjacent white (3) and gray (6) matter along the operative approach - normal-appearing white (4) and gray (5) matter remote from the tumor zone Statistics included Kruskal-Wallis and Bonferroni-corrected Mann-Whitney tests, ROC analysis and Spearman correlation.
**Results or Findings:** There were 18 patients with glioblastoma, 5 patients with anaplastic oligodendroglioma, 4 patients with anaplastic astrocytoma. Cerebral blood flow (CBF) allowed to differentiate between zones 1 and 2 with higher values in zone 1. T1, T2 and proton density values could differentiate between zones 2 and 3 with higher values in zone 2. T2 values could also afford to discriminate between zones 3 and 4 with lower values in zone 3. No difference was shown between relaxomertic or perfusion properties of zones 5 and 6. Significant inverse correlation was found between T2 and CBF in zone 1.
**Conclusion:** ASL perfusion and MR relaxometry possess complementary power for differentiation of glioma subregions and adjacent brain matter and thus represent a rationale for combined application.
**Limitations:** Sample size.
Possible anatomical differences in relaxometric values between brain regions independent of tumor presence.
**Ethics Committee Approval:** Approval granted.
**Funding for this study:** Russian Foundation for Basic Research grant №18-29-01018.
**Author Disclosures:**
Dipl.Phys. Eduard Pogosbekian: Nothing to disclose
Pavel Nikitin: Nothing to disclose
Andrey Bykanov: Nothing to disclose
David Pitskhelauri: Nothing to disclose
Artem Batalov: Nothing to disclose
Anastasia Tyurina: Nothing to disclose
Ivan Chekhonin: Nothing to disclose
Igor Nikolaevich Pronin: Nothing to disclose
Natalia Zakharova: Nothing to disclose

### RPS 311-6

**Assessment of subcortical brain volume changes in breast cancer patients undergoing treatment: a pilot study**

*A. S. C. Verde*, B. Sousa, A. Oliveira-Maia, F. Cardoso, C. Matos, N. Papanikolaou; Lisboa/PT

**Purpose:** Prior studies have focused on the investigation of chemotherapy-related cognitive impairment (CRCI) in breast cancer patients, as supported by neuroimaging findings. In this study, we aim at investigating whether chemotherapy treatment induces subcortical volumetric changes.
**Methods or Background:** Brain images of 29 breast cancer patients – 19 endocrine-treated and ten chemotherapy-treated– were prospectively acquired on a 3T MRI at two-time points, namely, before and six months after treatment. Additionally, 27 gender- and age-matched healthy controls, provided by the Open Access Series of Imaging Studies (OASIS) Cross-Sectional dataset, were included in the analysis. Firstly, T1-weighted images were segmented into 15 subcortical structures using FIRST from FSL (FMRIB software library). Secondly, its respective volumes were quantified using FSLstats and normalized using the V-scaling factor from SIENAX. A two-way repeated-measures ANOVA was performed, using age and total brain volume as study covariates. Post-hoc Tukey tests were employed and p-values obtained were adjusted for multiple comparisons using FDR.
**Results or Findings:** After controlling for age and total brain volume, a significant main effect of the participants' group in the volumes of the bilateral thalamus, putamen, nucleus accumbens, and right pallidum was found. Particularly, Tukey test results corrected for multiple comparisons showed that at six months there were statistically significant volume reductions in the bilateral thalamus (left: p=0.011, right: p=0.006) of the chemotherapy-treated patients compared with the healthy controls, while for the endocrine-treated group similar comparisons to healthy controls were non-significant (left: p=1.000, right: p=0.750).
**Conclusion:** There is evidence of thalamic volume reduction in chemotherapy-treated breast cancer patients, which may reflect attention and emotion dysregulations in these patients.
**Limitations:** Reduced number of chemotherapy-treated patients.
**Ethics Committee Approval:** MR acquisitions approved by the local EC.
**Funding for this study:** BOUNCE project funded by the EU Horizon 2020 research and innovation program.
**Author Disclosures:**
Albino Oliveira-Maia: Nothing to disclose
Fátima Cardoso: Nothing to disclose
Celso Matos: Nothing to disclose
Berta Sousa: Nothing to disclose
Nickolas Papanikolaou: Advisory Board: Advantis Medical SysteFounder: MRIcons
Ana Sofia Castro Verde: Nothing to disclose

### RPS 311-7

**Improved imaging of therapy-naive high-grade glioma by turbo spin-echo black blood sequences**

*N. Sollmann*, J. Gempt, C. Zimmer, J. S. Kirschke, T. Finck; Munich/DE
*(nico.sollmann@tum.de)*

**Purpose:** To investigate the value of contrast-enhanced (CE) turbo spin echo black blood (BB) sequences for imaging of therapy-naive high-grade gliomas (HGGs).
**Methods or Background:** Patients with WHO grade III or IV gliomas and no oncological treatment were retrospectively included (March 2019 to January 2020). 3-Tesla MRI comprised CE BB and CE turbo field echo (TFE) sequences. The lack/presence of tumor-related contrast enhancement and satellite lesions were evaluated by two readers. Sharper delineation of tumor boundaries (1, bad; 2, intermediate; 3, good delineation) and vaster expansion of HGGs into the adjacent brain parenchyma on CE BB imaging were the endpoints. Furthermore, contrast-to-noise ratios (CNRs) were calculated.
**Results or Findings:** Fifty-four patients were included (mean age: 61.2±15.9 years, 64% male). The vast majority of HGGs (51/54) showed contrast enhancement in both sequences, while two HGGs as well as one of six detected satellite lesions were depicted in CE BB imaging only. Tumor boundaries were significantly sharper (R1: 2.43±0.71 vs. 2.73±0.62, p<0.001; R2: 2.44±0.74 vs. 2.77±0.60, p=0.001), while the spread of HGGs into the adjacent parenchyma was larger when considering CE BB sequences according to both readers (larger spread in CE BB sequences: R1: 23 patients; R2: 20 patients). The CNR for CE BB sequences significantly exceeded that of CE TFE sequences (43.4±27.1 vs. 32.5±25.0, p=0.0028).
**Conclusion:** Our findings suggest that BB imaging may considerably improve delineation of therapy-naive HGGs when compared with established TFE imaging.
**Limitations:** The retrospective character of the study and the evaluation of tumor boundaries and size only in qualitative manner represent this study's limitations.
**Ethics Committee Approval:** This study was approved by the local Institutional Review Board (registration number: 340/16 S).
**Funding for this study:** Not applicable.
**Author Disclosures:**
Jan S Kirschke: Nothing to disclose
Claus Zimmer: Nothing to disclose
Jens Gempt: Nothing to disclose
Nico Sollmann: Nothing to disclose
Tom Finck: Nothing to disclose

### RPS 311-8

**Recurrence assessment in glioblastoma: 11C-methionine PET vs perfusion magnetic resonance imaging (MRI)**

*A. C. Igual Rouilleault*, I. Soriano Aguadero, J. F. Bastidas, P. Malmierca Ordoqui, A. Paternain Nuin, R. Garcia de Eulate, M. Calvo Imirizaldu, J. Arbizu, P. Dominguez Echavarri; Pamplona/ES
*(aigual@unav.es)*

**Purpose:** To compare dynamic susceptibility contrast perfusion MRI (DSC-MRI) and 11C -MET PET (MET-PET) accuracy in differentiating radiation necrosis from tumor recurrence in patients with treated glioblastoma.
**Methods or Background:** We studied 19 patients with treated glioblastoma undergoing DSC-MRI and MET-PET in the follow-up evaluation due to new contrast enhancement in T1-weighted images, including a total of 25 examinations. In DSC-MRI, regions of interest (ROI) with maximum relative cerebral blood volume (rCBV) in the area with contrast enhancement was compared to the average value of a reference ROI in the contralateral white matter to obtain the relative rCBV ratio. For MET-PET, we calculated the target-to-background ratio (TBR) in the areas with increased uptake. We studied the diagnostic accuracy of both imaging modalities (ROC-AUC), explored their concordance (Kappa index) and compared rCBV and TBR values between radiation necrosis and tumor recurrence diagnosis (U-Mann-Whitney test). A subanalysis was conducted after excluding patients under antiangiogenic treatment.
**Results or Findings:** DSC-MRI analysis showed a sensitivity and specificity of 81% and 100%, respectively, and positive predictive value and negative predictive value of 100% and 75% (AUC: 0.83). As for the MET-PET, statistics revealed a sensitivity and specificity of 100% and 78%, respectively, and positive predictive value and negative predictive value of 89% and 100%(AUC: 0.84), with a moderate concordance between both examinations(kappa:0.6). After excluding patients with antiangiogenic therapies, DSC-MRI sensitivity increased by 9%, not detecting changes in MET-PET results(kappa:0.7). The rCBV and TBR were higher in the recurrence group than in the necrosis group(p=0.007 and p=0.006).
**Conclusion:** Both techniques seem excellent in the recurrence assessment of treated glioblastoma patients. In patients treated with antiangiogenic therapies, MET-PET should be preferred.
**Limitations:** Small sample size
Lack of histological final diagnosis in most patients.
**Ethics Committee Approval:** Not applicable.
**Funding for this study:** No.
**Author Disclosures:**
Pablo Dominguez Echavarri: Nothing to disclose
Ignacio Soriano Aguadero: Nothing to disclose
Patricia Malmierca Ordoqui: Nothing to disclose
Juan Fernando Bastidas: Nothing to disclose
Javier Arbizu: Nothing to disclose
Alba Cristina Igual Rouilleault: Nothing to disclose
Marta Calvo Imirizaldu: Nothing to disclose
Reyes Garcia de Eulate: Nothing to disclose
Alberto Paternain Nuin: Nothing to disclose

### RPS 311-9

**ASL for brain tumours: do we really need CBF maps?**
*W. Teunissen*[1], A. Lavrova[2], E. Warnert[1], V. Cheremisin[2], M. van den Bent[1], M. Smits[1]; [1]Rotterdam/NL, [2]St. Petersburg/RU
*(whtteunissen@gmail.com)*

**Purpose:** Cerebral blood flow (CBF) in intra-axial tumours can be measured with arterial spin labelling (ASL), an advanced MRI technique in which perfusion-weighted images (PWI) are acquired and quantified based on a kinetic model. The aim of this study was to compare non-quantitative PWI with quantitative CBF maps to assess whether quantification could be omitted.
**Methods or Background:** We identified a retrospective consecutive cohort who underwent ASL in addition to routinely performed DSC perfusion MRI at 3T in 2019 for surveillance of intra-axial brain tumours. ASL derived CBF maps were computed with AWServer. PWI consisted of the averaged subtractions of the unlabelled minus the labelled acquisitions as provided directly by the scanner without additional post-processing. After visual quality assessment, ROIs of approximately 70mm2 were drawn manually within a representative part of the tumour and copied to the contralateral normal appearing white matter (nawm) to calculate ratios. Pearson's correlation coefficients of CBF and PWI ratios were calculated. Subset analyses were done separating primary/secondary tumours and enhancing/non-enhancing lesions.
**Results or Findings:** We measured 183 lesions within 122 patients. Mean age was 54y(±13), 77 were male. We found a high correlation of PWI and CBF ratios (r=0.969,p<0.001), also in a subset of enhancing glioma (n=80,r=0.956,p<0.001), non-enhancing glioma (n=56,r=0.997,p<0.001), enhancing metastasis (n=30,r=0.943,p<0.001) and non-enhancing lymphoma (n=7,r=0.944,p=0.0013). Correlation in enhancing lymphoma was lower (n=10,r=0.833,p=0.0027).
**Conclusion:** The results show an excellent correlation of the ratios of non-quantitative PWI and quantitative CBF ratios. Such high correlation can be expected when there is no violation of the underlying assumptions for quantification of CBF from PWI. However, these results suggest that for lymphoma with high PWI these assumption are not valid.
**Limitations:** Lack of follow-up data for definitive diagnosis: part of future research.
**Ethics Committee Approval:** Approved (ErasmusMC ethics committee).
**Funding for this study:** None.
**Author Disclosures:**
Vladimir Cheremisin: Nothing to disclose
Esther Warnert: Nothing to disclose
Anna Lavrova: Nothing to disclose
Wouter Teunissen: Nothing to disclose
Martin van den Bent: Nothing to disclose
Marion Smits: Nothing to disclose

### RPS 311-10

**Comparison of capability for molecular-based assessment in various brain tumour patients between 3D gradient echo-based CEST imaging and 2D spin echo-based CEST imaging**
*K. Murayama*[1], Y. Ohno[1], M. Yui[2], K. Yamamoto[2], M. Ikedo[2], S. Hanamatsu[1], A. Iwase[1], T. Fukuba[1], H. Toyama[1]; [1]Toyoake, Aichi/JP, [2]Ohtawara/JP

**Purpose:** To compare the capability for molecular-based assessment using 2D and 3D chemical exchange saturation transfer imaging at 3.5 ppm (APT-weighted image) in various brain tumor patients.
**Methods or Background:** 46 consecutive patients with brain tumors were examined with APT-weighted image by using 2D spin echo and 3D gradient echo sequences at a 3T MR system. Then, all patients were divided into benign and malignant intra-axial tumor and extra-axial tumor. In each lesion, magnetization transfer ratio asymmetry (MTRasym) was assessed by ROI measurements. To determine the relationship between two methods measurement of MTRasym, Spearman's rank correlation was performed. Student's t-test was performed to determine the difference between malignant and benign tumors. Finally, diagnostic accuracy to differentiate glioblastoma was compared between two methods by McNemar test.
**Results or Findings:** 3D APT-weighted image had significant and excellent correlation with 2D APT-weighted image (r=0.71, p<0.0001). The limits of agreement between 2D and 3D APT-weighted image was enough small. MTRasym had significant difference between low-grade and high-grade gliomas (2D: p=0.002, 3D: p<0.001). Diagnostic performance to differentiate glioblastoma from high-grade gliomas of 3D APT-weighted image was superior to that of 2D APT-weighted image.
**Conclusion:** 3D APT-weighted image appears to be as useful as 2D APT-weighted image for molecular-based assessment and can be considered at least as valuable as 2D APT-weighted image in various brain tumor patients.
**Limitations:** Limited study population is one of the limitations in this study.
**Ethics Committee Approval:** This study was retrospective study and written informed consent from each subject was waved. This study was approved by the institutional review board of Fujita Health University Hospital.
**Funding for this study:** This study was technically supported by Canon Medical Systems Corporation. Ohno, Murayama, Toyama: Research grants/ Canon Medical Systems Corporation. Yui, Yamamoto, Ikedo: Employee/ Canon Medical Systems Corporation
**Author Disclosures:**
Masato Ikedo: Employee: Canon Medical SysteCorporation
Kazuhiro Murayama: Grant Recipient: Canon Medical SysteCorporation
Satomu Hanamatsu: Nothing to disclose
Kaori Yamamoto: Employee: Canon Medical SysteCorporation
Hiroshi Toyama: Nothing to disclose
Masao Yui: Employee: Canon Medical SysteCorporation
Yoshiharu Ohno: Grant Recipient: Canon Medical SysteCorporation
Akiyoshi Iwase: Nothing to disclose
Takashi Fukuba: Nothing to disclose

### RPS 311-12

**Image subtraction maps for longitudinal FLAIR imaging in patients with glioma may facilitate early detection of tumour progression**
*N. Sollmann*, M. Gutbrod-Fernandez, E. Burian, J. Gempt, C. Zimmer, J. S. Kirschke; Munich/DE
*(nico.sollmann@tum.de)*

**Purpose:** Progression of glioma is frequently characterized by increases or enhanced spread of a hyperintensity in fluid attenuated inversion recovery (FLAIR) sequences. However, changes in FLAIR signal over time can be subtle, and conventional (CONV) visual reading is time-consuming. The purpose of this study was to compare CONV reading to reading of subtraction maps (SMs).
**Methods or Background:** FLAIR datasets of 3-Tesla MRI acquired at two different time points (mean inter-scan interval: 5.4±1.9 months) were considered per patient, derived from a consecutive series of 100 patients (mean age: 49.0±13.7 years) diagnosed with glioma (81 glioma WHO grade III&IV). Two readers performed CONV and SM reading assessing alterations in tumor-associated FLAIR signal over time (stable/unchanged or progressive), diagnostic confidence (1-very high to 5-very low diagnostic confidence), and time needed for reading. Gold-standard (GS) reading including all available clinical and imaging information revealed progressive FLAIR signal in 61 patients.
**Results or Findings:** SM reading revealed FLAIR signal increases in a larger proportion of patients according to evaluations of both readers (R1: 61 patients / R2: 60 patients identified with FLAIR signal increase vs. R1: 45 patients / R2: 44 patients for CONV reading) with significantly higher diagnostic confidence (R1: 1.29±0.48, R2: 1.26±0.44 vs. R1: 1.73±0.80, R2: 1.82±0.85; p<0.0001). This resulted in increased sensitivity (99.9% vs. 73.3%) with maintained high specificity (98.1% vs. 98.8%) for SM reading when compared to CONV reading. The time needed for SM reading was significantly lower compared to CONV assessment (p<0.0001).

**Conclusion:** SM reading may improve diagnostic accuracy and sensitivity while reducing reading time, thus potentially enabling earlier detection of disease progression.
**Limitations:** The retrospective character and qualitative analysis approach reflect this study's limitations.
**Ethics Committee Approval:** This retrospective study was approved by the local Institutional Review Board.
**Funding for this study:** Not applicable.
**Author Disclosures:**
Jan S Kirschke: Nothing to disclose
Claus Zimmer: Nothing to disclose
Jens Gempt: Nothing to disclose
Magaly Gutbrod-Fernandez: Nothing to disclose
Nico Sollmann: Nothing to disclose
Egon Burian: Nothing to disclose

### RPS 311-13

**Diffusion tensor imaging (DTI): a better understanding of spinal cord tumours**

*A. Ignatius*, P. S. Babu, R. Ravi; Chennai/IN
*(dr.amalanignatius@gmail.com)*

**Purpose:** The primary diagnosis of spinal intramedullary tumour suspected lesions is difficult and often it requires diagnostic spinal biopsy with the risk of neurological deficits. The DTI helps in identifying the type of tumour. The aim of the study is to assess the integrity of the spinal cord tracts in spinal tumours, analyse the DTI parameters and correlate them with HPE, prognosticate the tumour, plan surgery and predict post-operative damage
**Methods or Background:** The prospective study was conducted in the Barnard Institute of Radiology, MMC & RGGGH. The consecutive subjects undergoing Spinal MR for spinal cord tumours were invited to participate in this study. RESOLVE DTI was performed using a 1.5-T scanner (Avanto, Siemens). The tractography, FA, ADC (MD), E1 (AD), E2, E3 & RD values were analysed with HPE and compared with age/sex-matched normal controls.
**Results or Findings:** Ependymoma (n=2) caused the dispersion of fibres while astrocytoma (n=3) and ganglioglioma (n=2) caused the disruption of fibres. Tuberculoma (n=3) caused mild dispersion of fibres. The extramedullary tumours(n=4) caused compression of fibres while intramedullary tumours (n=10) caused dispersion and disruption of fibres. Mean FA values were decreased in ganglioglioma (0.223), ependymoma (0.451), astrocytoma (0.351), spinal intramedullary metastasis (0.380),tuberculoma (0.430) and extramedullary tumours (0.498). ADC values were increased in all tumours (n=14) except in tuberculoma (n=3) where it is decreased (0.916).
**Conclusion:** The mean-FA values were decreased in all tumours (n=14) while mean-ADC values were significantly increased except in tuberculoma where the mean-ADC values were decreased. The high-grade tumours caused the disruption of fibres while low-grade tumours caused the dispersion of fibres. DTI avoids paralytic biopsies, predicts the prognosis and avoids postoperative complication.
**Limitations:** Further studies with larger cohorts are needed to infer a definite conclusion.
**Ethics Committee Approval:** Institutional-Ethical committee approval.
**Funding for this study:** Nil.
**Author Disclosures:**
R Ravi: Nothing to disclose
Peter S. Babu: Nothing to disclose
Amalan Ignatius: Nothing to disclose

### RPS 311-14

**Comparative analysis of arterial spin labeling and dynamic contrast perfusion imaging for quantitative perfusion measurements of brain tumours**

*S. Ahmed*, S. Tiwari, T. Yadav, P. K. Garg, P. S. Khera, B. Sureka; Jodhpur/IN
*(ahmedsabha03@gmail.com)*

**Purpose:** This is a prospective comparative study between perfusion metrics obtained from arterial spin labeling (ASL) technique and dynamic susceptibility contrast perfusion (DSC) in glial neoplasms. To assess the utility of rCBF (cerebral blood flow) maps derived from Arterial spin labeling in pre-operative assessment of primary neoplasms of brain and study its correlation with the rCBF derived from DSC perfusion.
**Methods or Background:** Forty two cases of newly diagnosed intra-axial histologically proven primary brain tumors assessed on a GE DISCOVERY 3T MR system were included in our study. The ASL derived CBF and DSC derived relative CBF of 42 glioma patients were normalized (ASL-nCBF and DSC-nrCBF) for data analysis. A comparison between the two technique derived perfusion values was drawn using paired t test and correlated using Pearson correlation coefficient.
**Results or Findings:** Out of 42 patients ,  24 were high grade neoplasms (15 glioblastoma multiforme, 1 anaplastic ependymoma, 1 anaplastic oligodendroglioma, 4 lymphomas and 3 medulloblastoma)  18 were low grade neoplasms ( 3 pilocytic astrocytoma,1 pilomyxoid astrocytoma,3 grade II gliomas, 2 grade II astrocytoma, 6 grade II oligodendroglioma, 2 grade II ependymoma and 1 hemangioblastoma).  {27 males and 15 females- mean age 29.78 }. A significant correlation was demonstrated between ASL normalised TBF and DSC normalised TBF using contralateral grey matter (R-0.836) and white matter (R- 0.864).
**Conclusion:** A significant correlation was demonstrated between the ASL and DSC derived normalised perfusion parameters in brain tumors using multiparametric MRI. Our study establishes the ground for claims of ASL to be a viable alternative tool for DSC perfusion.
**Limitations:** Small sample size Pseudocontinous ASL was used perfusion assessment. A multi-delay ASL may serve as a better technique for correlation with T2* perfusion.
**Ethics Committee Approval:** Approved
**Funding for this study:** None
**Author Disclosures:**
Sarbesh Tiwari: Author: nothing to disclose
Sabha Ahmed: Nothing to disclose
Pawan Kumar Garg: Nothing to disclose
Pushpinder Singh Khera: Nothing to disclose
Binit Sureka: Nothing to disclose
Taruna Yadav: Nothing to disclose

### RPS 311-15

**Intracranial cystic lesions: wherever there is fluid**

*F-E. Badi*, M. Sabiri, S. Lezar, F. Essodegui; Casablanca/MA
*(badi.fatima.ezzahrae@gmail.com)*

**Purpose:** Analyze the different characteristics of intracranial cystic lesions on CT and MRI. Discuss the imaging features that's allow differentiation between the various intracranial cystic lesions. Propose a simplified algorithm allowing to make differential diagnosis of intracranial cystic lesions.
**Methods or Background:** Intracranial cystic lesions are very common findings in neuro-imagery. These lesions can be asymptomatic. At present, there's no established classification for brain cysts. But some features can help to differentiate these lesions, such as the location, the appearance of the wall of the cyst and his thickness, enhancement, the components of the cyst.
**Results or Findings:** The differential diagnosis of intracranial cystic lesions includes a broad spectrum of conditions: normal variants (cavum of the septum pellucidum, Rathke's cyst.), developmental cystic lesions (arachnoid cysts, epidermoid and dermoid cysts.), vascular cystlike structures (choroid plexus cyst.), infectious cysts (hydatid cysts, abscess.) and neoplasic cysts (Glioblastoma, metastasis.). MRI is the method of choice to establish the diagnosis of these cystic lesions by providing information about the anatomic location, size, shape and signal of the lesions as well as their enhancement and their mass effect on adjacent structures.
**Conclusion:** Understanding the spectrum of appearances of the various intracranial cystic lesions improves the diagnostic yield, enables one to understand their pathogenesis, and facilitates patient care.
**Limitations:** None
**Ethics Committee Approval:** Not applicable
**Funding for this study:** Not applicable
**Author Disclosures:**
Fatiha Essodegui: Nothing to disclose
Fatima-Ezzahare Badi: Nothing to disclose
Mouna Sabiri: Nothing to disclose
Samira Lezar: Nothing to disclose

### Research Presentation Session: Neuro

### RPS 411

### Neurological trauma

### RPS 411-1

**Discriminating cognitive motor dissociation from disorders of consciousness through structural MRI**

*P. Pozeg*, J. Jöhr, R. A. Meuli, K. Diserens, V. Dunet; Lausanne/CH
*(polona.pozeg@chuv.ch)*

**Purpose:** Detection of awareness in patients with disorders of consciousness is crucial for diagnosis, treatment, and end-of-life decisions. However, the standard neurobehavioral bedside assessments often overlook subtle signs of consciousness due to fluctuating arousal and other comorbidities hindering the patient's interaction. This retrospective study aimed to develop an MRI-based score for brain lesion assessment to distinguish between patients with and without covert awareness as defined by a novel Motor Behavior Tool.
**Methods or Background:** Retrospective data of 128 patients with severe brain injury were split into a development and validation set. We assessed their brain MRIs for lesions in 18 brain regions. Using logistic regression and support vector machine, we first identified a combination of damaged brain structures with a high relevance to discriminate between the patients with and without awareness. For each classifier we constructed an MRI-based score for lesion assessment and estimated its optimal diagnostic cut-off point. We evaluated the performance of the two classifiers on the validation dataset.
**Results or Findings:** The brain regions identified as relevant predictors of the negative outcome were highly overlapping between two classifiers. They included the left mesencephalon, right basal ganglia, right thalamus, right parietal cortex, and left frontal cortex. When tested on the validation dataset, support vector machine showed higher specificity (0.97, 95% CI: 0.85, 1) than logistic regression (0.90, 95% CI: 0.75, 0.97), whereas sensitivity level was at 0.67 for both classifiers (95% CI: 0.24, 0.94 and 0.22, 0.96, respectively).
**Conclusion:** The MRI-based score assessing brain lesions in patients with disorders of consciousness accurately detected patients with residual awareness. It can valuably complement behavioral evaluation as it is time-efficient and requires only a conventional MRI.
**Limitations:** n/a
**Ethics Committee Approval:** The local ethics committee (CER-VD-142/09) approved the study.
**Funding for this study:** Swiss National Science Foundation
**Author Disclosures:**
Jane Jöhr: Nothing to disclose
Vincent Dunet: Nothing to disclose
Polona Pozeg: Nothing to disclose
Reto Antoine Meuli: Nothing to disclose
Karin Diserens: Nothing to disclose

### RPS 411-2

**Reduced rate of brain waste clearance for TBI patients: glymphatic function assessed via MRI within a short time**
*J. Bao*, X. Wang, Y. Zhang, J. Cheng; Zhengzhou/CN
*(baoguojianfeng@gmail.com)*

**Purpose:** Dementia is commonly observed in traumatic brain injury (TBI), however, there is no consensus on the mechanism of how TBI promotes susceptibility to the development of dementia. Recent studies have shown that TBI significantly affects the glymphatic function on the rate of clearance of fluorescence tracer in mice. Herein, the TBI glymphatic function was assessed via MRI within a short time (1 hour) by measuring the rate of brain waste clearance.
**Methods or Background:** This study includes 16 TBI patients and 7 relative healthy controls with no history of TBI. T1W brain images were obtained three times with 20 min intervals after 4 hours lumbar intrathecal injections of gadolinium. Along the pathway of CSF propagation, 4 regions in each hemisphere were predefined. And the signal intensity of CSF nearby above regions was extracted for analyses. The rate of T1 signal change of two time points was simply calculated using the following equations R_2=((S_2-S_1))⁄S_1 [illegible]d R_3=((S_3-S_1))⁄S 1, where R is the rate and S is the signal intensity of corresponding time point.
**Results or Findings:** Significantly higher signal change rates were observed in 7 predefined CSF ROIs in TBI patients compared to controls within 1 hour scan after 4 hour injection gadolinium. The TBI patients show relatively slow clearance ability of gadolinium tracer for almost all predefined CSF regions.
**Conclusion:** Besides previously reported the delayed enhancement after 24 hours injection of tracer, this work proved the glymphatic dysfunction can be assessed by signal intensity dynamic change rate within 20 min after 4 hour injection of the tracer as well. The deduced rate of tracer clearance in TBI patients may reflect damage of brain glymphatic system.
**Limitations:** The number of subjects.
**Ethics Committee Approval:** Ethics Committee of the Zhengzhou University
**Funding for this study:** NNSF (No.81601470)
**Author Disclosures:**
Jingliang Cheng: Nothing to disclose
Jianfeng Bao: Nothing to disclose
Xiao Wang: Nothing to disclose
Yong Zhang: Nothing to disclose

### RPS 411-4

**Diagnostic accuracy of dual-layer spectral CT for detecting post-traumatic prevertebral haematoma of the cervical spine**
*S. Sedaghat*, M. Both, O. Jansen; Kiel/DE
*(samsedaghat1@gmail.com)*

**Purpose:** To investigate the diagnostic value of dual-layer spectral detector computed tomography (SDCT) in detecting posttraumatic prevertebral hematoma of the cervical spine
**Methods or Background:** Thirty-eight patients with posttraumatic imaging of the cervical spine were included in this study and received both SDCT and MRI examinations. MRI was set as reference and electron density (ED) images were compared to conventional CT (CCT) images alone.
**Results or Findings:** 18 prevertebral hematomas were identified. CCT showed a sensitivity of 33-50% and a specificity of 75-80%, while for ED reconstructed images sensitivity was 77-83% and specificity 85-90%. Accuracy increased from 55-66% to 84% by using ED images. The sizes of prevertebral hematoma on CCT or ED images were not significantly under- or overestimated compared to the MRI reference. There was a significant difference between the two readers for measuring hematoma sizes on CCT (p=0.04). Readers showed an excellent inter-rater reliability (kappa = 0.82) for C + ED images and a moderative inter-rater reliability (kappa = 0.44) for CCT.
**Conclusion:** Diagnostic accuracy for detecting post-traumatic prevertebral hematoma is improved on SDCT by using combined electron density reconstructions compared to conventional images alone.
**Limitations:** The main limitation is the small number of patients.
**Ethics Committee Approval:** The study was approved by the responsible Institutional Review Board (IRB).
**Funding for this study:** None.
**Author Disclosures:**
Sam Sedaghat: Nothing to disclose
Marcus Both: Nothing to disclose
Olav Jansen: Nothing to disclose

### RPS 411-6

**Brain glymphatic system is impaired in TBI found with MRI: the first human study**
*X. Wang*, J. Bao, Y. Zhang, J. Cheng; Zhengzhou/CN
*(daisywangxiao@outlook.com)*

**Purpose:** The pathology of neurodegeneration following the traumatic brain injury (TBI) is still unknown. Recently, a new brain waste clearance system, 'glymphatic system', was proved to be damaged in TBI rodent. Herein, the functionality of human brain glymphatic system of TBI patients and related healthy controls was evaluated using MRI.
**Methods or Background:** The glymphatic function was assessed by T1W MRI with intrathecal gadolinium injection as tracer. Images were obtained from TBI (n = 16) and relatively healthy controls (n = 7) at 4 time points. Along the cerebrospinal fluid (CSF) drawing pathway, Fig.1 predefined 4 regions of interest (ROIs), were automatically generated using standard brain atlas. Each ROI was subsequently split into sub-ROIs including CSF and gray matter (GM) separately for analyzing the signal enhancement characteristics. The percentage changes of signal were used for group comparisons.
**Results or Findings:** After 24-hour injection, the percentage changes of T1 signal intensity were significantly higher in TBI patients than controls for CSF and GM, but not for WM in all ROIs (not show here). The CSF enhancement seems more pronounced than GM enhancement. The higher T1 signal in TBI patient at 24 h can be attributed to the reduced efficiency of the contrast agent clearance.
**Conclusion:** The significantly delayed enhancements in CSF and GM in TBI group indicates that the brain glymphatic system is impaired in TBI. Glymphatic dysfunction may provide a new clue to understand its pathogenesis of the cognitive impairments along with TBI in later life. What's more, this study reconfirmed the MRI scan with gadolinium intrathecal administration may hold a potential to explore the glymphatic function in human.
**Limitations:** None
**Ethics Committee Approval:** This study was approved by Ethics Committee of the Zhengzhou University.
**Funding for this study:** NNSF (No. 81601470)
**Author Disclosures:**
Jingliang Cheng: Nothing to disclose
Jianfeng Bao: Nothing to disclose
Xiao Wang: Nothing to disclose
Yong Zhang: Nothing to disclose

### Research Presentation Session: Neuro

### RPS 511

### Foetal and paediatric neurological imaging

### RPS 511-1

**Magnetic resonance spectroscopy of the paediatric cerebellum: what is the range of normal metabolite ratios?**

*V. J. Balian*, D. Connolly, S. Mordekar, N. Hoggard; Sheffield/UK
*(balianvartan@gmail.com)*

**Purpose:** Proton magnetic resonance spectroscopy (1H-MR spectroscopy) is a valuable tool in brain MR imaging used to identify metabolic changes in neurological disorders. Its diagnostic role is well established in adults, particularly for cerebellar pathology. In children this has not occurred due to difficulty in obtaining normal control data. We aim to define a range of normal cerebellar metabolite ratios in children, and identify when normal adult levels are reached.
**Methods or Background:** Healthy children between the ages of 0 and 16 were prospectively included over the study period from November 2018 to November 2020. Metabolite concentrations of n-acetylaspartate (NAA), creatine (Cr) and choline (Ch) and NAA/Cr, NAA/Cr (height), Cho/Cr, and NAA/Ch ratios were acquired in the superior vermis and right cerebellar hemisphere in each participant using 1H-MR single voxel point-resolved spectroscopy (PRESS) at 3 T with echo time (TE) 144 ms, and repetition time (TR) 2000ms.
**Results or Findings:** In the 80 children who met the inclusion criteria, we showed that metabolite ratios increase steadily under the age of 3, but thereafter follow a stable pattern. From the age of 3 onwards, mean ± standard deviation metabolite ratios in the superior vermis were 0.99 ± 0.07 (NAA/Cr), 0.94 ± 0.06 (NAA/Cr (h)), 0.90 ± 0.08 (Ch/Cr), and 1.10 ± 0.08 (NAA/Ch); and ratios in the right cerebellar hemisphere were 1.07 ± 0.16 (NAA/Cr), 0.96 ± 0.10 (NAA/Cr (h)), 0.92 ± 0.08 (Ch/Cr), and 1.17 ± 0.15 (NAA/Ch).
**Conclusion:** We have shown for the first time that the paediatric range of normal cerebellar metabolite ratios acquired by long TE 1H-MR spectroscopy reach adult levels by the age of 3 onwards.
**Limitations:** Relatively small sample size (although largest in current literature); and some data were collected retrospectively.
**Ethics Committee Approval:** Yes.
**Funding for this study:** No funding.
**Author Disclosures:**
Vartan Jacques Balian: Nothing to disclose
Nigel Hoggard: Nothing to disclose
Santosh Mordekar: Nothing to disclose
Daniel Connolly: Nothing to disclose

### RPS 511-2

**Foetal brainstem visualisation using super-resolution and postmortem MRI – a first step towards AI-based prognostication in foetal neuroimaging**

*G. O. Dovjak*[1], G. Gruber[2], P. Brugger[1], M. Weber[1], E. Schwartz[1], D. Prayer[1], G. Kasprian[1]; [1]Vienna/AT, [2]Krems/AT
*(gregor.dovjak@meduniwien.ac.at)*

**Purpose:** The fetal posterior fossa is a complex anatomic compartment that consists of different functional systems. This study aimed to quantitatively assess prenatal brainstem pathologies with reconstructed “super-resolution” fetal MRI and postmortem data using biometry. The resulting insights aim to increase diagnostic accuracy and characterization of disorders in hindbrain segmentation.
**Methods or Background:** Fetuses with brainstem malformations and an available fetal MRI, postmortem MRI and a confirmatory autopsy were included. Three orthogonal T2-weighted prenatal brain sequences were algorithmically reconstructed into a three-dimensional “super-resolution” isovoxel dataset. Based on this data, as well as on high resolution isovoxel T2-weighted postmortem sequences, the volumes of the midbrain, pons, medulla oblongata and cerebellum were segmented using ITK-SNAP. Further, the area and diameters of these structures were quantified.
**Results or Findings:** 29 fetuses with a mean gestational age of 26.9±5.3, ranging from 18+3 to 37+5 gestational weeks were included. The brainstem findings included aqueductal stenosis, z-shaped brainstem, pontocerebellar hypoplasia, elongated and thin brainstem, brainstem disconnection, and dysplastic tectum. The associated cerebellar pathologies consisted of cerebellar hypoplasia, rhombencephalosynapsis, and dysplastic/hypoplastic vermis. All substructures of the fetal brainstem and cerebellum could be consistently measured in both modalities. The super-resolution brainstem volumetry allowed for a more detailed assessment of the brainstem disorders compared to standard two-dimensional evaluation.
**Conclusion:** A detailed prenatal three-dimensional analysis of posterior fossa structures is valid and possible using novel super-resolution reconstruction. Correlating prenatal super-resolution MRI data with postnatal neurodevelopmental long-term outcomes using computerized imaging based learning methods will optimize prenatal counseling of posterior fossa malformations. This will help to reduce anxiety of parents with ambiguous results during fetal organ screening examinations and improve parental counseling.
**Limitations:** Retrospective, small case amount
**Ethics Committee Approval:** n/a
**Funding for this study:** n/a
**Author Disclosures:**
Ernst Schwartz: Nothing to disclose
Gregor Oliver Dovjak: Nothing to disclose
Peter Brugger: Nothing to disclose
Gerlinde Gruber: Nothing to disclose
Daniela Prayer: Nothing to disclose
Gregor Kasprian: Nothing to disclose
Michael Weber: Nothing to disclose

### RPS 511-3

**The prenatal origins of human brain asymmetry: lessons learned from a cohort of foetuses with body lateralisation defects**

*P. Kienast*[1], E. Schwartz[1], M. C. Diogo[1], G. Gruber[2], P. Brugger[1], L. Bartha-Doering[1], M. Weber[1], D. Prayer[1], G. Kasprian[1]; [1]Vienna/AT, [2]Krems/AT
*(pat.kienast@gmail.com)*

**Purpose:** This study investigated whether body lateralization defects or ciliopathies have a morphological effect on physiological asymmetry patterns in fetal brains.
**Methods or Background:** Structural brain asymmetries are linked to asymmetries in the functional representation of cognitive functions and abilities. Knowledge about structural brain asymmetries of human fetuses with body lateralization defects—congenital diseases in which visceral organs are partially or completely incorrectly positioned—can improve our understanding of the developmental origins of hemispheric brain asymmetry. This study investigated structural brain asymmetry in 21 fetuses, who were diagnosed with different types of lateralization defects (situs inversus totalis, situs inversus abdominalis, situs ambiguus and isolated dextrocardias), five fetuses with ciliopathies; and 26 age-matched healthy control cases, between 22 and 34 gestational weeks (GW) of age. A database of 4,007 1.5 Tesla fetal MRIs was accessed and searched for the corresponding diagnoses. Specific temporal lobe brain asymmetry indices were quantified using in vivo, super-resolution processed MR brain imaging data.
**Results or Findings:** Results revealed that perisylvian fetal structural brain lateralization patterns and asymmetry indices did not differ between cases with lateralization defects, ciliopathies, and normal controls.
**Conclusion:** Molecular mechanisms involved in the definition of the right/left body axis—including cilium-dependent lateralization processes—appear to occur independently from those involved in the early establishment of structural human brain asymmetries. Atypically inverted early structural brain asymmetries are similarly rare in individuals with lateralization defects and may have a complex, multifactorial, neurodevelopmental background with currently unknown postnatal functional consequences.
**Limitations:** The main limiting factor of the study was the small number of MRIs with sufficiently well represented brains of fetuses who suffered from lateralization defects.
**Ethics Committee Approval:** Approval-number 1900/2016, MUV.
**Funding for this study:** No author has received payment specifically for the development of this study.
**Author Disclosures:**
Lisa Bartha-Doering: Nothing to disclose
Ernst Schwartz: Nothing to disclose
Mariana Cardoso Diogo: Nothing to disclose
Patric Kienast: Nothing to disclose
Peter Brugger: Nothing to disclose
Gerlinde Gruber: Nothing to disclose
Daniela Prayer: Nothing to disclose
Gregor Kasprian: Nothing to disclose
Michael Weber: Nothing to disclose

**RPS 511-4**

**"SyMRI"-based quantitative mapping of the human foetal brain**

*V. Schmidbauer*, G. O. Dovjak, M. S. Yildirim, B. Ulm, D. Prayer, G. Kasprian; Vienna/AT
*(schmidbauervictor@gmail.com)*

**Purpose:** Based on a single multi-dynamic multi-echo (MDME) sequence, the MR data post-processing software "SyMRI" generates a variety of MR contrasts characterizing tissue-specific properties. The aim of this study is to assess the feasibility of "SyMRI" in the early prenatal characterization of brain maturation.
**Methods or Background:** 47 fetuses were examined using a standardized fetal MRI protocol (1.5 Tesla). MDME sequence (acquisition time: 3 min. 20 sec.)-based post-processing was performed using "SyMRI" (Synthetic MR AB, Linköping, Sweden; Version 11.1.5). T1-relaxation time (T1R), T2-relaxation time (T2R) (ms), and proton density (PD) (%) of the germinal matrix were determined.
**Results or Findings:** Fetal motion limited the use of "SyMRI" in 39/47 (82.98%). Non-motion degraded images of diagnostic quality were available in 8/47 (17.02%) [gestational age (GA): 23+6; 24+6; 25+4; 25+4; 25+5; 26+6; 32+4; 34+0]. T1R, T2R, and PD of the germinal matrix were determined in six fetuses: range GA: 23+6 – 26+6 (T1R: 1195 – 1399/T2: 137 – 150/PD: 75.4 – 82.7).
**Conclusion:** "SyMRI" for MDME-based MR post-processing is feasible to provide MR contrasts of diagnostic image quality, although limited by fetal motion. Maturational changes of transient brain structures such as the germinal matrix can be quantified in utero and in vivo. In a small sample of non-motion distorted MDME acquisitions, higher GA is consistently associated with higher T1R of the germinal matrix.
**Limitations:** Small sample size
**Ethics Committee Approval:** All procedures were carried out in accordance with the Declaration of Helsinki.
**Funding for this study:** None
**Author Disclosures:**
Victor Schmidbauer: Nothing to disclose
Mehmet Salih Yildirim: Nothing to disclose
Barbara Ulm: Nothing to disclose
Gregor Oliver Dovjak: Nothing to disclose
Daniela Prayer: Nothing to disclose
Gregor Kasprian: Nothing to disclose

**RPS 511-5**

**Neuraxial deformation stress in "Complex" chiari malformation**

M. D. Papachristou[1], *R. Ward*[2], V. Agarwal[2]; [1]Warrendale, PA/US, [2]Pittsburgh, PA/US
*(Wardr2@upmc.edu)*

**Purpose:** 1. To review the radiographic features of "Complex" Chiari 2. To learn metrics of neuraxial deformation stress and integrate them into clinical reports 3. To gain awareness of algorithms used by neurosurgeons to plan appropriate surgical strategy.
**Methods or Background:** Chiari I Malformation (CMI) is a relatively common abnormality familiar to most radiologists. Cerebellar tonsillar herniation accompanied by additional abnormalities is often referred to as "Complex" Chiari (CC). Both CMI and CC patients present generally with cervicomedullary syndrome (CMS), although some authors maintain distinct clinical presentations for these disease processes. Relevant abnormalities may be grouped into deformities of the CVJ (e.g. Basilar invagination, retroflexed dens) and central nervous system (e.g. cervicomedullary kink) components. Because these abnormalities contribute to the overall disease process, such patients often require different surgical procedures to achieve symptom relief. Morphologic features of Complex Chiari may be described in terms of reference lines such as those defined by McRae and Chamberlain. More recently, the neurosurgical community has increasingly recognized the importance of neuraxial deformation stress (NDS) in the pathogenesis of CMS. NDS is a general term encompassing forces acting to deform the long-axis of the CNS, which, for this subject, concerns the normal cranio-caudal axis of the brainstem and upper cervical spinal cord. Mechanisms of NDS may be subdivided into ventral brainstem compression (VBSC) and stretch-induced myelopathy (SIM). The risk of ventral brainstem compression and stretch-induced myelopathy associated with skull base/CVJ variants is subsumed into certain radiographic metrics: clivo-axial angle (CXA) and Grabb's line (pB-C2). Familiarity with the imaging findings and relevant metrics of Complex Chiari can improve radiologists' relevance to our neurosurgical colleagues and the patients we both serve.
**Results or Findings:** N/a
**Conclusion:** N/a
**Limitations:** N/a
**Ethics Committee Approval:** N/a
**Funding for this study:** No disclosures.
**Author Disclosures:**
Marios D Papachristou: Nothing to disclose
Rebecca Elizabeth Ward: Nothing to disclose
Vikas Agarwal: Nothing to disclose

**RPS 511-6**

**Sex-related differences in extremely preterm neonates – a quantitative MR data analysis of infra- and supratentorial regions**

*V. Schmidbauer*, G. O. Dovjak, M. C. Diogo, M. S. Yildirim, K. Goeral, D. Prayer, G. Kasprian; Vienna/AT
*(schmidbauervictor@gmail.com)*

**Purpose:** DTI parameters [fractional anisotropy (FA)/apparent diffusion coefficient (ADC)] and tissue-specific properties [T1-relaxation time (T1R)/T2-relaxation time (T2R)] allow for a quantitative MRI assessment. The aim of this study is to investigate sex-related maturity differences of infra- and supratentorial regions in extremely preterm infants by means of a quantitative MR approach.
**Methods or Background:** 35 extremely preterm neonates [born <28 weeks gestational age (GA)] (female: n=17/male: n=18) were examined at term-equivalent age using a standardized neonatal MRI protocol (1.5 Tesla). Quantitative analysis was applied in the midbrain and the left/right posterior limb of the internal capsule (PLIC). DTI data post-processing was performed [IntelliSpace Portal (Philips Healthcare Systems; Version 10)] to determine FA-/ADC-values. T1R/T2R was determined based on multi-dynamic multi-echo sequence post-processing using "SyMRI" (Synthetic MR AB, Linköping, Sweden; Version 11.1.5). A Pearson's correlation analysis was performed to assess correlations between T1R/T2R and FA-/ADC-values. ANCOVA (covariate: GA at MRI) was used for group comparison. A paired t-test was used to compare the left and right PLIC in both female and male neonates.
**Results or Findings:** No consistent correlations were observed between T1R/T2R and FA-/ADC-values. T1R of the midbrain was significantly decreased in female compared to male infants (p=0.015). ADC-values/T1R (decreased)/FA-values (increased) differed significantly between the left and right PLIC in both female (ADC: p=0.002/T1R/FA: p<0.001) and male (ADC/T1R/FA: p<0.001) neonates.
**Conclusion:** DTI parameters and tissue-specific properties respond to different aspects of brain maturation. T1R metrics reveal sex-related maturity differences that indicate a more advanced state of brain maturation in female compared to male neonates. A consistently more advanced state of maturity of the left compared to the right PLIC was found using both mapping approaches.
**Limitations:** Small sample size
**Ethics Committee Approval:** All procedures were performed in accordance with the Declaration of Helsinki.
**Funding for this study:** None
**Author Disclosures:**
Victor Schmidbauer: Nothing to disclose
Mehmet Salih Yildirim: Nothing to disclose
Mariana Cardoso Diogo: Nothing to disclose
Gregor Oliver Dovjak: Nothing to disclose
Daniela Prayer: Nothing to disclose
Katharina Goeral: Nothing to disclose
Gregor Kasprian: Nothing to disclose

### Research Presentation Session: Neuro

### RPS 611

### Ichaemia and infarction in the brain

**RPS 611-1**

**Myosteatosis is correlated with poor clinical outcome in patients who undergo mechanical thrombectomy for acute ischaemic stroke**

*M. Bonatti*[1], A. Comai[1], R. Valletta[1], F. Lombardo[2], F. Ferro[1]; [1]Bolzano/IT, [2]Negrar/IT
*(matteobonatti@hotmail.com)*

**Purpose:** To assess the impact of sarcopenia and myosteatosis on clinical outcome in patients successfully reperfused after acute ischemic stroke.
**Methods or Background:** In this IRB-approved retrospective cohort study, we included 166 patients (84F/82M, 70±13years) who underwent technically successful mechanical thrombectomy for acute ischemic stroke in our

Institution between Jan2016 and Dec2019. 90days mRS was retrieved from Institutional database. ASPECTS and collateral score were assessed on pre-operative CT/triphasic CT-angiography(CTA). Skeletal muscle area (SMA) and mean muscle attenuation at the level of C5 were calculated using free software (ImageJ v1.52). ASPECTS, collateral score, SMA and muscle attenuation were compared to clinical outcome (90days mRS 0-2=good outcome; 3-6=poor outcome).
**Results or Findings:** ASPECTS was significantly higher in patients with good clinical outcome than in those with poor clinical outcome (8.7±1.4 vs. 7.6±2.1, p=0.0006, Mann-Whitney test). The presence of good/intermediate collaterals was significantly associated with good clinical outcome (p=0.0013, Fisher's exact test). SMA was not significantly different between patients with good and poor clinical outcome (41.97±10.29mm2 vs. 41.66±10.59 mm2, p=n.s., Mann-Whitney test). Mean muscle attenuation was significantly higher in patients with good clinical outcome than in those with poor clinical outcome (47.15±9HU vs. 43.5±8HU, p=0.0202, Mann-Whitney test).
**Conclusion:** Beyond other well-known predictors, myosteatosis represents a risk factor for poor clinical outcome in patients who underwent mechanical thrombectomy for acute ischemic stroke. On the other hand, low neck muscle mass didn't correlate with poor outcome.
**Limitations:** Retrospective study. Mechanical thrombectomy performed by four different interventional radiologists.
**Ethics Committee Approval:** IRB-approved retrospective cohort study
**Funding for this study:** None
**Author Disclosures:**
Matteo Bonatti: Nothing to disclose
Riccardo Valletta: Nothing to disclose
Fabio Lombardo: Nothing to disclose
Alessio Comai: Nothing to disclose
Federica Ferro: Nothing to disclose

### RPS 611-2

**Endovascular treatment of intracranial aneurysms in small peripheral vessel segments – efficacy and intermediate follow-up results of flow diversion with the silk vista baby low-profile flow diverter**

*M-S. Schüngel*, K-T. Hoffmann, U. Quäschling, S. Schob; Leipzig/DE
*(Marie-Sophie.Schuengel@medizin.uni-leipzig.de)*

**Purpose:** Low-profile flow diverter stents (FDS) quite recently amended peripheral segments as targets for hemodynamic aneurysm treatment, however, reports on outcomes, especially later than 3months, are scarce. This study therefore reports our experience with the novel silk vista baby (SVB) FDS and respective outcomes after eight and eleven months with special respect to specific adverse events.
**Methods or Background:** 44patients harbouring 47aneurysms treated with the SVB between June 2018 and December 2019 were included. Clinical, procedural and angiographic data were collected. Follow-ups were performed on average after three, eight and eleven months, respectively. Treatment effect was assessed using the O´Kelly Marotta (OKM) grading system.
**Results or Findings:** Overall, angiographic follow-ups were available for 41patients/ 45aneurysms. Occlusion or significant reduction in aneurysmal perfusion (OKM:D1, B1-B3 and A2-3) was observed in 98% of all aneurysms after eight months. Only 2% of the treated aneurysms remained morphologically unaltered and without an apparent change in perfusion (OKMA1). Adverse events in the early post-interventional course occurred in seven patients: Permanent and transient stent occlusion, acute infarction and progressive inflammatory mass effect were observed in one patient, respectively. Two patients suffered hemodynamically relevant device-induced vasospasm. Excepting two patients, however, all patients recovered completely within 90days after endovascular treatment. One death occurred in context of a major SAH due to an acutely ruptured, multi-segmental dissecting vertebra-basilar aneurysm.
**Conclusion:** The SVB achieves sufficient occlusion rates of intracranial aneurysms originating from peripheral segments, which are comparable to prior established conventional FDS with acceptably low complication rates. However, alteration of a hemodynamic equilibrium in distal localizations requires special attention to prevent ischemic events.
**Limitations:** Limitations are a small patient collective and missing long-term angiographic follow-up data of our cohort.
**Ethics Committee Approval:** Approval was given by the institutional ethics committee.
**Funding for this study:** None
**Author Disclosures:**
Karl Titus Hoffmann: Nothing to disclose
Marie-Sophie Schüngel: Nothing to disclose
Stefan Schob: Nothing to disclose
Ulf Quäschling: Nothing to disclose

### RPS 611-3

**Artificial intelligence in acute stroke: an early health technology assessment of vessel-occlusion detection on computed tomography**

*K. G. van Leeuwen*[1], F. J. A. Meijer[1], S. Schalekamp[1], M. Rutten[2], E. van Dijk[1], T. Govers[1], M. De Rooij[1], B. Van Ginneken[1]; [1]Nijmegen/NL, [2]'s-Hertogenbosch/NL
*(kicky.vanleeuwen@radboudumc.nl)*

**Purpose:** To demonstrate the cost-effectiveness of artificial intelligence (AI) software to aid in the detection of intracranial vessel occlusions in stroke compared to standard care by performing early health technology assessment.
**Methods or Background:** We used a Markov based model from a societal perspective in a UK setting to demonstrate the potential value of an AI tool reported in expected incremental costs (IC) and effects (IE) in quality adjusted life years (QALYs). Initial population existed of patients suspected of stroke based on symptoms and exclusion of other causes as demonstrated by non-contrast cerebrum CT. Input parameters for the model were predominantly based on stroke registry data from the UK and complemented with pooled outcome data from large randomized trials. Parameters were varied to demonstrate model robustness.
**Results or Findings:** The AI strategy with its base-case parameters (6% missed diagnoses of intra-arterial therapy eligible patients by clinicians, $40 per AI analysis, 50% reduction of missed vessel occlusions by AI) resulted in modest cost-savings and incremental QALYs over the projected lifetime (IC: -$156, -0.23%; IE: +0.01 QALYs, +0.07%) per ischaemic stroke patient. Within a ninety-day window after treatment no financial (IC: +$60) and negligible QALY (IE: +0.0001) gain was observed. For each yearly cohort of patients in the UK this translates to a total cost saving of $11 million.
**Conclusion:** We showed that computer aided thrombus detection in emergency care has the potential to increase health and save costs. Results may contribute to the debate on the investments, financial accountability and reimbursement for the clinical use of AI technology.
**Limitations:** Parameter values of the model were based on results from previous studies.
**Ethics Committee Approval:** N.a.
**Funding for this study:** This work has not received any funding.
**Author Disclosures:**
Ewoud van Dijk: Nothing to disclose
Frederick Jan Anton Meijer: Nothing to disclose
Matthieu Rutten: Nothing to disclose
Bram Van Ginneken: Founder: Thirona Shareholder: Thirona Other: Delft Imaging Other: Mevis Medical Solutions
Kicky Gerhilde van Leeuwen: Nothing to disclose
Tim Govers: Nothing to disclose
Steven Schalekamp: Nothing to disclose
Maarten De Rooij: Nothing to disclose

### RPS 611-4

**Prognostic evaluation of cerebral venous thrombosis using clinical, structural imaging characteristics and clot burden**

*J. S C*, S. Nandikoor, S. Kumar, V. Kamath; Bangalore/IN
*(jayashreesc@gmail.com)*

**Purpose:** Cerebral venous thrombosis (CVT) is an uncommon cause of stroke with varied presentations and outcomes. The study aims to perform prognostic evaluation of CVT using clinical, imaging characteristics and extent of clot
**Methods or Background:** Prospective observational study conducted in a tertiary care center (April 2018 to May 2019) including patients with symptomatic CVT. Demographic data, clinical presentation and Modified Rankin Scale (mRS) (at admission, discharge and follow up (1 and 3 month)) were documented. MRI brain was evaluated for extent of parenchymal involvement and venogram for extent of dural sinuses and cerebral venous thrombosis. Clot burden score (CBS) was computed as the sum of the thrombosis degree (1-partial or 2-complete) in dural sinuses / cerebral veins. The parenchymal changes and CBS was correlated with mRS score.
**Results or Findings:** 61 (M:37; F:24) patients were included (age: 9 to 77years). Poor mRS (at discharge and/or followup) was seen in patients presenting with focal neurological deficit, altered sensorium, large area of parenchymal involvement, cytotoxic edema and haemorrhage ($p < 0.05$). Cerebral vein CBS had moderate correlation with mRS score in patients who had no parenchymal involvement at presentation and follow up ($p< 0.05$). Deep cerebral venous system CBS in patients with small area of parenchymal involvement ($p< 0.05$) and superficial cerebral venous system CBS in patients with hemorrhagic infarct showed moderate correlation with mRS on follow up ($p< 0.05$). Total CBS and cerebral sinus CBS in patients with hemorrhagic infarct at presentation ($p< 0.05$) had moderate correlation with mRS.
**Conclusion:** Clinical and structural imaging characteristics can be of help in predicting the clinical outcome. Quantitative clot burden measurement convey additional prediction on outcomes of CVT.

**Limitations:** chronic CVT - not included in study
**Ethics Committee Approval:** Has been approved
**Funding for this study:** No funding sources
**Author Disclosures:**
Shrivalli Nandikoor: Nothing to disclose
Savith Kumar: Nothing to disclose
Vikram Kamath: Nothing to disclose
Jayashree S C: Nothing to disclose

### RPS 611-5

**High-resolution MRI demonstrates signal abnormalities of the 3rd cranial nerve in giant-cell arteritis patients with 3rd cranial nerve impairment**

*S. Mournet*; Paris/FR
*(sandy.mournet@gmail.com)*

**Purpose:** To determine the sensitivity and specificity of high resolution (HR) MRI for detecting signal abnormalities of cranial nerves (CN) in giant cell arteritis (GCA) patients presenting with diplopia.
**Methods or Background:** This IRB-approved retrospective single-center study included GCA patients who underwent 3T HR MRI from December 2014 to January 2020. Two radiologists, blinded to all data, individually assessed for the presence of enhancement of the 3rd, 4th and/or 6th CN on post-contrast HR imaging and high-signal intensity on HR T2-WI, for signal abnormalities of extraocular muscles and the brainstem, and for inflammatory changes of the ophthalmic and extracranial arteries. A Fisher's Exact test was used to compare patients with or without diplopia.
**Results or Findings:** 64 patients (42/64 (66%) women and 22/64 (34%) men, mean age 76.3 +/- 8 years) were included. 14/64 (21.9%) presented with diplopia. 3rd CN enhancement was detected in 7/8 (87.5%) patients with 3rd CN impairment, as compared to no patients with 4th or 6th CN impairment or to patients without diplopia, p<0.001. 3rd CN abnormal high-signal intensity on HR T2-WI was detected in 4/5 (80%) patients with 3rd CN impairment versus none of other patients, p<0.001. Sensitivity, specificity, predictive positive value and negative predictive value for detecting 3rd CN signal abnormalities were of 0.88, 1, 1 and 0.99 and 0.8, 1, 1 and 0.98 for post-contrast HR imaging and HR T2-WI, respectively.
**Conclusion:** HR MRI had excellent diagnostic sensitivity and specificity when detecting signal abnormalities of the 3rd CN in GCA patients presenting with 3rd CN impairment.
**Limitations:** However, one should remain cautious given the lack of a control group consisting of non-GCA patients.
**Ethics Committee Approval:** Approbation by an Institutional Review Board.
**Funding for this study:** No financial support to disclose
**Author Disclosures:**
Sandy Mournet: nothing to disclose

### RPS 611-6

**Subarachnoid haemorrhage, CT head and lumbar puncture - can our words influence clinicians actions?**

*M. Spurr*, R. Hunt; Bristol/UK
*(matthew.spurr1@nhs.net)*

**Purpose:** Subarachnoid haemorrhage (SAH) guidelines advise a non-contrast computed tomography (CT) head followed by lumbar puncture (LP) if the scan is negative. This project aimed to assess if LPs are performed appropriately and establish whether report wording impacts LP rate.
**Methods or Background:** CT head studies were analysed at a tertiary Neurosurgical centre over 2 months and examinations performed for SAH identified. LP rate was assessed following a negative CT examination. Language within reports was evaluated to assess if it affected LP rate. All CT head reports could be categorised according to how the report concluded. There were 4 styles: 1. No advice provided 2. CT cannot exclude SAH 3. No contraindication to LP 4. LP recommended.
**Results or Findings:** 2433 CTs were analysed; 88 were performed for SAH. Overall 48% of patients with a negative scan underwent LP. If no advice was given LP rate was 23%. CT cannot exclude SAH, 44%; no LP contraindication, 67% and LP recommended, 79%.
**Conclusion:** CT report wording impacted LP rate. The most effective way for a Radiologist to influence clinical practice is to make a recommendation.
**Limitations:** Study size
**Ethics Committee Approval:** None required
**Funding for this study:** None
**Author Disclosures:**
Rebecca Hunt: Nothing to disclose
Matthew Spurr: Nothing to disclose

### RPS 611-7

**Risk assessment of haemorrhagic transformation of middle cerebral artery occlusion acute ischaemic stroke using multimodal imaging: a case-control study**

*C. Li*; Shanghai/CN
*(11111220032@fudan.edu.cn)*

**Purpose:** To identify the multimodal image parameters most predictive of haemorrhagic transformation (HT) in acute ischaemic stroke (AIS) with middle cerebral artery occlusion (MCAO).
**Methods or Background:** MCAO AIS patients who underwent baseline (CT perfusion) CTP/ (CT angiography) CTA + MRI/ (susceptibility-weighted imaging) SWI within 12 hours of symptom onset and follow-up SWI/MR angiography within 3 weeks were included from January 2016 to December 2019. Baseline CTP data were postprocessed to generate maps of different perfusion parameters. Collateral flow was assessed on the baseline CTA. The infarct volume, imaging markers of cerebral small vessel disease and MCAO recanalization were assessed on the baseline MRI/ baseline SWI/ follow-up MR angiography. Outcome event, HT was defined according to European Cooperative Acute Stroke Study II criteria on follow-up SWI imaging. Multivariate logistic regression analysis identified independent predictors of HT and the receiver operating characteristic curve selected an optimal threshold.
**Results or Findings:** 33 (52%) developed HT of 63 consecutive MCAO AIS patients who met inclusion criteria from 181 ones. Multivariate logistic analysis revealed poor collateral flow (odds ratio, 8.003; 95% confidence interval, 6.912-8.949; P=0.021) and relative permeability surface (PS) (odds ratio, 13.254; 95% confidence interval, 10.611-19.926; P<0.001) were independent predictors of HT. The poor collateral flow predicted HT with 36% sensitivity and 93% specificity. The optimal relative PS threshold for predicting HT was 2.3 (area under the curve, 0.853; 95% confidence interval, 0.811-0.889), with 94% sensitivity and 60% specificity.
**Conclusion:** For AIS patients with MCAO, the strongest predictors of HT on the multimodal image protocol were poor collateral flow on CTA and increased relative PS on CTP.
**Limitations:** Not applicable.
**Ethics Committee Approval:** The Ethics Committee of Huashan Hospital, Fudan University (2020-939).
**Funding for this study:** The National Natural Science Foundation of China (81771788).
**Author Disclosures:**
Chanchan Li: Nothing to disclose

### Research Presentation Session: Neuro

### RPS 711

### Imaging the brain

### RPS 711-1

**The onset of schizophrenia prior to the end of brain maturation alters grey matter volume loss**

*R. Antulov*[1], A. Ruzic Barsic[2], D. Miletić[3], G. Rubesa[3]; [1]Esbjerg/DK, [2]Opatija/HR, [3]Rijeka/HR
*(ronald.antulov@outlook.com)*

**Purpose:** Brain maturation (BM) is considered completed around the age of 25, when prefrontal cortex maturation has been achieved. Previous studies showed that early onset schizophrenia, prior to the age of 18, causes a more severe clinical presentation. The aim of our study was to investigate grey matter volume (GMV) alterations between schizophrenia patients (SP) with disease onset (DO) before completed BM and after completed BM.
**Methods or Background:** The study included 100 SP which were divided into two groups of similar size according to DO – before the age of 25 (BABM) and after the age of 26 (AABM). The control group (CP) consisted of 50 healthy individuals. Brain magnetic resonance imaging was acquired on a 1.5 T scanner. Variance and co-variance analysis for GMV in correlation with the age of DO (BABM and AABM), as well as in correlation with the number of psychotic episodes (PE), including interactions and co-variables for total brain volume and age were calculated. Voxel-based morphometry (VBM) analyses were performed between groups.
**Results or Findings:** A greater number of PE resulted in GMV reduction in both groups, but more in BABM. GMV reduction caused by multiple PE is much less pronounced in AABM. VBM analyses detected GMV reduction in the frontal cortex and the cerebellum when BABM was compared to AABM and CP respectively.
**Conclusion:** Early DO of schizophrenia, prior to full BM, results in GMV reduction in comparison with healthy subjects and SP with later DO after completed BM. A more prominent effect of schizophrenia on the developing brain compared to the effect on a fully developed brain is suggested.
**Limitations:** The study groups should include more subjects.
**Ethics Committee Approval:** Study approved by Ethical Committee of the Clinical Hospital Center Rijeka, Croatia.
**Funding for this study:** None.
**Author Disclosures:**
Antonija Ruzic Barsic: Nothing to disclose
Gordana Rubesa: Nothing to disclose
Ronald Antulov: nothing to disclose
Damir Miletić: Nothing to disclose

### RPS 711-2

**Aberrant dynamic functional network connectivity in cirrhotic patients without overt hepatic encephalopathy**

*J-Y. Shi*, H-J. Chen; Fuzhou/CN
*(sjy0311926@163.com)*

**Purpose:** This study aimed to explore dynamic functional network connectivity (FNC) differences and to elucidate their association with neurocognitive changes in cirrhotic patients.
**Methods or Background:** 54 cirrhotic patients and 42 controls underwent resting-state functional magnetic resonance imaging. Psychometric hepatic encephalopathy score(PHES)was used to assess neurocognitive function. Independent component analysis was performed to identify the components of seven intrinsic brain networks, including sensorimotor (SMN), auditory, visual, cognitive control (CCN), default mode (DMN), subcortical (SC), and cerebellar networks. Sliding window correlation approach was employed to calculate dynamic FNC. FNC states were determined by k-means clustering method, and then functional state analysis was conducted to measure dynamic indices.
**Results or Findings:** The patients showed decreased FNC in State 2, involving the connectivity between posterior subsystem of DMN and CCN (represented by bilateral insular cortex), and in State 3, involving the connectivity between SMN (represented by bilateral precentral gyrus) and SC(represented by bilateral putamen and caudate). The patients spent significantly longer time in State 4 that was with weakest FNC across all networks. We observed a significant correlation between PHES and fraction time/mean dwell time in State 4.
**Conclusion:** Aberrant dynamic FNC may be the underlying mechanism of neurocognitive impairments in cirrhosis. Dynamic FNC analysis may potentially be utilized in investigating cirrhosis-related neuropathological processes.
**Limitations:** The cross-sectional design.
**Ethics Committee Approval:** The Research Ethics Committee of Fujian Medical University Union Hospital. All subjects provided their written informed consent.
**Funding for this study:** The National Natural Science Foundation of China (No. 81501450 and 82071900) and Academic Exchange Fund for Postgraduates of Fujian Medical University
**Author Disclosures:**
Jia-Yan Shi: Nothing to disclose
Hua-Jun Chen: Nothing to disclose

### RPS 711-5

**Prognostic value of brain atrophy in patients with essential tremor treated with MRgFUS thalamotomy: a VBM study**

*E. Tommasino*, F. Bruno, M. Varrassi, A. Catalucci, A. Gagliardi, M. R. Antenucci, L. Pertici, V. Pagliei, A. Splendiani; L'Aquila/IT
*(emanuele.tommasino@gmail.com)*

**Purpose:** Essential Tremor is one of the most common movement disorders and since the FDA approval, MRgFUS Vim thalamotomy is rapidly becoming an effective minimally invasive therapeutic option for the treatment of tremor. Skull density ration (SDR) was proposed as an indication for MRgFUS eligibility. Nevertheless, the role of brain tissue interposed between the target and the ultrasound transducers has never been explored. Therefore, the purpose of our study was to analyse data from our experience to evaluate the correlation between periprocedural technical findings (Accumulated Thermal Dose and Area), instrumental MRI findings, clinical outcome scores, Supratentorial Grey Matter (ST – GM), White Matter (ST – WM), Cerebral Spinal Fluid (ST – CSF) and their possible predictive value.
**Methods or Background:** Forty patients were addressed to the MRgFUS Thalamotomy. Pre-treatment T1-Weighted Sequences automatically segmented in WM, GM and Cerebral Spinal Fluid (CSF) using SPM 12. For each subject, ST- GM, WM, CSF and Total Intracranial Volume (ICV) were computed with The PeekAtlas software toolbox.
**Results or Findings:** In the pre-treatment and procedural settings, ST-GM was significantly positively correlated with the Accumulated Thermal Dose ($p<0,001$) whereas the ATD temperature negatively correlated with ST-CSF and ST-TIV ($p<0,001$). Interestingly, the Accumulated Thermal dose Area negatively correlated with ST-WM and ST-TIV ($p<0,001$).
**Conclusion:** Ultrasound propagation speed is lower in fluids than brain tissues. In addition, WM has an attenuation rate of 1.5 higher than the GM. The difference in the Accumulated Thermal Dose may therefore be explained by the different acoustic properties of normal brain tissues interposed between the transducers and the VIM.
**Limitations:** We did not evaluate the density of the brain tissues but just the volume.
**Ethics Committee Approval:** Local ethical committee approval was obtained.
**Funding for this study:** n/a
**Author Disclosures:**
Alessio Gagliardi: Nothing to disclose
Emanuele Tommasino: Nothing to disclose
Alessandra Splendiani: Nothing to disclose
Marco Varrassi: Nothing to disclose
Maria Rosaria Antenucci: Nothing to disclose
Leonardo Pertici: Nothing to disclose
Alessia Catalucci: Nothing to disclose
Federico Bruno: Nothing to disclose
Valeria Pagliei: Nothing to disclose

### RPS 711-6

**The cerebral perfusion and microstructure integrity pattern in advanced Parkinson's disease**

*Z. Liu*, Y. Zhang, H. Wang, D. Xu, H. You, F. Feng; Beijing/CN
*(m18246157638_1@163.com)*

**Purpose:** To explore the features of cerebral perfusion and microstructural integrity of the whole brain in advanced PD patients and to further investigate the possibility of a relationship between these features and cognition in advanced PD patients
**Methods or Background:** Forty-two subjects (21 PD patients and 21 healthy controls（HC) were recruited with the performance of arterial spin labeling (ASL), diffusion tensor imaging (DTI ) and 3D T1 weighted imaging (3D-T1WI) on a 3T MR scanner. The cerebral blood flow (CBF) of the whole brain, the fractional anisotropy (FA), axial diffusivity (AD), radial diffusivity (RD), mean diffusivity (MD) and grey matter (GM) volume of subcortical and cerebellar regions were measured. Mann-Whitney U-test was used to compare these parameters between PD and HC groups. Spearman’s correlations were used to test correlations between Montreal Cognitive Assessment (MoCA) /Mini-Mental State Examination (MMSE) and CBF/DTI measurements.
**Results or Findings:** The cerebellum CBF, the AD, RD and MD in left_crusII and vermis_VIIIa , FA in the left subthalamic nucleus, right substantia nigra were negatively correlated with MoCA and MMSE in PD. The CBF in bilateral thalamus, right red nucleus, right substantia nigra, most cerebellum regions, bilateral hippocampus and parahippocampus was significantly higher in PD compared to HC. Right thalamus, left_crusI, left_crusII, left_VIIb and right_VIIIb in PD showed significantly higher AD, RD and MD than HC. PD had a significant decrease in GM volume in bilateral thalamus and right globuspallidusexterna.
**Conclusion:** Although hypoperfusion was found in some cortical areas, hyperperfusion and microstructure impairments were detected in most subcortical and cerebellum regions, which may influence cognition, can also be detected. Importantly, right thalamus, left_crusI, left_crusII, left_VIIb and right_VIIIb may be the most crucial regions in the patholgy of PD.
**Limitations:** The sample size is relatively small.
**Ethics Committee Approval:** Ethics Committee of Peking Union Medical College Hospital
**Funding for this study:** None
**Author Disclosures:**
Feng Feng: Nothing to disclose
Hui You: Nothing to disclose
Yiwei Zhang: Nothing to disclose
Dan Xu: Nothing to disclose
Zhaoxi Liu: Nothing to disclose
Han Wang: Nothing to disclose

### RPS 711-7

**Dorsal and ventral pathways interaction for cognitive load evaluation in fMRI lie detection**

I. Dykan, L. Myroniak, K. Lisovyi, D. Gryshchak, O. Zakomornyi, I. Kobzar, M. Makarchuk, *O. Omelchenko*; Kiev/UA

**Purpose:** Cognitive load evaluation is necessary for development of fMRI lie detection method. We propose a unique pattern of the ventral pathway with mesolimbic system activation and ventral pathway with default mode network deactivation for cognitive load evaluation.
**Methods or Background:** Group of 10 healthy volunteers (5F, 5M, aged 21-48) was studied using Philips Ingenia 3.0T. Blocked design fMRI was used. Personally false characteristics were used for stimulation. Stimulation was made vocally. Volunteers were instructed to agree with false characteristics.

For fMRI we used EPI pulse sequence: TR/TE=3000/30ms. Activation and deactivation were modelled with GLM as opposite contrasts. Connectivity was evaluated with ICA. Analysis was made with FSL (Oxford, GB).
**Results or Findings:** Activation medial occipital lobes, medial and inferior temporal lobes bilaterally, left inferior lateral parietal cortex, left medial prefrontal, superior frontal and premotor cortex, left caudate nucleus and nucleus accumbens were shown. Deactivation of lateral occipital cortex, pre-/postecentral gyri, posterior inferior frontal gyri, superior parietal lobuli, precuneus and posterior cingulate cortex bilaterally were shown. Thus, activation of ventral pathway with mesolimbic system, and deactivation of dorsal pathway with default mode network were shown. Extensive pattern of activation and deactivation might indicate the high cognitive load. Dorsal pathway and default mode network regions deactivation might indicate the egocentric frame of reference and short memory suppression. Ventral pathway and mesolimbic regions activation might indicate the allocentric frame of reference, long term memory, emotional and reward-related response activation.
**Conclusion:** Unique pattern of ventral pathway with mesolimbic system activation and dorsal pathway with default mode network deactivation might be used for cognitive load evaluation and fMRI lie detection method development.
**Limitations:** This is experimental study for fMRI lie detection method development.
**Ethics Committee Approval:** Approved by local ethics committee.
**Funding for this study:** No additional funds were used.
**Author Disclosures:**
Mykola Makarchuk: Nothing to disclose
OleksanZakomornyi: Nothing to disclose
Iryna Dykan: Nothing to disclose
Liudmyla Myroniak: Nothing to disclose
Dmytro Gryshchak: Nothing to disclose
Kostiantyn Lisovyi: Nothing to disclose
Oleksii Omelchenko: Consultant: Institute of Nuclear Medicine and Radiology
Ihor Kobzar: Nothing to disclose

### RPS 711-8

**The radio-clinical spectrum of Dyke-Davidoff-Masson syndrome**
B. Baysal, A. Orhan Varoğlu, *M. B. Doğan*; Istanbul/TR
*(mbilaldogan@hotmail.com)*

**Purpose:** Dyke-Davidoff-Masson syndrome (DDMS) is a rare condition presenting with cerebral hemiatrophy, which can be congenital or may develop in early childhood. The diagnosis of the disease is based on clinical and imaging findings and is important in terms of approach and treatment.
**Methods or Background:** In this study, clinical and radiological examinations of eleven patients with a diagnosis of DDMS are discussed. Epidemiological characteristics of the patients, etiological risk factors, presenting symptoms, lateralization of hemiatrophy, and other CT and/or MRI results were evaluated. Descriptive statistics were used to describe the characteristics of the disease.
**Results or Findings:** Eleven patients (4 female, 7 male) were included in our study. The mean age of the patients was 40 years. Six patients had hemiatrophy in the unilateral hemisphere and associated falcin displacement. Atrophy of the basal ganglia was detected in 6 patients and Wallerian degeneration was seen in 6 patients, also. Parenchymal sequel (10/11, 90.9%) and Ipsilateral lateral ventricle (9/11, 81.8%) are the most common imaging findings. Thickening of the skull vault was observed in 6 patients. Elevation of the left petrous ridge (6/11, 54,5%) and mastoid air-cells enlargement (3/11, 27.2%) are other findings. Enlargement of frontal sinus was seen in 4 patients. In addition to parenchymal and skull changes, vascular pathologies such as capillary malformation are observed in 6 patients.
**Conclusion:** In Dyke-Davidoff-Masson syndrome, MRI is the preferred imaging method for evaluating cerebral hemiatrophy and structural bone changes. It is important to diagnose the condition at the earliest, treatment, and optimum control of seizures.
**Limitations:** The main limitation of our study is its retrospective nature and, therefore, insufficient detailed clinical evaluation.
**Ethics Committee Approval:** n/a
**Funding for this study:** n/a
**Author Disclosures:**
Begumhan Baysal: Nothing to disclose
Asuman Orhan Varoğlu: Nothing to disclose
Mahmut Bilal Doğan: Nothing to disclose

### RPS 711-9

**FLAIR Hyperintensity in the subarachnoid space: differentials**
*J. Miranda Bautista*[1], J. A. Guzmán de Villoria Lebiedziejewski[1],
I. Herrera Herrera[1], I. Garrido Morro[1], P. Fernández García[1],
P. Menéndez Fernández-Miranda[2], G. Moreno Zamarro[1], J. Amorim Sortino[1],
P. Elvira Ruiz[1]; [1]Madrid/ES, [2]Cantabria/ES
*(mirandabautistajuan@gmail.com)*

**Purpose:** The main objectives of this review are to illustrate the most frequent causes of CSF hyperintensity in the subarachnoid space in FLAIR sequence, to describe the different mechanisms by which they are produced, and to review the relevance of the rest of the imaging findings to guide the radiologist among the differential diagnoses.
**Methods or Background:** The FLAIR sequence allows suppression of the CSF signal, which increases the detection of pathology that affects the CSF of the subarachnoid space. The hyperintensity of the CSF in this sequence has been described in numerous diseases of various etiologies: tumor (leptomeningeal carcinomatosis, lymphomatosis, glioneuronal tumors and gliomatosis), inflammatory (meningitis), vascular (vascular malformations and Moya-moya disease), CSF hypercellularity (HaNDL headache), or blood (SAH). However, this finding has also been a source of diagnostic errors; for example, in patients treated with supplemental oxygen or other non-pathological processes.
**Results or Findings:** In this educational exhibit, we expose a pictorial review of the main differential diagnosis of FLAIR subarachnoid space hyperintensity based on several patients diagnosed in our institution.
**Conclusion:** FLAIR Hyperintensity of the CSF in the subarachnoid space can be found in diverse entities, including pathological and non-pathological processes, that the radiologist should be aware of. The clinical context, CSF analysis, and other coexisting imaging findings are essential to establish the differential diagnosis.
**Limitations:** There were no limitations to this exhibit.
**Ethics Committee Approval:** Not applicable
**Funding for this study:** Not applicable
**Author Disclosures:**
Pablo Menéndez Fernández-Miranda: Nothing to disclose
Joaquim Amorim Sortino: Nothing to disclose
Isabel Herrera Herrera: Nothing to disclose
Juan Adan Guzmán de Villoria Lebiedziejewski: Nothing to disclose
Juan Miranda Bautista: Nothing to disclose
Irene Garrido Morro: Nothing to disclose
Pilar Fernandez Garcia: Nothing to disclose
Gonzalo Moreno Zamarro: Nothing to disclose
Pascual Elvira Ruiz: Nothing to disclose

### RPS 711-10

**Management of carotid cave aneurysms**
M. Güdük, *A. Arslan*, A. Dinçer; Istanbul/TR
*(arslanaydan@gmail.com)*

**Purpose:** To analyze the follow-up of incidentally diagnosed carotid cave aneurysms, and figure out if extrapolating results from other intracranial aneurysms is reasonable for them.
**Methods or Background:** Saccular intracranial aneurysms can cause severe morbidity and mortality, and with the advent of imaging techniques, incidental diagnosis of them is more common. Even when they are diagnosed incidentally, a treatment is chased either surgical or endovascular. Carotid cave aneurysms are a rare group, and they seem to have a more benign course compared to other intracranial aneurysms, probably related with physical enveloping effect of the surrounding structures. Yet, their surgery is a serious challenge technically for the neurosurgeon, and with its severe morbidity and mortality for the patient. Endovascular techniques also have its own risks, too. The age, and gender of patients, the size, laterality, and MR-angiographic follow-up of aneurysms were reported. Their clinical results were noted.
**Results or Findings:** Fifty-six patients who had incidentally diagnosed 59 carotid cave aneurysms were followed-up. No patient was operated, but 15 patients had endovascular treatment for 15 aneurysms.None of the patients had carotid cave aneurysms related SAH during follow-up, and none of the aneurysms had shown growth. Two patients who had endovascular treatment, had ischemic complications with minor neurologic deficits.
**Conclusion:** Follow-up can be a reasonable option for the incidental aneurysms that are located and confined to the carotid cave.
**Limitations:** Our research had some limitations. First of all, it had a limited number of patients (55 cases) with a single center. It was correlated with DSA in only 15 cases due to its invasiveness. The intradural-extradural distinction with the CISS sequence in aneurysm was demonstrated only in a limited number of patients.
**Ethics Committee Approval:** Yes
**Funding for this study:** Non
**Author Disclosures:**
Mustafa Güdük: nothing to disclose
Aydan Arslan: Nothing to disclose
Alp Dinçer: nothing to disclose

### RPS 711-11

**The BACH (Brain Aging in Congenital Heart Disease) study: the burden of small vessel disease**

*L. Melazzini*[1], P. Vitali[1], F. Savoldi[1], M. Codari[2], A. Giamberti[1], F. Secchi[1], M. Chessa[1], E. Callus[1], F. Sardanelli[1]; [1]Milan/IT, [2]Stanford, CA/US

**Purpose:** Adults with congenital heart disease have recently outnumbered children with heart defects. This large population is at increased risk of developing cerebrovascular disease across the lifespan. The BACH (Brain Aging in Congenital Heart Disease) prospective cohort study aims at assessing the clinical impact of MRI signs of cerebral small vessel disease in adults with conotruncal anomalies.
**Methods or Background:** 23 adult patients with surgically-repaired tetralogy of Fallot and 23 age- and sex-matched healthy controls were recruited at our cardiovascular referral centre. Both groups underwent 3D-T1-weighted, 3D-FLAIR and 2D-T2*-weighted brain MRI scans. White matter hyperintensities (WMHs) masks were obtained using a machine-learning approach. An automated method was applied for sub-classifying WMHs into periventricular and deep. Cerebral microbleeds were manually counted. Patients were also administered a thorough neuropsychological examination.
**Results or Findings:** Total, deep and periventricular WMH volumes did not differ between the study groups (p=0.468). However, a statistically significant association was found between deep WMH volume and patients' performance at the frontal assessment battery (r=-0.650, p=0.012). Cerebral microbleeds were higher in patients than in controls (p<0.001) but were not significantly associated with cognitive performance.
**Conclusion:** Sub-classifying WMHs revealed useful for detecting a specific link to worse cognitive performance. Moreover, we found more cerebral microbleeds in the patients' group. Small vessel disease in patients could derive from a synergistic effect of cyanosis prior to surgical correction, surgically induced brain damage and patients' susceptibility to developing cardiovascular diseases. If confirmed on larger samples, our findings may warn physicians to adopt preventive strategies and promptly act on risk factors of cognitive deterioration in congenital heart disease.
**Limitations:** Preliminary results. Cross-sectional study.
**Ethics Committee Approval:** Yes.
**Funding for this study:** This study was partially supported by Ricerca Corrente funding from the Italian Ministry of Health to IRCCS Policlinico San Donato.
**Author Disclosures:**
Paolo Vitali: Nothing to disclose
Francesco Secchi: Nothing to disclose
Francesco Sardanelli: Nothing to disclose
Edward Callus: Nothing to disclose
Marina Codari: Nothing to disclose
Luca Melazzini: Nothing to disclose
Alessandro Giamberti: Nothing to disclose
Massimo Chessa: Nothing to disclose
Filippo Savoldi: Nothing to disclose

### RPS 711-12

**Sex differences in imaging of patients with multiple sclerosis**

*N. Fileva*, D. Zlatareva, V. Hadjidekov; Sofia/BG
*(nevena.fileva@yahoo.com)*

**Purpose:** Multiple sclerosis is a chronic inflammatory disease affecting young adults with the predominance of the female sex. Many studies show that the disease has a different progression pattern between males and females.
**Methods or Background:** We performed a 3 Tesla MRI exam using a standard head and spine coil in 210 patients diagnosed with MS – 64 males and 146 females (mean age - 41,08, SD = 11.277). We had clinical information including age of initial symptoms, disease duration, EDSS score, and some clinical symptoms for each of them. Using the MRI findings we divided them into groups according to the atrophy stage, the lesion count, and location.
**Results or Findings:** We reached a statistically significant difference in the rate of global atrophy in male patients (U = 3357.0, p=0.001, r=0.23). Our results showed no sex predominance in lesion count. Depending on the specific location the lesions, affecting corpus callosum were found more often in males, rather than in females. The disease disability, measured by the EDSS score, showed higher linear progression again in male patients.
**Conclusion:** There were significant differences in the rate of atrophy and disease progression in males in comparison to females.
**Limitations:** We have no information of the form of the disease in each patients.
**Ethics Committee Approval:** Not applicable
**Funding for this study:** No fundings
**Author Disclosures:**
Dora Zlatareva: Nothing to disclose
Nevena Fileva: Nothing to disclose
Vassil Hadjidekov: Nothing to disclose

### RPS 711-13

**Disentangling cortical function and its spatial topography reveals the substantial genetic influence of connectivity acting through topography in a cohort of healthy young adults**

*B. Burger*[1], K-H. Nenning[1], E. Schwartz[1], D. Margulies[2], A. Goulas[3], H. Liu[4], J. Dauwels[5], D. Prayer[1], G. Langs[1]; [1]Vienna/AT, [2]Paris/FR, [3]Hamburg/DE, [4]Charlestown, MA/US, [5]Singapore/SG
*(bianca.burger@meduniwien.ac.at)*

**Purpose:** We used functional magnetic resonance imaging (fMRI) to study genetic influence on disentangled variability of human functional connectivity strength and its spatial cortical topography in a cohort of young healthy subjects. We evaluated the distribution of genetic influence on these two aspects.
**Methods or Background:** To investigate genetic influence we used the data of 231 participants labeled either as monozygotic or dizygotic twins from the HCP S1200 ICA-FIX denoised dataset. Only twins with 4 resting state fMRI runs available were chosen. Two runs were used for discovery analysis and the remaining for replication. To examine disentangled connectivity strength and topography, we first aligned functional units of each subject to the corresponding units of a reference. This functional alignment allowed us to observe variability in topography and functional connectivity strength of corresponding functional regions independently. Genetic influence was estimated using separate twin models for both features and each unit, after sex and motion were regressed out. We also analysed genetic influence for entangled functional connectivity and compared it to influence on disentangled connectivity strength using a paired t-test.
**Results or Findings:** After disentangling variability of connectivity strength and spatial topography, genetic influence on connectivity strength decreased significantly on average (left hem: corrected p-value < 0.0001; right hem: corrected p-value < 0.0001) and is more homogeneous throughout the cortex ranging from 2.92% to 42%, compared to genetic influence on spatial topography ranging from and 0% to 67%. A replication experiment yielded consistent results.
**Conclusion:** Results suggest that a substantial fraction of genetic influence on functional connectivity architecture shapes variability of its spatial topography.
**Limitations:** Applied twin models estimate only additive effects, not interactions.
**Ethics Committee Approval:** Consent was provided following Wu-Minn HCP Consortium guidelines.
**Funding for this study:** This project was supported by Medical University of Vienna.
**Author Disclosures:**
Karl-Heinz Nenning: Nothing to disclose
Georg Langs: Nothing to disclose
Ernst Schwartz: Nothing to disclose
Hesheng Liu: Nothing to disclose
Justin Dauwels: Nothing to disclose
Daniela Prayer: Nothing to disclose
Daniel Margulies: Nothing to disclose
Alexandros Goulas: Nothing to disclose
Bianca Burger: Nothing to disclose

### RPS 711-14

**Correlation of hippocampal and parietal lobe volumes with visual rating scales in mild cognitive impairment patients**

*I. Soriano Aguadero*[1], A. C. Igual Rouilleault[1], R. Garcia de Eulate[1], P. Dominguez Echavarri[1], M. Vidorreta Díaz de Cerio[2], O. Fernández González[2], M. Riverol Fernández[1], B. Echeveste[1], M. Calvo Imirizaldu[1]; [1]Pamplona/ES, [2]Madrid/ES
*(isoriano@unav.es)*

**Purpose:** To evaluate the correlation between visual rating scales and volumetry with MRI in patients with mild cognitive impairment (MCI).
**Methods or Background:** Ninety-five (95) patients who underwent MRI due to MCI were retrospectively analysed. Two independent radiologists rated atrophy in medial temporal lobe (MTA) and posterior (PA) bilaterally, according to Scheltens' and Koeman's scales, respectively. Afterwards they made a consensus rank for both scales. The hippocampal and parietal lobe volumes were collected with a morphometry function (Syngovia Brain Morphometry, Siemens Healthineers, Erlangen, Germany). A Spearman's rank correlation coefficient was used to compare volume against scales.
**Results or Findings:** Mean volume of right and left hippocampus decreased from 3.15 and 3.25 ml in grade 0 of the Scheltens' scale to 2.89 and 2.85 ml in grade 3, respectively. In parietal lobe, volume changed from 104.77 and 107.81 ml in grade 0 of the Koeman's scale to 88.84 and 97.89 ml in grade 3, for right and left sides. Analysing MTA, correlation was statistically significant in both right (-0.209, p=0.042) and left (-0.230, p=0.025) hippocampal volumes compared to visual assessment (Scheltens' scale). Statistical differences in Spearman's rank correlation were also found when comparing right (-0.220, p=0.032) and left (-0.221, p=0.031) parietal lobe volumes with visual posterior atrophy scale (Koeman's scale).
**Conclusion:** Increasing values in Scheltens' and Koeman's scales have a statistically significant correlation with decreasing hippocampal and parietal lobe volumes. Ongoing long-term follow up in these patients will allow to analyse if either of this techniques can better predict transformation from MCI to Alzheimer´s disease.
**Limitations:** Small sample size.
**Ethics Committee Approval:** Yes.
**Funding for this study:** Not applicable.
**Author Disclosures:**
Pablo Dominguez Echavarri: Nothing to disclose
Beatriz Echeveste: Nothing to disclose
Marta Vidorreta Díaz de Cerio: Employee: Siemens Healthinners
Mario Riverol Fernández: Nothing to disclose
Ignacio Soriano Aguadero: Nothing to disclose
Alba Cristina Igual Rouilleault: Nothing to disclose
Marta Calvo Imirizaldu: Nothing to disclose
Reyes Garcia de Eulate: Nothing to disclose
Oscar Fernández González: Employee: Siemens Healthineers

### RPS 711-15

**Differentiating opportunistic CNS infection from disease infiltration in haematological malignancy**

*J. K. Ruffle*, E. Lim, R. Gnanadurai, H. H-C. H. Lee, R. Miller, H. Hyare; London/UK

**Purpose:** Haematological malignancies are a heterogeneous group of conditions wherein individuals are both at risk of intracranial dissemination and opportunistic infection secondary to treatment-induced immunosuppression. Differentiating CNS infection and disease infiltration in these patients is valuable, yet often difficult as the imaging appearances can be strikingly similar. We sought to characterise the similarity – and evaluate dissimilarity – between these processes.
**Methods or Background:** MR brain studies for all adult patients with haematological malignancy at University College London Hospitals between 2007-17 were retrieved and deep phenotyped for imaging abnormality across numerous anatomical distributions, in addition to the presence of pathological enhancement, diffusion restriction and haemorrhage. Clinical and laboratory were also retrieved.
**Results or Findings:** 109 patients met inclusion criteria (56 male; cohort mean age 48 years). Lymphoma and leukaemia formed the majority of haematological diagnoses, wherein 28 had proven intracranial infection and 15 intracranial metastatic spread. Across infection and disease, the commonest anatomical distributions of abnormality were multifocal parenchymal (34.9%), focal parenchymal (29.4%) and leptomeningeal (11.9%). Pathological enhancement was the most frequently observed abnormality (46.8%), followed by haemorrhage (22.9%) and restricted diffusion (19.3%). Logistic regression could differentiate CNS infection from disease infiltration with an AUC of 0.85 where, with OR >1 favouring infection, and <1 favouring CNS disease infiltration, significantly predictive imaging features were haemorrhage (OR 24.61, p=0.02), enhancement (OR 0.17, p=0.04) and an extra-dural location (OR 0.06, p=0.05).
**Conclusion:** Both CNS infection and disease infiltration have variable appearances and can be difficult to distinguish. We provide preliminary models of key radiological findings that may help in differentiating them.
**Limitations:** Future studies should further develop these preliminary models both across multiple hospital sites and with a larger sample.
**Ethics Committee Approval:** Not applicable
**Funding for this study:** Not applicable
**Author Disclosures:**
Robert Miller: Nothing to disclose
Heather Hoi-Ching Lee: Nothing to disclose
Harpreet Hyare: Nothing to disclose
Roshina Gnanadurai: Nothing to disclose
Emma Lim: Nothing to disclose
James K. Ruffle: Nothing to disclose

### Research Presentation Session: Neuro

### RPS 811

### Developments in neuroradiology

### RPS 811-2

**Exploring the epidemiology of motion artefacts using natural language processing of clinical MRI reports**

*S. Demir*, A. Lio Goncalves Filho, S. Cauley, M. Gee, P. Schaefer, O. Rapalino, S. Yi Huang, J. Conklin; Boston, MA/US
*(sdemir@mgh.harvard.edu)*

**Purpose:** Determine the frequency, distribution and risk factors for motion artifacts in brain MRI based on clinical radiologists' reports.
**Methods or Background:** All brain MRI scans performed in 2019 at a single institution (n=26,173). Presence of motion artifacts in clinical reports were determined using regular expression matching and graded with a pre-defined severity scoring system(mild/moderate/severe/non-diagnostic). Clinically significant motion was defined as "severe" or "non-diagnostic". Two radiologists also visually scored the presence of motion artifacts for a random sample of 100 MRI scans using a validated visual rating system. Logistic regression analysis was used to determine the clinical factors associated with motion (age, study indication, patient location, and MRI protocol).
**Results or Findings:** Prevalence of motion artifacts described in clinical MRI reports was 10.7% (n=2807). Motion was described as severe in 2.9% (n=771) of brain MRI reports, and 0.6% (n=153) described the study as non-diagnostic, thus total clinically significant motion was 3.9%. The report grading showed a strong positive correlation with mean and maximum radiologist visual scores (Spearman's rho = 0.69 and 0.74, respectively). Logistic regression analysis revealed that patient location (ED or inpatient), indication (altered mental status, stroke, seizure, and hydrocephalus), and age (pediatric and elderly patients) were associated with clinically significant motion artifacts.
**Conclusion:** Clinically significant motion artifact is relatively common, with an estimated prevalence of 3.9% based on retrospective review of 26,173 brain MRI reports. Motion is more common in ED/Inpatients as well as pediatric and geriatric patients, therefore future artifact mitigation strategies may benefit from focusing on these patient groups.
**Limitations:** Natural language processing of radiologist reports provides a method for estimating the prevalence of motion artifacts, but likely underestimates mild artifacts not mentioned by the reporting radiologist.
**Ethics Committee Approval:** IRB approved and HIPAA compliant.
**Funding for this study:** No external funding.
**Author Disclosures:**
Stephen Cauley: Nothing to disclose
Pamela Schaefer: Nothing to disclose
Otto Rapalino: Nothing to disclose
Susie Yi Huang: Nothing to disclose
John Conklin: Nothing to disclose
Serdest Demir: Nothing to disclose
Augusto Lio Goncalves Filho: Nothing to disclose
Michael Gee: Nothing to disclose

### RPS 811-3

**The usefulness of subtraction images for accurate diagnosis of pituitary microadenomas in dynamic contrast-enhanced magnetic resonance imaging**

*Z. Ghomi*, M. Sanei Taheri, S. Sabouri; Tehran/IR
*(zgh1881@gmail.com)*

**Purpose:** Dynamic contrast-enhanced Magnetic resonance imaging (MRI) is the modality of choice for the diagnosis of pituitary microadenomas; however, it may be associated with a relatively false-negative rate especially in small lesions. We aim to evaluate the usefulness of subtraction for enhancing visual detection of pituitary microadenomas.
**Methods or Background:** This was a retrospective cross-sectional study. We evaluated patients with clinically established diagnosis prolactinoma, growth hormone (GH) producing adenoma with acromegaly and Cushing's disease. Routine dynamic MRI of the pituitary gland and obtained subtraction MRI images were scrutinized separately on different sessions by two radiologists blinded to study design and patient data. The investigator's opinion on the presence or absence of a lesion and lesion size were collected.
**Results or Findings:** Fifty patients including 40 (80%) female and 10 male (20%) with a mean age of 34.88± 10.95 years were enrolled. Dynamic MRI images were reported positive in 39 patients (78%) and negative in 11 patients (22%). Subtraction images were described as positive in all patients (100%); including all patients with negative dynamic MRI and the difference was statistically significant (p-value: 0.002). Undetected lesions on dynamic MRI had a mean size of 2.95±1.61 mm and a significant inverse correlation was noted between lesion size and negative report of dynamic MRI (p-value: 0.007).
**Conclusion:** Our results confirmed that subtraction images can successfully identify all lesions detectable with conventional dynamic MRI as well as improving visualization of lesions undetected on conventional dynamic MRI; especially in small lesions.
**Limitations:** Due to the rare nature of GH- or ACTH-producing microadenomas, our study population included very few numbers of these lesions.
**Ethics Committee Approval:** The study was approved by the Ethical Committee of Shahid Beheshti University of Medical Science, Tehran, Iran
**Funding for this study:** No funding to declare.
**Author Disclosures:**
Morteza Sanei Taheri: Nothing to disclose
Sofia Sabouri: Nothing to disclose
Zahra Ghomi: Nothing to disclose

### RPS 811-4

**Radiological cervical foraminal grading systems: a systematic review**
*J. Meacock*, M. Schramm, S. Selvanathan, S. Currie, D. Stocken, D. Jayne, S. Thomson; Leeds/UK
*(James.Meacock@nhs.net)*

**Purpose:** A systematic review to evaluate the existing radiological grading systems that are used to assess cervical foraminal stenosis.
**Methods or Background:** The importance of imaging the cervical spine using CT or MRI in evaluating cervical foraminal stenosis is widely accepted however there is no consensus for standardised methodology to assess the compression of the cervical nerve roots. In collaboration with the University of Leeds a search strategy was developed and performed for reports published before 01.02.2020.
**Results or Findings:** 6952 articles were identified with 59 articles satisfying the inclusion criteria. Most reports involved multiple imaging modalities; MRI (69%), CT (50%) and plain X-rays (14%). Standard axial and sagittal imaging were used most with 53% including oblique views. This systematic review showed that the most mature grading for cervical foraminal stenosis is in the form of systems described by Kim et al. 2015 and Park et al. 2013. Area analysis was performed in 48% and global degenerative analysis with a focus on the neuroforamina was included in 41% of reports.
**Conclusion:** Imaging of the cervical nerve root canals is mostly performed using MRI and is reported using subjective terminology. The Park and Kim systems for classifying the degree of stenosis of the nerve root canal have been described. Oblique fine cut images derived from three dimensional MRI datasets may yield more consistency, better clinical correlation, enhanced surgical decision making and outcomes.
**Limitations:** Clinical application of these scoring systems is limited by their reliance on non-standard imaging (Park), limited validation against clinical symptoms and surgical outcome data.
**Ethics Committee Approval:** Local ethical approval was obtained
**Funding for this study:** This study was partly funded by the LGI Neurosurgery Research Fund. Dr. Meacock is supported by a RCS Research Fellowship and an NIHR grant.
**Author Disclosures:**
James Meacock: Nothing to disclose
Moritz Schramm: Nothing to disclose
Senthil Selvanathan: Nothing to disclose
David Jayne: Nothing to disclose
Deborah Stocken: Nothing to disclose
Stuart Currie: Nothing to disclose
Simon Thomson: Nothing to disclose

### RPS 811-5

**The correlation between DIR, FLAIR and T2W imaging sequences with EDSS and determine the diagnostic accuracy of three sequences in the detection of MS lesions at different anatomical regions**
*A. D. Almutairi*, R. Mahmud, S. Suppiah; Selangor/MY
*(alrha123@hotmail.com)*

**Purpose:** The purpose of our study was to evaluate the dependability of the correlation between the lesion load measurement of double inversion recovery (DIR), fluid-attenuated inversion recovery (FLAIR) and T2 weighted imaging (T2WI) sequences with the expanded disability status scale (EDSS) to determine the role of DIR technique as a practical test for clinical disability.
**Methods or Background:** 97 patients were included in our study, imaging performed on a 3T Siemens Skyra MRI scanner using DIR, FLAIR, and T2W_TSE sequences. EDSS was used to investigate the physical disability. The diagnostic accuracy of DIR, FLAIR, and T2WI sequences evaluated in different anatomical regions. Sensitivity and specificity were tested by the receiver operating characteristics (ROC) curve. Spearman correlations were performed to identify the significant relationships between the load measurement and EDSS scores at different regions with the sequences.
**Results or Findings:** Our results demonstrated that the highest correlation coefficients between the number of lesions and EDSS were in the infratentorial region (r= 0.584, p<0.001) for the DIR sequence. It was found that the number of lesions in infratentorial is the best predictor for EDSS. The highest sensitivity (84.6%) and specificity of 81% were observed for the lesions in infratentorial at the cut-off point of 3.5.
**Conclusion:** DIR can describe physical and cognitive dysfunction. Because of the higher potential of the DIR sequence to identify MS lesions and overcome the technical defect of conventional MRI sequences for the diagnosis of cortical lesions, we recommend adding DIR sequence routinely in patients with MS
**Limitations:** None
**Ethics Committee Approval:** The study was approved by the research ethics committee of the Faculty of Medicine and Health Sciences, Universiti Putra Malaysia on 30th January 2018, with a reference approval number UPM/TNCPI/RMC/KEUPM/1.4.18.2 (JKEUPM)
**Funding for this study:** No funding was obtained for this study
**Author Disclosures:**
Othman Alomair: Nothing to disclose
Hasyma Abu Hassan: Nothing to disclose
Subapriya Suppiah: nothing to disclose
Rozi Mahmud: Consultant: non
Abdullah Dhaifallah Almutairi: Speaker: non

### RPS 811-6

**Lesion load assessment among multiple sclerosis patients using DIR, FLAIR and T2WI sequences**
*A. D. Almutairi*[1], R. Mahmud[1], S. Suppiah[1], H. Abu Hassan[1], O. Alomair[2]; [1]Selangor/MY, [2]Riyadh/SA
*(alrha123@hotmail.com)*

**Purpose:** Magnetic resonance imaging (MRI) is a diagnostic imaging technique that is widely used to diagnose lesions in neurological disorders such as multiple sclerosis (MS). The aim of our study was to investigate MS lesion load at different anatomical regions in the brain using double inversion recovery (DIR), fluid-attenuated inversion recovery (FLAIR), and T2- weighted imaging (T2WI) sequences
**Methods or Background:** A total of 97 MS patients were included in our retrospective study. All images were obtained using 3T Scanner (Siemens Skyra). The images from the DIR, FLAIR, and T2WI sequences were compared on axial planes with identical anatomic positions and the number of lesions was assigned to their anatomical region. The contrast ratios were calculated from the mean values of the signal intensity among lesion/normal appearing white matter, lesions/CSF and lesion/ normal appearing grey matter
**Results or Findings:** DIR displayed a significantly higher number of MS lesions in all anatomical regions comparing to FLAIR and T2W_TSE (p < 0.05). Our results indicated that in DIR, the number of lesions in the cortical region was significantly higher than FLAIR and T2WI techniques. The highest contrast ratio observed for DIR in all regions except for lesion/CSF which FLAIR showed the highest ratio
**Conclusion:** DIR is a valuable technique for better delineation, greater contrast measurements, and the increasing total number of MS lesions, as well as more intracortical lesions in MS patients, compared to FLAIR and T2WI. It is strongly recommended to add DIR sequence in daily routine imaging sequences
**Limitations:** Non
**Ethics Committee Approval:** The study was approved by the research ethics committee of the Faculty of Medicine and Health Sciences, Universiti Putra Malaysia on 30th January 2018, with a reference approval number UPM/TNCPI/RMC/KEUPM/1.4.18.2
**Funding for this study:** No funding was obtained for this study
**Author Disclosures:**
Othman Alomair: Nothing to disclose
Hasyma Abu Hassan: Nothing to disclose
Subapriya Suppiah: Nothing to disclose
Rozi Mahmud: Consultant: non
Abdullah Dhaifallah Almutairi: Author: non

### RPS 811-7

**Influence of ageing and gadolinium exposure on T1, T2 and T2*-relaxation in healthy women with and without prior exposure to gadoterate meglumine at 3.0-T brain MR imaging**
*K. B. Krug*[1], C. Burke[1], K. Weiss[2], P. A. Baltzer[3], K. Rhiem[1], D. Maintz[1], M. Schlamann[1], M. Hellmich[1]; [1]Cologne/DE, [2]Hamburg/DE, [3]Vienna/AT
*(Barbara.krug@uk-koeln.de)*

**Purpose:** The effects of aging and intravenous application of gadolinium based contrast agents (GBCA) on MRI-measures in Deep brain nuclei remain poorly understood. We aimed to assess the effects of aging and GBCA-exposure on MRI-measurements in deep brain nuclei.
**Methods or Background:** This prospective, single-center study enrolled 100 healthy participants of our high-risk screening center for hereditary breast cancer, who had received at least six doses of macrocyclic GBCA or were newly entering the program. All participants underwent unenhanced 3.0-T brain MRI including quantitative T1, T2 and R2*-mapping. The relaxation times were derived from ROI measurements in the brain nuclei performed by a radiologist and a neuroradiologist. Statistical analysis was based on descriptive evaluations and multivariate analyses.
**Results or Findings:** The participants (exposed group: 49, controls: 51) were aged 42±9 years. In a multivariable model, age had a comparatively strong impact on R2* ($p<0.001$-0.01), T2 ($p=0.005$-0.048) and T1-relaxation times ($p<0.001$-0.037) for the majority of brain nuclei, mostly affecting the substantia nigra, globus pallidus (GP), nucleus ruber, thalamus and dentate nucleus (DN). The effect of prior GBCA-administration on T1-relaxation times was comparatively less pronounced, yielding statistically significant results in the DN, GP and pons ($p=0.019$-0.033).
**Conclusion:** The effects of aging and gadolinium-exposure on MRI-measurements of deep brain nuclei was observed in a homogeneous group of middle-aged healthy females. Aging had a pronounced effect on T2 and R2*-relaxation more than any effect of former GBCA-applications on measured T1-relaxation times.
**Limitations:** The main limitation of the presented study lies in the interindividual comparison of healthy young women of the same age group. The lack of an intraindividual approach will be addressed in an ongoing study including attenders of the screening program.
**Ethics Committee Approval:** Approval of the institutional ethics review board (file reference 17-240).
**Funding for this study:** None
**Author Disclosures:**
Pascal A.T. Baltzer: Nothing to disclose
Kerstin Rhiem: Nothing to disclose
David Maintz: Nothing to disclose
Christina Burke: Nothing to disclose
Martin Hellmich: nothing to disclose
Kilian Weiss: Nothing to disclose
Kathrin Barbara Krug: Nothing to disclose
Marc Schlamann: Nothing to disclose

### RPS 811-8

**MR prevalence of incidental findings on brain MR: a retrospective analysis of 36,808 MRI scans**

*V. K. Venugopal*, V. Mahajan, H. Mahajan ; Delhi/IN
*(vasanthdrv@gmail.com)*

**Purpose:** To analyze the frequency of benign incidental findings on Brain MR in a multicenter MRI report database on a python based web platform using keyword and substring match.
**Methods or Background:** The study was conducted on retrospective reports of MR scans from six imaging centers between June 2016 to January 2020. These reports were loaded on a python based search platform CARPL data miner. The platform is enabled by MongoDB for handling the huge amount of unstructured data(modality, report, department etc). The platform keeps the recent search results stored in a Redis database, an in-memory database, thus, making the search faster for similar queries. Searches were performed for the frequency of the following incidental findings: Basal ganglia calcifications, empty sella/ partial empty sella, sinusitis, mastoid cell effusion, arachnoid cyst, calcified granuloma, mega cisterna magna, cavum septum pellucidum (CSP), hyperostosis frontalis interna, developmental venous anomaly (DVA), cavum vergae and pineal cyst.
**Results or Findings:** From a total of 128,397 MRI reports done across these six centers, there were 36, 808 MRI Brain studies. The frequency of basal ganglia calcifications, empty sella/ partial empty sella, sinusitis, mastoid cell effusion, arachnoid cyst, calcified granuloma, mega cisterna magna, CSP, hyperostosis frontalis interna, DVA, cavum vergae, pineal cyst were: 28111 (22 %), 2527 (2 %), 1727 (1 %), 1197 (1 %), 857 (1%), 596 (0.5%), 368 (0.3%), 310 (0.2%), 189 (0.1%), 172 (0.1%), 161 (0.1%), 96 (0.1%).
**Conclusion:** We report MR prevalence of incidental findings on brain MR. Python-based web data-miner is helpful in identifying these incidental findings in large retrospective report datasets.
**Limitations:** None
**Ethics Committee Approval:** Not applicable
**Funding for this study:** Not applicable
**Author Disclosures:**
Harsh Mahajan : Other: Research collaboration, Predible Health Other: Research collaboration, Lunit INC Other: Research collaboration, Qure.ai Other: Research collaboration, General Electric Company Other: Research collaboration, Lunit Inc. Other: Director, Mahajan Imaging Pvt Ltd Other: Research collaboration, Koninklijke Philips NV
Vasantha Kumar Venugopal: Other: Research collaboration, Koninklijke Philips NV Other: Research collaboration, General Electric Company Other: Research collaboration, Predible Health Other: Research collaboration, Qure.ai Other: Research collaboration, Lunit Inc.
Vidur Mahajan: Other: Head of Research at CARING

### RPS 811-9

**Magnetic resonance neurography in the evaluation of peripheral neuropathy and plexopathy**

*P. Lenin*, M. Z. Mohammad Zakir, A. Kale, Y. J. Kirubha, K. Sudhakar, N. Chidambaranathan; Chennai/IN
*(pradhaplenin@gmail.com)*

**Purpose:** To evaluate the role of MR Neurography (MRN) in plexopathy, peripheral neuropathy and to localize the abnormality with its extent and to characterize the aetiology. In addition to evaluate the role of MRN in grading the nerve injuries into Neuropraxia, Axonotmesis and Neurotmesis.
**Methods or Background:** 125 patients with different clinical suspicions referred to our department for MRN. The study included both traumatic and non-traumatic cases. The study was done on 3 Tesla MRI machine with the standard departmental protocol using T1W, T2W, PDW-SPAIR, STIR, DWIBS, 3D-Nerve view sequences, and post-contrast T1W sequence if required. The calibre, signal intensity, course, fascicular pattern, and enhancement pattern of the nerve analysed. Then the data was collected, processed, analysed and results were tabulated. We correlated the results of clinical examination, nerve conduction study, histopathology (in case of nerve sheath tumours) and intra-operative findings (in case of nerve injuries) with MRN diagnosis.
**Results or Findings:** Of the 125 patients, 40 had neurogenic tumour, 2 had median nerve fibrolipomatous hamartoma, 5 had acute brachial neuritis, 8 had radiation plexitis, 21 had post-traumatic brachial plexopathy, 25 had post-traumatic neuropathic changes in peripheral nerves, 3 patient had infective neuritis, 10 patients had malignant brachial plexopathy, 2 patients had CIDP as evidenced by imaging findings of MR Neurography. 6 patients had no abnormality in MRN. Follow up histopathology report correlated well with MRN findings
**Conclusion:** MRN is a non-invasive, reliable, and novel imaging technique giving detailed anatomical information to diagnose plexopathy and peripheral neuropathy. Nerve injuries are graded with higher reliability in MRN which helps the surgeon in planning the course of treatment effectively.
**Limitations:** The presence of haemorrhage and excess oedema limits the grading the nerve injury.
**Ethics Committee Approval:** Not applicable
**Funding for this study:** Not applicable
**Author Disclosures:**
Alok Kale: Nothing to disclose
Natesan Chidambaranathan: Nothing to disclose
Yvette Kirubha Jayakar David Livingstone: Nothing to disclose
K Sudhakar: Nothing to disclose
Mohammad Zuber Mohammad Zakir: Nothing to disclose
Pradhap Lenin: Nothing to disclose

### RPS 811-10

**Evaluation of brain morphometry DTI and perfusion MRI in patients with mild cognitive impairment**

E. Wong Pak Yew, *K. Rahmat*, N. F. Muhammad, T. Li Kuo, N. Ramli, T. Maw Pin; Kuala Lumpur/MY
*(kartini@ummc.edu.my)*

**Purpose:** To determine the differences in brain morphometry, cerebral white matter lesion, diffusion tensor imaging (DTI) indices and arterial spin labelled (ASL) perfusion between subjects with mild cognitive impairment (MCI) and cognitively normal control (CNC).
**Methods or Background:** A total of 28 subjects underwent MRI neurodegenerative protocol. Brain morphometry was processed using Freesurfer to obtain surface area, subcortical brain volume and cortical thickness. A template-based ROI was used to obtain fractional anisotropy (FA), mean diffusivity (MD), axial diffusivity (AD) and radial diffusivity (RD) values. Pulsed Arterial spin labelling (PASL) was evaluated using FSL software suite. White matter lesion burdens were evaluated visually by Fazekas score and quantitatively by automated lesion segmentation tool method.
**Results or Findings:** MCI group showed significant volume loss in both hippocampi, both amygdala and corpus callosum ($p<0.05$) compared to CNC. Reduced cortical thickness in both parahippocampal, both entorhinal, right posterior cingulate and right temporal lobe in MCI group. MCI group had significantly lower FA, higher MD, AD and RD values in white matter tracts predominantly in the hippocampus and cingulate gyrus when compared to CNC ($p<0.05$). DTI parameters particularly FA, MD and RD were significantly correlated with MoCA and VCAT scores. White matter lesion volume via quantitative assessment were significantly higher in MCI group.

**Conclusion:** Morphometric analysis, DTI and white matter lesion assessment are useful for evaluation of MCI patients and aid in their early diagnosis.
**Limitations:** Data collection and analysis in progress.
**Ethics Committee Approval:** Medical ethics was approved by the UMMC Medical Research Ethics Committee in July 2018 (MREC ID NO 201832-6071).
**Funding for this study:** The funding of the study was provided by the University of Malaya Research Grant (Grant Code: RP052B-17HTM)
**Author Disclosures:**
Tan Maw Pin: Nothing to disclose
Norlisah Ramli: Nothing to disclose
Tan Li Kuo: Nothing to disclose
Kartini Rahmat: Nothing to disclose
Eustace Wong Pak Yew: Nothing to disclose
Nadia Fareeda Muhammad: Nothing to disclose

### RPS 811-12

**Quantitative DTI measurements in non-compressive and compressive cervicodorsal myelopathy with the degree of compression correlation**
*H. G. E. Hassan*, R. Ali Maarouf, N. Ramzy Mohamady,
M. A. Farouk Abo-Elela; Cairo/EG
*(doctor_hendgalal@yahoo.com)*

**Purpose:** DTI is a specific and sensitive method in evaluation of spinal cord trauma abnormalities in regions that appeared normal on conventional MRI, assessment of the functional integrity of the axons of the compressed spinal cord can improve prognostic prediction.
**Methods or Background:** thirty patients (15 female and 15 male patients their ages ranged from 16 to 65 years) presented with clinical symptoms of spinal cord compression (unilateral or bilateral numbness of upper limbs or back pain). Diffusion tensor sequence was added to conventional MRI study. FA and ADC were measured in all cases in three sites (at, above, and below the site of lesion); the last two measures are considered as control
**Results or Findings:** Statistically significant difference of (FA) and (ADC) between compressive and non-compressive lesions and control with p value < 0.05, resultant cut-off value of FA = 0.385 with sensitivity 71%, specificity 100%, and cutoff value of ADC = 1.35 with sensitivity 61.3 %, specificity 88.3%. There is positive correlation between degrees of cord compression and FA value which have clinical impact on specification of clinical symptoms.
**Conclusion:** The more increase of FA and decrease of ADC values implies a better prognosis in acute lesions and clinical recovery.There is positive correlation between degrees of cord compression and FA value which have clinical impact on specification of clinical symptoms.
**Limitations:** further study is advised with specific pathological category with additional post-operative clinical data and follow-up of the DTI results correlation could add relevant value.
**Ethics Committee Approval:** This study was approved by the Research Ethics Committee of the Faculty of Medicine at Ain Shams University in Egypt in 2018 and extended to 2020; Reference Number of approval: MD 76/2018.
**Funding for this study:** This study had no funding from any resource.
**Author Disclosures:**
Hend Galal Eldeen Hassan: Nothing to disclose
Mohamed AFarouk Abo-Elela: Nothing to disclose
Rania Ali Maarouf: Nothing to disclose
Norhan Ramzy Mohamady: Nothing to disclose

### RPS 811-13

**A 6-year review of imaging studies used to investigate patients with papilloedema**
*A. A. Ahmmed*[1], J. Olson[2]; [1]Bristol/UK, [2]Aberdeen/UK
*(afsara.a.ahmmed@gmail.com)*

**Purpose:** To examine what imaging studies patients with suspected papilloedema that presented to a tertiary emergency eye service received and if this complies with national consensus guidelines.
**Methods or Background:** The Association of British Neurologists consensus guidelines in 2018 for the diagnosis and management of idiopathic intracranial hypertension state all patients found to have papilloedema should have CT/MRI head and venography to rule out a space occupying lesion and venous sinus thrombosis. A database of clinical records from all attendances to the emergency eye service was searched to identify all episodes where papilloedema was the listed diagnosis from February 2013 to June 2019. Electronic patient records were used to determine what imaging they had and what it showed retrospectively. Those with no report from their imaging studies were excluded. If a patient was seen multiple times, the imaging relating to their first visit with a diagnosis of papilloedema was noted.
**Results or Findings:** There were 179 entries with papilloedema as the diagnosis. Imaging reports were not available for 29 entries and there were 19 duplicate entries, giving 131 cases. Ninety-six patients had a CT or MRI head (73%) and 64 patients (49%) had a CT or MR venogram.
**Conclusion:** Imaging was not performed as per national guidelines. Concordance with the guideline could be improved through teaching sessions to referring clinicians as well as educational posters within the radiology department. A reaudit could take place in six months.
**Limitations:** The study was conducted retrospectively, which resulted in 29 cases being excluded due to inaccessible imaging reports. All cases of suspected papilloedema may have not been identified using the filtering method used.
**Ethics Committee Approval:** This study was a service improvement project, hence no ethics committee approval was required.
**Funding for this study:** Nil
**Author Disclosures:**
Afsara Anisa Ahmmed: Nothing to disclose
John Olson: Nothing to disclose

### RPS 811-14

**Ultrasound-guided motor unit scanning EMG (UltraMUSE)**
*S. Maitland*, J. Hall, R. Whittaker; Newcastle/UK
*(stu.maitland@newcastle.ac.uk)*

**Purpose:** Needle EMG is a neurophysiological procedure performed ‘blind’ with no sense of the position of the needle within the motor unit. As a result, no information on motor unit dimensions or internal structure is available. We have developed a simple to use technique combining EMG and ultrasound to perform targeted motor unit transects in human muscles.
**Methods or Background:** Ultrasound guidance was achieved using a Philips EPIQ with eL18-4 linear transducer, frequency 2-20MHz. The ultrasound recordings were captured using a video capture card and synchronised to EMG recording using the audio-in channel. We stimulated the peroneal nerve until alternation was visible in a single motor unit in the tibialis anterior muscle, and targeted this with a 28g concentric needle electrode. By placing the needle through to the far side of the twitch and withdrawing in small increments by hand, around 20 motor unit potentials were recorded at each position. Needle position was determined post-hoc to allow a 2D motor unit scan to be reconstituted.
**Results or Findings:** Here, we present the first recordings of this new technique. We demonstrate that the movement resolution of the human operator is around 1-2mm, which is sufficient to allow a transect of a motor unit. We establish the motor unit potential at each location, and how this evolves as the needle position moves. The corridor length of each motor unit transect is around 6mm.
**Conclusion:** This quick and easy technique holds the potential to allow rigorous sampling of motor unit activity, reducing the inter-operator variability of needle EMG and improving the sensitivity to conditions including Motor Neurone Disease.
**Limitations:** Further work is ongoing modelling the diagnostic accuracy of this technique.
**Ethics Committee Approval:** Newcastle Medical School Ethics Committee approval received.
**Funding for this study:** NIHR Newcastle BRC
**Author Disclosures:**
Stuart Maitland: Nothing to disclose
Julie Hall: Nothing to disclose
Roger Whittaker: Nothing to disclose

### RPS 811-15

**Looking outside the box: a review of incidental findings outside lumbar spinal canal causing back pain on routinely performed MRI lumbar spine**
*R. Kanwal*, M. Rauf, B. Y. Faiz, S. Gul, A. Rana; Islamabad/PK
*(raanakanwal@hotmail.com)*

**Purpose:** 1. To document the prevalence of findings additional to lumbar spinal canal pathologies on routinely performed MRI lumbar spine. 2. To educate radiologists to search for causes of back pain other than discogenic disease by reviewing all the visible structures acquired in MRI lumbar spine.
**Methods or Background:** Lower backache is one of the well-recognized indications for acquiring MRI lumbar spine with reported prevalence to be 60-70 percent. Apart from expected spinal pathologies, radiological imaging helps to recognize the incidental findings which are unexpected yet can explain patient's symptoms. We reviewed these pathologies on MRI spine and determined their prevalence which can have an impact on clinical management.
**Results or Findings:** We reviewed 350 MRI lumbar spine of patients with lower backache performed from January till June 2020. Among recognized incidental findings classification was made into vascular, oncological, infectious, congenital and others. Among the included patients 117 were females; while 233 were males. Out of total 350 patients 80 had extra-spinal incidental findings with most commonly seen finding being renal cyst seen in 33 (41.2%) patients. Others had simple ovarian cyst 13 (16.2%), complex ovarian cyst 5 (6.2%), ovarian mass 2 (2.5%), vertebral metastasis 9 (11.2%), prostatic carcinoma 2 (2.5%), cervical carcinoma 1 (1.2 %), rectal carcinoma 1 (1.2%), vertebral body fracture 2 (2.5%), retroperitoneal fibromatosis 1 (1.2%), para-aortic lymphadenopathy 3 (3.7%), cholelithiasis 1 (1.2%), congenital anomaly 3 (3.7%) and hydronephroureter due to urolithiasis 4 (5%).
**Conclusion:** Our study points out uncommonly reported incidental findings that can explain patient's symptoms. It provides a road map towards spinal and non-spinal structures assessment in MRI lumbar spine.
**Limitations:** No
**Ethics Committee Approval:** Approved
**Funding for this study:** Not needed.
**Author Disclosures:**
Belqees Yawar Faiz: Nothing to disclose
Atif Rana: Nothing to disclose
Raana Kanwal: Nothing to disclose
Salma Gul: Nothing to disclose
Maria Rauf: Nothing to disclose

### Research Presentation Session: Paediatric

### RPS 312

### CT and MRI in paediatrics

### RPS 312-2

**Haematopoietic stem cell transplantation (HSCT) in children: the basics and how to identify the main abdominal complications**

*C. Ponich Clementino*, L. Suzuki, D. Otto, M. Lebouche, R. Correia, M. Rocha; São Paulo/BR
*(caponich@gmail.com)*

**Purpose:** To review the most common indications for HSCT in the pediatric population, as well as revise briefly the central principles and different types of transplantation. To discuss the most common abdominal complications, clinical manifestations, imaging findings, and relation to the three posttransplantation phases. To propose a simple diagram that aids radiologists to suggest the most accurate diagnosis in this scenario.
**Methods or Background:** HSCT is currently an option of treatment for many benign and malignant hematologic disorders, as well as some solid tumours. In children, some of the main indications include leukemia, lymphoma, neuroblastoma, and Wilms tumour. It consists of extracting stem cells from another individual (allogeneic transplantation) or from the patient himself (autologous transplantation) and injecting them into the host. Previously, the patient has to undergo a conditioning therapy, in which virtually all of the patient's hematopoietic cells will be cleared from the organism. The patient becomes more susceptible to contract various bacterial, viral and fungal infections, as well as other conditions that affect the liver, gastrointestinal tract, urinary tract and the lymphatic system.
**Results or Findings:** Complications following HSCT include acute and chronic graft-versus-host disease (GVHD), infectious enterocolitis, neutropenic colitis, thrombotic microangiopathy, benign pneumatosis, sinusoidal obstruction syndrome, hemorrhagic cystitis, and posttransplant lymphoproliferative disorder (PTLD). Imaging findings in ultrasound and computed tomography, such as bowel wall thickening and mesenteric fat stranding, are common in many of these conditions and often mislead to an equivocal diagnosis.
**Conclusion:** Abdominal complications following HSCT may present a diagnostic challenge for radiologists due to overlapping clinical symptoms and imaging findings. It is important for radiologists to know what clinical data and key imaging findings will help them narrow down the diagnosis and allow an adequate treatment.
**Limitations:** No limitations
**Ethics Committee Approval:** Not required
**Funding for this study:** Non
**Author Disclosures:**
Carolina Ponich Clementino: Nothing to disclose
Melody Lebouche: Nothing to disclose
Raphael Correia: Nothing to disclose
Deborah Otto: Nothing to disclose
Marina Rocha: Nothing to disclose
Lisa Suzuki: Nothing to disclose

### RPS 312-3

**Normal pancreatic thickness values in healthy children: MRI values**

E. Fatihoğlu, *S. Aydın*, E. Karavaş; Erzincan/TR
*(sonaydin89@hotmail.com)*

**Purpose:** Normal pancreatic thickness values on ultrasound (US) have been defined in literature. However, there is insufficient information about normal pancreatic measurements acquired from computed tomography (CT) or magnetic resonance imaging (MRI). We aim to define normal pancreatic thickness measurements acquired from different localizations.
**Methods or Background:** A retrospective evaluation was made of the abdominal MRI examinations of 105 pediatric patients. Patients with any pancreatic disease, or chronic gastrointestinal inflammatory disease were excluded from the study. Measurements were taken from T2-weighted images
**Results or Findings:** The study population comprised 51 (48.6%) males and 54 (51.4%) females with a median age of 11 years. The median normal pancreatic thickness of head, neck, body and tail portions were measured as 18 mm, 10 mm, 14 mm, and 14 mm, respectively. N. A positive correlation was determined between age, height, weight, body mass index (BMI) and pancreatic thickness in all the anatomic localizations measured.
**Conclusion:** The defined normal ranges were found to be mostly consistent with previously determined US and CT-based values. Pancreatic thickness was determined to be positively correlated with age, height, weight, and BMI for all four localizations. Knowledge of the normal pancreatic thickness values will increase the diagnostic accuracy of radiologists for pancreatic diseases.
**Limitations:** A larger population would enable more definitive normal ranges. Patients younger than 4 years of age were not included in this study. The inclusion of more centers in the study would have increased the reproducibility and reliability of the data. Moreover, the interobserver variability data were of only two researchers. Data were collected using only T2-weighted images.
**Ethics Committee Approval:** Approval for this retrospective study was granted by the Institutional Review Board. Informed consent was waived because of the retrospective nature.
**Funding for this study:** None
**Author Disclosures:**
Erdal Karavaş: Nothing to disclose
Sonay Aydın: Nothing to disclose
Erdem Fatihoğlu: Nothing to disclose

### RPS 312-4

**Power injector usage for paediatric CT angiography: a single centre experience**

*M. Pop*, C. Patricia Carmen, D. C. Cucoranu, I. P. Simu; Tirgu Mures/RO
*(pop.marian@gmail.com)*

**Purpose:** To evaluate the safety of power injector usage for pediatric CT angiography when using central versus peripheral access, by assessing the incidence of adverse events.
**Methods or Background:** We retrospectively evaluated data of 399 pediatric CT angiography examinations performed 2015-2020 in a tertiary cardiovascular center. All examinations were done using a pressure-limited power injector (300 psi) and room-temperature contrast medium. Recorded data included patient age and weight, cannulation type, site of venous access, type and volume of contrast agents, flow rate and adverse events (e.g. extravasation, allergic reactions).
**Results or Findings:** The median patients age was 349 days, with a male: female ratio of 1.27. The most frequently site for peripheral cannulation was the left antecubital fossa (159 cases, 39.8%) and the most frequently used canula size was 24 gauge(143 cases). A central line was used in 74(18.5%) cases. The average flowrate was significantly higher (2.03 vs 0.99 ml/s) when using a peripheral canula. Using peripheral access we have encountered three cases of extravasation (0.75 %), 8 minor allergic reactions and one anaphylactic shock. All cases resolved favorably. Using a central line was not associated with incidents due to contrast medium injection; however, in two cases we found the central line to be placed in an artery.
**Conclusion:** The usage of power injectors in CT angiography is generally safe when using either peripheral or central venous access. Even with a rigorous systematic approach and a properly trained team the presence of incidents is not zero.
**Limitations:** Small sample size and single center experience. Lack of data regarding the central line type (single/multi lumen, diameter, length). 45.7% of patients were infants, unable to express complaints.
**Ethics Committee Approval:** IUBCvT 4920/2020
**Funding for this study:** n/a
**Author Disclosures:**
Chiscariu Patricia Carmen: Nothing to disclose
Iunius Paul Simu: Nothing to disclose
Dragos Constantin Cucoranu: Nothing to disclose
Marian Pop: Nothing to disclose

### RPS 312-5

**Silent anatomic deformities of the paediatric chest: more often than we think**

*D. Bako*[1], O. Yapici Ugurlar[2]; [1]Van/TR, [2]Istanbul/TR
*(deryabakokeskin@gmail.com)*

**Purpose:** To evaluate the frequency of silent anatomic malformations and variations of the pediatric chest, which were not even recognized by the physicians.
**Methods or Background:** Thorax CT scans of 592 children were retrospectively reviewed. 82 children who had undergone chest wall surgery or suspected of having chest wall deformity, chest wall mass, scoliosis, chest wall or clavicular fracture were excluded. The frequency of silent chest wall deformities evaluated and compared with age subgroups and sex.
**Results or Findings:** 118 of 510 patients had silent anatomic deformity (malformation or variation) of chest wall (23.1%). These deformities or variations were depicted in 50 of 204 girls and 68 of 306 boys. The most common deformity was the prominent convexity of the costal cartilage (n=36, 30.5%). While there was no statistically significant difference between sex and the frequency of deformities; significant difference ($p < 0.001$) existed in frequency between age subgroups.
**Conclusion:** We detected that the frequency of silent anatomic variation / malformations in child population is about 23%. It means that approximately every fourth child has some silent deformity of the chest wall. A high rate depicts that asymptomatic asymmetries-lesions of the chest wall are mostly benign and there is no need for further imaging. The other important finding is an increase in the frequency of malformation / variations with age, which supports the hypothesis that these subtle malformations/variations are mostly developmental, not congenital and becoming more prominent with age and puberty. The other conclusion and next hypothesis might be that these silent malformations/variations are expected to be even more frequent in adult population.
**Limitations:** It was based on data from a single center and sample size is relative small.
**Ethics Committee Approval:** Available (Van Regional Training and Research Hospital)
**Funding for this study:** Van Regional Training and Research Hospital
**Author Disclosures:**
Ozge Yapici Ugurlar: Nothing to disclose
Derya Bako: Nothing to disclose

### RPS 312-7

**Let's play: using games to perform fMRI in 3-5 year-old children**

*M. T. Podgórski*, M. Podgórska, J. Błasiak, N. Smiech, K. B. Biernacka, T. Puzio, P. Grzelak; Lodz/PL
*(podgorskimt@gmail.com)*

**Purpose:** The main aim of the study was to prepare the interactive application than can be used as a motor paradigm in fMRI study. Secondary goal was to decrease the age of paediatric patients than can undergo the fMRI study when compared with the standard fMRI protocol.
**Methods or Background:** The application was prepared according to authors' scenario. Child has to squeeze the rubber figure powering up the car waiting at the start of the track. The figure is connected through the silicon tube with the receiver and the pace of squeezing corresponds with energy accumulated in car (active block lasting 30 sec.). During next 30 sec. child observes how the car moves through the track (passive block). The schema with different tracks of increasing difficulty is repeated 5 times. Thirty children aged 3-5 years were examined according to the standard and interactive motor paradigms (5 girls and 5 boys in each year of life).
**Results or Findings:** None of ten of the 3-years old children were able to fulfill the examination. In 4-years old one out of ten was able to undergo the standard paradigm, while fife the interactive one. In a group of 5-years old children three undergo the standard paradigm and seven the interactive one. On the visual inspection examination with the interactive paradigm resulted with less artifacts.
**Conclusion:** Application of a game as a motor paradigm can decrease the age of children able to undergo the fMRI study and improve examination quality.
**Limitations:** Only healthy children were examined. Children with neurological problems will be tested next to confirm usefulness of the application.
**Ethics Committee Approval:** Local bio-ethical committee agreement no. 109/2015
**Funding for this study:** The study was funded by Narodowe Centrum Nauki as a scientific grant SONATA (gran no: 2016/21/D/NZ4/03721)
**Author Disclosures:**
Nina Smiech: Nothing to disclose
Tomasz Puzio: Nothing to disclose
Michał Tomasz Podgórski: Nothing to disclose
Monika Podgórska: Nothing to disclose
Piotr Grzelak: Nothing to disclose
Joanna Błasiak: Nothing to disclose
Katarzyna Barbara Biernacka: Nothing to disclose

### Research Presentation Session: Paediatric

### RPS 412

### Ultrasound in paediatrics

### RPS 412-1

**Accordance of histopathology with imaging diagnosis of congenital pulmonary lesions using prenatal ultrasound, prenatal MRI and postnatal CT: a single centre experience**

*D. Kifjak*, F. Prayer, H. Prosch, M. Weber, G. Gruber, A. Scharrer, A. Hojreh, D. Prayer, G. Kasprian; Vienna/AT
*(daria.kifjak@meduniwien.ac.at)*

**Purpose:** Evaluation of diagnostic accuracy of prenatal ultrasound, prenatal MRI and postnatal CT in congenital pulmonary malformations. Histopathology after lung lesion resection served as gold standard.
**Methods or Background:** In this retrospective, single-center study, 32 fetuses with histopathologically verified congenital pulmonary lesions evaluated by prenatal ultrasound, prenatal MRI and postnatal CT during the first year of life were included. Prenatal ultrasound and prenatal MRI were performed at an average gestational age of 22+3 gestations weeks (GW) (range 12+16 to 32+5 GW) and 27+2 GW (21+5 to 38+3 GW), respectively. Routine fetal MRI was performed according to ISUOG guidelines. Afterwards, diagnosis based on prenatal ultrasound, prenatal MRI and postnatal CT was compared to histopathological findings.
**Results or Findings:** Histologically proven congenital lung lesions included 14 cases of congenital pulmonary airway malformation (CPAM), seven cases of hybrid lesions, six cases of bronchopulmonary sequestration, three cases of bronchial atresia and two cases of bronchogenic cysts. Prenatal sonography findings agreed with histopathological results in twelve of 32 (38%) cases. In 23 of 32 (72%) cases fetal MRI diagnosis was consistent with the histopathological results. Histopathological diagnosis confirmed CT findings in 26 of 32 (81%) cases.
**Conclusion:** In our study we succeeded showing that fetal MRI is an excellent tool for the diagnosis of congenital pulmonary lung lesions, thereby complimenting prenatal ultrasound screening. Moreover, fetal MRI facilitates personalized perinatal management of cases with congenital pulmonary lung lesions.
**Limitations:** Different gestational age (gestational weeks) during prenatal MRI and prenatal ultrasound.
**Ethics Committee Approval:** Approved by local ethics committee (EK# 1343/2020).
**Funding for this study:** No funding.
**Author Disclosures:**
Helmut Prosch: Nothing to disclose
Florian Prayer: Nothing to disclose
Anke Scharrer: Nothing to disclose
Daria Kifjak: Nothing to disclose
Azadeh Hojreh: Nothing to disclose
Gerlinde Gruber: Nothing to disclose
Daniela Prayer: Nothing to disclose
Gregor Kasprian: Nothing to disclose
Michael Weber: Nothing to disclose

### RPS 412-4

**Pseudo-testicular sign: a new ultrasonographic feature of the ovaries in female neonates with ambiguous genitalia due to congenital adrenal hyperplasia**

*S. Dehghani Dashtabi*, S. A. Alamdaran, A. Vahabi, S. Hashemian, Y. Nikooiyan; Mashhad/IR
*(soroosh.dehghani@gmail.com)*

**Purpose:** A common cause of ambiguous genitalia is congenital adrenal hyperplasia (CAH). The imaging features of the uterus and ovaries in infants with CAH have not been thoroughly investigated. We aimed to investigate the gynecologic findings in ultrasound exams of these patients.

**Methods or Background:** This case series study was performed in the radiology and endocrine departments of Akbar Children's Hospital, Mashhad, Iran, during 2016-2018. The diagnosis of CAH was confirmed using medical history, physical examination, laboratory tests, and ultrasound exams (indicated by the enlarged cerebriform adrenal gland in ultrasound imaging). Ultrasound variables including uterus size, uterus-to-cervix ratio, echogenicity and size of the ovary, and presence of follicles and/or cysts in the ovaries were collected for all patients. After a two-month course of treatment, a second ultrasound was performed to evaluate the response to treatment. Data were analyzed in SPSS version 22 for Windows.
**Results or Findings:** Overall, 18 female patients with a mean age of 16.83±5.44 days were studied. The adrenal enlargement and cerebriform appearance were reported in all patients. We studied the ovarian follicular appearance and found that 14 patients (78%) had no follicles in either of the ovaries, showing the pseudo-testicular sign. In the second ultrasound, all patients had bilateral multi-follicular ovaries, and cerebriform adrenal enlargements were resolved in all patients except one (8%).
**Conclusion:** The pseudo-testicular appearance of ovaries is an auxiliary sign in female infants with CAH, obviating the need for other laboratory tests and karyotyping and preventing misdiagnosis and loss of time.
**Limitations:** The small number of our cases was our main limitation.
**Ethics Committee Approval:** The study was approved by the Ethics Committee, Mashhad University of Medical Sciences (MUMS) (approval code: IR.MUMS.MEDICAL.REC.1398.803).
**Funding for this study:** This study was funded by the Vice-Chancellor for Research, MUMS (grant number 981056).
**Author Disclosures:**
Somayeh Somayeh Hashemian: Nothing to disclose
Yasaman Nikooiyan: Nothing to disclose
Armin Vahabi: Nothing to disclose
Soroosh Dehghani Dashtabi: Nothing to disclose
Ali Seyed Ali Alamdaran: Nothing to disclose

### RPS 412-5

**Shearwave elastography of healthy paediatric testicular tissue and correlation with age**
*I. Sarı Karabağ*, M. Ceyhan Bilgici; Samsun/TR
*(iremsari@yahoo.com)*

**Purpose:** Ultrasound elastography is a newly developed technique that is used for assessment of tissue elasticity in a fast and noninvasive way. Recently, numerous studies proved its capability in differentiating between normal and diseased tissue. The aim of this study is to determine the testicular stiffness change throughout childhood. Besides, correlation with age, we wanted to manifest baseline standard values for pediatric testicular elasticity.
**Methods or Background:** A total of 91 pediatric patients (mean age; 7,49±5,06 years, range; 1 month- 17 years) with no known systemic or testicular disease were enrolled in the study. First, B mode ultrasound had been done and testicular parenchyma was evaluated. During B mode ultrasound, patients with incidental testicular parenchymal lesions were discarded from the study. Then, testicular volume calculations were done for healthy testicular tissue. Lastly, testicular elasticity was measured for each patient with Siemens Acuson S2000 (Mountain View, CA, USA).
**Results or Findings:** Statistically, there was a strict correlation of testicular volume with age p<0,001). There was no significant correlation of tissue elasticity within different age groups. The mean shear-wave velocities in ≤5, 6-10, 11-15 and > 16 years were 0,728 ± 0,063, 0,714 ± 0,048, 0,723 ± 0,067, 0,702 ± 0,036 m/s, respectively.
**Conclusion:** Shear wave elastography demonstrated pediatric testicular elasticity doesn't have correlation with age. However, with the basal pediatric testicular elasticity values, this study will be beneficial for future pediatric testicular elasticity researches including focal scrotal masses and diffuse testicular diseases.
**Limitations:** No limitations.
**Ethics Committee Approval:** Approved
**Funding for this study:** No fund has been received.
**Author Disclosures:**
Meltem Ceyhan Bilgici: Nothing to disclose
Irem Sarı Karabağ: Nothing to disclose

### Research Presentation Session: Physics in Medical Imaging

### RPS 313

### Physics in medical imaging developments

### RPS 313-1

**Robustness and reproducibility of radiomics in virtual monoenergetic images of dual-energy CT**
*A. Euler*, D. Cester, M. Eberhard, T. D. J. Sartoretti, N. Lohaus, H. Alkadhi, B. Baessler; Zurich/CH

**Purpose:** To evaluate the robustness and reproducibility of radiomic features from Virtual Monoenergetic Images (VMIs) of different energy levels and to assess their impact on machine-learning-based classification as a function of radiation dose and dual-energy CT (DECT) approach for dual-source (DS-DE) and split-filter DECT (SF-DE).
**Methods or Background:** Our phantom consisted of 4 kiwi fruits, 4 onions, 4 apples, and 4 oranges, chosen to reflect different HU, shapes, and tissue textures. It was imaged in dual-energy mode on a DS-DE and SF-DE at two radiation doses of 5 and 15mGy. To assess test-re-test robustness, all scan setups were repeated after repositioning the phantom. For each setup, VMIs at 40,50,75,120,190keV were reconstructed. Datasets were pre-processed and a total of 1218 radiomic features were extracted (pyRadiomics; 3D-Slicer). The robustness of each feature was tested by calculating the concordance-correlation-coefficients (CCCs). A Random Forest classifier was repeatedly trained with different data splitting and feature subsets, to assess a) the impact of the setup parameters and b) the relative importance of the features and its stability.
**Results or Findings:** Test re-test robustness was relatively homogeneous across the settings with 80% robust features. SF-DE showed a larger variation of the number of robust features compared to DS-DE. Reproducibility of features differed substantially among VMI levels (as low as 32.8%) and depended on DECT. In regards to the classification task, a subset of 20 features achieved high performance for every parameter combination (accuracy and F-Score higher than 0.99).
**Conclusion:** Robustness of radiomic features was high within each VMI while comparisons among different VMI levels and DECT scanners decreased robustness. Machine-learning-based classification was, however, unaffected and achieved high performance independent of parameter combination.
**Limitations:** Phantom study.
**Ethics Committee Approval:** Not needed because of design as phantom study
**Funding for this study:** No funding
**Author Disclosures:**
Matthias Eberhard: Nothing to disclose
Davide Cester: Nothing to disclose
Bettina Baessler: Nothing to disclose
Thomas Daniel Jean Sartoretti: Nothing to disclose
Niklas Lohaus: Nothing to disclose
Andre Euler: Nothing to disclose
Hatem Alkadhi: Nothing to disclose

### RPS 313-2

**Micro-beam vs broad-beam radiation-driven effects on lungs studied by x-ray phase contrast 3D virtual histology**
*M. Romano*[1], A. Bravin[2], J. A. Laissue[3], M. Wright[4], V. Djonov[3], P. Coan[1]; [1]Munich/DE, [2]Grenoble/FR, [3]Berne/CH, [4]Roseville, CA/US
*(mariele.romano@lmu.de)*

**Purpose:** The aim of this study is to visualize and characterize the effects of both standard and novel spatially-fractionated radiotherapy (RT) methods using a label-free, three-dimensional (3D), full organ coverage, ex-vivo X-ray phase contrast imaging (XPCI)-based approach for virtual 3D histology technique. We focussed on the effects of different X-ray beam configurations on healthy rat lungs.
**Methods or Background:** Consisting of air sacs separated by thin tissue layers, lungs are ideally suited to XPCI, allowing small airways visualization with high contrast. Thirty-three healthy rat lungs were irradiated at the European Synchrotron (ESRF) with X-ray micrometre-wide and standard seamless broad beams delivering different doses. Rats were sacrificed 12 months after irradiation, lungs removed, fixed and embedded in paraplast blocks which were imaged by XPCI micro-computed tomography using a 20 keV monochromatic beam and voxel sizes of 1.63^3 and 0.65^3 µm3.
**Results or Findings:** The 3D nature of XPCI datasets was exploited to investigate the radio-induced pulmonary lesions. The effects of irradiation were strongly dependent both on the delivered dose and on the beam geometry. Fibrotic tissues were segmented and quantified. Conventional broad beam irradiations produced the largest amount of fibrotic tissue while spatially-fractionated RTs caused isolated scars with small iron accumulations and calcium deposits in damaged blood vessels.
**Conclusion:** The proposed method is well suited for 3D virtual histology of lung tissue and allows visualization, discrimination and detailed quantification of radio-altered tissues in the entire lung of healthy rats.
**Limitations:** n/a
**Ethics Committee Approval:** Animal care procedures conformed to the guidelines of the French government and were approved by the ESRF Internal Evaluation Committee for Animal Welfare and Rights.
**Funding for this study:** This study was supported by the German Research Foundation within the Research Training Group GRK2274.
**Author Disclosures:**
Alberto Bravin: Nothing to disclose
Valentin Djonov: Nothing to disclose
Michael Wright: Nothing to disclose
Mariele Romano: Nothing to disclose
Jean Albert Laissue: Nothing to disclose
Paola Coan: Nothing to disclose

### RPS 313-3

### A phantom study to investigate the reproducibility of radiomics variables with different magnetic resonance imaging (MRI) parameters

*R. N. Mitchell-Hay*, T. Ahearn, A. Murray; Aberdeen/UK

**Purpose:** A phantom study to investigate the reproducibility of Radiomics variables with different Magnetic Resonance Imaging (MRI) parameters.
**Methods or Background:** MRI has multiple different alterable parameters. This is particularly relevant in Radiomics which produces quantitative variables through high throughput analysis of the medical imaging. One of the limitations of Radiomics is reproducibility. This phantom study aims to investigate the repeatability of Radiomic variables across a range of different TR and TE values. The T1 Mapping and ECV Standardization Program phantom (Captur et al., 2016) (T1MES) was scanned under a range of different parameters, 10 times under the same conditions on a Philips 3T Achieva dStream using a body coil over a period of 4 months. The TE time ranged from 80 to 110ms while the TR ranged from 3000 to 5000ms. Radiomics analysis was performed on the same segmented section of the phantom across all acquired sequences. Intraclass Correlation Coefficient (ICC) was calculated across the different TE and TR ranges to investigate the reproducibility of Radiomics variables.
**Results or Findings:** Of 1596 variables calculated, 187 variables had excellent reproducibility (ICC >0.9) across the range of TE, while 82 variables had an excellent reproducibility across a range of TR values. 664 had good reproducibility (ICC >0.75) across the range of TEs, with 541 across the range of TR values.
**Conclusion:** Reproducibility of Radiomics variables in MRI is dependent on the imaging parameters and careful consideration of this, in combination with variable selection, is required when applying Radiomics within MRI.
**Limitations:** Small sample size and performed on a single site.
**Ethics Committee Approval:** Not applicable as a phantom study.
**Funding for this study:** RSAT funded Dr Rosalind Mitchell-Hay salary. Kind permission for use of T1MES phantom was given (Captur et al., 2016).
**Author Disclosures:**
Rosalind Nina Mitchell-Hay: Nothing to disclose
Alison Murray: Nothing to disclose
Trevor Ahearn: Nothing to disclose

### RPS 313-4

### X-ray dark-field computed tomography allows for the detection of radiation-induced lung damage in early stages

*F. T. Gassert*, R. Burkhardt, D. Pfeiffer, A. Fingerle, A. Sauter, J. Wilkens, F. Pfeiffer; Munich/DE

**Purpose:** To show the benefit of dark-field computed tomography imaging for the detection of radiation-induced lung damage in early stages compared to attenuation based imaging.
**Methods or Background:** We compared attenuation based CT imaging to dark-field (DF) CT imaging in a murine model of radiation-induced lung damage in the right lung (n=6) and a control group (n=6). Animals were scanned before irradiation and 12, 16, 20 and 24 weeks thereafter. Three radiologists assessed the images twice for the presence of lung damage and rated their confidence on a scale from 1 to 5. The inter-rater- and intra-rater-reliability was determined and rated with Cohen's $\kappa$ respectively Fleiss' $\kappa$. For the quantitative analysis the ratio of the mean pixel value of the right and left lung (⟨R⟩) was calculated. Results of the irradiated group were compared with the respective control group by using a t-test.
**Results or Findings:** The sensitivity of DF CT for radiation-induced lung damage in the reader study was significantly higher at 12 weeks (Att: 36.7%, DF: 53.3%, p=0.023) and at 16 weeks (Att: 50.0%, DF: 91.7%, p<0.001). The overall confidence of the readers was significantly higher when reading DF images (Att: 3.48, DF: 4.77, p<0.001). Both the average Intra-Rater-Reliability (Att: $\kappa$=0.82, DF: $\kappa$=0.91) and the Inter-Rater-Reliability (Att: $\kappa$=0.66, DF: $\kappa$=0.75) were higher for DF imaging. For attenuation based imaging the difference of the ratio ⟨R⟩ between the control group and the irradiated group became significant after 20 weeks (p=0.011), while for DF imaging it was already highly significant after 16 weeks (p=0.003).
**Conclusion:** This small animal study demonstrates that dark-field CT imaging allows for the detection of radiation-induced lung damage in early stages and, in that respect, is superior to conventional CT.
**Limitations:** n/a
**Ethics Committee Approval:** n/a
**Funding for this study:** n/a
**Author Disclosures:**
Andreas Sauter: Nothing to disclose
Florian Tilman Gassert: Nothing to disclose
Rico Burkhardt: Nothing to disclose
Jan Wilkens: Nothing to disclose
Alexander Fingerle: Nothing to disclose
Franz Pfeiffer: Nothing to disclose
Daniela Pfeiffer: Nothing to disclose

### RPS 313-5

### Influence of spectrum and patient size on dose conversion factors for chest radiography

*A. S. L. Dedulle*[1], N. Fitousi[2], H. Bosmans[2]; [1]Heverlee/BE, [2]Leuven/BE
*(an.dedulle@qaelum.com)*

**Purpose:** Chest posterior-anterior radiography is a commonly performed examination. The need for size-specific dosimetry was highlighted in earlier studies. In this study, the influence of patient size and spectrum on the dose conversion factors is investigated to evaluate how specific the conversion factors should be.
**Methods or Background:** A Monte Carlo (MC) framework dedicated to projection radiography was used with a set of 44 voxel phantoms. A standard chest PA geometry was simulated for 6 spectra (half value layer 3.8–8.8mmAl). The derived Monte Carlo patient-specific effective dose and organ dose conversion factors (lung, breast, skin, thyroid) were correlated with the body mass index (BMI) of the phantoms.
**Results or Findings:** All the derived MC conversion factors, besides the thyroid, were found to decrease with increasing BMI due to the shielding function of adipose tissue. The correlation was not significant (p>0.05) for the thyroid, because the increase in adipose tissue is less pronounced in the neck region. All MC conversion factors, but the ones for skin, were found to increase with increasing beam energy; for skin, this relationship was inverse. Increasing the beam energy might have a dose saving effect for the skin, but could increase the dose to other organs. Therefore, for similar entrance air kerma, the organ doses may vary a lot depending on the used spectrum. For example, patient-specific effective dose changed by 75% between the lowest and highest beam energy.
**Conclusion:** Dose conversion factors in chest radiography are influenced by both patient size and spectrum. As estimation of patient size and input of beam quality in projection radiography are not always straightforward, it is important to have the right tools when moving towards personalized dosimetry.
**Limitations:** Not applicable.
**Ethics Committee Approval:** Not applicable.
**Funding for this study:** PhD grant VLAIO [HBC.2016.0233].
**Author Disclosures:**
An Saskia Luc Dedulle: Research/Grant Support: grant VLAIO [HBC.2016.0233] Employee: Qaelum NV
Hilde Bosmans: Board Member: Qaelum NV Founder: Qaelum NV
Niki Fitousi: Employee: Qaelum NV

### RPS 313-7

### Validation of a statistical method for low-contrast detectability as a simple tool for QC in tomosynthesis

*N. Paruccini*[1], R. Villa[1], N. Oberhofer[2], P. Caricato[1], V. Faccenda[1], E. De Ponti[1]; [1]Monza/IT, [2]Bolzano/IT
*(n.paruccini@asst-monza.it)*

**Purpose:** The aim of this work was to validate an innovative and simplified method for threshold contrast evaluation in tomosynthesis based on a single acquisition of a homemade test tool.
**Methods or Background:** A homogeneous region was used to calculate threshold contrast for details of varying size with a statistical approach. A circular insert of aluminium was used to evaluate Spatial Modulation effect. An included aluminium step wedge permitted to express the threshold contrast also as an absolute quantity in terms of mmAl. Validation of the statistical method was carried out comparing contrast detail curves calculated with the CDMAM image analysis method. Cause Both CDMAM and statistical method (SM) suffer the presence of scatter radiation, acquisitions were performed in scattering free geometry, using 2 mm of aluminium block close to the collimator. Furthermore, SDNR was calculated for each aluminium step of the SM Phantom, from 0.2 to 1 mm thickness.
**Results or Findings:** In scatter free condition, the [c-d] curve calculated with SM doesn't depend on the ROI size. CDMAM and SM of projection images have been analysed. Contrast detail curves calculated with the proposed SM for threshold resulted in good accordance with the curves obtained with the reference CDMAM method, within 20% for all the detail sizes.
**Conclusion:** The statistical approach for threshold contrast evaluation, based on a single acquisition of a simple test object, has been proven to be a reliable means for relative and absolute image quality evaluation in tomosynthesis. This method might be an alternative to large_area SNDR and DQE assessment, too
**Limitations:** Tomosynthesis images are usually acquired without the presence of the grid, introducing a significant non-uniformity caused by scattering radiation. Scattering free condition was used to overcome this critical issue.
**Ethics Committee Approval:** Not required
**Funding for this study:** Nothing
**Author Disclosures:**
Nadia Oberhofer: Nothing to disclose
Raffaele Villa: Nothing to disclose
Nicoletta Paruccini: Nothing to disclose
Elena De Ponti: Nothing to disclose
Paolo Caricato: Nothing to disclose
Valeria Faccenda: Nothing to disclose

### RPS 313-8

### A patient-specific model for the estimation of the well-aerated lung volume from CT images: application to COVID-19 patients

L. Berta, C. De Mattia, F. Rizzetto, D. Lizio, S. Carrazza, A. Vanzulli, *A. Torresin*; Milan/IT

**Purpose:** to propose a patient-independent model for the well-aerated volume estimation (WAVE) using CT images of the lung
**Methods or Background:** The model was applied to the histograms of lungs segmented in CT images of the chest. Two cohorts of 20 patients each with healthy lungs (cohort1, from emergency department of our hospital) and with 4DCT acquisitions (cohort2, from a public database) were used to test the independence of our model from reconstruction parameters and respiratory cycle. Data points around the first peak, corresponding to the healthy parenchymal tissue, were fitted with a Gaussian function and WAVE.f was defined by integrating the fitted curve between the histogram range. WAVE.f was compared to another equivalent metric based on histogram thresholds (WAVE.th). Finally, WAVE.f and first order radiomic features were calculated for a third cohort of 20 COVID-19 patients and results were compared with those relative to cohort1.
**Results or Findings:** Average values of (84±2)% and (75±4)% were found for WAVE.f and WAVE.th for healthy subjects. Differences of 1%, 2% and up to 14% were found comparing WAVE.f values on images of patients from cohort1 reconstructed with a moderate iterative strength and FBP algorithm, 1 and 3 mm of slice thickness and different reconstruction kernels. Results from cohort2 showed independence of WAVE.f and WAVE.th from the respiratory motion in 80% and 20% of the cases, respectively. Patients from cohort1 were significantly different from COVID-19 patients for all the calculated metrics.
**Conclusion:** Unlike other metrics derived from CT histogram for the quantification of healthy lung parenchyma based on fixed thresholds, the proposed model is able to consider the inter- and intra-subject variability.
**Limitations:** Absolute values of WAVE metric is not yet validated clinically using functional data
**Ethics Committee Approval:** n/a
**Funding for this study:** n/a
**Author Disclosures:**
Francesco Rizzetto: Nothing to disclose
Luca Berta: Nothing to disclose
Domenico Lizio: Nothing to disclose
Angelo Vanzulli: Nothing to disclose
Alberto Torresin: Nothing to disclose
Cristina De Mattia: Nothing to disclose
Stefano Carrazza: Nothing to disclose

### RPS 313-9

### Comparison of attenuation measurements in unenhanced CT images and virtual non-contrast images calculated from Twin Beam Dual Energy CT scans

*D. Rieger*, S. C. Krämer, H. Seifarth; Esslingen/DE

**Purpose:** Twin Beam Dual Energy offers dual-Energy features on a single detector CT-scanner. The purpose of this study was to assess the reliability of attenuation values measured in virtual non-contrast images in various regions of the body using this technique compared to true unenhanced images.
**Methods or Background:** 100 patients after endovascular aortic repair (EVAR) were included in the study. For each patient, a triphasic scan was performed consisting of a single energy unenhanced scan followed by a dual-energy arterial and venous phase scan. Virtual non-contrast images were created. Two independent readers measured attenuation values in the thrombus outside the aortic stent, the psoas muscle, the subcutaneous fat and the liver. The measurements of both readers were averaged. A Wilcoxon signed rank test was used to assess differences between the density values for each region.
**Results or Findings:** Correlation between measurements from the two readers was good in all regions and for all scan series. There were no significant differences between attenuation values from true unenhanced and virtual non-contrast images in the aortic thrombus (mean 41,3 ± 9,5 HU; 41,2± 13,8 and 40,1 ± 13,3HU; p>0.05). For the other regions attenuation values differed significantly. Mean values were -106,7 ±15,3; -101,4±9,1 and -100,2 ±9,5; p<0.01 for subcutaneous fat; 43,1±56,7; 56,6 ± 6.7 and 58,0 ±7,4 for the psoas muscle and 43,9 ±10,1; 45,3 ± 9,4 and 53,4 ±9,7 for the liver (p<0.05 for all measurements).
**Conclusion:** Using Twin Beam Dual Energy CT technique, attenuation values measured in virtual non-contrast images differ significantly from values obtained from true unenhanced images in perfused tissues. Attenuation measurements in thrombus did not differ between virtual non-contrast and true unenhanced images.
**Limitations:** Only four types of tissue were analyzed
**Ethics Committee Approval:** Approval for retrospective data analysis
**Funding for this study:** Klinikum Esslingen
**Author Disclosures:**
Harald Seifarth: Nothing to disclose
Stefan C. Krämer: Nothing to disclose
David Rieger: Nothing to disclose

### RPS 313-10

### Testing CT image reconstruction methods using a 3D printed anthropomorphic thorax vessel phantom: deep learning vs iterative and filtered-back projection

*I. Hernandez-Giron*[1], G. Streekstra[2], J. den Harder[2], O. V. V. Ivashchenko[1], W. Veldkamp[1]; [1]Leiden/NL, [2]Amsterdam/NL
*(irene.debroglie@gmail.com)*

**Purpose:** To evaluate quantitatively commercial deep learning (DL) CT reconstruction image quality performance, versus filtered back-projection (FBP) and iterative reconstruction (IR), using an anthropomorphic phantom with uniform and textured background sections.
**Methods or Background:** An in-house anthropomorphic-thorax PMMA phantom, mimicking a thin patient section (300x200x29mm3), containing two 3D-printed elliptical lung vessels inserts (material_VisijetEX200) and spine (material_Teflon), was scanned (Canon Aquilion Prism-CT, clinical thorax protocol, varying mA, [CTDIvol=4.0-(2.1-clinical dose)-1.0-0.5-0.2mGy], x4 repetitions). Images were reconstructed with FBP, AIDR3De (IR) (FC08-kernel both) and AiCE-lung (DL). The phantom materials attenuation (HU) and SNR in PMMA-thorax parts were analyzed. Noise properties (inter-acquisitions standard deviation maps) were compared across backgrounds [uniform_(PMMA-thorax); textured-lung_vessels], dose and reconstruction.
**Results or Findings:** The highest averaged attenuation differences (AIDR3De_baseline, (material,Average_HU_Diff,[HU_range]) were found for the lowest dose and high attenuation materials; AIDR3De_vs_FBP: (PMMA-thorax, (Average_HU_Diff=10HU), [-30,-1HU]); (Teflon-spine, (Average_HU_Diff=-54HU), [-62,-47HU]); (VisijetEX200-vessels, (Average_HU_Diff=-21HU), [-60,1HU]; (air,(Average_HU_Diff=13HU), [-5,31HU]). For AIDR3De_vs_AiCE: (PMMA-thorax, (Average_HU_Diff=-0.5HU), [-3,3HU]); (Teflon-spine, (Average_HU_Diff=-27HU), [-32,-25HU]); (VisijetEX200-vessels, (Average_HU_Diff=-19HU), [-40,-3HU]; (air, (Average_HU_Diff=8HU), [6,12HU]). Average PMMA_SNR increased with dose at different rates per reconstruction. From lowest to highest dose, values were [FBP_SNR=(1.1-2.3-3.4-4.8-6.4)]; [AIDR3De_SNR=(4.2-4.9-6.0-7.6-10.0)]; [AiCE-Lung_SNR=(3.9-3.8-4.5-5.1-6.0)]. Noise maps were homogeneous at all dose-reconstructions combinations for uniform backgrounds (PMMA-thorax,Teflon-spine). They were non-homogeneous and enhanced in vessel edges for textured backgrounds (vessel_inserts) for AIDR3De and AiCE.

**Conclusion:** Attenuation and noise properties differences were found in an anthropomorphic textured lung vessel phantom between deep learning, iterative (current clinical standard) and FBP reconstruction. Stronger noise reduction was observed in the uniform phantom parts. Noise was enhanced in vessel edges for IR and DL-based reconstruction compared to FBP.
Our result suggest that anthropomorphic textured background phantoms better represent complex patient anatomy and are better suited for evaluation of IR and DL-based reconstructions.
**Limitations:** The phantom represents a simplified small-sized patient, containing textured lung vessels's complexity, except parenchyma. Alternative tissue-equivalent materials can be investigated.
**Ethics Committee Approval:** Phantom-study-N/A
**Funding for this study:** NWO-Talent-Programma_Personal_Veni_grant (17378-Through_the_eyes_of_AI)
NWO-OTP_grant-CLUES-project_13592:Phantom creation
**Author Disclosures:**
Oleksandra V. V Ivashchenko: Nothing to disclose
Wouter Veldkamp: Nothing to disclose
J.M. den Harder: Nothing to disclose
Irene Hernandez-Giron: Research/Grant Support: The phantom was created under NWO-OTP grant (CLUES-project: 13592) Research/Grant Support: Irene Hernandez received a NWO-Talent Programma personnal Veni grant (project 17378-Through the eyes of AI)
Geert Streekstra: Nothing to disclose

### Research Presentation Session: Radiographers

### RPS 314

### Investigations in radiography

### RPS 314-1

**Perception of breast screening clients towards male radiographers working in breast screening**

*M. Iles*, F. Zarb, D. Mizzi; Msida/MT
*(m.iles@student.maastrichtuniversity.nl)*

**Purpose:** To evaluate the perception of breast cancer screening clients towards male radiographers performing mammography screening in Malta.
**Methods or Background:** The research consisted of a quantitative, prospective, non-experimental, and descriptive study design. Questionnaires were distributed to all women, aged 50-69 years, who were invited and attended either prevalent or incident breast screening mammography at a local breast screening unit. A total of 204 questionnaires were collected between February and March 2020.
**Results or Findings:** Thirty-five percent of participants were aged 50-54 years; 50.5% completed their highest level of education at secondary level and 81.4% of participants were invited for an incident screening round. The participants were mostly (87.7%) Maltese. Twenty-nine percent stated they would agree to have a male radiographer performing their mammogram, but they would feel more comfortable having a female radiographer. Also, 31.4% of participants stated that a male radiographer would not affect their future attendance to breast cancer screening appointments.
**Conclusion:** Even though several women would accept a male radiographer performing their mammogram, further evidence based research is required on the perception of women towards male radiographers performing screening mammography before implementing in the local scenario. Further studies are needed to also include symptomatic women.
**Limitations:** Due to the COVID-19 pandemic and subsequent closure of the local breast screening unit, only a small sample was obtained. This resulted in a limited distribution of demographics limiting comparison to literature findings.
**Ethics Committee Approval:** Approval was obtained from the University of Malta Research Ethics Committee (2657_30082019).
**Funding for this study:** No specific grant from any funding agency in the public, commercial or non-profit sectors was received.
**Author Disclosures:**
Deborah Mizzi: Nothing to disclose
Maria Iles: Nothing to disclose
Francis Zarb: Nothing to disclose

### RPS 314-2

**Profile-based kV and NI selection to evaluate auto prescription**

*N. Sogge*[1], K. Jensen[1], H. K. Andersen[1], F. Rupcich[2], J. Glittum[1], M. Svanteson[1]; [1]Oslo/NO, [2]Wauwatosa, WI/US
*(nicsog@ous-hf.no)*

**Purpose:** Auto Prescription can be applied to optimize radiation dose and image quality across patient sizes by automatically adjusting kV and Noise Index (NI) as a function of patient size and clinical task. Size Adjusted Noise Index (SANI) may also be enabled to further tune the noise and dose level.
**Methods or Background:** Image quality of chest CT and abdominal/pelvic CT were compared using the hospital's original protocol and a protocol based on parameters suggested by the Auto Prescription algorithm. Each patient was examined with both protocols on the same scanner. We tested which kV and NI Auto Prescription would give for different patient sizes and adjusted this to the hospital's requirements. Four radiologists compared the two abdominal/pelvic exams in a random order. 16 patients with AP+Lat diameters between 46-57cm were evaluated for liver contrast, sharpness psoas muscle, perirenal fascia and overall impression. The following scale was used: considerably/slightly worse, no difference, slightly/considerably better. Ordinal logistic regression analysis was performed on the radiologists' score. Chest images will be evaluated with the criteria: pulmonary vessels, segmental bronchi, lung parenchyma, border pleura - thoracic wall and overall impression.
**Results or Findings:** The score percentage (better – no difference) for the new abdominal/pelvic protocol compared to the original protocol were on liver contrast: 62,5%-18,8%, sharpness of psoas muscle: 45,3%-37,5%, perirenal fascia: 32,8%-50,0%, and overall impression: 50,0%-25,0%. Regression analysis showed the differences were significant. With one exception, CTDI was either lower with the new protocol or within 10% of the original CTDI. 12 of the 16 cases had lower CTDIs. The chest examination is work in progress.
**Conclusion:** Auto Prescription proved useful for optimizing image quality or dose with patient size.
**Limitations:** n/a
**Ethics Committee Approval:** Institutional approval.
**Funding for this study:** n/a
**Author Disclosures:**
Nicolas Sogge: Other: The Oslo University Hospital has a collaboration with GE Healthcare.
Jan Glittum: Author: The Oslo University Hospital has a collaboration with GE Healthcare.
Kristin Jensen: Other: The Oslo University Hospital has a collaboration with GE Healthcare.
Mona Svanteson: Other: The Oslo University Hospital has a collaboration with GE Healthcare.
Franco Rupcich: Employee: Employee at GE Healthcare.
Hilde Kjernlie Andersen: Other: The Oslo University Hospital has a collaboration with GE Healthcare.

### RPS 314-3

**The impact of ASiR-V and kVp variations on CT image quality and radiation dose**

*S. D. Moerup*[1], J. G. Stowe[2], H. Precht[1], S. J. Foley[2]; [1]Odense/DK, [2]Dublin/IE
*(sdmo@ucl.dk)*

**Purpose:** To investigate how ASiR-V and kVp changes in Computed tomography (CT) affects radiation dose and image quality, when using automatic tube current modulation (ATCM) for different sized phantoms.
**Methods or Background:** A Liver-Phantom with two different liver inserts (QRM, Moehrendorf, Germany) was repeatedly scanned on a 16cm detector CT scanner (GE Healthcare, Milwaukee, WI, USA). Extension rings, representing fat, were additionally applied to the phantom to simulate patients of different sizes (small: 30cm diameter, medium: 35cm and large: 40cm). Scans were performed with consistent pitch (0.992), rotation time (0.5s), slice thickness (0.625) and collimation (80mm), while other parameters were varied (kVp: 80/100/120/140; Noise Index: 13/22; mA interval 80-720, ASiR-V: 30/60/100%). CTDI and DLP was recorded for each scan and image quality was assessed using objective metrics in predefined anatomic areas (HU, SD and CNR). Radiation dose and image quality was evaluated using ANOVA.
**Results or Findings:** A total of 216 scans were performed, resulting in 432 measurements of image quality. Radiation dose reductions (1-85%) were noted for all phantom sizes and kVp settings when more ASiR-V is applied. Regarding image quality, no significant differences in HU was found between ASIR-V levels (p=0.064). For a majority of the scans the lowest SD and highest CNR was obtained at 60% ASiR-V, with SD increasing at 100% ASiR-V because of the large reduction in tube current applied (80 mA). Notably, image quality in the low contrast area of the liver insert was impaired when the large phantom was scanned with 100% ASiR-V and either 80/100kVp (NI 22).
**Conclusion:** Substantial radiation dose reductions are possible with increasing ASiR-V levels. However, image quality deteriorates when 100% ASiR-V is applied due to low applied tube current.

**Limitations:** Not applicable
**Ethics Committee Approval:** Not applicable
**Funding for this study:** Not applicable
**Author Disclosures:**
Helle Precht: Nothing to disclose
John Gerard Stowe: Nothing to disclose
Svea Deppe Moerup: Nothing to disclose
Shane J Foley: Nothing to disclose

### RPS 314-4

**Predictors of image quality at human foetal post-mortem micro CT**

*I. C. Simcock*, S. C. Shelmerdine, D. Langan, N. Sebire, O. J. Arthurs; London/UK
*(ian.simcock@gosh.nhs.uk)*

**Purpose:** To determine the main factors determining diagnostic image quality for human foetal whole body post-mortem micro-CT.
**Methods or Background:** Post-mortem micro-CT is a non-invasive, high resolution (up to 5 µm) imaging method which can provide high diagnostic accuracy particularly for fetuses below 20 weeks gestation, however the main predictors of image quality are yet to be determined. In this prospective, single centre study, we imaged all fetuses referred for micro-CT over a 3 year period on a Nikon Micro-CT scanner. Axial images of the head and chest were retrospectively assessed by 2 paediatric radiologists for maceration and image quality. Simple and multivariable linear regression models were fitted for outcome variables, with demographics, maceration score and tissue preparation considered as predictor variables.
**Results or Findings:** 258 foetal micro-CT scans were evaluated with mean foetal weight 64.8g (range 2.6 - 300) and mean gestational age of 16 weeks (range 11 – 24). Diagnostic image quality was recorded in 497/516 (95.9%) head or chest images. Maceration score was the strongest positive indicator for poor image quality ($p<0.001$), with gestational weight being a weak negative predictor of image quality ($p<0.001$).
**Conclusion:** Low weight, non-macerated fetuses yielded the highest diagnostic image quality scores with micro-CT, emphasising its usage as an excellent clinical tool for early pregnancy loss.
**Limitations:** Although maceration and gestational weight were identified as predictors of image quality, it is believed that other factors will also play a role in image quality and will require investigation.
**Ethics Committee Approval:** Ethical approval was granted for this study (13/LO/1994 and 17/WS/0089).
**Funding for this study:** Supported by the National Institute for Health Research (NIHR), Medical Research Council, Royal College of Radiologists, Great Ormond Street Hospital Children's Charity and NIHR GOSH/ICH Biomedical Research Centre.
**Author Disclosures:**
Ian C. Simcock: Nothing to disclose
Dean Langan: Nothing to disclose
Neil Sebire: Nothing to disclose
Susan Cheng Shelmerdine: Nothing to disclose
Owen Arthurs: Nothing to disclose

### RPS 314-5

**Automated artifact detection in uniform mammography images: transfer learning approach**

*E. Alukić*, N. Mekis, J. Žibert; Ljubljana/SI
*(erna.alukic@zf.uni-lj.si)*

**Purpose:** The aim was to build a system for the automatic detection of artifacts in mammographic images that can be used in practice as an analysis tool for the daily quality control of flat-field homogenous images in mammography systems.
**Methods or Background:** A deep learning system was designed for recognition of the most frequent artifacts in mammography (contrast non-uniformity, ghosting, lines, dead pixels and acquisition errors). Total of 542 mammographic images were acquired from 7 different mammography units. A deep learning approach was performed with the convolutional neural network VGG19 (CNN), which was trained to detect the given artifacts by transfer learning approach. For each artifact a separate CNN was trained and evaluated by measuring the AUCs in the ROC analysis.
**Results or Findings:** The evaluation results were measured by AUC scores for each individual artifact with sensitivity and specificity at Youden cutoff points. The AUCs results were: contrast non-uniformity 0.995, ghosting 0.986, lines0.954, dead pixels and acquisition errors 0.997 respectively. The overall detection results were better in a comparison with the results achieved by conventional machine learning approaches. At the same time the transfer learning approach, that was used in our modelling, proved to be efficient even in the case of small amount of training material (images of artifacts), which are usually needed in huge amounts for deep learning approaches.
**Conclusion:** The developed system was proven to be efficient in case of selected artifacts detection on the flat-field homogenous mammography images. The lowest score was observed at line detection, but even though the success rate was 95.4%.
**Limitations:** No limitations.
**Ethics Committee Approval:** It was not necessary.
**Funding for this study:** No funding.
**Author Disclosures:**
Nejc Mekis: Nothing to disclose
Janez Žibert: Nothing to disclose
Erna Alukić: Nothing to disclose

### RPS 314-6

**Intra/inter-observer repeatability in liver computed tomography volumetry in patients undergoing radioembolisation simulation**

*A. Masperi*, L. Bombelli, V. Cubadda; Milan/IT
*(andrea.masperi1992@gmail.com)*

**Purpose:** Recently, angiography can be associated using MDCT at the same time (Angio-CT suite), which may provide useful precise information, regarding the volumes of future treated liver area in pre-treatment for Trans-Arterial Radio Embolization (TARE). The careful evaluation of MDCT is an essential step for the treatment planning, because it may provide unique volumetric data (CTVs), which are information useful for an effective and safe TARE.
**Methods or Background:** We select 17 consecutive patients, who underwent pre-treatment work-up for TARE, in our Institution, between May 2019 and February 2020. For each patient, four specific parameters are evaluated from MDCT achieved during treatment simulation: healthy liver volume (HLV), the whole hepatic parenchyma (THV=healthy liver and TTV= tumor) involved by TARE, and whole liver volume (WLV). Four independent observers: R1 (expert Interventional Radiologist), T1, T2 and T3 (Radiographers, with different experience in the field of Interventional Radiology) are involved in the imaging analysed. The intraclass correlation coefficient (ICC) is used to quantify intra-inter observer repeatability and accuracy.
**Results or Findings:** All the four observers detect the same number of hepatic lesion(s) per patient. Regarding the three radiographers, the intra-observer reliability for CTVs is very high (0.997 to 1.000). Also Inter-observer reproducibility between radiographers is excellent regarding CTVs, (0.965 to 0.999). The accuracy of radiographers evaluation is very high (0.964 to 0.999).
**Conclusion:** The high intra-inter observer reproducibility shows that a properly trained radiographers might have the same accuracy as interventional radiologists, in assessing liver CTVs data for planning TARE.
**Limitations:** The limited number of patients undergoing pre-therapeutical imaging so far leads to a limitation to this study, although promising in the first analysis.
**Ethics Committee Approval:** The ethics committee of our institute approves the study (trial ID:2234 - protocol: hepatic volumetry).
**Funding for this study:** None
**Author Disclosures:**
Luca Bombelli: Nothing to disclose
Valerio Cubadda: Nothing to disclose
Andrea Masperi: Nothing to disclose

### Research Presentation Session: Radiographers

### RPS 414

### Professional issues in radiography

### RPS 414-1

**A critical appraisal of the quality of guidelines for radiation protection in interventional radiology using the AGREE II tool: a EuroAIM initiative**

*M. Zanardo*[1], R. Gerasia[2], L. Giovannelli[3], G. Scurto[2], P. Cornacchione[4], A. Cozzi[1], D. Catania[1], S. Durante[5]; [1]Milan/IT, [2]Palermo/IT, [3]Trento/IT, [4]Rome/IT, [5]Bologna/IT

**Purpose:** The growing interest in radiation protection and the importance of awareness in radiation safety resulted in the production of several clinical practice guidelines, with an unavoidable variability in terms of overall quality. The aim of this review was to assess the methodological quality of guidelines for patient safety in interventional radiology department and to compare their recommendations.
**Methods or Background:** In September 2020, a systematic search was performed using MEDLINE, EMBASE, National Guideline Clearinghouse, and National Institute for Health and Clinical Excellence, to find guidelines regarding radiation protection in interventional radiology departments. The evaluation of guidelines quality was performed independently by three authors using the AGREE II tool. We excluded those guidelines that were not primarily focused on radiation protection or not mainly focused on interventional radiology.
**Results or Findings:** Our literature search identified 106 citations, and after applying exclusion criteria, 11 guidelines were eventually included. Guidelines were published between 2009 and 2018 and the overall quality of included guidelines was acceptable (median 72%, IQR 64%–83%), with only one considered low-quality (<60%). “Scope and Purpose”, “Clarity of Presentations”, and “Editorial Independence” had the best domain results (87%, 76%, and 75% respectively), while “Applicability”, “Rigor of Development”, and “Stakeholder Involvement” the worst (46%, 49%, and 52% respectively). Most of the authors were interventional radiologists (168/224, 75%).
**Conclusion:** The overall quality of all guidelines was acceptable, with one-third of them reaching a “high-quality” score. The domain “Applicability” showed the lowest median result suggesting that possible strategies for implementing guideline recommendations in clinical practice should be researched.
**Limitations:** AGREE II tool does not assess the consistency of guidelines.
**Ethics Committee Approval:** Not applicable.
**Funding for this study:** Not applicable.
**Author Disclosures:**
Andrea Cozzi: Nothing to disclose
Diego Catania: Nothing to disclose
Patrizia Cornacchione: Nothing to disclose
Stefano Durante: Nothing to disclose
Giuseppe Scurto: Nothing to disclose
Moreno Zanardo: Nothing to disclose
Lorenzo Giovannelli: Nothing to disclose
Roberta Gerasia: Nothing to disclose

### RPS 414-2

**A systematic review of reasonable and feasible adjustments to facilitate patients with autism spectrum disorders undergoing MRI examinations**
*N. Stogiannos*[1], S. Carlier[2], J. Harvey-Lloyd[3], A. Brammer[4], K. Cleaver[5], J. McNulty[6], C. S. d. Reis[2], C. Malamateniou[1]; [1]London/UK, [2]Lausanne/CH, [3]Suffolk/UK, [4]Manchester/UK, [5]Greenwich/UK, [6]Dublin/IE
*(nstogiannos@yahoo.com)*

**Purpose:** To summarise all evidence-based interventions used within radiography practice when scanning patients with autism spectrum disorders (ASD) in the MR environment.
**Methods or Background:** A systematic review of the literature was conducted from July to October 2020. The methodology of this study was in line with the Preferred Reporting Items for Systematic Reviews and Meta-Analyses (PRISMA) guidelines. An electronic search was performed across nine databases. Specific keywords were used for this search, with Boolean operators. Filters included: English language, studies published after 2010, only full-text, and peer-reviewed studies. The included studies were critically evaluated using the Critical Appraisal Skills Programme (CASP) tools. Two reviewers conducted searches and the final eligible studies were determined by consensus between two independent researchers.
**Results or Findings:** Twenty-three studies were eligible for inclusion. This review indicates that current methods to facilitate MRI examinations for ASD patients were based on the following core themes: 1) image acquisition and optimisation techniques, 2) image post-processing software to improve image quality, 3) technology-assisted distraction techniques, 4) simulation approaches, 5) psychological interventions, while a core component of the majority of the interventions was 6) effective communication with patient and carers.
**Conclusion:** MRI can present challenges for neurotypical patients. Given the communication difficulties, sensory sensitivities, and increased anxiety of ASD individuals it is vital to employ customised interventions to improve the experience of ASD patients when undergoing MRI examinations to allow truly patient-centred care. These might be person-centred and technology-enabled, and a standardisation of these protocols should occur for the benefit of the patients.
**Limitations:** Not applicable
**Ethics Committee Approval:** Not applicable
**Funding for this study:** The Society and College of Radiographers CORIPS grant scheme
**Author Disclosures:**
Sarah Carlier: Nothing to disclose
Claudia Sa dos Reis: Nothing to disclose
Karen Cleaver: Nothing to disclose
Jonathan McNulty: Nothing to disclose
Nikolaos Stogiannos: Nothing to disclose
Andrea Brammer: Nothing to disclose
Jane Harvey-Lloyd: Nothing to disclose
Christina Malamateniou: Nothing to disclose

### RPS 414-4

**Anxiety and claustrophobia evaluation in patients undergoing magnetic resonance imaging**
B. Lopes, *K. B. Azevedo*, A. Abrantes, S. I. Rodrigues, L. Ribeiro, B. Vicente, R. P. P. Almeida; Faro/PT

**Purpose:** The purpose of this research was to evaluate the anxiety and claustrophobia levels in patients undergoing Magnetic Resonance Imaging of the Head, Extremities or Breast.
**Methods or Background:** This is a quantitative and descriptive-correlational research. Data collection was done in the Magnetic Resonance area from the Imaging Department. All the patients undergoing head, extremities or breast scans were invited to fulfill the sociodemographic, anxiety (State-Trait Anxiety Inventory) and claustrophobia (Questionnaire of Claustrophobia for Patients Undergoing Magnetic Resonance Imaging) before the procedure. Data were analysed using SPSS version 26.
**Results or Findings:** The internal consistency of the anxiety and claustrophobia scales was above 0,9. The mean age of participants was 46 years. Most of the patients were moderately anxious and the majority of the patients related low concern related to claustrophobic environment of the Magnetic Resonance equipment. Anxiety is correlated with claustrophobia (r=0,47 p=0,000) and both variables are correlated with female genre (anxiety has a r=0,379 and p=001 / claustrophobia has a r=0,429 and p=0,000).
**Conclusion:** Anxiety is positively correlated with anxiety, which means that higher the anxiety, higher the claustrophobia that patients feel. Also, the female genre is associated with higher levels of both anxiety and claustrophobia, which requires closer attention.
**Limitations:** Data was collected in only one institution, composed by 3 hospitals, which may difficult generalizations to other regions of the country. Data collection from patients is a difficult process, which limited the sample size. Results would be more robust if there is a larger sample. The fulfillment of the survey was onsite, which may influence the answers provided.
**Ethics Committee Approval:** Authorization (Refª 87-2018) from the Administration Board, after the agreement from the Ethics Commission.
**Funding for this study:** No funding
**Author Disclosures:**
António Abrantes: Nothing to disclose
Kevin Barros Azevedo: Nothing to disclose
Rui Pedro Pereira Almeida: Nothing to disclose
Bianca Vicente: Nothing to disclose
Sónia Isabel Rodrigues: Nothing to disclose
Bruno Lopes: Nothing to disclose

### RPS 414-3

**The design and prototype testing of a novel device to protect the use of DR detectors in radiography practice**
*E. Conroy*, A. S. Karam, E. O'Cearbhaill, A. Toohey, L. A. Rainford; Dublin/IE
*(emily.conroy@ucdconnect.ie)*

**Purpose:** Digital radiography (DR) detectors are removed from their normal X-ray housing when imaging patients who require modified examinations e.g. imaging patients in wheelchairs or trauma situations, with a subsequent increased risk of damage to the DR detector. A flexible supporting prototype was to be developed with the aim of stabilizing the DR detector outside of its routine housing for: antero-posterior (AP) chest x-ray, horizontal beam lateral and “skyline” knee projections.
**Methods or Background:** Prototype components needed to remain outside the area of investigation and be fully supportive of the DR detector for use in portrait and landscape positions. The prototype needed to be lightweight, easy to transport and facilitate efficient positioning by the radiographer. The device was required to be chemically resistant to allow for repeated cleaning with Actichlor and Cliniwipes and facilitate cleaning between consecutive patients.
**Results or Findings:** A prototype was developed, using an off-the-shelf monitor arm and a 3D printable hook component. The monitor arm could hold a maximum weight of 10kg: the Carestream detector (3.63kg) and the detector holder (3kg) had a a combined weight of 6.63kg. A range of motion was possible at the 3 joints (180, 360 and 360 degrees), allowing for a sufficient degree of flexibility. The hook component was 3D printed and attached to the off-the-shelf monitor arm.

**Conclusion:** The device was tested and demonstrated particular potential for AP chest imaging for patients in wheelchairs. Further research is warranted to improve the prototype design for X-ray table use and radiographer feedback was collated to inform further development.
**Limitations:** Not applicable
**Ethics Committee Approval:** Institutional waiver from full ethics application confirmed
**Funding for this study:** None
**Author Disclosures:**
Aliaa Sherif Karam: Nothing to disclose
Andrea Toohey: Nothing to disclose
Eoin O'Cearbhaill: Nothing to disclose
Louise A. Rainford: Nothing to disclose
Emily Conroy: Nothing to disclose

### Research Presentation Session: Radiographers

### RPS 514

### Safety in radiography

### RPS 514-1

**Dosimetric comparison of two radiotherapy techniques in irradiation of breast cancer**

*B. S. M. Vaz*[1], A. F. L. Abrantes[2], S. I. Rodrigues[2], L. P. V. Ribeiro[2], M. Ramos[2], F. Serra[2], B. Vicente[2], R. P. P. Almeida[2]; [1]Vila Nova de Cacela/PT, [2]Faro/PT
*(brunav7@hotmail.com)*

**Purpose:** To compare and analyze the dosimetric aspects of three-dimensional conformational radiation therapy (3DCRT) and Volumetric Modulated Arc Therapy (VMAT) techniques in irradiation of breast cancer.
**Methods or Background:** A quantitative and descriptive-correlational study was carried out and a total of 47 patients were included, of which 24 underwent 3DCRT and 19 were treated with VMAT. In dosimetric analysis we obtained dose-volume histograms (HDV) evaluation. Besides that, parameters such as conformity index, homogeneity index and body dose values were calculated in order to compare both techniques. The dose prescribed was 50 Gy in 25 fractions with conventional fractionation to the whole breast. The dose prescribed was 50 Gy in 25 fractions with 2 Gy per fraction, 1 fraction per day for 5 days per week to the whole breast.
**Results or Findings:** Dosimetric analysis showed that VMAT presents a greater coverage of the planning target volume (PTV) according to definition proposed by ICRU Report 83. Conformity and Homogeneity indexes are also higher in patients who underwent irradiation with VMAT technique (between 0.11 to 0.16). However, 3DCRT presents, on average, a greater protection of the organs at risk. Data showed that VMAT may have an effect of “low dose exposure bath” on adjacent healthy organs, mainly on the contralateral breast.
**Conclusion:** In conclusion, VMAT presents advantages comparing to 3DCRT in cases where the cervical and axillary ganglionar nodes are involved to do the treatment. However, in selected cases based in stage and patient anatomy 3 DCRT can be a preferred technique because it achieves better protection of the organs at risk.
**Limitations:** Sample size.
**Ethics Committee Approval:** Ethics committee approved the study
**Funding for this study:** No funding
**Author Disclosures:**
Magda Ramos: Nothing to disclose
Rui Pedro Pereira Almeida: Nothing to disclose
Sónia Isabel Rodrigues: Nothing to disclose
Fábio Serra: Nothing to disclose
Antonio Fernando Lagem Abrantes: Nothing to disclose
Bianca Vicente: Nothing to disclose
Bruna Sofia Mangas Vaz: Nothing to disclose
Luís Pedro Vieira Ribeiro: Nothing to disclose

### RPS 514-2

**Deep-learning reconstruction in CT**

*S. D. Moerup*[1], J. G. Stowe[2], H. Precht[1], S. J. Foley[2]; [1]Odense/DK, [2]Dublin/IE
*(sdmo@ucl.dk)*

**Purpose:** To investigate the influence of the new deep-learning image reconstruction TrueFidelity on image quality for cardiac computed tomography angiography scanning in different size phantoms.
**Methods or Background:** Four sizes of cardiac phantom (small: 30cm, medium: 35cm, large: 40cm, XL: 60cm) (QRM, Moehrendorf, Germany) were scanned on the Revolution Apex CT scanner (GE Healthcare, USA) using combinations of ASiR-V (50/70%), kV assist with a standard cardiac protocol. Radiation dose was recorded by CTDI and DLP and each scan reconstructed with three levels of TrueFidelity (low (TF-L) medium (TF-M) and high (TF-H)). Image quality was assessed using objective metrics in predefined areas (SD and CNR) and subjectively by evaluating the nine calcifications in the phantom. Descriptive statistics was performed.
**Results or Findings:** By increasing ASIR-V from 50-70%, radiation dose reduced by 42%, 81% and 101% for the small, medium and large phantoms respectively, with a slight (1%) increase for the XL phantom. When using ASiR-V50%, increasing TrueFidelity strength lead to image noise reductions (17-56%) for the small, medium and large phantom. For ASiR-V70%, image noise is reduced (0-45%) for both small and medium phantom. For the large and XL phantom noise increases 11% for TF-L, but reduces (4-37%) for TF-M and TF-H. CNR improves (4-105%) for all levels of TrueFidelity when using ASiR-V50%. For ASiR-V70% the CNR decreased (4-15%) for TF-L for all phantoms and increased (4-64%), for TF-M and TF-H. For the small, medium and large phantom 7/8 calcifications were visible with TrueFidelity, the XL phantom 5/6 calcifications.
**Conclusion:** TrueFidelity reduces image noise and improves the CNR, the largest noise reduction (56%) is obtained with ASiR-V50%. For the XL phantom the change in noise and CNR was significantly lower.
**Limitations:** Not applicable
**Ethics Committee Approval:** Not applicable
**Funding for this study:** Not applicable
**Author Disclosures:**
Helle Precht: Nothing to disclose
John Gerard Stowe: Nothing to disclose
Svea Deppe Moerup: Nothing to disclose
Shane J Foley: Nothing to disclose

### RPS 514-3

**Evaluation of residual activity in a nuclear medicine department**

*A. D. S. Queirós*, S. I. Rodrigues, L. P. V. Ribeiro, A. F. L. Abrantes, J. Rosas, R. P. P. Almeida, J. Pinheiro; Faro/PT
*(andredsqueiros@gmail.com)*

**Purpose:** The exposure to ionizing radiation by radiographer in Nuclear Medicine departments is inevitable, as this area has the highest rates of residual activity. The main objective of this study was to ascertain to which doses are the staff exposed in the Radiopharmacy and the Injection Room (IR).
**Methods or Background:** Activity measurements were performed at different points of the department and at different distances (0m, 0.5m and 1m) of the radioactivity waste. These measurements were taken for 63 days, with a survey-meter Geiger-Müller.
**Results or Findings:** In Radiopharmacy, the mean dose rate values obtained were 77.16, 1.14, 0.57 and 0.52 μSv/h at distances of 0m, 0.5m, 1m and 1.5m respectively, and in the Injection Room, mean dose rate values of 1.75, 0.31 and 0.19 μSv/h were observed at distances of 0m, 0.5m and 1m. At 1.5m, being a general point in this area, a value of 0.361 mSv/year was obtained, and in the IR at a distance of 1m, the value of 0,132 mSv/year was obtained for each radiographer.
**Conclusion:** We conclude that the effective dose values are lower than the dose limits legally established (20 mSv per year) and that management of radioactive waste is greatly optimized.
**Limitations:** Not applicable
**Ethics Committee Approval:** Ethics committee approved the study
**Funding for this study:** No funding was received for this work.
**Author Disclosures:**
Joao Pinheiro: Nothing to disclose
Rui Pedro Pereira Almeida: Nothing to disclose
Sónia Isabel Rodrigues: Nothing to disclose
André Daniel Santos Queirós: Nothing to disclose
Joana Rosas: Nothing to disclose
Antonio Fernando Lagem Abrantes: Nothing to disclose
Luís Pedro Vieira Ribeiro: Nothing to disclose

### RPS 514-4

**Establishment of local diagnostic reference levels and trigger values analysis in a cath-lab department**

A. Cortez[1], *J. Santos*[1], J. Costa[2], G. Paulo[1]; [1]Coimbra/PT, [2]Braga/PT
*(joanasantos@estescoimbra.pt)*

**Purpose:** This study aims to assess the examination dose values in interventional cardiology procedures to establish diagnostic reference levels and analyse number of procedures that exceed trigger values.

**Methods or Background:** Data about the procedure type, operator identification, procedure access, patient characteristics (gender, age and weight), fluoroscopy time (min), Kerma Area Product - PKA (Gy.cm2), was retrospectively collected, for a period of 12 months. Patients were categorised in three weight groups: low (<65kg), standard (65kg to 75kg) and high (>75kg).
**Results or Findings:** The majority of the 2540 examinations were diagnostic procedures (65%) and the more common access was via the right radial artery (92%). Significant differences (p<0,05) in fluoroscopy time and PKA values, between procedure access, patient weight group and type of procedure were found. The trigger level of PKA (>300Gy.cm2) value was exceeded in 0,5% of the procedures, lower than the incidence found in the literature. The local DRL values for diagnostic and therapeutical procedures were respectively 39 and 111Gy.cm2, in line with values published in the literature.
**Conclusion:** The dose distribution is in line with the ones found in the literature. The percentage of patients that exceed one of trigger levels is lower when compared with the literature, however a patient follow-up plan should be established.
**Limitations:** It was not possible to obtain Air Kerma at reference point retrospectively in order to analyse that trigger value.
**Ethics Committee Approval:** This study was approved by the institutional ethical committee.
**Funding for this study:** This study was performed without funding.
**Author Disclosures:**
Graciano Paulo: Nothing to disclose
Angela Cortez: Nothing to disclose
Joana Santos: Nothing to disclose
João Costa: Nothing to disclose

### Research Presentation Session: Radiographers

### RPS 614

### Education and training in radiography

### RPS 614-1

**Information seeking preferences among patients undergoing radiotherapy in Malta**
*C. Borg*, G. v. Dijk, K. Borg Grima; Msida/MT
*(clarissa.borg.16@um.edu.mt)*

**Purpose:** Adequate information is necessary to help cancer patients cope with their disease and treatment. The aim of this study was to investigate whether radical cancer patients undergoing radiotherapy, seek information regarding their pathology. The objectives of this study were to identify any correlation between the sources of information used by cancer patients and the information-seeking tendencies of these patients. Moreover, the topics searched by the patients, and during which time frame patients mostly sought information, were all investigated factors.
**Methods or Background:** This study used a quantitative cross-sectional research design, in which an already established questionnaire was distributed by intermediaries to consenting patients.
**Results or Findings:** In this research, 42 patients undergoing radical radiotherapy consented to participate. The results demonstrated that the information seeking tendencies were affected by the patient's educational level. Moreover, the most frequently searched topics included 'diagnosis', 'tests of investigations', 'radiotherapy' and 'side effects.' In this study, participants tended to search for information at the time of diagnosis and prior to radiotherapy. The preferred sources of information were 'Nurses' (66.7%), 'Websites' (54.8%), 'Written Materials' (50%), 'General Practitioners' (50%), 'Radiographers' (47.6%) and 'Friends/Family' (47.6%). The participants who used internet as an information source searched for treatment options (54.8%), cancer diagnosis (52.4%) and possible treatment side effects (50.0%). However, 28% of the participants admitted that the information found online was difficult to understand.
**Conclusion:** This study indicated that cancer patients tend to search for more information than that provided initially.
**Limitations:** The amount of information sought throughout the treatment journey might have varied and patients could have found it difficult to recall the amount of information they sought and all the sources used.
**Ethics Committee Approval:** Ethical approval was obtained from the university research ethics committee, University of Malta.
**Funding for this study:** None
**Author Disclosures:**
Karen Borg Grima: Nothing to disclose
Gerbert van Dijk: Nothing to disclose
Clarissa Borg: Nothing to disclose

### RPS 614-2

**Management of patients with autism spectrum disorders in Swiss medical imaging departments**
*S. Carlier*[1], C. S. d. Reis[1], C. Malamateniou[2]; [1]Lausanne/CH, [2]London/UK

**Purpose:** To understand and explore the strategies currently in use to imaging patients with autism spectrum disorder (ASD) by radiographers in Swiss medical imaging departments. To develop recommendations for imaging ASD patients.
**Methods or Background:** An online questionnaire on REDCap platform to collect information on demographics, the strategies used, barriers and facilitating factors during the management of ASD patients was developed, piloted and disseminated via the Swiss Professional Body/(ASTRM). Descriptive statistics and thematic analysis were used according to the nature of the questions to analyse data.
**Results or Findings:** A hundred radiographers participated in the study but only 60 reported having managed an ASD patient. Nineteen out of 121(15.7%) radiographers declared that the condition of the patient was not communicated before the examination. The main strategy used to manage the patient was to use the help from carers who accompanied the patient (50/111; 45%). The main difficulties identified by the participants were: lack of training (35/92, 38.04%), lack of guidelines (24/92; 26.08%) and poor communication (15/92; 16.3%).
**Conclusion:** The lack of knowledge/training and the lack of local guidelines about autism seem to impact in the management of ASD patients. The caregivers were identified as playing an important role during the examination. Improvement in communication routes in clinical settings is vital for better managements and clinical services for these patients. Education about autism and local guidelines are recommended to help radiographer to manage ASD patients.
**Limitations:** The spread of the questionnaire was realised during summer holidays, which could contribute to a lower participation. The different parts of ASTRM complicated the dissemination of the questionnaire. Switzerland has three main languages however the questionnaire was developed only in French.
**Ethics Committee Approval:** Ethics approval was obtained for this study from Swiss Ethics Vaud.
**Funding for this study:** No funding.
**Author Disclosures:**
Sarah Carlier: nothing to disclose
Claudia Sa dos Reis: Nothing to disclose
Christina Malamateniou: nothing to disclose

### RPS 614-4

**Practice and safety guidelines in interventional radiology: what the radiographer should know**
*R. Gerasia*[1], A. Roletto[2], N. Giannotti[3], G. S. Gallo[1], C. Tafaro[1], A. Cozzi[2], M. Zanardo[2], S. Durante[4], D. Catania[2]; [1]Palermo/IT, [2]Milan/IT, [3]Sydney/AU, [4]Bologna/IT
*(roberta.gerasia@gmail.com)*

**Purpose:** The aim of this narrative review was to integrate major evidence about the multi-layered role of radiographers in the practice of interventional radiology (IR), summarising relevant recommendations for safe handling of X-ray equipment, radiation protection, procedural assistance, quality assurance, image and data management. We also outlined learning strategies and continuing education goals for both radiographers in training and practicing radiographers, in order to foster an ever-better integration in the IR workflow.
**Methods or Background:** No ethics committee approval was needed for this narrative review. In September 2020, an in-depth literature search (Medline/EMBASE) targeting practice guidelines for IR was performed. Recommendations were divided: before procedure; during procedure; after procedure; considerations about education.
**Results or Findings:** Nine guidelines were considered eligible. Widely recommended pre-procedural checks include the verification of patient identity and procedure site, management of relevant clinical data, and assistance to procedure planning. During the procedure, the radiographer must be aware of how the exposure settings should be tailored according to each patient's clinical and anatomical features to ensure appropriate image quality while delivering the lowest level of dose achievable. Radiation dose must be continuously monitored and patient-specific radiation dose data must be recorded in the patient's medical folder at the end of the IR procedure.
**Conclusion:** Radiographers have clearly defined and paramount roles in all steps of IR procedures, particularly providing correct management of x-ray equipment, procedural assistance, and radiation protection planning. A strong learning attitude from direct experience in the IR suite should be developed in addition to constant in-house training and advanced IR courses offered by academic institutions. A working culture that protects patient safety should be attained by multilateral integration with radiologists, medical physicists and nurses.
**Limitations:** Review not systematically conducted.
**Ethics Committee Approval:** Not applicable.
**Funding for this study:** None.
**Author Disclosures:**
Andrea Cozzi: Nothing to disclose
Diego Catania: Nothing to disclose
Nicola Giannotti: Nothing to disclose
Giuseppe Salvatore Gallo: Nothing to disclose
Stefano Durante: Nothing to disclose
Andrea Roletto: Nothing to disclose
Moreno Zanardo: Nothing to disclose
Roberta Gerasia: Nothing to disclose
Corrado Tafaro: Nothing to disclose

### Research Presentation Session: Radiographers

### RPS 714

### Quality assurance in radiography

### RPS 714-1

**Diagnostic reference levels in computed tomography examinations by clinical indication**
R. Marques[1], S. I. Rodrigues[2], A. F. L. Abrantes[2], L. P. V. Ribeiro[2], K. B. Azevedo[2], R. P. P. Almeida[2], *B. Vicente*[2]; [1]Portimao/PT, [2]Faro/PT
*(biancaicvicente@gmail.com)*

**Purpose:** The present work intends to define the Diagnostic Reference Levels (DRL) in computed tomography examinations by clinical indications, as literature suggest that these are lower than the Diagnostic Reference Levels by anatomical regions, inducing a reduced exposure in the patients submitted to this procedures.
**Methods or Background:** By registering dosimetric parameters related to CT scans of the skull, chest, spine, abdomen, pelvis, angio and uro-CT through the examination of the file dose protocol grouped into 12 different clinical indications and using a sample of 20 patients per each clinical indication. A final total of 240 examinations were considered.
**Results or Findings:** The Diagnostic Reference Levels established for the CT-Brain examination with clinical indication of trauma, stroke and vascular pathology was 884mGy.cm; 884 mGy.cm; 1772.50 mGy.cm, respectively. For the CT-Face examination (trauma) the DRL obtained was 410 mGy.cm, for the CT-Cervical, Dorsal and Lumbar Spine examination (trauma) it was 410 mGy.cm, 677 mGy.cm, 577.3 mGy.cm, respectively, for the chest examination (Pulmonary Thrombo Embolism) 383.0 mGy.cm, for the abdomen-pelvic examination (pathology of the urinary system and colon pathology) it was 1305.5 mGy.cm, 782.3 mGy.cm, respectively.
**Conclusion:** It was concluded that there was a great variation in the results obtained within the same exam, which leads to the conclusion that the DRLs should be suitable for the clinical indication and not just for an anatomical region. The obtained values were below comparing to the DRL's by anatomical regions in the following cases: CT-Brain exams (trauma and stroke), Face-CT (trauma), Cervical, Dorsal and Lumbar Spine (trauma), CT-Chest (Thrombo Embolism).
**Limitations:** Sample size.
**Ethics Committee Approval:** Ethics committee approved the study and written informed consent was delivered to the participants.
**Funding for this study:** No funding was received for this work.
**Author Disclosures:**
Kevin Barros Azevedo: Nothing to disclose
Rui Pedro Pereira Almeida: Nothing to disclose
Sónia Isabel Rodrigues: Nothing to disclose
Raquel Marques: Nothing to disclose
Antonio Fernando Lagem Abrantes: Nothing to disclose
Bianca Vicente: Nothing to disclose
Luís Pedro Vieira Ribeiro: Nothing to disclose

### RPS 714-2

**Longterm ultrasound follow-up in patients with small gallbladder polyps**
S. R. Rafaelsen, P. O. Otto, *M. R. V. Pedersen*; Vejle/DK
*(Malene.Roland.Vils.Pedersen@rsyd.dk)*

**Purpose:** The aim of the study was to examine long-term follow-up growth in gallbladder polyps <6 mm and explore the risk of developing gallbladder cancer.
**Methods or Background:** Abdominal ultrasonography reports from 2007 to 2009 were reviewed in order to include patients diagnosed with gallbladder polyps < 6 mm. The patients were invited to a final follow-up ultrasonography of the gallbladder from October 2019 to February 2020. A total of 154 patents were included ( 100 woman and 53 men).
**Results or Findings:** Gallbladder polyps were confirmed in 101 patients (65.6%) and not visible in 53 patients (34.4). A total of 49 patients had a single polyp (31.8%), and 52 (33.8%) had multiple polyps. A total of 15 patients experienced a growth of the polyps of 2 mm or more. The median gallbladder polyp size was 4.0 mm (range 2.0-5.9 mm) at baseline and median size was 4.0 mm (range 1.7-15.0 mm) during follow up. None had developed gallbladder cancer.
**Conclusion:** Gallbladder polyps <6 mm has a low probability of growth. None of the patients with small gallbladder polyps developed gallbladder cancer. The need for follow-up in patients with small gallbladder polyps is questionable.
**Limitations:** One limitation of this study is the small study size.
**Ethics Committee Approval:** The study was approved by the Institutional Review Board of the University Hospital of Southern Denmark and the local Danish data protection agency. Patients consent was mandatory.
**Funding for this study:** None
**Author Disclosures:**
Malene Roland Vils Pedersen: Nothing to disclose
Peter O. Otto: Nothing to disclose
Sören R. Rafaelsen: nothing to disclose

### RPS 714-3

**Design and development of a 3D printable holder for digital radiography detector safety**
*A. S. Karam*, E. Conroy, L. A. Rainford, E. O'Cearbhaill; Dublin/IE
*(aliaa.karam@ucdconnect.ie)*

**Purpose:** In digital radiography (DR), electronic detectors transform X-rays into digital images. These expensive detectors are highly vulnerable outside of their routine housing under the X-ray table. If damaged while in use, may require replacement at significant cost. To overcome this problem, there is a need to develop a holder to stabilize and secure the detector in a range of adjustable positions within a clinical setting. This study aims to design a novel inexpensive 3D printable based holder to fully support DR detectors when imaging using modified techniques either on the X-ray table or in a wheelchair.
**Methods or Background:** The holder was mechanically designed, modeled, and assembled using the Autodesk Inventor software. The Stress Analysis feature was employed to simulate the holder's performance with movement constraints and applied forces.
**Results or Findings:** The designed holder consists of seven parts connected with joints allowing movements in different planes. The holder's free end includes a hook and latch creating a safe grasp for the detector. Using polylactic acid (PLA), the most readily available 3D printable material, the maximum Von Mises stress, displacement, and the safety factor on the holder were 0.551 MPa, 0.147 mm, and 15, respectively.
**Conclusion:** The yield stress of PLA is greater than maximum stress generated on the holder. Maximum displacement was minor, and the safety factor was greater than one. Thus, the designed holder should be capable of fully supporting the detector without material failure, under the static loading conditions analyzed. These results provide a potential solution to reduce the risk of DR detector damage in clinical practice.
**Limitations:** Not Applicable
**Ethics Committee Approval:** Not Applicable
**Funding for this study:** The authors would like to acknowledge funding from the Health Research Board, Dublin, Ireland.
**Author Disclosures:**
Aliaa Sherif Karam: Nothing to disclose
Eoin O'Cearbhaill: Nothing to disclose
Louise A. Rainford: Nothing to disclose
Emily Conroy: Nothing to disclose

### RPS 714-4

**Differences in display size of sequential posteroanterior chest radiographs on PACS: a potential source of interpretative error**
*A. Stirling*, A. Murray, A. N. Murphy, M. Brennan, J. Murray; Dublin/IE

**Purpose:** Sequential digital radiography unit acquired (DR) posteroanterior chest radiographs (CXRs) are routinely displayed at different sizes on PACS

monitors. The purpose of this study was to determine the cause of these differences, the percentage of radiographs affected, the relative change in magnification seen and if radiologists were aware of this display difference.
**Methods or Background:** Differences in routine radiographer collimation pre-acquisition and image cropping (shuttering) post acquisition were noted. From 3 different centres, 300 posteroanterior (PA) erect CXRs with prior comparative studies were viewed side-by-side on a standard landscape display monitor. Variation in display size (i.e. the extent of magnification) was calculated using the number of detector elements (Dels) in the autofitted axis of the radiograph, when compared with the prior study. Correlation between age, sex and extent of magnification between images was recorded. Following this, a national survey was circulated to see if radiologists were aware of this phenomenon.
**Results or Findings:** Large variations in display size were noted. The mean extent of magnification between sequential PA chest radiographs was ±6.8% (range 0 – 21.6%) when viewed side-by-side. 98% of CXRs had some degree of variability in display size. There was no significant difference in the extent of variation in magnification based on age or gender. 86% of the radiologists who responded to the survey (n=132) were unaware of any display size variability.
**Conclusion:** Sequential DR acquired chest radiographs are routinely displayed with varying degrees of magnification on PACS monitors due to differences in radiographer practice and auto-fit display settings. Most radiologists are unaware of these differences which could lead to perceptive error.
**Limitations:** Possible response bias in survey
**Ethics Committee Approval:** Yes
**Funding for this study:** None to declare
**Author Disclosures:**
Michael Brennan: Nothing to disclose
John Murray: Nothing to disclose
Aoife Murray: Nothing to disclose
Aaron Stirling: Nothing to disclose
Alexandra N Murphy: Nothing to disclose

### RPS 714-6

**Quality of life and quality of work-life among radiographers: a comparative study between Portugal and England**

*I. Pinto*[1], L. P. V. Ribeiro[2], S. I. Rodrigues[2], B. Vicente[2], A. F. L. Abrantes[2], R. P. P. Almeida[2]; [1]Loulé/PT, [2]Faro/PT
*(inespinto54@gmail.com)*

**Purpose:** Nowadays, Quality of Life (QL),as well as Quality of Work Life (QWL), represent an important factor in any human being. Physical and psychological well-being, environmental factors, personal relationships and career factors influence a person's life and, when positives, provide a greater personal achievement. The purpose of this study is to analyze QL and QWL among radiographers and to compare the results between these professionals in two different countries.
**Methods or Background:** A total of 83 radiographers participated, of which 51 worked in Portugal and 32 in England. For the accomplishment of this study two online questionnaires were applied, one to assess QL and another concerning QWL. Both combined allowed the assessment of individuals general QL.
**Results or Findings:** Despite the sample's homogeneity, some differences were observed between radiographers of the two countries.The differences found are mostly work-related questions, in which radiographers from England presented more satisfactory response averages than Portuguese radiographers concerning, respectively, the ability for innovation (3,78 vs 3,08),staff equality (3,53 vs 3,04),professional fulfilment (3,72 vs 3,37),satisfaction in QWL (3,25 vs 3,78),among others. In addition,significant differences were found between radiographers from both countries concerning personal relationships dimension (p=0,001).It was found positive correlations between QL and QWL, mostly in environmental (r=0,439;p=0,000), physical (r=0,266;p=0,017) and psychological (r=0,255;p=0,023) factors.
**Conclusion:** It was possible to verify that radiographers from Portugal present a greater QL unlike England, which presents a better QWL of its professionals. This study may lead to an awareness and to an effort to improve radiographer's QL and QWL in order to achieve a greater personal satisfaction and, consequently,a better performance of their professional practice.
**Limitations:** Sample size
**Ethics Committee Approval:** Ethics committee approved the study and written informed consent was delivered to the participants.
**Funding for this study:** No funding was received for this work.
**Author Disclosures:**
Rui Pedro Pereira Almeida: Nothing to disclose
Bianca Vicente: Nothing to disclose
Sónia Isabel Rodrigues: Nothing to disclose
Antonio Fernando Lagem Abrantes: Nothing to disclose
Inês Pinto: Nothing to disclose
Luís Pedro Vieira Ribeiro: Nothing to disclose

### Research Presentation Session: Vascular

### RPS 315

### Vascular ultrasound and MR

### RPS 315-1

**Ultrasonography for the diagnosis of extra-cranial carotid occlusion: diagnostic test accuracy meta-analysis**

*D. M. Rojoa*[1], A. Lodhi[1], F. J. Raheman[2], N. Kontopodis[3], C. Ioannou[3], N. Labropoulos[4], G. Antoniou[1]; [1]Manchester/UK, [2]Leicester/UK, [3]Heraklion/GR, [4]New York, NY/US
*(Djamila.rojoa@doctors.org.uk)*

**Purpose:** The correct diagnosis of internal carotid artery (ICA) occlusion is crucial as it limits unnecessary intervention, whereas correct identification of patients with severe ICA stenosis is paramount in decision making and selecting patients who would benefit from intervention. We aimed to evaluate the accuracy of ultrasonography (US) in the diagnosis of ICA occlusion.
**Methods or Background:** We conducted a systematic review in compliance with the Preferred Reporting Items for a Systematic Review and Meta-analysis (PRISMA) of diagnostic test accuracy studies. We interrogated electronic bibliographic sources using a combination of terms to identify studies assessing the diagnostic accuracy of US in ICA occlusion. We used a mixed-effects logistic regression bivariate model to estimate summary sensitivity and specificity and developed hierarchical summary receiver operating characteristic (HSROC) curves.
**Results or Findings:** We identified 23 studies reporting a total of 5,675 arteries of which 722 were proven to be occluded. The reference standard was digital subtraction or cerebral angiography in all but two studies, which used surgery to ascertain a carotid occlusion. The pooled estimates for sensitivity and specificity were 0.97 (95% confidence interval (CI) 0.94 to 0.99) and 0.99 (95% CI 0.98 to 1.00), respectively. The diagnostic odds ratio was 3,846.15 (95% CI 1,375.74 to 10,752.65). The positive and negative likelihood ratio were 114.71 (95% CI 58.84 to 223.63) and 0.03 (95% CI 0.01 to 0.06), respectively.
**Conclusion:** US is a reliable and accurate method in diagnosing ICA occlusion. US can be used as a screening tool with cross-sectional imaging being reserved for ambiguous cases.
**Limitations:** The fact that there have been multiple advances in technology since some of the old papers were written means that the diagnostic errors due to technological limitations are now significantly reduced.
**Ethics Committee Approval:** None
**Funding for this study:** None
**Author Disclosures:**
Ahmad Lodhi: Nothing to disclose
George Antoniou: Nothing to disclose
Nicos Labropoulos: Nothing to disclose
Djamila Mubashchira Rojoa: Nothing to disclose
Christos Ioannou: Nothing to disclose
Firas J Raheman: Nothing to disclose
Nikos Kontopodis: Nothing to disclose

### RPS 315-2

**Existence of helical flow of superior vena cava in healthy young males: a 4D flow MRI study**

*H. Pu*; Chengdu/CN
*(phxia0409@126.com)*

**Purpose:** Helical flow is considered to be of positive significance to human blood circulation, but its existence in superior vena cava (SVC) has not been well confirmed in vivo. This study aimed to investigate whether helical flow exists in SVC of healthy young males, and to describe the development and characteristics of this flow pattern with 4D flow magnetic resonance imaging (MRI).
**Methods or Background:** 4D flow MRI data with full coverage of SVC and brachiocephalic veins (BVs) were acquired with a 3-Testa MRI in 8 young male volunteers. Hemodynamic parameters in SVC including velocity, velocity vector, pathline, streamline and flow waveforms were obtained with a specialized commercial post-processing software based on the 4D flow MRI data.
**Results or Findings:** This study found that: 1) the helical flow appeared in diastole of cardiac cycle and mainly located at the upper and middle segments of SVC; 2) with the decrease of blood velocity, the helical flow area had the tendency of gradual extension; 3)the flow pathline of left and right BVs formed helical flows respectively, and there were two development types: twining and untwining; 4)the flow waveform of the SVC presented as a double peak shape, some cases have a centrifugal blood flow during diastole.

**Conclusion:** Helical flow does exist in SVC in healthy young males. It seems to have the physiological function of ensuring the venous return rate and preventing venous thrombus. This finding might help to explain the occurrence and development of potential SVC disease, such as thrombosis.
**Limitations:** Single-center research and small sample may cause sampling error.
**Ethics Committee Approval:** The Ethical Review Committee of the West China Hospital of Sichuan University (Chengdu, Sichuan, China) approved this research.
**Funding for this study:** the National Natural Science Foundation of China [grant number 81601462], et al.
**Author Disclosures:**
MA Huaxia Pu: Nothing to disclose

### RPS 315-3
**Two dimensional vessel wall diffusion anisotropy detects age related alterations of diffusivity in human carotids**
*S. L. Leber*, E. M. Hassler, F. Gunzer, G. Silbernagel, T. Metzner, H. Deutschmann, G. Reishofer; Graz/AT
*(stefan.leber@medunigraz.at)*

**Purpose:** The application of magnetic resonance (MR) diffusion tensor imaging on carotid arteries is a relatively new, non-invasive method to monitor arterial health and disease progression. High resolution diffusion weighted imaging (DWI) in combination with a two-dimensional (2D) diffusion gradient sampling scheme has recently been demonstrated as feasible for evaluating fractional anisotropy (FA) in the vessel wall of human carotids. The aim of our study was to detect age related alterations of diffusivity in human carotids in a clinical context.
**Methods or Background:** We used DWI in combination with a 2D gradient at 3T to evaluate FA of carotid vessel walls in older patients (mean age 65.2 years) and compare them to a healthy control group of younger patients (mean age 47.6 years). DWI was carried out using a read-out segmented EPI (rs-EPI) sequence.
**Results or Findings:** Our group comparison revealed significantly decreased fractal anisotropy values in carotids of older patients compared to healthy controls ($p< 0.05$).
**Conclusion:** Our work demonstrates that FA in human carotid vessel walls changes with age and support the hypothesis that 2D vessel wall diffusion anisotropy is feasible for detecting age related architectural changes in blood vessels and monitor arterial health. Furthermore we show the applicability of 2D vessel wall diffusion anisotropy in a clinical context.
**Limitations:** Since group size was limited in our study, our findings need to be validated on a larger independent cohort.
**Ethics Committee Approval:** The study was approved by the ethics committee of the Medical University of Graz.
**Funding for this study:** No funding was received.
**Author Disclosures:**
Eva Maria Hassler: Nothing to disclose
Hannes Deutschmann: Nothing to disclose
Stefan L. Leber: Nothing to disclose
Günther Silbernagel: Nothing to disclose
Felix Gunzer: Nothing to disclose
Gernot Reishofer: Nothing to disclose
Thomas Metzner: Nothing to disclose

### RPS 315-4
**Transferring catheter interventions into the MR environment**
*C. M. Reich*[1], S. Schauer[2], M. Schmid[2], K. Höger[3], A. Hegel[3], F. Cranz[4], M. Azhar[5], M. Steinmetz[5], A. Melzer[1]; [1]Leipzig/DE, [2]Dettingen an der Erms/DE, [3]Pforzheim/DE, [4]Wessling/DE, [5]Göttingen/DE
*(ChristophMartin.Reich@medizin.uni-leipzig.de)*

**Purpose:** Over the last years, researchers and clinicians have successfully shown the advantage of MRI-guidance for multiple procedures. Our research group takes part in joint research and development projects focussing on catheter-based interventions using MRI-guidance instead of the currently used x-ray fluoroscopy.
**Methods or Background:** The development process to realize MRI-guided vascular interventions starts with MRI compatible instruments. The paradigm shift from MRI as a diagnostic to a therapeutic imaging modality makes technological adaptions necessary. Taken the aforementioned issues together, a need is generated for a workflow for MRI-guided interventions.
**Results or Findings:** Novel instrument designs and MRI marker technology are developed and first prototypes are being tested. Besides passive and semi-active markers, we aim for semi-active instruments. A technical setup is developed to support interventionalists during the procedure in a way they are accustomed to from the catheter laboratory. Our approach is to use everyday technology to establish an interventional MRI suite that can be operated by minimally trained staff. A clinical workflow was compiled, taking into consideration MR Safety aspects, effective handling of the MRI scanner and predefined sequences for the desired use case. Among other aspects, this is supposed to provide guidance for the clinical personnel.
**Conclusion:** The three milestones: MRI compatible instrument, clinical workflow, and technical setup were accomplished in a preliminary project. The applicability and usability of each aspect need to be proven and evaluated in in-vitro/ex-vivo trials. Afterwards, necessary adaptations can be made before animal studies will be carried out.
**Limitations:** To assure MR Safety, further investigations in conformity with standards have to be carried out.
**Ethics Committee Approval:** No approval required
**Funding for this study:** German Federal Ministry of Education and Research, „KMU-innovativ: Medizintechnik", contract numbers 13GW0242B, 13GW0288D; "Bildgeführte Diagnostik und Therapie – Neue Wege in der Intervention", 13GW0365C
**Author Disclosures:**
Felix Cranz: Employee: Bavaria Medizin Technologie GmbH
Senta Schauer: Employee: EPflex Feinwerktechnik GmbH
Andreas Melzer: Nothing to disclose
Michael Steinmetz: Other: DZHK (German Center for Cardiovascular Research), partnersite Goettingen
Alexander Hegel: Employee: ADMEDES GmbH
Mueed Azhar: Other: DZHK (German Center for Cardiovascular Research), partnersite Goettingen
C. Martin Reich: Nothing to disclose
Michael Schmid: Employee: EPflex Feinwerktechnik GmbH
Kim Höger: Employee: ADMEDES GmbH

### RPS 315-5
**One stop giant cell arteritis: the Oxford experience of GCA sonographer development during the COVID-19 pandemic crisis**
*G. Sallemi*, S. Dubey, M. Adamson, J. Thorne, J. P. E. White, K. Partington, R. A. Luqmani; Oxford/UK
*(germana.sallemi@ouh.nhs.uk)*

**Purpose:** Demonstrate the presence of the “halo” sign in ultrasound patients with suspected Giant Cell Arteritis (GCA), confirming diagnosis due to a daily pandemic clinic.
**Methods or Background:** Prior pandemic, our GCA ultrasound performed twice week clinic/scanning assessing 15 patients/session, including newly suspected and follow-up patients. Patients required 20 minute-slot, resulting in late clinics and delays in establishing new patient’s timely diagnosis. The risks of high dose steroid during the pandemic and early diagnosis was imperative due to diagnostic uncertainty if partially pre-treated. We reviewed records from patients between November 2019 to July 2020, including 17 weeks seen ‘pre-COVID’ and 17 weeks of 5 days per week service, termed ‘intra-COVID’. All patients underwent clinical evaluation and ultrasound in longitudinal and transverse plane: axillary, common, parietal and frontal arteries evaluated as our institutional protocol. Those hypoechoic, non-compressible peri-luminal arterial wall-thickening (or halo sign) were considered ultrasound ‘positive’; increasingly used as diagnostic standard.
**Results or Findings:** 139 patients with suspected GCA, 63 pre-COVID period vs 76 intra-COVID were reviewed. Diagnosis of GCA made in 33.3% pre-COVID and 42% intra-COVID ($p=0.289$). Ultrasound sensitivity improved from 38.1% to 68.6% ($p<0.0001$). Proportion of patients diagnosed within 7days of referral improved 61.6% ($p<0.0001$), from 31.7% to 93.3%. Median number of days from referral to assessment fell over four-fold, from 12.5 to 3. Median number of days steroid exposure fell over seven-fold from 15 days to 2 days.
**Conclusion:** The pandemic led to the development of a sonographer daily ultrasound/clinic one-stop service for patients with suspected GCA or with a flare of symptoms, achieving an efficient system with regards to patient pathway, diagnosis and earlier treatment.
This model is used as our institution’s gold standard of care planning emerging from the pandemic.
**Limitations:** None
**Ethics Committee Approval:** None
**Funding for this study:** None
**Author Disclosures:**
Matthew Adamson: Nothing to disclose
Raashid A Luqmani: Nothing to disclose
Jonathan P E White: Nothing to disclose
Jessica Thorne: Nothing to disclose
Germana Sallemi: Nothing to disclose
Shirish Dubey: Nothing to disclose
Karen Partington: Nothing to disclose

### Research Presentation Session: Vascular

### RPS 415

### Vascular CT and angiography

### RPS 415-1

**Feasibility of ultra-low dose contrast media in pancreatic arteries depiction using the combination of advanced virtual monoenergetic imaging and high-concentration contrast medium**

*J. Li*[1], X. Chen[2], H. Xue[1], J. Zhengyu[1]; [1]Beijing/CN, [2]Chengdu/CN
*(mzy_lyy@163.com)*

**Purpose:** To evaluate the feasibility of ultra-low dose (224mgI/kg) contrast media (CM) in the depiction of pancreatic arteries, by using the combination of advanced virtual monoenergetic imaging (VMI+) and high-concentration (400mgI/ml) CM.
**Methods or Background:** From April 2019 to May 2020, 41 patients (26 men, 15 women; mean age, 56.0 years), who underwent both normal dose CM (ND-CM, 320mgI/kg) and low dose CM (LD-CM, 224mgI/kg) thoracoabdominal enhanced CT (with or without pelvic CT) for tumor follow-up, were prospectively included. The VMI+ at the energy level of 40-kev for the LD-CM images was reconstructed. The CT attenuation, signal-to-noise ratios (SNRs) and contrast-to-noise ratios (CNRs) of abdominal artery, celiac artery and superior mesenteric artery (SMA) as well as the depiction of pancreatic arteries were recorded and compared between the ND-CM images and LD-CM images, the ND-CM images and VMI+ of the LD-CM images respectively. Paired t-test, Wilcoxon-signed rank test and McNemar's test were used for analysis.
**Results or Findings:** Comparing to the ND-CM images, SNRs of aorta and celiac artery in VMI+ LD-CM images were significantly lower (all $p < 0.05$), but the mean CT value and CNRs in VMI+ LD-CM images is significantly higher (all $p < 0.001$). There was no significant difference of SNR of SMA between ND-CM images and VMI+ LD-CM images (p=0.223). There were no significant differences of qualitative scores of all pancreatic arteries between the ND-CM images and VMI+ LD-CM images in the (all p> 0.05).
**Conclusion:** Ultra-low dose CM (224mgI/kg) could be used in the depiction of pancreatic arteries, by using the combination of VMI+ and high-concentration (400mgI/ml) CM.
**Limitations:** Firstly, the duration between two examinations is long; Secondly, we didn't use constant injection duration; Finally, we didn't further group by BMI.
**Ethics Committee Approval:** Yes
**Funding for this study:** Na
**Author Disclosures:**
Jin Zhengyu: Nothing to disclose
Juan Li: Nothing to disclose
Xinyue Chen: Nothing to disclose
Huadan Xue: Nothing to disclose

### RPS 415-2

**Impact of false lumen outflow variability on adverse event risk stratification in patient with uncomplicated type B aortic dissection**

*M. Codari*, M. J. Willemink, D. Mastrodicasa, M. Madani, G. Mistelbauer, V. Turner, V. Hinostroza, M. Fischbein, D. Fleischmann; Stanford, CA/US
*(mcodari@stanford.edu)*

**Purpose:** False lumen outflow (FLO) has emerged as an independent imaging predictor for late adverse events (LAE) in patients with initially uncomplicated Stanford type B aortic dissection (uTBAD). Before it can be clinically used, reproducibility and subsequent effects on risk stratification should be evaluated. We investigated the impact of inter-observer variability of visually assessed aortic branches drainage patterns and derived FLO values on LAE risk stratification.
**Methods or Background:** We selected 40 patients who underwent CTA for acute uTBAD. Left subclavian, visceral, renal, and iliac arteries were visually classified as arising from the true lumen, false lumen, or both (defining their drainage pattern). Then, we derived FLO values and resulting 1-year, 2-years, and 5-years LAE probabilities. Assessments were done twice by independent observers. Agreement was assessed using Cohen's kappa and Bland-Altman analysis. Wilcoxon tests were used to compare paired samples. Finally, we classified patients according to derived risk scores and evaluated number of reclassifications.
**Results or Findings:** Agreement varied across aortic branches with a median kappa (interquartile range) of 0.54 (0.23–0.61). FLO differed significantly among observers: 638.8 (434.4–837.5) vs. 828.8 (633.1–1268.8) mL/min, p<0.001. Resulting LAE probabilities varied significantly: 1-year probability 7.4% (5.1–15.0%) vs. 5.6% (3.6–15.0%), p<0.001; 2-years probability 13.6% (9.5–26.6%) vs. 10.5% (6.6–26.6%), p=0.001; 5-years probability 28.1% (20.2–50.2%) vs. 22.0% (14.4–50.2%), p=0.001, respectively. Bland-Altman analyses showed suboptimal agreement. As the results of different FLO assessments, 8/40 (20%) patients were reclassified to a different risk class.
**Conclusion:** Inter-observer variability of FLO significantly impacts risk stratification in uTBAD patients. Computer-aided solutions may allow to improve its reproducibility.
**Limitations:** Single center, retrospective.
**Ethics Committee Approval:** This study was approved by our institutional ethical research board (IRB-51630). Patient informed consent was waived due to the retrospective design of the study.
**Funding for this study:** AHA grant (18POST34030192), NIBIB grant (5T32EB009035).
**Author Disclosures:**
Dominik Fleischmann: Nothing to disclose
Virginia Hinostroza: Nothing to disclose
Michael Fischbein: Nothing to disclose
Marina Codari: Nothing to disclose
Gabriel Mistelbauer: Nothing to disclose
Valery Turner: Nothing to disclose
Martin J. Willemink: Founder: Segmed, Inc. Grant Recipient: American Heart Association
Mohammad Madani: Nothing to disclose
Domenico Mastrodicasa: Grant Recipient: National Institute of Biomedical Imaging and Bioengineering

### RPS 415-3

**Extracorporeal membrane oxygenation (ECMO) 'return' cannulae: now you see them, now you don't!**

*J. K. Ruffle*, K. Pinto, J. Doyle, C. Russell, C. Bleakley, S. Ledot, S. R. Desai; London/UK

**Purpose:** Extracorporeal membrane oxygenation (ECMO) is a proven technique for supporting patients with severe acute respiratory failure (SARF). However, iatrogenic complications are recognised and may be problematic. Among recognised adverse effects is the mal-positioning of ECMO cannulae and confirming satisfactory positioning — in particular, of the radiolucent tip of the return cannula (RC) — with computed tomography (CT) is important. We investigated the utility of CT in accurately identifying ECMO RC positioning.
**Methods or Background:** Retrospective review of CT scans in consecutive ECMO patients treated between January 2019 and September 2020. On reconstructed contrast-enhanced coronal CT images, where visible, two observers independently recorded i) the distance from the tip of RC to the cavo-atrial junction (CAJ) and, where appropriate, ii) the proximity of the RC to the inter-atrial septum (IAS). In those without a visible RC tip, a 4 cm length from the radio-opaque end was assumed.
**Results or Findings:** The tip of the RC was visible in 32/50 (64%) patients; measurement agreement for RC positioning was excellent (r=0.93, p<0.0001). The median distance of the RC from the CAJ was -0.90cm (interquartile range (IQR), -2.05cm — +0.94cm). In 17/50 (34%) patients, the cannula tip was a median distance 1.4 cm (IQR 1.01-2.40cm) beyond the CAJ. Of these, 13/17 (76%) RC - sited in the right atrium - were adjacent to the IAS, whereas 4/17 (23%) were in the SVC.
**Conclusion:** The radiolucent tip of the ECMO RC was not visible on CT in nearly one-third and, importantly, of those in the right atrium, the majority were adjacent to the IAS. This has important practical implications for radiologists and intensivists managing patients with SARF.
**Limitations:** Future study should relate these findings to complementary imaging modalities.
**Ethics Committee Approval:** Not applicable.
**Funding for this study:** Not applicable.
**Author Disclosures:**
Caroline Bleakley: Nothing to disclose
Stephane Ledot: Nothing to disclose
Clare Russell: Nothing to disclose
James K. Ruffle: Nothing to disclose
James Doyle: Nothing to disclose
Karen Pinto: Nothing to disclose
Sujal R. Desai: Nothing to disclose

### RPS 415-4

**Is the undetermined stroke genesis really unknown? - a retrospective study**

*M. V. M. Micelli*, U. Russo, E. G. Puglielli, N. Caputo; Teramo/IT
*(micelli.mvm@gmail.com)*

**Purpose:** Carotid web (CW) is a rare entity characterized by a nonatherosclerotic fibrous band of the internal carotid artery (ICA). The overall prevalence of CW is 1%, with an uncertain clinical effect. The aim of the study is to evaluated the correlation between CWs incidence and large vessel occlusion ischemic strokes (LVOIS).
**Methods or Background:** All consecutive LVOIS-patients treated by endovascular trombectomy performed at our Interventional Radiology Division, were enrolled in the study. Site of occlusion, risk factors, CTA of the neck were retrospectively evaluated per each patient. Moreover, strokes were classified in 5 subtypes using the TOAST classification ( Trial of Org 10172 in Acute Stroke Treatment classification). Exclusion criteria were posterior-strokes, known causes of ischemic stroke, absence of neck-imaging.
**Results or Findings:** During the last 3 years, 85 LVOIS-patients were eligible for endovascular trombectomy procedures. 11 of 85 cases didn't performed imaging of neck and were excluded from the study. 63 of 74 (85.1%) patients presented an anterior-LVOIS and 13 of them (20.6%) were classified as undetermined strokes using TOAST classification. CWs were retrospectively observed in 6 of 63 cases. 5 of 6 CWs were ipsilateral to the anterior-LVOIS and they occurred in 2 men and 3 women (mean age of 66 ± 7years), with no smoking history, a BMI<32, no other arteriopathies at the imaging, no apparent risk factors.
**Conclusion:** CWs prevalence in our anterior-LVOIS population and in our undetermined anterior-LVOIS patients were respectively 9.5% and 38.4%. CWs diagnosis were easily dismissed but it could be an underappreciated risk factor for ischemic strokes.
**Limitations:** Our data is referring to a quite small sample but we are enrolling more patients.
**Ethics Committee Approval:** No necessary for this retrospective study.
**Funding for this study:** No funding to declare.
**Author Disclosures:**
Umberto Russo: Nothing to disclose
Maria Valeria Marcella Micelli: nothing to disclose
Nevia Caputo: Nothing to disclose
Edoardo Gaetano Puglielli: Nothing to disclose

### RPS 415-5

**Inter-observer analysis of morphologic risk predictors in aortic dissection**

M. J. Willemink, *D. Mastrodicasa*, M. Madani, G. Mistelbauer, A. M. H. Sailer, V. Turner, V. Hinostroza, M. Codari, D. Fleischmann; Stanford, CA/US
*(domenico.mastrodicasa@gmail.com)*

**Purpose:** Morphologic features such as (i)maximum aortic diameter, (ii)relative false-lumen circumference, (iii)false-lumen drainage, and (iv)number of identifiable intercostal arteries, have recently emerged as independent predictors of late complications in patients with uncomplicated type-B aortic dissection (uTBAD). We sought to determine the inter-observer variability of these manually extracted features (i-iv), which is a prerequisite for incorporation into a clinical risk prediction model.
**Methods or Background:** Forty-seven patients who underwent CTA for acute uTBAD were included. Each CT-scan was evaluated twice by different observers. (i)Maximum aortic diameter(mm) was measured orthogonal to the aortic centerline; (ii)relative false-lumen circumference (angular degrees) was measured in the same plane; (iii)false-lumen drainage (mL/min) was measured as the sum of estimated branch vessel blood flow arising off the false-lumen, and (iv)intercostals (n) at the dissection level were counted. Differences between measurements were compared with paired t-tests. Reliability was evaluated with intraclass correlation coefficients (ICCs) and agreement with Bland-Altman plots.
**Results or Findings:** Repeated measurements of maximum aortic diameter 37.1±5.6 mm vs. 35.8±5.0 mm (3.5%,p=0.047) were significant but small, while other imaging features differed more substantially: relative circumference of false lumen 242.2±29.1° vs. 256.3±30.2° (5.8%,p=0.004), false lumen drainage 904.9±459.6 mL/min vs. 714.6±372.1 mL/min (21.0%,p<0.001), and intercostal arteries n=15.4±3.5 vs. n=12.9±3.6 (16.2%,p<0.001). Inter-observer reliability was poor to moderate with ICCs of 0.69(0.51-0.82) for maximum aortic diameter, 0.43(0.16-0.64) for relative false-lumen circumference, 0.71(0.52-0.84) for false-lumen drainage, and 0.52(0.27-0.70) for number of intercostals. Bland-Altman analyses indicated suboptimal agreement with a non-zero bias for all four features and relatively wide 95%-confidence intervals.
**Conclusion:** With the exception of maximum aortic diameter, the reproducibility of emerging imaging features is limited. New tools supporting manual feature-extraction or the development of new, reproducible automated methods are needed.
**Limitations:** Retrospective study
**Ethics Committee Approval:** IRB-approved
**Funding for this study:** American Heart Association
**Author Disclosures:**
Dominik Fleischmann: Research/Grant Support: Siemens Healthineers
Virginia Hinostroza: Nothing to disclose
Marina Codari: Nothing to disclose
Gabriel Mistelbauer: Research/Grant Support: cool IT GmbH
Valery Turner: Shareholder: Segmed, Inc
Anna M. H. Sailer: Nothing to disclose
Martin J. Willemink: Grant Recipient: Philips Healthcare Consultant: Arterys, Inc Shareholder: Segmed, Inc
Mohammad Madani: Nothing to disclose
Domenico Mastrodicasa: Nothing to disclose

### RPS 415-6

**Automatic assessment of morphologic risk predictors in aortic dissection: a reliability study**

*J. Hop*, G. Mistelbauer, L. Hahn, M. Codari, D. Mastrodicasa, M. Madani, D. Fleischmann, M. J. Willemink; Stanford, CA/US
*(j.f.hop@umcg.nl)*

**Purpose:** Recent studies have shown that morphologic features such as maximum aortic diameter and false-lumen arc length are independently associated with late adverse-events in uncomplicated type-B aortic dissection (uTBAD) patients. We developed an automatic method to assess these morphologic features on CTA-images. Our aim was to evaluate the reliability of these automatically-derived measurements.
**Methods or Background:** Baseline CT-scans of 36 uTBAD-patients were retrospectively evaluated. We developed a method to identify the centerline and create multiplanar-reformations. For the current study, the centerline was determined based on ground-truth segmentations. We automatically assessed the orthogonal plane with the maximum aortic diameter. In this plane, we estimated the maximum aortic diameter by fitting an ellipse to the aortic boundary and false-lumen arc length by describing the arc's contour using a Freeman chain-code. To validate this approach, an observer manually measured the maximum aortic diameter and false-lumen arc length using iNtuition-software. Automatic and manual measurements were compared using paired t-tests. Reliability was evaluated with intraclass correlation-coefficients (ICCs) and agreement with Bland-Altman plots.
**Results or Findings:** The automatically-derived mean maximum aortic diameter (39.4±5.5mm) was slightly larger than the manual diameter (37.9±6.6mm,p=0.001). The automatically derived mean false-lumen arc length (80.8±18.3mm) was similar to the manual measurement (79.0±18.2 mm,p=0.374). Inter-method reliability was good to excellent with ICCs of 0.91(0.82-0.95) for maximum aortic diameter and 0.79(0.62-0.89) for false-lumen arc length. Bland-Altman analyses indicated a small mean difference of 1.5±2.6mm (4.8±6.8%) and a 95%-confidence interval of -3.6–6.7mm for maximum aortic diameter. Similarly, we found a small mean difference of 1.8±11.9mm (3.3±16.6%) and 95%-confidence interval of -21.6–25.1mm for false-lumen arc length.
**Conclusion:** Our fully automatic method allowed for reliable measurements of the maximum aortic diameter and false-lumen arc length on CT-images of uTBAD-patients.
**Limitations:** Retrospective study
**Ethics Committee Approval:** IRB-approved
**Funding for this study:** American Heart Association
**Author Disclosures:**
Dominik Fleischmann: Research/Grant Support: Siemens Healthineers
Lewis Hahn: Consultant: Arterys, Inc
Marina Codari: Nothing to disclose
Gabriel Mistelbauer: Research/Grant Support: cool IT GmbH
Martin J. Willemink: Consultant: Arterys, Inc Grant Recipient: Philips Healthcare Shareholder: Segmed, Inc
Mohammad Madani: Nothing to disclose
Domenico Mastrodicasa: Nothing to disclose
Joost Frederik Hop: Nothing to disclose

### RPS 415-7

**Evaluation of hybrid technique (three-phase scintigraphy with CT angiography) for prediction of treatment outcomes in patients with acute and chronic critical lower limb ischaemia**

*O. Leshchinskaya*, N. Kudryashova, I. Mikhailov, O. Lbova, B. Kozlovskiy, A. Panyukova, L. Kokov; Moscow/RU
*(o.leshchinskay@icloud.com)*

**Purpose:** Main predictors of limb salvage with open or endovascular interventions are level of arterial occlusion, degree of ischemia and severity of microcirculatory disturbance. Our aim was to predict the outcomes in before and following intervention using hybrid technique.
**Methods or Background:** Between 2018-2020 113 patients with lower limb ischemia 106/113 acute (ALI) and 7/113 chronic (CCLI) have been examined using Hybrid technique. We used GE, Discovery 670 NM/CT scanner. For

Scintigraphy we used 99mTc-pyrophosphate (500MBq, 2.85mSv) or 99mTc-MIBI (500MBq, 4.5mSv). 31/113 (27%) were referred to surgery and follow-up Hybrid examinations performed in 17/31 (55%) of patients a week after intervention due to undetermined clinical symptoms.
**Results or Findings:** In 12/17 (71%) of patients at follow-up studies CTA demonstrated restoration of the blood flow and scintigraphy shown increase of tissue blood-flow in the zones of hypoperfusion, regression of infiltrative-necrotic changes and positive dynamics of calculated indices. 5/17 (29%) had no evidence of revascularization or aggravation of ischemia. 3/5 patients had necrosis and reperfusion syndrome with signs of nonviable tissue on scintigraphy, which led to amputations. Other 2/5 patients had persistent ischemia without positive dynamics on scintigraphy and medical treatment was initiated. 99mTc-pyrophosphate studies had an advantage due to the ability to identify foci of aseptic necrosis.
**Conclusion:** Our initial results indicate the reliability of the hybrid technique in diagnosis of the severity of ischemic changes in ALI and CCLI thus predicting the outcomes prior to and following the intervention. Hybrid technique characterizes both tissue viability and the state of the arterial vessels to choose the method of revascularization, along with predicting probability of limb salvage and/or reducing a level of amputation.
**Limitations:** Relatively small number of patients
**Ethics Committee Approval:** Yes
**Funding for this study:** Non
**Author Disclosures:**
Olga Valerievna Leshchinskaya: Nothing to disclose
Alexandra Panyukova: Nothing to disclose
Igor Mikhailov: Nothing to disclose
Natalia Kudryashova: Nothing to disclose
Leonid Kokov: Nothing to disclose
Boris Kozlovskiy: Nothing to disclose
Olga Lbova: Nothing to disclose

### RPS 415-8
**Stenting of the left renal vein**
*O. B. Zhukov*; Moscow/RU
*(ob.zhukov@yandex.ru)*

**Purpose:** We are presents the results left renal vein stenting in 35 patients with proven hemodynamically significant compression of the vein in "aorto-mesenteric tweezers".
**Methods or Background:** Men - 7 aged 39 - 54 years (average 44.3 years), women - 28 aged 31 - 64 years (average 34.7 years). All patients were examined according to the accepted algorithm: USS, CT and MRA. MRI. All were examined by a urologist and/or a gynecologist. Direct contrast phlebography (PCF) in the overwhelming majority of cases (33–94%) preceded the decision to perform stenting of the LPV. In two cases (6%), direct renal pdebography ended with LPV stenting. The first stenting of the left renal vein was performed on February 24, 2003.
**Results or Findings:** In the overwhelming majority of cases (4 men and 26 women), patients underwent simultaneous operations, including stenting of the left renal vein and spiral embolization or sclerotherapy (often in combination) of testicular, ovarian veins. In 4 cases, in women, at different times (from 6 months to 1 year) prior to stenting of LPV, endovascular interventions were performed for May-Turner syndrome. The stenting of the left common iliac vein was performed. In 5 cases (3 men, 2 women), only stenting of the left renal vein was performed. In all cases, technical success was achieved
**Conclusion:** LPV stenting is an effective and safe method of treatment of compression syndrome accompanying venous hypervolemia, which has many clinical manifestations. Currently, pelvialgia, relapse of varicocele or ovariko-varix, hematuria, progressive pelvic varicose veins in the form of pathological venous drainage of the cavernous bodies of the penis, progression of external or internal hemorrhoids are indications for in-depth follow-up of these patients and possible subsequent stenting of LPV.
**Limitations:** n/a
**Ethics Committee Approval:** n/a
**Funding for this study:** n/a
**Author Disclosures:**
Oleg B. Zhukov: Nothing to disclose

### RPS 415-9
**Preoperative aortic geometry in predicting perioperative peri-stent false lumen patency for type B aortic dissection after thoracic endovascular repair**
*H. Pu*; Chengdu/CN
*(phxia0409@126.com)*

**Purpose:** The study aimed to investigate whether pre-operative three-dimensional aortic geometric parameters can predict the perioperative peri-stent false lumen patency (FLP) in patients with Type B aortic dissection (TBAD) after thoracic endovascular aortic repair (TEVAR).
**Methods or Background:** We retrospectively collected the pre- and post-operative imaging data of patients with TBAD who underwent TEVAR. According to the false lumen (FL) status confirmed by follow-up computed tomography angiography (CTA) at 1 week after TEVAR, 51 patients with peri-stent FLP were enrolled as group-FLP, while 43 patients with peri-stent FL obliteration (FLO) were enrolled as control (group-FLO). Pre-operative aortic geometry parameters, including diameters, area and volume of true lumen (TL) and FL, intimal tear number, branch involvement, aortic arch angulation, et al. Logistic regression was employed to evaluate parameters which can predict the risk of perioperative peri-stent FLP in post-TEVAR TBAD patients.
**Results or Findings:** Maximum area of the upper segment of FL and aortic arch angulation were independent risk factors for perioperative peri-stent FLP after TEVAR. ROC curve showed that maximum area of the upper segment of FL had higher predictive efficiency [AUC = 0.924; cut-off value: 2.69 $cm^2$] for perioperative peri-stent FLP after TEVAR compared with aortic arch angulation [AUC = 0.766; cut-off value: 48°].
**Conclusion:** Pre-operative maximum area of the upper segment of FL might be a good predictor for perioperative peri-stent FLP after TEVAR. The greater the maximum area of the upper segment of FL, the less likely it was for the obliteration of FL.
**Limitations:** Retrospective design, single-center research and relatively small sample may cause sampling error.
**Ethics Committee Approval:** The Ethical Review Committee of the West China Hospital of Sichuan University (Chengdu, Sichuan, China) approved this research.
**Funding for this study:** the National Natural Science Foundation of China [grant number 81601462].
**Author Disclosures:**
MA Huaxia Pu: Nothing to disclose

### Research Presentation Session: Vascular

### RPS 515
### Aneurysms and arteriosclerosis

### RPS 515-1
**Retrograde use of the outback re-entry catheter in complex infrainguinal arterial recanalisations**
*L. Patrone*[1], D. Akhtar[1], Z. Ahmed[1], P. Kalia[1], B. Dharmarajah[1], E. Blessing[2]; [1]London/UK, [2]Karlsbad/DE
*(Lorenzo.patrone@nhs.net)*

**Purpose:** We present our experience with retrograde, infrainguinal recanalizations using the Outback re-entry catheter in challenging chronic total occlusions.
**Methods or Background:** We report data from a retrospective multi-centre registry of high-volume centres. Procedural outcomes included technical success (defined as successful wire passage and delivery of adjunctive therapy with <30% residual stenosis), as well as safety (peri-procedural complications)
**Results or Findings:** 45 consecutive patients underwent retrograde, infrainguinal recanalization attempts with the Outback re-entry catheter between February 2015 and August 2020. Thirty (66.7 %) patients had history of open vascular surgery in the index limb. In 4 patients (8.9 %), recanalizations were even more challenging due to previous surgical removal and/or ligation of the proximal superficial artery. Retrograde access site of the Outback catheter was the femoropopliteal segment in 31 (68.9 %) patients and crural vessels in 14 (31.1 %) patients. The re-entry target sites were as follows: common femoral artery in 31 (68.9 %) patients, superficial femoral artery in 9 (20.0%) patients, popliteal artery in 1 (2.2 %) and below-the-knee arteries in 2 (4.4 %) patients. In 4 patients (8.9 %), the needle of the re-entry device was targeted to an inflated balloon, inserted via the antegrade route. The Outback re-entry catheter was placed via a 6-French sheath in 19 (42.2 %) cases and sheathless in 26 (57.8 %) cases. Technical success was achieved in 41 (91.1 %) patients, 30-day amputation free survival was at 100%. There were 2 (4.4 %) instances of distal embolizations and 3 (6.7 %) minor bleeding episodes.
**Conclusion:** Retrograde use of the Outback re-entry catheter in infra-inguinal chronic total occlusions provides an effective and safe endovascular adjunct, when conventional antegrade and retrograde recanalization attempts have failed.
**Limitations:** Retrospective study
**Ethics Committee Approval:** Not needed
**Funding for this study:** None

**Author Disclosures:**
Erwin Blessing: Consultant: Abbott, Shockwave, Cardinal Health
Zain Ahmed: Nothing to disclose
Brahman Dharmarajah: Nothing to disclose
Parul Kalia: Nothing to disclose
Lorenzo Patrone: Consultant: Abbott, Terumo, Pathfinder Medical, Shockwave
Daoud Akhtar: Nothing to disclose

### RPS 515-2

**Pledget reinforcement and tractional compression as adjunctive techniques for suture mediated closure in percutaneous endovascular aneurysm repair (pEVAR): a retrospective observational cohort study**

*L. Patrone*[1], M. Najem[2], M. Malina[1], D. Akhtar[1], Z. Ahmed[1], N. S. Theivacumar[1]; [1]London/UK, [2]Bedford/UK
*(Lorenzo.patrone@nhs.net)*

**Purpose:** Percutaneous endovascular aneurysm repair (pEVAR) are commonly utilised and require the use of suture-mediated closure (SMC) devices to ensure adequate femoral artery haemostasis. Despite the use of such devices, puncture related complications remain relatively common. We introduced two new adjuncts (pledget reinforcement and tractional compression) along with SMC to reduce such puncture related complications. The aim of the study was to assess the efficacy and safety of the new adjunct techniques compared to the previous year data prior to this new introduction of adjunct techniques.
**Methods or Background:** The two adjuncts described were introduced in our department in June 2018. 61 (in 32 patients) percutaneous common femoral artery punctures as part of routine pEVAR utilising adjunct techniques (Adjunct Group) were retrospectively compared to 89 punctures (in 46 patients) in the preceding year who had standard percutaneous closure with SMC (Standard Group). All patients had a CT Angiogram at one month and puncture complications (failure to achieve haemostasis requiring surgical cut down/forming hematoma, occlusion of puncture vessel requiring a surgical repair, pseudo aneurysm formation, wound infection, chronic pain) were objectively recorded at time of procedure.
**Results or Findings:** 61 percutaneous femoral punctures (in 31 patients) utilising adjunct techniques for closure (Adjunct Group) were retrospectively compared to 89 punctures (in 46 patients) closed with standard SMC technique (Standard Group). The use of adjunctive techniques led to a significant reduction in overall puncture related complications (3/61 (4.9%) vs. 20/89 (22.5%), p=0.0106), and the need for emergent surgical repair following failed haemostasis (2/61 (3.3%) vs. 13/89 (14.6%), p=0.037).
**Conclusion:** The pledget reinforcement and tractional compression of SMC for pEVAR reduces puncture related complications and increase the confidence to offer percutaneous techniques for more patients.
**Limitations:** Retrospective
**Ethics Committee Approval:** Obtained
**Funding for this study:** None
**Author Disclosures:**
Mojahid Najem: Nothing to disclose
Zain Ahmed: Nothing to disclose
Martin Malina: Nothing to disclose
Lorenzo Patrone: Nothing to disclose
Daoud Akhtar: Nothing to disclose
Nada Selva Theivacumar: Nothing to disclose

### RPS 515-3

**The ipsilateral retrograde approach to the treatment of femoropopliteal lesions: a systematic review**

*L. Patrone*, D. Akhtar, Z. Ahmed, P. Kalia, A. Antonietti, M. Covani, M. Malina; London/UK
*(Lorenzo.patrone@nhs.net)*

**Purpose:** The endovascular treatment of femoropopliteal lesions is an integral part of managing patients with peripheral arterial disease. The antegrade approach is the most widely used technique with good evidence for its safety and efficacy. Retrograde approach is nowadays increasingly used to maximise the chances of procedural success. The objective of this systematic review is therefore to assess the safety and effectiveness of the ipsilateral retrograde approach to femoropopliteal lesions.
**Methods or Background:** A systematic review conforming to the PRISMA standards was undertaken. MEDLINE, EMBASE and The Cochrane Register were searched between 1st January 1988 and 1st January 2020. Full-text, English language peer-reviewed articles pertaining to peripheral arterial disease, endovascular intervention and access site were included.
**Results or Findings:** A total of 8,599 papers were screened, of which 38, involving 1,940 patients undergoing 2,184 retrograde procedures, were included. The mean number of patients per study was 51.1, with three studies including <10 and four >100. A retrograde approach was used as the primary access route in 45.1% (648/1,438) of procedures with relevant data. Primary technical success was achieved in 87.9% (1,920/2,184, 63.5%-100%) with a reported complication rate of 11.1% (235/2,117, 0% - 27.1%).
**Conclusion:** The ipsilateral retrograde approach to femoropopliteal lesions has good primary technical success and a low rate of complications, and has a promising role as a bailout, or even a primary access technique in complex lesions. Patient positioning, puncture site and technique, lesion anatomy and the size of catheters and devices used are important considerations to achieve the best outcomes.
**Limitations:** Overall, the quality of evidence was poor, with just 7 papers deemed to be of high quality with a low risk of bias.
**Ethics Committee Approval:** Not required
**Funding for this study:** No funds
**Author Disclosures:**
Zain Ahmed: Nothing to disclose
Alberto Antonietti: Nothing to disclose
Marco Covani: Nothing to disclose
Martin Malina: Nothing to disclose
Parul Kalia: Nothing to disclose
Lorenzo Patrone: Consultant: Abbott, Terumo, Pathfinder Medical, Shockwave
Daoud Akhtar: Nothing to disclose

### RPS 515-5

**Comparative diagnostic role of CTA vs NC-MRA in CVA patients**

*R. Rastogi*, S. Parashar, N. Jain, V. Pratap; Moradabad/IN
*(rajulrst@yahoo.co.in)*

**Purpose:** Cerebrovascular accidents (CVA) are a major cause of mortality and morbidity in middle and elderly age groups. It can be ischemic or hemorrhagic and thrombotic or embolic. Digital subtraction angiography (DSA) is a gold standard investigation for evaluating patients with CVA. Due to the invasive nature of DSA, computed tomography angiography (CTA) has long been used as a primary noninvasive imaging tool to evaluated patients with CVA. Angiography can also be performed with magnetic resonance imaging, both with & without contrast. Introduction: CTA serves as a primary noninvasive imaging tool in evaluation of patients with CVA as nearly half of these patients do not have treatable underlying cause. Due to increasing awareness regarding the radiation exposure, contrast-induced nephrotoxicity and iodine sensitivity, magnetic resonance angiography (MRA) is gaining more and more attention. Noncontrast MRA (NC-MRA) can be performed utilizing 3D-time of flight sequence which provides results comparable to that of CTA. Hence, we performed a study to evaluate comparable role of NC-MRA and CTA .
**Methods or Background:** Fifty patients with cerebrovascular accidents were evaluated with CTA & NC-MRA in tandem on the same day. The results obtained were statistically evaluated and conclusions were drawn.
**Results or Findings:** Detection of aneurysm in intracranial and stenoses in intracranial as well as extracranial vessels can be detected with good accuracy by NC-MRA. The results of NC-MRA was comparable to that obtained by CTA except in very small aneurysm (<3mm) and early stenosis (20-30%) which rarely affect immediate patient management.
**Conclusion:** MRA is comparable in accuracy to CTA in detection of clinically-significant ICA stenosis as well as in detecting occlusive disease of intracranial arteries. Hence, NC-MRA can be a good substitute to CTA.
**Limitations:** 1.5T MR scanner
Small sample volume
**Ethics Committee Approval:** Approved
**Funding for this study:** None
**Author Disclosures:**
Neha Jain: Nothing to disclose
Rajul Rastogi: Nothing to disclose
Sagar Parashar: Nothing to disclose
Vijai Pratap: Nothing to disclose

### RPS 515-6

**Association between PET functional imaging and CT/MR morphological findings at CT/MR imaging at baseline and during follow-up in patients affected by large vessel vasculitis**

*M. Ferrari*, F. Monelli, G. Besutti, L. Spaggiari, F. Muratore, C. Salvarani, P. Mancuso, P. Giorgi Rossi, P. Pattacini; Reggio Emilia/IT
*(m21011990@gmail.com)*

**Purpose:** To evaluate the association between baseline PET uptake and MR/CT contrast-enhanced angiography findings (aneurysmal dilatations, stenosis and arterial wall thickening) present at baseline or appeared after at least 6 months follow-up, in patients with giant cell (GCA) or Takayasu arteritis (TA). The association between baseline thickening and appearance of stenosis/dilatations was also evaluated.
**Methods or Background:** All consecutive GCA or TA patients who simultaneously performed PET and CT or MR (<6 months distance) were included. Images were retrospectively reviewed to assess, for each of 12 vascular segments, stenosis (<50%/50-74%/>75%), dilatations

(absence/presence), thickening (3mm/4mm/>4mm), and PET uptake (0-3, with grade>2 indicating active disease). Follow-up CT/MR examinations performed 6 to 30 months after baseline PET were also reviewed.
**Results or Findings:** Ninety-eight patients were included (age 57±18,8 years, 81% females, 55 TA and 43 GCA), for a total of 1176 arterial segments. For 31 patients, a follow-up CT/MR was available. Baseline PET uptake showed weak or no association with baseline stenosis (p=0.19) and dilatations alone (p=0.37), when considering stenosis and/or dilatations a borderline significant association was observed (stenosis/dilatation in 9% PET inactive vs 15% in PET active segments, p=0.08); while it was highly associated with baseline thickening (thickening in 15% PET inactive vs 32% in PET active segments, p<0.001). Baseline PET uptake was associated with appearance at follow-up of stenosis and/or dilatations (1,5% vs 11% stenosis/dilatations in PET inactive vs active segments, p=0.003). Baseline thickening was not associated with follow-up appearance of stenosis and/or dilatations (p=0.78).
**Conclusion:** PET uptake is highly associated with CT/MR arterial wall thickening at baseline. Baseline PET uptake, but not thickening, may predict morphological vascular changes during follow-up.
**Limitations:** Further analyses are necessary to evaluate the association of imaging findings with clinical outcomes.
**Ethics Committee Approval:** AVEN number:724/2020
**Funding for this study:** None.
**Author Disclosures:**
Francesco Muratore: Nothing to disclose
Lucia Spaggiari: Nothing to disclose
Pierpaolo Pattacini: Nothing to disclose
Filippo Monelli: Nothing to disclose
Marco Ferrari: Nothing to disclose
Carlo Salvarani: Nothing to disclose
Pamela Mancuso: Nothing to disclose
Paolo Giorgi Rossi: Nothing to disclose
Giulia Besutti: Nothing to disclose

### Research Presentation Session: Oncologic Imaging

### RPS 316

### Gastrointestinal oncology

### RPS 316-1

**Analysis of main preoperative prognostic factors in locally advanced rectal cancer: an essential role of the EMVI**

*B. Desana*, A. Balbo-Mussetto, G. Gallo, E. Soncin, D. Molinar, A. Macera, A. Fornari, M. Petracchini, S. Cirillo; Turin/IT
*(benedetta.desana@me.com)*

**Purpose:** Treatment of locally advanced rectal cancer foresees neoadjuvant chemo-radiotherapy, followed by radical surgery. Based on outcome of histological examination, patients are then subjected to adjuvant chemotherapy. With this approach there is little control over the development of metachronous metastases. Clinical and Magnetic Resonance (MR) data have been analyzed in order to identify the most important presurgical prognostic factor.
**Methods or Background:** Data of fifty-nine consecutive patients who performed a staging MR for rectal cancer have been retrospectively evaluated. After neoadjuvant therapy, all of them underwent surgery with curative intent and were monitored for at least two years through clinical-instrumental follow-up. Univariate and multivariate analyses using COX model were performed in order to identifying the main risk factors for the development of metachronous metastases. Kaplan-Meier analysis was made to evaluate the cumulative incidence of metastases between different groups. A ROC (Relative Operating Characteristics) curve was executed to compare the most relevant parameters of MR and Pathological Anatomy (PA).
**Results or Findings:** Metachronous metastases appeared in 17/59 patients. Univariate analyses demonstrated the statistically significant relevance of parameters like extra-mural vascular invasion in MR (mrEMVI; HR=4.06, p=0.009) and in PA (pEMVI; HR=5.74; p=0.003), positivity of lymphnodes (HR=10.16, p<0.001) and radial margins infiltration (HR=13.42, p<0.001) at histological examination. Multivariate analysis of MRI parameters showed an increased risk of significant metastasis only in mrEMVI positive patients (HR=3.195, p 0.045). The difference between EMVI-related ROC curves in MRI and AP was not statistically significant (p=0.2904).
**Conclusion:** EMVI currently appears to be early prognostic factor that best predicts the risk of metachronous metastasis. In anticipation of new presurgical personalized therapies it could be used as a benchmark for choosing the most appropriate approach.
**Limitations:** Low sample quantity.
**Ethics Committee Approval:** Ethical committee approval was obtained.
**Funding for this study:** No founding.
**Author Disclosures:**
Benedetta Desana: Nothing to disclose
Daniela Molinar: Nothing to disclose
Enrico Soncin: Nothing to disclose
Massimo Petracchini: Nothing to disclose
Annalisa Balbo-Mussetto: Nothing to disclose
Stefano Cirillo: Nothing to disclose
Alberto Fornari: Nothing to disclose
Giulia Gallo: Nothing to disclose
Dear Annalisa Macera: Nothing to disclose

### RPS 316-2

**Radiomic analysis performed on 68Ga-DOTANOC PET/CT allows discriminating primary pancreatic neuroendocrine tumour grading**

A. Bevilacqua, D. Calabrò, *S. Malavasi*, C. Ricci, R. Casadei, D. Campana, S. Fanti, V. Ambrosini; Bologna/IT
*(s.malavasi@unibo.it)*

**Purpose:** To evaluate the effectiveness of radiomic features (RFs) computed on 68Ga-DOTANOC PET/CT to discriminate grade 1 (G1) and grade 2 (G2) pancreatic neuroendocrine tumours (panNET).
**Methods or Background:** Twenty-eight patients with primary panNET (M:F=13:15; mean age: 56 years old [17-78]; G1=13, G2=15) whose grading was assessed after surgical excision were enrolled in this study. First and second-order RFs were computed on the standardized uptake value (SUV) maps pertaining to the whole lesion. 1770 coupled texture RFs initially were considered, but those with a high correlation were subsequently discarded. The linear discriminant analysis was used on the remaining couples to build as many discriminative radiomic models (RMs), and their discrimination capability was assessed using two-tail Wilcoxon rank-sum test on the RF space and the Bonferroni correction (p-value=0.001). The Area under the curve (AUC) of the receiver operating characteristic (ROC) was used to assess RM performances, together with sensitivity, specificity and accuracy. The best RM was selected as that showing the lowest p-value and the highest AUC.
**Results or Findings:** The RF couple made of first-order skewness and second-order sum entropy shows the highest significant performance (p-value=0.0003) with sensitivity=93%, specificity=85%, accuracy=89%, achieved with 2 false positive and 1 false negative.
**Conclusion:** The radiomic model identified shows a high statistical significance in discriminating G2 from G1 primary panNET and it is promising for the validation step. This preliminary study suggests that the texture-based RFs computed on 68Ga-DOTANOC PET/CT could represent a valid tool for tumour grade assessment, potentially useful in panNET not amenable to biopsy before surgery.
**Limitations:** The limited number of sample size.
**Ethics Committee Approval:** 164/2017/O/Oss
**Funding for this study:** Not applicable
**Author Disclosures:**
Diletta Calabrò: Nothing to disclose
Davide Campana: Nothing to disclose
Silvia Malavasi: Nothing to disclose
Riccardo Casadei: Nothing to disclose
Valentina Ambrosini: Nothing to disclose
Stefano Fanti: Nothing to disclose
Claudio Ricci: Nothing to disclose
Alessandro Bevilacqua: Nothing to disclose

### RPS 316-3

**Identifying high-risk colon cancer on CT: can a radiomics signature improve radiologist's performance for T staging?**

*E. K. Hong*[1], Z. Elkarghali[1], F. Landolfi[1], N. Bogveradze[1], P. Bos[1], S. J. Park[2], J. M. Lee[2], R. G. H. Beets-Tan[1]; [1]Amsterdam/NL, [2]Seoul/KR
*(amyh0803@gmail.com)*

**Purpose:** To assess the role of radiomics in detection of high-risk (pT3-4) colon cancer and develop a combined model that combines both radiomics and CT staging of colon cancer.
**Methods or Background:** We included 292 colon cancer patients who underwent pre-operative CT and primary surgical resection within two months. Three-dimensional segmentations and CT staging of primary colon tumours were done. From each 3D segmentation of colon tumour, radiomic features were automatically extracted. Logistic regression analysis was performed to identify associations between radiomic features and high-risk (pT3-4) colon tumours. A combined model that integrated both radiomics and CT staging was developed and their diagnostic performance was compared with that of conventional CT staging. 10-fold cross-validation was used to validate the performance of the model and CT staging.
**Results or Findings:** The model that combined radiomic features and CT staging demonstrated a significantly better performance in detection of high-risk colon tumours in training set (AUC = 0.799, 95% CI: 0.720 – 0.839 for combined model and AUC = 0.697, 95% CI = 0.538 – 0.756 for CT staging only, p < 0.001 for difference). Cross-validation results also demonstrated significantly better detection performance of combined model (AUC = 0.727, 95% Confidence Interval (CI): 0.621 – 0.777 for combined model and AUC = 0.628, 95% CI = 0.558 – 0.689 for CT staging only, Boot CI = 0.099).
**Conclusion:** CT radiomic features of primary colon cancer, combined with CT staging, can improve the detection of high-risk colon cancer patients.
**Limitations:** Tumour segmentations were manually done by 3 radiologists.
**Ethics Committee Approval:** This is a retrospective study and local institutional boards from two tertiary centers approved and waived the requirement for informed consent.
**Funding for this study:** None
**Author Disclosures:**
Zuhir Elkarghali: Nothing to disclose
Sae Jin Park: Nothing to disclose
Jeong Min Lee: Nothing to disclose
Eun Kyoung Hong: Nothing to disclose
Regina G. H. Beets-Tan: Nothing to disclose
Federica Landolfi: Nothing to disclose
Nino Bogveradze: Nothing to disclose
Paula Bos: Nothing to disclose

### RPS 316-4

**Radiomic analysis could allow detecting misassigned bioptic grading when discriminating G1 and G2 primary pancreatic neuroendocrine tumours imaged with 68Ga-DOTANOC PET/CT**

*S. Malavasi*, D. Calabrò, A. Bevilacqua, C. Ricci, R. Casadei, D. Campana, S. Fanti, V. Ambrosini; Bologna/IT
*(s.malavasi@unibo.it)*

**Purpose:** Evaluating whether radiomic analysis applied to 68Ga-DOTANOC PET/CT examinations could allow detecting misassigned bioptic grading, when discriminating grade 1 (G1) and grade 2 (G2) pancreatic neuroendocrine tumours (panNET).
**Methods or Background:** Thirty-three patients with low grade primary panNET assessed through pathological bioptic samples underwent 68Ga-DOTANOC PET/CT. Seven lesions with volume lower than 1cm3 were excluded from the study and standardized uptake value (SUV) maps were extracted for the remaining twenty-six patients (M:F=11:15; mean age:64 y.o. [48-85]; G1:G2=18:8). 60- first and second-order radiomic features (RFs) were computed on SUV maps of the whole tumour volume, discarding those showing high linear correlation. A discriminative radiomic model (RM) was generated for each of the surviving RFs. The area under the curve (AUC) were computed on the receiver operating characteristic curve. To evaluate models' discrimination capability, the two-tail Wilcoxon rank-sum test was applied (p-value≤0.05) on the features space and the RF with the lowest p-values and the highest AUC were selected.
**Results or Findings:** The best performing RM is based on second-order correlation which provides a significant (p-value=0.028) separation between G2 and G1 panNET, with AUC=0.78, sensitivity=88%, specificity=78%, accuracy=81%. More importantly, the only FN is a lesion with borderline ki67=3 index, detected as FN in all the other RMs, this leading to questioning if it was correctly discriminated by the bioptical sample examination.
**Conclusion:** This preliminary study showed that this single RF well discriminates low grade panNET. Considering that tumour grade based on bioptical samples is not representative of the entire lesion, RFs may be used as tool to overcome this limitation, assigning a correct grade in all patients, especially in cases with borderline ki67.
**Limitations:** Reduced patients' sample size and lack of grading assessment on tumour surgically excided.
**Ethics Committee Approval:** 164/2017/O/Oss
**Funding for this study:** Not applicable
**Author Disclosures:**
Diletta Calabrò: nothing to disclose
Davide Campana: Nothing to disclose
Silvia Malavasi: Nothing to disclose
Riccardo Casadei: Nothing to disclose
Valentina Ambrosini: Nothing to disclose
Stefano Fanti: Nothing to disclose
Claudio Ricci: Nothing to disclose
Alessandro Bevilacqua: Nothing to disclose

### RPS 316-5

**PET/MRI for the preoperative diagnosis and staging of peritoneal carcinomatosis: a prospective multireader study**

*N. Vietti Violi*[1], S. Gavane[2], P. Argiriadi[2], A. Law[2], S. Heiba[2], E. Bekhor[2], J. S. Babb[2], D. Labow[2], B. Taouli[2]; [1]Lausanne, VD/CH, [2]New York, NY/US
*(nviettivioli@gmail.com)*

**Purpose:** To assess the diagnostic performance of FDG-PET/MRI for diagnosis and staging of peritoneal carcinomatosis (PC) using surgical Sugarbaker's PCI (PC index) as the reference.
**Methods or Background:** 14 adult patients (mean age:57±12y) with PC were prospectively included in 2016-2018 in a single center. They underwent PET/MRI prior to surgery (14d, range:1-63d) that were reviewed independently by 2radiologists and 2nuclear medicine physicians. According to surgical PCI, the abdomen was divided in 13regions, scored 0-3 according to tumor burden. Each region score was summed to reach PCI (0-39). Radiologists read 2sets separated by 6w: 1)T2wi+DWI, and 2)T2wi+contrast-enhanced sequences. Nuclear medicine physicians read images based on PET images+T2wi. Hybrid PET/MRI PCI was created by combining the data. Inter-reader agreement was measured using weighted-kappa. For each set, radiological PCI was compared to the surgical PCI on a per-patient and per-region basis using generalized estimating equations based on logistic regression.
**Results or Findings:** Mean surgical PCI was 10±8 [range 0-24,PCI 0(n=2),PCI 1-10(n=8),PCI 11-19(n=1),PCI 20-24(n=3)]. Interreader agreement was almost perfect for all sets for PCI (Kappa:0.81-0.98). Per-patient pooled sensitivity, specificity, accuracy were 75%(18/24), 50%(2/4), 71.4%(20/28) for MRI; 66.7%(16/24), 50%(2/4), 64.3%(18/28) for PET and 91.7%(22/24), 50%(2/4), 85.7%(24/28) for PET/MRI, without statistical difference. MRI and PET/MRI achieved 100% sensitivity and specificity for cutoff PCI of 20. Per-region sensitivity, specificity, accuracy were 37%(54/146), 78.4%(171/218), 61.8%(225/364) for MRI, 17.8%(26/146), 95%(207/218), 64.3%(234/364) for PET and 52.7%(77/146), 66.5%(145/218), 60.4%(220/364) for PET/MRI, with statistical difference between sets for sensitivity (p:0.001–0.035) and specificity (p<0.001).
**Conclusion:** PET/MRI improves sensitivity and accuracy for PC detection on per-patient basis, and has high diagnostic performance in patients with high tumor burden. The performance of PET/MRI is lower per-region.
**Limitations:** This study is limited by its small sample size.
**Ethics Committee Approval:** Local ethics committee approved the study.
**Funding for this study:** No funding.
**Author Disclosures:**
Amy Law: Nothing to disclose
James S Babb: Nothing to disclose
Sherif Heiba: Nothing to disclose
Naik Vietti Violi: Nothing to disclose
Bachir Taouli: Nothing to disclose
Eliahu Bekhor: Nothing to disclose
Pamela Argiriadi: Nothing to disclose
Daniel Labow: Nothing to disclose
Somali Gavane: Nothing to disclose

### RPS 316-6

**PET/MRI for M-staging of rectal cancer: a primary economic evaluation**

*F. G. Gassert*[1], J. Rübenthaler[1], F. T. Gassert[1], M. R. Makowski[1], S. O. Schönberg[2], M. F. Froelich[2]; [1]Munich/DE, [2]Mannheim/DE

**Purpose:** Correct identification of the TNM state in primary staging of rectal cancer has critical implications on patients' management. Initial evaluations revealed a high sensitivity and specificity for whole body (wb) PET/MRI in detection of metastasis allowing the application of local ablative techniques. Nevertheless, cost-effectiveness compared to standard procedure using pelvic MRI + chest and abdominopelvic CT (SCI) is yet to be investigated and therefore was aim of this study.
**Methods or Background:** A decision model including the diagnostic modalities wbPET/MRI and SCI was created based on Markov simulations estimating lifetime costs and quality-adjusted life years (QALYs). Deterministic sensitivity of diagnostic parameters and costs was determined and probabilistic sensitivity analysis using Monte-Carlo Modelling was applied.
**Results or Findings:** In the base-case scenario, the strategy wbPET/MRI resulted in total costs of $ 52,186 whereas total costs of SCI were at $ 51,672. WbPET/MRI resulted in an expected effectiveness of 3.542 QALYs versus 3.535 QALYs for SCI. This resulted in an incremental cost-effectiveness ratio of $ 70,291 per QALY for PET/MRI. Therefore, from an economic point of view wbPET/MRI was identified as an adequate diagnostic alternative to SCI.
**Conclusion:** Based on the results of the analysis use of wbPET/MRI was identified as a feasible diagnostic strategy for initial staging of rectal cancer from a cost-effectiveness perspective.
**Limitations:** Regarding limitations, our Markov-model does not differ between local tumor-states as this would outrange the scope of this study. Additionally, we assumed biopsy to be performed in approximately two third of unclear cases in SCI. Although this number may vary in clinical practice, sensitivity analysis showed high robustness of results to variation of input parameters.
**Ethics Committee Approval:** Not applicable.
**Funding for this study:** No funding was received.
**Author Disclosures:**
Florian Tilman Gassert: Nothing to disclose
Stefan Oswald Schönberg: Nothing to disclose
Matthias Frank Froelich: Nothing to disclose
Marcus R. Makowski: Nothing to disclose
Felix Gerhard Gassert: Nothing to disclose
Johannes Rübenthaler: Nothing to disclose

### RPS 316-7

**CT for lymph node staging of colon cancer: not only size but also location and number of lymph node count**

*E. K. Hong*[1], F. Landolfi[1], F. Castagnoli[1], S. J. Park[2], J. M. Lee[2], R. G. H. Beets-Tan[1]; [1]Amsterdam/NL, [2]Seoul/KR
*(amyh0803@gmail.com)*

**Purpose:** To evaluate the diagnostic accuracy of imaging features to predict lymph node status of colon cancer using CT.
**Methods or Background:** 317 Colon cancer patients who underwent primary surgical treatment were included. The number of lymph nodes according to the anatomical location, size, cluster, degree of attenuation, shape, presence of internal heterogeneity and irregular outer border of the lymph node were assessed and compared according to histological lymph node status.
**Results or Findings:** The largest short diameter of lymph node and presence of internal heterogeneity of lymph node showed significant association with malignant lymph node status (P<0.001 and P=0.041, respectively). The ROC curve analysis revealed AUC of 0.703 for the largest short diameter of lymph node (P<0.001), and AUC of the presence of internal heterogeneity was 0.630 (P<0.001). In addition, our study showed that a total number of lymph nodes, regardless of size, (P=0.022) and number of lymph nodes in the peritumoral area (P<0.001) and along the mesenteric vessels (P<0.001) on CT demonstrated significant association with malignant status of lymph nodes in colon cancer.
**Conclusion:** There were significant associations between lymph node status and imaging features of lymph nodes on CT in colon cancer patients. The largest short diameter of lymph node and presence of internal heterogeneity can be effectively used to predict the malignant status of lymph node in colon cancer patients. Also, the number of lymph nodes near the colonic tumor should be considered in the assessment of colon cancer lymph node involvement on CT.
**Limitations:** This study was limited by a lack of lesion based assessment of radiologic-pathologic data.
**Ethics Committee Approval:** This was a retrospective study. Institutional IRB approved the study and waived informed consent.
**Funding for this study:** None
**Author Disclosures:**
Francesca Castagnoli: Nothing to disclose
Sae Jin Park: Nothing to disclose
Jeong Min Lee: Nothing to disclose
Eun Kyoung Hong: Nothing to disclose
Regina G. H. Beets-Tan: Nothing to disclose
Federica Landolfi: Nothing to disclose

### RPS 316-8

**Comparing PET-derived semiquantitative parameters and radiomic features in discriminating G1 and G2 primary pancreatic neuroendocrine tumours in 68Ga-DOTANOC PET/CT**

*S. Malavasi*, D. Calabrò, A. Bevilacqua, C. Ricci, R. Casadei, D. Campana, S. Fanti, V. Ambrosini; Bologna/IT
*(s.malavasi@unibo.it)*

**Purpose:** To assess whether PET-derived semiquantitative parameters (PDSPs) extracted from 68Ga-DOTANOC PET/CT and single radiomic features (RFs) can differentiate low grade pancreatic neuroendocrine tumour (panNET).
**Methods or Background:** 13 patients with G1 and 15 with G2 primary panNET demonstrated by pre-surgical 68Ga-DOTANOC PET/CT were included in this study (M:F=13:15; mean age: 56 years old [17-78]). Tumour grading was assessed after surgical excision evaluation. Total lesion receptorial expression (TLRE), Receptorial tumour volume (RTV), and SUVmax were analysed together with 60 first and second-order RFs computed on standardized uptake value (SUV) maps of the primary lesion whole volume. To prevent overfitting, only single RF were considered to generate discriminative radiomic models, where linearly correlated RFs were removed. Discrimination capability was assessed through the two-tail Wilcoxon rank-sum test with Bonferroni correction (p-value<0.0031). Receiver operating characteristic and area under the curve (AUC) were computed. Features with the lowest p-values and highest AUC were selected.
**Results or Findings:** RTV is the only PDSP yielding a significant separation (p-value=0.03) between G2 and G1 panNET patients (Sensitivity=67%, Specificity=92%, Accuracy=79%). Indeed, SUVmax (Sensitivity=93%, Specificity=38%, Accuracy=68%, p-value=0.71) and TLRE (Sensitivity=87%, Specificity=54%, Accuracy=71%, p-value=0.20) were not significant and led to worse performance, accordingly. On the contrary, the first-order kurtosis provided the best performance (p-value=0.0009) at all, with Sensitivity=93%, Specificity=77%, Accuracy=86%.
**Conclusion:** Despite PDSPs are more easily accessible by clinicians, RFs proved to be more accurate in discriminating tumour grading in well-differentiated panNETs. The use of this radiomic model, if validated on a larger population, could represent a novel non-invasive approach to avoid biopsy before surgery, especially in selected patients not amenable to biopsy or with poor health conditions.
**Limitations:** Reduced patients' sample size.
**Ethics Committee Approval:** 164/2017/O/Oss
**Funding for this study:** Not applicable
**Author Disclosures:**
Diletta Calabrò: Nothing to disclose
Davide Campana: Nothing to disclose
Silvia Malavasi: nothing to disclose
Riccardo Casadei: nothing to disclose
Valentina Ambrosini: Nothing to disclose
Stefano Fanti: Nothing to disclose
Claudio Ricci: Nothing to disclose
Alessandro Bevilacqua: Nothing to disclose

### RPS 316-9

**The use of Lipiodol in hepatocellular carcinoma: multipotent applicability or common contrast media?**

*M. C. Langenbach*[1], T. J. Vogl[2], J-E. Scholtz[2], L. Basten[2], R. Hammerstingl[2], T. Gruber-Rouh[2]; [1]Cologne/DE, [2]Frankfurt a. Main/DE
*(marcel.langenbach@me.com)*

**Purpose:** To evaluate the potentials of Lipiodol in diagnosis, therapy and outcome-to-therapy prediction of hepatocellular carcinoma (HCC) beyond the established application as a lesion-visualizer, drug carrier and micro-embolic agent.
**Methods or Background:** In total, 250 patients with HCC were evaluated regarding the potentials of Lipiodol. First, 60 patients with hepatic LI-RADS 3 and 4 lesions in MRI received a Lipiodol angiography directly followed by an unenhanced CT to identify enhancement patterns allowing a differentiation of benign vs. malign lesions. Second, 204 patients with HCC suspicious liver lesions underwent a CT-guided biopsy, 67 unenhanced, 26 with i.v. contrast, and 96 with prior Lipiodol marking of the suspected lesion. Third, 30 patients with secured HCC received a Lipiodol-based cTACE. Enhancement patterns in angiography and CT were analyzed regarding the outcome prediction.
**Results or Findings:** Lipiodol shows potential benefits for all steps in tumor diagnosis and treatment. Clearly detectable lesions in Lipiodol-based angiography with a homogeneous or lacunar enhancement in post-angiographic CT allow for differentiation of LI-RADS 3 and 4 lesions as malign (sensitivity 86.1%, specificity 100%). The lesion hitting rate was significantly better for lesions prior marked with Lipiodol compared to i.v. contrast or unenhanced lesions (78.6% vs. 73.2% vs. 65.2%, overall 72.2%, p=0.038), this benefit is increased in small lesions and cirrhotic liver. Therapy monitoring during cTACE is possible, strong enhancing, hypervascularized lesions show a significant better response (p=0.008; rho=0.475) allowing an early adaption of the therapy protocol.
**Conclusion:** Use of Lipiodol beyond the role for TACE might be beneficial in several steps of diagnosis and treatment of HCC for selected patients: in differentiation of LI-RADS 3/4 lesions in benign vs. malign, in CT-guided biopsies, and in therapy outcome prediction of cTACE during treatment.
**Limitations:** Retrospective
**Ethics Committee Approval:** Approved
**Funding for this study:** Research grant (Guerbet)
**Author Disclosures:**
Lajos Basten: Nothing to disclose
Jan-Erik Scholtz: Nothing to disclose
Renate Hammerstingl: Nothing to disclose
Tatjana Gruber-Rouh: Nothing to disclose
Thomas J. Vogl: Nothing to disclose
Marcel Christian Langenbach: Research/Grant Support: Guerbet GmbH

### Research Presentation Session: Oncologic Imaging

### RPS 416

### Genitourinary oncology

### RPS 416-2

**The value of radiomic features extracted from preoperative contrast-enhanced computed tomography (CECT) in predicting tumor mutation burden (TMB) of bladder cancer patients**

*X. Tang*, W-F. Yan, Y-l. Gong, Z-G. Yang; Chengdu/CN

**Purpose:** Tumor mutation burden (TMB) is an emerging biomarker for predicting the response of immunotherapy in bladder cancer (BLCA). We aim at investigating the value of radiomic features in predicting the TMB status of BLCA patients.
**Methods or Background:** Totally, 75 patients with BLCA were enrolled. Radiomic features extracted from volume of interest of preoperative contrast-enhanced computed tomography (CECT) were obtained in each case. Unsupervised hierarchical clustering analysis was performed based on the radiomic profile. Logistic regression and least absolute shrinkage and selection operator (LASSO) regression analysis were used to test each feature's power in TMB-prediction. Finally, a TMB-predicting model was established using radiomic features.
**Results or Findings:** The hierarchical clustering analysis divided the total cohort into group A (32.0%, 24/75) and group B (68.0%, 51/75) with high radiomic similarity within each group while distinct radiomic profile between groups. Patients in group A had significantly higher proportion of high TMB against those in group B (66.7% [16/24] vs. 41.2% [21/51], p=0.039). TMB regarded as a continuous factor was also higher in group A than group B (median TMB: 8.32 vs. 4.97 mut/Mb, p=0.049). Based on five radiomic features selected by logistic and LASSO regression, a TMB-predicting model was built and evaluated using different approaches which revealed the good performance of the model.
**Conclusion:** We firstly explored the connection between radiomics and TMB in BLCA and found that radiomic features from preoperative CECT can satisfactorily predict TMB status. Our findings will facilitate doctors in decision-making of TMB test in BLCA patients.
**Limitations:** This is a retrospective study with small sample size.
**Ethics Committee Approval:** No ethical approval or informed consent was needed for this study because all patients' data from TCIA and TCGA databases were publicly and freely available.
**Funding for this study:** Not applicable
**Author Disclosures:**
Xin Tang: Nothing to disclose
You-ling Gong: Nothing to disclose
Wei-Feng Yan: Nothing to disclose
Zhi-Gang Yang: Nothing to disclose

### RPS 416-3

**Diagnostic imaging approach to retroperitoneal lesions**

*S. Nagarajan*, D. Jagannathan, G. Kathirvelu, K. Geetha; Chennai/IN
*(cbesuriya@gmail.com)*

**Purpose:** To illustrate and classify the different types of retroperitoneal masses and their imaging features. To give a systematic approach to identifying and classifying primary retroperitoneal masses. Identify the imaging findings and its correlate with the HPE findings.
**Methods or Background:** Retroperitoneum is a compartmentalized space bounded anteriorly by posterior parietal peritoneum and posteriorly by transversalis fascia extending from diaphragm superiorly to pelvic brim inferiorly. The abdominal retroperitoneum is divided by fascial planes into the anterior and posterior pararenal spaces and perirenal space.
**Results or Findings:** IMAGING APPROACH CT and MR imaging can demonstrate important characteristics of retroperitoneal tumors. Step 1: Intra or Retroperitoneal - Beak sign, Embedded organ sign, Displacement of bowel loops. Step 2: Classification of retroperitoneal masses into primary and secondary masses. Step 3: Assessment of the tumor - Tumor composition(fat,cyst,solid,calcification), Vascularity(hypervascular/moderately vascular/hypovascular), Location. Step 4: Extent of the tumor for biopsy and surgical planning. SAMPLE CASES 1. Castlemans Disease 2. Paraganglioma 3. Primitive Neuroectodermal Tumor 4. Retroperitoneal Lymphoma 5. Retroperitoneal Necrotic Lymphadenopathy 6. Retroperitoneal Leiomyosarcoma 7. Retroperitoneal Lipomatosis 8. Undifferentiated Pleomorphic Sarcoma 9. Aggressive Fibromatosis 10. Liposarcoma 11. Mature Teratoma 12. Ganglioneuroma 13. Retroperitoneal Metastasis From Serous Papillary Carcinoma Of Ovary 14. Erdheim Chester Disease 15. Myxoid Liposarcoma
**Conclusion:** CT and MRI can contribute to tumor diagnosis, though histological confirmation is often required because of the considerable overlap of imaging features. Attention to diagnostic clues is essential in making an accurate radiologic diagnosis or narrowing the differential diagnosis and in obtaining clinically significant information. Cross-sectional imaging is key to the pre-operative staging and planning of retroperitoneal masses. Imaging also helps to select and guide the site to biopsy from these usually large and heterogeneous neoplasms.
**Limitations:** Not Applicable
**Ethics Committee Approval:** Not Applicable
**Funding for this study:** Not Applicable
**Author Disclosures:**
Suriyaprakash Nagarajan :
K Geetha: nothing to disclose
Gopinathan Kathirvelu: Nothing to disclose
Devimeenal Jagannathan: Nothing to disclose

### RPS 416-4

**Added value of MRI for extraperitoneal findings in potential CRS-HIPEC candidates: seeing the whole picture**

*I. Van'T Sant-Jansen*, E. Nerad, C. Rijsemus, M. P. Engbersen, D. M. J. Lambregts, R. G. H. Beets-Tan, A. Aalbers, N. Kok, M. J. Lahaye; Amsterdam/NL

**Purpose:** In colorectal cancer patients the selection of suitable cytoreductive surgery and hyperthermic peritoneal chemotherapy (CRS-HIPEC) candidates is based on the location and extent of peritoneal metastases (PM) and the presence of extraperitoneal metastases. MRI is increasingly being used to assess the extent of PM, however, the significance of extraperitoneal findings in these scans has never been evaluated before.
**Methods or Background:** Colorectal cancer patients who had undergone an additional MRI scan after standard diagnostic work-up with CT for preoperative staging between January 2016-January 2020 were selected. CT and MRI reports were reviewed for new extra-peritoneal findings (EPF) on MRI and the MRI findings concerning lesions previously indicated as equivocal (uncertain benign/malignant) on CT. Reference standard were surgical results or follow-up imaging.
**Results or Findings:** In 163 included patients 60 findings (58/163 patients) were noted on MRI: twenty-seven (45%) were new findings and thirty-three (55%) were on CT equivocal findings. Of the 33 equivocal findings 25 were 'rejected as malignant', 4 'confirmed malignant', 3 'more likely malignant', and 1 as 'less likely malignant' based on MRI. In 30 patients (18%) a new finding or an equivocal finding scored as 'more likely malignant' or 'confirmed malignant' on MRI could have contributed to a more complete CRS-HIPEC. CRS-HIPEC was cancelled in three patients (2%) because of EPF on MRI. Six EPF were missed on imaging altogether.
**Conclusion:** MRI had an added value in more than a third of the patients due to extraperitoneal findings that were undetected or indeterminate on CT and had a clinical impact in 20% of the patients. Therefore, MRI seems a logical addition to the diagnostic workup of potential CRS-HIPEC candidates.
**Limitations:** The retrospective nature of the study.
**Ethics Committee Approval:** Yes.
**Funding for this study:** None.
**Author Disclosures:**
Max Lahaye: Nothing to disclose
Elias Nerad: Nothing to disclose
Maurits Peter Engbersen: Nothing to disclose
Doenja Marina Johanna Lambregts: Nothing to disclose
Charlotte Rijsemus: Nothing to disclose
Niels Kok: Nothing to disclose
Regina G. H. Beets-Tan: Nothing to disclose
Iris Van'T Sant-Jansen: Nothing to disclose
Arend Aalbers: Nothing to disclose

### RPS 416-5

**Feasibility of ultra-fast dynamic perfusion 18F-NaF PET in prostate cancer as enabled by digital photon counting PET/CT**

*K. Binzel*, C. L. Wright, E. Folefac, D. A. Diaz Pardo, D. G. Stover, Y-l. Hsieh, M. V. Knopp; Columbus, OH/US

**Purpose:** To assess the feasibility of ultra-fast frame rate dynamic perfusion of 18F-NaF PET/CT for patients with advanced prostate cancer.
**Methods or Background:** Twenty patients with suspicion of recurrent prostate cancer were imaged as part of this initial feasibility and methodology development study. Patients were imaged on a digital photon counting system (Philips Vereos) for 15 minutes over a single bed position, beginning at the time of injection of 185MBq 18F-NaF, followed by whole body imaging at 75 minutes post-injection. Listmode data from the dynamic acquisition was reconstructed with time frames of 1, 9, and 15 seconds, in addition to the standard 1 minute per frame. Regions of interest were placed over any suspected bone tumors as well as regions of degenerative changes, blood pool, and a region of healthy bone.
**Results or Findings:** Using the 1 min/frame images for reference, regions of interest were placed in a reliable manner. The ultra-short 1 and 9 sec/frame dynamic curves gave exceptionally detailed uptake intensity curves of the perfusion in both bone lesions and areas of degenerative changes. The 15 sec frame rate can be used to visualize post perfusion phase uptake characteristics of each region.
**Conclusion:** One second per frame image reconstruction of dynamic perfusion 18F-NaF PET/CT data is consistently achievable even at a low dose of 185MBq. Quantification of tumor features such as vascularity, perfusion and uptake kinetics appear to be robustly and accurately assessable.
**Limitations:** Evaluation and further optimization of the count sparse reconstruction parameters will improve the quantitative readouts from such short frame time image sets.
**Ethics Committee Approval:** Performed with approval of University Institutional Review Board
**Funding for this study:** ODSA TECH 13-060 (IPP), R01 CA-195513, NCI 5U24CA180803 grants
**Author Disclosures:**
Chadwick Lewis Wright: Nothing to disclose
Dayssy A. Diaz Pardo: Nothing to disclose
Katherine Binzel: Nothing to disclose
Edmund Folefac: Nothing to disclose
Michael Vinzenz Knopp: Nothing to disclose
Yu-lung Hsieh: Nothing to disclose
Daniel G. Stover: Nothing to disclose

### RPS 416-6

**The prognostic value of radiomics features extracted from computed tomography (CT) in patients with localised clear cell renal cell carcinoma (ccRCC) after nephrectomy**
*X. Tang*, T. Pang, W-F. Yan, W. Qian, Y-l. Gong, Z-G. Yang; Chengdu/CN

**Purpose:** Radiomics is an emerging field of quantitative imaging. The prognostic value of radiomics analysis in patients with localized clear cell renal cell carcinoma (ccRCC) after nephrectomy remains unknown.
**Methods or Background:** Computed tomography images of 167 eligible cases were obtained from the Cancer Imaging Archive database. Radiomics features were extracted from the region of interest contoured manually for each patient. Hierarchical clustering was performed to divide patients into distinct groups. Prognostic assessments were performed by Kaplan-Meier curves, COX regression and least absolute shrinkage and selection operator COX regression. Besides, transcriptome mRNA data were also included in the prognostic analyses. Endpoints were overall survival (OS) and disease-free survival (DFS).
**Results or Findings:** Hierarchical clustering groups from nephrographic features and mRNA can divide patients into different prognostic groups while clustering groups from corticomedullary or unenhanced phase couldn't distinguish patients' prognosis. In multivariate analyses, 7 OS-predicting and 6 DFS-predicting features were identified in nephrographic phase. Similarly, 7 OS-predictors and 7 DFS-predictors were confirmed in mRNA data. In contrast, limited prognostic features were found in corticomedullary (1 OS-predictor and 2 DFS-predictors) and unenhanced phase (2 OS-predictors and 2 DFS-predictors). Prognostics models combining both nephrographic features and mRNA showed improved C-index than any model alone (C-index: 0.943 and 0.881 for OS- and DFS-predicting, respectively).
**Conclusion:** We firstly investigated the prognostic significance of preoperative radiomics signatures in ccRCC patients. Radiomics features obtained from nephrographic phase had stronger predictive ability than features from corticomedullary or unenhanced phase. Multi-omics models combing radiomics and transcriptome data could further increase the predictive accuracy.
**Limitations:** This is a retrospective study and require further external validation from other centers.
**Ethics Committee Approval:** No ethical approval or informed consent was needed because all patients' data from TCIA and TCGA databases were public.
**Funding for this study:** Not applicable.
**Author Disclosures:**
Wenlei Qian: nothing to disclose
Xin Tang: Nothing to disclose
You-ling Gong: Nothing to disclose
Tong Pang: Nothing to disclose
Wei-Feng Yan: Nothing to disclose
Zhi-Gang Yang: Nothing to disclose

### Research Presentation Session: Oncologic Imaging

### RPS 516

### CT in oncology

### RPS 516-1

**Incidence, risk factors and CT characteristics of radiation recall pneumonitis induced by immune checkpoint inhibitor in lung cancer**
*F. Cousin*; Liège/BE

**Purpose:** Radiation recall pneumonitis (RRP) is a delayed radiation-induced lung toxicity triggered by systemic agents, typically anticancer drugs. Immune checkpoint inhibitors (ICIs) have recently been identified as potential causal agents of RRP but its real incidence and potential risk factors remain unknown.
**Methods or Background:** Medical records and CTs of patients treated with programmed death 1 (PD-1) or programmed death ligand 1(PD-L1) inhibitors for advanced lung cancer between 2014 and 2019 at our tertiary center, and who had a previous history of lung irradiation were retrospectively analyzed. We identified RRP as lung CT modifications occurring in the irradiation field > 6 months after conventionally fractionated radiotherapy completion and >1 year after stereotactic body radiation therapy. Clinical and dosimetric data were analyzed to identify potential risk factors for RRP.
**Results or Findings:** Among 348 patients treated with ICIs, data from 80 eligible patients were analyzed (mean age, 69 years [standard deviation, 8.3]; 45 men). Fifteen patients (18.8%) presented with RRP. Median time between end of radiotherapy and RRP was 450 days (range, 231 – 1859). No risk factor was significantly associated with RRP. ICI-related pneumonitis was associated with RRP in 33.3% of cases ($p=0.0021$), developing either concomitantly or after RRP. Incidence of grade ≥ 3 pneumonitis in the RRP population was 13.3 %.
**Conclusion:** We demonstrated a high incidence of RRP (18.8%) in our population of previously irradiated patients treated with ICIs for lung cancer. We identified no risk factors for RRP, but an association was noted between RRP and ICI-related pneumonitis.
**Limitations:** Retrospective single institution analysis, additional prospective studies with larger sample sizes are required to confirm our observations. RPP has no clear definition in the literature.
**Ethics Committee Approval:** This study received approval from our institutional review board.
**Funding for this study:** No
**Author Disclosures:**
Francois Cousin: Nothing to disclose

### RPS 516-2

**Multiparametric functional imaging in lung cancer: the importance of tumour radiomic heterogeneity beyond FDG PET/CT and lung biopsy**
*T. Polidori*, D. Caruso, G. Guido, E. Lucertini, M. Zerunian, B. Bracci, M. Polici, M. Rossi, A. Laghi; Rome/IT
*(tiziano.polidori13@gmail.com)*

**Purpose:** [18F]-Fluorodeoxyglucose Positron Emission Tomography-Computed Tomography (FDG PET/CT) has a central role in the lung nodules' characterization even if, with SUV<2.5, percutaneous CT-guided Lung Biopsy (CTLB) is needed to assess nodule nature. In that scenario, CT Texture Analysis (CTTA) could be a non-invasive imaging biomarker. The aim of the study is to test CTTA ability in differentiating malignant from benign lung nodules.
**Methods or Background:** Patients that underwent FDG PET/CT followed by CTLB between January 2013 and December 2018 were retrospectively enrolled. Were included patients with lung nodule SUV<2.5 and histological diagnosis. Exclusion criteria: nodules with SUV>2.5, patients who refused CTLB or received oncological treatment before CTLB, indeterminate pathology report, motion artifacts on chest CT. Two radiologists in consensus performed CTTA on preliminary CT performed for CTLB with a dedicated software. Statistics included a comparison between malignant and benign neoplasms distribution (2-tailed T-test or Mann-Whitney test according to normal/non-normal data distribution), P-values<0.05 were considered significant. CTTA accuracy was test with Receiver operating characteristics (ROC) curve.
**Results or Findings:** From an initial population of 1178, 46 patients encountered inclusion criteria. Pathologist reported 27/46 (59%) with malignant and 19/46 (41%) with benign nodules. CTTA showed lower values of Kurtosis and higher values of Skewness in malignant lesions (all $P \leq 0.0013$ and all filtered TA $P<0.024$ respectively). ROC curve showed significant AUC for Kurtosis ($P<0.001$; AUC 0.654) and Skewness ($P<0.001$; AUC 0.642) at medium filtration.

**Conclusion:** CTTA is a promising radiological tool which may allow the characterization of benign and malignant lung nodules even in those cases without an altered glucose metabolism.
**Limitations:** Small population sample; only first order texture parameters.
**Ethics Committee Approval:** This study was approved by our local institutional review board and written informed consent was obtained from all study participants.
**Funding for this study:** No.
**Author Disclosures:**
Damiano Caruso: Nothing to disclose
Michele Rossi: Nothing to disclose
Marta Zerunian: Nothing to disclose
Benedetta Bracci: Nothing to disclose
Tiziano Polidori: Nothing to disclose
Andrea Laghi: Nothing to disclose
Michela Polici: Nothing to disclose
Gisella Guido: Nothing to disclose
Elena Lucertini: Nothing to disclose

### RPS 516-3

**Skeletal muscle mass and skeletal muscle radiodensity: two sides of the same medal or two different medals?**

*L. Weerink*[1], B. van Leeuwen[2], T. Kwee[2], G. de Bock[2]; [1]Almelo/NL, [2]Groningen/NL

**Purpose:** Muscle wasting is associated with inferior outcome following surgical treatment of solid malignancies. Radiological measurement of sarcopenia is a widespread method to detect muscle wasting. Measurement of myosteatosis might present an alternative strategy. Aim of this study was to evaluate if the presence of myosteatosis is related to the presence of sarcopenia and if myosteatosis can be used to predict the presence of sarcopenia.
**Methods or Background:** For this analysis, data were used from the PICNIC cohort including patients undergoing surgical cancer treatment. The total psoas index (TPI) for detection of sarcopenia and psoas radiodensity (PRD) for detection of myosteatosis on the L3 level were estimated on CT images. Age and Body Mass Index (BMI) were registered, BMI≤25 was considered normal. Gender-specific 25-percentile cut-off values were applied to determine low TPI and PRD. Spearman's correlations coefficient was calculated. To determine if a low PRD relates to a low TPI logistic regression analyses were performed.
**Results or Findings:** A total of 372 patients were included. 79 patients (24.2%) had a low TPI and 85 patients (26%) had a low PRD. 29 (8.9%) patients had both a low TPI and low PRD. BMI was normal in 35.5%. The correlation between TPI and PRD was 0.011 (P:0.84). A low PRD related to the presence of a low TPI (OR adjusted for BMI and age:2.02, 95-CI:1.12-3.64, P:0.019.
**Conclusion:** This study showed that in patients undergoing surgical cancer treatment the presence of myosteatosis combined with a normal BMI relates to the presence of sarcopenia. Therefore, even though the correlation between sarcopenia and myosteatosis in general is low, presence of myosteatosis in patients with a normal BMI can function as a warning for the presence of sarcopenia.
**Limitations:** Not applicable.
**Ethics Committee Approval:** Not applicable.
**Funding for this study:** None.
**Author Disclosures:**
Geertruida de Bock: Nothing to disclose
Linda Weerink: Nothing to disclose
Thomas Kwee: Nothing to disclose
Barbara van Leeuwen: Nothing to disclose

### RPS 516-4

**The prognostic significance of radiomic features from chest CT in advanced non-small cell lung cancer (NSCLC) patients with EGFR-T790M mutation receiving second-line osimertinib treatment**

*X. Tang*, Y. Li, Y-l. Gong, Z-G. Yang; Chengdu/CN

**Purpose:** Osimertinib is approved for treating advanced non-small cell lung cancer (NSCLC) patients with EGFR-T790M mutation after progression on first-line EGFR-TKIs. We aim at investigating the prognostic value of radiomic features in advanced NSCLC patients receiving second-line osimertinib therapy.
**Methods or Background:** Chest CT images before osimertinib treatment were collected from 140 metastatic NSCLC patients with EGFR-T790M mutation. Radiomics features were extracted from the volume of interest. Hierarchical clustering was used to identify patients with different radiomic patterns. Radiomics' ability in predicting progression-free survival (PFS) was analyzed by COX and LASSO COX regression.
**Results or Findings:** The predictive ability of CT morphological features was limited. The PFS-predicting ability was only achieved in 1/11 CT morphological features, i.e. lobulation (HR, 95%CI: 1.94, 1.14-3.32). Hierarchical clustering groups from contrast-enhanced phase can divide patients into two groups of discrepant prognosis (mPFS: 17.0-Mo vs. 11.8-Mo, P=0.007), while clustering groups from unenhanced CT phase shared similar prognosis (mPFS: 12.3-Mo vs. 13.9-Mo, P=0.972). In univariate analyses, sixty-six and eight features from contrast-enhanced and unenhanced CT were predictors of PFS, respectively, indicating great potential of contrast-enhanced CT features in prognostic prediction. We then performed LASSO COX and backward COX regression using contrast-enhanced CT features, and an 8-feature-based radiomic model was developed (C-index: 0.701). Adding the radiomic model to the clinical model can substantially increase its C-index from 0.725 to 0.787 and strengthen its performance in decision curve analysis.
**Conclusion:** We firstly explored the prognostic value of radiomics in advanced NSCLC patients receiving second-line osimertinib therapy. Our study will help doctors in dicision-making process.
**Limitations:** It's a retrospective study.
**Ethics Committee Approval:** This study was approved by West China Hospital Research Ethics Board.
**Funding for this study:** This study was supported by 1·3·5 project for disciplines of excellence, West China Hospital, Sichuan University (ZYGD18013).
**Author Disclosures:**
Yuan Li: Nothing to disclose
Xin Tang: Nothing to disclose
You-ling Gong: Nothing to disclose
Zhi-Gang Yang: Nothing to disclose

### RPS 516-5

**Orbital metastases: common pathologies and imaging pearls**

*J. O'Mahony*, A. Nasoodi, C. Johnston; Dublin/IE
*(omathuns@tcd.ie)*

**Purpose:** Orbital metastases present a diagnostic challenge for the busy radiologist as the imaging features are often subtle, and can be easily overlooked on cross-sectional imaging of the brain or head and neck using routine oncological protocols. Although accounting for only 4% of all adult orbital tumours, orbital metastases must be considered in patients with a history of malignancy. This is particularly relevant in the presence of known metastases, progressive disease, or symptoms of visual impairment, pain on eye movements or proptosis at presentation.
**Methods or Background:** The most common primary malignancies associated with orbital metastases are carcinoma of the breast, prostate, melanoma and lung. We share our experience as one of the major oncology centres in Ireland through a series of illustrative cases from our institution that demonstrate the common imaging appearances of orbital metastases. We also illustrate diagnostic pearls which enable diagnosticians to identify and characterise such lesions more efficiently.
**Results or Findings:** Metastatic lesions may be found within any compartment of the orbit and careful attention must be paid to identify any abnormalities as lesion detection may be difficult on axial imaging alone, particularly in the setting of a routine brain protocol. To this end, adopting a more structured approach with utilisation of multi-planar reconstruction of the images cannot be over-emphasised.
**Conclusion:** Orbital Metastases must be considered in the context of patient with known metastatic disease and may be easily overlooked on routine axial oncological imaging. Careful attention to the orbits with multi-planar reconstruction of images is essential to identification of subtle orbital pathology.
**Limitations:** Primary orbital malignancy not assessed
**Ethics Committee Approval:** Not required
**Funding for this study:** None
**Author Disclosures:**
Afshin Abdollahzadeh Nasoodi: Nothing to disclose
Johnny O'Mahony: Nothing to disclose
Ciaran Johnston: Nothing to disclose

### RPS 516-6

**Dosimetry and biokinetic modelling of thyroid cancer patients treated with radioiodine as part of an international multi-centre study within MEDIRAD**

*J. Taprogge*[1], F. Leek[1], M. Bardiès[2], F. Courbon[2], U. Eberlein[3], M. Lassmann[3], M. Luster[4], D. Vallot[2], G. Flux[1]; [1]Sutton/UK, [2]Toulouse/FR, [3]Würzburg/DE, [4]Marburg/DE

**Purpose:** MEDIRAD investigates the effects of low-dose radiation exposure in the medical field. Work package 3 aims to recruit 100 thyroid cancer patients for a multi-national multi-centre observational clinical study to determine biokinetics of radioiodine in thyroid cancer patients and to measure the range of absorbed doses to healthy tissues and thyroid remnants.
**Methods or Background:** Four European centres were set-up and five SPECT and SPECT/CT systems were calibrated to allow for quantitative imaging of high-activity I-131. Flexible image acquisition protocols were developed based on local requirements and availability of equipment. A modified biokinetic model based on the ICRP-128 I-131 model was implemented in Monolix2019R1. Feasibility of the modelling was assessed using retrospective data of 23 patients for whom biokinetic data was available for thyroid remnant, blood, protein-bound-iodine and whole body.
**Results or Findings:** Five SPECT or SPECT/CT systems were set up for quantitative imaging with system volume sensitivities ranging from 62 to 92 cps/MBq. 87 patients have been recruited for the study to date. The modified ICRP-128 model was able to accurately reproduce the activity retention of the retrospective data. The modified model has slower transfer rate constants from blood to thyroid (0.12 day-1) compared to ICRP-128 (7.26 day-1). Thyroid to blood transfer in both models was found to be comparable (30 day-1 and 36 day-1).
**Conclusion:** A network of centres able to perform quantitative I-131 imaging was set-up to support a series of clinical studies and recruitment is ongoing. Initial results have shown that iodine bio-kinetics change after thyroidectomy. The model will be further adapted using data acquired as part of the multi-centre multi-national prospective study.
**Limitations:** n/a
**Ethics Committee Approval:** Obtained.
**Funding for this study:** The MEDIRAD project received funding from the Euratom research and training programme 2014-2018 under grant agreement No 755523.
**Author Disclosures:**
Delphine Vallot: Nothing to disclose
Manuel Bardiès: Consultant: Theragnostics Consultant: IPSEN Consultant: MEDPACE
Michael Lassmann: Research/Grant Support: IPSEN
Frederic Courbon: Nothing to disclose
Glenn Flux: Nothing to disclose
Jan Taprogge: Nothing to disclose
Francesca Leek: Nothing to disclose
Markus Luster: Nothing to disclose
Uta Eberlein: Nothing to disclose

### RPS 516-7

**CT based radiomic approach on first line Pembrolizumab in lung cancer**

*E. Lucertini*, M. Zerunian, G. Guido, T. Polidori, D. Caruso, C. Rucci, F. Pucciarelli, M. Polici, A. Laghi; Rome/IT

**Purpose:** To assess whether CT-derived texture parameters extrapolated from baseline examinations can predict overall survival (OS) and progression-free survival (PFS) in patients with advanced non-small-cell lung cancer (NSCLC) treated with Pembrolizumab as a first line.
**Methods or Background:** Twenty-one patients with NSCLC were prospectively enrolled; all participants underwent contrast enhanced CT (CECT) at baseline and during Pembrolizumab treatment. Response to therapy was assessed both with clinical assessment and iRECIST criteria. Two radiologists in consensus drew a volume of interest of the tumor at baseline CECT on portal-venous phase, extracting the following first level histogram based texture parameters: mean, standard deviation, entropy, kurtosis, skewness and mean value of positive pixels (MPP). ROC curves, a univariate Kaplan-Meyer analysis and Simple Cox proportional hazard regression analysis were performed to evaluate the prognostic value of texture analysis.
**Results or Findings:** Twelve (57%) patients showed partial response to therapy while nine (43%) had confirmed progressive disease. Among texture parameters MPP at fine and medium filter showed an AUC of 72% and 74% respectively (P<0.001). Kaplan-Meyer analysis showed that MPP under 56.2 were significantly associated with lower OS and PFS in patients with advanced NSCLC treated with Pembrolizumab (P <0.0035). Further, Cox proportional analysis showed a significant correlation between MPP4 and OS (P=0.0038; HR=0.89 [CI 95%:0.83,0.96]).
**Conclusion:** MPP could be used as predictive imaging biomarker of OS and PFS in patients with NSCLC with first line immune treatment.
**Limitations:** The study has a small population sample. No test validation and no multivariate analysis have been performed.
**Ethics Committee Approval:** After the approval of the institutional ethics committee, all patients have accepted and signed the written informed consent.
**Funding for this study:** No sources of funding have been used for this study.
**Author Disclosures:**
Damiano Caruso: Nothing to disclose
Francesco Pucciarelli: Nothing to disclose
Carlotta Rucci: Nothing to disclose
Marta Zerunian: Nothing to disclose
Tiziano Polidori: Nothing to disclose
Andrea Laghi: Nothing to disclose
Michela Polici: Nothing to disclose
Gisella Guido: Nothing to disclose
Elena Lucertini: Nothing to disclose

### RPS 516-8

**Dual-energy CT assessment of skeletal bone marrow involvement in multiple myeloma**

*R. Gu*[1], A. Amlani[1], O. A. Westerland[1], A. Green[1], U. Haberland[2], M. Streetly[1], M. Antonelli[1], I. Dregely[1], V. Goh[1]; [1]London/UK, [2]Frimley Camberley/UK
*(renyang.gu@kcl.ac.uk)*

**Purpose:** In multiple myeloma, accurate and objective assessment of skeletal bone marrow involvement would enable more precise monitoring of disease burden. We aimed to develop a quantitative dual-energy CT approach for assessing whole skeletal disease burden and to correlate CT with clinical metrics.
**Methods or Background:** Consecutive patients with suspected or confirmed myeloma underwent CT from vertex to below knees on a third-generation dual-source scanner, generating 120 kVp-equivalent images and corresponding three-material decomposition calcium-subtracted attenuation maps. The skeleton was segmented on the 120 kVp-equivalent images using Hounsfield unit (HU) thresholding and application of a Chan-Vese morphological operation. Skeletal marrow attenuation values were generated from the corresponding calcium-subtracted maps using the segmented marrow mask overlay. Associations of whole skeleton calcium-subtracted attenuation with bone marrow biopsy infiltration percentage, haemoglobin level and age were assessed with Spearman's rank correlation; significance was at 5%.
**Results or Findings:** 20 patients (11 females; median 68 years; 12 relapsed, 8 new myeloma) were evaluated; 14 patients had subsequent bone marrow biopsy. Median (IQR) of mean skeletal attenuation was -56.0 HU (-65.6, -44.9HU). There was a strong positive correlation with bone marrow plasma cell infiltration percentage (Spearman's rho: + 0.91, p < 0.001); a negative correlation with haemoglobin level (Spearman's rho: -0.77, p < 0.001) but no correlation with age (Spearman's rho: -0.19, p = 0.4).
**Conclusion:** Whole skeletal assessment is feasible and associated with the degree of bone marrow plasma cell infiltration. This may provide an additional objective measure of marrow disease burden to the current assessment of focal lytic bone lesions.
**Limitations:** It was an exploratory study with a small number of patients.
**Ethics Committee Approval:** REC number 13/EM/0409.
**Funding for this study:** No funding.
**Author Disclosures:**
Adrian Green: Nothing to disclose
Matthew Streetly: Nothing to disclose
Vicky Goh: Research/Grant Support: Siemens Healthcare
Ulrike Haberland: Employee: Siemens Healthcare
Renyang Gu: Nothing to disclose
Olwen Amy Westerland: Nothing to disclose
Ashik Amlani: Nothing to disclose
Isabel Dregely: Nothing to disclose
Michela Antonelli: Nothing to disclose

### RPS 516-9

**Development of perfusion CT and its importance for evaluating pancreatic diseases: a quantitative meta-analysis**

*N. Vats*, H-U. Kauczor, W. Stiller, S. Skornitzke; Heidelberg/DE
*(neha.vats@med.uni-heidelberg.de)*

**Purpose:** This meta-analysis evaluated changes in perfusion computed tomography (pCT) acquisition protocols over ~40 years, assessing its potential to differentiate pancreatic diseases.
**Methods or Background:** 491 pCT studies from 1982-2020 gathered from PubMed using key terms (pancreas/pancreatic, dynamic/perfusion, and computed tomography/CT) were included. 50 studies met inclusion criteria: original data, human study, at least 5 datasets. Sample size, CT acquisition parameters, and quantitative perfusion parameters (Blood flow (BF), Blood volume (BV) and permeability) were extracted. Year-wise mean acquisition parameters were calculated and linear regression plots computed over the years. Perfusion measurements were grouped into 4 categories of pathology: normal pancreas (control), pancreatitis, carcinoma, and non-carcinoma. Weighted-ANOVA followed by Student's t-test was performed for comparing quantitative measurements for different pathologies.
**Results or Findings:** Over the years, pCT acquisition parameters varied considerably, with the number of studies and sample size increasing after 2010. Mean tube potential and current-time product have slightly decreasing (2010-2014:92±13.0kVp, 2015-2019:88.8±5.0kVp) and increasing (2010-2014:101.4±51.4mAs, 2015-2019:150.9±68.7mAs) trends respectively, with tissue coverage also increasing, but without a significant trend in effective dose. Lowest averaged perfusion measurements were found in carcinoma (BF: 29.3±10.9ml/100ml/min, BV: 9.5±8.9ml/100ml, permeability: 23.7±10.8ml/100ml/min) compared to non-carcinoma (BF: 109.6±39.9ml/100ml/min, BV: 14.4±7.7ml/100ml, permeability: 30.2±16.5ml/100ml/min), pancreatitis (BF: 64.0±22.4ml/100ml/min, BV: 3.1±5.0ml/100ml, permeability: 33.7±15.6ml/100ml/min), and control (BF: 100.9±30.9ml/100ml/min, BV: 20.0±6.0ml/100ml, permeability:

36.9±13.4ml/100ml/min). Weighted-ANOVA shows BF (p<0.0001) and BV (p<0.0001) differ significantly between pancreatic pathologies but permeability does not (p=0.1131). Student's t-test shows significant differences between control/pancreatitis, control/carcinoma, and carcinoma/non-carcinoma for BF and BV, and between pancreatitis/carcinoma, and pancreatitis/non-carcinoma for BF.
**Conclusion:** Results illustrate the need for standardization of pCT. Quantitative perfusion parameters (BF and BV) can differentiate between pancreatic pathologies. Thus, pCT could qualify as a biomarker for diagnosing pancreatic diseases.
**Limitations:** Limited number of pancreatic pCT studies reporting perfusion parameters
**Ethics Committee Approval:** Not applicable
**Funding for this study:** BMBF-grant 031L0163
**Author Disclosures:**
Neha Vats: Nothing to disclose
Wolfram Stiller: Nothing to disclose
Hans-Ulrich Kauczor: Nothing to disclose
Stephan Skornitzke: Nothing to disclose

### Research Presentation Session: Emergency Imaging

### RPS 317

### Developments in emergency imaging

### RPS 317-1

**Pseudo-pneumatosis of the gastrointestinal tract: its incidence and the accuracy of a checklist developed by artificial intelligence (AI) techniques to reduce the misinterpretation of pneumatosis**

*A. A. Esposito*[1], L. Castoldi[1], S. Zannoni[1], E. Casiraghi[1], C. Giannitto[1], E. Avola[1], O. Catalano[2], G. Carrafiello[1]; [1]Milan/IT, [2]Boston, MA/US
*(andrea.esposito@policlinico.mi.it)*

**Purpose:** To assess the incidence of pseudo-pneumatosis in patients who underwent an emergency abdominal CT and to verify the performance of imaging features, selected by artificial intelligent techniques, to reduce this misinterpretation.
**Methods or Background:** We searched for patients who underwent an emergency abdominal CT between 2012 and 2019 and whose radiological reports contained the word pneumatosis, retrieving 71 patients. Surgical findings, clinical outcomes and reevaluation of the CT scans, were used as reference standard. Five imaging signs were considered useful for differentiating pneumatosis versus pseudo-pneumatosis: gas location, dissecting gas in the bowel wall edge, a circumferential gas pattern, intramural gas beyond a gas-fluid/faecal level, the presence of alterations associated with bowel ischemia. An unbiased performance evaluation on individual features was obtained by training and testing decision trees through a Leave One Out (LOO) strategy. Most accurate variables (accuracy >75%) were selected, and used to train and test boosted Random Forest classifiers through LOO.
**Results or Findings:** Of the 71 patients, 27 resulted as pseudo-pneumatosis (38% of error). The most significant features to diagnose pneumatosis were: the presence of dissecting gas (96% of accuracy), the presence of intramural gas beyond a gas-fluid/faecal level (86% of accuracy), the presence of at least one alteration associated with bowel ischemia (86% of accuracy), a circumferential gas pattern (78% of accuracy). When pooled the selected variables obtained 96% of accuracy.
**Conclusion:** In patients who undergo an emergency abdominal CT, the incidence of pseudo-pneumatosis can be high. The use of a checklist including four imaging signs, can be useful to reduce this overestimation.
**Limitations:** The low number of patients.
It is a retrospective monocentric study
**Ethics Committee Approval:** The study was approved by the Ethics Committee, and patient's informed consent was waived for its retrospective nature.
**Funding for this study:** No.
**Author Disclosures:**
Onofrio Catalano: Nothing to disclose
Caterina Giannitto: Nothing to disclose
Laura Castoldi: Nothing to disclose
Andrea Alessandro Esposito: Nothing to disclose
Stefania Zannoni: Nothing to disclose
Elena Casiraghi: Nothing to disclose
Emanuele Avola: Nothing to disclose
Gianpaolo Carrafiello: Nothing to disclose

### RPS 317-2

**Soft tissue assessment on multidetector computed tomography (MDCT) can improve imaging classification of incomplete pelvic ring injuries: a cohort study**

*L. Gudauskas*, R. Karthikeyan, S. Choudhary, R. Rajakulasingam, S. Balamoody; Birmingham/UK

**Purpose:** Pelvic soft tissue assessment, in addition to fracture patterns on computed tomography (CT) can improve identification and classification of pelvic ring injuries in patients with polytrauma.
**Methods or Background:** A cohort of consecutive patients with incomplete pelvic ring injuries who underwent examination under anaesthesia were identified from the operative database of an adult major trauma centre over 10 years. Pelvic CT scans were retrospectively reviewed for signs of pelvic ligament injury. The findings assessed were - obscured retropubic fat plane, thickening of symphyseal ligaments, symphysis pubis width, ischial spine and sacral avulsion fractures, thickening of sacrospinous and sacrotuberous ligaments, obscured anterior and posterior sacroiliac fat plane and sacroiliac joint width. Pelvic ring injury was classified according to Young- Burgess classification and compared with operative findings.
**Results or Findings:** Obscured retropubic fat plane and symphysial ligament thickening identified pubic symphysial injury at CT, irrespective of the presence of a pelvic binder and degree of diastasis (APC type 1, n=15). Assessment of posterior pelvic ligaments and pre-defined fat planes allowed classification of APC type 2 (n=13) and type 3 (n=3) injuries at imaging, which were confirmed with operative findings. Sacral bony avulsion was the commonest pattern of sacrospinous and sacrotuberous ligament injury.
**Conclusion:** Soft tissue assessment can identify pelvic ring injury on CT scans, when diastasis may not be readily apparent with a pelvic binder. Pelvic ring ligaments should be assessed in addition to fractures when interpreting CT scans in polytrauma patients, to improve identification and classification of pelvic ring injuries.
**Limitations:** Retrospective study design. Patients with pelvic trauma not included if they did not have examination under anaesthesia or operative intervention.
**Ethics Committee Approval:** Approved by Clinical Governance Board, formal Ethics Committee review not required.
**Funding for this study:** None.
**Author Disclosures:**
Sharon Balamoody: Nothing to disclose
Surabhi Choudhary: Nothing to disclose
Ravichandran Karthikeyan: Nothing to disclose
Linas Gudauskas: Nothing to disclose
Ramanan Rajakulasingam: Nothing to disclose

### RPS 317-3

**Effects of the COVID-19 lockdown on the radiological examination outcomes**

*V. Vingiani*[1], A. Posa[2], A. F. Abadia[3], G. Belmonte[4], C. R. Rutigliano[5], L. Pasqualetto[1], A. Presidente[1], M. Catalano[2], G. B. Berte[2]; [1]Sorrento/IT, [2]Rome/IT, [3]Denver, CO/US, [4]Potenza/IT, [5]Andria/IT
*(vincenzovingiani@gmail.com)*

**Purpose:** The aim of this multicentric study is to illustrate how the COVID-19 pandemic lockdown affected the workload and outcomes of radiological examinations in emergency radiology.
**Methods or Background:** The exams performed in the radiology departments of four Italian hospitals during three weeks of the Italian lockdown were retrospectively reviewed and compared to the exams conducted during the same time-period in 2019. Only exams coming from the emergency department (ED) were included. Two radiologists from each hospital defined the cases as positive or negative findings, based on independent blind readings of the imaging studies and correlation with the clinical indication. In case of differences in the evaluation, consensus was reached amongst them via discussion. Continuous measurements are presented as median and interquartile range, while categorical measurements are presented as frequency and percentages; p-values were calculated using T-test, Mann-Whitney test and the Chi-squared test.
**Results or Findings:** There were 745 patients [53% male; 62 years (44-78)] who underwent radiological examinations in 2020 vs. 2623 [52% male; 56 years (35-76)] in 2019 (p<.001). Furthermore, the total number of ED exams dropped from 3206 (2019) to 939 (2020), with a relative increase of CT examinations from 23% to 33% (p<.001). The percentage of patients with a positive finding was significantly higher in 2020 (355, 48%) compared to 2019 (684, 26%) (p<.001).
**Conclusion:** Our findings showed that despite the reduction of emergency radiological examinations there was a relative increase of positive cases. These significant findings are crucial to ensure better organization of radiology department and patient management in similar health emergencies in the future.

**Limitations:** Study results might change according to the level of lockdown applied.
**Ethics Committee Approval:** Institutional review boards approved this retrospective multi-center study.
**Funding for this study:** Not applicable.
**Author Disclosures:**
Giovanna Berte Bertè: nothing to disclose
Gianfranco Belmonte: Nothing to disclose
Claudia Rutigliano Rutigliano: nothing to disclose
Alfonso Presidente: Nothing to disclose
Alessandro Posa: nothing to disclose
Andres Felipe Abadia: nothing to disclose
Luigi Pasqualetto: nothing to disclose
Marialavinia Catalano: nothing to disclose
Vincenzo Vingiani: nothing to disclose

### RPS 317-4

**Bleeding haematomas in COVID-19 patients related to fibrinogen levels and anticoagulation**

*V. Picasso*, L. Ball, F. Rapetti, A. Pezzino, G. Bovio, P. Pelosi, L. E. Derchi; Genoa/IT
*(virgi.picasso@gmail.com)*

**Purpose:** Bleeding/non bleeding hematomas in Covid 19 patients, correlated to the degree of anticoagulation and fibrinogen levels.
**Methods or Background:** Major arterial or venous thromboembolism occurs frequently in patients with severe Covid-19 disease, especially in the ICU setting. Anticoagulant therapy is given to these patients, with Its inherent risks. We reviewed the contrast enhanced CT studies of 100 patients admitted to the ICU and emergency room between February 29th and May 30th, 2020 for COVID disease to identify presence of active bleeding and/or hematomas.
**Results or Findings:** At the time of CT scan, most patients were treated with anticoagulant therapy (low molecular weight heparin – LMWH – every 24h: 33; LMWH every 12h: 30; sodium heparin in continuous infusion: 33, no therapy: 4). Ninety-one (91%) studies had been requested for pulmonary assessment or for suspected pulmonary arteries thrombosis, 9 (9%) for suspected bleeding. We observed 24 (24%) cases of hematoma in thoracoabdominal regions, of which 13 were located in the deep abdominal muscles in the retroperitoneal area, 10 were actively bleeding and required treatment.
**Conclusion:** Patients with versus those without hematomas had comparable D-Dimer levels (1167 vs. 1576 mcg/L, p=0.107) and anticoagulation regimens (p=0.276). Mortality was similar in patients with and without bleeding at the CT scan (37.5% vs. 42.1%, p=0.891).
**Limitations:** None
**Ethics Committee Approval:** N/a
**Funding for this study:** N/a
**Author Disclosures:**
Virginia Picasso: Nothing to disclose
Francesca Rapetti: Nothing to disclose
Giulio Bovio: Nothing to disclose
Paolo Pelosi: Nothing to disclose
Lorenzo E. Derchi: Nothing to disclose
Lorenzo Ball: Nothing to disclose
Angelo Pezzino: Nothing to disclose

### RPS 317-5

**Prognostic value of the initial chest radiography in patients admitted with SARS-CoV-2 pneumonia**

*J. M. Plasencia Martinez*, I. Herves Escobedo, M. Martín-Cascón, A. Blanco Barrio, J. M. Gómez-Verdú, S. Alemán-Belando, C. Jiménez Pulido, A. Moreno Pastor, I. M. González Moreno; Murcia/ES
*(plasen79@gmail.com)*

**Purpose:** To determine the chest X-ray (CXR) ability to predict the outcome in COVID-19 hospitalized patients.
**Methods or Background:** Brixia-Score (BS) and bilateral pneumonia (BN) in the initial CXR, inflammatory laboratory biomarkers, SaO2/FiO2 ratio, ARDS, death, non-invasive mechanical ventilation (NIMV) and intensive care unit (ICU) admissions were assessed in hospitalized patients with SARS-CoV-2 pneumonia in the first pandemic wave by intraclass correlation coefficient (ICC), Chi2, Student t and Spearman tests.
**Results or Findings:** 117 patients (62 men-53%-), age 57.03±15.47 years, were admitted between March-13 and April-27, 2020. 38/117 (32.5%) patients suffered from ARDS, 12/117 (10.3%) needed NIMV and 18/117 (15.4%) were admitted to ICU or died. The BS-ICC was 0.833 (95CI%0.752-0.887). BS was greater in males (6.03±3.42 vs 4.78±3.04, P=0.04) and older patients (61.47±13.91 -BS>5- vs 53.18±15.58 -BS≤5- years; P=0.003). BS>5 patients had higher ferritin (862±744 vs 562±648; P=0.04), lactate-dehydrogenase (658±250 vs 512±210, P<0.001) and C-reactive protein (CRP; 11±10 vs 7±7; P=0.006) and lower lymphocyte (0.78±0.48 vs 1.1±0.67x103; P<0.001) levels. CRP (10.4±9 vs 6.38±7.3; P=0.027) and lymphocyte (0.85±0.55x103 vs 1.2±0.71x103; P<0.001) levels worsened for BN. BS and SaO2/FiO2 ratio correlated moderately (R=-0.435; P<0.001). BS>5 (23/38-60%- vs 28/78-35.9%-; OR 2.7-95CI%1.2-6.1-, P=0.012) and BN (32/38-84%- vs 48/74-69.5%-; OR 2.88-95CI%1.07-7.81-, P=0.032) were more frequent in ARDS. BS>5 (vs≤5) led to earlier hospital (symptoms-onset-to-hospital-admission days: 7.82±3.9 vs 9.75±6.86; P=0.06) and ICU (hospitalization-to-ICU days : 2±1.4 vs 4.2±1.9; P=0.04) admissions, and longer hospital stay (9.1±6.5 vs 6.0±4.9 days; P=0.004), but BN (8.8±5.0 vs 9.4±7.4; P=0.644; 3±2.1 vs 3.0±0.0; P=1; and 8.2±5.9 vs 6.0±5.4; P=0.083, respectively) did not.
**Conclusion:** CXR for SARS-CoV-2 pneumonia diagnosis can be an early, inexpensive, and reproducible biomarker of the later systemic inflammatory response, hospital stay, prompt hospitalization and ICU admission need, providing a timely advantage.
**Limitations:** Small sample
**Ethics Committee Approval:** Yes
**Funding for this study:** No
**Author Disclosures:**
Sergio Alemán-Belando: Nothing to disclose
Juana María Plasencia Martinez: Nothing to disclose
Isabel María González Moreno: Nothing to disclose
Ignacio Herves Escobedo: Nothing to disclose
Ana Moreno Pastor: Nothing to disclose
Miguel Martín-Cascón: Nothing to disclose
José Miguel Gómez-Verdú: Nothing to disclose
Ana Blanco Barrio: Nothing to disclose
Cristina Jiménez Pulido: Nothing to disclose

### RPS 317-6

**Improved diagnostic accuracy of computer-aided peripheral embolism detection by CTPA on virtual monochromatic images compared to conventional polychromatic images**

*E. Langius*[1], I. M. Nijholt[1], E. De Boer[1], J. Nijboer-Oosterveld[1], S. Walen[1], J. W. van den Berg[1], P. A. de Jong[2], M. F. Boomsma[1]; [1]Zwolle/NL, [2]Utrecht/NL
*(elinelangius@gmail.com)*

**Purpose:** Pulmonary embolism (PE) is a potentially life threatening condition and therefore requires quick diagnosis and treatment. In this study, the diagnostic accuracy of computer-aided PE detection by computed tomography pulmonary angiography (CTPA) on conventional polychromatic images (CPI) was compared with virtual monochromatic images (VMI) retrospectively obtained with a detector-based spectral CT scanner (SDCT).
**Methods or Background:** Paired CPI and 60 keV VMI of 113 consecutive patients suspected of PE were independently evaluated by two radiologists using computer aided PE detection software (CAD). Sensitivity and specificity of the radiologists on VMI and CPI were compared using the McNemar test. In addition, the odds of false positive (FP) PE CAD markers were determined on VMI and CPI. The image quality of VMI and CPI was compared by determining signal-to-noise ratio (SNR) and contrast-to-noise ratio (CNR).
**Results or Findings:** The number of FP CAD markers per scan on VMI was significantly reduced compared to the number per scan on CPI (p < 0.001). The odds of FP PE markers also decreased significantly on VMI (OR 0.51, p <0.001). Improved image quality of VMI compared to CPI was found to largely explain the lower odds of false positive PE markers on VMI. All diagnostic measures of radiologists using CAD to detect PE improved on VMI vs. CPI, albeit not always statistically significant.
**Conclusion:** Diagnostic accuracy of computer aided PE detection on CTPA improved on VMI compared to routinely used CPI. This can largely be attributed to improved image quality on VMI.
**Limitations:** Reference standard not an absolute standard of truth. Radiologists blinded to clinical information, not image type. The mportance of detecting peripheral emboli is unclear.
**Ethics Committee Approval:** Approved by the local Institutional Review Board of Isala (no. 190209).
**Funding for this study:** Funded by Isala and UMCU.
**Author Disclosures:**
Martijn F. Boomsma: Nothing to disclose
Erwin De Boer: Nothing to disclose
Jacqueline Nijboer-Oosterveld: Nothing to disclose
Stefan Walen: Nothing to disclose
Jan Willem van den Berg: Nothing to disclose
Eline Langius: Nothing to disclose
Pim A. De Jong: Nothing to disclose
Ingrid M. Nijholt: Nothing to disclose

### Research Presentation Session: Radiation Protection / EuroSafe Imaging

### RPS 200

### Professional issues: radioprotection

### RPS 200-1

**European Diagnostic Reference Levels (DRLs) for Computed Tomography (CT) applications in nuclear medicine: results from the MEDIRAD project**

*G. Verfaillie*, Y. D'Asseler, K. Bacher; Ghent/BE
*(Gwenny.Verfaillie@UGent.be)*

**Purpose:** Unlike in diagnostic radiology, published national Diagnostic Reference Levels (DRLs) in Europe for CT acquisitions used in hybrid imaging are limited. This study aims to propose the first European DRLs for specific applications of CT in hybrid SPECT/CT and PET/CT.
**Methods or Background:** As part of the EU Horizon 2020 MEDIRAD project, a CT dosimetry survey was conducted from October 2018 until February 2020. Participating nuclear medicine departments were asked to provide patient-specific information (gender, age, length and weight) and CT dose indicators (CTDIvol, DLP) from the most frequently performed diagnostic SPECT/CT and PET/CT examinations. Per scan type, data from 30 adult patients (weight of 70 ± 10 kg) were collected. European DRLs were defined as the 75th percentile value from the distribution of the median CTDIvol and DLP values.
**Results or Findings:** Data were received from 50 nuclear medicine departments in 20 European countries. As expected, CT doses were lower for attenuation correction than for localisation or diagnostic hybrid CT scans. Suggested European DRLs for CTDIvol (and DLP) of 18F-FDG half body PET/CT examinations are 2.5 mGy (230 mGy.cm) and 6.3 mGy (580 mGy.cm) for attenuation correction only and localisation CT scans, respectively. A value of 6.4 mGy (160 mGy.cm) is proposed for attenuation correction only 18F-FDG brain PET/CT studies. For 99mTc-bone (trunk) and parathyroid SPECT/CT examinations, a DRL of 4.4 mGy (160 mGy.cm) for attenuation correction and localisation CT scans is suggested. Finally, the proposed European DRL for attenuation correction CT scans of 99mTc-cardiac SPECT/CT studies is 3.1 mGy (60 mGy.cm).
**Conclusion:** European DRLs for typical CT applications in nuclear medicine were derived. The large variations in observed CT doses highlights the need for optimisation.
**Limitations:** n/a
**Ethics Committee Approval:** n/a
**Funding for this study:** H2020 Euratom Grant ID 755523
**Author Disclosures:**
Gwenny Verfaillie: Nothing to disclose
Klaus Bacher: Nothing to disclose
Yves D'Asseler: Nothing to disclose

### RPS 200-2

**A comparison of Computed Tomography (CT) justification practices between core hours and on-call hours in Irish CT centres**

*A. Dowley*, S. J. Foley, J. Potočnik, T. Thiel, M-L. Ryan; Dublin/IE
*(anna.dowley@ucdconnect.ie)*

**Purpose:** Radiation dose from CT scans are among the highest in diagnostic imaging. In Ireland, CT scans account for 5.7% of examinations but contribute to 55% of the collective dose. There is a legislative requirement to justify CT examinations. Also, health service resources are stretched and appropriate use of CT is crucial to maximise efficiency. The purpose of this study was to audit justification of CT examinations in Irish CT departments, during standard working and on-call hours.
**Methods or Background:** CT referrals (six common exam types) were randomly sampled from a three-month period in 2019 from five public Irish hospitals. Two experienced radiographers independently assessed referrals for adequate completion and justification in accordance with UK iRefer guidelines (2), with any disagreements being addressed by a third radiographer. Each referral was further assessed by a consultant radiologist (20+ years experience) for justification in an on-call setting.
**Results or Findings:** 1158 CTs were reviewed, of which 488 of which were performed on-call. 11% of exams were not justified- 12% during working hours and 9% during on-call times. Justification per anatomical region varied widely- 28% of CT pulmonary angiogram scans were unjustified, compared to 3% of Kidney, Ureter and Bladder CTs. Similarly, justification between hospitals varied widely, from 8% of scans being unjustified up to 21%. 37% of scans were deemed inappropriate for completion during on-call hours. 43.8% of justified exams had a clinical finding related to the reason the scan was completed, compared with 20.0% findings for unjustified exams.
**Conclusion:** Notable differences exist in CT justification between working and on-call hours, among different examination types and between hospitals. Continued efforts in justification can save both resources and unnecessary radiation dose.
**Limitations:** Limit in examination numbers.
**Ethics Committee Approval:** Approved by institutional ethics review.
**Funding for this study:** None.
**Author Disclosures:**
Thomas Thiel: Nothing to disclose
Jaka Potočnik: Nothing to disclose
Marie-Louise Ryan: Nothing to disclose
Shane J Foley: Nothing to disclose
Anna Dowley: Nothing to disclose

### RPS 200-3

**Performance evaluation of Computed Tomography (CT) examinations at highest radiation doses**

*I. Fitton*, E. Charpentier, C. Van Ngoc Ty, O. Clement; Paris/FR
*(isabelle.fitton@aphp.fr)*

**Purpose:** Dose data and image scoring were chosen to be representative and appropriate as indicators of CT exams performance to evaluate professional practices in CT.
**Methods or Background:** The highest radiation dose levels (HRDL) were determined and set on two CT scanners (Siemens SOMATOM® Definition Edge and Siemens SOMATOM® Force) dedicated to emergency and conventional examinations. HRDL values were fixed by calculating the 99.5th percentile of DLP distributions for all CT exams acquired during 4 months on each CT equipment. CT examinations exceeding HRDL were collected over 15 months. Clinical practices checking were based first, on the interaction with the CT console by the team by studying the dose reduction difference between actual and projected total DLP for three thresholds at 5 %, 10 %, and 15 %. Second, image quality and diagnostic performance were visually assessed by one reader on a 5-point-Likert scale.
**Results or Findings:** Over 30038 exams, 153 exams were superior to HRDL. We observed that 33 % of these patients had a dose reduction higher than 5 %. Mean image score was 3.7 ± 1.1. 3.26% of CT exams higher than HDRL were not interpretable.
**Conclusion:** Analyzing CT examinations of patients exposed to the highest radiation doses is an adequate approach for evaluating professional practices in radiology.
**Limitations:** Some patient morphology data or examination indication, for example, overweight or patient suffering from polytrauma, had an impact on the HRDL triggering without possibilities to interact with the CT console for improving image quality or dose.
**Ethics Committee Approval:** Retrospective study. Submitted to the ethic committee of our institution.
**Funding for this study:** This work was awarded by The French Society of Radiology and SHAM.
**Author Disclosures:**
Etienne Charpentier: Nothing to disclose
Isabelle Fitton: Nothing to disclose
Olivier Clement: Nothing to disclose
Claire Van Ngoc Ty: Nothing to disclose

### RPS 200-4

**Out-of-field patient shielding in medical imaging: current legislative documents and recommendations reviewed by EURADOS WG12**

O. Ciraj Bjelac[1], *H. de las Heras Gala*[2], C. Candela-Juan[3], J. Dabin[4], D. Faj[5], A. Gallagher[6], F. de Monte[7], G. Simantirakis[8], M. Sans Merce[9]; [1]Belgrade/RS, [2]Oberschleissheim/DE, [3]Valencia/ES, [4]Mol/BE, [5]Osijek/HR, [6]Limerick/IE, [7]Padova/IT, [8]Athens/GR, [9]Geneva/CH

**Purpose:** The objective of this work is to extensively review current national legislation and recommendations on the use of out-of-field shielding in x-ray imaging, including those from national authorities and professional bodies as well as international organizations.
**Methods or Background:** The review was performed within the activities of EURADOS Working Group 12 covering available recommendations for adult, pregnant and paediatric patients in radiography, fluoroscopy, computed tomography, mammography and dental radiology. It included a comprehensive search of 80 legislative documents and recommendations from 34 countries and 6 international organizations.
**Results or Findings:** Some national legislation does not explicitly mention patient shielding, or do not state in which cases it should be used. Other national documents (such as guidelines and publications of professional bodies) explicitly consider the use of patient shielding as a means of good practice or require its availability without further specification of its use. When patient shielding is recommended, it is only suggested if it does not compromise the diagnostic task (e.g. it does not interfere with the primary beam or influences the automatic exposure control system), or if it serves to reassure the patients and comforters. In addition, the documents often mention that shielding should only be considered if organs at risk are within 5 cm from the field edge. Among those documents explicitly considering this subject, between 29 % (in computed tomography) and 43 % (in dental radiology) discourage the use of patient shielding for that modality or in general.
**Conclusion:** Very large discrepancies exist between recommendations, even from organizations of the same country. A common position seems advisable for the efficient information of the public and the proper education of professionals.
**Limitations:** The review mainly includes European countries.
**Ethics Committee Approval:** Not required
**Funding for this study:** EURADOS Working Group 12
**Author Disclosures:**
Jeremie Dabin: Nothing to disclose
Olivera Ciraj Bjelac: Nothing to disclose
Dario Faj: Nothing to disclose
Hugo de las Heras Gala: Nothing to disclose
Georgios Simantirakis: Nothing to disclose
Chrysoula Theodorakou: Nothing to disclose
Cristian Candela-Juan: Nothing to disclose
Francoise Louise Malchair: Nothing to disclose
Francesca de Monte: Nothing to disclose
Aoife Gallagher: Nothing to disclose
Marta Sans Merce: Nothing to disclose

### RPS 200-5

**Comparison of peak skin dose assessment between RDIM software and radiochromic film measurements in interventional procedures: impact of the patient's BMI**

*P. E. Colombo*[1], M. M. J. Felisi[1], S. Riga[1], D. Curto[1], C. Dillion[2], S. Massey[2], F. Barbosa[1], A. Rampoldi[1], A. Torresin[1]; [1]Milan/IT, [2]Scottsdale, AZ/US
*(paolae.colombo@ospedaleniguarda.it)*

**Purpose:** To evaluate the accuracy of the algorithm that calculates the skin dose distribution in interventional radiology procedures, provided by the radiation dose index monitoring software NEXO[DOSE]® (Bracco Injeneering SA, Lausanne).
**Methods or Background:** To calculate the skin dose, the software uses exposure parameters taken from the Radiation Dose Structured Report, angiographic system information and other factors, such as backscatter factor. The software had been previously validated on phantoms. GafChromic® XR-RV3 films were positioned under the patient's back. 22 interventional procedures (including prostatic artery embolization, uterine fibroid embolization, transjugular intrahepatic portosystemic shunt, endovascular aneurysm repair, transcatheter aortic valve implantation and transarterial chemoembolization) were performed in three angiographic rooms with two Philips Integris Allura FD20 and a Siemens Artis Zeego. The Body Mass Index (BMI) was evaluated for each patient. Student's t-test was used to compare peak skin dose (PSD) differences for patients with different BMI.
**Results or Findings:** The PSD values range from 0.18 Gy to 9.90 Gy. The mean deviation between estimated and measured PSD is -4%±14%. For patients with BMI>25 kg/m2 the mean of the absolute PSD differences is 0.44±0.12 Gy, while for patients with BMI<25 kg/m2 it is 0.11±0.01 Gy.
**Conclusion:** We observed a good correlation (r=0.980, p<0.0001) between film measurements and software estimates even if a significant dependence on the patient's BMI was observed (p<0.05). This study suggests that NEXO[DOSE]® software can be considered as a valuable dose calculation tool to optimize existing practices and to monitor the follow-up required for patients who have received high doses. A significant improvement can come from using personalized phantoms that consider the increase of scatter radiation in the case of higher BMI values.
**Limitations:** Limited number of cases.
**Ethics Committee Approval:** Approved by the Institutional Review Board (596-13102020).
**Funding for this study:** No funding.
**Author Disclosures:**
Steve Massey: Employee: PACSHealt, LLC
Colby Dillion: Employee: PACSHealt, LLC
Fabiane Barbosa: Nothing to disclose
Denise Curto: Nothing to disclose
Alberto Torresin: Nothing to disclose
Marco Maria Jacopo Felisi: Nothing to disclose
Paola E. Colombo: Nothing to disclose
Antonio Rampoldi: Nothing to disclose
Stefano Riga: Nothing to disclose

### RPS 200-7

**Dose reduction potential using patient-specific prefilters in single-energy Computed Tomography (CT) within the performance limits of current diagnostic CT systems**

*J. Steidel*, L. Klein, J. Maier, S. Sawall, M. Kachelrieß; Heidelberg/DE
*(joerg.steidel@dkfz.de)*

**Purpose:** To assess the dose reduction potential of patient-specific prefilters within the limitations of current diagnostic single energy CT systems.
**Methods or Background:** CT scans of child, adult and obese abdomen phantoms were simulated with tube voltages between 35 and 150 kV with tin (0 to 2 mm) and copper (0 to 5 mm) prefilters in 0.1 mm steps. Tube current modulation was on for all scans. CTDI was used as a measure of dose and determined with Monte Carlo simulations of a 32 cm CTDI phantom and validated with measurements. Soft tissue and iodine contrast performance was quantified using the contrast-to-noise-ratio at unit dose (CNRD) and compared to the standard protocols to obtain dose reduction values. Current CTs were assumed to operate from 70 to 150 kV in 10 kV steps, and up to 1000 mAs.
**Results or Findings:** Using patient-specific prefilters achieved 17%, 32% and 29% dose reduction for child, adult and obese phantoms for soft tissue contrast, and 3%, 44% and 28% for iodine contrast. Here, no significant difference between tin and copper prefilters was observed. Optimal prefilter thickness depends on patient size and contrast modality. Therefore, fine filter thickness gradations are needed. Dose reduction for iodine contrast is mostly achieved due to the decrease of tube voltage. Reducing tube voltage to 50 kV increases dose reduction of the child to 39% with tin and 57% with copper prefilters.
**Conclusion:** Patient-specific prefilters provide significant dose reductions for soft tissue contrast with current CT systems. Dose reduction for iodine contrast requires tube voltage reduction in combination with copper prefilters.
**Limitations:** Our results are based on simulations. Measurements are not possible because today's CT systems do not have such a variety of prefilter thicknesses.
**Ethics Committee Approval:** Not applicable
**Funding for this study:** Not applicable
**Author Disclosures:**
Laura Klein: Nothing to disclose
Jörg Steidel: Nothing to disclose
Marc Kachelrieß: Nothing to disclose
Stefan Sawall: Nothing to disclose
Joscha Maier: Nothing to disclose

### RPS 200-8

**Comparison of Size-Specific Dose Estimates (SSDE) and CTDIvol values in patients undergoing CT examinations of abdomen and pelvis**

*S. Joshi*; Lalitpur/NP
*(swetajoshi95@gmail.com)*

**Purpose:** To compare CTDIvol and SSDE values in patients undergoing CT scans of the abdomen and pelvis and to determine their relationship with patient size (patient weight and BMI).
**Methods or Background:** Background: Computed Tomography Dose Index volume (CTDIvol) underestimates the radiation dose being given to small children and smaller adults and overestimates the dose to larger patients. The use of Size Specific Dose Index (SSDE) helps convert CTDIvol into more patient size specific radiation dose and is a better measure of estimated patient dose. Method: Purposive sampling was used for data collection and 96 adults were included. Effective diameters were calculated from the AP and Lateral diameters at the mid-liver region. These were used to determine the conversion factors which were then used to convert the CTDIvol values to SSDE.
**Results or Findings:** The average CTDIvol was found to be 9.42 ± 3.26 mGy and the average SSDE was found to be 13.48 ± 3.53 mGy. Moderate positive correlation(r=+0.52) was found between CTDIvol and patient weight and low positive correlation(r=+0.17) was found between SSDE and patient weight. Similarly, moderate positive correlation(r=+0.5) was found between CTDIvol and patient BMI and low positive correlation(r=+0.14) was fond between SSDE and patient BMI.
**Conclusion:** In comparison with SSDE, CTDIvol seemed to underestimate the patient dose estimate by 30.11%. CTDIvol values showed dependency with patient weight and BMI, this dependency was significantly reduced when those values were converted into SSDE. Thus, providing more concrete evidence to the fact that SSDE values are a more reliable patient dose estimate since it addresses the patient's size.
**Limitations:** Neither risk nor organ doses were determined in this work. SSDE values were calculated using the effective diameter and not the water equivalent diameter.
**Ethics Committee Approval:** Yes
**Funding for this study:** No
**Author Disclosures:**
Sweta Joshi: Nothing to disclose

### RPS 200-9

**Estimation of radiation exposure and assessment of local diagnostic reference levels in the endovascular therapy of subarachnoid haemorrhage related vasospasm**

*M. Opitz*, A. Wetter, S. Zensen, D. Bos, M. Forsting, C. Deuschl, N. Guberina; Essen/DE

**Purpose:** This study aims to determine local diagnostic reference levels (LDRLs) of endovascular therapy in patients with cerebral vasospasm (CVS) due to spontaneous subarachnoid hemorrhage (sSAH).
**Methods or Background:** In a retrospective study design, LDRLs and achievable dose (AD) were assessed for all consecutive patients undergoing selective intra-arterial vasodilator administration or additional mechanical angioplasty for CVS treatment. All procedures were performed at the flat-panel angiography-systems Allura Xper (Philips Healthcare). Interventional procedures were differentiated according to the type of procedure and the number of probed vessels.
**Results or Findings:** Altogether 494 consecutive neurointerventional procedures of 121 patients with CVS due to sSAH could be included. The following diagnostic reference values, AD and mean values could be determined: (I) (ICA bilateral+VA) DRL 80.29Gy*cm², AD 67.77Gy*cm², mean 70.12Gy*cm²; (II) (ICA bilateral) DRL 59.15Gy*cm², AD 49.88Gy*cm², mean 50.25Gy*cm²; (III) (ICA unilateral) DRL 34.70Gy*cm², AD 27.12Gy*cm², mean 33.19Gy*cm²; (IV) (VA unilateral) AD 32.57Gy*cm², mean 34.25Gy*cm², (V) (Nimodipine+TBA) DRL 130.17Gy*cm², AD 108.56Gy*cm², mean 106.09Gy*cm² and (VI) (Nimodipine+stent) DRL 107.60Gy*cm², AD 81.13Gy*cm², mean 86.53Gy*cm². Kruskal-Wallis-test confirmed significant dose difference considering the number of probed vessels ($p<0.001$). Mann-Whitney-U-test revealed significant differences of DAP and fluoroscopy time between chemical-only and additional mechanical angioplasty ($p<0.001$).
**Conclusion:** The LDRLs of intra-arterial chemical angioplasty are substantially lower compared with diagnostic reference levels (DRLs) proposed for other therapeutic interventions. The total dose may be reduced efficiently by probing only the target vessels during repeated applications.
**Limitations:** The DAP allows to contrast radiation exposure during the course of time, for different procedures of the same anatomic area, at different devices and centers. To estimate stochastic risks of radiation exposure the effective dose is a more straightforward value.
**Ethics Committee Approval:** Ethical approval was approved by the ethical committee of our institution (20-9604-BO).
**Funding for this study:** No funding.
**Author Disclosures:**
Michael Forsting: Nothing to disclose
Nika Guberina: Nothing to disclose
Denise Bos: Nothing to disclose
Cornelius Deuschl: Nothing to disclose
Sebastian Zensen: Nothing to disclose
Axel Wetter: Nothing to disclose
Marcel Opitz: Nothing to disclose

### RPS 200-10

**The Image Gently mission: one voice through many languages**

*D. P. Frush*[1], R. Yurchuck[1], F. Fahey[2], S. T. Treves[2], D. Gress[3], D. d. C. T. Bustelo[4], K. E. Applegate[5]; [1]Durham, NC/US, [2]Boston, MA/US, [3]Reston, VA/US, [4]Curitiba - Paraná/BR, [5]Zionsville, IN/US
*(dfrush@stanford.edu)*

**Purpose:** The Image Gently (IG) Alliance mission, to improve safe and effective imaging care of children worldwide has advanced through campaigns with eight campaign posters adhering to simple, resonant messaging. An early successful partnership with the Brazilian Society of Nuclear Medicine resulted in Portuguese translations of the two nuclear medicine posters and a call for additional translations.
**Methods or Background:** A committee that met regularly with strong international and intersocietal relationships formalized the process: (1) initially targeting common languages, (2) identifying project lead(s), (3) providing access to the publisher adobe acrobat through DropBox and Google Drive for templates and PDFs of all English versions, (4) leveraging networks for translators including IAEA (as a convener), ESPR, and the World Federation of Pediatric Imaging, (5) attributing the translating society and a statement that the translation accuracy is the responsibility of the translator, (6) placing posters on both IG and translating society websites.
**Results or Findings:** Completed translations: Farsi, German, Portuguese, Romanian, Russian, and Spanish; ongoing Albanian, Bulgarian, Chinese, Estonian, Finnish, French, Latvian, Lithuanian, Macedonian, Norwegian, Serbo-Croatian, and Swedish. Several corrections in the English versions also resulted.
**Conclusion:** Based on a successful model for informed radiation use in children, poster translations can result in potentially more influential message penetration into regions. Image Gently's translation initiative has served as a model and inspiration for Image Wisely to pursue similar international collaboration(s), such as translated publications.
**Limitations:** Challenges included occasional difficulties navigating the adobe acrobat software, accuracy of translations especially with English catchphrases (implied meanings such as “Back to Basics” may not translate well), reliance on volunteer efforts, and inability, without follow up survey, in determining impact/value.
**Ethics Committee Approval:** Not applicable
**Funding for this study:** None
**Author Disclosures:**
Dustin Gress: Nothing to disclose
Kimberly E Applegate: Nothing to disclose
Robin Yurchuck: Nothing to disclose
Frederic Fahey: Nothing to disclose
Dolores del Carmen Tanus Bustelo: Nothing to disclose
S. Ted Treves: Nothing to disclose
Donald P. Frush: Nothing to disclose

### RPS 200-11

**Novel application of a comparison metric between software simulation and radiochromic film skin dose maps in high dose interventional angiographic procedures**

*M. M. J. Felisi*[1], S. Riga[1], P. E. Colombo[1], M. M. Vincenzi[1], C. Dillion[2], S. Massey[2], A. Torresin[1]; [1]Milan/IT, [2]Scottsdale, AZ/US
*(marco.felisi@unimi.it)*

**Purpose:** Feasibility study of a comparison metric to evaluate the skin dose maps accuracy in interventional radiology procedures, provided by the algorithm implemented in the Radiation Dose Index Monitoring (RDIM) software NEXO[DOSE]® (Bracco Injeneering SA, Lausanne).
**Methods or Background:** The RDIM software uses the Radiation Dose Structured Report information for skin dose maps estimation. The algorithm had been previously validated on geometrical phantom in terms of Peak Skin Dose (PSD) and dose profiles, using the gold standard GafChromic® XR-RV3 films, positioned under the phantom. Although in the simplest cases the information relating to the PSD is sufficient, in more complex ones, as occurs in clinical routine, it is necessary to have information relating to the entire dose map and not only in the most exposed area. To address this need, the gamma analysis method, usually used in radiotherapy to verify treatment plans, was adapted to this issue. This metric was tuned by comparing dose maps obtained with the software and GafChromic® XR-RV3 for 8 tests performed on phantom with angiography systems of two different vendors. Gamma analysis was performed using FilmQA Pro software (Ashland, Inc, Covington, KY, USA).
**Results or Findings:** Dose difference and distance to agreement acceptance criteria were established, using the simple cases performed on phantom. Gamma criteria of 12%/10mm has been found to be appropriate as a tolerance level with a passing rate of 90%. The obtained tolerance values will be used to assess the accuracy also in complex clinical cases.
**Conclusion:** The proposed metric appears to be promising for evaluating dose distributions in diagnostic procedures, allowing a full 2D skin dose maps validation.
**Limitations:** Results obtained in preliminary studies on phantom tests.
**Ethics Committee Approval:** Not Available
**Funding for this study:** No Funding
**Author Disclosures:**
Monica Maria Vincenzi: Nothing to disclose
Steve Massey: Employee: PACSHealt, LCC, Scottsdale, AZ, USA
Colby Dillion: Employee: PACSHealt, LCC, Scottsdale, AZ, USA
Alberto Torresin: Nothing to disclose
Marco Maria Jacopo Felisi: Nothing to disclose
Paola Enrica Colombo: Nothing to disclose
Stefano Riga: Nothing to disclose

### RPS 200-12

**Effective dose conversion factors from air-kerma area product in three high dose abdominal interventional angiographic procedures**

*S. Riga*, M. M. J. Felisi, D. Curto, P. E. Colombo, A. Torresin; Milan/IT
*(stefano.riga@unimi.it)*

**Purpose:** Establishment of conversion factors to evaluate effective dose (E) from air-kerma area product (KAP) in three interventional angiographic procedures: trans-arterial chemoembolization (TACE) endovascular aneurysm repair (EVAR), transjugular intrahepatic portosystemic shunt (TIPS) procedures. These procedures involve high-doses and the patient exposure estimation is strongly recommended in the European Directive 2013/59/EURATOM.
**Methods or Background:** Radiation Dose Index Monitoring software NEXO[DOSE]® (Bracco Injeneering SA, Lausanne) was used to extract 60 procedures with more than 6000 irradiation events. For each irradiation event, the software collects information from the Radiation Dose Structured Report

(RDSR) about kV, primary/secondary angles, source-to-isocenter distance, additional filtration, air-kerma at the interventional radiological point and KAP. Effective dose was calculated using the rotation module for PCXMC 2.0 (STUK, Helsinki, Finland) based on a Monte Carlo simulations and on the mathematical ORNL phantoms model. The PCXMC software processed the dosimetric and geometrical data obtained from the RDSR.
**Results or Findings:** In addition to the inherent filtration of 3 mm of Al, additional Cu and Al filtrations are taken into account for high (0.1mm/1mm), normal (0.4mm/1mm) and low fluoroscopy (0.9mm/1mm) mode, and no filtration for cine-fluorography. The X-ray beam quality may lead to a relevant impact to the effective dose calculation. The achieved E/KAP conversion factors for TACE, EVAR and TIPS procedures are estimated in 0.28±0.03 mSv/mGy·cm2, 0.19±0.02 mSv/mGy·cm2 and 0.34±0.04 mSv/mGy·cm2 respectively. The differences obtained reflect the different position and beam quality of the considered procedures.
**Conclusion:** Effective dose conversion factors from KAP were assessed using ICRP 103 tissue weighting factors and considering the different filtration. These factors can be integrated in the RDIM system to estimate population doses from KAP and to compare different procedures.
**Limitations:** Limited number of exams analyzed.
**Ethics Committee Approval:** Not applicable.
**Funding for this study:** No funding.
**Author Disclosures:**
Denise Curto: Nothing to disclose
Alberto Torresin: Nothing to disclose
Marco Maria Jacopo Felisi: Nothing to disclose
Paola Enrica Colombo: Nothing to disclose
Stefano Riga: Nothing to disclose

### RPS 200-13

**Radiation exposure of CT-guided percutaneous manual and power drill bone biopsy**

*S. Zensen*, S. Selvaretnam, M. Opitz, D. Bos, J. Haubold, J. Theysohn, M. Forsting, N. Guberina, A. Wetter; Essen/DE

**Purpose:** Apart from the commonly applied manual needle biopsy, CT-guided percutaneous biopsies of bone lesions can be performed with battery-powered drill biopsy systems. Due to assumably different radiation doses and procedural duration, the aim of this study is to examine radiation exposure and establish local diagnostic reference levels (DRLs) of CT-guided bone biopsies of different anatomical regions.
**Methods or Background:** In this retrospective study, dose data of 187 patients who underwent CT-guided bone biopsy with a manual or powered drill biopsy system performed at one of three different multi-slice CT were analyzed. Between January 2012 and November 2019, a total of 27 femur (A), 74 ilium (B), 27 sacrum (C), 28 thoracic vertebrae (D) and 31 lumbar vertebrae (E) biopsies were included. Radiation exposure was reported for volume-weighted CT dose index (CTDIvol), dose-length product (DLP) and effective dose.
**Results or Findings:** CTDIvol and DLP of manual versus powered drill biopsy were (median,IQR): A: 56.9(41.4-128.5)/66.7(37.6-76.2)mGy, 410(203-683)/303(128-403)mGy·cm, B: 83.5(62.1-128.5)/59.4(46.2-79.8)mGy, 489(322-472)/400(329-695)mGy·cm, C: 97.5(71.6-149.2)/63.1(49.1-83.7)mGy, 627(496-740)/404(316-515)mGy·cm, D: 67.0(40.3-86.6)/39.7(29.9-89.0)mGy, 392(267-596)/207(166-402)mGy·cm, E: 100.1(66.5-162.6)/62.5(48.0-90.0)mGy, 521(385-619)/315(240-452)mGy·cm. Radiation exposure with powered drill was significantly lower for ilium and sacrum, while procedural duration was not increased for any anatomical location. Local DRLs could be depicted as follows (CTDIvol/DLP): A: 90.9mGy/522mGy·cm, B: 90.3mGy/530mGy·cm, C: 115.5mGy/740mGy·cm, D: 86.6mGy/578mGy·cm, E: 115.0mGy/546mGy·cm.
**Conclusion:** Use of powered drill bone biopsy systems for CT-guided percutaneous bone biopsies can significantly reduce the radiation burden compared to manual biopsy for specific anatomical locations such as ilium and sacrum and does not increase radiation dose or procedural duration for any of the investigated locations.
**Limitations:** Limitations are the retrospective design and that there were no equivalent numbers of manual and powered drill biopsies. Possibly operator dependent variability affected radiation dose.
**Ethics Committee Approval:** This study has obtained IRB approval.
**Funding for this study:** n/a
**Author Disclosures:**
Michael Forsting: Nothing to disclose
Jens Theysohn: Nothing to disclose
Nika Guberina: Nothing to disclose
Denise Bos: Research/Grant Support: Research grant within the University Medicine Essen Academy (UMEA) program, funded by the German Research Foundation (DFG; grant FU356/12-1) and the Faculty of Medicine, University of Duisburg-Essen
Johannes Haubold: Research/Grant Support: Research grant within the University Medicine Essen Academy (UMEA) program, funded by the German Research Foundation (DFG; grant FU356/12-1) and the Faculty of Medicine, University of Duisburg-Essen
Sebastian Zensen: Nothing to disclose
Axel Wetter: Nothing to disclose
Marcel Opitz: Nothing to disclose
Sumitha Selvaretnam: Nothing to disclose

### RPS 200-14

**Institutional Diagnostic Reference Levels (iDRLs) for fluoroscopic diagnostic and interventional procedures**

*J. Tristram*[1], A. Steuwe[1], F. Kröpil[1], C. Thomas[2], G. Antoch[1], J. Boos[3]; [1]Düsseldorf/DE, [2]Krefeld/DE, [3]Münster/DE
*(juliana.tristram@hhu.de)*

**Purpose:** To follow the call for action by the European Society of Radiology Eurosafe imaging campaign and implement institutional diagnostic reference levels (iDRLs) for fluoroscopic diagnostic and interventional procedures.
**Methods or Background:** 3812 fluoroscopic procedures performed on a monoplanar angiography system (device 1), a biplane angiography system (device 2) and a digital fluoroscopy device (device 3) were included in the IRB approved retrospective study (1926 men, 1886 women; 61.5±16.5 years, range 18–107 years). Dose area product (DAP), cumulative air karma (CAK), fluoroscopy time (FT), patient information, and procedure information were extracted using the institutional dose monitoring system. IDRLs were defined according to the recommendations of the International Commission on Radiological Protection (ICRP) as the median dose and calculated for the three most frequent procedures per device. When available national DRLs were compared with these iDRLs.
**Results or Findings:** IDRLs (DAP/CAK/FT) for device 1 were 1603 cGycm²/ 74 mGy/ 14.4 min for lower limb angiography (n=216), 11444/635/19.4 for abdominal vessel intervention (n=155), and 14587/942/24.5 for transarterial chemoembolization (TACE, n=84). For device 2 iDRLs were 9994/632/25.2 for cerebral and cervical thrombectomy (n=387), 7924/398/8.9 for cerebral diagnostic angiography (n=298) and 3113/195/5.5 for diagnostic angiography of the carotid arteries (n=122). For device 3 iDRLs were 638/31/0.6 for iod swallow (n=452), 409/15/0.9 for colon contrast enema (n=123) and 414/16/0.8 for upper gastrointestinal fluoroscopy (n=95). All iDRLs were lower than the national DRLs ranging from 13.6% for colon contrast enema to 63.4% for TACE.
**Conclusion:** We implemented iDRLs for fluoroscopic diagnostic and interventional procedures which enable a comprehensive dose analysis and comparison with national DRLs.
**Limitations:** The retrospective study design did not allow a complexity analysis of the procedures.
**Ethics Committee Approval:** Ethics committee approval was granted by the institutional review board.
**Funding for this study:** None
**Author Disclosures:**
Johannes Boos: Nothing to disclose
Christoph Thomas: Nothing to disclose
Juliana Tristram: Nothing to disclose
Feride Kröpil: Nothing to disclose
Gerald Antoch: Nothing to disclose
Andrea Steuwe: Nothing to disclose

### RPS 200-15

**Exposure of the population to ionising radiation from diagnostic medical imaging procedures in France in 2017**

*S. Dreuil*, C. Michel; Fontenay-aux-Roses Cedex/FR
*(serge.dreuil@irsn.fr)*

**Purpose:** This study aims to establish data on the exposure of the French population to ionising radiation from medical imaging for diagnosis purposes (conventional, dental and interventional radiology, computed tomography and nuclear medicine) for the year 2017 and to analyse variation in such data.
**Methods or Background:** The study was performed based on diagnostic imaging procedures taken from the "échantillon généraliste des bénéficiaires", a sample on a 1/97th scale of the healthcare consumption of the population covered by the main French health insurance schemes.
**Results or Findings:** 45.4% of the French population underwent one or more diagnostic imaging procedure in 2017 (43.8% in 2012), this percentage falling to 32.7% when dental radiology procedures are excluded. The evolution observed on the mean frequency of imaging procedures and the average per caput annual effective dose was generally minor, the latter stabilising at 1.53 mSv per caput (vs. 1.56 mSv in 2012). Nuclear medicine, the third contribution to the collective effective dose (11.3%) behind conventional radiology (11.8%), recorded the greatest increase over the 2012-2017 period (+44%). However, computed tomography remained the most significant contribution to the exposure faced by the population (74.2 %) by far. Only a small percentage of patients - but representing several hundreds of thousands of patients throughout France - combined multiple computed tomography examinations, leading to high effective doses, potentially exceeding 100 mSv over 3 years.
**Conclusion:** The strong increase recorded between 2002 and 2012 for the average per caput annual effective dose due to diagnostic medical imaging is no longer observed between 2012 and 2017. Nevertheless, attention should be paid to patients undergoing multiple imaging procedures over a short period of time.
**Limitations:** Not applicable.
**Ethics Committee Approval:** Not applicable.
**Funding for this study:** Not applicable.
**Author Disclosures:**
Serge Dreuil: Nothing to disclose
Célian Michel: Nothing to disclose

### RPS 200-16

**Evaluation of possible effectiveness and detriment of using patient lead pad for reducing staff dose in interventional radiology**

*T. Viana Miranda Lima*, T. Reyes Del Castillo, M. Heinrich, S. Zihlmann, R. Lopez Benitez, J. E. Roos; Luzern/CH
*(thiago.lima@luks.ch)*

**Purpose:** To evaluate the usefulness of commercially available scatter reduction lead pads on staff radiation exposures in interventional radiology.
**Methods or Background:** For this study an Alderson radiation therapy phantom, was irradiated in our Siemens Artis Icono angiograph device in three scenarios of protective pad usage (no use of the protective pad, correct use of the pad and incorrect use of pad). Different levels of incorrect pad positioning (pad covering the FOV) were also evaluated: 5%, 15% and 50% of the FOV. A Raysafe i3 real-time dosimeter system was used to evaluate accumulative dose (mSv) and dose rates (mSv/h). Dosimeters positions simulated operator's left eye (closer to the x-ray tube), over the lead apron, and thyroid. Doses and dose rates were compared in respect to its usefulness (dose reduction) or detriment (higher dose). Additionally, patient exposure was evaluated by the procedure DAP.
**Results or Findings:** Operator doses ranged from 3.15 ± 0.04 µSv to 67.89 ± 2.92 µSv (over the lead apron); from 0.43 ± 0.01 to 16.43 ± 0.91 (thyroid); and from 1.52 ± 0.08 to 44.77 ± 1.83 µSv (left eye). In terms of dose, at the perfect positioning of the pad (no FOV obstruction) we obtained in average 68% dose reduction compared to 67% (5% FOV obstruction), 63% (15% obstruction) and an average dose increase of 779% (50% obstruction). This pattern was observed for all dosimetry locations. Similar order of magnitude was observed to the patient exposure.
**Conclusion:** Our results showed that exposure reduction (around 60%) for the operator is possible when these protective pads are used, however, the incorrect use may introduce a detriment in both operator and patient exposure.
**Limitations:** Our results cover a limited number or devices.
**Ethics Committee Approval:** Not applicable
**Funding for this study:** Not applicable
**Author Disclosures:**
Stefanie Zihlmann: Nothing to disclose
Justus Erasmus Roos: Nothing to disclose
Mirjam Heinrich: Nothing to disclose
Tomas Reyes Del Castillo: Nothing to disclose
Thiago Viana Miranda Lima: Nothing to disclose
Ruben Lopez Benitez: Nothing to disclose

### RPS 200-18

**See the Invisible: an augmented reality simulation of scatter radiation embedded in endovascular simulator as a game changer in the physician's awareness**

*G. Bartal*, S. Fargun, I. Gal, N. Conway, A. Porat; Tel Aviv-Yafo/IL
*(gbartal@gmail.com)*

**Purpose:** State of the art angiography systems are an integral part of Interventional Radiology (IR), yet they come with a price of significant radiation exposure to the patients and personnel. Intelligent and safe use of this hardware improves performance and minimizes radiation exposure. Our aim was to develop and evaluate performance of “See-the-Invisible”, radiation awareness application.
**Methods or Background:** “See the Invisible” is a radiation alertness software package add-on, connected to the ANGIO Mentor endovascular simulator (Simbionix, 3D Systems Healthcare). Based on published exposure data on distance and angulation, four 3D models were created for each C-Arm angulation. The four 3D models represent scatter from 1000 mGy\min, 150-1000 mGy\min, 50-15 mGy\min, <50 mGy\min, and outer layer <1 mGy\min respectively.
**Results or Findings:** The simulator launches an external TCP server application, activating the Augmented Reality add-on, receiving synchronized scatter data as four layers of exposure. The 3D models shape, color and size are modified based on data interpolation corresponding to the simulation case’s current C-Arm movement and other imaging parameters. The radiation scatter is a combination of four 3D models pre-loaded by the simulation, representing the four layers of the radiation exposure. The room’s simulated scatter radiation is displayed using real-time deformation of the 3D models. The server sends the information received from simulator client to any display devices clients (phones/tablets) and they render for pre-calculated meshes in the simulated 3D environment.
**Conclusion:** Real time augmented reality simulation of scatter radiation during simulation of endovascular interventions creates virtual environment expected to improve the interventionist’s skills in a better procedure performance with lowest possible radiation exposure. Further evaluation using validated questionnaires is underway.
**Limitations:** Laboratory Investigation
**Ethics Committee Approval:** Not required
**Funding for this study:** Not required
**Author Disclosures:**
Shani Fargun: Employee: Simbionix 3DSystems
Idan Gal: Employee: Simbionix 3DSystems
Amir Porat: Employee: Simbionix 3DSystems
Gabriel Bartal: Consultant: Simbionix 3DSystems
Neithan Conway: Employee: Simbionix 3DSystems

### RPS 200-19

**Child protection beyond gender-based violence in crisis and humanitarian settings of northeast Nigeria: a radiation protection perspective**

*A. A. C. Igwegbe *[1], L. R. Owoade[2], K. Kwaji[1], M. Abubakar[1], F. B. Nkubli [1], C. Chukwuemeka Nzotta[3], A. Ali[1], U. Mohammed Sani[4]; [1]Maiduguri/NG, [2]Ibadan/NG, [3]Awka/NG, [4]Kano/NG
*(igwegbea@gmail.com)*

**Purpose:** To determine radiation doses received by school-aged female children and to establish diagnostic reference levels (DRLs)
**Methods or Background:** Fifty (50) school – aged female children,< 18 years of age were recruited from a tertiary hospital in Northeast Nigeria for this study. Demographic data such as age and gender; anthropometric data such as weight, height, Body Mass Index, and anteroposterior chest thickness and technical exposure parameters were obtained. Calibrated thermoluminescent dosimeter chips (TLD 100) were used to measure entrance skin dose. Descriptive statistics were used to summarise the data while percentiles were used to establish DRLs. Pearson’s correlation was used to determine the relationship between doses received with anthropometric and technical parameters. DRL was set at the 75th percentile of the median patient dose distributions for the various age groups studied. Statistical significance was set at $p < 0.05$.
**Results or Findings:** The results of the established DRLs for the various age groups in this study were;4Years - <10Year, 1.56 mGy; 10Years - <14Years, 1.23 mGy; 14Years - <18Years, 2.12 mGy. Pearson correlation shows statistically significant ($p = 0.044$) moderate positive relationship ($r = 0.353$) between weight with entrance skin dose among the 10 Years - <14 Years age group. A significant ($p = 0.035$) strong negative relationship ($r = - 0.703$) was also observed between entrance skin dose with kVp among the 14 Years - < 18 Years age group.
**Conclusion:** Age specific DRL for plain chest radiography marked by variations in measured entrance skin doses was established for school aged female children. A statistically significant strong positive relationship between kVp and entrance skin dose was observed in this study.
**Limitations:** Only chest radiography examination was considered
**Ethics Committee Approval:** Ethics committee approval was obtained for this study
**Funding for this study:** n/a
**Author Disclosures:**
Kafuli Kwaji: Author: Author
Aiman Amin Chidera Igwegbe : Author: speaker
Umar Mohammed Sani: Author: Author
Christian Chukwuemeka Nzotta: Author: Author
Alhaji Ali: Author: Author
Flavious Bobuin Nkubli : Author: Author
Latifat Ronke Owoade: Author: Author
Mathew Abubakar: Author: Author

### Research Presentation Session: Education & Professional issues

### RPS 100

### Professional issues: education and training

### RPS 100-1

**Radioimmunotherapy with yttrium-90 ibritumomab tiuxetan for relapsed or refractory B-cell non-Hodgkin's lymphoma: a single institution experience in Japan**

*K. Maruyama*, K. Utsunomiya, Y. Kono, Y. Ueno, N. Tanigawa; Osaka/JP
*(maruyamk@hirakata.kmu.ac.jp)*

**Purpose:** Yttrium-90 ibritumomab tiuxetan therapy (90Y-IT) was approved for treating relapsed or refractory B-cell non-Hodgkin's lymphoma (NHL). As yet however, how long survive after 90Y-IT and what kind of factor affect the efficacy remains uncertain. Objective: We investigated the efficacy of 90Y-IT for NHL.
**Methods or Background:** Methods: We retrospectively examined 65 patients (mean age 68, range 45-87 years) with relapsed or refractory NHL who received 90Y-IT between October 2012 and September 2017. NHL included 46 Follicular lymphoma (FL),13 Mucosa associated lymphoid tissue lymphoma (MALT), and 6 Mantle cell lymphoma (MCL). Response rates (complete response, CR; partial response, PR; stable disease, SD; progressive disease, PD), overall survival (OS), and progression-free survival (PFS) were analyzed. The correlations between OS and baseline variables such as age and F-18 fluorodeoxyglucose (FDG) uptake were analyzed.
**Results or Findings:** Results: The overall response rate to 90Y-IT was 86% (CR 63%; PR 23%). The median follow-up between 90Y-IT and the latest clinical record was 51 months (range 0.9 to 95 months). The median OS was not reached. For evaluable patients excluding who were censored (lost to follow-up) the OS and PFS rates were 77% and 28% at 5 years, respectively. Of a total of 65 patients 10 patients died. 5 FL and 2 MALT died after 1 year from 90Y-IT. Three (50%) of the six patients with MCL did not respond and died within 1 year in PD. The baseline variables were not significantly correlated with OS.
**Conclusion:** Radioimmunotherapy is active against relapse or refractory FL and MALT. On the other hand, efficacy data for 90Y-IT in MCL are limited.
**Limitations:** Time limits. Longer follow-up needs.
**Ethics Committee Approval:** This study was conducted according to the principles of the Declaration of Helsinki.
**Funding for this study:** No funding.
**Author Disclosures:**
Kaoru Maruyama: Nothing to disclose
Keita Utsunomiya: Nothing to disclose
Noboru Tanigawa: Nothing to disclose
Yasuhiro Ueno: Nothing to disclose
Yumiko Kono: Nothing to disclose

### RPS 100-2

**A novel online training platform for improving the accuracy of frontline clinicians in suspected COVID-19 chest x-ray interpretation**

*S. Ather*, F. Gleeson, A. Novak, J. Bahra, A. Acharya; Oxford/UK
*(msarim@hotmail.com)*

**Purpose:** The incidence of coronavirus disease 2019 (COVID-19) continues to rise in the UK. Identification of COVID-19 using conventional radiography (chest X-rays) in the Emergency Department (ED) is a crucial skill for frontline clinicians. Report and Image Quality Control (RAIQC) is an original web-based tool designed to improve the image reporting ability of clinicians. We set out to evaluate the use of this platform for improving the accuracy of COVID-19 identification on chest X-rays via a multicentre service improvement study.
**Methods or Background:** 112 clinicians working in EDs across five regional hospitals were recruited over a six-month period. Participants completed online training in interpreting sixty anonymised chest X-rays. Accuracy of X-ray reporting was assessed before and after training.
**Results or Findings:** All recruits completed the initial assessment. 56 recruits completed all three training components. The initial mean accuracy of clinicians in identifying COVID-19 on chest X-rays was 43%. The mean accuracy was 57% amongst recruits who completed all three online training components. Participants who completed all training components had an improved reporting speed.
**Conclusion:** Online training can improve the accuracy of frontline clinicians in identifying COVID-19 on chest X-rays.
**Limitations:** This study assessed participant reporting accuracy in a virtual learning environment. Further work is required to evaluate the real-life accuracy rates of ED COVID-19 X-ray detection following the completion of online training.
**Ethics Committee Approval:** - Permission was sought from the OUH Caldicott Guardian and information governance team to use anonymised patient imaging for the study. - Study participants were informed that their anonymised performance data would be used for service improvement purposes.
**Funding for this study:** - Oxford Hospitals Charity - Innovate UK - Pfizer Ltd. Funding provided for the development and creation of the online learning platform. Funders did not have any editorial influence.
**Author Disclosures:**
Sarim Ather: Research/Grant Support: Innovate UK Research/Grant Support: Pfizer Ltd Research/Grant Support: Oxford Hospitals Charity
Fergus Gleeson: Nothing to disclose
Alex Novak: Nothing to disclose
Jasdeep Bahra: Nothing to disclose
Anita Acharya: Nothing to disclose

### RPS 100-3

**Airway, breathing, COVID-19? Adapting resuscitation training in the radiology department to ensure patient and staff safety in the COVID-19 pandemic**

*S. Memarian*, W. Pettit, M. Naik, G. Treanor, M. Cooper, J. Danin, A. J. Jameel; London/UK

**Purpose:** The creation of the 2019 Radiology Specific Resus Training (RSR), was to aid radiology staff in responding to a deteriorating patient and perform effective resuscitation. As safety to staff and patients is paramount, where patients in the radiology department often have unknown Covid status, we have adapted the course to meet the unique challenges of the Covid-19 pandemic.
**Methods or Background:** Following discussion with key stakeholders including resuscitation, paediatric and imaging teams (specifically MR safety) the previous RSR course now incorporates Covid-19 into each original scenario: anaphylaxis and contrast reactions, paediatric basic life support, maternal collapse and resuscitation of an adult in the MR scanner (adult basic life support) with special reference to staff safety and personal protective equipment (PPE).
**Results or Findings:** This two hour course consists of an 'e-component' and a practical RSR component, which provides 'hands on' learning from the scenarios presented in the e-learning module. This “e-RSR” encompasses all of the above scenarios with the following aims: 1. Provide key knowledge prior to the practical session, using modern distance learning platforms. 2. Ensure the practical session is effective and time focused - reducing any potential exposure to staff. We audited the effectiveness of the new e-RSR course at delivering resuscitation training with a pre- and post course questionnaire.
**Conclusion:** Due to Covid-19 we amended the successful roll out of the radiology specific resuscitation (RSR) training programme, demonstrating the ability of our department to swiftly adapt resuscitation training to the Covid-19 pandemic. The new e-RSR course provides our radiology staff with the skills and confidence to manage acutely unwell paediatric and adult patients with a variety of Covid status.
**Limitations:** Covid-19 imposed restrictions and limited resus teams availability impacting sample size for audit.
**Ethics Committee Approval:** n/a
**Funding for this study:** n/a
**Author Disclosures:**
Mehrengise Cooper: Nothing to disclose
Gillian Treanor: Nothing to disclose
William Pettit: nothing to disclose
Mitesh Naik: Nothing to disclose
Joanna Danin: Nothing to disclose
Sabrina Memarian: Nothing to disclose
Ayesha Juvairia Jameel: Nothing to disclose

### RPS 100-4

**The effects of multidisciplinary team meetings on clinical practice for colorectal, lung, breast, and prostate cancer: a systematic review**

*L. Koco*, H. Weekenstroo, R. Mann; Nijmegen/NL
*(lejla.koco@radboudumc.nl)*

**Purpose:** The aim of this systematic review is to identify the effects and value of multidisciplinary team meetings (MDTM) for lung, breast, colorectal and prostate cancer.
**Methods or Background:** For this systematic review, performed following PRISMA guidelines, studies examining the impact of MDTMs on breast, colorectal, lung, and prostate cancer care pathways were included. Endpoints include impact on management plan and survival. Electronic databases

PUBMED, EMBASE, Cochrane Library and Web of Science were searched for articles published between 2000-2019. Screening and data extraction were performed by two independent researchers. Risk of bias and level of evidence were assessed using ROBINS-I tool and GRADE scale.
**Results or Findings:** 35 of 11,875 articles were selected; evaluating colorectal (20), lung (8), prostate (6) and breast (1) cancer. Risk of Bias was scored serious to critical and reported results are heterogeneous. Results showed that management plans were changed in 1.6%-58% of cases after MDTMs; 10 of 14 studies showed an increase of more than 20%. All studies evaluating surgery showed a significant impact of MDTMs on surgery type; most studies showed a significant reduction of overall performed surgery after MDTM. Most studies showed a significant increase in CT and MRI imaging in the MDT cohorts. No significant impact on colonoscopy and ultrasound diagnostics was identified. Nine studies assessed survival, of which six studies showed survival rate increased significantly with MDTM discussions and three studies did not show significant differences.
**Conclusion:** Despite heterogeneous data, MDTMs commonly change the management plan, decreasing surgery and increasing imaging. Survival seems to be increased in patients discussed in MDTMs. To further explore the impact of MDTMs on the quality of healthcare, high-quality research is needed.
**Limitations:** Meta-analysis wasn't possible due to the heterogeneity of data.
**Ethics Committee Approval:** Not necessary
**Funding for this study:** None
**Author Disclosures:**
Harm Weekenstroo: Nothing to disclose
Ritse Maarten Mann: Nothing to disclose
Lejla Koco: Nothing to disclose

### RPS 100-5

**Staff radiologists' experience and subspecialisation: how does it affect proofreading behaviour when reviewing resident preliminary reports?**
*J. Vosshenrich*, P. Brantner, J. Cyriac, D. Boll, E. M. Merkle, T. Heye; Basel/CH
*(jan.vosshenrich@usb.ch)*

**Purpose:** To investigate if level of experience and subspecialization of attendings impact the report review process using a data mining approach.
**Methods or Background:** 131,397 radiology reports dictated from 07/2017-08/2020 in our tertiary care radiology department were included. Reports were automatically analyzed by a custom-developed report comparison tool. It quantifies the amount of edits on residents' preliminary reports by attendings during proofreading using the Jaccard Index (range: 0-1). A Jaccard index of 0.85 e.g. equals an edit distance of 15%. Years/months of post-fellowship experience as staff radiologist at the time of review were allocated to each report. Three levels of experience were defined: (1) ≤ 5 years, (2) 6-10 years (3) >10 years. Welch ANOVA was used to compare groups overall, per modality and subspecialty section. Relationship of Jaccard distance and experience was assessed with linear regression analysis.
**Results or Findings:** With increasing experience, staff radiologists performed less edits during review of resident reports overall (Jaccard distance: 18% [≤5 years' experience] vs. 14% [6-10 years' experience] vs. 9% [>10 years' experience]; p<.001). Similar decreases were seen for all distinct radiology subspecialty sections (e.g. MSK: 28% [≤5 years] vs. 20% [6-10 years] vs. 9% [>10 years]; p<.001) and imaging modalities (e.g. CT: 15% [≤5 years] vs. 11% [6-10 years] vs. 9% [>10 years]; p<.001). Linear regression analysis confirmed a strong negative relationship between experience and Jaccard distance (r=-0.79 [95%-CI: -0.72,-0.84]; p<.001). Attendings performed more edits on reports from their personal subspecialty compared to reports of imaging studies outside their field of expertise (Jaccard distance: 15% vs. 11%; p<.001).
**Conclusion:** Staff radiologists' level of experience and subspecialization seem to impact proofreading behavior when reviewing resident reports significantly. Further investigation how this affects radiology report quality are needed.
**Limitations:** n/a
**Ethics Committee Approval:** n/a
**Funding for this study:** n/a
**Author Disclosures:**
Philipp Brantner: Nothing to disclose
Tobias Heye: Nothing to disclose
Joshy Cyriac: Nothing to disclose
Daniel Boll: Nothing to disclose
Elmar M. Merkle: Nothing to disclose
Jan Vosshenrich: Nothing to disclose

### RPS 100-6

**How COVID-19 kick-started online learning in medical education: the DigiMed study**
*F. Stoehr*[1], L. Müller[1], A. Brady[2], F. Hahn[1], A. Mähringer-Kunz[1], C. Düber[1], T. Emrich[1], D. Pinto[3], R. Kloeckner[1]; [1]Mainz/DE, [2]Cork/IE, [3]Cologne/DE

**Purpose:** The coronavirus disease 2019 (COVID-19) pandemic led to far-reaching restrictions of social and professional life. Medical schools had to restructure their curriculum by switching to online learning. We aimed to evaluate students' attitudes to online learning to guide future development of medical education.
**Methods or Background:** 3286 medical students from 12 different countries participated in this cross-sectional study. On a 7-point Likert scale, participants rated the online learning situation before and during the pandemic at their medical schools, technical and social aspects, and the current and future role of online learning in medical education.
**Results or Findings:** Most medical schools managed the rapid switch to online learning (78%). Online learning led to unchanged or higher attendance of courses (70%). Possible downsides included motivational problems (42%) and the risk of social isolation (64%). Most were convinced that medical education lags behind current capabilities regarding online learning (78%) and estimated the proportion of online learning before the pandemic at only 14%. For the future, they wished for a more prominent role, involving around 42% of online learning.
**Conclusion:** This study demonstrates the positive attitude of medical students towards online learning. It reveals a discrepancy between what students demand and what the curriculum offers. The pandemic might be the long-awaited catalyst for a new "online era" in medical education.
**Limitations:** - selection bias, meaning that interested students are more likely to complete the questionnaire. - social desirability, meaning that participants choose the answer that they assume is favourable. - cross-sectional study design. Thus, results may not necessarily translate into long-term opinions.
**Ethics Committee Approval:** Institutional review board approval was waived by the Ethics Committee of the Medical Association of Rhineland-Palatinate.
**Funding for this study:** FS is supported by the Clinician Scientist Fellowship "Else Kröner Research College: 2018_Kolleg.05".
**Author Disclosures:**
Roman Kloeckner: Nothing to disclose
Lukas Müller: Nothing to disclose
Fabian Stöhr: Grant Recipient: Fabian Stoehr is supported by the Clinician Scientist Fellowship "Else Kröner Research College: 2018_Kolleg.05".This funding source had no role in the design, data collection, analysis, interpretation, writing, or decision to submit the results of this study.
Christoph Düber: Nothing to disclose
Aline Mähringer-Kunz: Nothing to disclose
Felix Hahn: Nothing to disclose
Tilman Stephan Emrich: Nothing to disclose
Daniel Pinto: Nothing to disclose
Adrian Brady: Nothing to disclose

### RPS 100-7

**How to operate proton therapy in the pandemic environment: a single center experience**
*M. Cherkashin*, N. Berezina, N. Vorobyov, A. Mikhaylov, A. Nikolaev, N. Kataev; Saint Petersburg/RU
*(mikhail.a.cherkashin@gmail.com)*

**Purpose:** To discuss organizational practices for proton therapy delivery in COVID-19 pandemic environment
**Methods or Background:** Novel coronavirus pandemic has affected on different therapeutic areas and patient access for cancer treatment this year was restricted. We've tried to organize processes for proton and external beam radiation therapy for COVID-positive cancer patients. Due to local regulations, verified SARS-CoV-2 patient should be isolated, and we've organized dedicated observational inpatient department. 2 infected patients (moderate pneumonia confirmed by CT and good respiratory function) were involved - prostate cancer and esophageal tumor. Prostate was treated by hypofractionation (5 fractions with endorectal balloons), esophageal cancer - by standard fractionation. Patients has isolated in wards and before each radiation treatment were disinfected in disinfection area and covered with PPE. Medical physicists and radiotherapists also use PPE level 2. Proton therapy treatment gentry was sanitized after each fraction.
**Results or Findings:** Radiation proton therapy for cancer in COVID patients was completed with standard clinical effect and good local control. Also both patients has positive respiratory status and were dismisses hospital for home treatment. After 3 months we haven't detected cancer progression (by MRI) and patients has reconvalescence for COVID with positive IgG antibodies and good clinical performance. Medical staff and other non-infected patients haven't any infection exposure and still COVID-free.
**Conclusion:** New pandemic environment in oncology hospitals demands to reassess infection control practices. Standard radiation cancer treatment is possible for coronavirus infected patients. Clear practices should be implemented - patient isolation in case of infection, disinfection before treatment, personal protective equipment usage for patient and involved healthcare providers.
**Limitations:** This is the ongoing case study in pandemic environment
**Ethics Committee Approval:** Study was approved by LEC
**Funding for this study:** Funded by Hospital
**Author Disclosures:**
Nikita Kataev: Nothing to disclose
Mikhail Cherkashin: Nothing to disclose
Natalia Berezina: Nothing to disclose
Aleksey Nikolaev: Nothing to disclose
Alexey Mikhaylov: Nothing to disclose
Nikolay Vorobyov: Nothing to disclose

### RPS 100-8

**Challenges encountered by radiologists during the COVID-19 pandemic in a limited-resource third-world setting**

*M. E. Abd El Bagi*[1], M. Mustafa[1], H. Khalil[2], M. Altahir[1], S. Alhassan[1], S. Mohammed[3], O. Elamin[4], H. Ahmed[1], T. Abdalhadi[3]; [1]Khartoum/SD, [2]Omdurman/SD, [3]Khartoum North/SD, [4]Shargelneel/SD
*(drm_bagi@hotmail.com)*

**Purpose:** COVID19 impact on radiologists in a limited resource setting.
**Methods or Background:** COVID19 hit Sudan on 13th March 2020. Access to advanced imaging is limited to those who can pay. Sudanese radiology residents and their supervisors were asked by an online questionnaire about their experience during the pandemic. This paper reports the actual challenges encountered by radiologists during the pandemic.
**Results or Findings:** The weekly meeting attendance varied from 70-100. 89 participants responded. 55 (61.8%) of the respondents were residents. 35(39.3%) of participants were affiliated with the private sector. Most departments 61 (68.5%) were partly shutdown.11(12.4%) of participants were not given a pass during the care few. 32(36%) could not find transport. Personal protective equipment (PPEs) was totally lacking for 34 (38.2%) of the participants. Turn-around times of radiology reports were normal in only 30(33.7%). Delay of swab results has caused unnecessary isolation for 59 (66.3%) of the participants. 76 (85.4%) of the 89 respondents did not get any training for COVID19. 45 (50.6%) of the respondents have sometimes suffered intimidation or aggression by co-patients or the forces. Only 27 (30.3%) of the participants expected their departments would do better in the future. 21 (23.6%) of the participants had their rent refused by landlords due to phobia.
**Conclusion:** There were many encountered challenges. Most serious was lack of training and shortage of PPE which lead to departments shut down. Though well equipped, the private sector is not the best set up for training and research. It is hoped that this report would help future preparedness and attract international logistic support to bridge the widening gap of global health inequalities.
**Limitations:** The results were not gendered specific.
**Ethics Committee Approval:** The study was approved by the Sudan Medical Specialities Board
**Funding for this study:** None.
**Author Disclosures:**
Somia Mohammed: Nothing to disclose
Hiba Khalil: Nothing to disclose
Tarteel Abdalhadi: Nothing to disclose
Mohammed Altahir: Nothing to disclose
Mohamed Elmutasim Abd El Bagi: Nothing to disclose
Hanaa Ahmed: Nothing to disclose
Madina Mustafa: Nothing to disclose
Suzan Alhassan: Nothing to disclose
Osman Elamin: Nothing to disclose

### RPS 100-9

**Contrast media in the water supply system: how to reduce the use of contrast media in CT scans**

*H. Dekker*, M. Prokop; Nijmegen/NL
*(heleen.dekker@radboudumc.nl)*

**Purpose:** To understand the environmental impact of iodinated contrast agents. To learn which measures can be taken to reduce the amount of contrast material in waste water. To understand the impact of these measures on contrast use and waste.
**Methods or Background:** For radiologists, contrast media are essential for diagnostic and interventional procedures. Data from The Netherlands shows that these contrast media are found in sewage water, surface water and drinking water. Contrast media cannot be removed from the water with current purification techniques and therefore pose a problem for drinking water preparation that has not been solved yet.
**Results or Findings:** At Radboudumc we have implemented the following approach: 1. Individually adjustment of the amount of intravascular iodinated contrast medium in CT to the weight of the patient and the clinical question. 2. Multi-patient injection system using bottle sizes of 50 – 500 ml adjusted to the expected aggregated use towards the upcoming scan hours. 3. Oral contrast media for abdominal CT largely replaced by water. 4. Collection of residues of contrast medium separately and dispose of them via the specific hospital waste. The following effects have been achieved by these measures: 1. The use of intravenous iodinated contrast medium is reduced by more than 10% 2. The use of oral contrast medium is reduced by more than 80% 3. Awareness and active anticipation has arisen among technicians.
**Conclusion:** Reducing the use of contrast media, both for intravenous and oral use, is a first step to limit the environmental impact of contrast media.
**Limitations:** n/a
**Ethics Committee Approval:** n/a
**Funding for this study:** n/a
**Author Disclosures:**
Mathias Prokop: nothing to disclose
Helena Dekker: nothing to disclose

### RPS 100-10

**Patient-centred care in diagnostic radiography: perceptions of service users, service deliverers, students, and educators**

*E. Hyde*[1], M. Hardy[2]; [1]Derby/UK, [2]Bradford/UK
*(e.hyde@derby.ac.uk)*

**Purpose:** To elicit the meaning of patient centred care (PCC) from the perspectives of service users (patients), service deliverers (radiographers and managers), radiography students and educators and determine whether these are aligned in terms of attached importance.
**Methods or Background:** A 2 stage approach was adopted. Stage 1 was a web-based attitudinal survey to explore understanding of PCC. Respondents were asked to indicate their level of agreement to paired (positive and negative phrasing) statements. Survey content was the same across participant groups but phrasing was tailored to respondent group. Pre-distribution piloting ensured suitability on survey language and questions. Data were analysed using descriptive statistics with impact of statement phrasing analysed using Wilcoxon signed rank test. Stage 2 was focus groups and telephone interviews with volunteer particpants from Stage 1. Situational vignettes were developed from Stage 1 responses and participants were asked to discuss these to elicit deeper understanding of PCC priorities from the differing perspectives. The interviews were audio-recorded and transcribed verbatim before being analysed thematically to draw out key themes. These were compared and contrasted across participant groups.
**Results or Findings:** While the core components of PCC identified by all groups were: technical competence; dignity and privacy; and human interaction, the interpretation and meaning placed on these and their reltive importance within PCC varied. Importantly, only patients identified the environment as important to PCC. Similarly only radiographers and radiography managers identified efficiency as important.
**Conclusion:** Patient centred care does not hold the same meaning for the different participant groups. In order to truly deliver PCC, the differing values and interpretations need to be aligned.
**Limitations:** None
**Ethics Committee Approval:** Ethical approval was granted by the University of Derby.
**Funding for this study:** This research was funded by the UK College of Radiographers Industry Partnership scheme.
**Author Disclosures:**
Emma Hyde: Nothing to disclose
Maryann Hardy: Nothing to disclose

### RPS 100-11

**Radiological "SATs" monitor: the use of "study ascribable times" to assess the impact of clinical workload on resident training in a resource-limited setting**

*C. E. Le Roux*; Cape Town/ZA
*(camilla.leroux@gmail.com)*

**Purpose:** Globally, increasing clinical demands threaten postgraduate radiology training programmes. Time-based assessment of clinical workload is optimal in the academic environment, where an estimated 30% of consultant time should ideally be devoted to non-reporting activities. There has been limited analysis of the academic radiologist workload in low- and middle-income countries.
**Methods or Background:** Departmental staffing and clinical statistics were reviewed for 2008 and 2017. The Royal Australian and New Zealand College of Radiologists 'study ascribable times' (RANZCR-SATs) for primary consultant reporting were used with the Royal College of Radiologists (RCR) 2012 guidelines for secondary review of resident reports, to estimate the total consultant-hours required for each year's clinical workload. Analyses were stratified by type of investigation (plain-film vs. special) and expressed as a proportion of the total annual available consultant working hours.
**Results or Findings:** Reporting all investigations required 90% and 100%, while reporting special investigations alone, demanded 53% and 69% of annual consultant working hours in 2008 and 2017, respectively. Between 2008 and 2017, the proportion of consultant time available for plain-film reporting decreased from 17% to 1%, while preserving 30% for non-reporting activities.
**Conclusion:** A time-based analysis of the academic radiologist's clinical workload, utilising the RANZCR-SATs and RCR 2012 guidelines for primary and secondary reporting, respectively, provides a reasonably accurate reflection of the service pressures in resource-constrained environments and has potential international applicability.
**Limitations:** A retrospective study was conducted and it is not possible to invoke a completely accurate and all-encompassing method of measuring radiologist workload and productivity.
**Ethics Committee Approval:** Granted
**Funding for this study:** None
**Author Disclosures:**
Camilla Engela Le Roux: Nothing to disclose

### RPS 100-12

**The COVID-19 outbreak on healthcare workers: survey on the perception of phase 1 of the HealthCare system: Italian radiographers' experience**
*C. Martini*[1], A. Devetti[2], M. Nicolò[3], C. Risoli[4], A. Tombolesi[5], O. Brazzo[6], D. Di Feo[7], W. Antonucci[8], M. Migliorini[9]; [1]Parma/IT, [2]Udine/IT, [3]Brescia/IT, [4]Piacenza/IT, [5]Torino/IT, [6]Bari/IT, [7]Florence/IT, [8]Barletta/IT, [9]Ferrara/IT
*(martinic@ao.pr.it)*

**Purpose:** To evaluate the Italian radiographers experience during the Phase 1 COVID-19 Outbreak.
**Methods or Background:** The COVID-19 (C19) outbreak had since spread rapidly worldwide. Many patients even with and without symptoms, underwent radiological examinations, with an high risk of infection for radiology department's staff. The first-hit European country to face the outbreak of C19 was Italy and the impact on radiographers (RT) was huge. It was a cross-sectional study consisting of a web-based survey to investigate the attitudes and the level of awareness of Healthcare Workers (HCW) during first wave C19 pandemic. The survey was administered via social media by the Italian Association of CT and Pediatric RT, Italian Risk Management Group, Italian Association of System and Telehealth Administrators, and Italian Federation of Scientific RT Societies, respectively. The survey was available anonymously online from March 7 th 2020 to May 31. The questions concerned: demographics, working environment, knowledge on Covid-19/SARS/MERS, radiological equipment for X-ray and CT, presence of dedicated paths/protocols, PPE, sanification and disinfection.
**Results or Findings:** A total of 1.109 responded to the survey. Of these, 35% were women. HCW were mostly RT (95%), from high-prevalence regions (68%). 73% worked in a public hospital getting defined protocol for generical infection, and pathway for COVID-19. While only 38% was aware to SARS/MERS protocol. 55% had a dedicated both X-ray and CT for COVID-19 patients, without exhaustive guidelines. Among the tested population, 40% performed a radiological exam in pairs. Only 21% of HCW considered PPE adequate for quality and quantity.
**Conclusion:** Healthcare institutions should have ongoing training for HCWs and discussions around protocol for infection transmission. Management strategies should be promptly undertaken to enhance safety and optimize resource allocation.
**Limitations:** Nothing to declare
**Ethics Committee Approval:** Nothing to declare
**Funding for this study:** Nothing to declare
**Author Disclosures:**
Camilla Risoli: Nothing to disclose
Alessandro Tombolesi: Nothing to disclose
Walter Antonucci: Nothing to disclose
Angie Devetti: Nothing to disclose
Marco Nicolò: Nothing to disclose
Oscar Brazzo: Nothing to disclose
Chiara Martini: Nothing to disclose
Matteo Migliorini: nothing to disclose
Daniele Di Feo: Nothing to disclose

### RPS 100-13

**Radiology residents' feedback on initial training by social media in a limited resource sub-Saharan setting during the COVID-19 pandemic**
*M. E. Abd El Bagi*, A. Jabir, K. Tia, M. M. Fadol, W. Almansoori, I. Abd Elgadir, T. Nori, E. Eltayeb, R. Abugsaisa; Khartoum/SD
*(profmutasim@gmail.com)*

**Purpose:** Residents' evaluation of training by social media.
**Methods or Background:** Radiology residents in limited-resource settings of Sub-Saharan Africa compete for the same British Fellowships and European Diploma exams with their privileged western counterparts. There is a lack of literature about radiology-infrastructure in Africa. Little is known about the use of social media in radiology training. The choice of the most suitable social media at the local circumstances was not studied before. This paper reports the hurdles facing local trainees and their evaluation of an initial voluntary training program using social media. Methods A quantitative questionnaire was posted on google forms software. Sudanese radiology residents were invited to participate via their What's App group. Expatriates and those trapped outside the country were excluded. Participants were asked about their experience during the first 3month peak of COVID19 spread in the country.
**Results or Findings:** There were 91 residents in the town of whom 53(59%) responded. 47 (88.7%) suffered difficulties related to the care few. Onsite training stopped completely for24(45.3%) of residents due to the pandemic. The most practical platform was What's App according to 27(50.9%) of the residents. 9( 17%) recommended virtual learning full time but 12(22.6%) indicated that virtual teaching should not replace onsite activities.34(64.7%) of residents reported technical internet inaccessibility due to a weak district internet signal or interrupted electric power supply. The care few procedures were restrictive.
**Conclusion:** The pandemic has challenged the training program with unparalleled hurdles. Social media use is unavoidable albeit privacy risk and discontinuity. WhatsApp was the most favored. A need exists for a booster external attachment training programs, perhaps sponsored by ECR and RCR.
**Limitations:** The results were not gender-specific.
**Ethics Committee Approval:** Sudan Medical Specialisation Board approval.
**Funding for this study:** None
**Author Disclosures:**
Tsabeeh Nori: Nothing to disclose
Eman Eltayeb: Nothing to disclose
Randa Abugsaisa: Nothing to disclose
Weam Almansoori: Nothing to disclose
Khalid Tia: Nothing to disclose
Abdallah Jabir: Nothing to disclose
Mohamed Elmutasim Abd El Bagi: Nothing to disclose
Iman Abd Elgadir: Nothing to disclose
Muhammed Mustafa Fadol: Nothing to disclose

### RPS 100-14

**Does levosimendan prevent contrast medium nephrotoxicity in rats?**
*I. Durur Subasi*[1], D. Kose[2], M. Yayla[3], B. Sirin[2], A. Karaman[2], I. Calik[2], F. Alper[2]; [1]Istanbul/TR, [2]Erzurum/TR, [3]Kars/TR
*(irmakdurur@yahoo.com)*

**Purpose:** To study the possible nephroprotective influences of levosimendan for contrast medium nephrotoxicity in the kidney of rat.
**Methods or Background:** With the permissions of IRB, the experiment consisted of 8 rat groups having 6 rats in each cage. Nephrotoxicity was provoked by one-day water withdrawal and injection of glycerol. Contrast medium nephrotoxicity was encouraged by IV delivery of iohexol. The first group was not given any medications as the control group. The other groups were given some medications as follows: Glycerol for the second group; contrast medium (iohexol 10 ml/kg) for third; glycerol and contrast medium for fourth; contrast medium and levosimendan 0.25 mg/kg for fifth; glycerol, contrast medium and levosimendan 0.25 mg/kg for sixth:, contrast medium and levosimendan 0.5 mg/kg for seventh; glycerol, contrast medium and levosimendan 0.5 mg/kg for eight groups.
**Results or Findings:** The serum blood urea nitrogen and creatinine levels, superoxide dismutase activity, glutathione, and malondialdehyde levels were considerably developed by levosimendan direction. Levosimendan significantly down-regulated the values of tumor necrosis factor-α, nuclear factor-kappa ß, and interleukin 6 mRNA. The histopathological marks of hyaline and hemorrhagic casts and tubular necrosis were improved in the levosimendan administered ones.
**Conclusion:** Levosimendan has nephroprotective properties attributable to its possessions falling oxidative distress and preventing the secretion of pro-inflammatory cytokines against contrast medium nephrotoxicity in rats.
**Limitations:** A limited number of rats.
Only two different (not multiple) dose administration.
**Ethics Committee Approval:** Exists.
**Funding for this study:** No funding.
**Author Disclosures:**
Duygu Kose: Nothing to disclose
Busra Sirin: Nothing to disclose
Muhammed Yayla: Nothing to disclose
Adem Karaman: Nothing to disclose
Fatih Alper: Nothing to disclose
Irmak Durur Subasi: Nothing to disclose
Ilknur Calik: Nothing to disclose

### RPS 100-15

**Medical devices in the abdominal x-ray: what the radiologist needs to know**

*D. C. Carvalho*, F. L. Ferreira, M. Morgado, A. T. Vilares, S. C. Dias, A. J. Madureira; Porto/PT

**Purpose:** To review and illustrate many of the abdominal and pelvic devices that a radiologist may encounter in X-ray studies, going through how to recognize them, as well as their indications and related complications.
**Methods or Background:** Due to technology advancements and the increasing number of imaging studies, it's getting more common for radiologists to find devices on abdominal X-rays, some of which they might not be familiar with. This can complicate the interpretation of the images. Failing to recognize the device(s) present in a certain X-ray study might compromise the accuracy of the diagnosis, and lead the radiologist to miss an incorrectly placed device or a related complication. It's very important for the radiologist to become familiar with the radiologic appearance of medical devices, and their evaluation should be considered as important as any other aspect in a radiologic examination.
**Results or Findings:** Medical devices in abdominal x-rays can be divided into a few major groups for simplification purposes, namely gastrointestinal, genitourinary, surgical and neurologic devices. Some of them are more commonly seen, such as nasogastric tubes, gastric banding, gastrostomy and jejunostomy, Foley catheters, ureteric stents, intrauterine contraceptive devices ou tubal sterilization devices, surgical clips or drains and endoprosthetic stents, just to name a few. Others are seen more rarely, such as neurostimulators, suprapubic cystotomies or nephrostomy tubes, embolization material, penile prostheses or a vaginal pessaries.
**Conclusion:** Since there is a wide variety of devices that can be found during an X-ray study of the abdomen and pelvis, it is important for the radiologist to be familiar with them, not only with their accurate placement, but also with complications that might be associated, so that he can timely inform the referring clinician.
**Limitations:** n/a
**Ethics Committee Approval:** n/a
**Funding for this study:** n/a
**Author Disclosures:**
Ana Teresa Vilares: Nothing to disclose
António J. Madureira: Nothing to disclose
Sílvia Costa Dias: Nothing to disclose
Fábio Lopes Ferreira: Nothing to disclose
Margarida Morgado: Nothing to disclose
Diogo Costa Carvalho: Nothing to disclose

### RPS 100-16

**Gender trends in authorships and publication impact**

*I. Molwitz*; Hamburg/DE

**Purpose:** To investigate publication numbers of female authors in 10 high to low impact radiological journals between 2007/8 and 2017/18.
**Methods or Background:** In this retrospective bibliometric analysis original research articles and reviews published in 2007/8 and 2017/18 in high, medium, and low impact journals according to Clarivate analytics web of science's journal citation reports were reviewed with regards to the gender of the first (FA) and senior author (SA). The adjusted event rate (AER) and adjusted odds ratio (AOR) were calculated by mixed logistic and multinomial logistic regression models to assess likelihood of female publications according to impact factor, journal, author position and combination.
**Results or Findings:** The proportion of female FA and female SA in N=6979 (2007/8) and N=7383 (2017/18) articles, increased by 58% and 61%, respectively. While most female authorships were continuously observed in medium impact journals, the strongest increase occurred for both female FA (AOR 2.0; $P < .0001$) and SA (AOR 2.1; $P < .0001$) in low impact journals. Female SA were significantly more likely to publish in a low (AOR 1.5) or medium ranking journal (AOR 1.8) than in a high ranking one. Same-sex male collaborations were more likely than female SA collaborations across journals of all impact spates.
**Conclusion:** A ten-year positive trend in the development of female authorships was observed in all impact areas. Gender disparities however still exist, particularly with regard to female senior positions and high impact factor.
**Limitations:** The analysis was focused on FA and SA and does not provide information about co-authors. Statistical analysis was corrected for authors of unclear gender.
**Ethics Committee Approval:** Institutional review board exemption was obtained.
**Funding for this study:** This work has not received any funding.
**Author Disclosures:**
Isabel Molwitz: Nothing to disclose

### RPS 100-17

**Everything you always wanted to know about contrast media (but were afraid to ask)**

*J. M. Felices Farias*, M. D. Abellan-Rivero, P. P. Ortuño-Lopez, V. Martinez-Jimenez, A. Perez-Ballesta, F. Barqueros Escuer, D. San Leandro Pardo, A. Puerta-Sales; El Palmar/ES
*(jmfelicesfarias@gmail.com)*

**Purpose:** - To learn the main information to know about the patient before contrast media use - To know the complications related to contrast media use and how to prevent them - To review which patients should be premedicated and how to premedicate.
**Methods or Background:** Contrast media is broadly used in Radiology departments. Nowadays they are mostly safe and complications related to contrast media are rare. The most common complications related to contrast media are the anafilactoid reactions and the acute kidney injury but there is not enough known which patients could develop them and how to properly prevent these kinds of complications.
**Results or Findings:** In clinical practice it is common to doubt if an image study might or not be performed because of potential complications, which could lead to infradiagnosis. Most of these situations could be avoided through an optimal premedication, but it is main to know which patients get benefit of the premedication and how to set it. An algorithm to recognize patients in risk to develop contrast media related complications is shown according to a review of the relevant risk factors. It is also purposed the suitable management of these patients in risk, based on potential adverse anafilactoid reactions or development of an acute kidney injury.
**Conclusion:** To use an algorithm to check the relevant information due to premedicate the patients in risk of contrast media related complications will allow clinicians and radiologists to avoid infradagnosis and to perform safer image studies.
**Limitations:** It is based on retrospective study.
**Ethics Committee Approval:** No need of ethics committee approval
**Funding for this study:** No funding received
**Author Disclosures:**
José Manuel Felices Farias: Speaker: Hospital Clinico Universitario Virgen de la Arrixaca Author: Hospital Clinico Universitario Virgen de la Arrixaca
Ana Perez-Ballesta: Author: Hospital Clinico Universitario Virgen de la Arrixaca
Maria Dolores Abellan-Rivero: Author: Hospital Clinico Universitario Virgen de la Arrixaca
David San Leandro Pardo: Author: Hospital Clinico Universitario Virgen de la Arrixaca
Victor Martinez-Jimenez: Author: Hospital Clinico Universitario Virgen de la Arrixaca
Pedro Pablo Ortuño-Lopez: Author: Hospital Clinico Universitario Virgen de la Arrixaca
Francisco Barqueros Escuer: Author: Hospital Clinico Universitario Virgen de la Arrixaca
Alejandro Puerta-Sales: Author: Hospital Clinico Universitario Virgen de la Arrixaca

### RPS 100-18

**Optimising patient and staff safety during a pandemic: practical infection control measures for MRI scanning**

*B. J. Nugent*; Edinburgh/UK
*(bnugent@btopenworld.com)*

**Purpose:** Lockdown measures such as postponing elective MRI scans were designed to ensure capacity for an expected surge in COVID-19 cases and to reduce the likelihood of nosocomial spread. With the easing of restrictions comes pressure to resume routine scanning and process the backlog of examinations. As criteria for infection control during this pandemic evolves, establishing current infection control processes and sharing expertise may encourage best practice.
**Methods or Background:** A snapshot survey, via email, asked UK MRI professionals what strategies were in place to try to protect against contracting COVID-19 in the workplace. The results led to a collaborative project to develop a series of infection control measures to consider.
**Results or Findings:** A learning resource developed providing practical and innovative steps, within an outpatient MRI patient pathway, to try to reduce the risk of infection emphasising the importance of: • Health surveillance checks • Eye protection for staff if a patient is not wearing a mask • Regular COVID-19 testing of staff • Tools/workflow intervention to ensure physical distancing • Cleaning/disinfection/decontamination guidance • Workplace/personal risk assessments • MRI safety labelling/classification of respiratory personal protective equipment (PPE)

**Conclusion:** Many of the proposals could be introduced into any outpatient setting and may assist in promoting a benchmark of infection control standards to follow, as further evidence about COVID-19 emerges. Maintaining recommended safe distances isn't possible when scanning. Identifying infected cases is problematic. A multitiered approach, which filters out the potentially infected before they reach the unit is required. The complex nature of MRI presents challenges to cleaning, disinfecting and decontamination with respiratory PPE not labelled for MRI safety conditions. These interventions may help reduce such risks and optimise service efficiency.
**Limitations:** Limited number of responses
**Ethics Committee Approval:** Not applicable
**Funding for this study:** Not applicable
**Author Disclosures:**
Barbara J. Nugent: Nothing to disclose

### E³ - Advanced Courses: Artificial Intelligence

### E³ 122

### Artificial intelligence (AI) in radiology: the basics you need to know

**E³ 122-1**
**A. Conventional machine learning vs deep learning**
Marleen de Bruijne; Rotterdam/NL

**Learning Objectives:**
1. To understand the difference between machine learning and deep learning.
2. To learn about the various conventional machine learning techniques.
3. To learn about pros and cons of conventional machine learning vs deep learning.
**Author Disclosures:**
Marleen de Bruijne: Research Grant/Support: NWO, TTW, Intel, Quantib

**E³ 122-2**
**B. Training data for deep learning: what is needed?**
Thomas Weikert; Basle/CH
*(thomasjohannes.weikert@usb.ch)*

**Learning Objectives:**
1. To learn about what is needed to train neural networks in radiology.
2. To learn about the sources of training data for radiology projects as well as their pros and cons.
3. To learn about the peculiarities of data in radiology and what they mean for the training of algorithms.

**E³ 122-3**
**C. Clinical applications of artificial intelligence (AI) in medical imaging**
Nickolas Papanikolaou; Lisbon/PT
*(nickolas.papanikolaou@research.fchampalimaud.org)*

**Learning Objectives:**
1. To learn about the current state of the art of AI applications in medical imaging.
2. To focus on the current challenges related to AI development and deployment in clinical conditions.
3. To understand how AI will transform medical imaging in the long term.
**Author Disclosures:**
Nickolas Papanikolaou: Owner: MRIcons LTD

### E³ - Advanced Courses: Artificial Intelligence

### E³ 222

### Artificial intelligence for image reconstruction: towards deep imaging?

**E³ 222-1**
**A. Deep learning for MRI reconstruction**
Kerstin Hammernik; London/UK

**Learning Objectives:**
1. To demonstrate how with deep learning we can learn the entire MRI reconstruction procedure.
2. To understand the advantages and disadvantages of using deep learning in MRI reconstruction.
3. To demonstrate the application of deep learning in the reconstruction of MRI musculoskeletal images.

**E³ 222-2**
**B. Deep learning in cardic MRI acquisition and reconstruction**
Daniel Rueckert; London/UK
*(D.Rueckert@imperial.ac.uk)*

**Learning Objectives:**
1. To understand how MRI acquisition time can be reduced with deep learning.
2. To understand the potential artefacts related to deep learning-based image reconstruction.
3. To demonstrate the applications of deep learning in the reconstruction of cardiovascular MRI data.
**Author Disclosures:**
Daniel Rueckert: Consultant: Heartflow, IXICO, Circle Cardiovascular Imaging

**E³ 222-3**
**C. Deep learning in CT image acquisition and reconstruction**
Mathias Prokop; Nijmegen/NL
*(mathias.prokop@radboudumc.nl)*

**Learning Objectives:**
1. To learn how deep learning can be used to improve CT image quality.
2. To understand how deep learning can be used to speed up CT image acquisition.
3. To learn about clinical applications of deep learning-based CT reconstruction.
**Author Disclosures:**
Mathias Prokop: Patent Holder: 4D noise suppresion, licensed to Canon Medical Systems; Research Grant/Support: Canon Medical Systems, Siemens Healthineers; Speaker: Canon Medical Systems, Siemens Healthineers

### E³ - Advanced Courses: Artificial Intelligence

### E³ 322

### Artificial intelligence and translations to clinical practice

**E³ 322-1**
**A. Artificial intelligence (AI) use cases**
Bibb Allen, Jr.; Birmingham, AL/US
*(bibb@mac.com)*

**Learning Objectives:**
1. To introduce the audience to the AI use cases developed by the American College of Radiology.
2. To learn about an ecosystem for developing AI algorithms that can be translated into clinical practice.
3. To discuss the hurdles and proposed solutions to getting regulatory approval for AI techniques.

**E³ 322-2**
**B. Challenges to objectively compare performance of AI applications**
Lena Maier-Hein; Heidelberg/DE

**Learning Objectives:**
1. To learn about the outcomes of a review of more than 150 challenges in medical imaging.
2. To learn about the metrics allowing the objective evaluation of AI algorithm performance.
3. To understand how new developments in AI challenges help to objectively evaluate the performance of algorithms.

**E³ 322-3**
**C. How far are we in getting AI into clinical practice?**
Luis Martí-Bonmatí; Valencia/ES
*(marti_lui@gva.es)*

**Learning Objectives:**
1. To critically review the current level of AI adoption in clinical practice.
2. To understand the need of data scientists working in radiology departments.
3. To discuss what next steps need to be taken in order to increase take-up in clinical practice.
**Author Disclosures:**
Luis Martí-Bonmatí: Advisory Board: QUIBIM

### E³ - Advanced Courses: Artificial Intelligence

### E³ 422

### Radiomics: principles and applications

**E³ 422-1**
**A. Radiomics: images are data!**
Giuseppe Cicchetti; Rome/IT
*(cicchetti.giuseppe88@gmail.com)*

**Learning Objectives:**
1. To understand the design of a pipeline to develop a radiomics signature.
2. To learn about the current state of the art in radiomics.
3. To learn about the impact of radiomics for precision medicine.

**E³ 422-2**
**B. Radiomics applications**
Tobias Penzkofer; Berlin/DE

**Learning Objectives:**
1. To learn about the state of the art of radiomics in prostate cancer.
2. To learn about the state of the art of radiomics in gliomas.
3. To learn about other promising application areas of radiomics.
**Author Disclosures:**
Tobias Penzkofer: Employee: Charité Universitätsmedizin Berlin; Research Grant/Support: Berlin Institute of Health, AGO, Aprea AB, ARCAGY-GINECO, Astellas Pharma Global Inc. (APGD), Astra Zeneca, Clovis Oncology, Inc., Dohme Corp, Elsevier, Holaira, Incyte Corporation, Karyopharm, Lion Biotechnologies, Inc., MedImmune, Merck Sharp, Millennium Pharmaceuticals, Inc., Morphotec Inc., NovoCure Ltd., PharmaMar S.A. and PharmaMar USA, Inc., Roche, Siemens Healthineers, and TESARO Inc.

**E³ 422-3**
**C. Multicentre studies for more robust radiomics signatures**
Martijn Starmans; Rotterdam/NL
*(m.starmans@erasmusmc.nl)*

**Learning Objectives:**
1. To introduce a framework for the rapid development of radiomics signatures.
2. To explain how the parameters of a radiomics pipeline can be automatically optimised.
3. To show the performance of the framework for a number of applications.

### E³ - Advanced Courses: Artificial Intelligence

### E³ 522

### Artificial intelligence and clinical decision support

**E³ 522-1**
**A. Clinical decision support workflow improved by artificial intelligence (AI)**
Erik R. Ranschaert; Tilburg/NL

**Learning Objectives:**
1. To learn how a decision support workflow can be supported and improved by AI.
2. To understand the different workflow parts in which AI can play a role.
3. To discuss how to evaluate the clinical value of AI in decision support.
**Author Disclosures:**
Erik R. Ranschaert: Advisory Board: Osimis, Diagnose.me; Consultant: Barco, Robovision; Share holder: Osimis, Diagnose.me

**E³ 522-2**
**B. Data mining and machine learning for integrated clinical decision support**
Giles Boland; Boston, MA/US
*(gboland@partners.org)*

**Learning Objectives:**
1. To understand how data mining can help in clinical decision support.
2. To learn about the needs and limitations of standardisation for AI-assisted clinical decision support.
3. To learn about the state of the art in AI-assisted clinical decision support.
**Author Disclosures:**
Giles Boland: Other: royalities Elsevier

**E³ 522-3**
**C. AI to predict treatment response**
Nandita M. deSouza; Sutton/UK

**Learning Objectives:**
1. To understand the role of AI in moving towards precision medicine.
2. To understand the current potential of AI for monitoring response.
3. To understand how to manage AI in a clinical workflow as a decision support tool.

### E³ - Advanced Courses: Artificial Intelligence

### E³ 622

### Challenges and solutions for introducing artificial intelligence (AI) in daily clinical workflow

**E³ 622-1**
**A. Implementation of AI algorithms in picture archiving and communication systems (PACS)**
Wouter B. Veldhuis; Utrecht/NL

**Learning Objectives:**
1. To learn about how to start experimenting with AI in daily clinical routine.
2. To learn about the developments of integrating multiple AI tools within one framework.
3. To learn about the processes to evaluate AI algorithms for clinical use cases.
**Author Disclosures:**
Wouter B. Veldhuis: Other: CoFounder of QuantibU

$E^3$ 622-2
**B. How to best complement human intelligence with AI**
Christian J. Herold; Vienna/AT
*(Christian.Herold@meduniwien.ac.at)*

**Learning Objectives:**
1. To understand the current and emerging concept for AI and machine learning in imaging.
2. To explore whether it is possible to successfully integrate AI into clinical practice today.
3. To learn how radiologists can be assisted by AI.

**Author Disclosures:**
Christian J. Herold: Advisory Board: Siemens Healthineers; Board Member: Context Flow; Research Grant/Support: Siemens Healthineers, Bracco, Bayer Healthcare and 115 other companies; Share holder: Hologic Inc.

$E^3$ 622-3
**C. AI, ethics, and radiology**
Adrian Brady; Cork/IE
*(adrianbrady@me.com)*

**Learning Objectives:**
1. To understand the ethical aspects related to data use in AI.
2. To learn about possible bias in AI algorithms.
3. To learn how to prepare radiology policies for AI.

$E^3$ 622-4
**D. AI in radiology: culture change**
Howard Fleishon; Atlanta, GA/US
*(hfleishon@outlook.com)*

**Learning Objectives:**
1. To review the possible changes in radiology practices and departments due to the implementation of AI workflows.
2. To present the possible impact of AI on radiology macroeconomics.
3. To discuss the educational innovations in introducing AI into radiology resident training.

### $E^3$ - Advanced Courses: Artificial Intelligence

*Jointly organised by the ESR and EIBALL*

### $E^3$ 722

### Making the invisible visible: pushing the boundaries in multimodality radiomic quantification

$E^3$ 722-1
**A. Unravelling the mysteries of the black box: does radiomics enhance or complement biomarker data?**
Ana Jiménez-Pastor; Valencia/ES
*(anajimenez@quibim.com)*

**Learning Objectives:**
1. To learn what radiomic analyses add to current biomarker evaluation.
2. To appreciate the limitations and pitfalls of such analyses.
3. To understand the most robust statistical methods for carrying out the analyses.

**Author Disclosures:**
Ana Jiménez-Pastor: Employee: QUIBIM SL

$E^3$ 722-2
**B. Working across modalities: how do we progress from redundant to relevant data?**
Philippe Lambin; Maastricht/NL

**Learning Objectives:**
1. To learn how radiomic outputs differ across modalities.
2. To appreciate the variability of the method and how to ensure data is robust.
3. To understand the relevance of the findings.

$E^3$ 722-3
**C. Using radiomics in the clinic: a decision support tool?**
Marius E. Mayerhöfer; Vienna/AT
*(marius.mayerhoefer@meduniwien.ac.at)*

**Learning Objectives:**
1. To learn the clinical scenarios where radiomic analyses may be helpful.
2. To appreciate the place of these analyses in the context of other quantifiable biomarkers.
3. To understand the implications of using these analyses in the clinic.

**Author Disclosures:**
Marius E. Mayerhöfer: Speaker: Siemens, BMS

### $E^3$ - Advanced Courses: How to Improve Your Expertise in Cardiothoracic Imaging

### $E^3$ 919

### Low-dose thoracic CT: only screening for lung cancer?

$E^3$ 919-1
**A. Overview of lung cancer screening activities in European countries**
Mario Silva; Parma/IT
*(mariosilvamed@gmail.com)*

**Learning Objectives:**
1. To be aware of the latest results of lung cancer screening trials.
2. To learn about the best technical standards for lung cancer screening.
3. To learn about the performance of artificial intelligence algorithms for lung cancer prediction.

$E^3$ 919-2
**B. Lung nodule management**
Anna Rita Larici; Rome/IT
*(annarita.larici@unicatt.it)*

**Learning Objectives:**
1. To be aware of the common causes of solitary pulmonary nodules.
2. To learn about key radiological features suggesting a benign cause.
3. To learn about the methods allowing identifying malignant nodules.

$E^3$ 919-3
**C. Coronary artery disease assessment as part of a lung cancer screening programme: how to do it?**
Rozemarijn Vliegenthart; Groningen/NL
*(r.vliegenthart@umcg.nl)*

**Learning Objectives:**
1. To become familiar with the importance and predictive power of coronary calcium assessment.
2. To discuss the possibilities of combining coronary calcium assessment and lung cancer screening.
3. To learn about the practical implementation of coronary calcium assessment in the routine chest.

**Author Disclosures:**
Rozemarijn Vliegenthart: Grant Recipient: Institutional Research grant, Siemens Healthineers; Other: honorarium as Associate Editor, Eur J Radiology

### $E^3$ - Advanced Courses: How to Improve Your Expertise in Cardiothoracic Imaging

### $E^3$ 1219

### Infections of the chest

$E^3$ 1219-1
**A. Pulmonary infections**
Jiri Neuwirth; Prague/CZ
*(neuwirthj@gmail.com)*

**Learning Objectives:**
1. To review the role and limitations of chest-x ray for diagnosing community-acquired pneumonia.
2. To learn about pulmonary infections in immunocompromised hosts.
3. To become familiar with CT signs suggesting fungal infection.

**E³ 1219-2**
**B. Tuberculosis (TB)**
Igor E. Tyurin; Moscow/RU

**Learning Objectives:**
1. To evaluate the imaging patterns of thoracic tuberculosis.
2. To review the different imaging features of the disease in immune compromised patients.
3. To discuss the current role of imaging in nontuberculous mycobacterial disease.

**E³ 1219-3**
**C. Infectious endocarditis**
Hatem Alkadhi; Zurich/CH

**Learning Objectives:**
1. To review the valvular consequences of untreated infections.
2. To become familiar with the CT signs of endocarditis in native valves.
3. To learn about the typical appearance of infectious complications after valvular surgery.

### E³ - Advanced Courses: How to Improve Your Expertise in Cardiothoracic Imaging

### E³ 1519

### Mediastinal and cardiac tumours in adults

**E³ 1519-1**
**A. Prevascular compartment of the mediastinum**
José Vilar; Valencia/ES
*(vilarsamper@gmail.com)*

**Learning Objectives:**
1. To become familiar with the main causes of prevascular tumours.
2. To review the CT features of thymic malignancies.
3. To learn when to suspect lymphoma and when to suggest percutaneous biopsy.

**E³ 1519-2**
**B. Paravertebral space**
Mariaelena Occhipinti; Florence/IT

**Learning Objectives:**
1. To become familiar with the posterior mediastinal pathology.
2. To review the typical and atypical features of neurogenic tumours.
3. To learn about less frequent causes of paravertebral space masses.

**E³ 1519-3**
**C. Cardiac masses: a survival guide**
Valentin E. Sinitsyn; Moscow/RU
*(vsini@mail.ru)*

**Learning Objectives:**
1. To learn how to differentiate thrombi from tumours of the cardiac cavities.
2. To review the main differential diagnosis of cardiac tumours.
3. To learn about the role of US, CT, and MRI for diagnosis and characterisation.
**Author Disclosures:**
Valentin E. Sinitsyn: Advisory Board: Comany Botkin AI (Russia); Consultant: Comany PET-Technology (Russia); Speaker: GE healthcare Satellite Symposium at ECR 2021

### E³ - Advanced Courses: How to Improve Your Expertise in Cardiothoracic Imaging

### E³ 1619

### Pulmonary embolism/pulmonary hypertension

**E³ 1619-1**
**A. Diagnosis of acute pulmonary embolism (PE)**
Marie-Pierre Revel; Paris/FR
*(marie-pierre.revel@aphp.fr)*

**Learning Objectives:**
1. To review the role of clinical probability scores and D-dimer assessment.
2. To learn about tips and tricks to optimise arterial opacification on CT.
3. To learn about key features to report in acute PE.
**Author Disclosures:**
Marie-Pierre Revel: Speaker: Lecture fees from MSD; Other: Travel support from GUERBET

**E³ 1619-2**
**B. Pulmonary hypertension**
Anagha P. Parkar; Bergen/NO

**Learning Objectives:**
1. To become familiar with the causes of pulmonary hypertension.
2. To learn about the radiological presentation of pulmonary artery hypertension.
3. To understand how imaging helps patient management.

**E³ 1619-3**
**C. The heart in pulmonary hypertension**
Karl-Friedrich Kreitner; Mainz/DE
*(Karl-Friedrich.Kreitner@unimedizin-mainz.de)*

**Learning Objectives:**
1. To learn about cardiac causes of pulmonary hypertension.
2. To become familiar with cardiac assessment in pulmonary hypertension.
3. To recognise the prognostic importance of right ventricle dysfunction in pulmonary hypertension.

### E³ - Advanced Courses: Interactive Teaching Sessions for Young (and not so Young) Radiologists

### E³ 121

### Musculoskeletal tumours

**E³ 121-1**
**A. Soft tissue tumours**
Filip M.H.M. Vanhoenacker; Antwerp/BE
*(filip.vanhoenacker@telenet.be)*

**Learning Objectives:**
1. To describe the imaging findings of soft tissue tumours.
2. To review the differential diagnosis of soft tissue tumours and tumour-like conditions.

**E³ 121-2**
**B. Bone tumours**
F. Bilge Ergen; Ankara/TR
*(bergen@delta-eur.com)*

**Learning Objectives:**
1. To describe imaging findings of musculoskeletal tumours and tumour-like conditions.
2. To recognise imaging pitfalls of musculoskeletal tumours and tumour-like conditions.

### E$^3$ - Advanced Courses: Interactive Teaching Sessions for Young (and not so Young) Radiologists

### E$^3$ 221

### Emergency and chest radiology

**E$^3$ 221-1**
**A. Dyspnoea in oncologic patients: how to approach it**
Cornelia M. Schaefer-Prokop; Amersfoort/NL
*(cornelia.schaeferprokop@gmail.com)*

**Learning Objectives:**
1. To learn about the role of imaging in oncologic patients with dyspnoea.
2. To understand the importance of clinical data for narrowing the differential diagnosis

**E$^3$ 221-2**
**B. Blunt thoracic trauma: from the plain film to CT**
Anastasia Oikonomou; Toronto, ON/CA
*(anastasia.oikonomou@sunnybrook.ca)*

**Learning Objectives:**
1. To learn about the spectrum of radiological findings in blunt thoracic trauma.
2. To emphasise the importance of CT reformations for the detection of severe complex traumatic injuries.

### E$^3$ - Advanced Courses: Interactive Teaching Sessions for Young (and not so Young) Radiologists

### E$^3$ 321

### Imaging of the liver

**E$^3$ 321-1**
**A. CT and MRI liver imaging reporting and data system (LI-RADS): how to use it and what to expect**
Anna Darnell; Barcelona/ES

**Learning Objectives:**
1. To become familiar with LI-RADS categories, definitions and algorithms.
2. To discuss the confusing features of LI-RADS with examples.
3. To understand what the probability of hepatocellular carcinoma is for each LI-RADS category.

**E$^3$ 321-2**
**B. Focal lesions in non-cirrhotic liver: how to diagnose, differentiate and manage**
Giuseppe Brancatelli; Palermo/IT
*(gbranca@yahoo.com)*

**Learning Objectives:**
1. To learn how to differentiate focal liver lesions in non-cirrhotic patients with CT and MRI.
2. To know how to manage non-cirrhotic patients with focal liver lesions.
**Author Disclosures:**
Giuseppe Brancatelli: Consultant: Bayer; Speaker: Bayer, GE Healthcare

### E$^3$ - Advanced Courses: Interactive Teaching Sessions for Young (and not so Young) Radiologists

### E$^3$ 421

### Small bowel imaging

**E$^3$ 421-1**
**A. CT and MR enterography: my technical tips for preparation and scanning**
António J.B.S. Madureira; Porto/PT
*(ajbmadureira@gmail.com)*

**Learning Objectives:**
1. To understand the importance of proper preparation for enterography.
2. To be familiar with technical details that ensures high imaging quality.

**E$^3$ 421-2**
**B. Detection of small bowel involvement in patients with peritoneal carcinomatosis before hyperthermic intraperitoneal chemotherapy (HIPEC)?**
Michael R. Torkzad; Stockholm/SE
*(michael.torkzad@gmail.com)*

**Learning Objectives:**
1. To understand how to optimise imaging techniques to detect peritoneal involvement of the small bowel.
2. To be familiar the various imaging characteristics of peritoneal involvement of the small bowel.

### E$^3$ - Advanced Courses: Interactive Teaching Sessions for Young (and not so Young) Radiologists

### E$^3$ 521

### Musculoskeletal radiology: arthropathies

**E$^3$ 521-1**
**A. Extremities**
Üstün Aydingöz; Ankara/TR
*(uaydingo@hacettepe.edu.tr)*

**Learning Objectives:**
1. To explain the key points in the differential diagnosis of common arthropathies in the extremities.
2. To describe the imaging findings of common arthropathies in the extremities as they relate to pathophysiology.

**E$^3$ 521-2**
**B. The axial skeleton**
Apostolos H. Karantanas; Iraklion/GR
*(akarantanas@gmail.com)*

**Learning Objectives:**
1. To explain the key points in the differential diagnosis of arthropathies in the axial skeleton.
2. To describe the imaging findings of arthropathies in the axial skeleton as they relate to pathophysiology.

### E³ - Advanced Courses: Interactive Teaching Sessions for Young (and not so Young) Radiologists

### E³ 621

### Neuroradiology: paediatric and adult

**E³ 621-1**
**A. Imaging in epilepsy: how to scan and find the suspect**
Michael Okujava; Tbilisi/GE

**Learning Objectives:**
1. To describe best imaging protocols when considering the patient's age.
2. To review common epileptogenic lesions and their imaging features.
3. To define contributions from advanced imaging.

**E³ 621-2**
**B. Imaging in movement disorders: keeping up with the neurologist**
Kader Karli Oguz; Ankara/TR
*(karlioguz@yahoo.com)*

**Learning Objectives:**
1. To review common movement disorders and basic mechanisms.
2. To define the role of radiologic examinations and review ideal imaging protocols.
3. To review diagnostic radiologic patterns in classified movement disorders.

### E³ - Advanced Courses: Interactive Teaching Sessions for Young (and not so Young) Radiologists

### E³ 721

### Cardiac imaging: an update

**E³ 721-1**
**A. Coronary artery disease - reporting and data system (CAD-RADS): a new tool for reporting coronary CT angiograms (CTAs)**
Bálint Szilveszter; Budapest/HU
*(szilveszter.balint@gmail.com)*

**Learning Objectives:**
1. To be familiar with standardised reporting of coronary CTA findings.
2. To know the impact of CAD-RADS in management and treatment.

**E³ 721-2**
**B. Athlete's heart**
Jan Bogaert; Leuven/BE
*(Jan.Bogaert@uz.kuleuven.ac.be)*

**Learning Objectives:**
1. To learn about the different entities that cause left ventricular thickening.
2. To understand the role of the different techniques in the study of this patients.

### E³ - Advanced Courses: Interactive Teaching Sessions for Young (and not so Young) Radiologists

### E³ 821

### Gynecological cancer imaging

**E³ 821-1**
**A. Cervical cancer**
Teresa Margarida Cunha; Lisbon/PT
*(tmargarida@gmail.com)*

**Learning Objectives:**
1. To describe the imaging findings of cervical cancer.
2. To learn about the new International Federation of Gynecology and Obstretrics (FIGO) classification and its implications.
3. To define the role of radiologic examinations and review ideal imaging protocols.
4. To recognise imaging pitfalls in cervical cancer.

**E³ 821-2**
**B. Ovarian cancer: new challenges**
Stephanie Nougaret; Montpellier/FR

**Learning Objectives:**
1. To be familiar with the histological subtype of the epithelial ovarian cancer.
2. To become familiar with the revised FIGO staging system.
3. To learn about the role of imaging in recognising "difficult to resect" disease.

### E³ - Advanced Courses: Interactive Teaching Sessions for Young (and not so Young) Radiologists

### E³ 921

### Imaging of the face

**E³ 921-1**
**A. Facial infections and complications**
Martin G. Mack; Munich/DE
*(m.mack@radiologie-muenchen.de)*

**Learning Objectives:**
1. To become familiar with the key concepts and imaging features of facial infections.
2. To learn how to integrate clinical findings with radiological features.

**E³ 921-2**
**B. Facial pain**
Alexandra Borges; Lisbon/PT
*(borgalexandra@gmail.com)*

**Learning Objectives:**
1. To become familiar with the key concepts and imaging features of facial pain.
2. To learn how to integrate clinical findings with radiological features.

### $E^3$ - Advanced Courses: Interactive Teaching Sessions for Young (and not so Young) Radiologists

### $E^3$ 1021

### Focal testicular lesions: challenging conditions

**$E^3$ 1021-1**
**A. Acutely painful lesions**
Vikram S. Dogra; Rochester, NY/US
*(Vikram_Dogra@URMC.Rochester.Edu)*

**Learning Objectives:**
1. To describe the imaging findings of tumours and tumour-like testicular lesions presenting clinically with acute scrotal pain.
2. To learn how to integrate clinical and imaging findings for the differential diagnosis between benign and malignant lesions.

**$E^3$ 1021-2**
**B. The small, incidentally detected lesion**
Paul S. Sidhu; London/UK
*(paulsidhu@btinternet.com)*

**Learning Objectives:**
1. To learn about the prevalence of small testicular lesions incidentally detected at US.
2. To learn how to improve characterisation of small incidentally detected testicular lesions.
3. To discuss how to manage the patient with small incidentally detected testicular lesions.
**Author Disclosures:**
Paul S. Sidhu: Advisory Board: Samsung Inc; Consultant: ITREAS; Speaker: Bracco, Siemens

### $E^3$ - Advanced Courses: Interactive Teaching Sessions for Young (and not so Young) Radiologists

### $E^3$ 1121

### Imaging of head and neck manifestations of systemic disease

**$E^3$ 1121-1**
**A. Autoimmune disorders: IgG, Sjogren's syndrome and others**
Minerva Becker; Geneva/CH

**Learning Objectives:**
1. To become familiar with the imaging characteristics of autoimmune disease manifestations in the head and neck.
2. To learn how to avoid interpretation pitfalls on CT, US and MRI.

**$E^3$ 1121-2**
**B. Lymphatic and haematopoietic neoplasms**
Frank Pameijer; Utrecht/NL
*(f.a.pameijer@umcutrecht.nl)*

**Learning Objectives:**
1. To become familiar with the imaging characteristics of lymphatic and haematopoietic neoplasms affecting the head and neck.
2. To learn how to avoid interpretation pitfalls on CT, US and MRI.

### $E^3$ - Advanced Courses: Interactive Teaching Sessions for Young (and not so Young) Radiologists

### $E^3$ 1221

### Breast lesions of uncertain malignant potential (B3 lesions): the management conundrum

**$E^3$ 1221-1**
**A. Imaging appearance of B3 lesions**
Pascal A.T. Baltzer; Vienna/AT

**Learning Objectives:**
1. To understand the entire spectrum of the lesions with uncertain malignant potential (B3).
2. To learn how B3 lesions appear on mammography, tomosynthesis, contrast-enhanced mammography, ultrasound, non-contrast and contrast-enhanced MRI.
3. To discuss their different biological behaviour in relation to contrast-enhanced imaging modalities.

**$E^3$ 1221-2**
**B. The clinical management of B3 lesions**
Simone Schiaffino; Milan/IT
*(schiaffino.simone@gmail.com)*

**Learning Objectives:**
1. To distinguish between low and high-risk B3 lesions.
2. To know the impact of needle size and imaging guidance on exclusion of associated malignancy.
3. To learn how to discuss the clinical management of B3 lesions in the multidisciplinary meeting.
**Author Disclosures:**
Simone Schiaffino: Speaker: GE Healthcare; Bracco Imaging

### $E^3$ - Advanced Courses: Interactive Teaching Sessions for Young (and not so Young) Radiologists

### $E^3$ 1321

### Imaging of the retroperitoneum

**$E^3$ 1321-1**
**A. Retroperitoneal lesions**
Subramaniyan Ramanathan; Doha/QA
*(drrmsubbu@gmail.com)*

**Learning Objectives:**
1. To illustrate the spectrum of primary retroperitoneal tumours and non-neoplastic disease.
2. To explain the key points in the differential diagnosis.
3. To learn how to avoid interpretation pitfalls.

**$E^3$ 1321-2**
**B. Subperitoneal, perirectal and deep pelvic lesions**
Mustafa Secil; Izmir/TR
*(mustafa.secil@deu.edu.tr)*

**Learning Objectives:**
1. To illustrate the spectrum of benign and malignant perirectal and deep pelvic lesions.
2. To discuss the imaging features of these lesions.
3. To understand how to narrow the differential diagnosis.

### E³ - Advanced Courses: Interactive Teaching Sessions for Young (and not so Young) Radiologists

### E³ 1421

### Cross-sectional imaging of bone metastases

**E³ 1421-1**
**A. Is it a bone metastasis (incidental lesions)?**
Sabine Weckbach; Heidelberg/DE
*(sabine.weckbach@med.uni-heidelberg.de)*

**Learning Objectives:**
1. To illustrate the imaging appearance of bone metastases.
2. To review the imaging protocols for evaluation of bone metastases.
3. To illustrate how to differentiate between metastases and other focal bone lesions.
4. To illustrate the pitfalls and limitations of imaging in assessment of metastatic bone disease.

**E³ 1421-2**
**B. Dedicated whole-body staging for bone metastases**
Giuseppe Petralia; Milan/IT
*(giuseppe.petralia@ieo.it)*

**Learning Objectives:**
1. To review current image acquisition and interpretation for whole-body MRI.
2. To illustrate the clinical applications for whole-body MRI in assessment of metastatic bone disease.
3. To illustrate the potential and limitations of whole-body MRI in assessment of metastatic bone disease.

### E³ - Advanced Courses: Interactive Teaching Sessions for Young (and not so Young) Radiologists

### E³ 1521

### Pelvic pain in female patients

**E³ 1521-1**
**A. Acute pain**
Francesca Iacobellis; Naples/IT
*(iacobellisf@gmail.com)*

**Learning Objectives:**
1. To become familiar with different uterine and adnexal pathologies presenting acute pelvic pain.
2. To learn about integrating clinical findings with imaging features before establishing a diagnosis.

**E³ 1521-2**
**B. Chronic pain**
Marcia C. Javitt; Haifa/IL
*(m_javitt@rambam.health.gov.il)*

**Learning Objectives:**
1. To become familiar with different uterine and adnexal pathologies presenting chronic pelvic pain.
2. To learn about integrating clinical findings with imaging features before establishing a diagnosis.

### E³ - Advanced Courses: Neuro

### E³ 118

### Metabolic disorders: common and complex

**E³ 118-1**
**A. Posterior reversible encephalopathy syndrome (PRES): still no name change?**
Sven Haller; Geneva/CH

**Learning Objectives:**
1. To discuss the pathophysiology and new insights of PRES.
2. To give an overview of imaging findings in PRES.
3. To learn how to recognise PRES differentials.
**Author Disclosures:**
Sven Haller: Advisory Board: EPAD; Consultant: Spineart, Wyss

**E³ 118-2**
**B. Acquired metabolic diseases**
Jasmina Boban; Novi Sad/RS
*(jasmina.konstantinovic@gmail.com)*

**Learning Objectives:**
1. To learn about the most important acquired metabolic diseases affecting the brain.
2. To understand the underlying pathology of intoxications.
3. To become familiar with drugs-related neurotoxicity.

**E³ 118-3**
**C. Prognostication of hypoxic-ischaemic injury (HII)**
Seamus Looby; Dublin/IE

**Learning Objectives:**
1. To explain imaging findings in HII.
2. To learn how to evaluate the prognosis.
3. To discuss the diagnosis of brain death.

### E³ - Advanced Courses: Neuro

### E³ 218

### Inflammation: the burning brain

**E³ 218-1**
**A. Vessel wall imaging: how to do it**
Jeroen Hendrikse; Utrecht/NL
*(J.Hendrikse@umcutrecht.nl)*

**Learning Objectives:**
1. To understand vessel wall imaging.
2. To learn when and how to use vessel wall imaging.
3. To learn about the possible pitfalls in using vessel wall imaging.

**E³ 218-2**
**B. Vasculitis: what can we see with new MRI techniques?**
Hans Rolf Jäger; London/UK
*(r.jager@ucl.ac.uk)*

**Learning Objectives:**
1. To discuss the imaging findings in CNS vasculitis.
2. To understand the value of each MRI technique in the evaluation of vasculitis.
3. To discuss the pathological findings and their clinical relevance.

**E³ 218-3**
**C. Autoimmune diseases: new pandemia?**
Philippe Demaerel; Leuven/BE

**Learning Objectives:**
1. To learn about the rising incidence of autoimmune mediated diseases.
2. To discuss the imaging findings suggestive of autoimmune encephalitis.
3. To understand the clinical scenarios and indications for imaging.

### E³ - Advanced Courses: Neuro

### E³ 318

### Brain tumours: new classification, new names, new challenges

**E³ 318-1**
**A. Glioblastoma: what is really important?**
Sotirios Bisdas; London/UK
*(s.bisdas@ucl.ac.uk)*

**Learning Objectives:**
1. To become familiar with the current classifications of glioblastoma.
2. To learn how to distinguish between glioblastoma and lymphoma.
3. To understand the value of advanced techniques in evaluating.

**E³ 318-2**
**B. Child with posterior fossa tumour: what next?**
Zoltán Patay; Memphis, TN/US

**Learning Objectives:**
1. To learn about the most common posterior fossa tumours in children.
2. To understand the challenges in the interpretation of images.
3. To appreciate the new classifications and genetic insights.

**E³ 318-3**
**C. Tumour or therapy-induced changes**
Pia C. Maly Sundgren; Lund/SE

**Learning Objectives:**
1. To learn about therapy-induced phenomena.
2. To appreciate diagnostic strategy in the differentiation between recurrence and therapy-induced changes.
3. To understand how to use advanced techniques in treatment monitoring.

### E³ - Advanced Courses: Neuro

### E³ 418

### Vascular disorders beyond stroke: small arteries and veins

**E³ 418-1**
**A. Cerebral amyloid angiopathy (CAA) and other small vessel diseases**
Fabrice Bonneville; Toulouse/FR
*(bonneville.f@chu-toulouse.fr)*

**Learning Objectives:**
1. To learn about imaging findings in CAA.
2. To discuss vascular dementia.
3. To review imaging findings in small vessel disease.

**E³ 418-2**
**B. Not every black dot on susceptibility weighted imaging (SWI) is a microbleed**
Paul M. Parizel; Perth, WA/AU

**Learning Objectives:**
1. To review differential diagnosis of multiple black dots on SWI.
2. To understand how to use SWI in neurological diseases.
3. To appreciate the value of SWI in routine brain protocols.
**Author Disclosures:**
Paul M. Parizel: Advisory Board: Agfa, icometrix, SmartReporting, Incepto Medical; Consultant: Gold Standard Phantoms; Share holder: icometrix, SmartReporting, Incepto Medical, Gold Standard Phantoms, GE, Siemens Healthineers

**E³ 418-3**
**C. Veins are different**
Pedro Vilela; Almada/PT
*(ferrovilela@sapo.pt)*

**Learning Objectives:**
1. To learn about venous diseases.
2. To review the imaging findings in venous sinus thrombosis.
3. To become familiar with venous infarcts.

### E³ - Advanced Courses: Neuro

### E³ 518

### Seizures: disrupted connections

**E³ 518-1**
**A. Imaging in seizures: how to identify the epileptogenic focus**
Christopher Hess; San Francisco, CA/US

**Learning Objectives:**
1. To discuss the "perfect" protocol in patients with seizures.
2. To learn about the structural MRI in seizures.
3. To discuss the value of functional MRI.

**E³ 518-2**
**B. Temporal lobe epilepsy (TLE)**
Núria Bargalló; Barcelona/ES
*(bargallo@clinic.ub.es)*

**Learning Objectives:**
1. To learn the anatomy of the temporal lobe necessary for the evaluation of TLE.
2. To adopt the "temporal lobe epilepsy protocol".
3. To review the underlying causes of temporal lobe epilepsy.

**E³ 518-3**
**C. Status epilepticus**
Paolo Vitali; Milan/IT

**Learning Objectives:**
1. To learn about the definition and clinical presentation of status epilepticus.
2. To understand CT and MRI findings in status epilepticus.
3. To discuss postictal imaging findings.

### EFOMP Workshop: New European Quality Controls for State-of-the-Art Innovations in Radiology and Artificial intelligence: The Role of the Medical Physicist

### EF 2

### European quality controls (QC) in innovation, safety, and artificial intelligence (AI)

### EF 2-1

**Artificial intelligence: which role for the medical physicist?**
Federica Zanca; Leuven/BE
*(federica.zanca@palindromo.consulting)*

**Learning Objectives:**
1. To understand the rationale and challenges for building a European Curricular and Professional Programme on AI for medical physicists.
2. To learn about a guideline curriculum covering theoretical and practical aspects of education and training for medical physicists in AI within Europe.
3. To become familiar with AI challenges and activities of medical physicists, through worked out examples.

### EF 2-2

**A universal model for breast dosimetry**
Ioannis Sechopoulos; Nijmegen/NL
*(ioannis.sechopoulos@radboudumc.nl)*

**Learning Objectives:**
1. To learn why a Europe wide consistent approach is necessary for medical physicists involved in clinical trials and where they fit in the multidisciplinary team.
2. To understand the rationale and challenges for the new guidances and its relevance to diagnostic accuracy and safety.
3. To become familiar with how the new guidances can be implemented in a practical manner.

**Author Disclosures:**
Ioannis Sechopoulos: Research Grant/Support: Siemens Healthcare, ScreenPoint, Sectra Benelux, Volpara Solutions, Canon Medical; Speaker: Siemens Healthcare

### EF 2-3

**The role of medical physics experts in clinical trials**
Natalie Abbott; Cardiff/UK
*(natalie.abbott@wales.nhs.uk)*

**Learning Objectives:**
1. To learn why a Europe wide consistent approach is necessary for medical physicists involved in clinical trials and where they fit in the multidisciplinary team.
2. To understand the rationale and challenges for the new guidance and its relevance to diagnostic accuracy and safety.
3. To become familiar with how the new guidances can be implemented in a practical manner.

### EuroSafe Imaging Session

*Jointly organised by the ESR and WHO*

### EU 1

### Ethics and radiation protection of the patient: a focus on medical imaging

### EU 1-1

**Ethics and radiation protection of the patient: the WHO perspective**
Jim Malone; Dublin/IE
*(jifmal@gmail.com)*

**Learning Objectives:**
1. To learn about the distinction between ethical behaviour and compliance with guidelines/the law.
2. To become familiar with a widely accepted set of ethical values and how to discern them in medicine and radiation protection (RP).
3. To understand that a personal moral compass alone is not acceptable when acting as a professional.

### EU 1-2

**Ethics in the context of EuroSafe Imaging**
Guy Frija; Paris/FR

**Learning Objectives:**
1. To learn the evolving position of ethics in radiation protection in medicine.
2. To identify ethical challenges for the implementation of the radiation protection system in medical imaging.
3. To appreciate the EuroSafe response to these developments.

### EU 1-3

**Ethics in radiation protection of patients: ICRP activities**
Francois Bochud; Lausanne/CH

**Learning Objectives:**
1. To learn about the existence of ICRP-138, a report setting out the ethics foundation of radiation protection.
2. To appreciate why ICRP is developing a new report on ethics of radiation protection in medical diagnosis/treatment.
3. To understand the concepts and values that will be introduced in the new ICRP report.

### EU 1-4

**Ethics in radiological practice**
Dina Husseiny Salama; Cairo/EG
*(drdinahusseiny@yahoo.com)*

**Learning Objectives:**
1. To learn about the importance of ethics for radiology in day-to-day practice.
2. To become familiar with ethical dilemmas that may arise when implementing the system of radiation protection in radiology.
3. To understand the importance of information for, and personal contact with, patients in day-to-day practice.

### EuroSafe Imaging Session

### EU 3

### Essential requirements for dose management systems (DMS): what do we really need?

### EU 3-1

**Dosimetric parameters of x-ray modalities as an input source for DMS**
Eliseo Vaño; Madrid/ES
*(eliseov@med.ucm.es)*

**Learning Objectives:**
1. To learn about the option of dosimetric quantities to be used for the different imaging modalities.
2. To appreciate the advantages to auditing the typical patient dose values compared to DRLs.
3. To understand the need to periodically validate the dosimetric values transferred to the DMS.

### EU 3-2

**Experience and problems with the implementation of DMS in clinical routine**
Virginia Tsapaki; Athens/GR

**Learning Objectives:**
1. To learn about the practical steps to follow to facilitate the process of procurement to installation.
2. To appreciate the importance of various DMS technical characteristics and how they can affect the clinical practice.
3. To understand the challenges related to the practical implementation of DMS into the daily clinical practice.

### EU 3-3
**Requirements and workflow of DMS in the clinical context of HIS, RIS, and PACS**
Peter Mildenberger; Mainz/DE
*(mildenbe@uni-mainz.de)*

**Learning Objectives:**
1. To learn about the requirements for standardised dose reporting.
2. To appreciate the integration profiles for interoperability between different vendors.
3. To understand the value of the integration of DMS into clinical information systems.

### EU 3-4
**Networking of individual dose management systems to create a centralised dose repository for benchmarking and national dose collection**
Sebastian T. Schindera; Aarau/CH
*(sschindera@aol.com)*

**Learning Objectives:**
1. To learn about the main objectives of a centralised dose repository.
2. To appreciate the requirements of establishing a centralised dose repository.
3. To understand the main challenges of a centralised dose repository.

### EuroSafe Imaging Session

### EU 4
### A historical overview of CT dose exposure: past, present, and future

### EU 4-1
**From past to present: how have advances in CT technology impacted radiation doses?**
Mahadevappa Mahesh; Baltimore, MD/US
*(mmahesh@jhmi.edu)*

**Learning Objectives:**
1. To understand how CT dose-reduction technology evolves.
2. To become familiar with modern CT dose-reduction technology.
3. To learn how advances in CT technology have impacted radiation doses.

**Author Disclosures:**
Mahadevappa Mahesh: Board Member: American College of Radiology, American Association of Physicists in Medicine

### EU 4-2
**Year 2021: what still needs to be done to reduce doses for CT?**
Virginia Tsapaki; Athens/GR

**Learning Objectives:**
1. To propose new strategies that should be implemented for CT dose reduction.
2. To become familiar with the tools available for CT dose management.
3. To learn what still needs to be done to reduce doses for CT.

### EU 4-3
**The road ahead: how can we overcome hurdles towards reliable clinical CT dosimetry?**
John Damilakis; Iraklion/GR

**Learning Objectives:**
1. To appreciate the value of personalised dosimetry in CT.
2. To understand how artificial intelligence can support the CT dose-reduction process.
3. To learn how to overcome obstacles towards reliable clinical CT dosimetry.

### EuroSafe Imaging Session
*Jointly organised by EuroSafe Imaging and ECR Subcommittee on Physics in Medical Imaging*

### EU 7
### Medical radiation exposure of patients

### EU 7-1
**Medical radiation exposure of patients in the United States: methodology, data sources, and results**
Mahadevappa Mahesh; Baltimore, MD/US
*(mmahesh@jhmi.edu)*

**Learning Objectives:**
1. To learn how different data sources can be used to estimate the number of imaging procedures.
2. To appreciate the difficulties in estimating radiation doses per imaging procedure.
3. To understand the method to estimate medical radiation exposure per person.

**Author Disclosures:**
Mahadevappa Mahesh: Board Member: American College of Radiology, American Association of Physicists in Medicine

### EU 7-2
**Medical radiation exposure of the paediatric population**
Donald P. Frush; Durham, NC/US
*(donald.frush@duke.edu)*

**Learning Objectives:**
1. To become familiar with the various imaging procedures performed in the paediatric population.
2. To appreciate how size matters and that imaging protocols are key to dose optimisation.
3. To understand the status of paediatric imaging in the Unites States as per NCRP 184.

**Author Disclosures:**
Donald P. Frush: Author: NCRP Report 184

### EU 7-3
**UNSCEAR's global report on radiation exposure of patients**
Peter Thomas; Yallambie, VIC/AU
*(peter.thomas@arpansa.gov.au)*

**Learning Objectives:**
1. To learn about the survey results of the global radiation exposure of patients.
2. To appreciate the challenges in coordinating data regarding the number of imaging procedures from various countries.
3. To understand how the model can be utilised to estimate radiation exposure.

### C

### Scientific and Educational Exhibits (C)

Full EPOS™ presentations are published at **epos.myESR.org** and can be cited by a Digital Object Identifier (DOI) if the presenter has chosen to publish the work.
